# Introns targeted by plant microRNAs: a possible novel mechanism of gene regulation

**DOI:** 10.1186/1939-8433-6-8

**Published:** 2013-04-15

**Authors:** Yijun Meng, Chaogang Shao, Xiaoxia Ma, Huizhong Wang

**Affiliations:** College of Life and Environmental Sciences, Hangzhou Normal University, Xuelin Street 16#, Xiasha, Hangzhou, 310036 P. R. China; College of Life Sciences, Huzhou Teachers College, Huzhou, 313000 P. R. China

**Keywords:** Plant microRNA, Pre-mRNA (the precursor of messenger RNA), Intron, RDR (RNA-dependent RNA polymerase), DCL (Dicer-like), Phased small RNA, Degradome

## Abstract

**Background:**

In plant cells, most microRNAs (miRNAs) perform cleavages of target mature mRNAs in the cytoplasm. A recent report of a miRNA pathway involved in DNA methylation in the rice nucleus raises the possibility that plant miRNAs could cleave intron-containing pre-mRNAs (the precursor of messenger RNAs) located in the nucleus.

**Results:**

In this study, we searched for the miRNA binding sites present within the introns of *Arabidopsis* (*Arabidopsis thaliana*) and rice (*Oryza sativa*) genes. All miRNA—intron interactions predicted to result in cleavages were validated by using the public degradome sequencing data. As a result, 40 miRNA—intron pairs involving 25 miRNAs in *Arabidopsis* and 1912 pairs involving 91 miRNAs in rice were identified. For several rice genes, not all transcription forms (alternative splicing variants) were under similar regulation by specific miRNAs. Certain transcripts could escape cleavages due to the absence of intronic miRNA binding sites within these sequences. In some instances, specific cleaved intron remnants could be converted to double-stranded RNAs (dsRNAs) by RNA-dependent RNA polymerase 2. These dsRNAs could then be processed into 21- and 24-nt phased sRNAs by the activity of Dicer-like 1 and 3, respectively. The resultant siRNAs have the potential to be incorporated into Argonaute (AGO)-associated silencing complexes and result in cleavages of target pre-mRNA sequences.

**Conclusions:**

A regulatory model, miRNA—targeting of intron-containing pre-mRNAs—phased sRNAs—targeting of mature mRNAs is proposed, which further expands the potential modes of action of plant miRNAs.

**Electronic supplementary material:**

The online version of this article (doi:10.1186/1939-8433-6-8) contains supplementary material, which is available to authorized users.

## Background

MicroRNAs (miRNAs) play an essential role in modulating gene expression levels in organisms (Carthew & Sontheimer 2009; Chen 2009; Jones-Rhoades et al. 2006; Voinnet 2009). In plants, miRNAs are involved in many essential biological processes, such as morphogenesis, hormone signaling, and developmental phase transition (Chen 2009; Jones-Rhoades et al. 2006). Mediated by RNA polymerase II, a miRNA gene (Lee et al. 2004; Xie et al. 2005) is firstly transcribed into pri-miRNA (primary microRNA). After two-step cleavages by Dicer-like 1 (DCL1), the pri-miRNA is processed into the pre-miRNA (precursor microRNA), then into a miRNA/miRNA* duplex. The duplex is exported to the cytoplasm, and the miRNA strand is selectively incorporated into specific Argonaute (AGO)-associated gene silencing complex. Based on the well-established model, a dominant portion of the plant miRNAs are associated with AGO1. These miRNAs could guide the silencing complexes to bind the transcripts containing highly complementary recognition sites. Then, site-specific cleavages could be performed in the cytoplasm (Jones-Rhoades et al. 2006; Voinnet 2009). In this regard, the mature mRNAs (messenger RNAs), but not the pre-mRNAs (the precursor of messenger RNA), of the protein-coding genes, should be the miRNA targets. However, a recent report in rice showed us that some of the 24-nt miRNAs associated with AGO4 complexes could mediate DNA methylation in the nucleus (Wu et al. 2010). This raises the possibility of cleavages of the nuclear-localized, intron-containing pre-mRNAs by certain AGO1-associated miRNAs.

In this study, we performed a large-scale search for the miRNA binding sites present within the introns of *Arabidopsis* (*Arabidopsis thaliana*) and rice (*Oryza sativa*). All the cleavage-based miRNA—intron interactions were validated by using public degradome sequencing data. As a result, 40 miRNA—intron pairs in *Arabidopsis*, and 1912 miRNA—intron pairs in rice were identified. Several interesting phenomena were observed. In some cases in rice, certain transcription forms (alternative splicing variants) of a gene could escape from the regulation by specific miRNA due to the absence of the binding sites within the intron. Also in rice, some cleaved intron remnants could be converted to double-stranded RNAs (dsRNAs) by RNA-dependent RNA polymerase 2 (RDR2). These dsRNAs could be further processed into 21- and 24-nt phased small RNAs (sRNAs) relying on the activity of DCL1 and DCL3, respectively. Some of the phased sRNAs could be incorporated into AGO1 or AGO4 clade proteins. Some of the AGO1-associated sRNAs possessed great potential to perform site-specific target cleavages. Based on the above results, a novel mode of action of the plant miRNAs in the nucleus, involving the regulatory cascade miRNA—intron—secondary phased sRNAs—targets, was proposed. We hope that this study inspires further research efforts on thorough elucidation of this novel action mode, which could advance the current understanding on the miRNA activities in plants.

## Results and discussion

### Identification of miRNA—intron pairs

The annotated introns of *Arabidopsis* and rice were collected from the *Arabidopsis* information resource (TAIR, release 10) (Huala et al. 2001) and the rice genome annotation project established by the institute for genome research (TIGR rice, release 6.1) (Yuan et al. 2003), respectively. All the introns with 30-nt flanking sequences at both ends were subjected to miRNA (miRBase (Griffiths-Jones et al. 2008), release 18) binding site prediction by using miRU algorithm (Dai & Zhao 2011; Zhang 2005). Plant degradome sequencing is quite efficient for the identification of miRNA cleavage sites on the targets (Addo-Quaye et al. 2008; German et al. 2009; German et al. 2008; Li et al. 2010; Pantaleo et al. 2010). In this regard, the predicted miRNA—intron pairs were subjected to degradome sequencing data-based validation by employing target plots as previously described (German et al. 2009; German et al. 2008; Meng et al. 2011). The publicly available degradome sequencing data sets (11 in *Arabidopsis* and 4 in rice) were utilized (see details in Methods). As a result, 40 miRNA—intron interactions involving 25 miRNAs in *Arabidopsis* and 1912 interactions involving 91 miRNAs in rice were obtained (Additional file 1: Figure S1, Additional file 2: Figure S2, Additional file 3: Figure S3 and Additional file 4: Figure S4, and Additional file 5: Table S1 and Additional file 6: Table S2). The number of the miRNA—intron pairs identified in *Arabidopsis* is much less than that in rice. This discrepancy is likely resulted from the smaller numbers of the annotated introns and the registered miRNAs in *Arabidopsis* when compared to those in rice (175,512 introns in *Arabidopsis* vs. 251,812 in rice; 328 miRNAs in *Arabidopsis* vs. 661 in rice). Together, these results indicate that certain plant miRNAs might perform cleavages on the intron-containing pre-mRNAs in the nucleus, which needs further validation.

**Figure 1:**
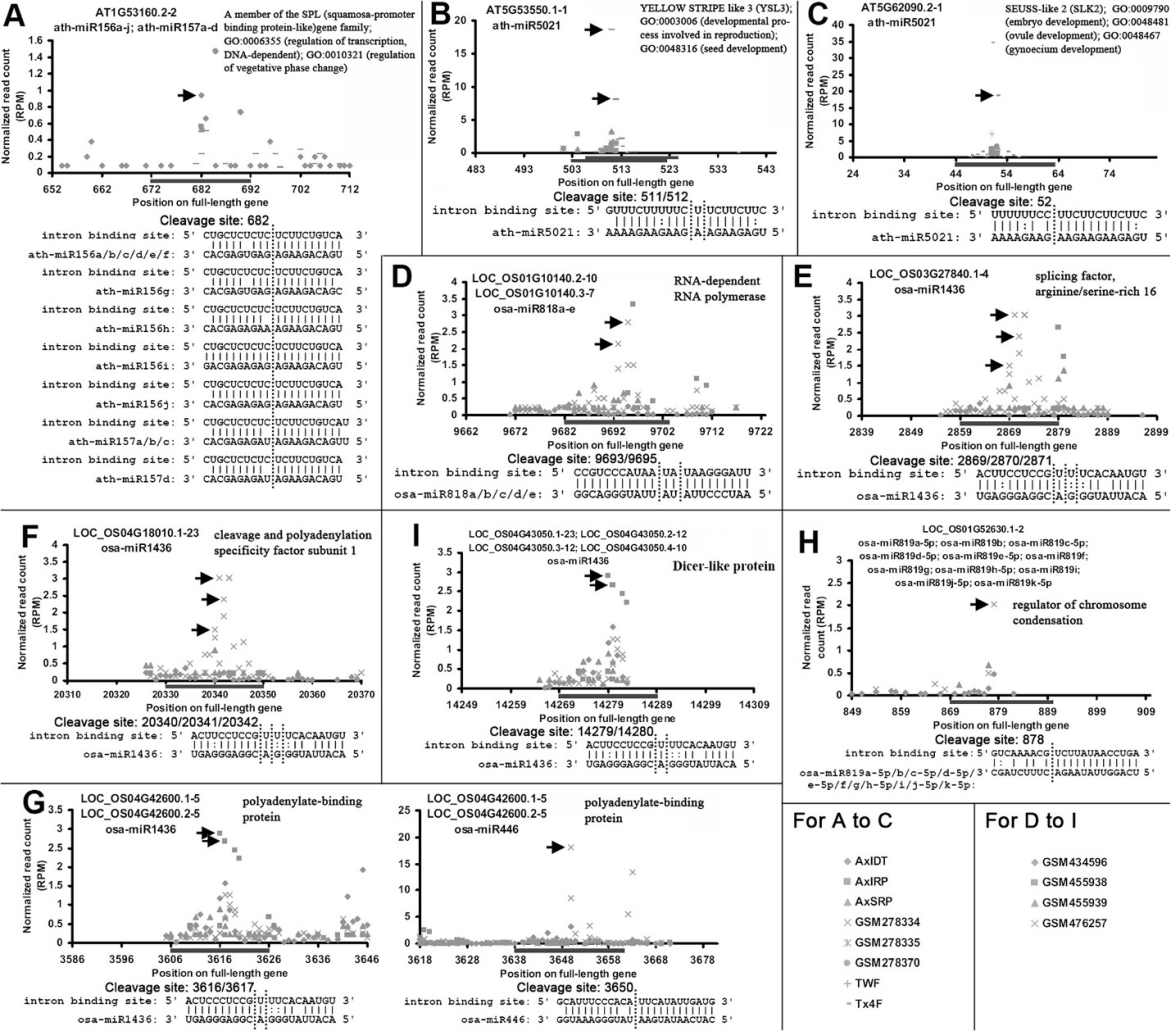
**Degradome sequencing data**-**based identification of microRNA** **(miRNA)—** **intron pairs.** (**A**) to (**C**) miRNA—intron pairs in *Arabidopsis*. (**D**) to (**I**) miRNA—intron pairs in rice. For (**A**) to (**I**), the *x* axes indicate the positions of the miRNA binding sites (marked by horizontal lines) on the full-length genes, and the *y* axes measure the intensity (normalized in RPM, reads per million) of the degradome signals. The prominent cleavage signals were marked by arrowheads on the target plots (t-plots). The figure keys for the degradome signatures are shown at the right bottom. The miRNAs, the intron IDs and the target gene annotations are shown on the top of each t-plot. For each t-plot, the alignment of the miRNA sequence against the binding site is shown, and the cleavage sites are indicated by vertical dotted lines.

Further functional investigations discovered several interesting target genes. In *Arabidopsis*, AT1G53160.2-2 (represents the 2^nd^ intron of the transcription form AT1G53160.2 of the gene *AT1G53160*) was regulated by ath-miR156 and ath-miR157 (Figure 1A). *AT1G53160* is an *SPL* (*squamosa*-*promoter binding protein*-*like*) gene family member, and it is involved in the regulation of developmental phase transition. AT5G53550.1-1 was regulated by ath-miR5021 (Figure 1B). *AT5G53550* encodes YSL3 (YELLOW STRIPE like 3) protein participating in seed development and reproduction. AT5G62090.2-1 was also regulated by ath-miR5021 (Figure 1C), and *AT5G62090* encodes SLK2 (SEUSS-like 2) involved in embryo, ovule and gynoecium development. Consistently, all these target genes play important roles in reproduction. In rice, several target genes were annotated to be involved in RNA-level biological processes, such as *LOC* _*OS01G10140* (RNA-dependent RNA polymerase) regulated by osa-miR818 (Figure 1D), *LOC* _*OS01G52630* (regulator of chromosome condensation) regulated by osa-miR819 (Figure 1H), and *LOC* _*OS03G27840* (splicing factor, arginine/serine-rich 16), *LOC* _*OS04G18010* (cleavage and polyadenylation specificity factor subunit 1) and *LOC* _*OS04G43050* (Dicer-like protein) regulated by osa-miR1436 (Figure 1E, F and I). More interestingly, the 5^th^ intron of *LOC* _*OS04G42600* (polyadenylate-binding protein) was simultaneously regulated by osa-miR1436 and osa-miR446, and the binding sites were neighboring to each other (Figure 1G).

**Figure 2:**
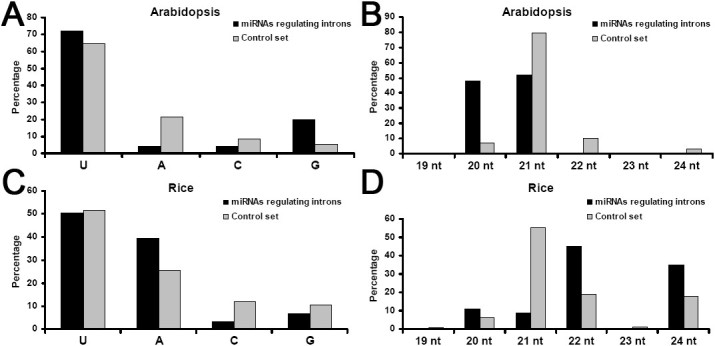
**Sequence characteristics of the microRNAs** **(miRNAs)** **targeting introns.** (**A**) 5’ terminal compositions of the miRNAs of *Arabidopsis*. (**B**) Length distribution patterns of the miRNAs of *Arabidopsis*. (**C**) 5’ compositions of the miRNAs of rice. (**D**) Length distribution patterns of the miRNAs of rice. For each plant, all of its miRBase-registered (release 18) miRNAs excluding those targeting introns were served as a control set.

The sequence characteristics, including 5’ terminal nucleotide compositions and sequence length, of the 25 miRNAs targeting introns in *Arabidopsis* and the 91 miRNAs in rice were analyzed. The remaining miRBase-registered (release 18) miRNAs of the two plants (i.e. 328–25 = 303 miRNAs in *Arabidopsis*; 661–91 = 570 miRNAs in rice) were recruited to create the control sets. The results showed that, in both plants, the 5’ terminal compositions of the miRNAs targeting introns were similar to the control sets (Figure 2A and C). However, significant differences of the sequence length were observed. In *Arabidopsis*, the miRNAs regulating introns are highly enriched in 20 nt, which is not the case for the control set (Figure 2B). In rice, compared to the control set, the miRNAs involved in intron regulation are not enriched in 21 nt, but in 22 and 24 nt (Figure 2D). The biological implications under the observed length discrepancies need further investigations.

### Certain transcription form(s) of a rice gene could escape from the regulation of a specific miRNA due to the lack of the miRNA binding site

**Figure 3:**
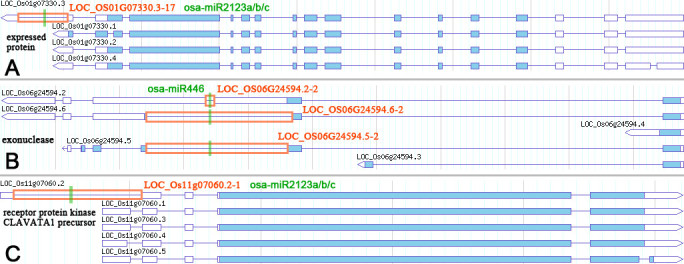
**Certain transcription form** **(s)** **of a rice gene could escape from the regulation of a specific microRNA** **(miRNA).** (**A**) The 17^th^ intron of LOC_OS01G07330.3, which does not exist in LOC_OS01G07330.1, LOC_OS01G07330.2 and LOC_OS01G07330.4, is regulated by osa-miR2123a/b/c. (**B**) The 2^nd^ introns of LOC_OS06G24594.2, LOC_OS06G24594.5 and LOC_OS06G24594.6 were regulated by osa-miR446, whereas LOC_OS06G24594.3 and LOC_OS06G24594.4 lacking the corresponding binding site escaped from this regulation. (**C**) For the five transcription forms of *LOC* _*OS11G07060*, only LOC_OS11G07060.2 with a miRNA binding site within its 1^st^ intron was regulated by osa-miR2123a/b/c. From (**A**) to (**C**), the introns containing miRNA binding sites (denoted by green vertical lines) were marked by orange boxes. The intron IDs and the miRNAs are listed. The graphic presentations of the transcription forms were retrieved from the rice genome annotation project (TIGR rice) (Yuan et al. 2003).

We found that, for the nine rice genes (*LOC* _*OS01g07330*, *LOC* _*OS04g01530*, *LOC* _*OS04g51809*, *LOC* _*OS06g24594*, *LOC* _*OS07g35920*, *LOC* _*OS09g34140*, *LOC* _*OS10g04674*, *LOC* _*OS11g07060* and *LOC* _*OS12g40419*), not all of their annotated (TIGR rice, release 6.1) transcription forms were under similar regulations of specific miRNAs. Instead, certain transcription forms could escape from the miRNA surveillance due to the lack of the corresponding miRNA binding sites (Additional file 7: Figure S5). For example, *LOC* _*OS01g07330* has four annotated transcription forms, i.e. LOC_OS01g07330.1, LOC_OS01g07330.2, LOC_OS01g07330.3 and LOC_OS01g07330.4. However, only LOC_OS01g07330.3 was regulated by osa-miR2123. The binding site of osa-miR2123 resides within the 17^th^ intron of LOC_OS01g07330.3, which could not be found on the other three transcription forms (Figure 3A). For another example, osa-miR446 regulated LOC_OS06g24594.2, LOC_OS06g24594.5 and LOC_OS06g24594.6 by recognizing the binding sites within the 2^nd^ introns of these transcripts. However, LOC_OS06g24594.3 and LOC_OS06g24594.4 could evade this regulation due to the lack of the corresponding introns (Figure 3B). Similarly, among the five transcription forms of *LOC* _*OS11G07060*, only LOC_OS11G07060.2 was under the surveillance of osa-miR2123 (Figure 3C). Based on these observations, the canonical notion that the plant genes are passively regulated by specific miRNAs may have to be revised. We speculate that, for certain genes, some of their transcription forms lost the miRNA binding sites during evolution, thus partially eliminating the regulatory effects of specific miRNAs.

### RDR2-dependent synthesis of dsRNAs from the cleaved intron remnants and DCL1/3-dependent production of phased sRNAs

The introns are spliced out and subject to degradation during mRNA maturation. However, based on our previous study, huge numbers of short sequences generated by sRNA high-throughput sequencing (HTS) could be mapped to the introns (data not shown). Focusing on our study, one question was raised here: once sliced by specific miRNAs, were the cleaved intron remnants totally subjected to degradation? Referring to the biogenesis model of the ta-siRNA (-acting small interfering RNA) (Allen et al. *trans*^2005^; Williams et al. 2005), we hypothesized that the cleaved remnants might be converted to dsRNAs, which could be further processed into phased secondary sRNAs through DCL-mediated dicing.

To test this possibility, firstly, the short sequences from the publicly available sRNA HTS data sets (see details of these HTS data sets in Additional file 8: Table S3 and Additional file 9: Table S4) were mapped onto the miRNA-regulated introns (see intron IDs in Additional file 5: Table S1 and Additional file 6: Table S2). Only the perfectly matched sRNAs were retained. Then, a Perl script was developed to search for the phase-distributed sRNAs within the introns. The search criteria were set as follows: (1) A defined set of phased sRNAs, it should be constituted by three or more continuous sRNA duplexes with constant sequence length (defined as phase step), such as 21 nt. (2) According to the enzymatic feature of DCLs, each sRNA duplex diced from the dsRNA synthesized from an intron should possess 2-nt 3’ overhangs at both ends. (3) Considering the possibility that the HTS data utilized in this study might not reflect the expression levels of all the phased sRNAs, the phased sRNA duplex with only one detectable strand was allowed. (4) For the overlapping sRNA sets sharing the same phase step, the longest set was retained for the analysis.

As a result, thousands of phased sRNA sets were discovered within the introns of rice, but not of *Arabidopsis*. According to the biogenesis model of ta-siRNAs, the miRNA cleavage sites on the target transcripts could define the start points of the phased sRNA sets (Allen et al. 2005; Williams et al. 2005). In this regard, another Perl script was developed to search for the phased sRNA sets with either ends resided within the intronic miRNA binding sites validated based on the degradome sequencing data (Additional file 1: Figure S1, Additional file 2: Figure S2, Additional file 3: Figure S3 and Additional file 4: Figure S4). Interestingly, for most of the sRNA sets identified, the corresponding miRNA binding sites located at the 5’ ends of these sets (Additional file 10: Data S1). This observation fits well with the notion that the 3’ remnants cleaved by specific miRNAs are more stable than the 5’ remnants.

The above results only indicate the possibility that the introns cleaved by specific miRNAs might be converted to dsRNAs for the production of phased sRNAs. We attempted to obtain more evidences. Considering the fact that the synthesis of the dsRNAs depends on RDR(s), and the production of the phased sRNAs relies on DCL(s), we utilized the following sRNA HTS data sets for further analysis: GSM520640 (prepared from the wild type rice seedlings, served as the control set for GSM520639, GSM520637 and GSM520638), GSM520639 (prepared from the rice mutant *rdr2*), GSM520637 (prepared from the rice mutant *dcl1*), GSM520638 (prepared from the rice mutant *dcl3*), GSM455965 (total RNA extracts from rice seedlings, served as the control set for the following six data sets), GSM455962 (sRNAs associated with the rice AGO1a protein), GSM455963 (sRNAs associated with the rice AGO1b protein), GSM455964 (sRNAs associated with the rice AGO1c protein), GSM520634 (sRNAs associated with the rice AGO4a protein), GSM520635 (sRNAs associated with the rice AGO4b protein) and GSM520636 (sRNAs associated with the rice AGO16 protein). The accumulation levels of each sRNA belonging to a phased set were calculated for all the above sRNA HTS data sets.

**Figure 4:**
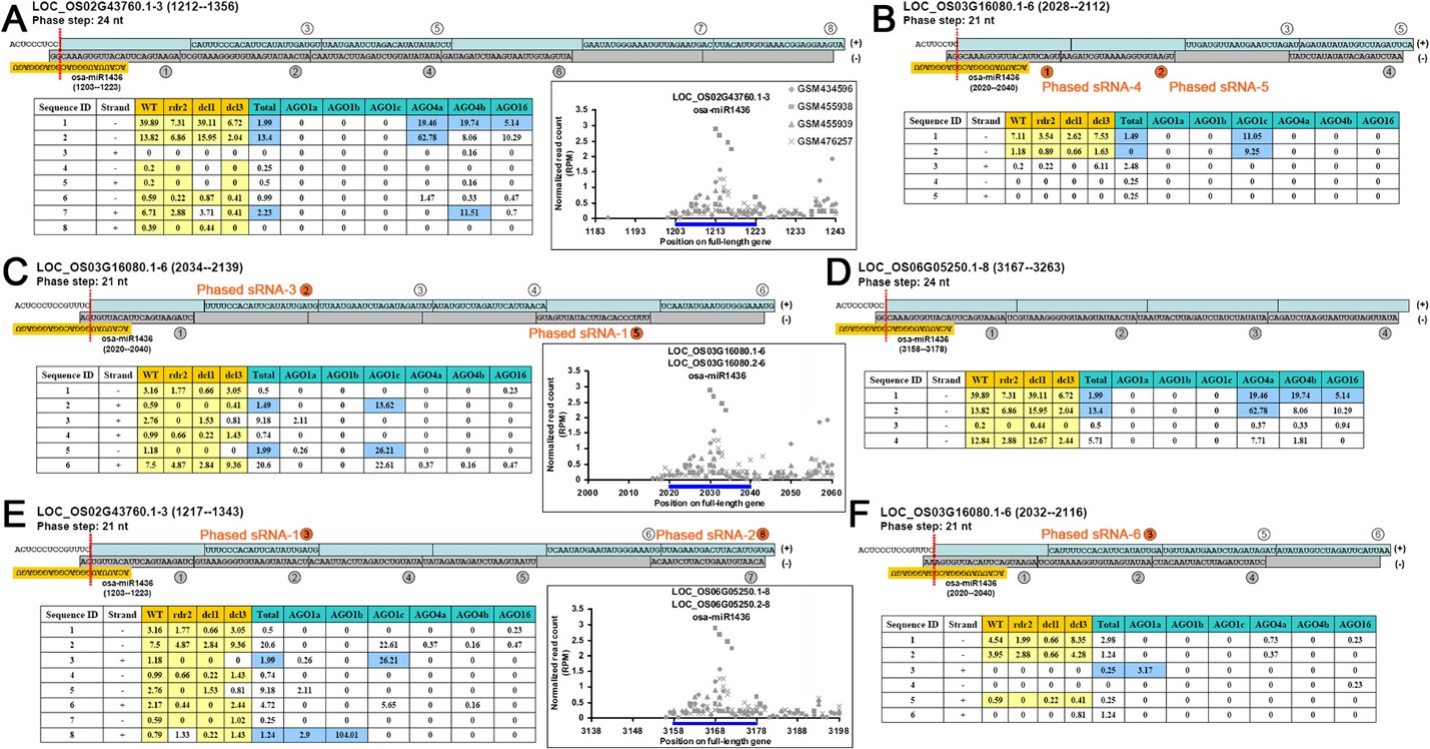
**Sequencing data**-**based evidences for the possible novel pathway microRNA** **(miRNA)—** **intron** **—phased small RNAs** **(sRNAs).** (**A**) Cleavage of the 3^rd^ intron of LOC_OS02G43760.1 by osa-miR1436 resulted in the production of 24-nt phased sRNAs. The region (i.e. 1212 nt to 1356 nt) for phased sRNA production on the full-length gene is shown. The sequence of osa-miR1436 along with its binding site is also shown. The sense strand of the targeted intron was highlighted in blue color, and the antisense strand was in gray. Based on the high-throughput sequencing (HTS) data, the sequences of the phased sRNAs detected by HTS were present in the corresponding boxes, and were numbered accordingly. The accumulation levels of these sRNAs in *rdr2*, *dcl1* and *dcl3*, and in different Argonautes are shown in the table. Similar illustrations apply to the other figure panels. (**B**) Cleavage of LOC_OS03G16080.1-6 by osa-miR1436 resulted in the production of 21-nt phased sRNAs. (**C**) Cleavage of LOC_OS03G16080.1-6 by osa-miR1436 resulted in the production of 21-nt phased sRNAs. (**D**) Cleavage of LOC_OS06G05250.1-8 by osa-miR1436 resulted in the production of 24-nt phased sRNAs. (**E**) Cleavage of LOC_OS02G43760.1-3 by osa-miR1436 resulted in the production of 21-nt phased sRNAs. (**F**) Cleavage of LOC_OS03G16080.1-6 by osa-miR1436 resulted in the production of 21-nt phased sRNAs. Target plots were provided to show the cleavage signals within the miRNA binding sites. For all the target plots, the *x* axes indicate the positions of the binding sites (marked by blue horizontal lines) on the full-length genes, and the *y* axes measure the intensity (normalized in RPM, reads per million) of the degradome signals. The figure keys for degradome signatures belonging to different sequencing libraries are shown on the first plot. The intron IDs and the miRNAs are shown on the top of each plot.

Several supportive evidences were obtained (Figure 4). For instance, the 3^rd^ intron of LOC_OS02G43760.1 was cleaved by osa-miR1436 (see the target plot between Figure 4A and B). Using the 3’ cleaved intron remnant as the template, the antisense strand was synthesized, resulting in the production of 24-nt phased sRNAs. Notably, the accumulation levels of most of these sRNAs were highly dependent on RDR2 and DCL3, but not DCL1. Some of these 24-nt sRNAs were enriched in AGO4 and AGO16 (Figure 4A). Another similar example is the 8^th^ intron of LOC_OS06G05250.1. The levels of all the phased sRNAs generated from this intronic region were influenced by the activities of RDR2 and DCL3. Among these sRNAs, two were associated with AGO4 and AGO16 (Figure 4D). Different from the 24-nt phased sRNAs, the accumulation levels of the 21-nt phased sRNAs produced from the 6^th^ intron of LOC_OS03G16080.1 highly depended on RDR2 and DCL1, but not DCL3. Besides, some of these 21-nt sRNAs were incorporated into the AGO1 protein complexes (Figure 4C). Another intriguing observation is that distinct sRNA phase sets could initiate from different cleavage sites within the same miRNA binding site. For example, three different sets of 21-nt phase step initiated from different cleavage sites within the binding site of osa-miR1436 on the 6^th^ intron of LOC_OS03G16080.1 (Figure 4B, C and F). All these cleavage sites were supported by the degradome sequencing data (see the target plot between Figure 4C and D). Another case was observed for the 3^rd^ intron of LOC_OS02G43760.1. Two sets with distinct phase steps (21-nt and 24-nt respectively) were initiated from different cleavage sites within the binding region of osa-miR1436 (Figure 4A and E). The results presented here strongly support our hypothesis that the miRNA-mediated cleavages of certain rice introns might result in dsRNA synthesis and phased sRNA production. This process is highly dependent on the activities of RDR2 and DCL1/3.

**Figure 5:**
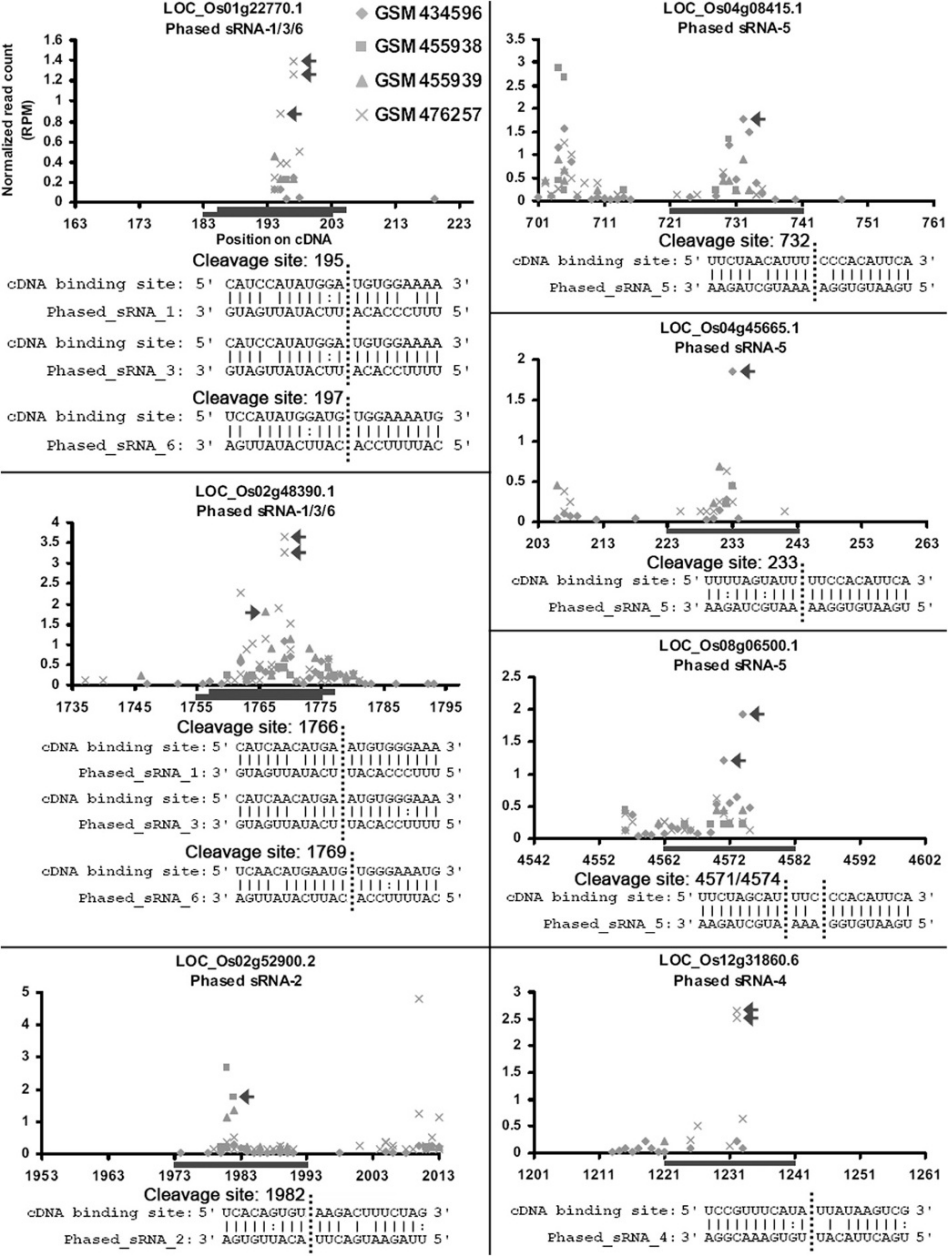
**Degradome sequencing data**-**based identification of the targets regulated by certain phased small RNAs** **(sRNAs).** As shown in Figure 4, the phased sRNAs enriched in Argonaute 1 (numbered and highlighted in orange color in Figure 4B, C, E and F) were selected for this analysis. For all the panels, the *x* axes indicate the positions of the miRNA binding sites (marked by blue horizontal lines) on the mature mRNAs, and the *y* axes measure the intensity (normalized in RPM, reads per million) of the degradome signals. The prominent cleavage signals were marked by arrowheads. The figure keys for degradome signals belonging to different libraries are shown on the first panel. The target transcript IDs and the phased sRNA IDs are shown on the top of each panel. For each plot, the alignment of the sRNA sequence against the binding site is shown, and the cleavage site(s) was (were) indicated by vertical dotted line(s).

Although the biogenesis of the intronic phased sRNAs is quite similar to ta-siRNAs, we did not know whether these sRNAs could cleave specific targets just as the ta-siRNAs. To this end, the intronic phased sRNAs enriched in AGO1 (refer to Figure 4) were selected for analysis. The rice mature mRNAs were used for target prediction in order to test whether these phased sRNAs could cleave targets in the cytoplasm. Degradome sequencing data-based validation showed that several transcripts, such as LOC_OS01G22770.1, LOC_OS02G48390.1, LOC_OS02G52900.2, LOC_OS04G08415.1, LOC_OS04G45665.1, LOC_OS08G06500.1 and LOC_OS12G31860.6, could be cleaved by specific phased sRNAs (Figure 5 and Additional file 11: Figure S6).

## Conclusions

### miRNA—intron—phased sRNAs—targets: a possible novel regulatory cascade reflecting a new mode of action of the plant miRNAs

According to the canonical model of miRNA biogenesis and action in plants (Jones-Rhoades et al. 2006; Voinnet 2009), the miRNA transcripts were processed and exported to the cytoplasm. The miRNA strands were then incorporated into the AGO1-associated silencing complexes, guiding the complexes to bind targets such as the mature mRNAs. Then, the target transcripts were cleaved by the AGO1 complexes in the cytoplasm. However, a recent study by Qi’s group demonstrated that certain miRNAs could mediate DNA methylation in the plant nucleus (Wu et al. 2010). In this regard, we hypothesized that some of the AGO1-associated miRNAs regulate the intron-containing pre-mRNAs in the nucleus. In this study, the possibility of the nuclear-localized interactions between the miRNAs and the introns was tested in two model plants. Especially in rice, dozens of predicted miRNA binding sites on the introns were supported by the degradome sequencing data.

**Figure 6:**
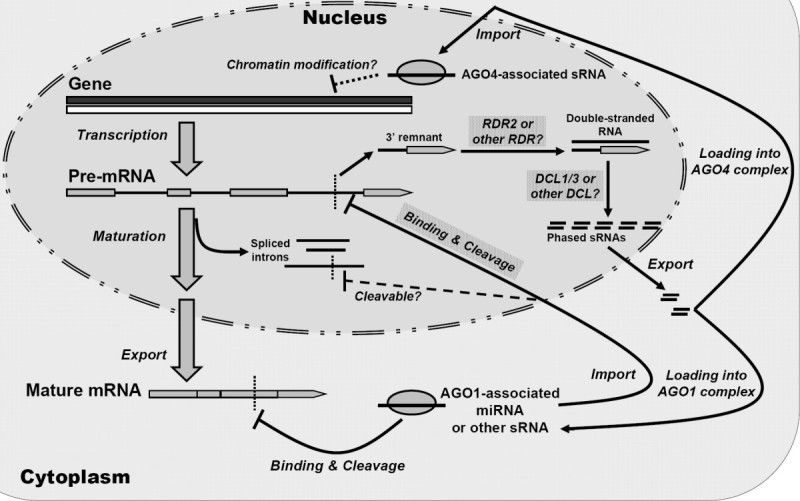
**Proposed model involving microRNA**—**intron regulation and secondary phased small RNA amplification in plants.**

Further investigation indicated that the cleaved intron remnants could be converted to dsRNAs for the production of phased secondary sRNAs. In rice, the generation of certain sRNA phase sets was demonstrated to rely on RDR2 and DCL1/3, which was quite similar to the biogenesis of the ta-siRNAs (Jones-Rhoades et al. 2006; Allen et al. 2005; Williams et al. 2005). Moreover, good correlation between the length of the phased sRNAs and their dependence on specific DCLs, and also their selective association with specific AGO proteins were clearly observed. Taken together, these evidences support the novel regulatory pathway “miRNA—intron—phased secondary sRNAs” (Figure 6). Target prediction and degradome sequencing data-based validation showed that some of the AGO1-associated phased sRNAs could mediate target cleavages in rice (Figure 5). However, whether or not the AGO4-associated phased sRNAs possess a chromatin-level regulatory activity needs to be further investigated.

Thus, we propose a novel additional regulatory cascade “miRNA—intron—phased secondary sRNAs—targets”. However, much more experimental efforts are needed to thoroughly elucidate this possible novel pathway. For example, we should experimentally exclude the possibility that the observed intronic cleavage signals originated from the retained (unspliced) introns in the cytoplasm. We hope that our study would advance the current understanding on the modes of action of miRNAs in plants.

## Methods

### Data resources

All the miRNAs of *Arabidopsis* and rice were retrieved from miRBase (http://www.mirbase.org/; release 18) (Griffiths-Jones et al. 2008).

The annotated transcription forms, the introns, and the gene annotations of *Arabidopsis* were retrieved from the FTP site of the *Arabidopsis* information resource (TAIR, release 10; ftp://ftp.arabidopsis.org/home/tair/Sequences/blast_datasets/TAIR10_blastsets/) (Huala et al. 2001), and those of rice were obtained from the FTP site of the rice genome annotation project established by the institute for genome research (TIGR rice, release 6.1;  ftp://ftp.plantbiology.msu.edu/pub/data/Eukaryotic_Projects/o_sativa/annotation_dbs/pseudomolecules/version_6.1/) (Yuan et al. 2003).

For the *Arabidopsis* degradome sequencing data sets, GSM278333, GSM278334, GSM278335 and GSM278370 were retrieved from Gene Expression Omnibus (GEO; http://www.ncbi.nlm.nih.gov/geo/) (Barrett et al. 2009), and AxIDT, AxIRP, AxSRP, Col, ein5l, TWF and Tx4F were retrieved from *Arabidopsis* PARE Database (http://mpss.udel.edu/at_pare/) (Nakano et al. 2006). For rice, the degradome data sets GSM434596, GSM455938, GSM455939 and GSM476257 were retrieved from GEO.

The sRNA HTS data sets of *Arabidopsis* and rice were downloaded from GEO, Next-Gen Sequence Databases (http://mpss.udel.edu/) (Nakano et al. 2006), and Cereal Small RNAs Database (CSRDB; http://sundarlab.ucdavis.edu/smrnas/) (Johnson et al. 2007). See Additional file 8: Table S3 and Additional file 9: Table S4 for detailed information.

### Prediction and validation of the intron targets of the miRNAs

Target prediction was performed by using miRU algorithm (Dai & Zhao 2011; Zhang 2005) with default parameters. The degradome sequencing data were utilized to validate the predicted miRNA—intron pairs. First, in order to allow cross-library comparison, the normalized read count (in RPM, reads per million) of a short sequence belonging to a specific degradome library was calculated by dividing the raw count of this sequence by the total counts of the library, and then multiplied by 10^6^. Then, two-step filtering was performed to extract the most likely miRNA—intron interactions. During the first step, the predicted miRNA binding sites along with the 50-nt surrounding sequences at both ends were collected. For the BLAST, all the collected degradome data sets were utilized at the same time to do a comprehensive search. It was based on the scenario that a miRNA—intron pair was considered to be the candidate once the cleavage signals were detected in any data set(s). The introns meeting the following criteria were retained: (1) there must be perfectly matched degradome signatures with their 5’ ends resided within 8—14 nt region away from the 5’ ends of the miRNAs; and (2) for a specific position within the 8—14 nt region (regarded as the potential cleavage site) there must be two or more distinct degradome signatures to support this position. These retained transcripts were subjected to a second BLAST, and the degradome signals along each full-length gene were obtained to provide a global view of the signal noise when compared to the signal intensity within a specific binding site. Referring to our previous study (Meng et al. 2011), the target plots (German et al. 2009; German et al. 2008) were drawn for the subsequent manual filtering. Only the introns with cleavage signals easy to be recognized were extracted as the miRNA—intron pairs.

## Electronic supplementary material

Additional file 1: Figure S1: Target plot-based validation of the microRNA—intron interactions in *Arabidopsis*. Only the miRNA binding sites (indicated by blue horizontal lines) surrounded by 20-nt sequences at both ends were shown. (PDF 58 KB)

Additional file 2: Figure S2: Global views of the degradome sequencing signatures along the full-length target genes of *Arabidopsis*. (PDF 2 MB)

Additional file 3: Figure S3: Target plot-based validation of the microRNA—intron interactions in rice. Only the miRNA binding sites (indicated by blue horizontal lines) surrounded by 20-nt sequences at both ends were shown. (PDF 446 KB)

Additional file 4: Figure S4: Global views of the degradome sequencing signatures along the full-length target genes of rice. (PDF 13 MB)

Additional file 5: Table S1: List of the microRNA—intron pairs in *Arabidopsis*. All these pairs were supported by degradome sequencing data. (XLS 16 KB)

Additional file 6: Table S2: List of the microRNA—intron pairs in rice. All these pairs were supported by degradome sequencing data. (XLS 127 KB)

Additional file 7: Figure S5: Observed distinct microRNA—intron regulatory patterns among different transcription models of a rice gene. (PDF 157 KB)

Additional file 8: Table S3: Small RNA high-throughput sequencing data sets of *Arabidopsis* used in this study. (XLS 23 KB)

Additional file 9: Table S4: Small RNA high-throughput sequencing data sets of rice used in this study. (XLS 26 KB)

Additional file 10: Data S1: Detailed information of all the introns with potential of producing phase-distributed secondary small RNAs after microRNA-mediated cleavages. (TXT 419 KB)

Additional file 11: Figure S6: Global views of the degradome sequencing signatures along the mRNAs targeted by specific phased small RNAs in rice. (PDF 190 KB)

Below are the links to the authors’ original submitted files for images.

Authors’ original file for figure 1

Authors’ original file for figure 2

Authors’ original file for figure 3

Authors’ original file for figure 4

Authors’ original file for figure 5

Authors’ original file for figure 6

## Abbreviations

miRNA: MicroRNA
mRNA: Messenger RNA
AGO: Argonaute
dsRNA: Double-stranded RNA
pre-mRNA: The precursor of messenger RNA
RDR: RNA-dependent RNA polymerase
DCL: Dicer-like
pri-miRNA: Primary microRNA
pre-miRNA: Precursor microRNA
sRNA: Small RNA
TAIR: The *Arabidopsis* information resource
TIGR: The institute for genome research
HTS: High-throughput sequencing
SPL: Squamosa-promoter binding protein-like
YSL3: YELLOW STRIPE like 3
SLK2: SEUSS-like 2
ta-siRNA: *Trans*-acting small interfering RNA
GEO: Gene Expression Omnibus
CSRDB: Cereal Small RNAs Database
RPM: Reads per million
t-plot: Target plot
UTR: Untranslated region
WT: Wild type.
