# Supplementary figures and images for: Introns targeted by plant microRNAs: a possible novel mechanism of gene regulation
Source: Rice (N Y). 2013 Apr 15;6:8. doi: 10.1186/1939-8433-6-8 (PMC4883735; doi:10.1186/1939-8433-6-8)

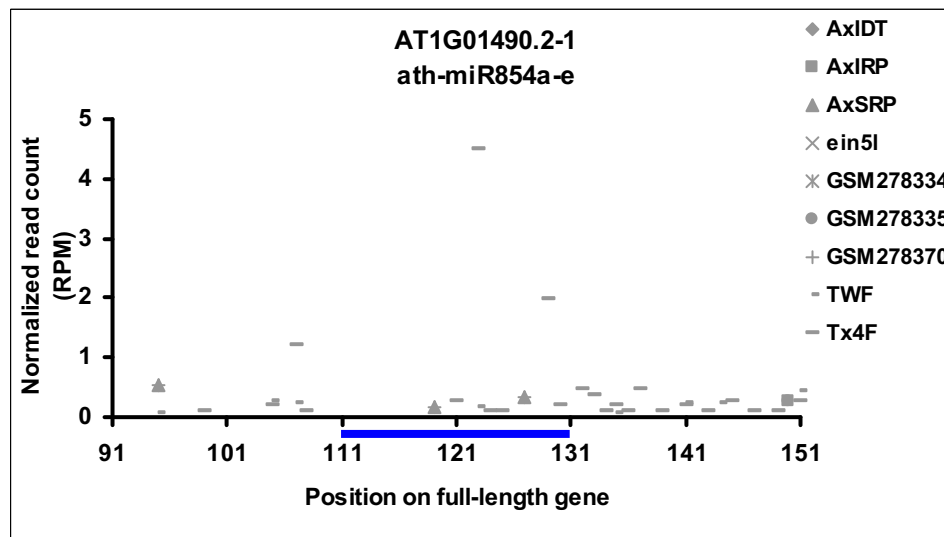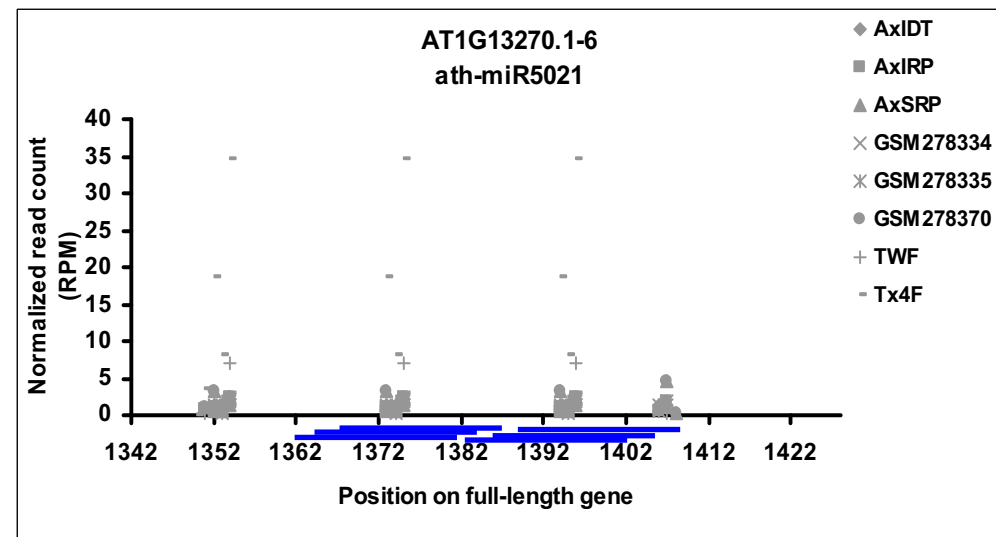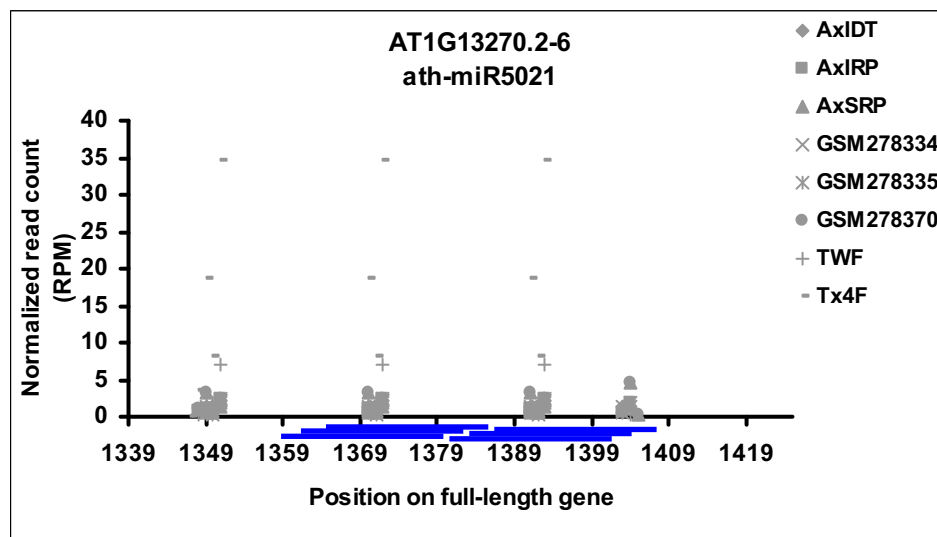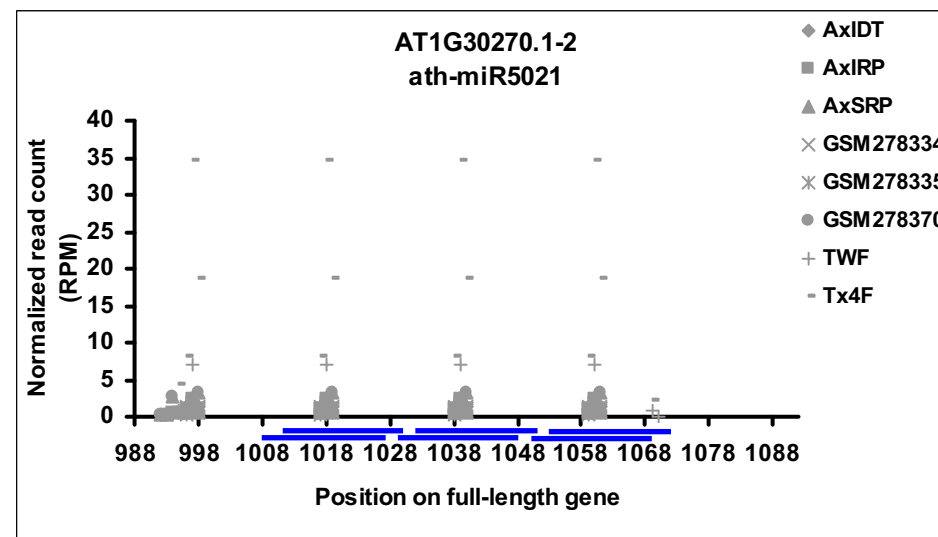

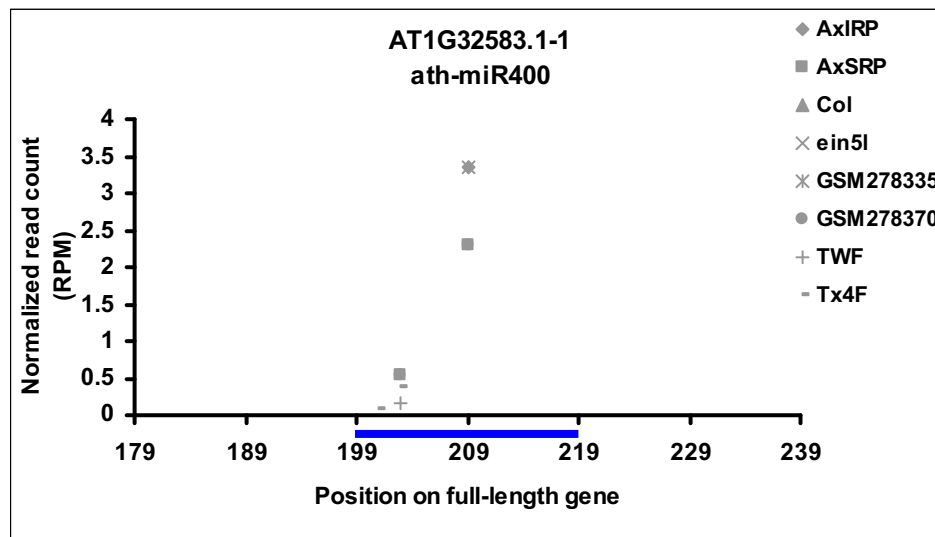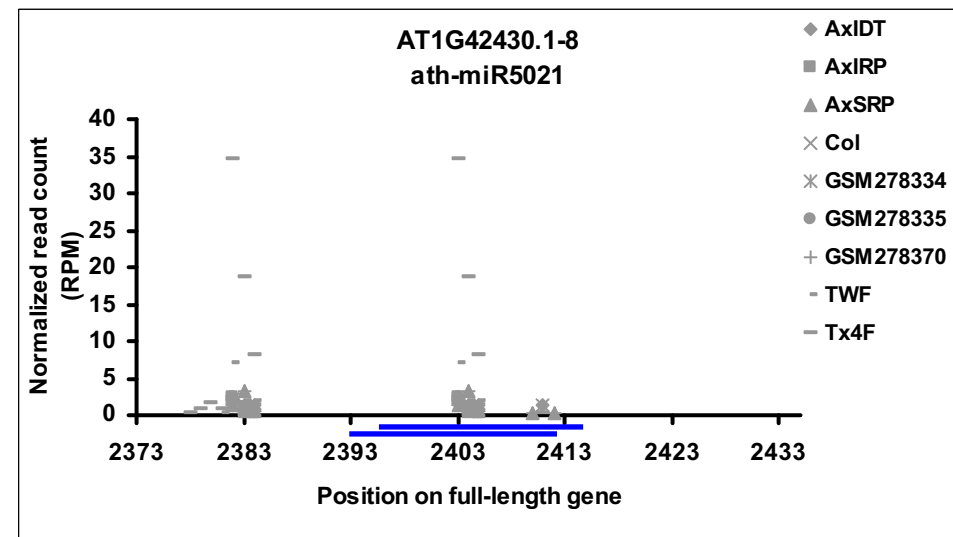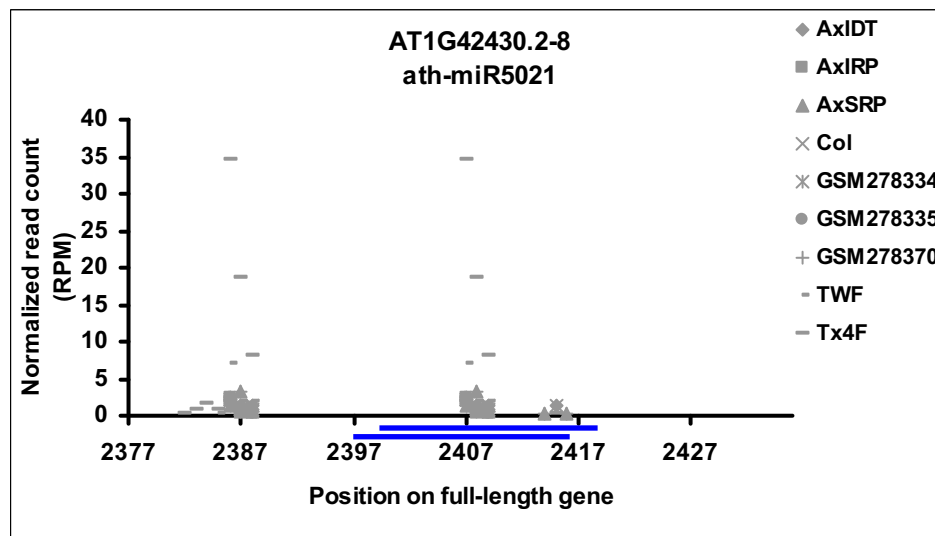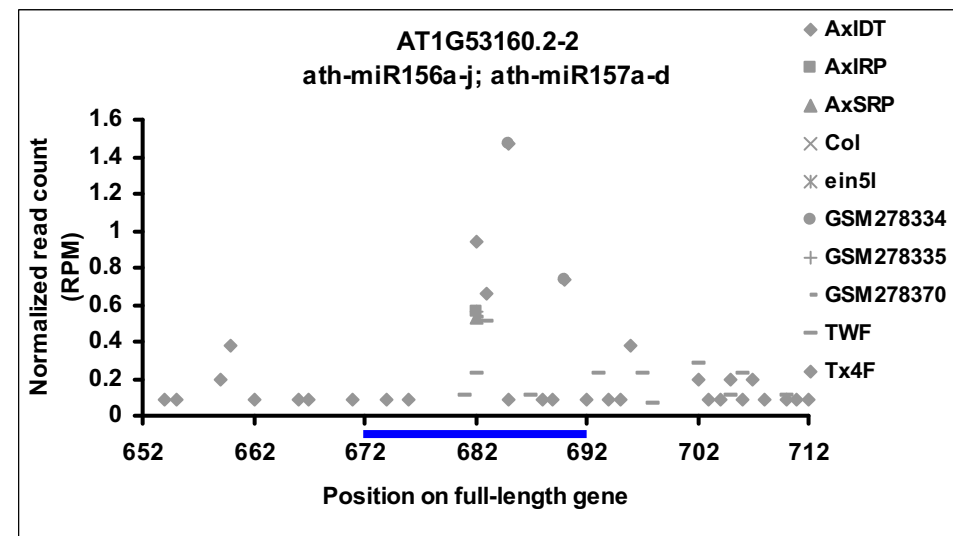

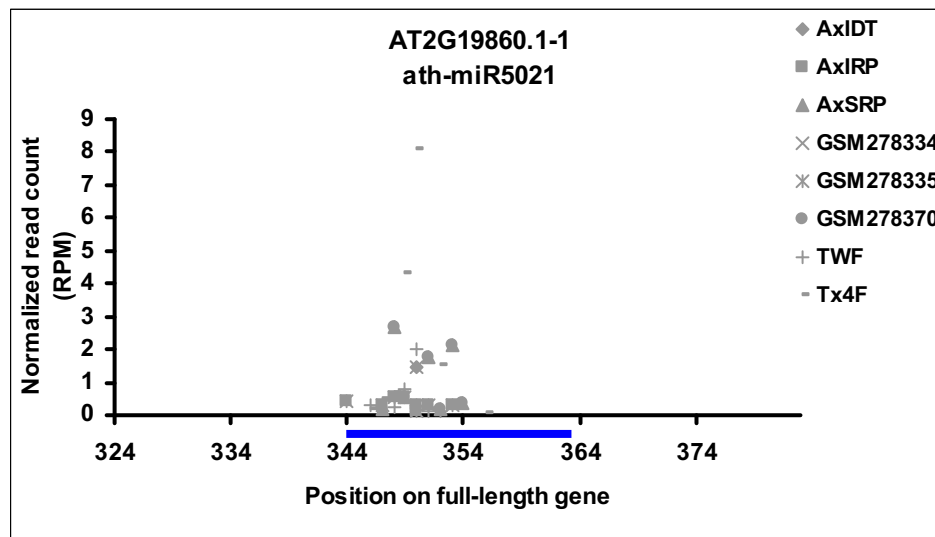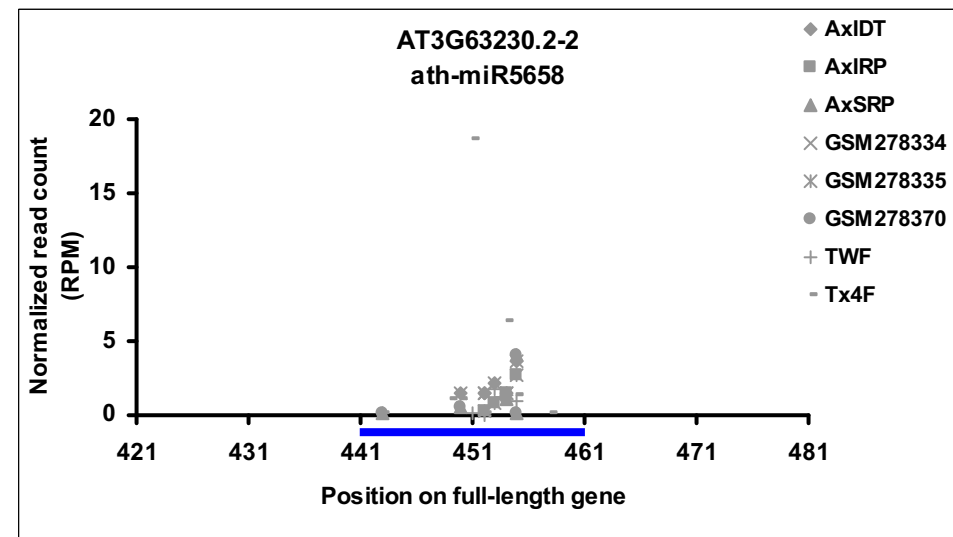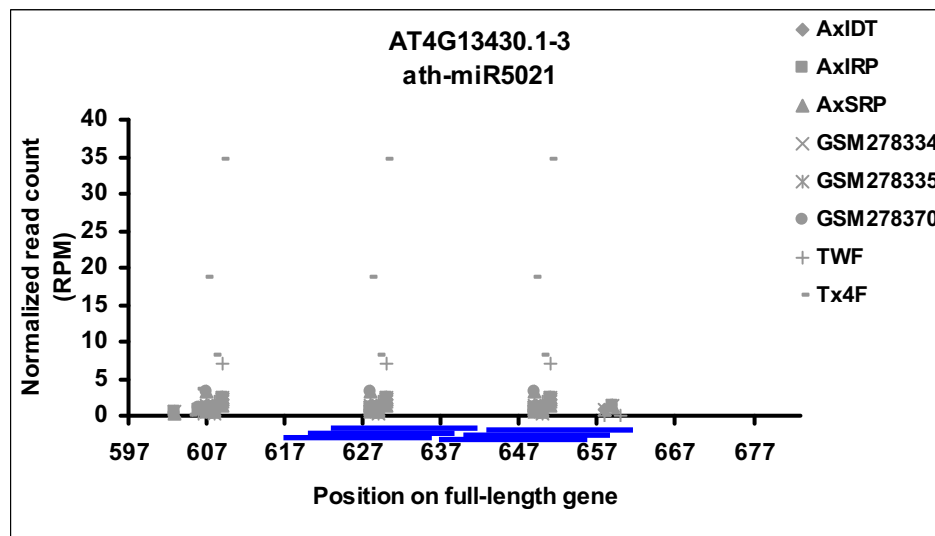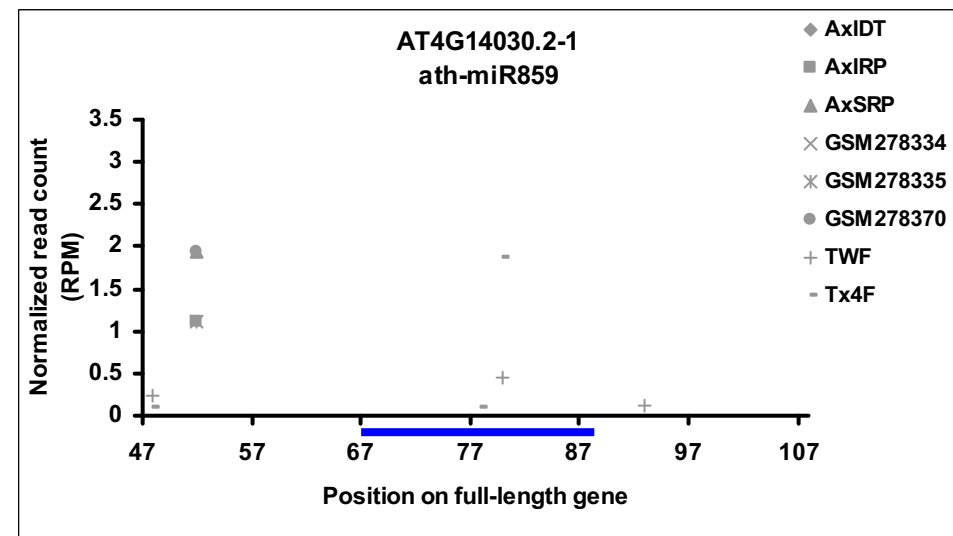

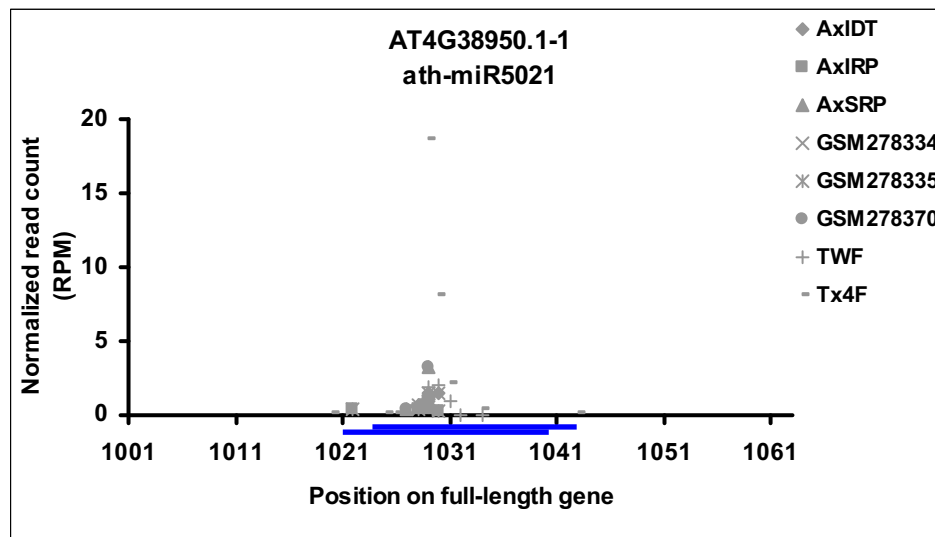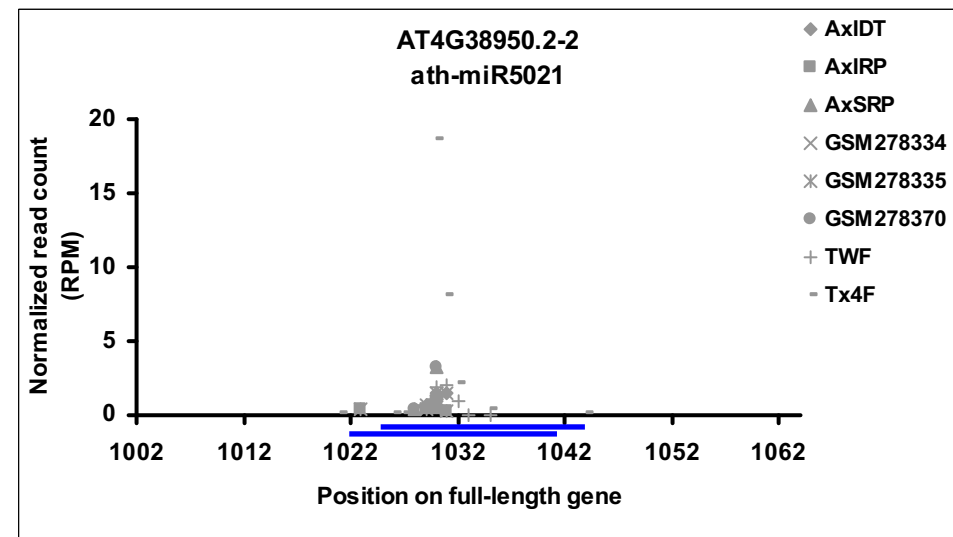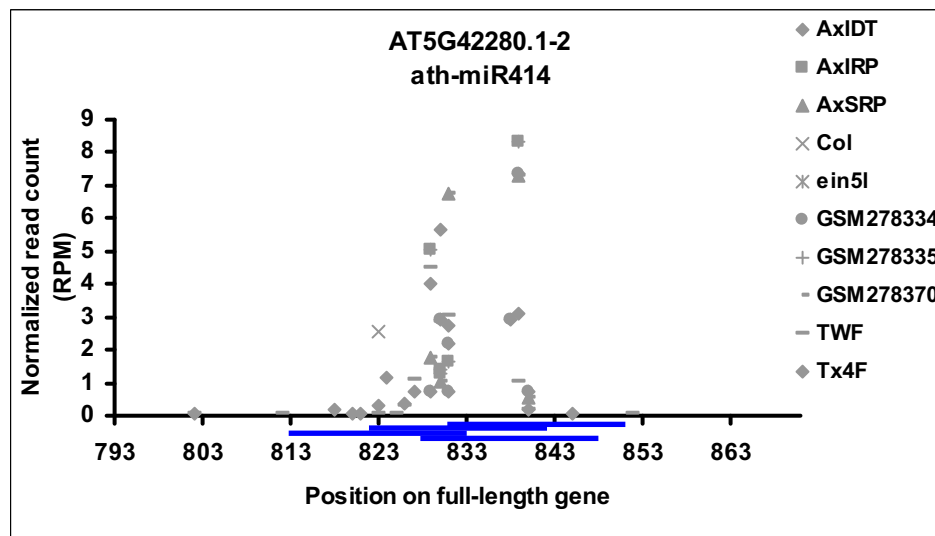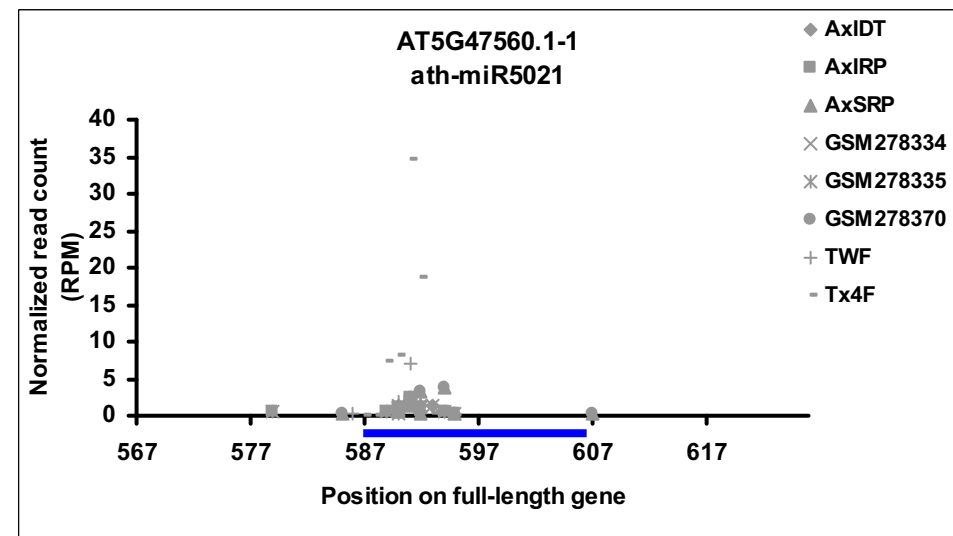

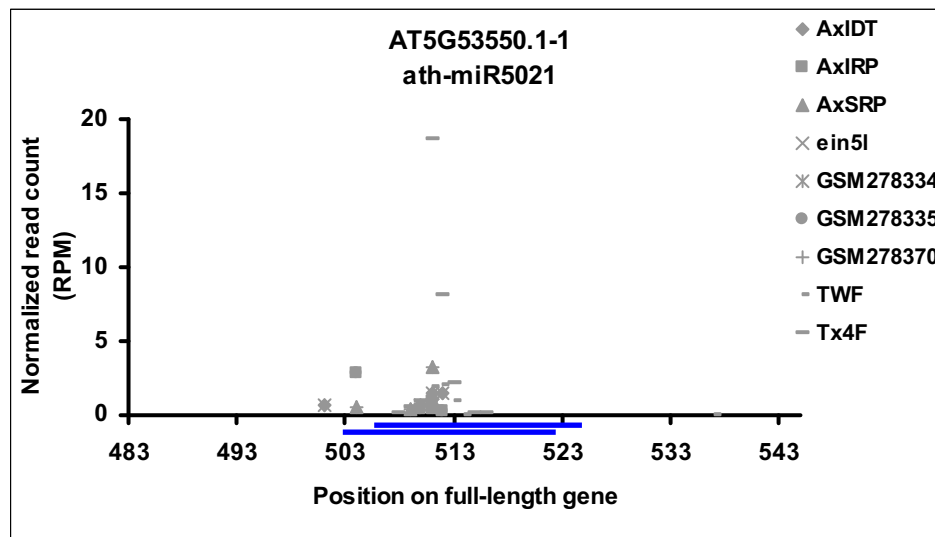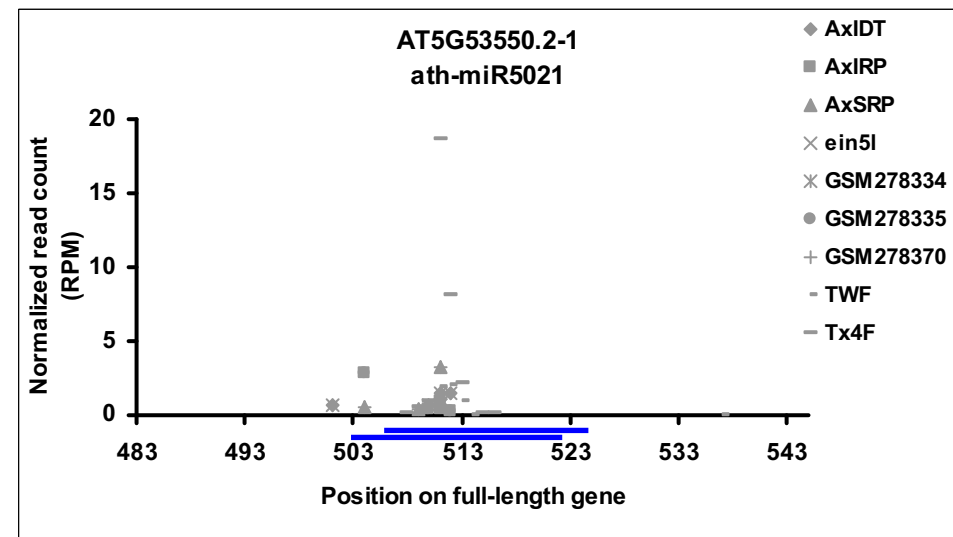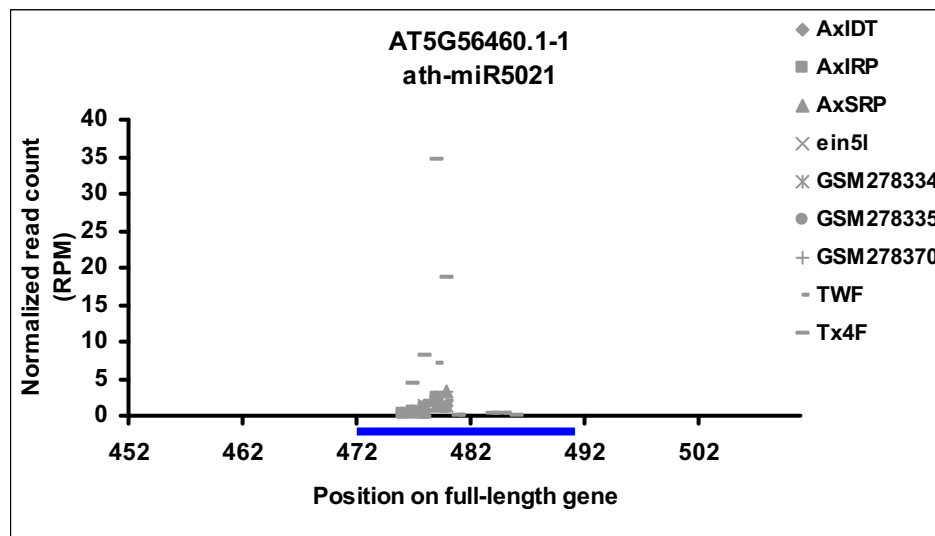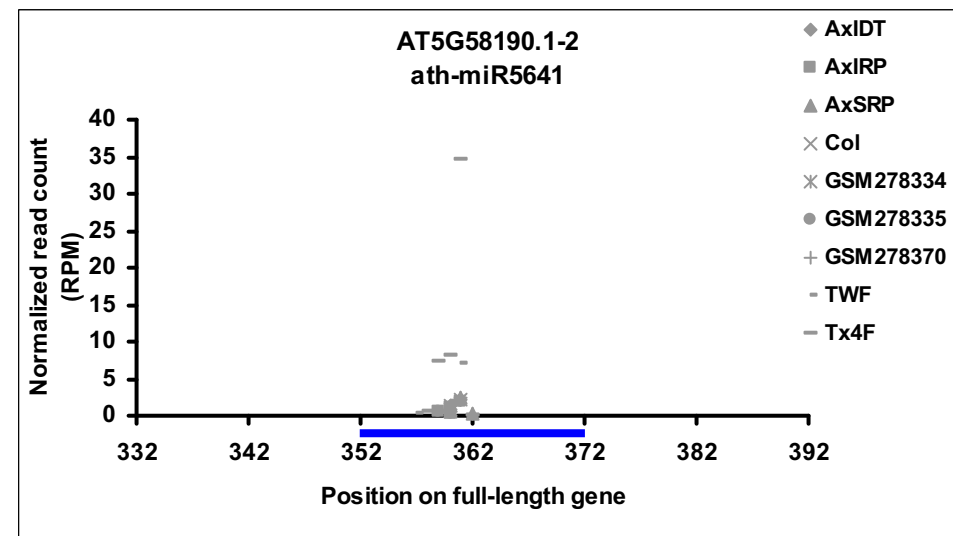

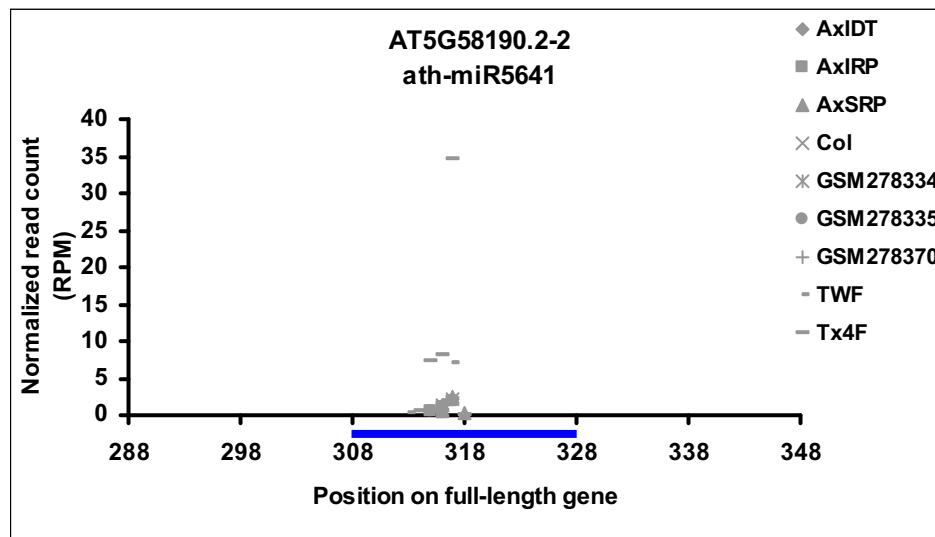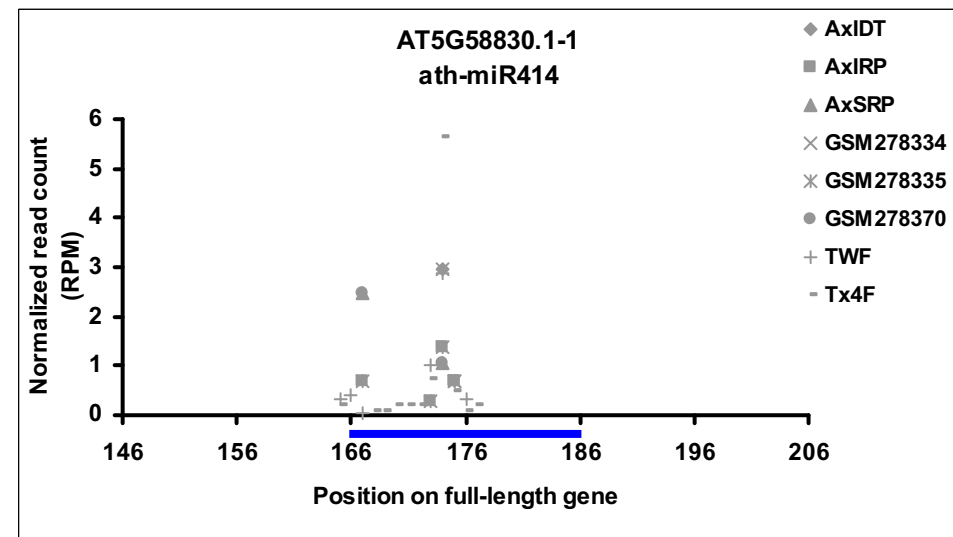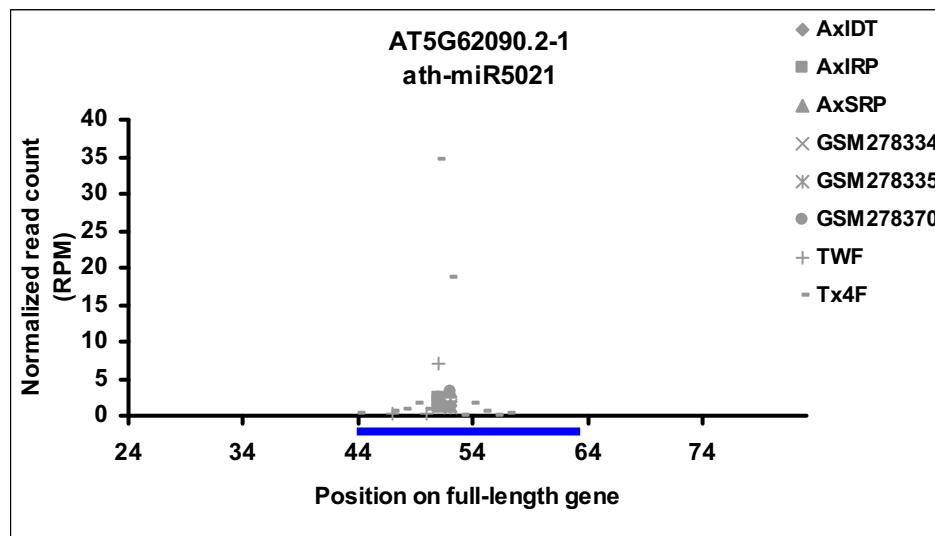

Supplement: Supplementary file 1 — Additional file 1: Figure S1: Target plot-based validation of the microRNA—intron interactions in Arabidopsis. Only the miRNA binding sites (indicated by blue horizontal lines) surrounded by 20-nt sequences at both ends were shown. (PDF 58 KB) [file 12284_2012_45_MOESM1_ESM.pdf]

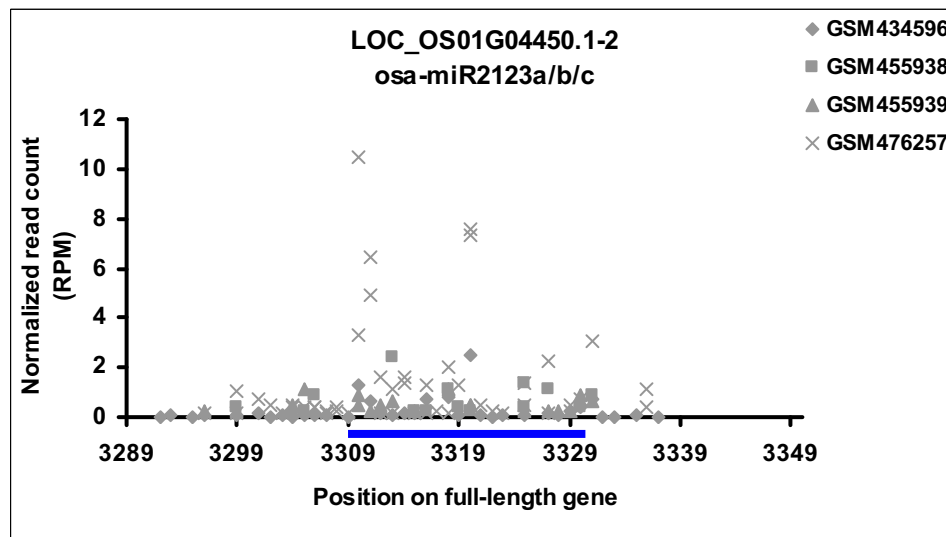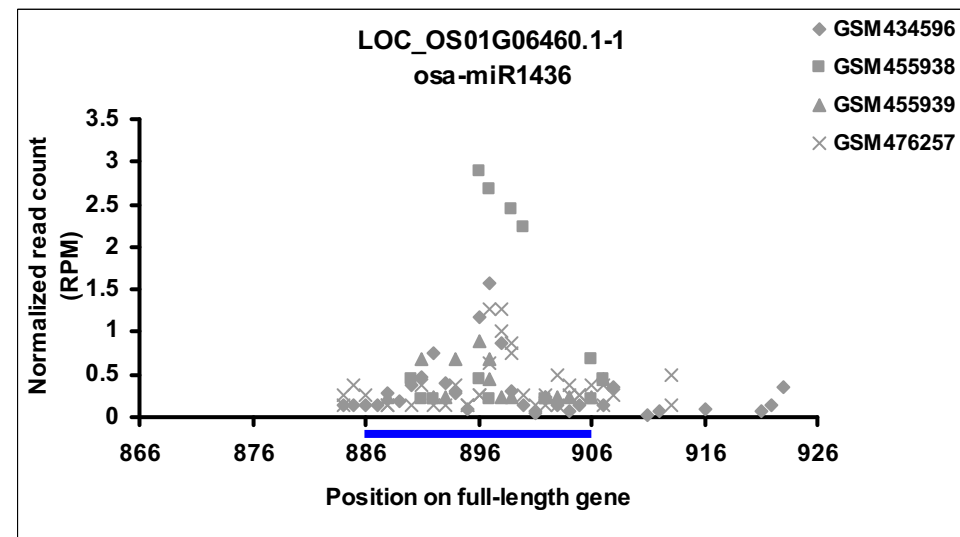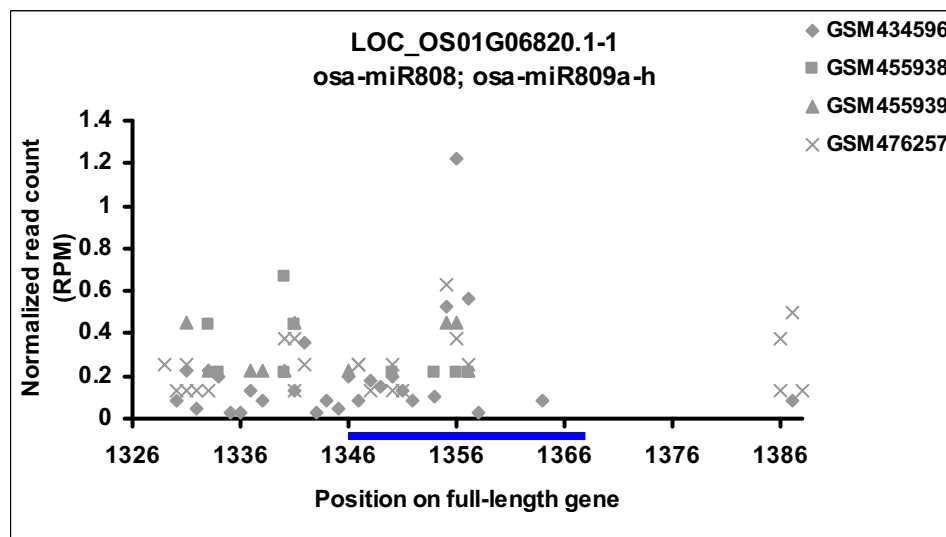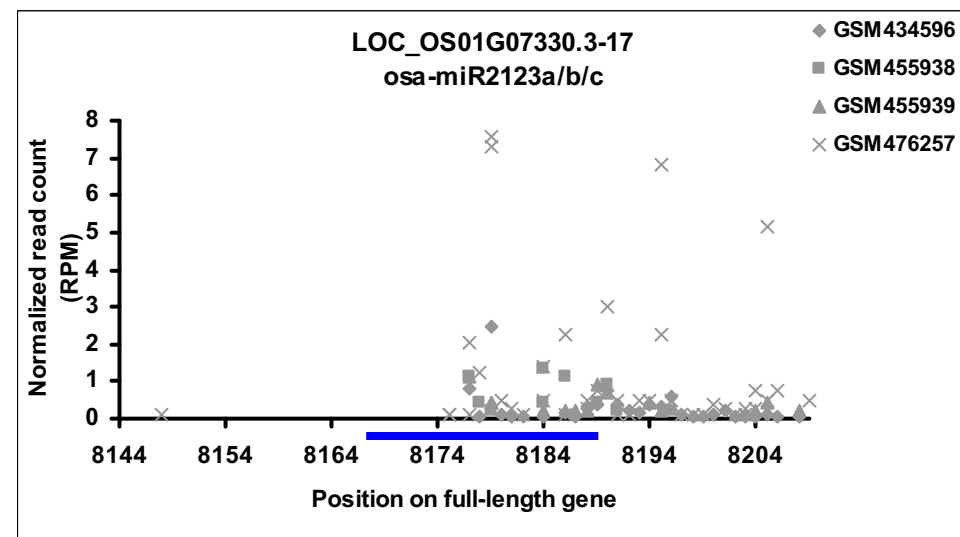

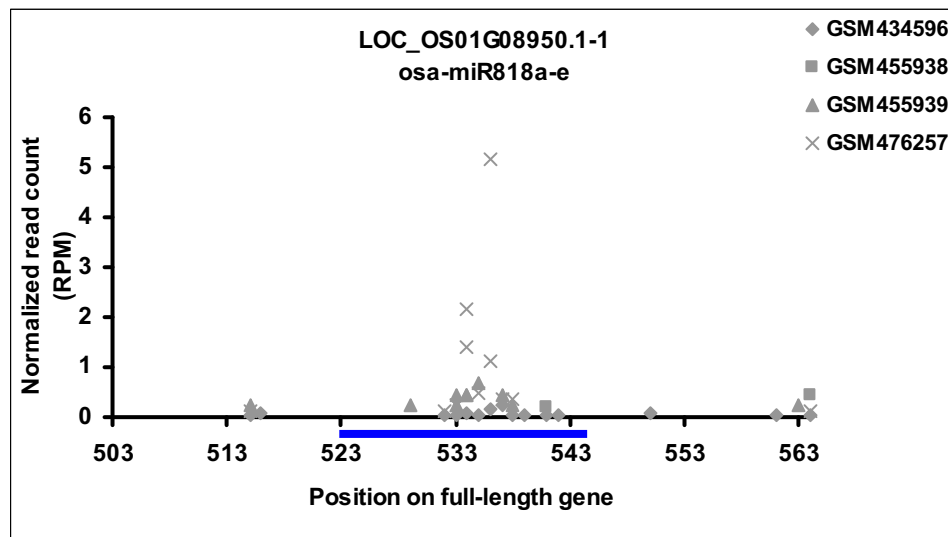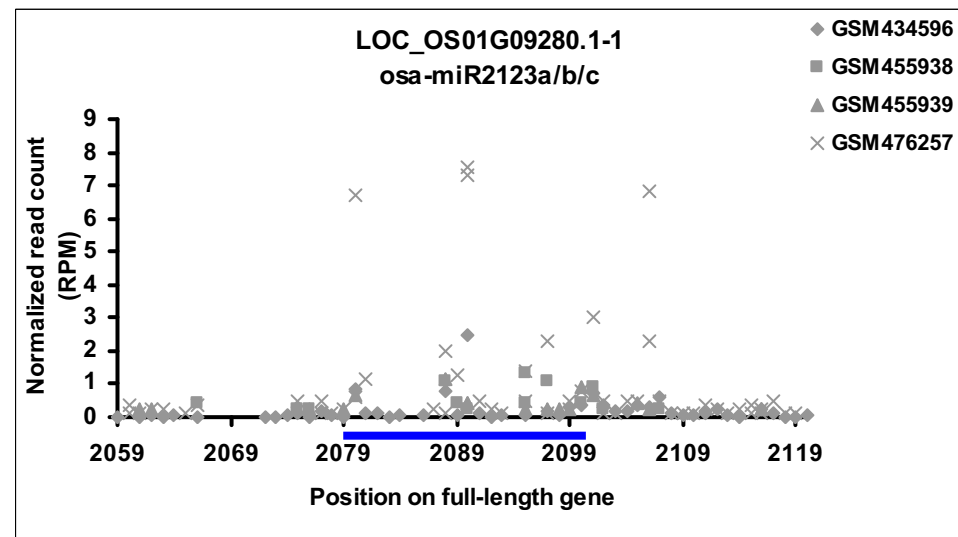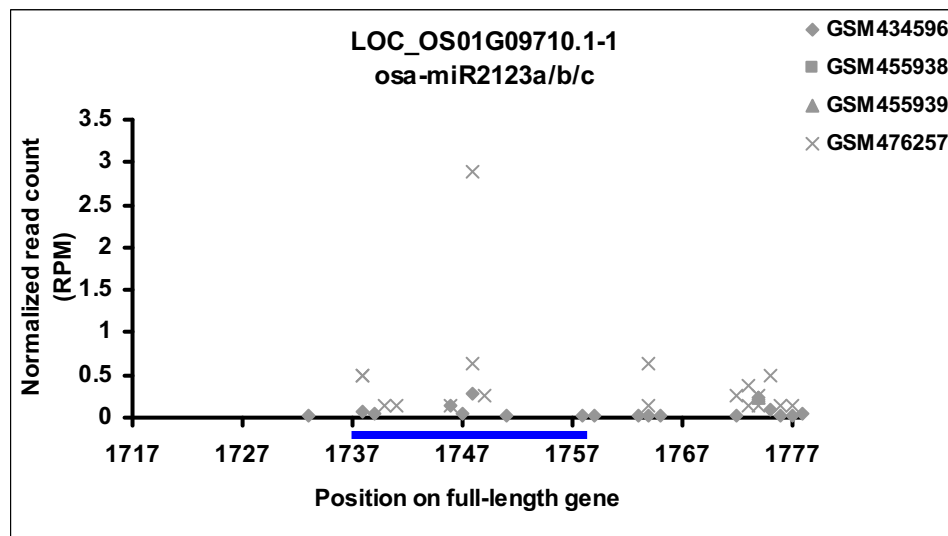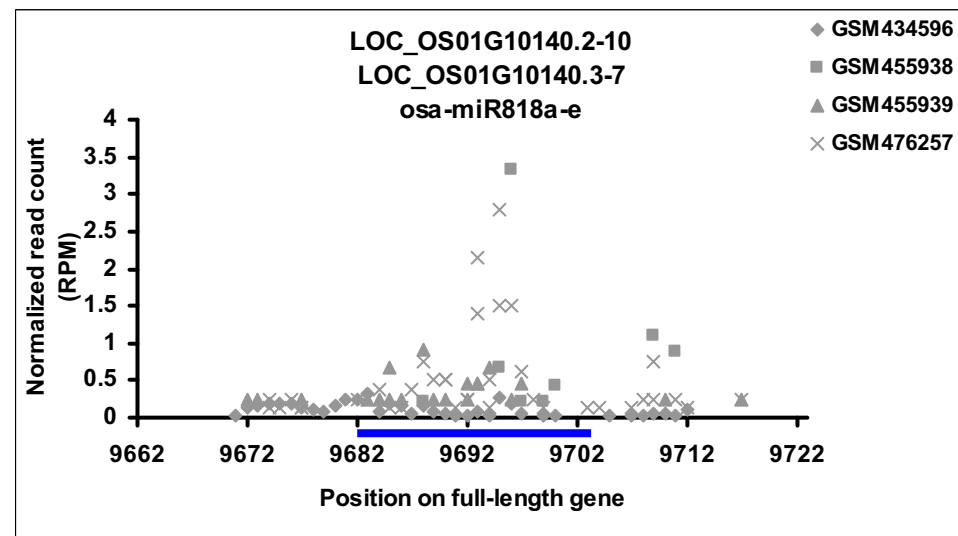

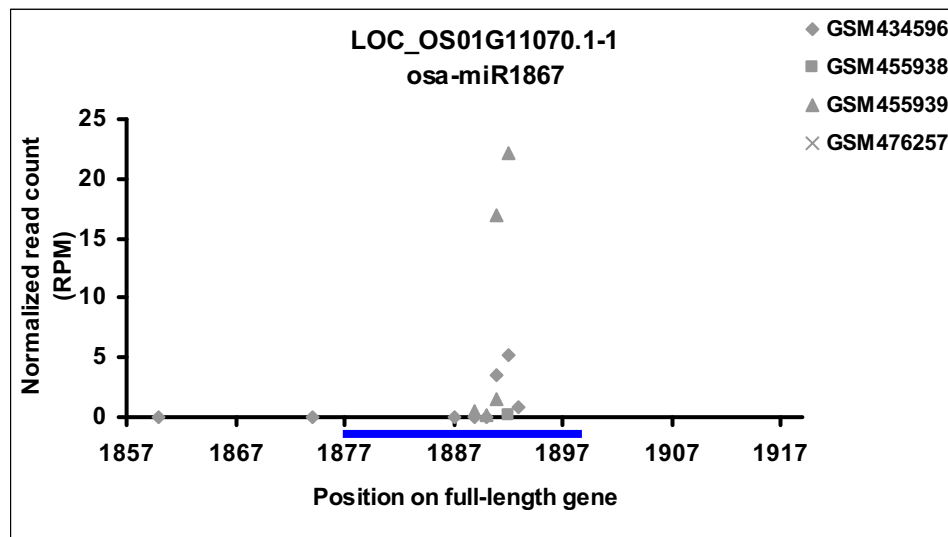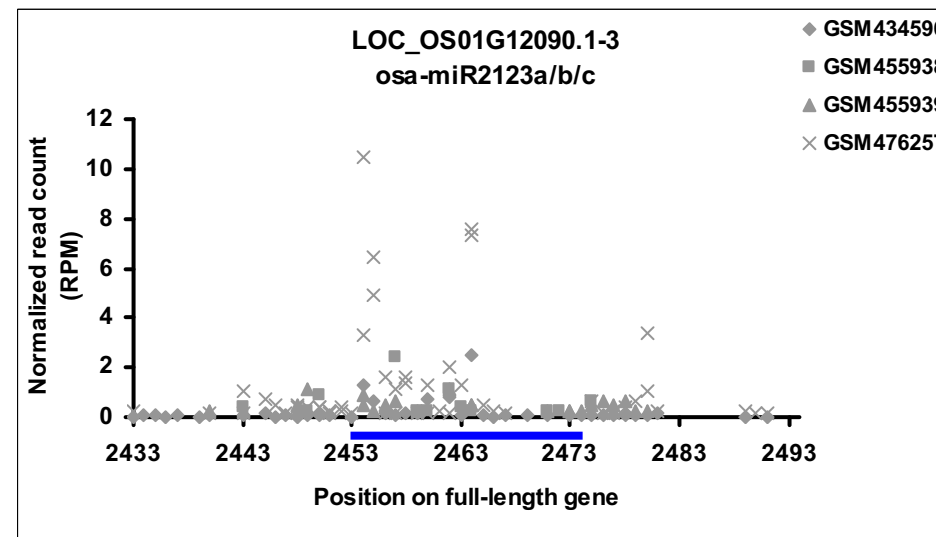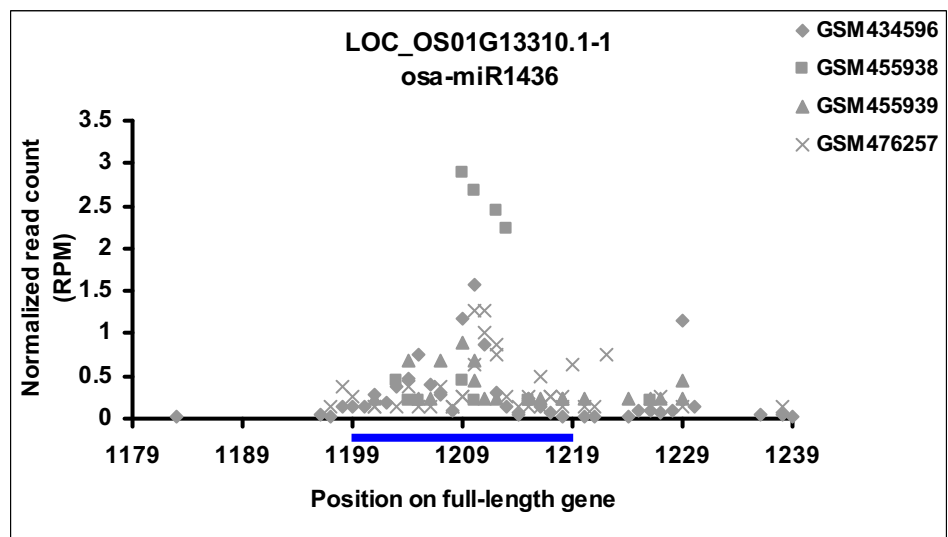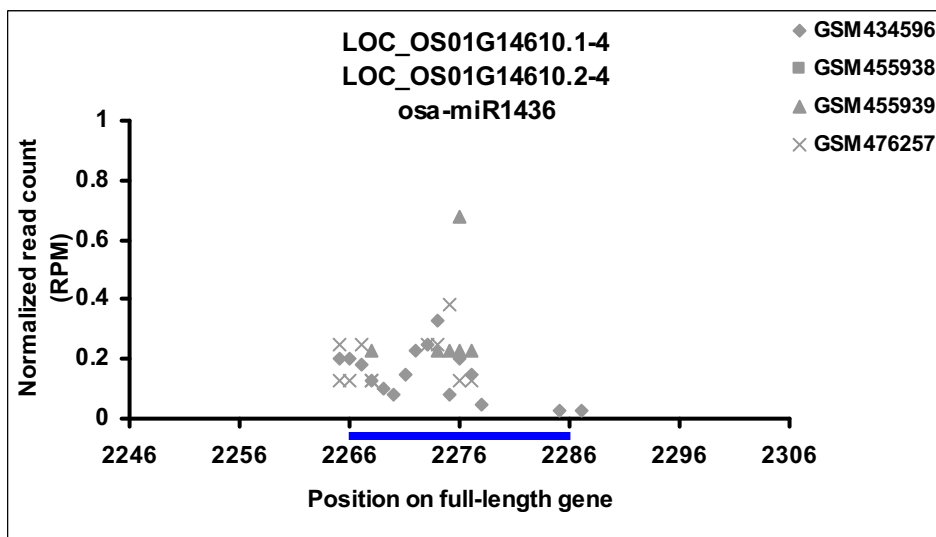

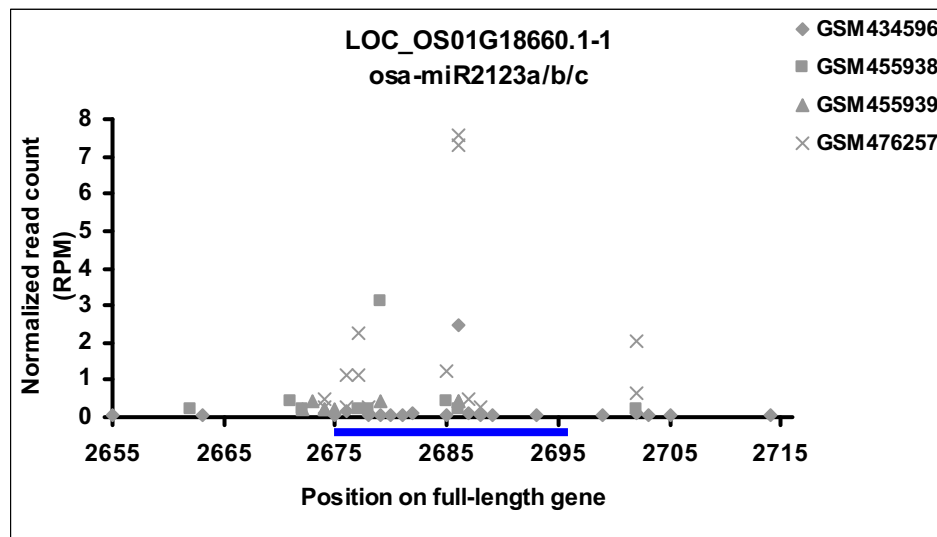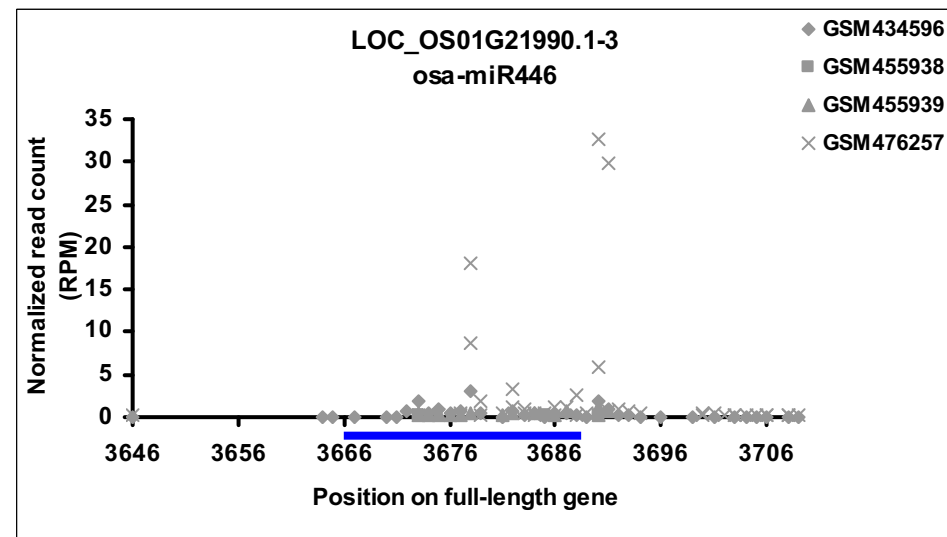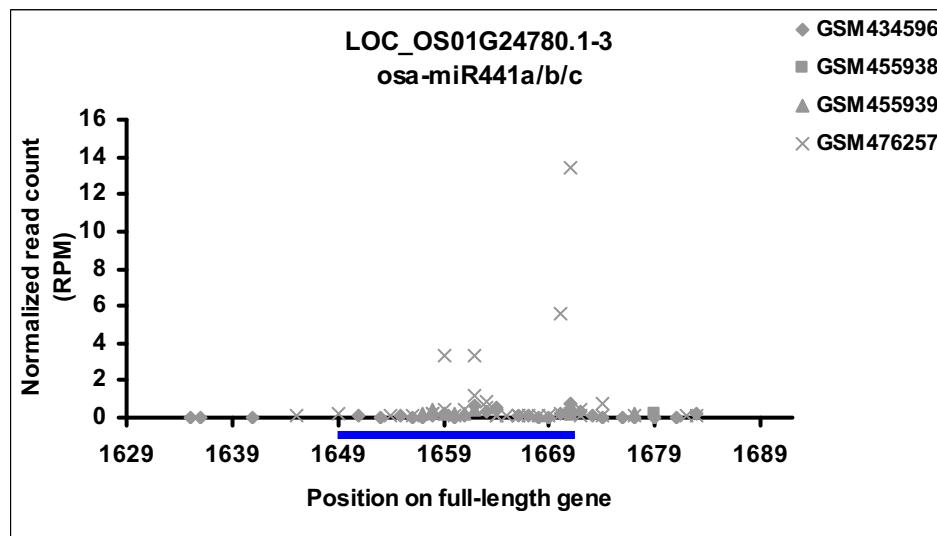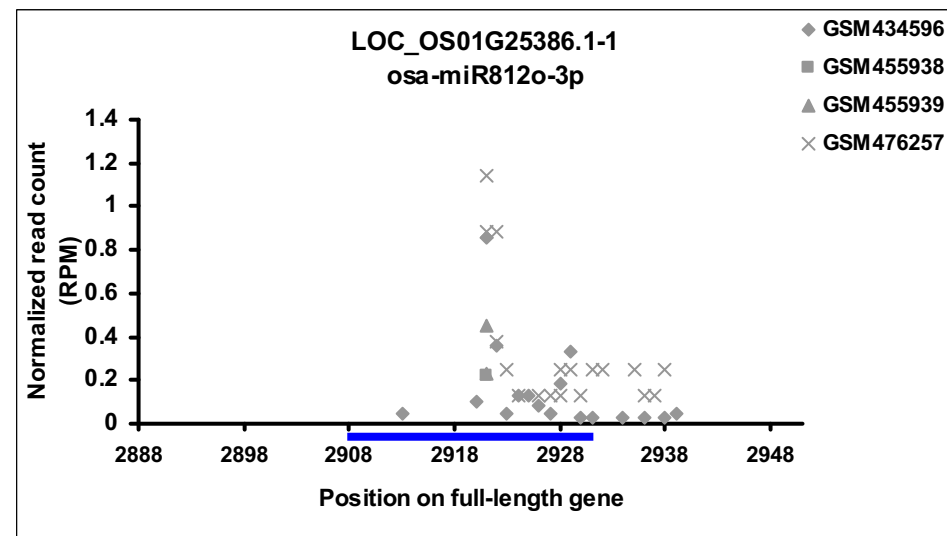

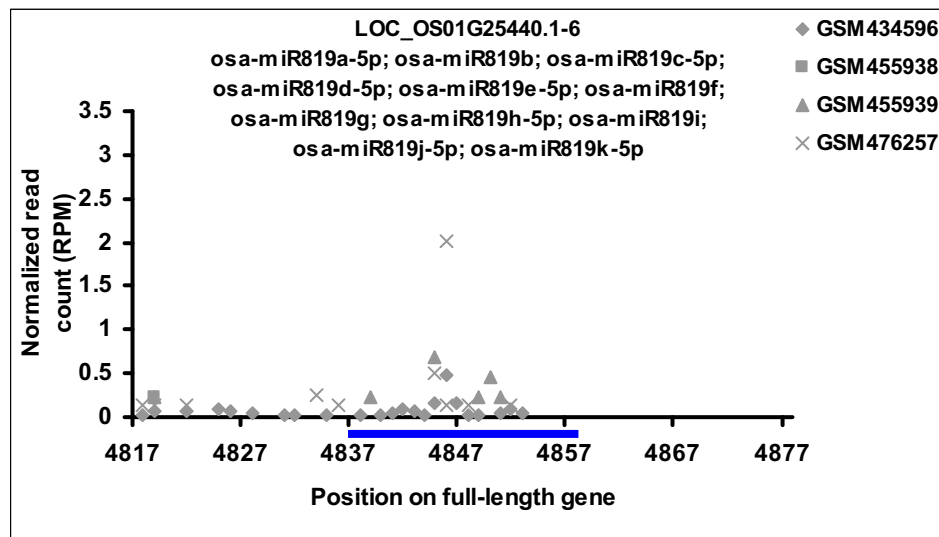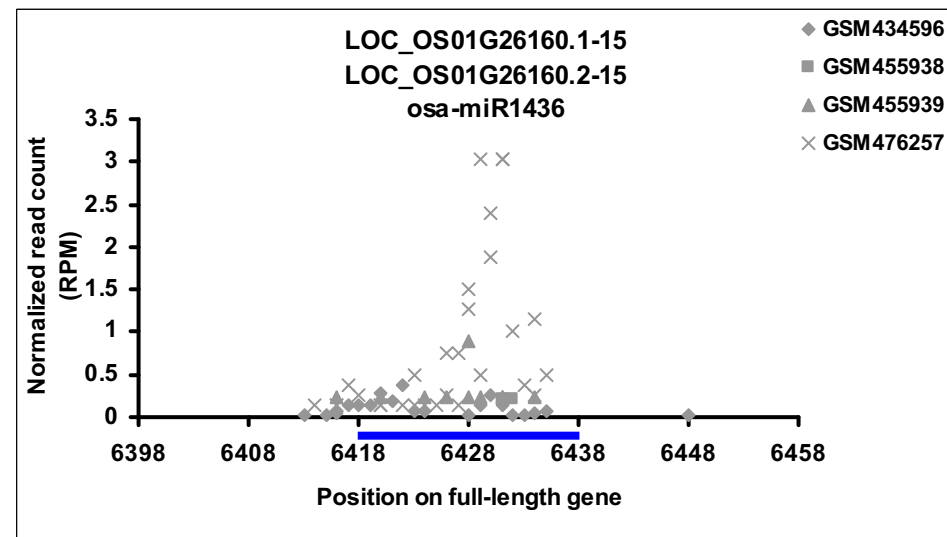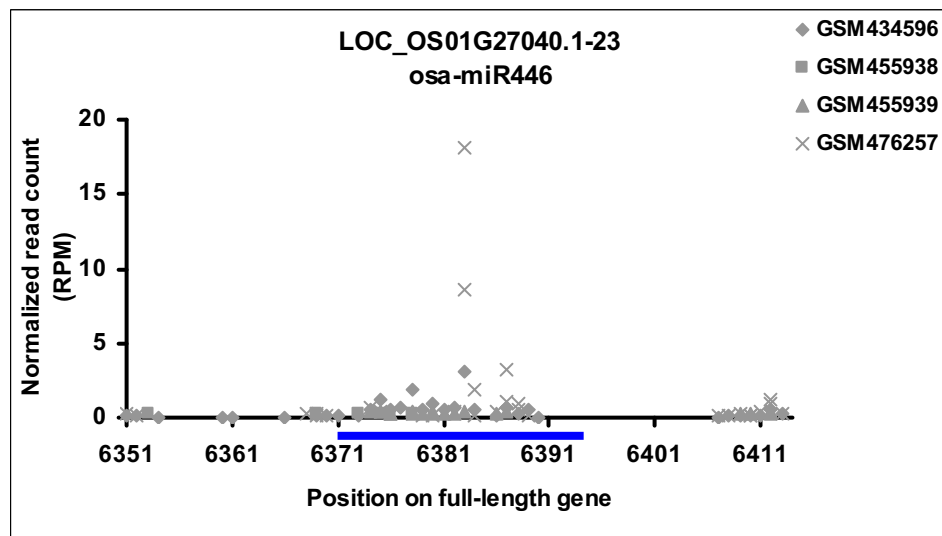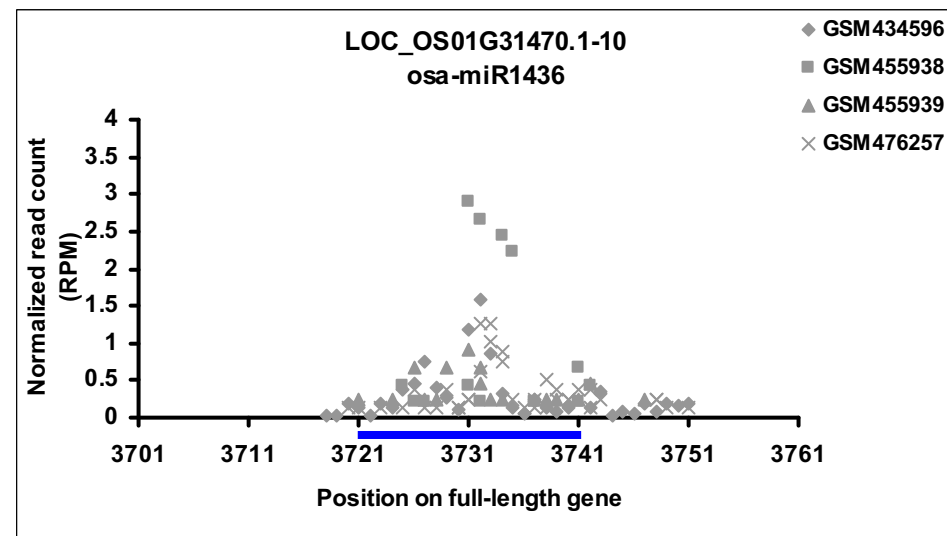

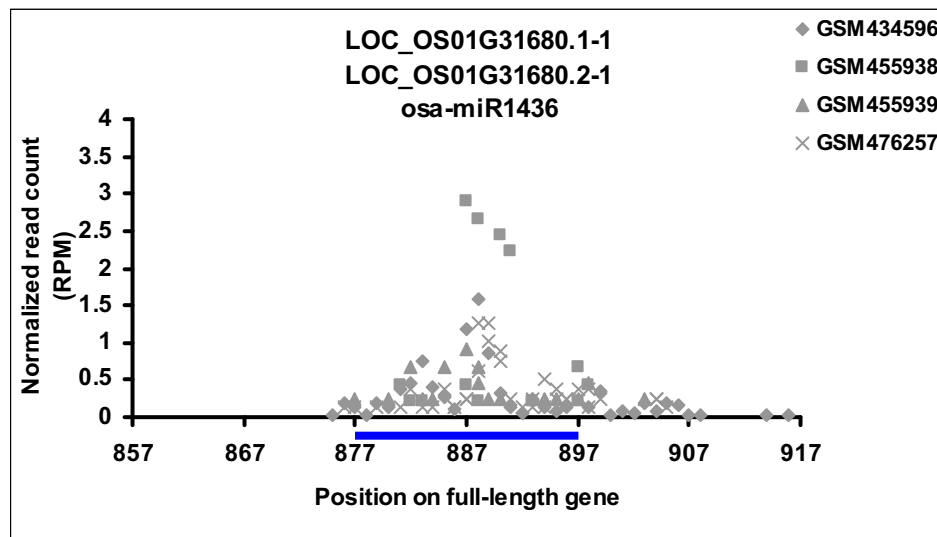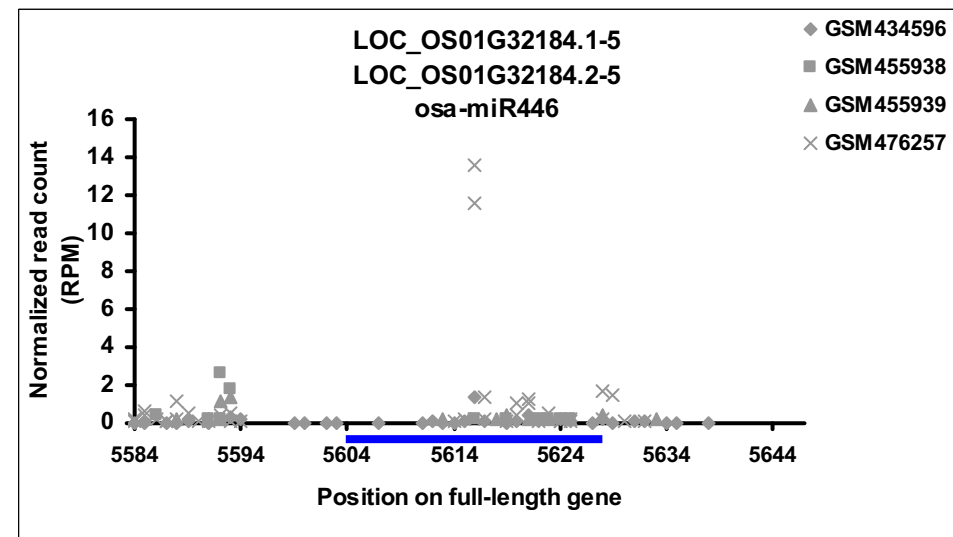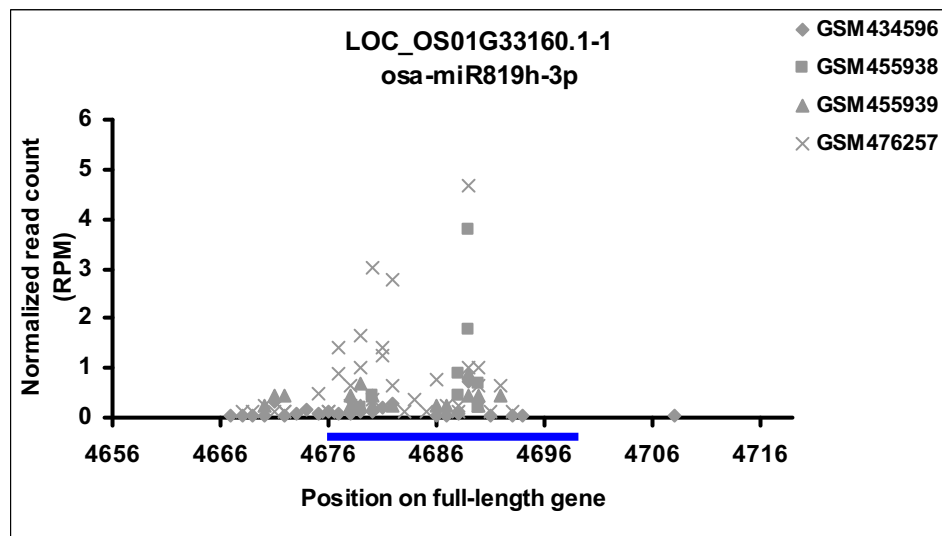

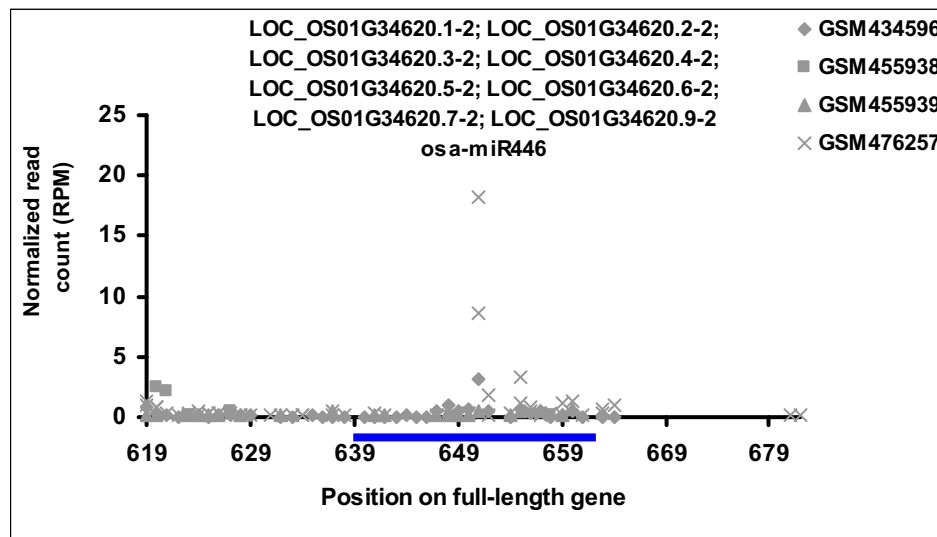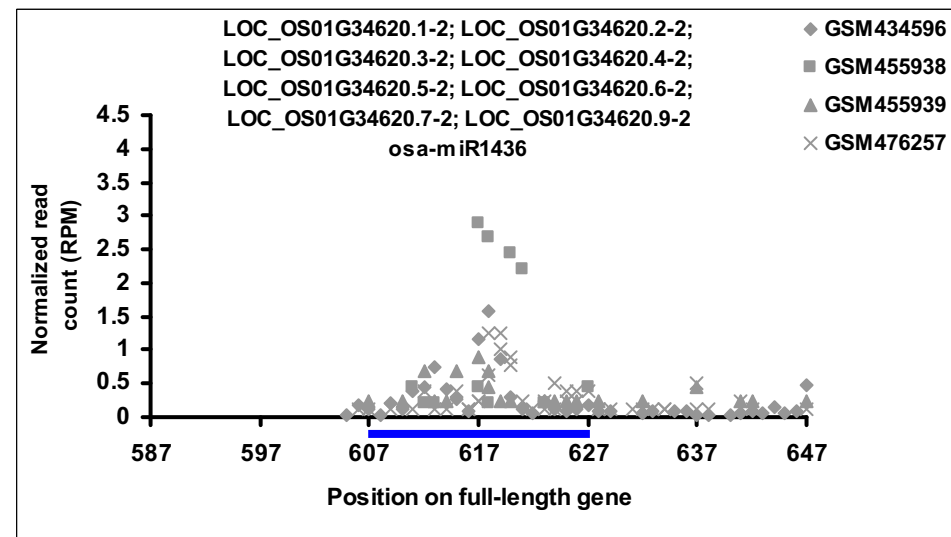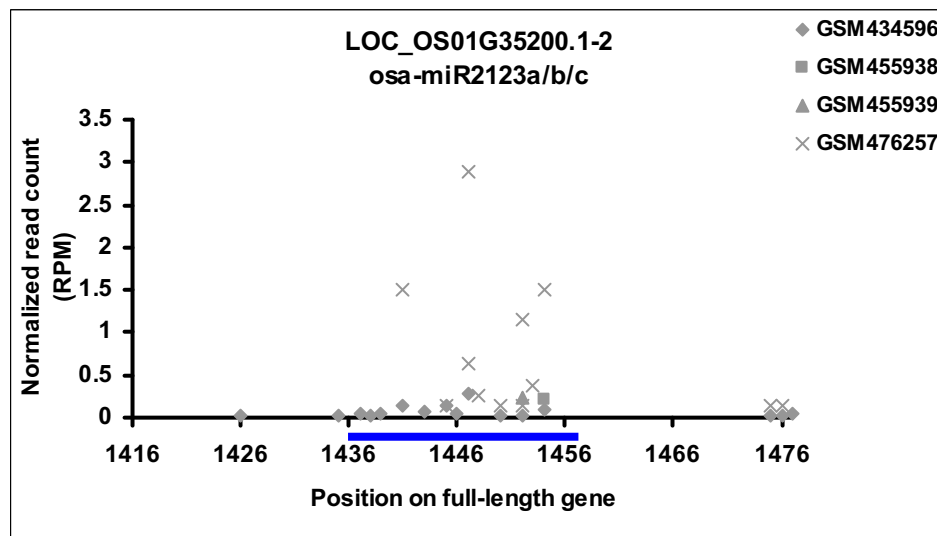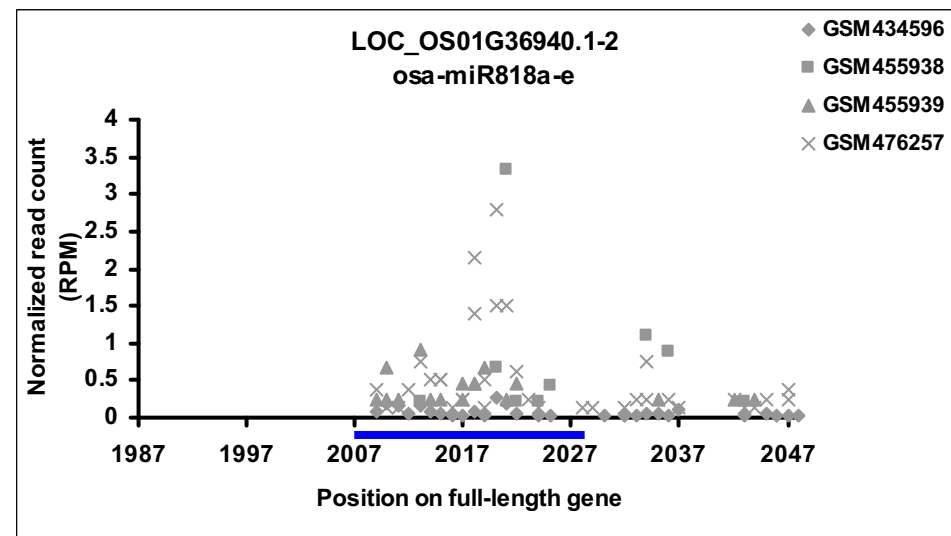

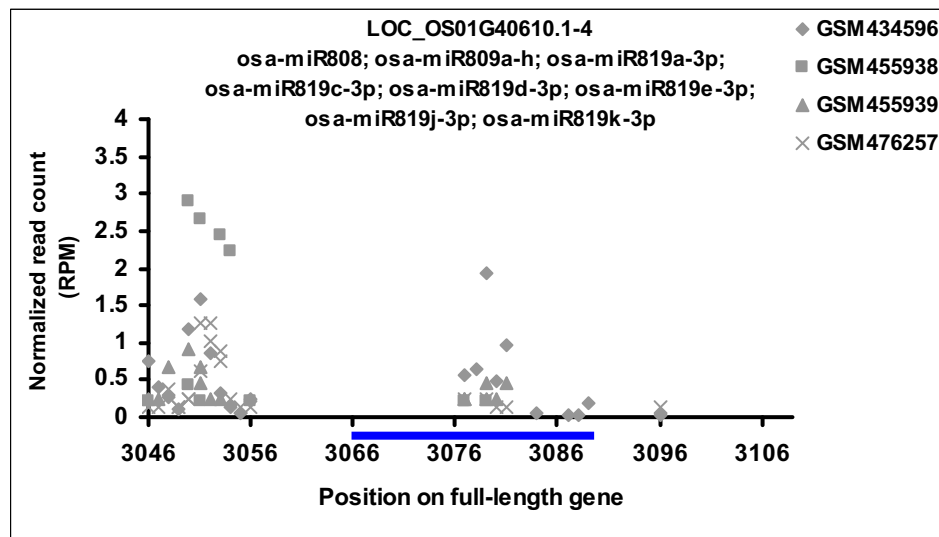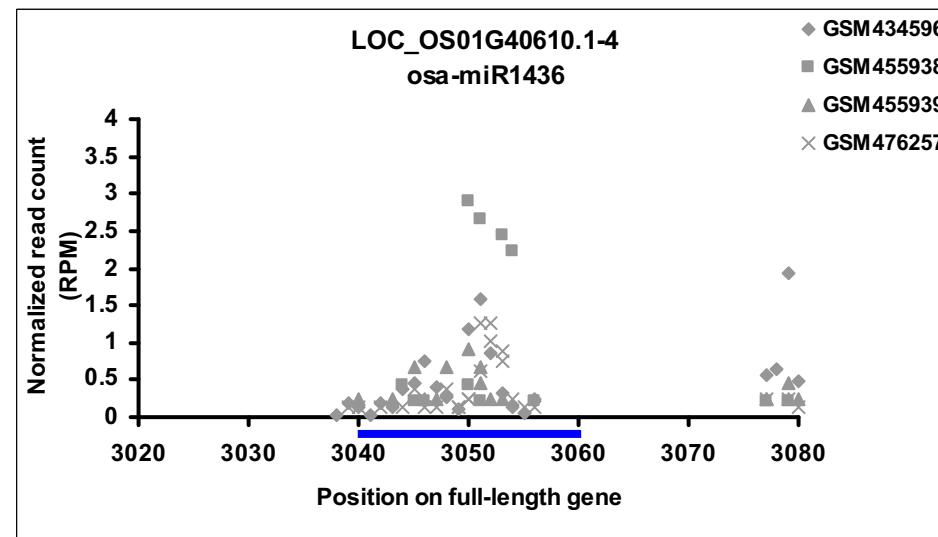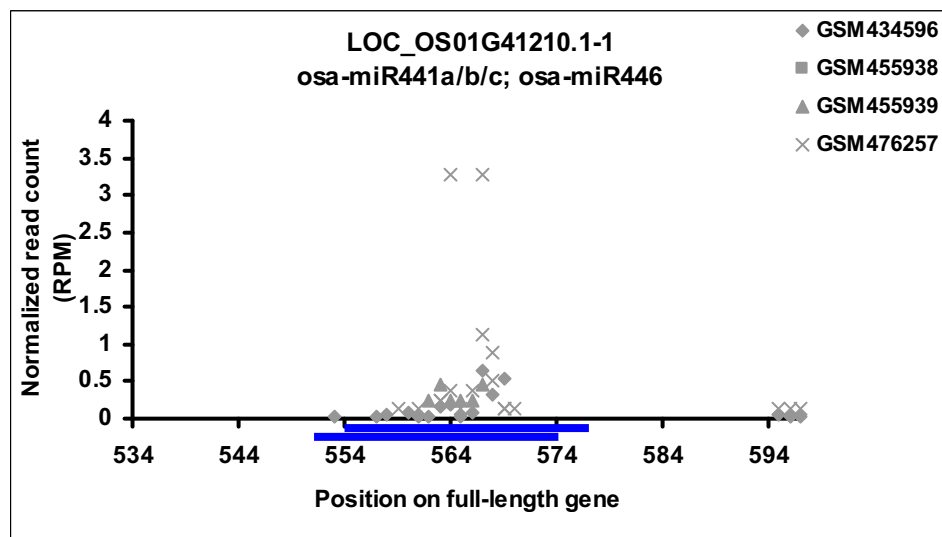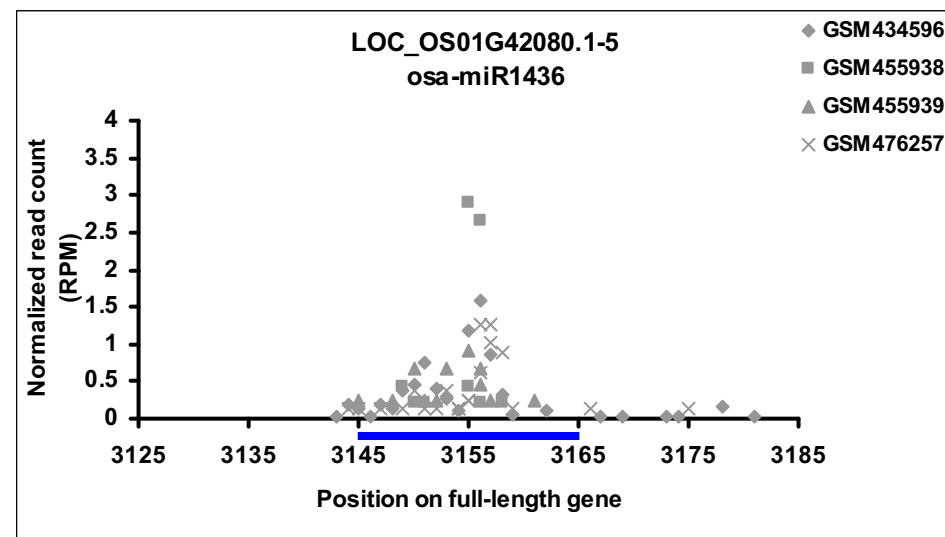

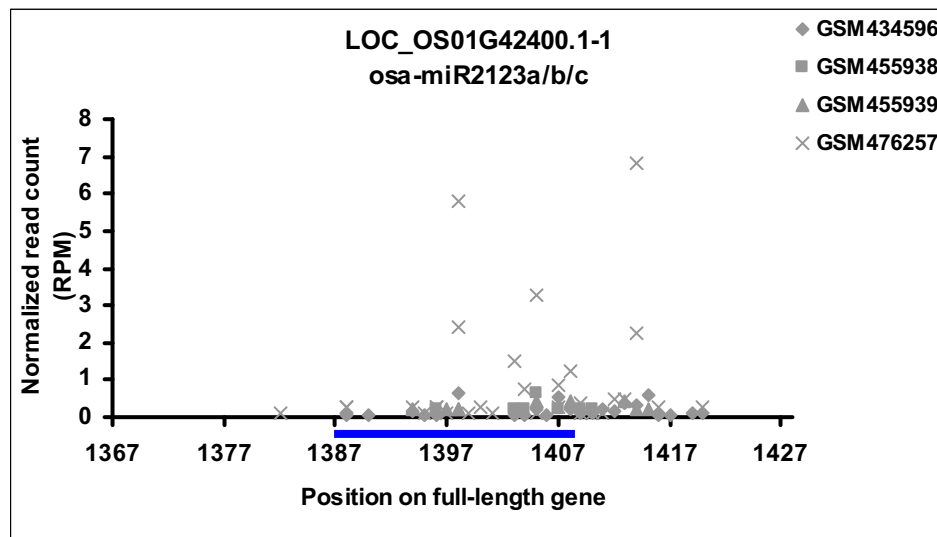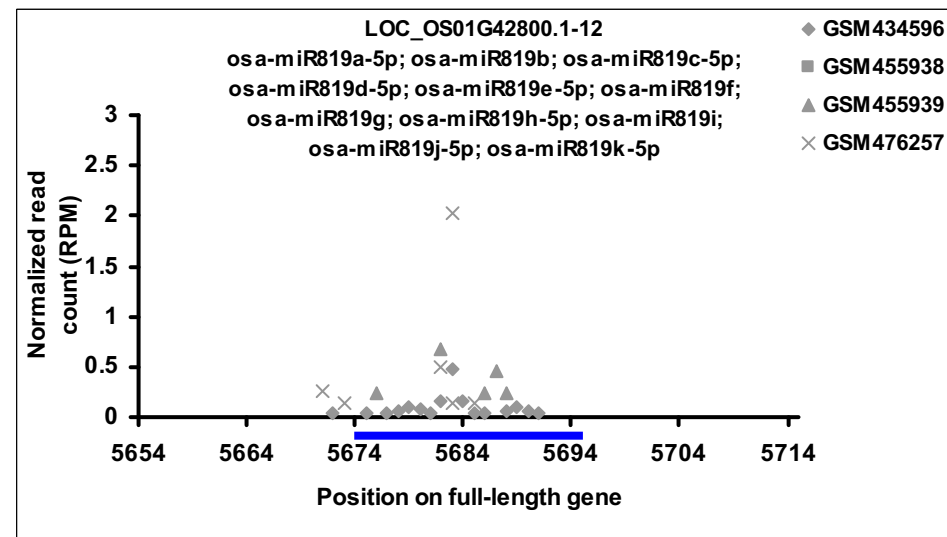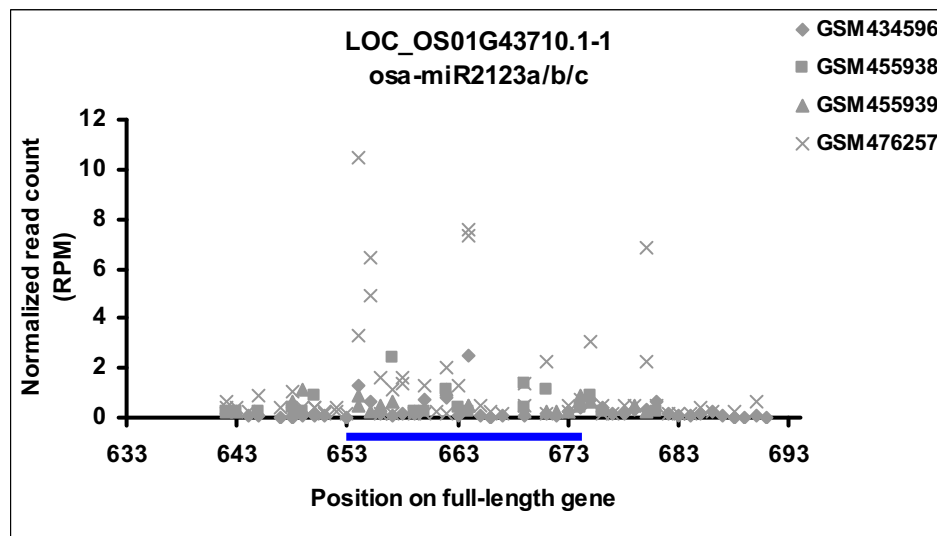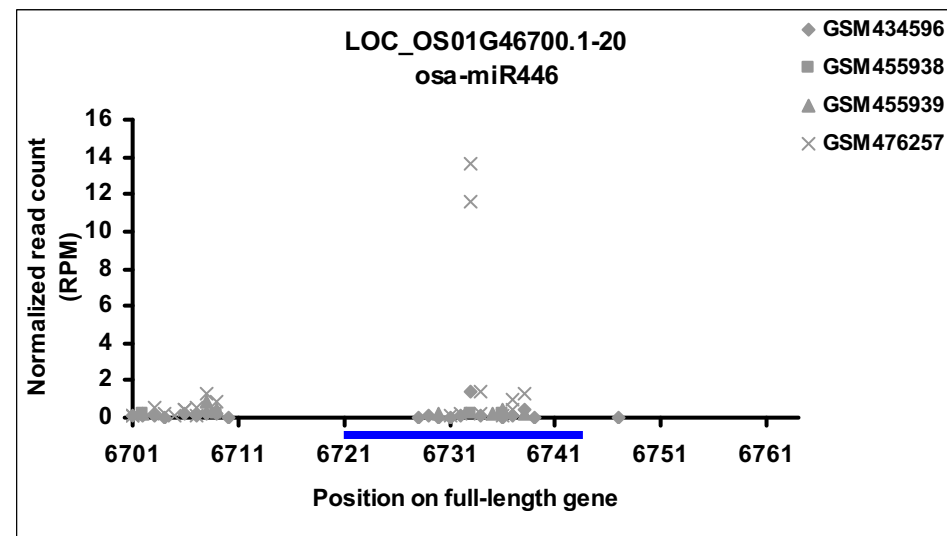

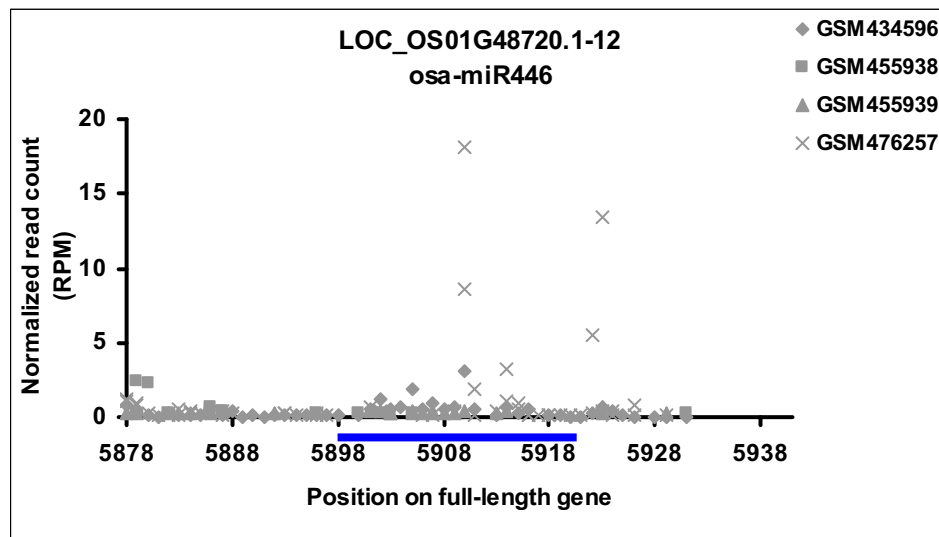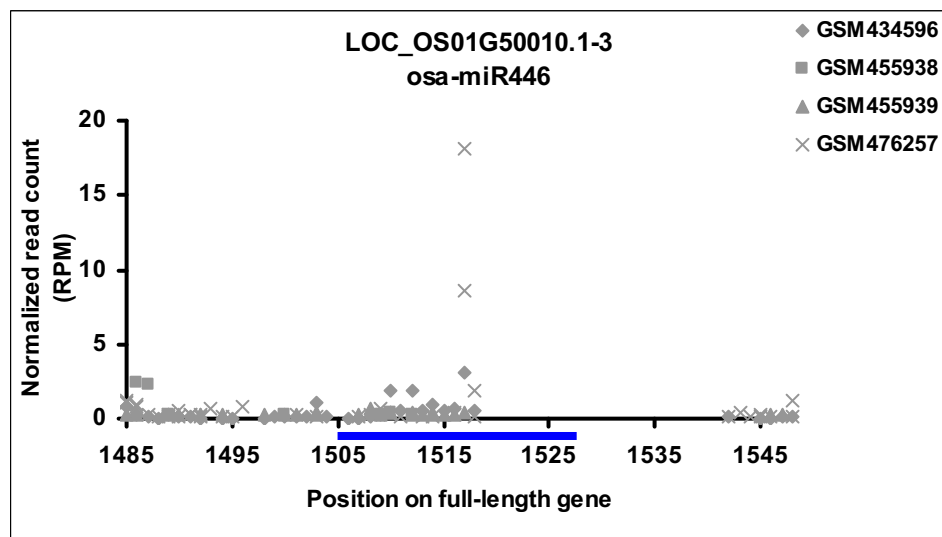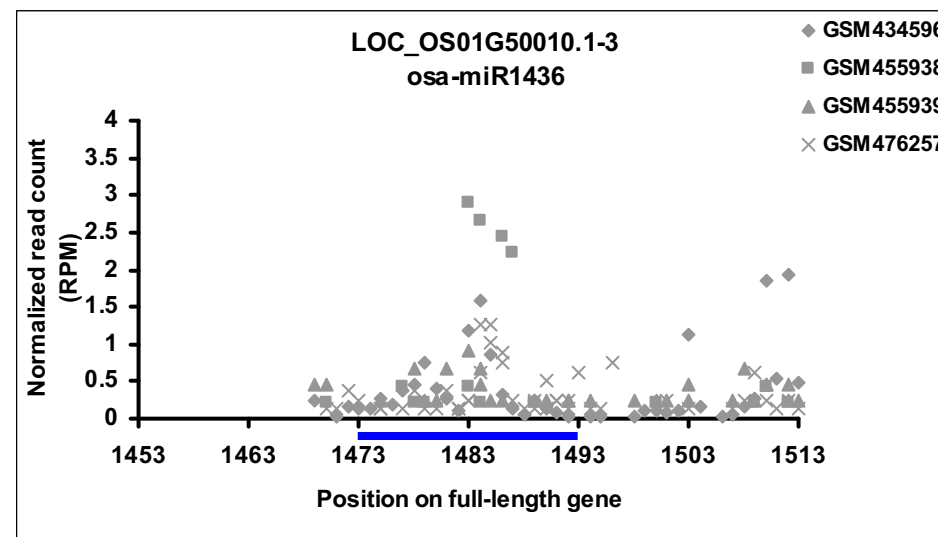

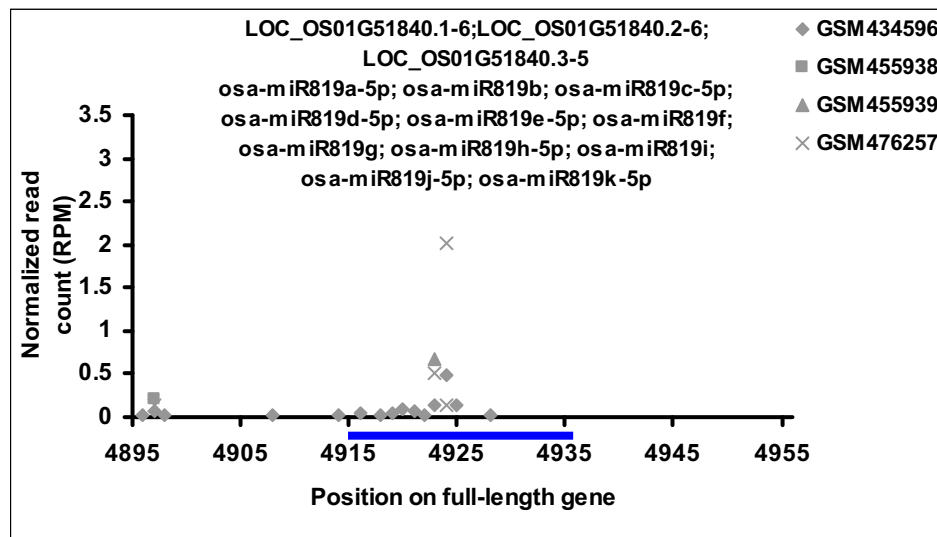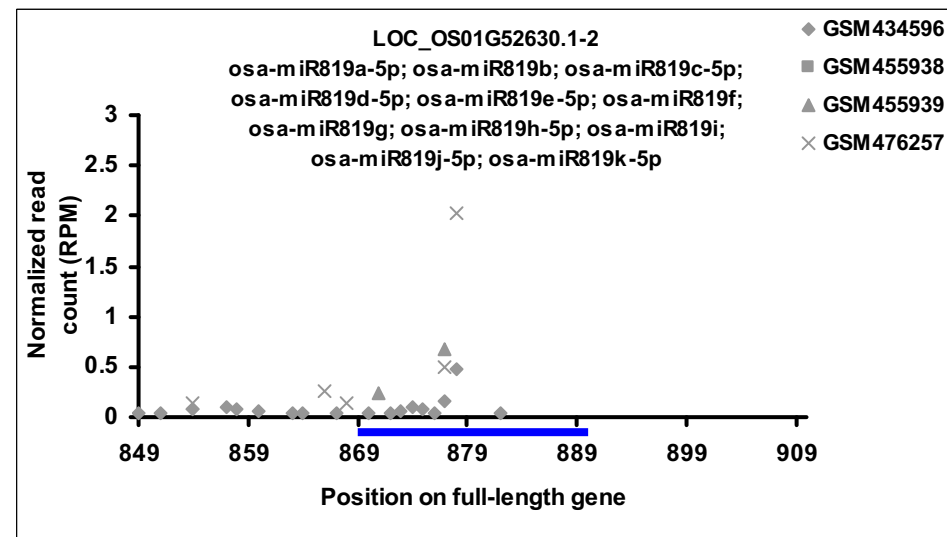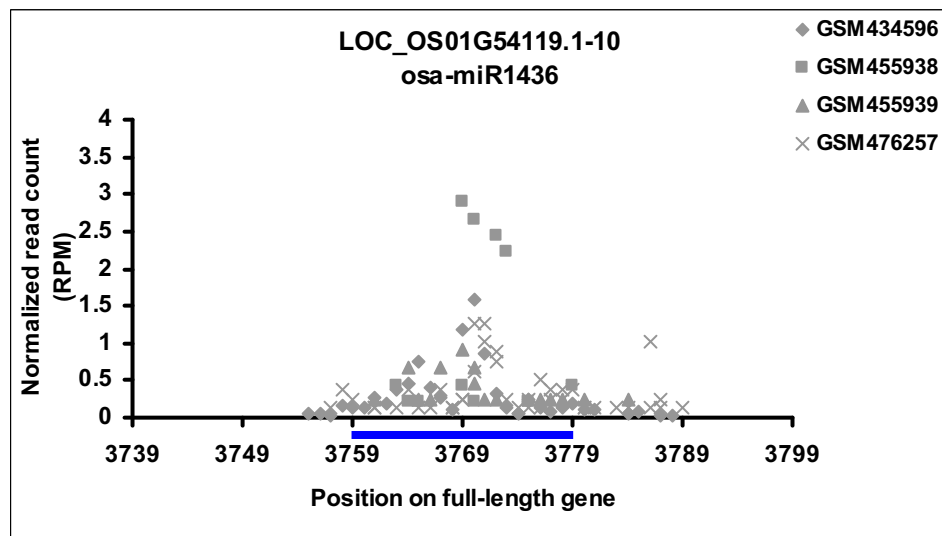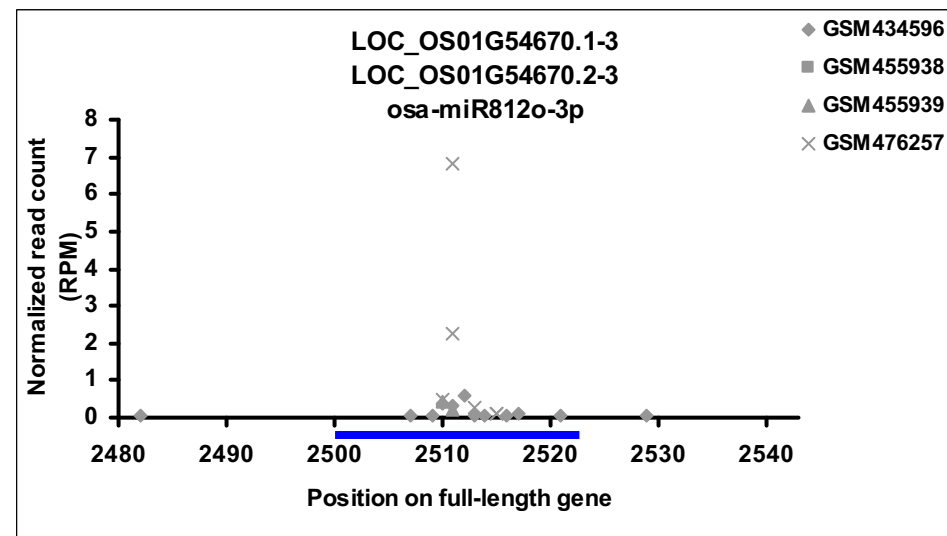

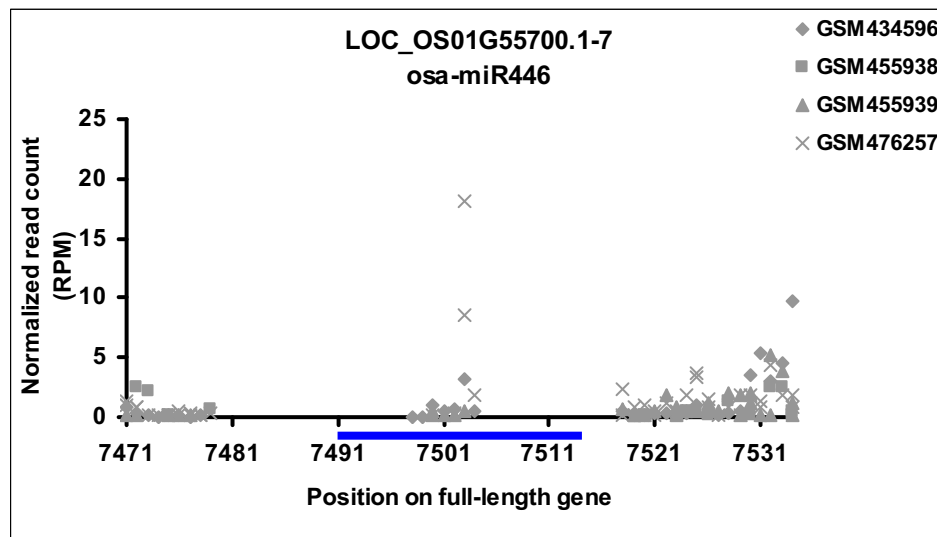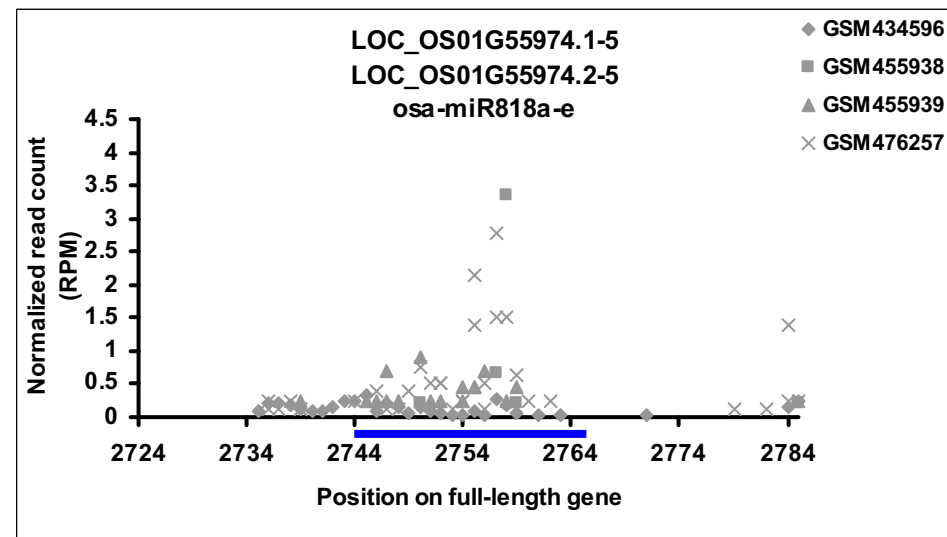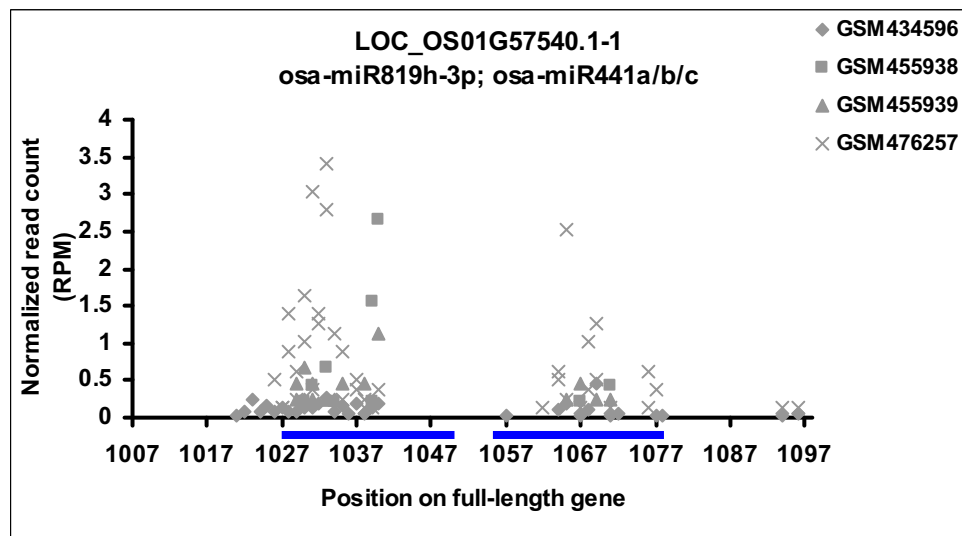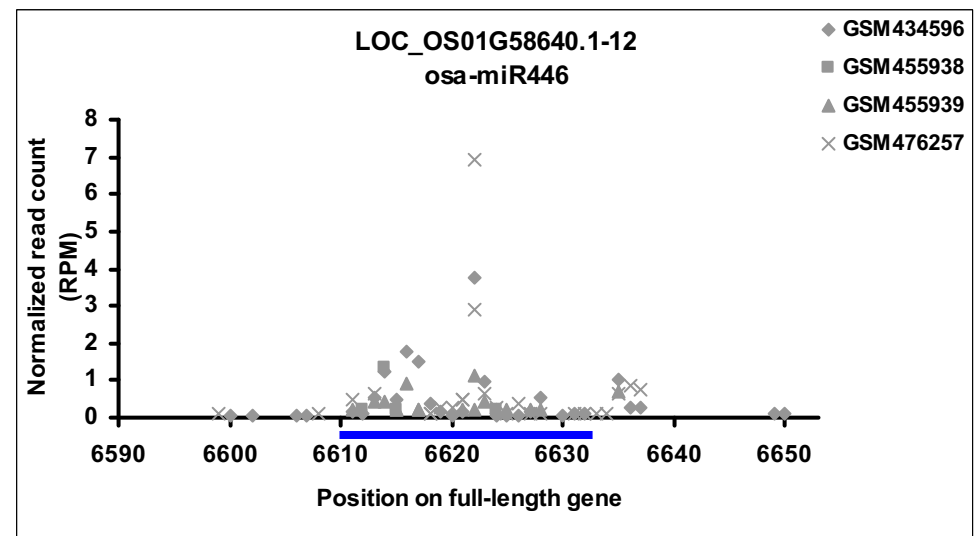

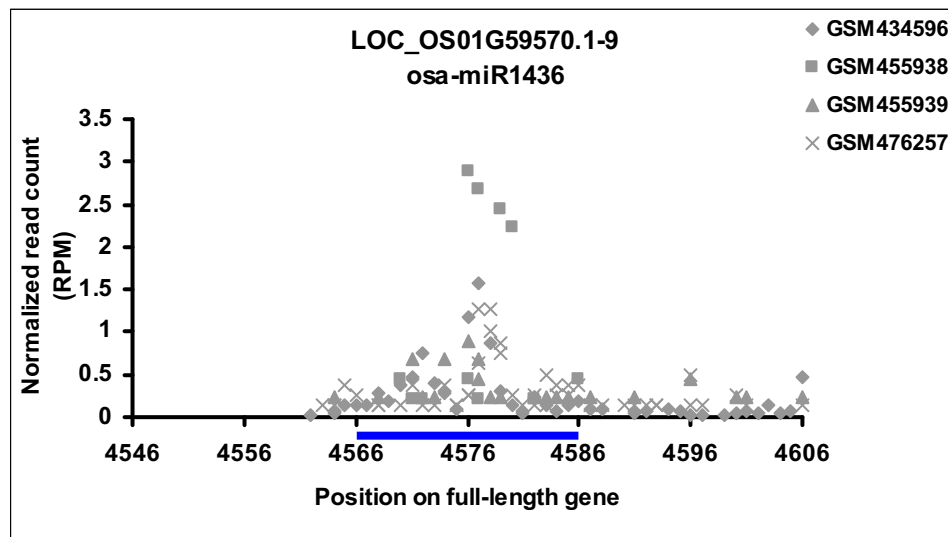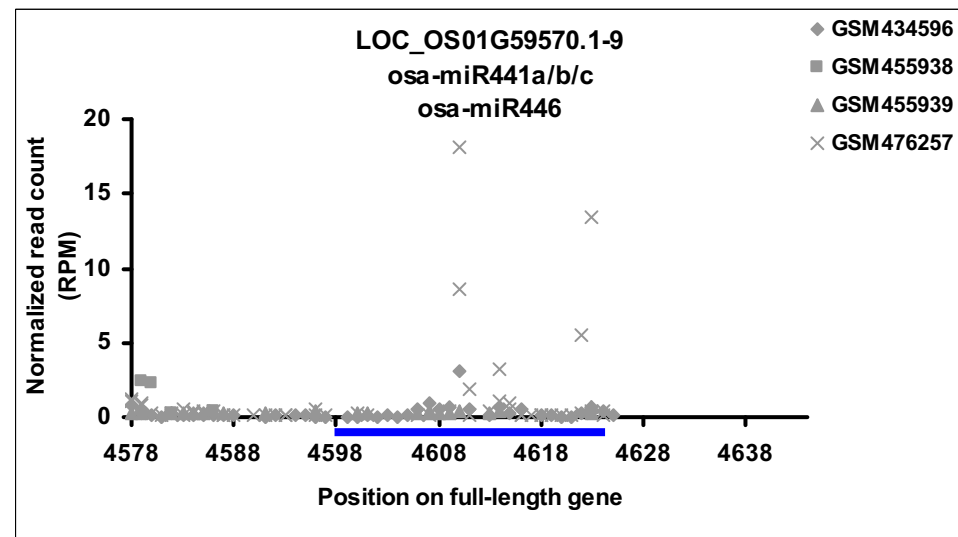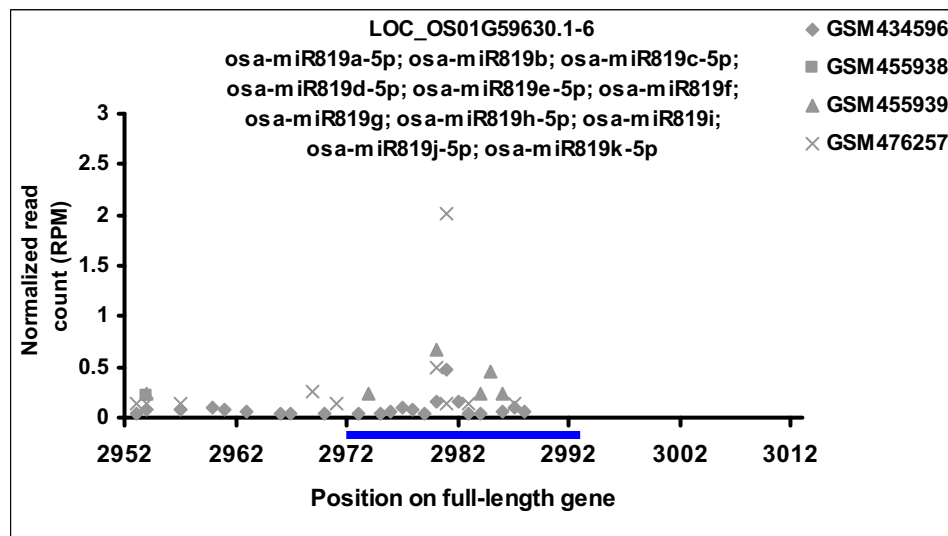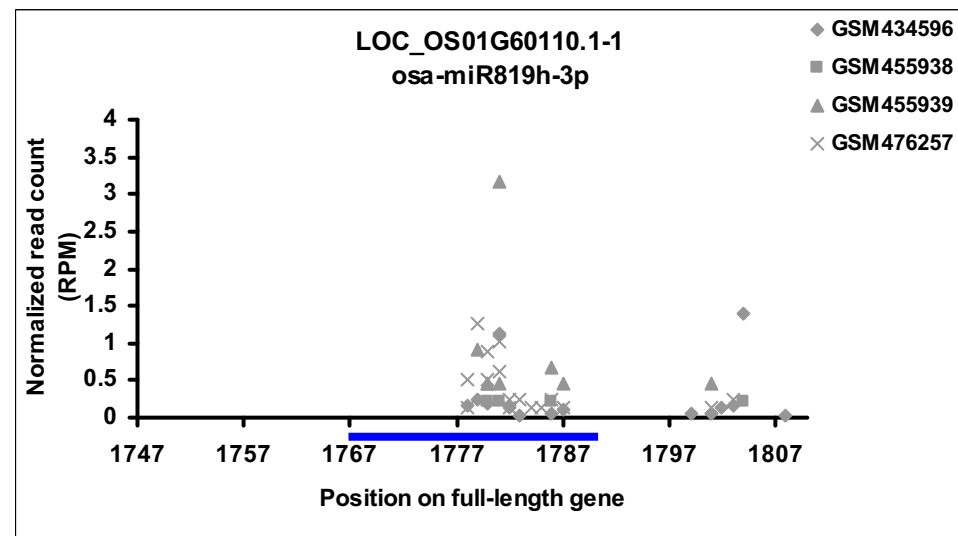

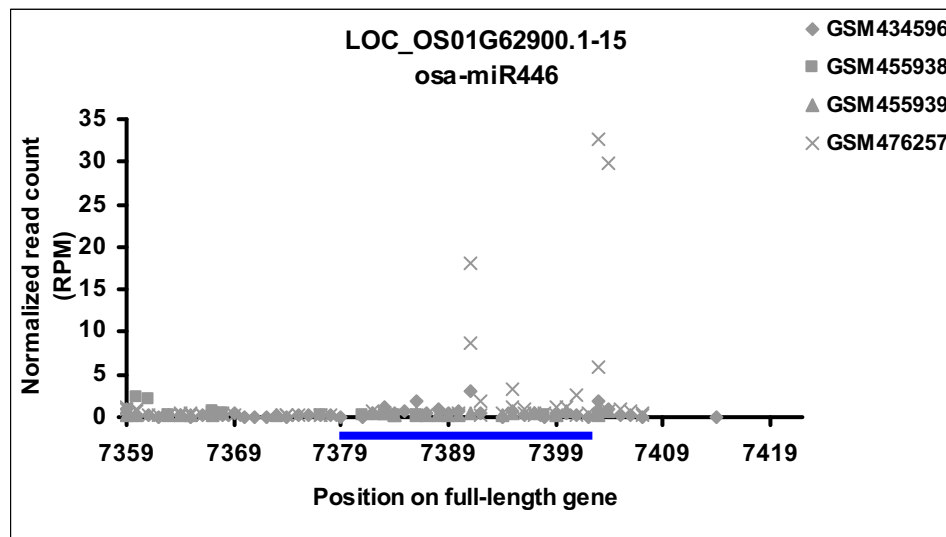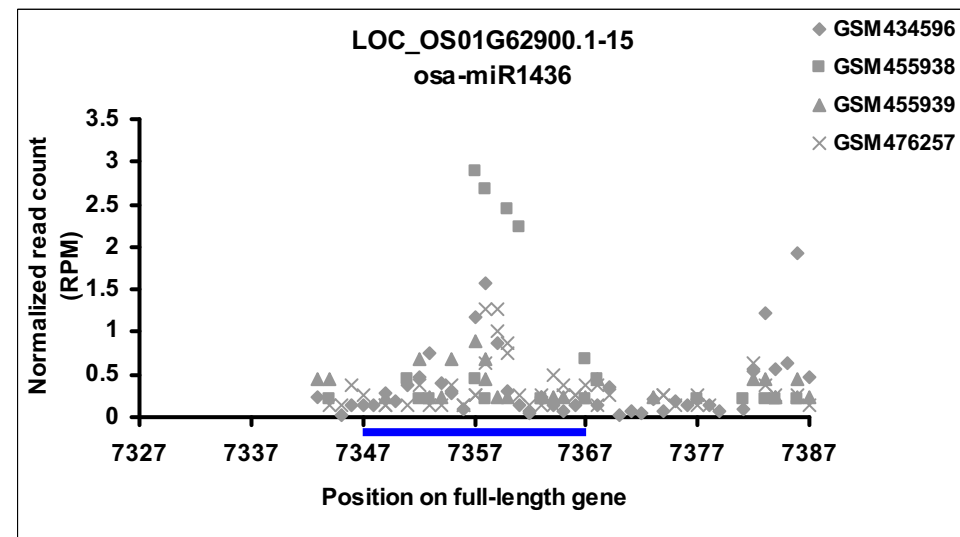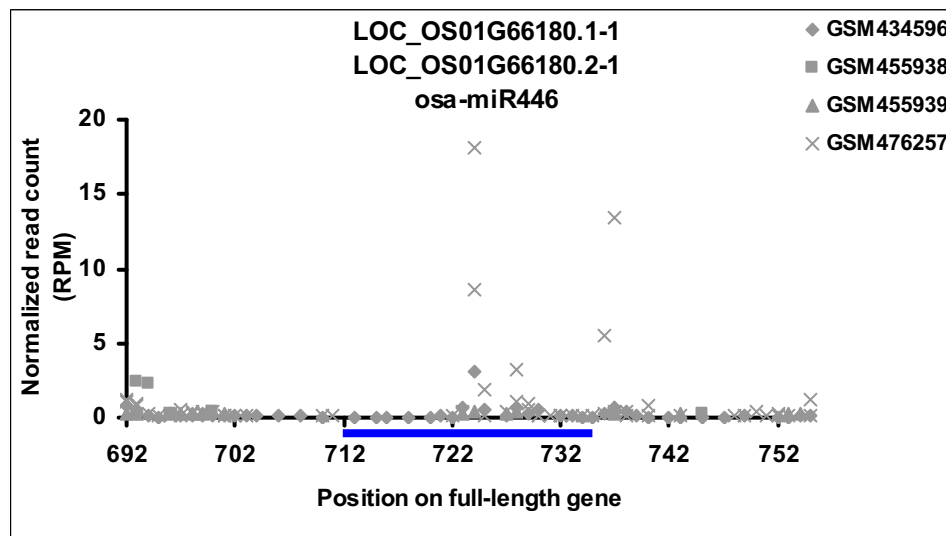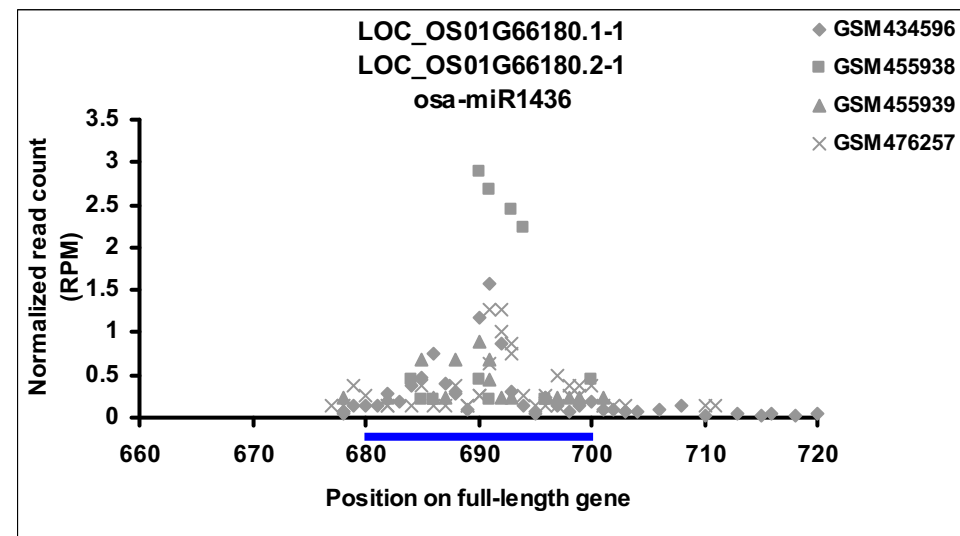

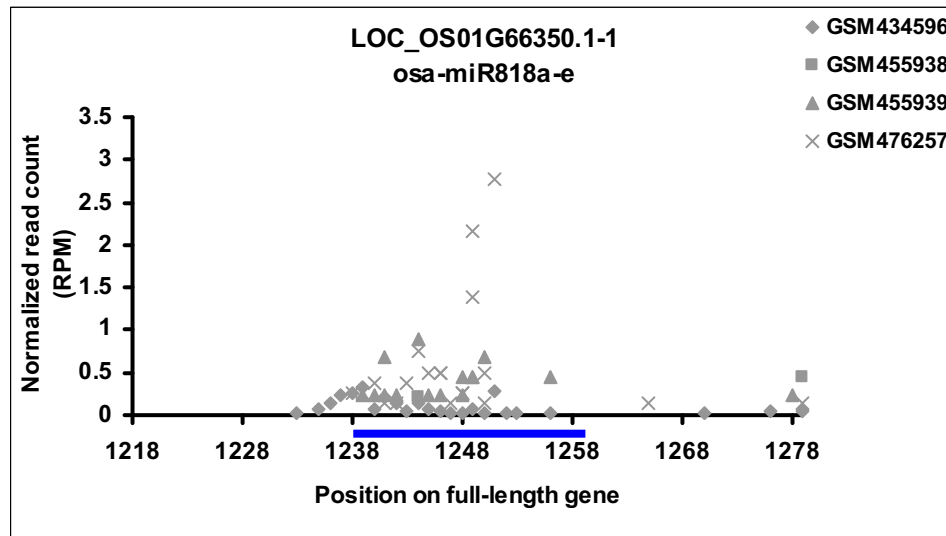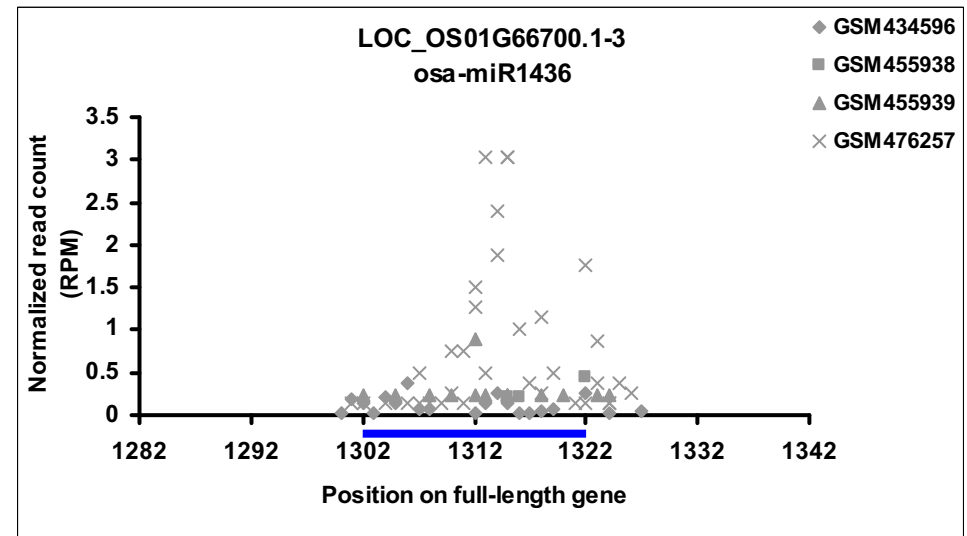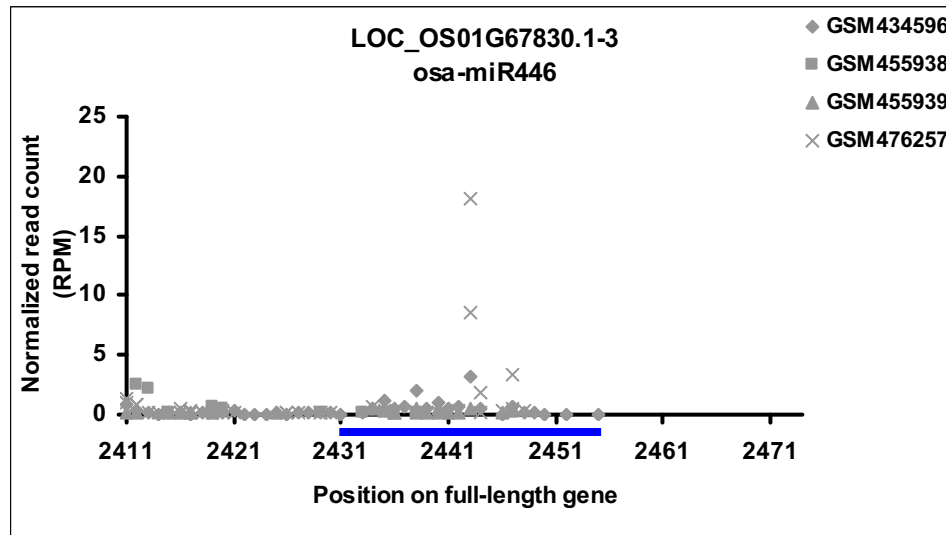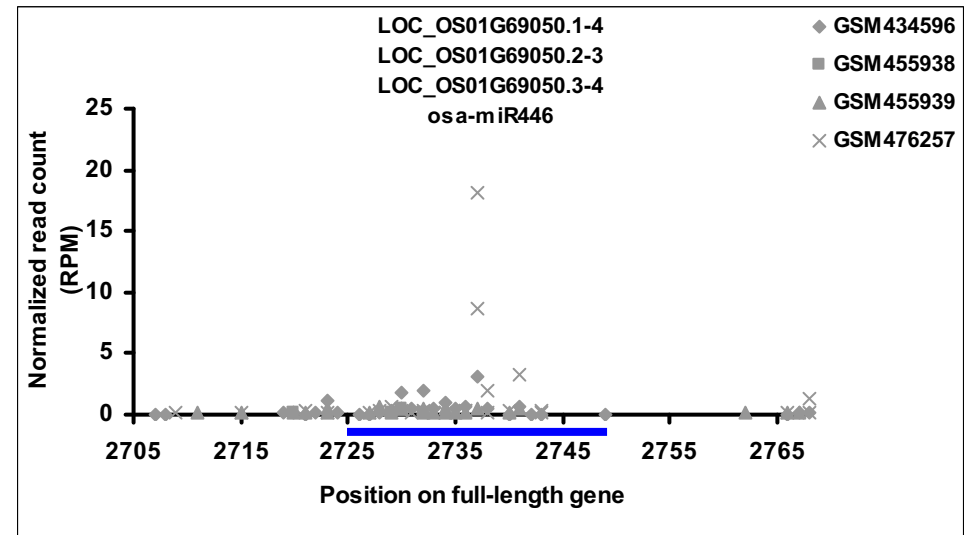

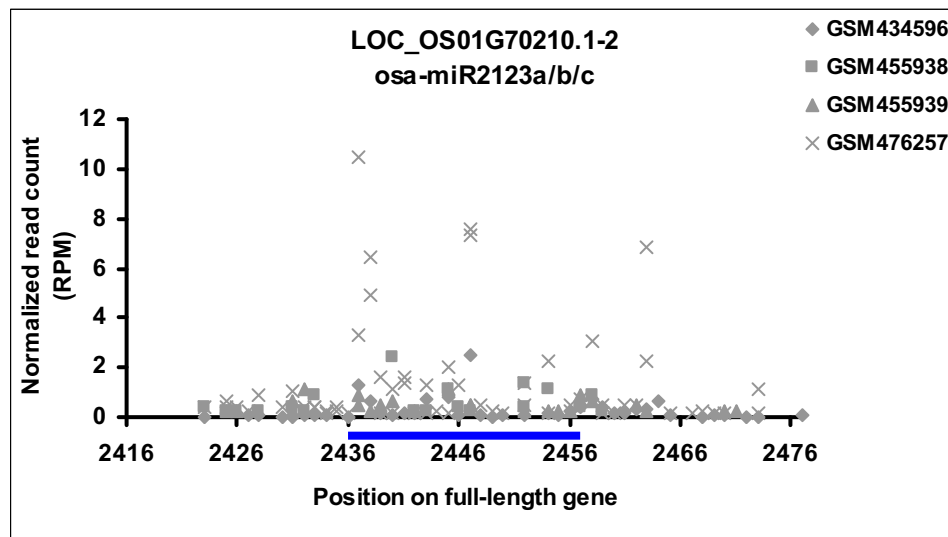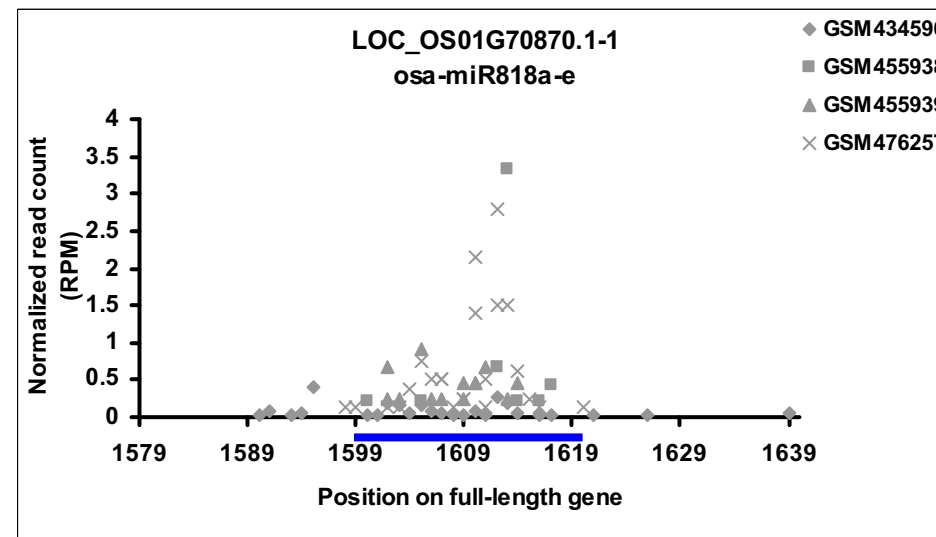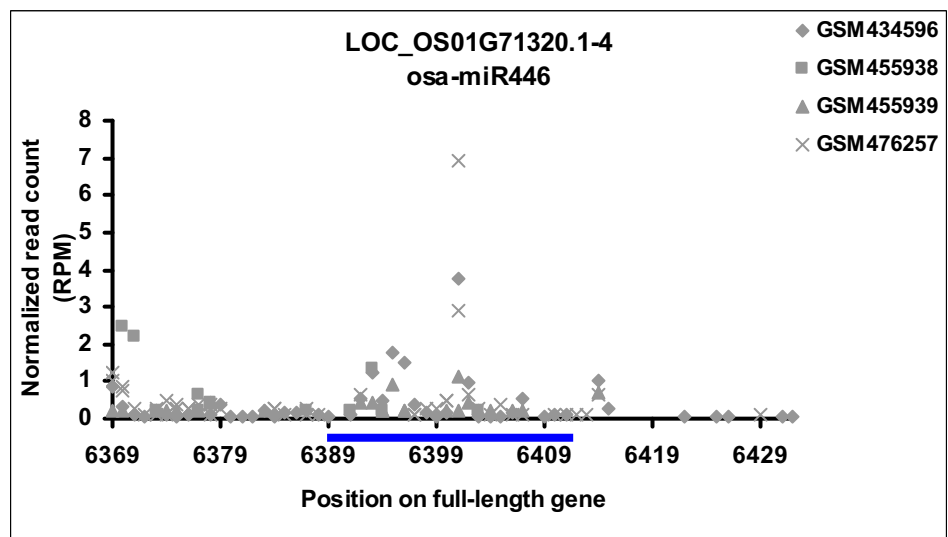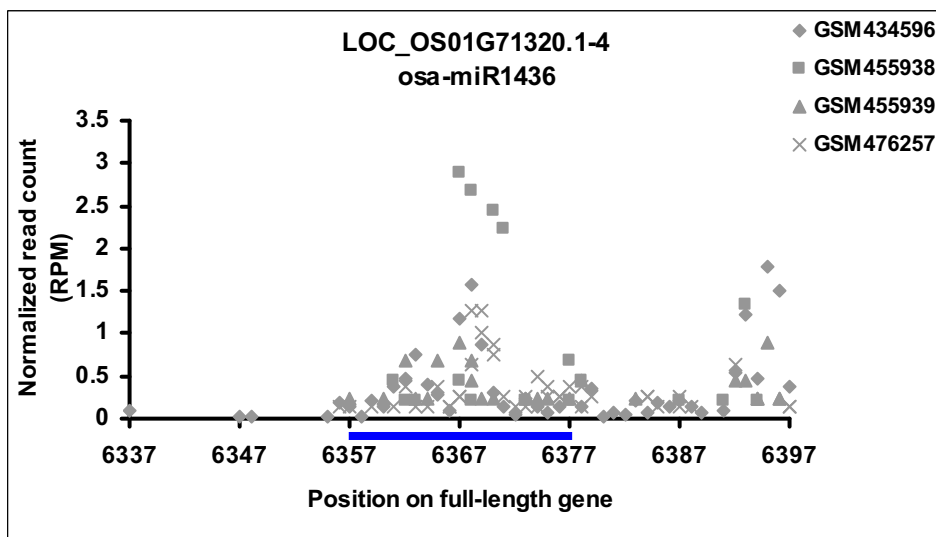

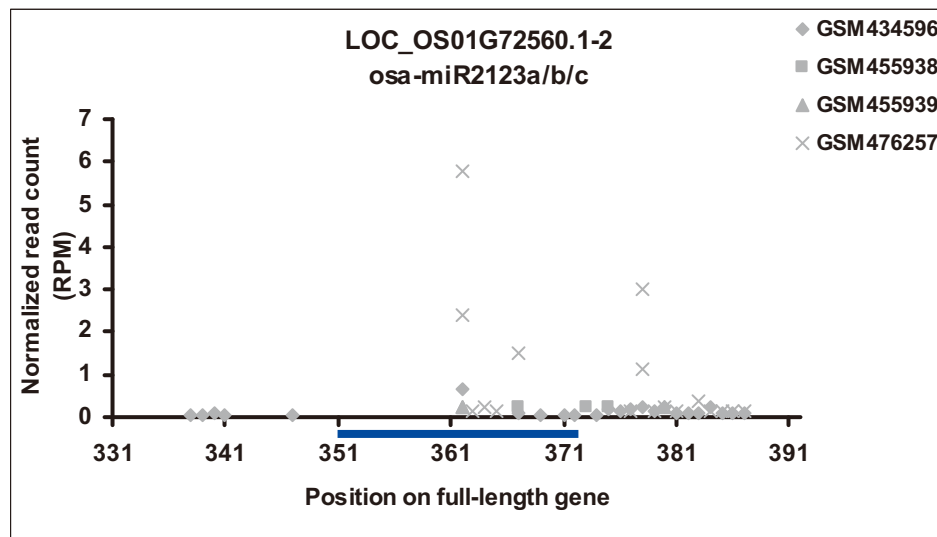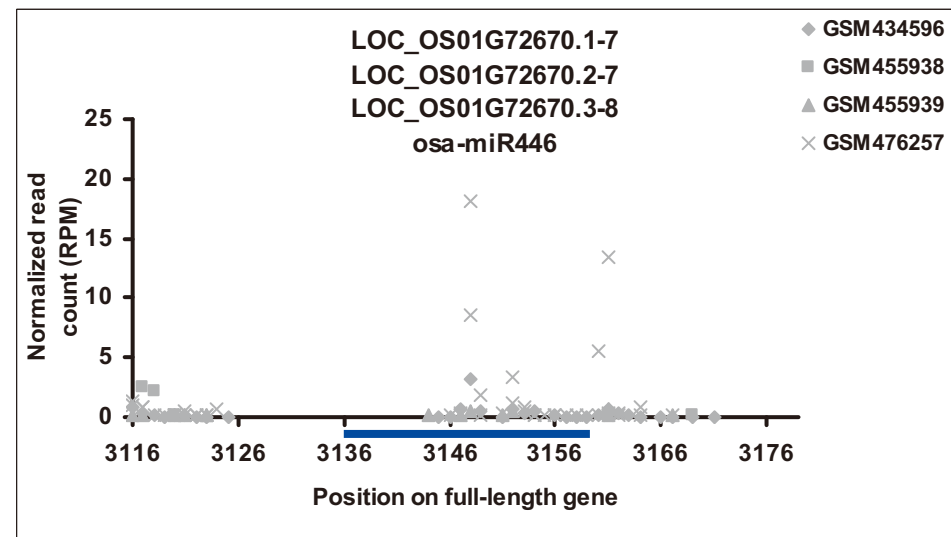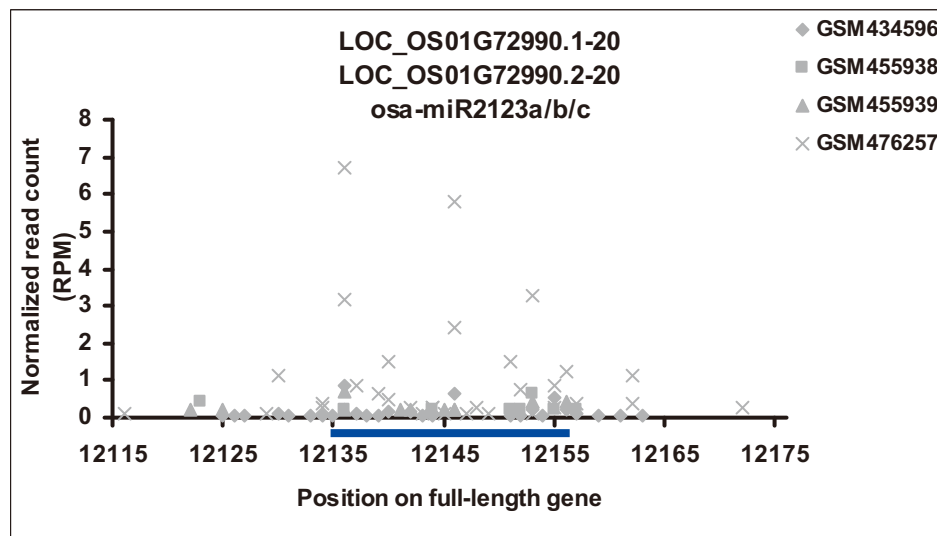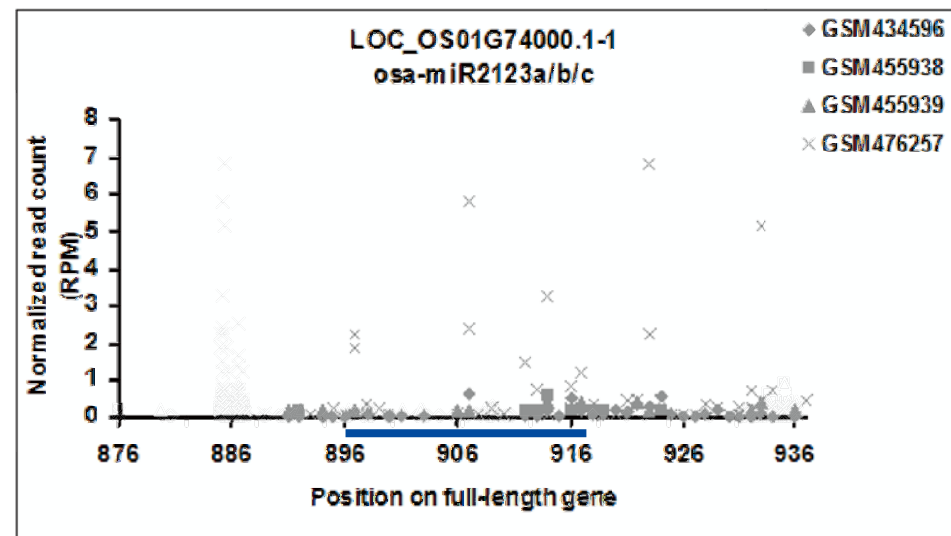

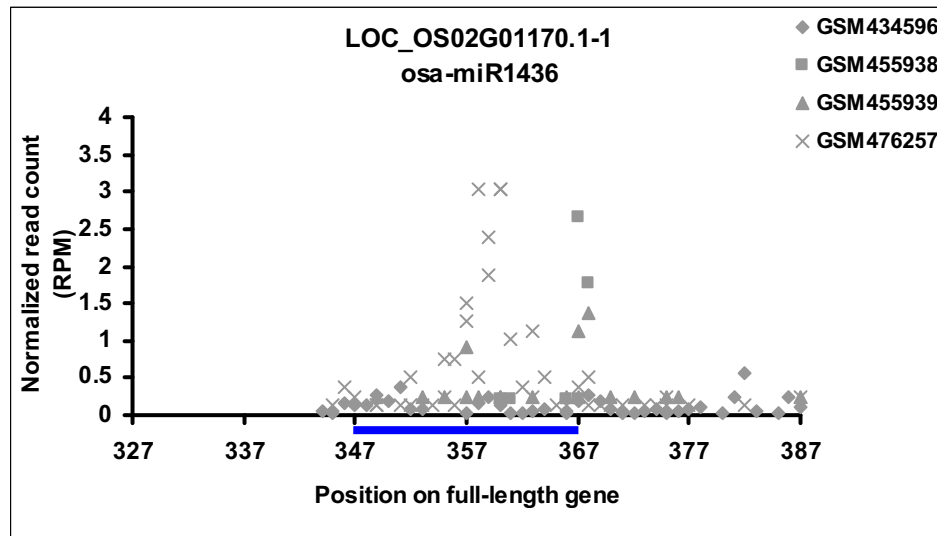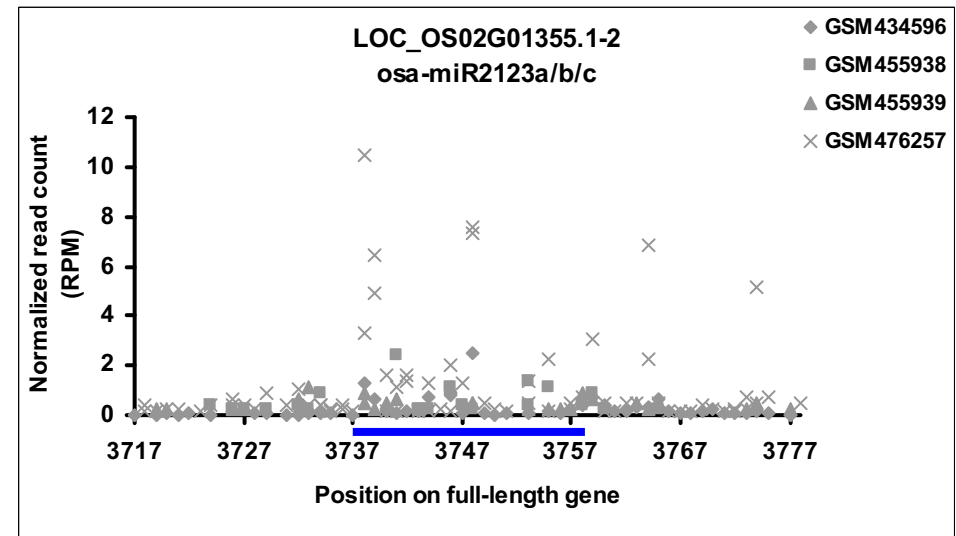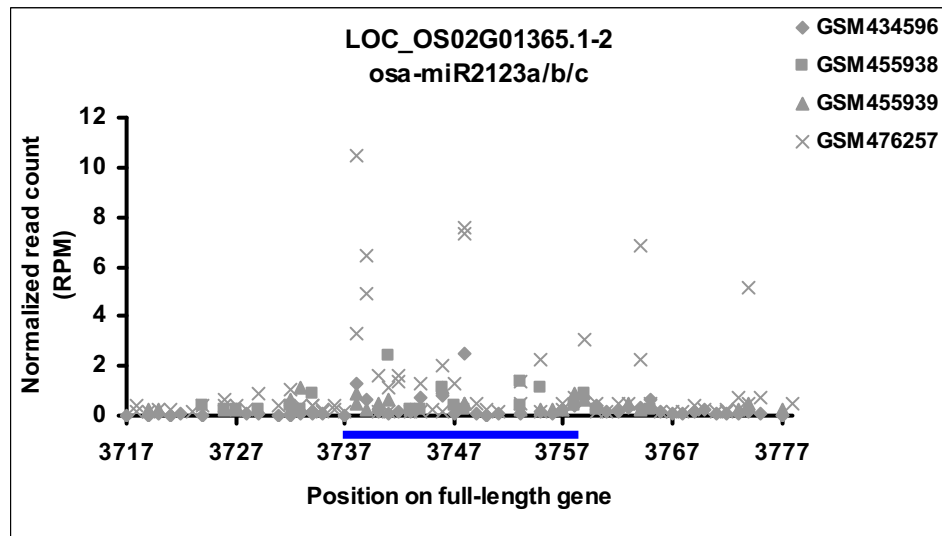

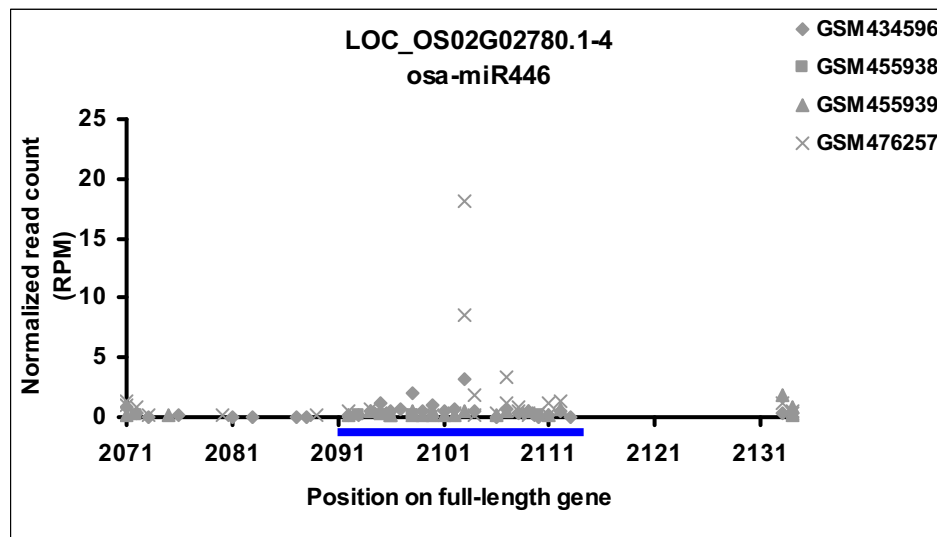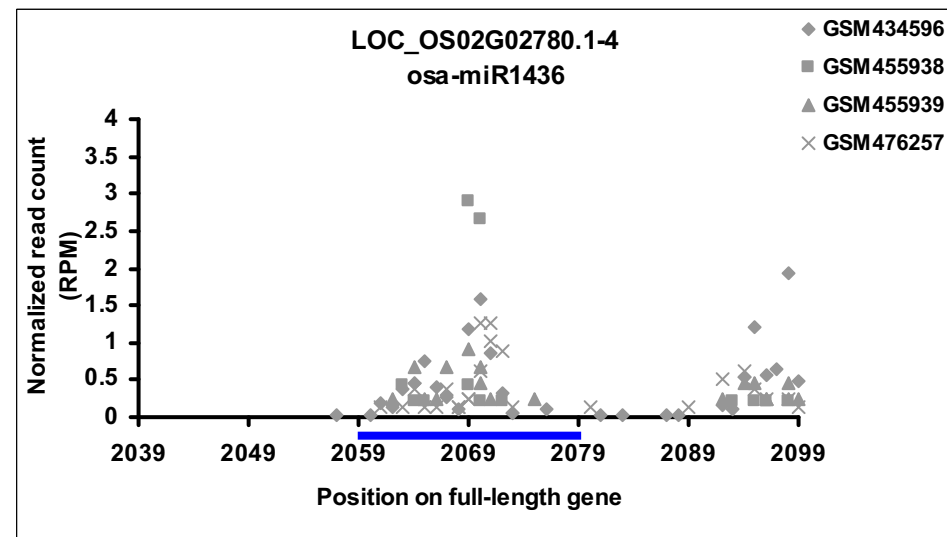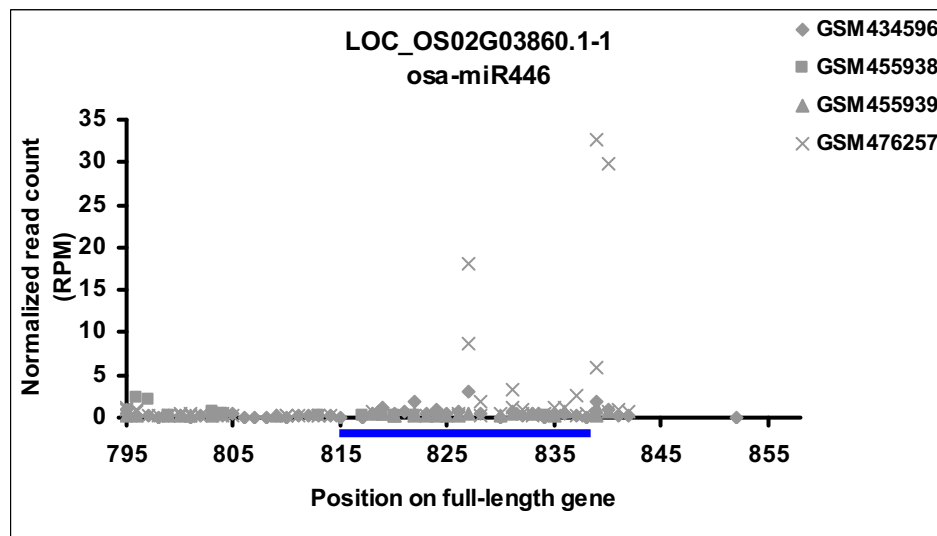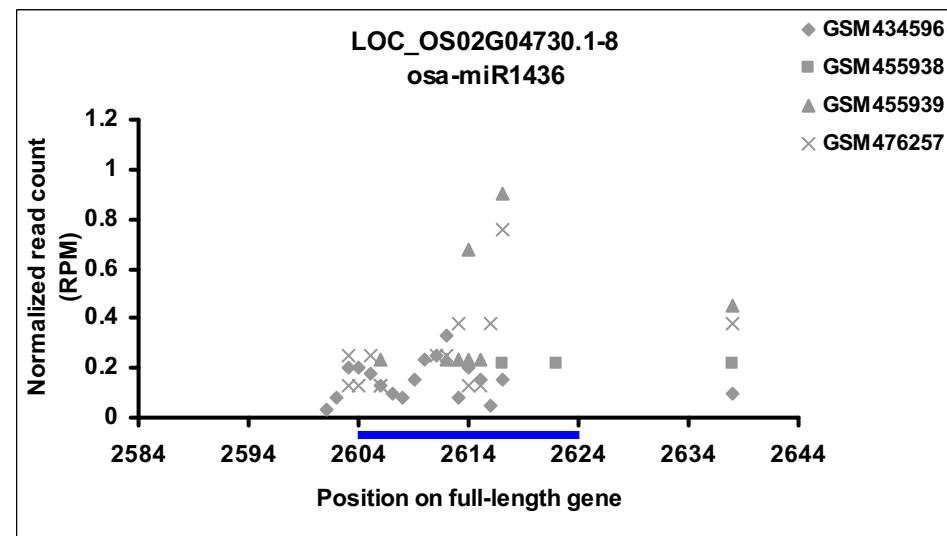

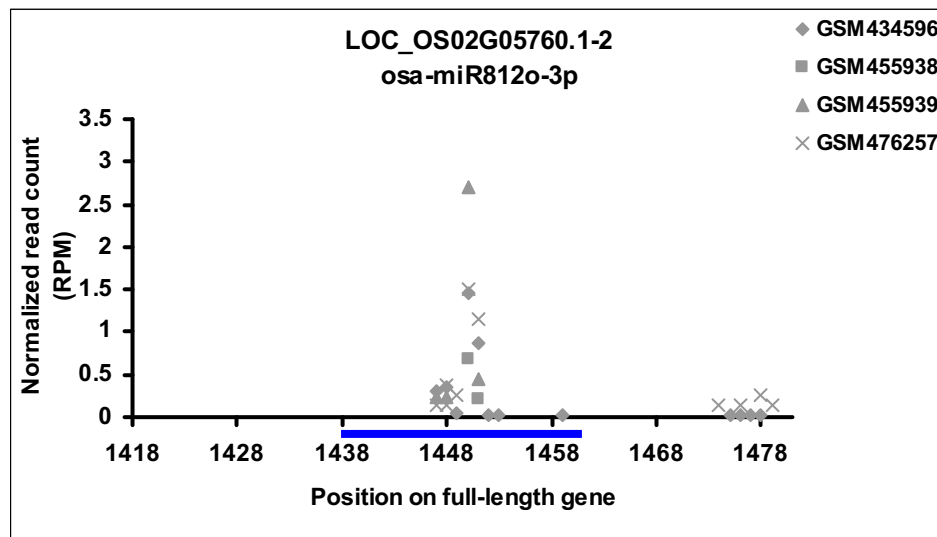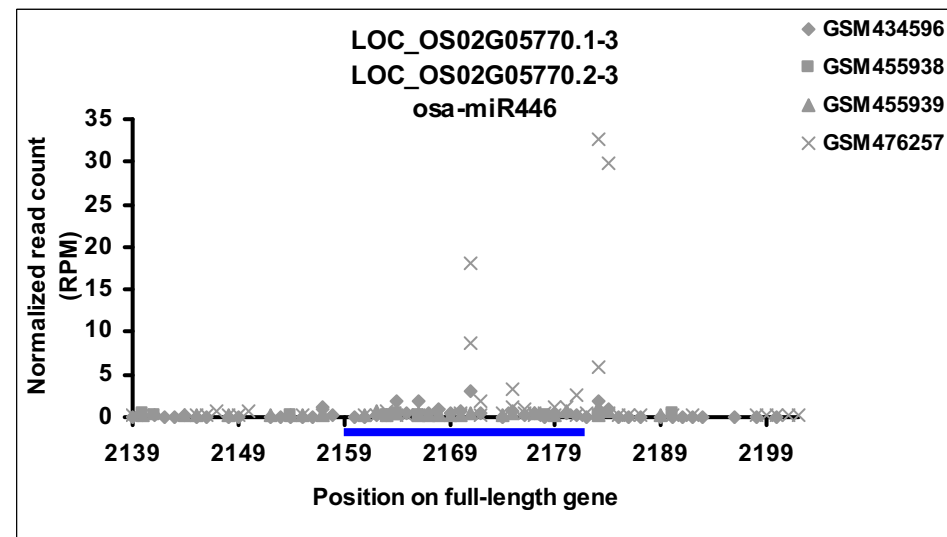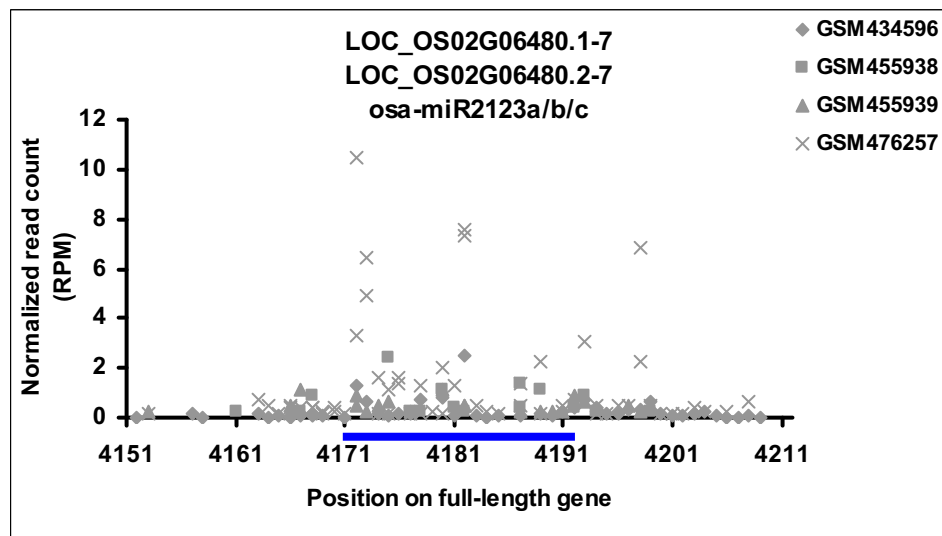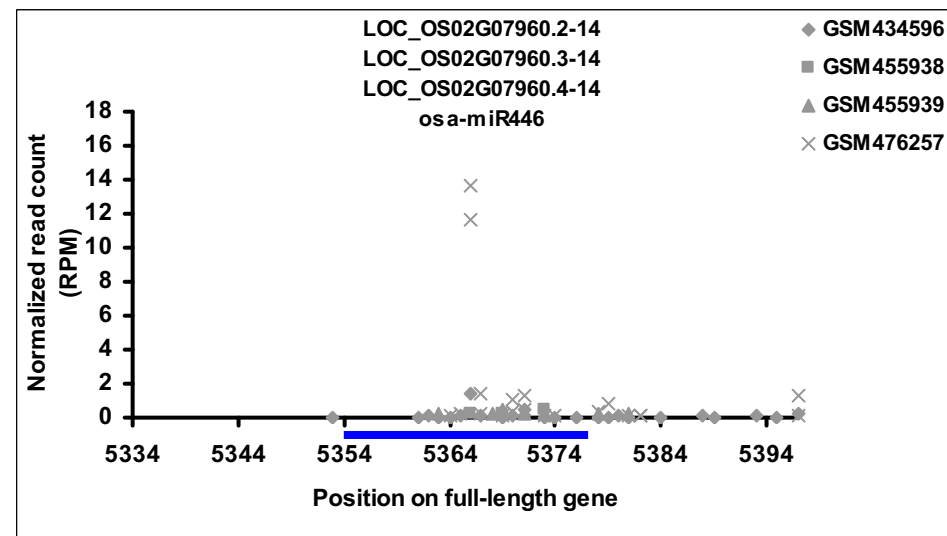

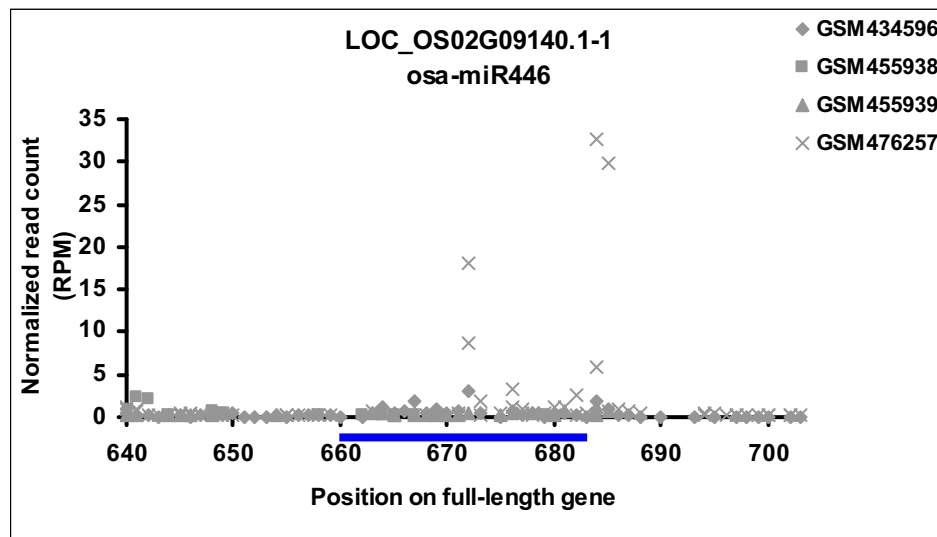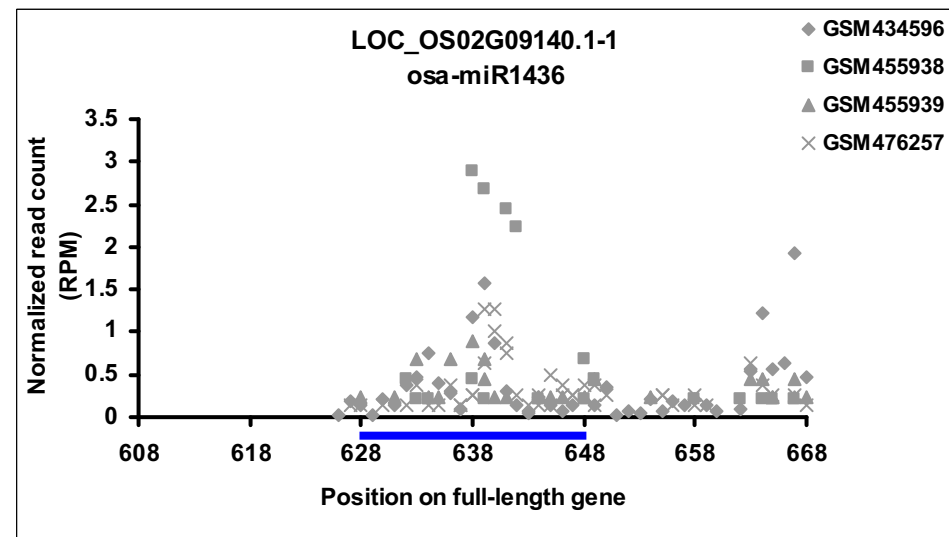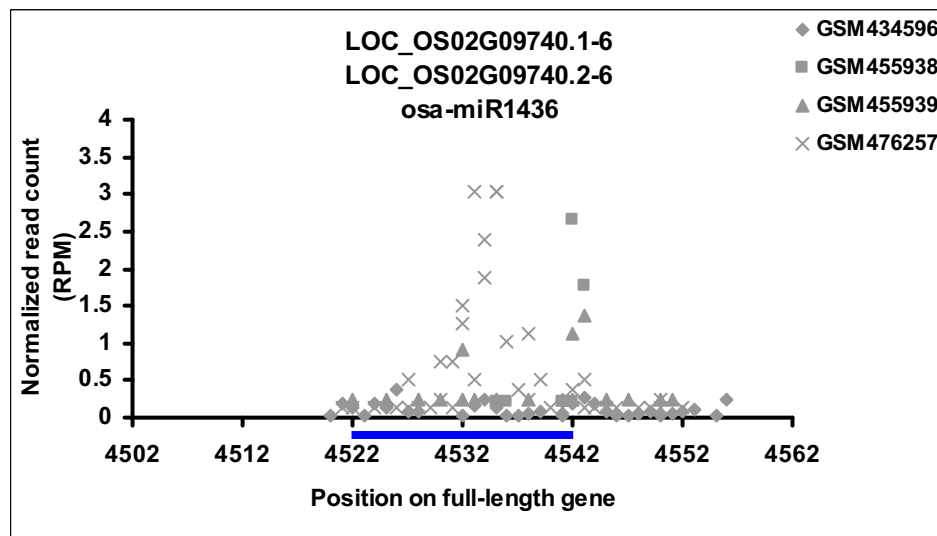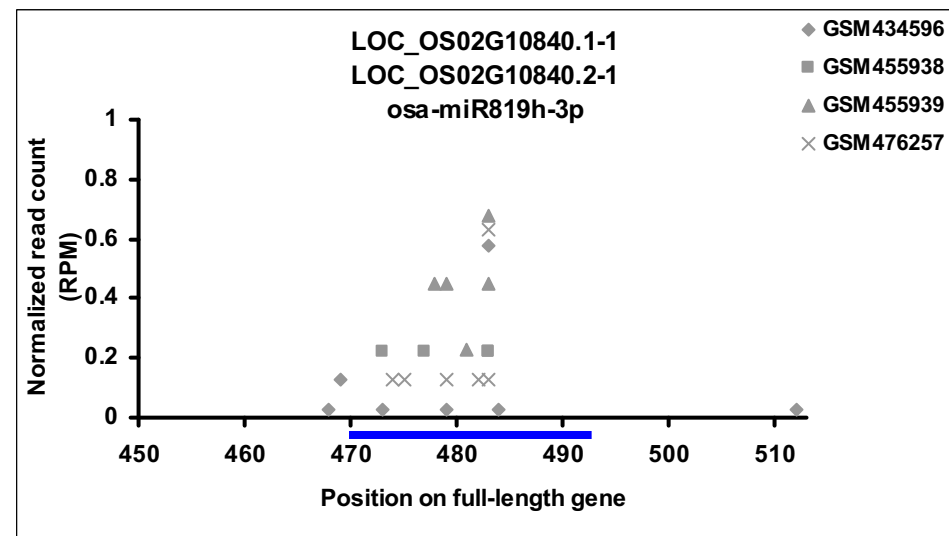

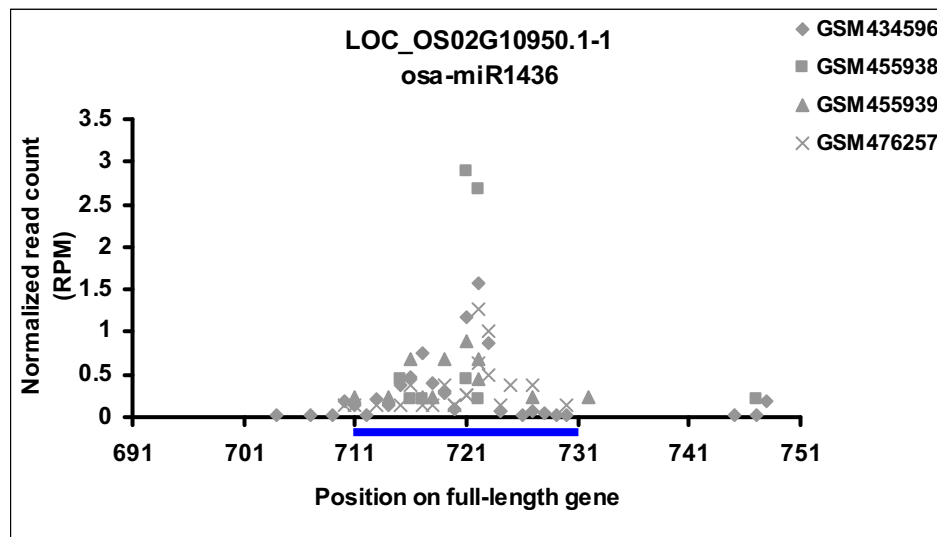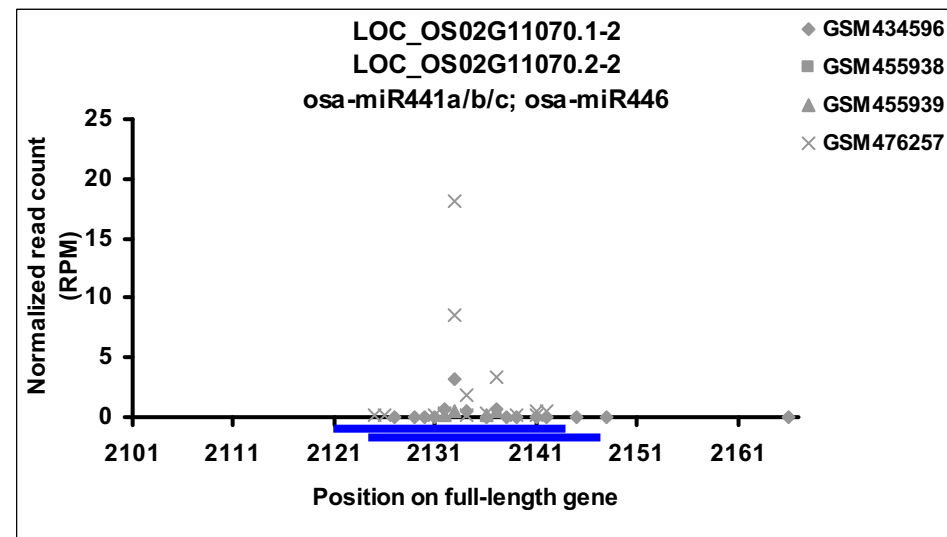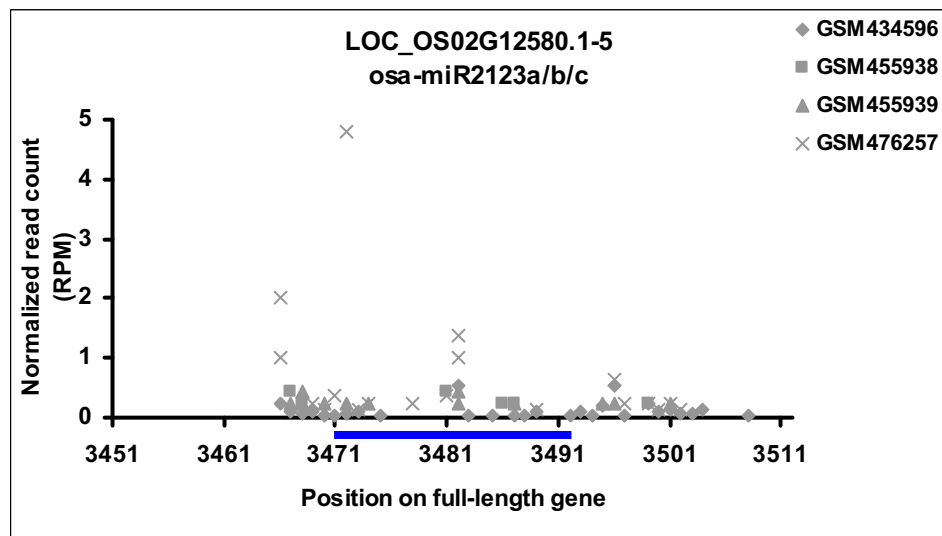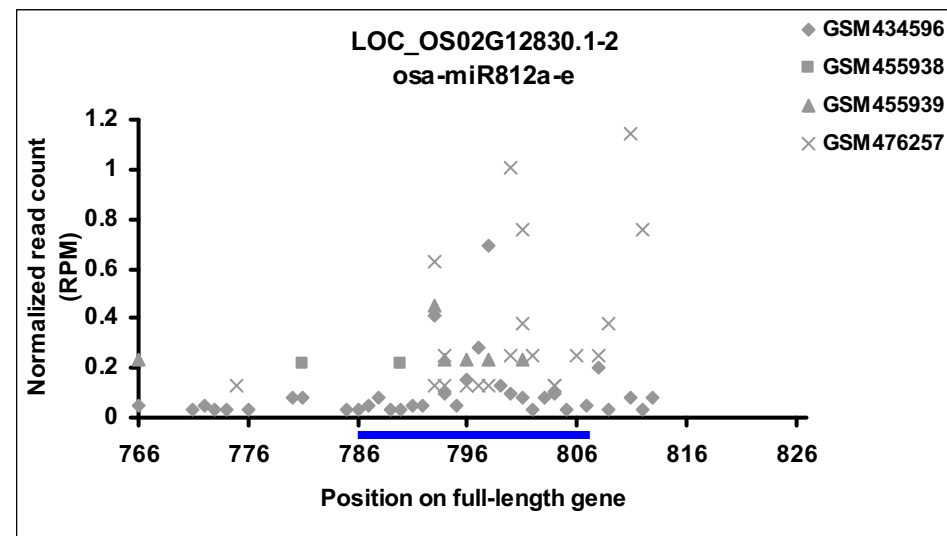

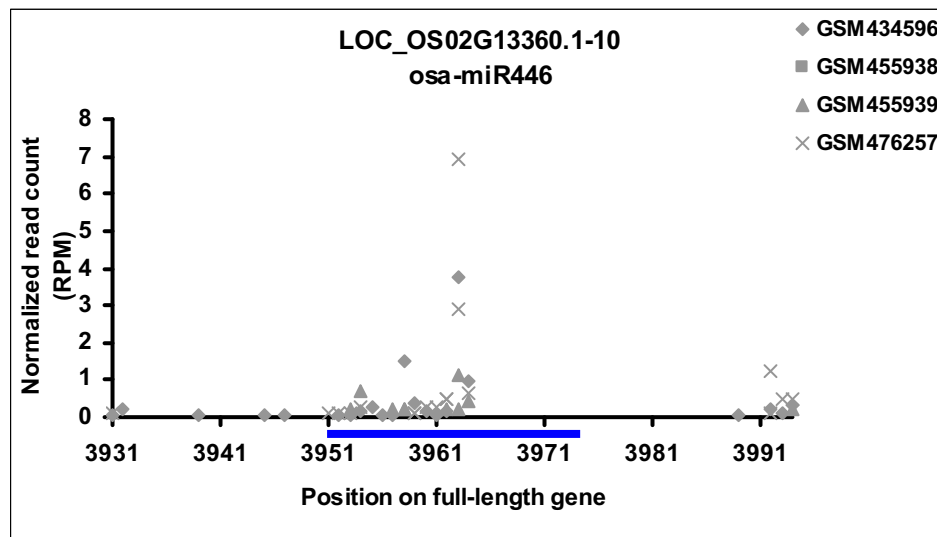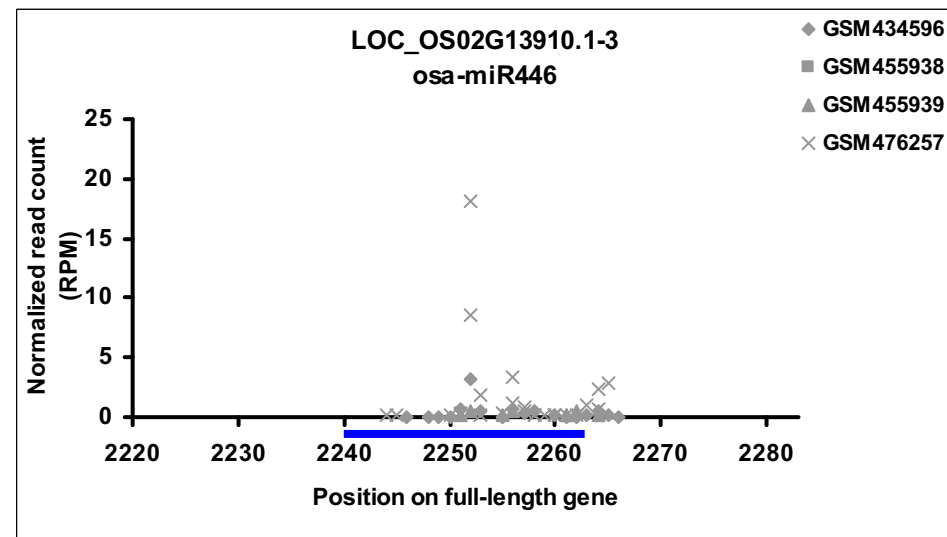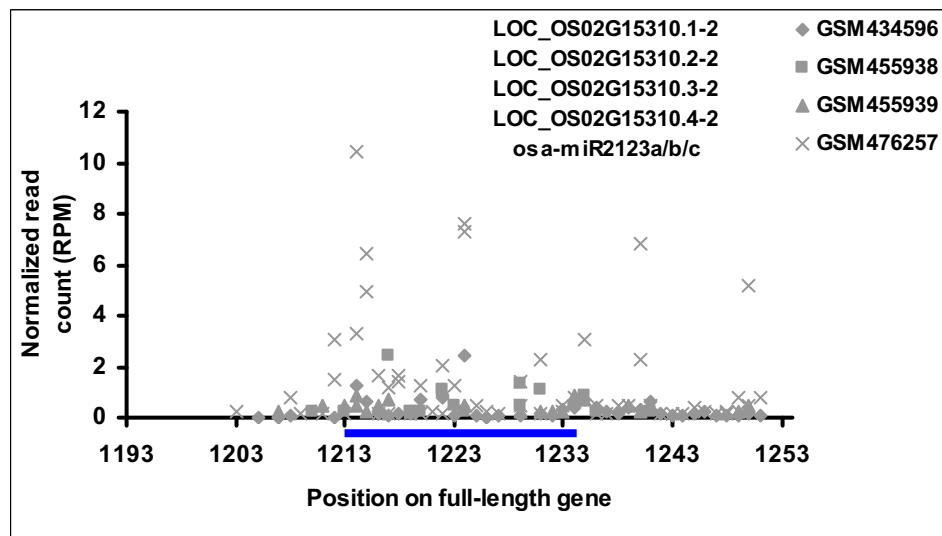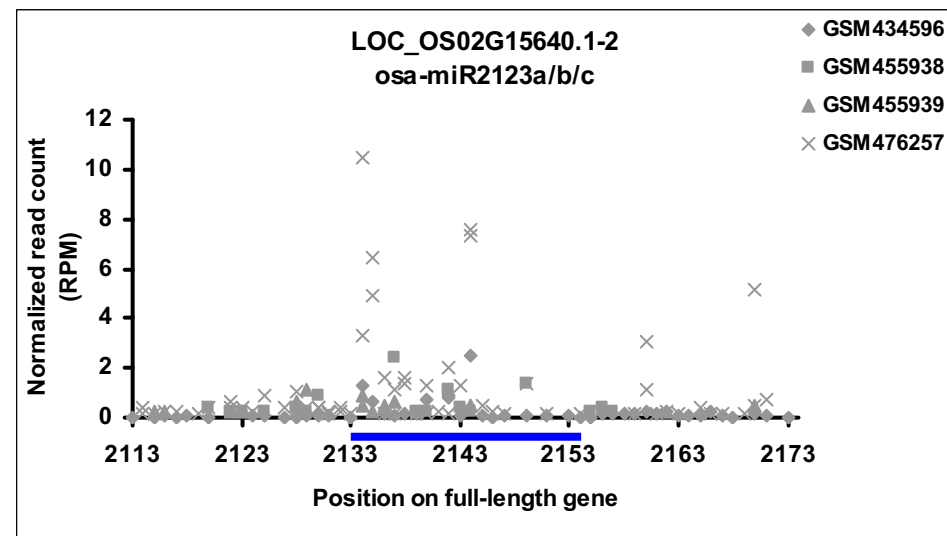

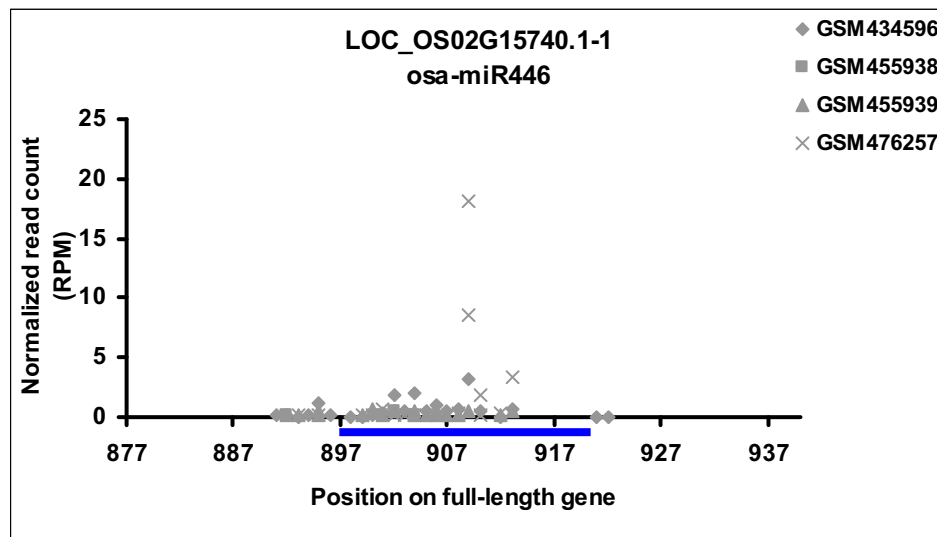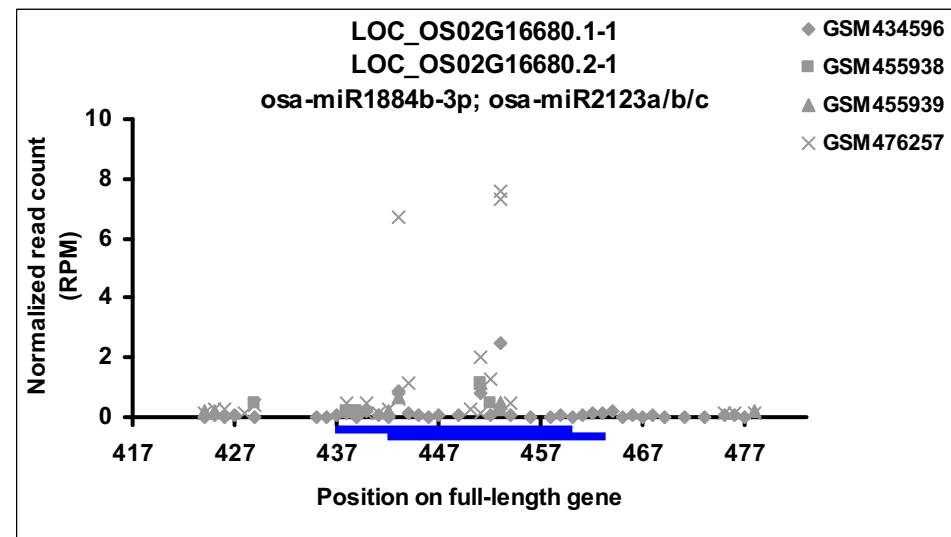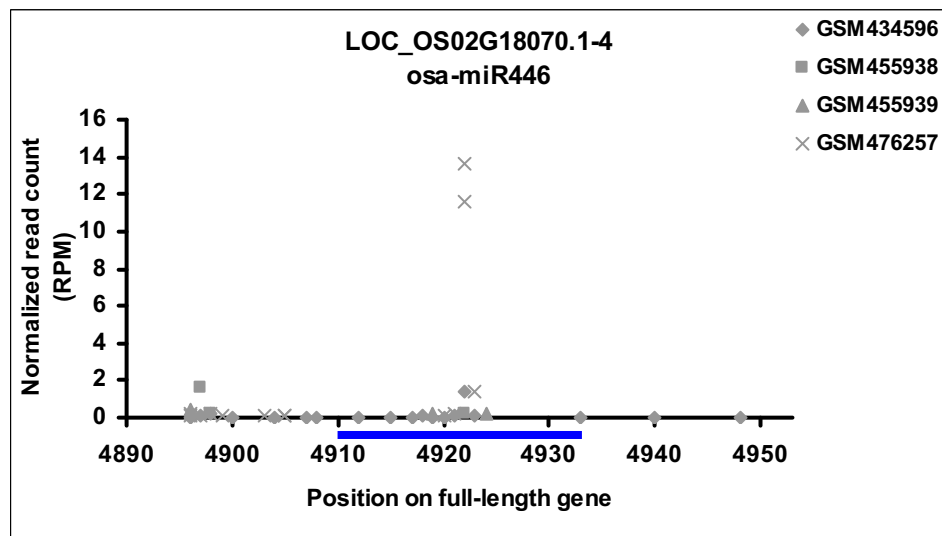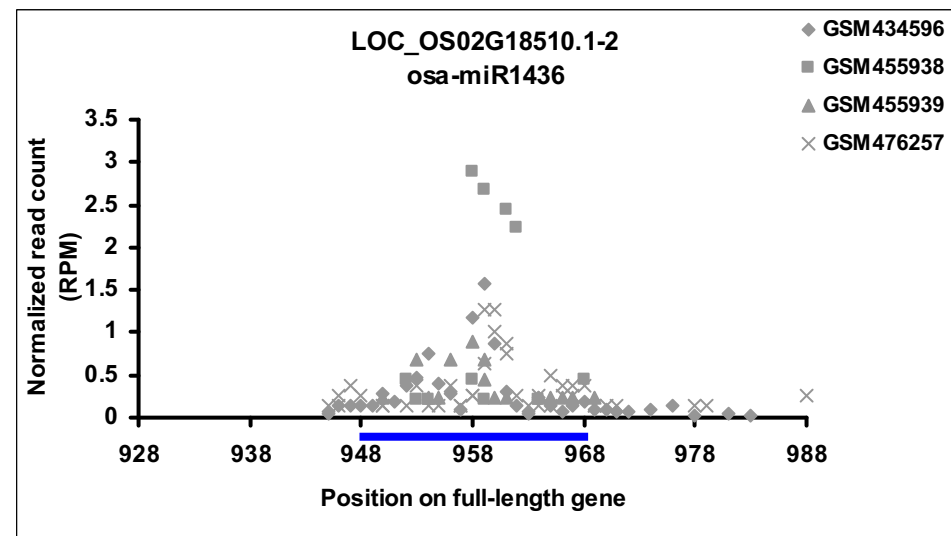

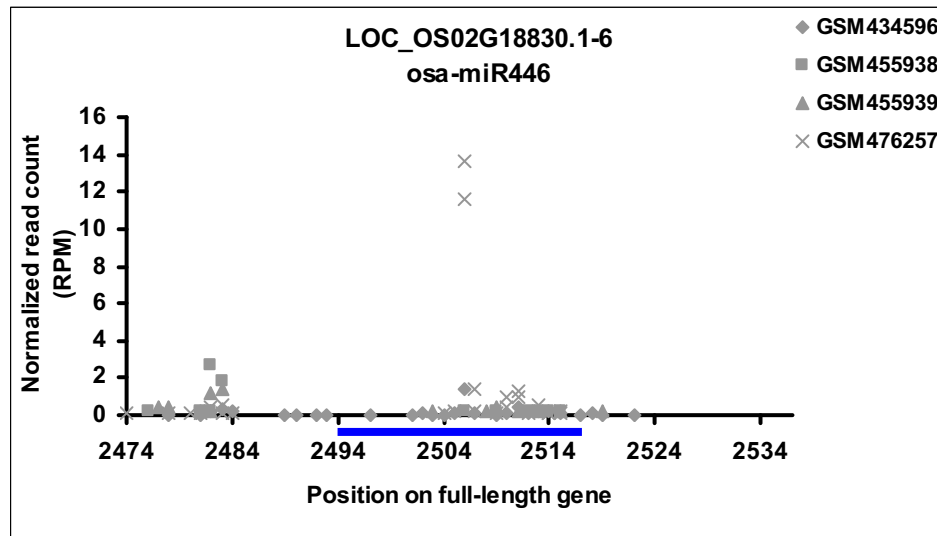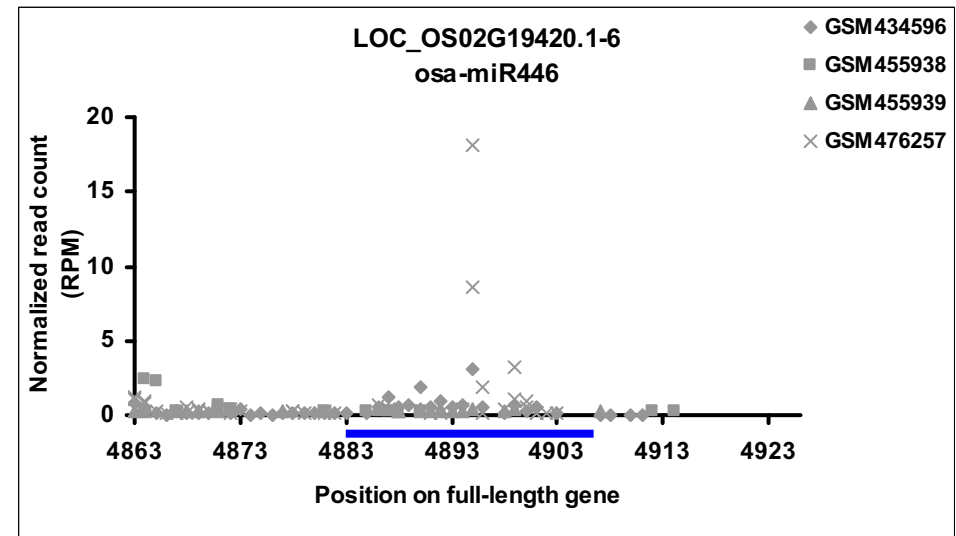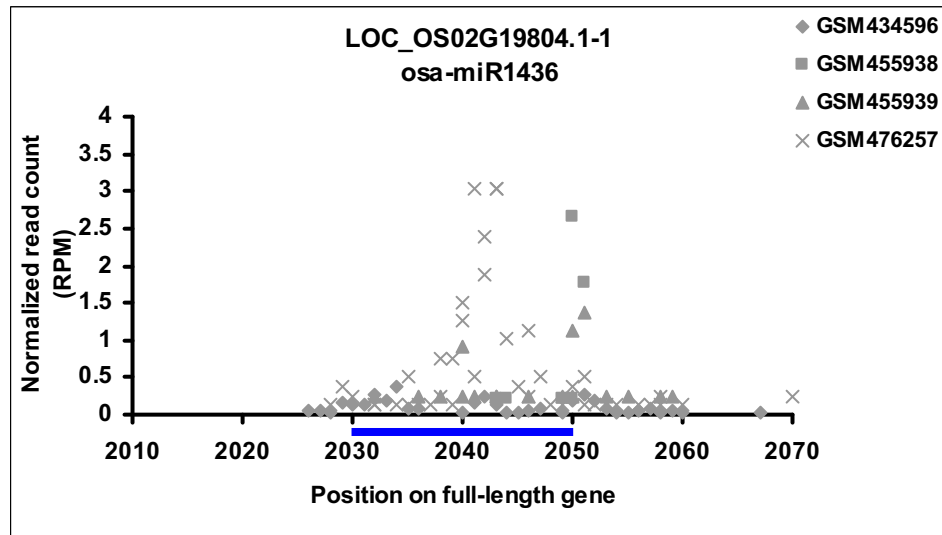

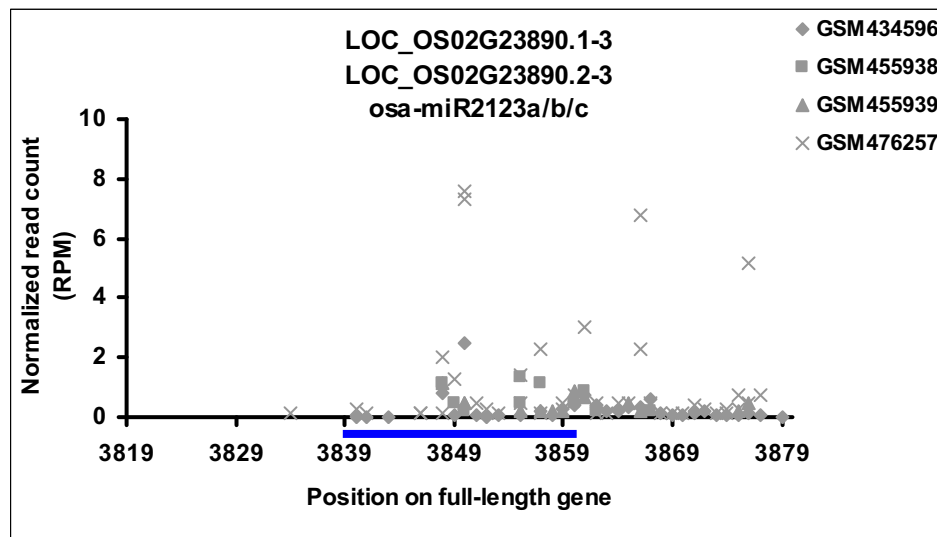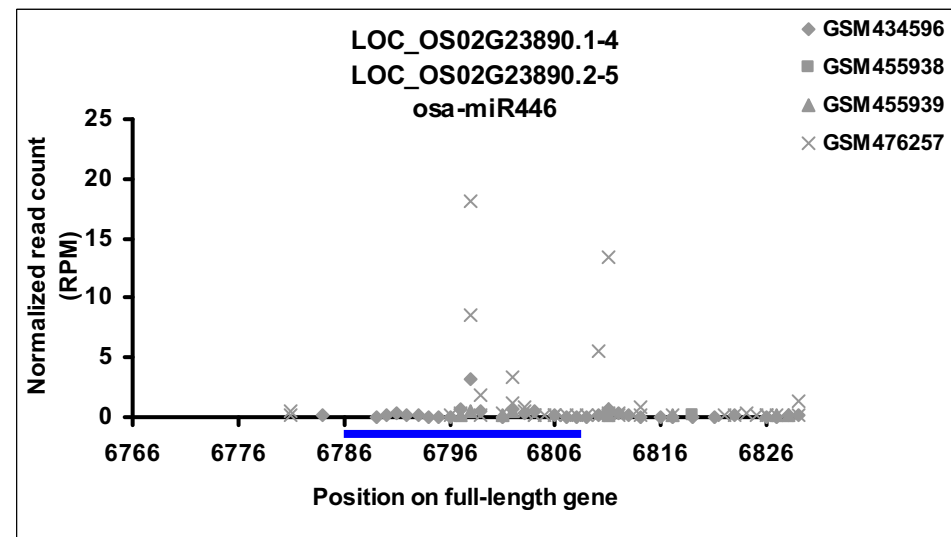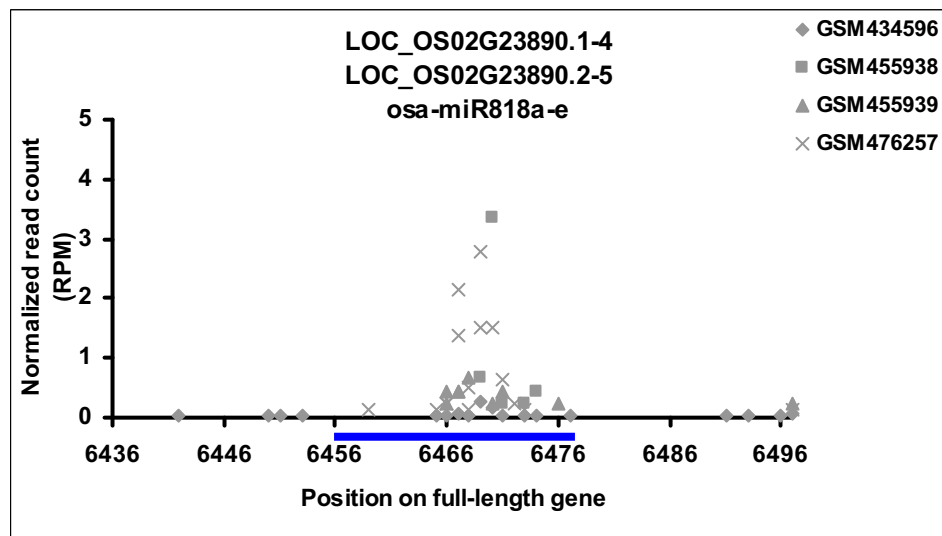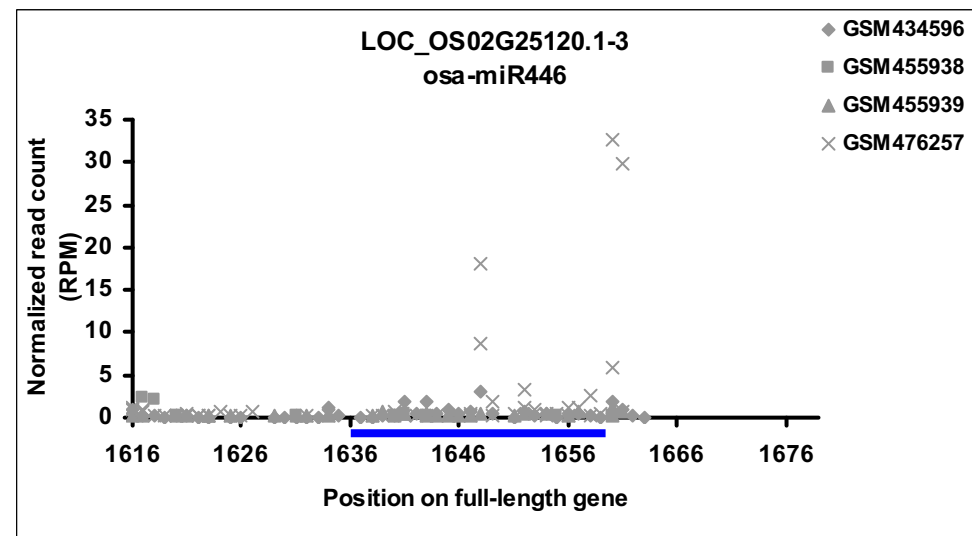

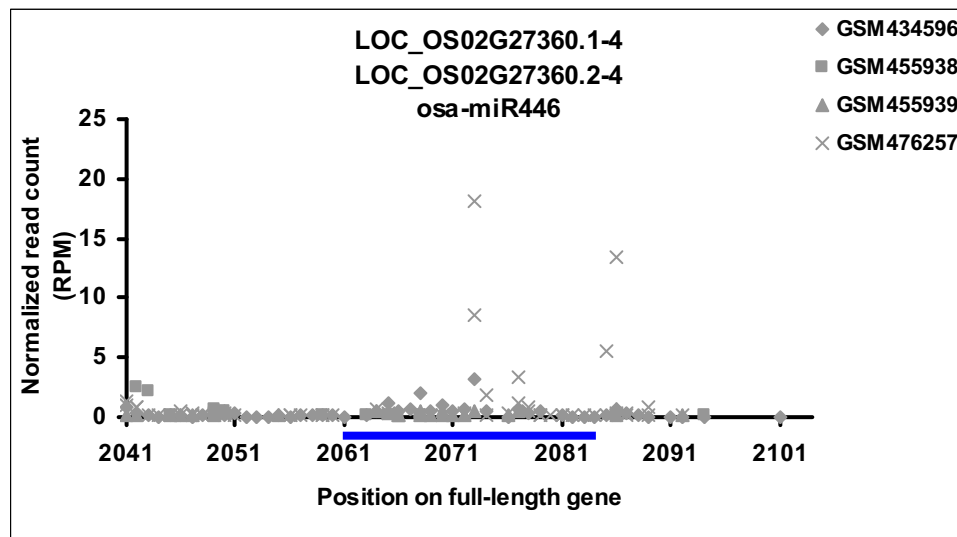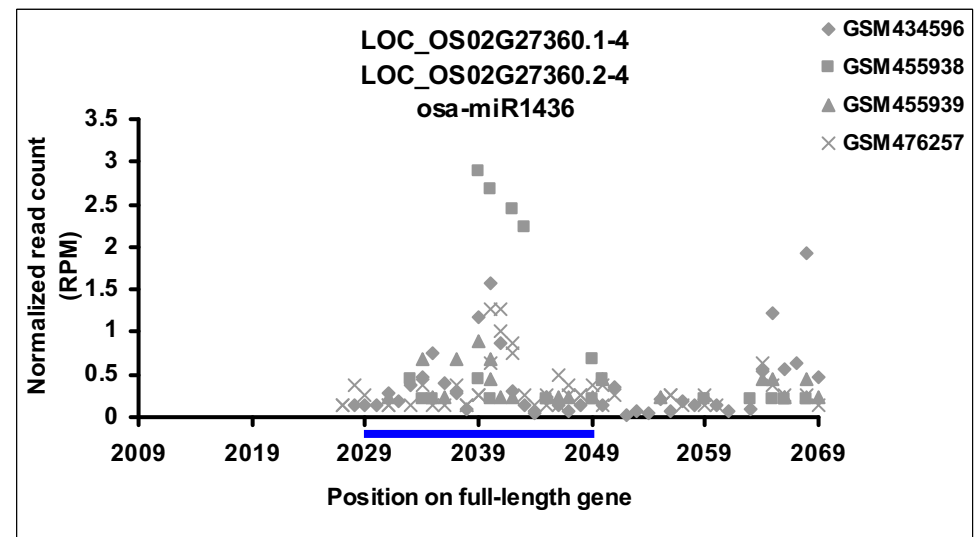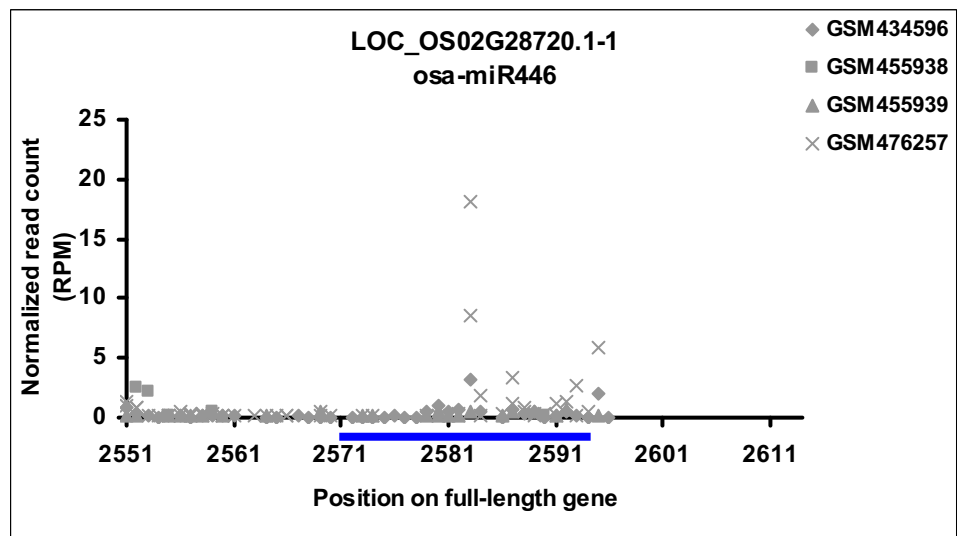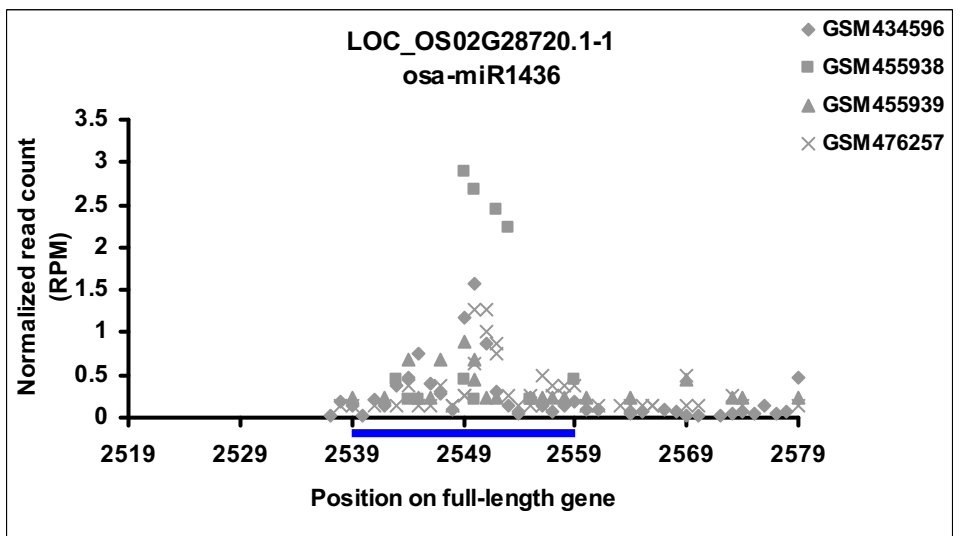

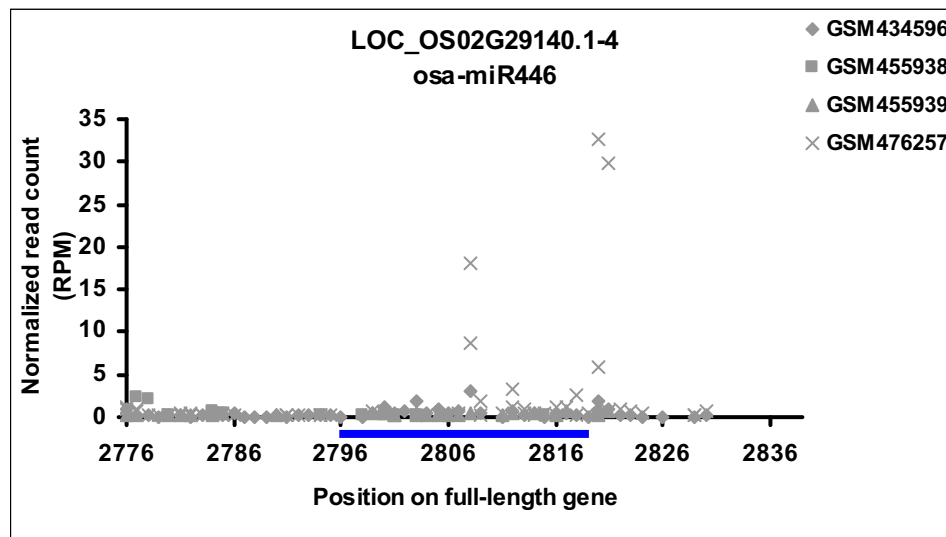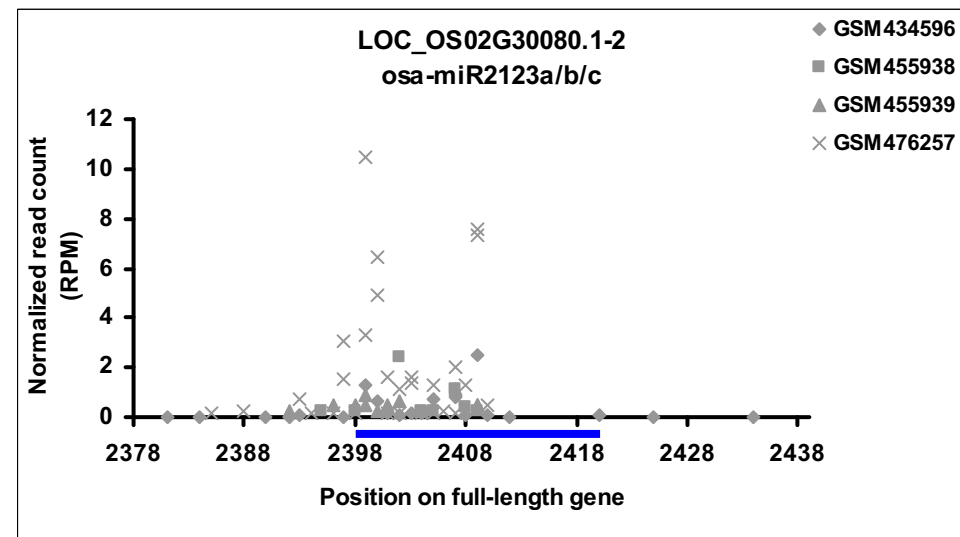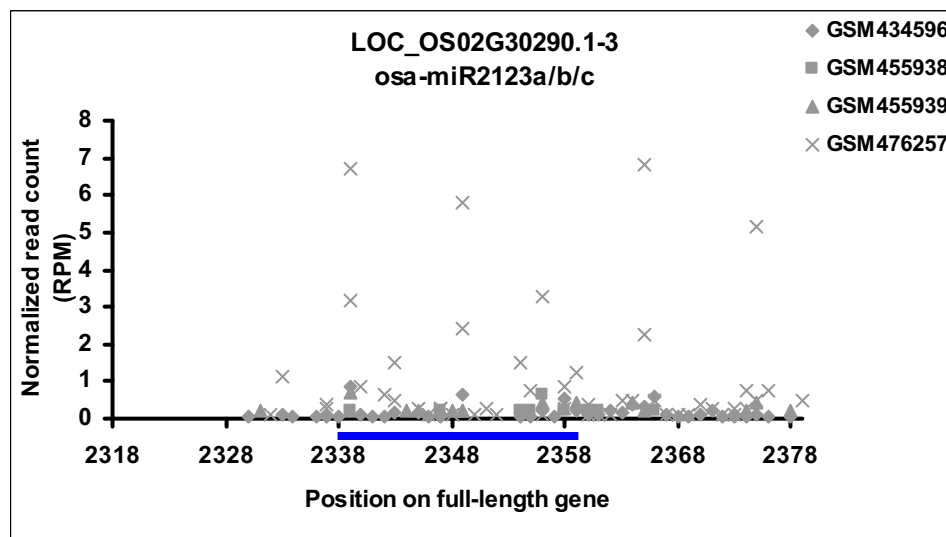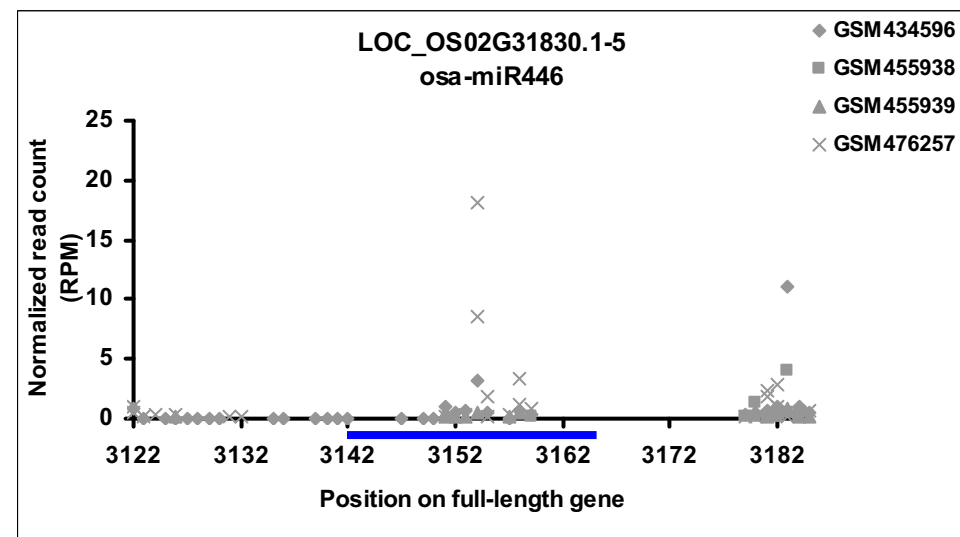

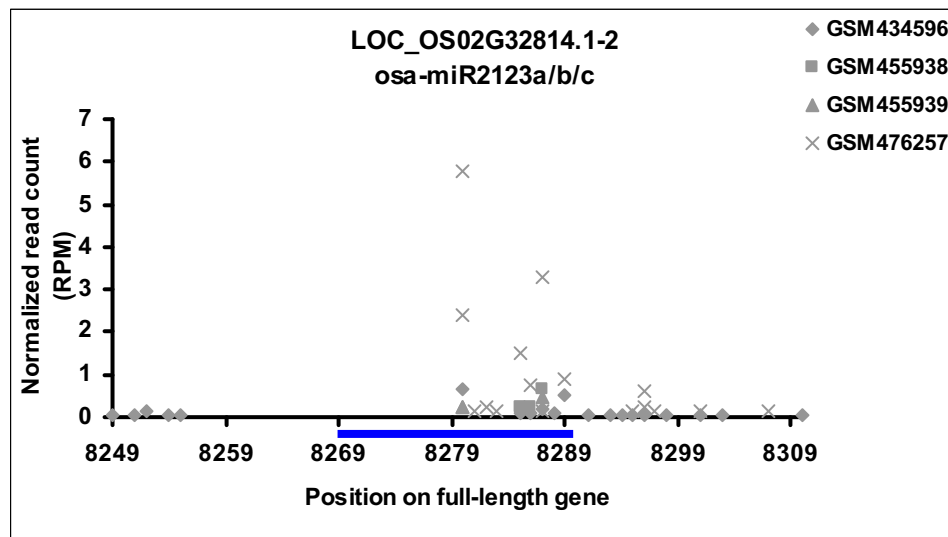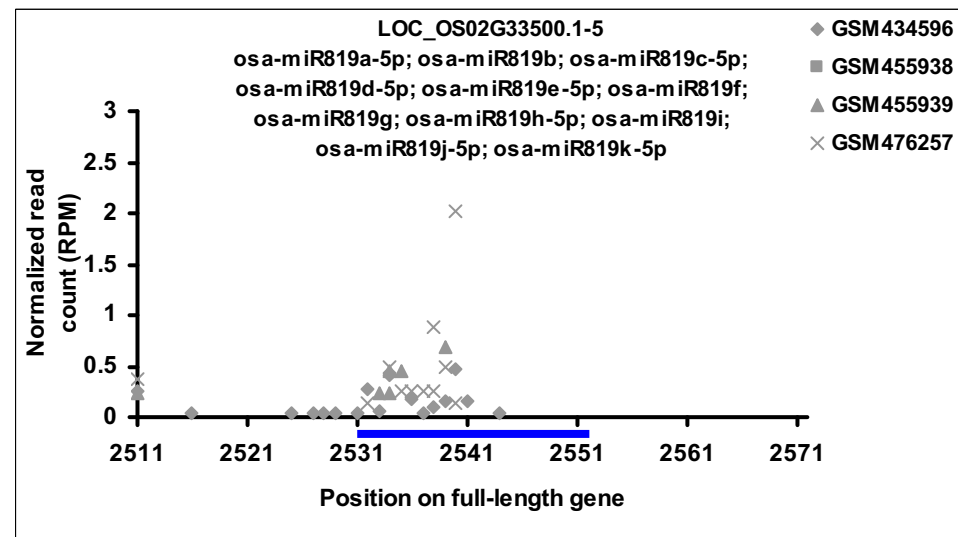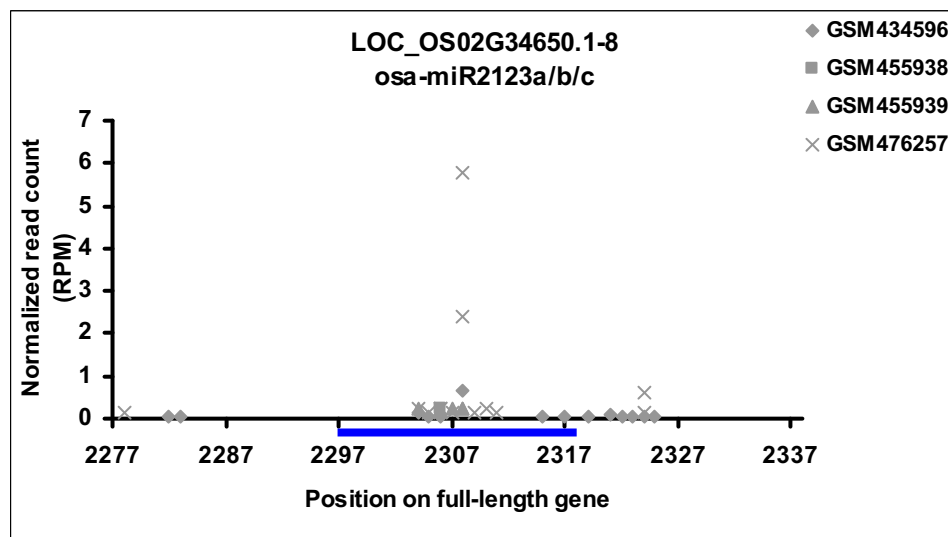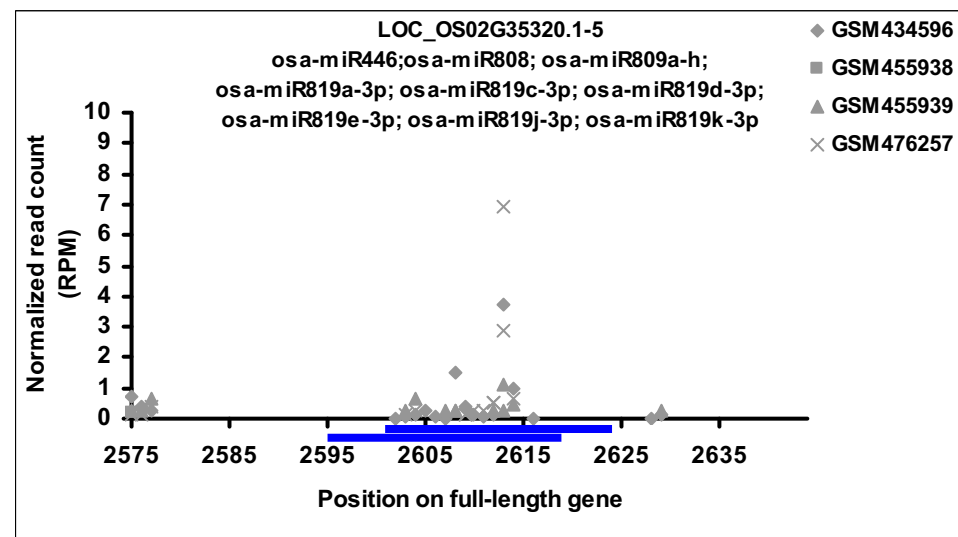

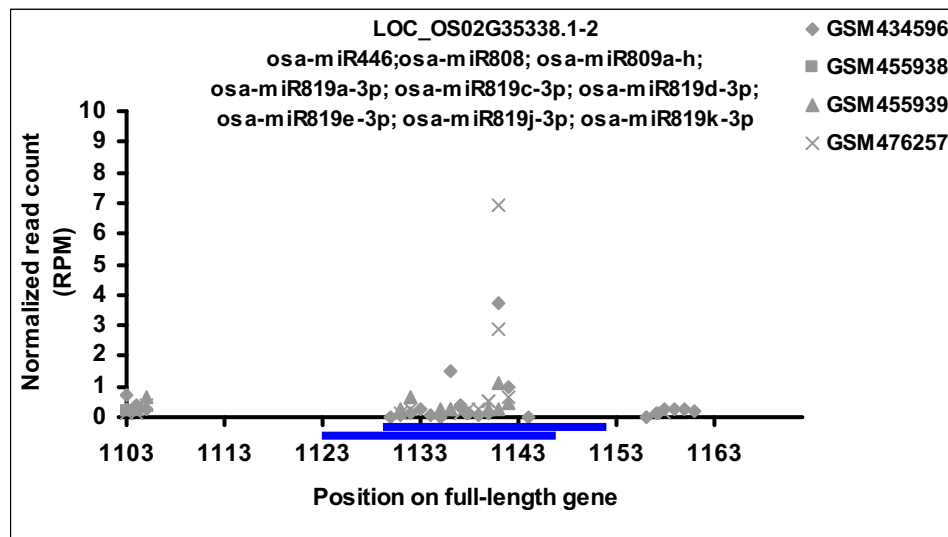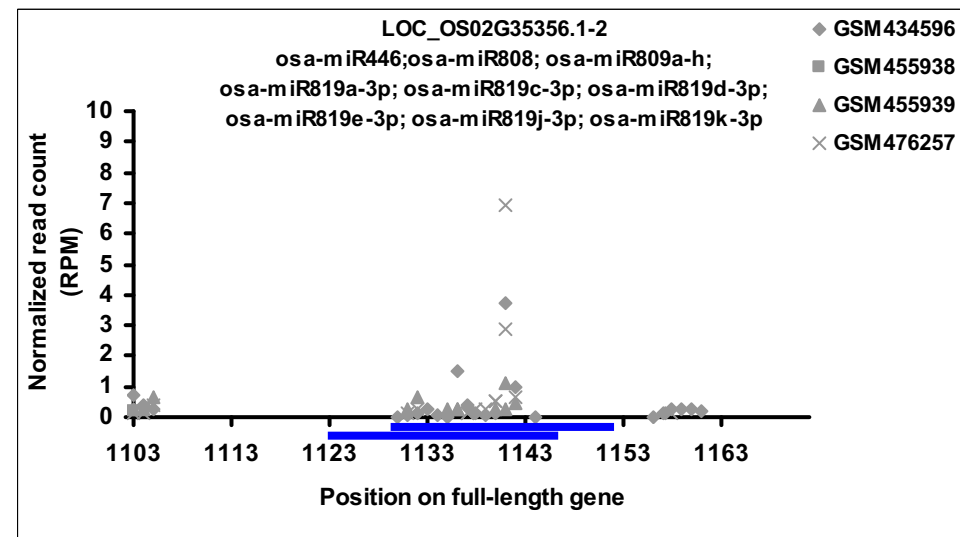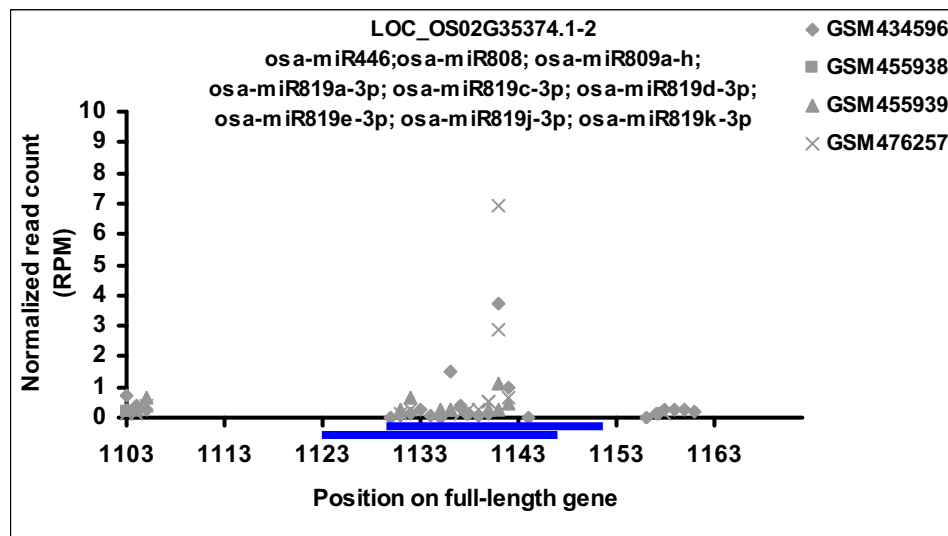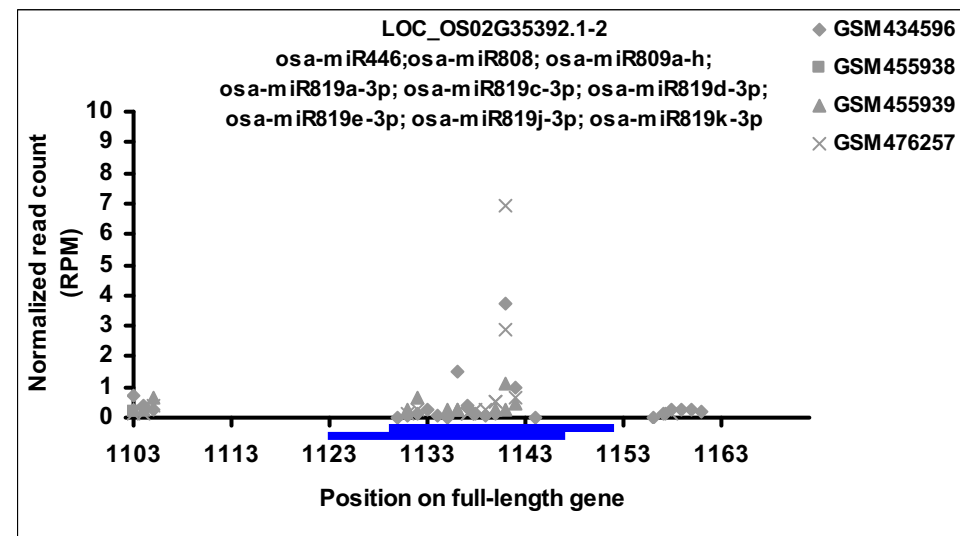

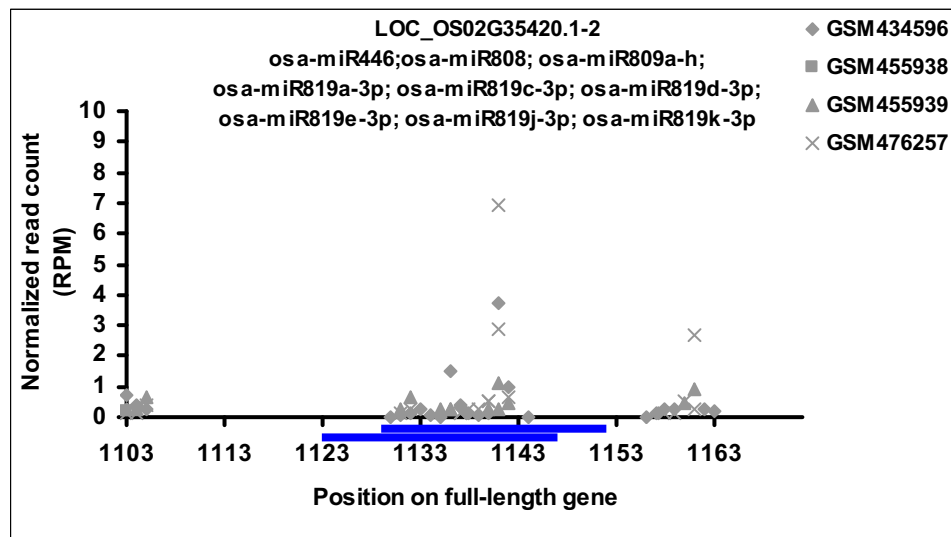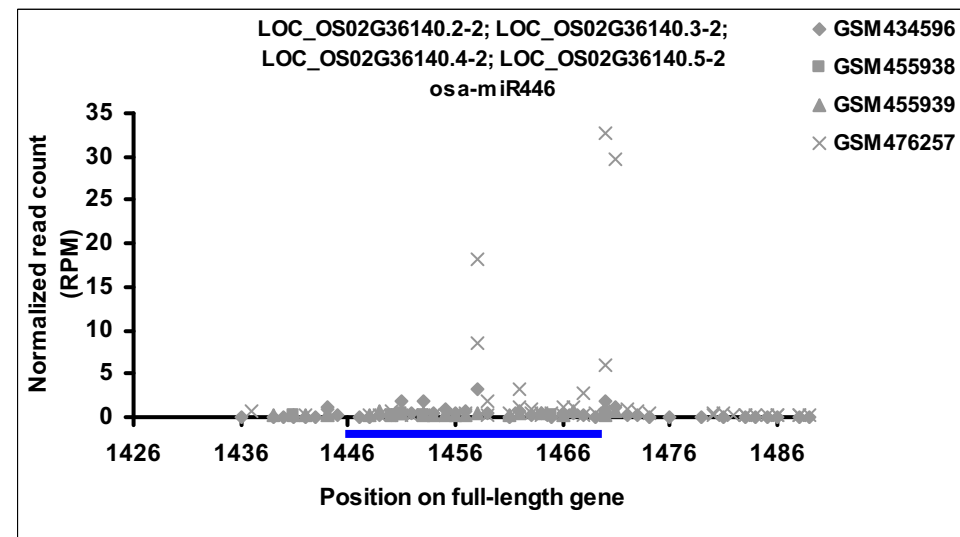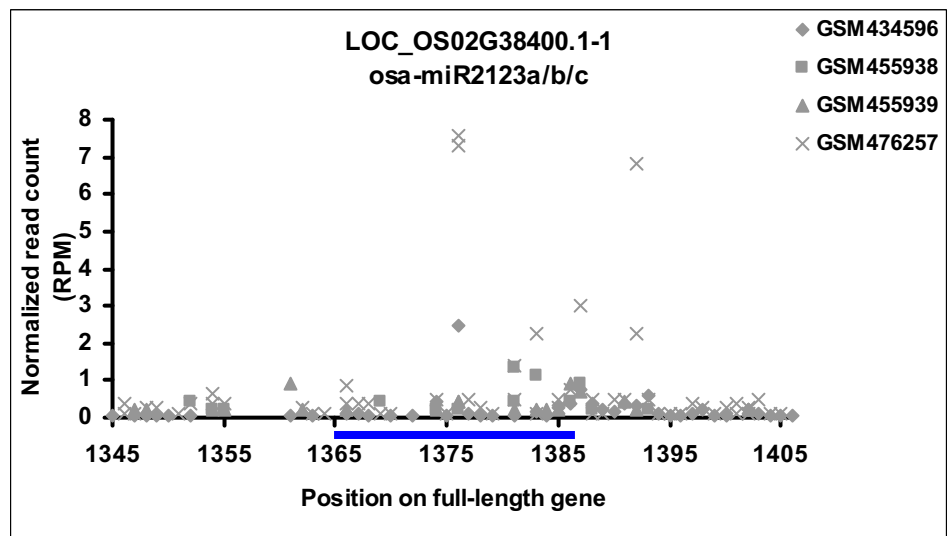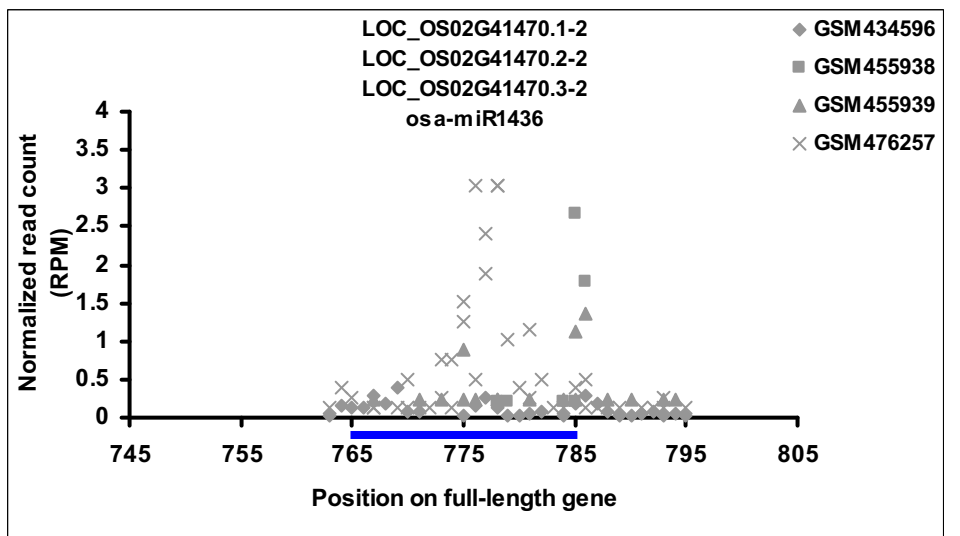

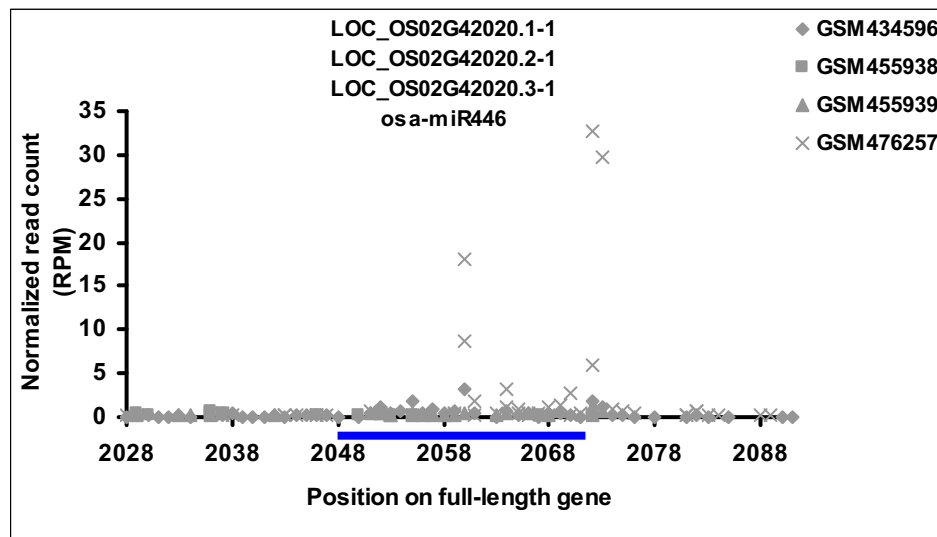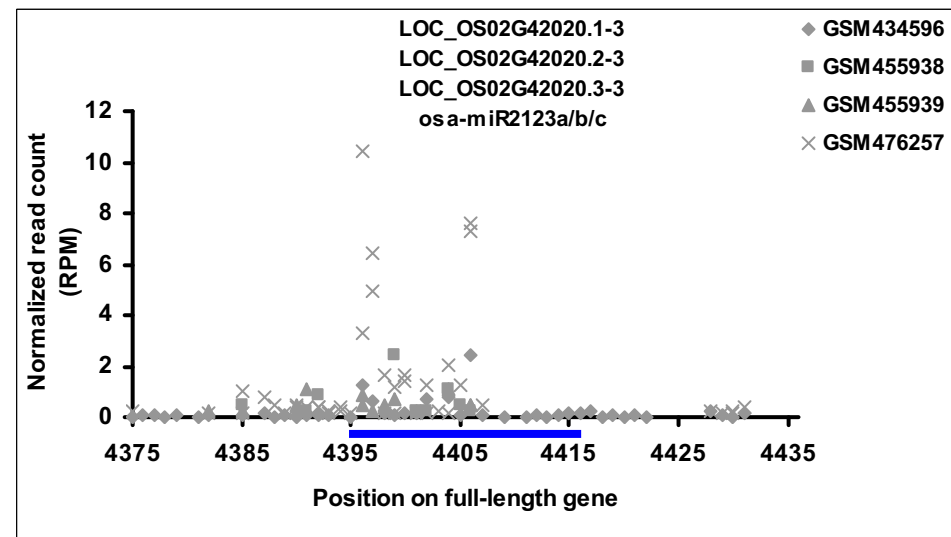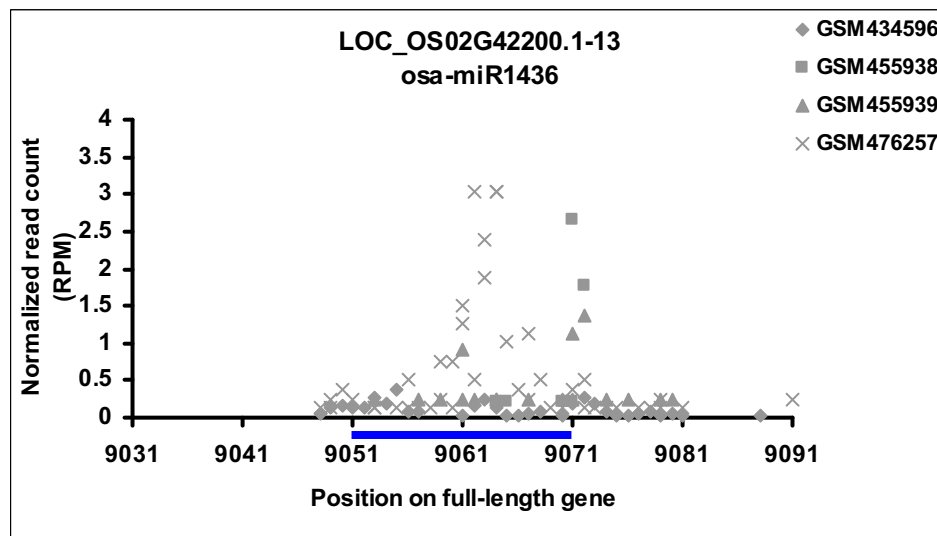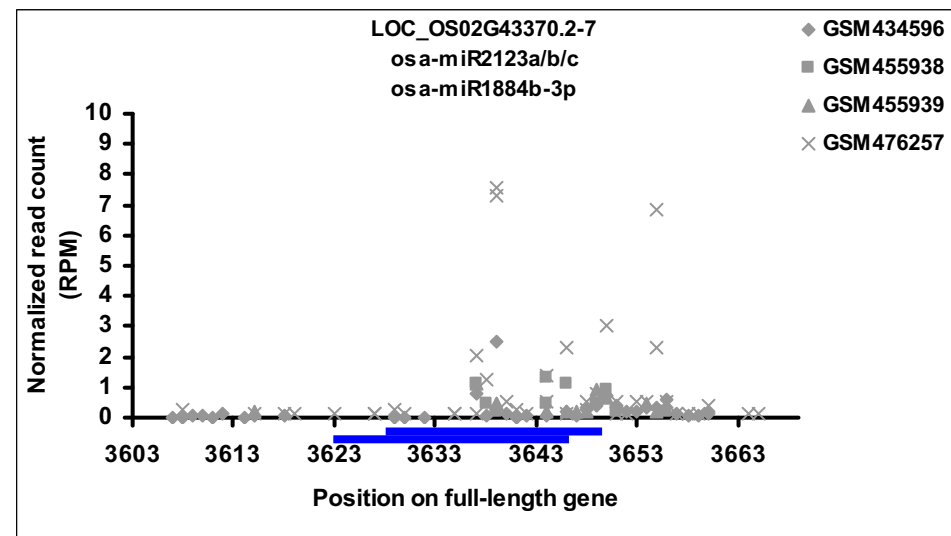

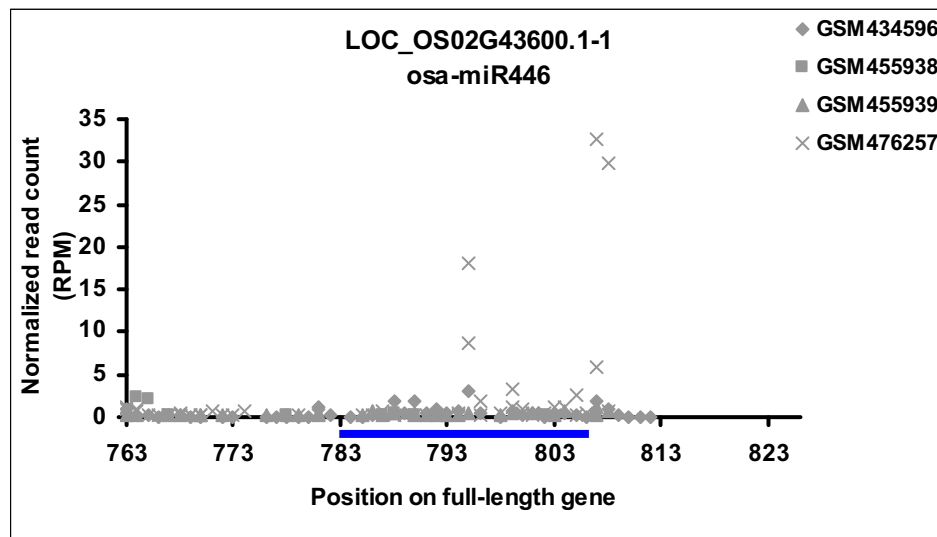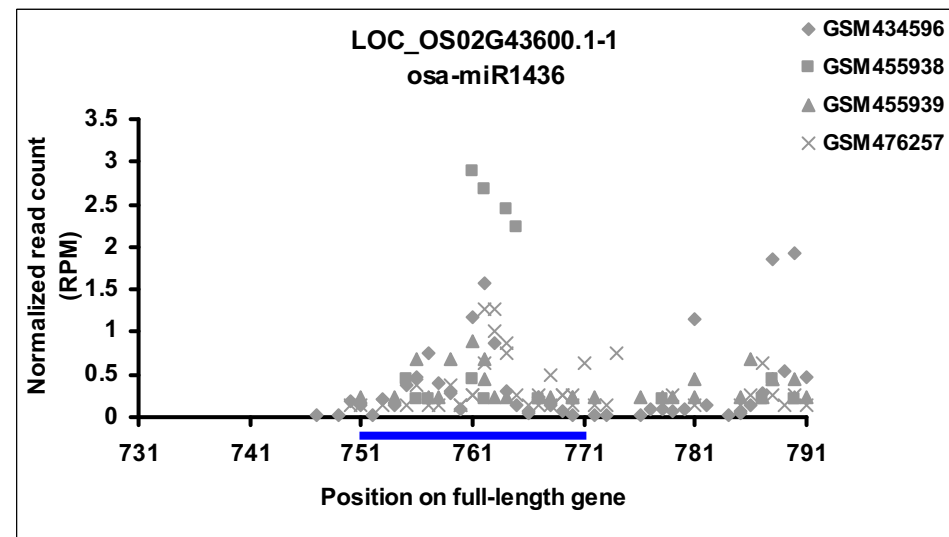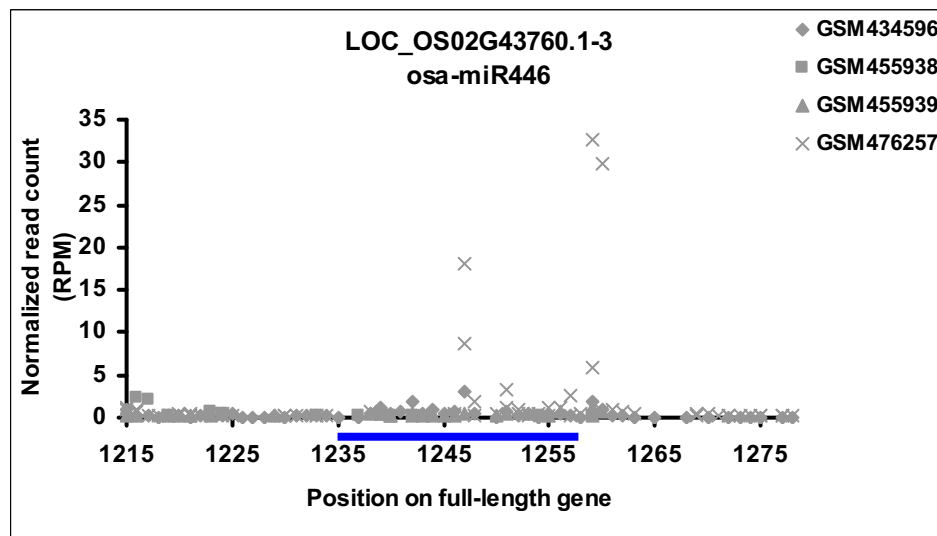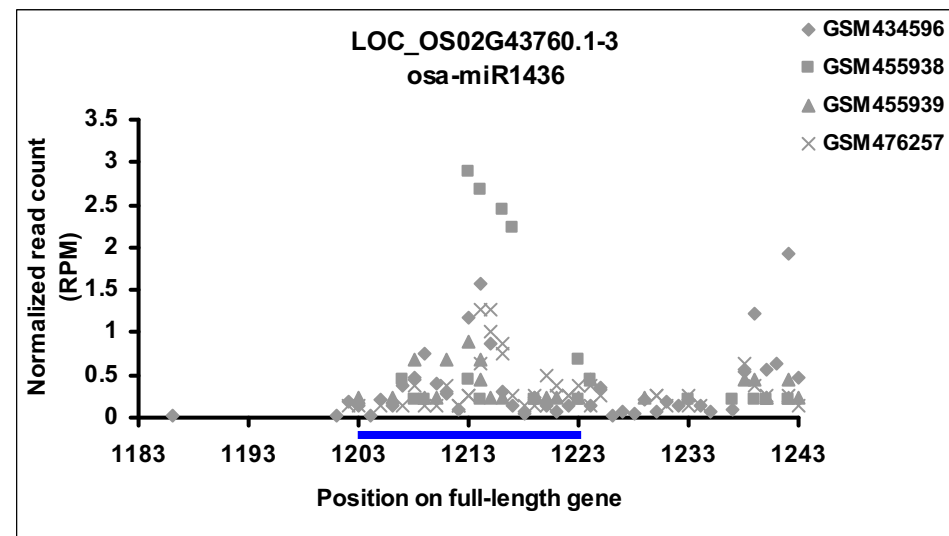

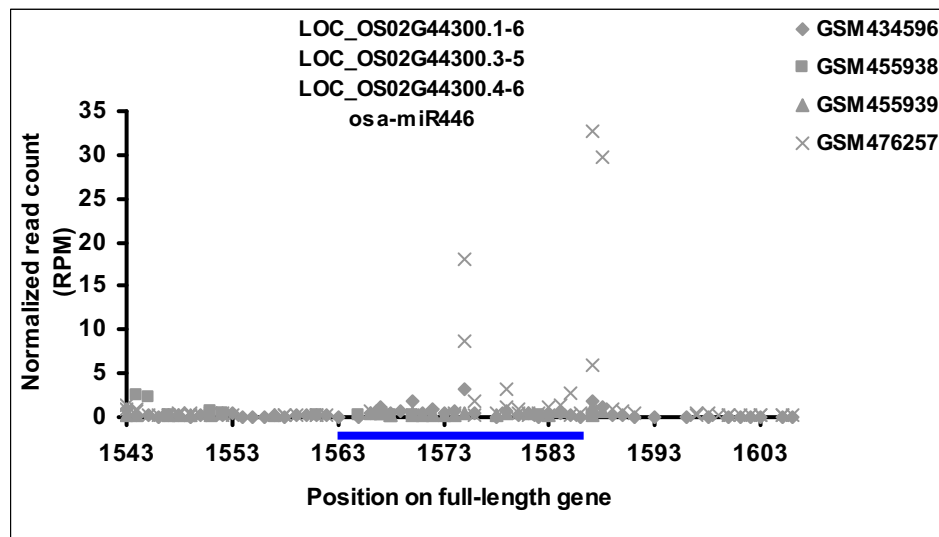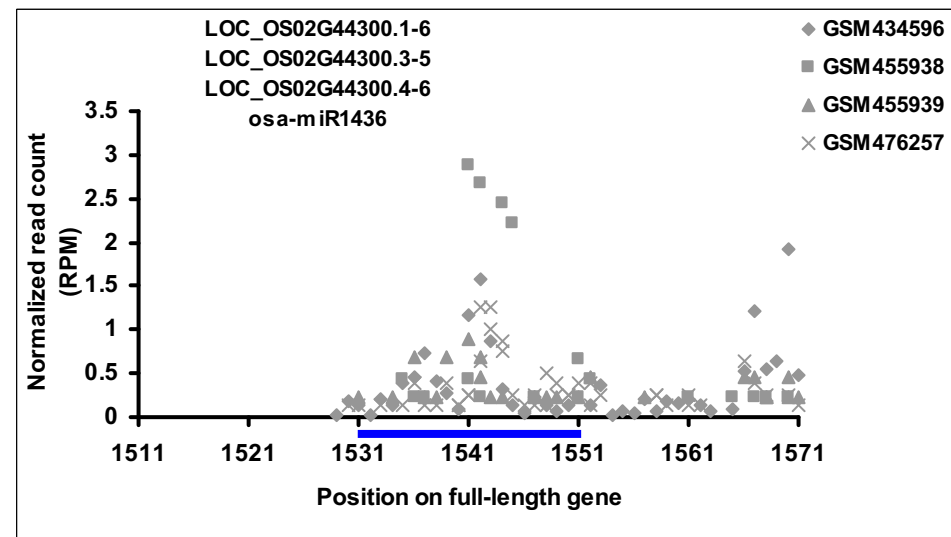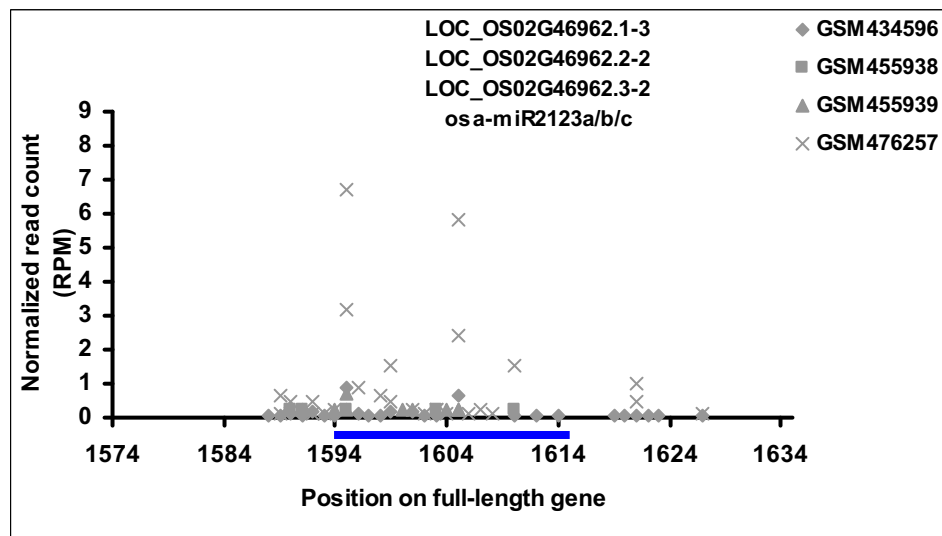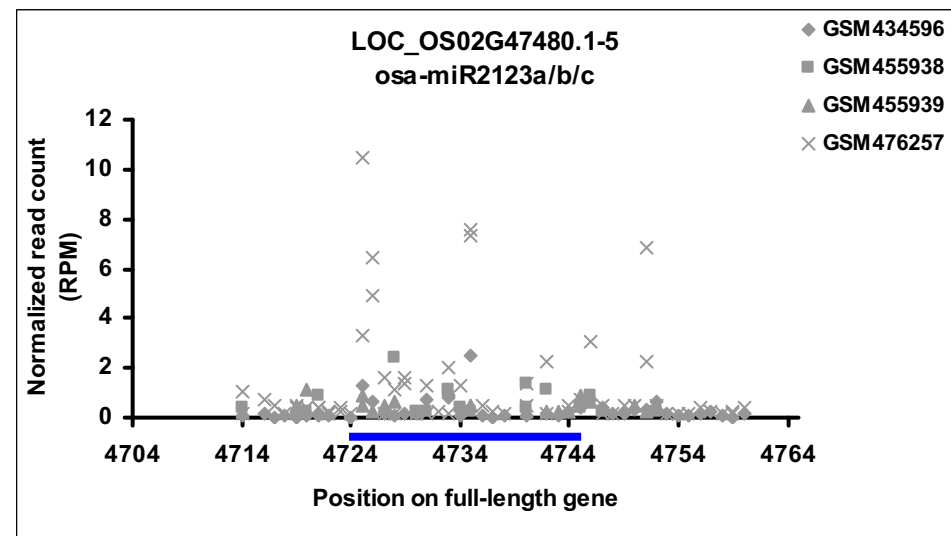

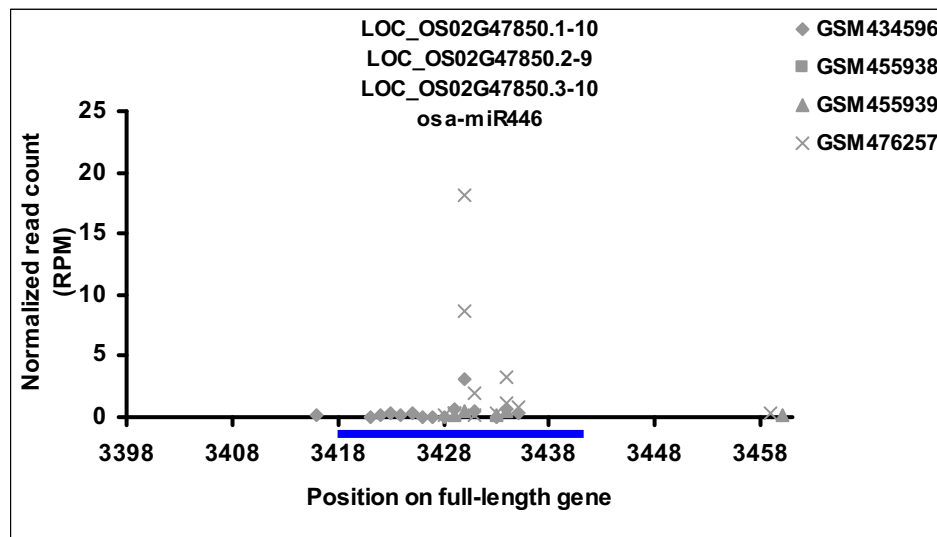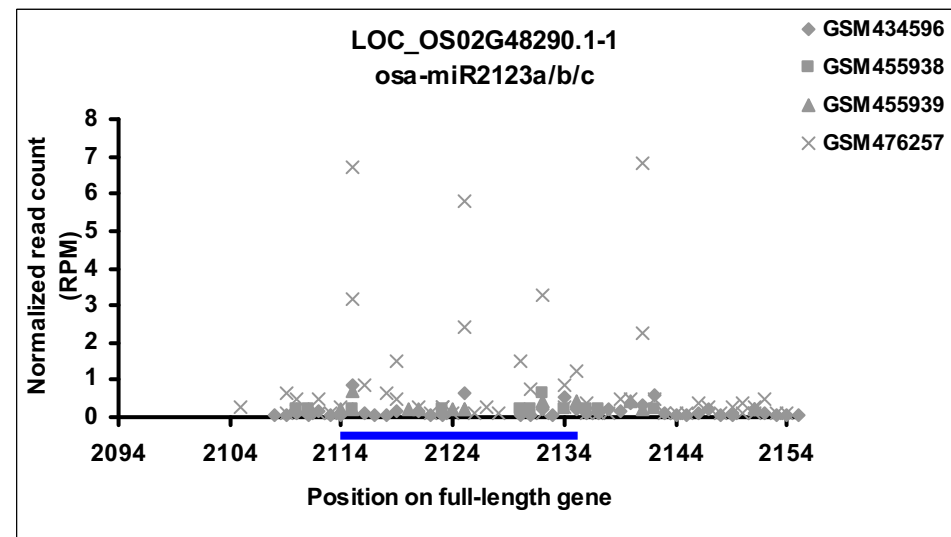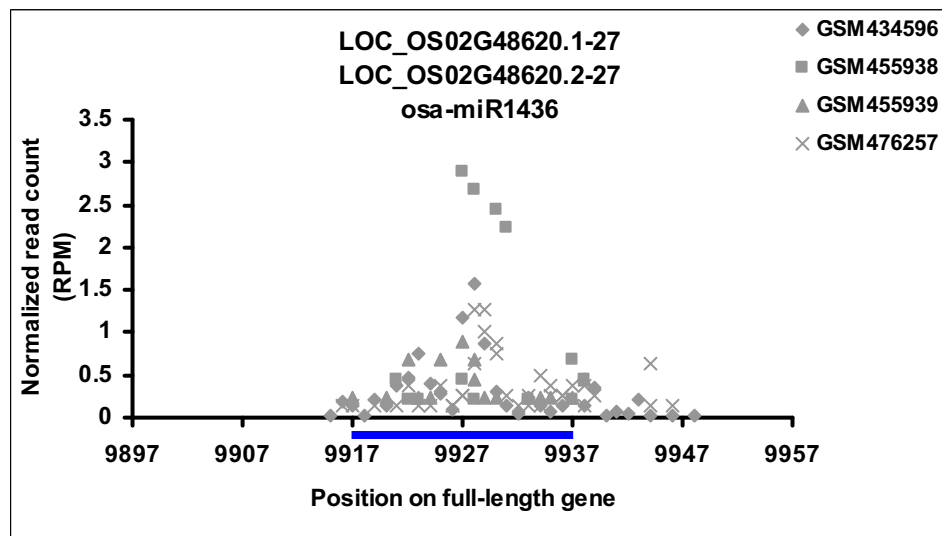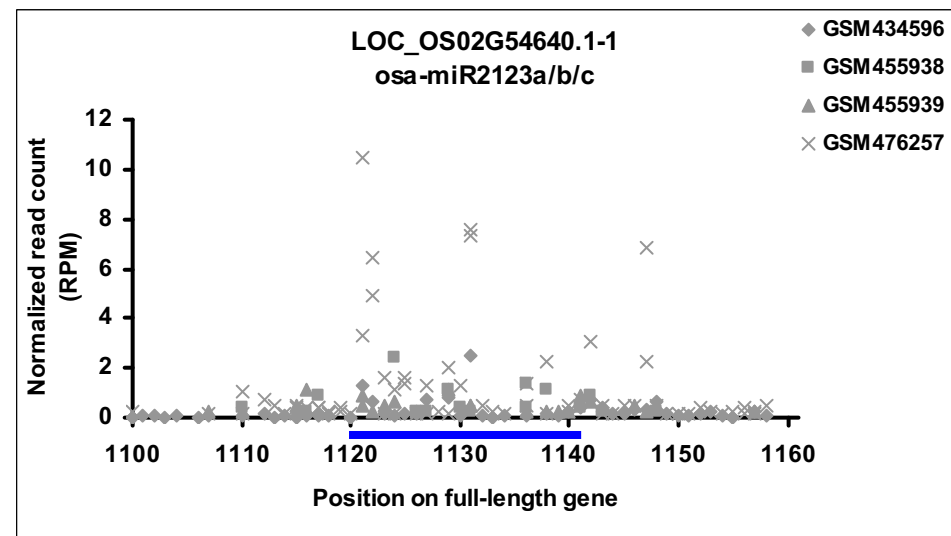

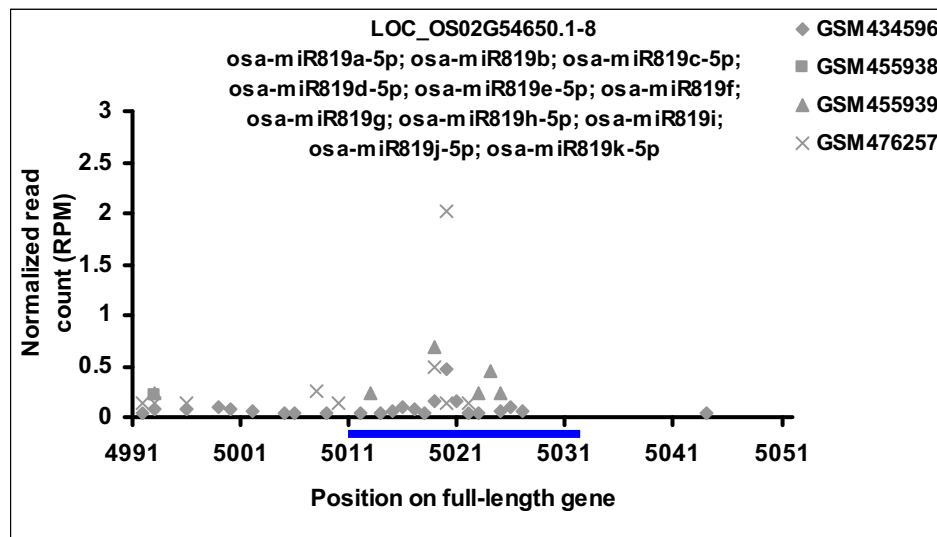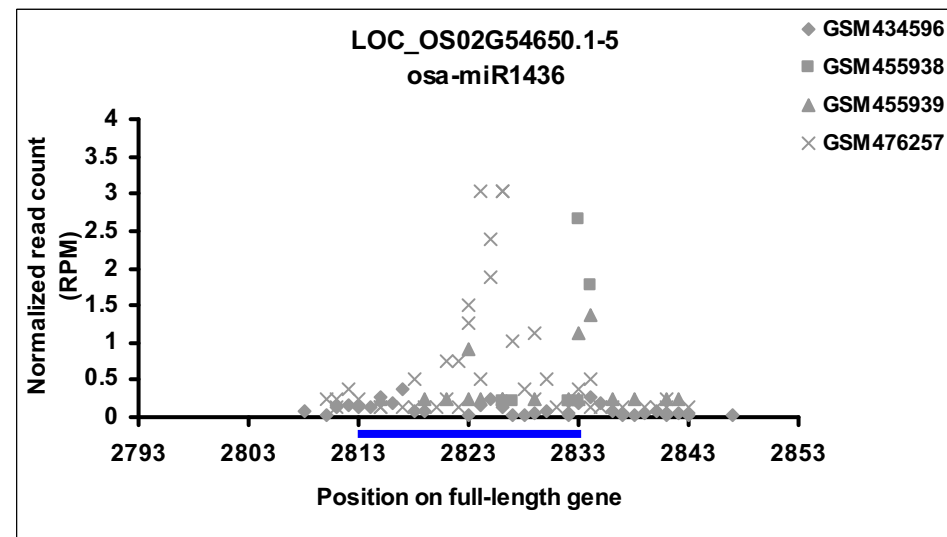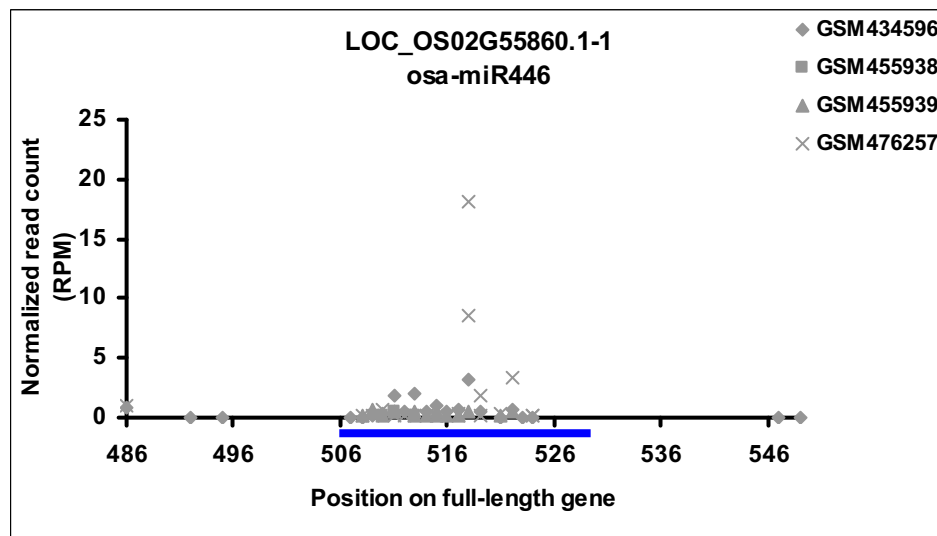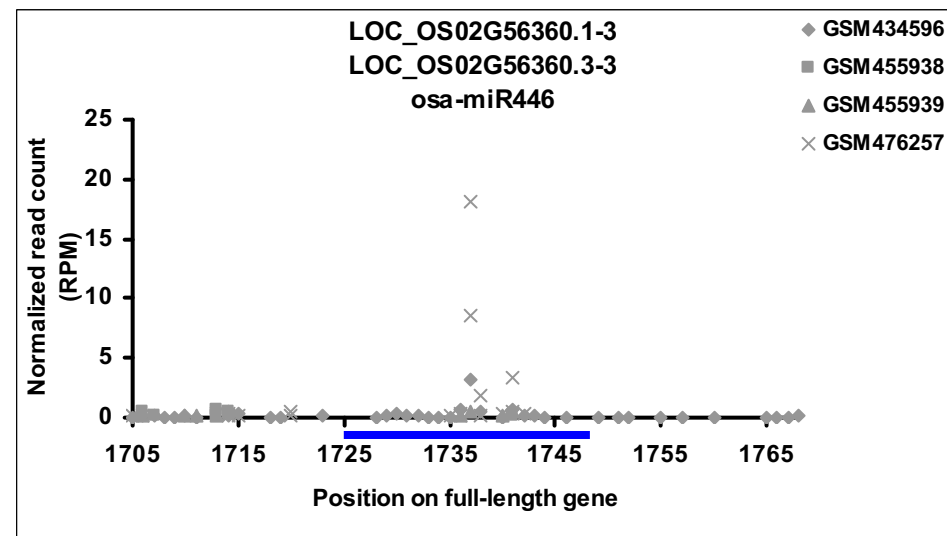

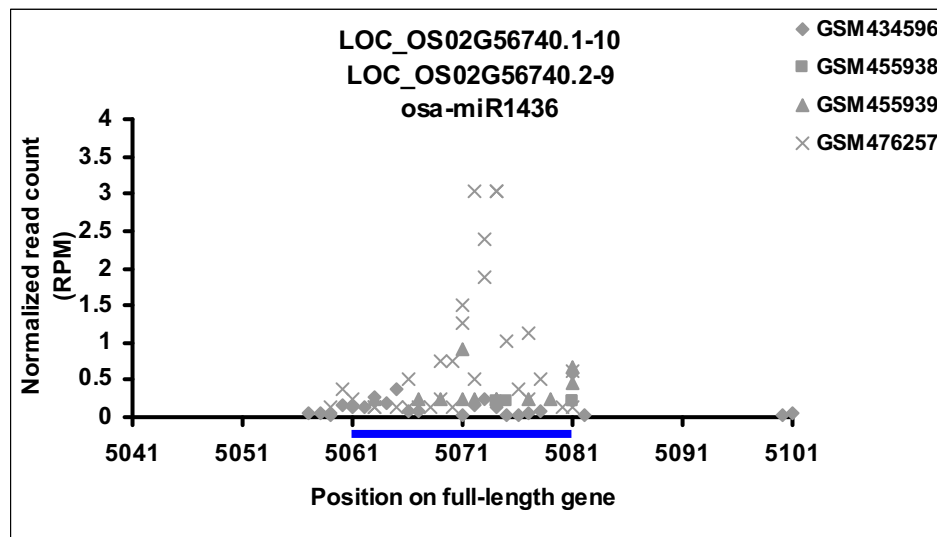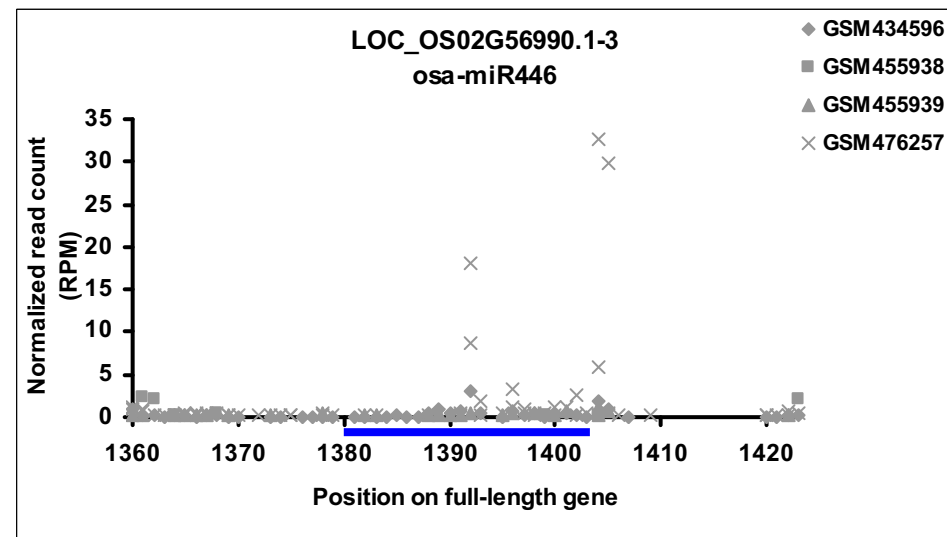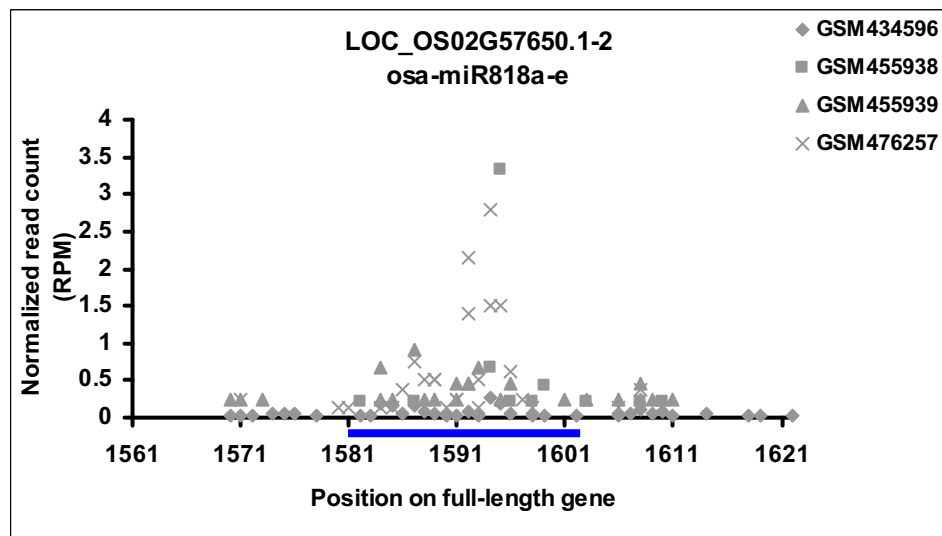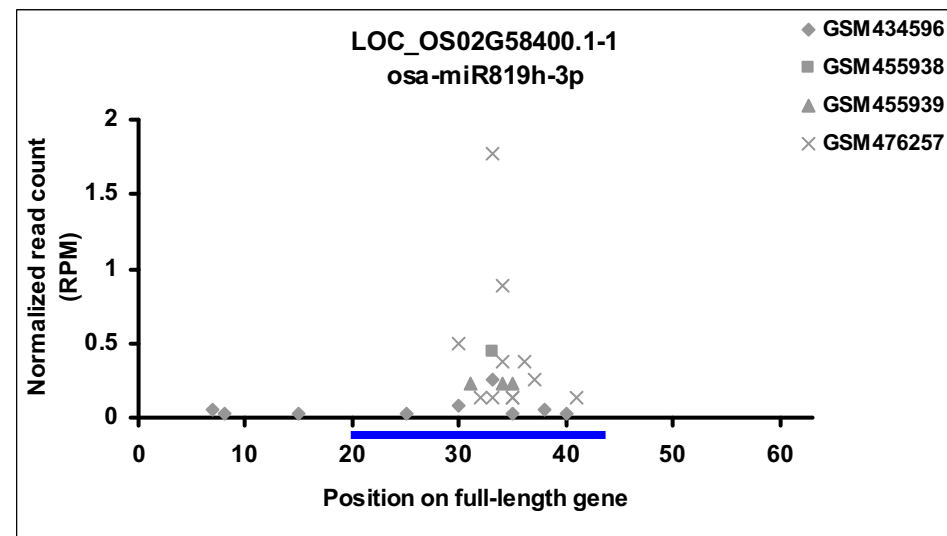

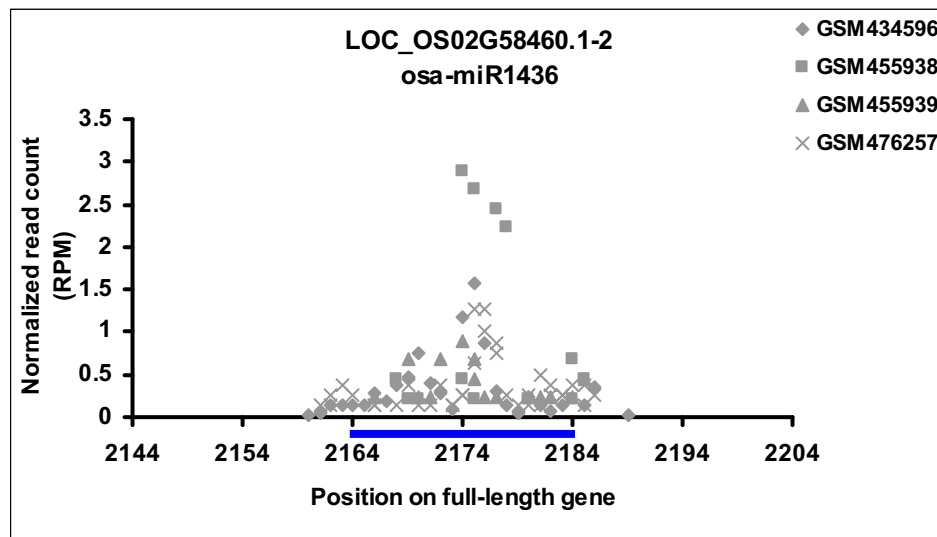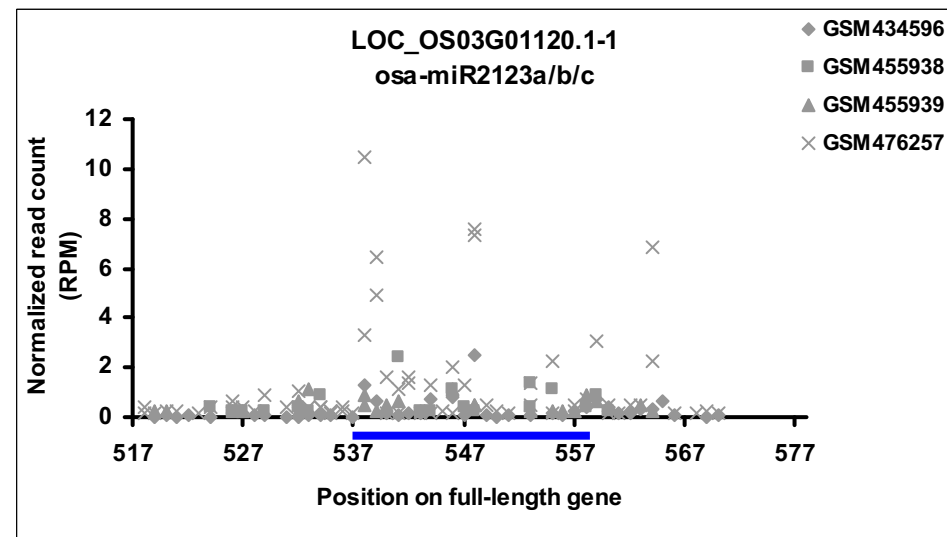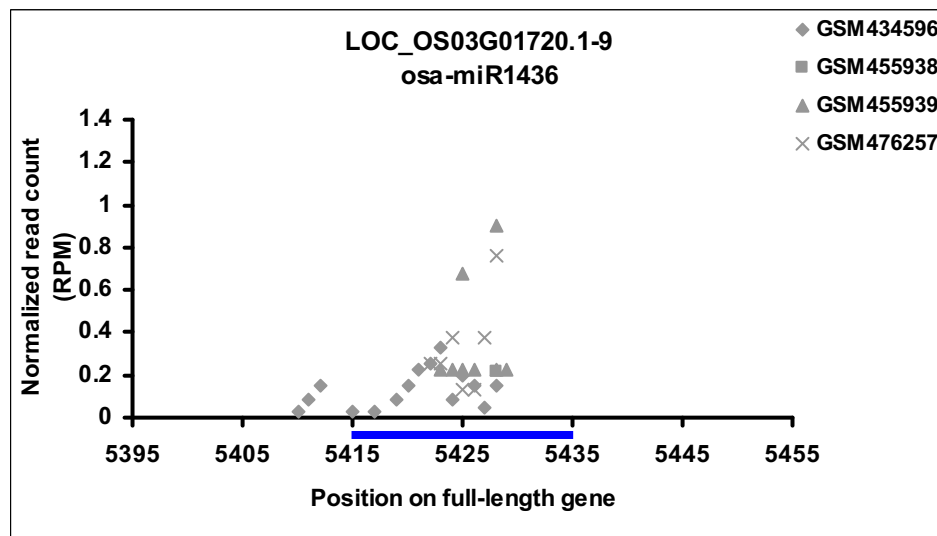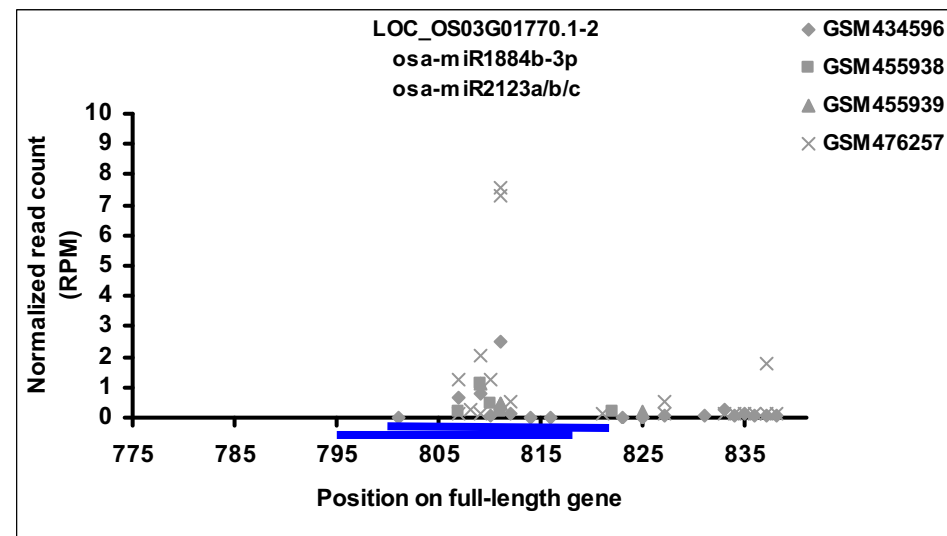

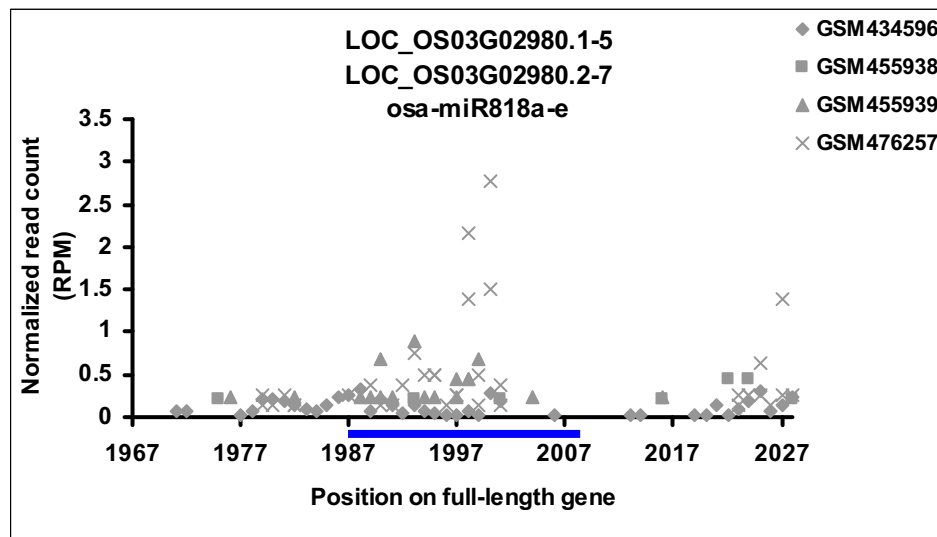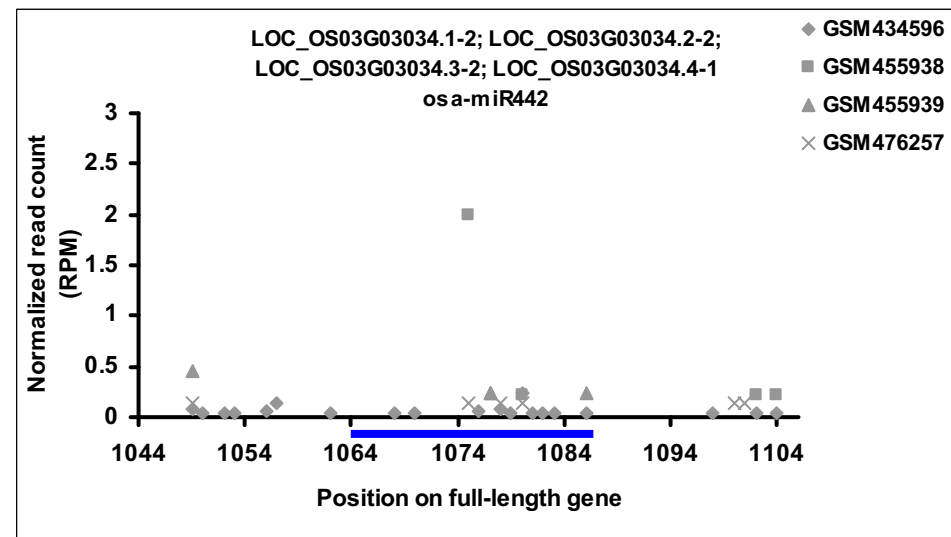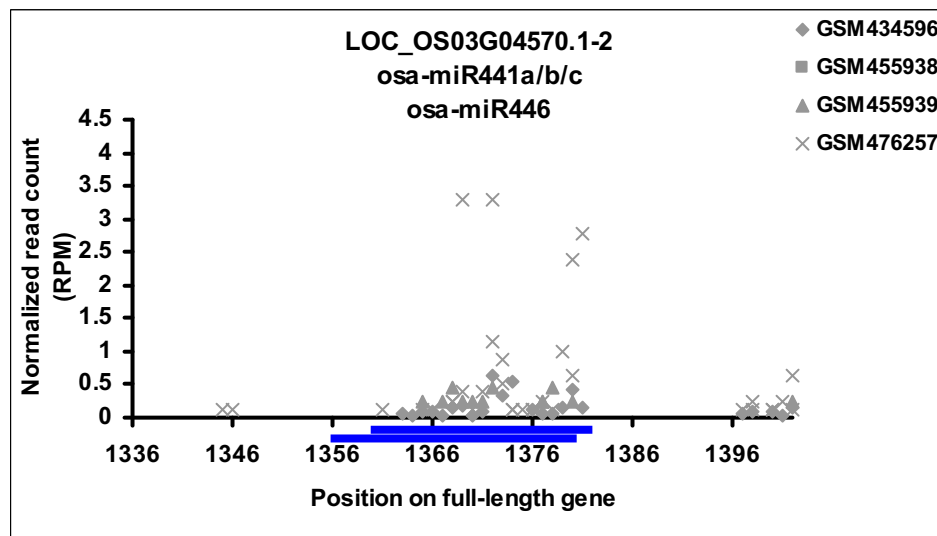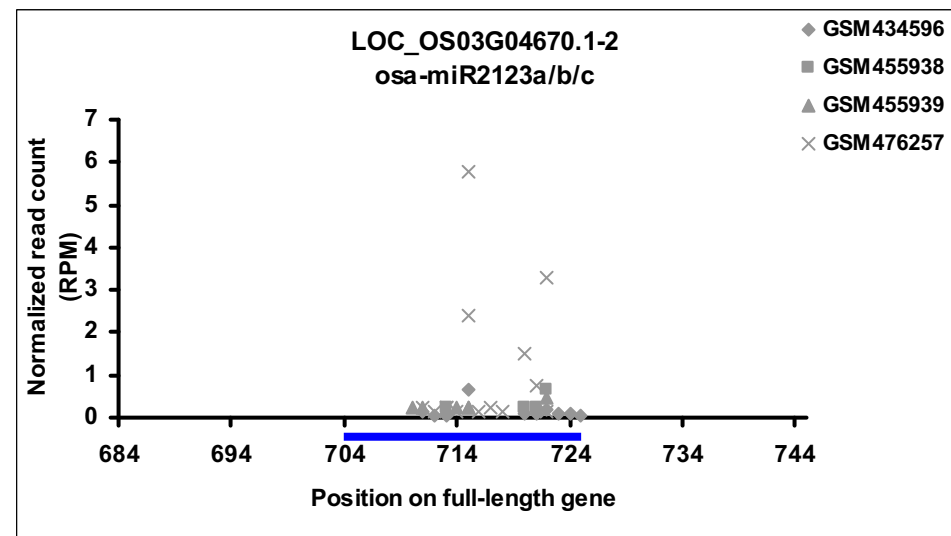

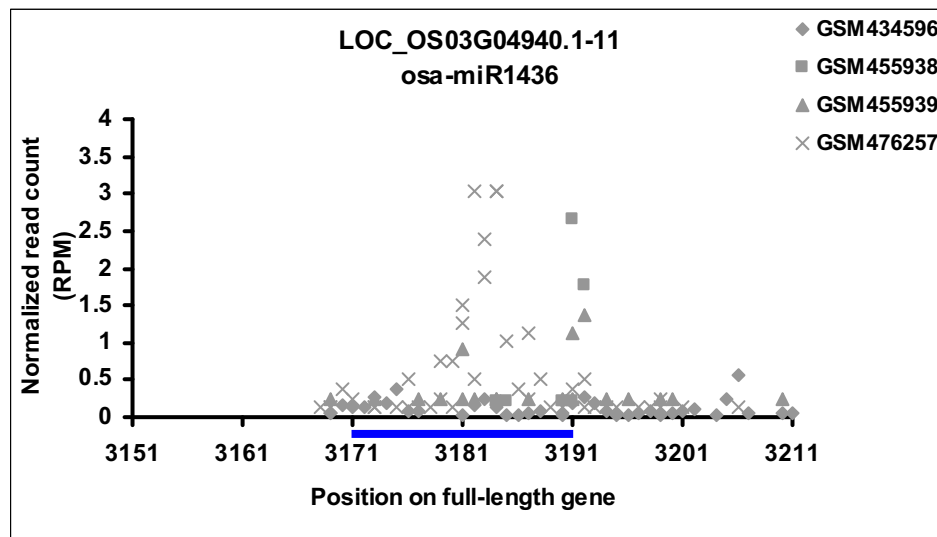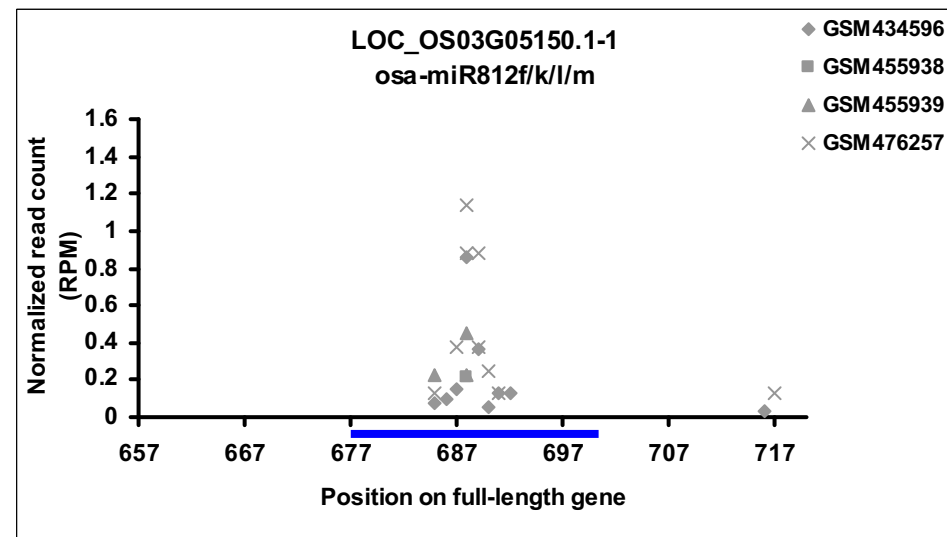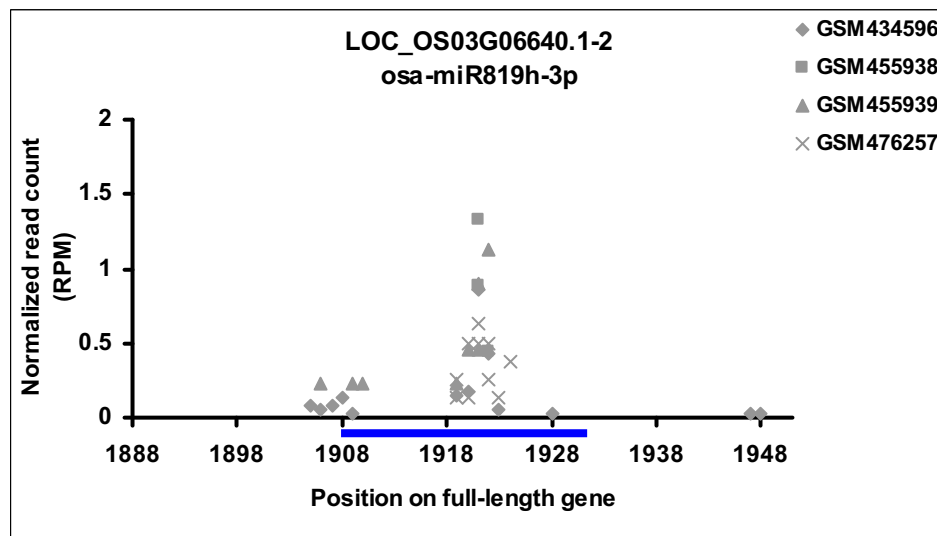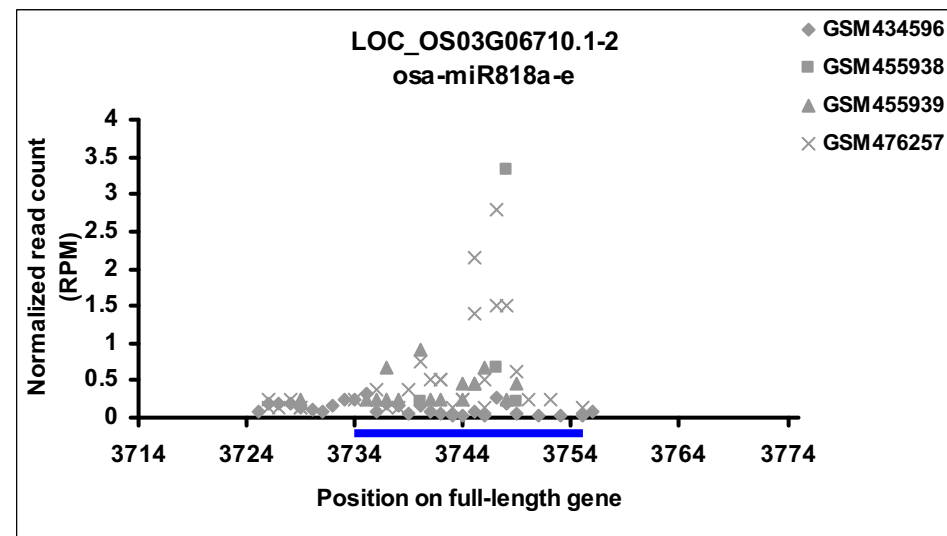

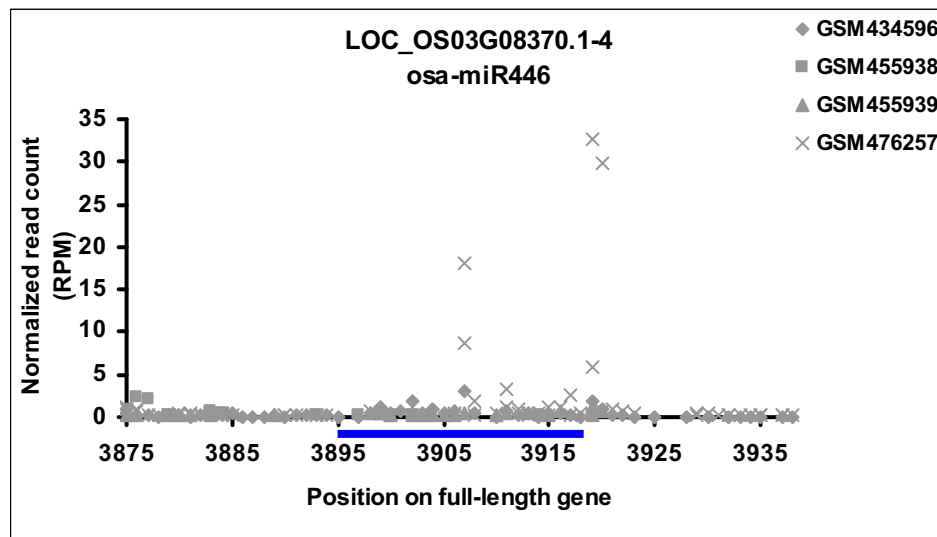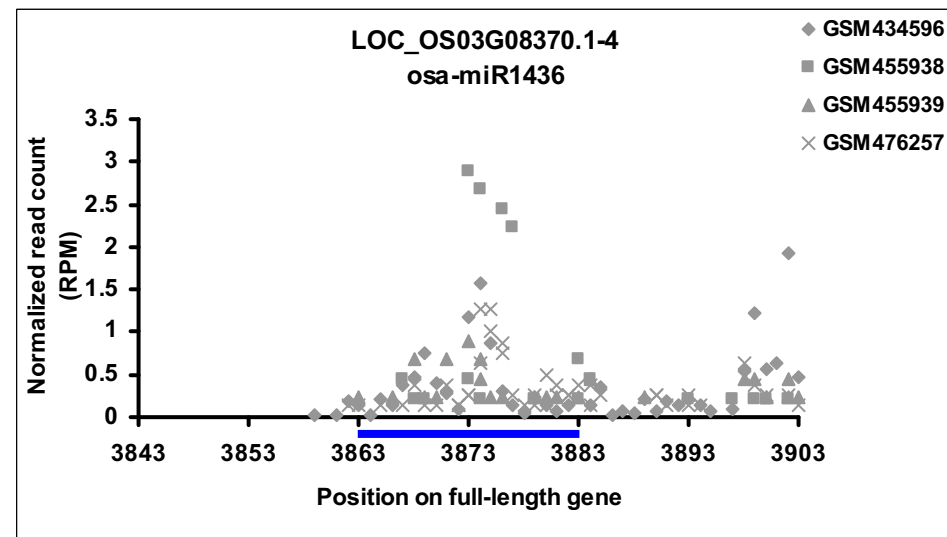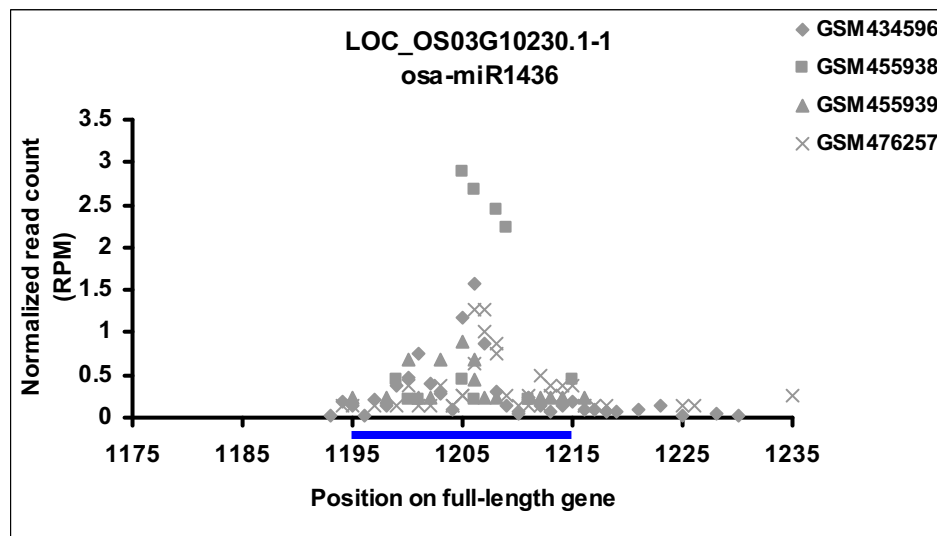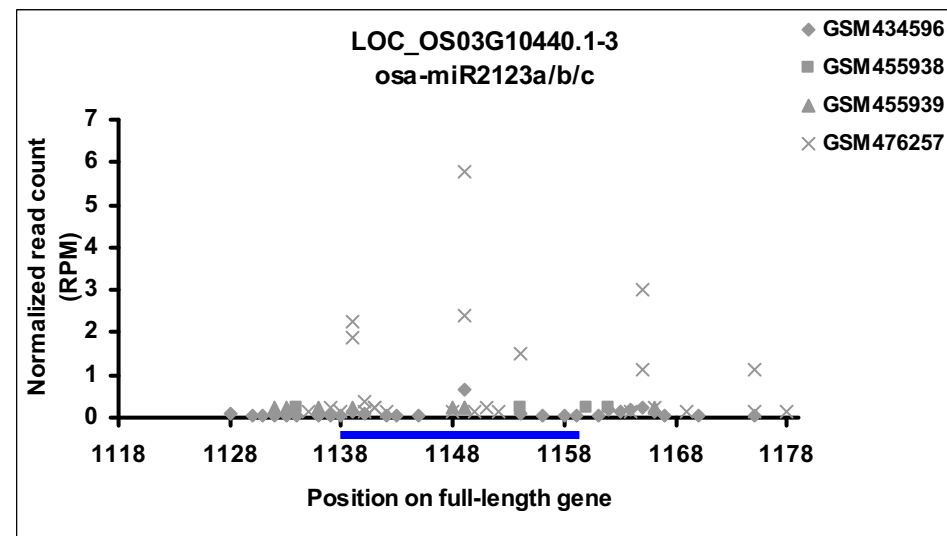

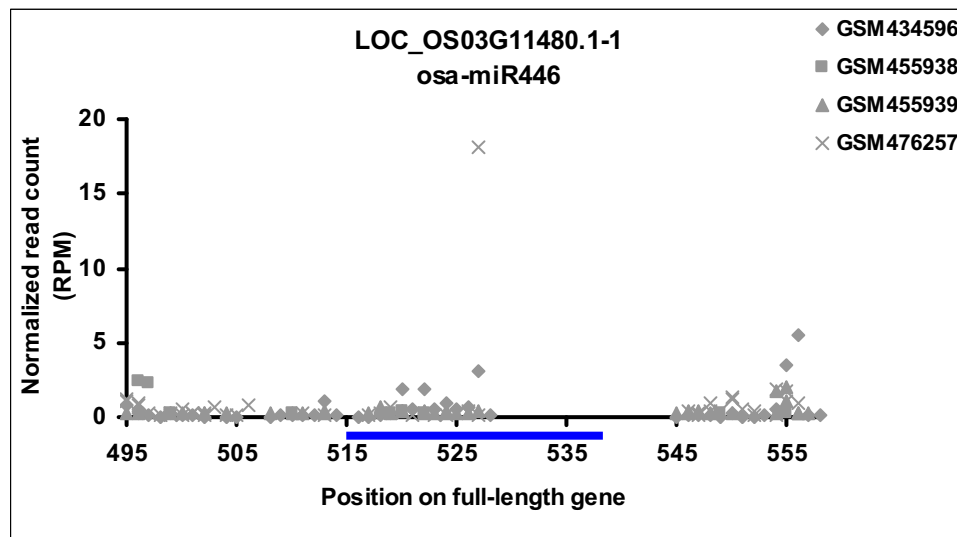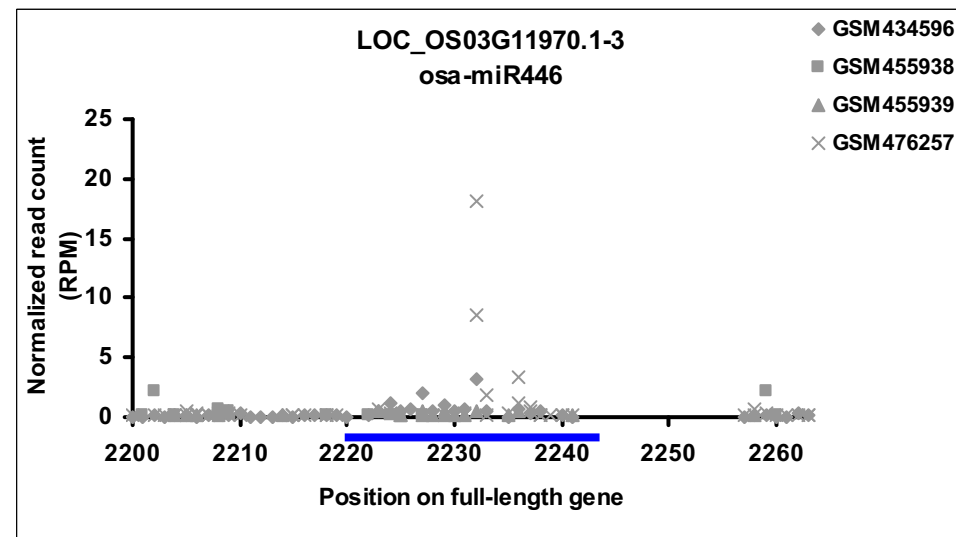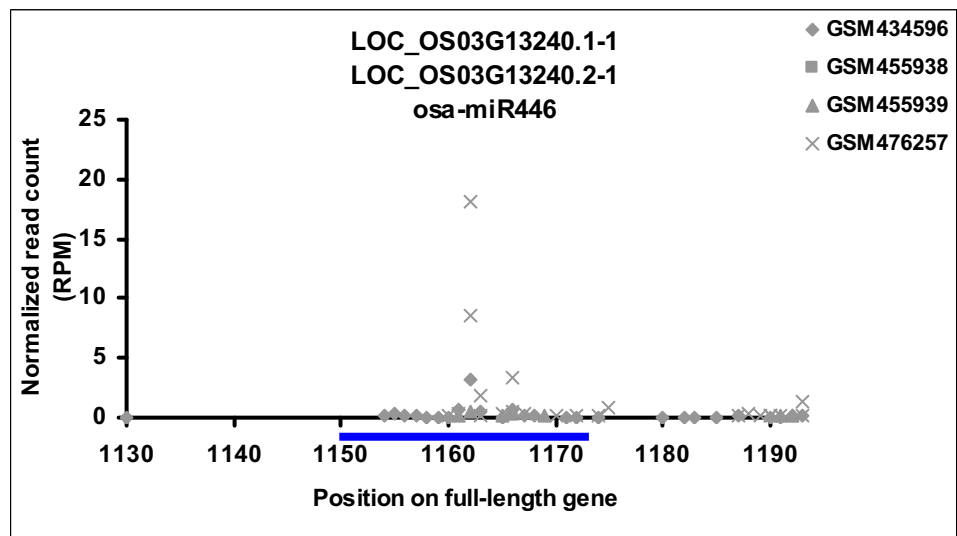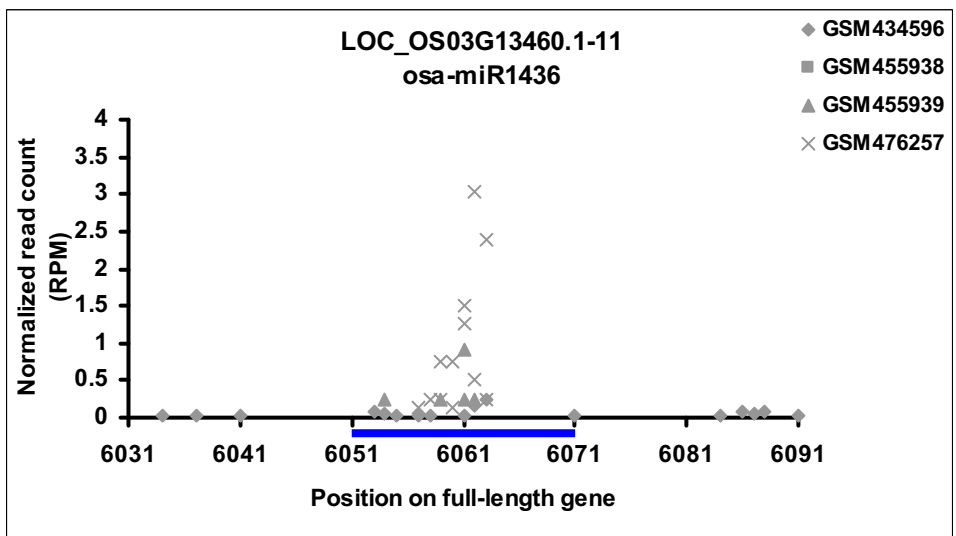

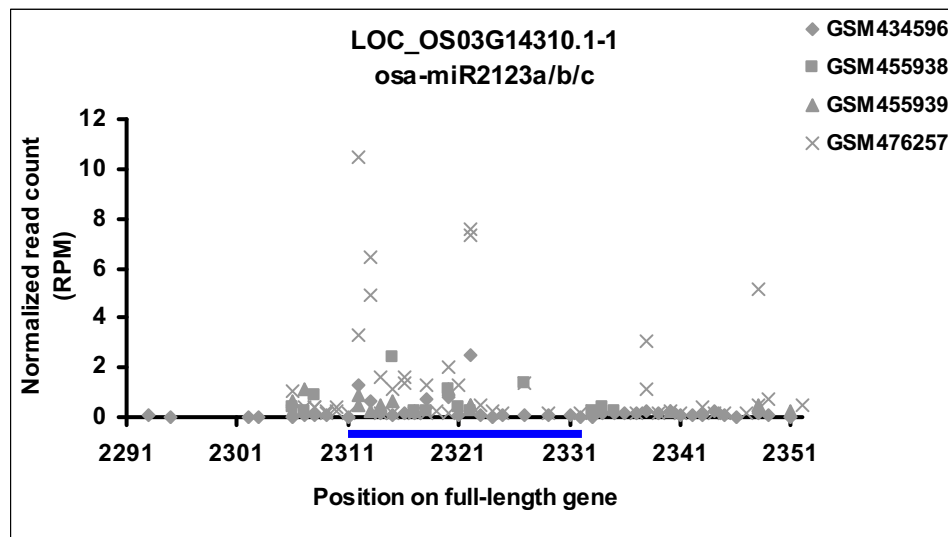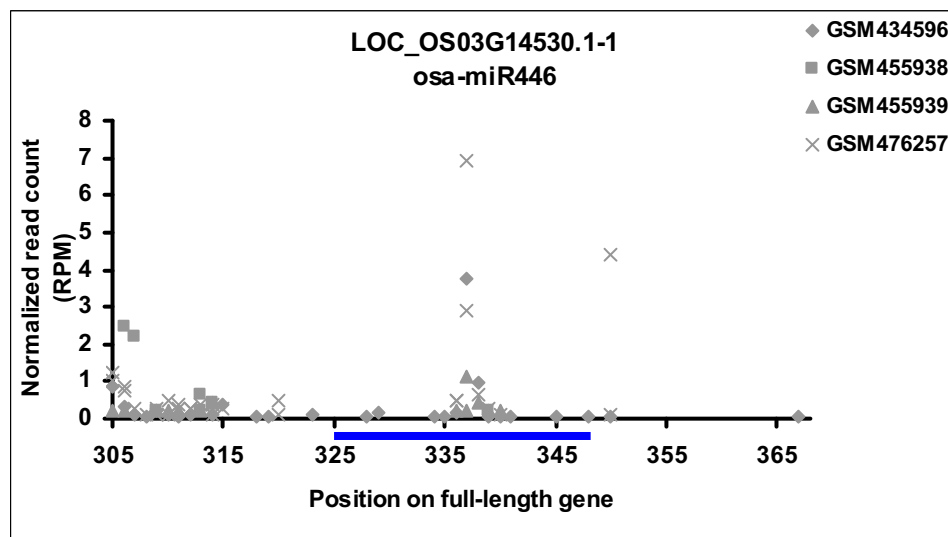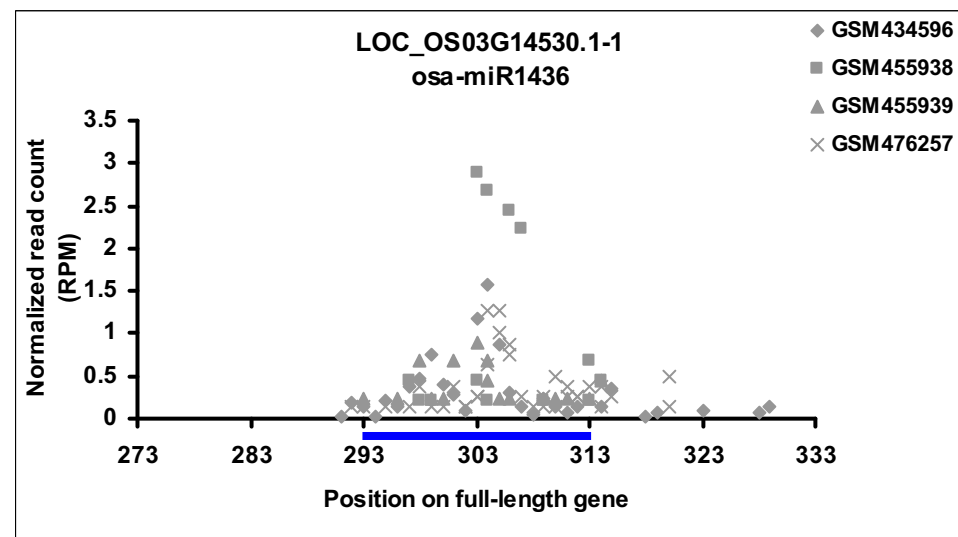

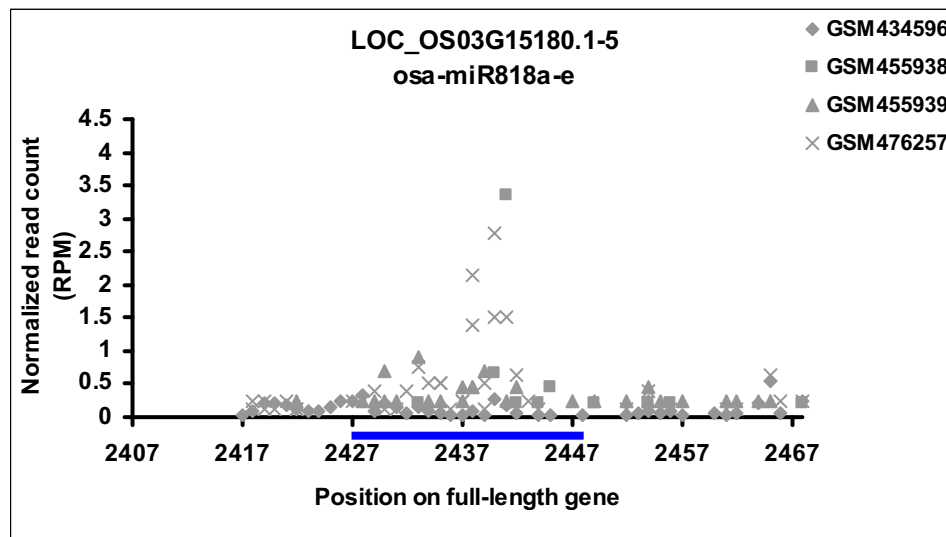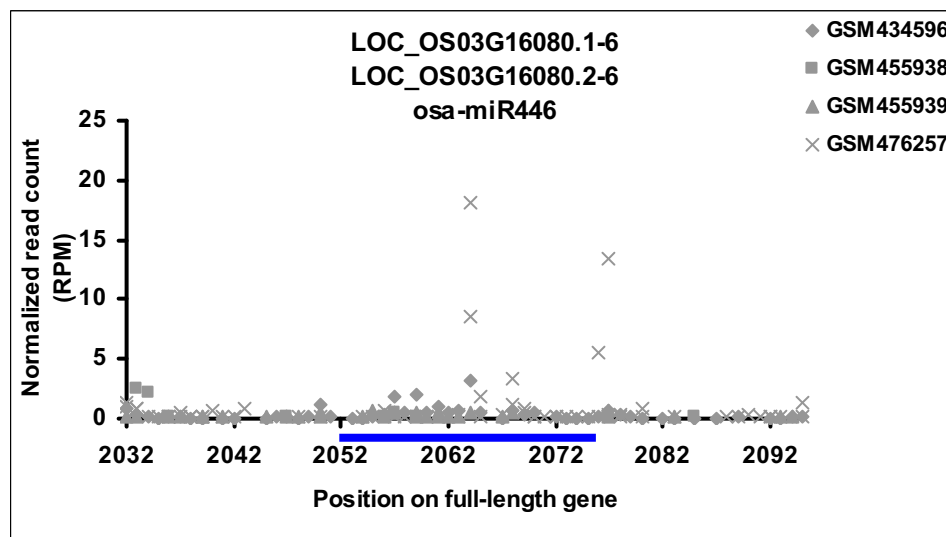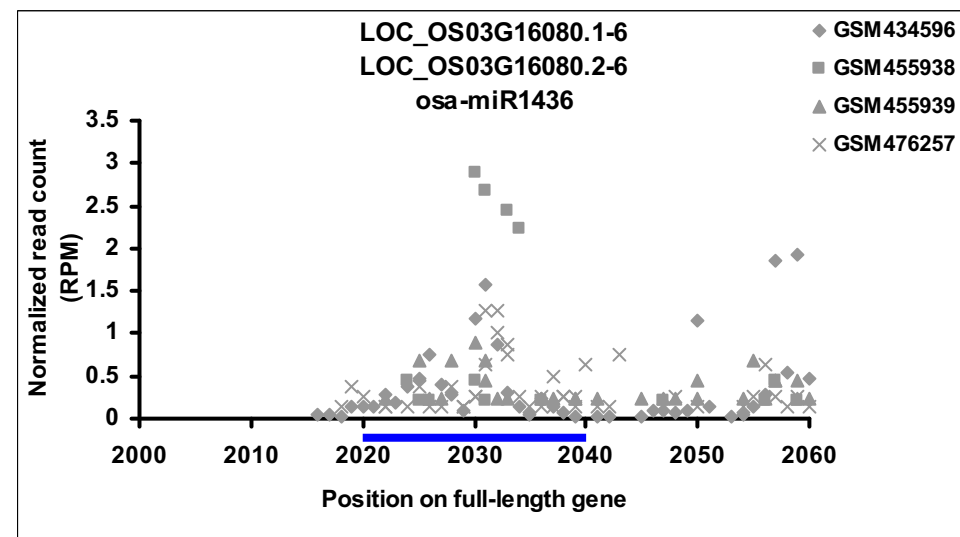

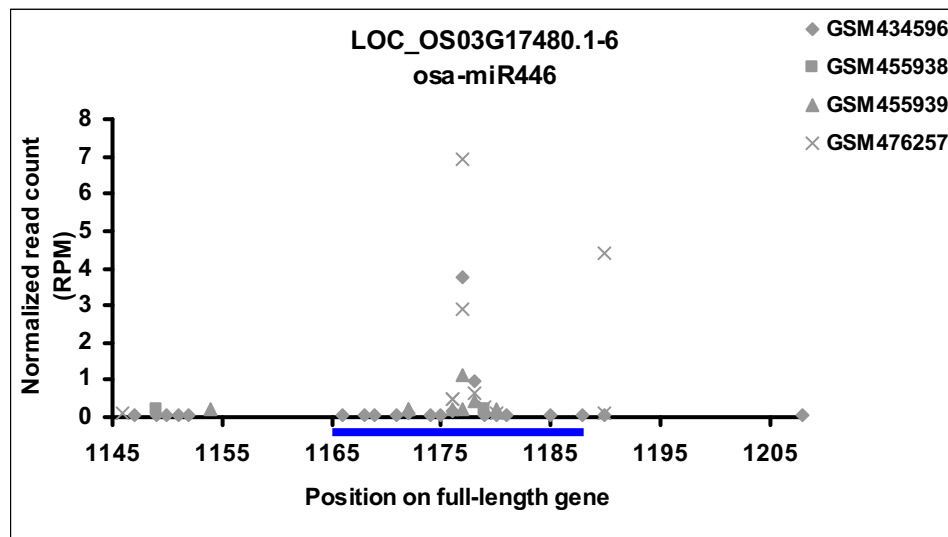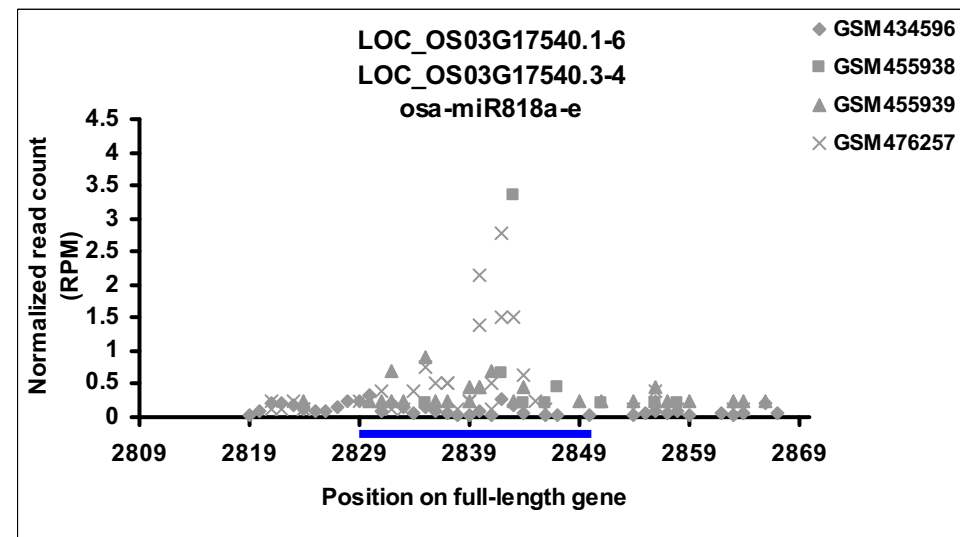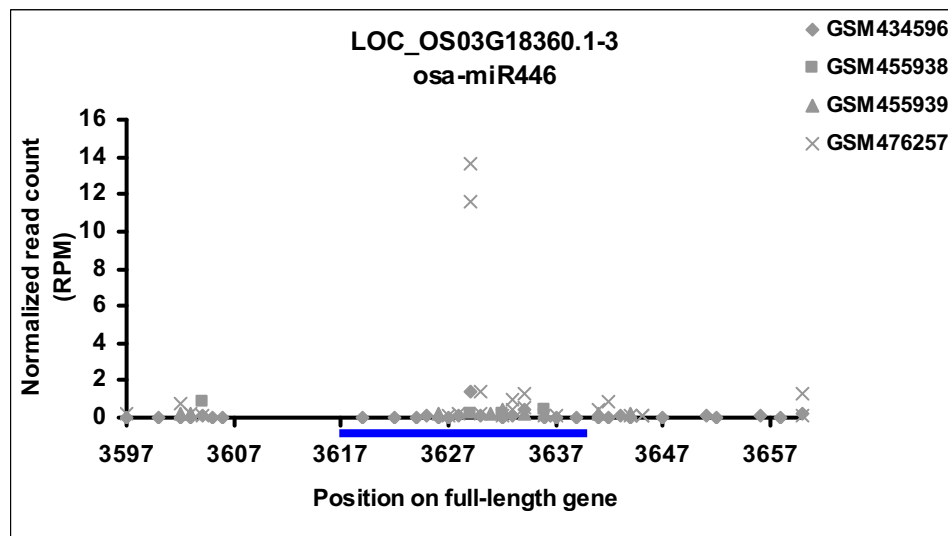

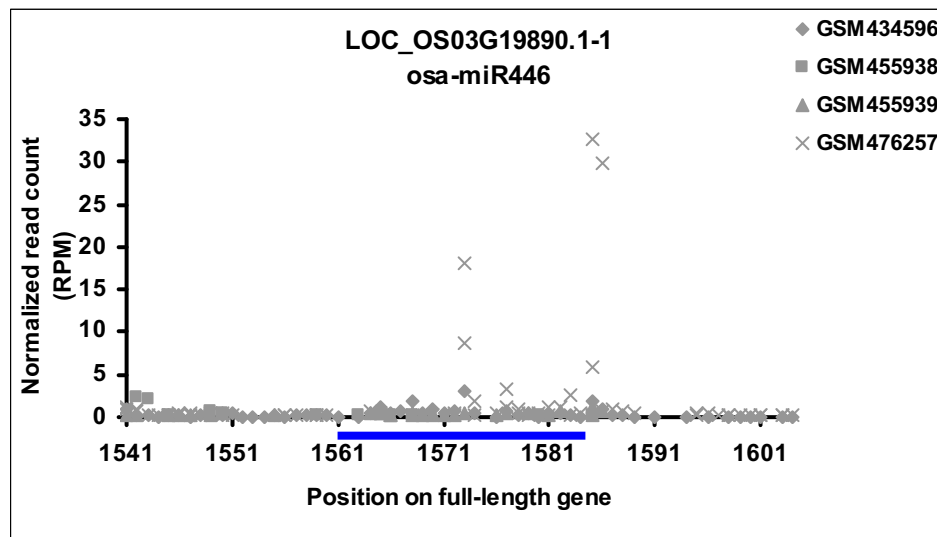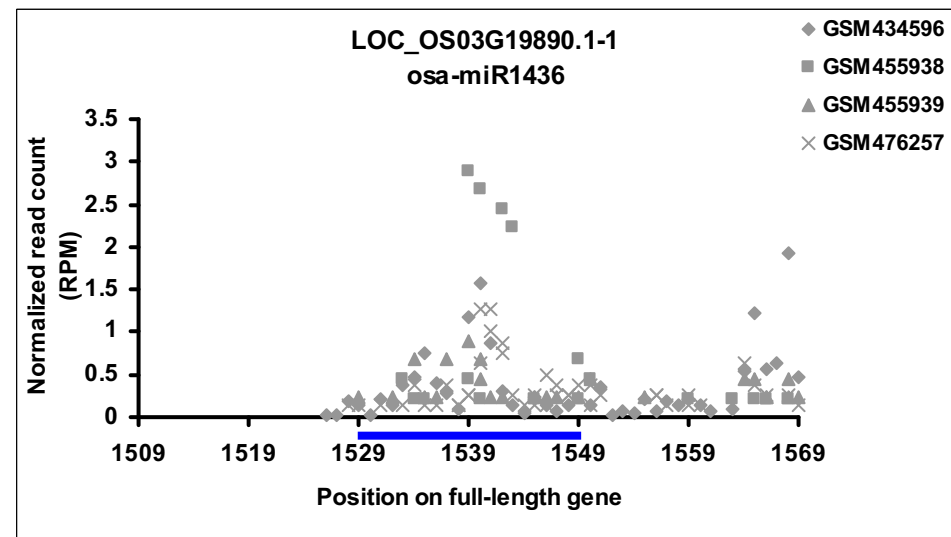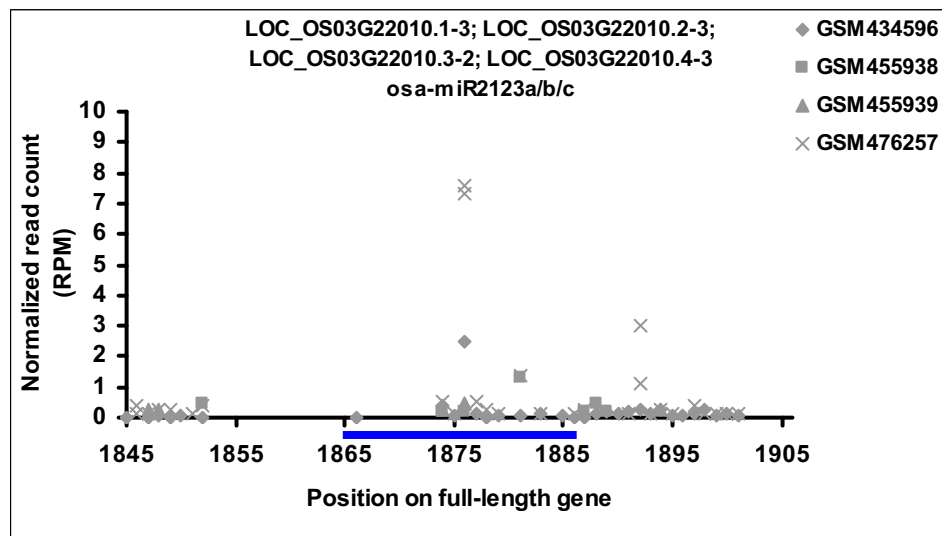

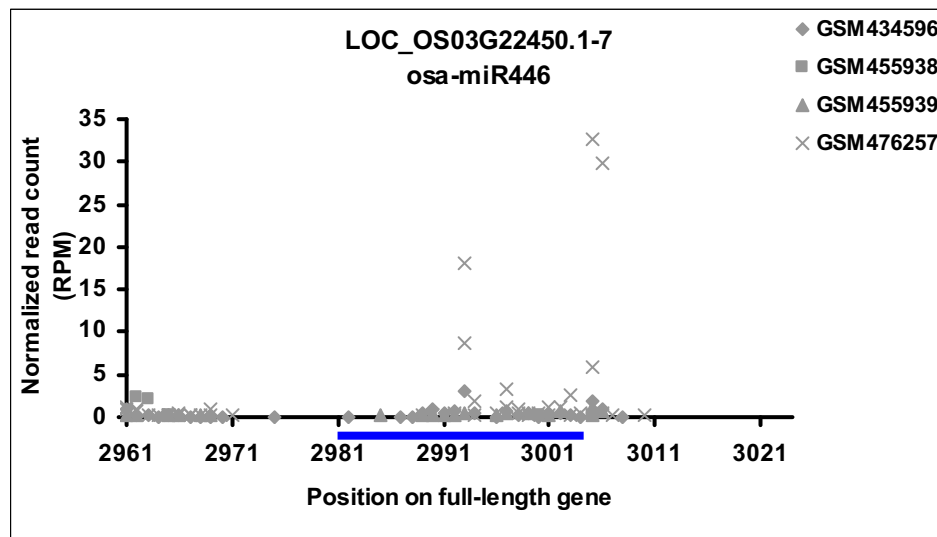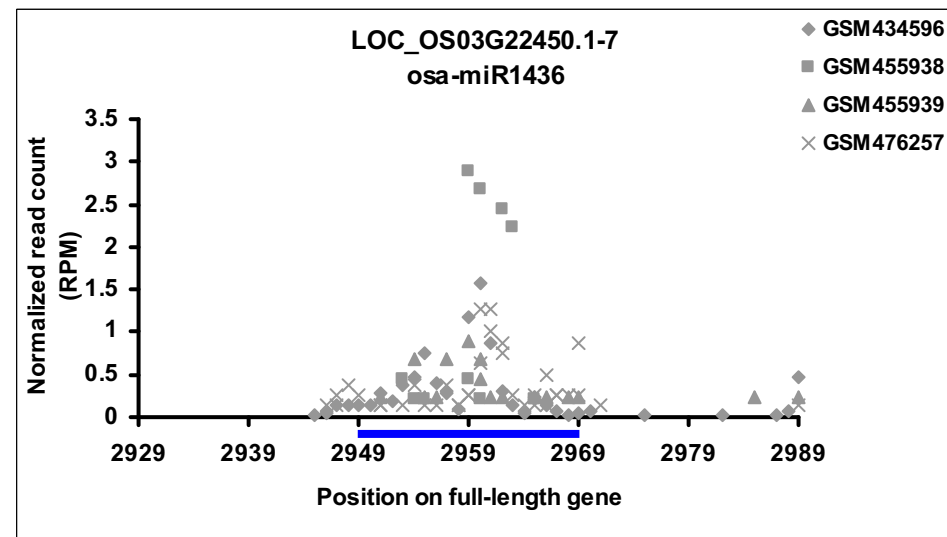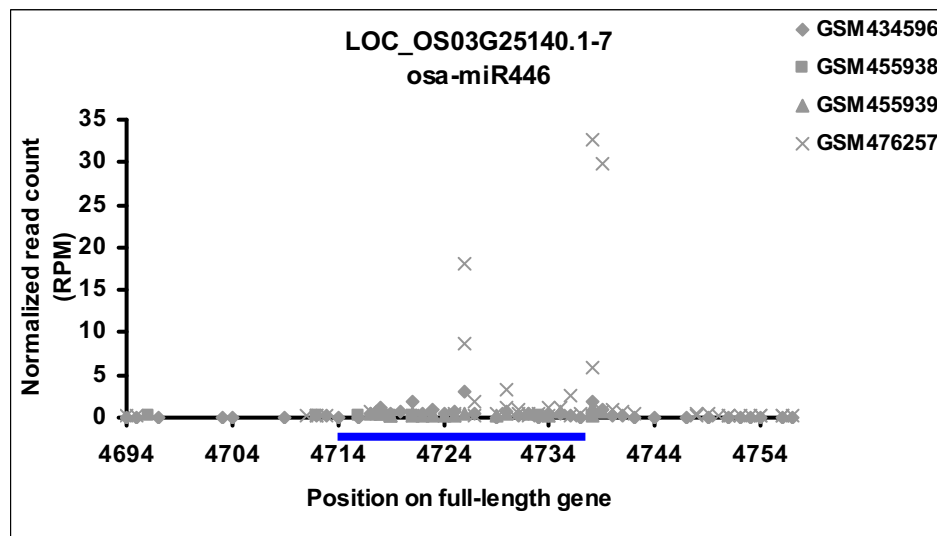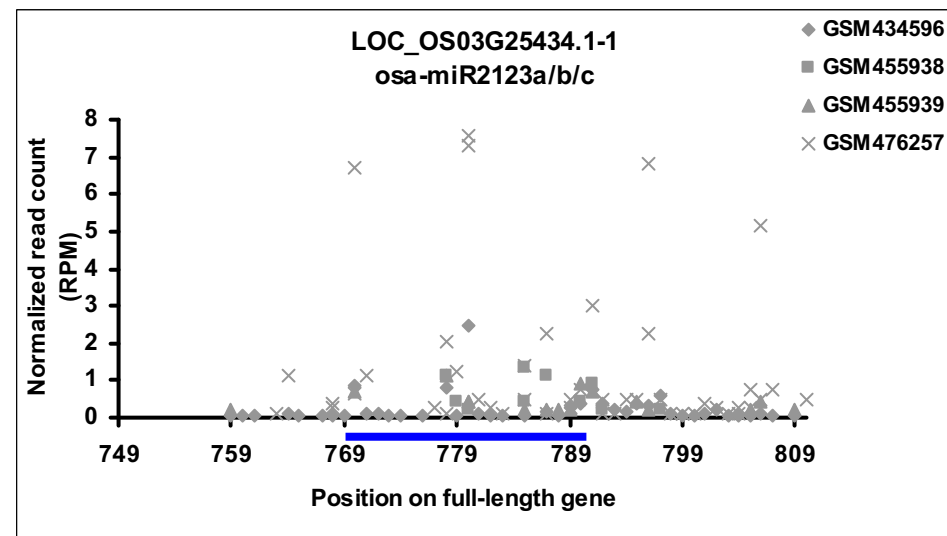

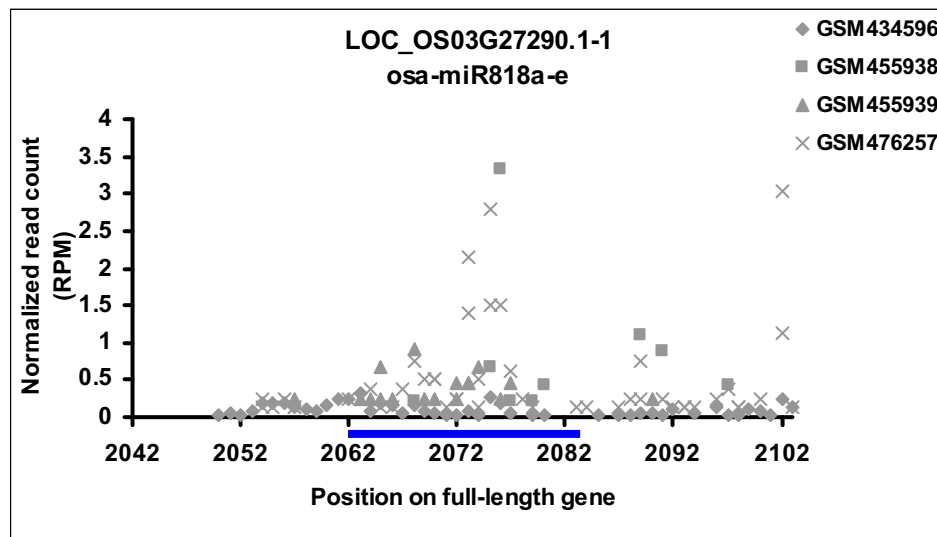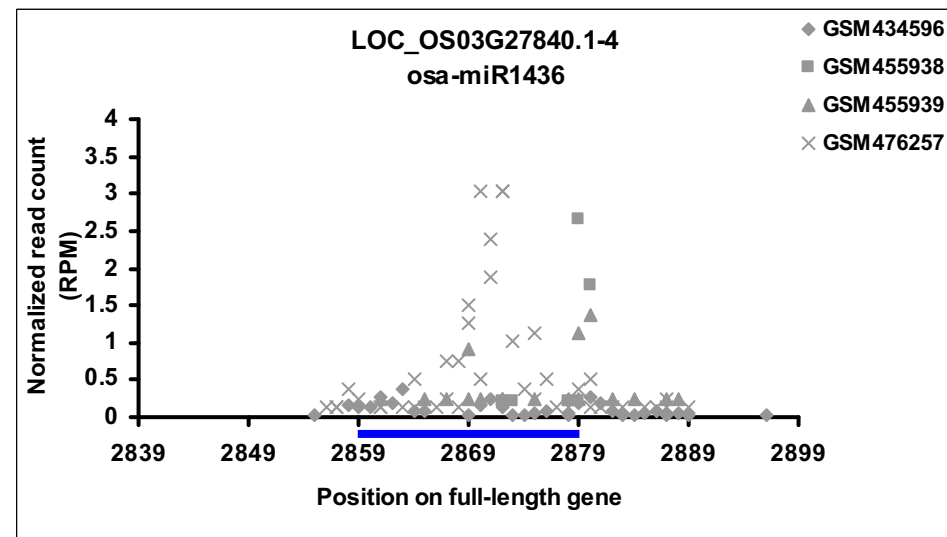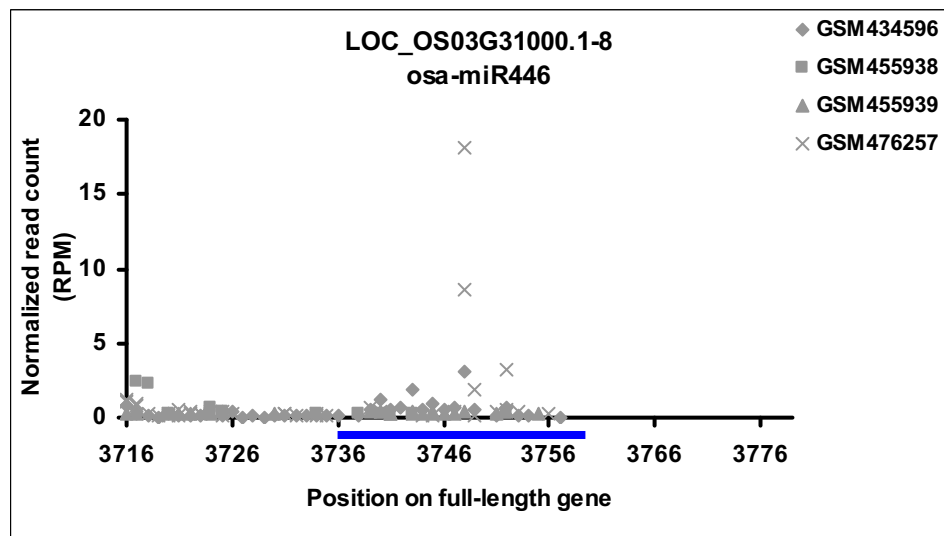

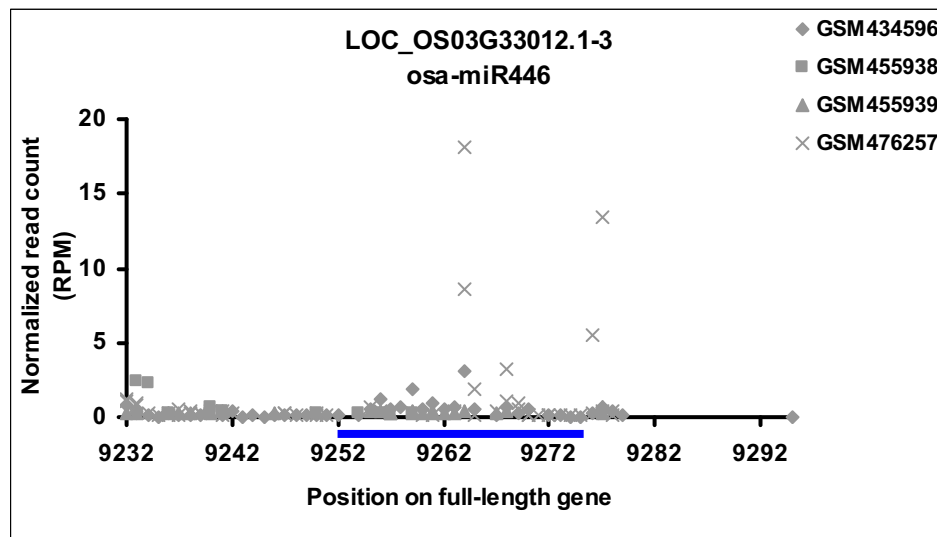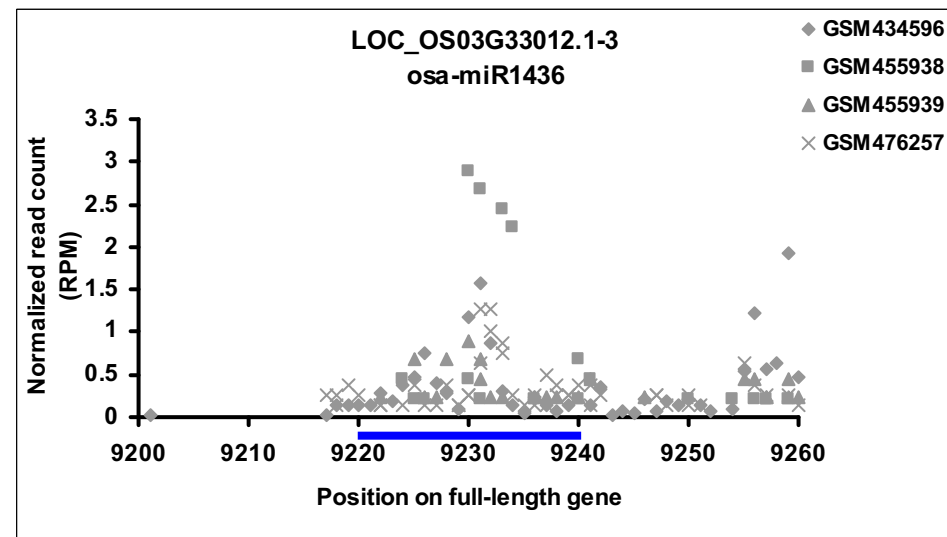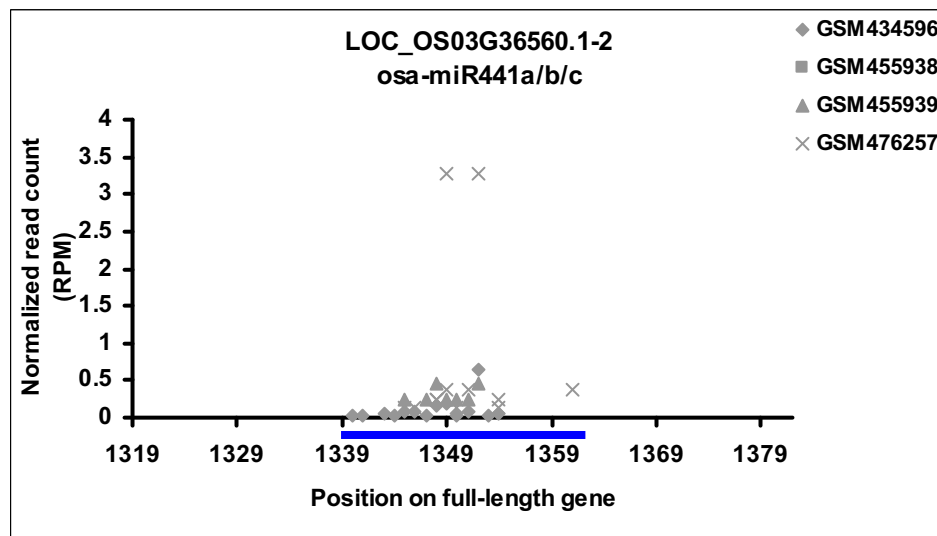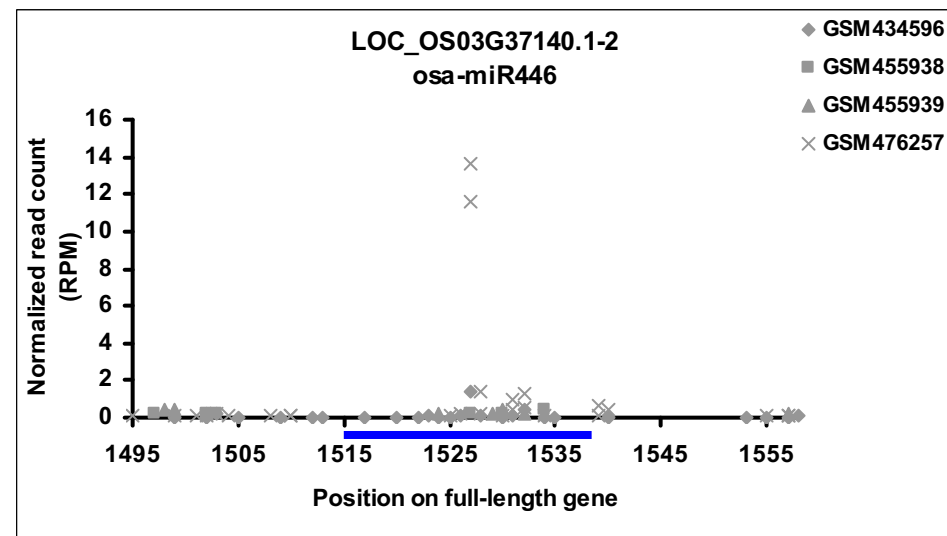

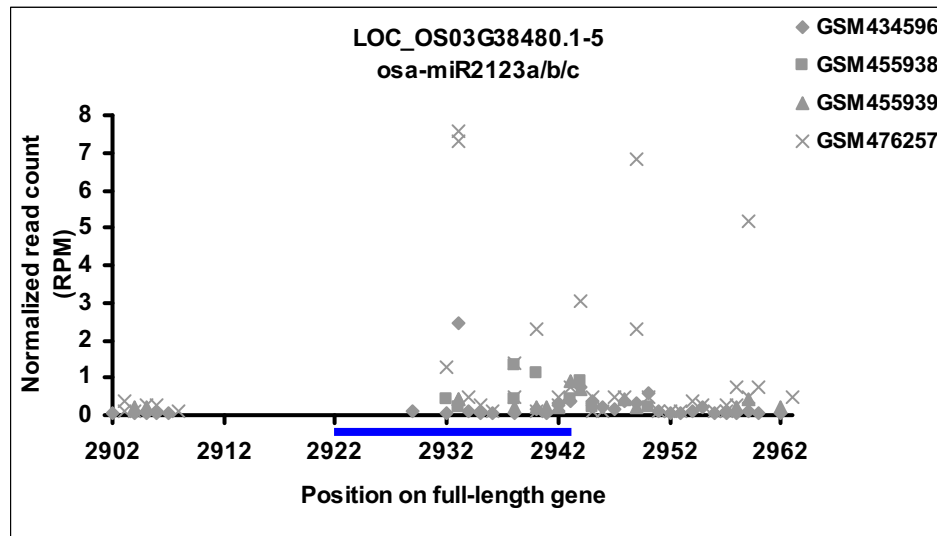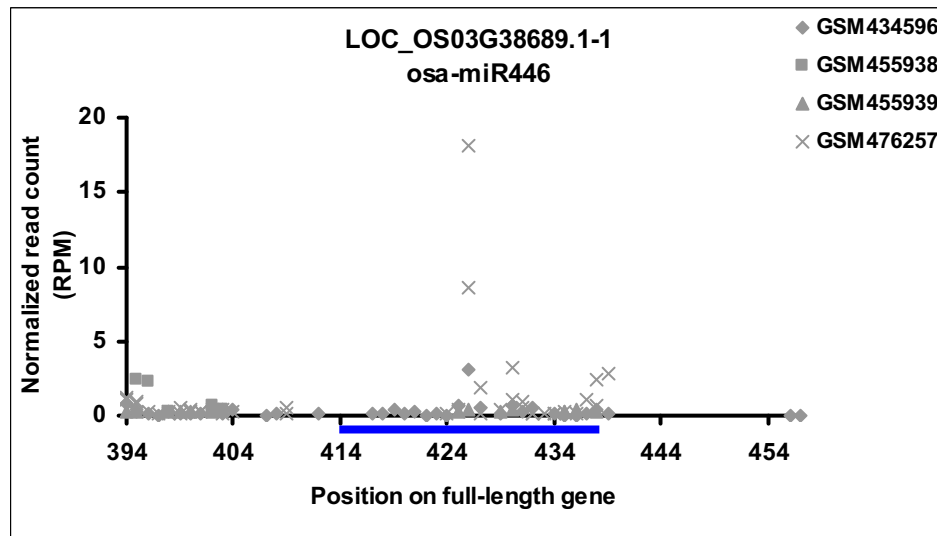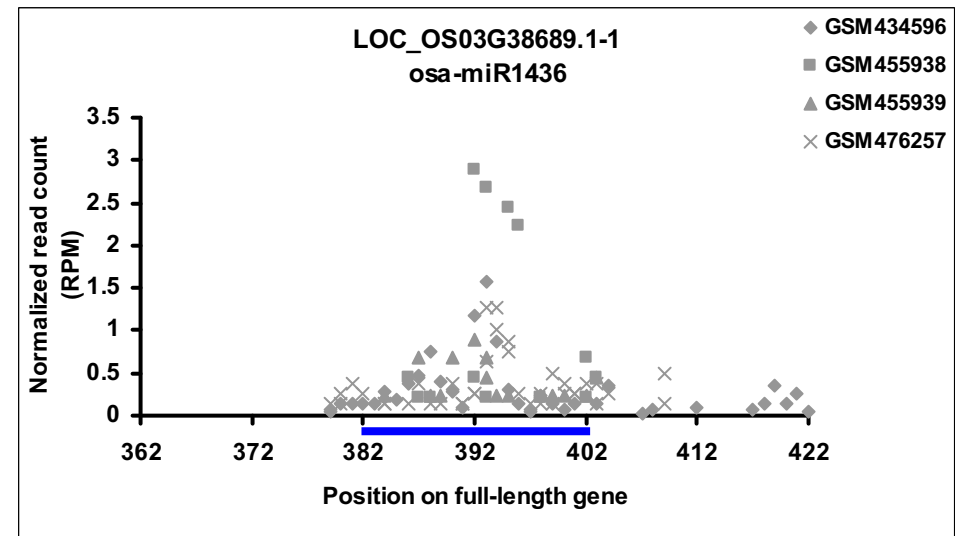

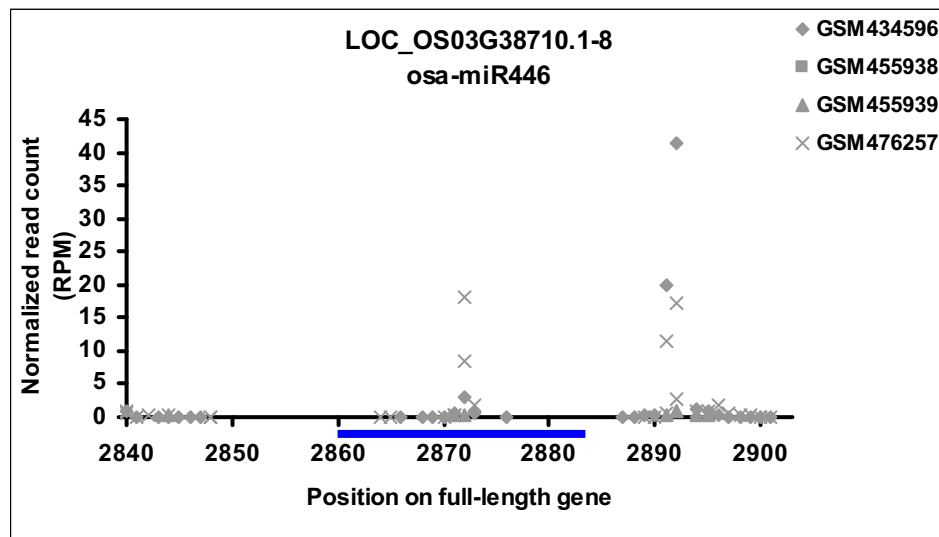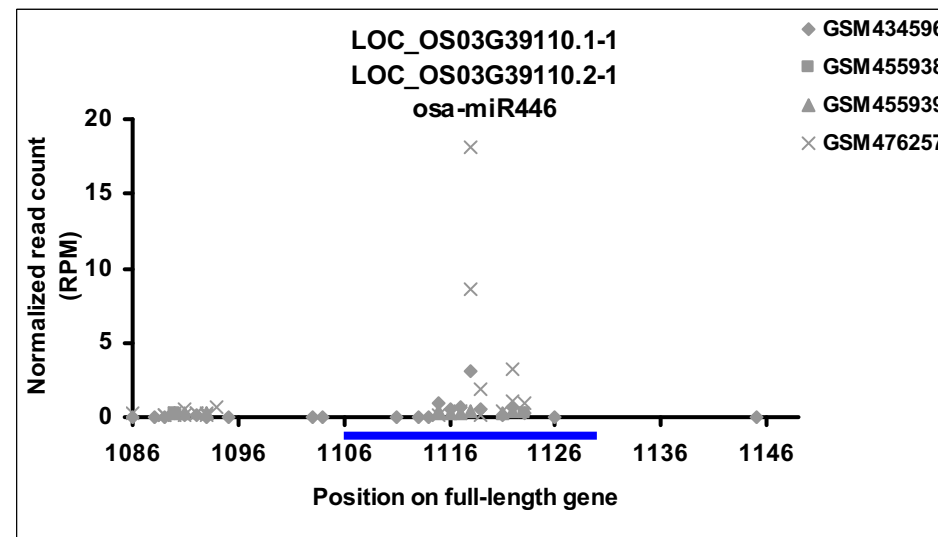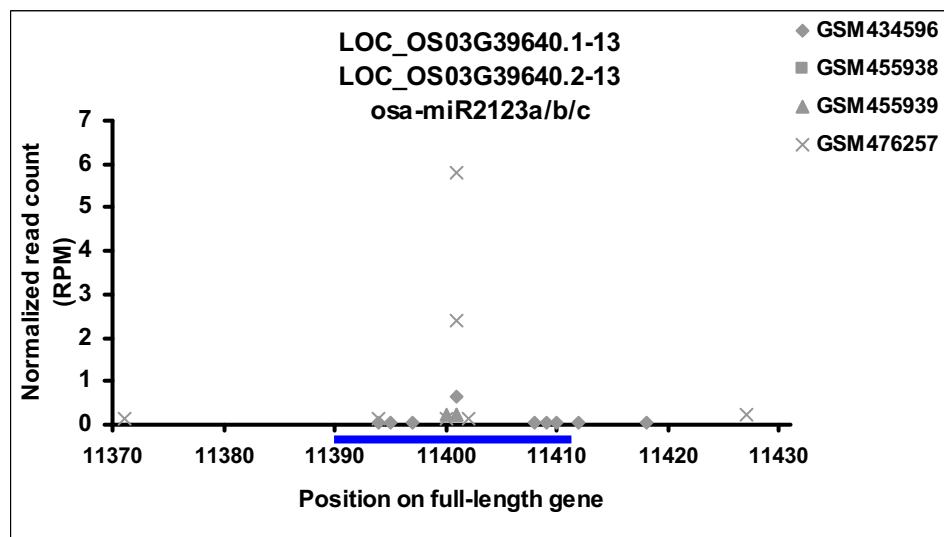

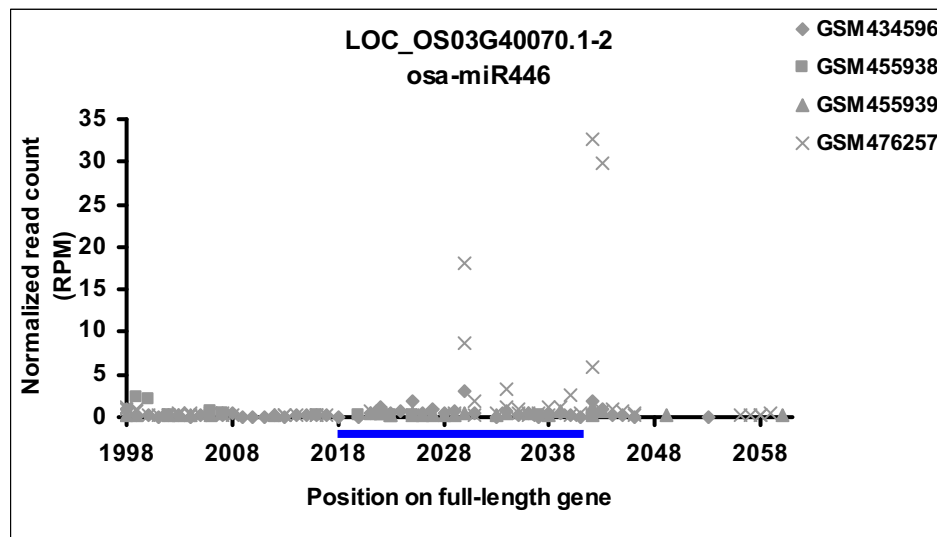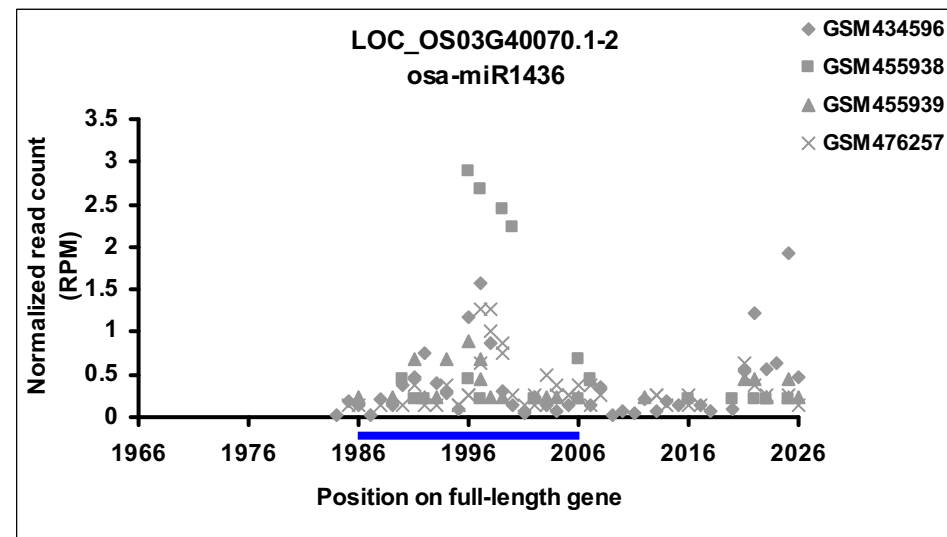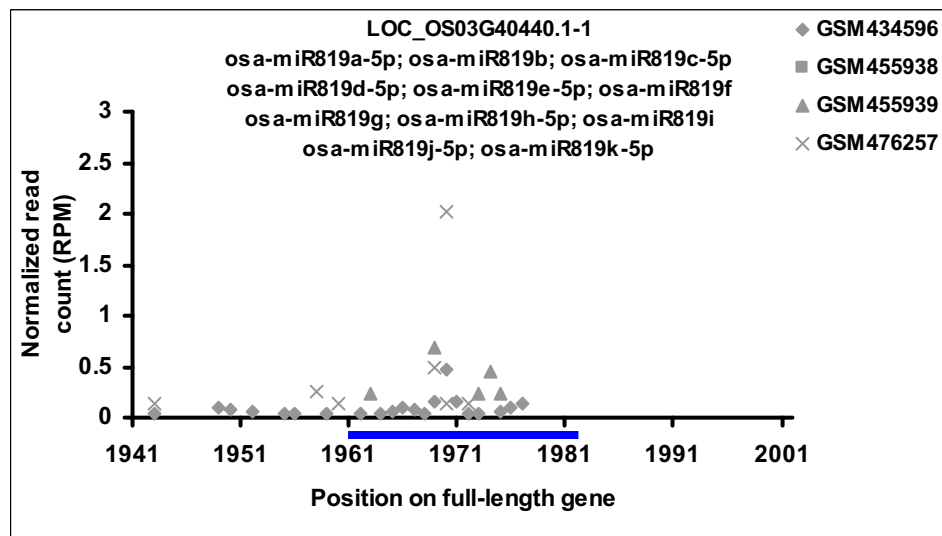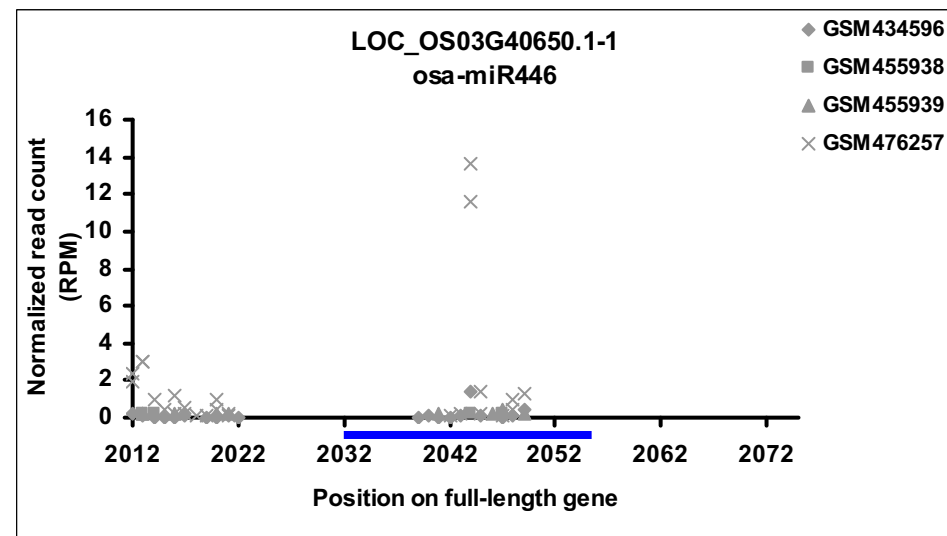

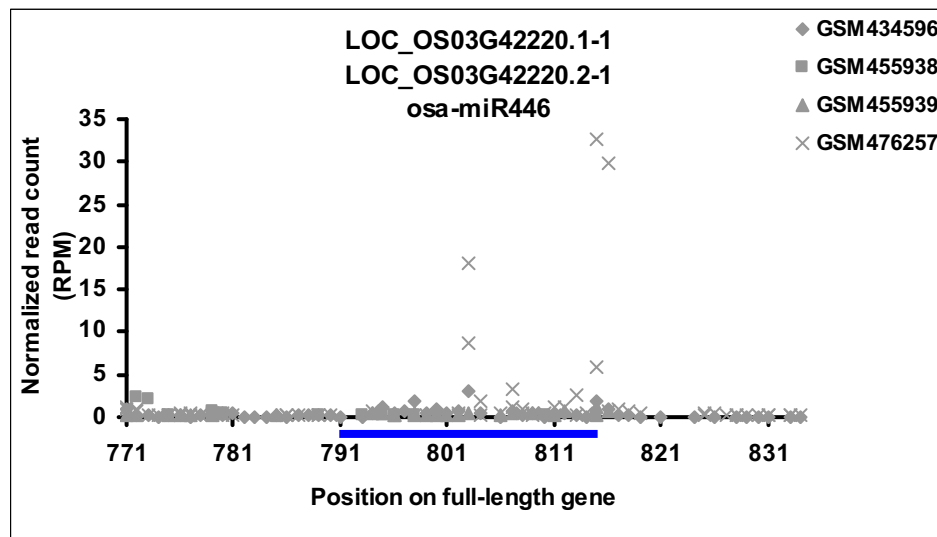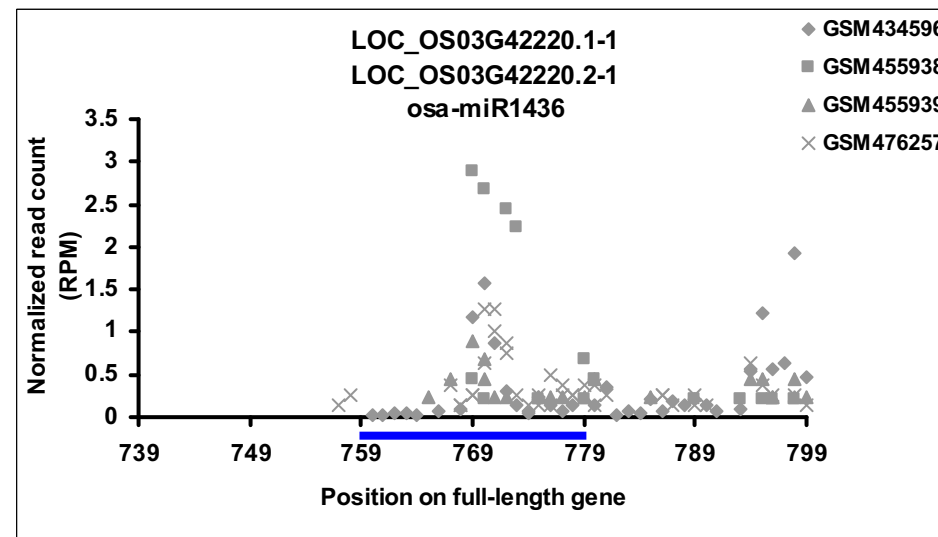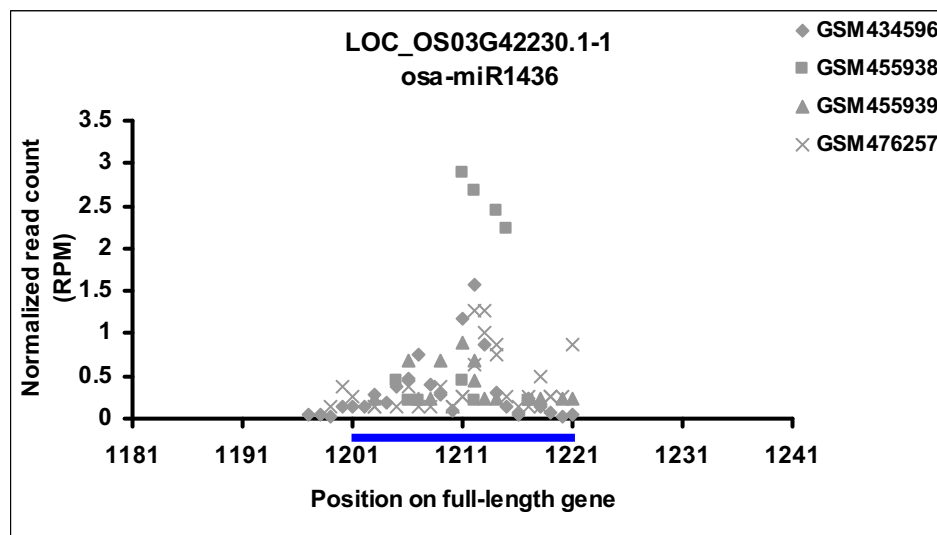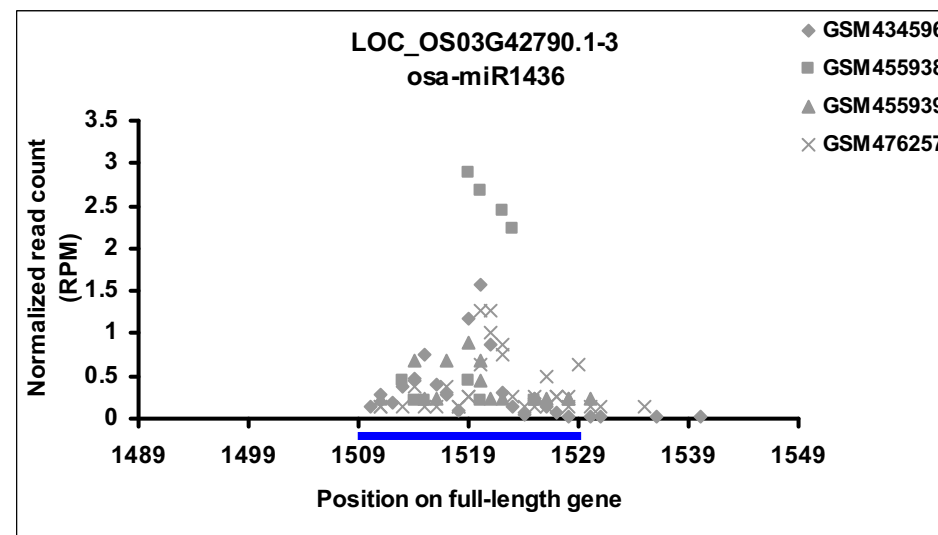

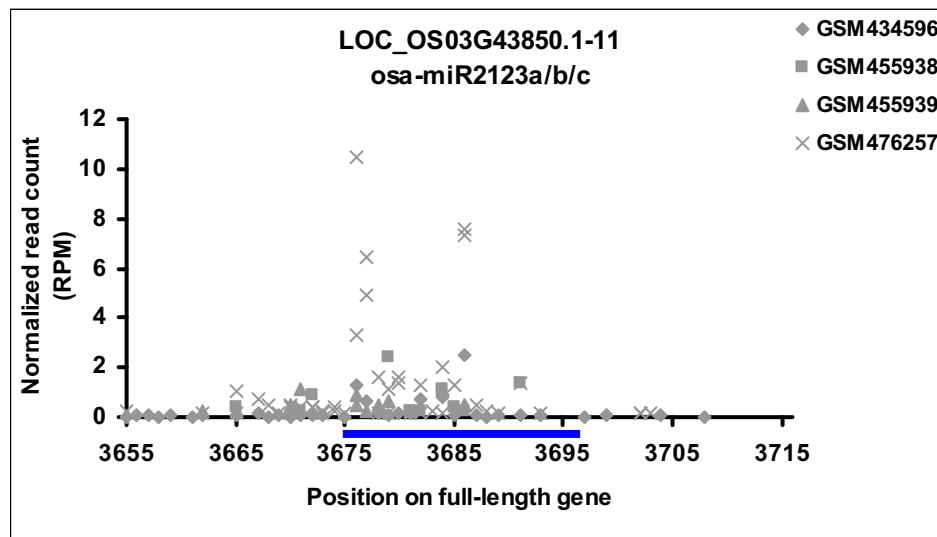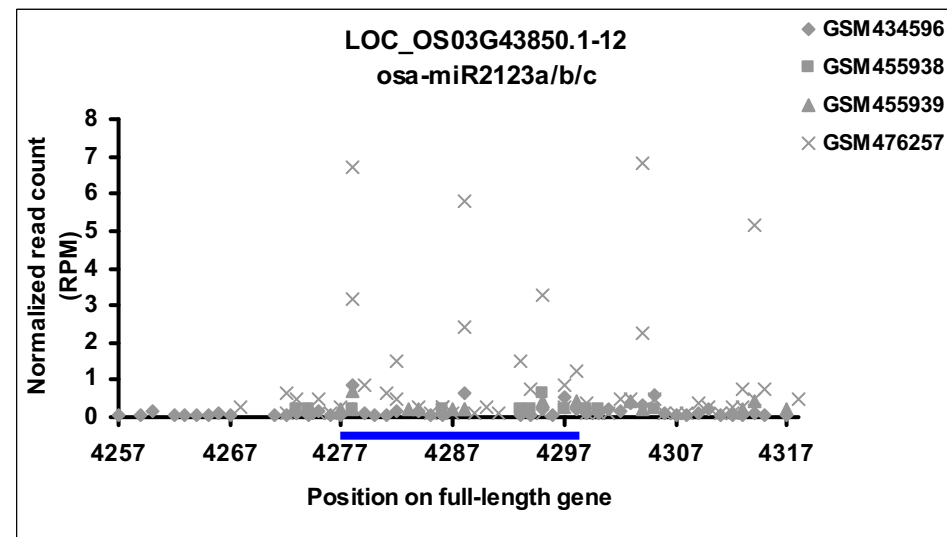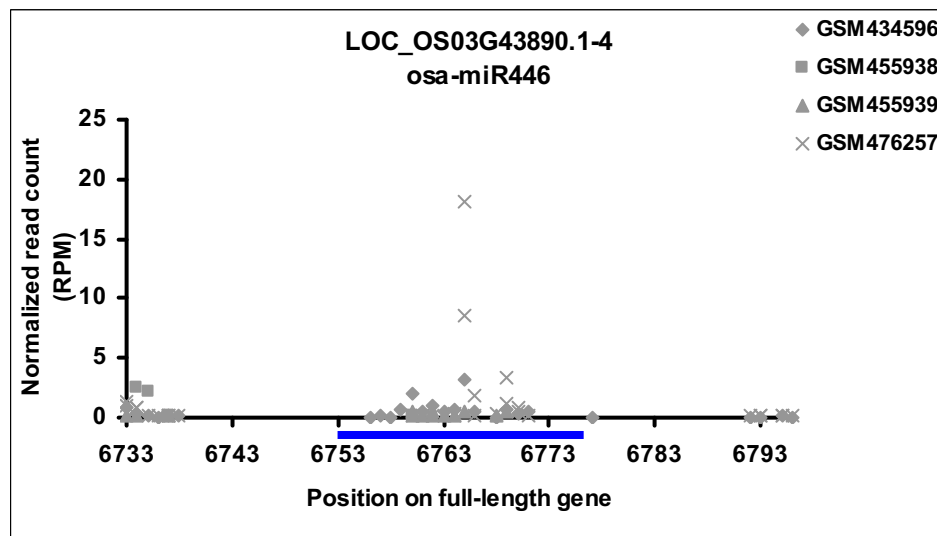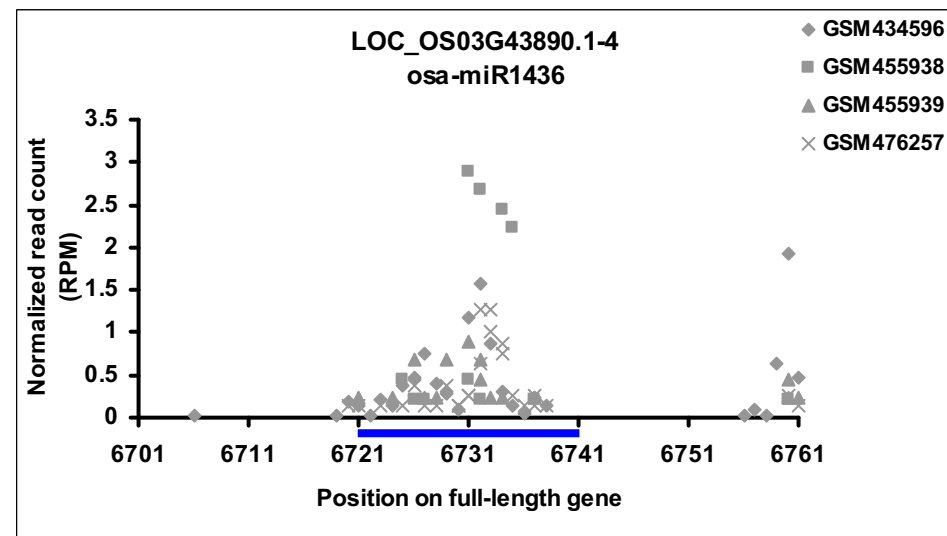

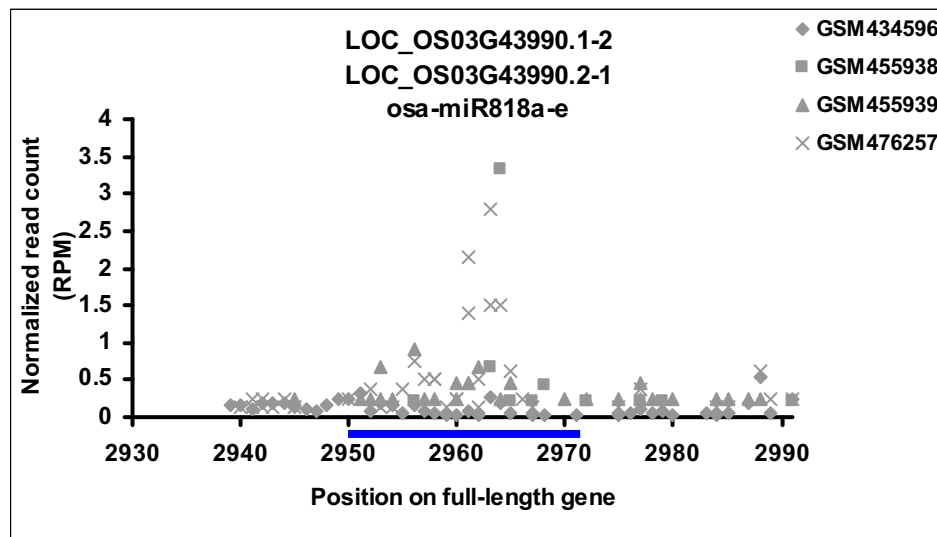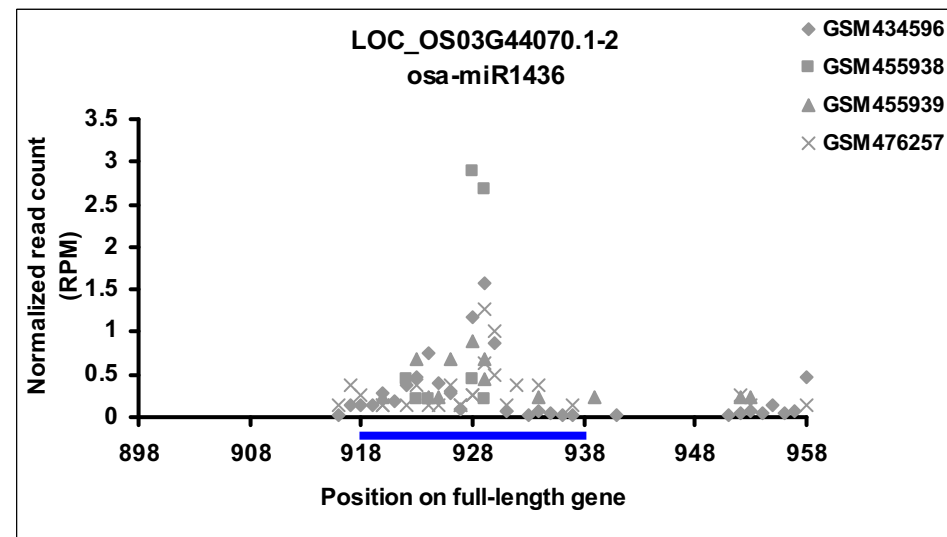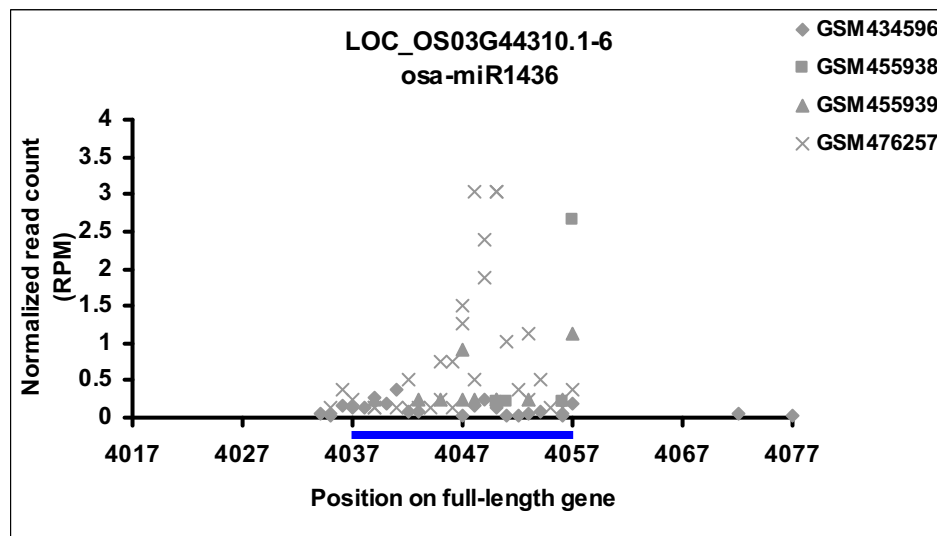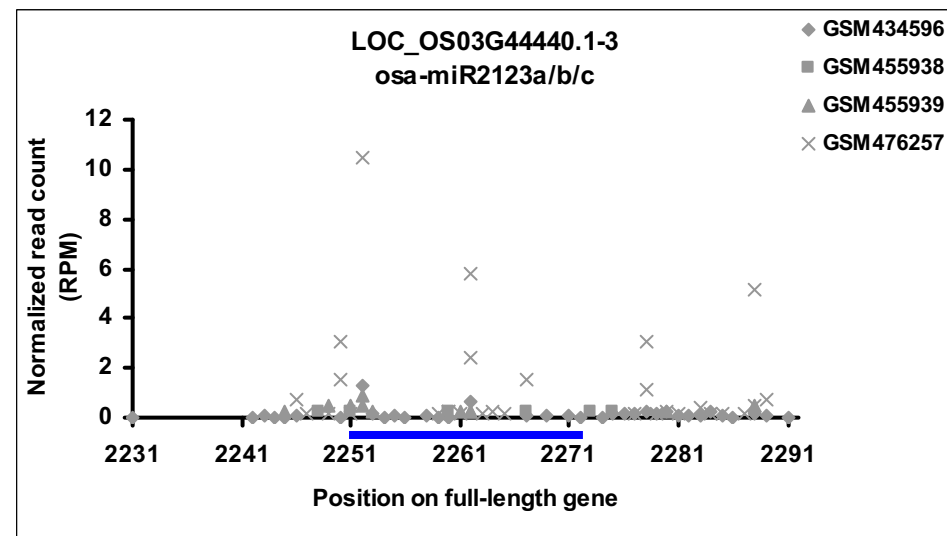

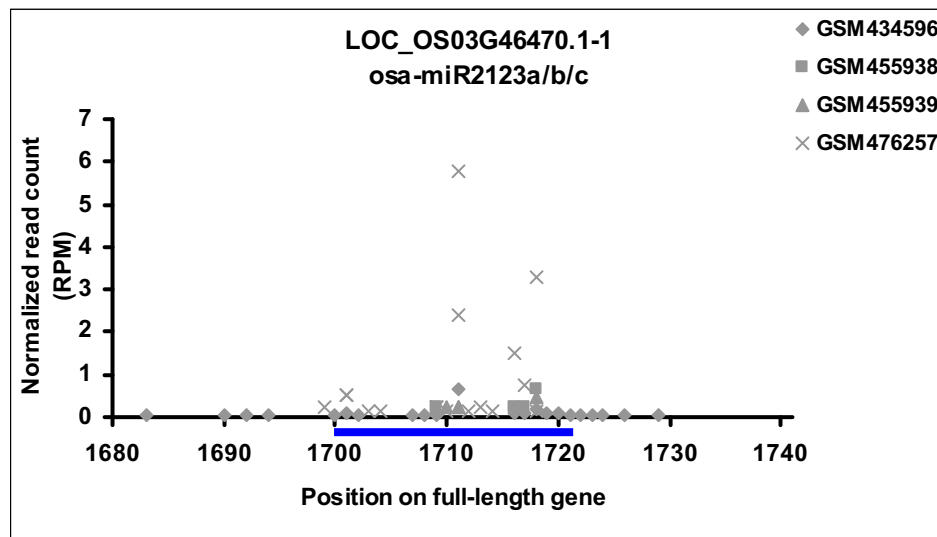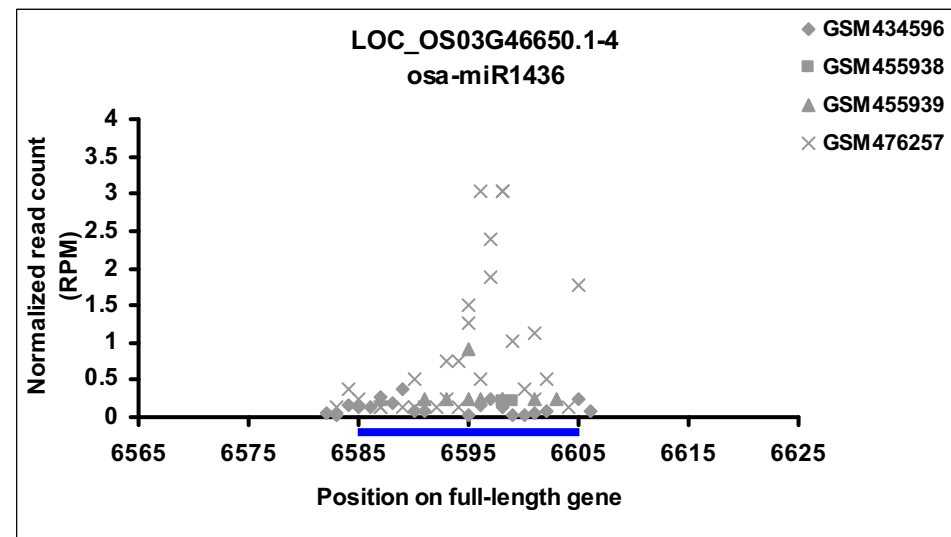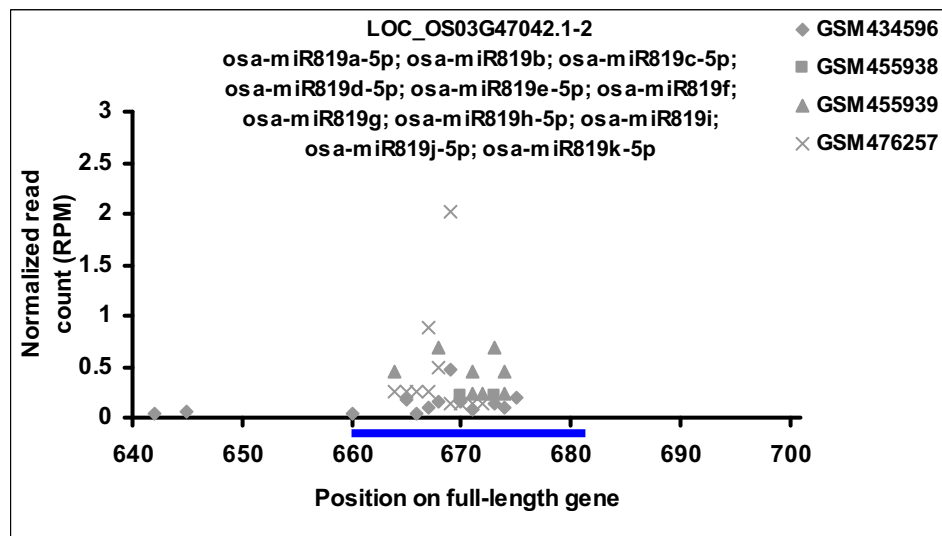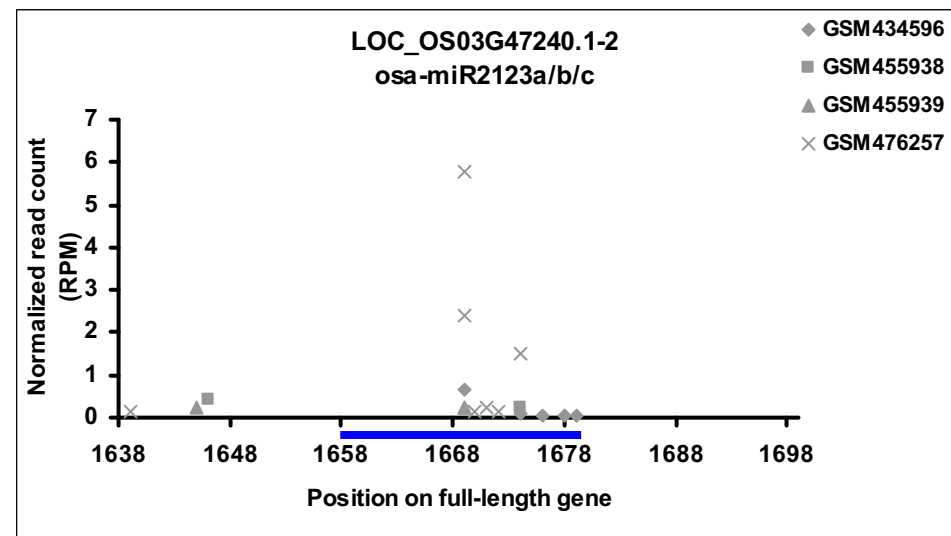

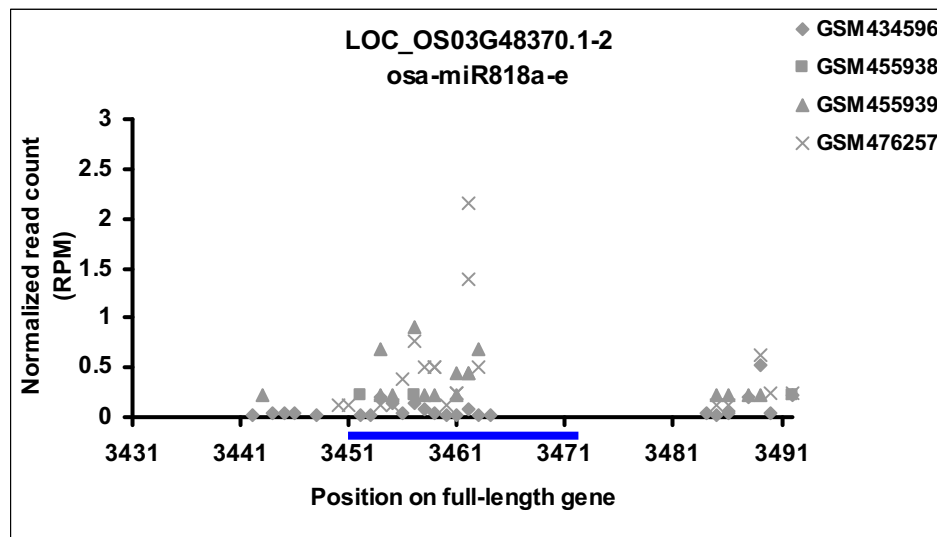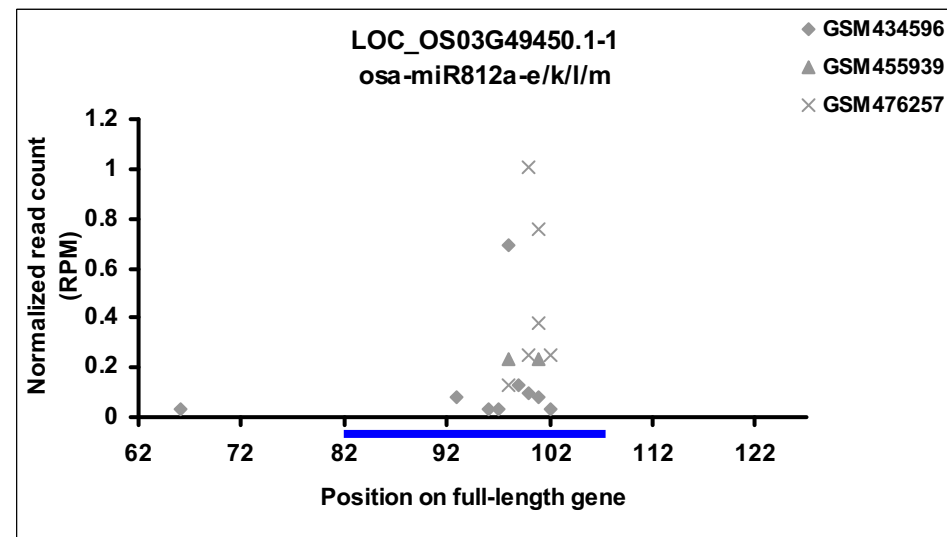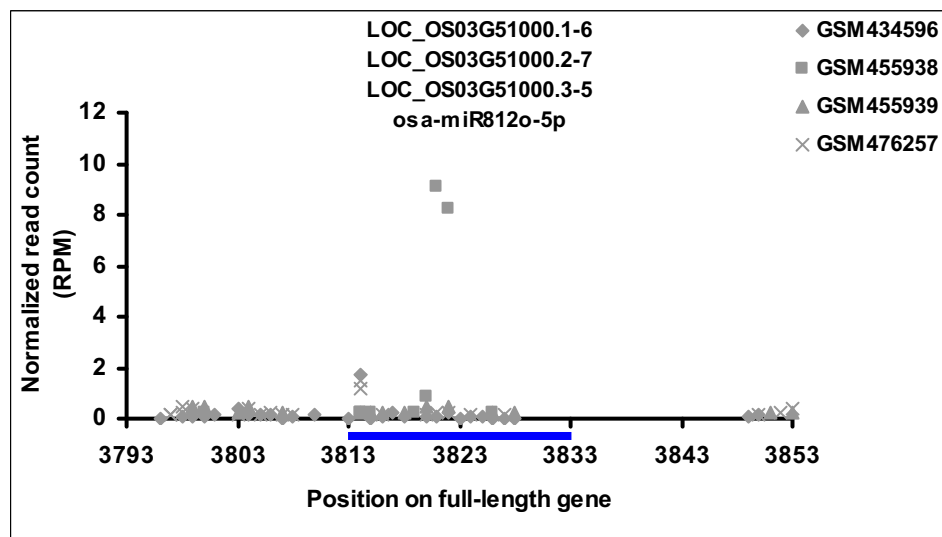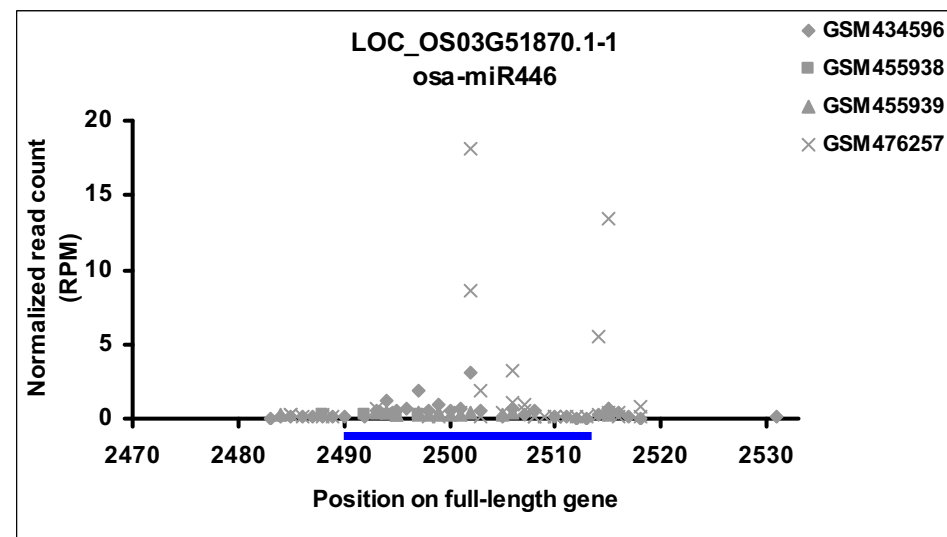

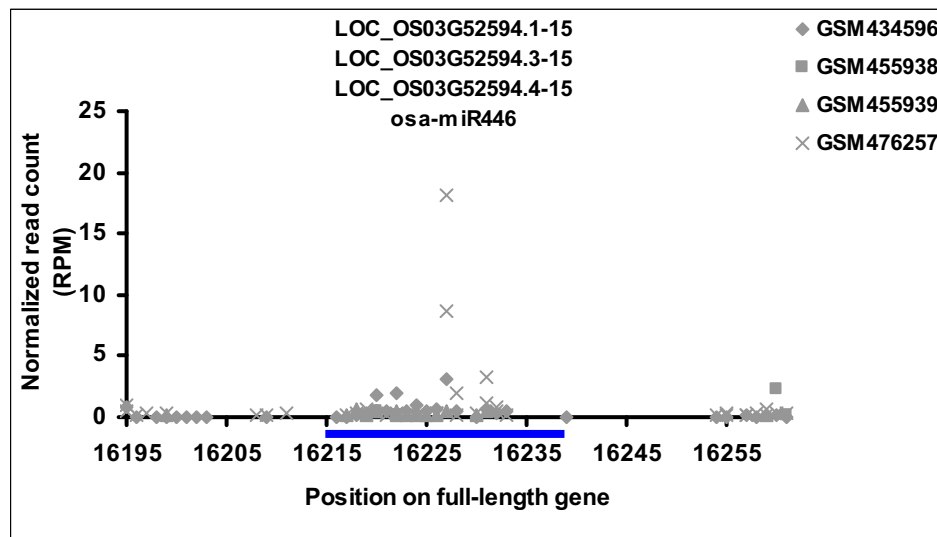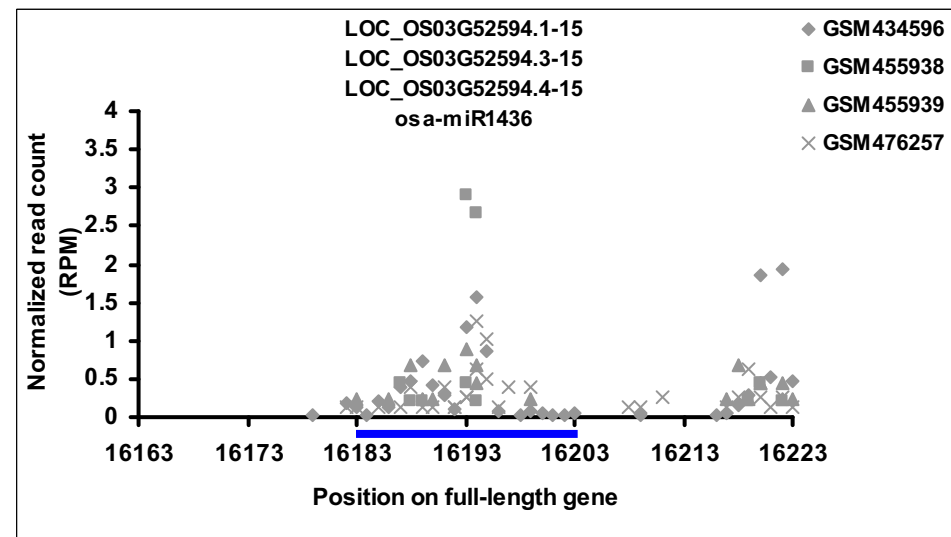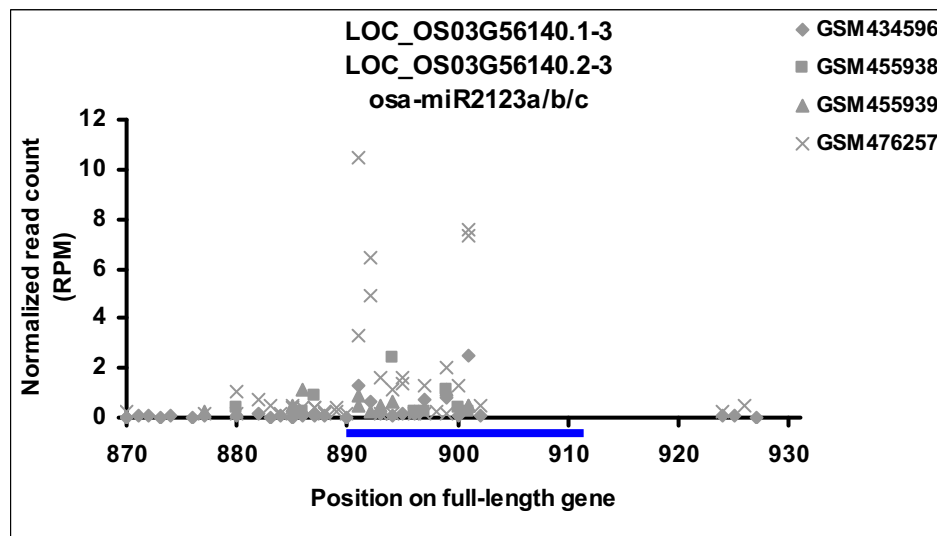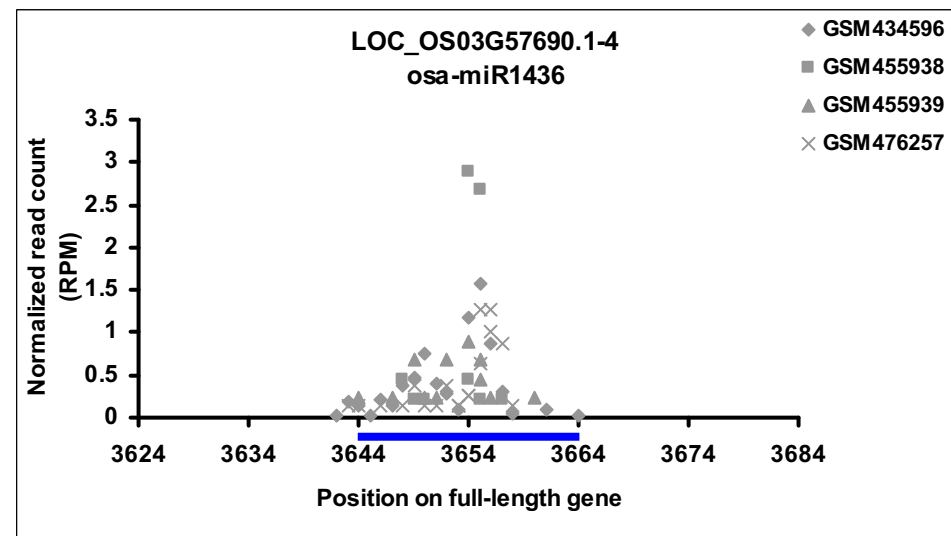

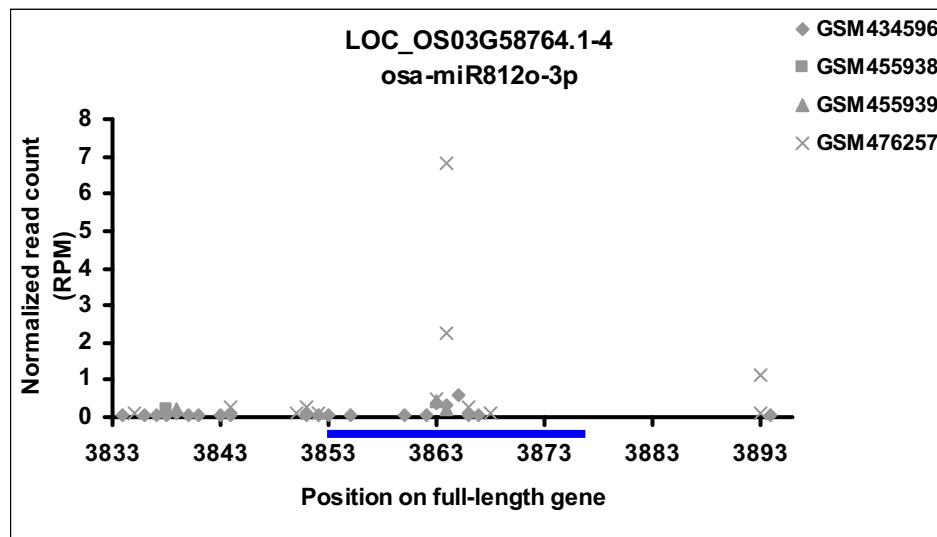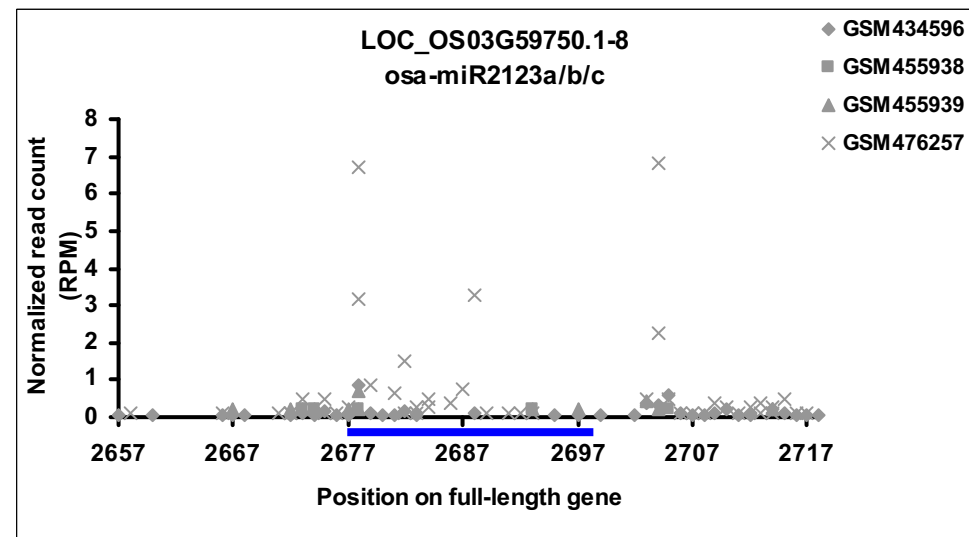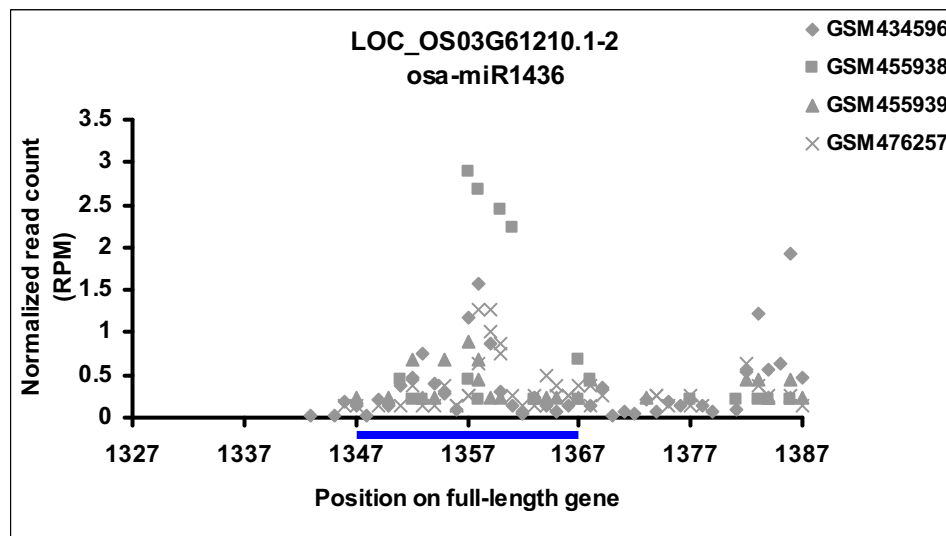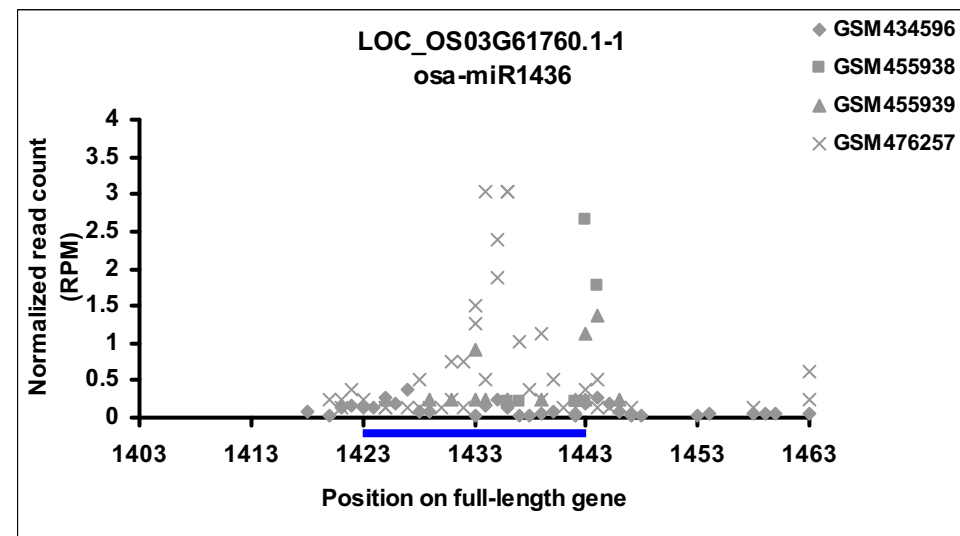

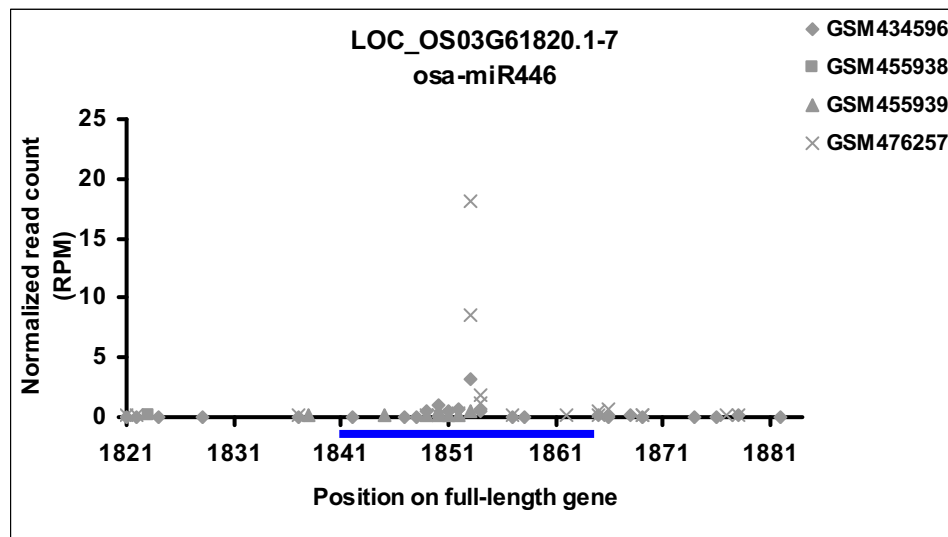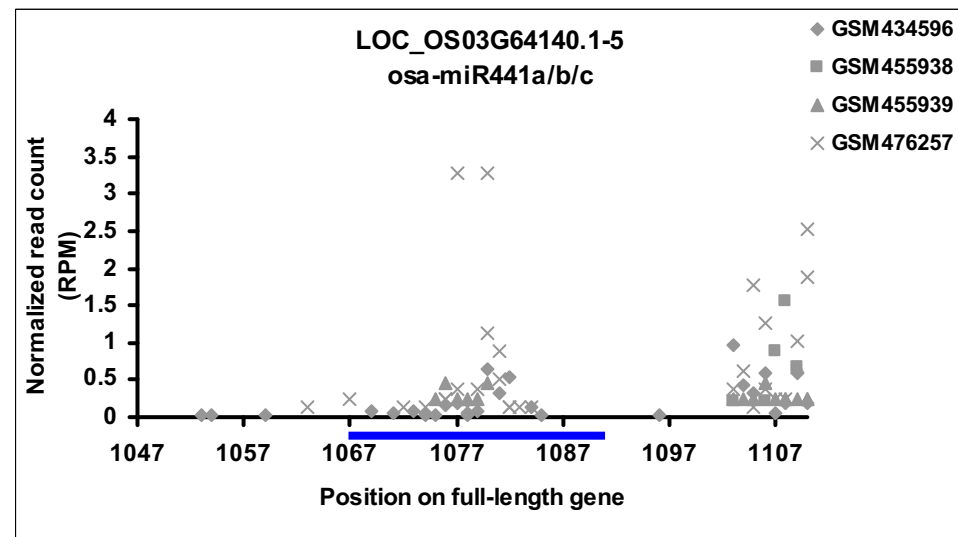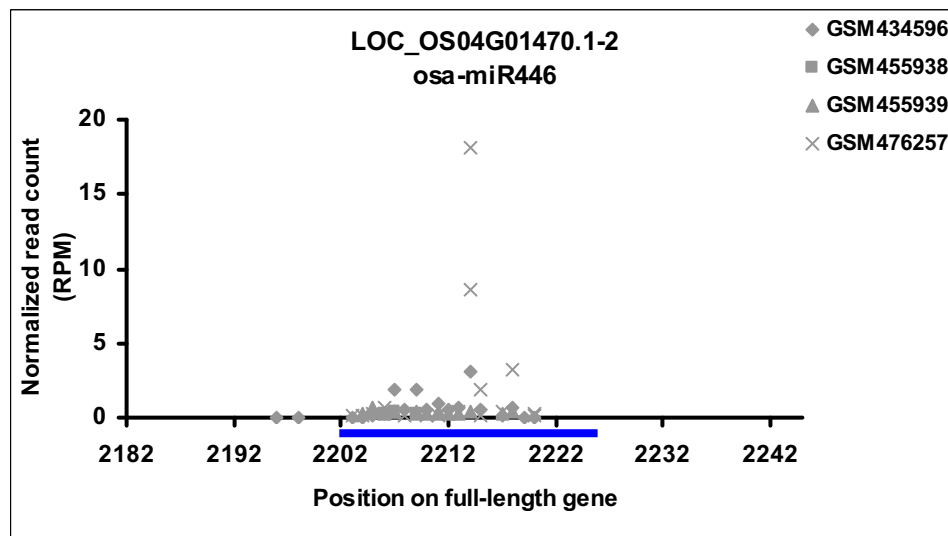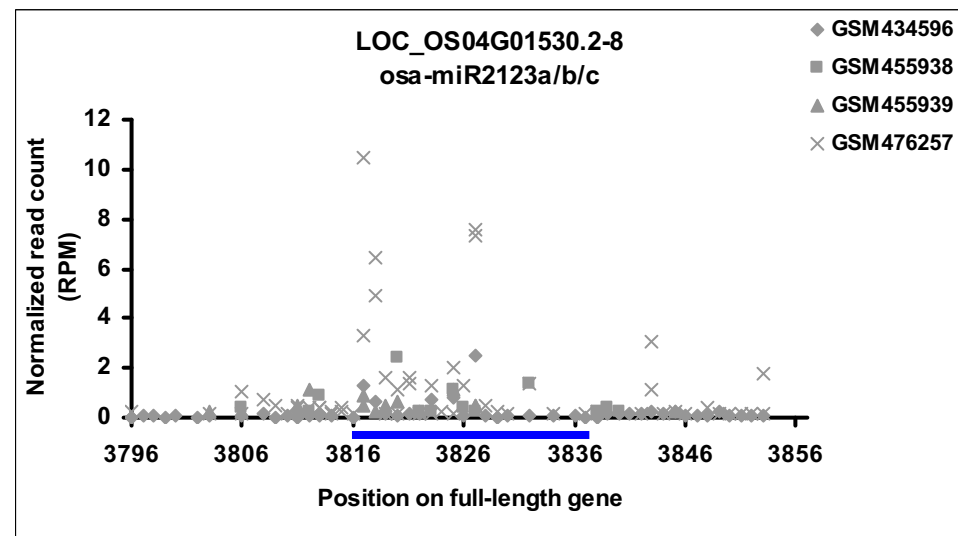

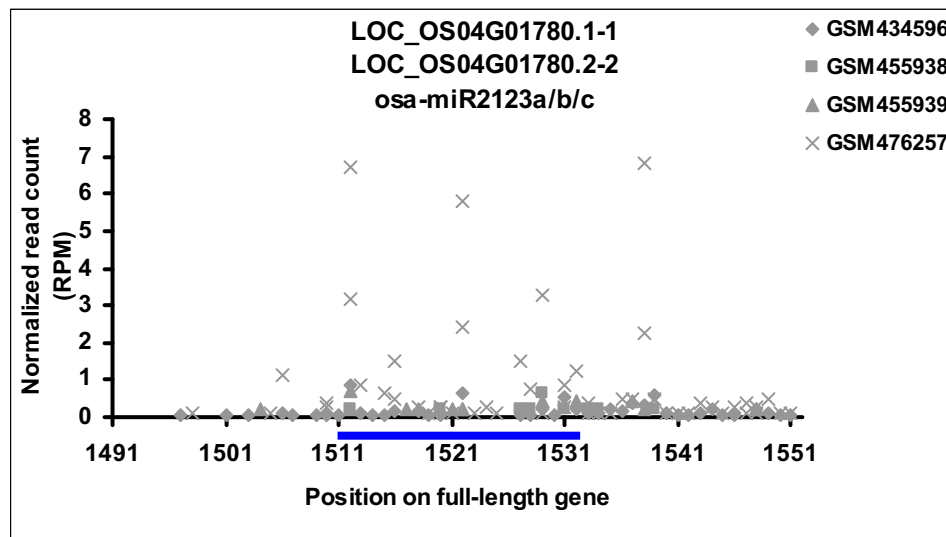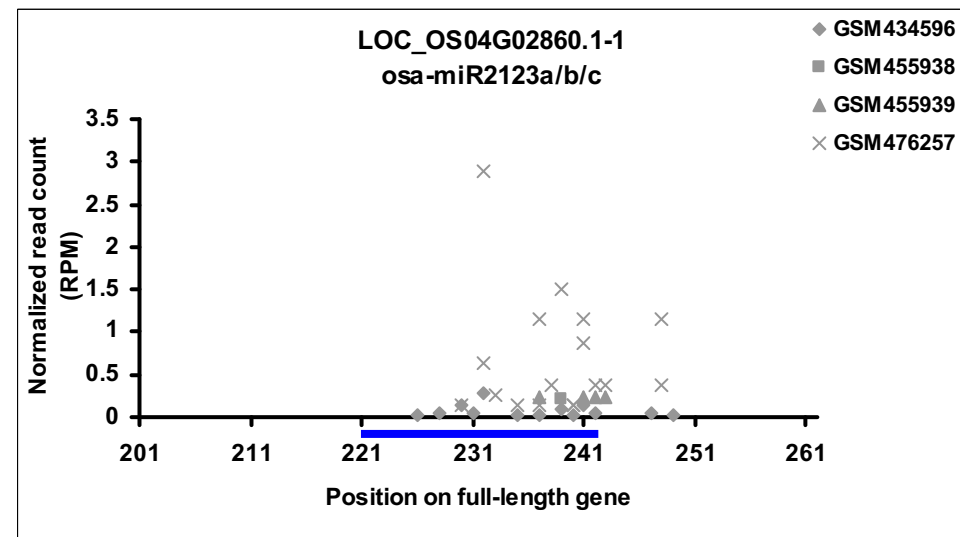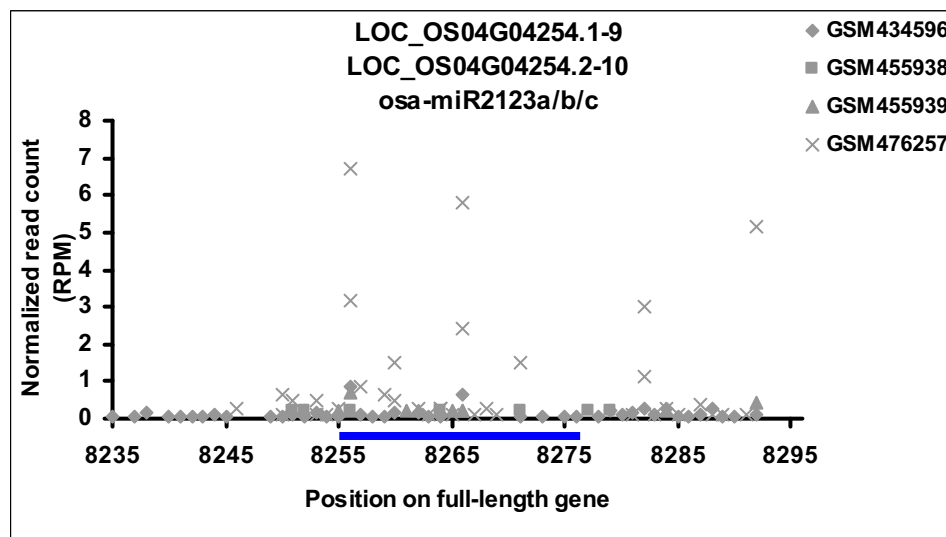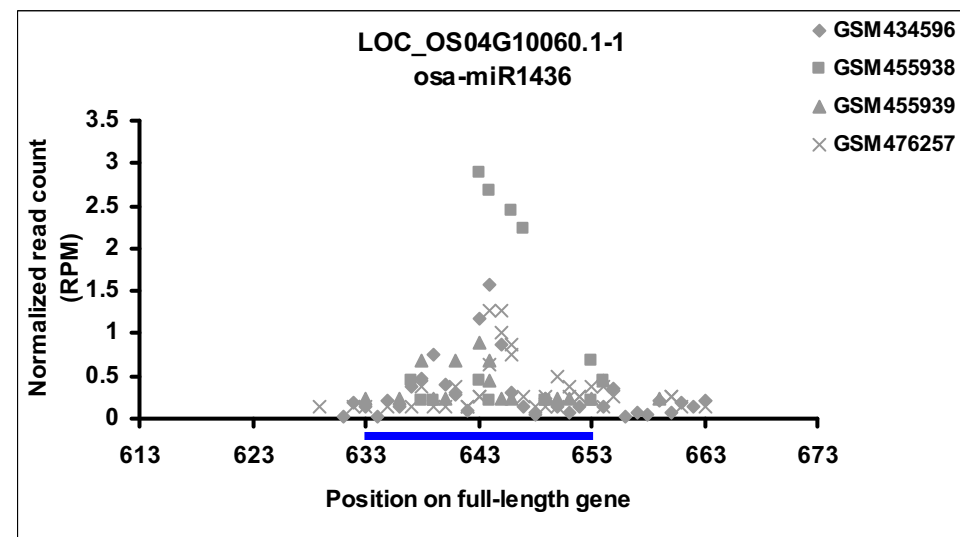

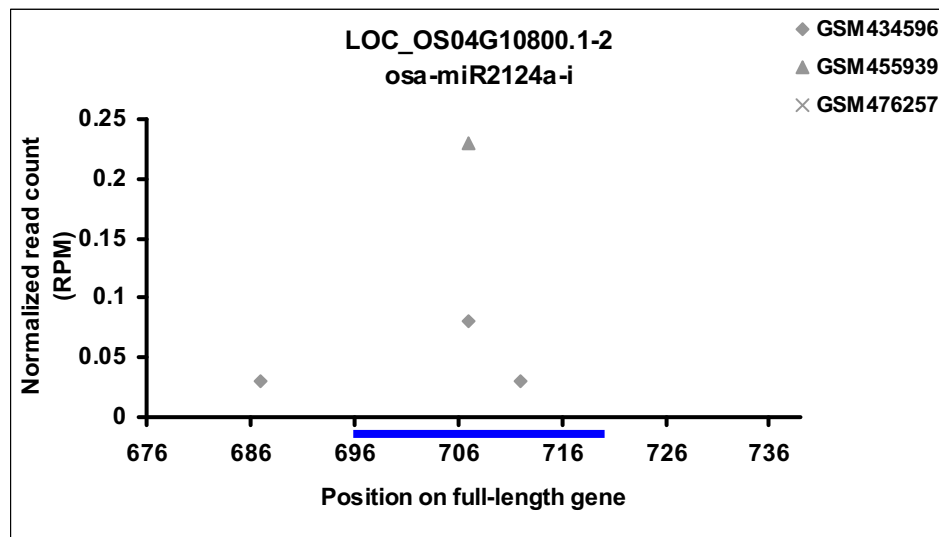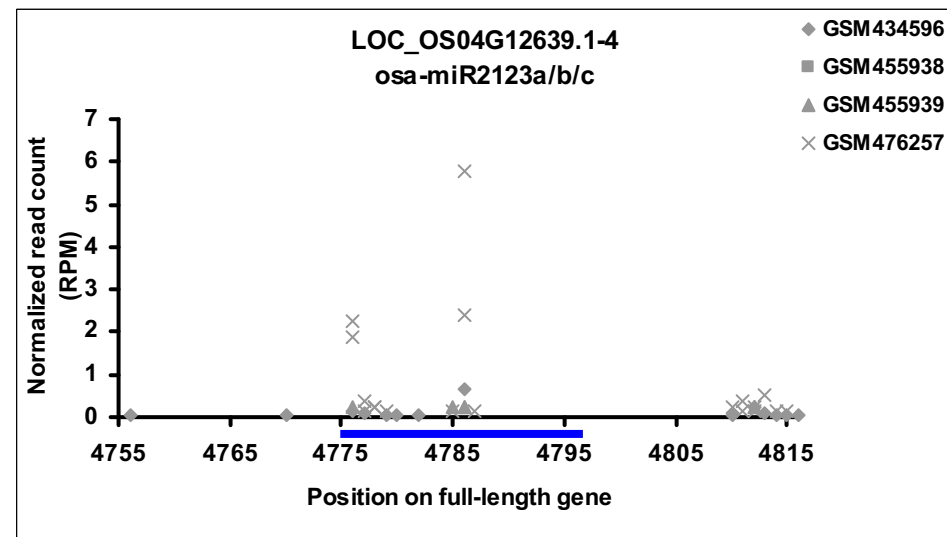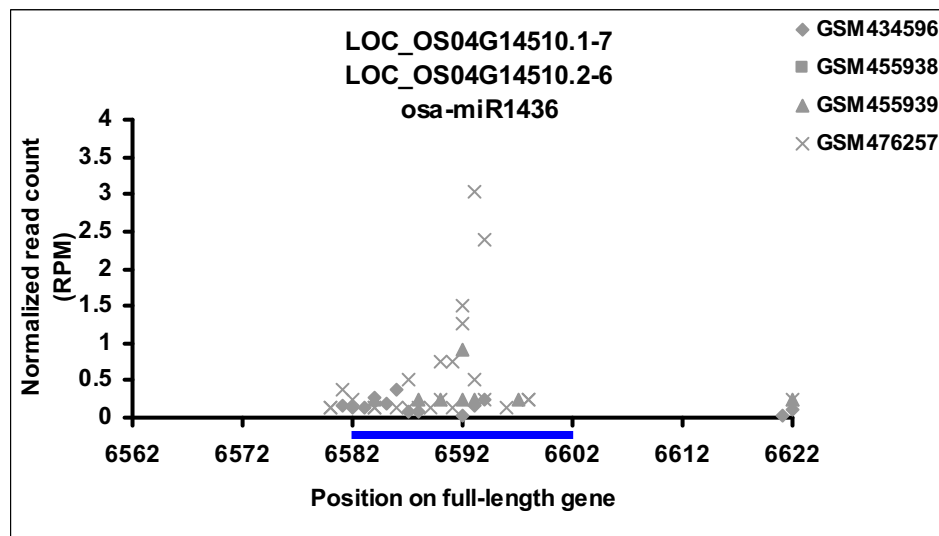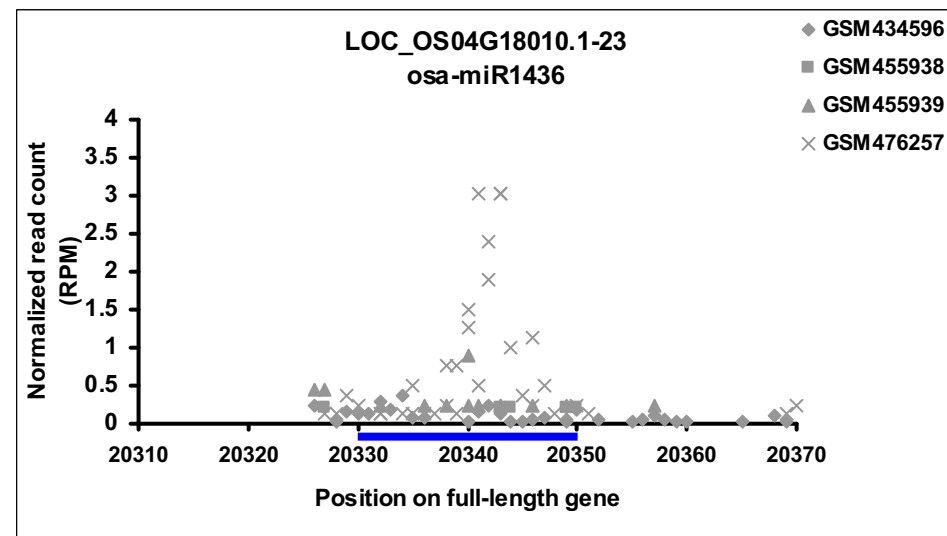

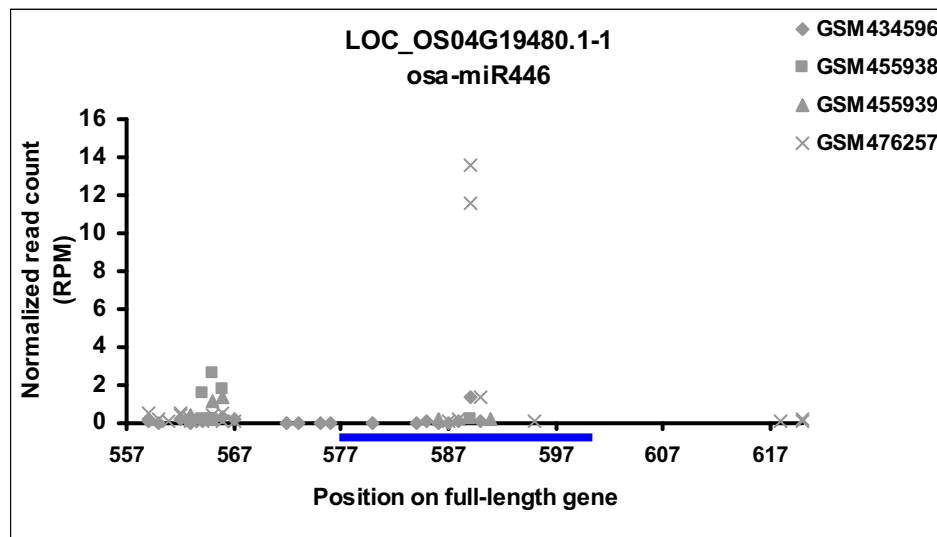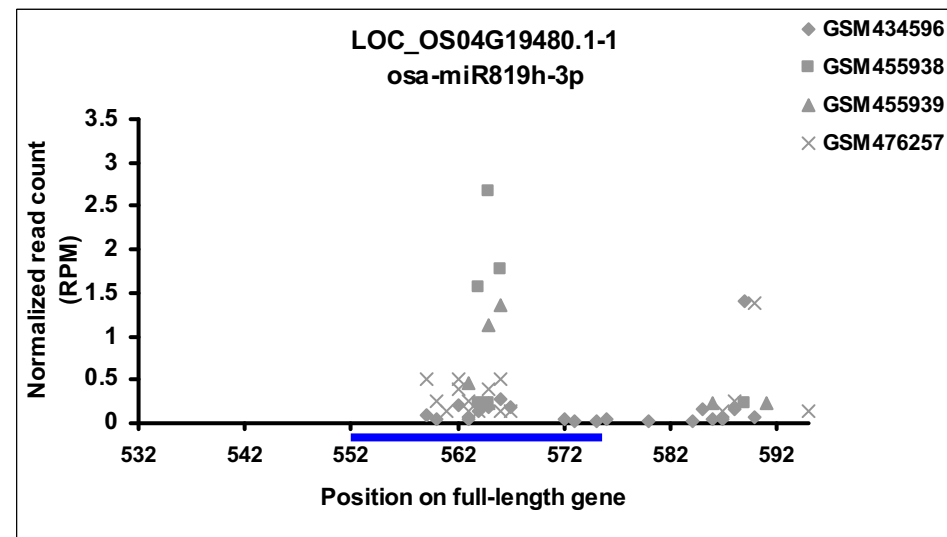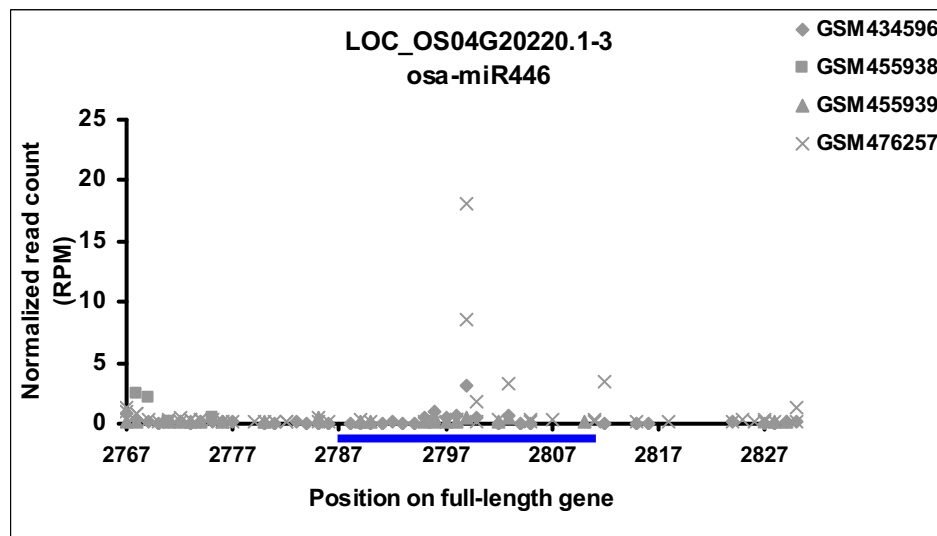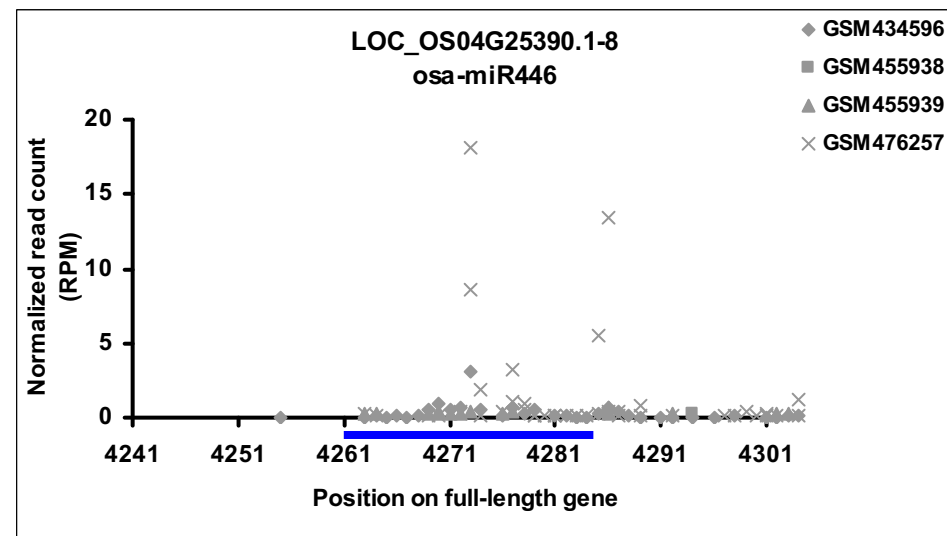

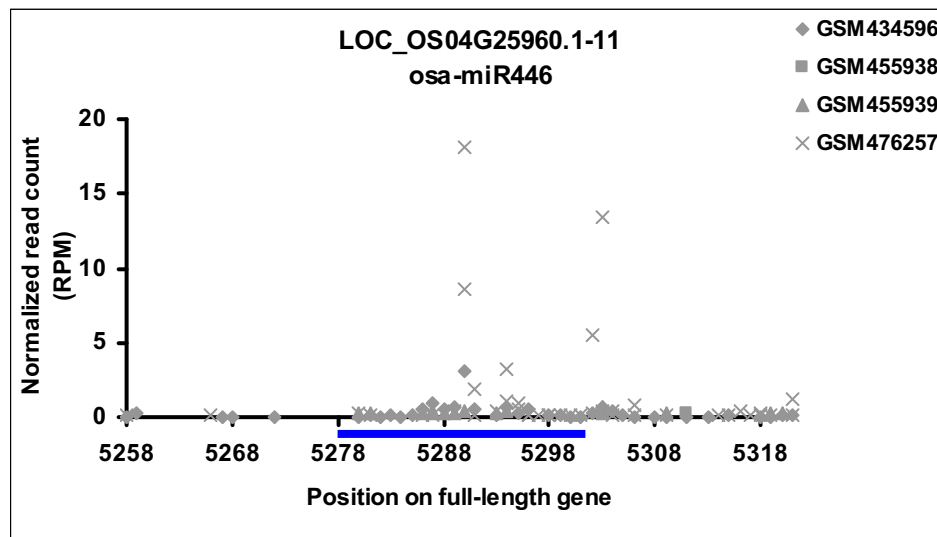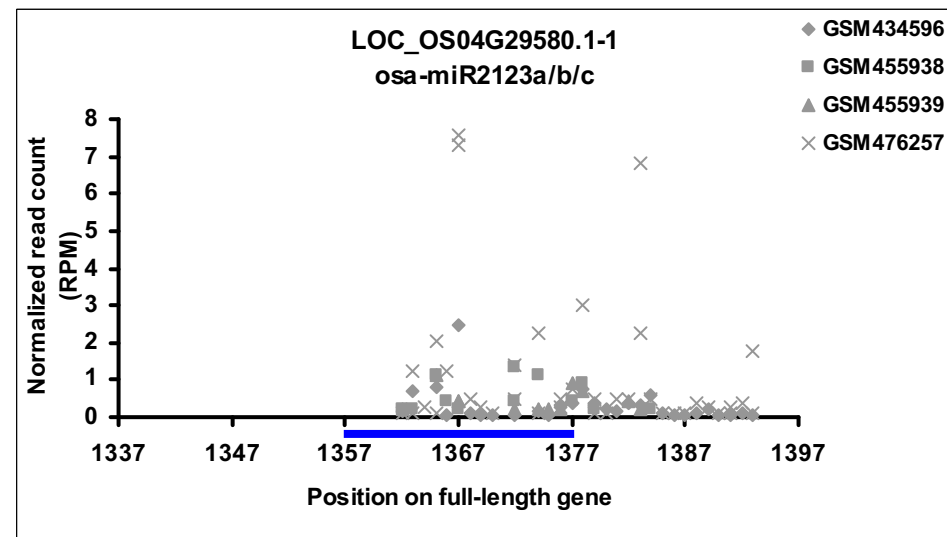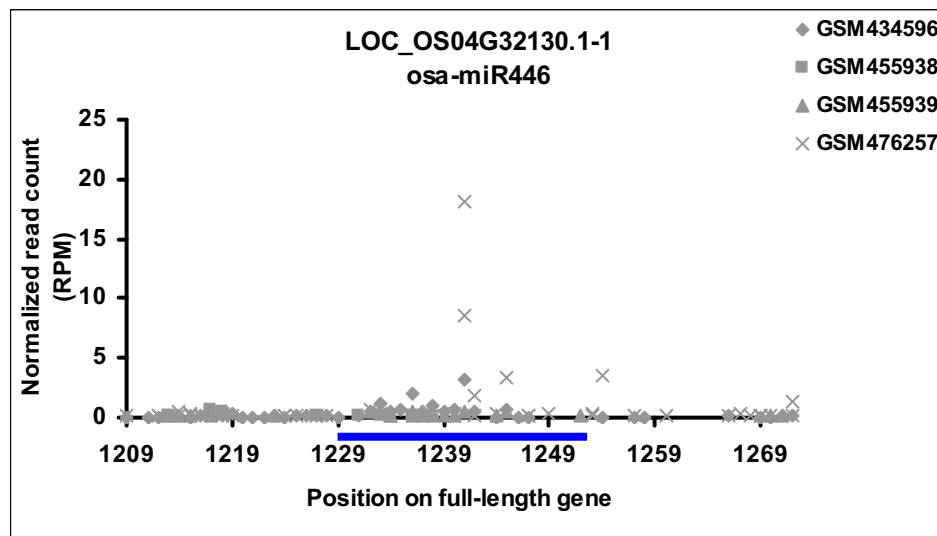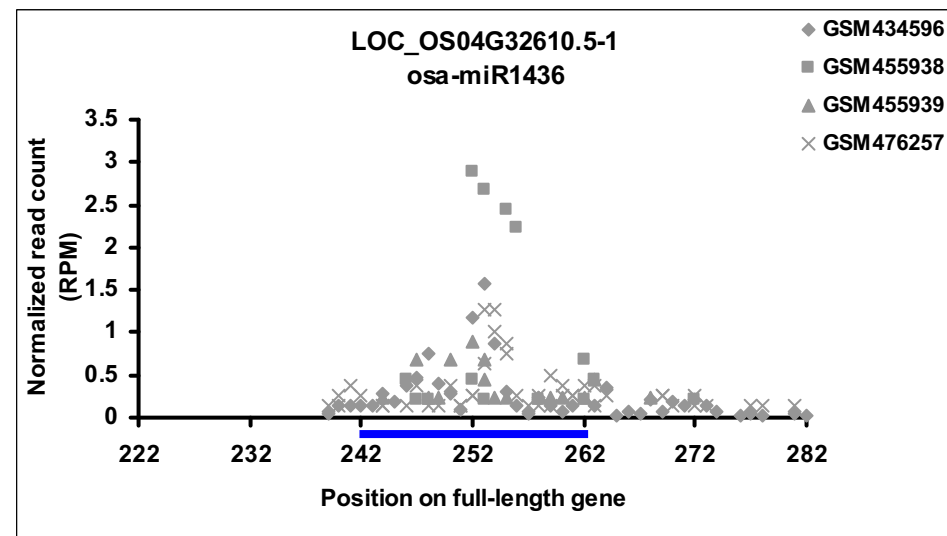

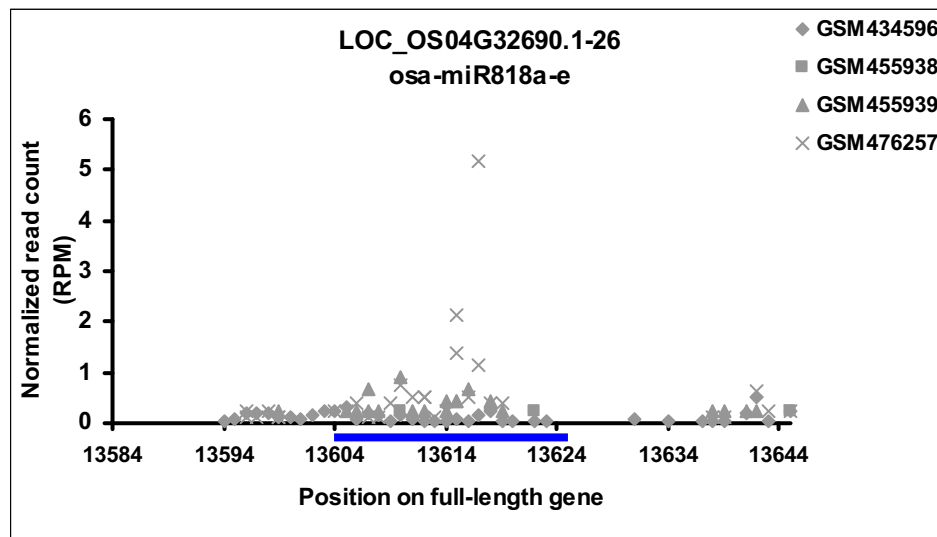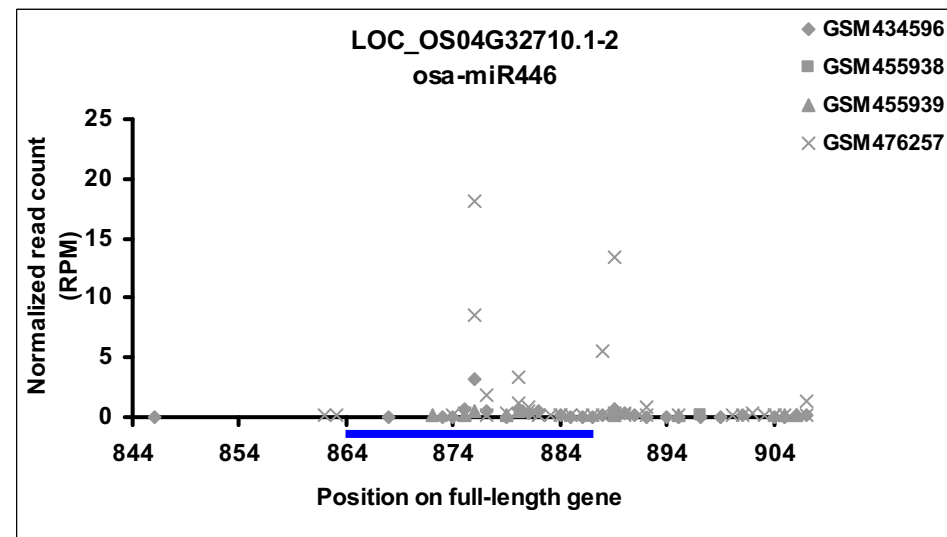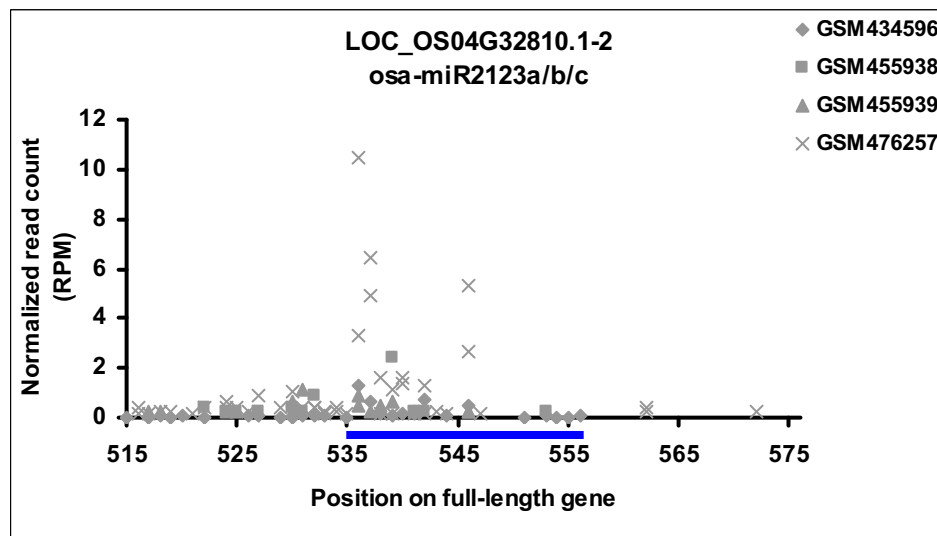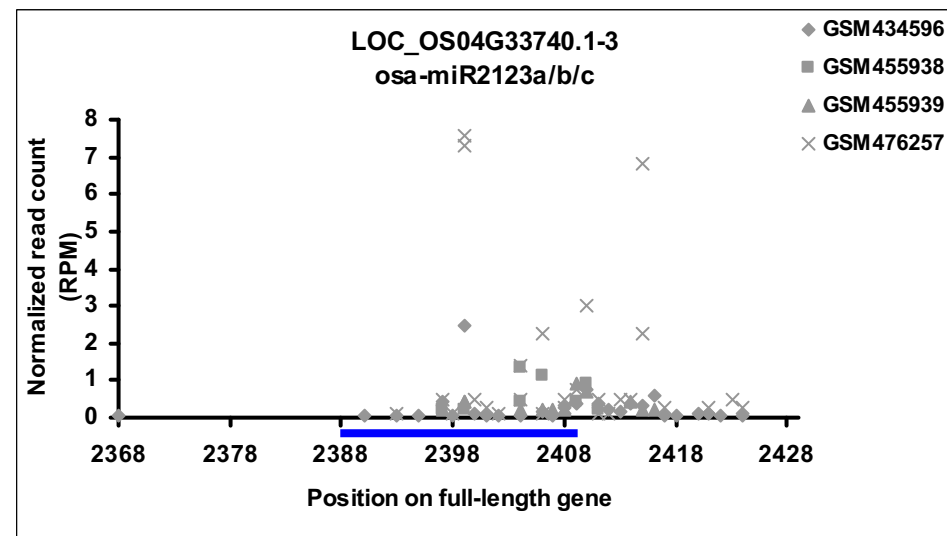

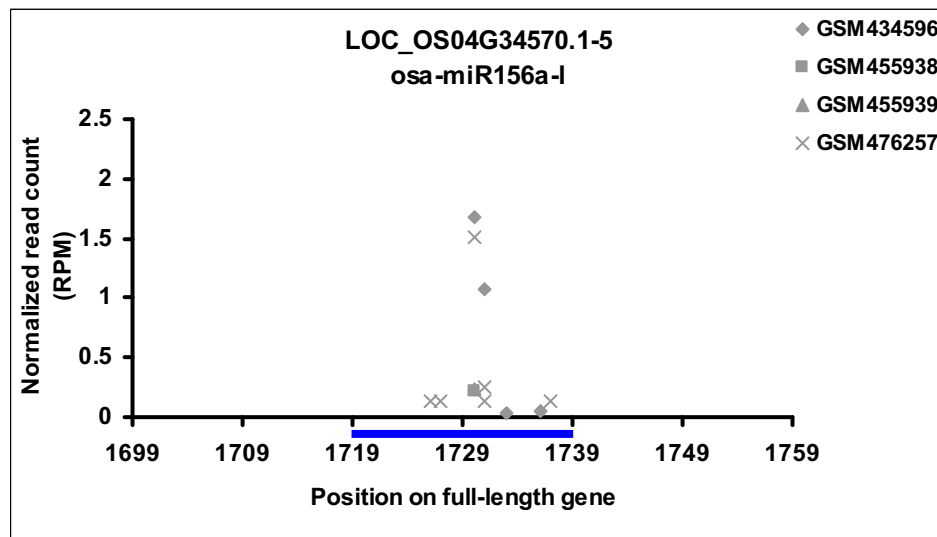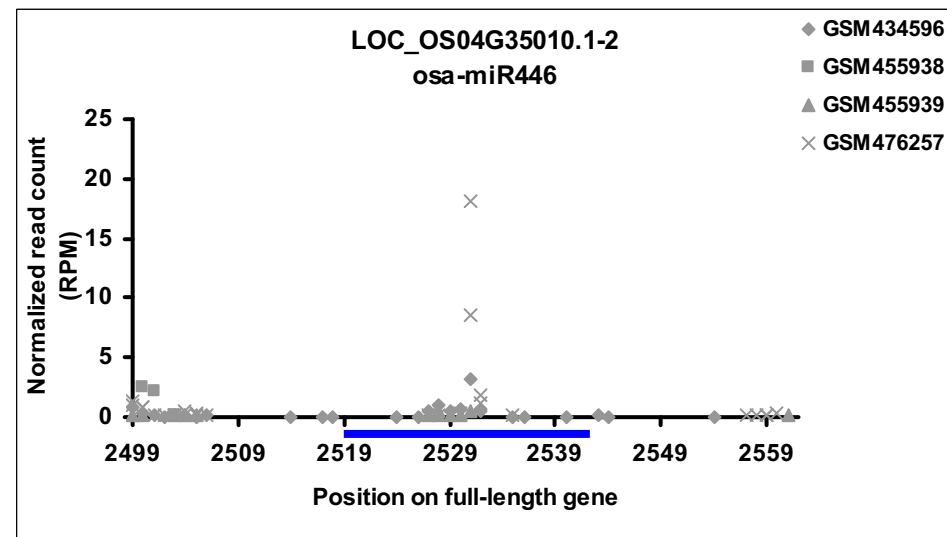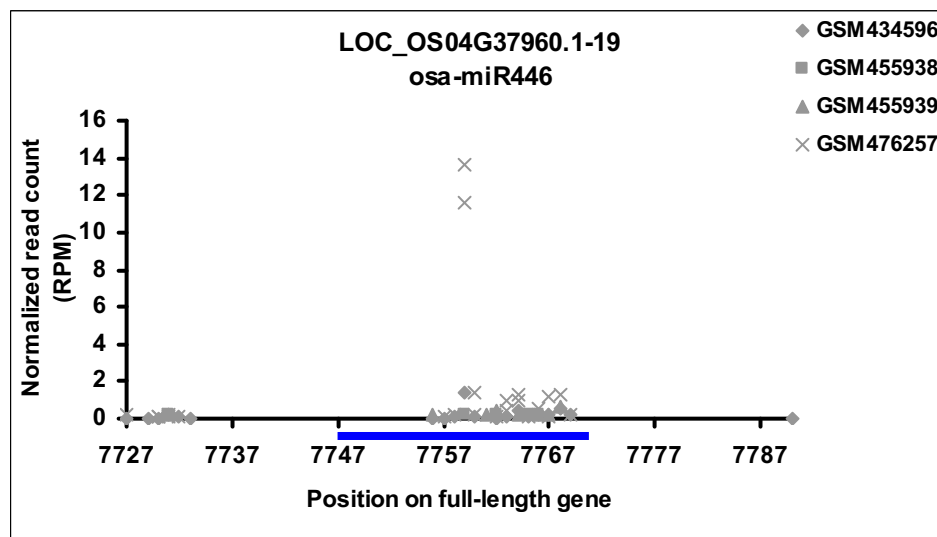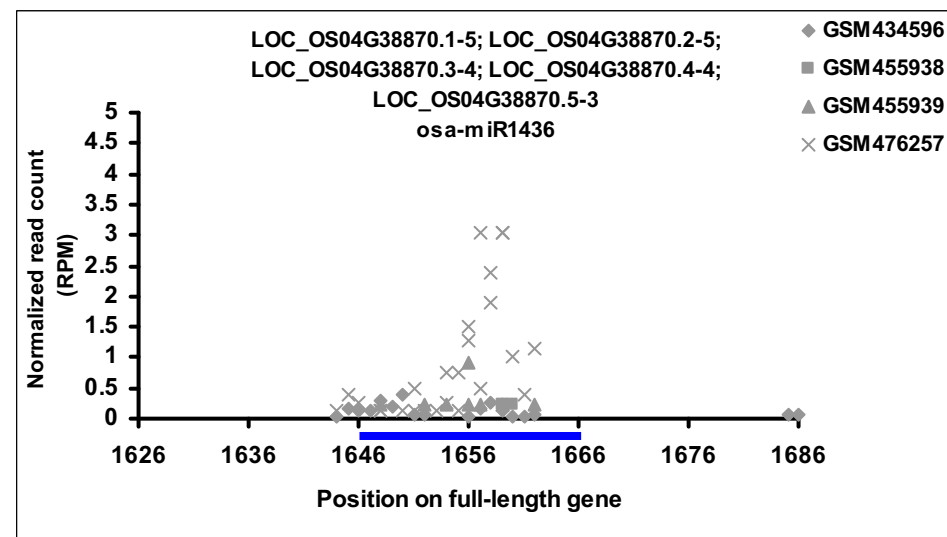

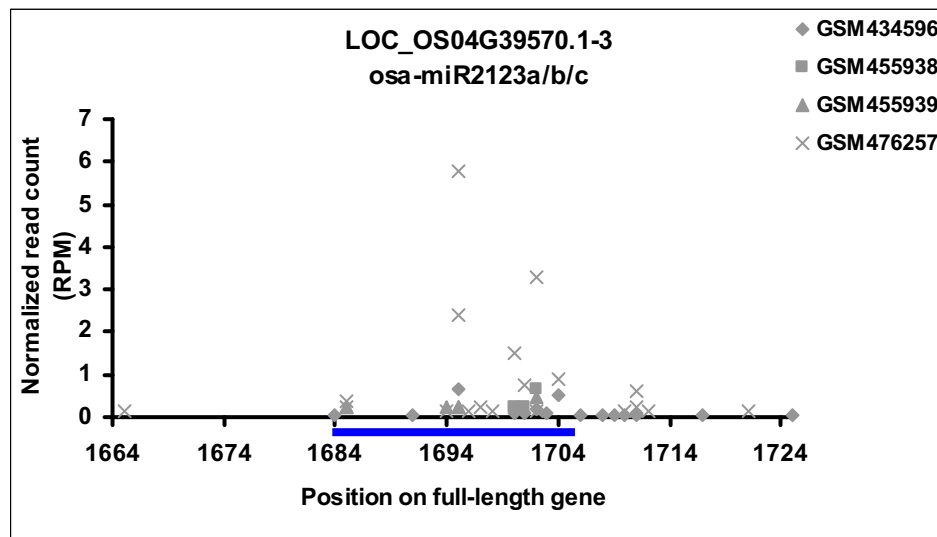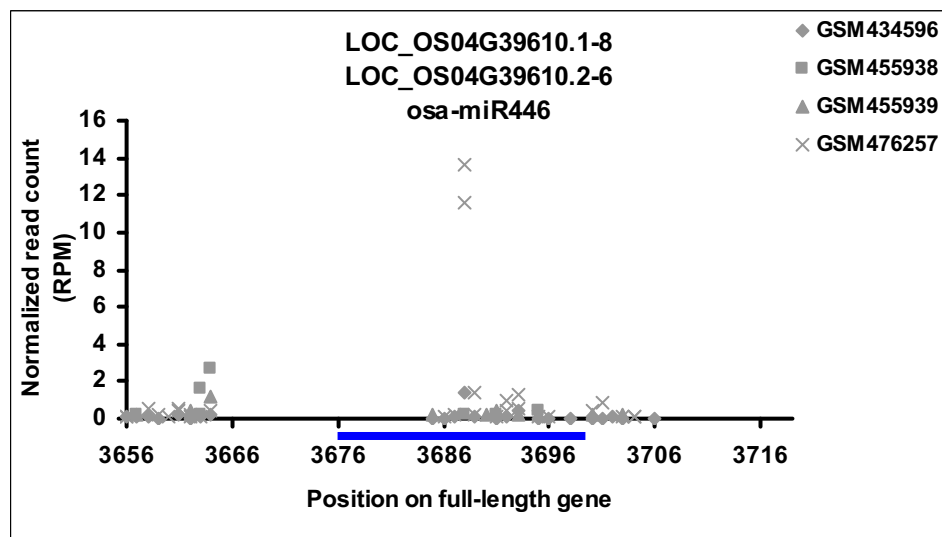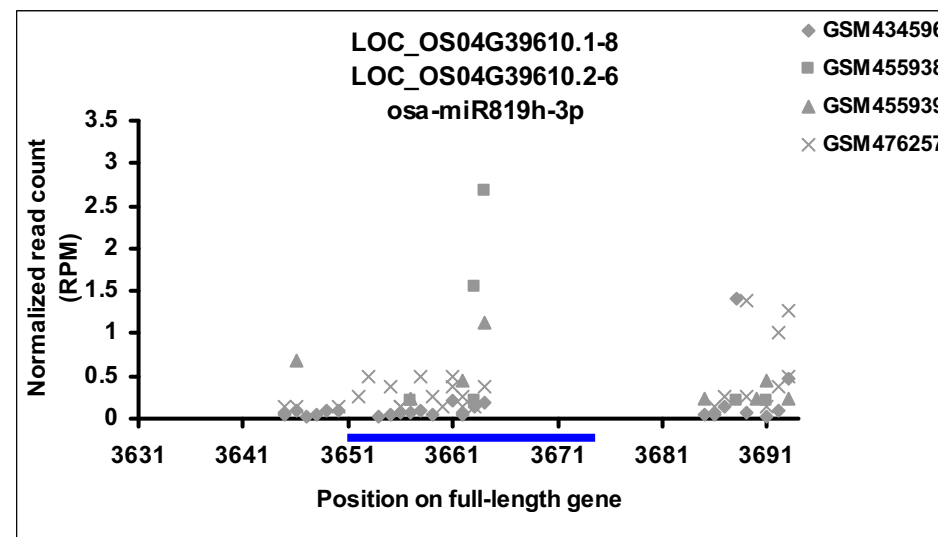

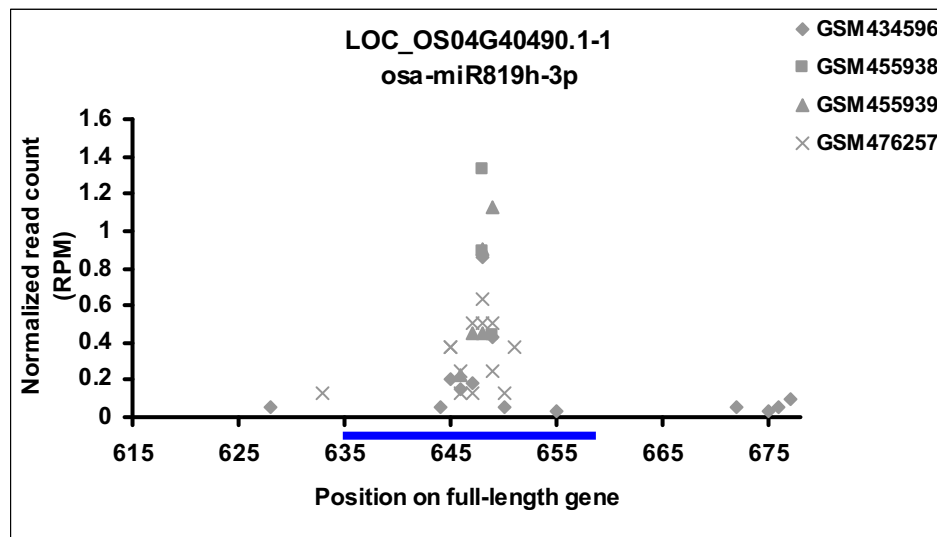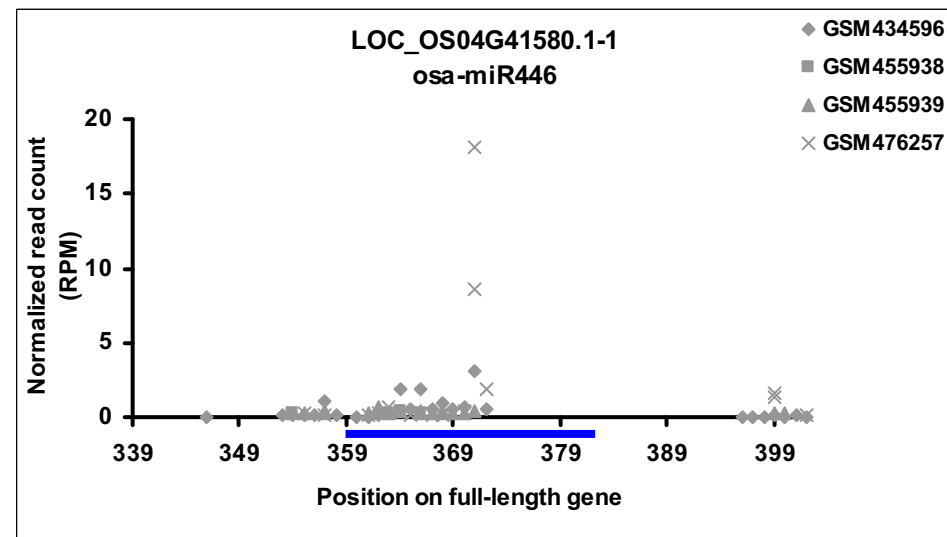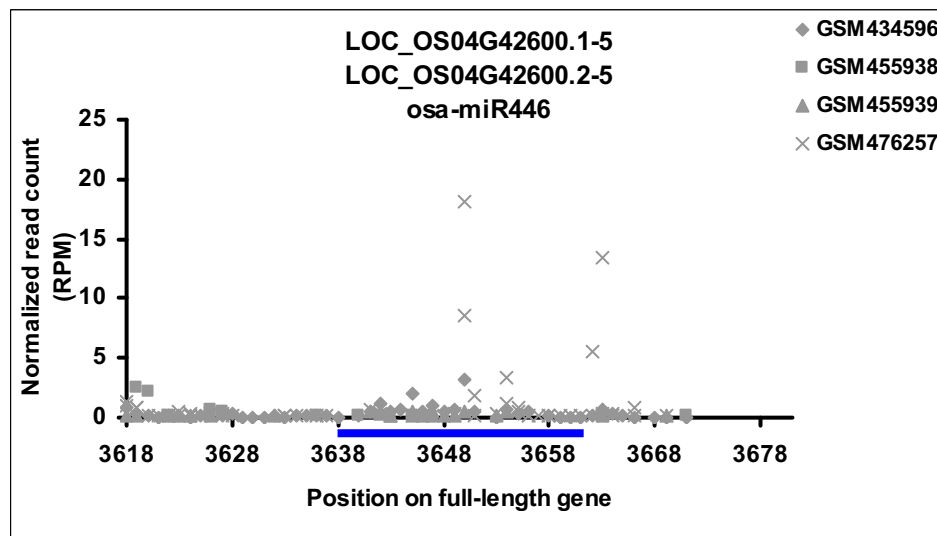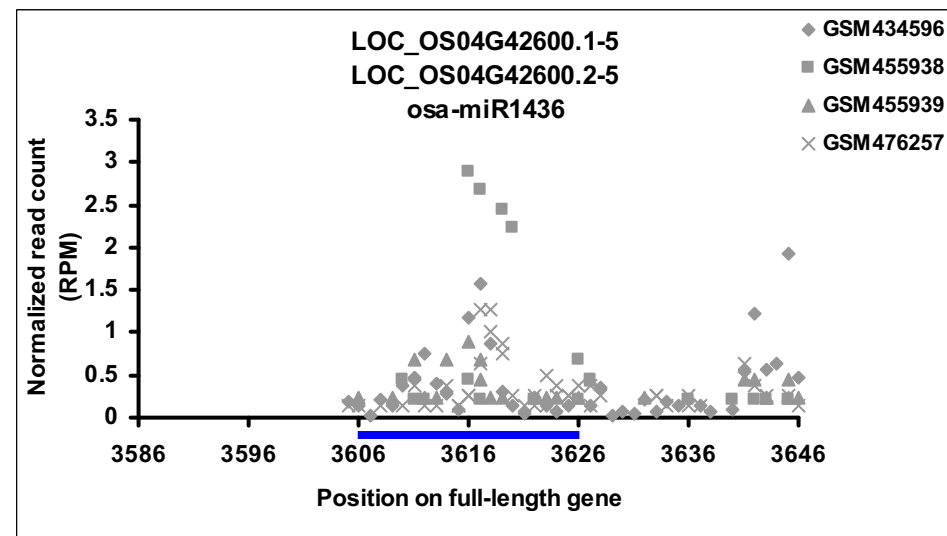

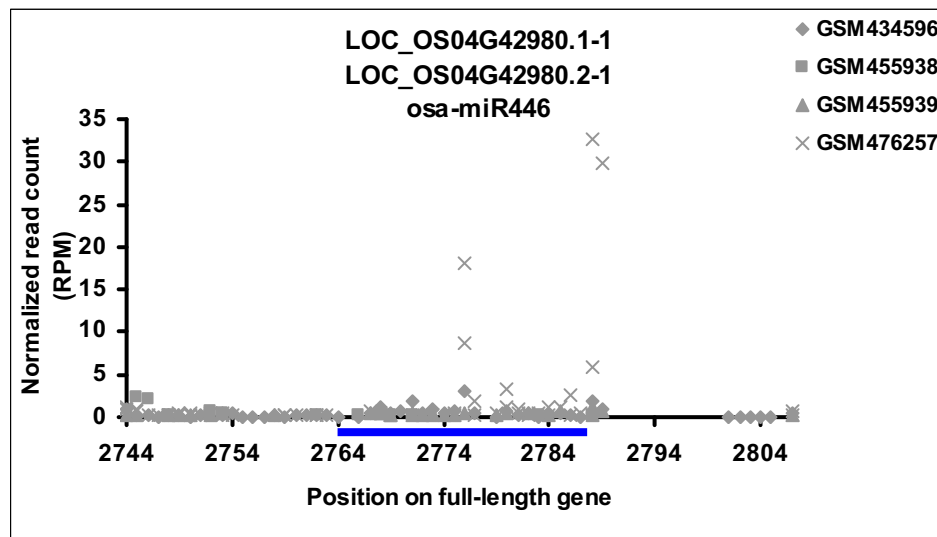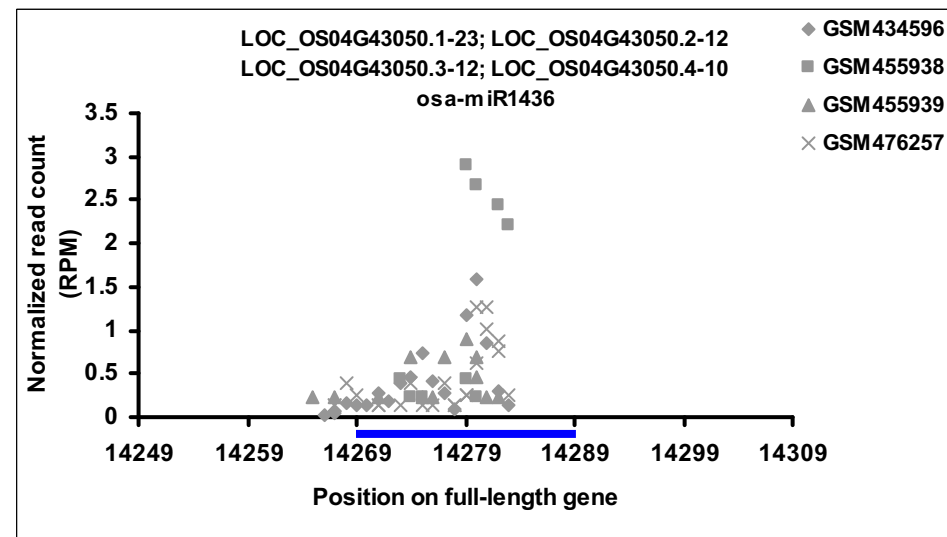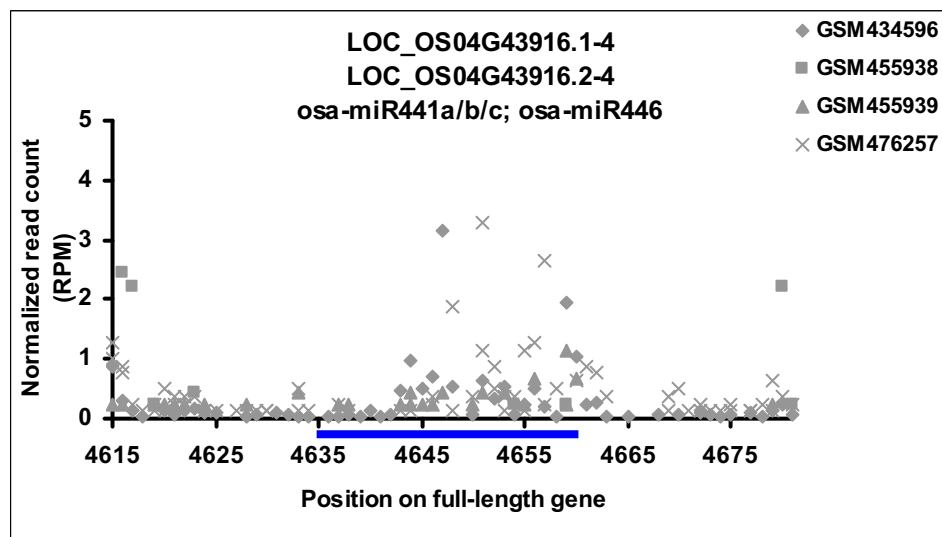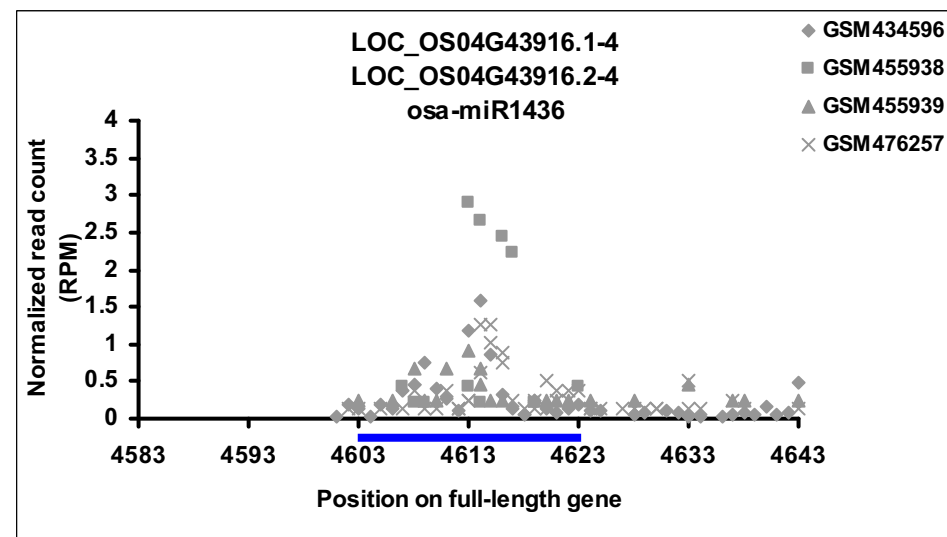

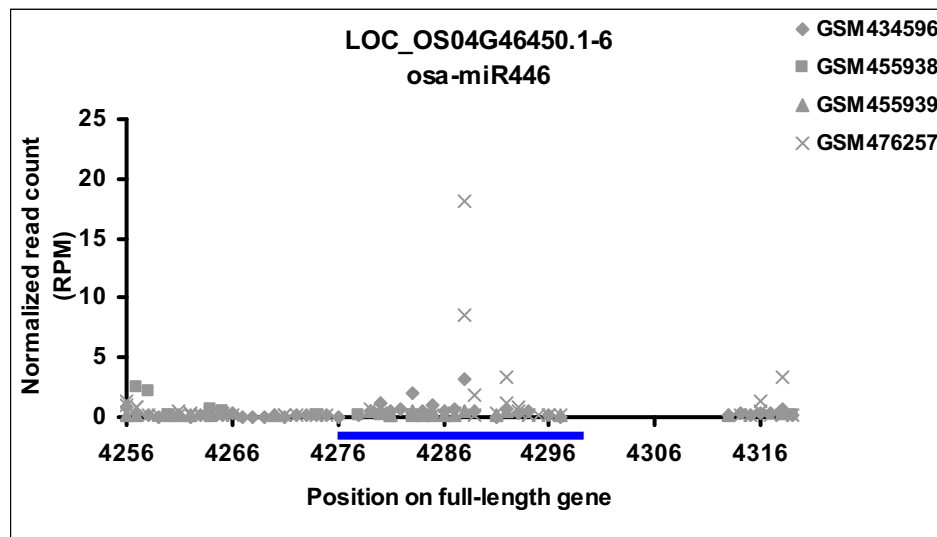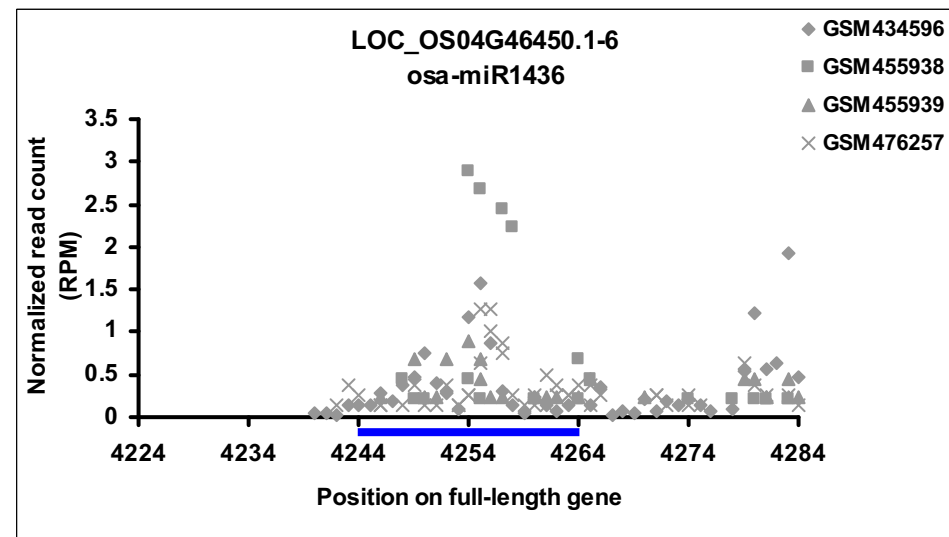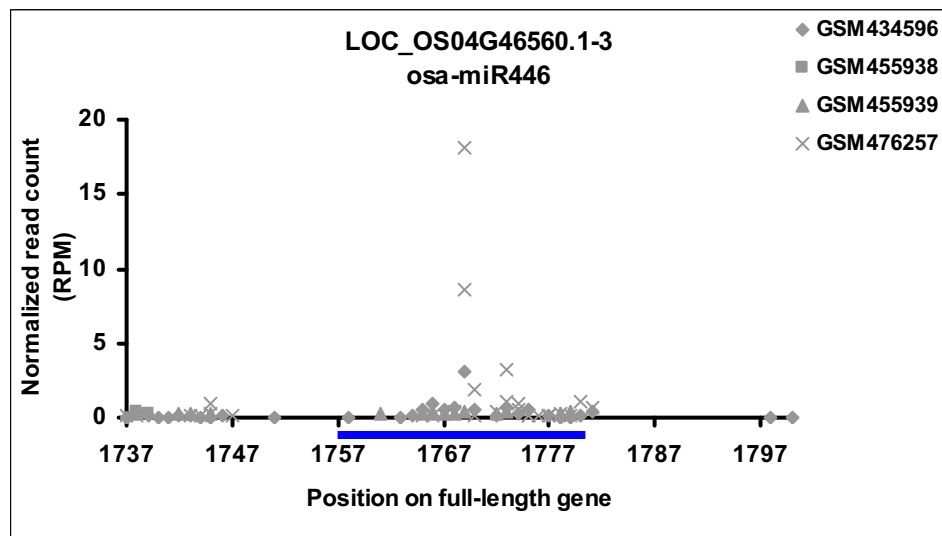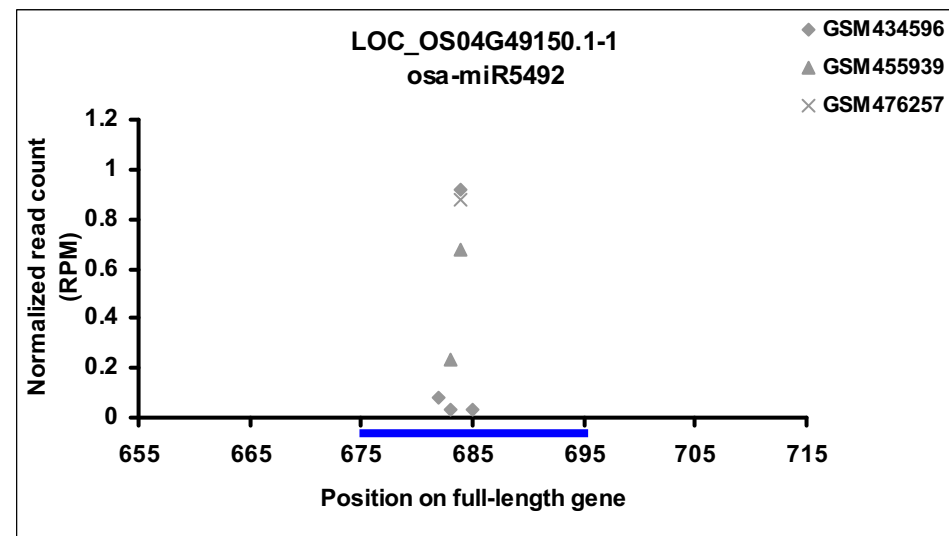

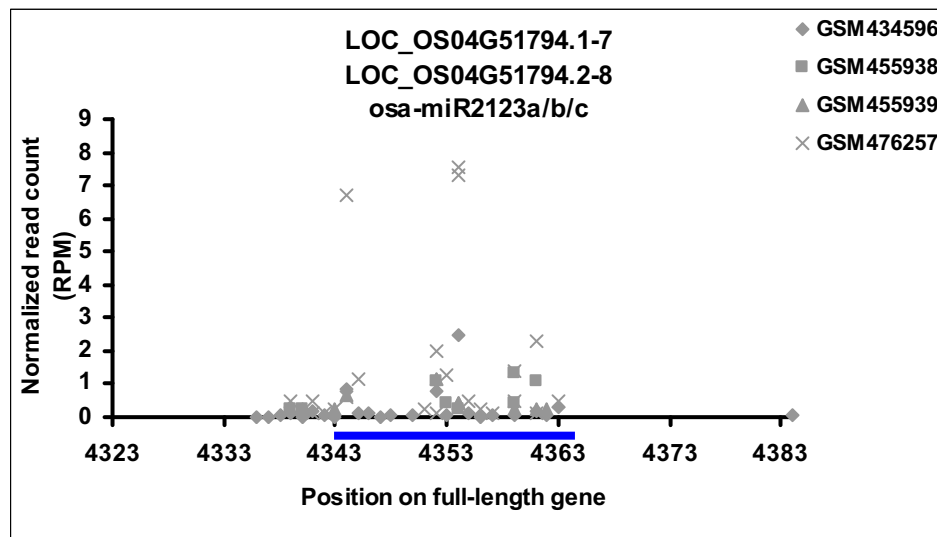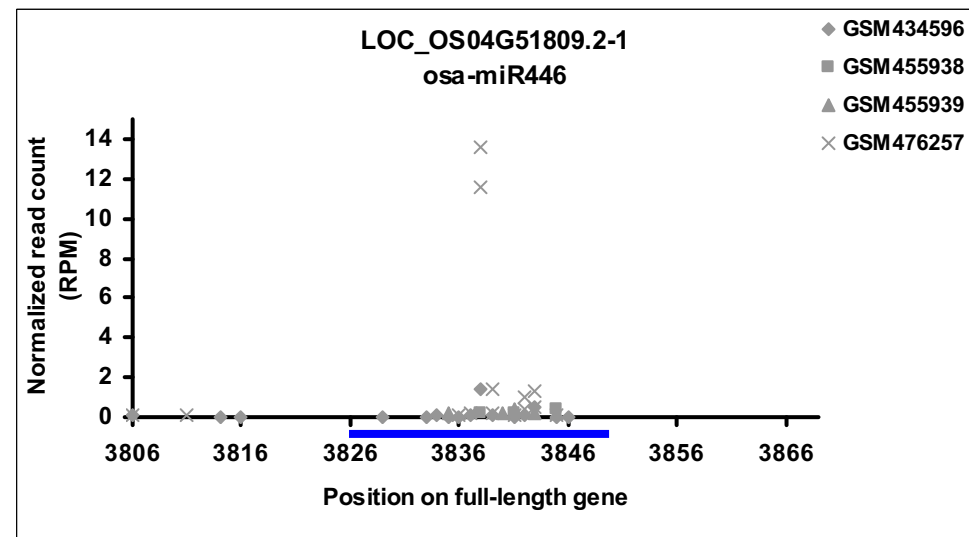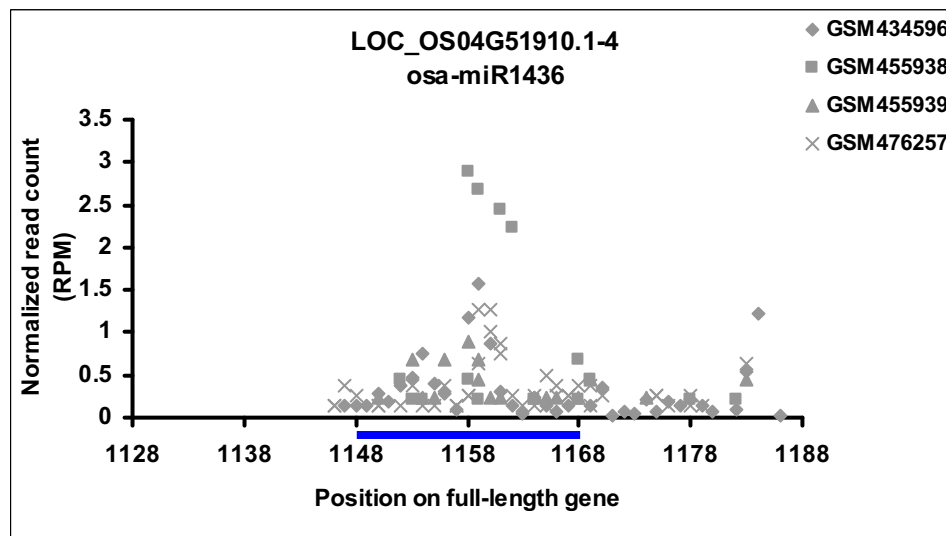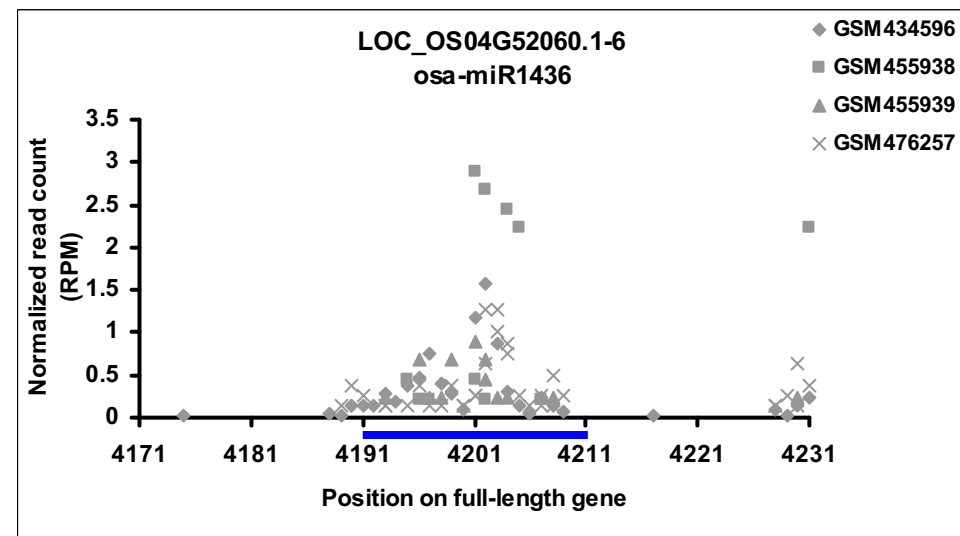

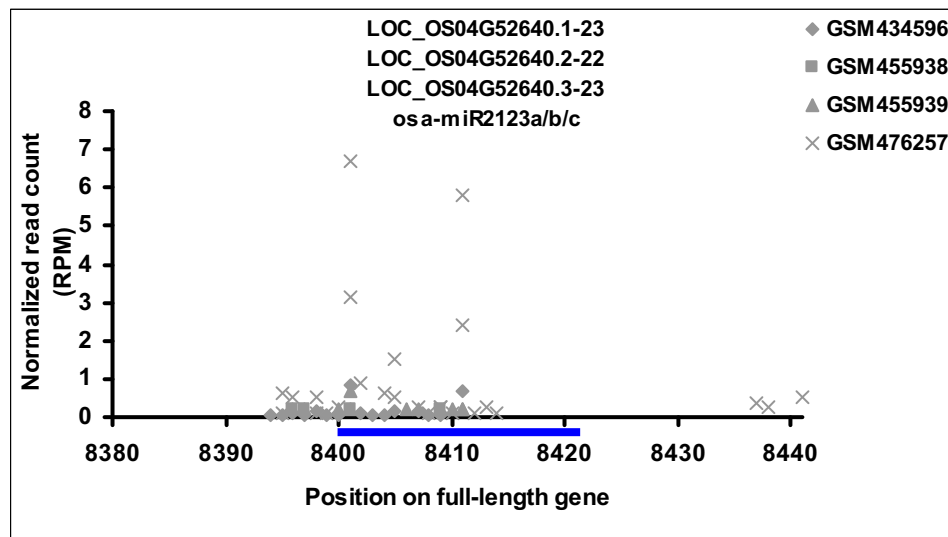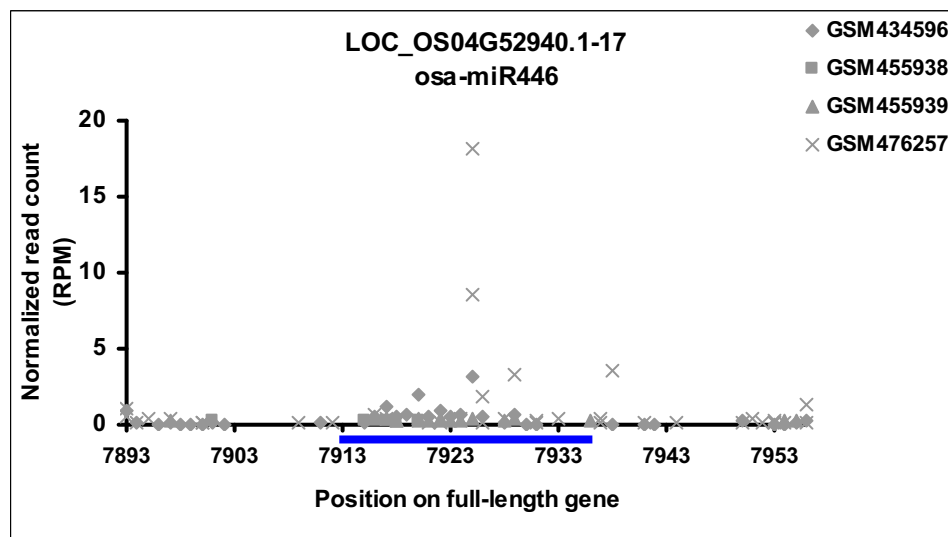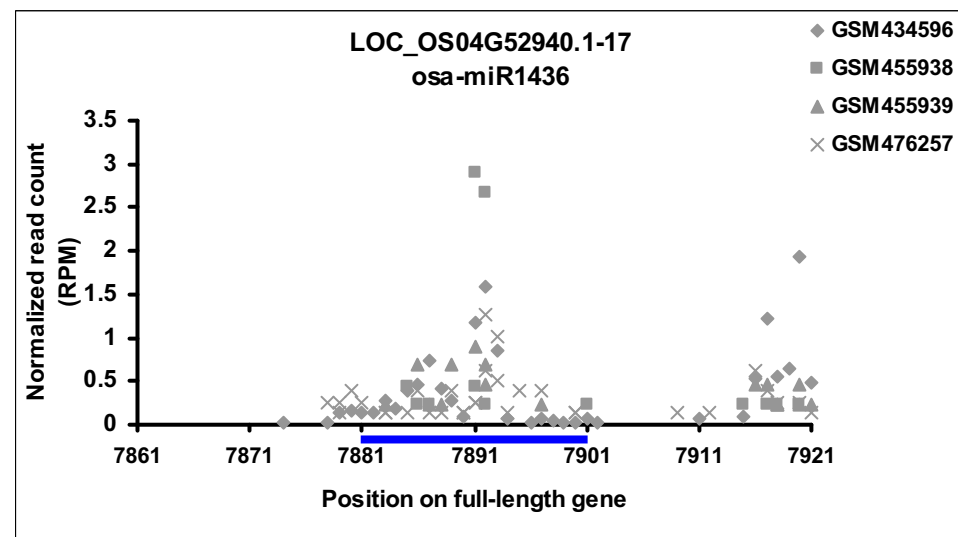

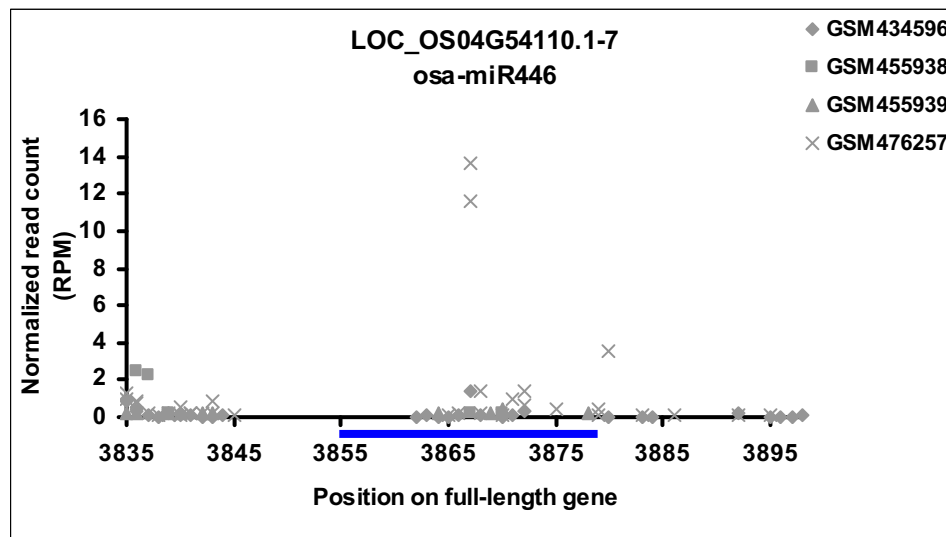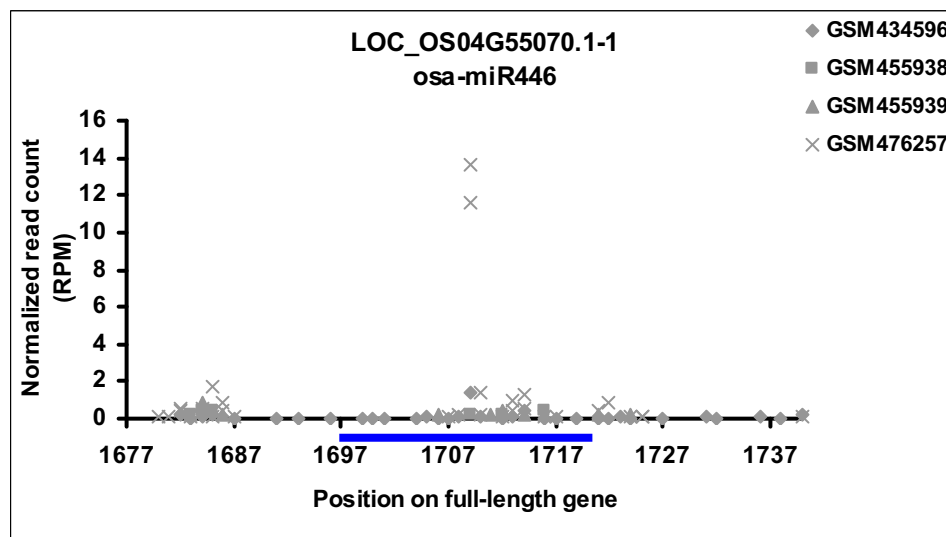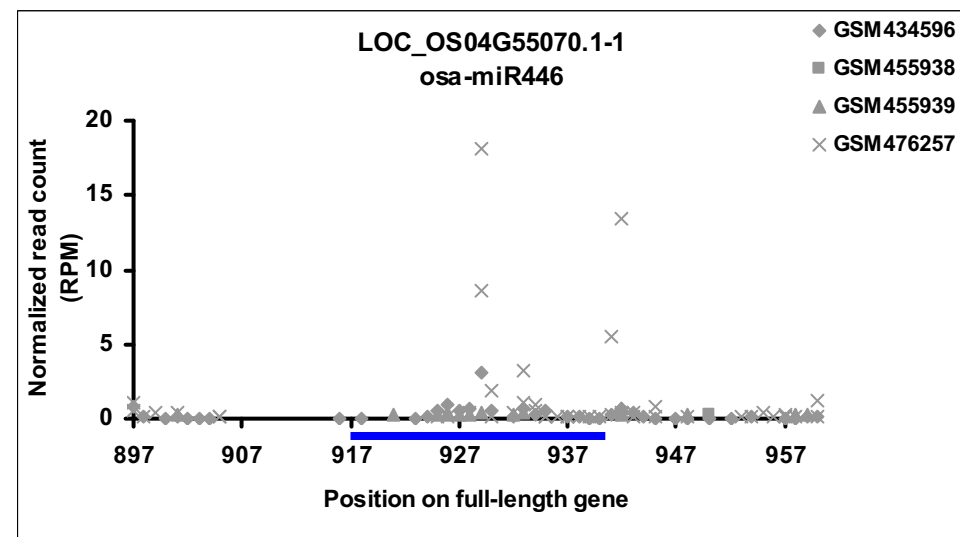

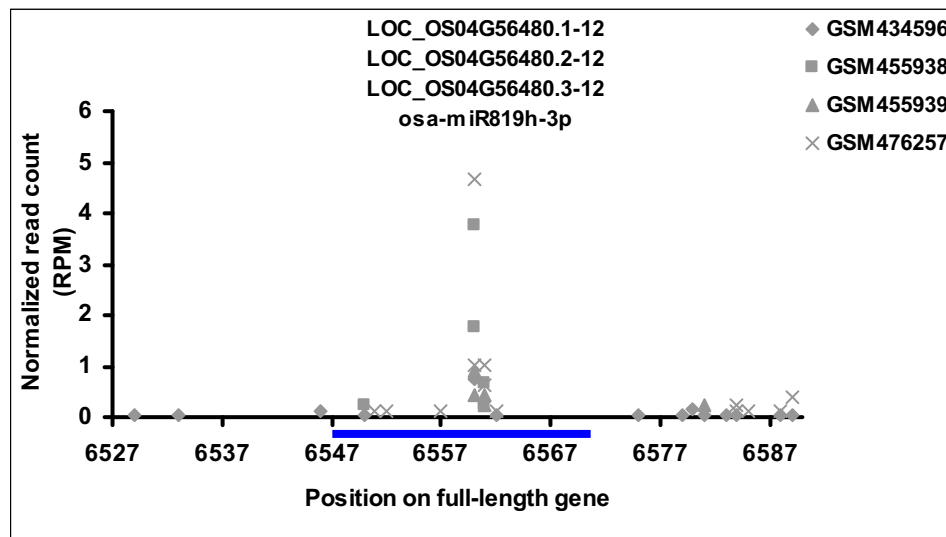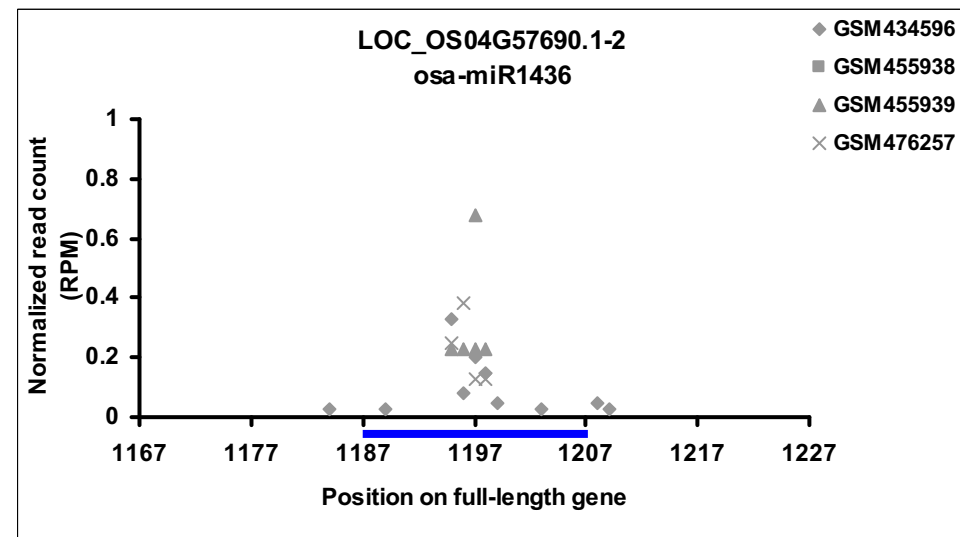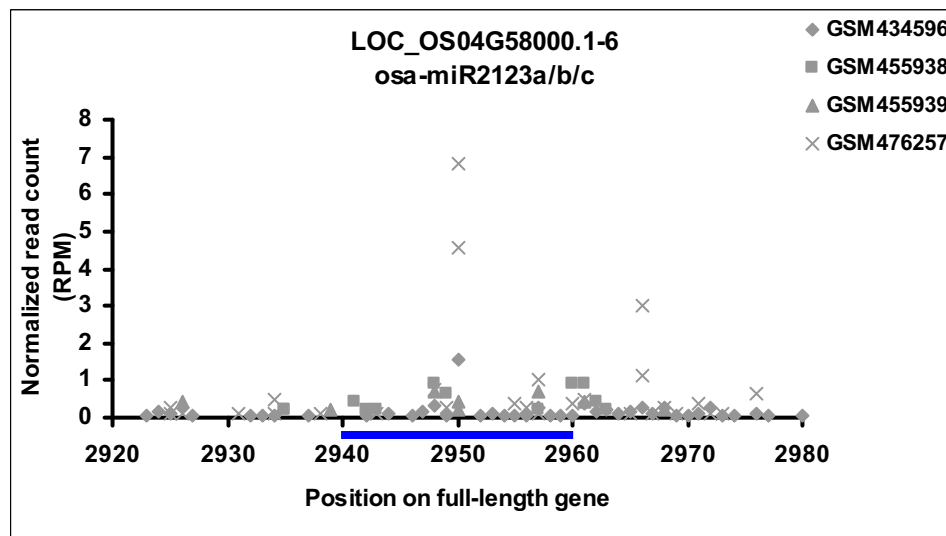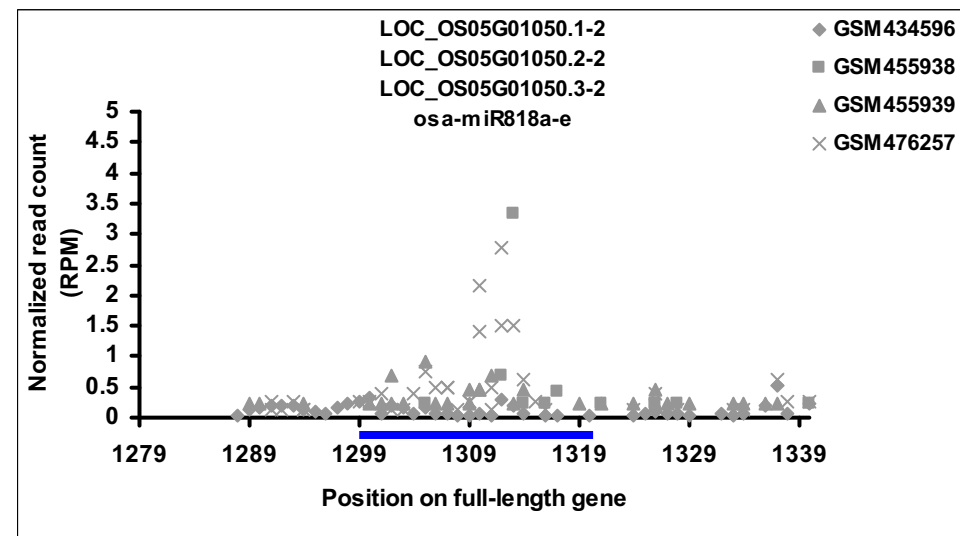

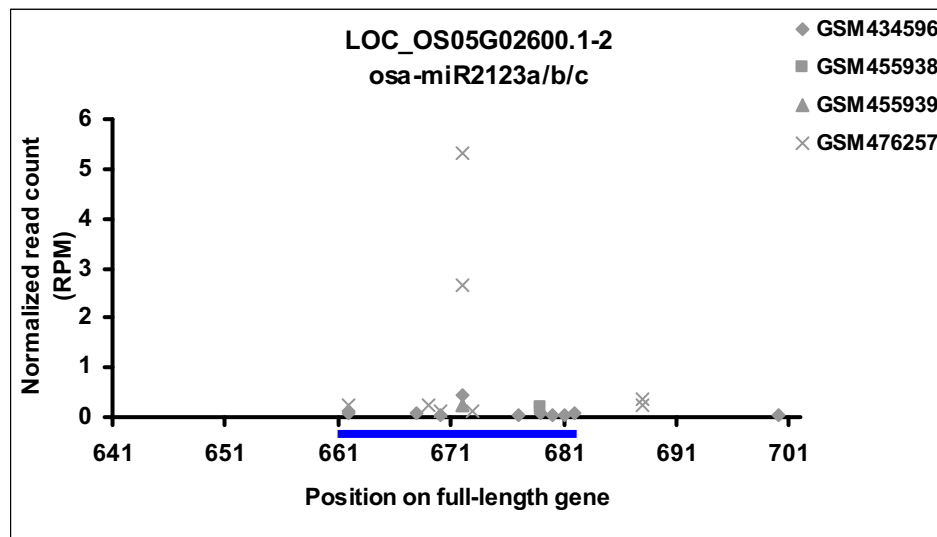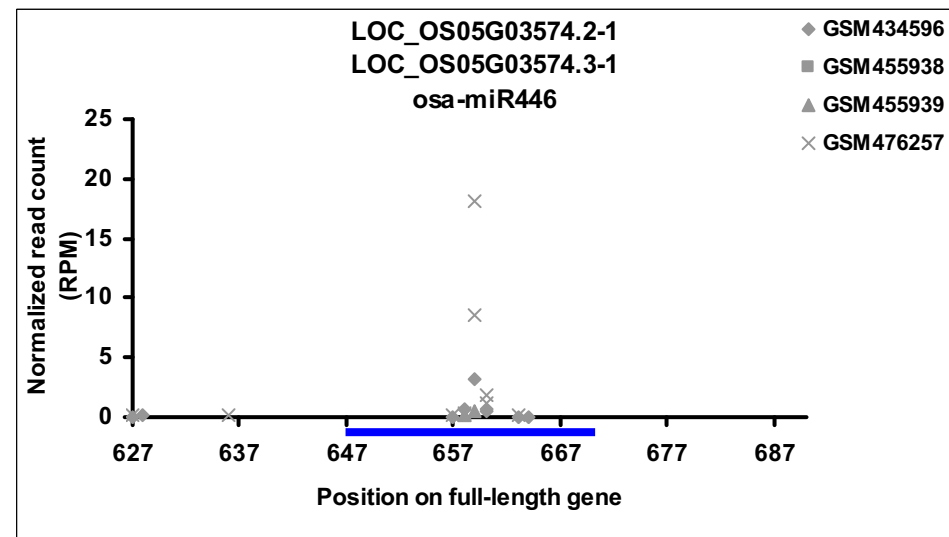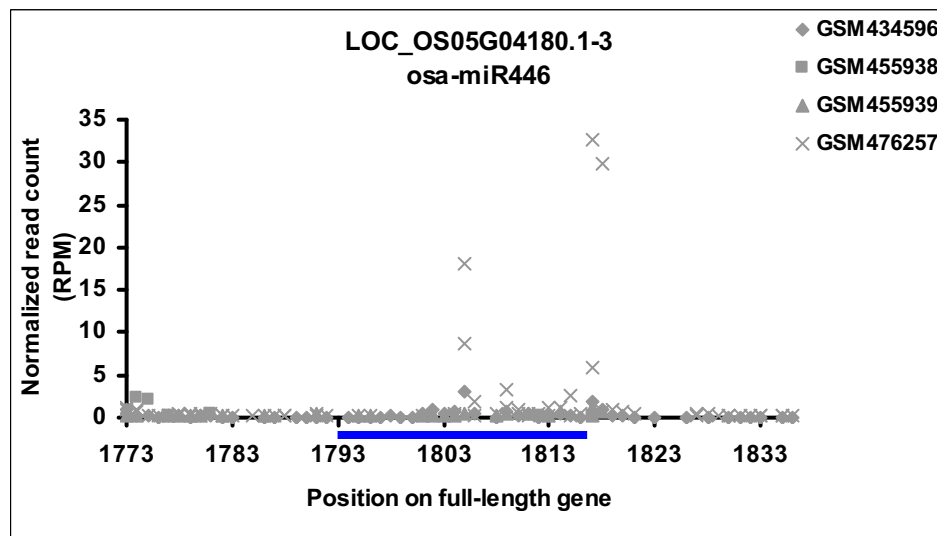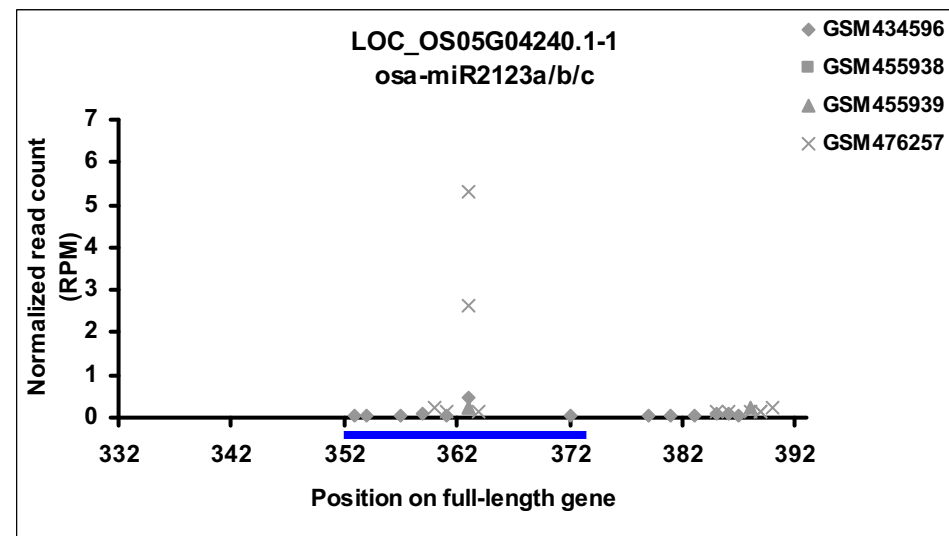

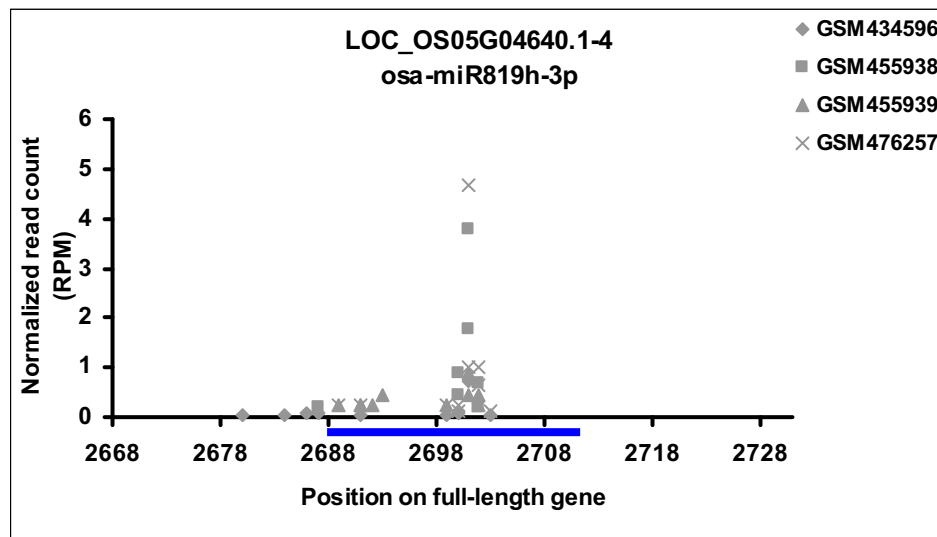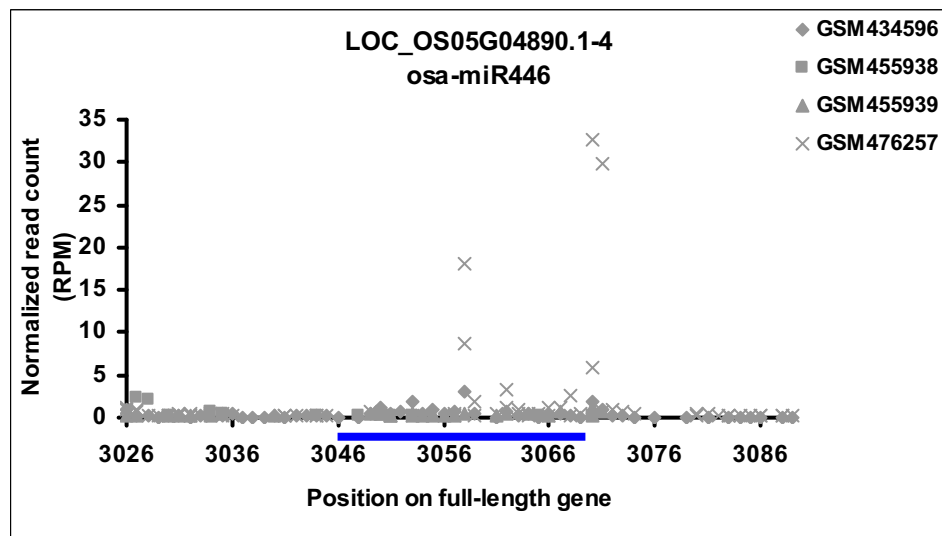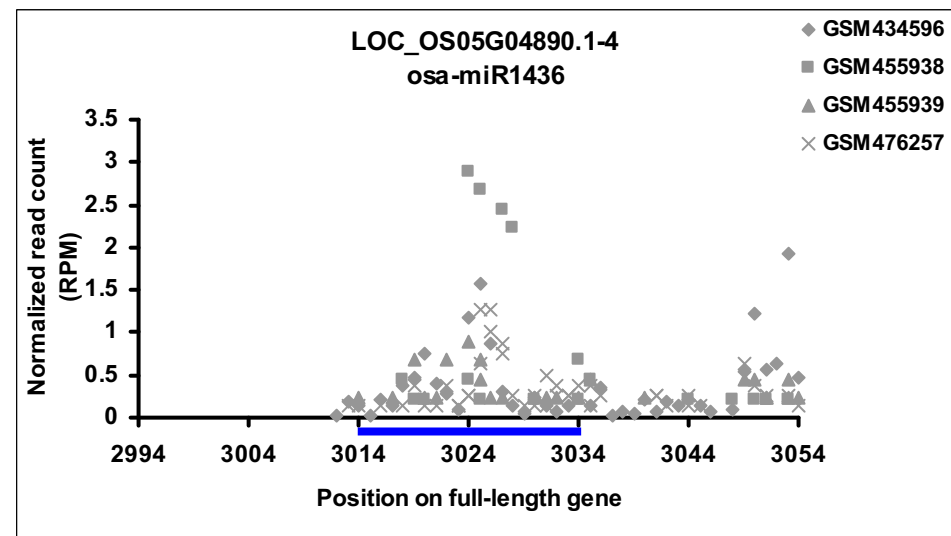

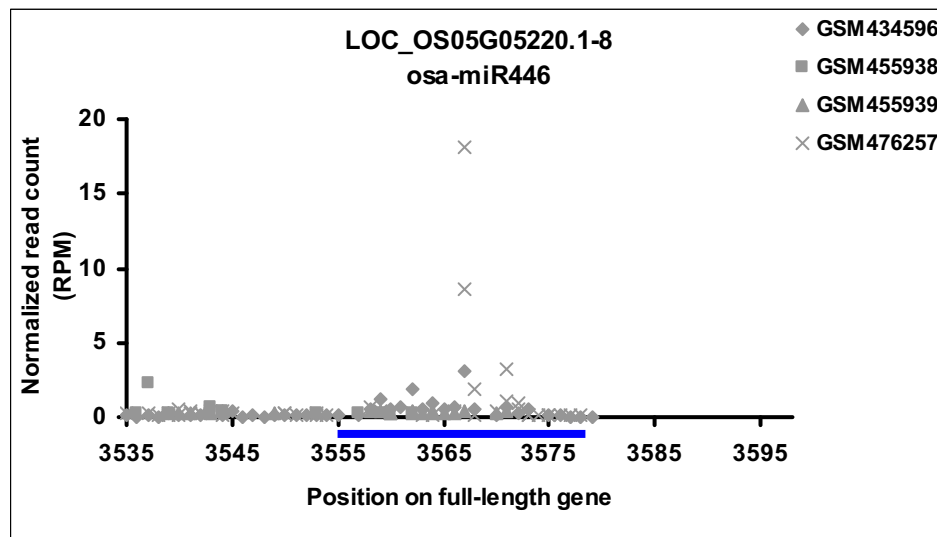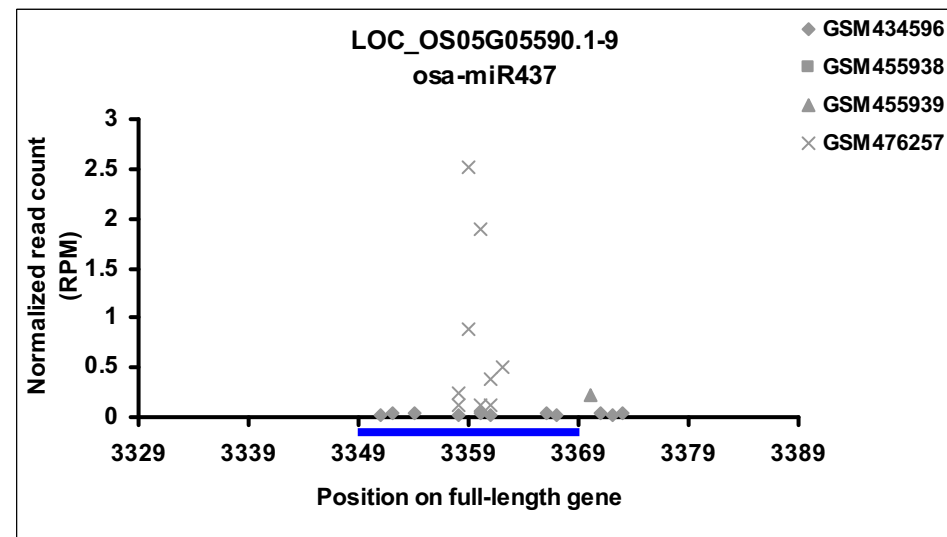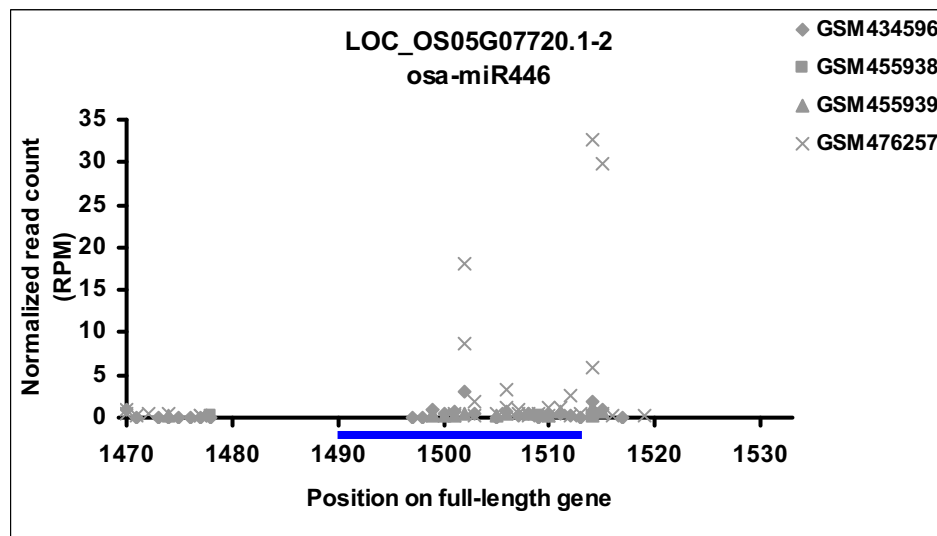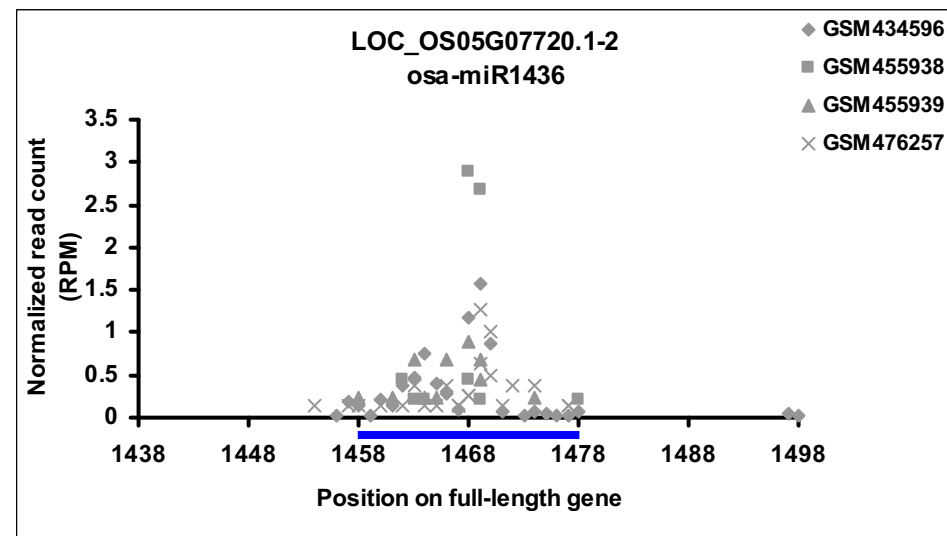

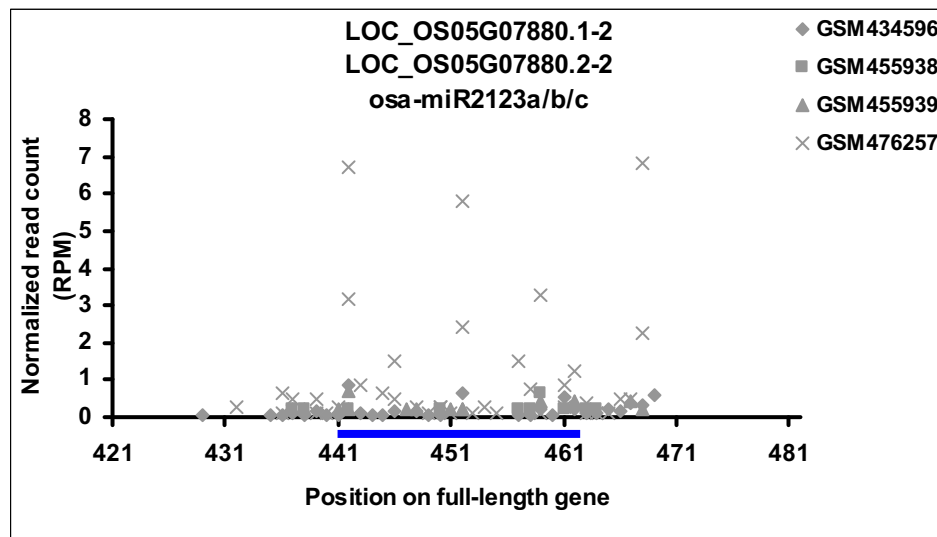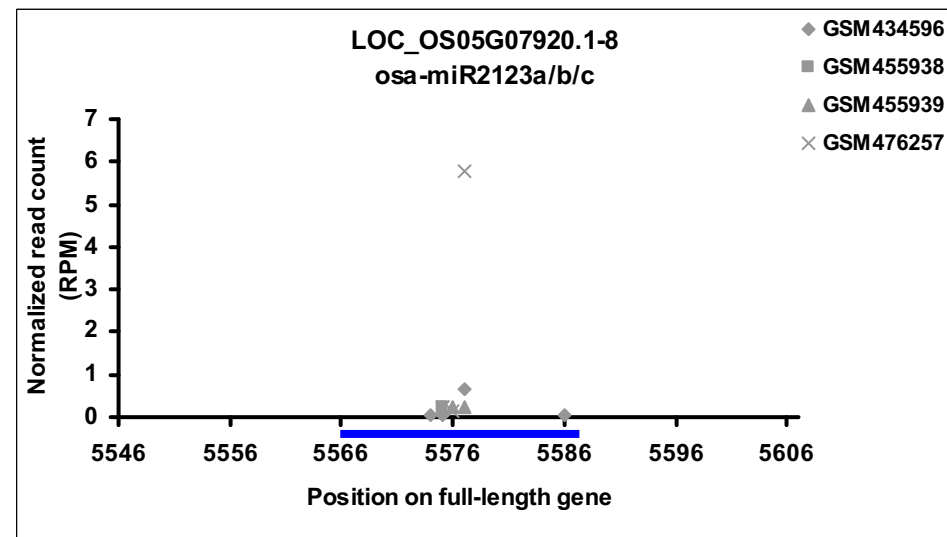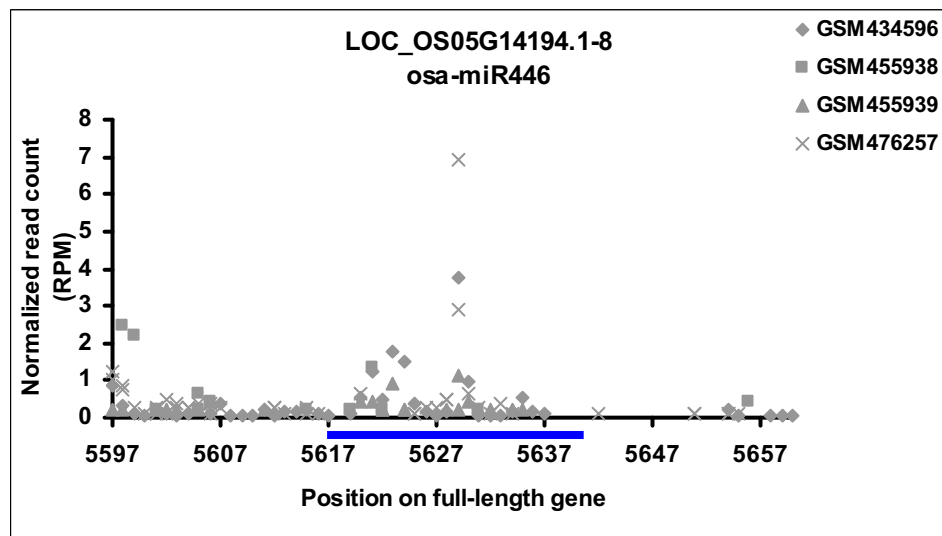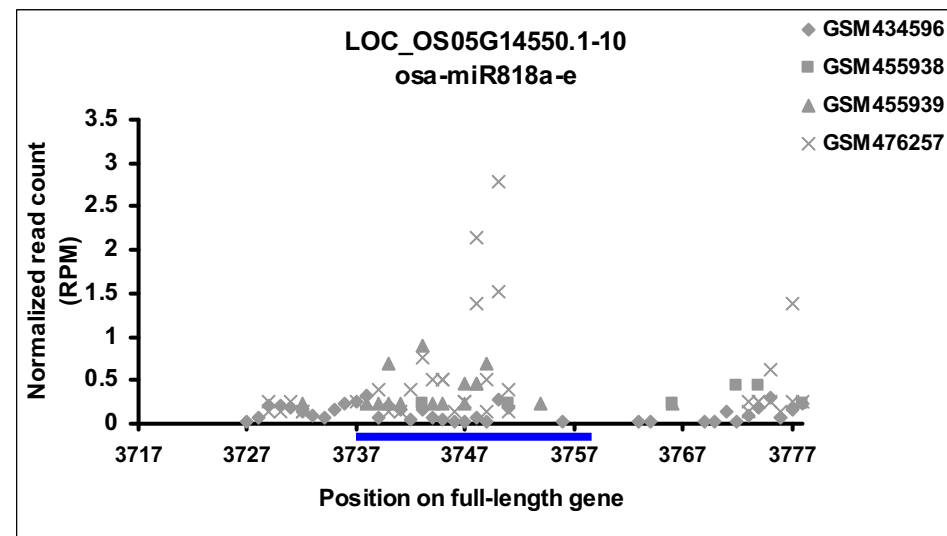

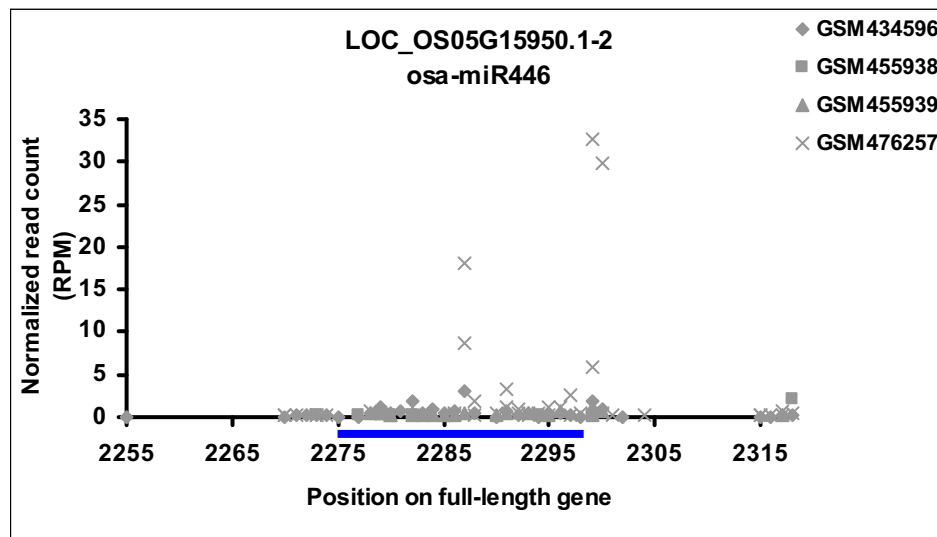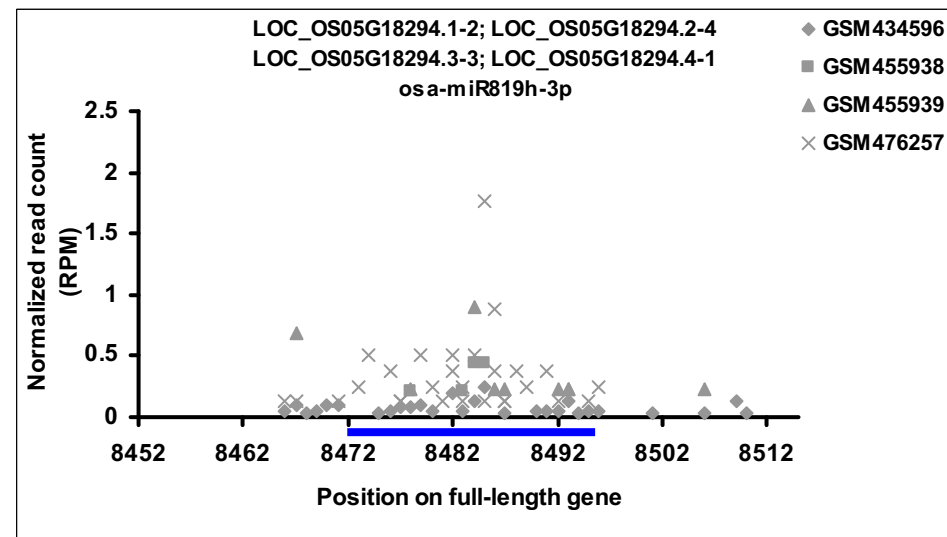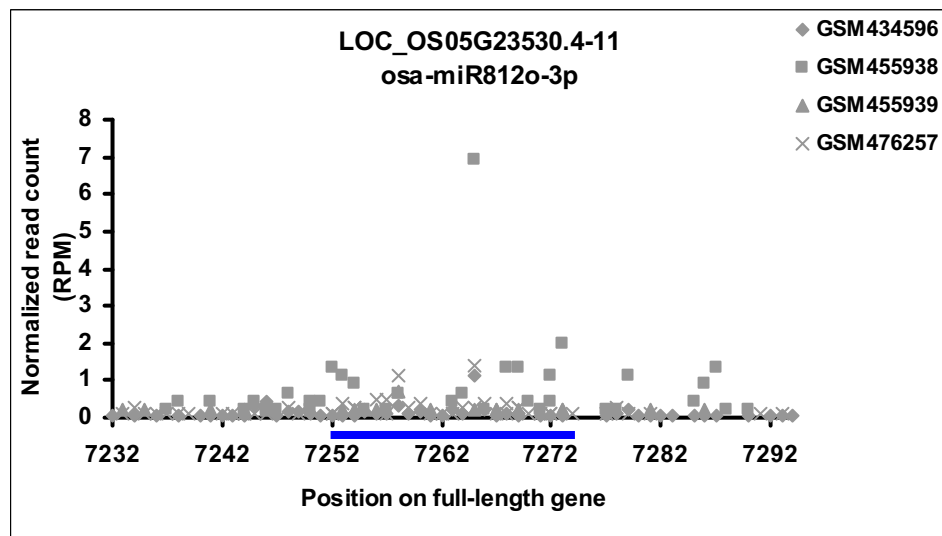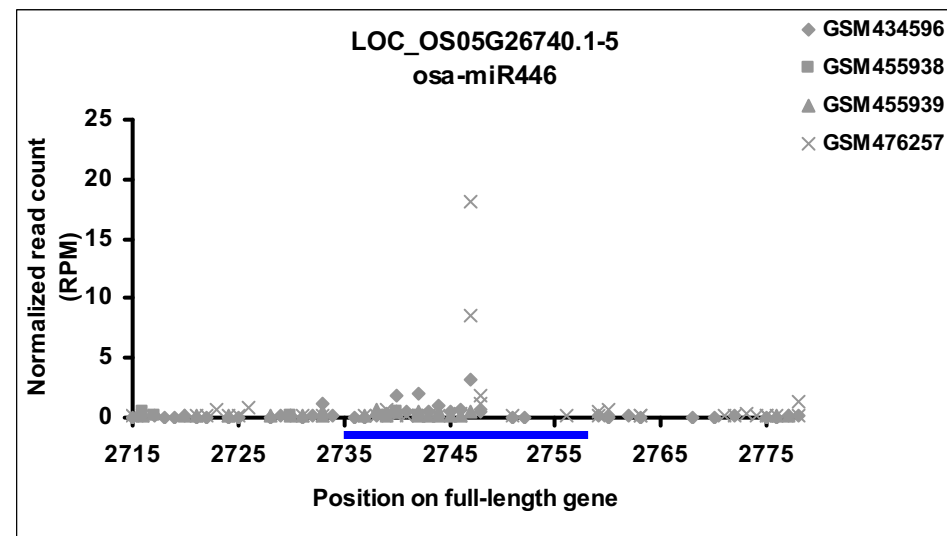

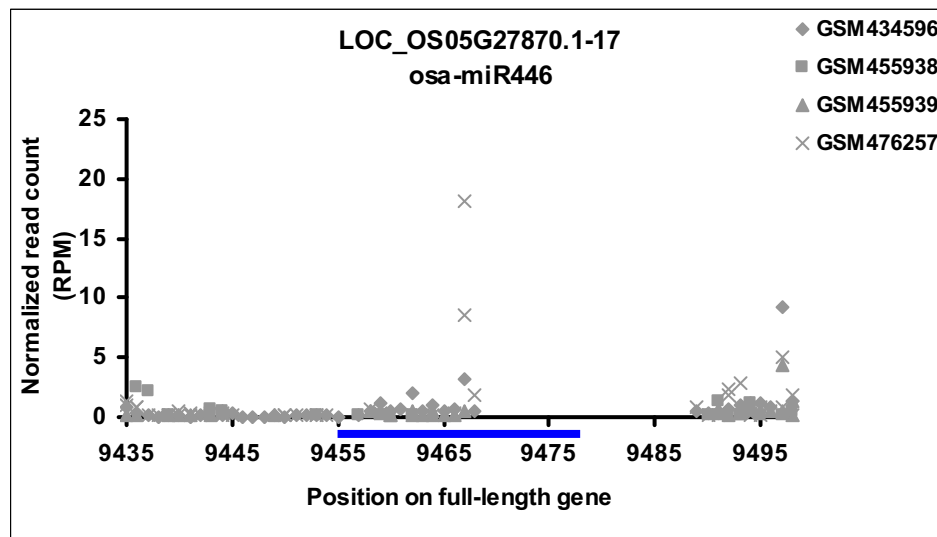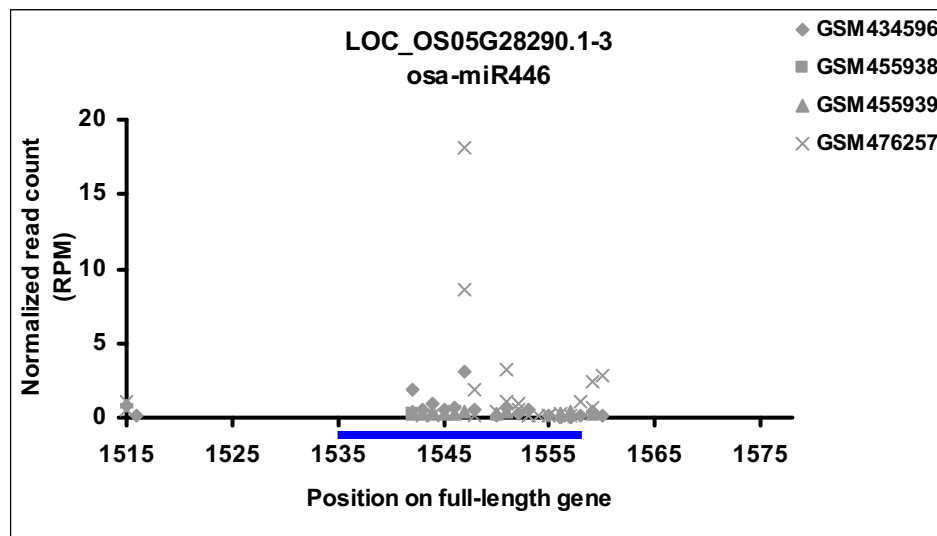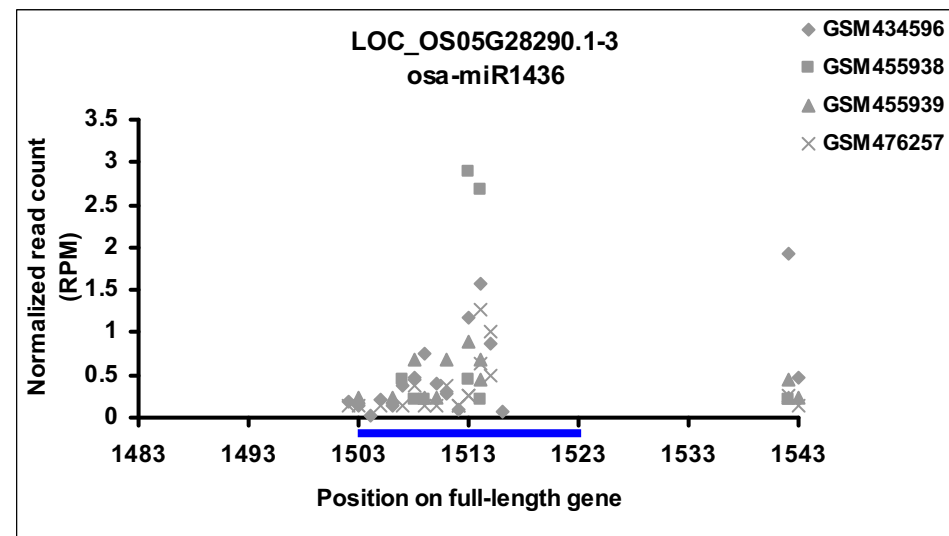

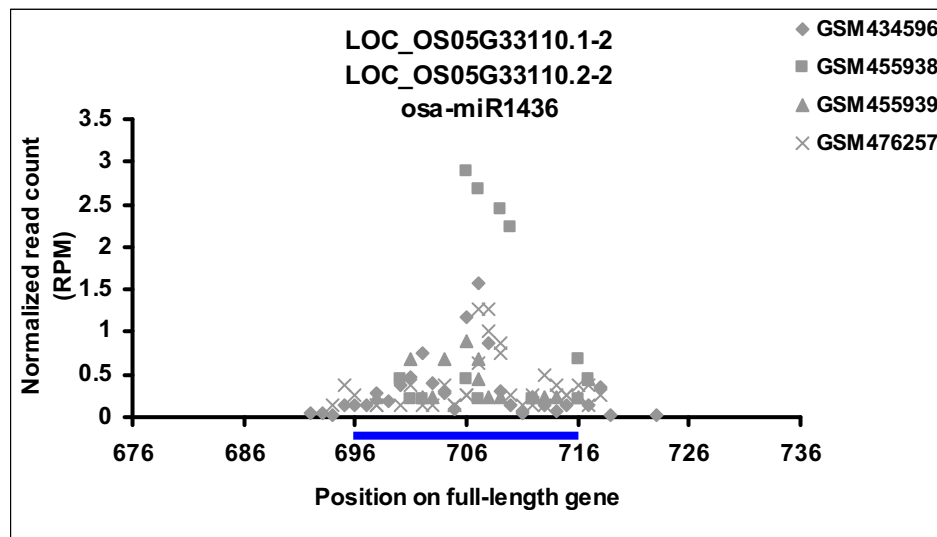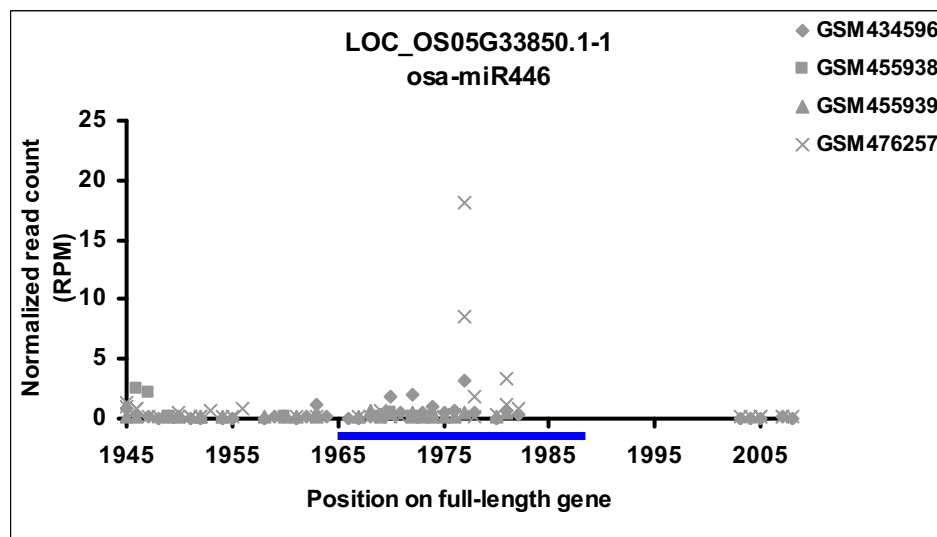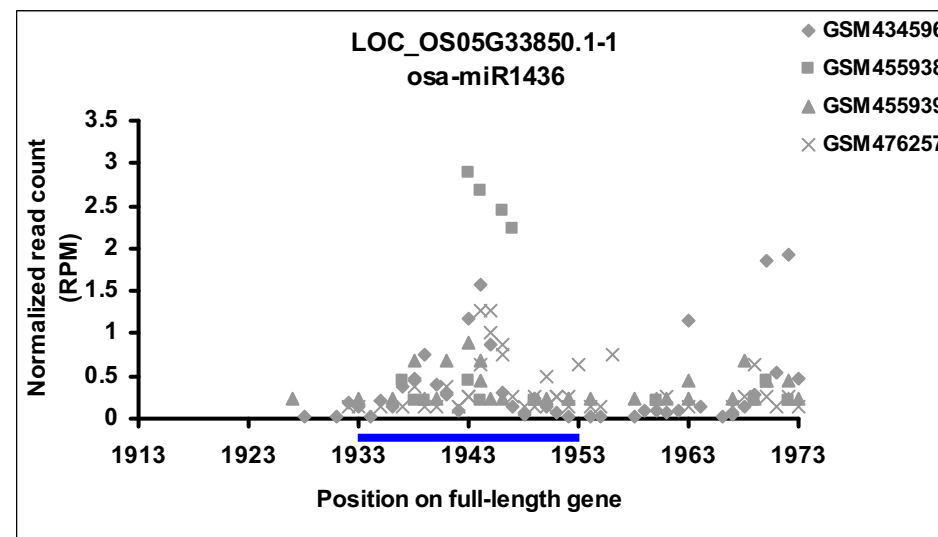

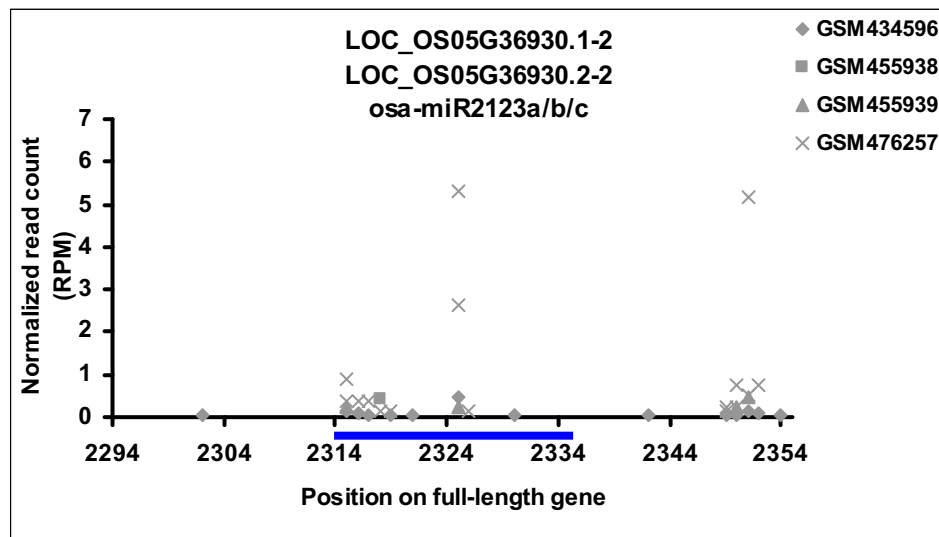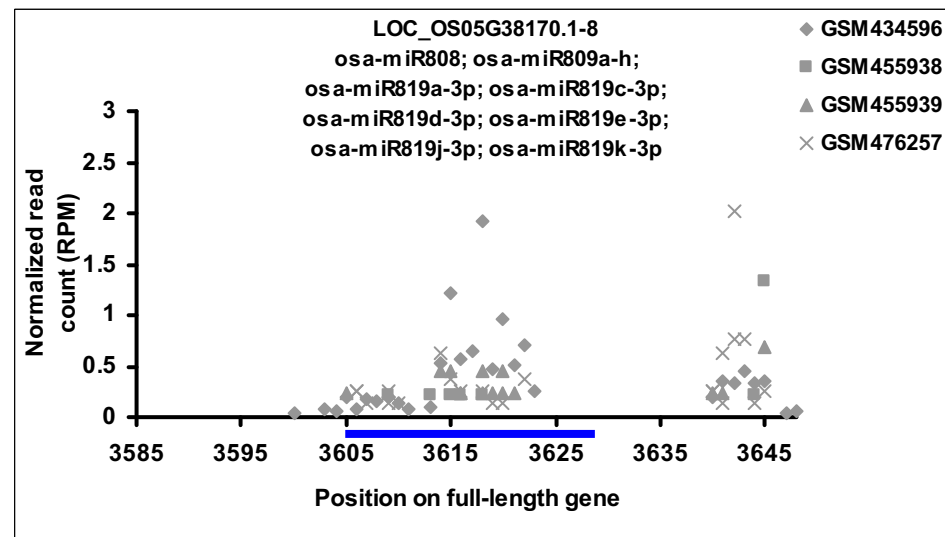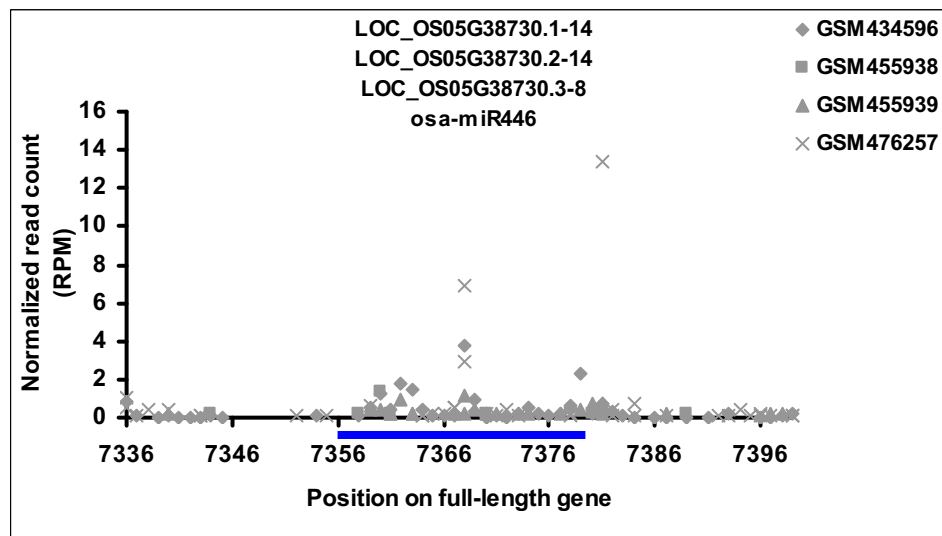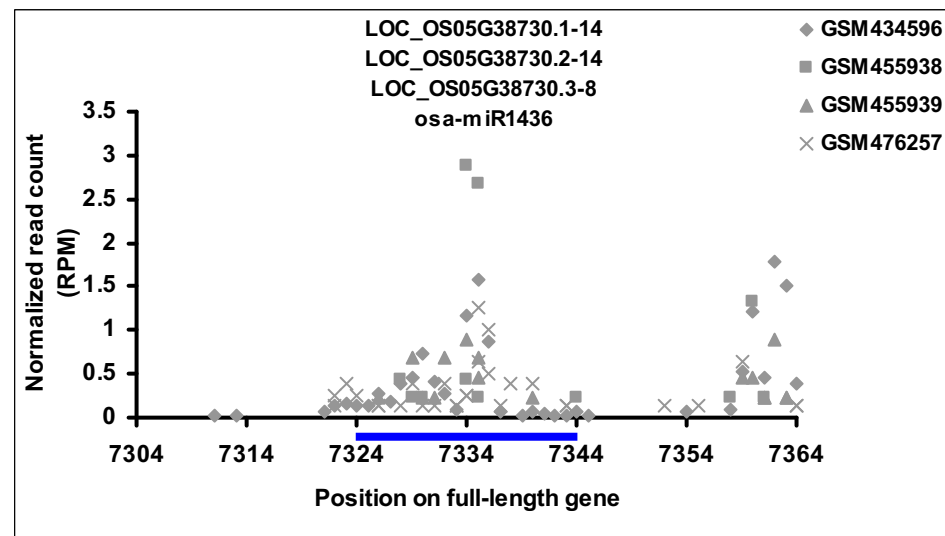

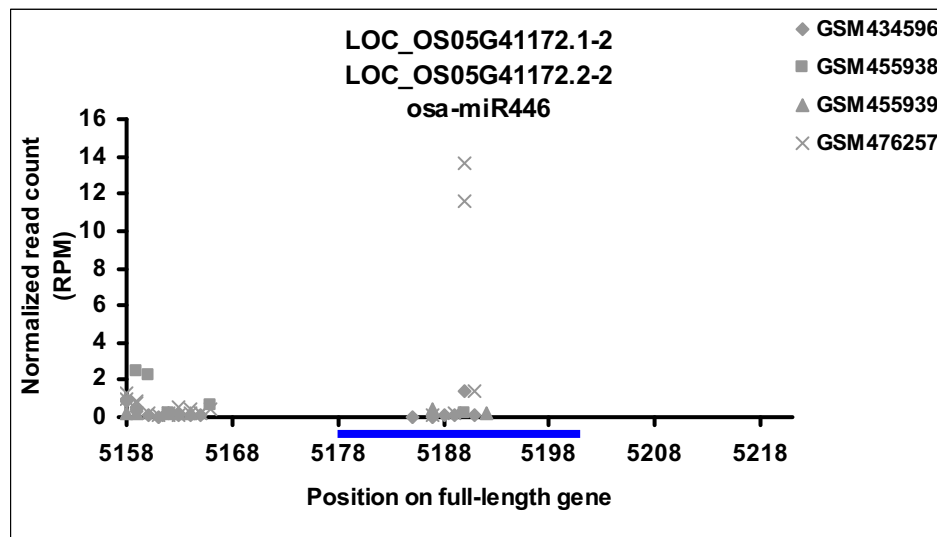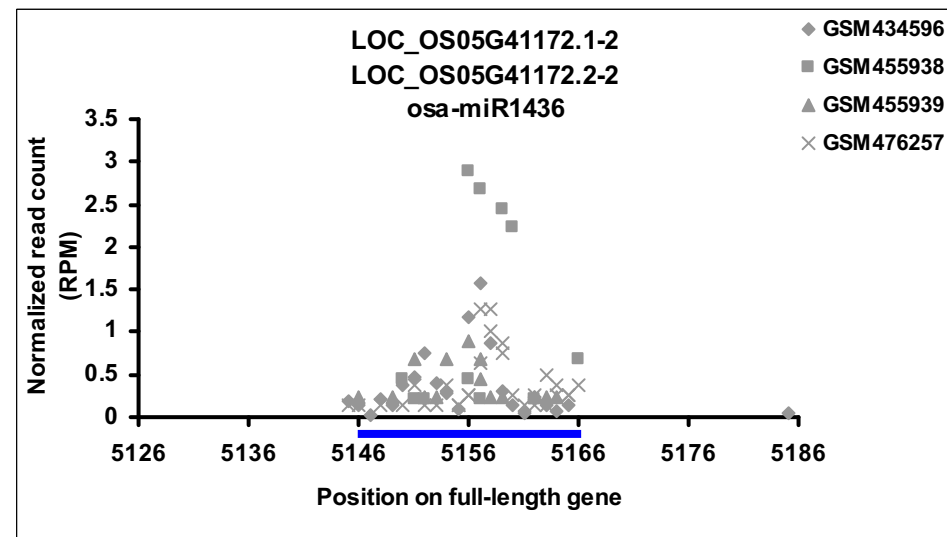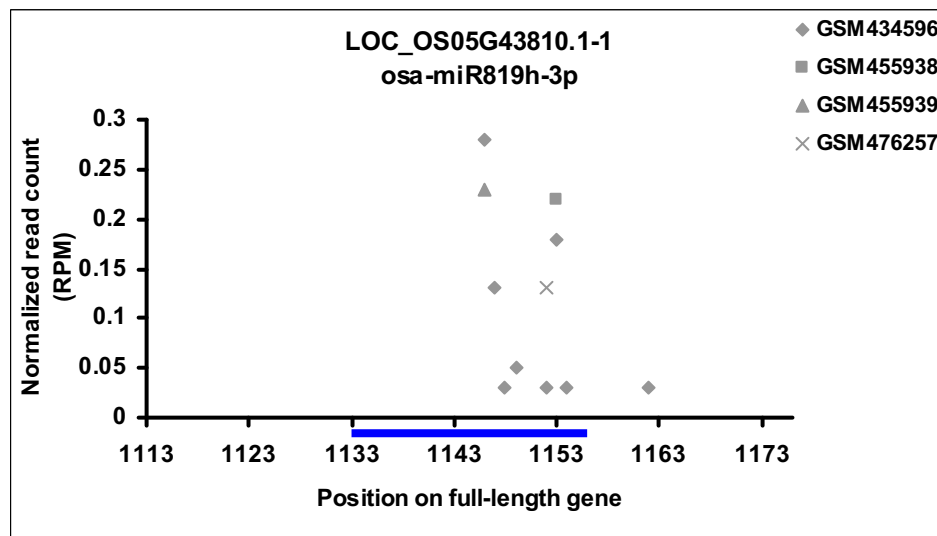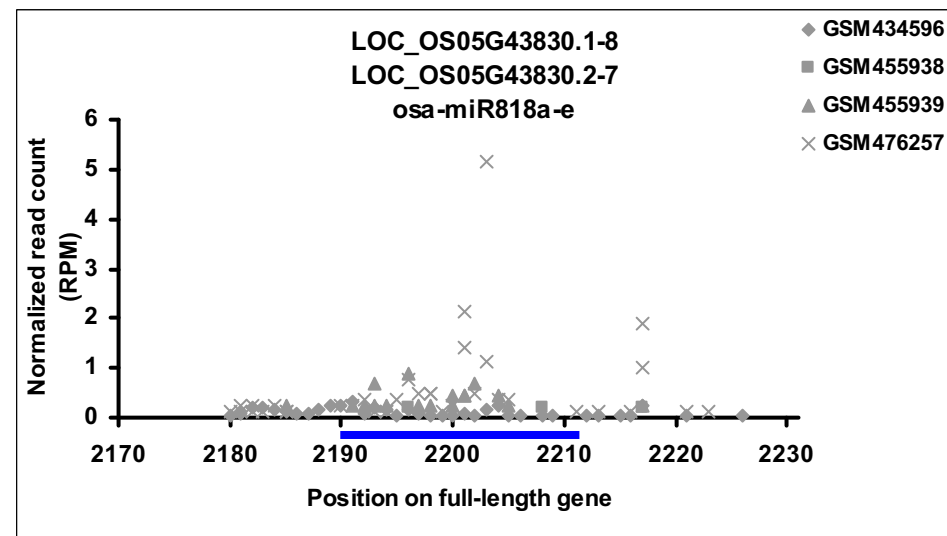

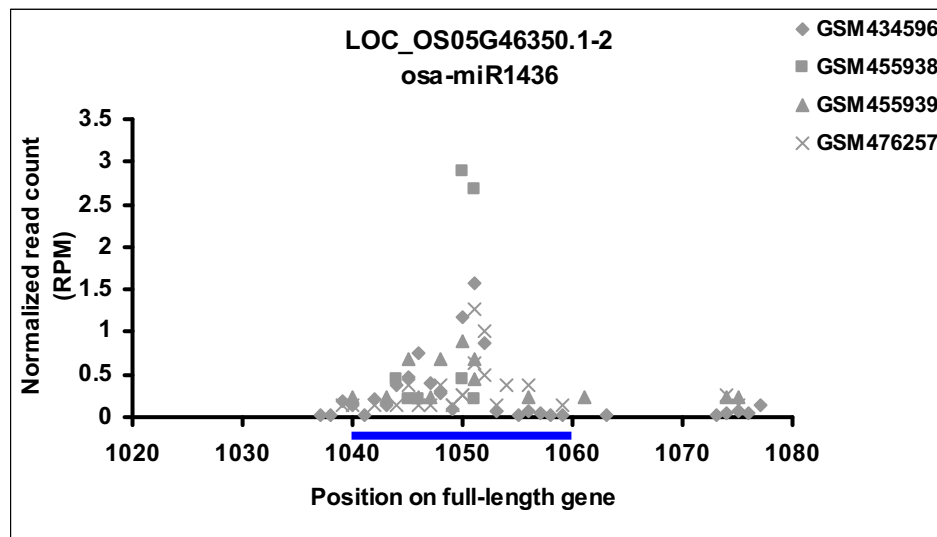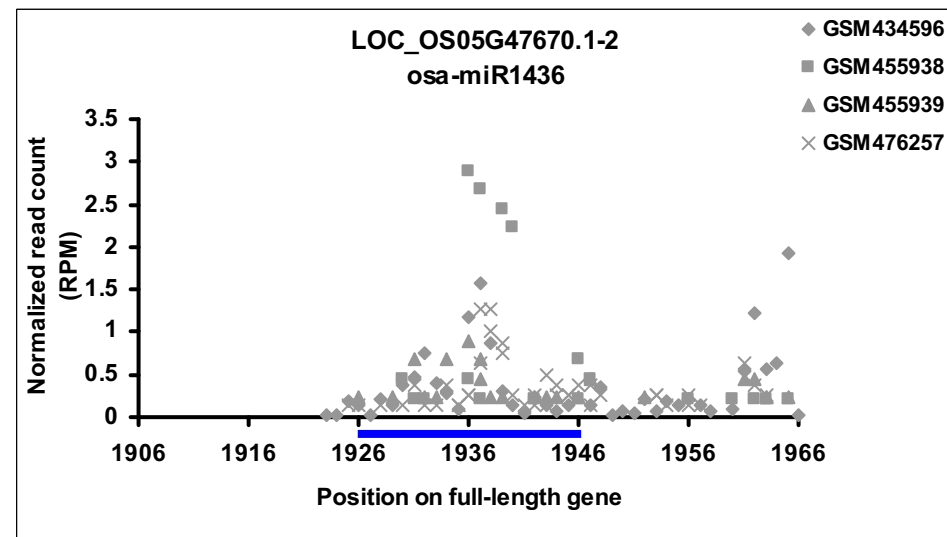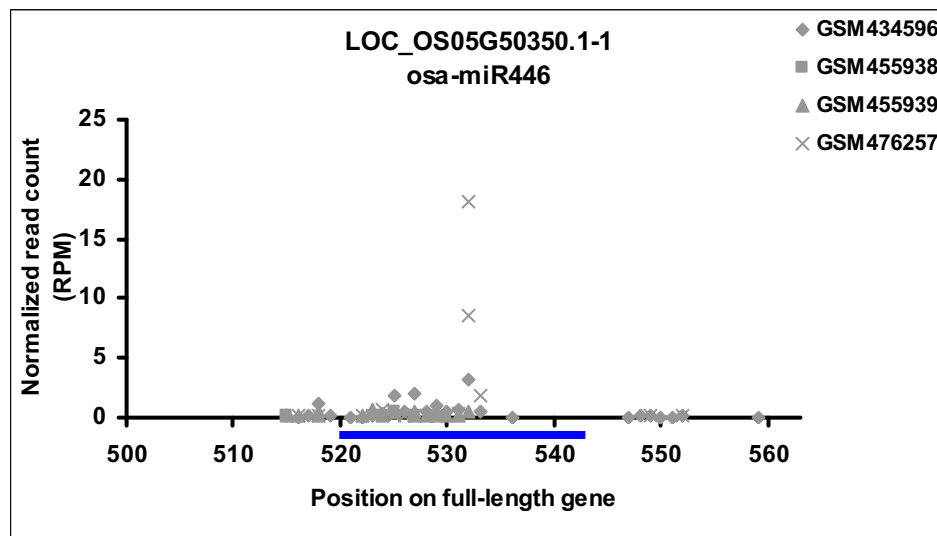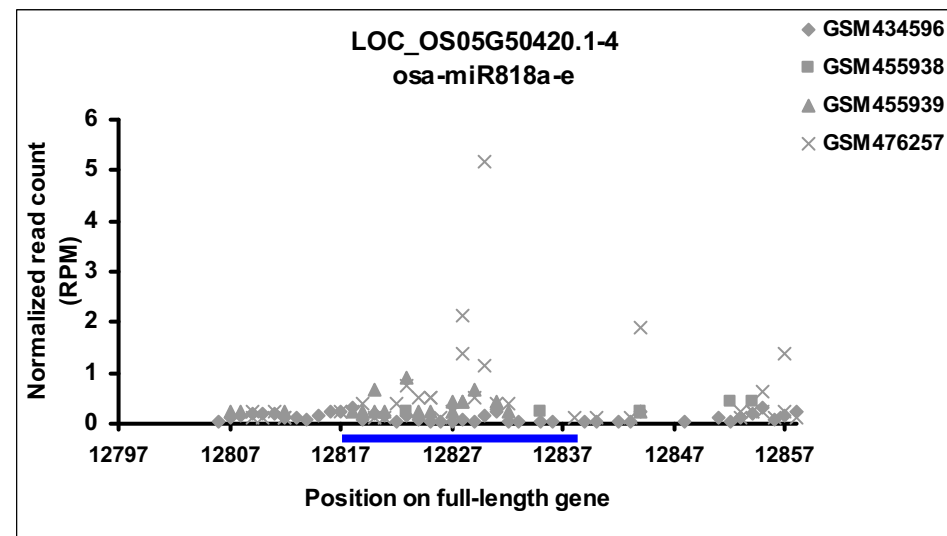

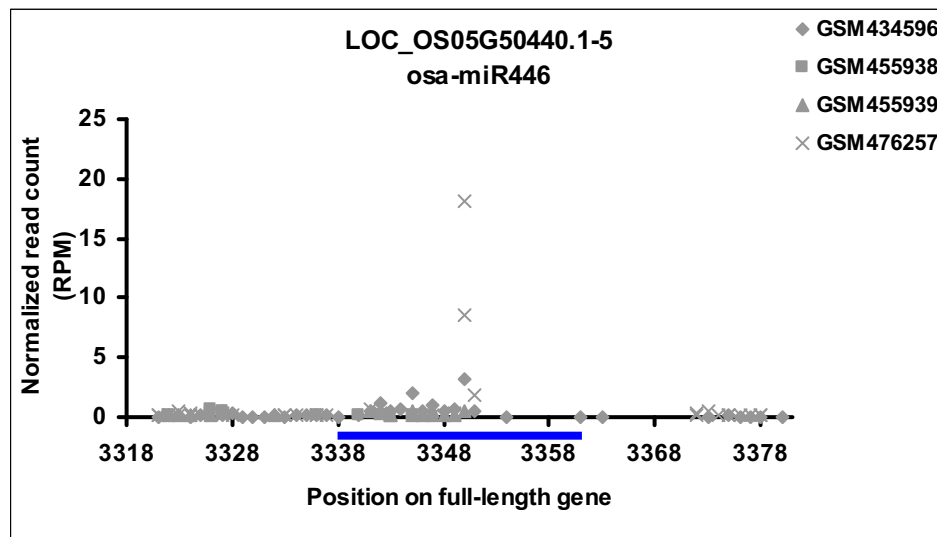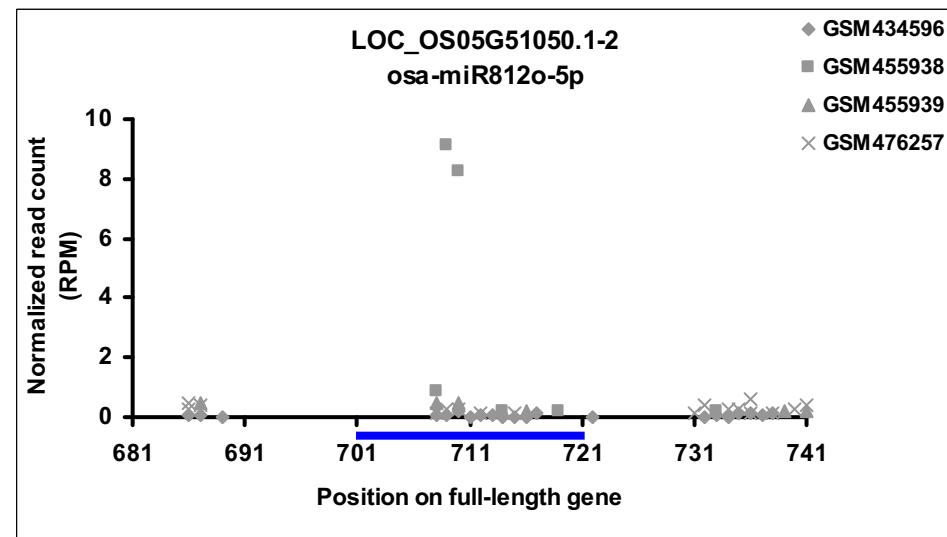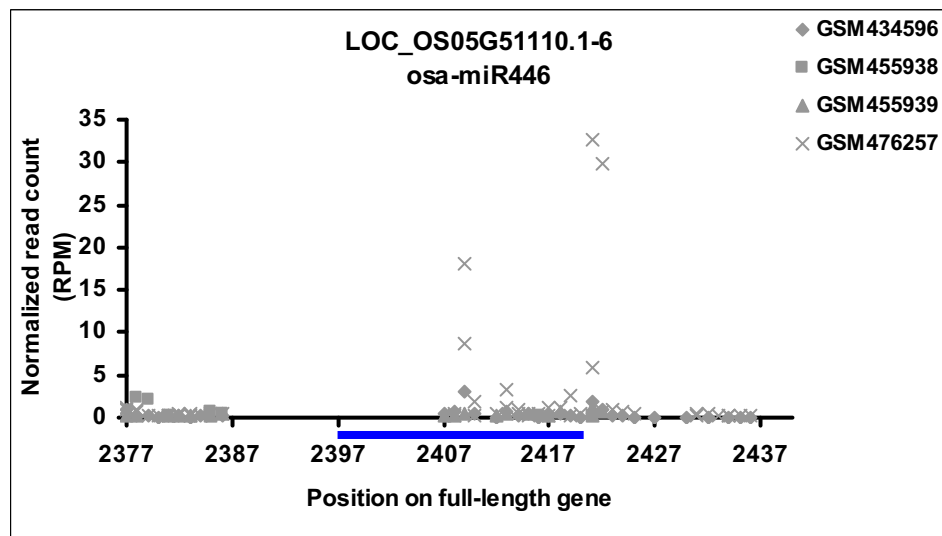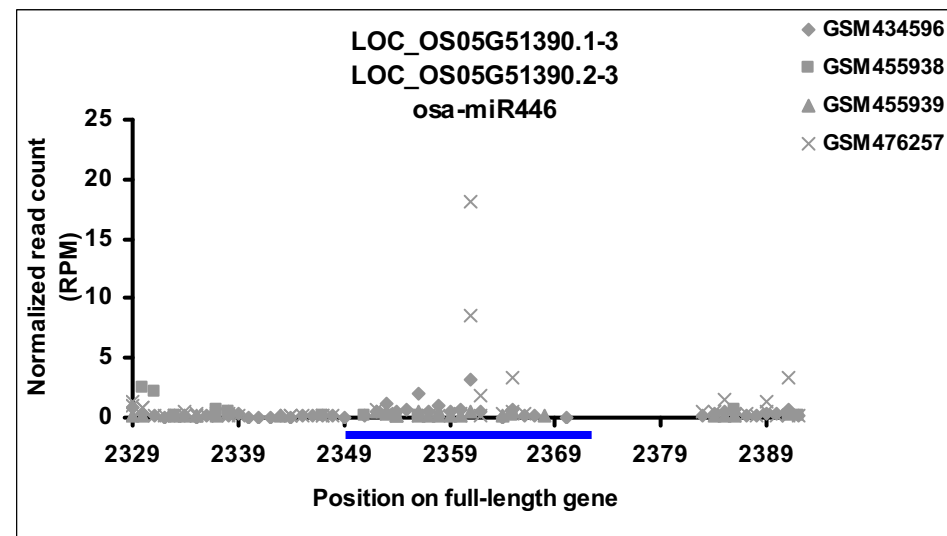

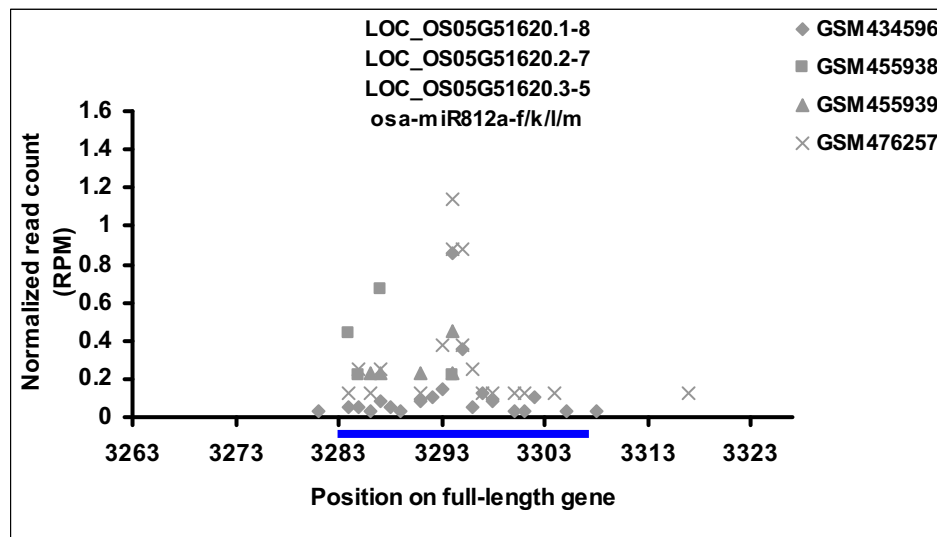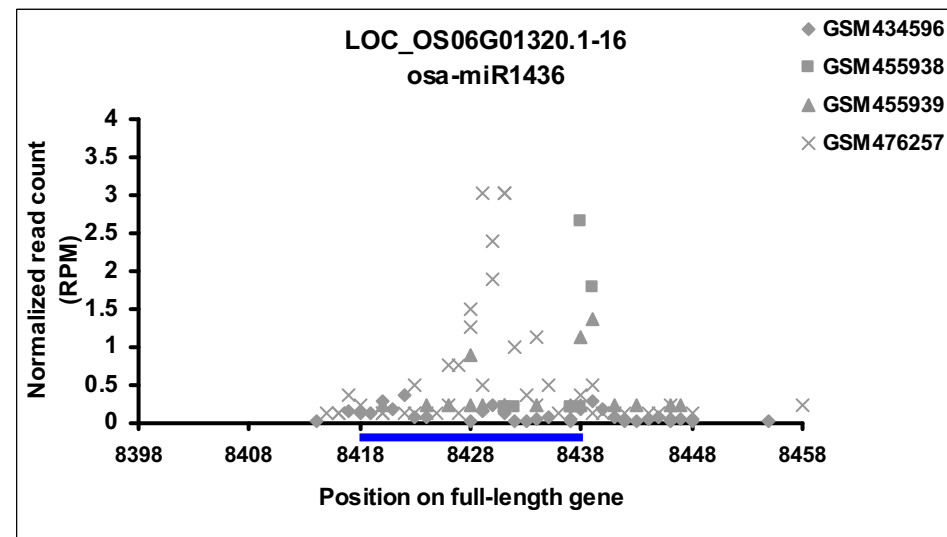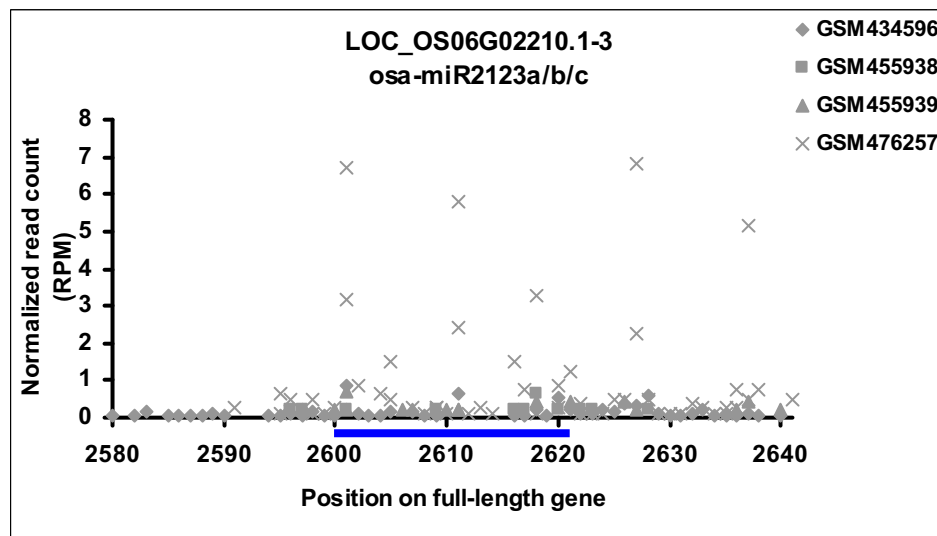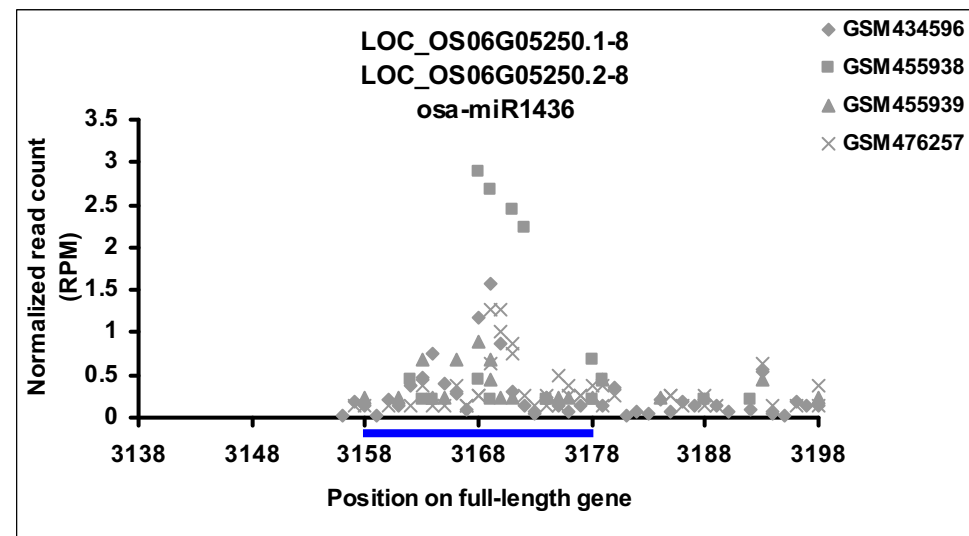

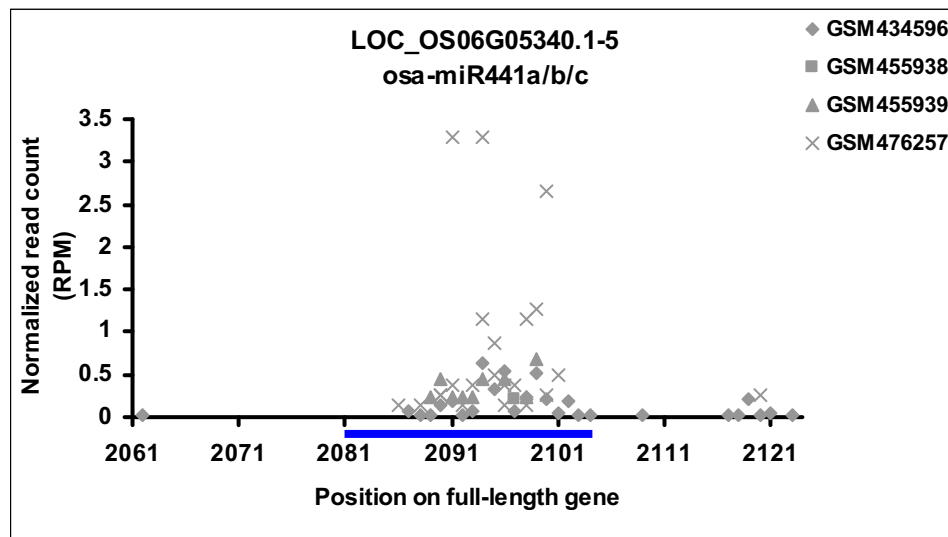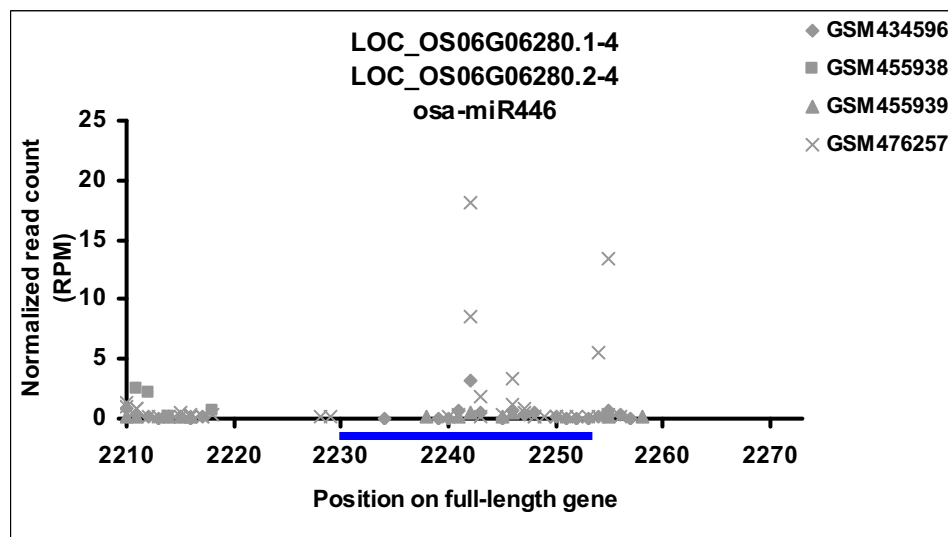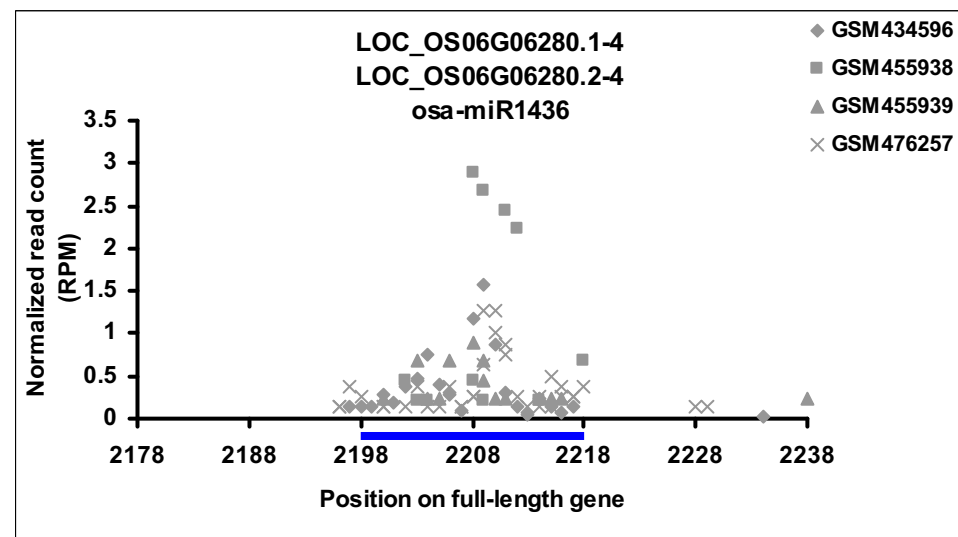

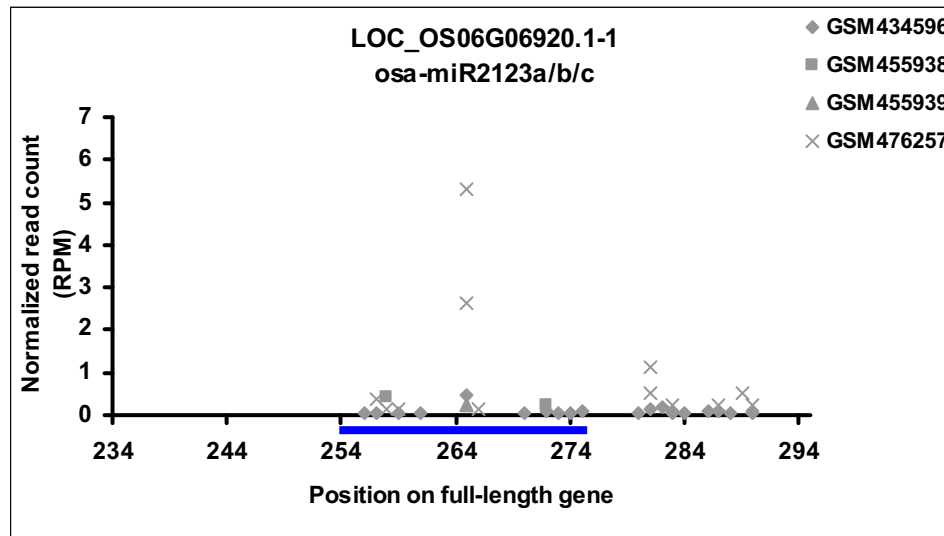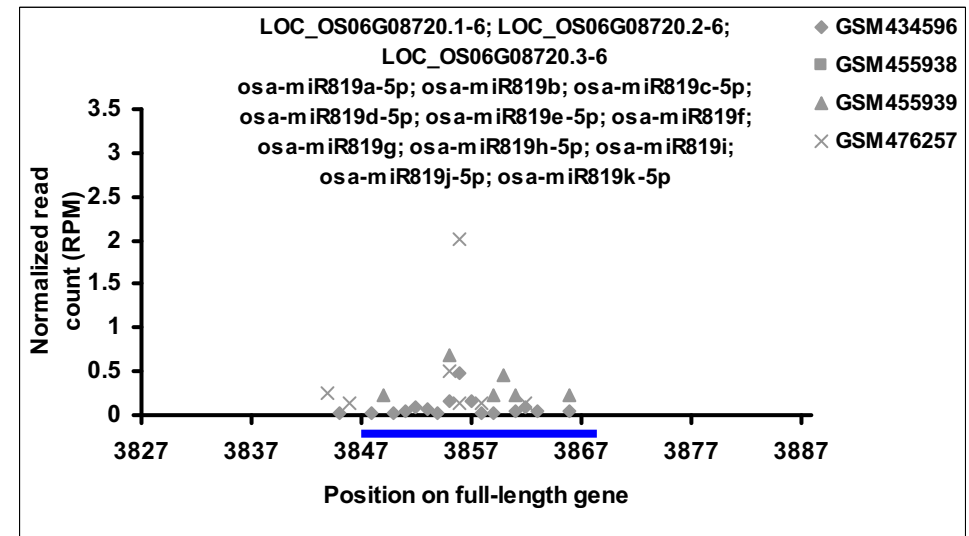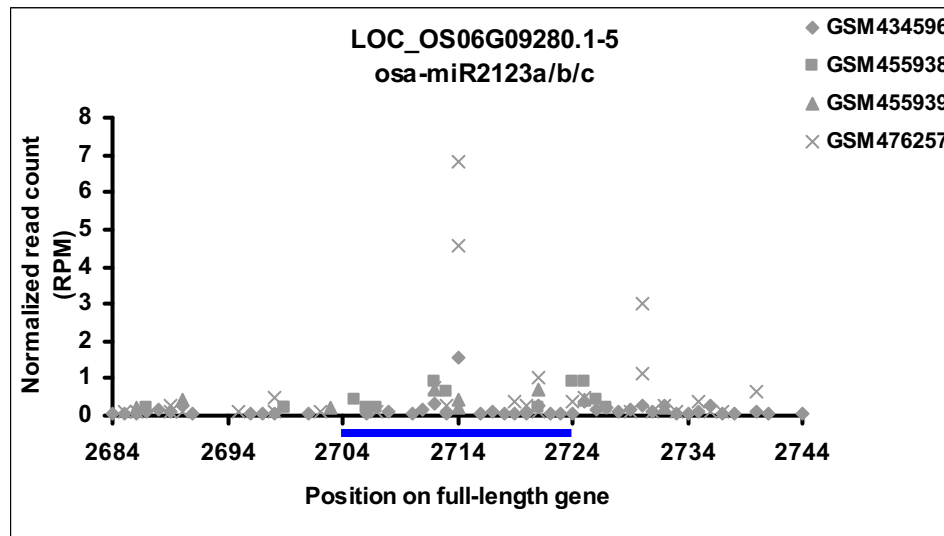

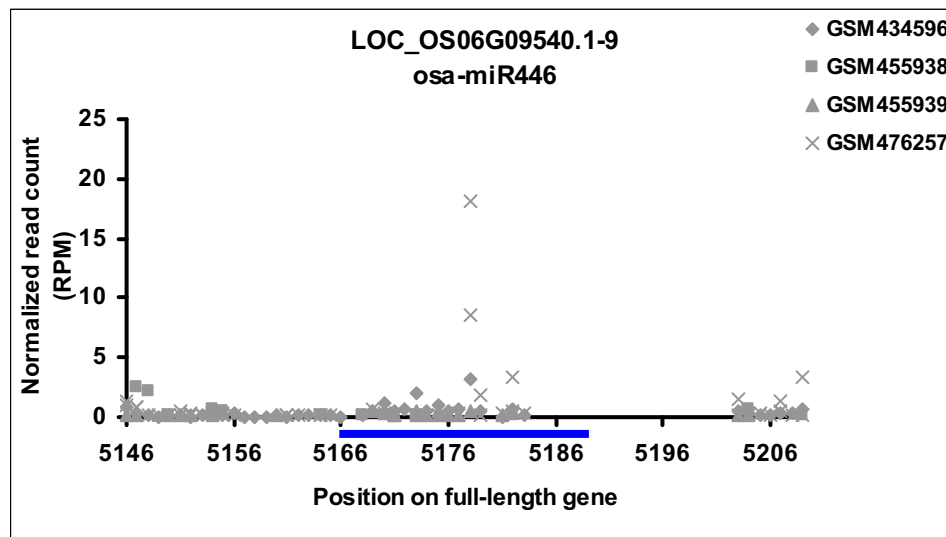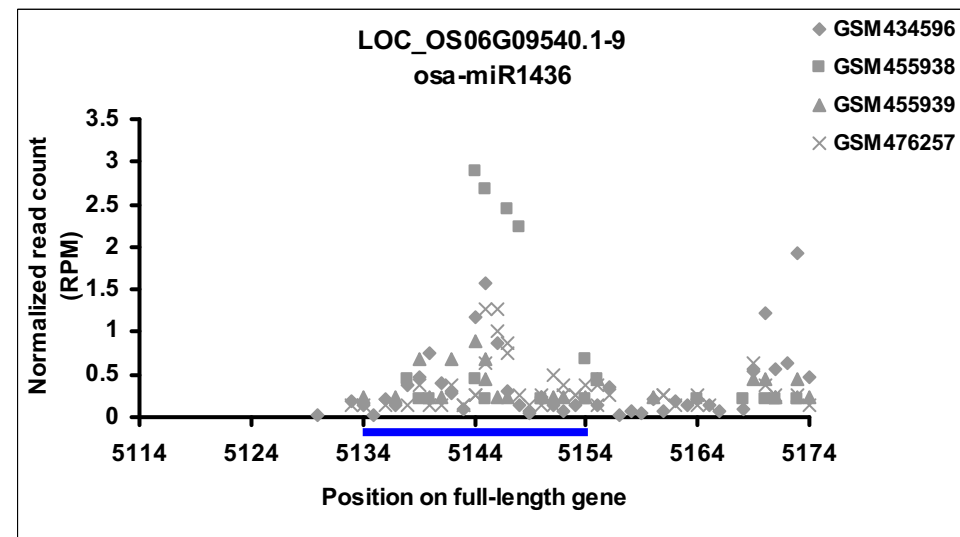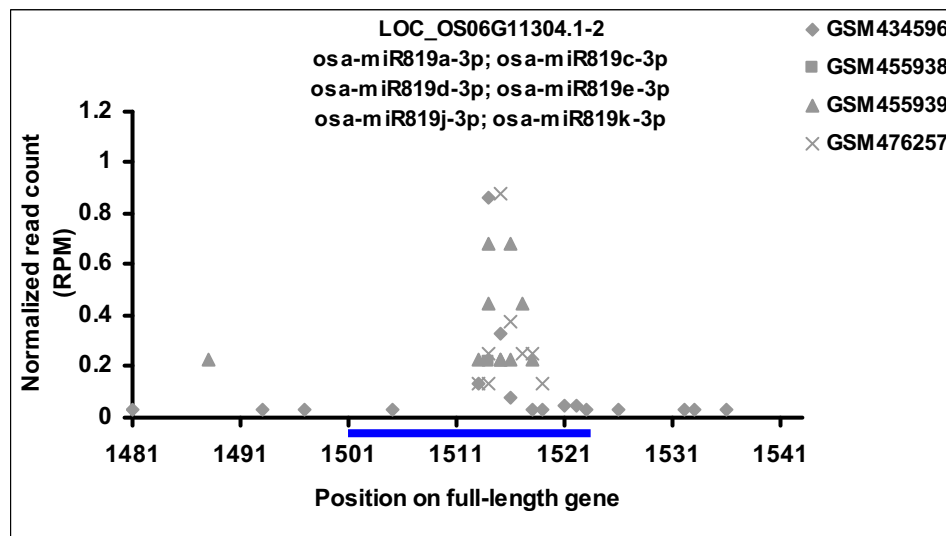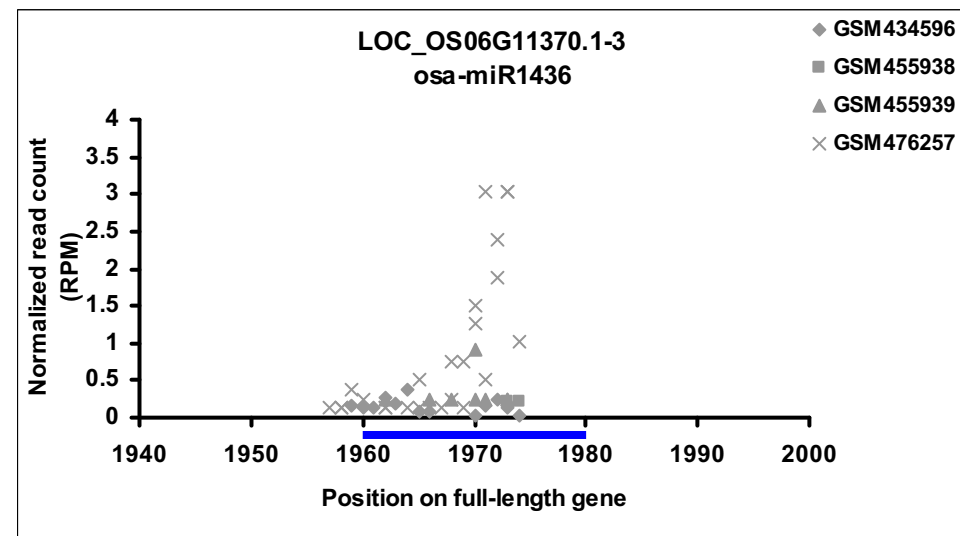

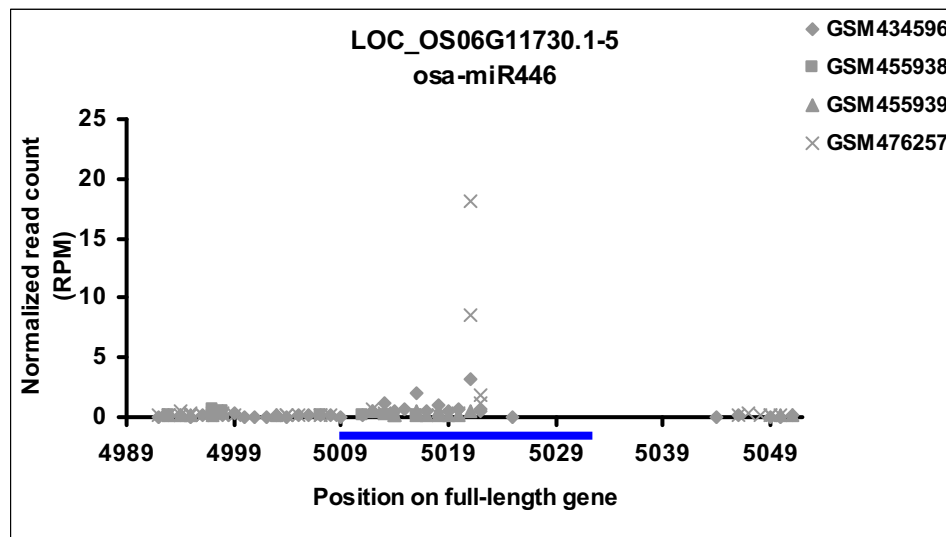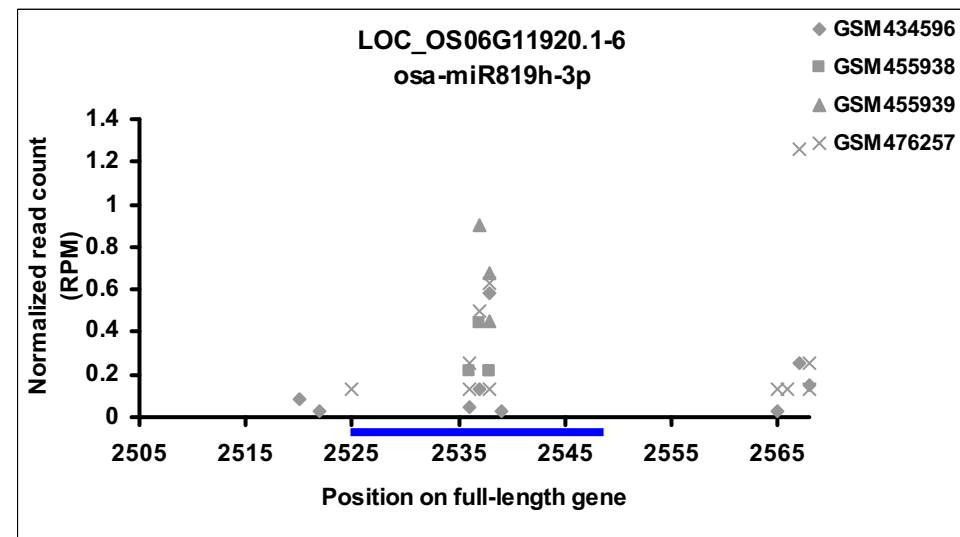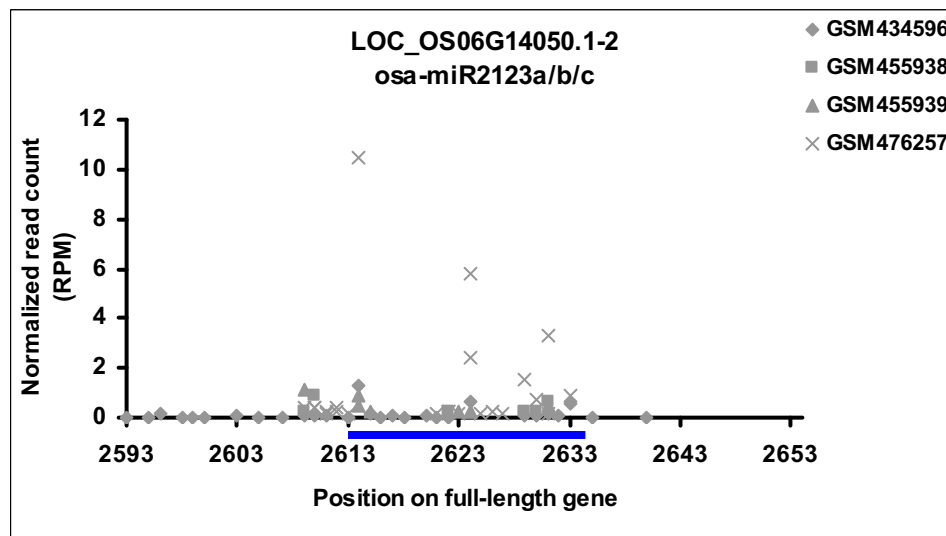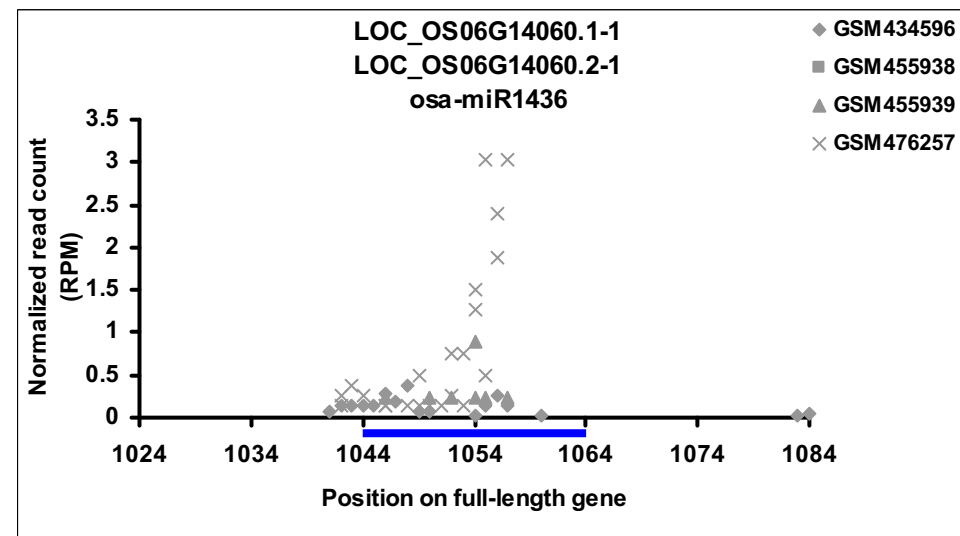

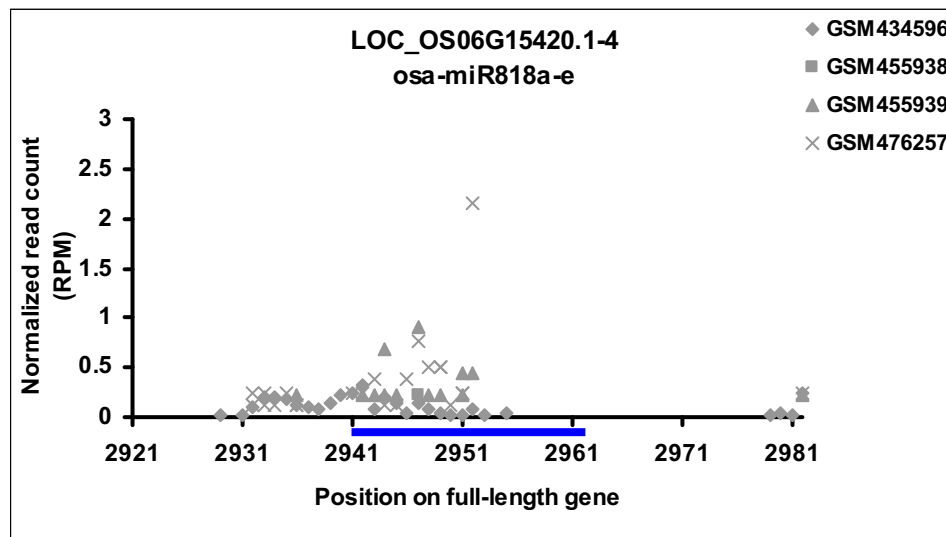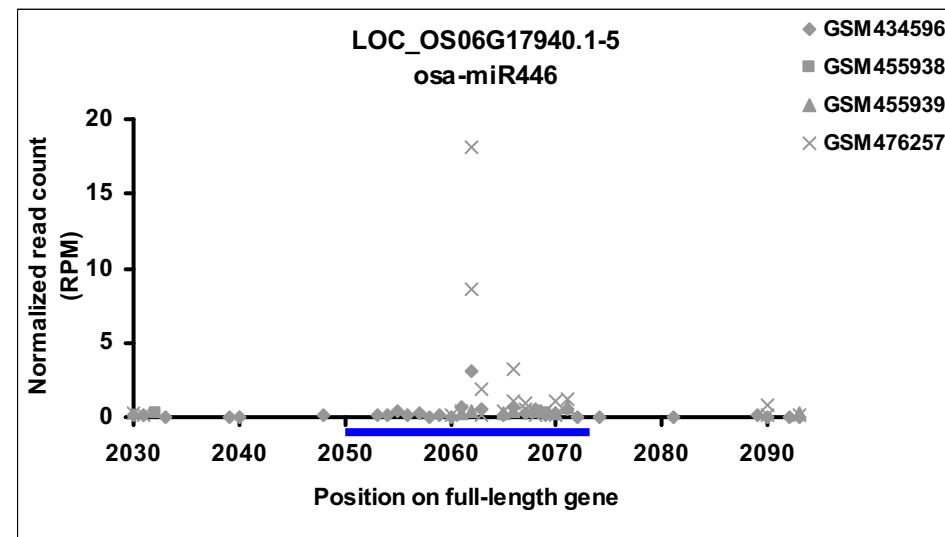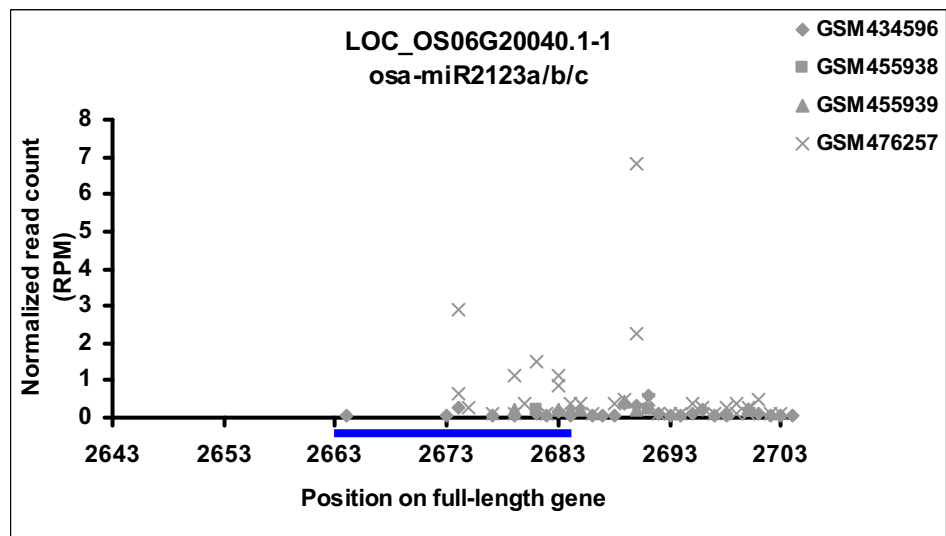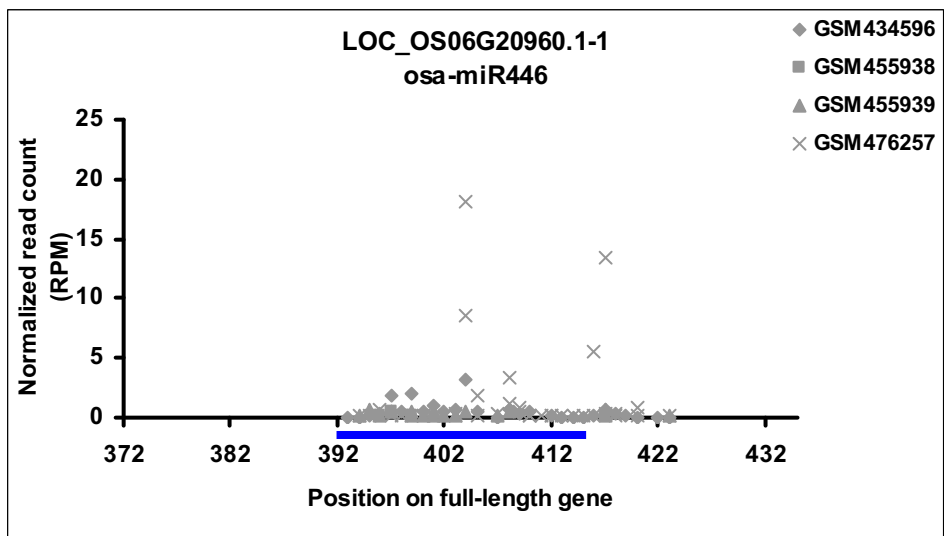

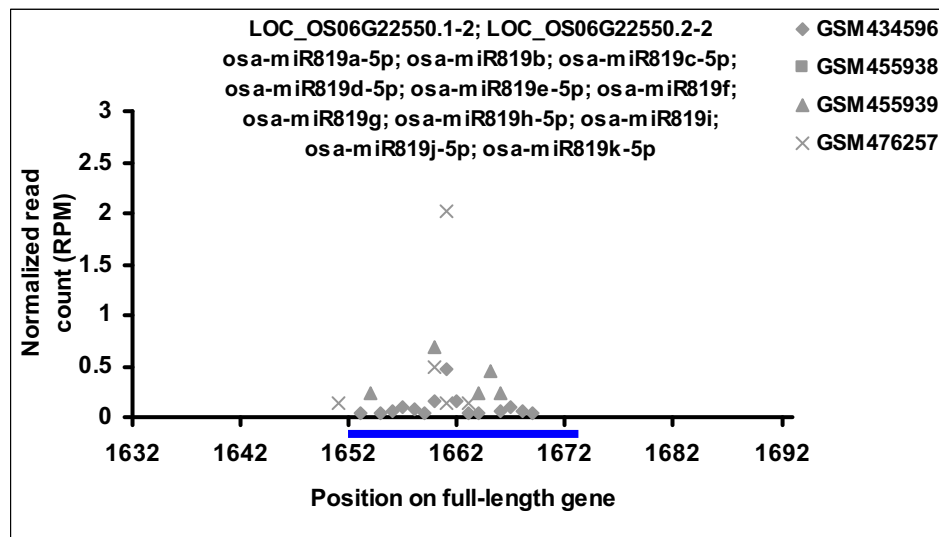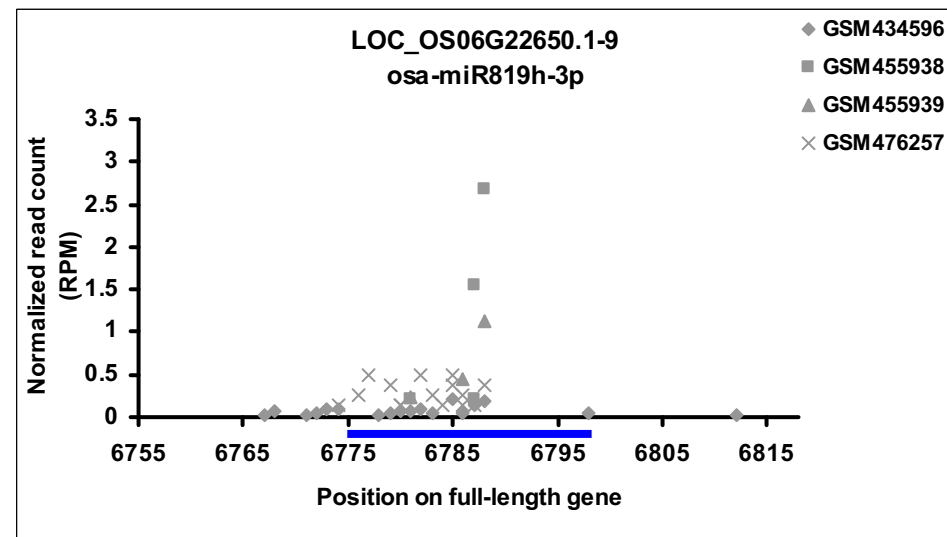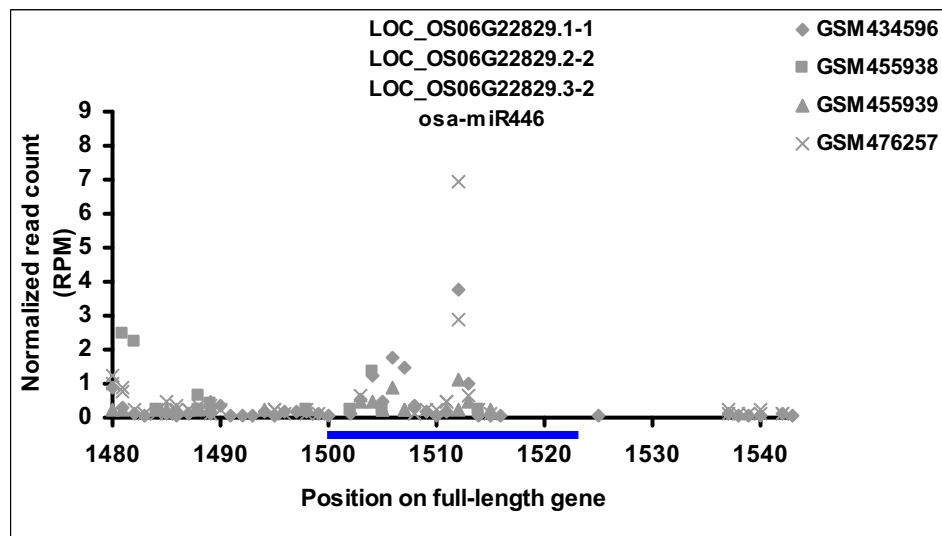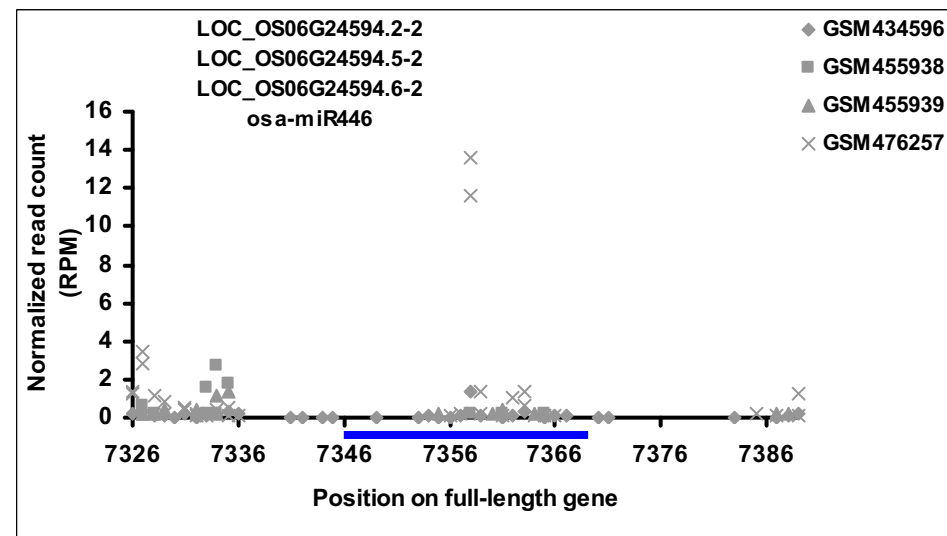

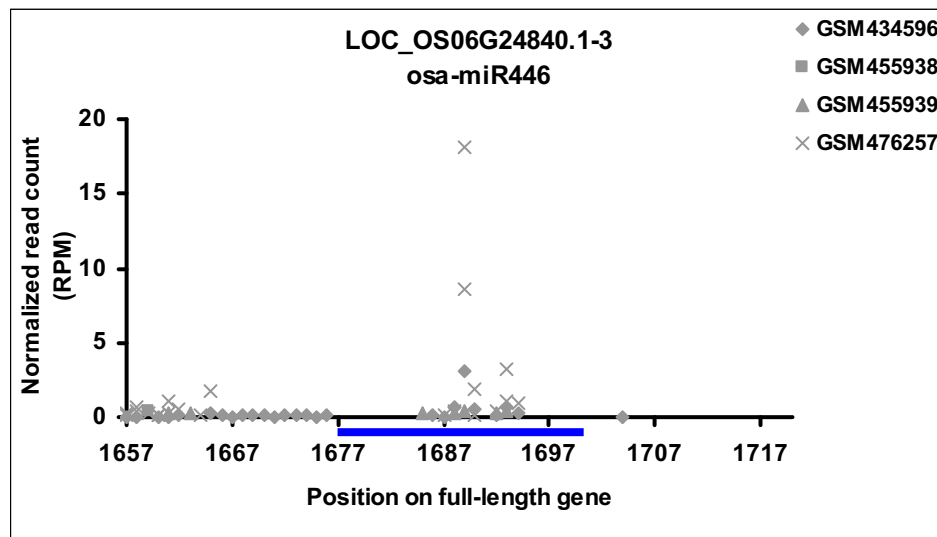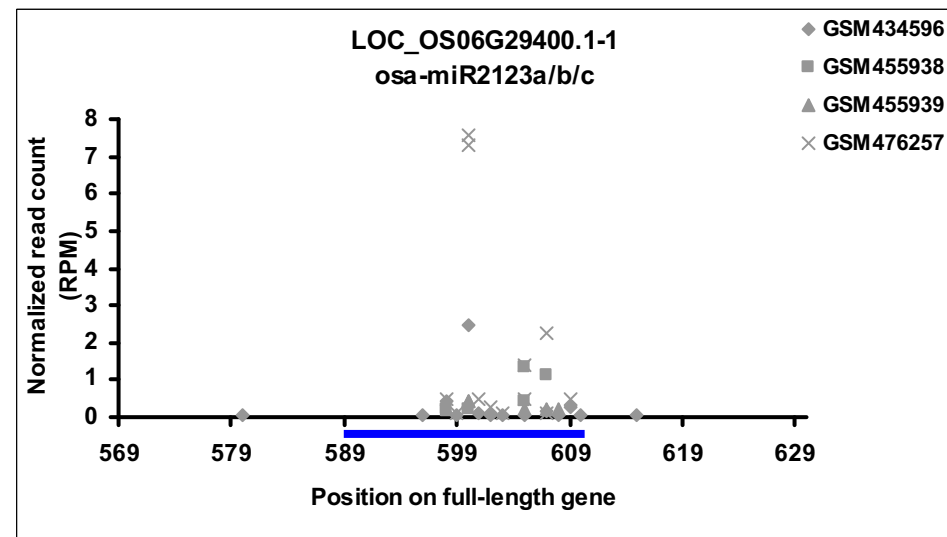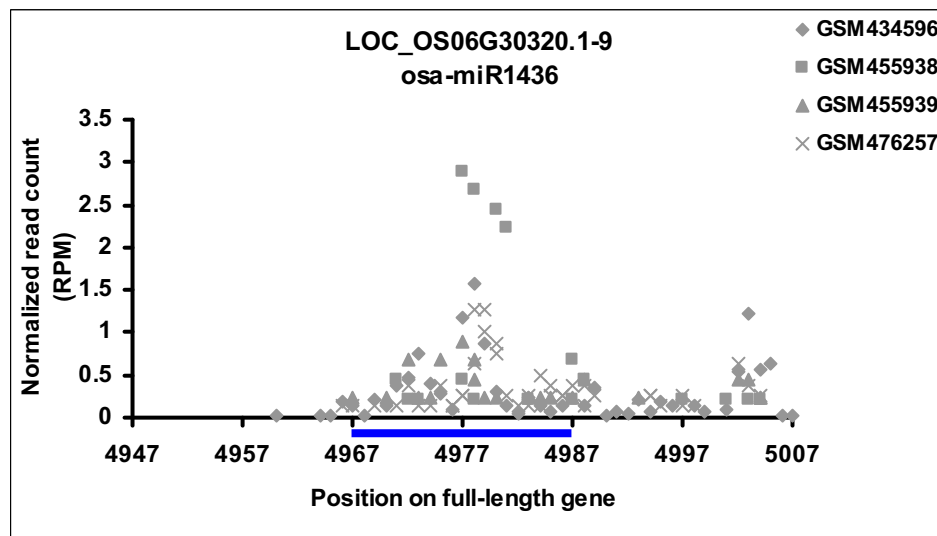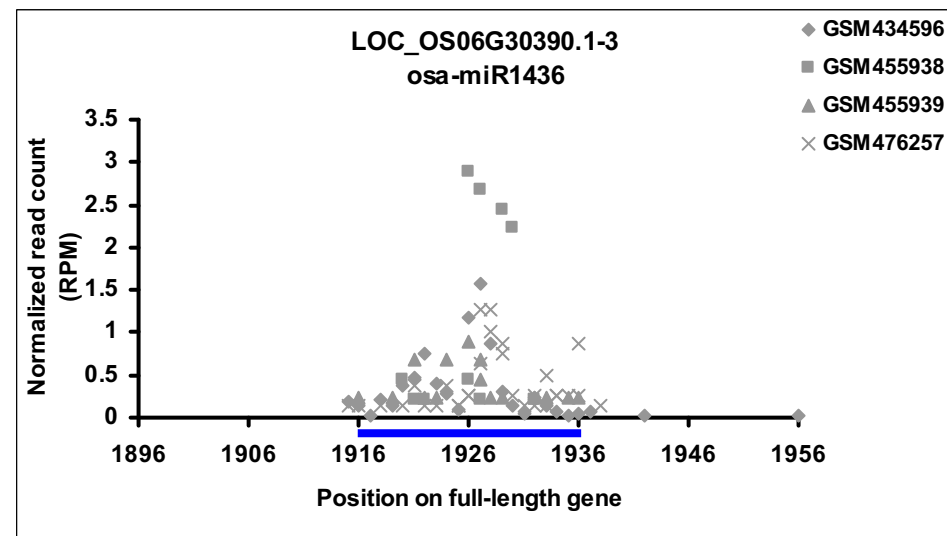

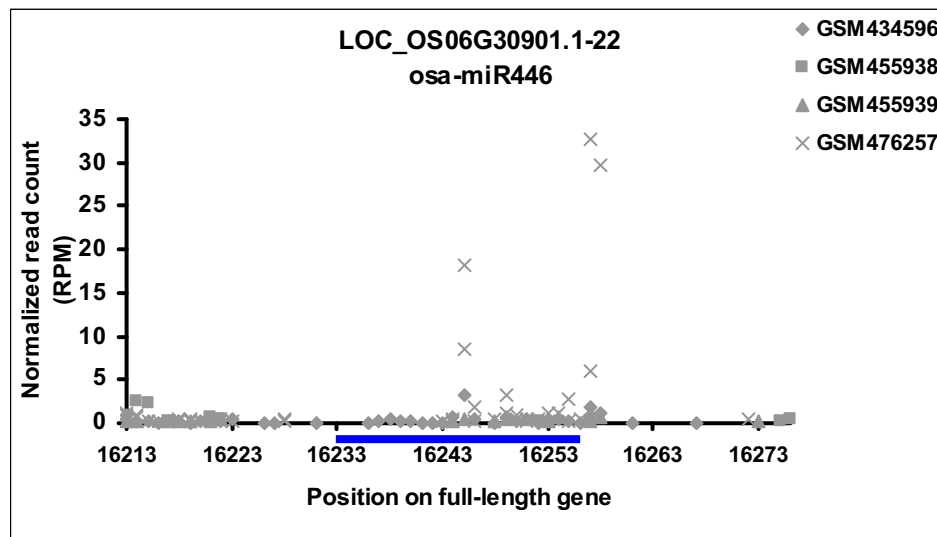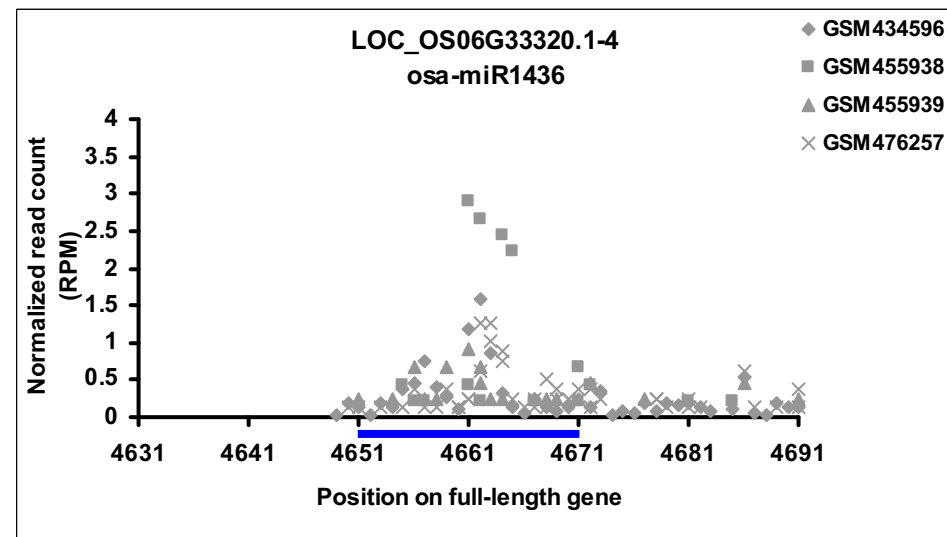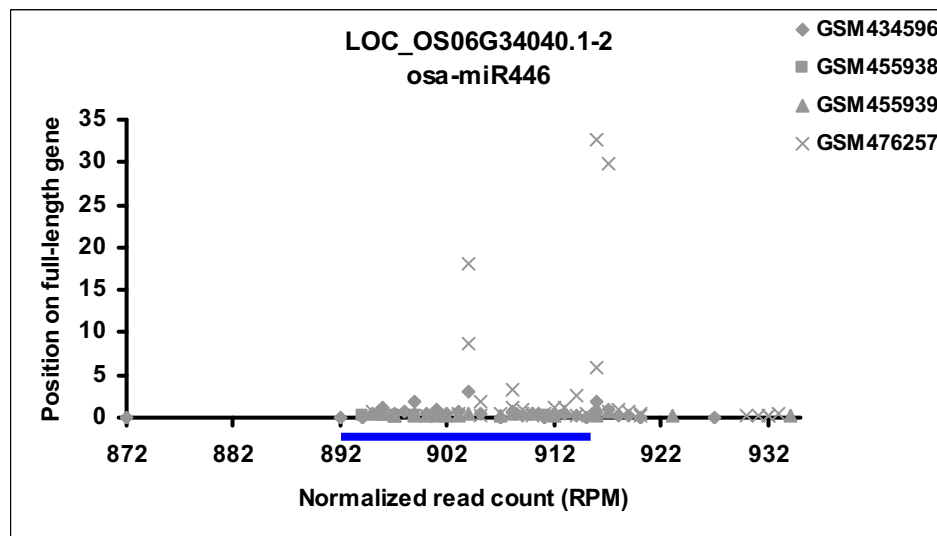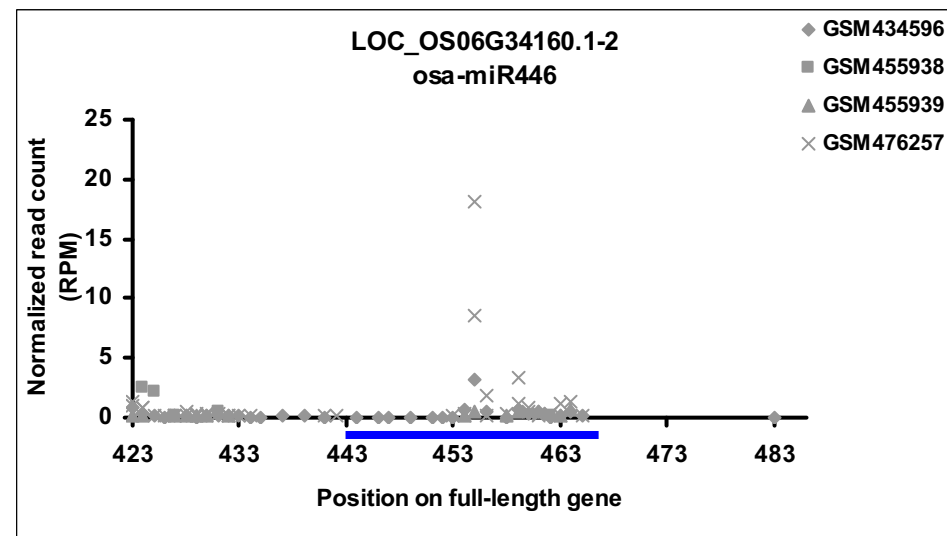

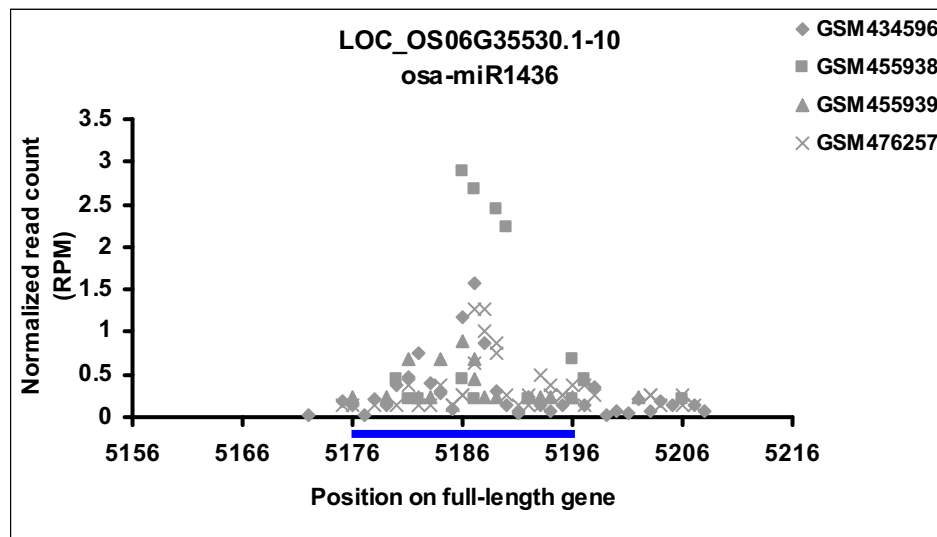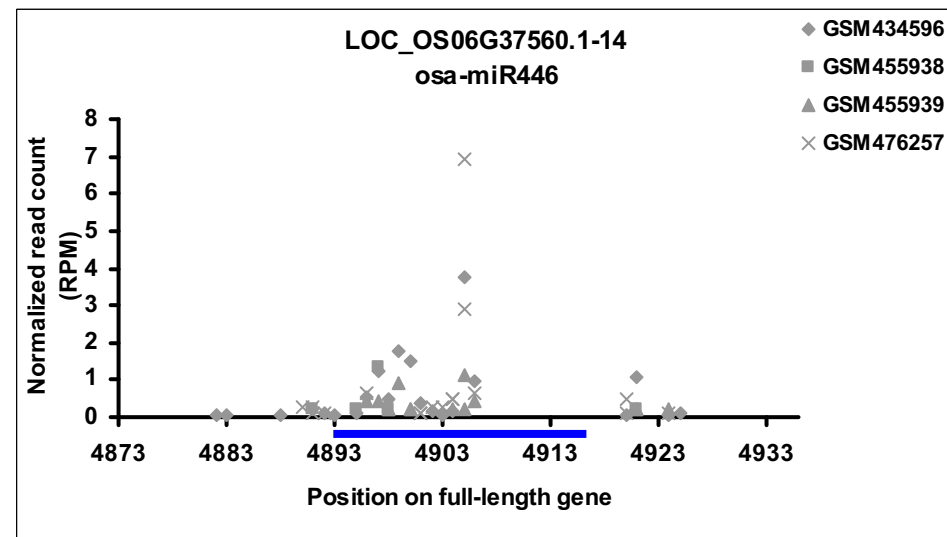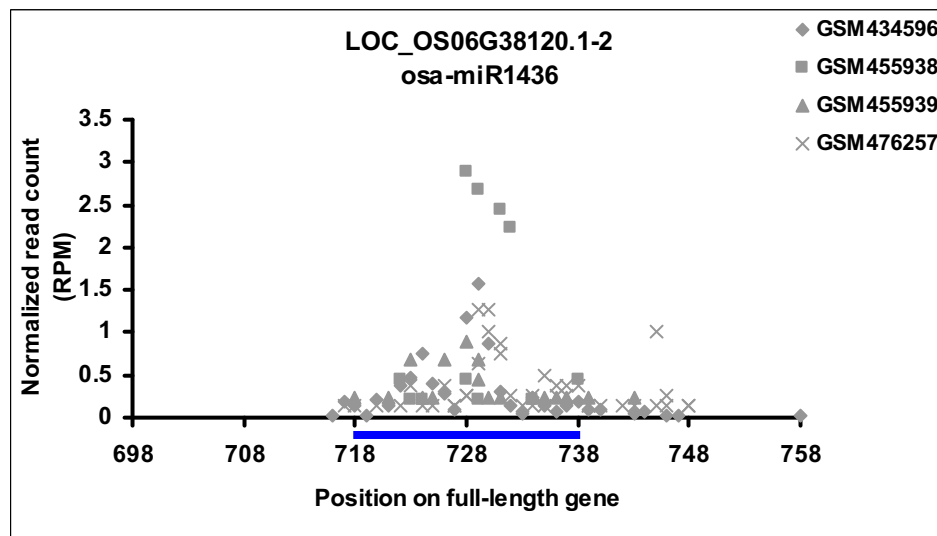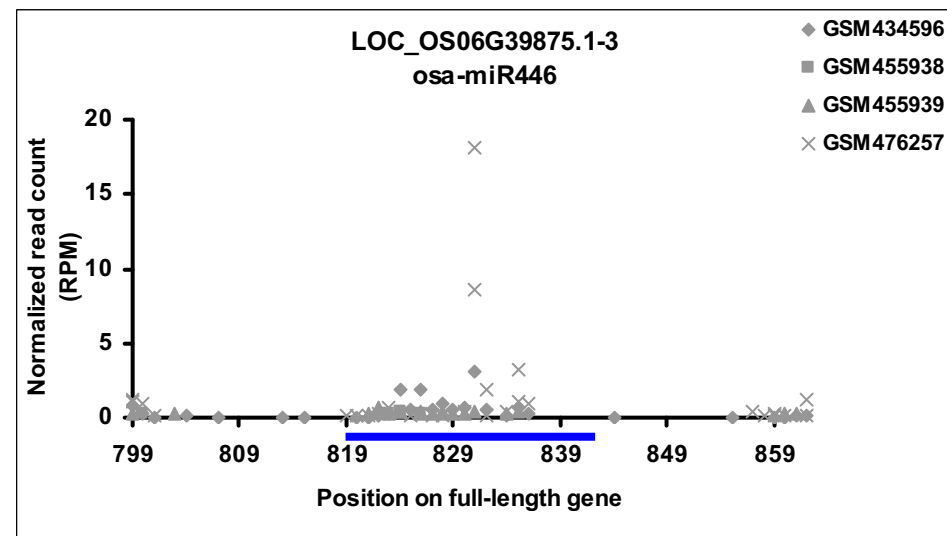

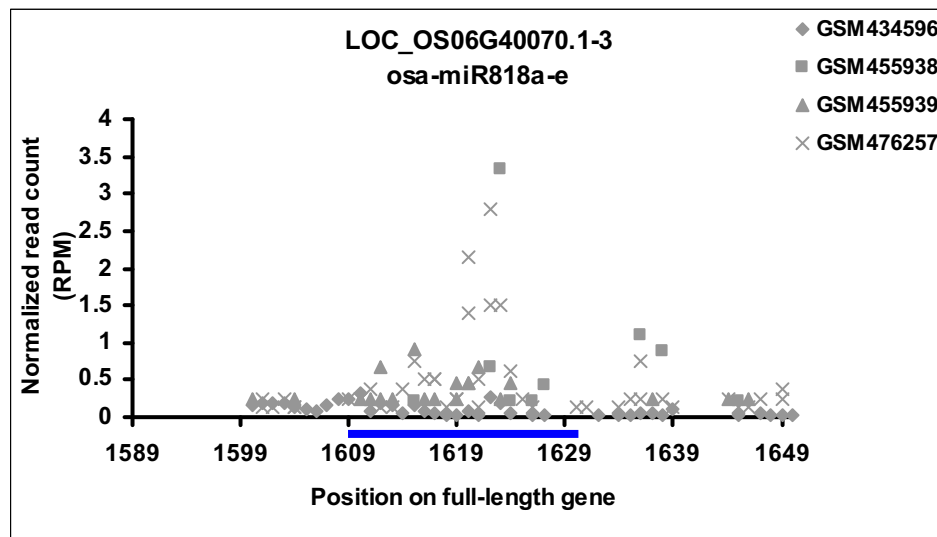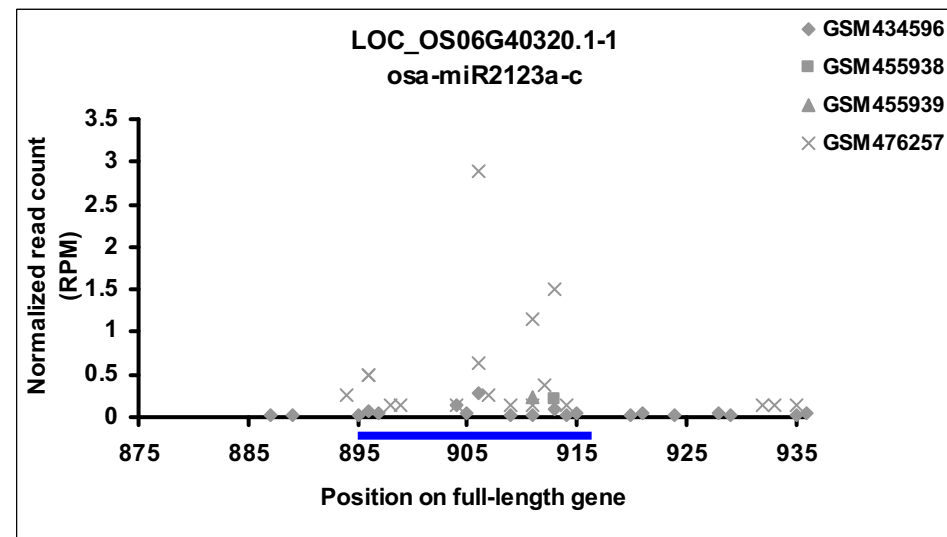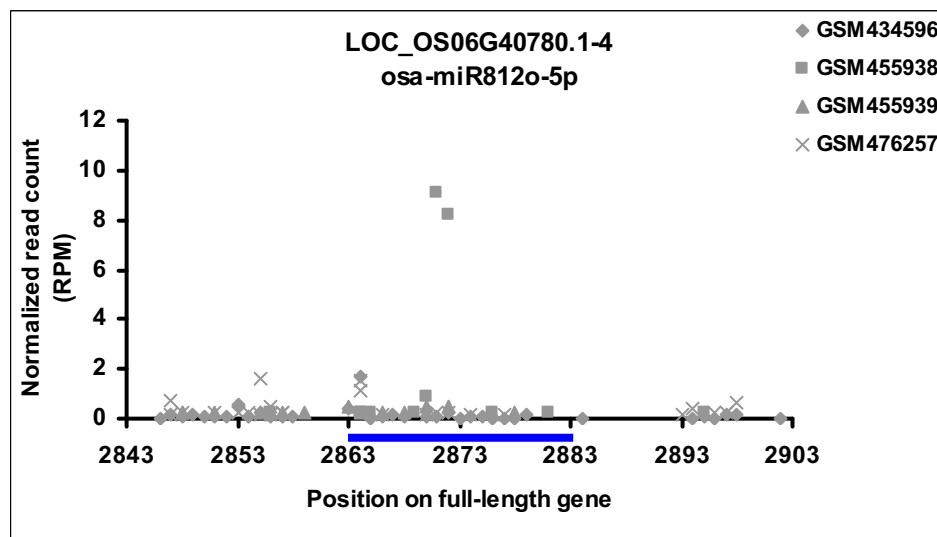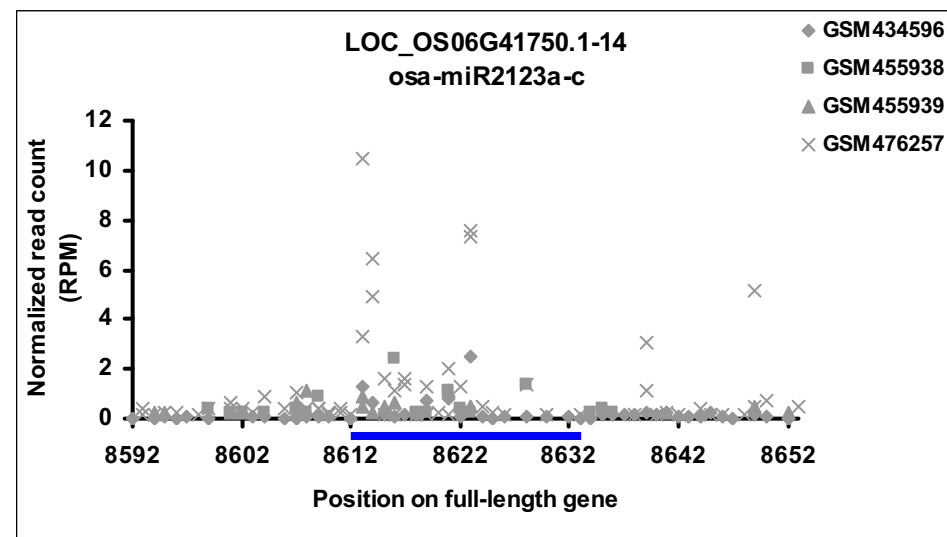

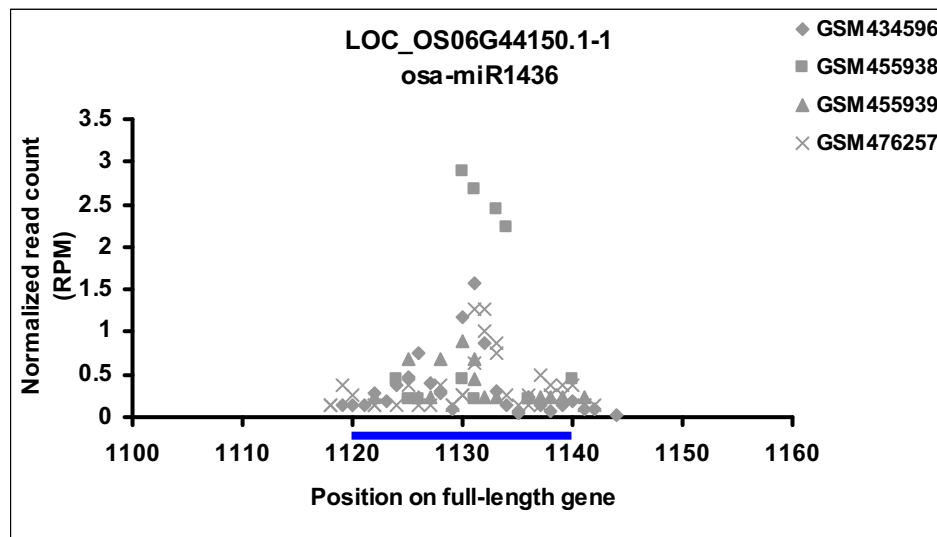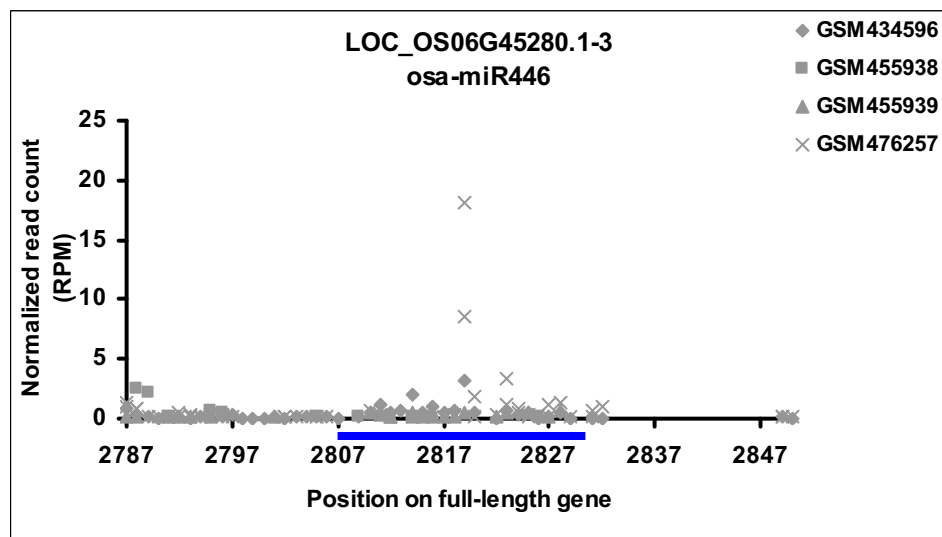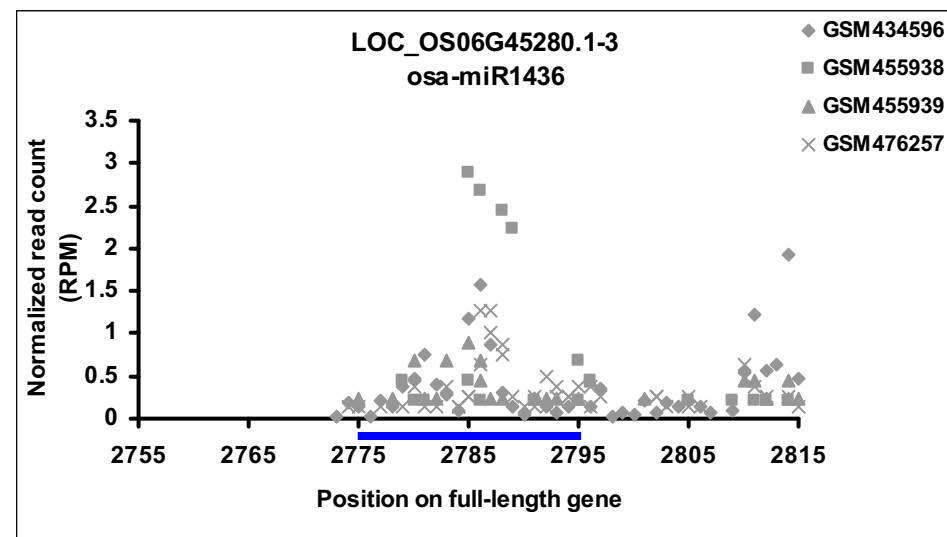

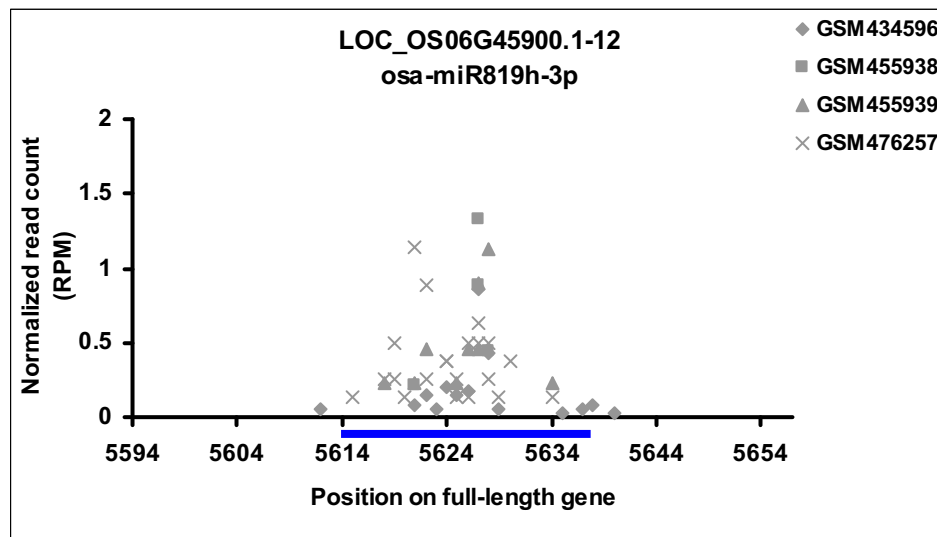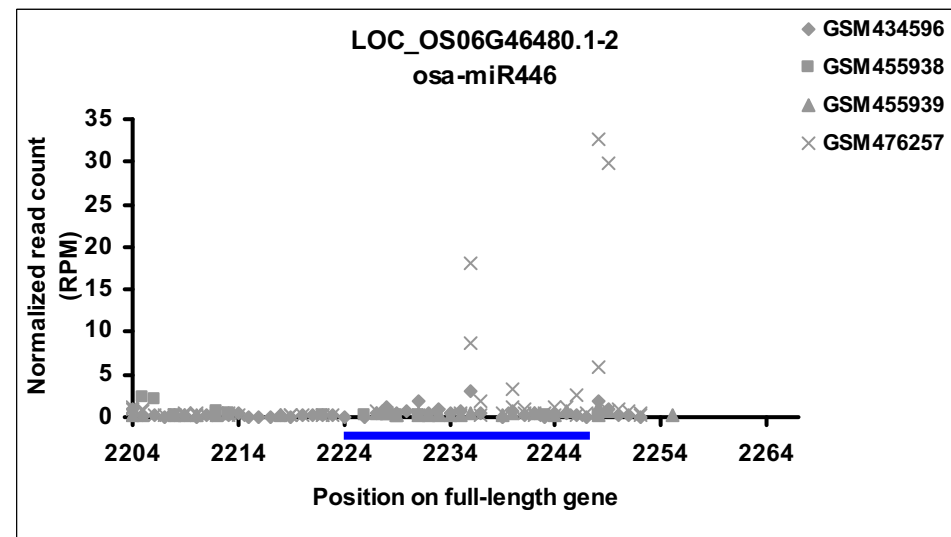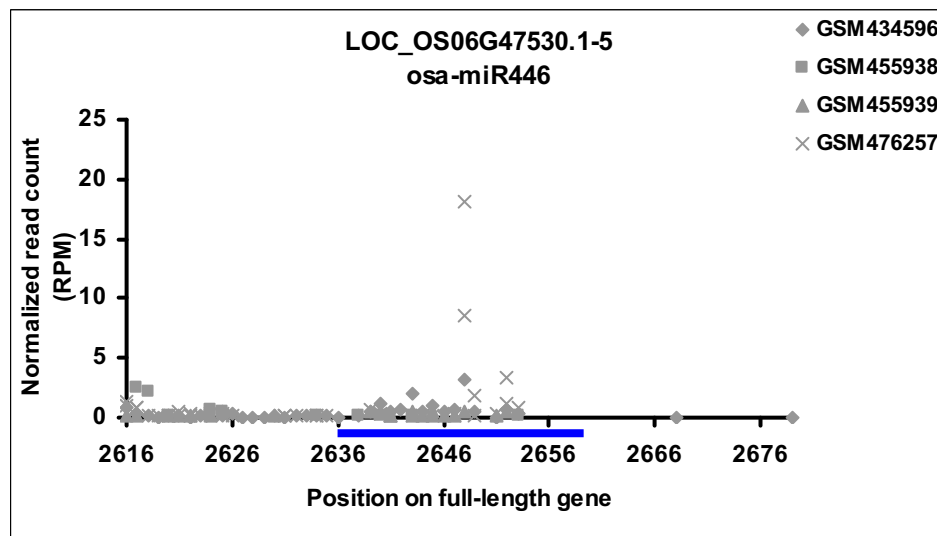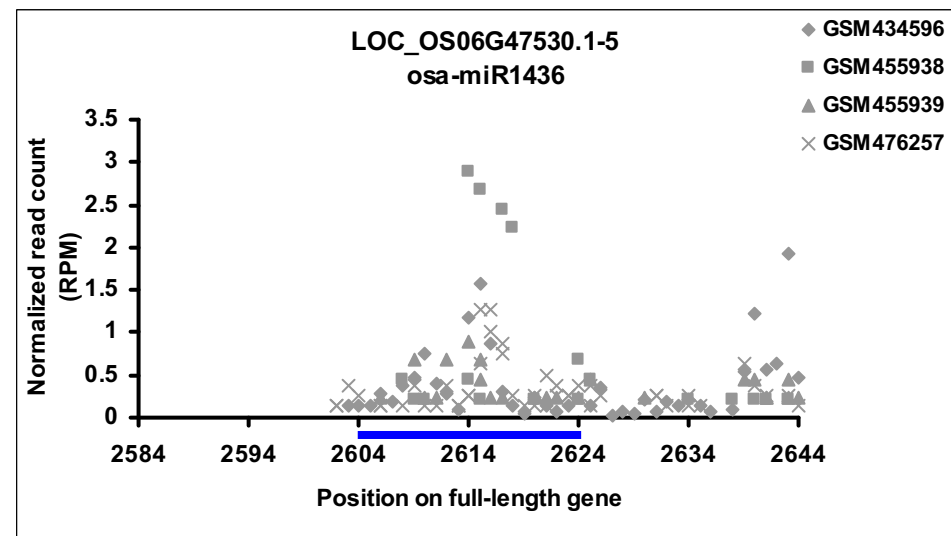

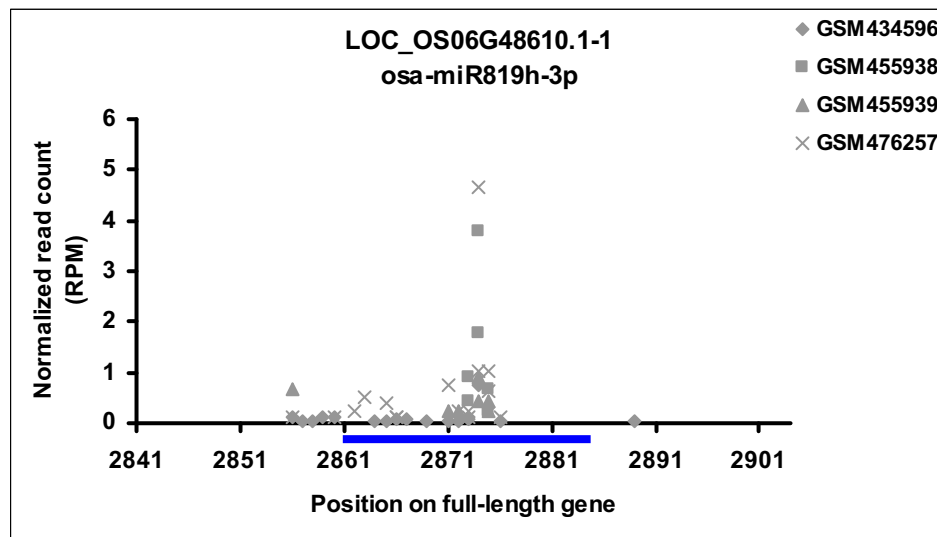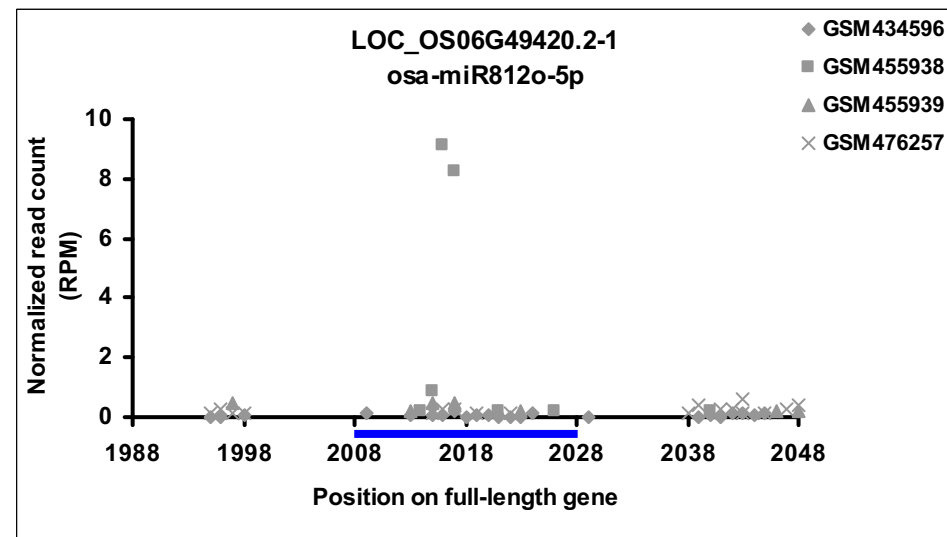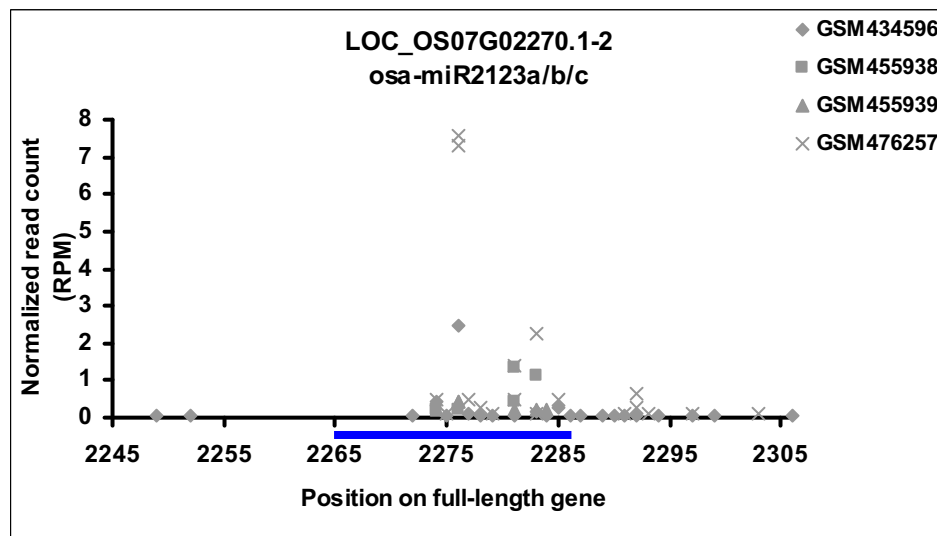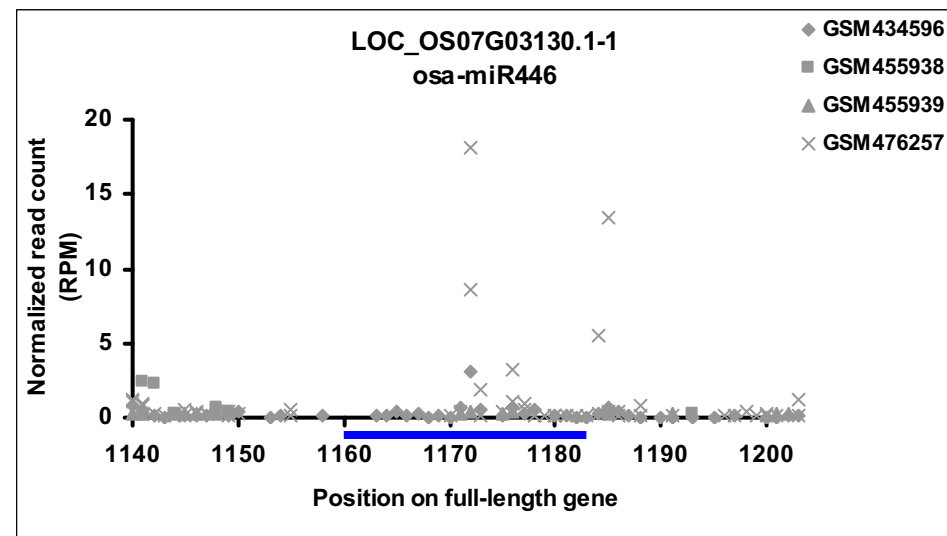

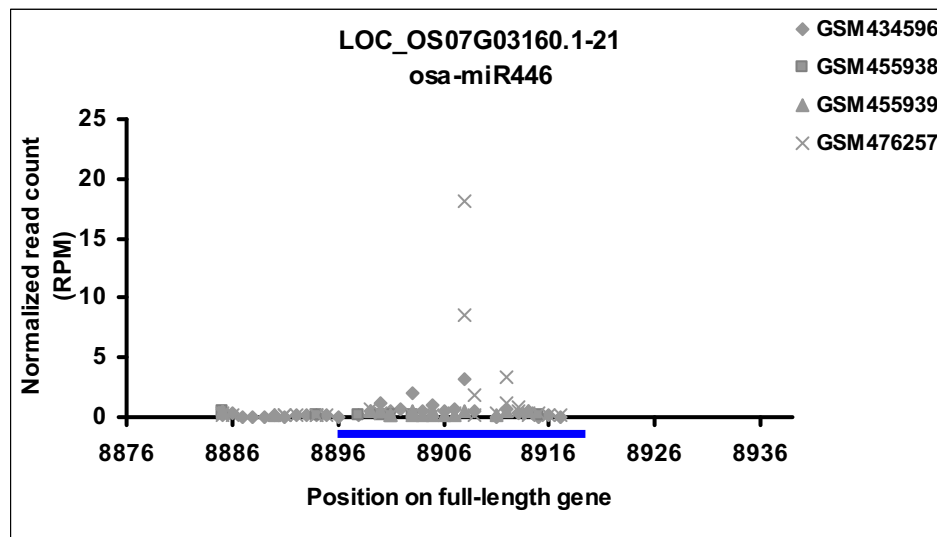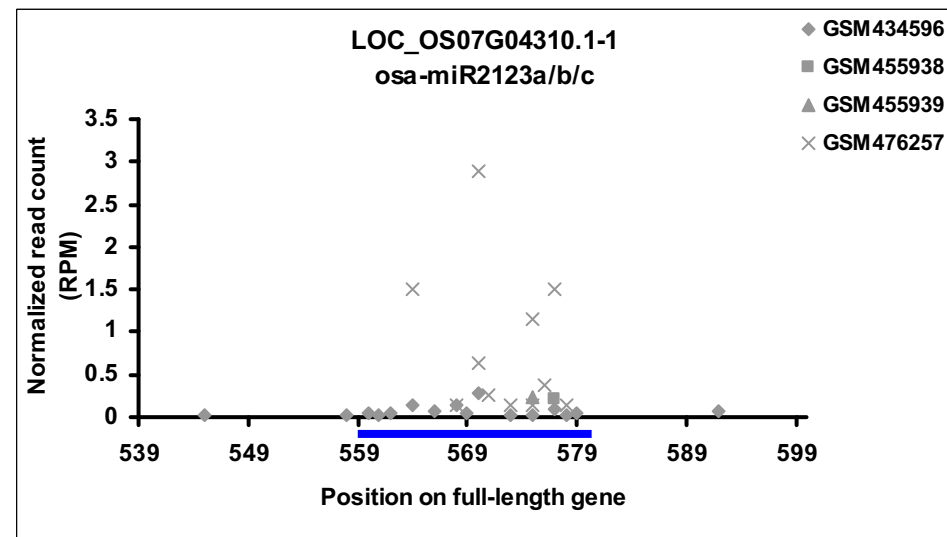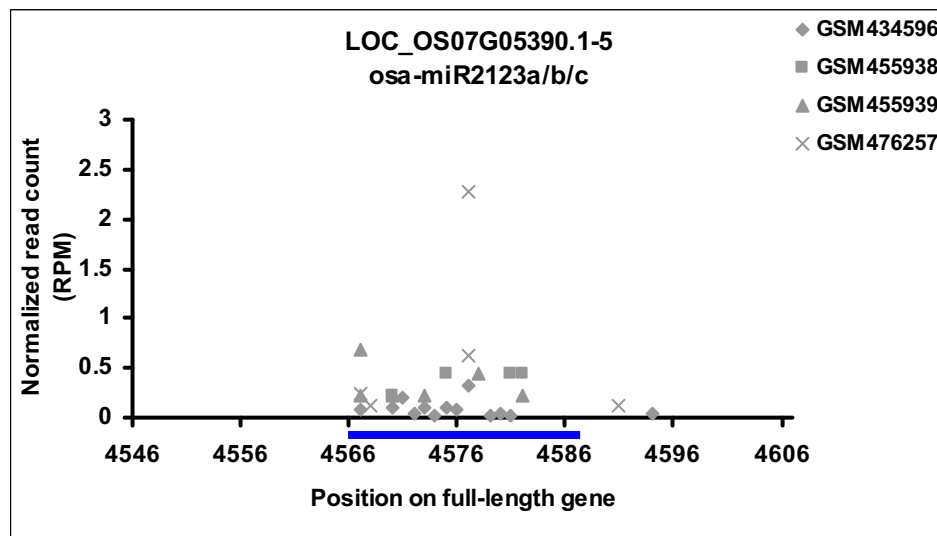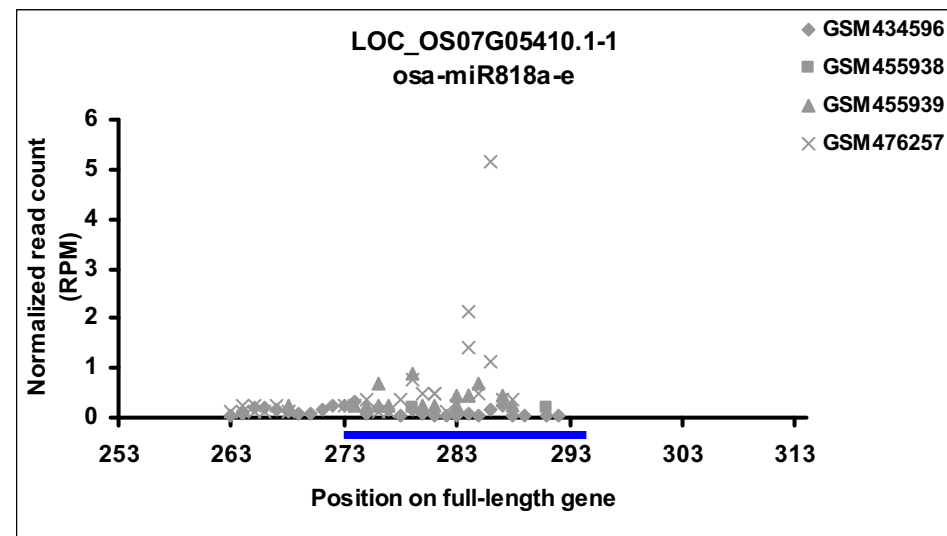

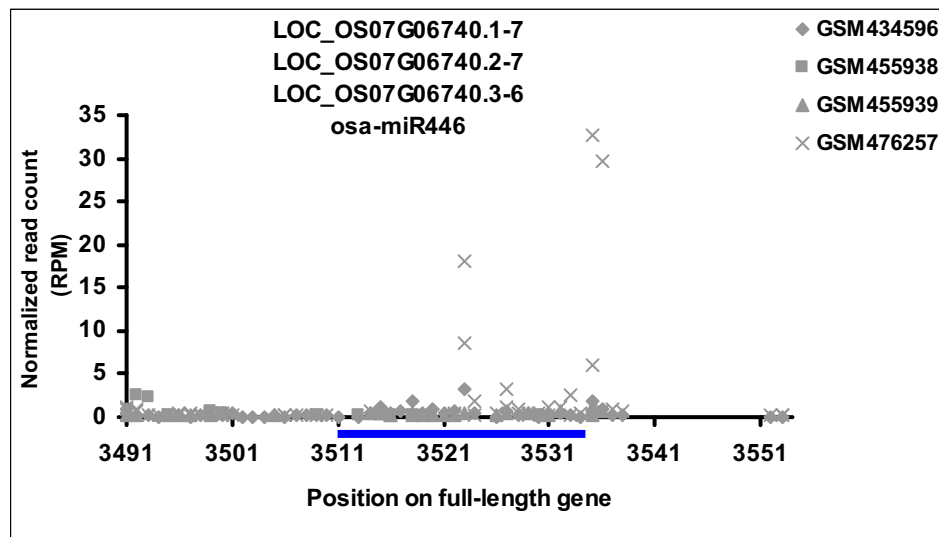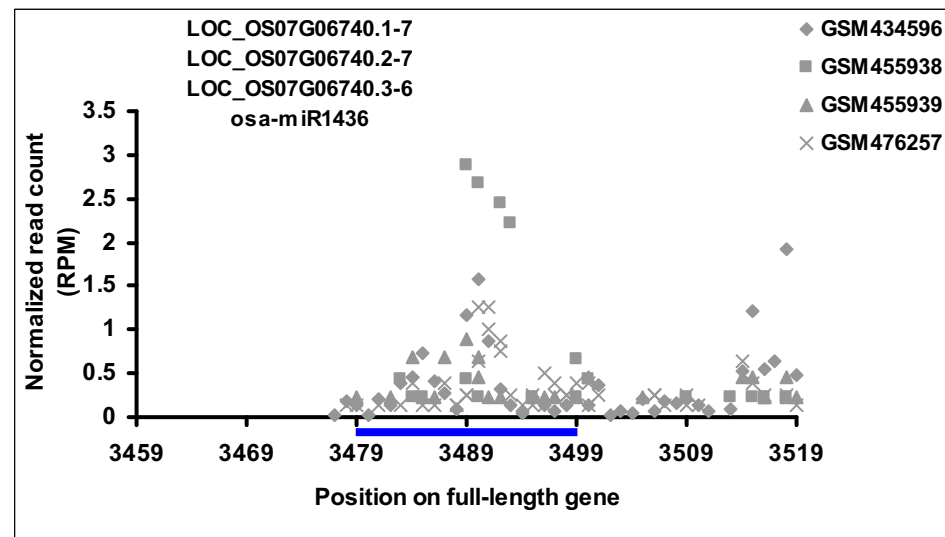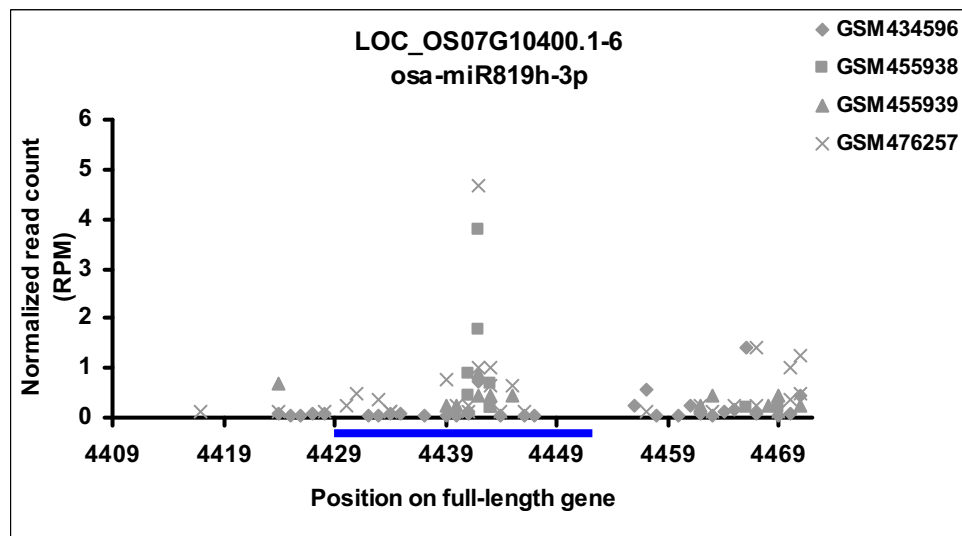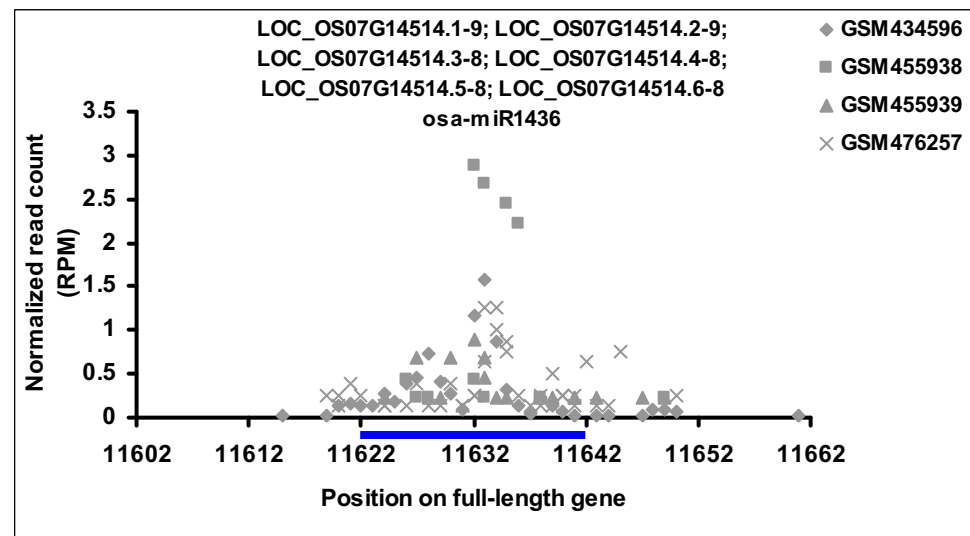

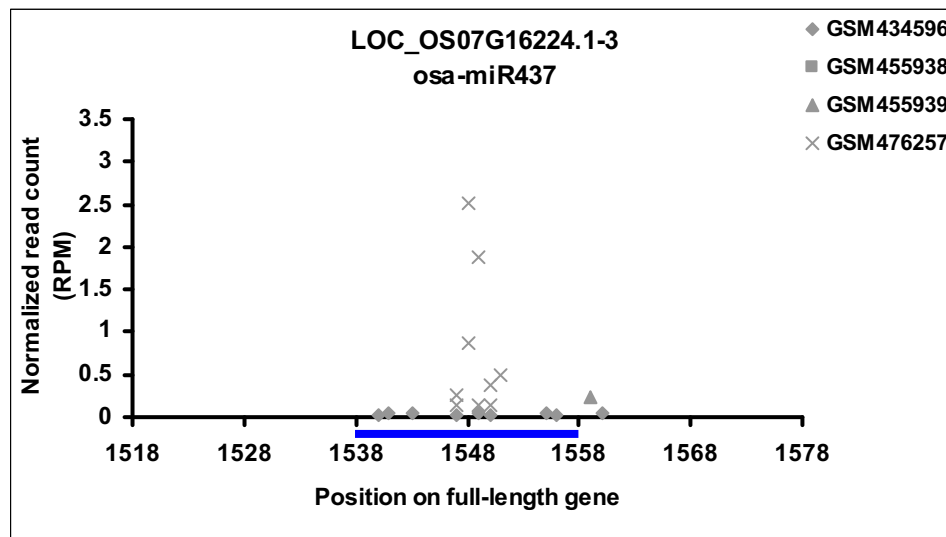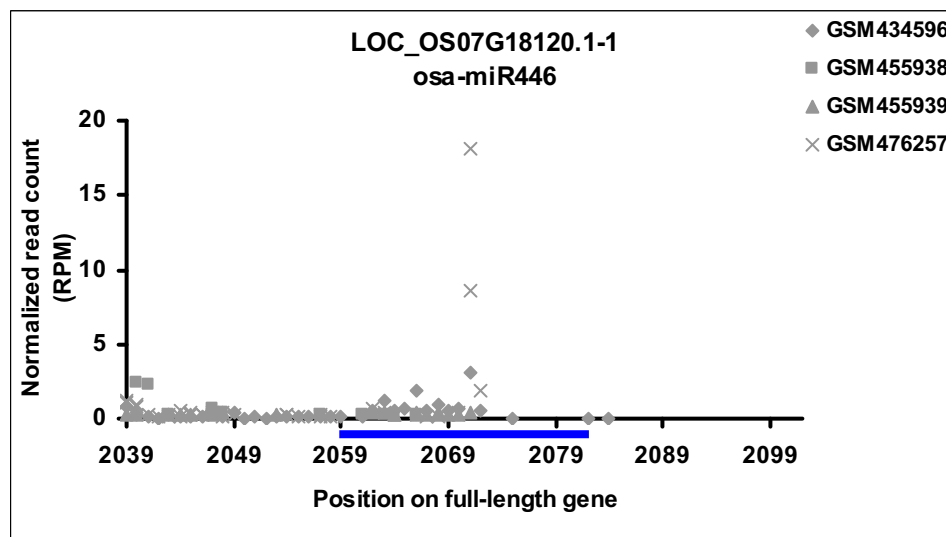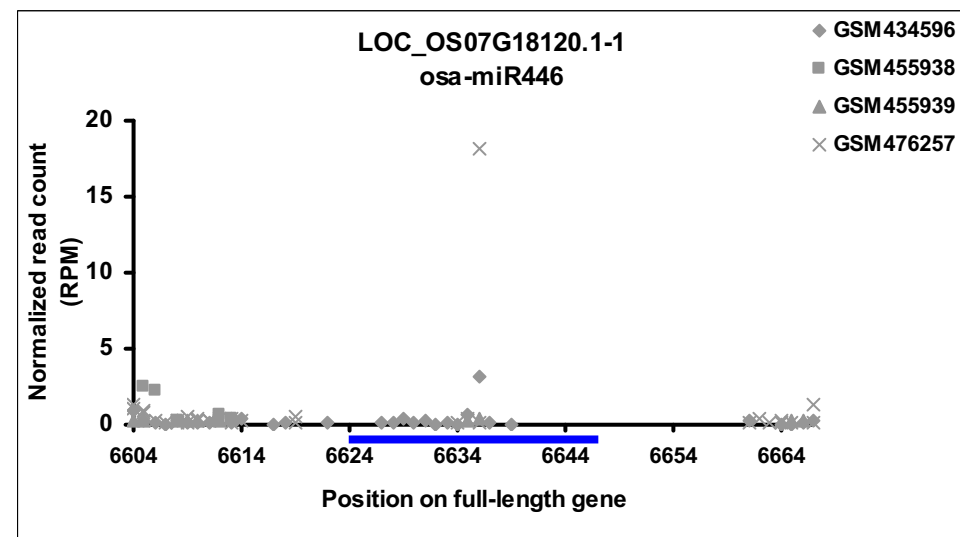

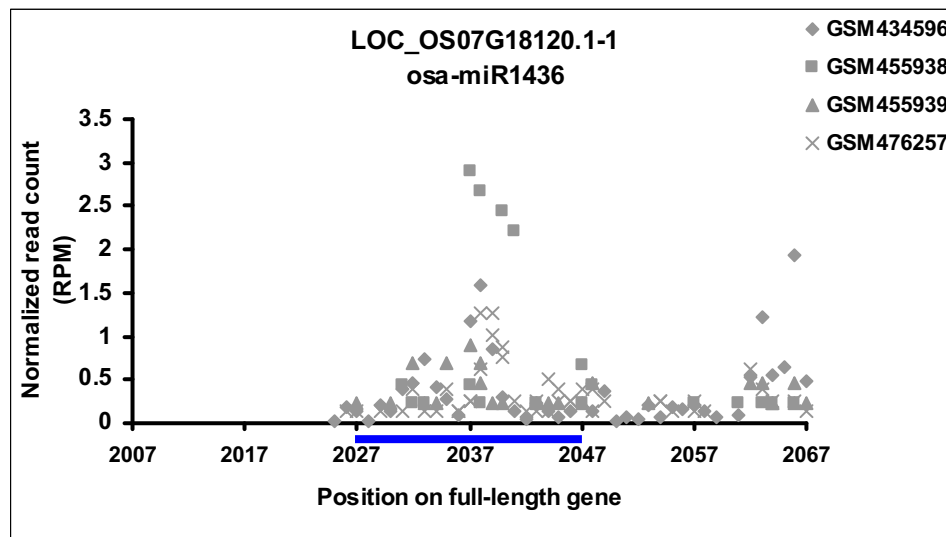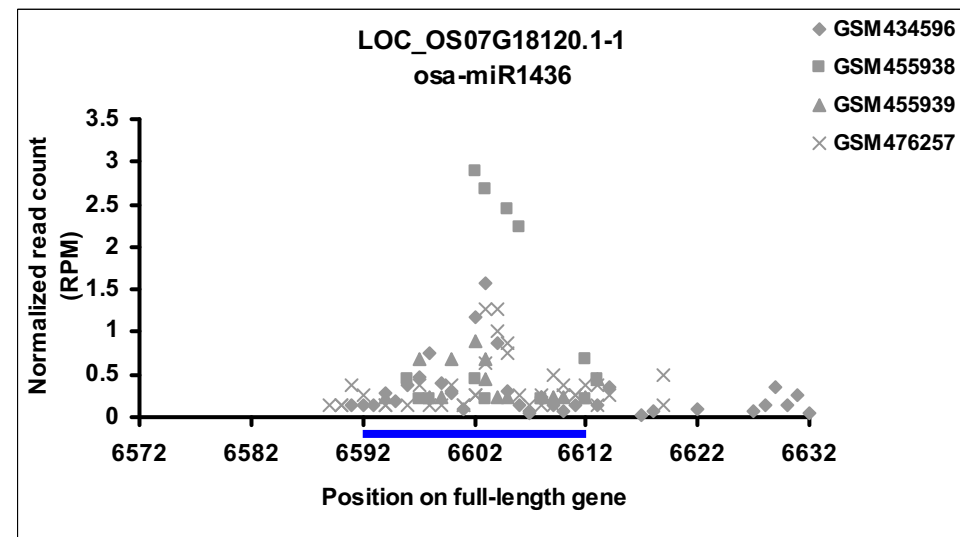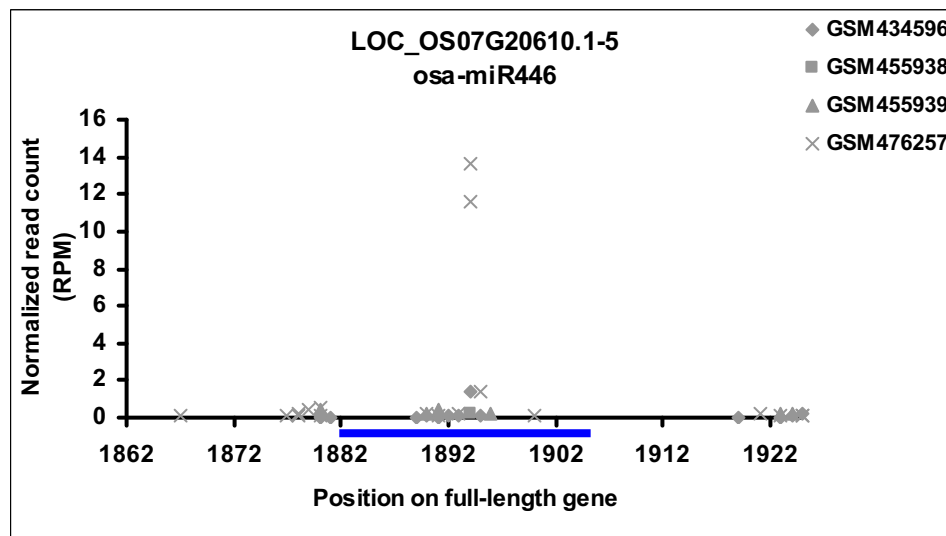

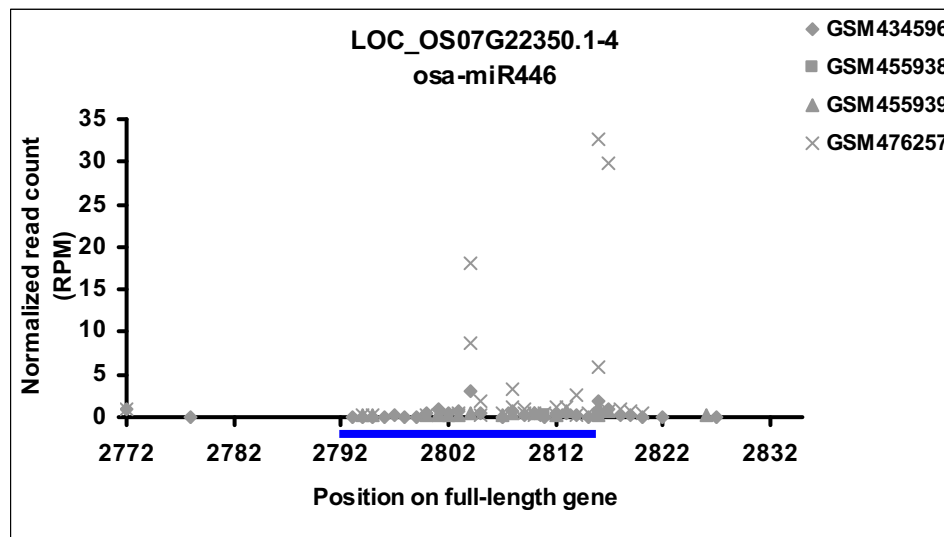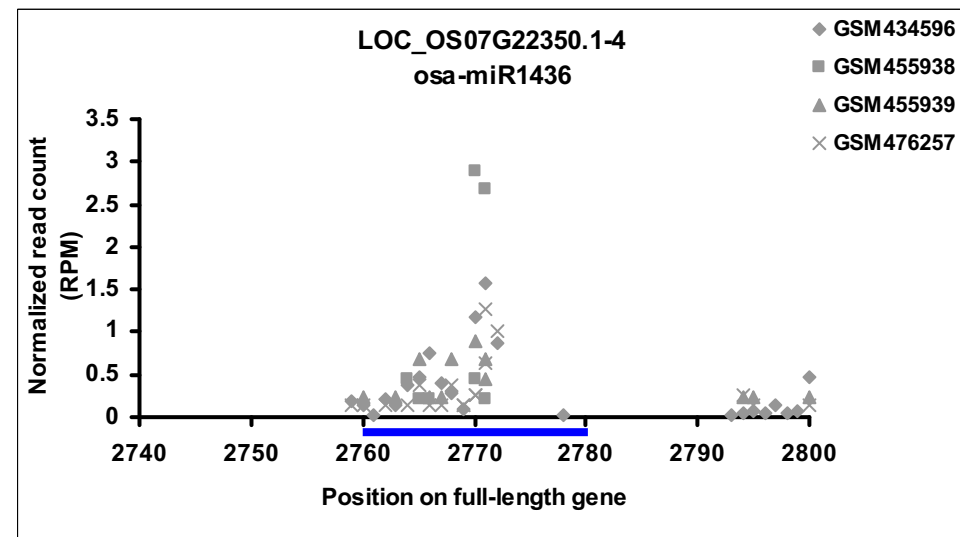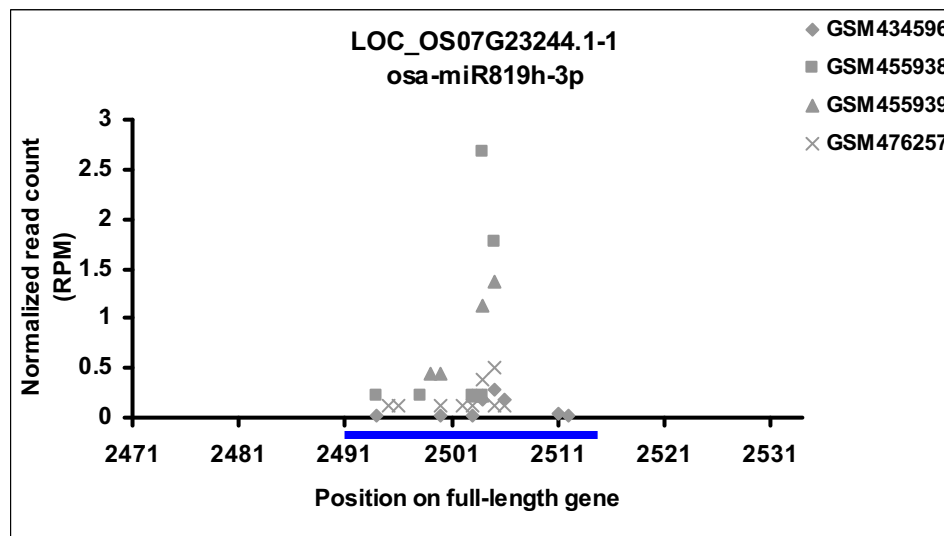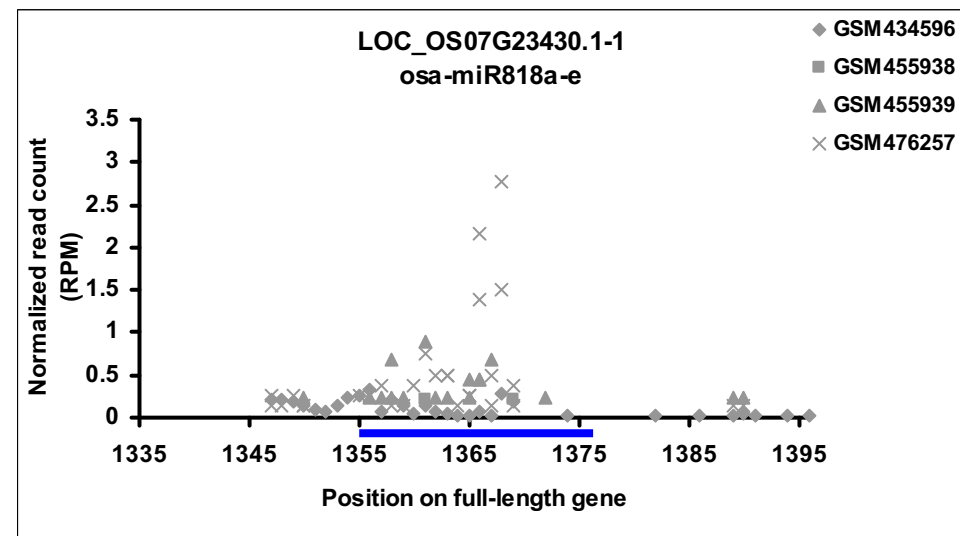

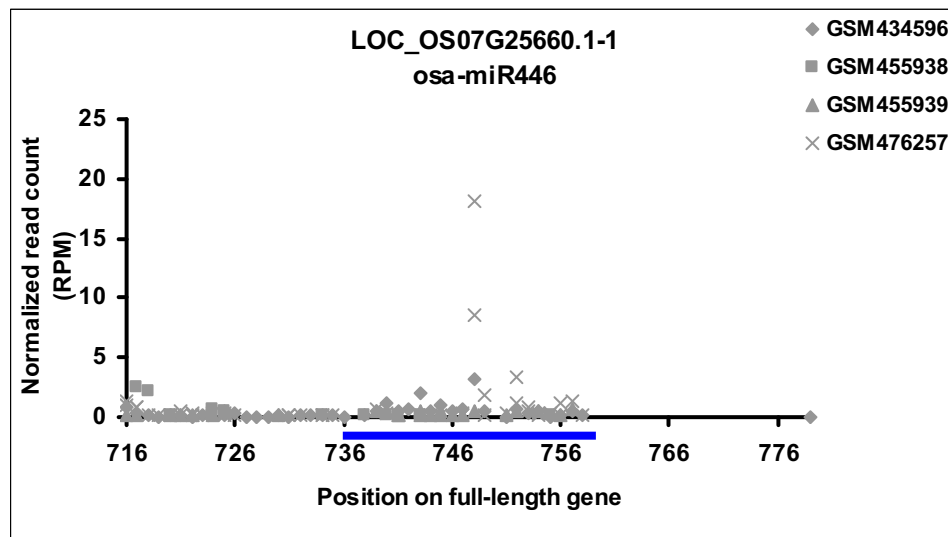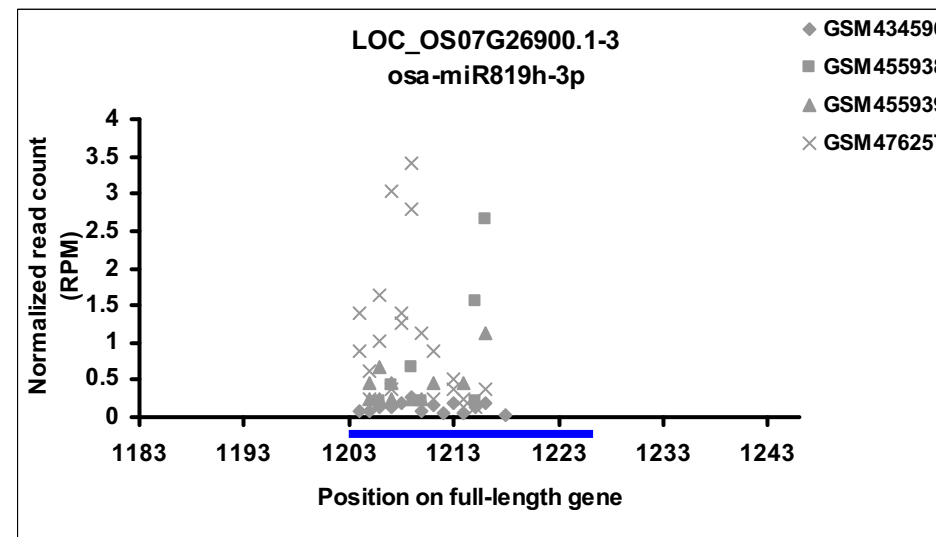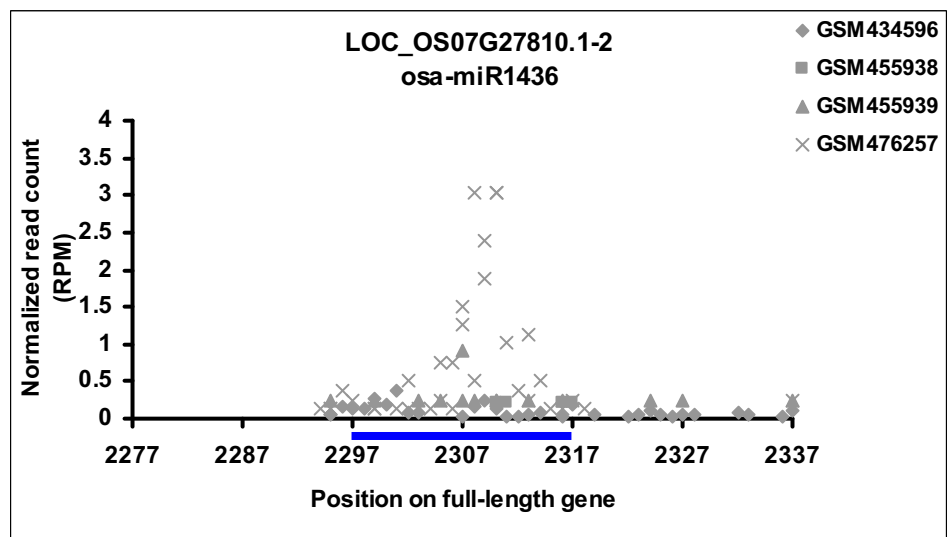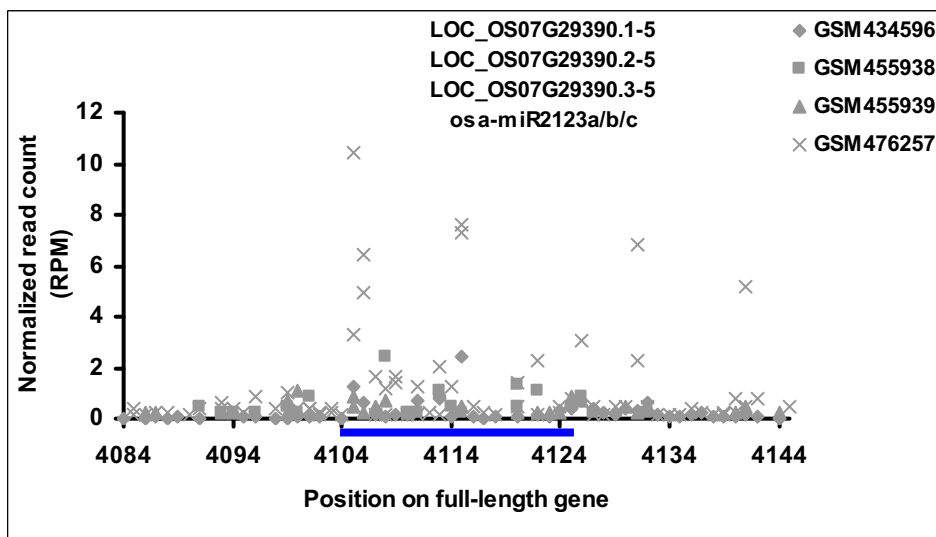

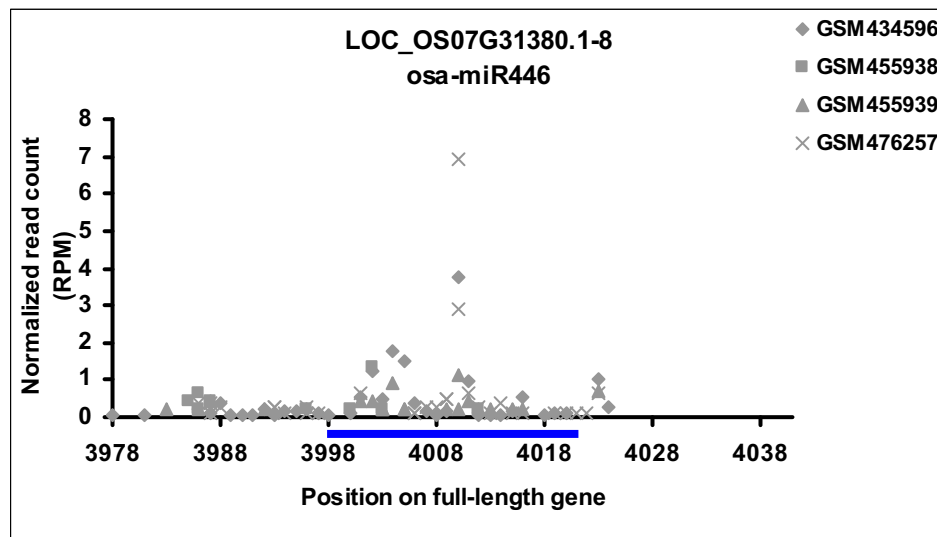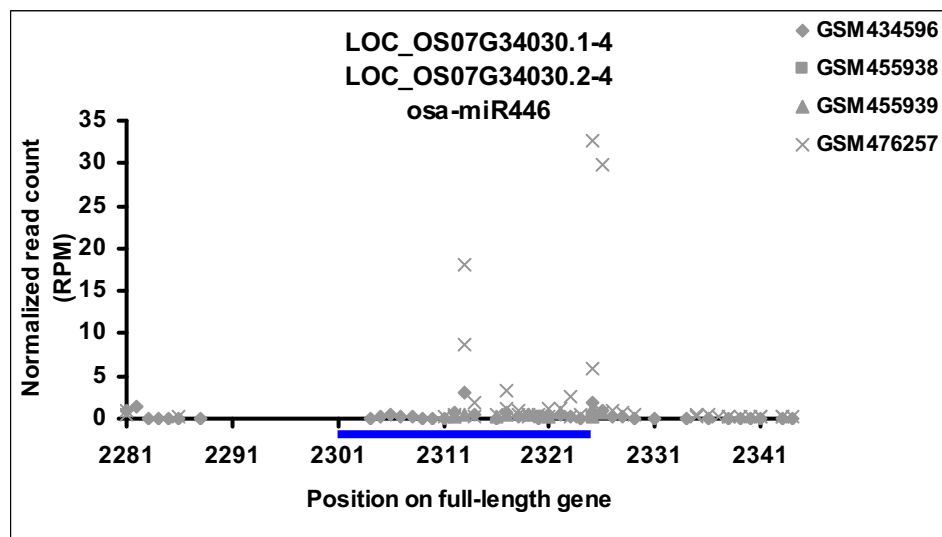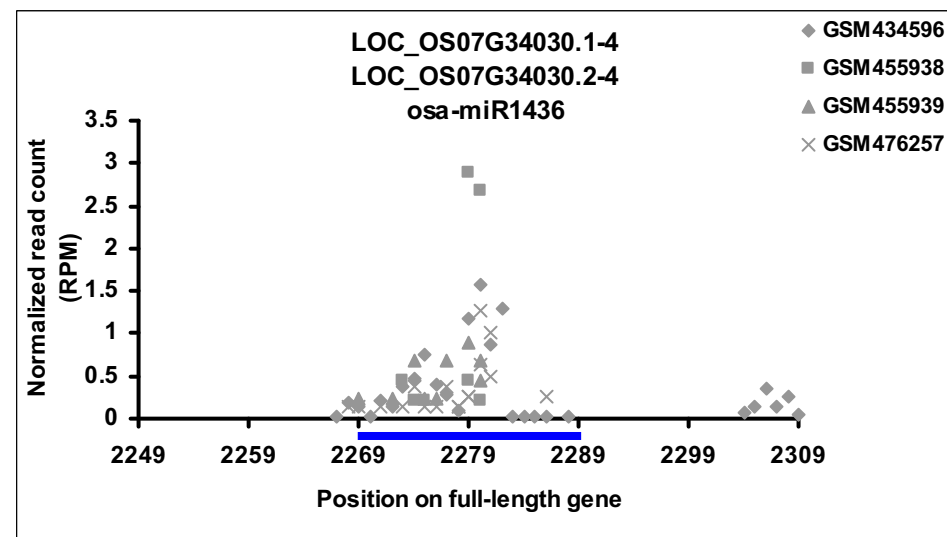

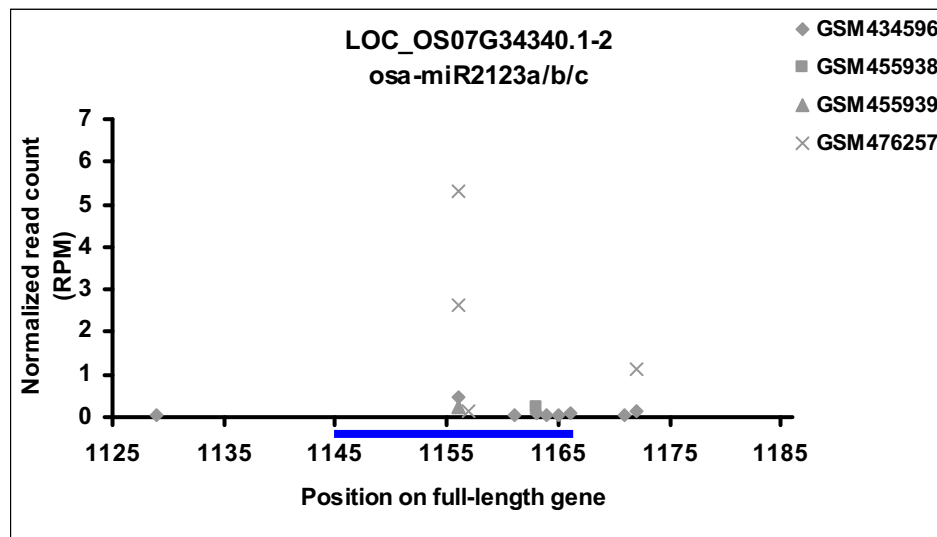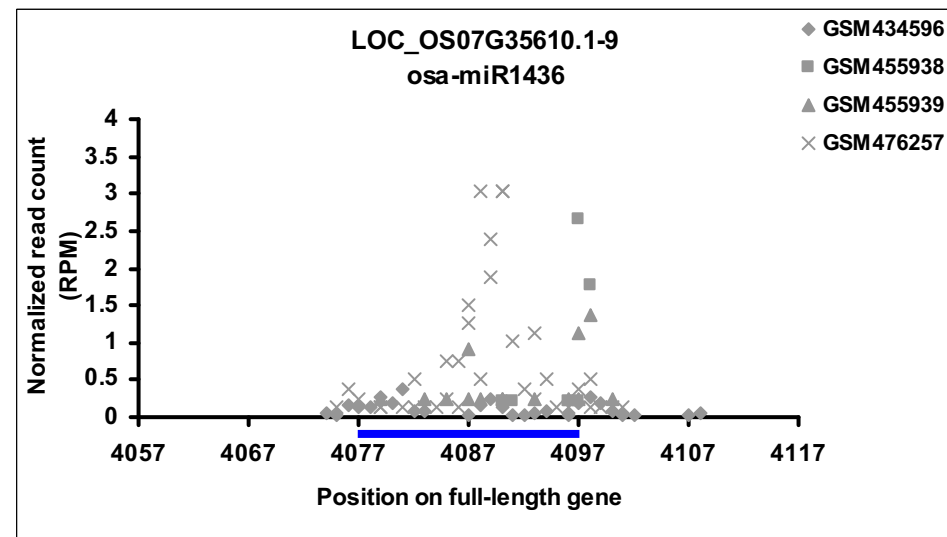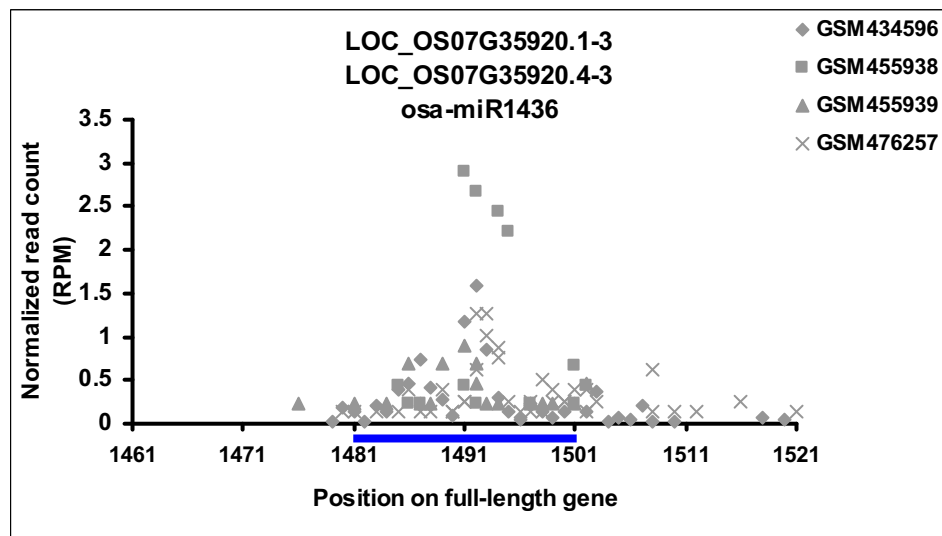

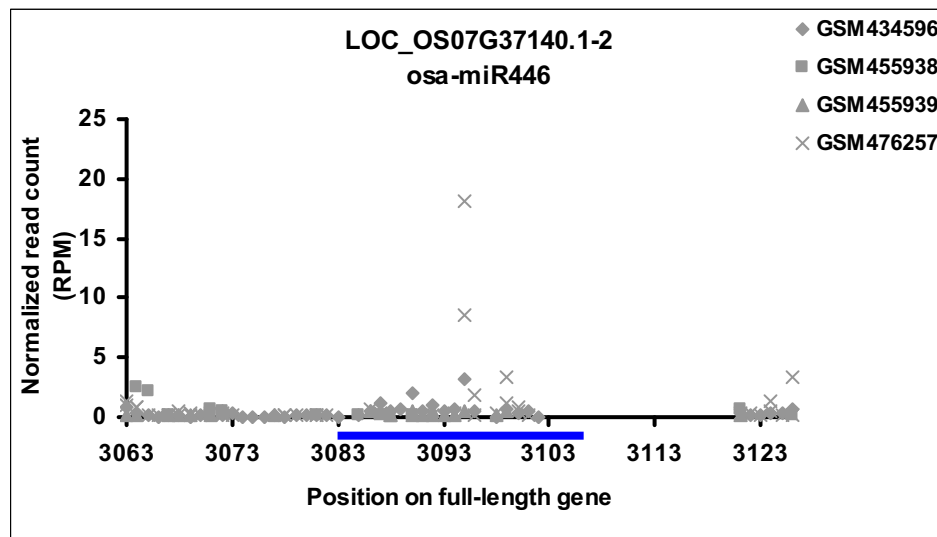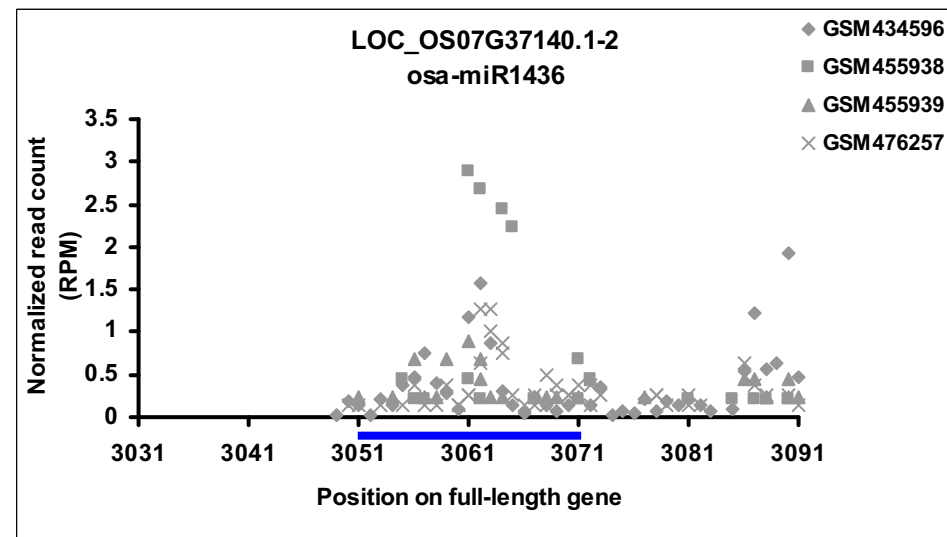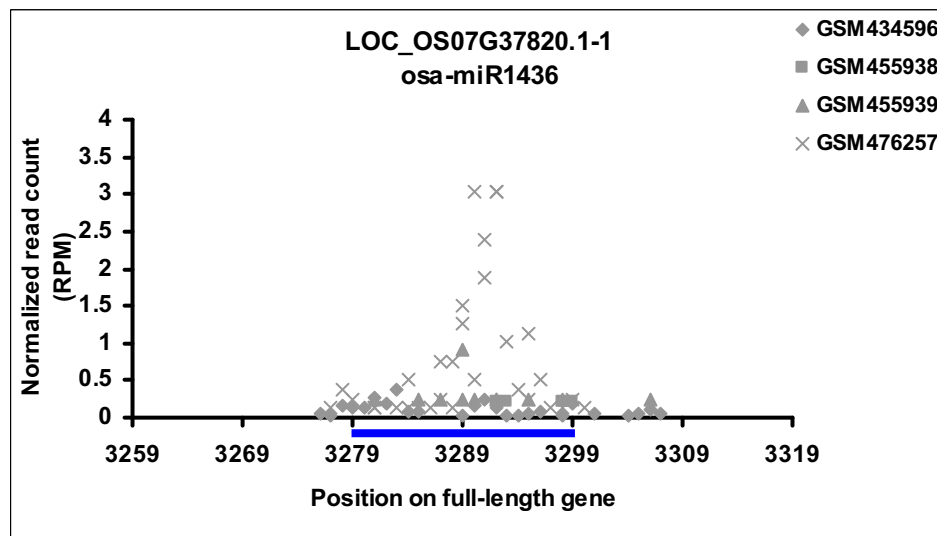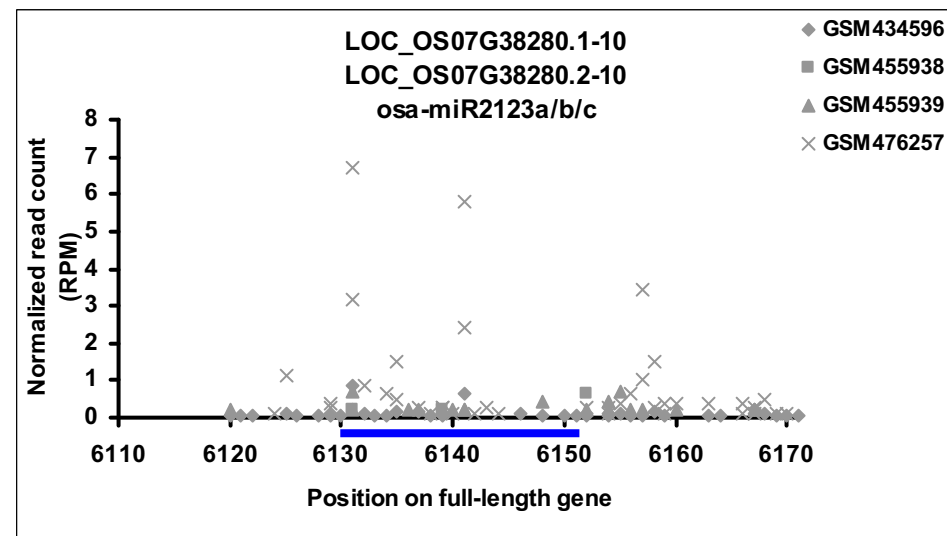

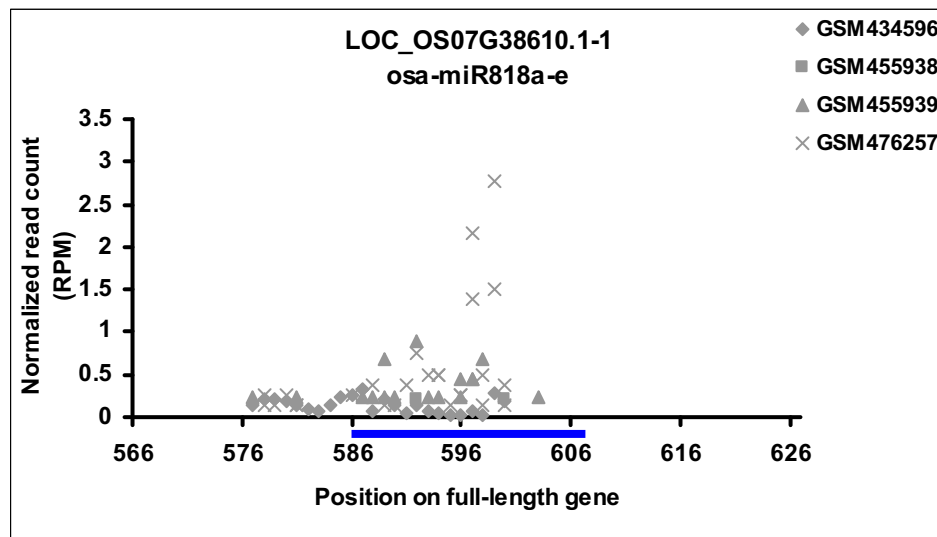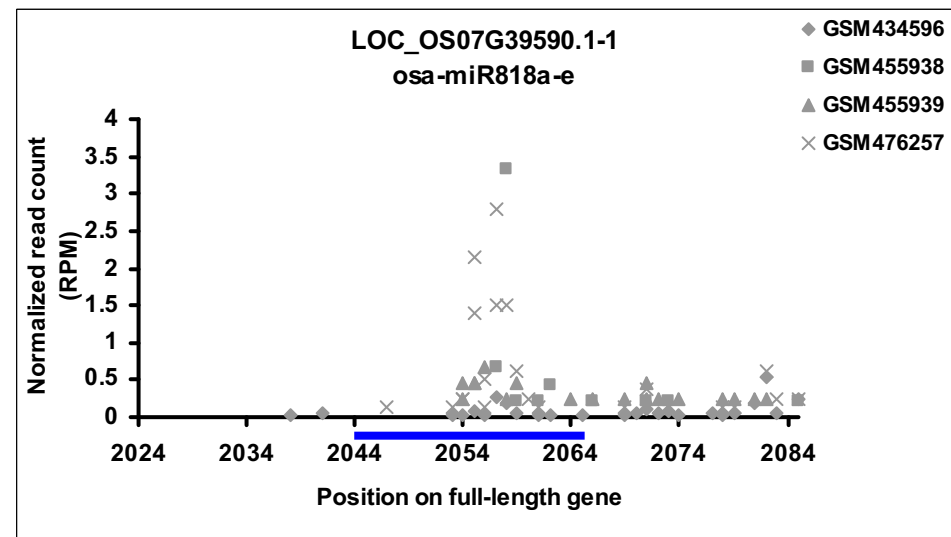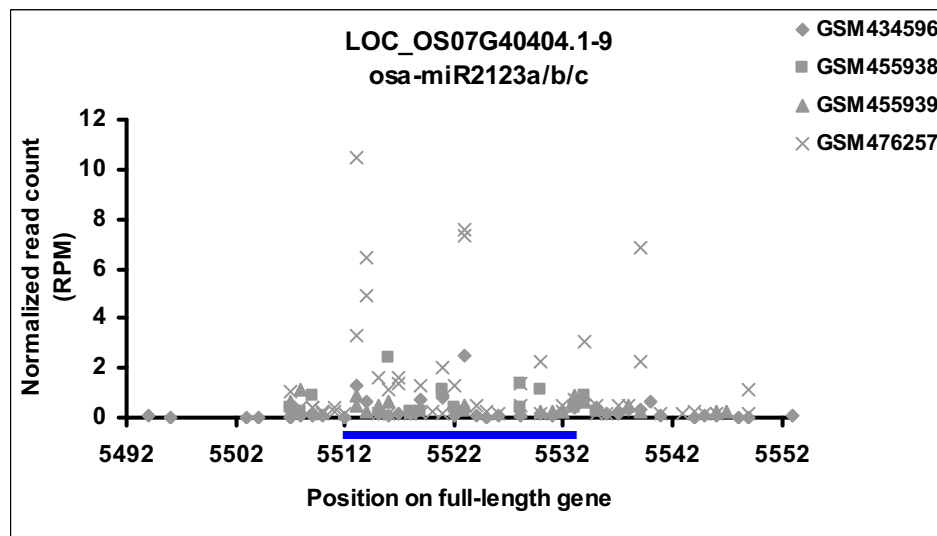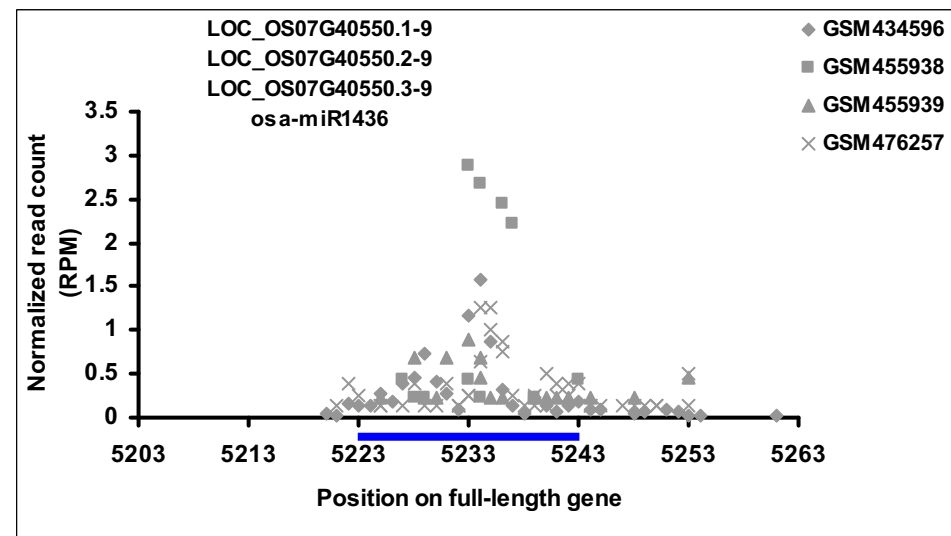

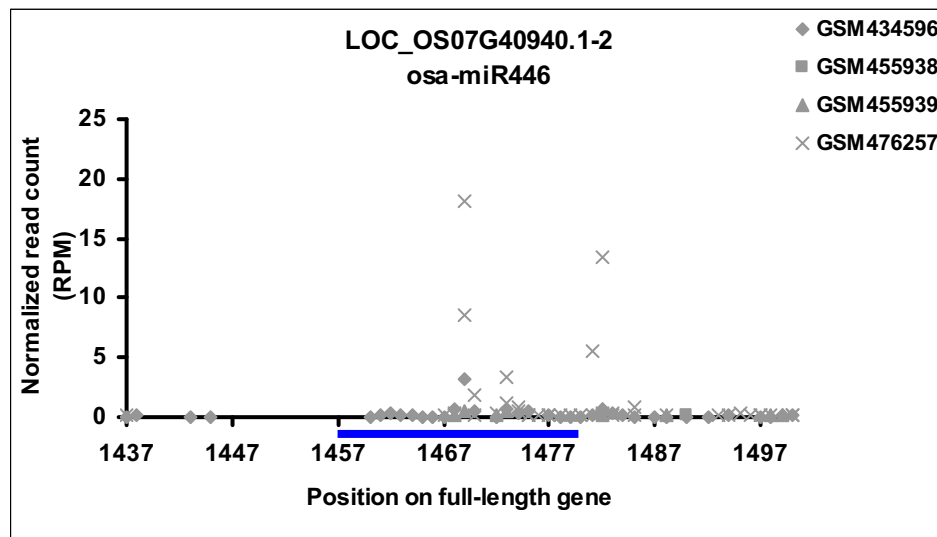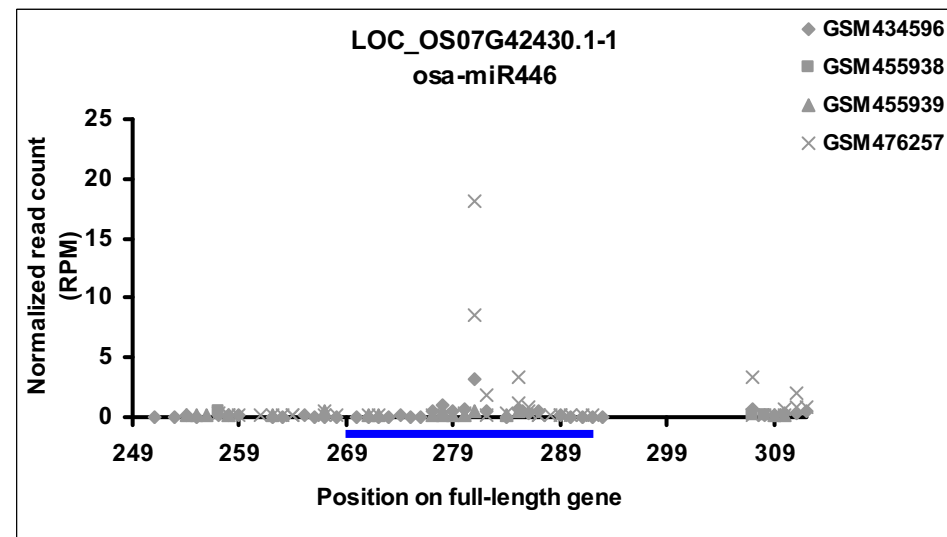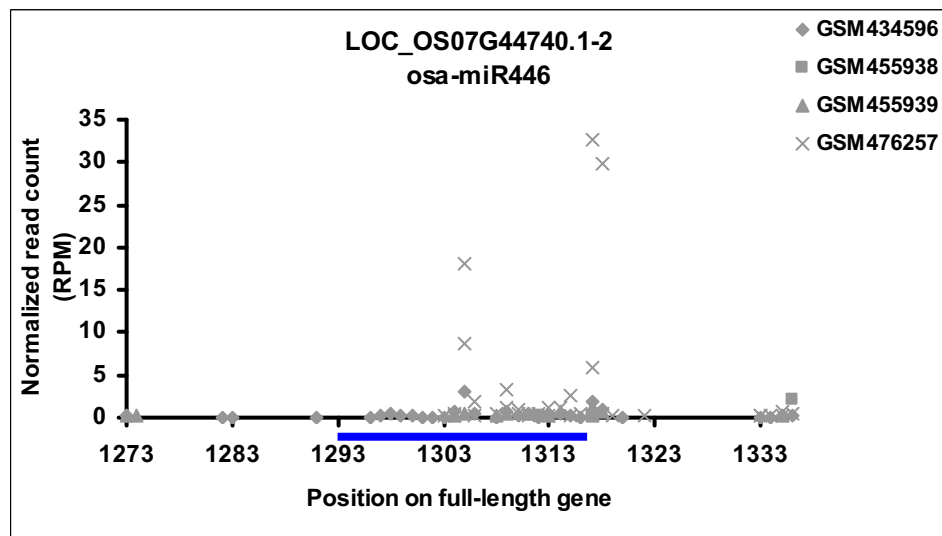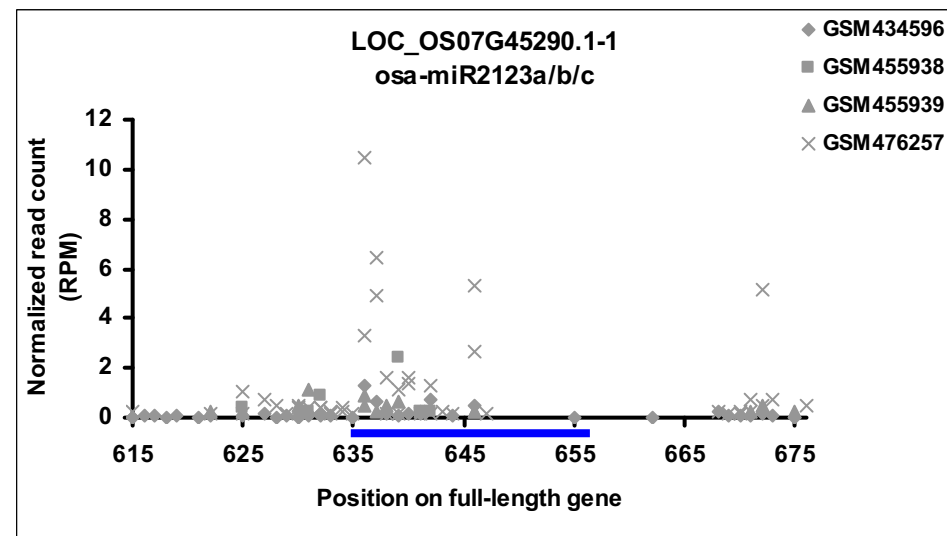

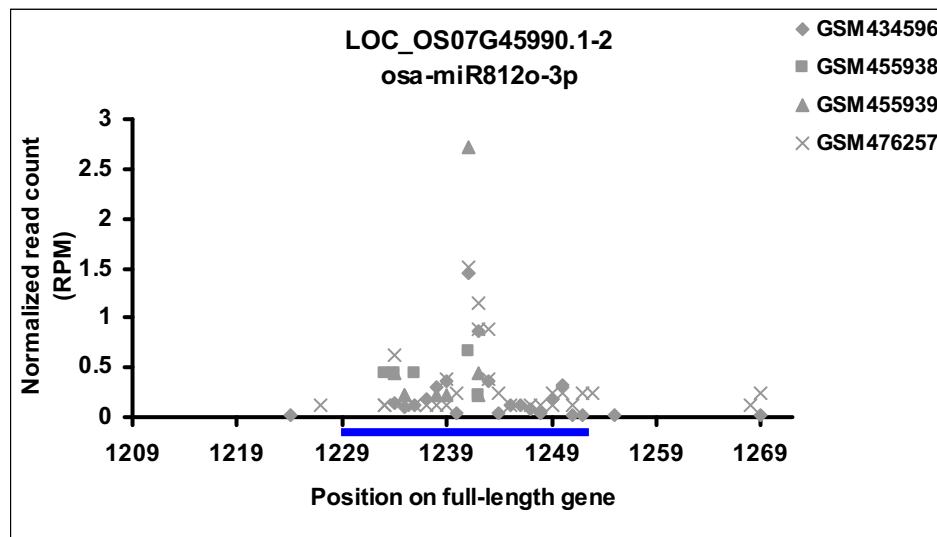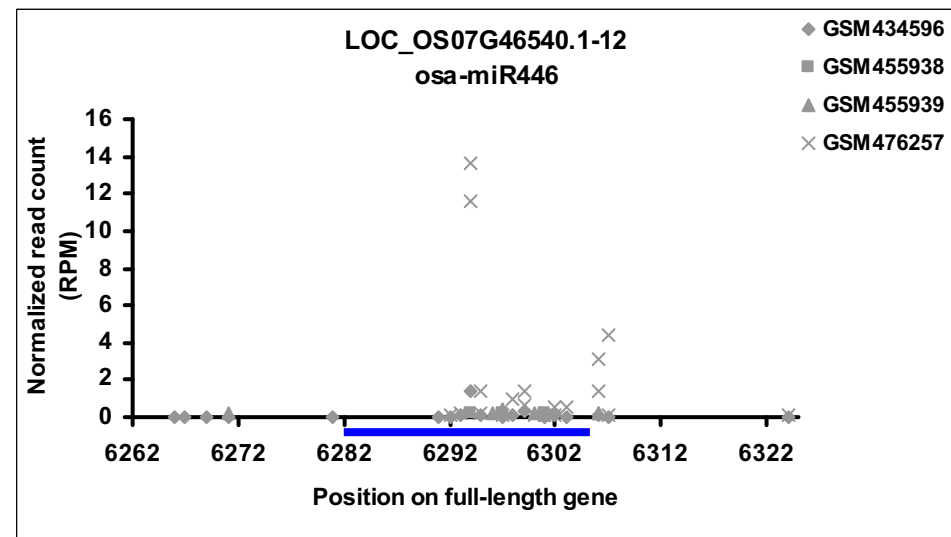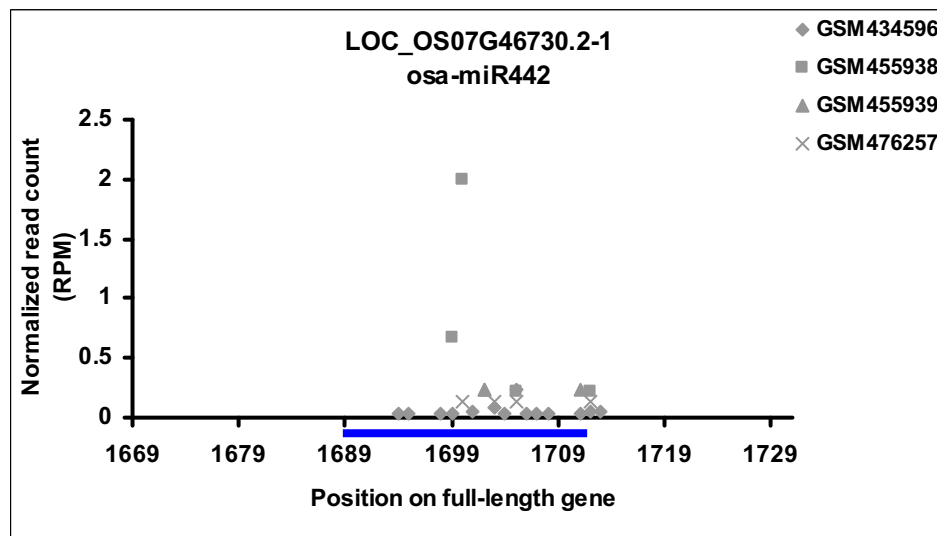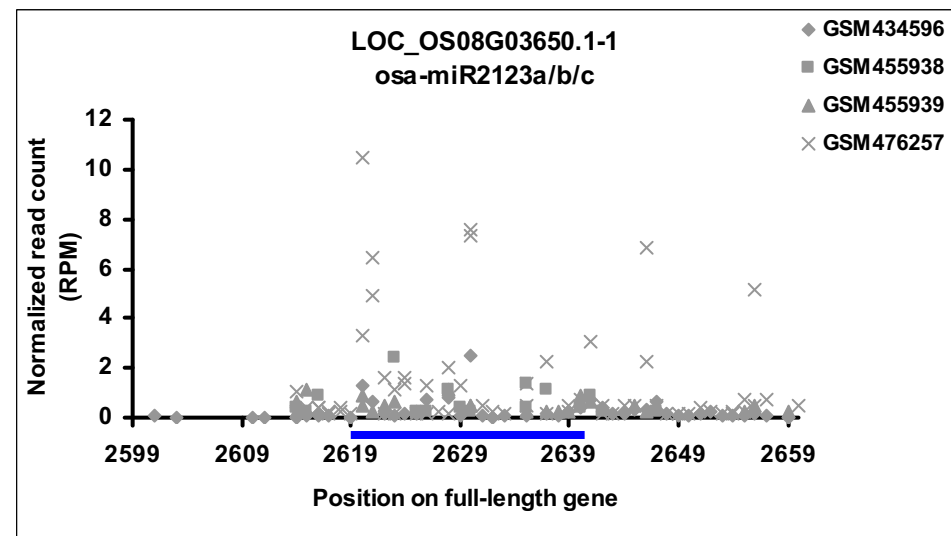

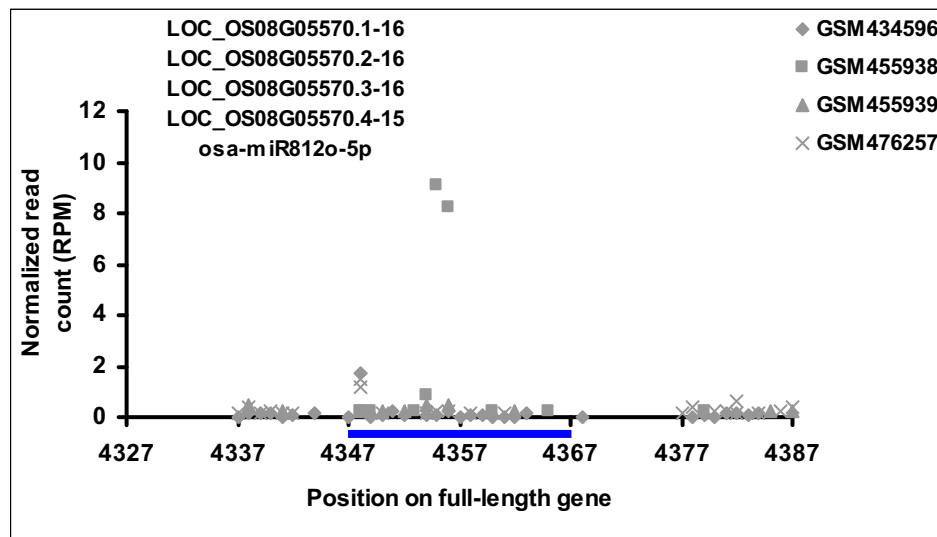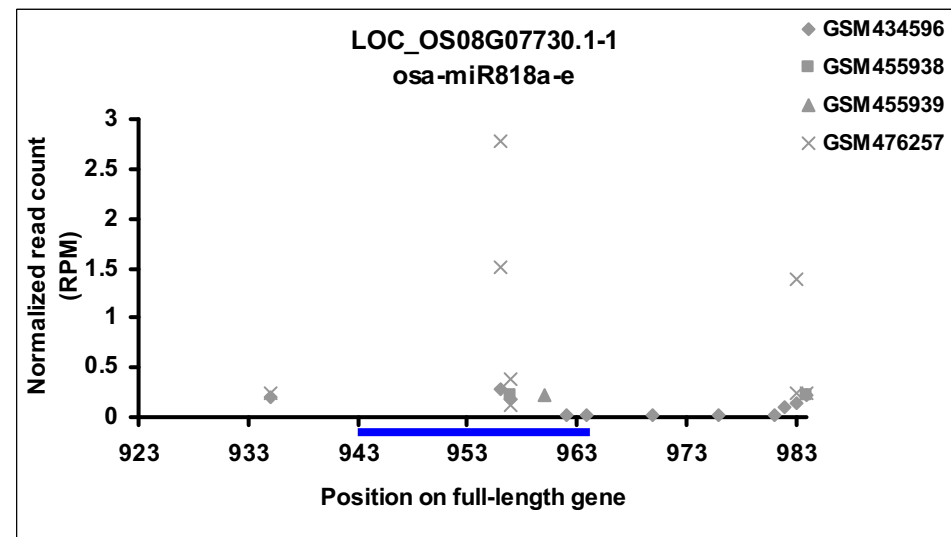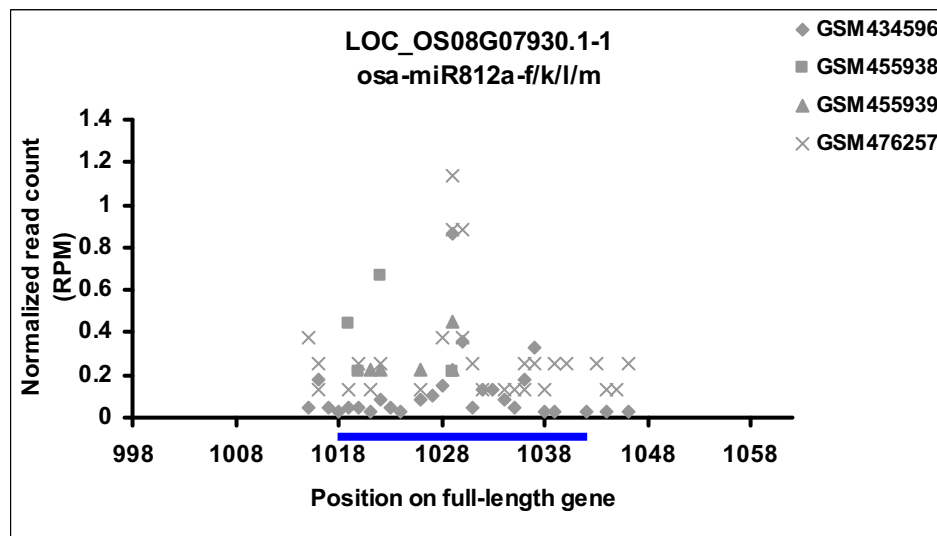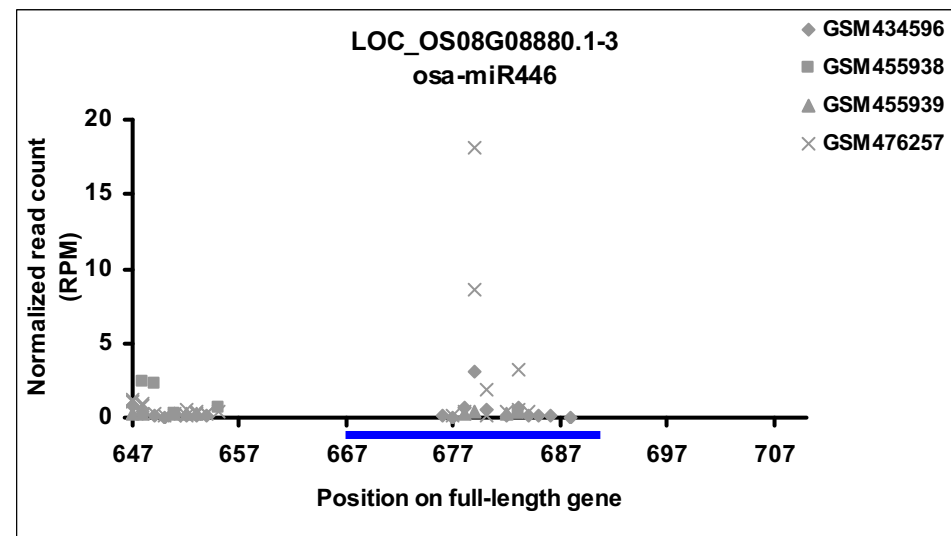

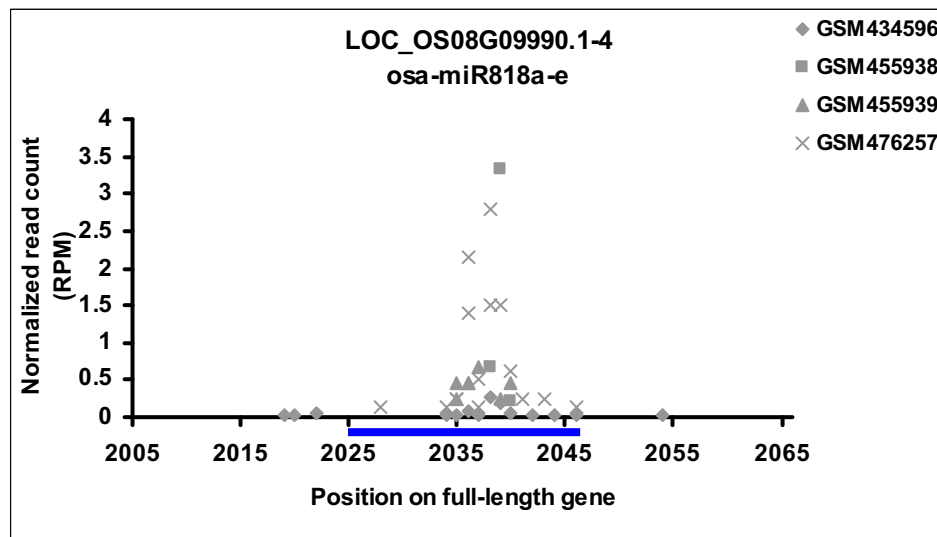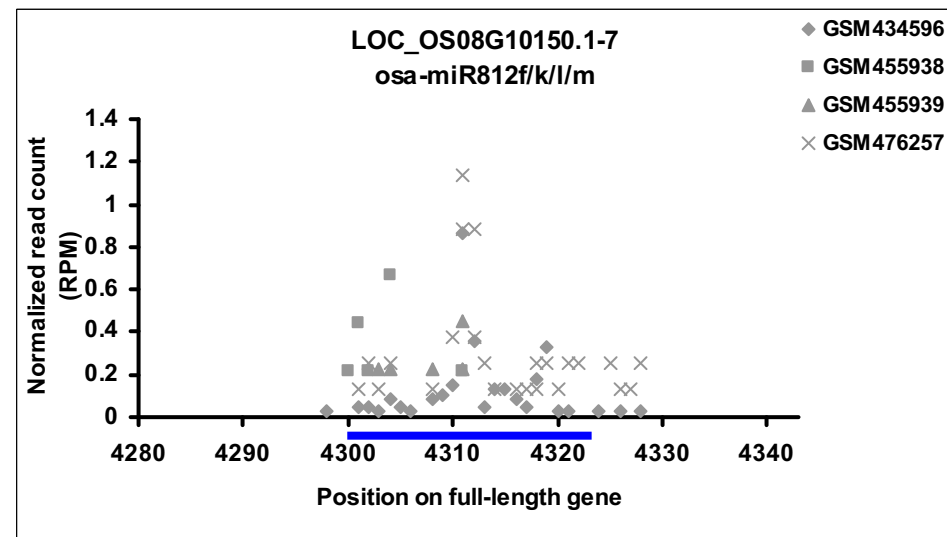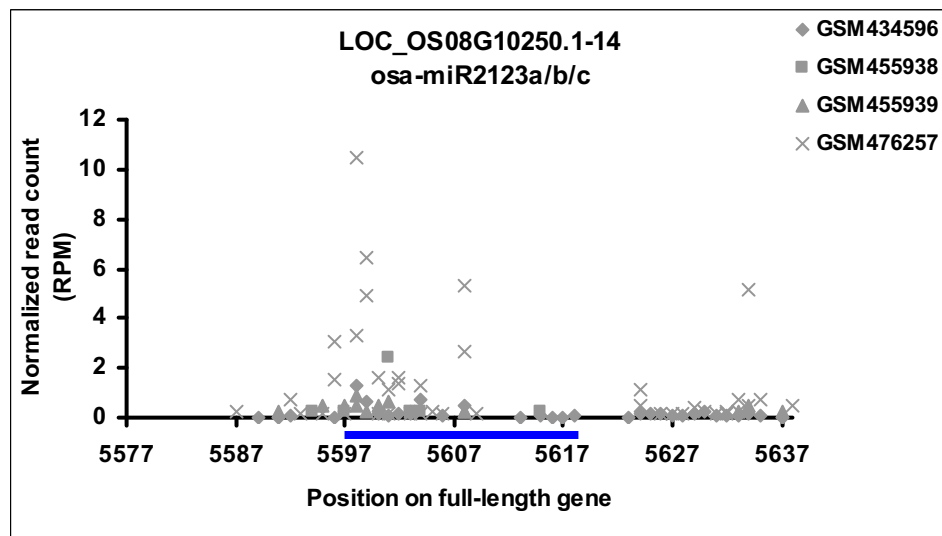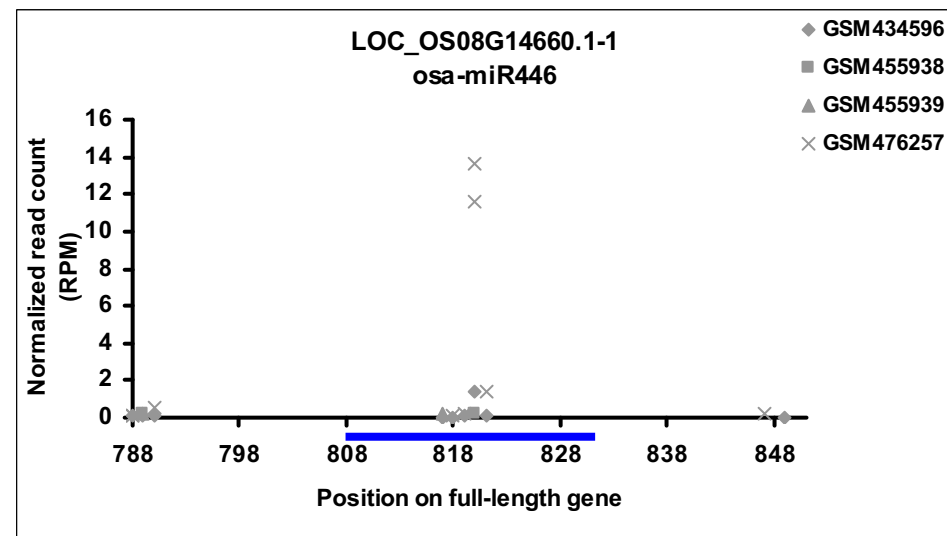

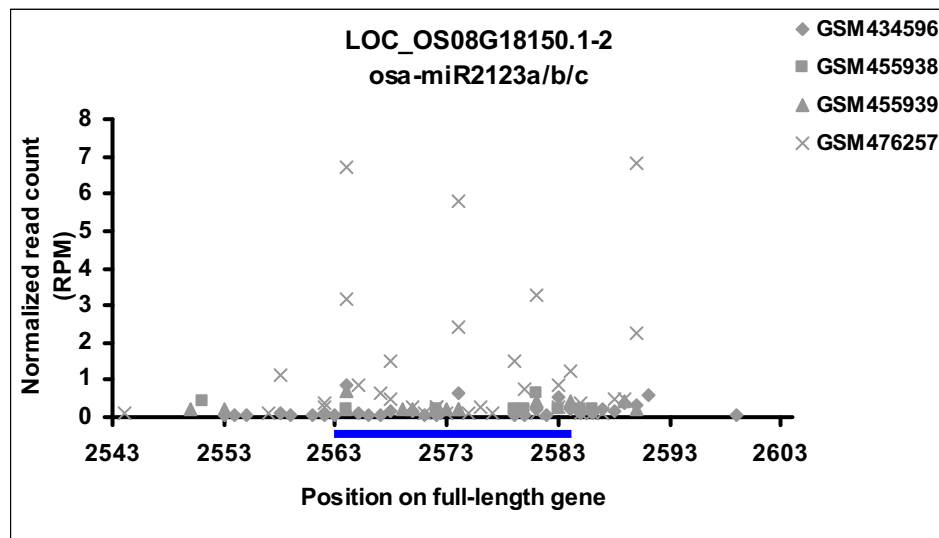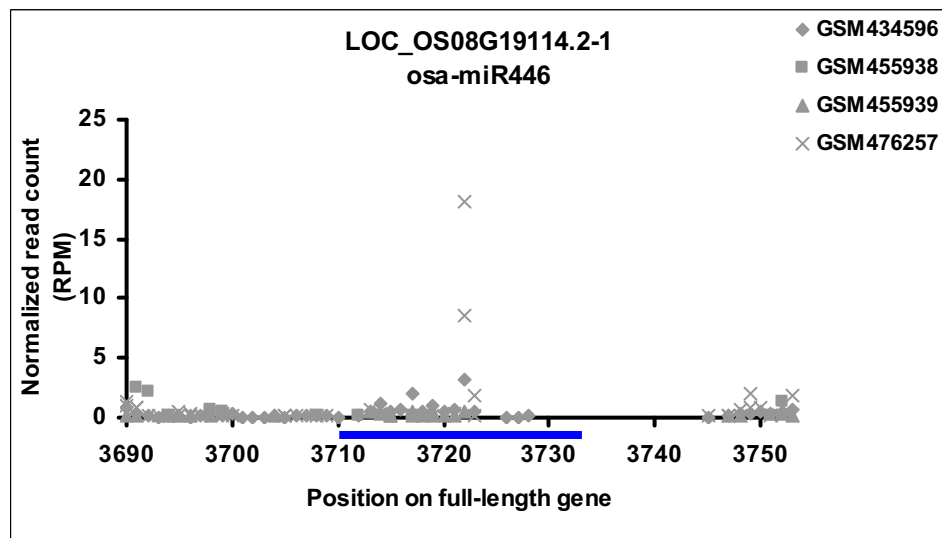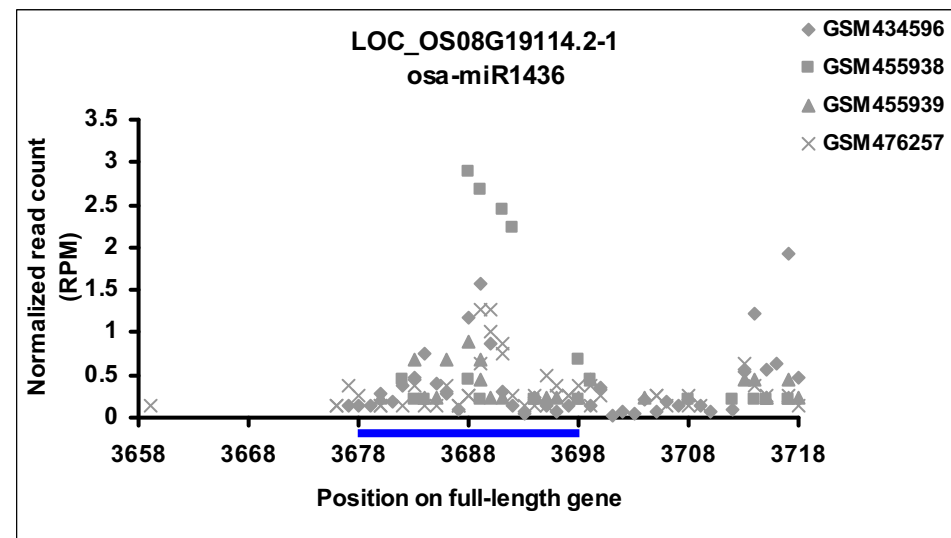

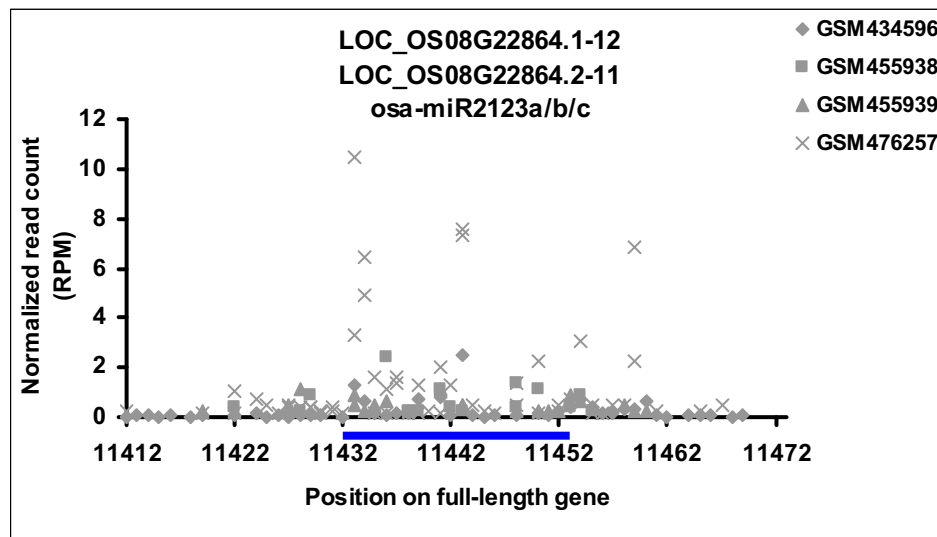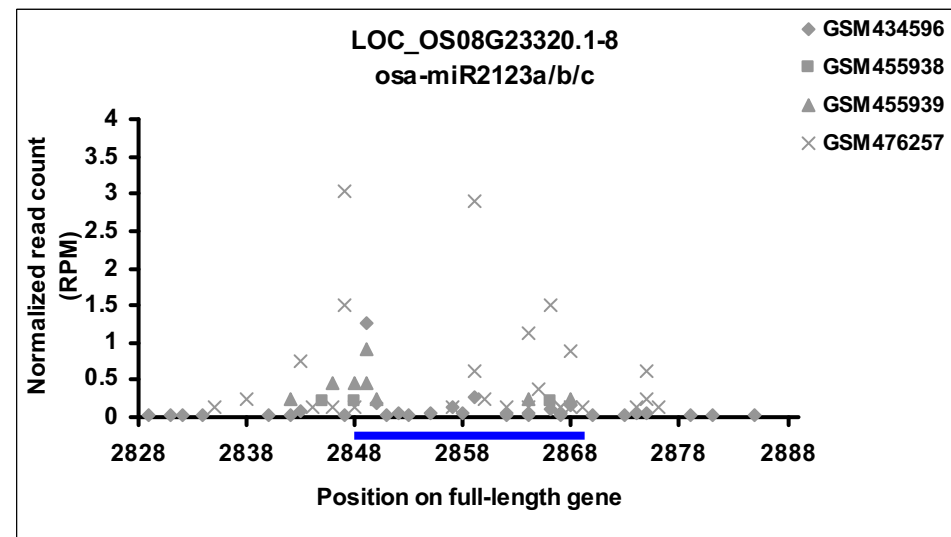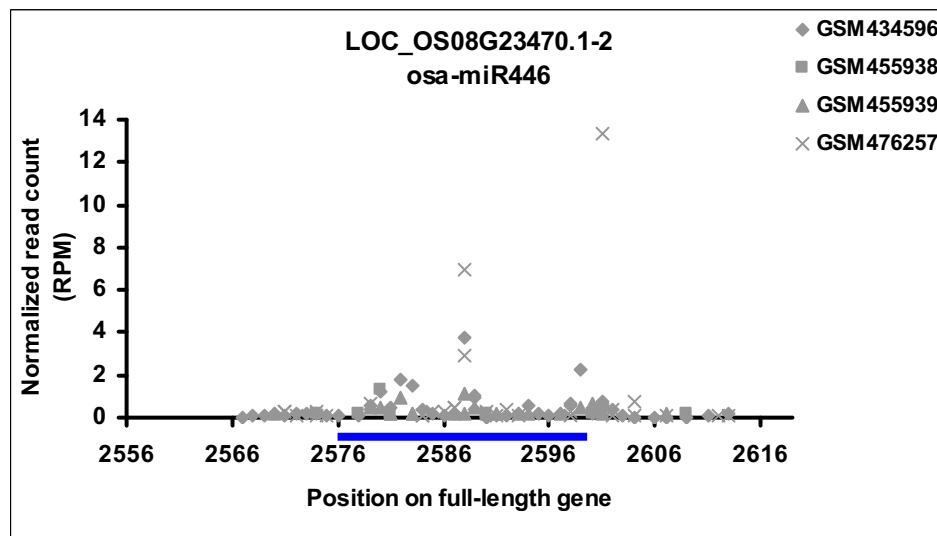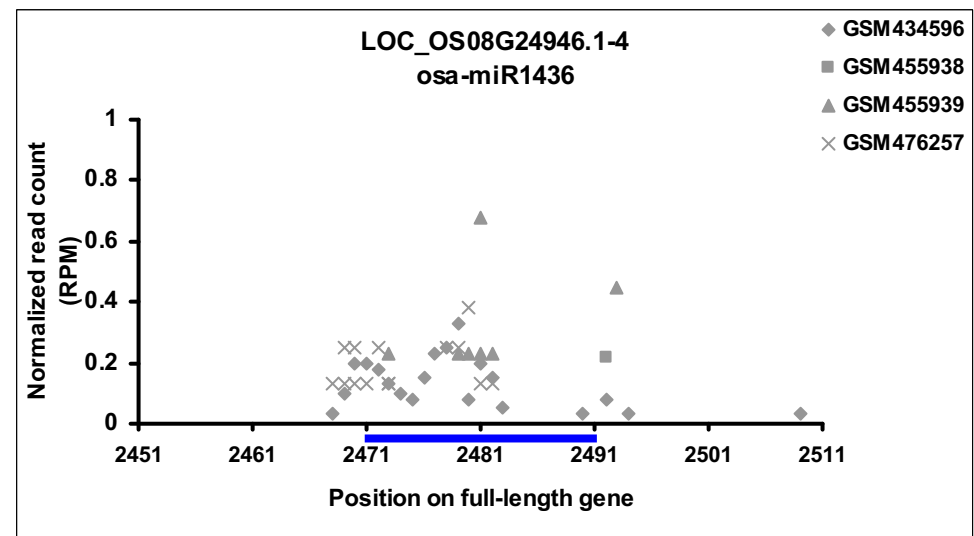

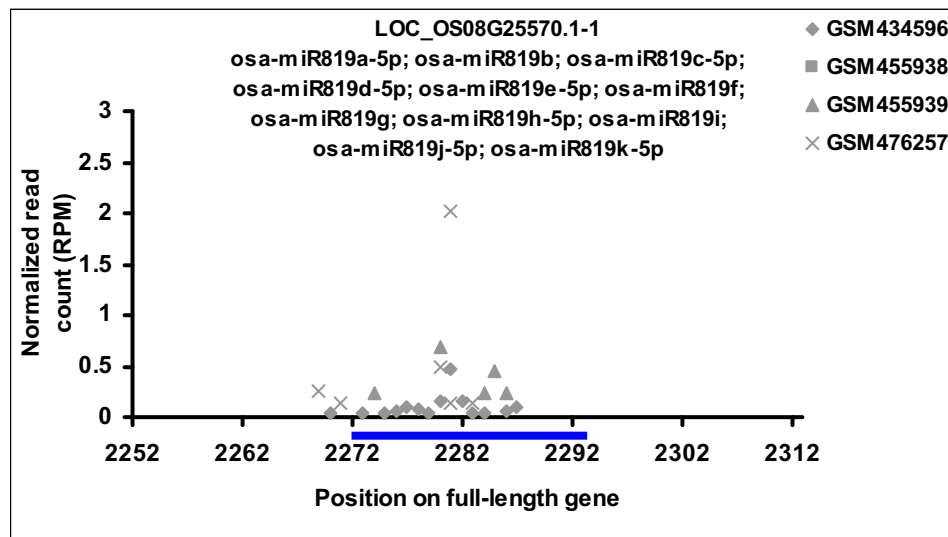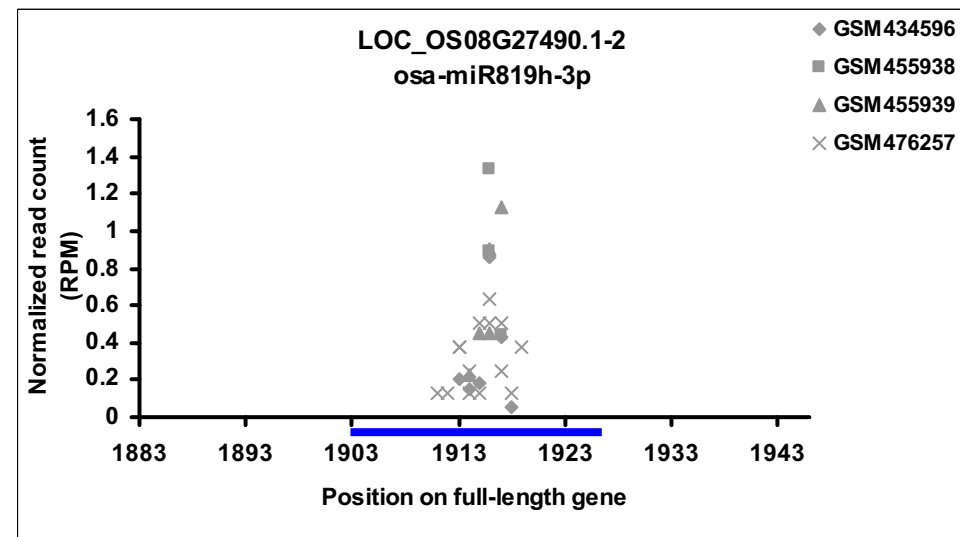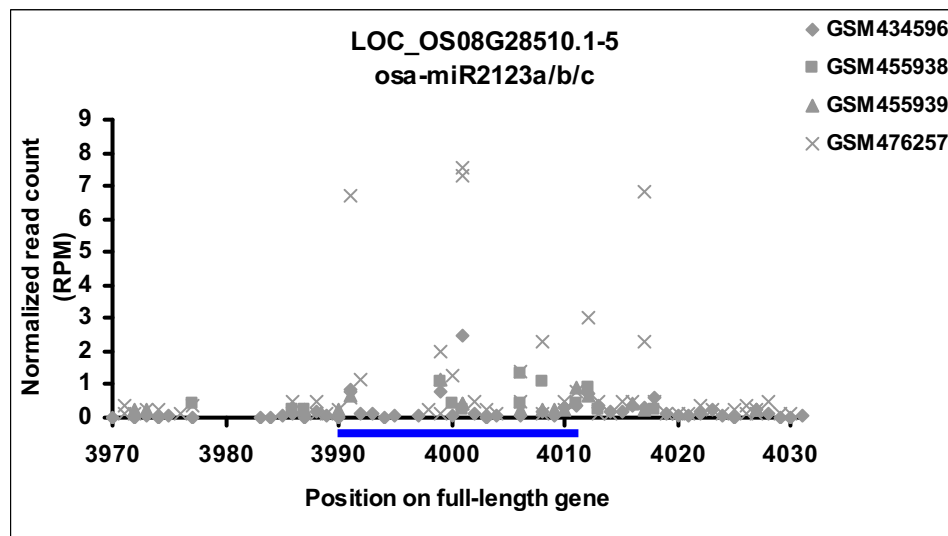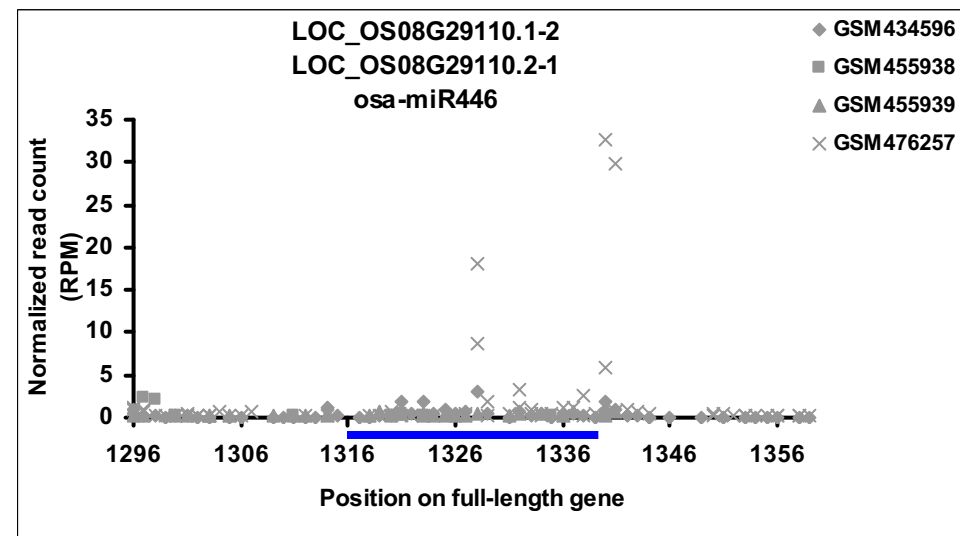

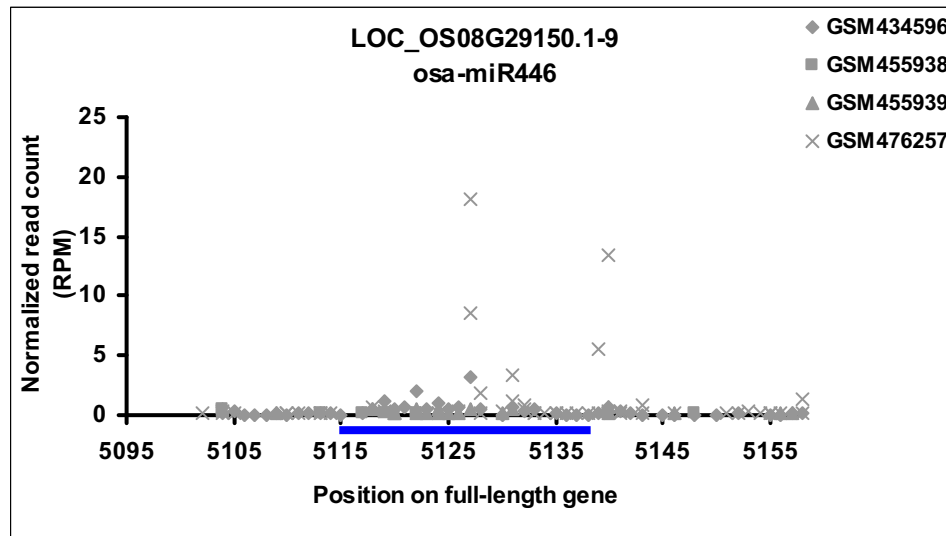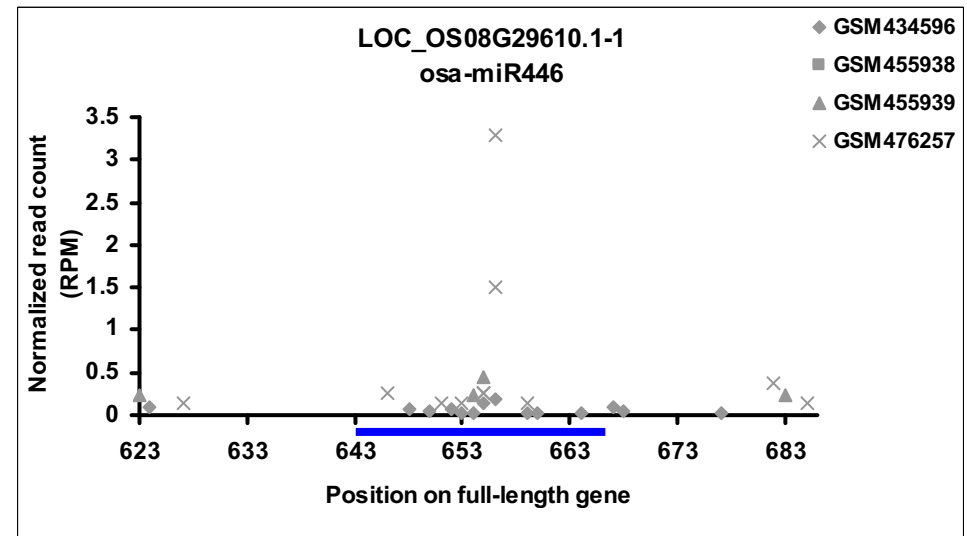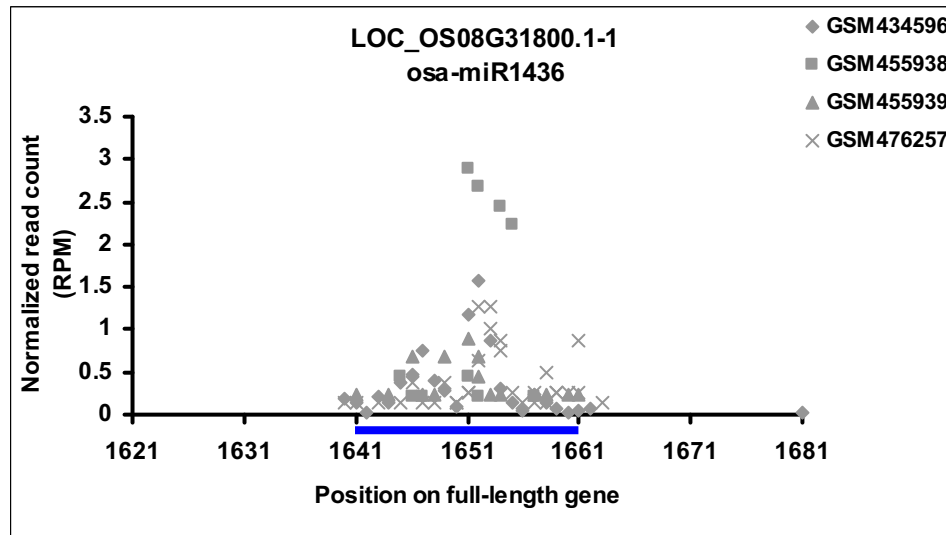

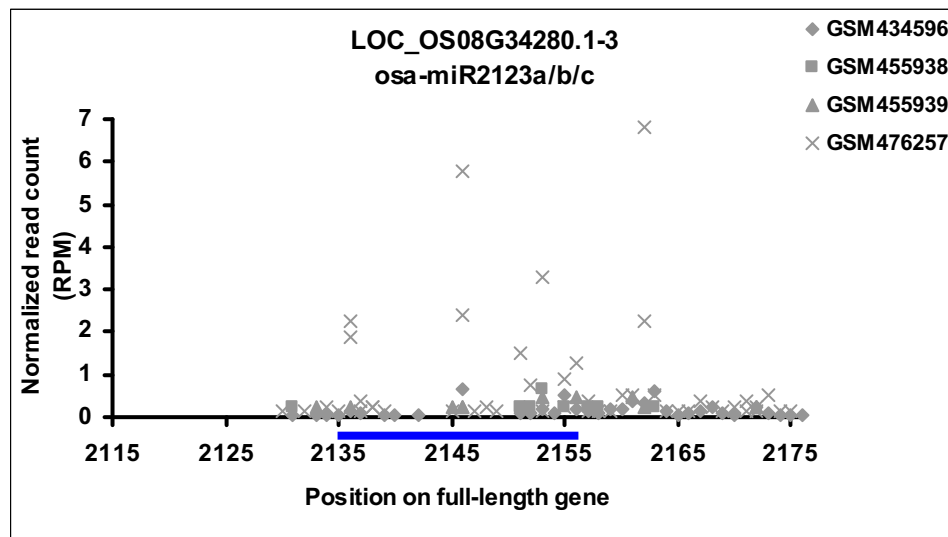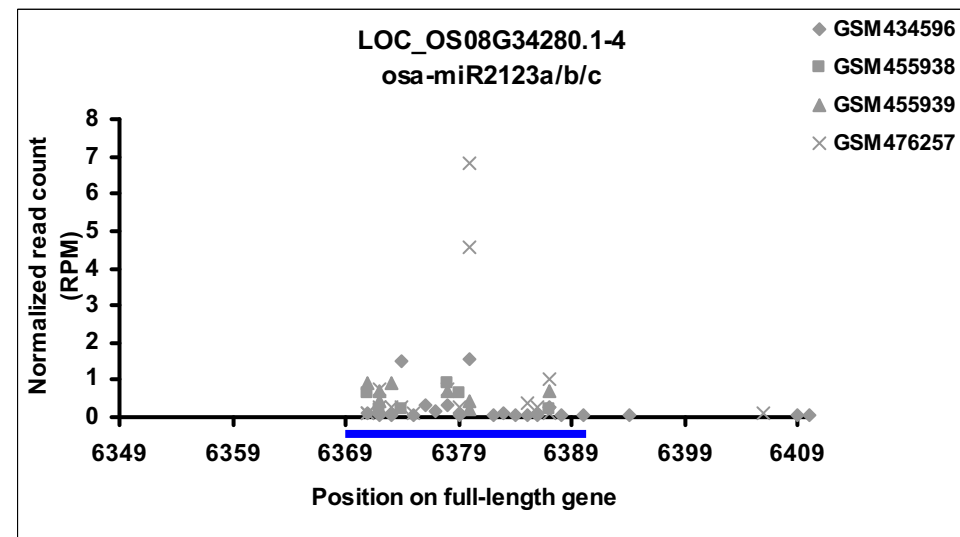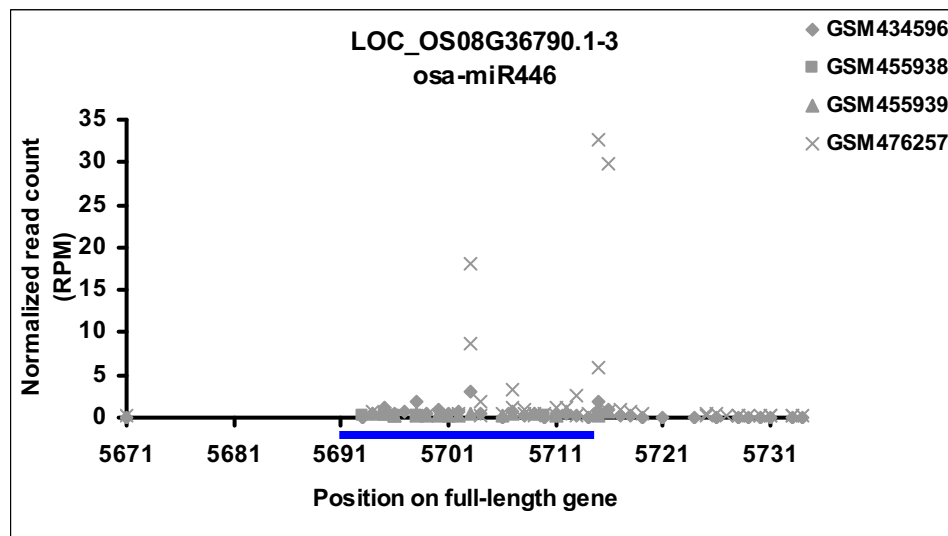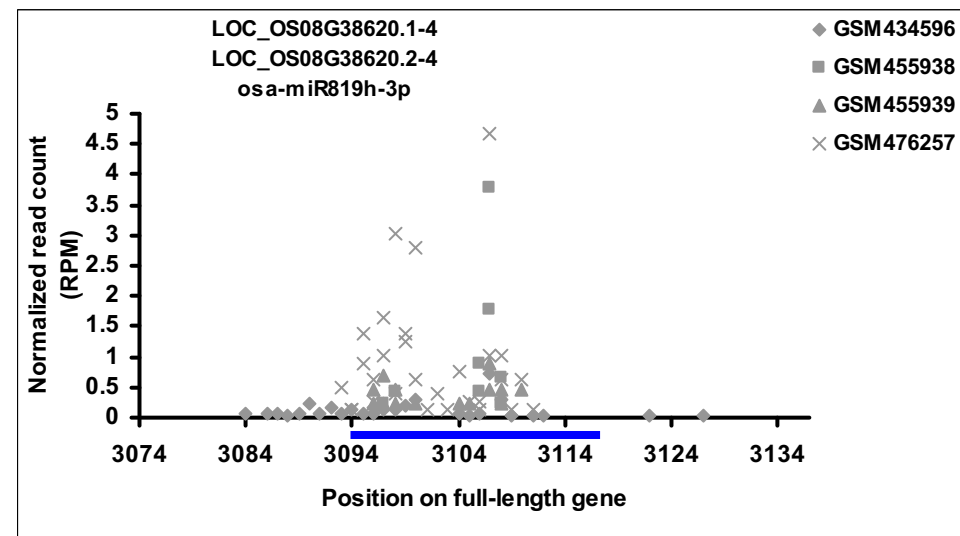

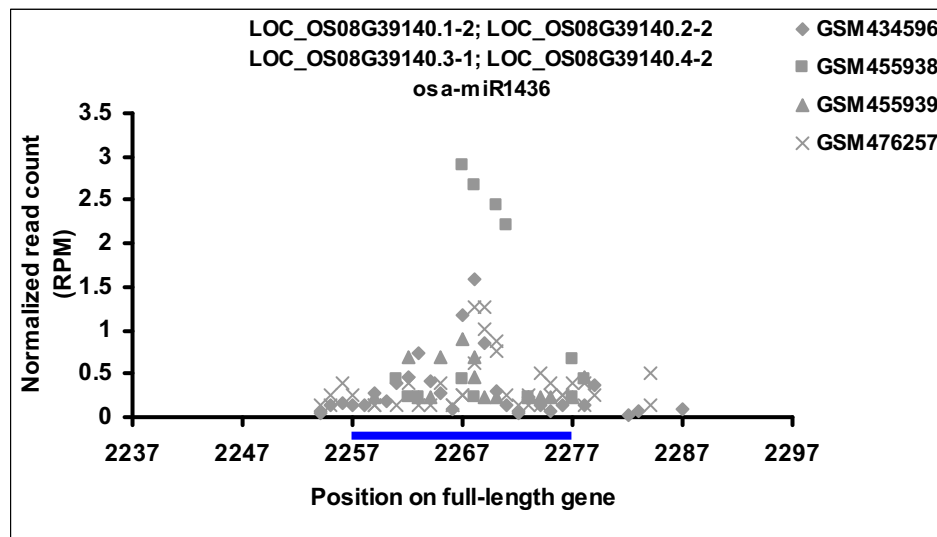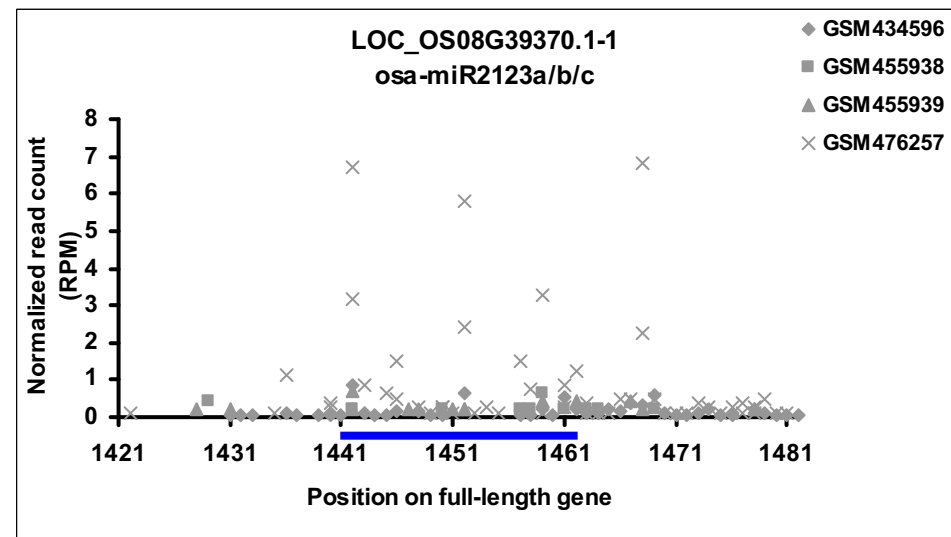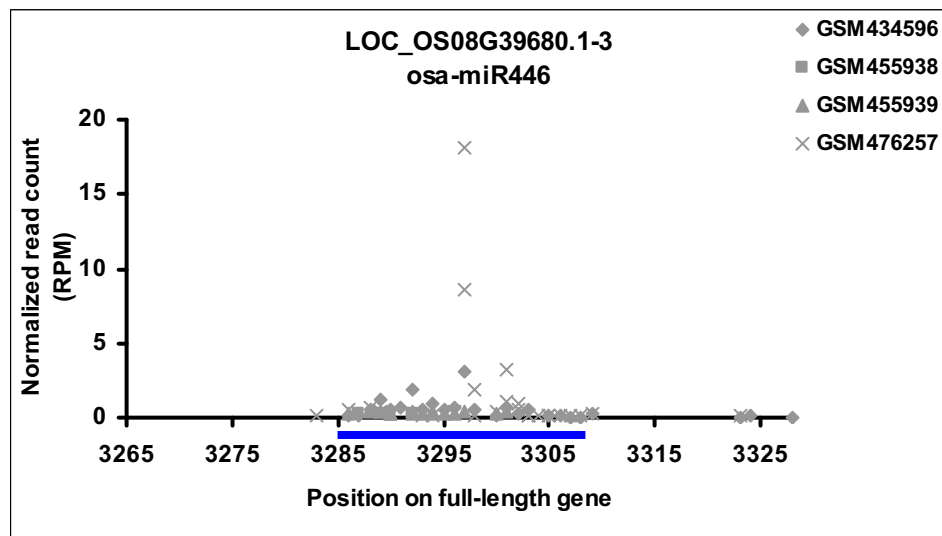

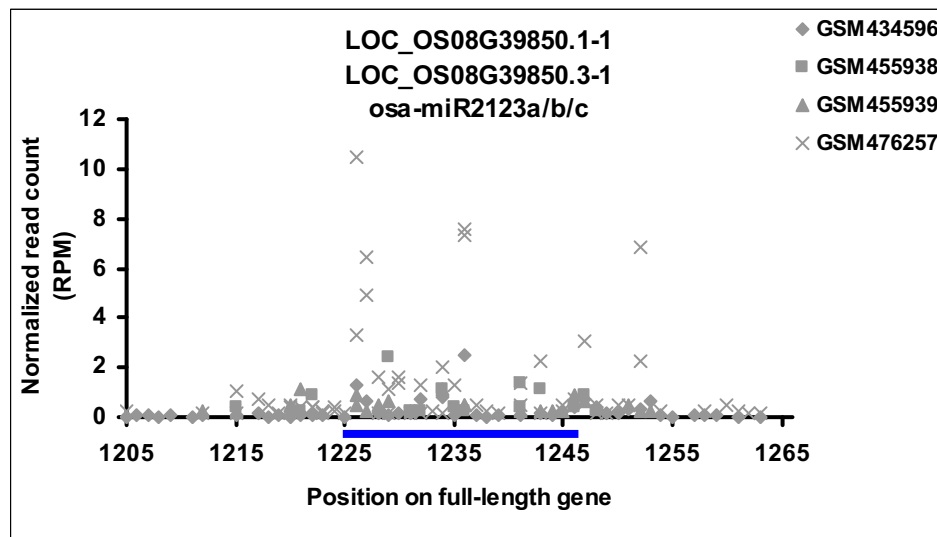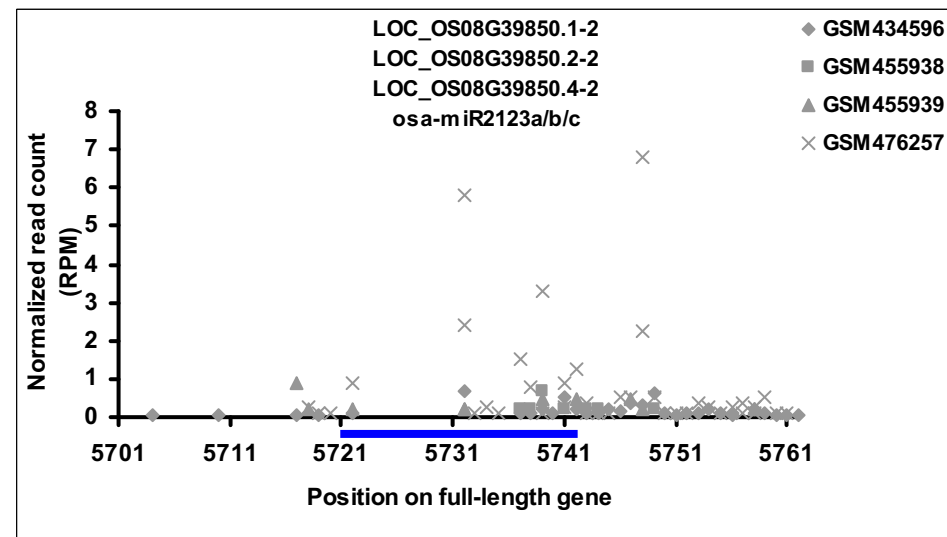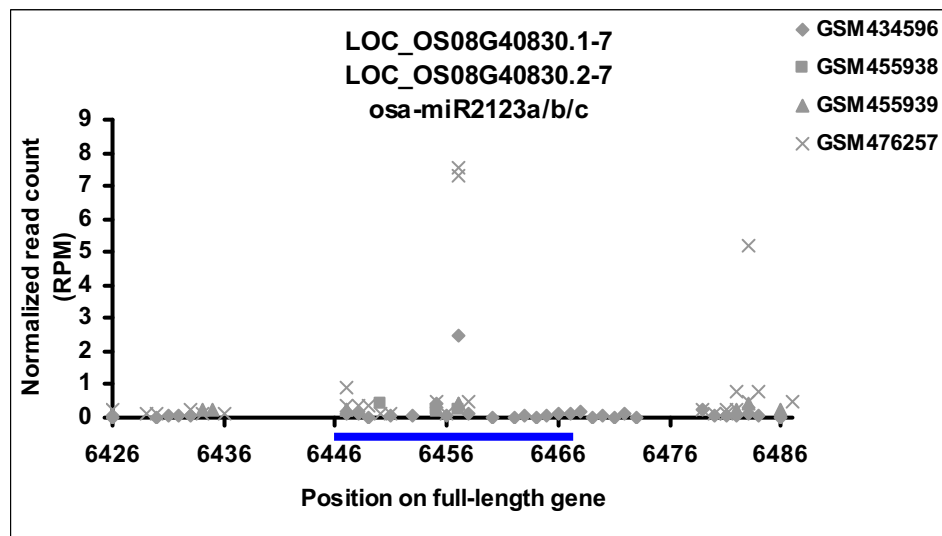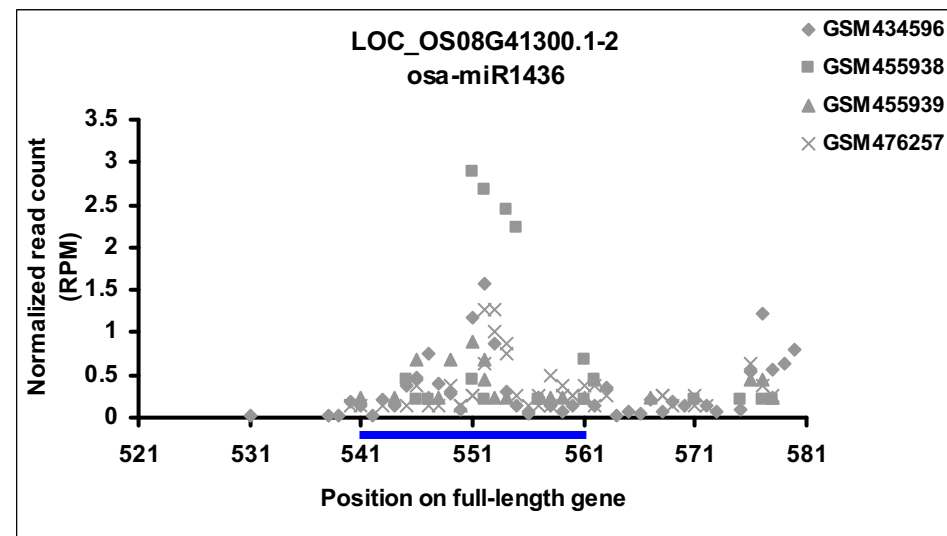

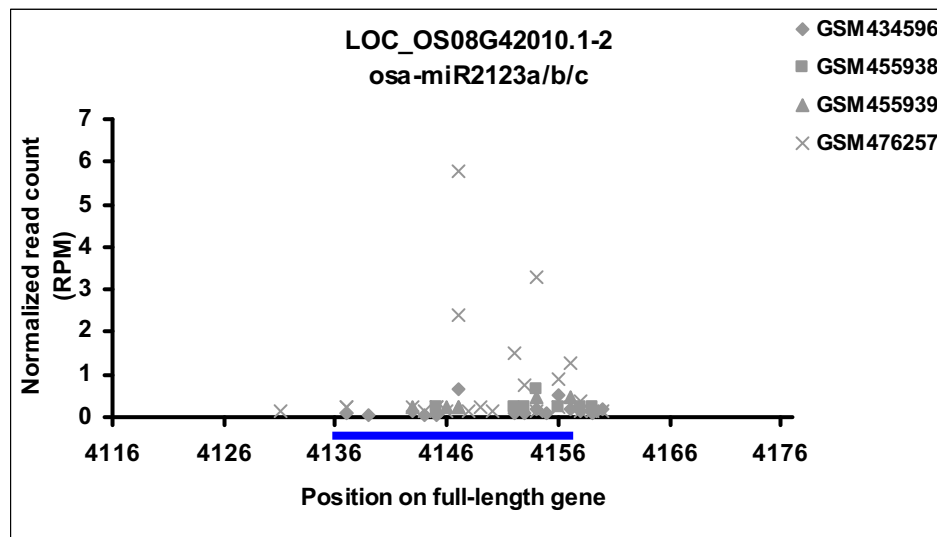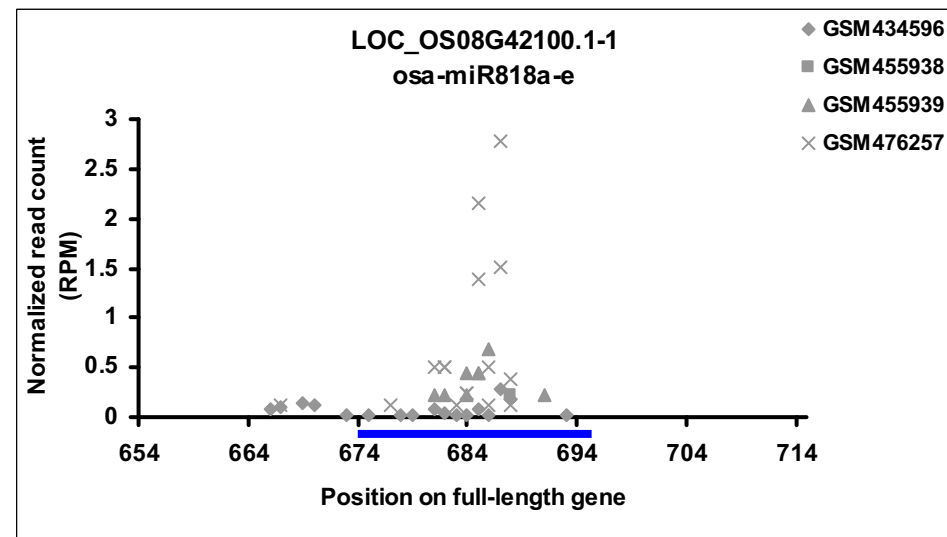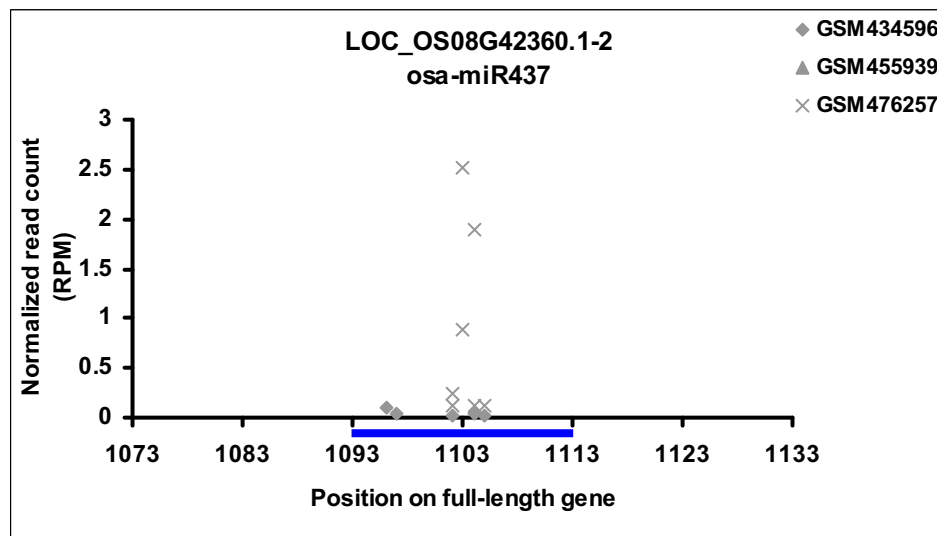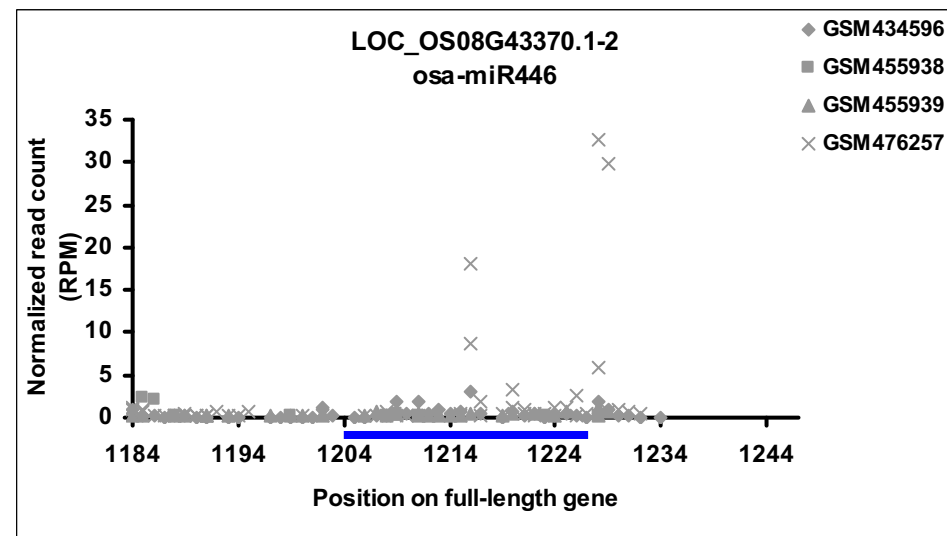

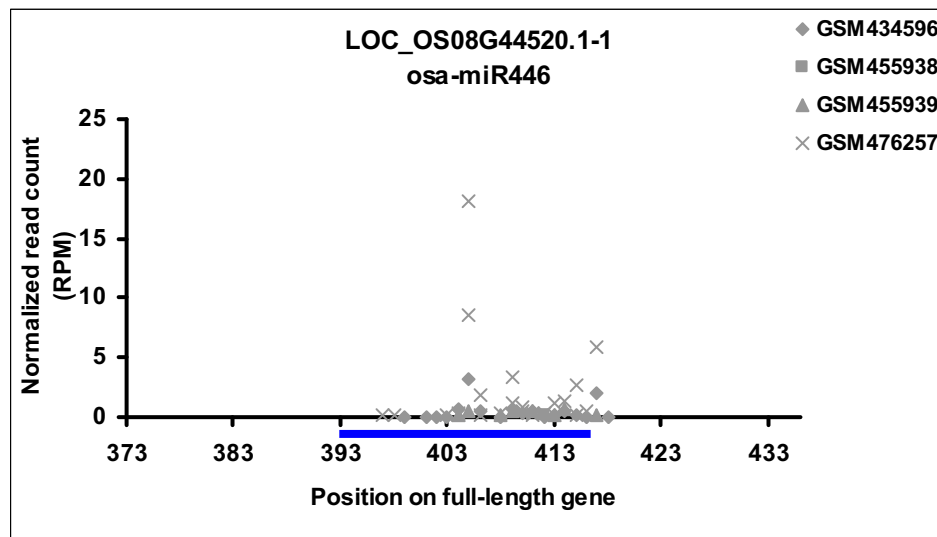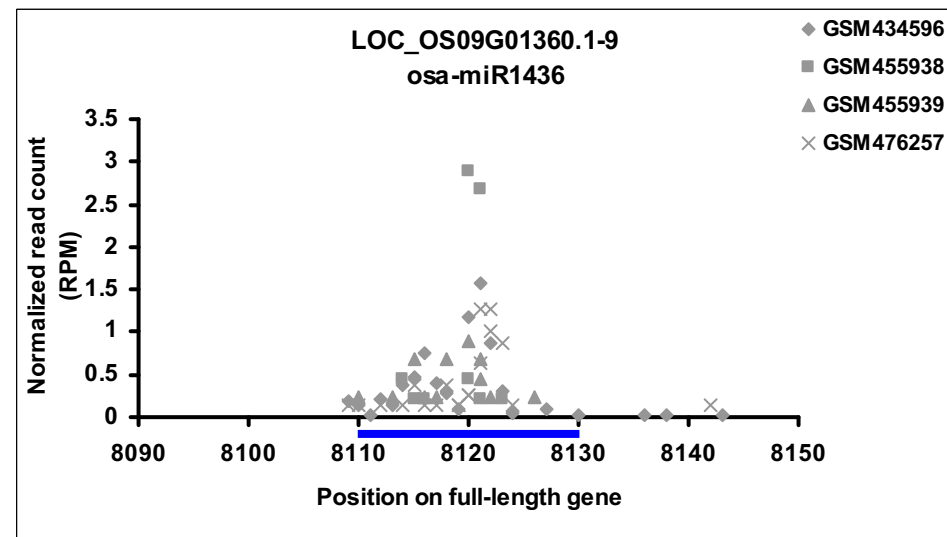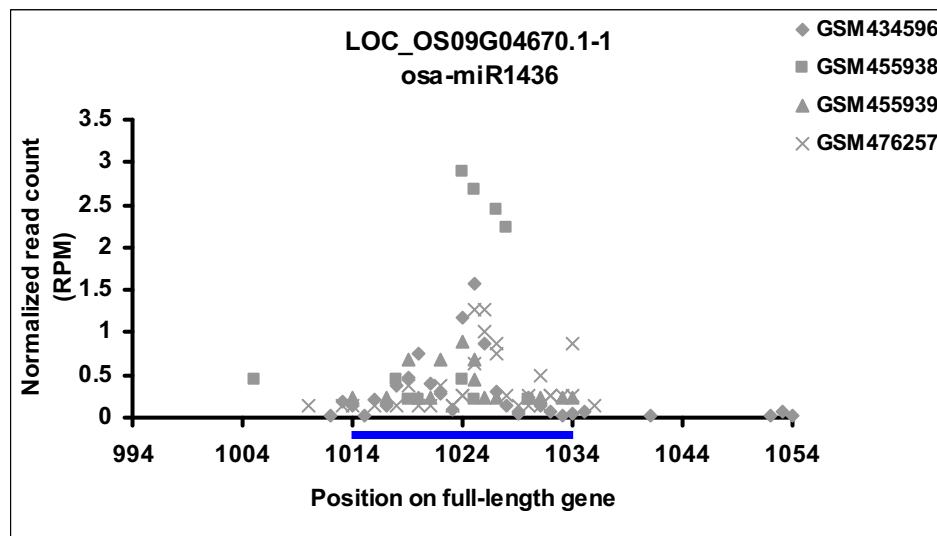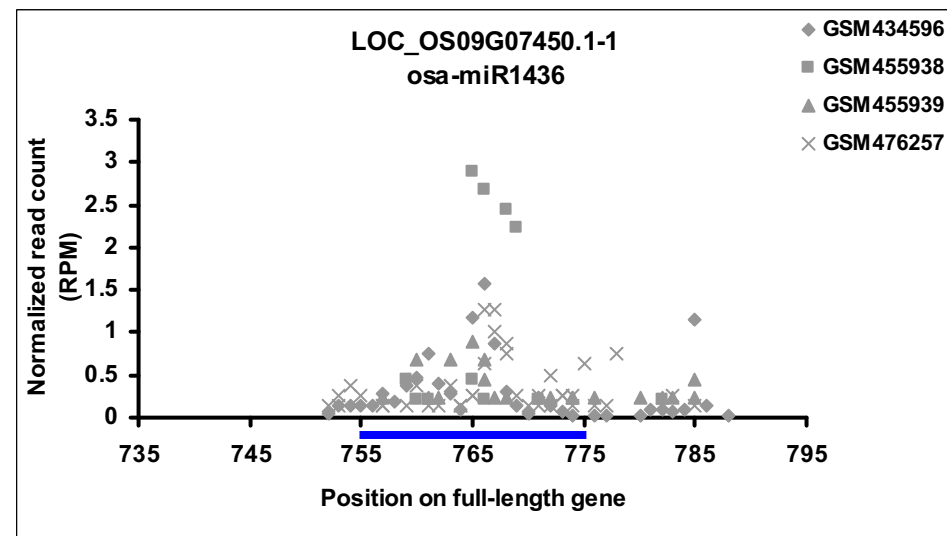

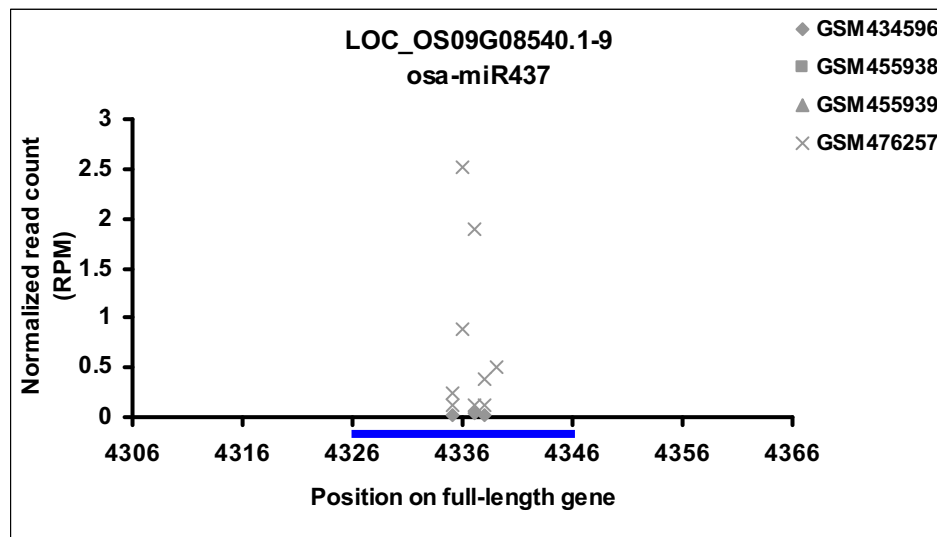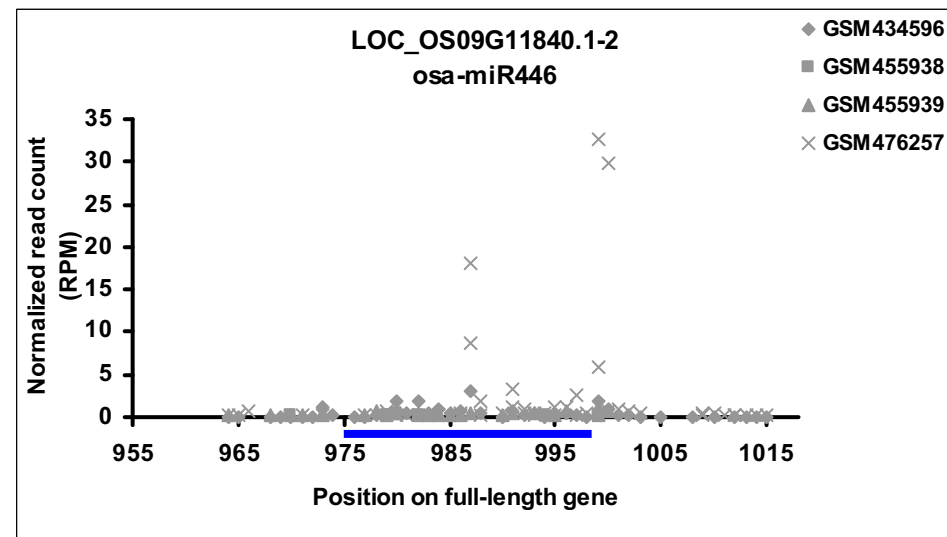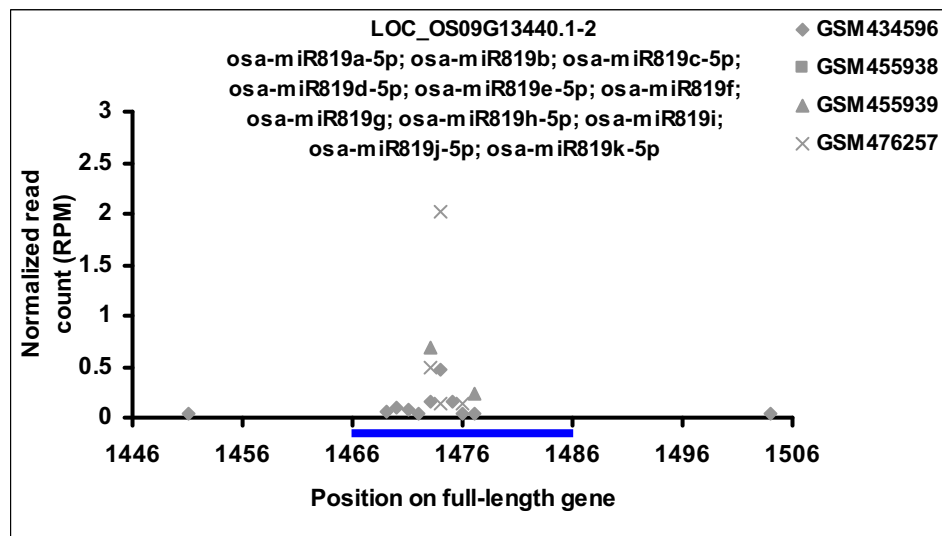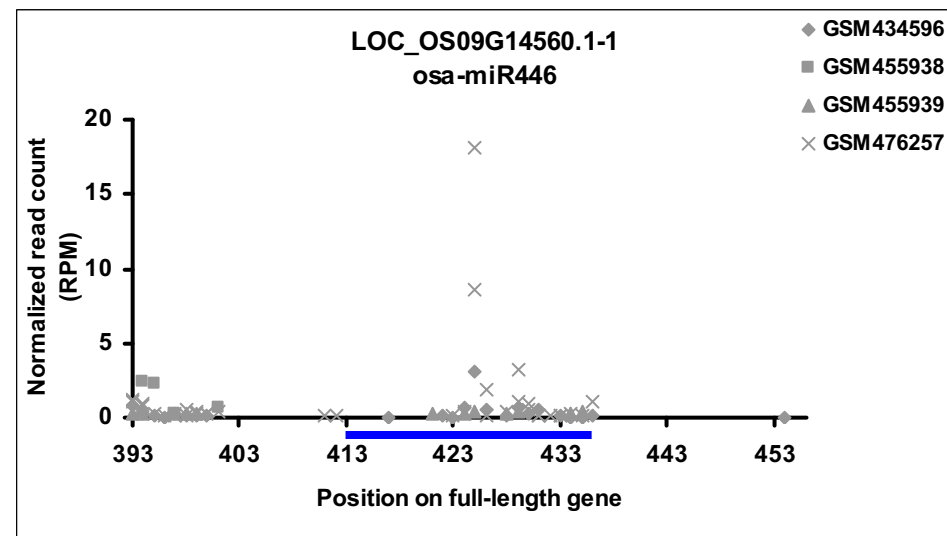

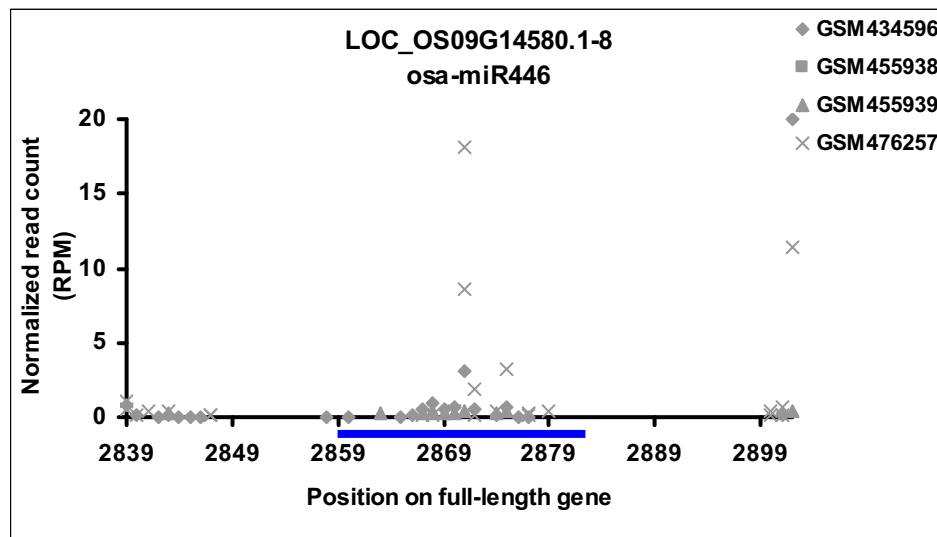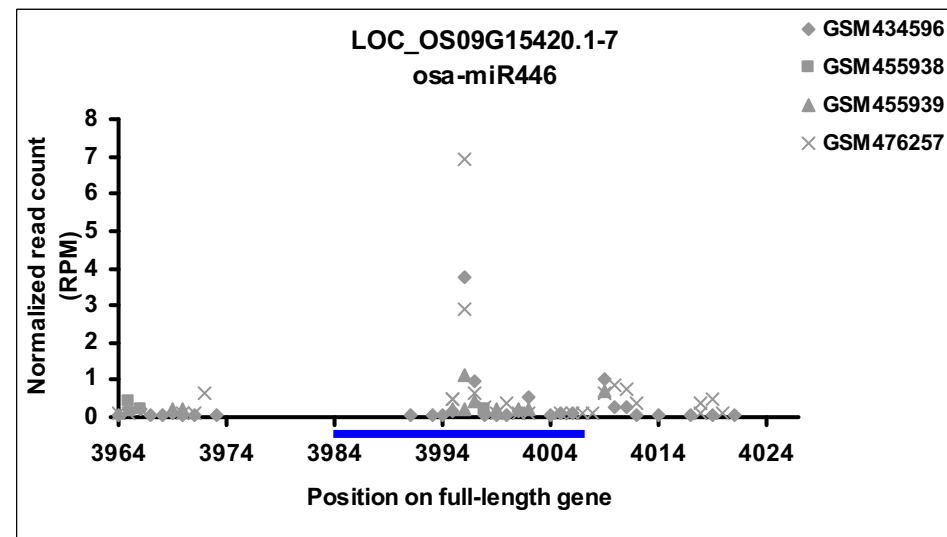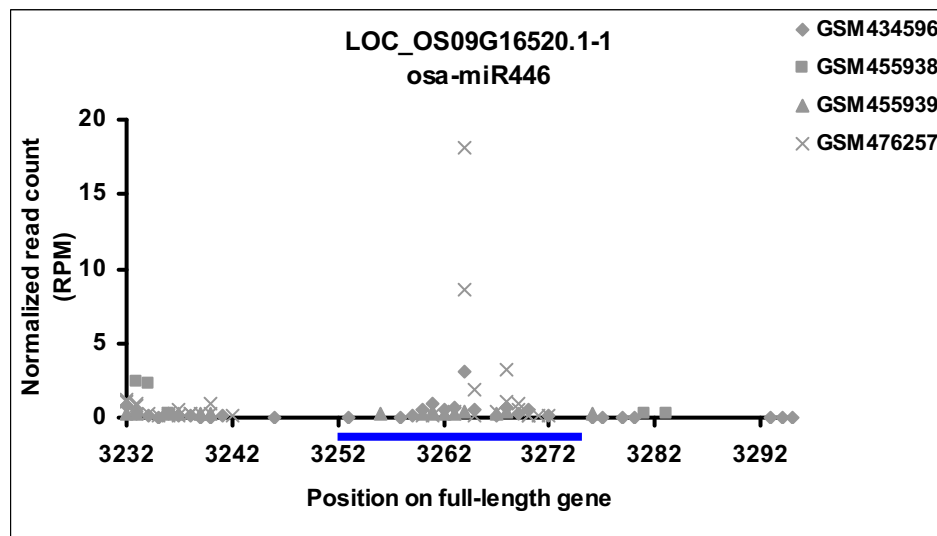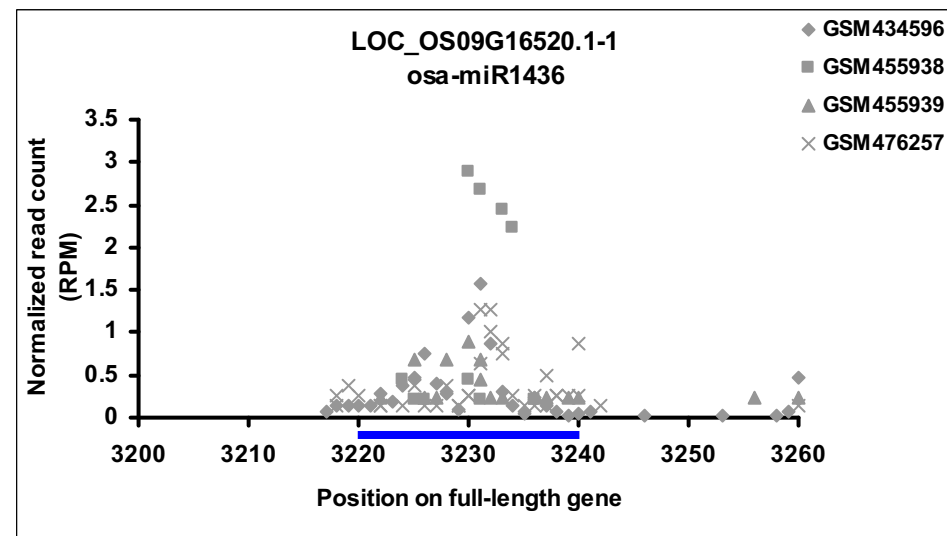

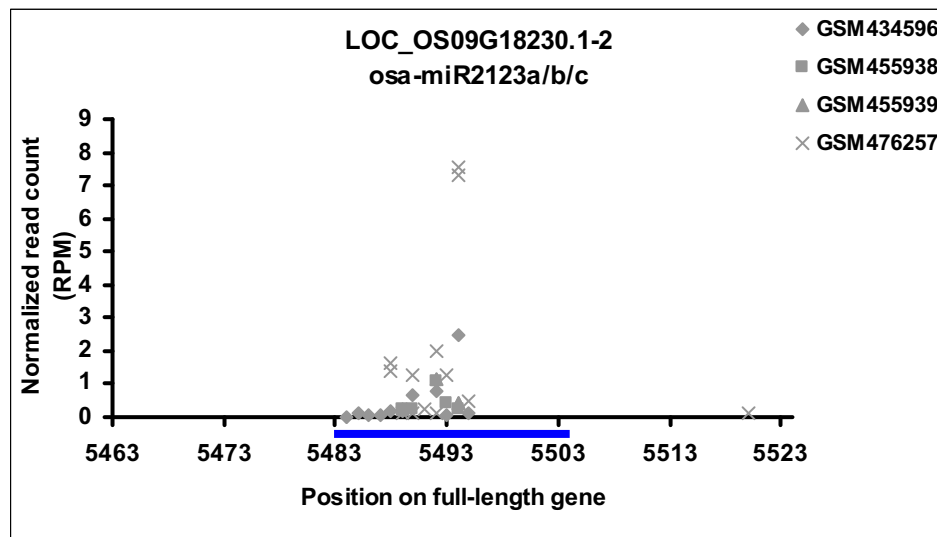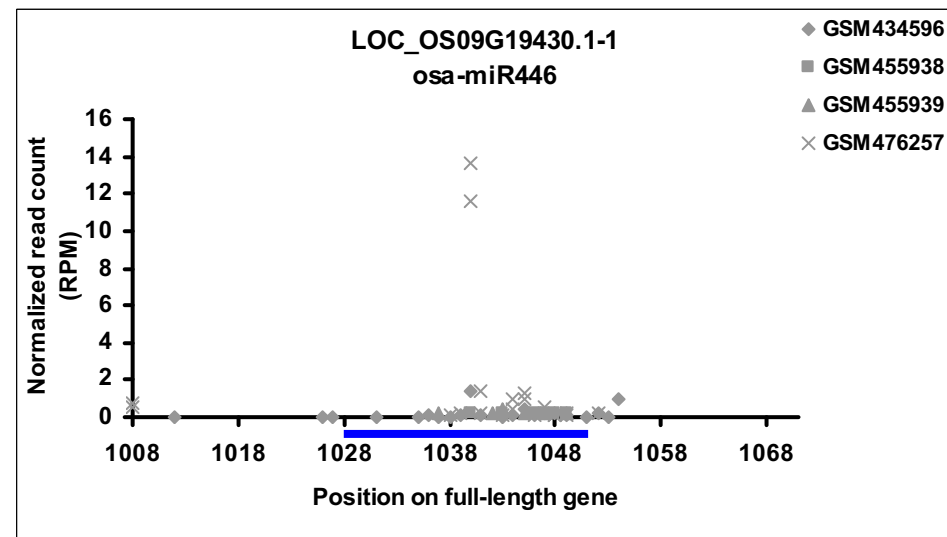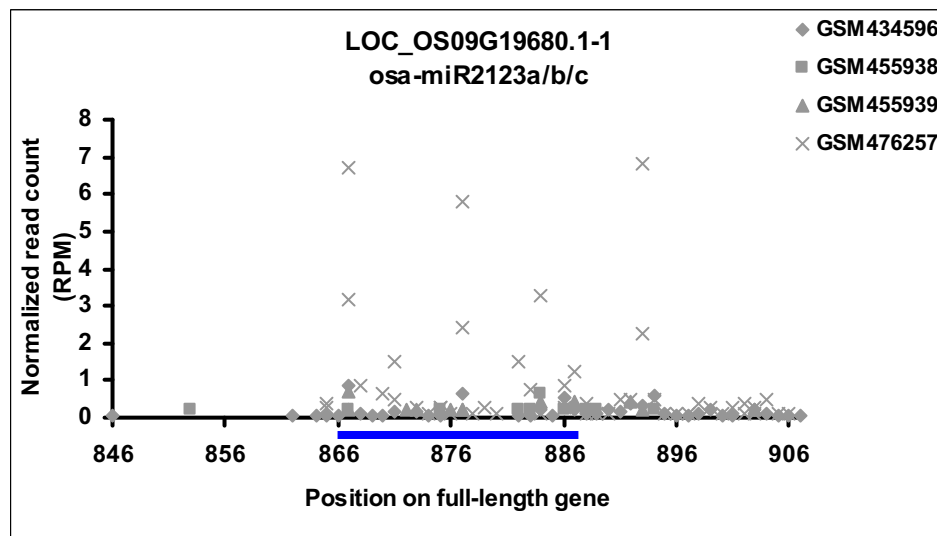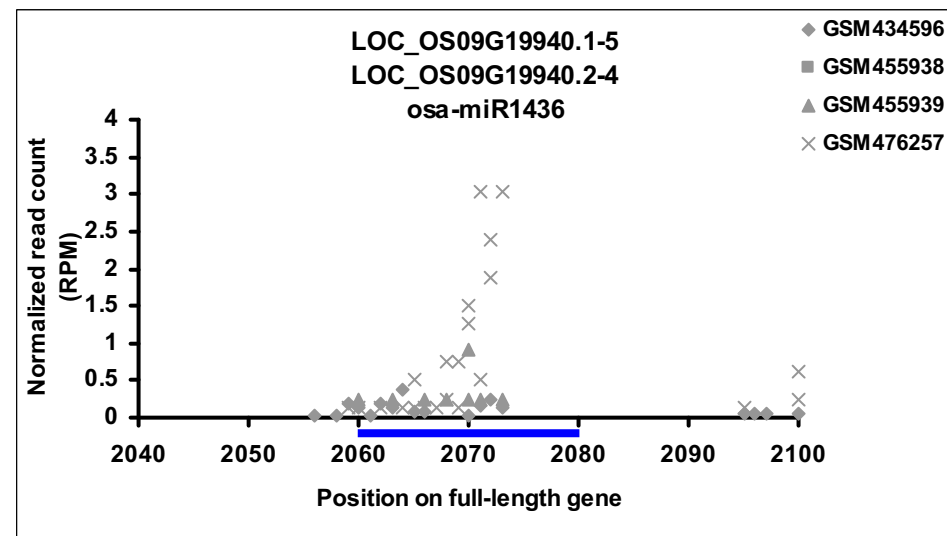

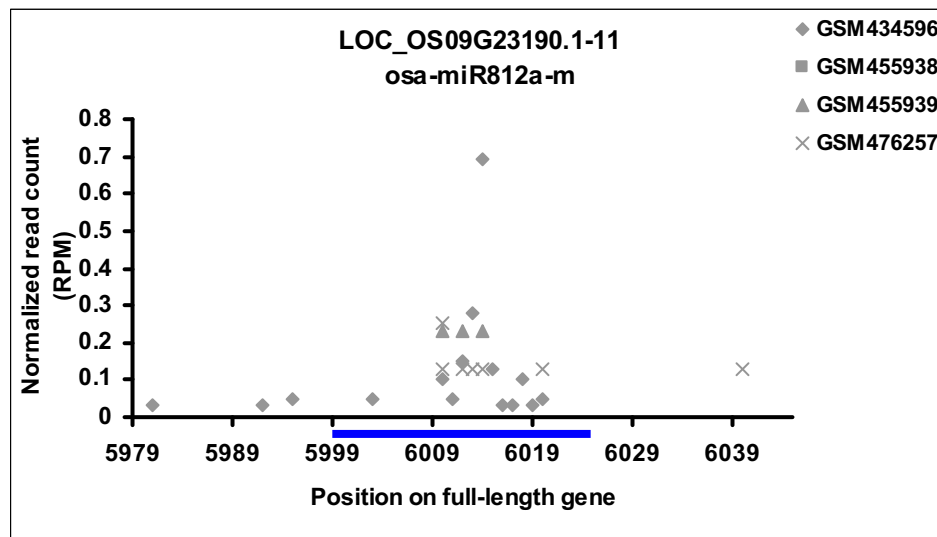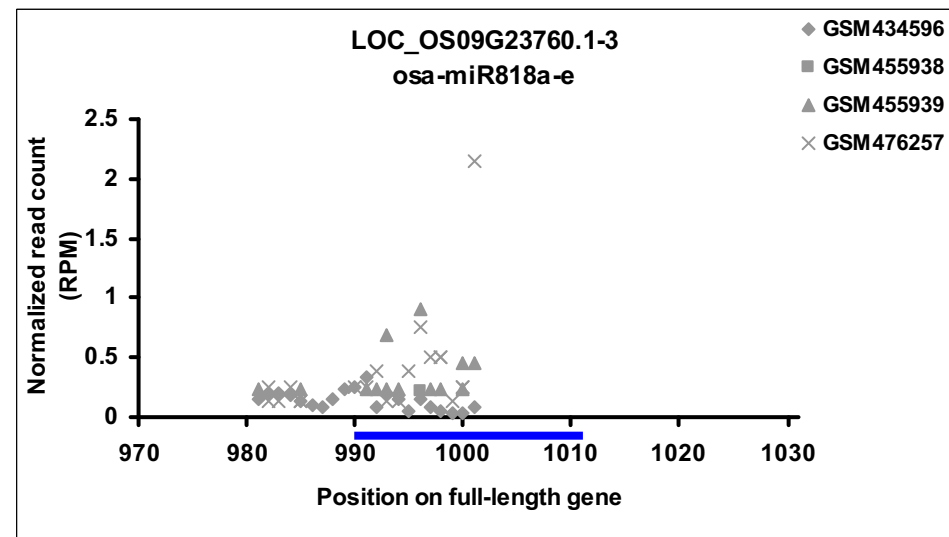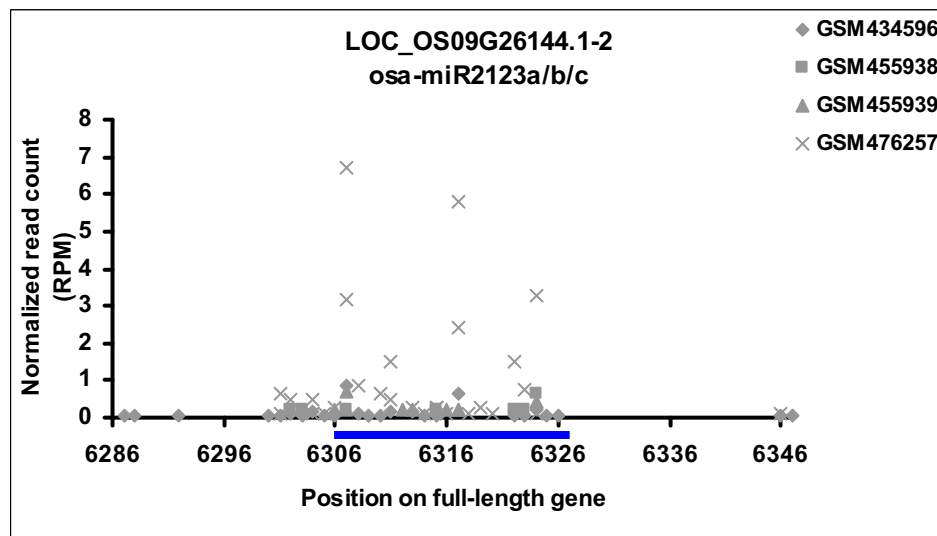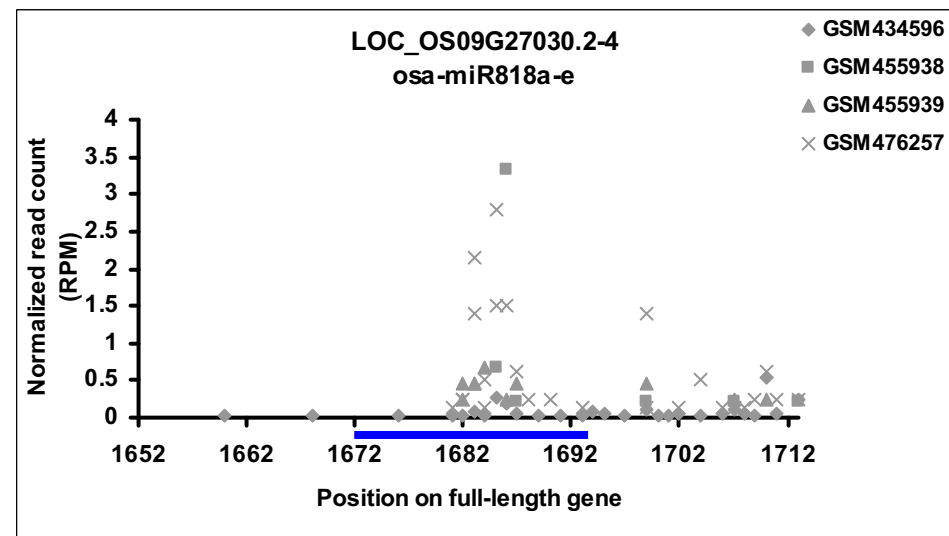

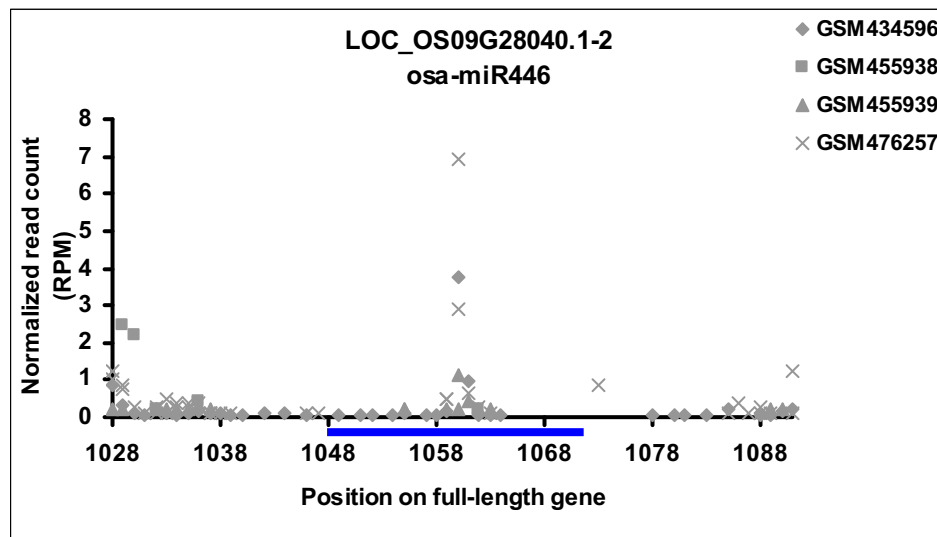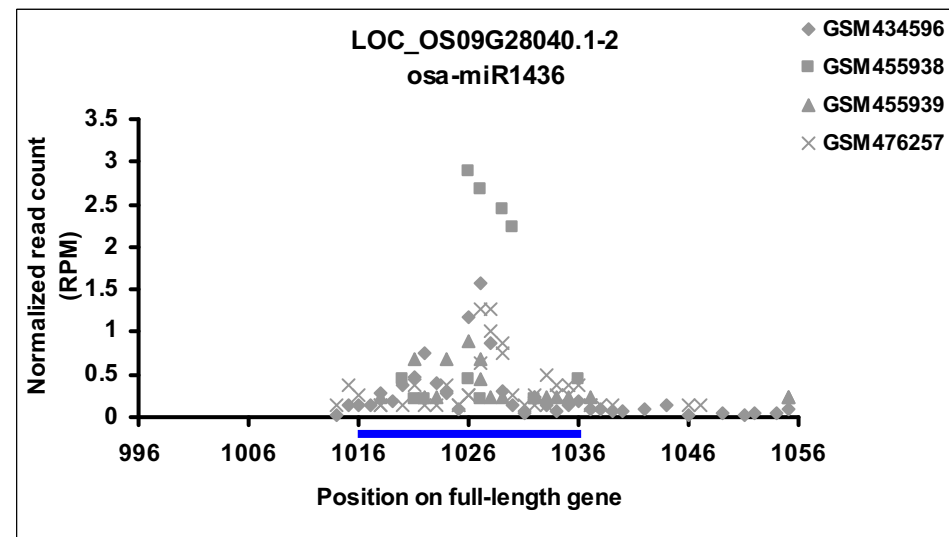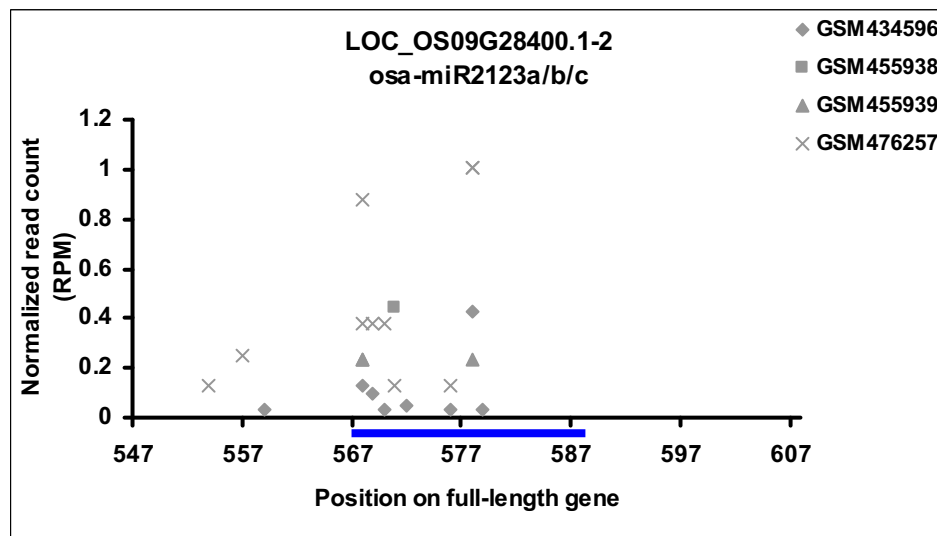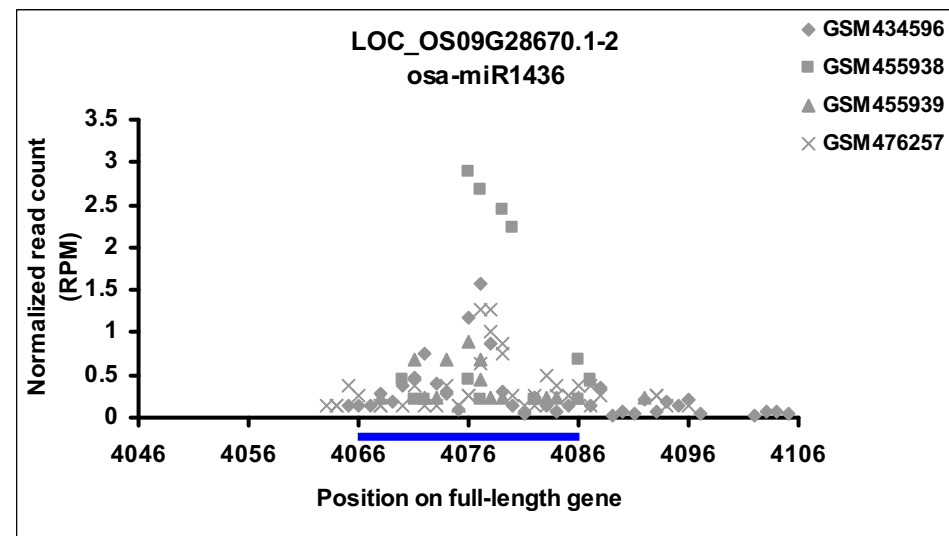

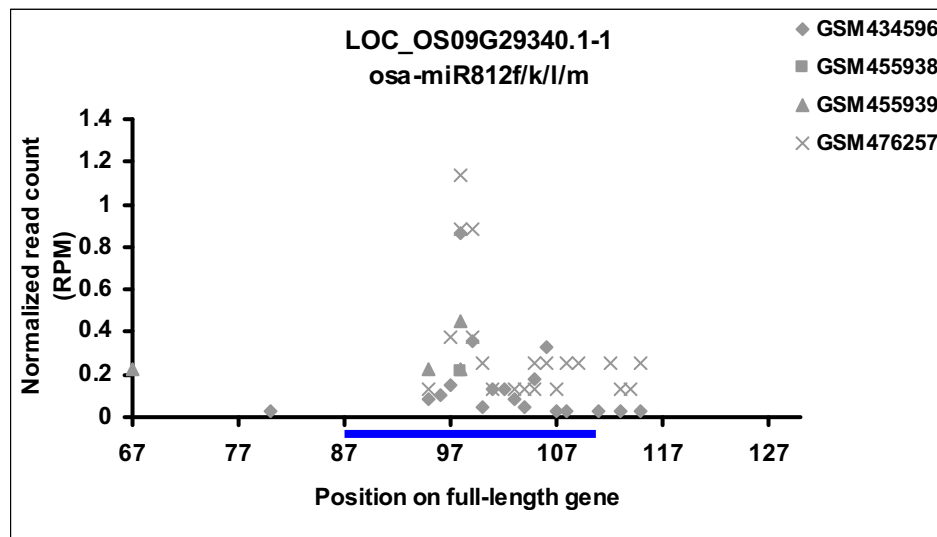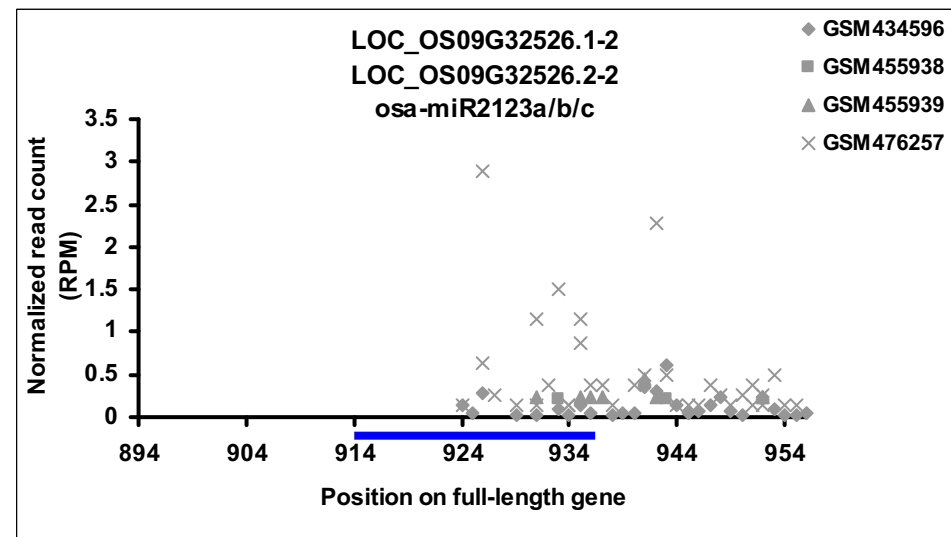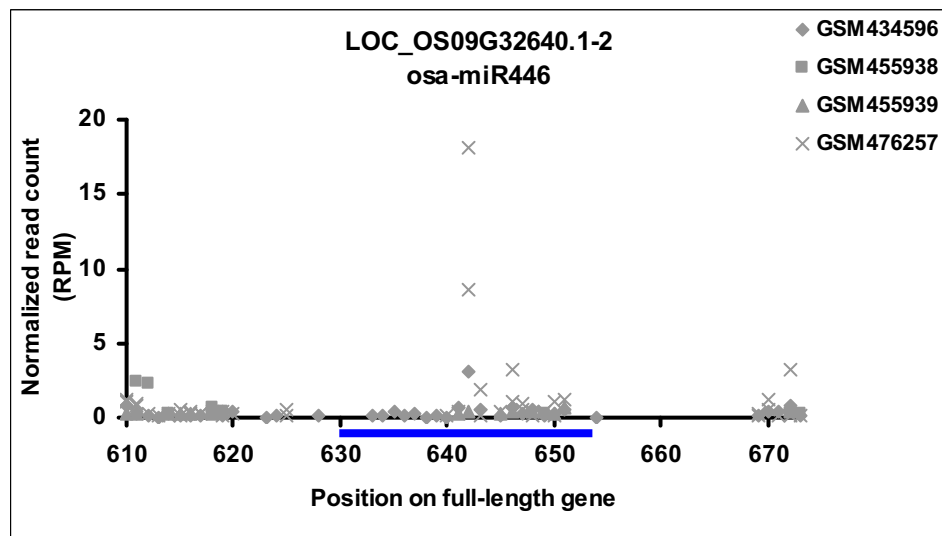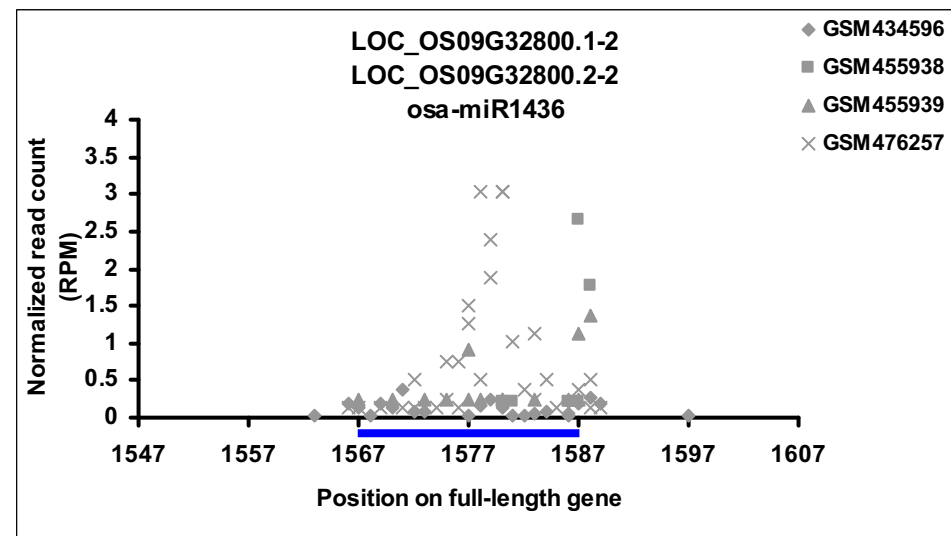

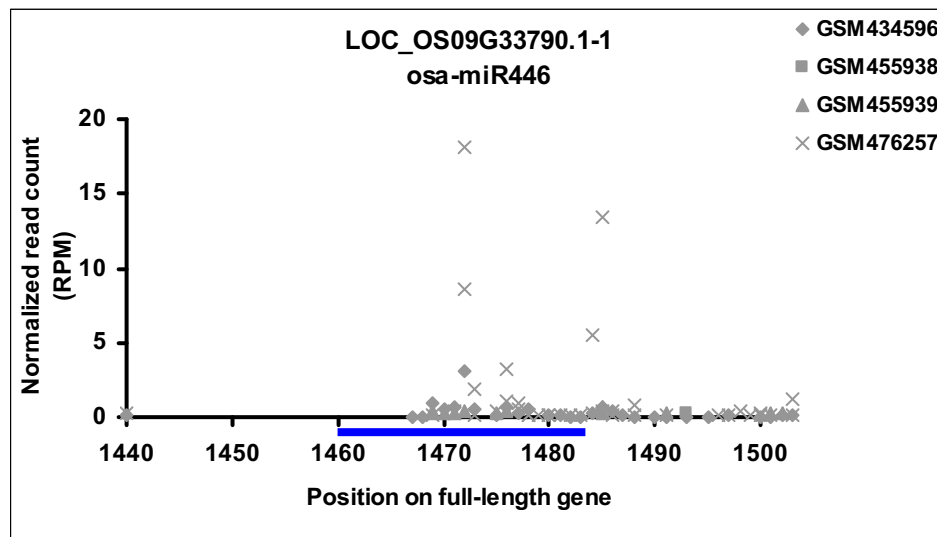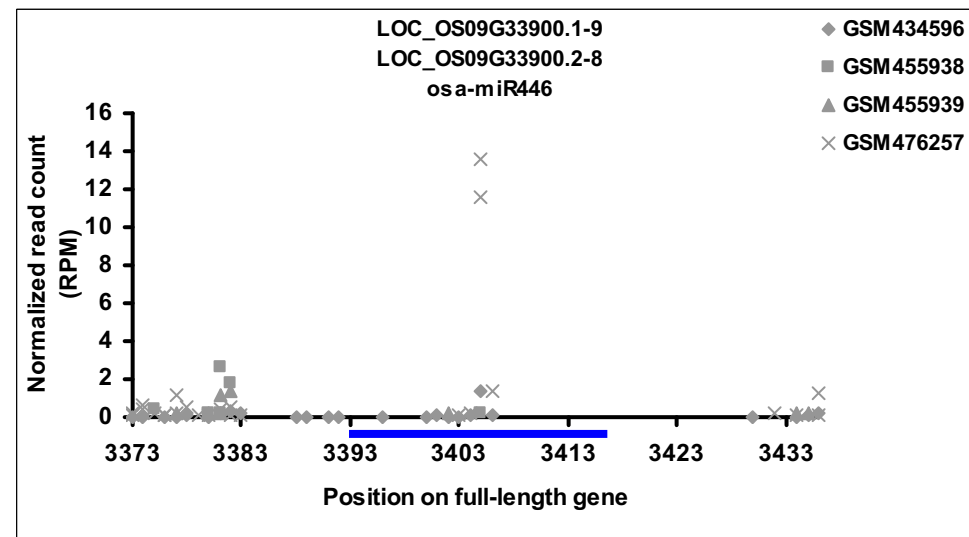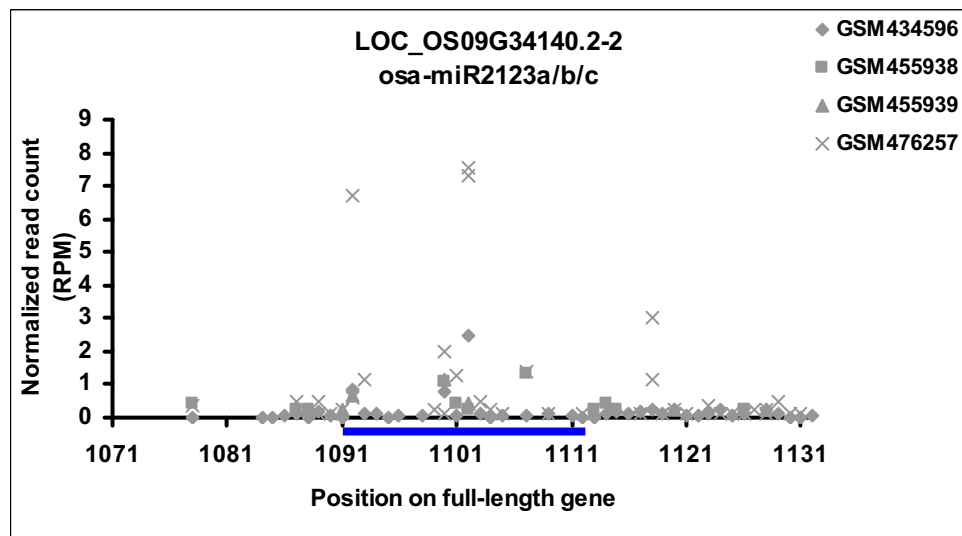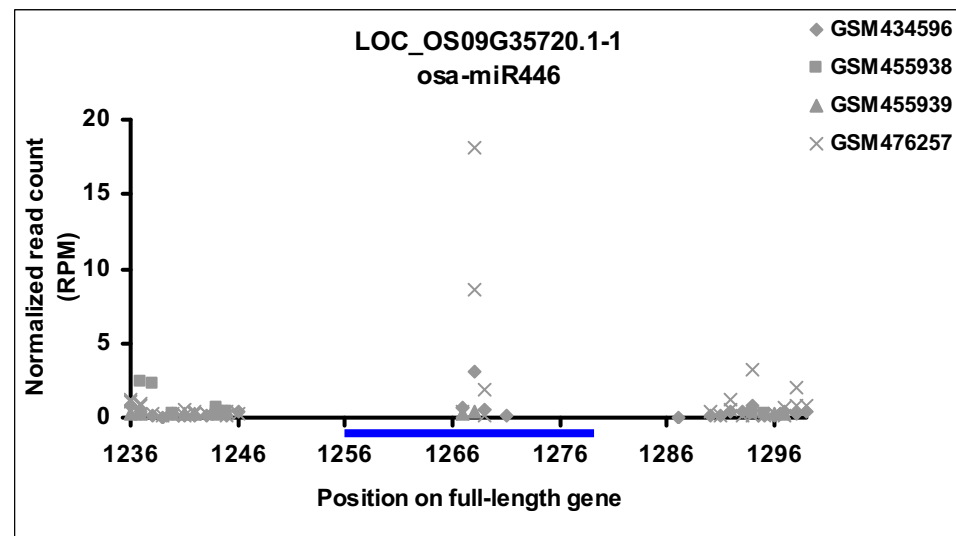

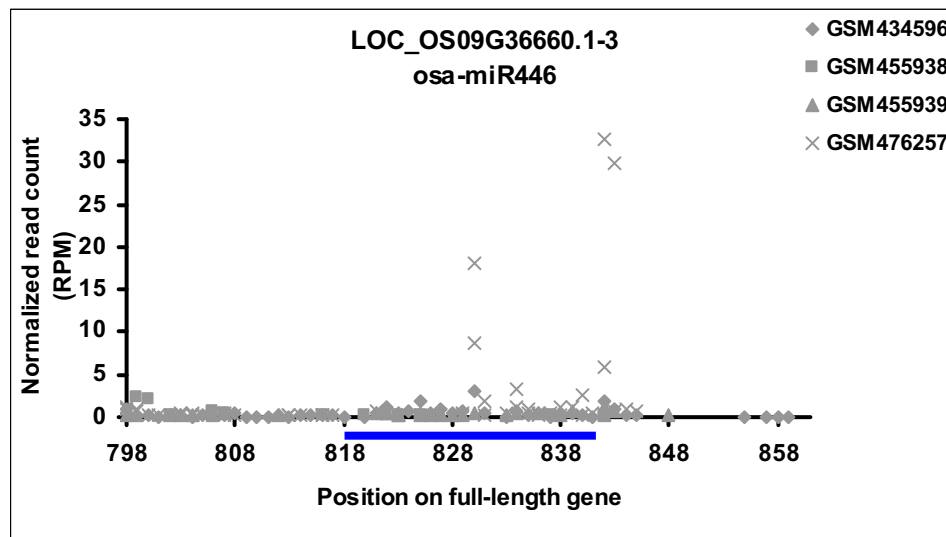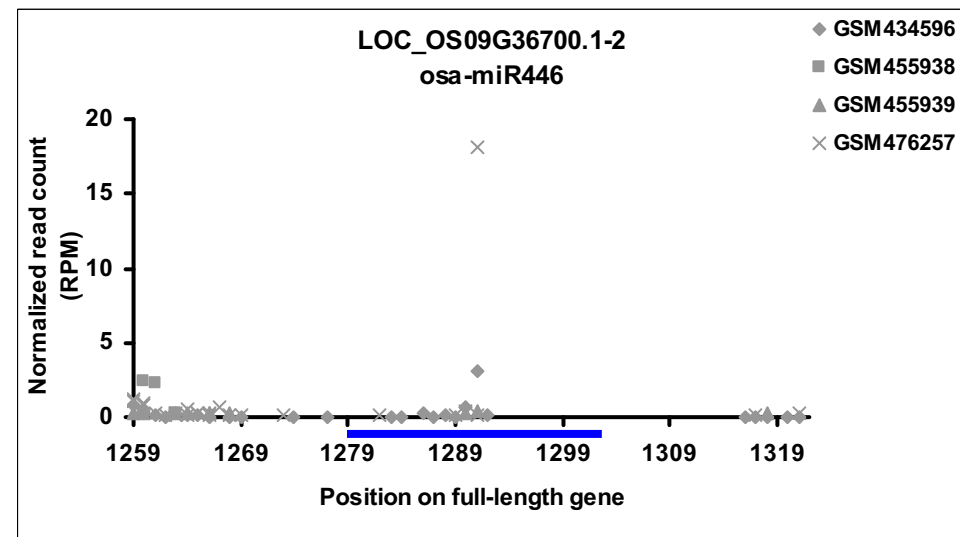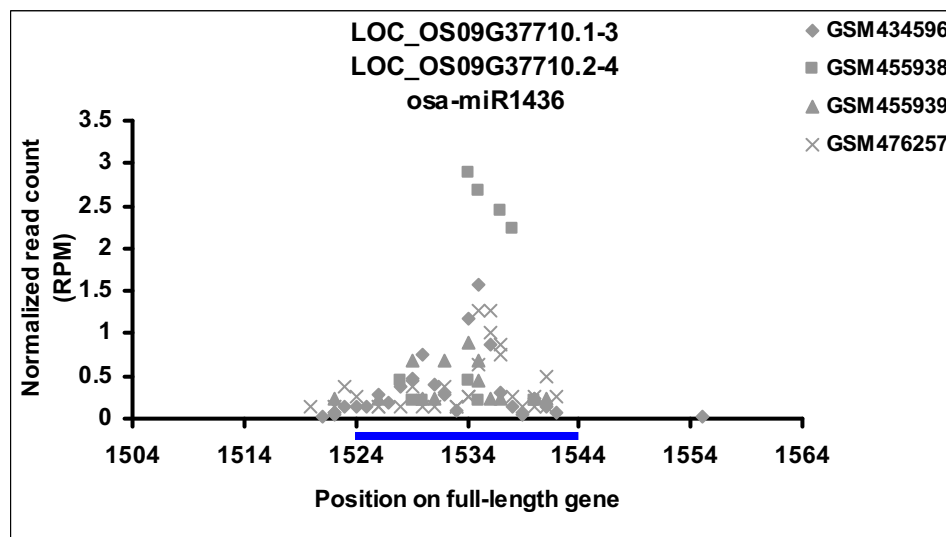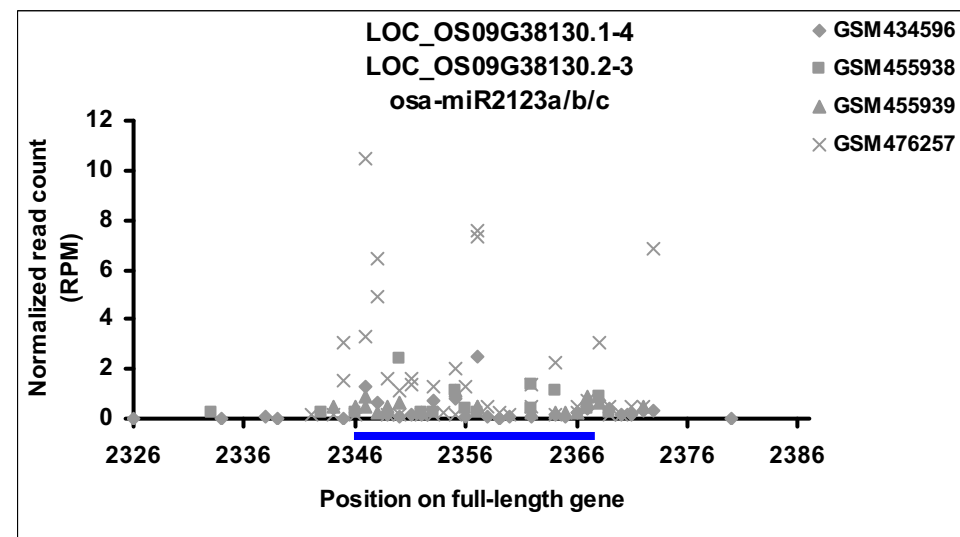

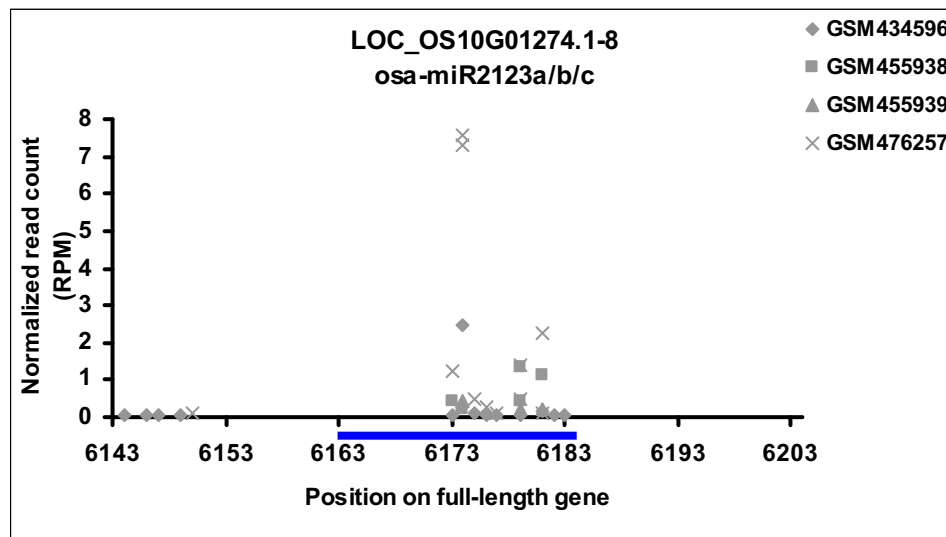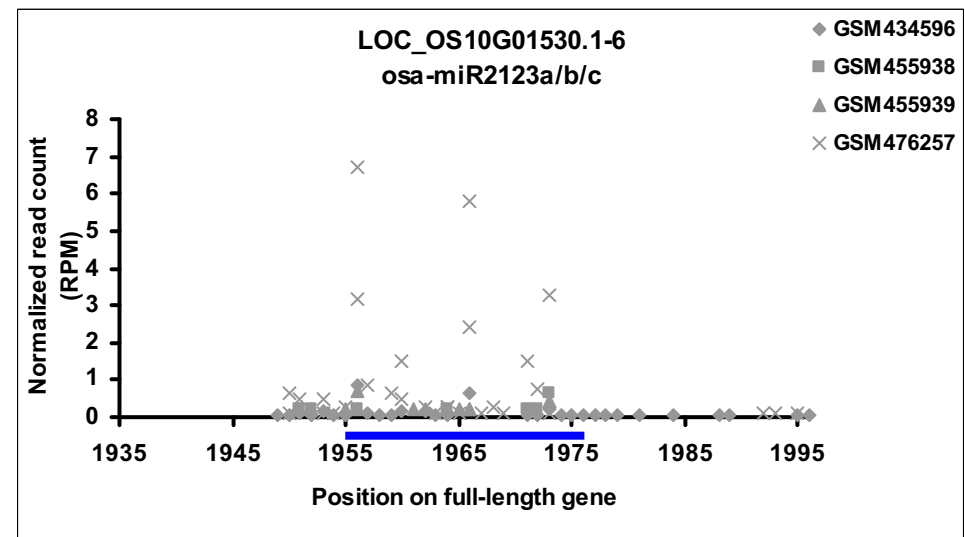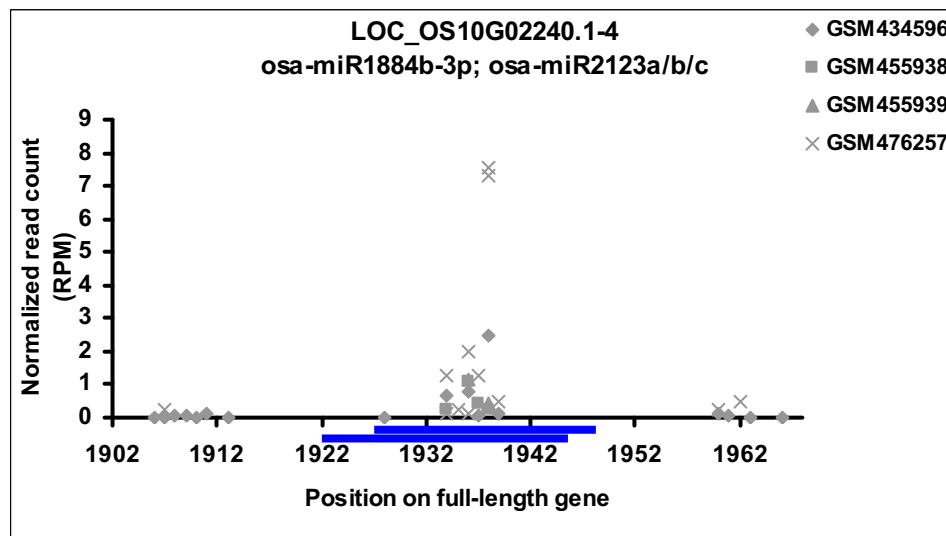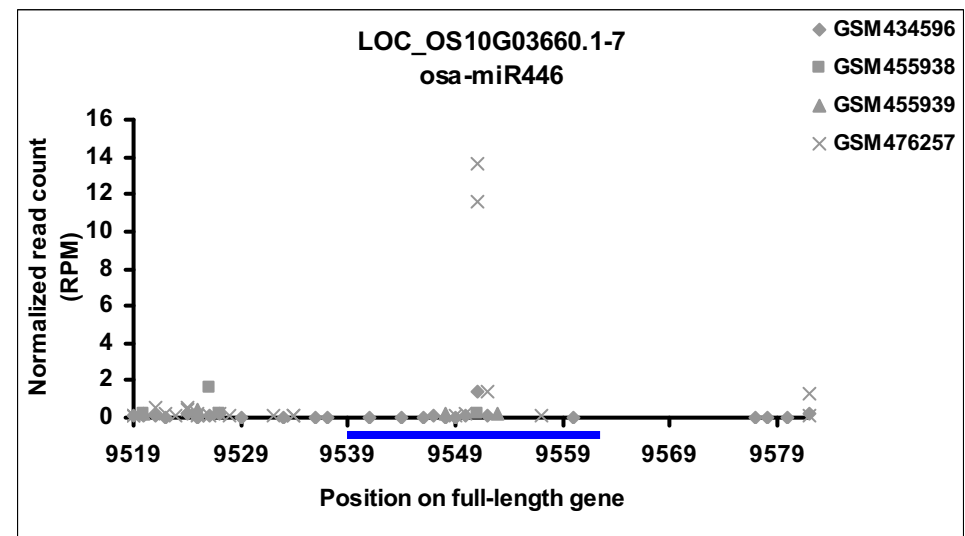

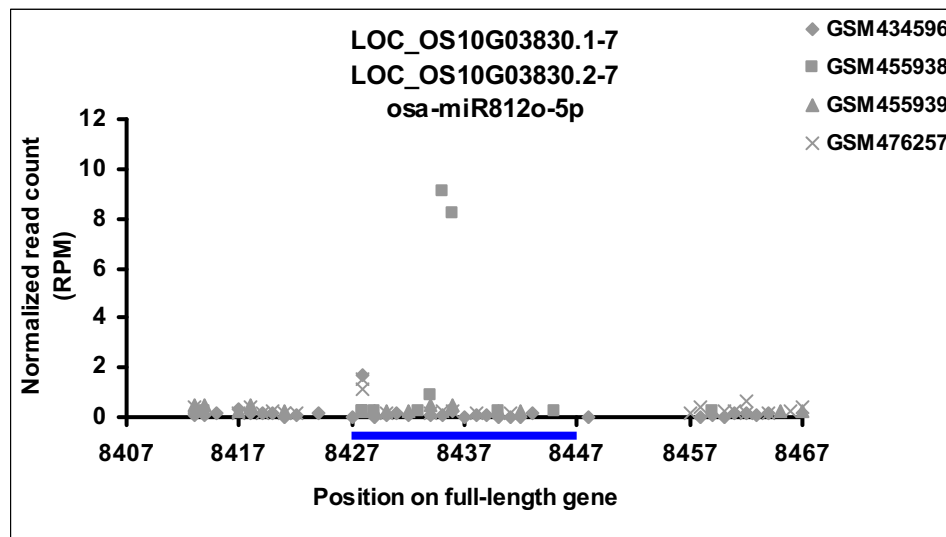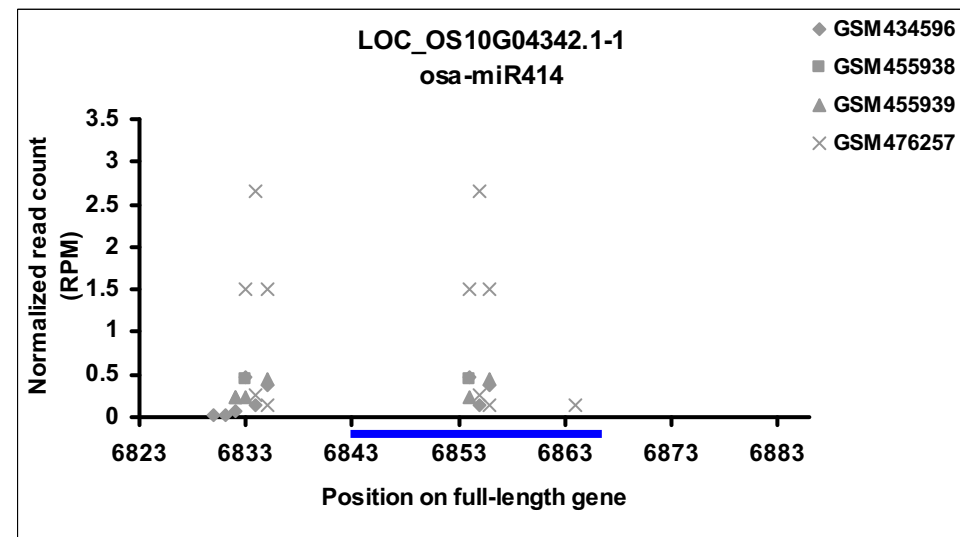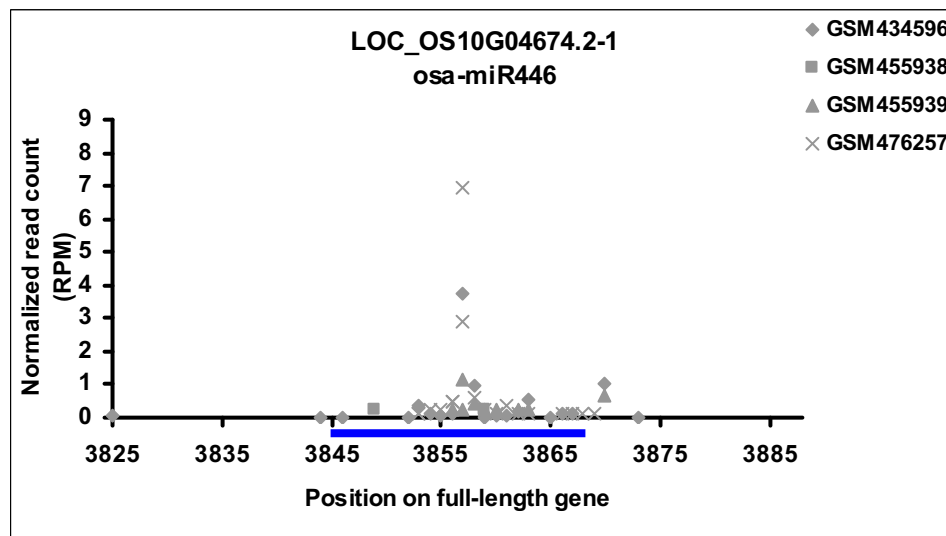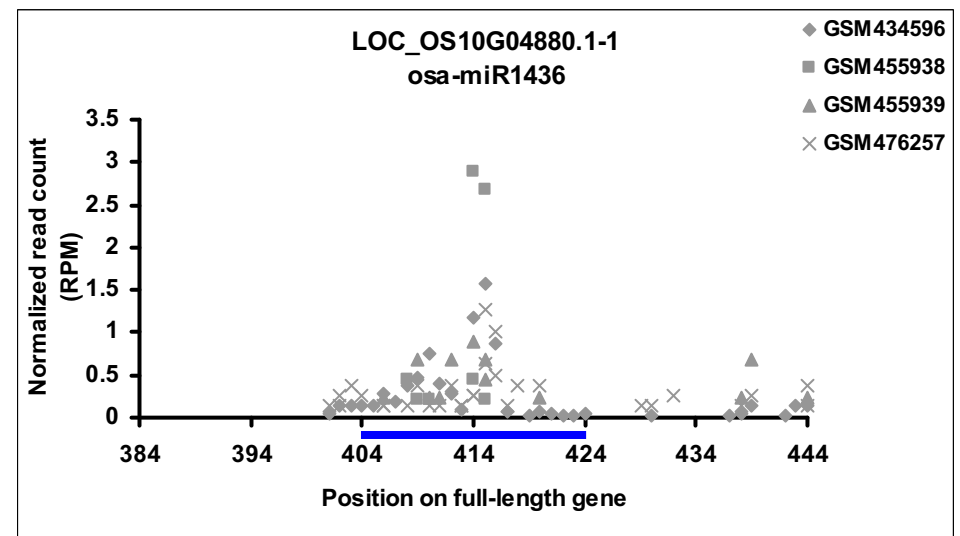

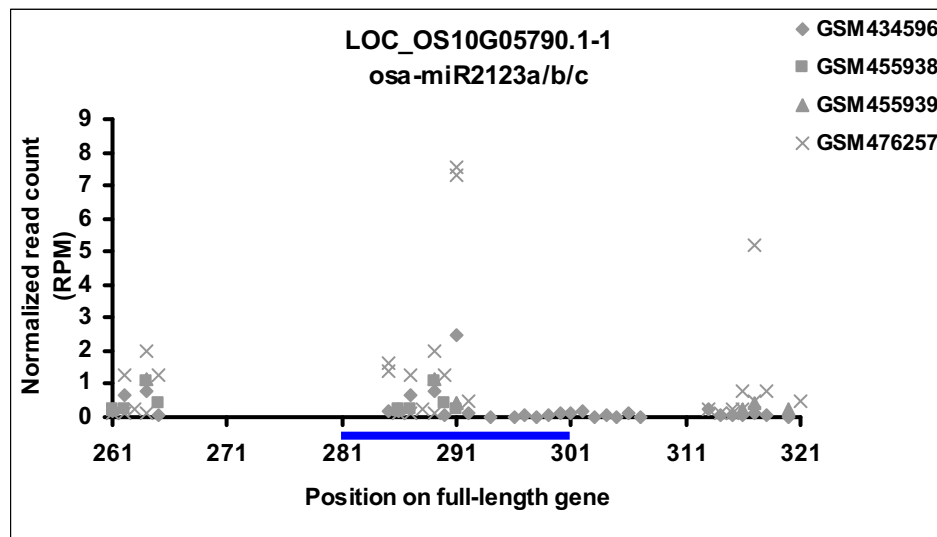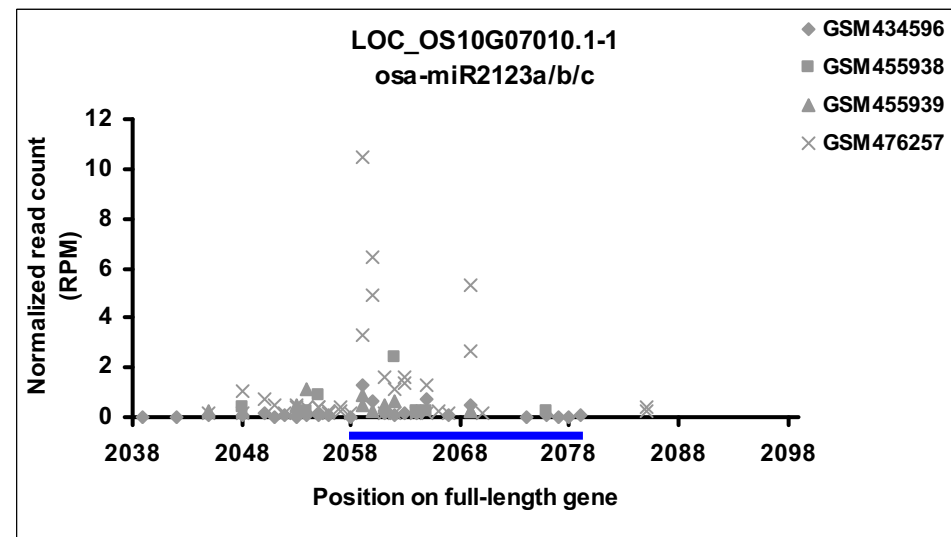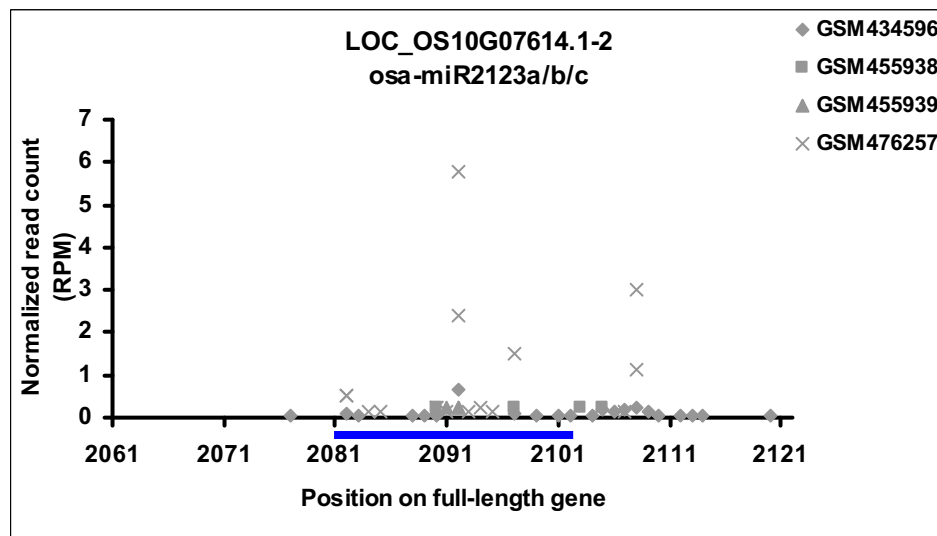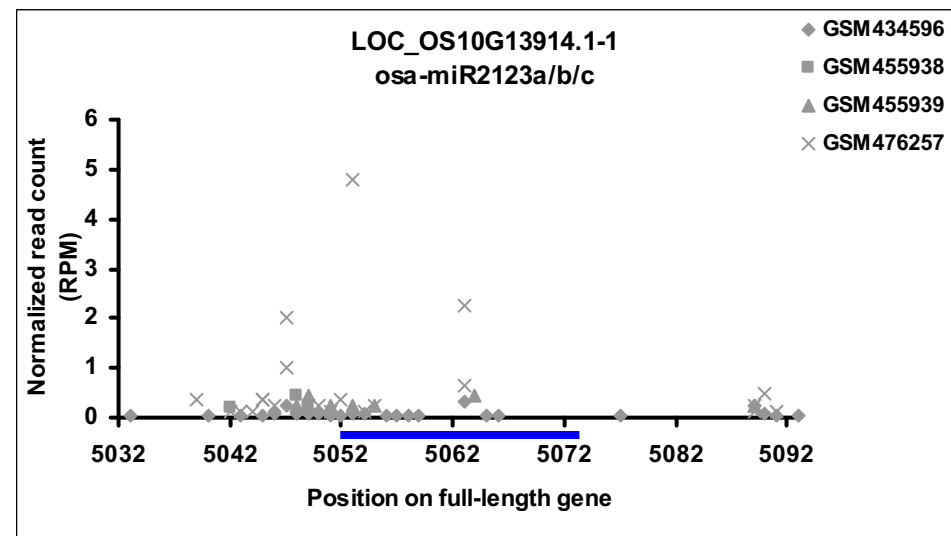

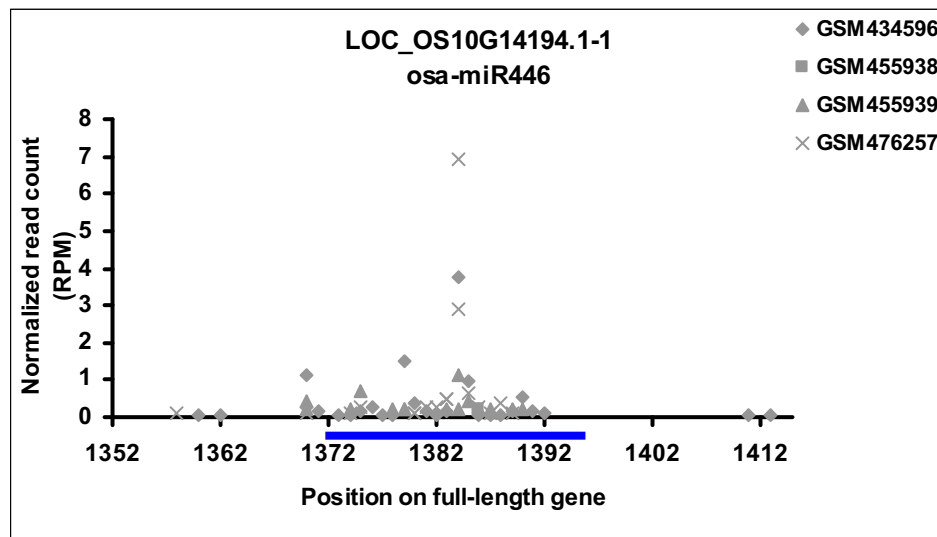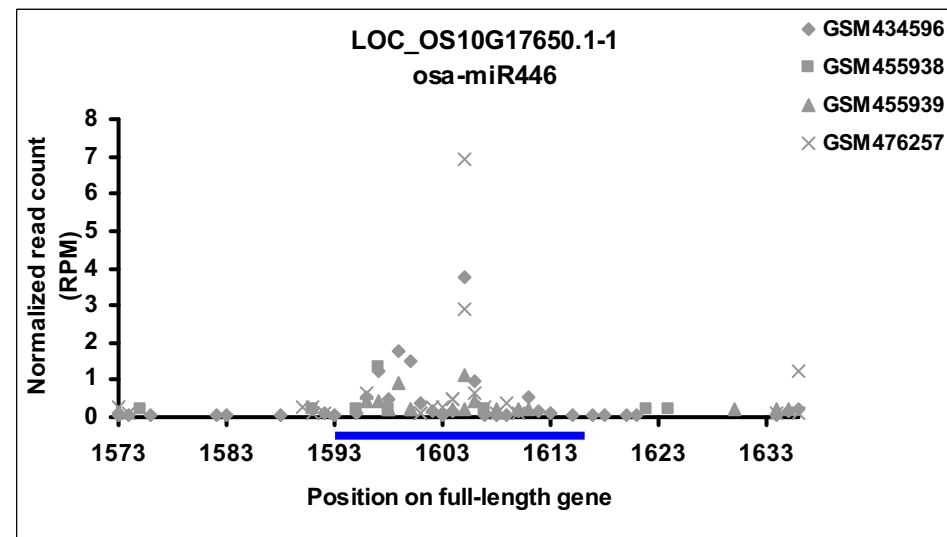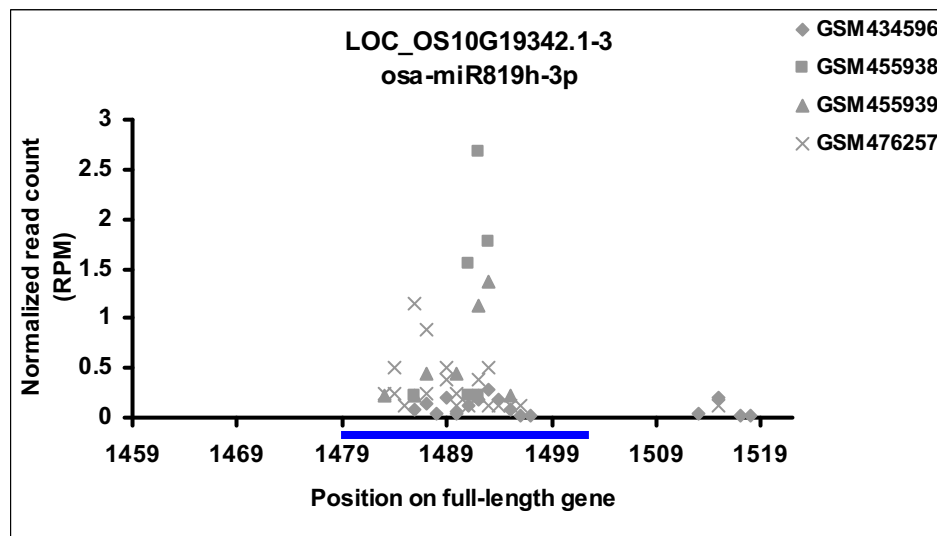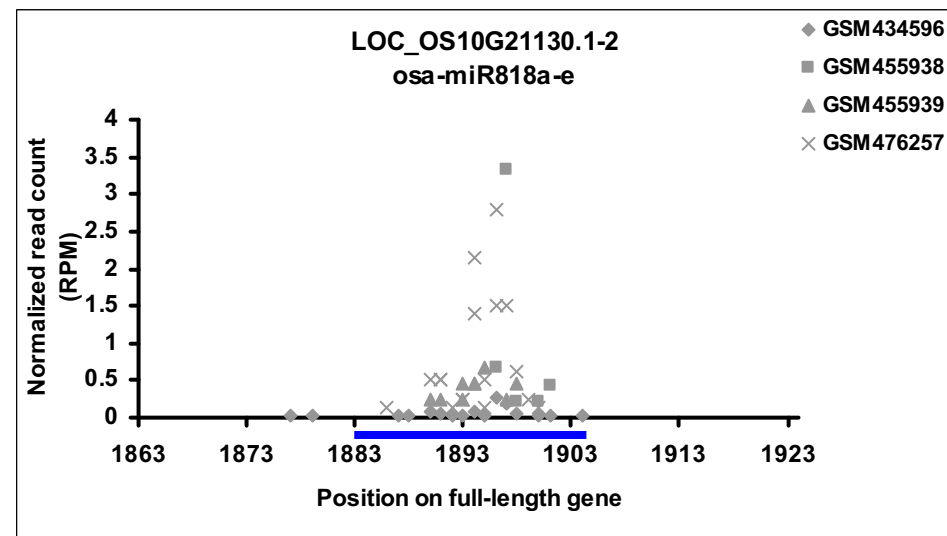

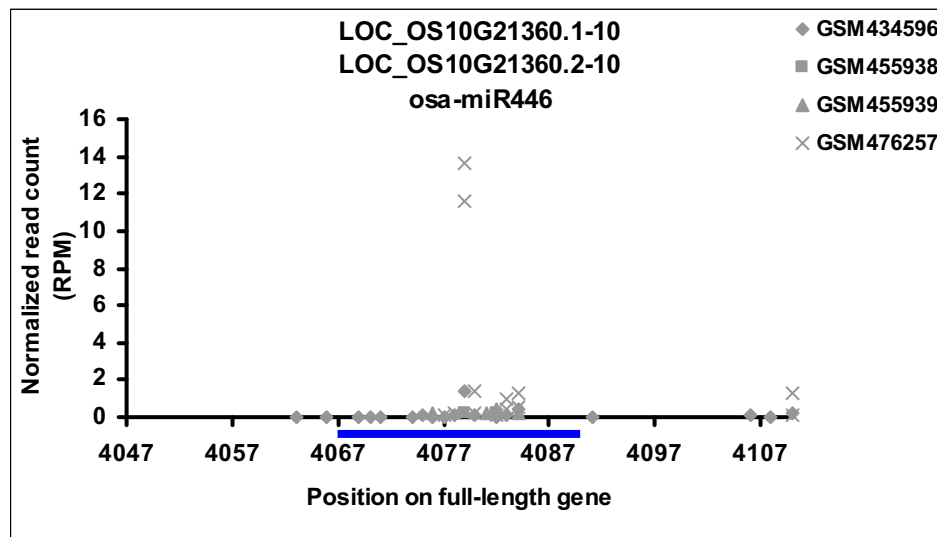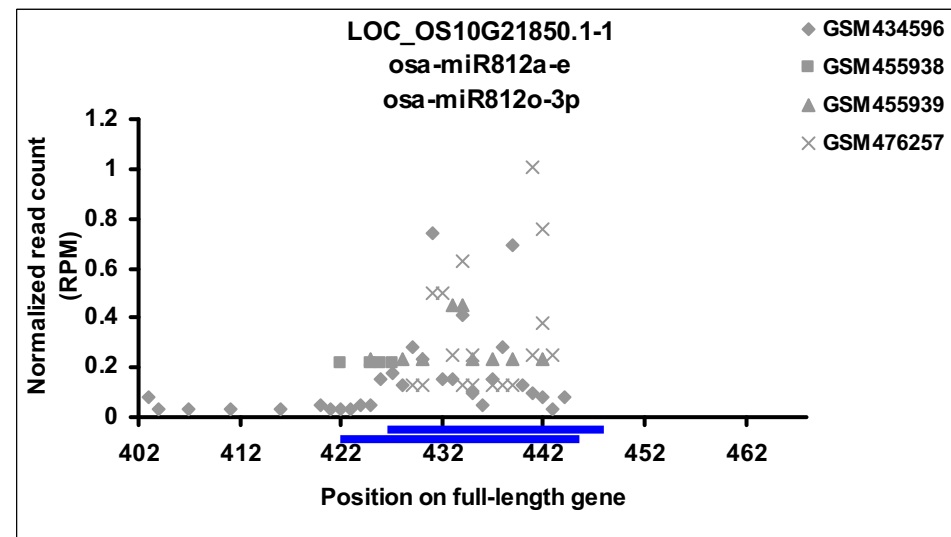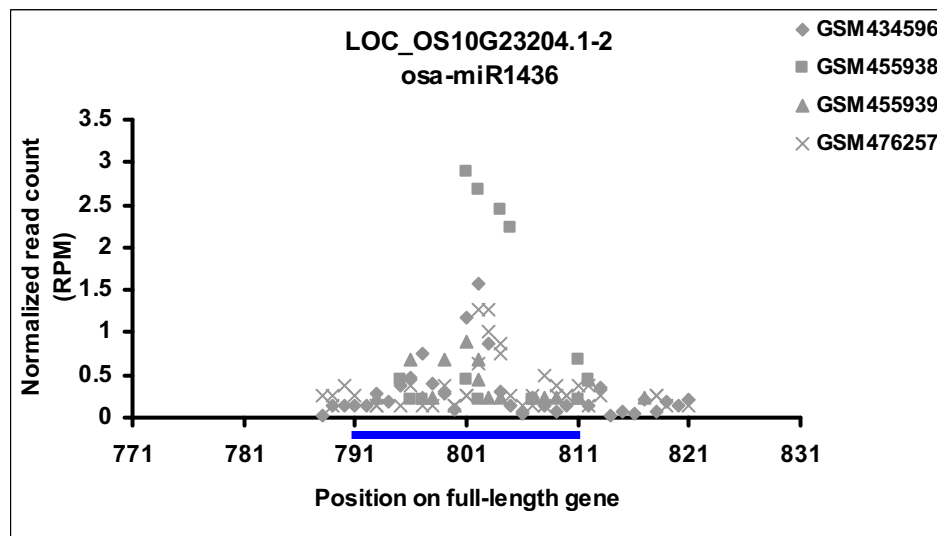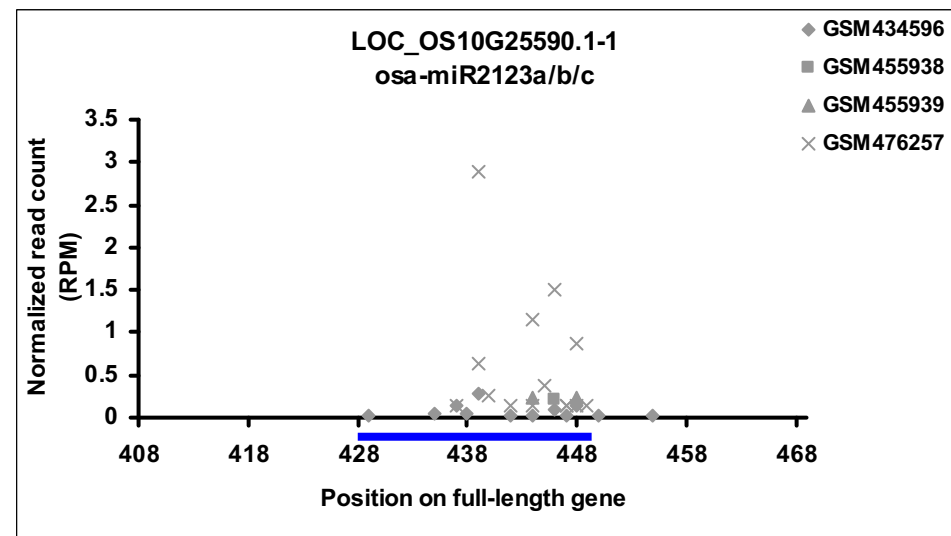

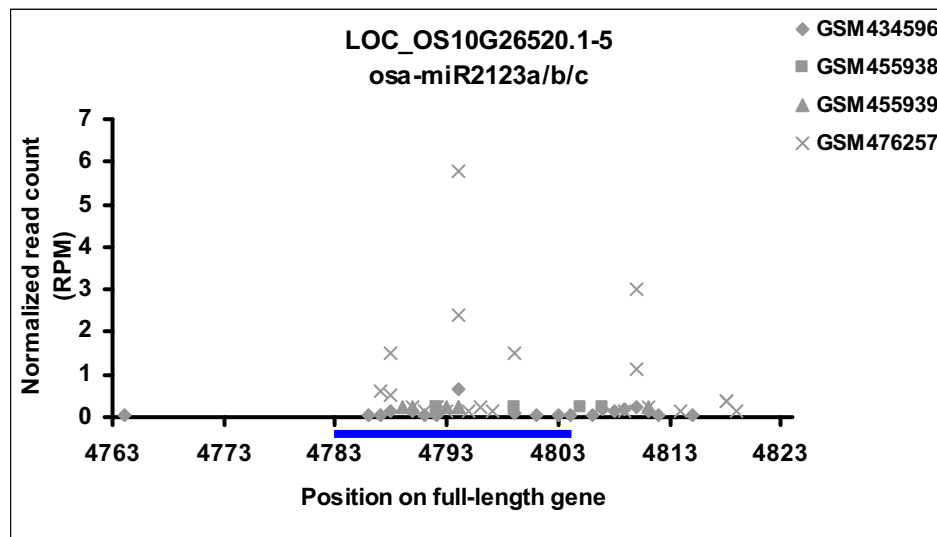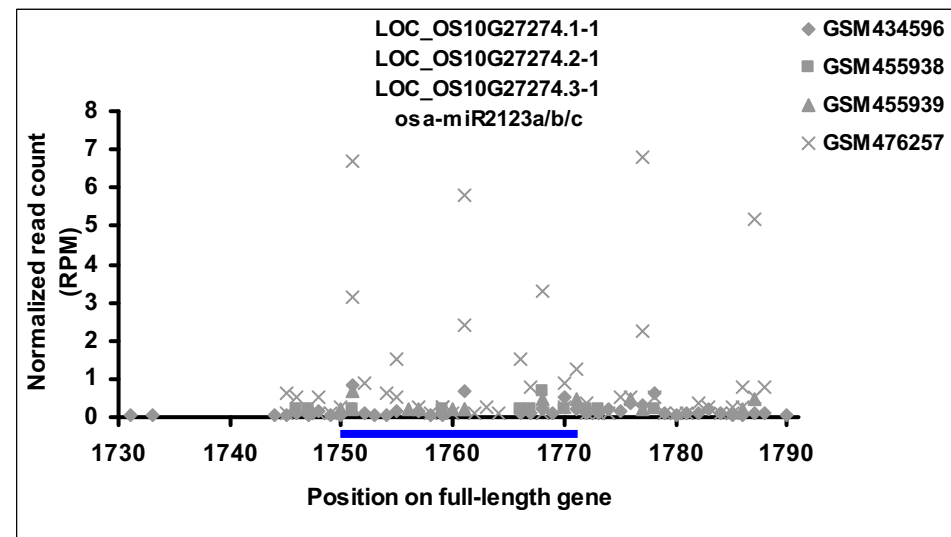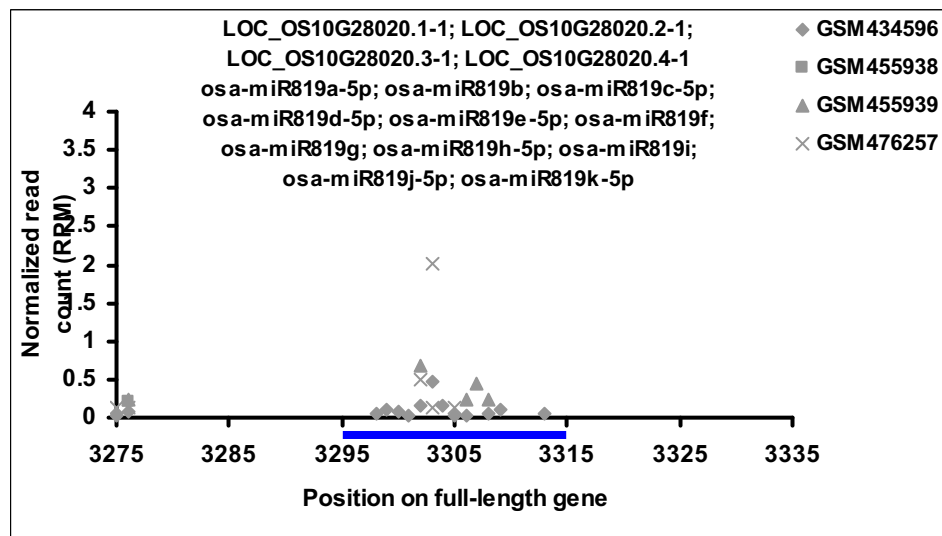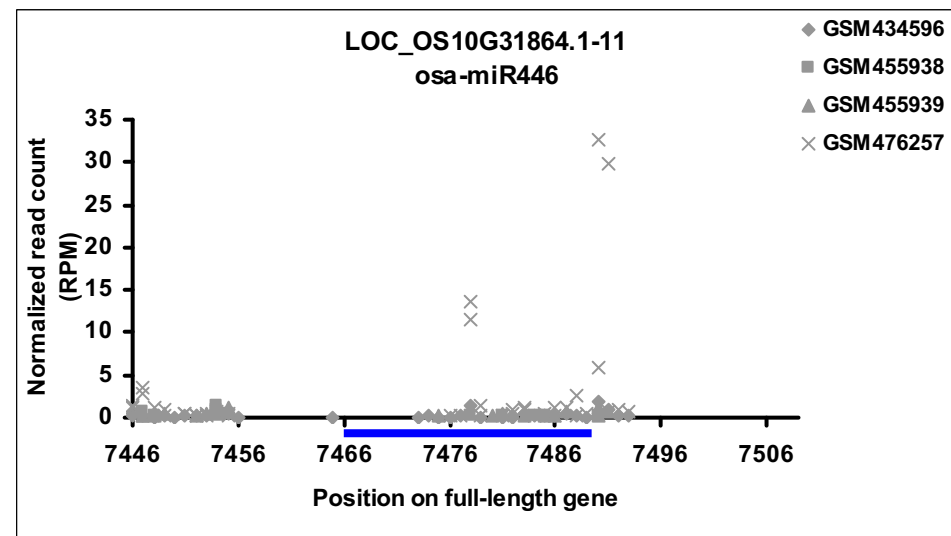

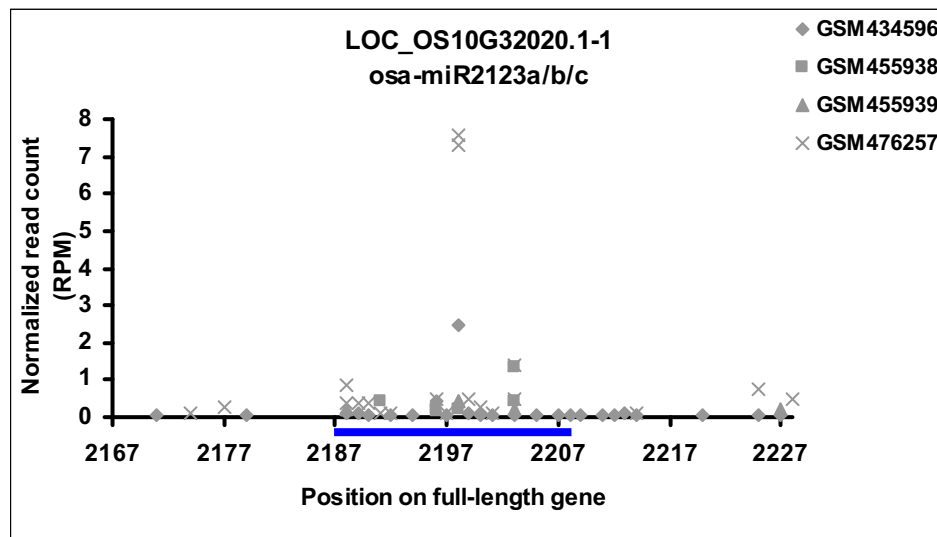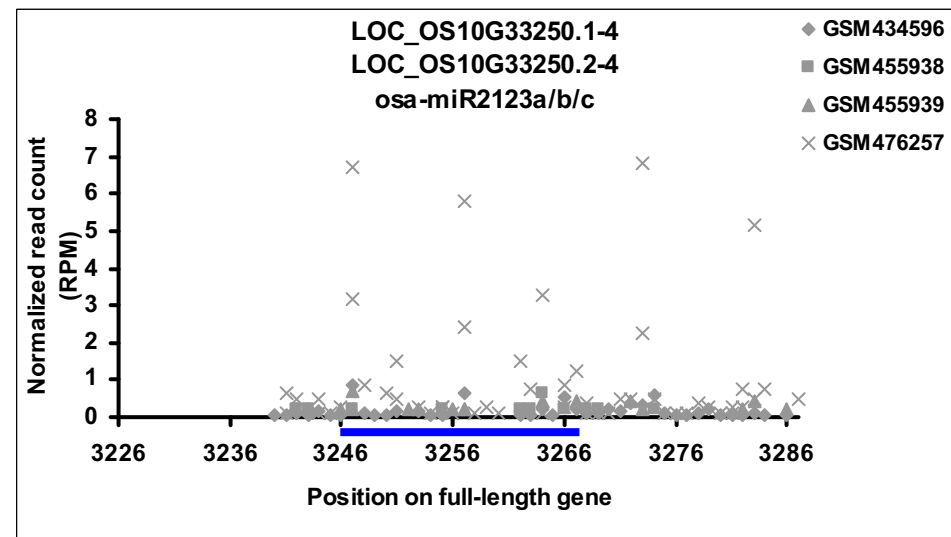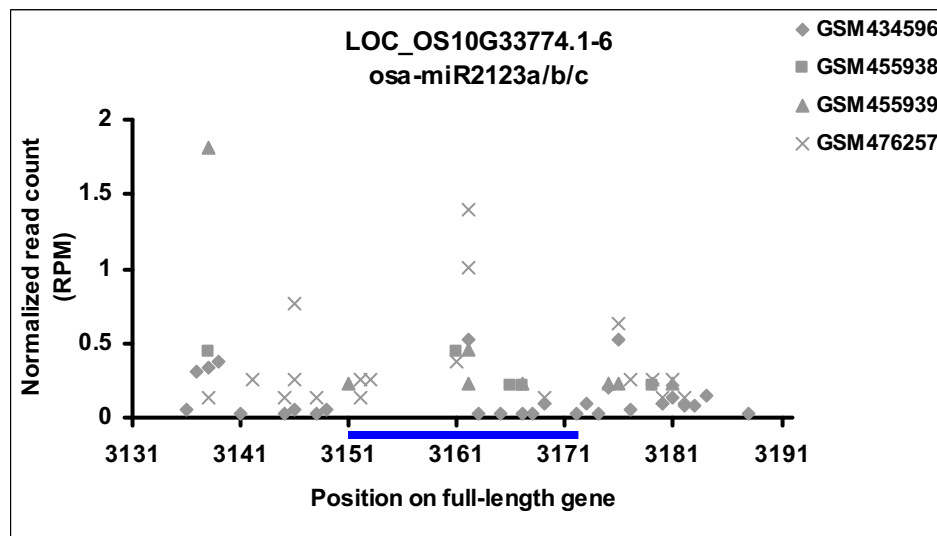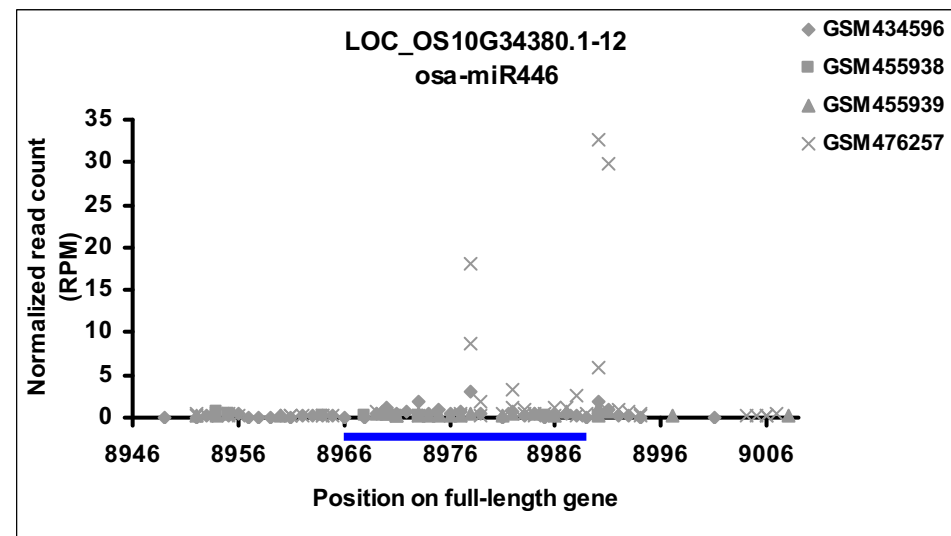

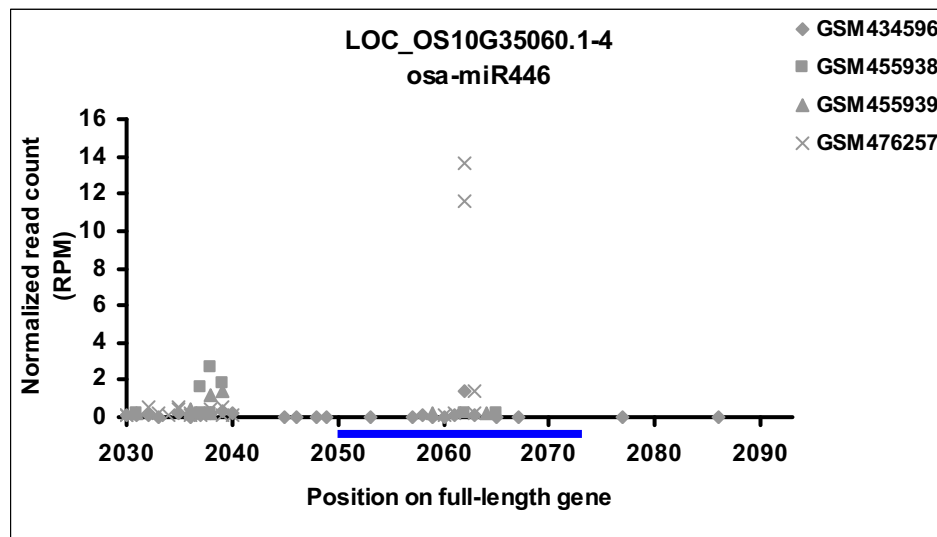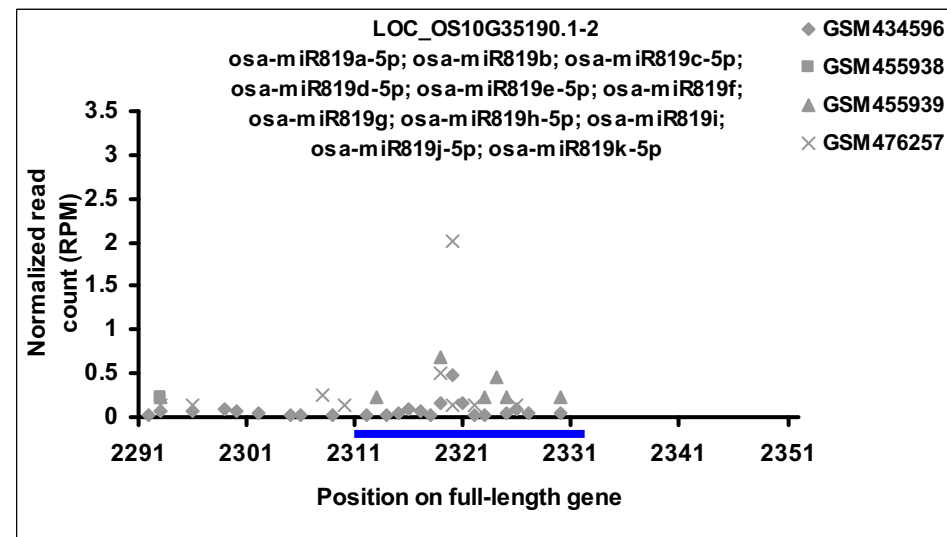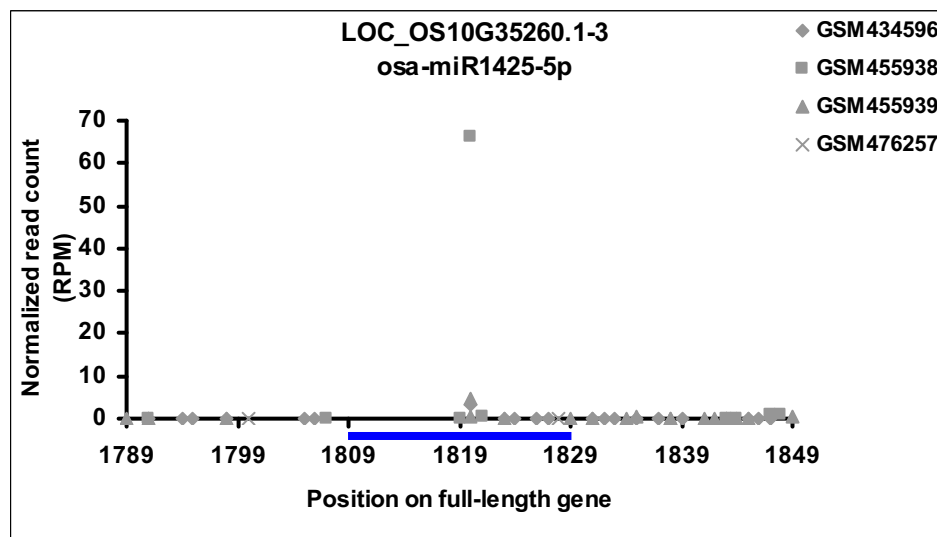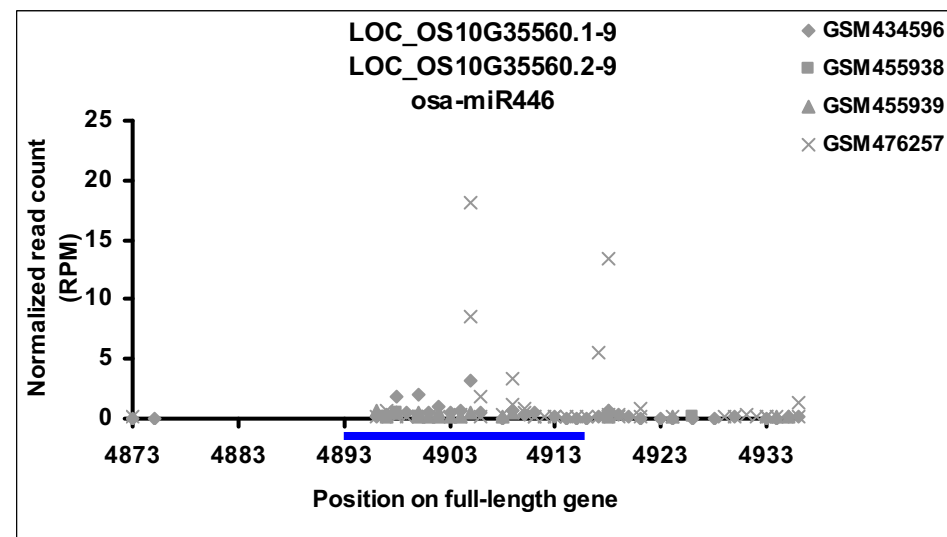

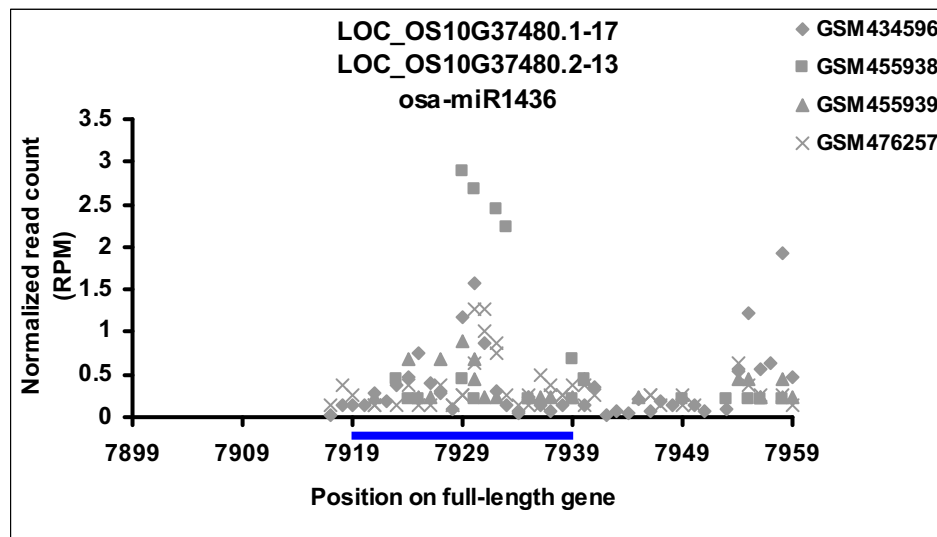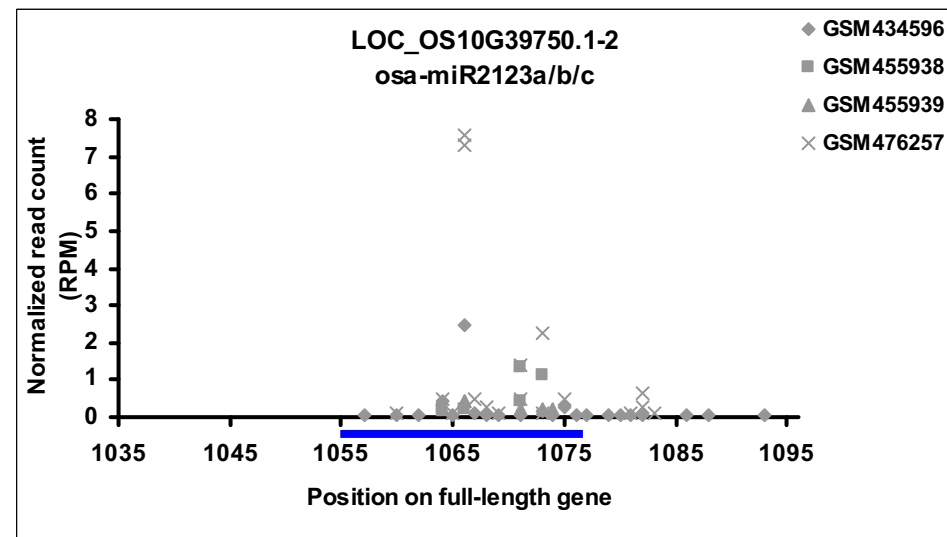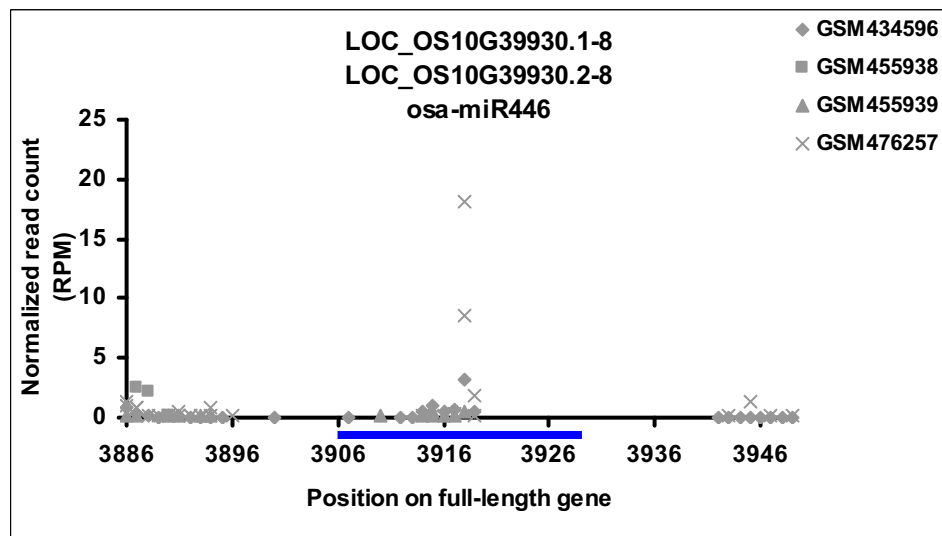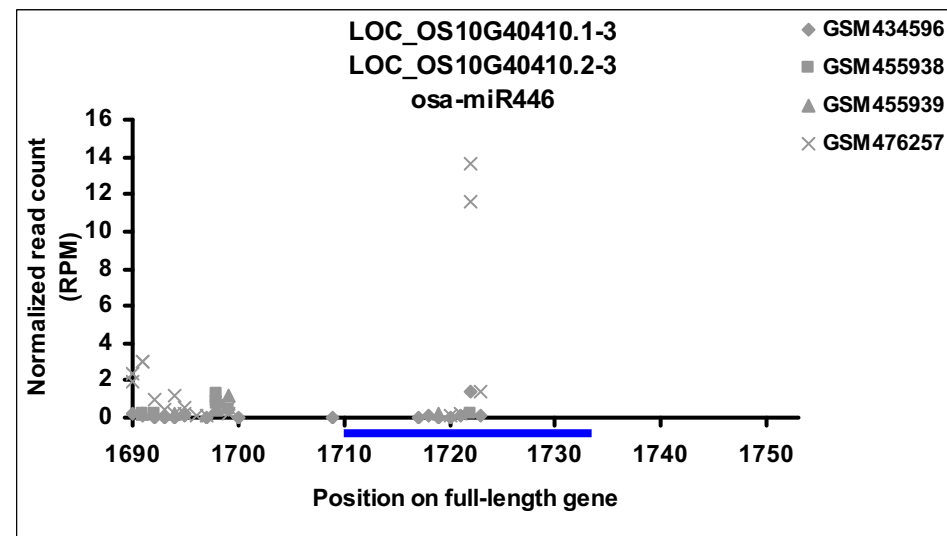

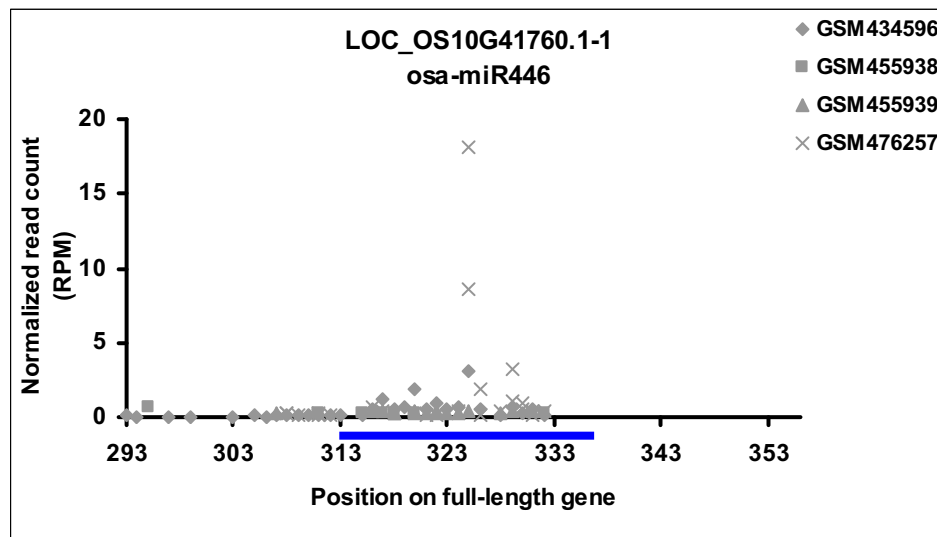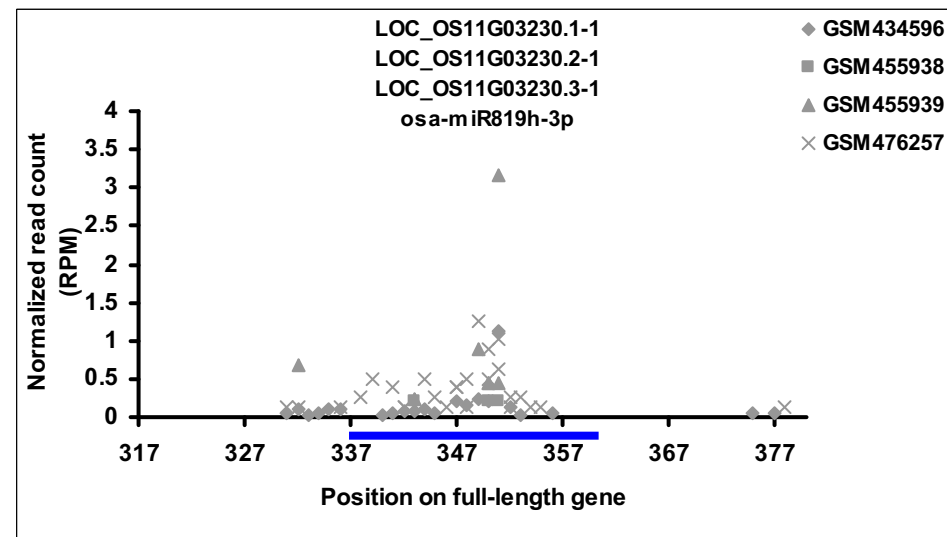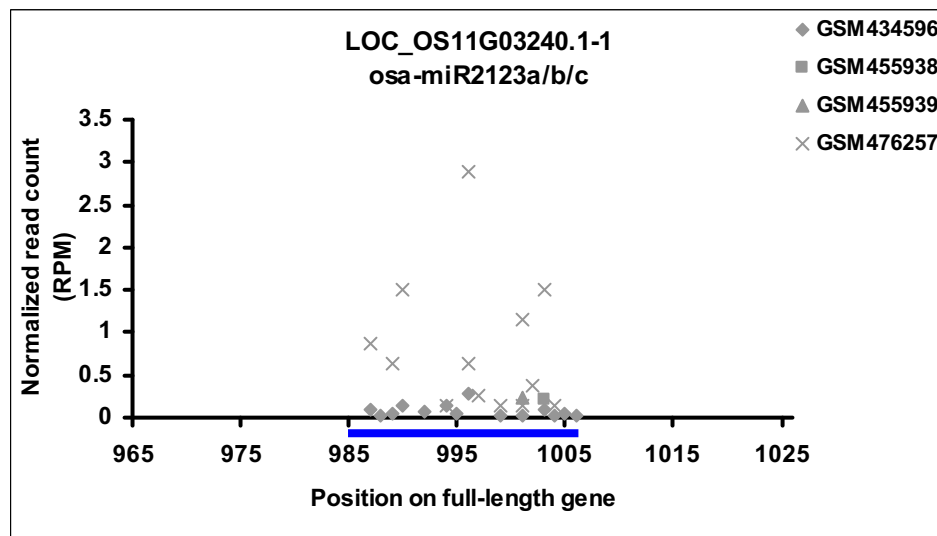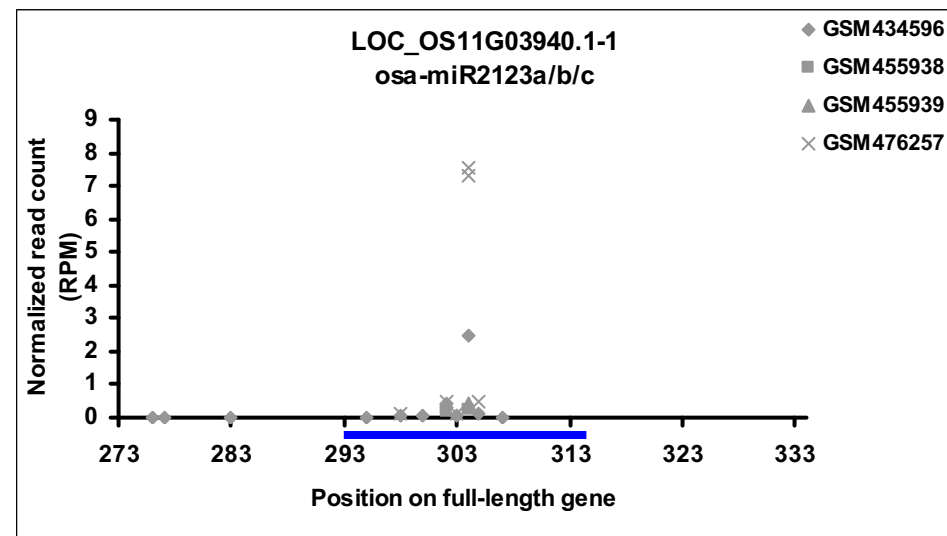

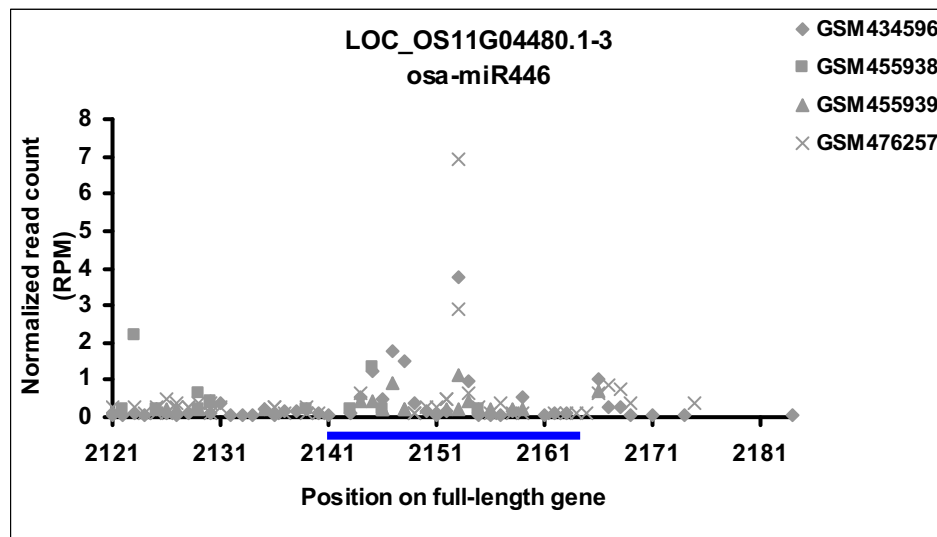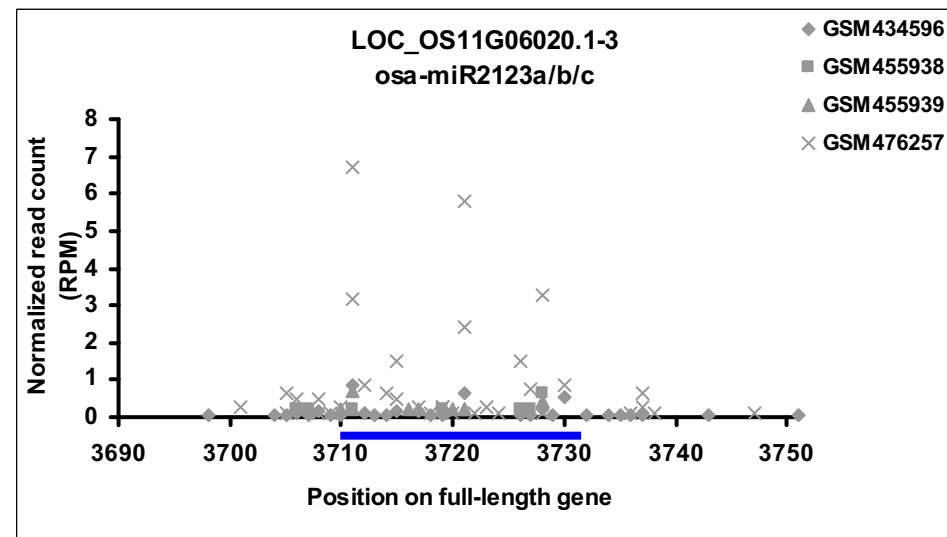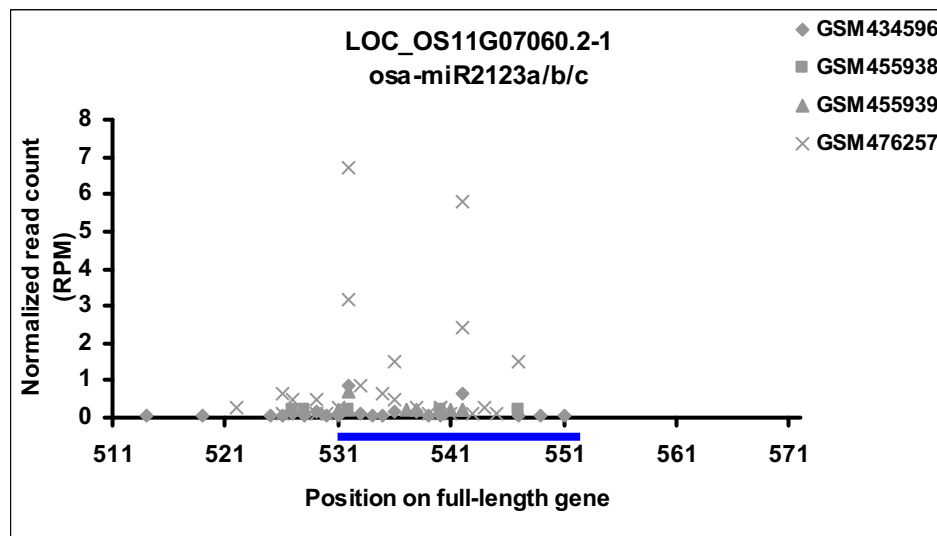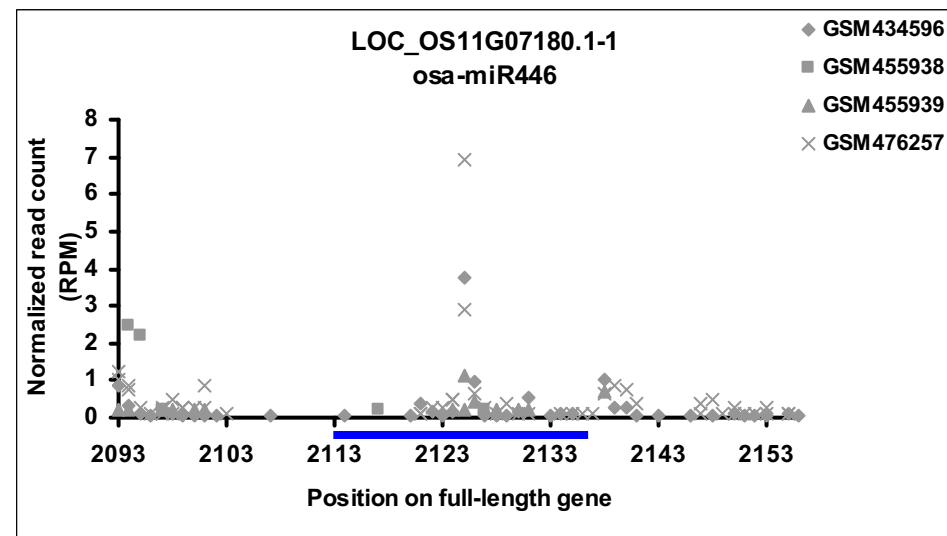

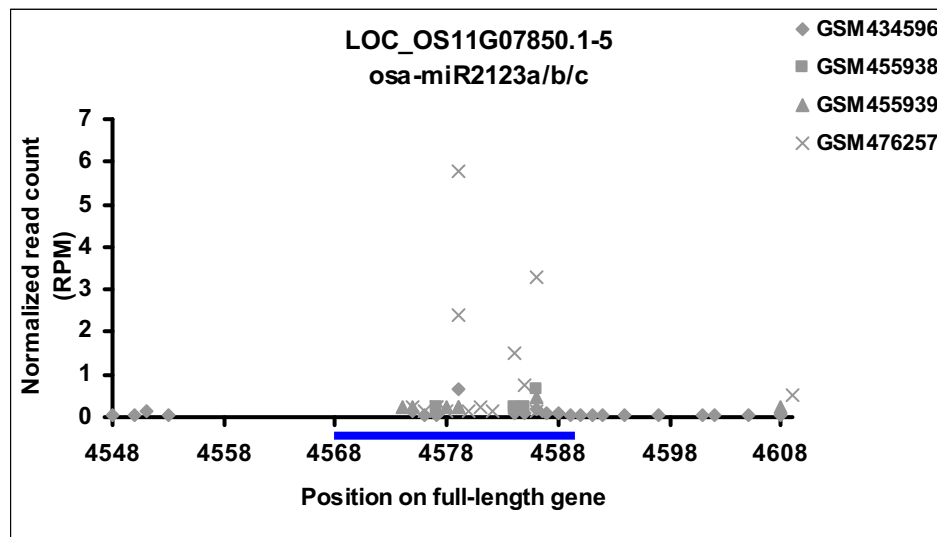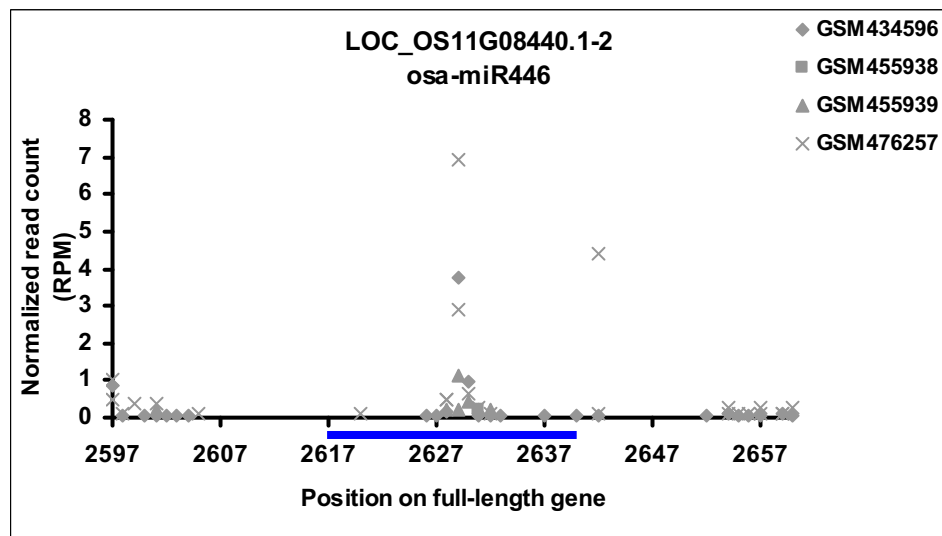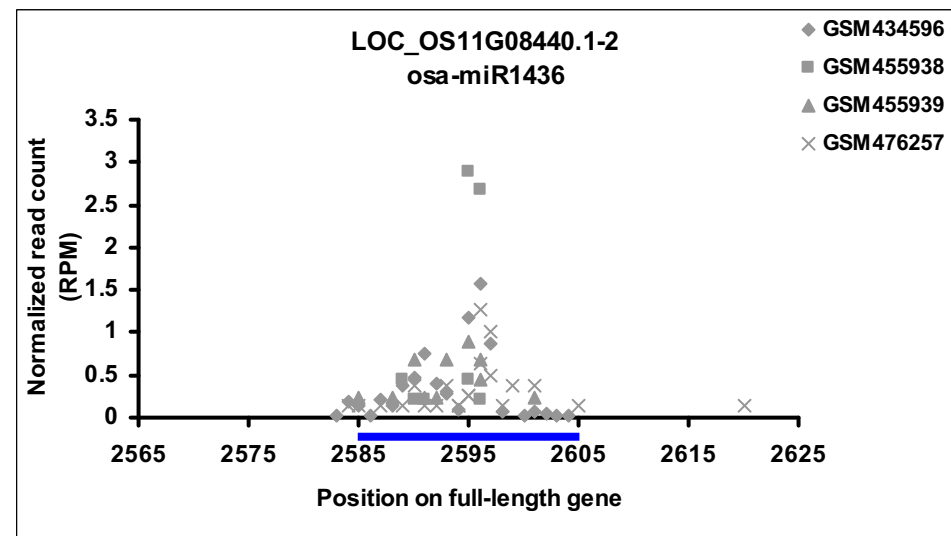

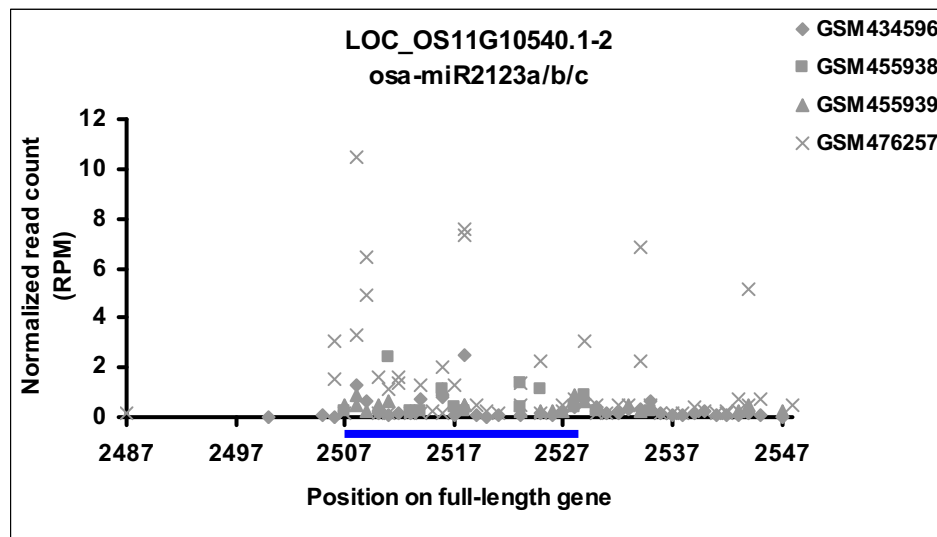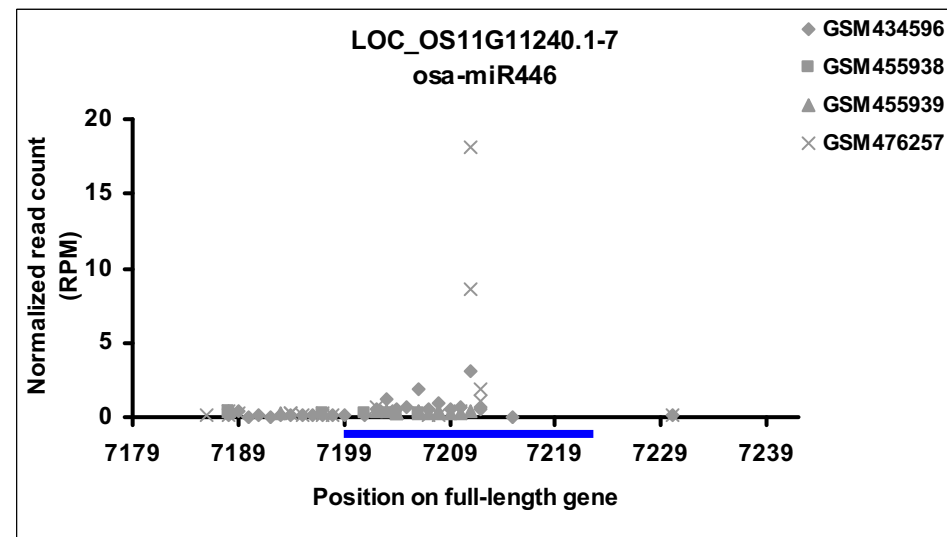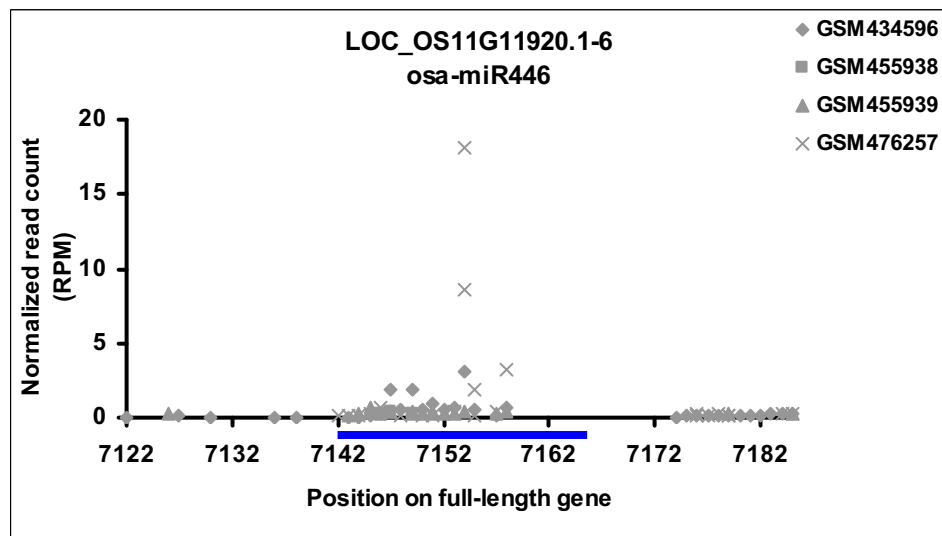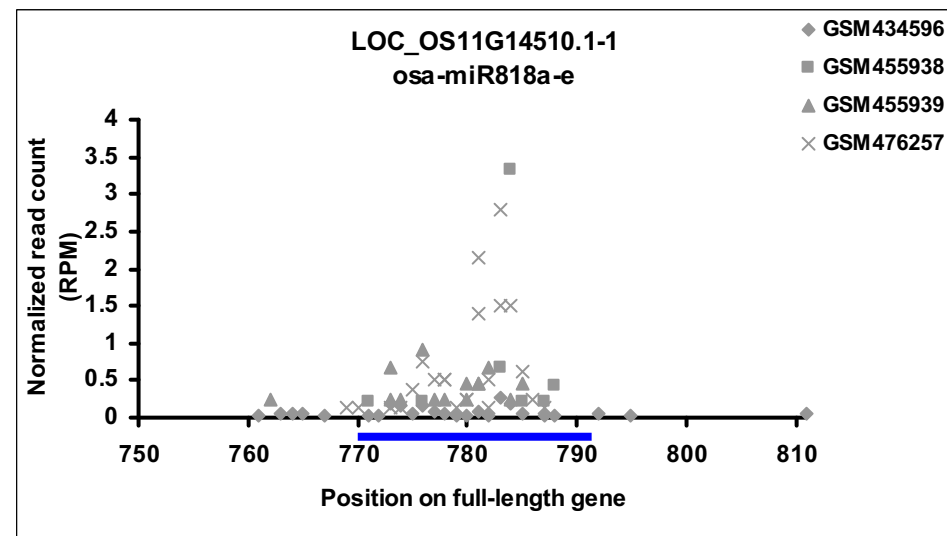

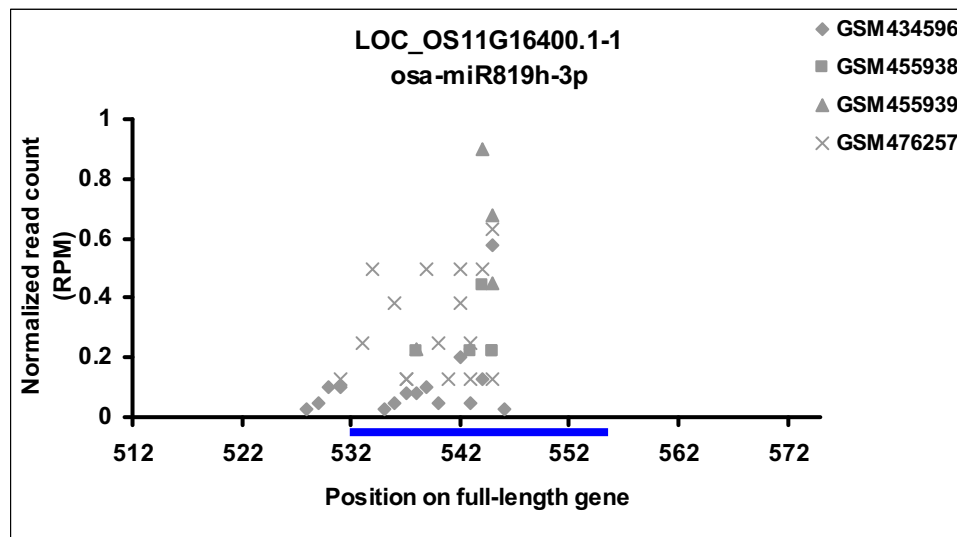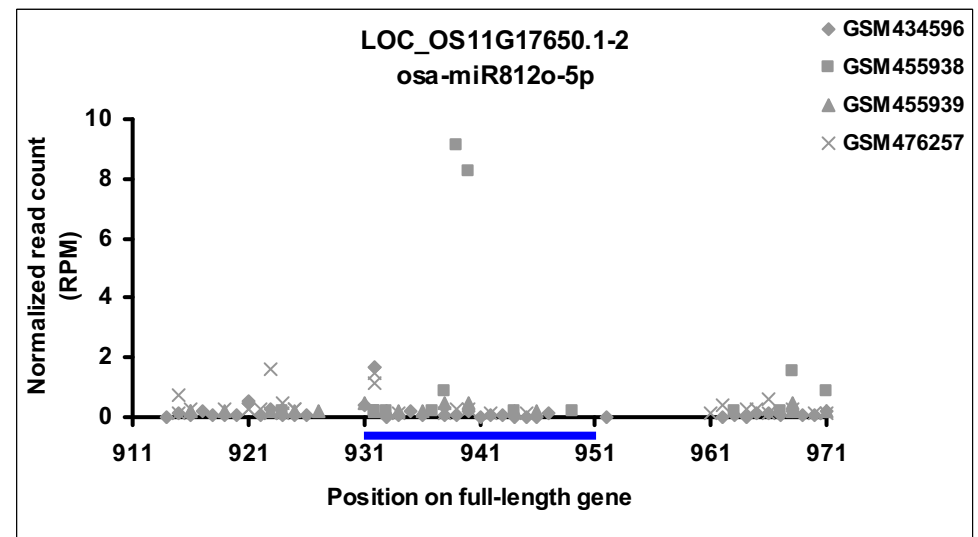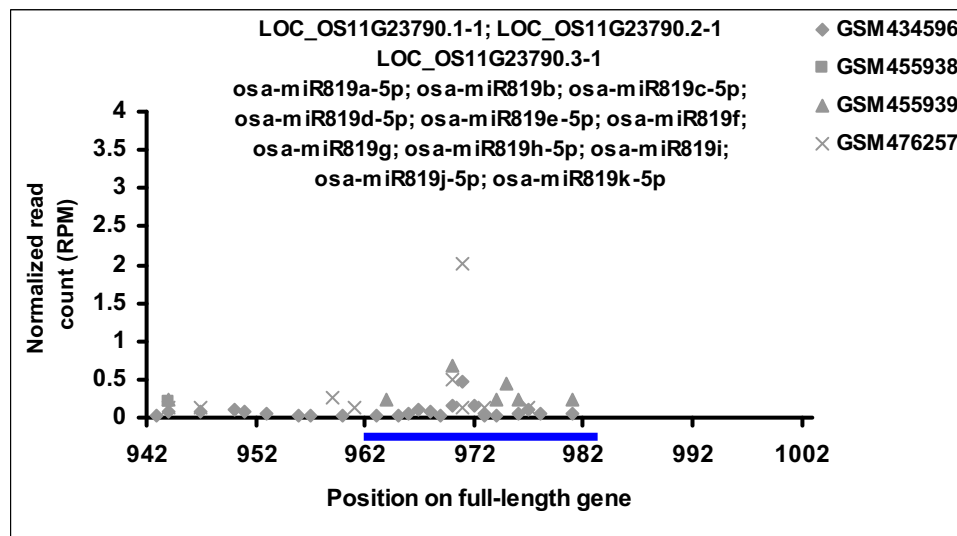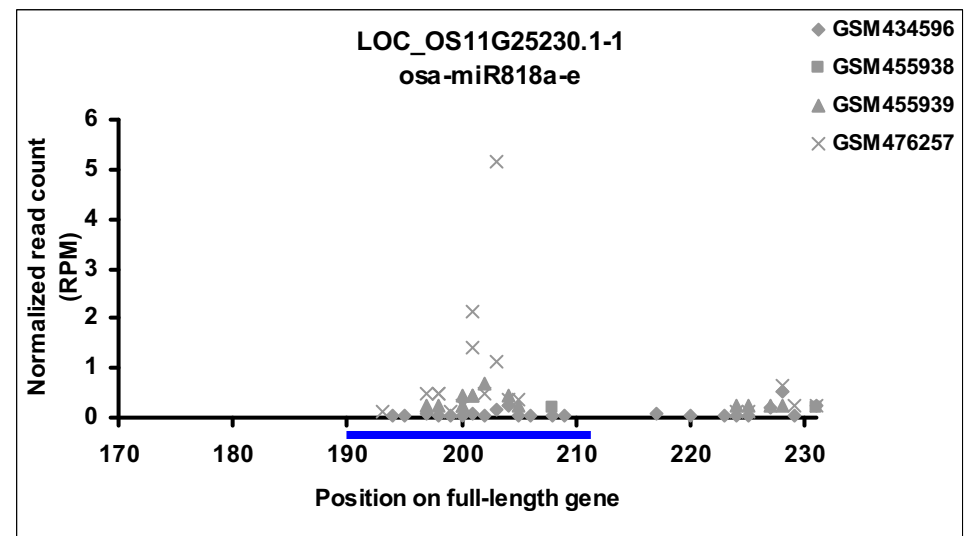

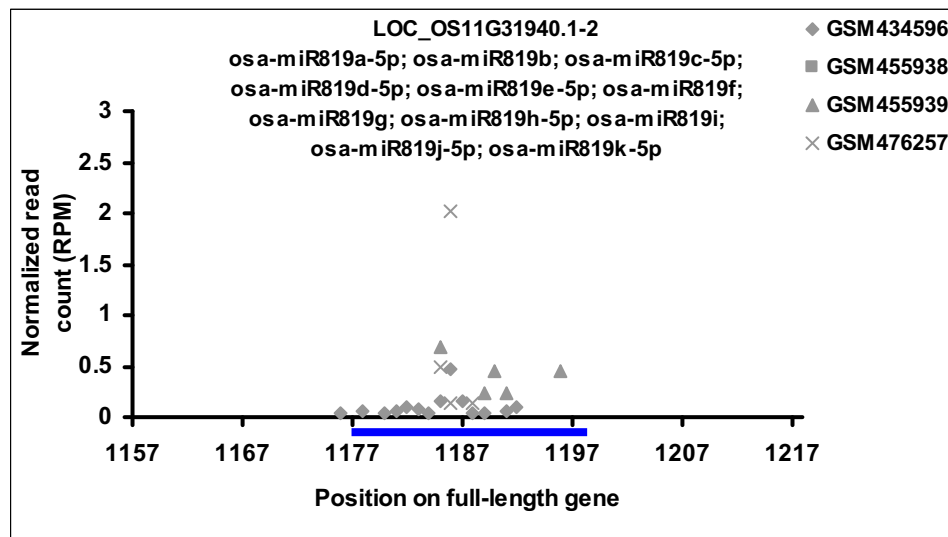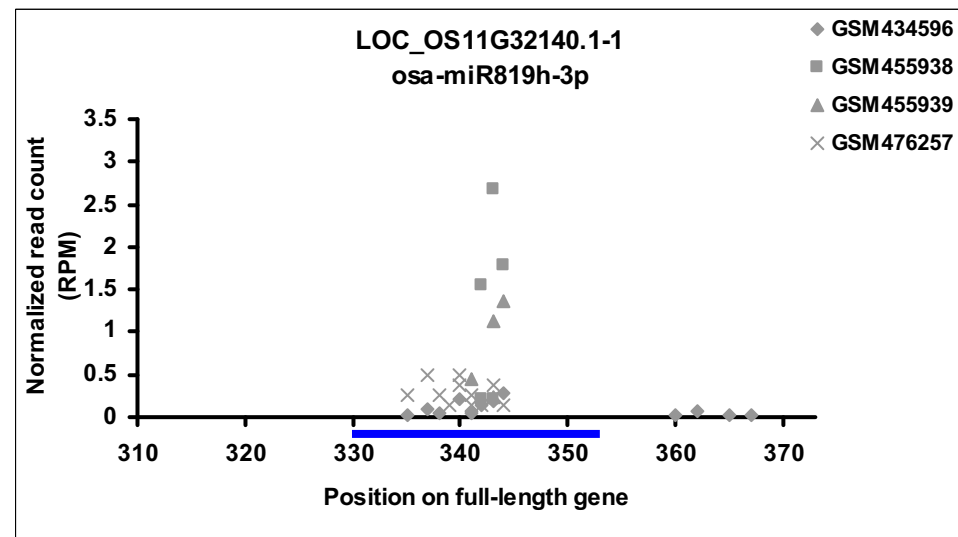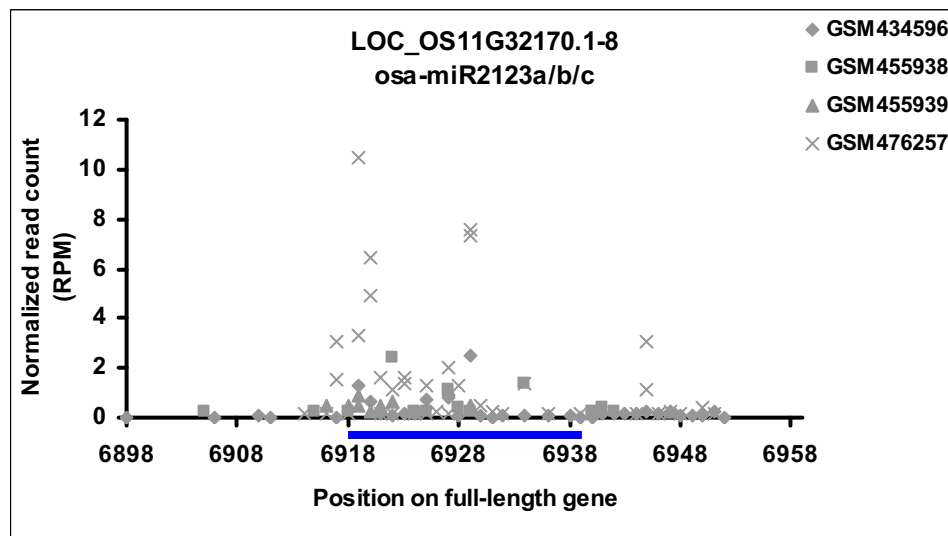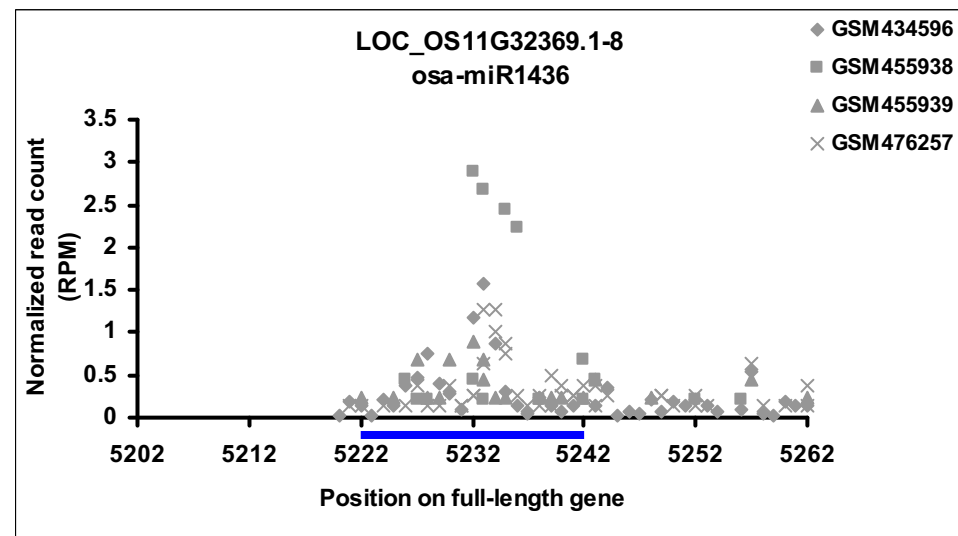

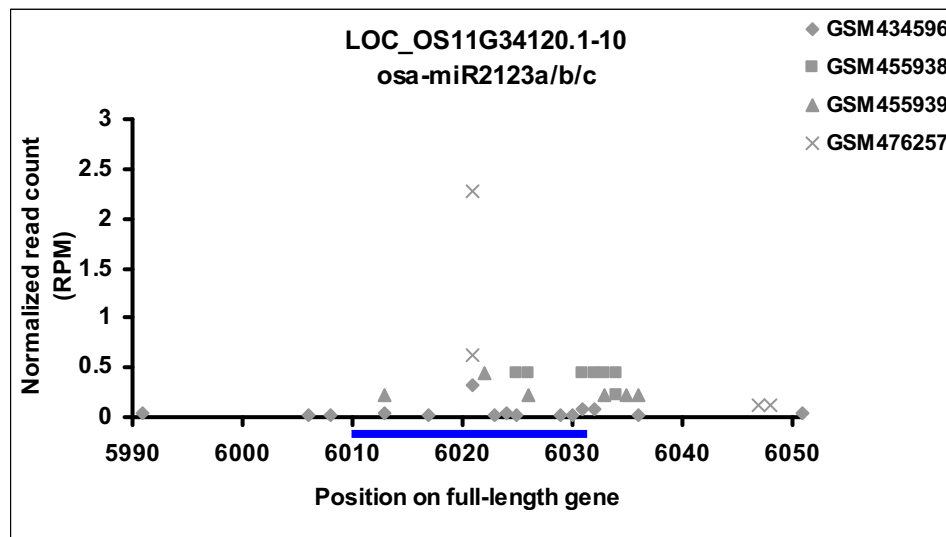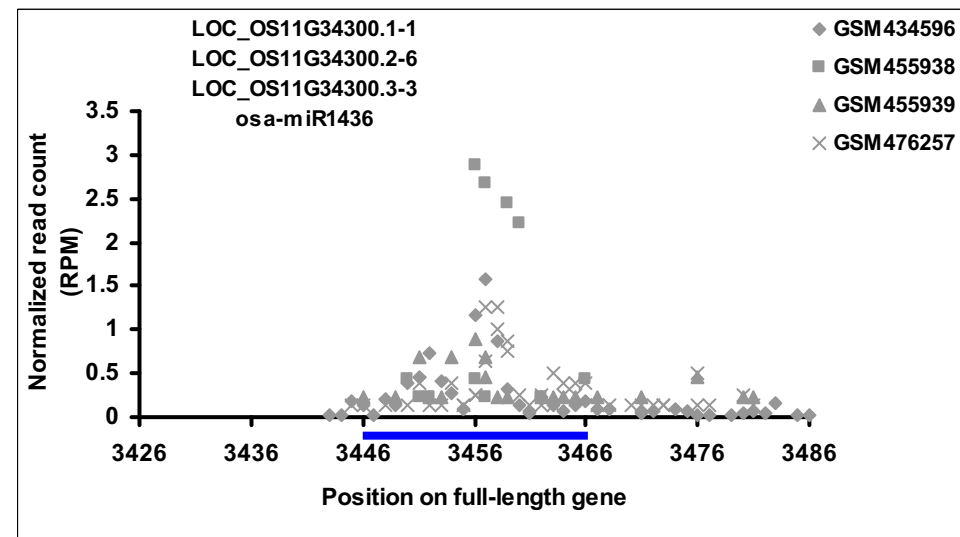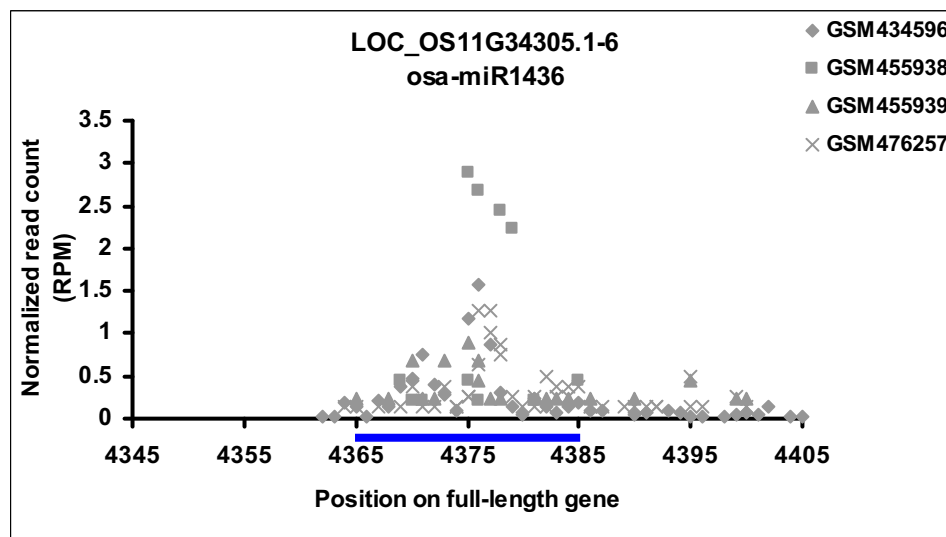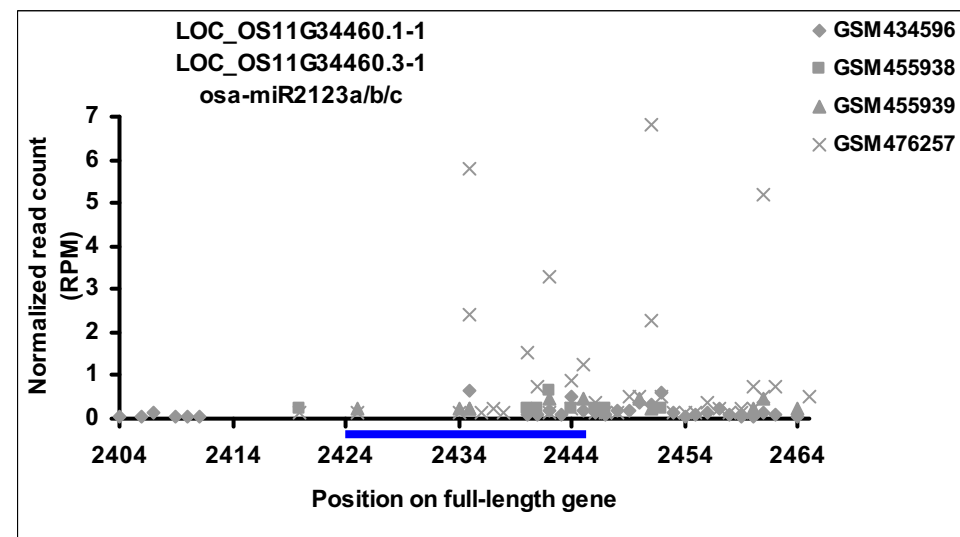

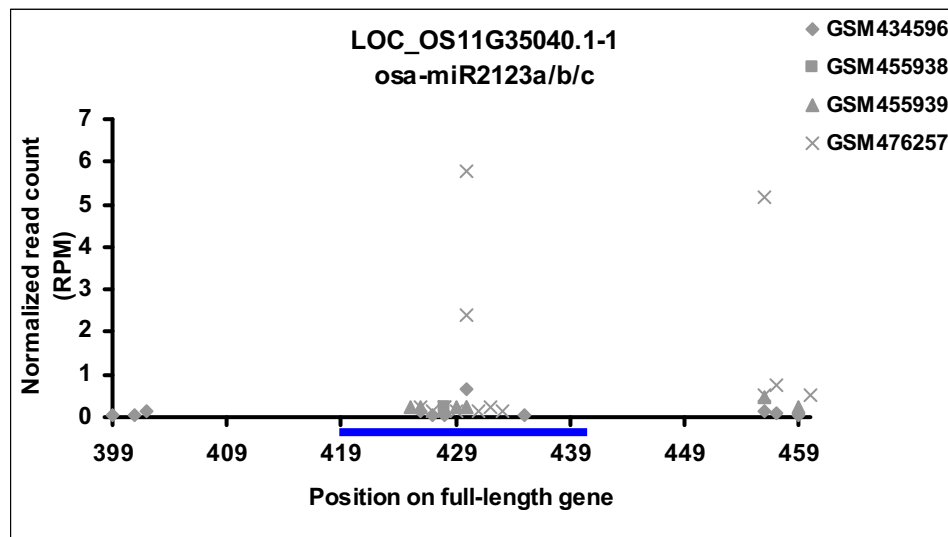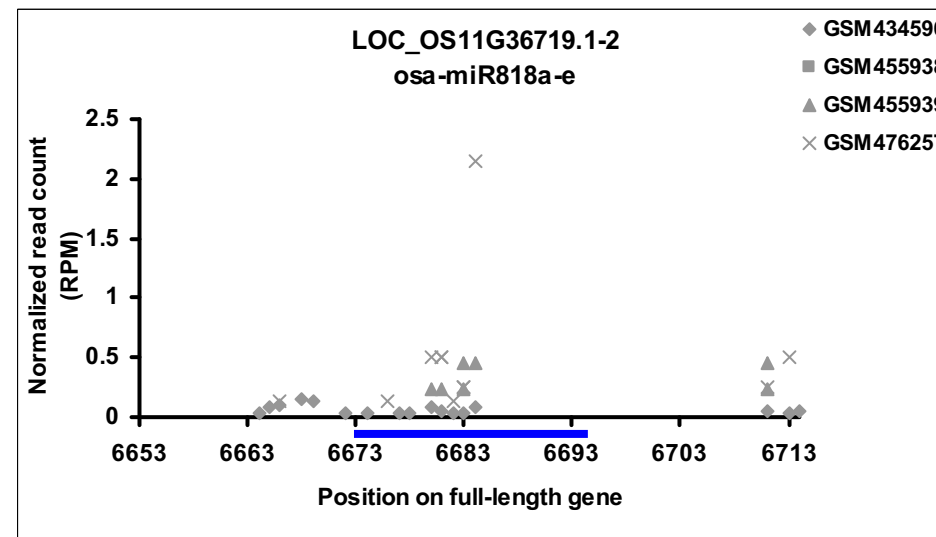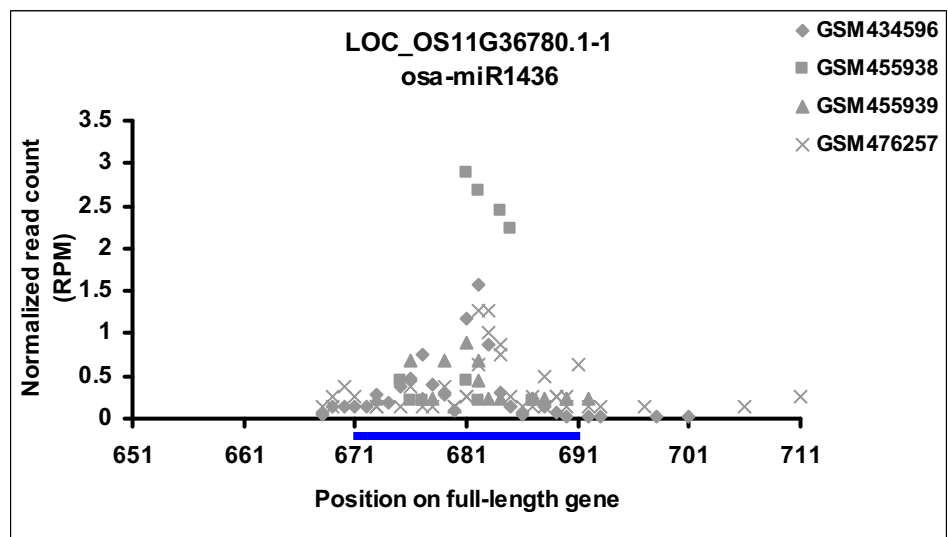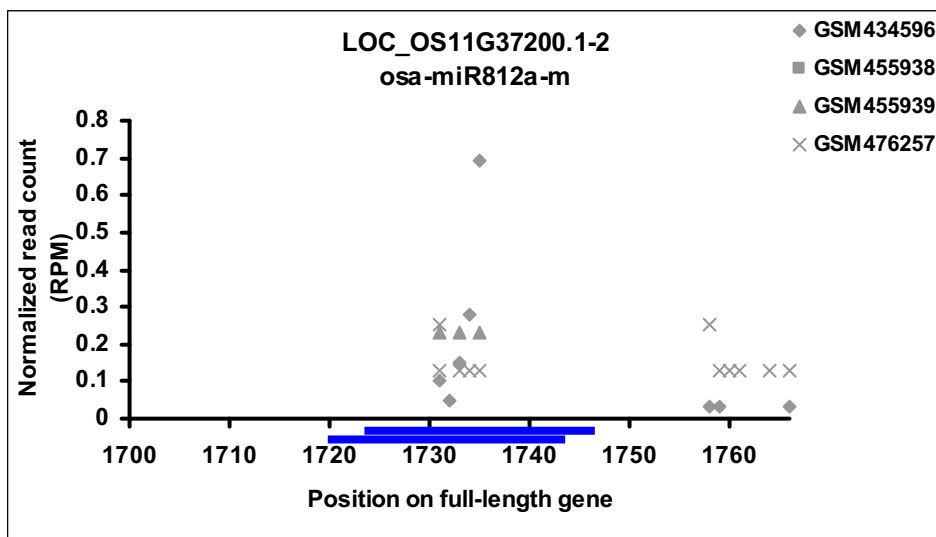

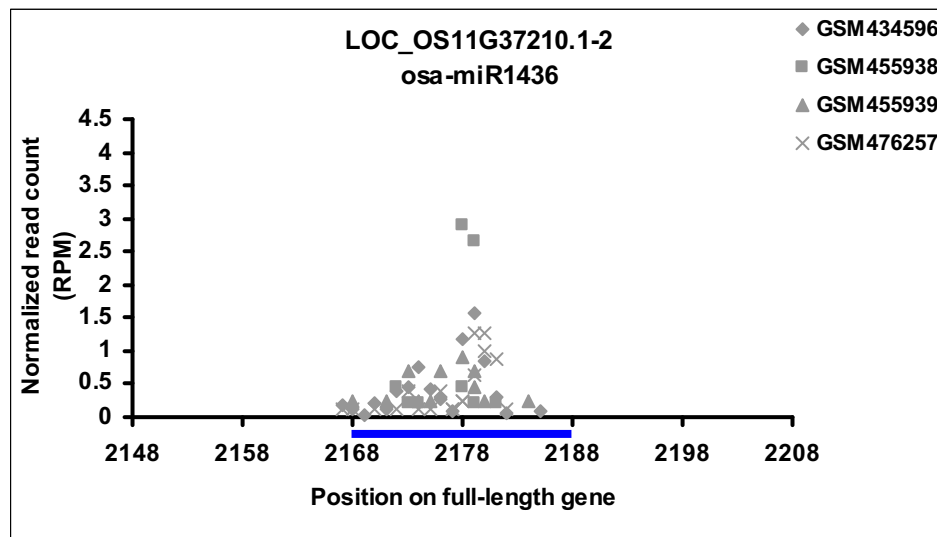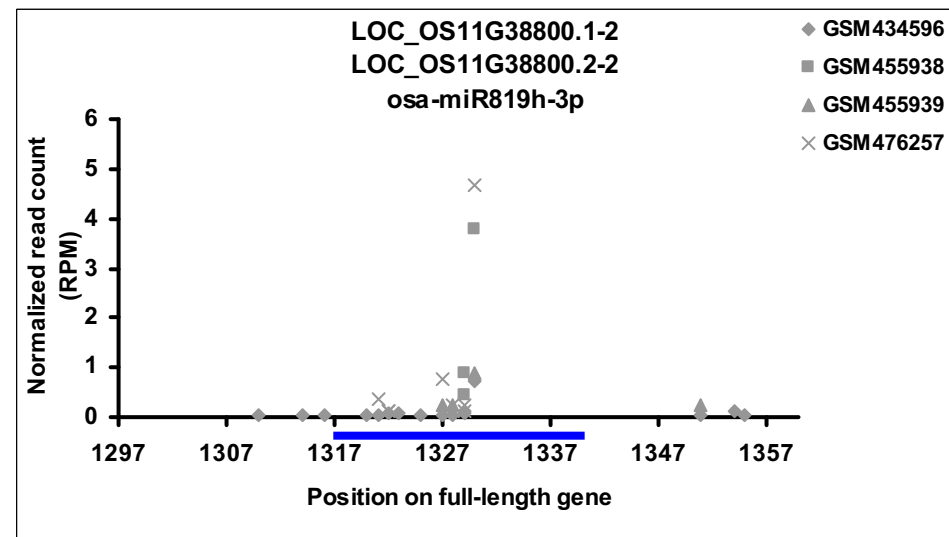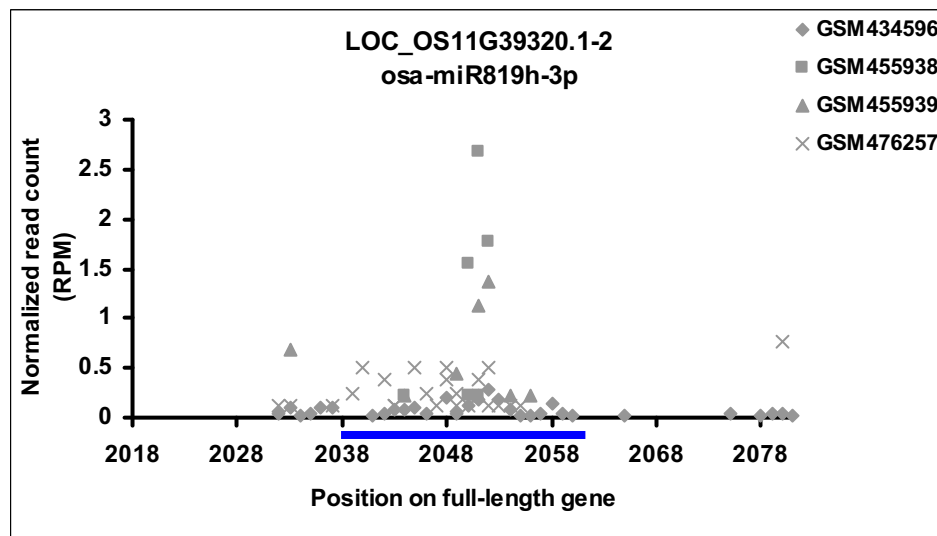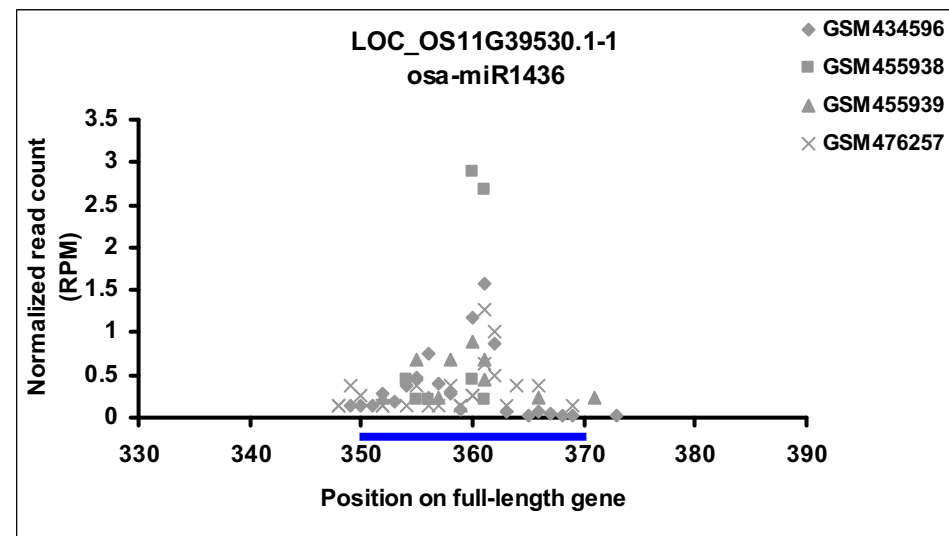

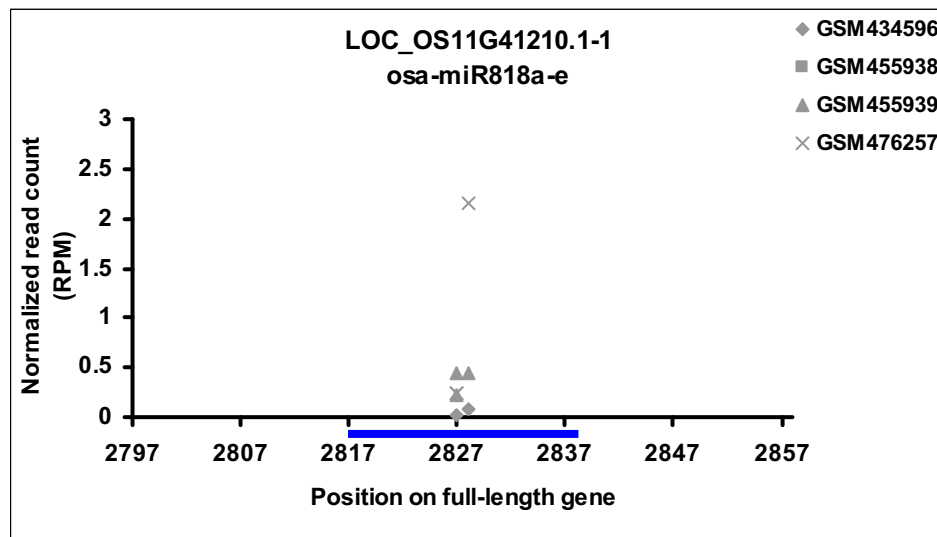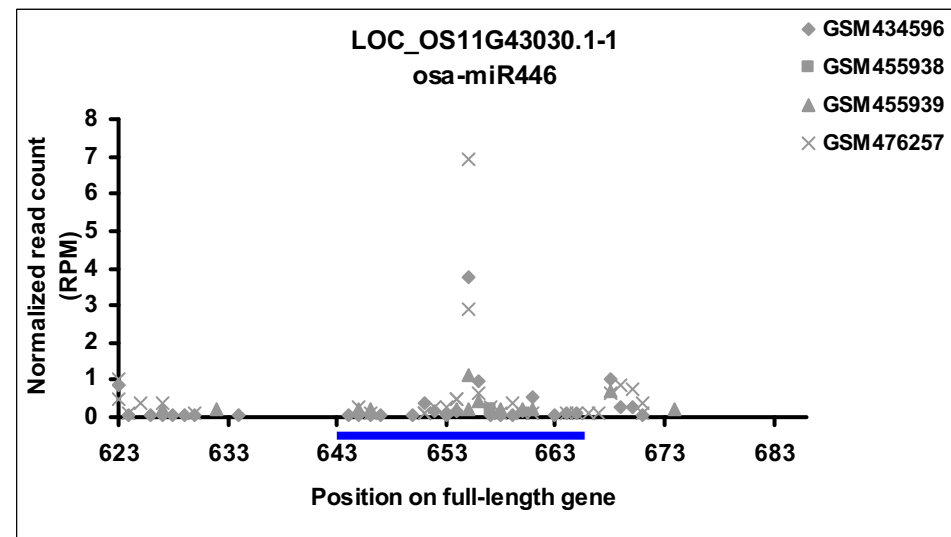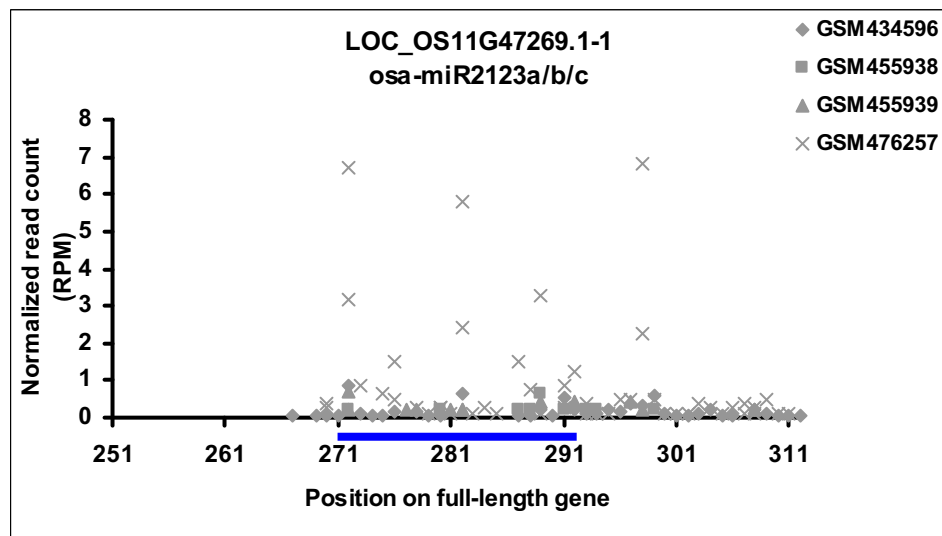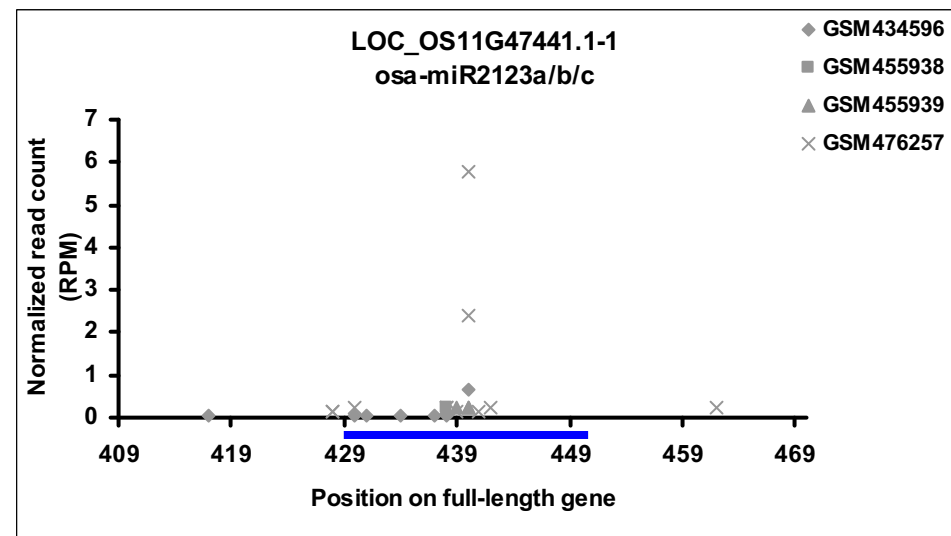

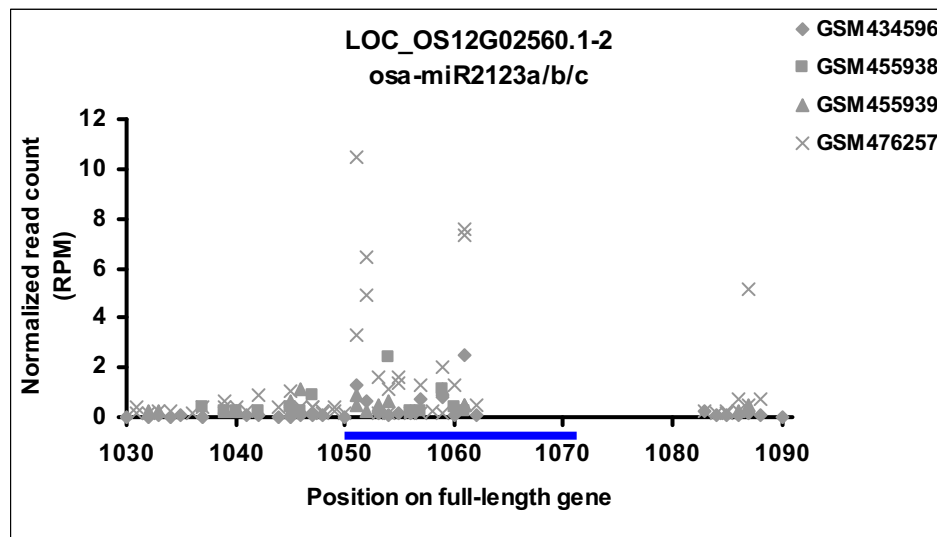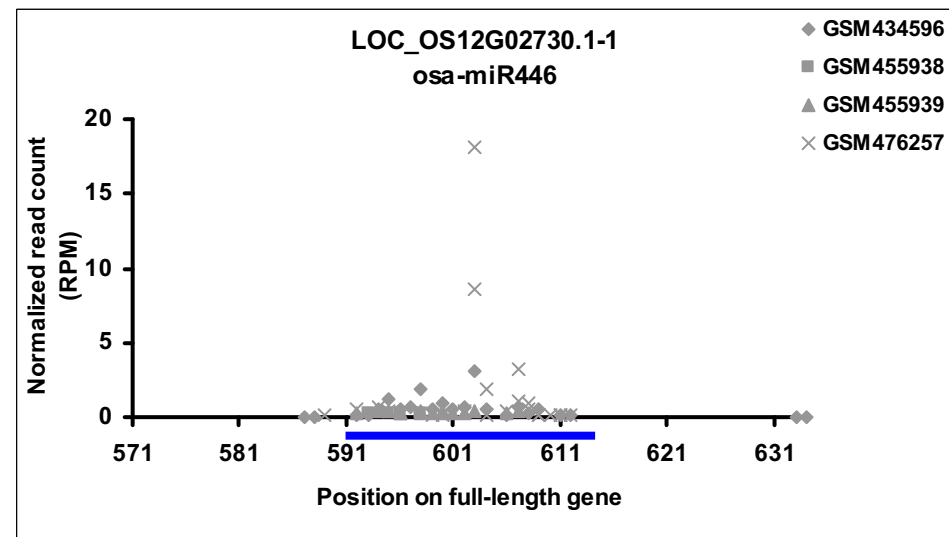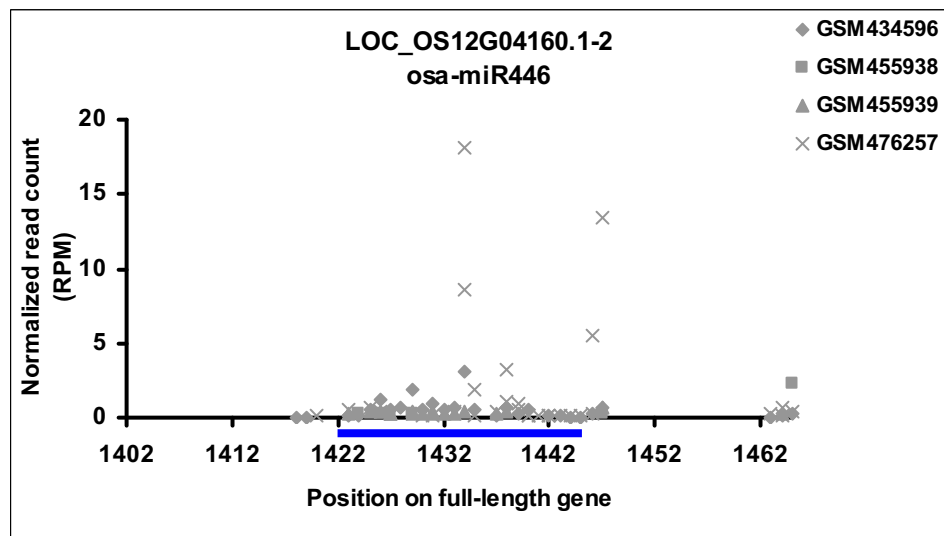

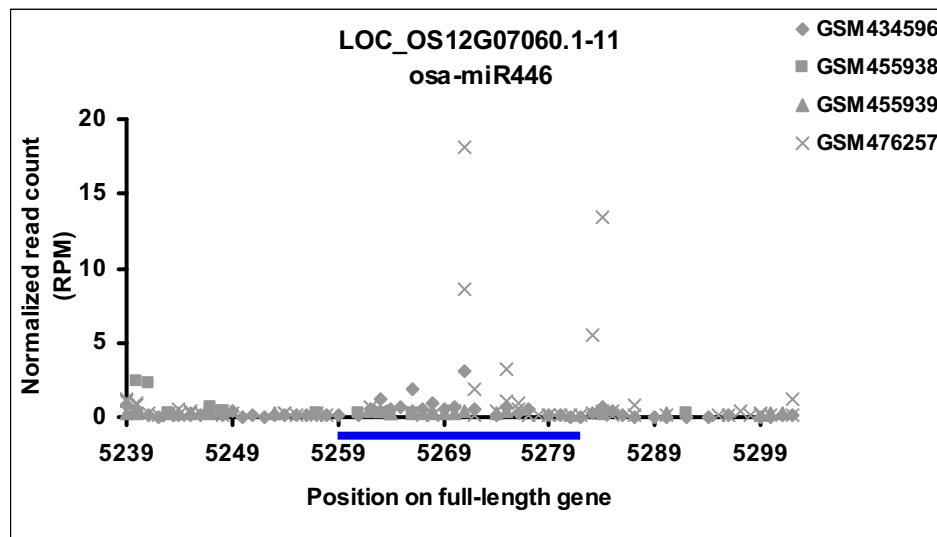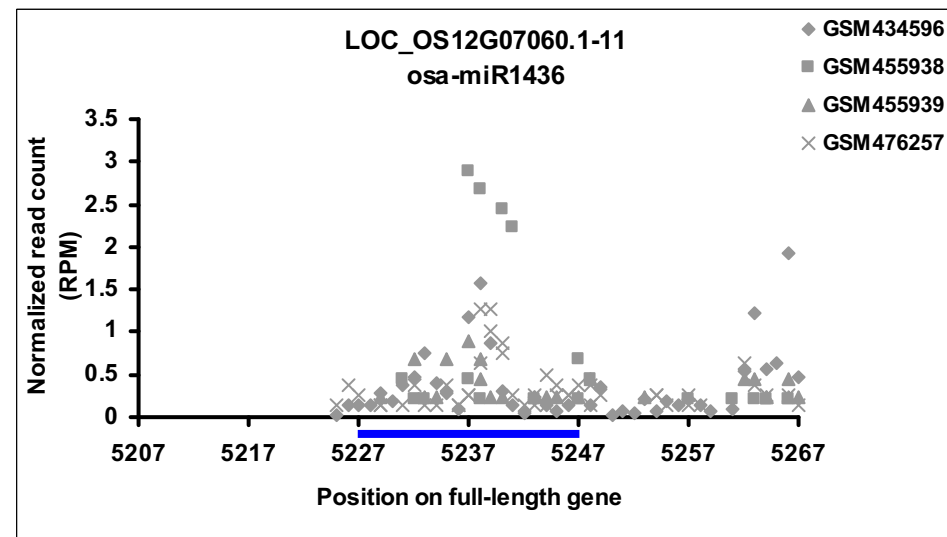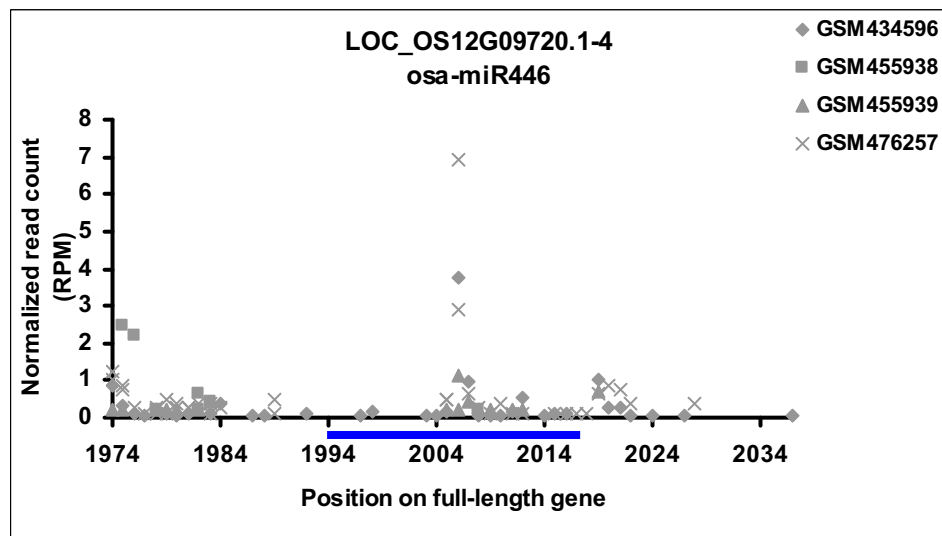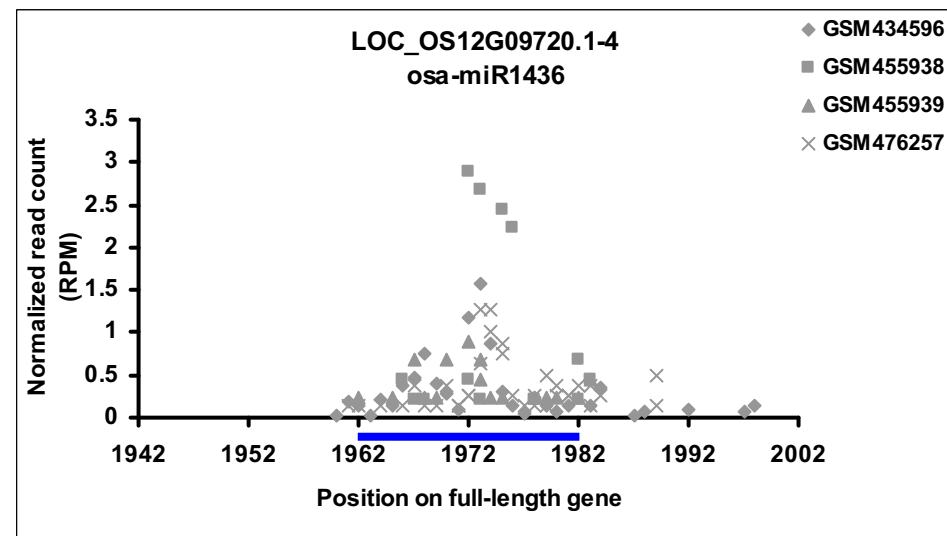

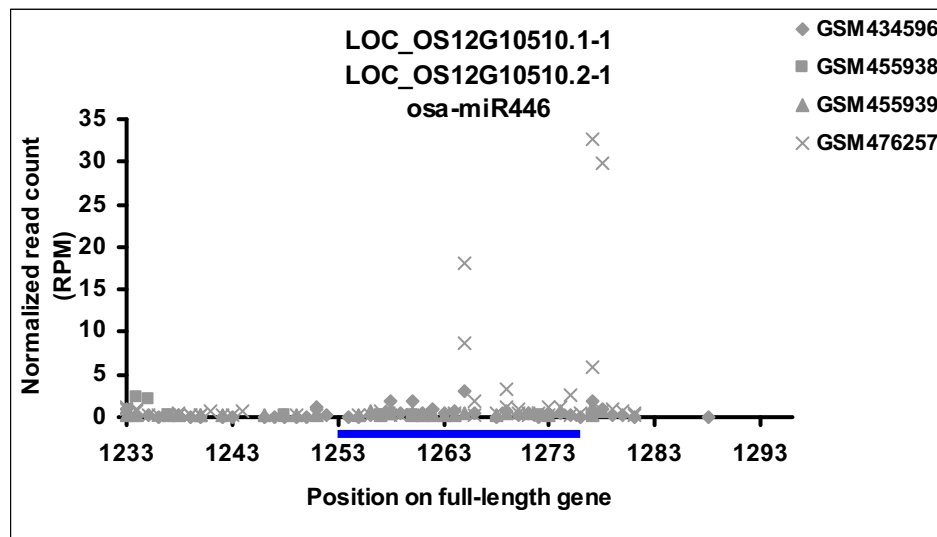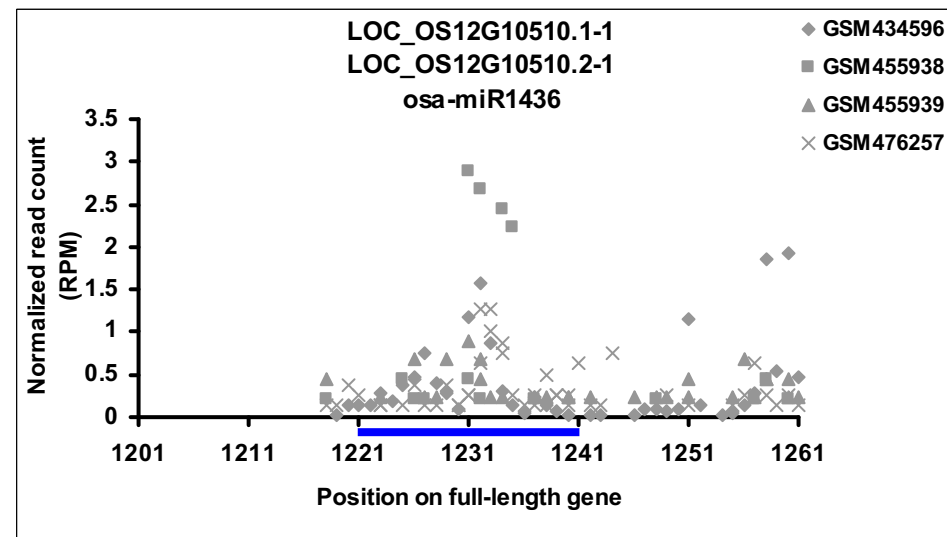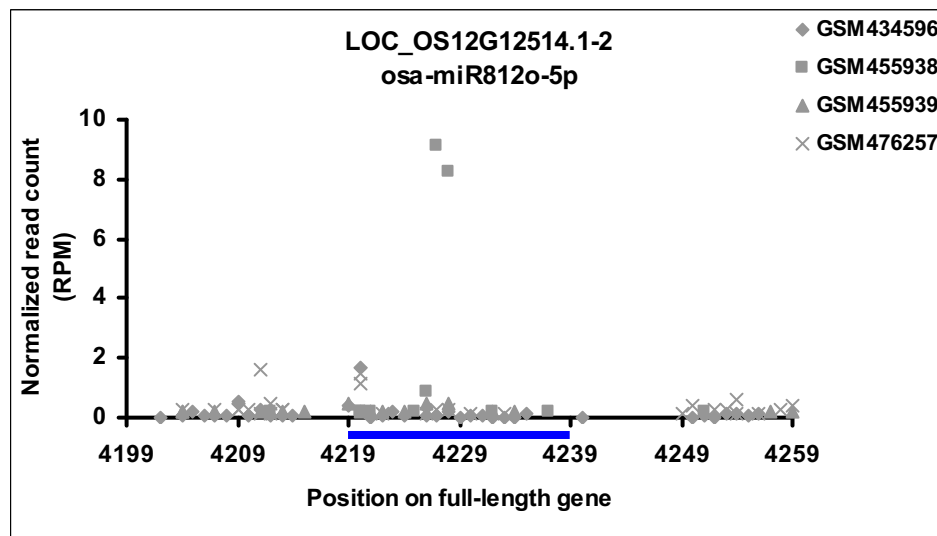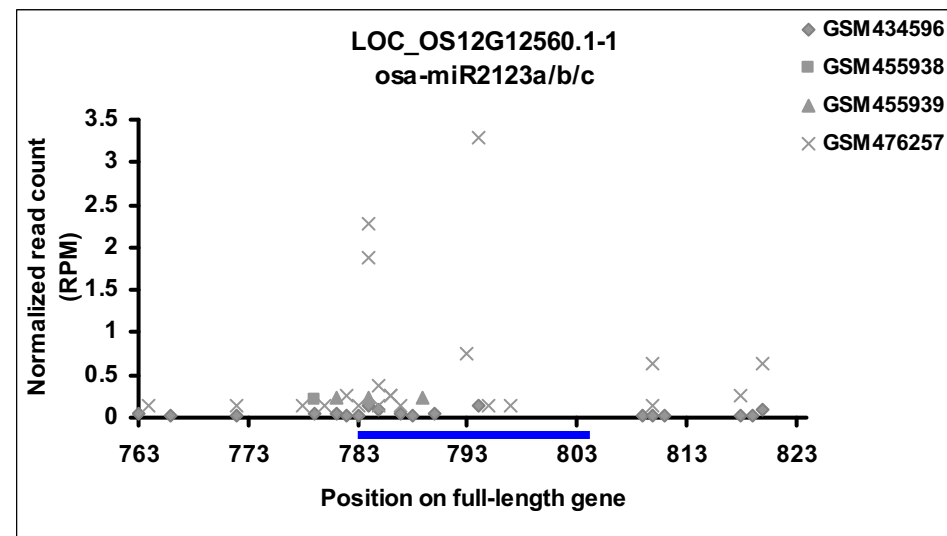

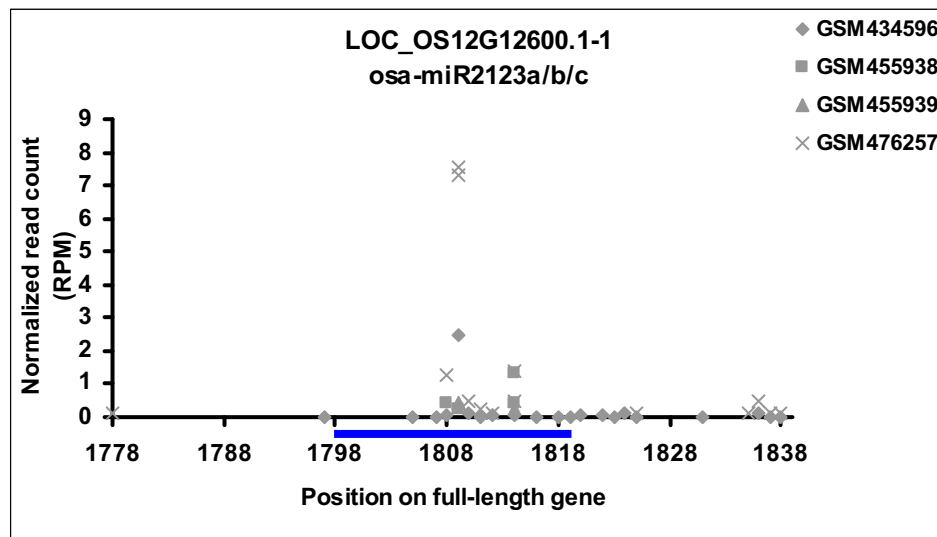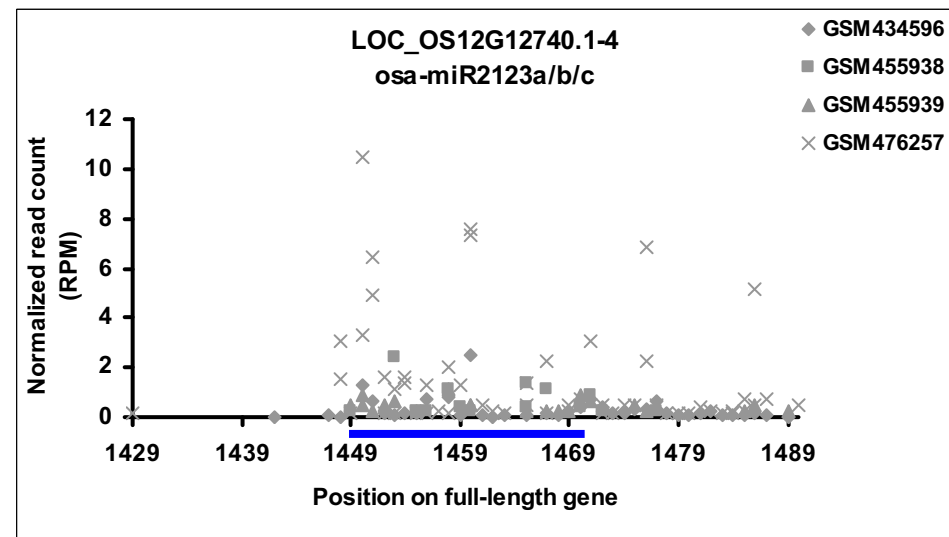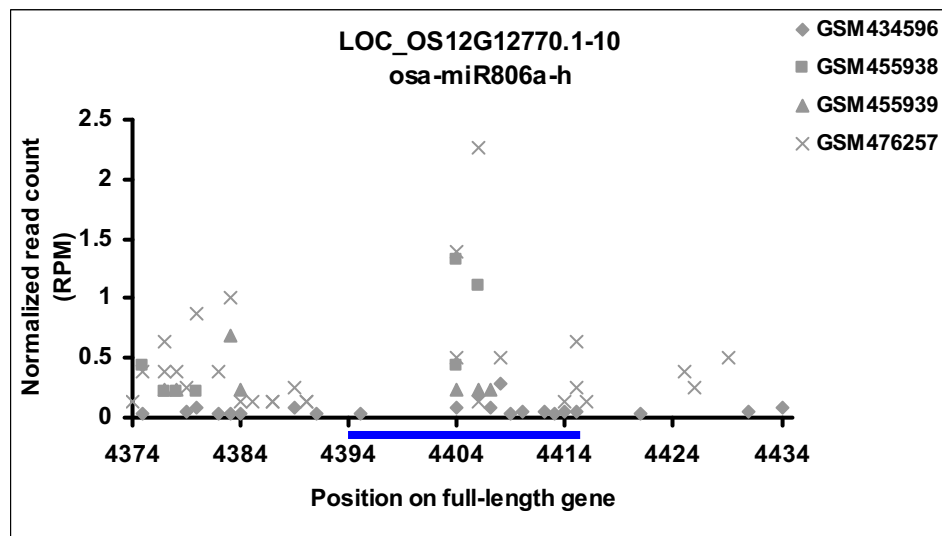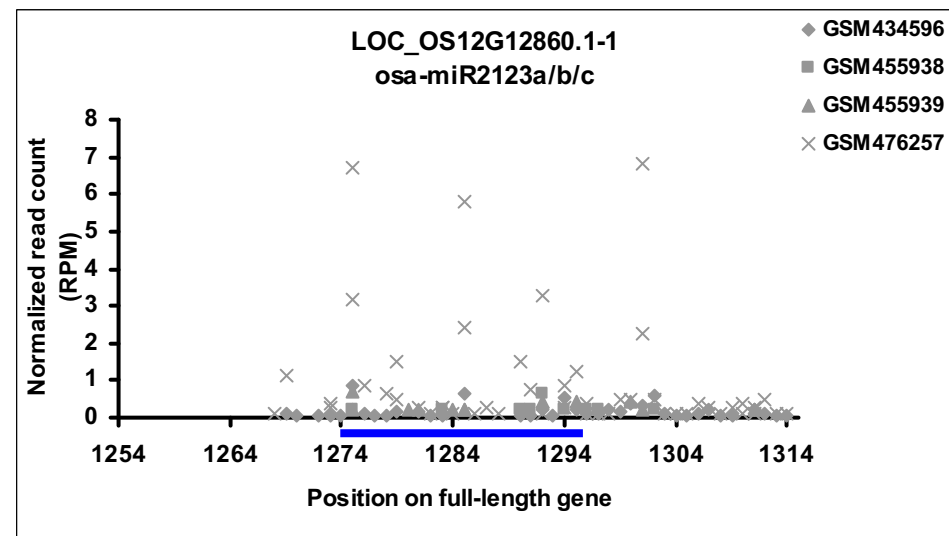

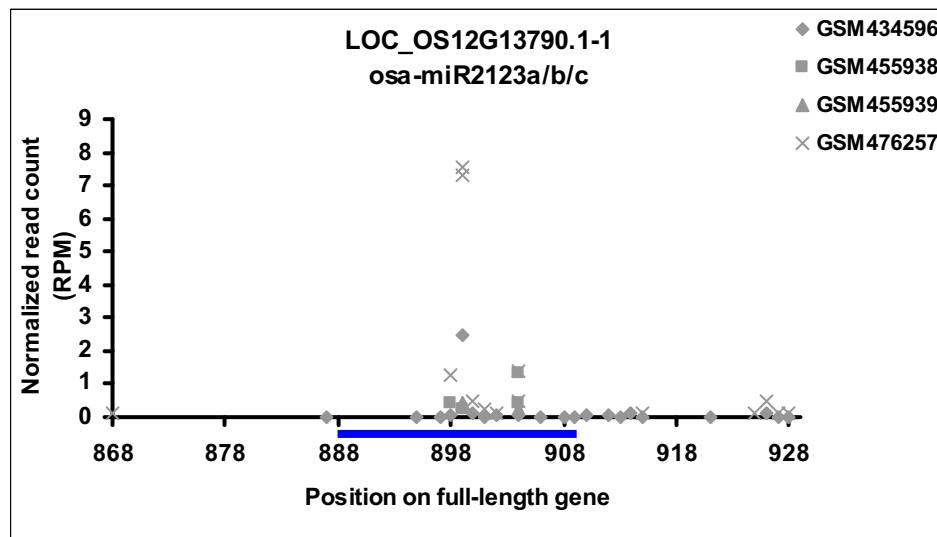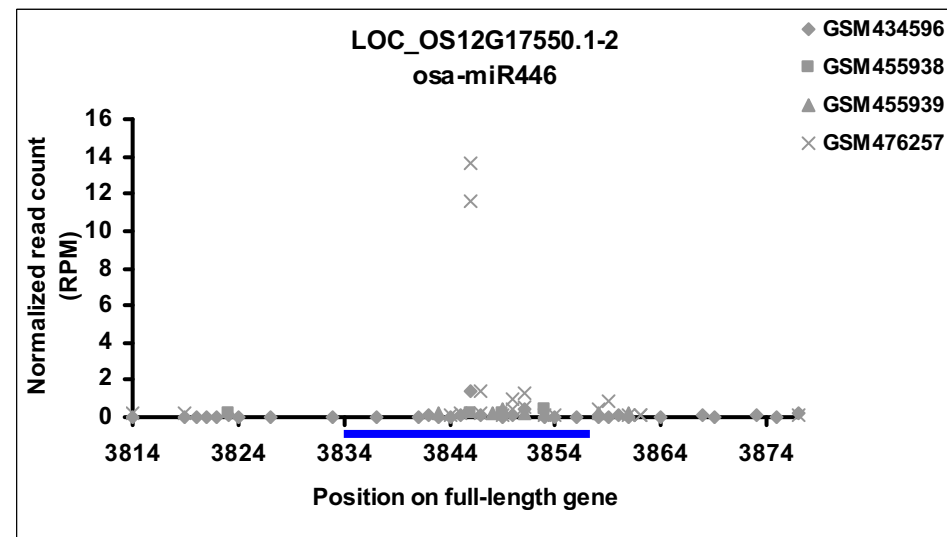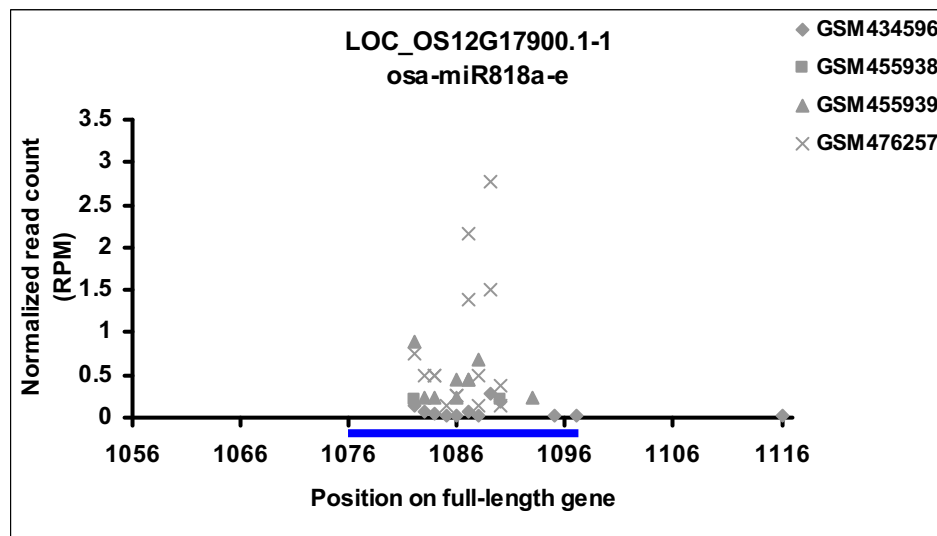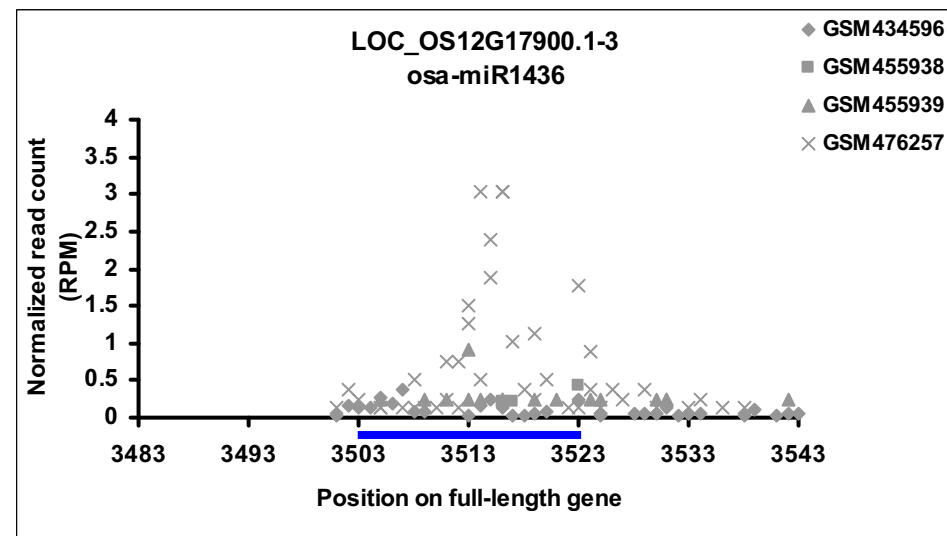

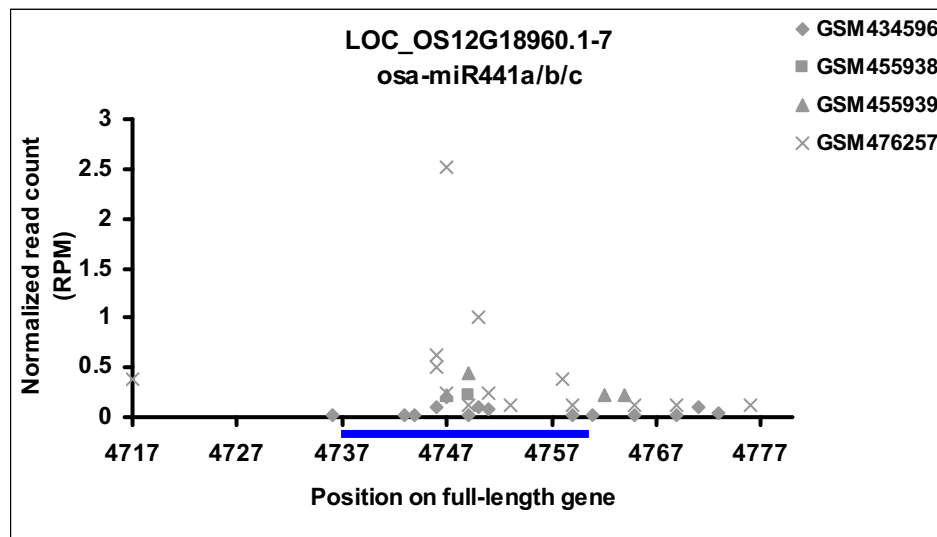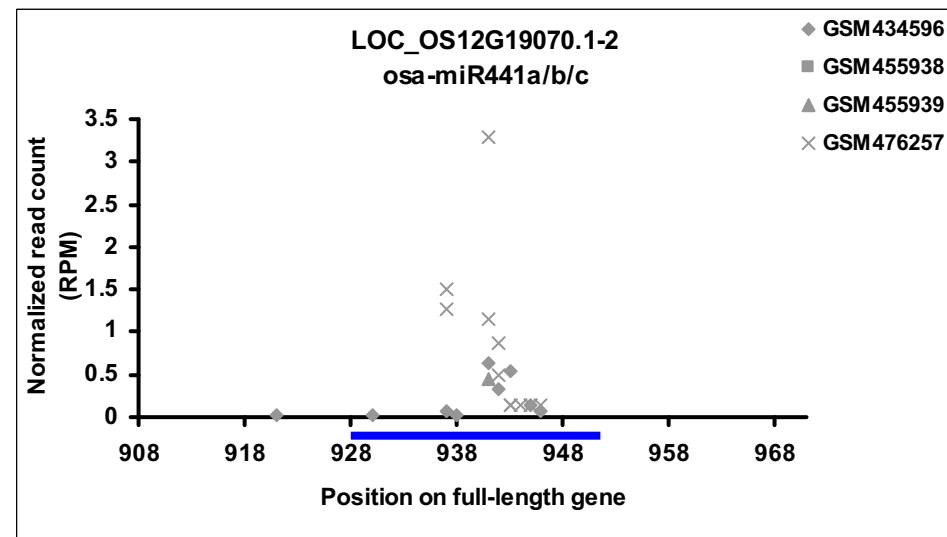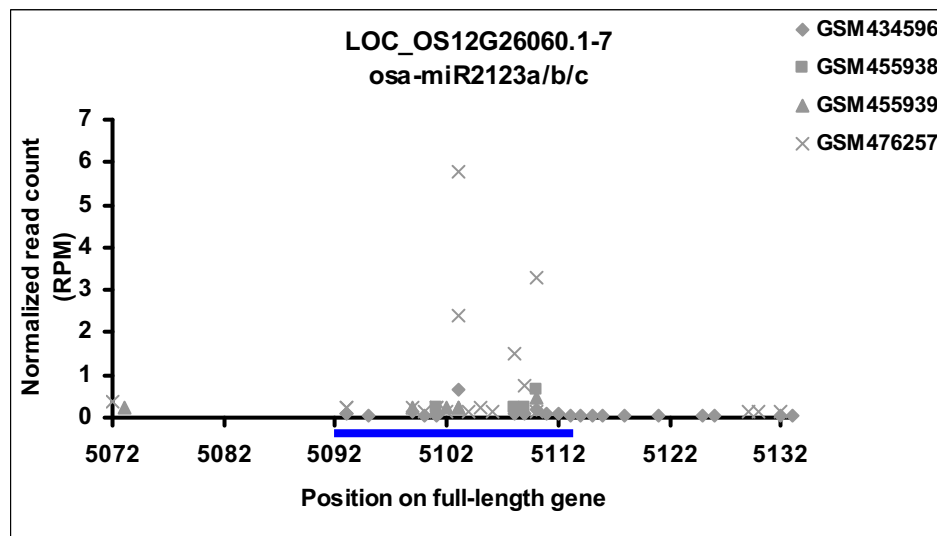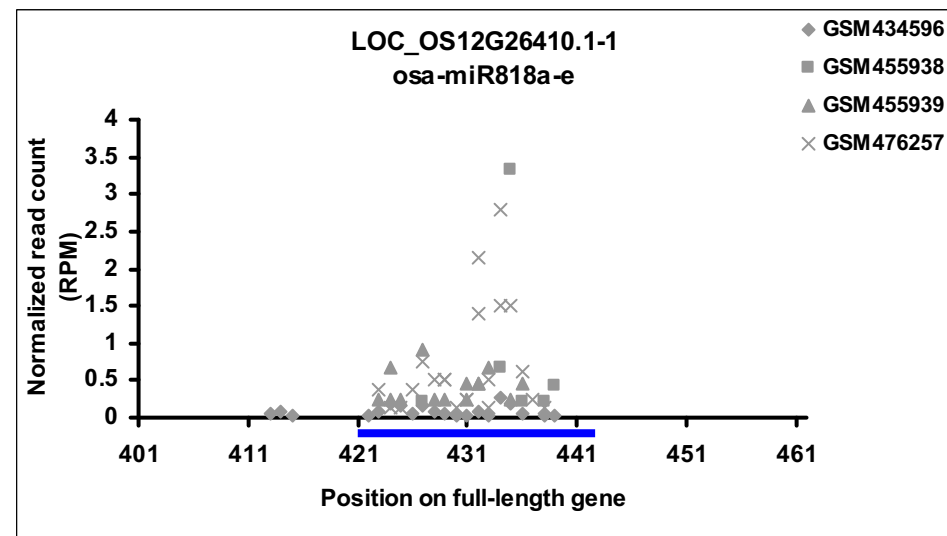

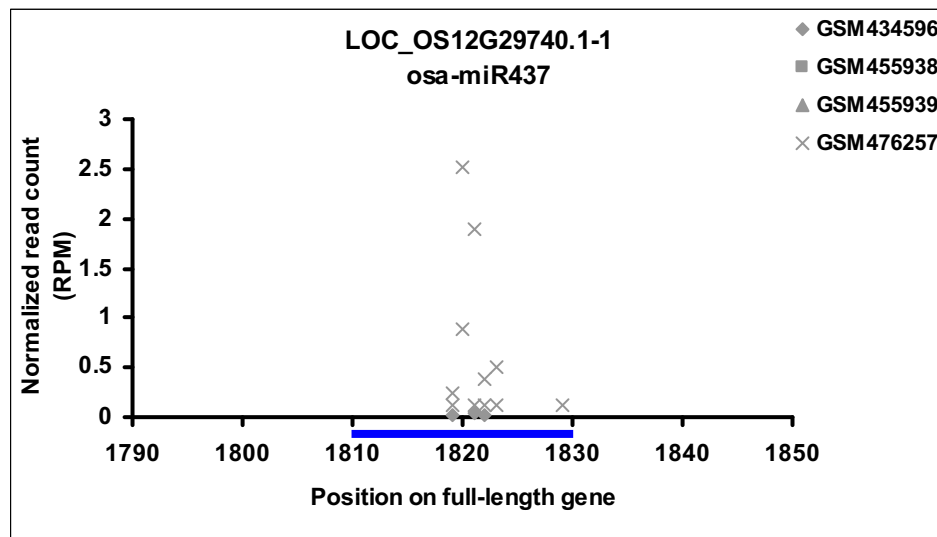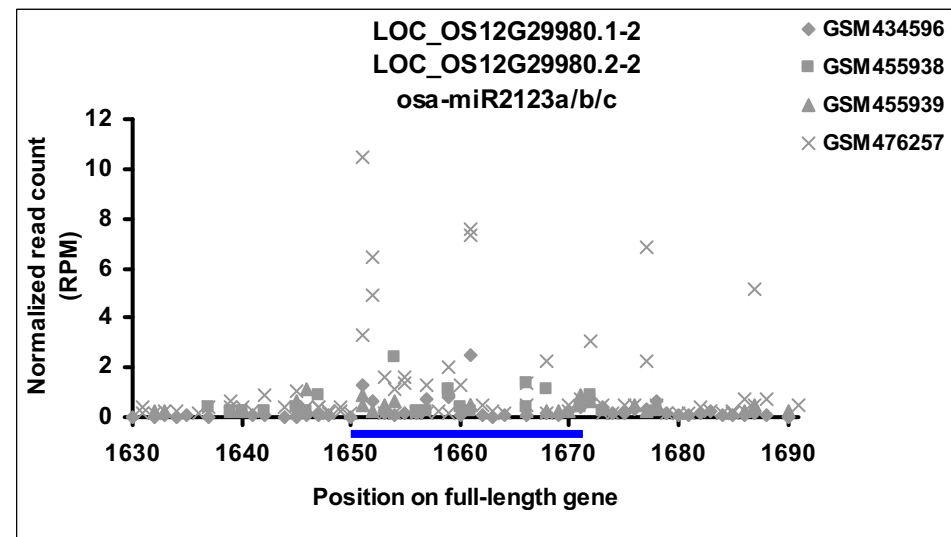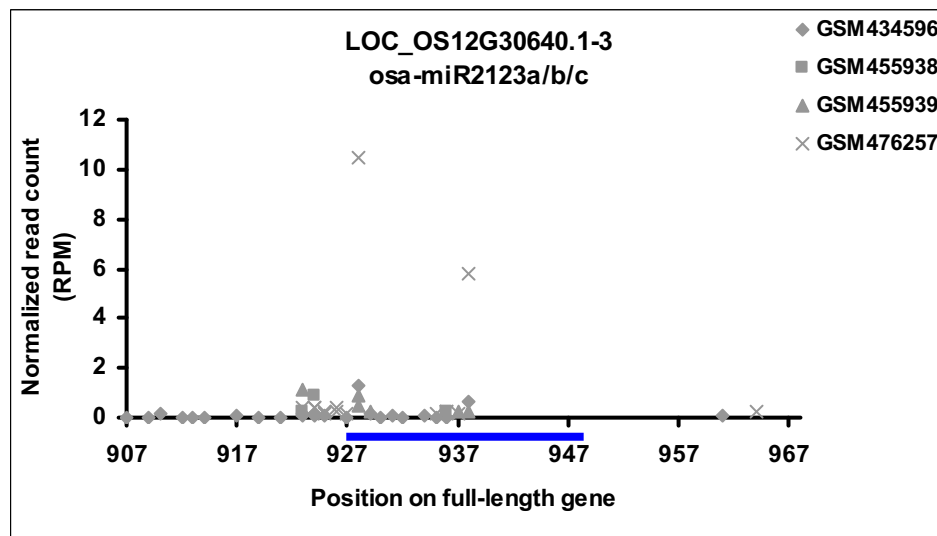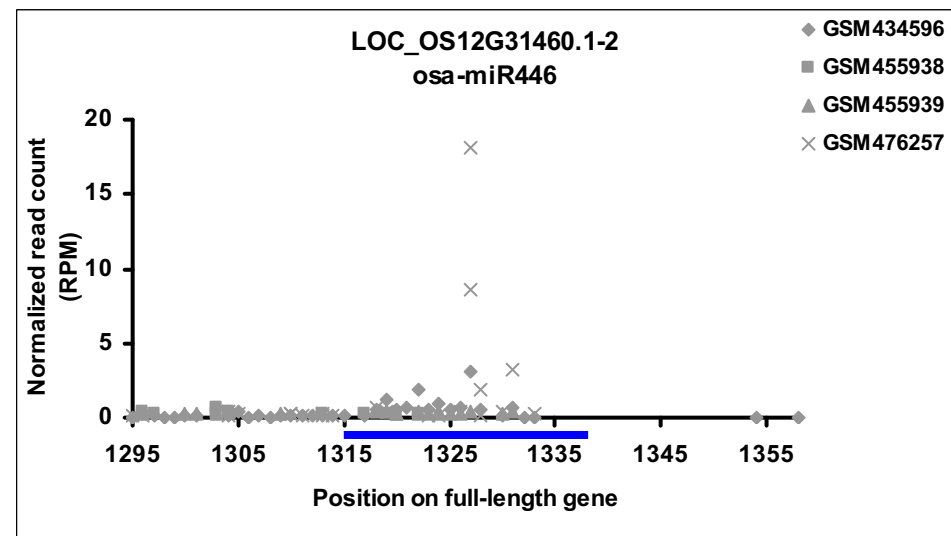

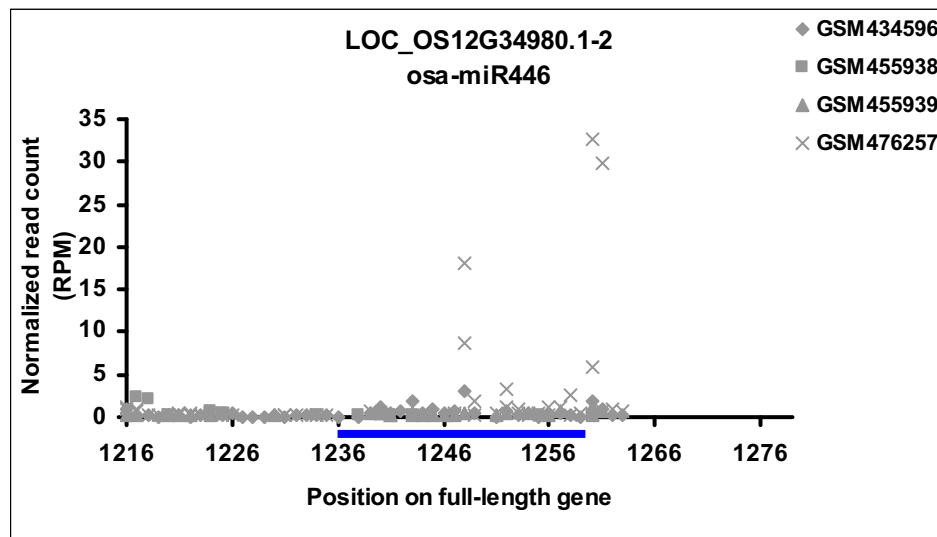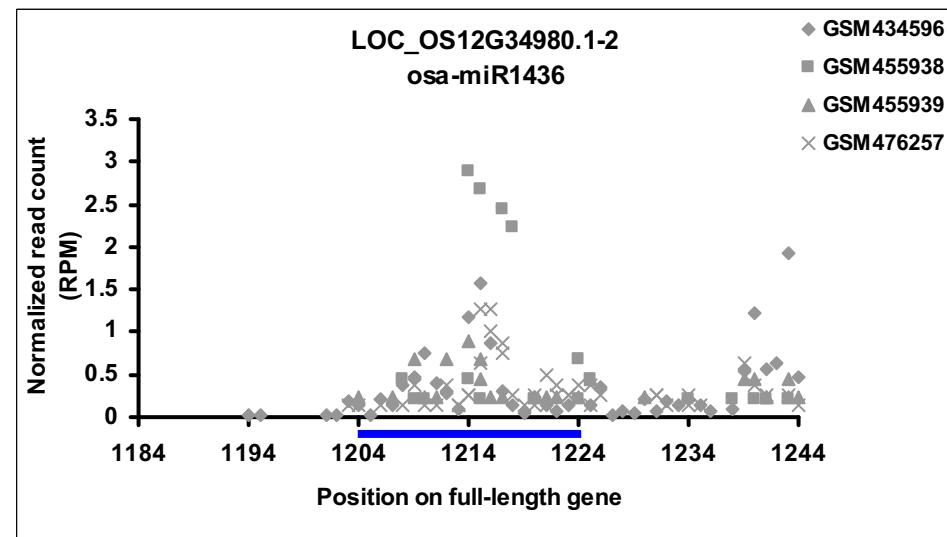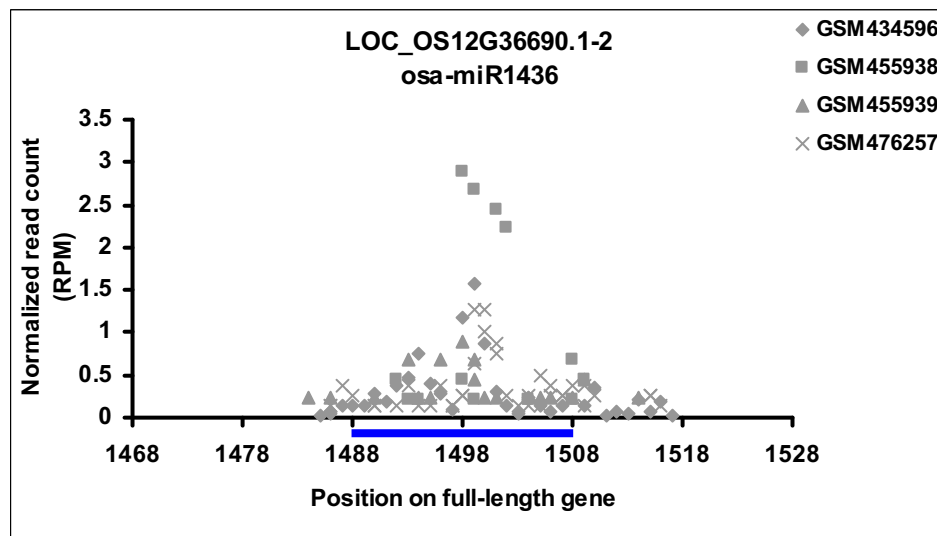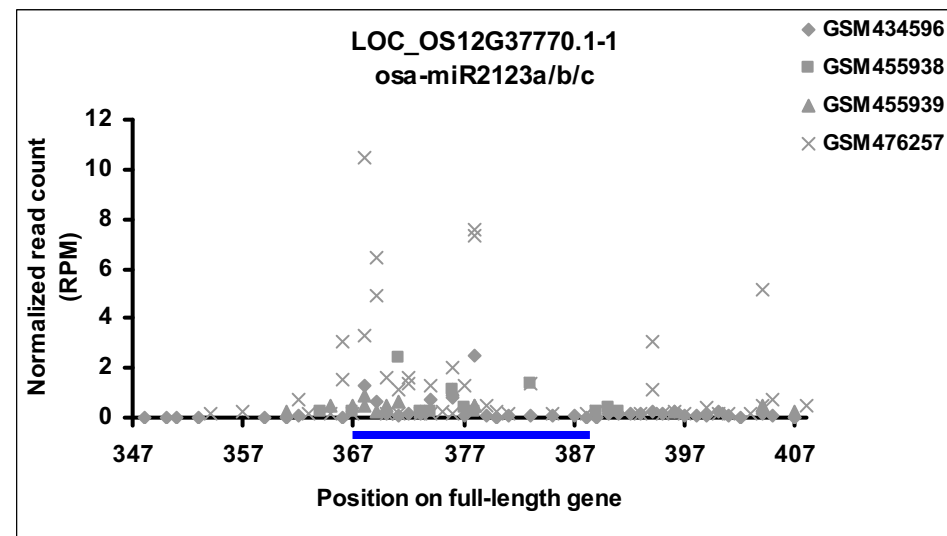

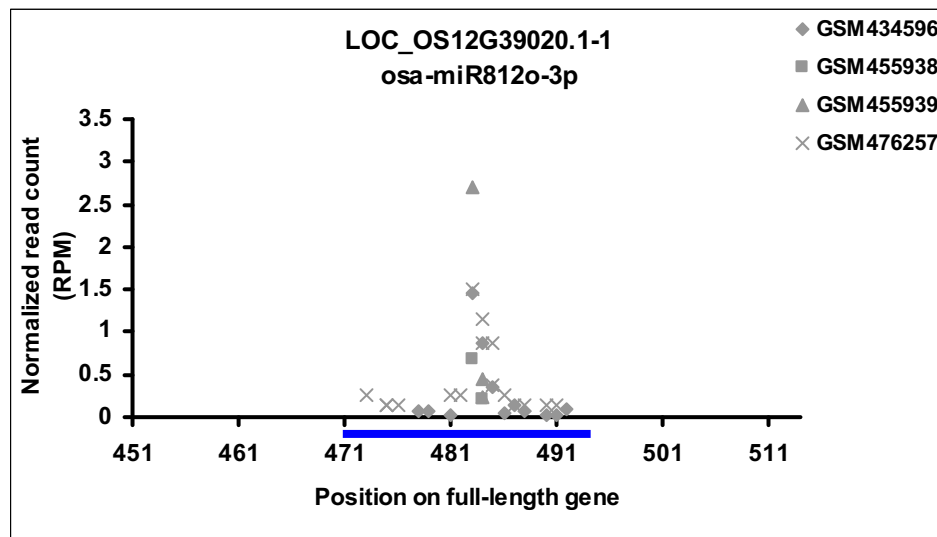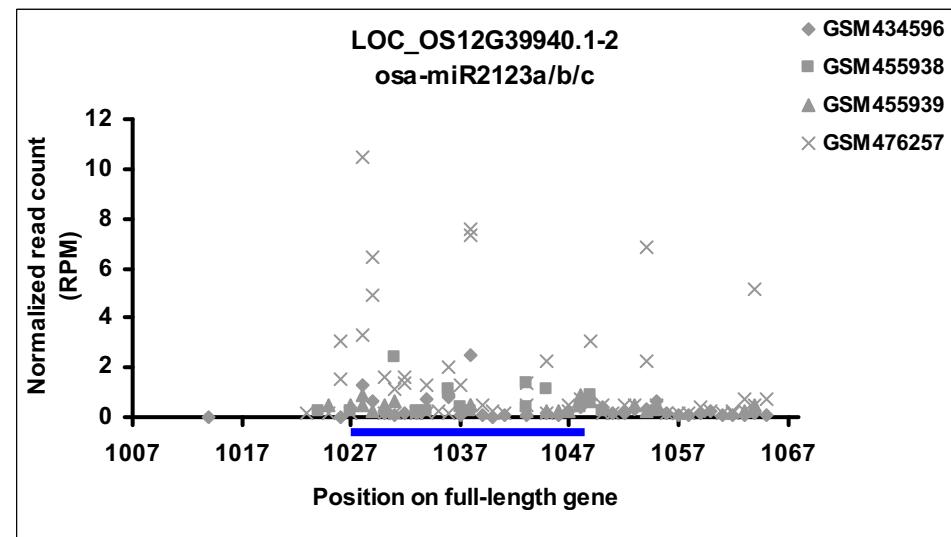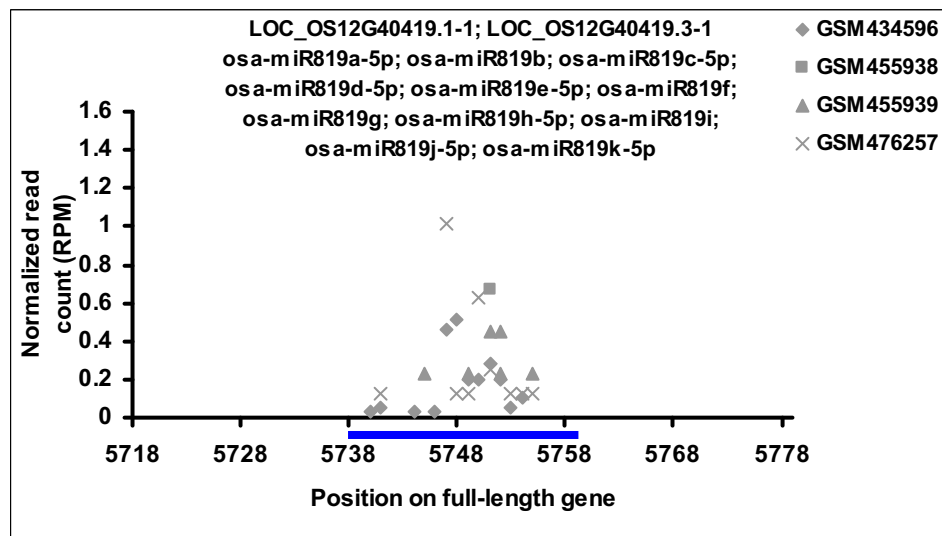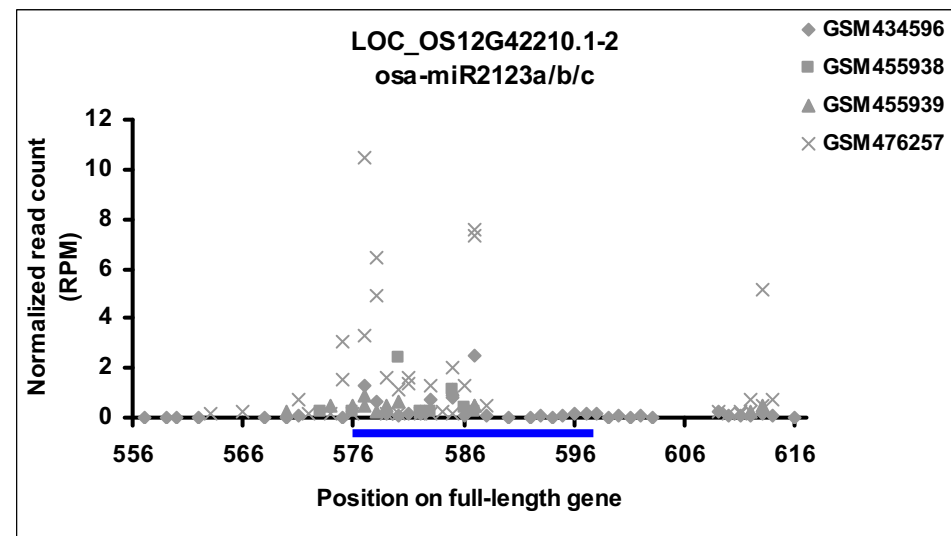

Supplement: Supplementary file 2 — Additional file 2: Figure S2: Global views of the degradome sequencing signatures along the full-length target genes of Arabidopsis. (PDF 2 MB) [file 12284_2012_45_MOESM2_ESM.pdf]

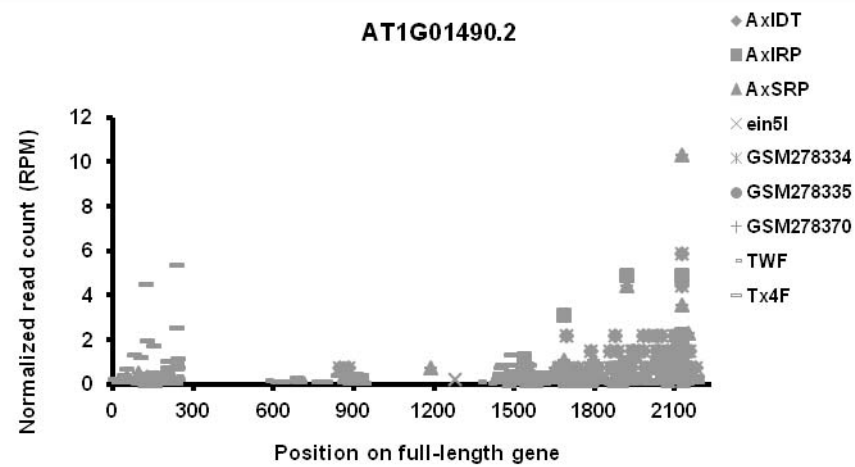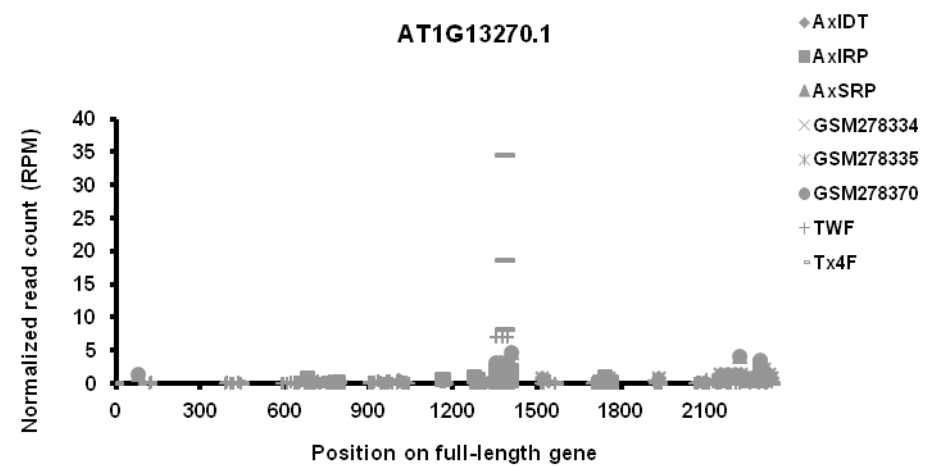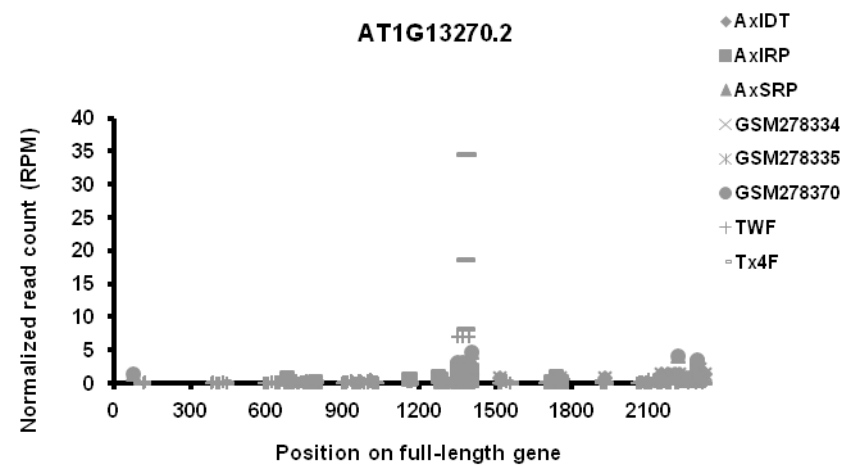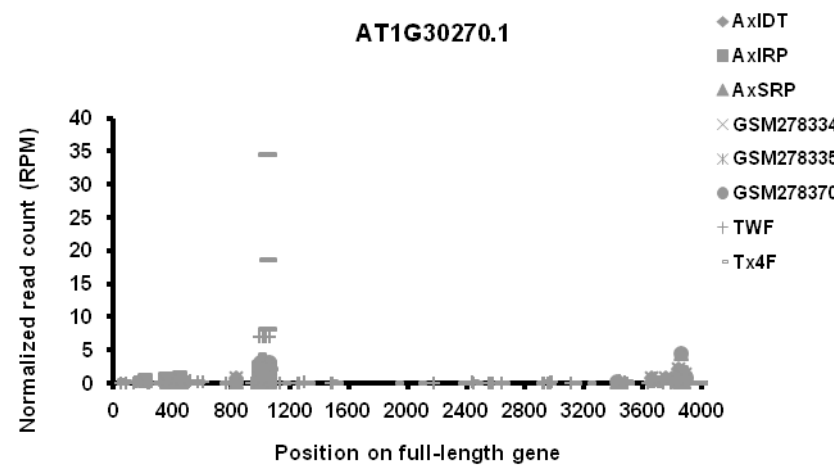

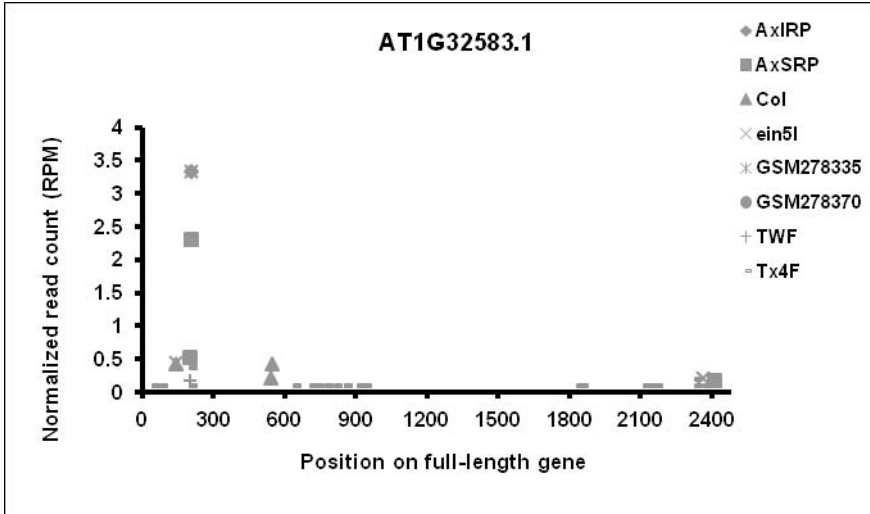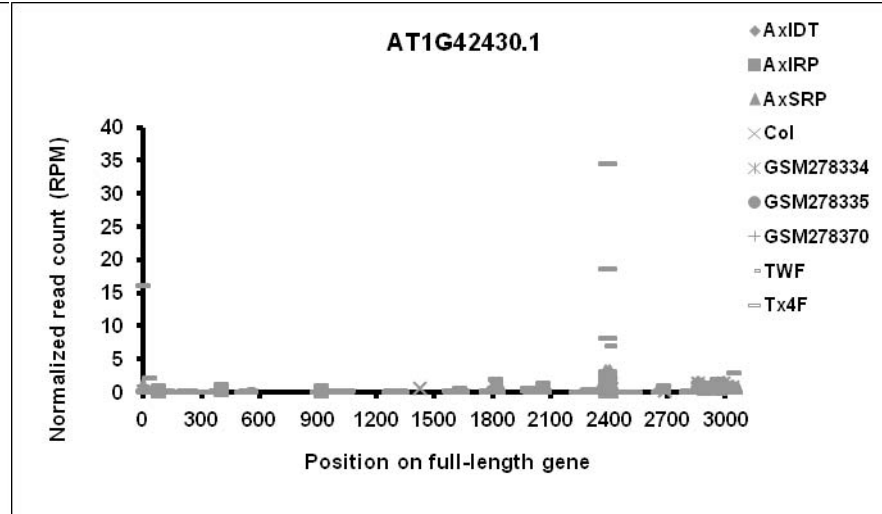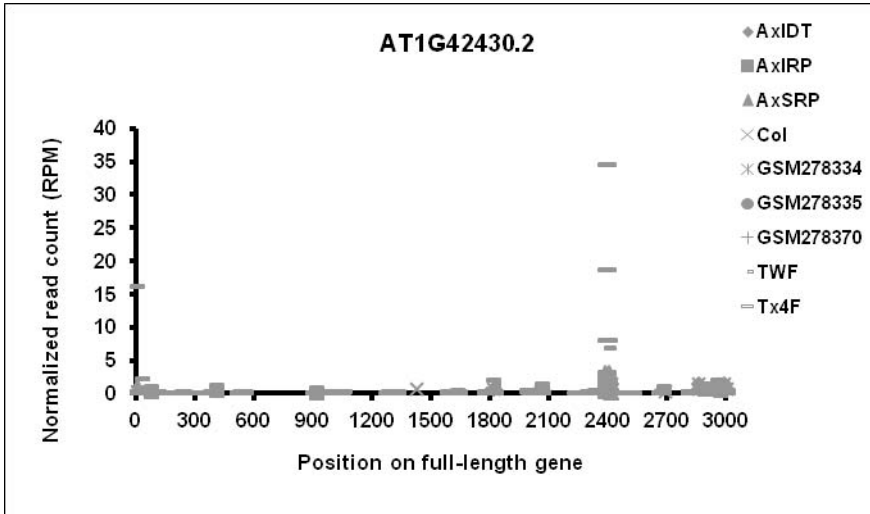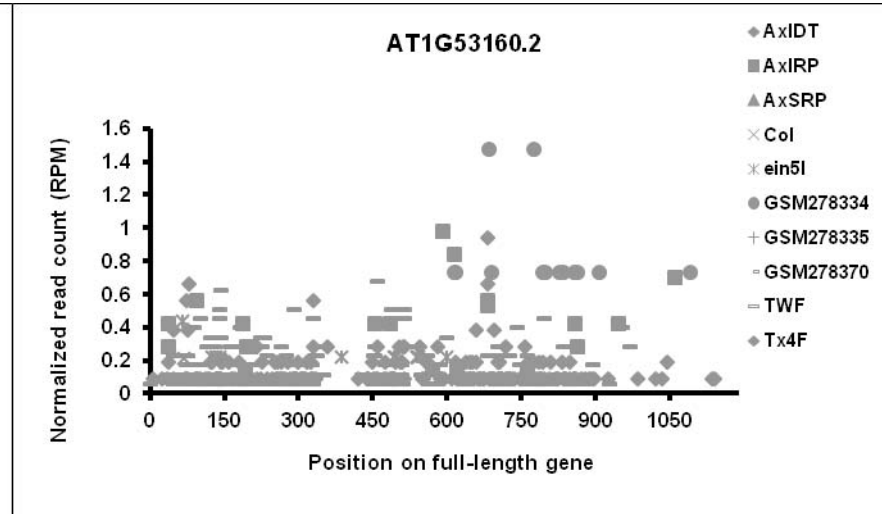

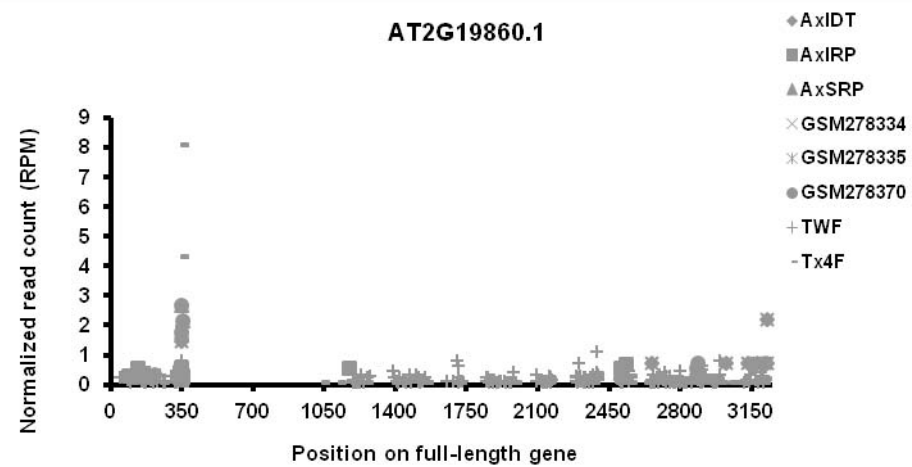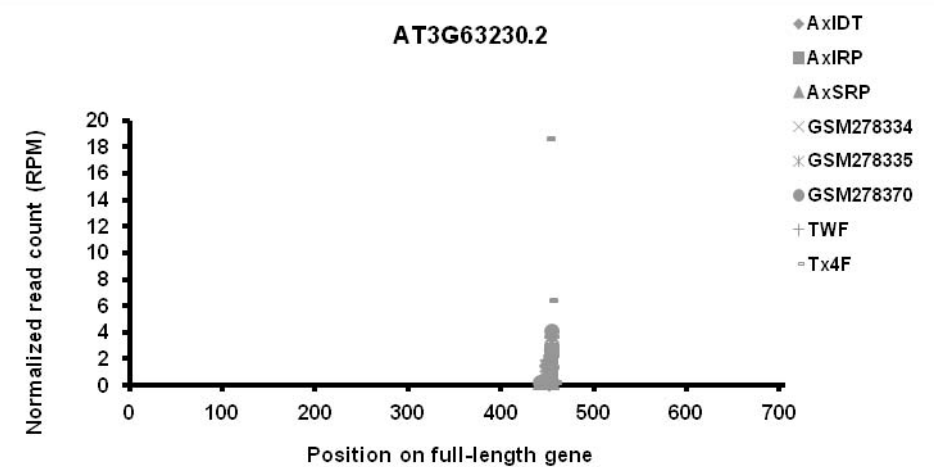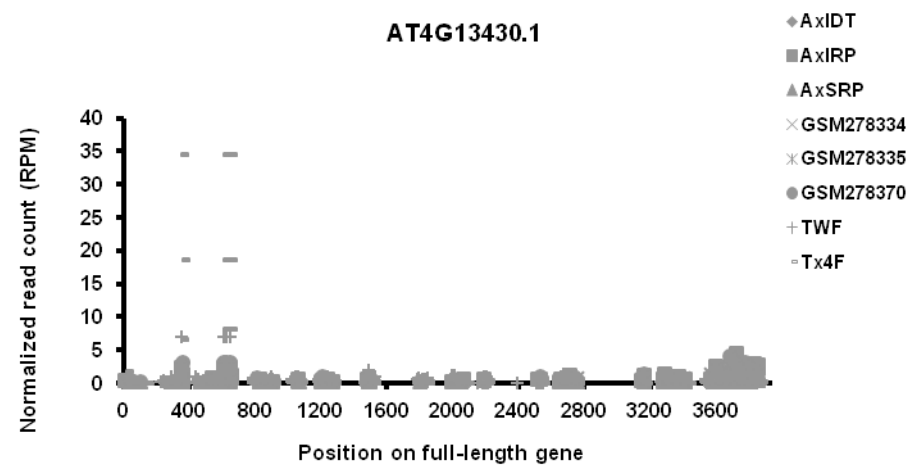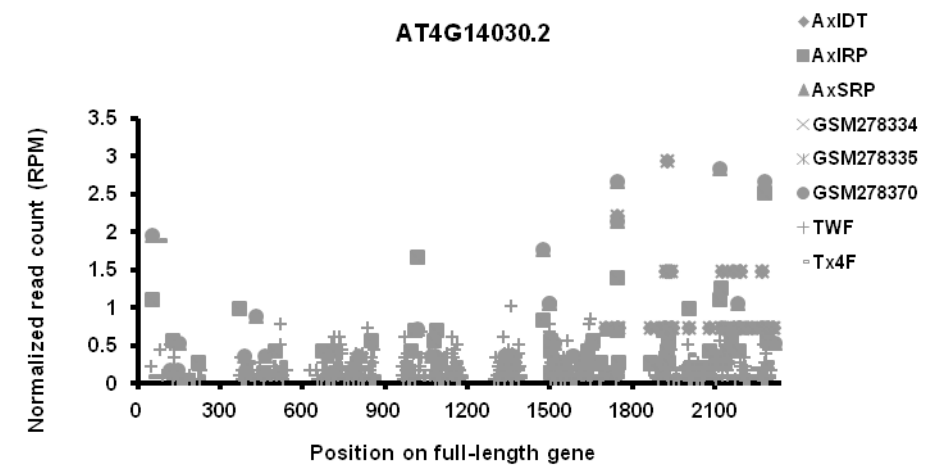

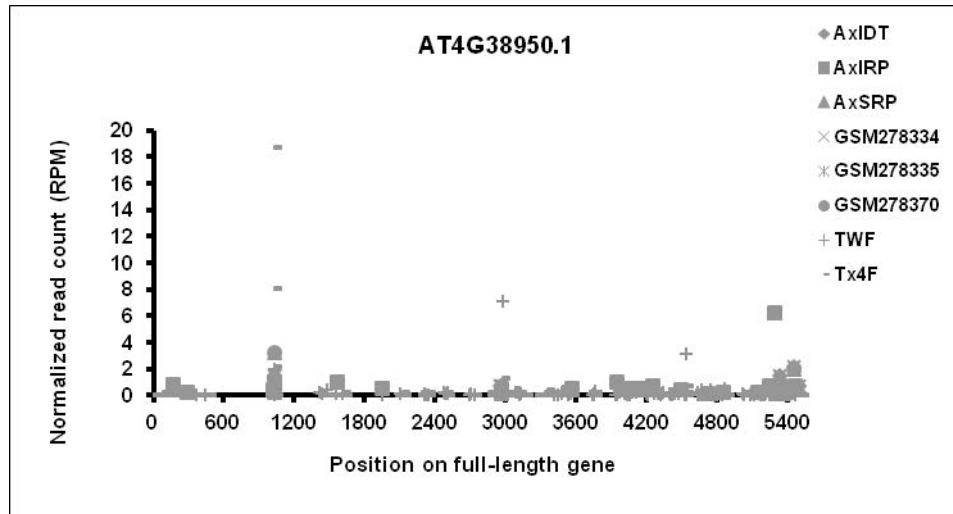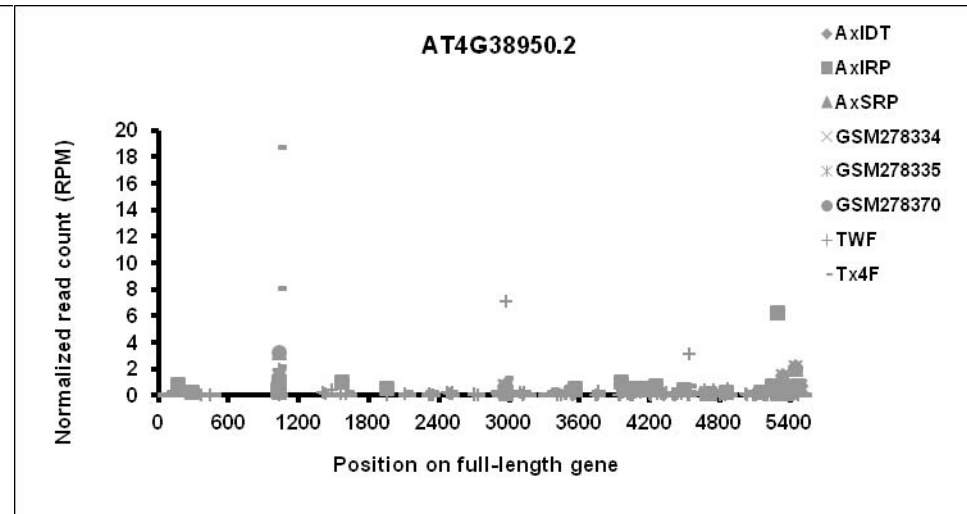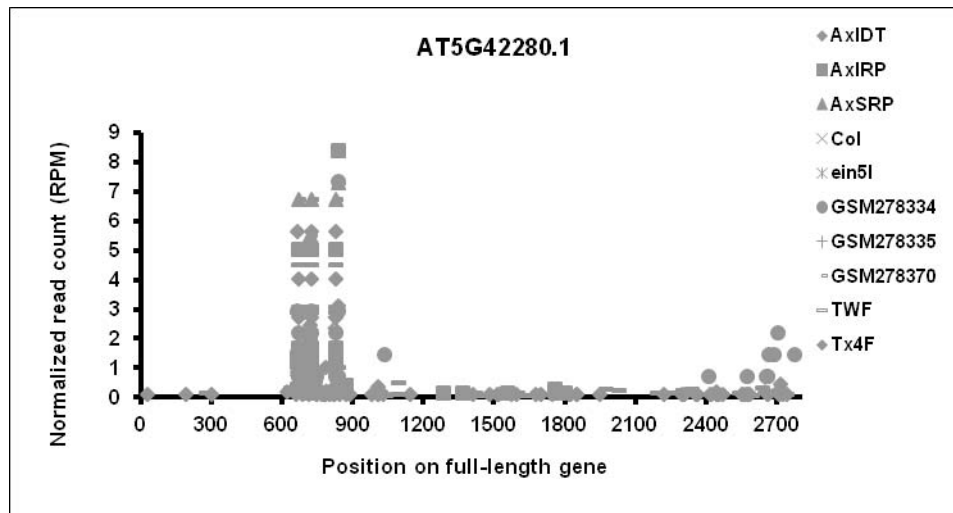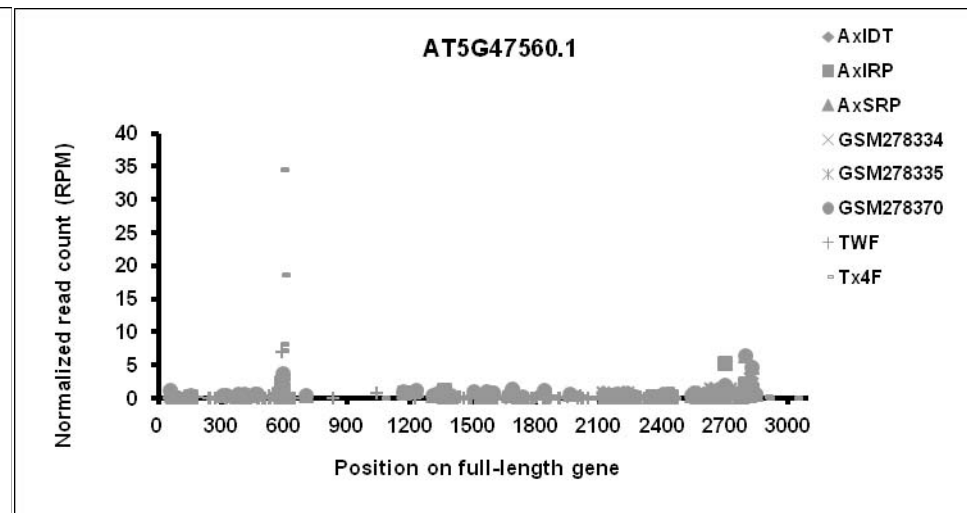

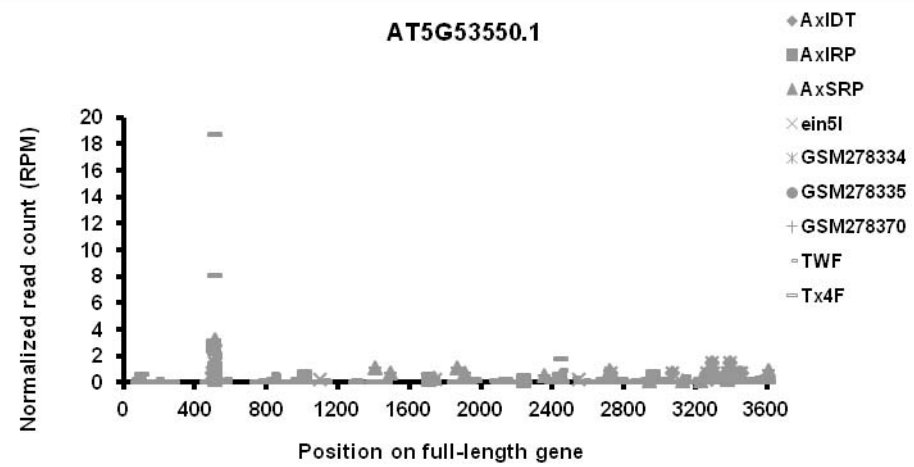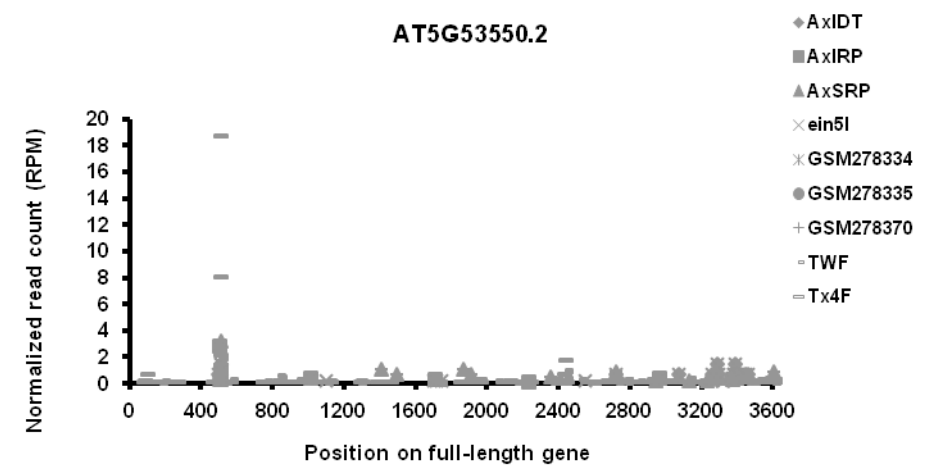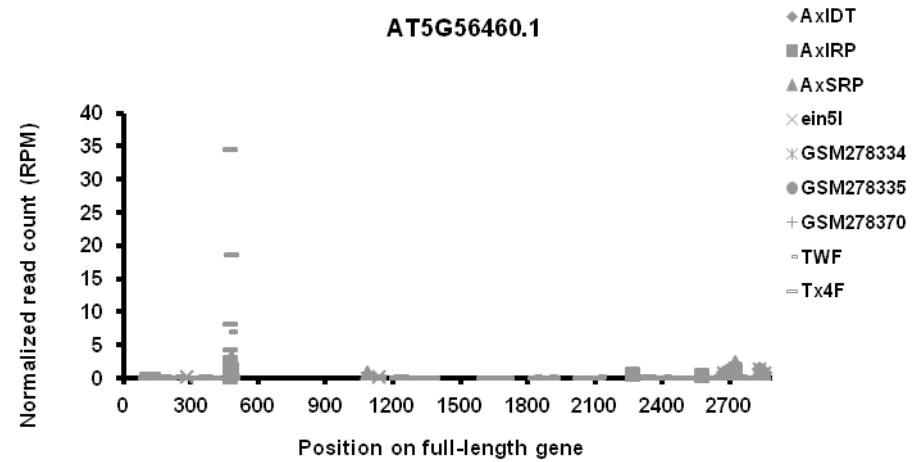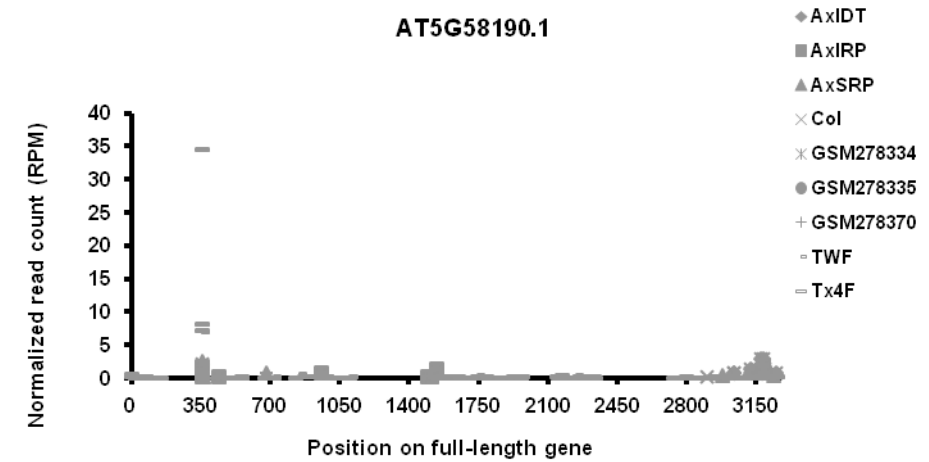

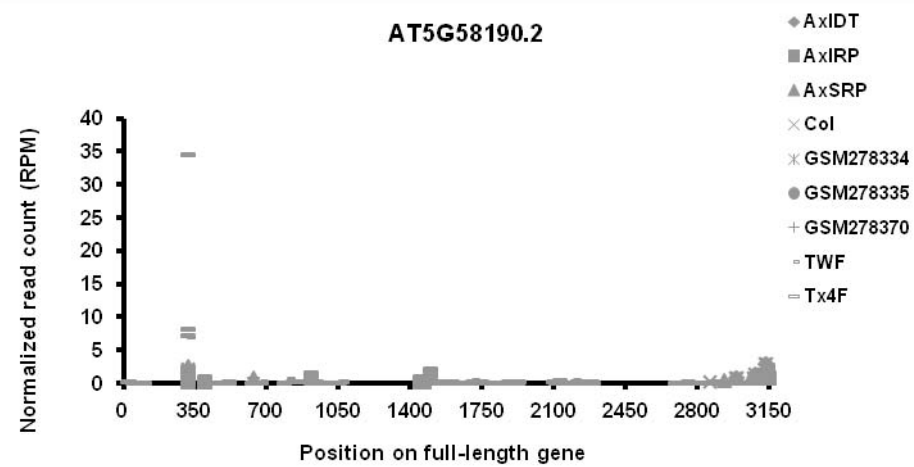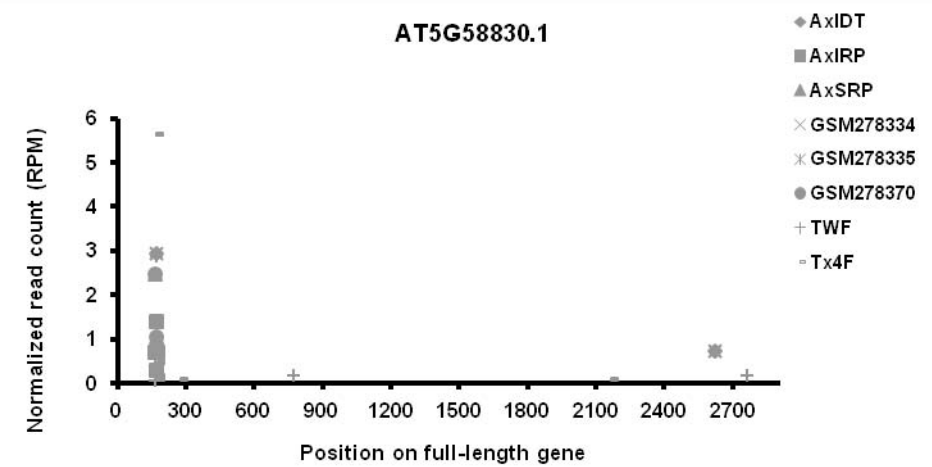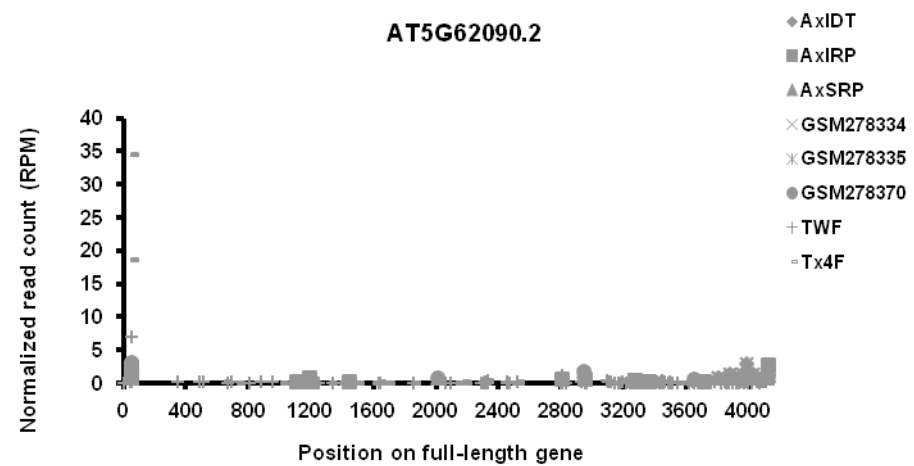

Supplement: Supplementary file 3 — Additional file 3: Figure S3: Target plot-based validation of the microRNA—intron interactions in rice. Only the miRNA binding sites (indicated by blue horizontal lines) surrounded by 20-nt sequences at both ends were shown. (PDF 446 KB) [file 12284_2012_45_MOESM3_ESM.pdf]

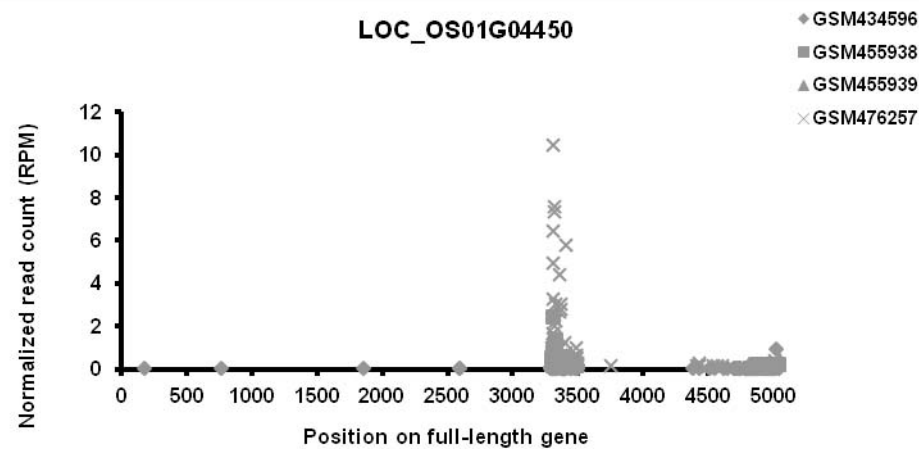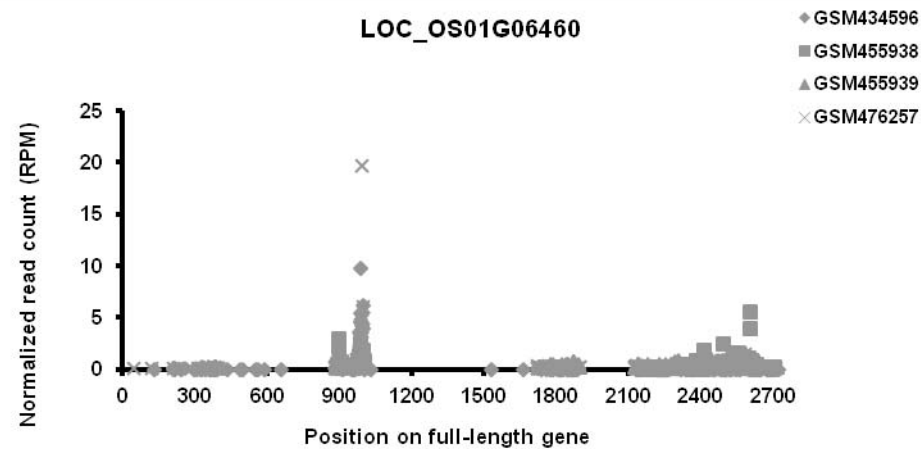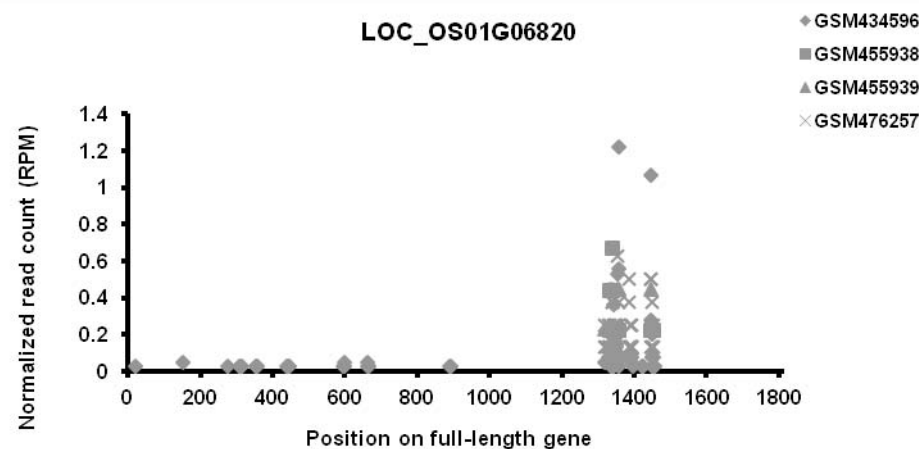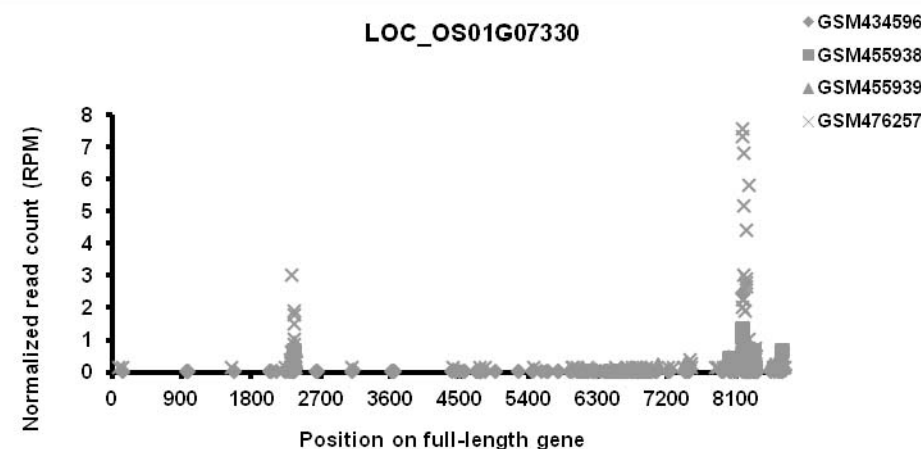

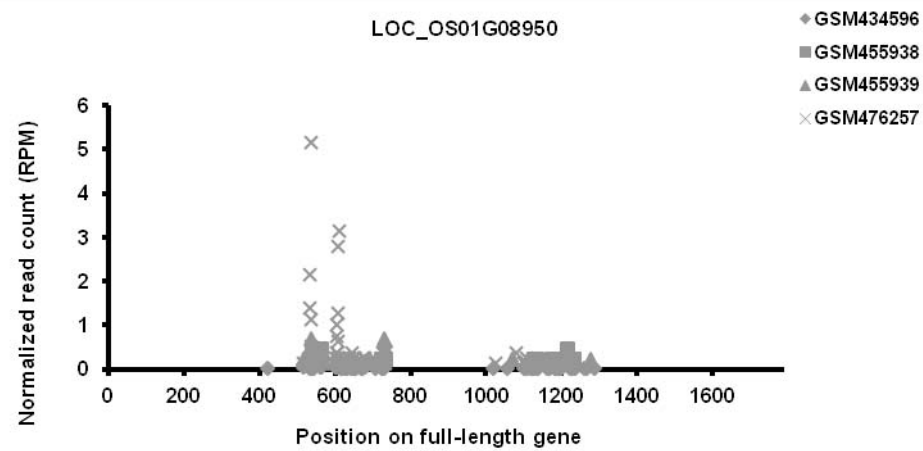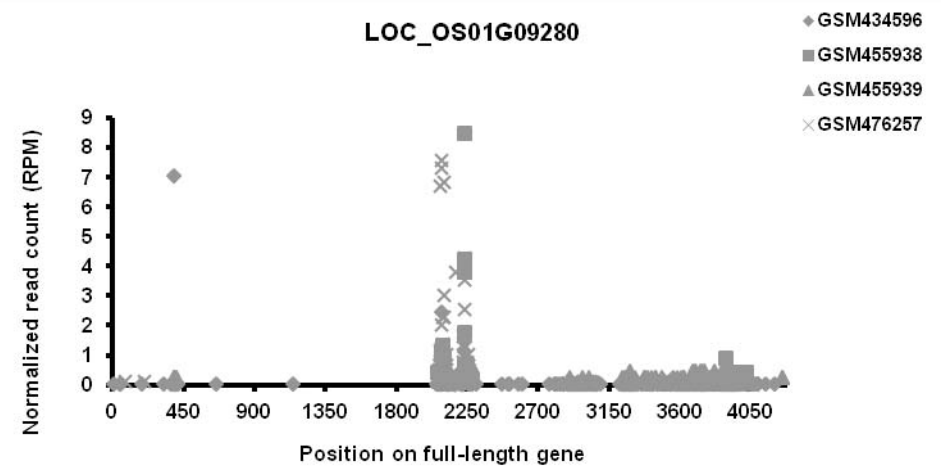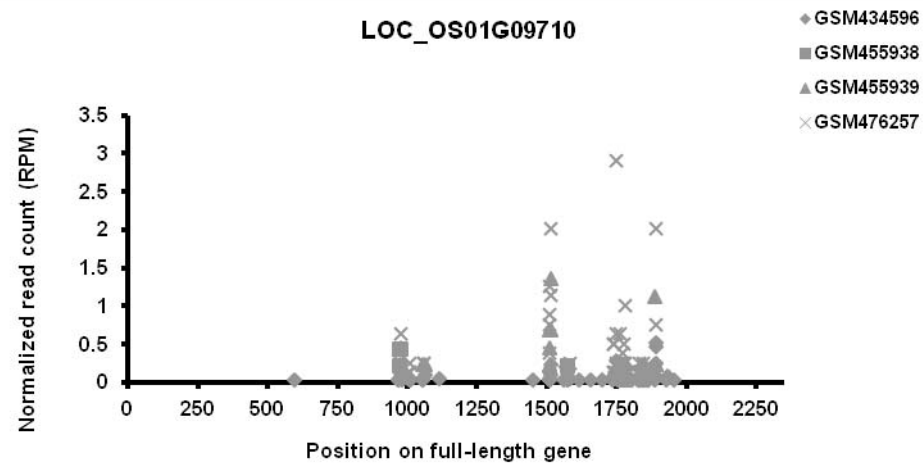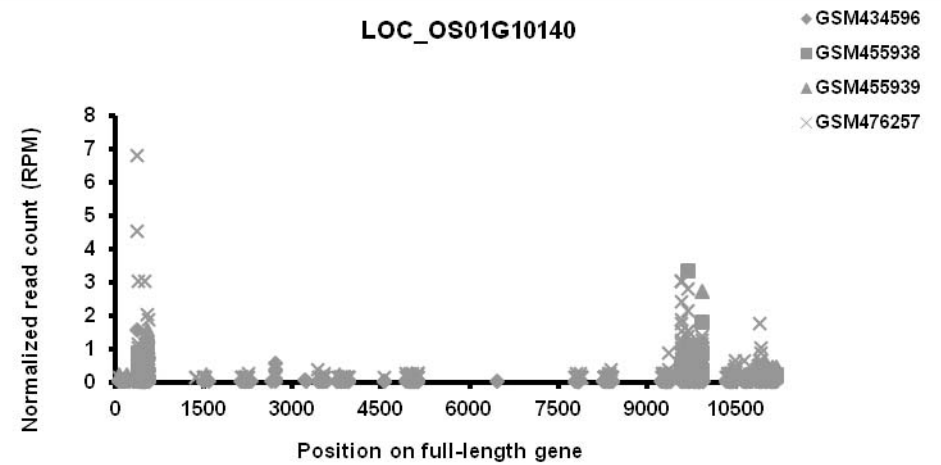

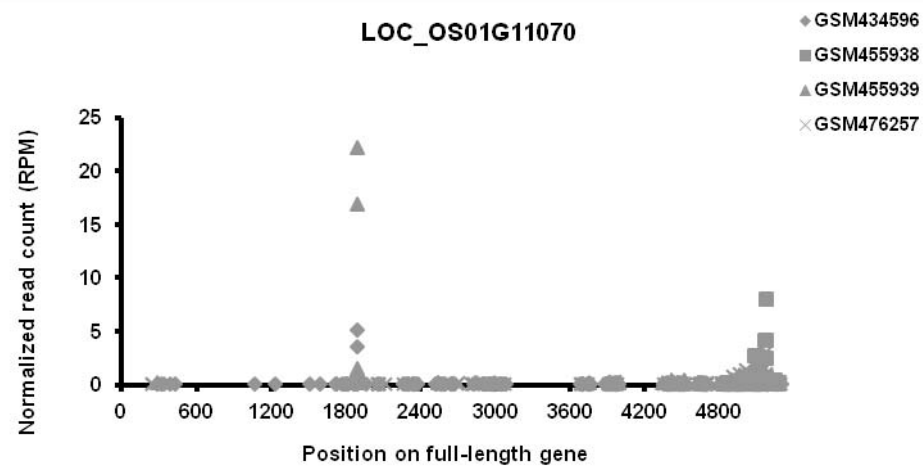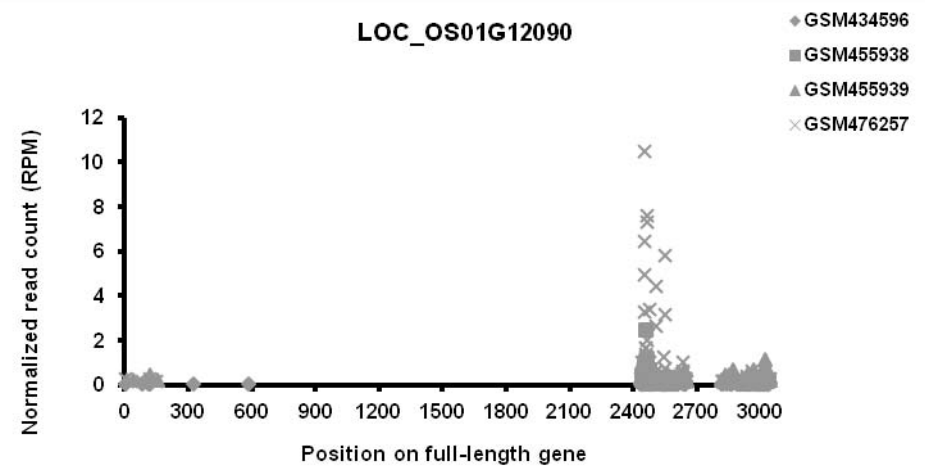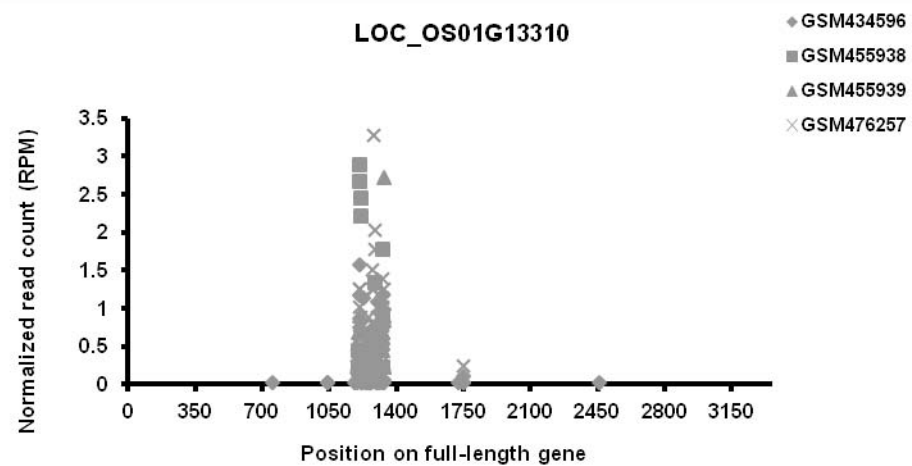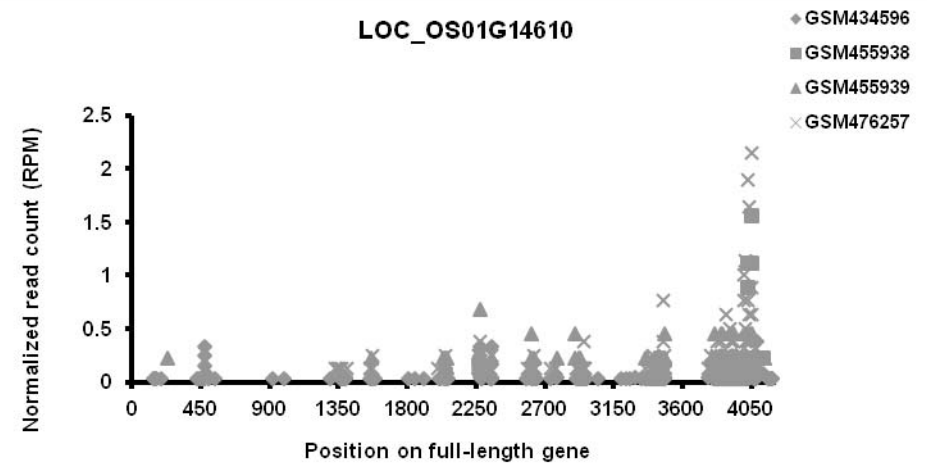

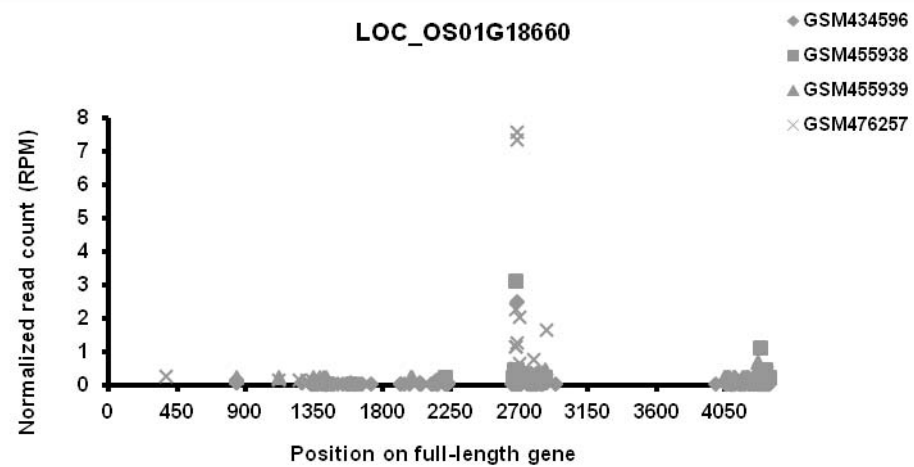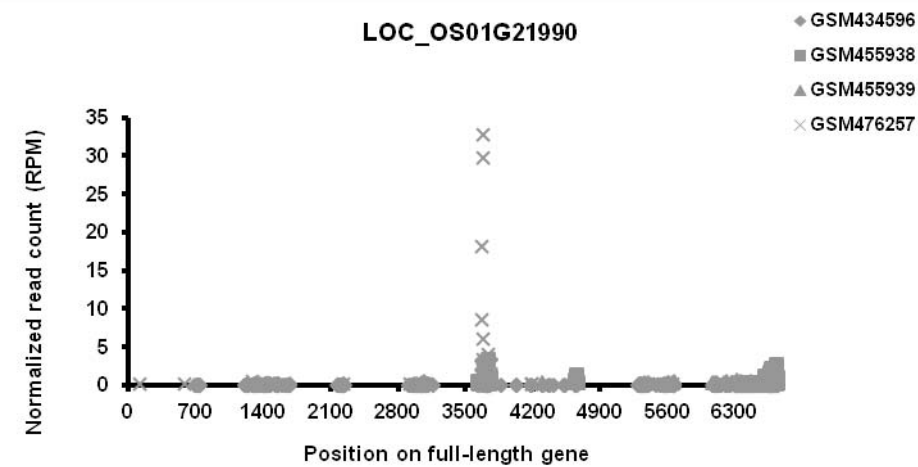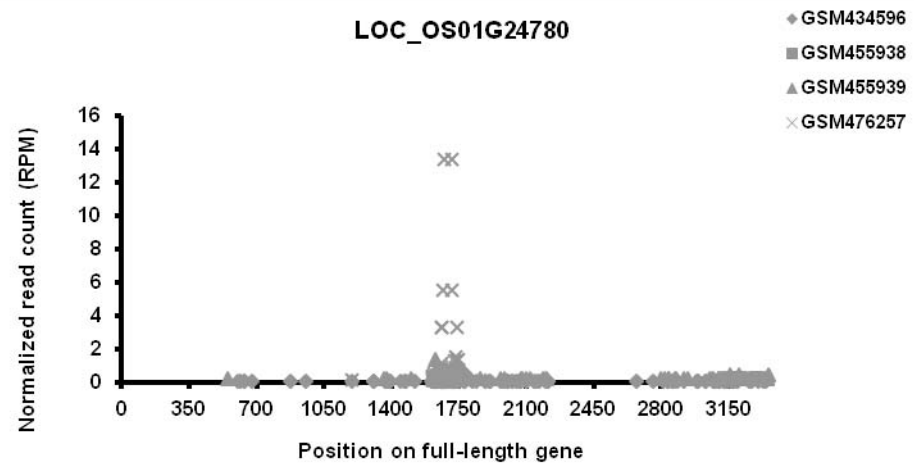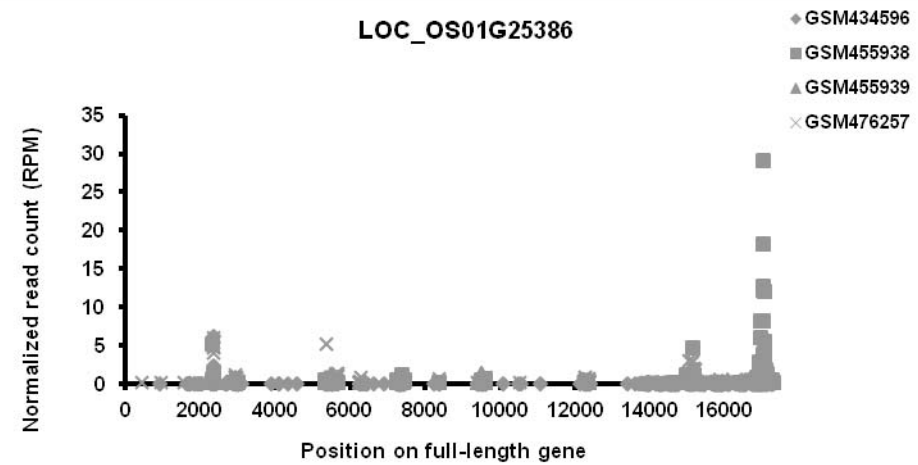

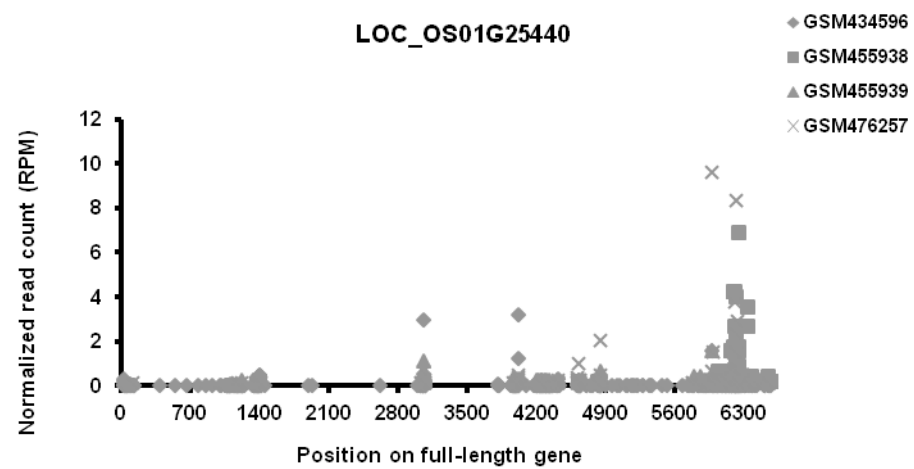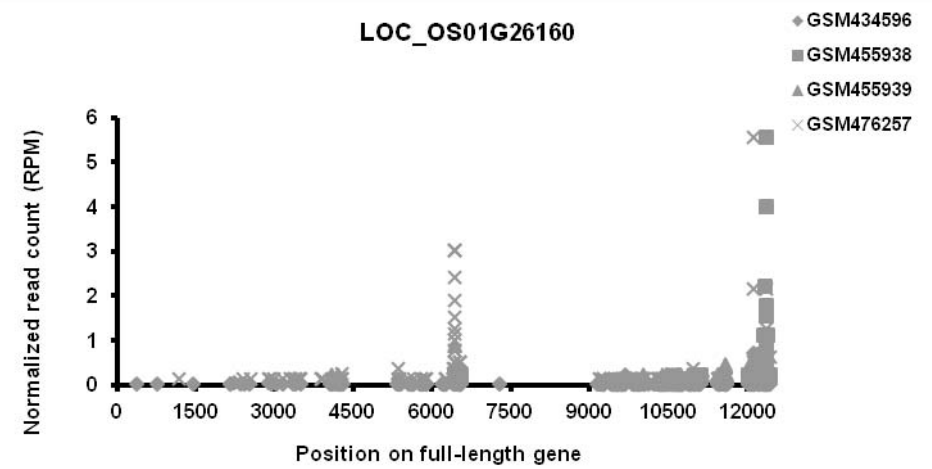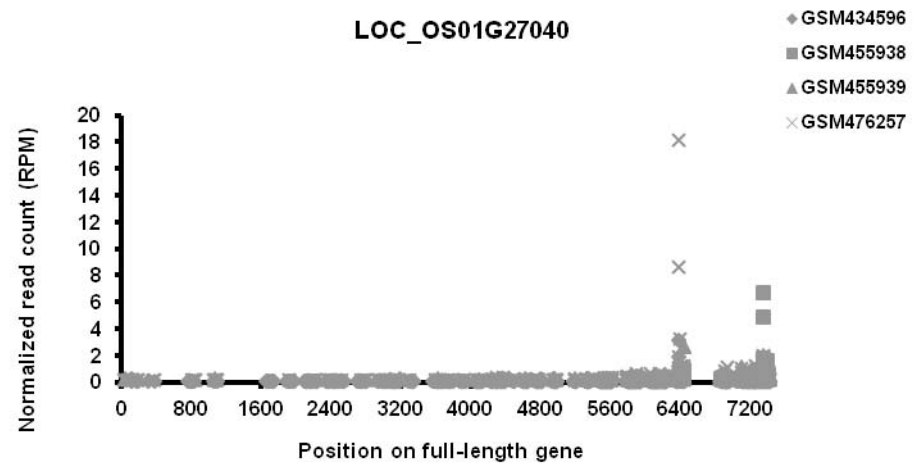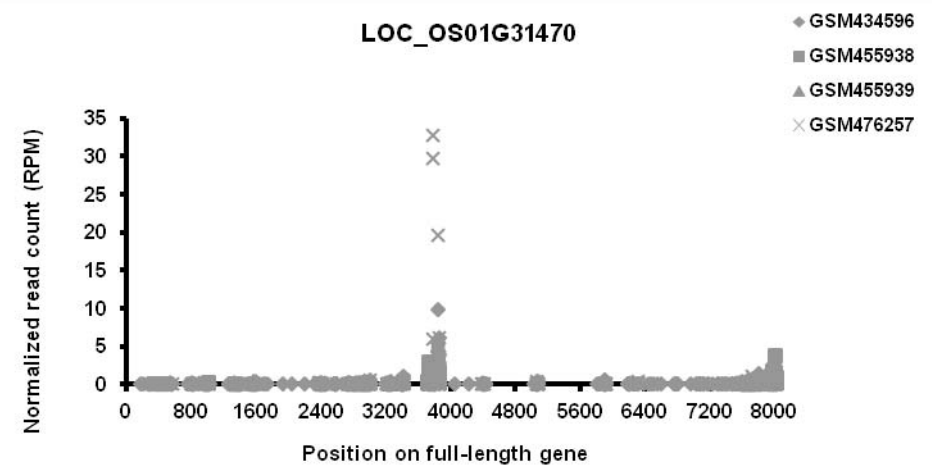

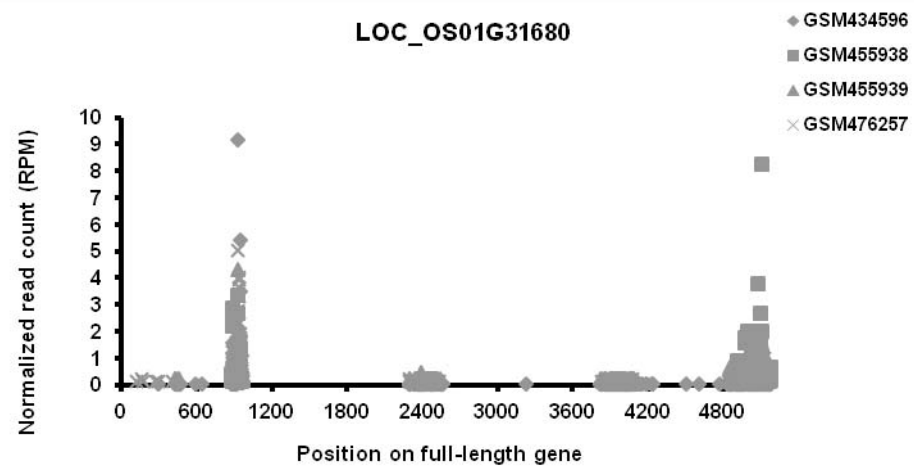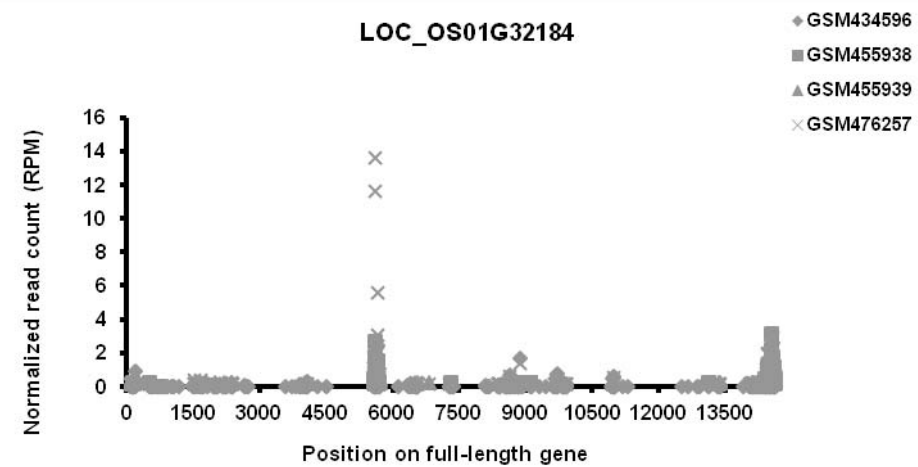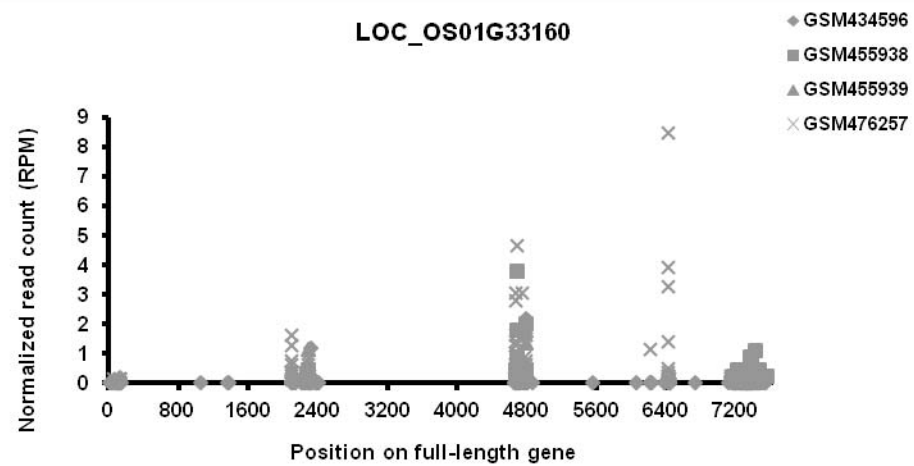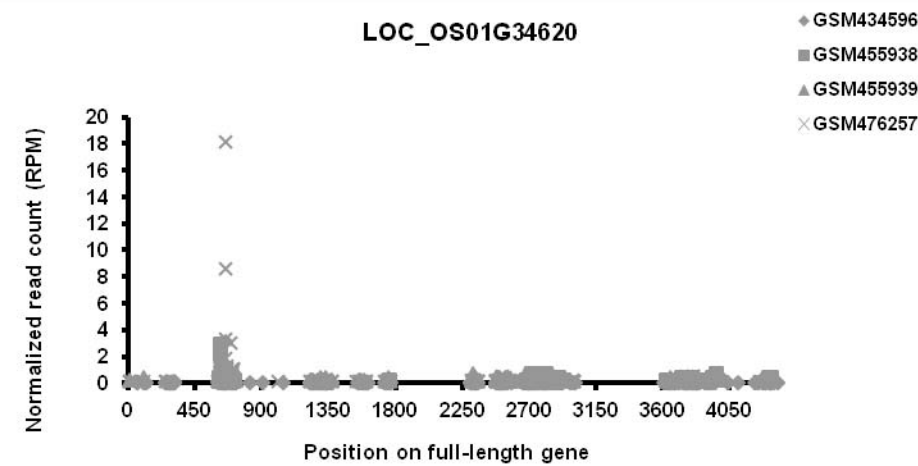

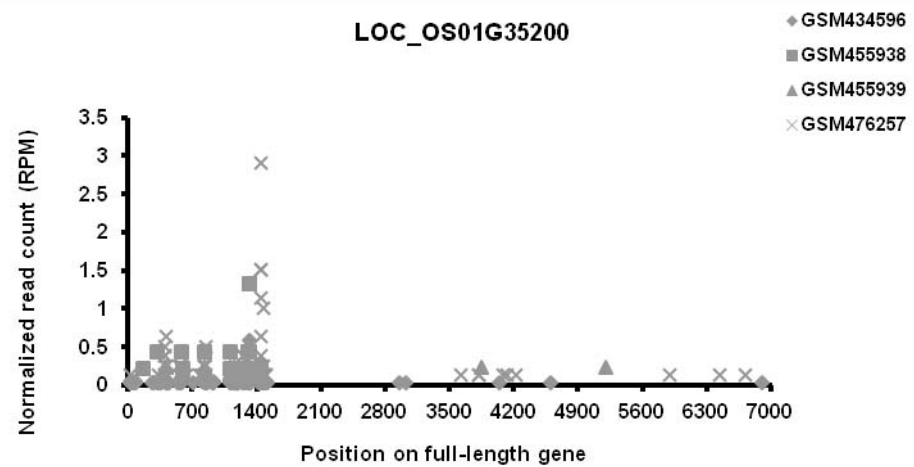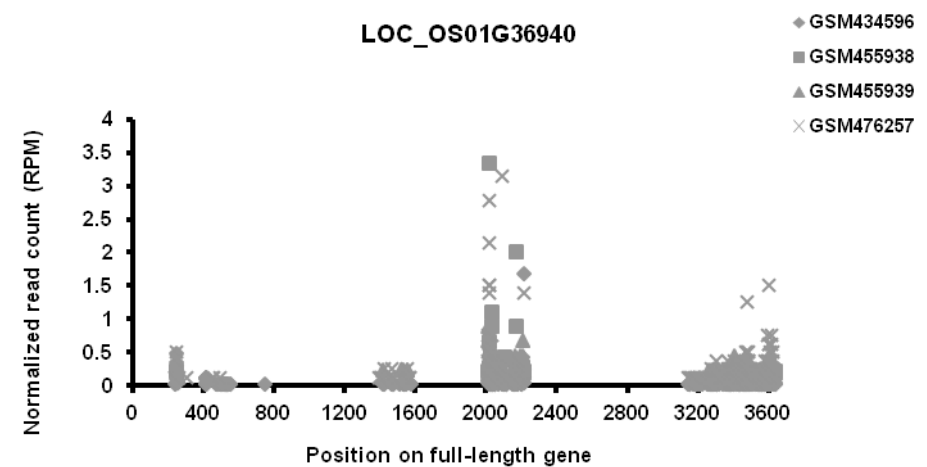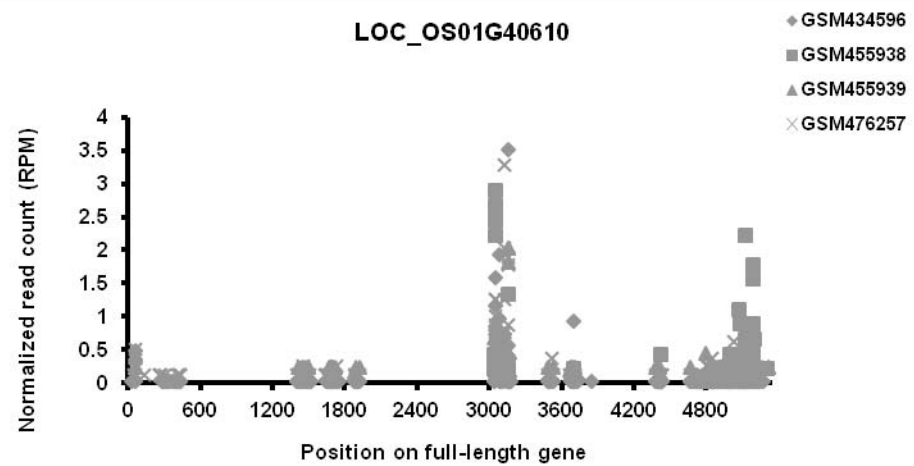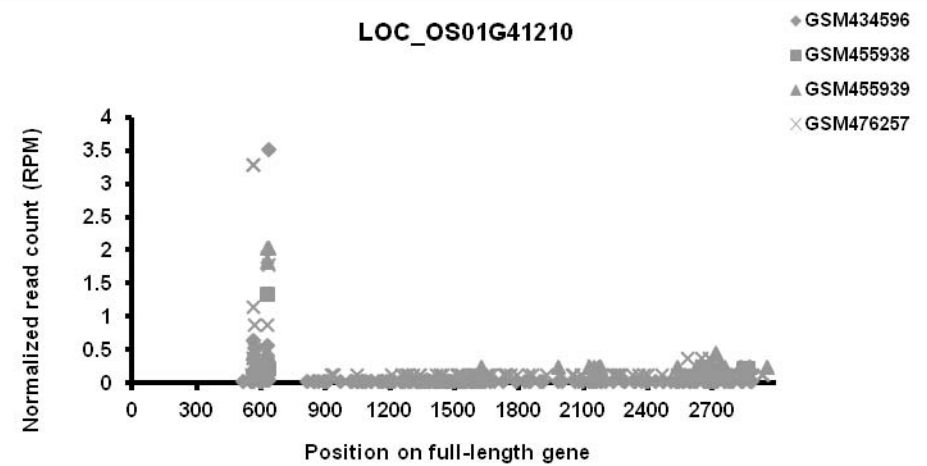

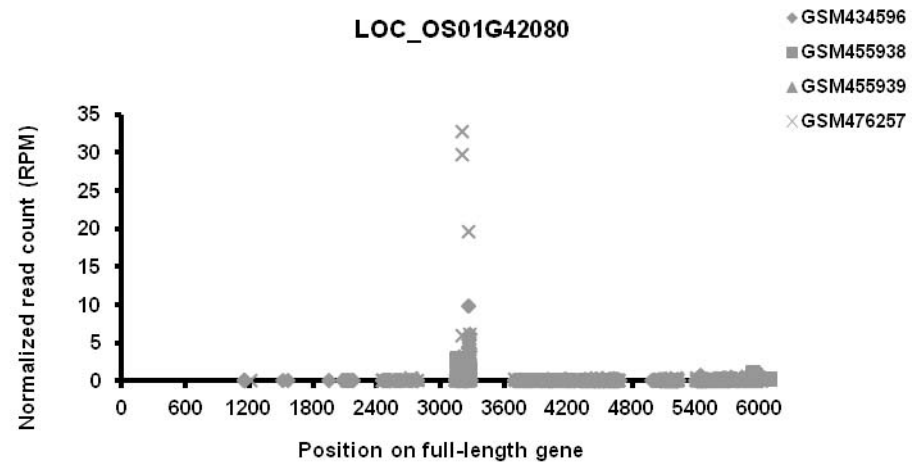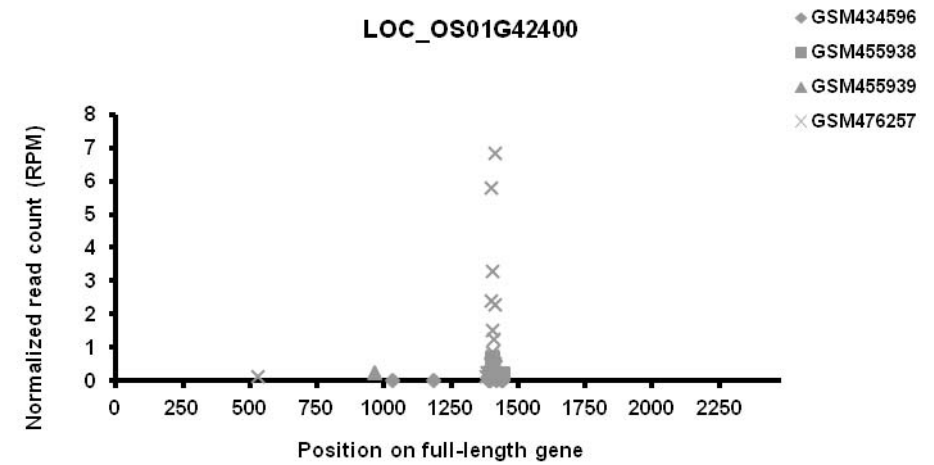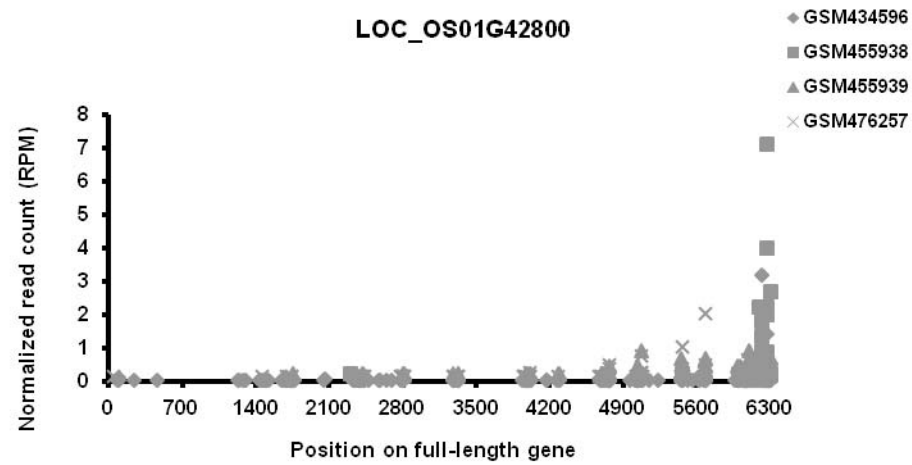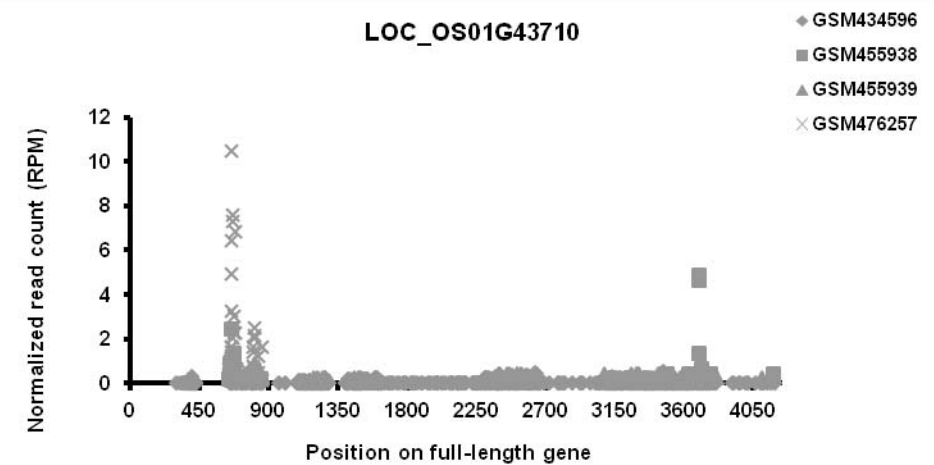

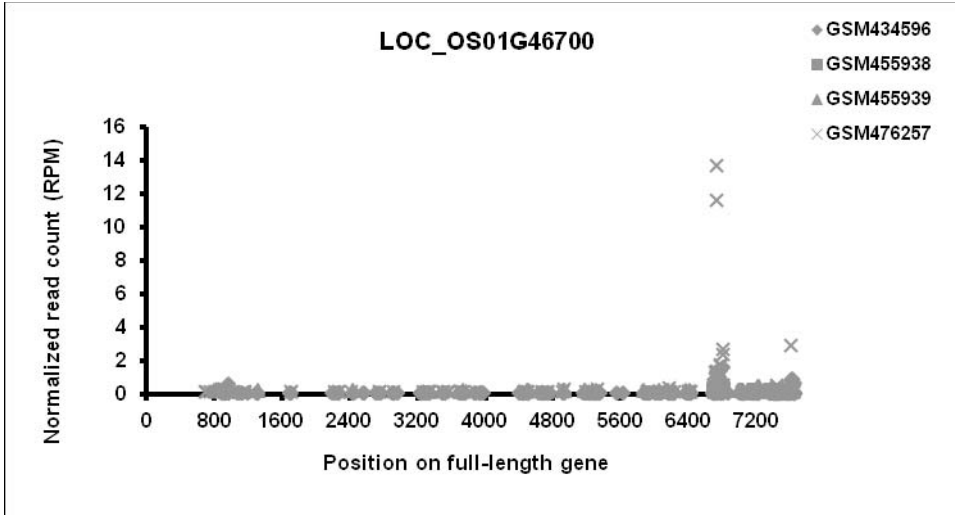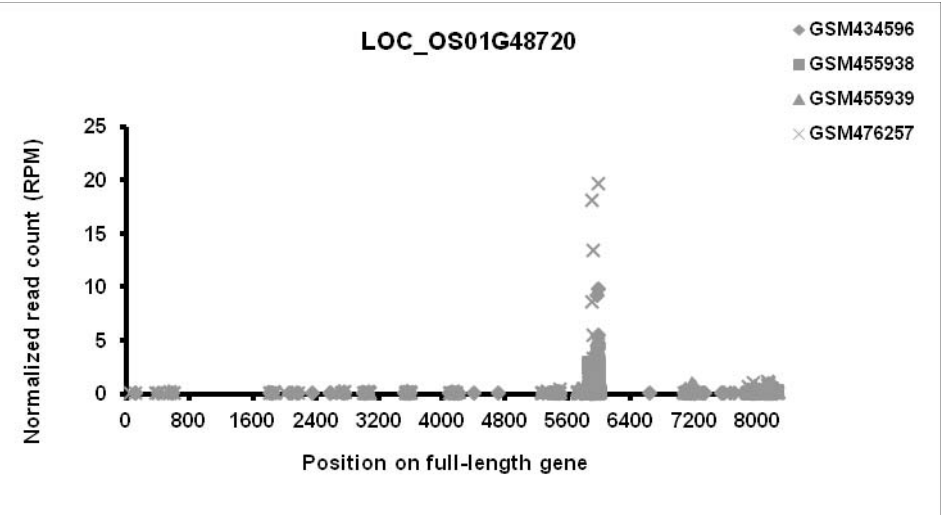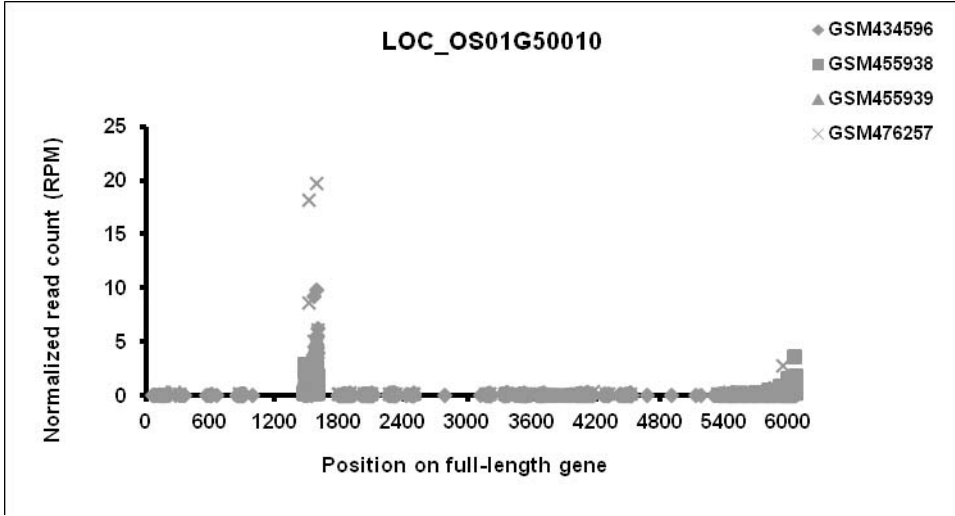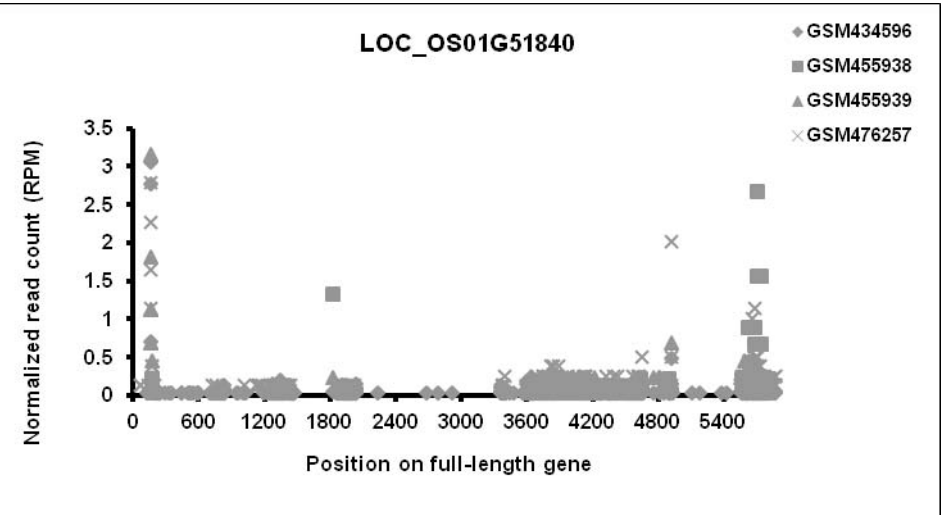

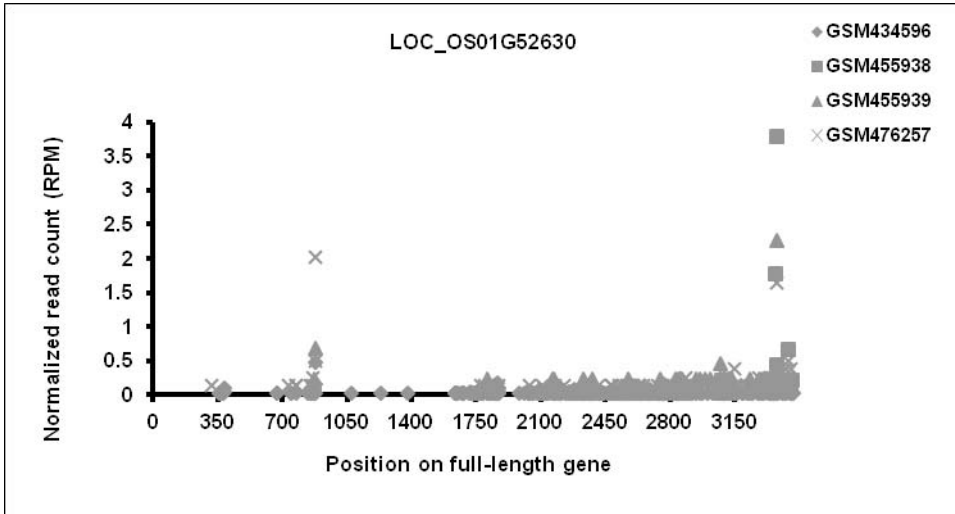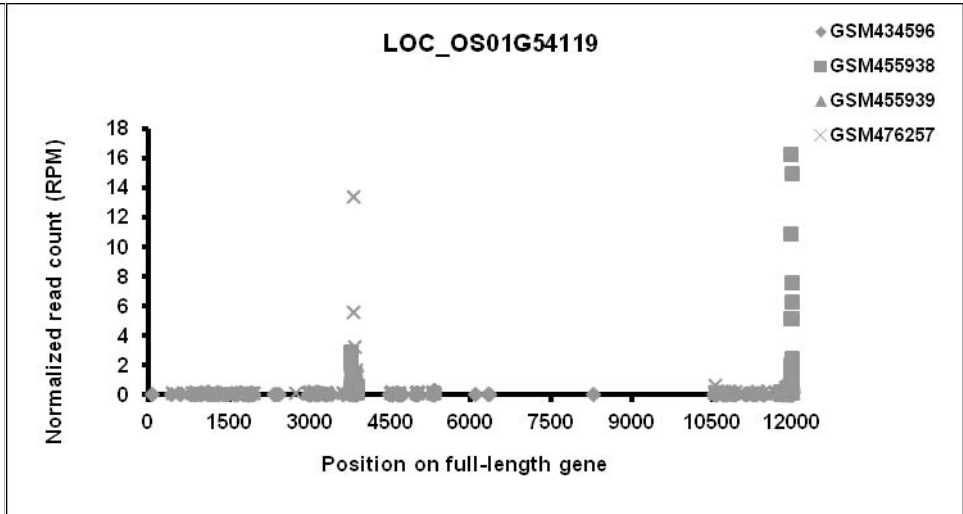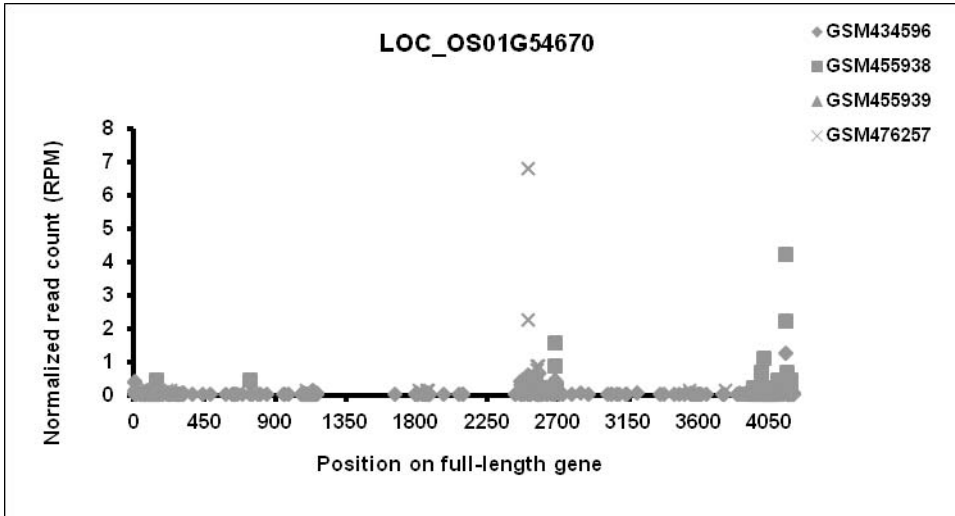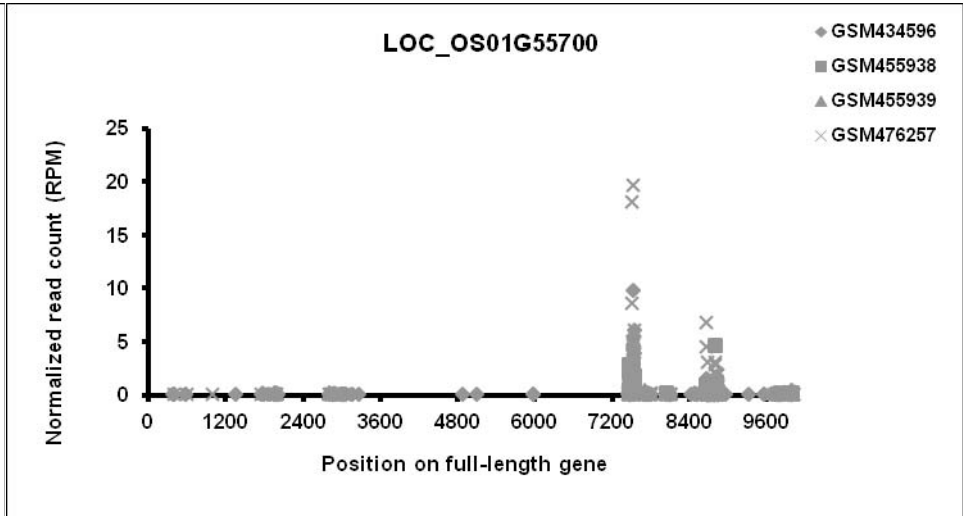

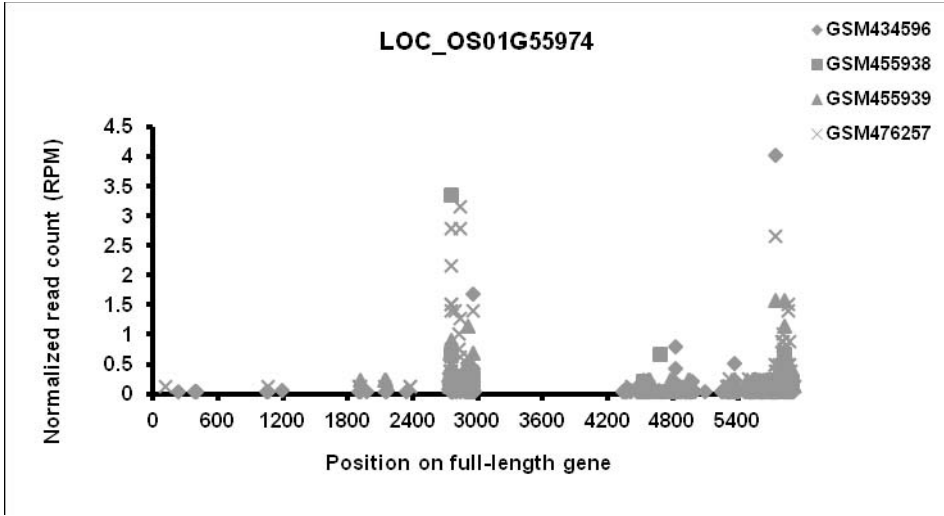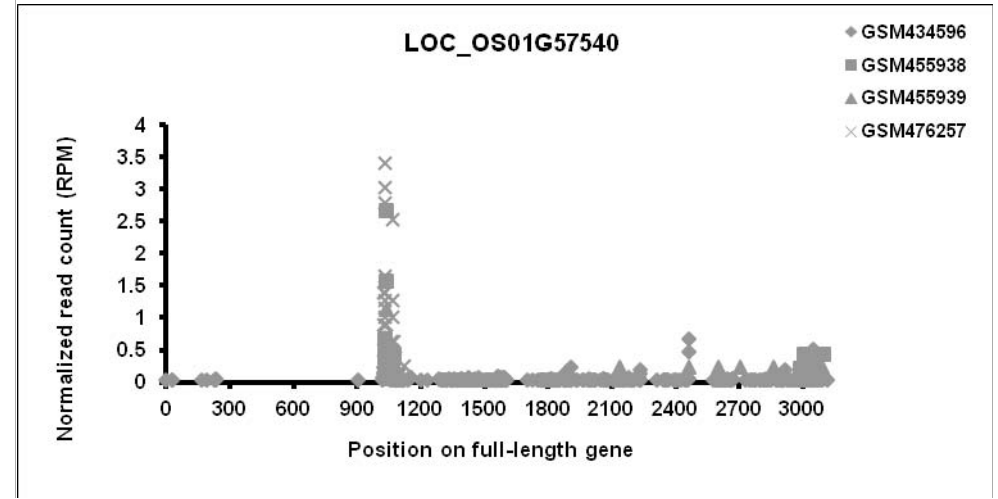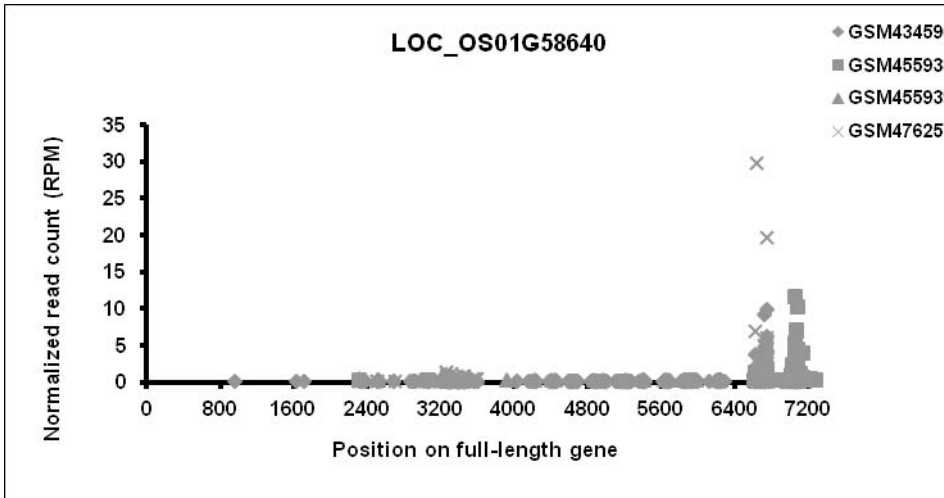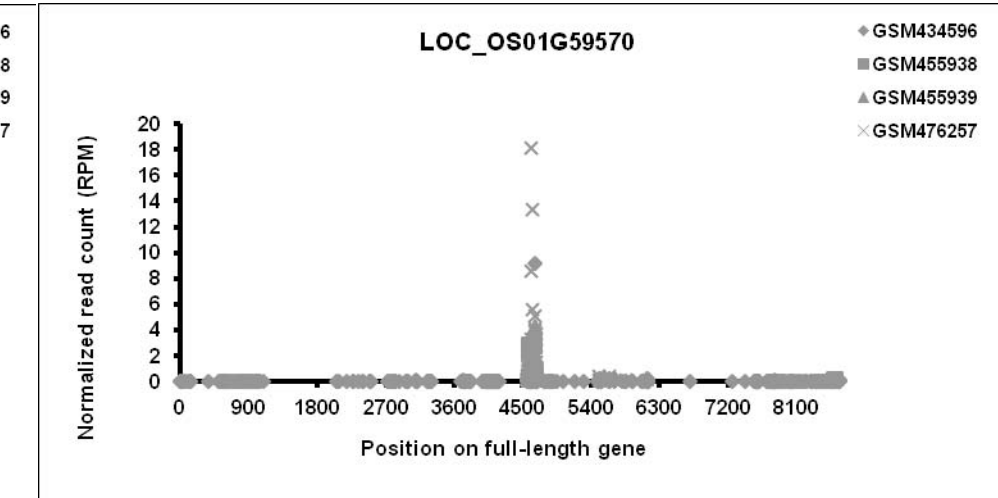

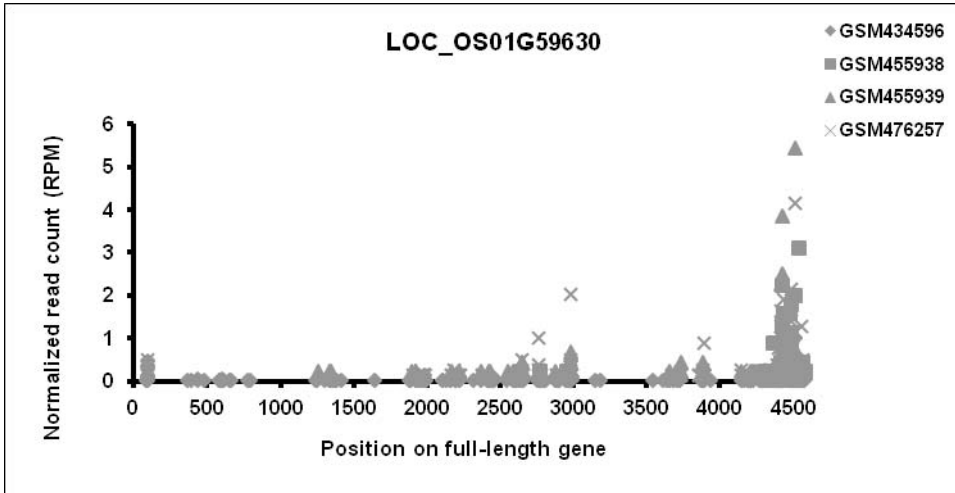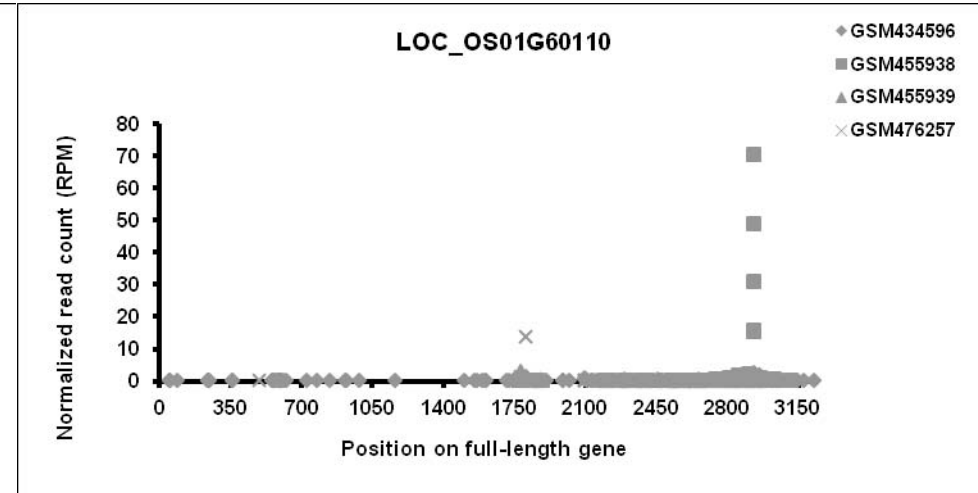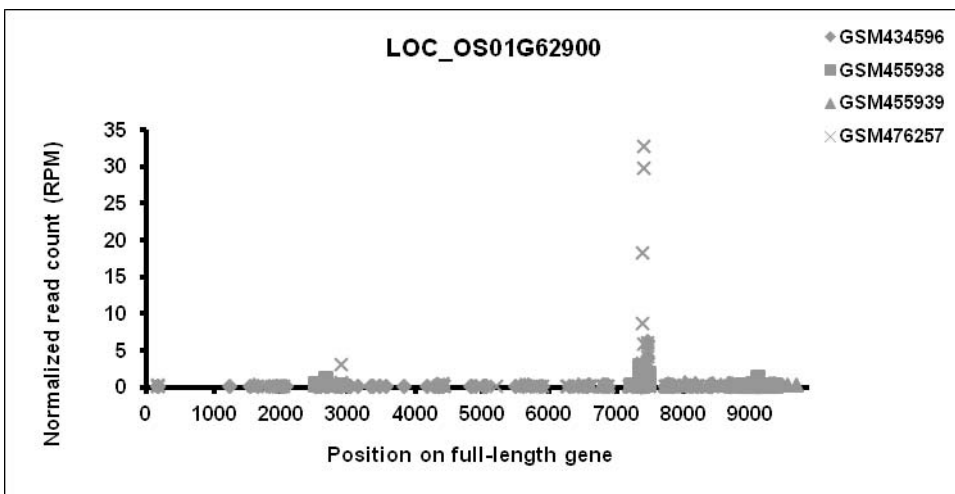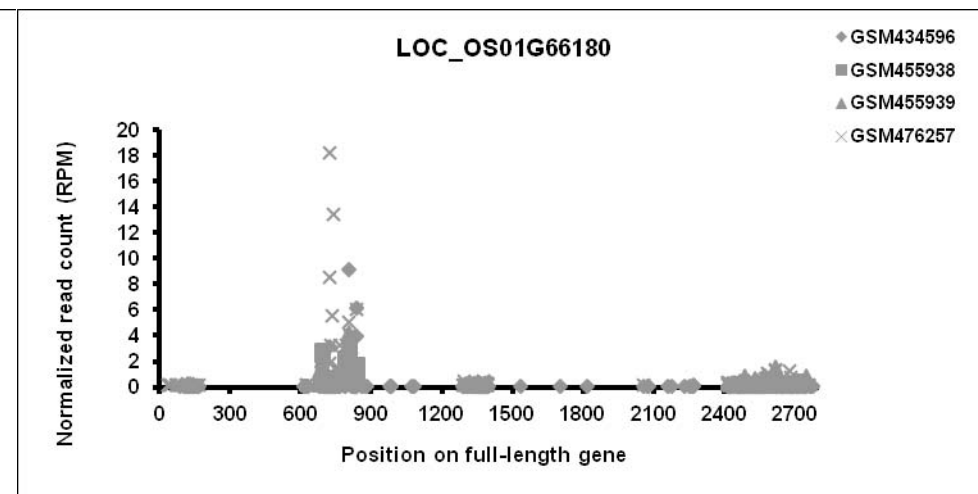

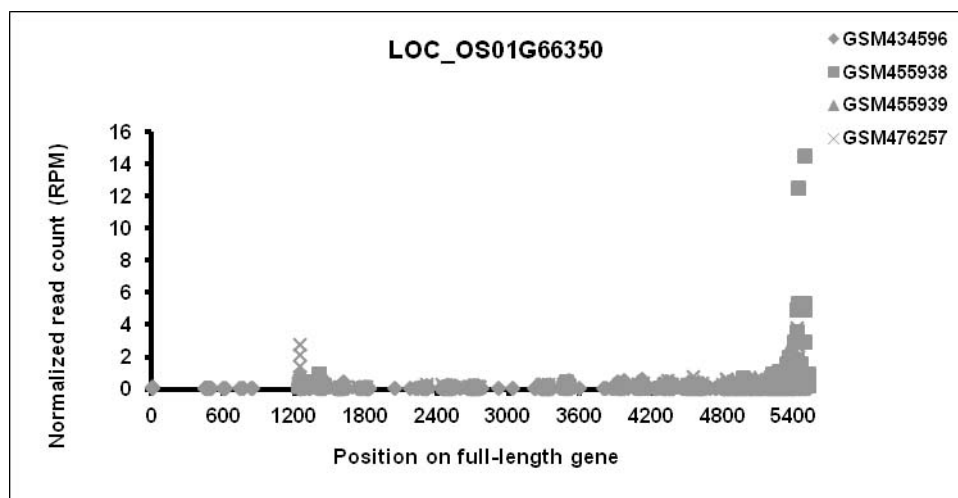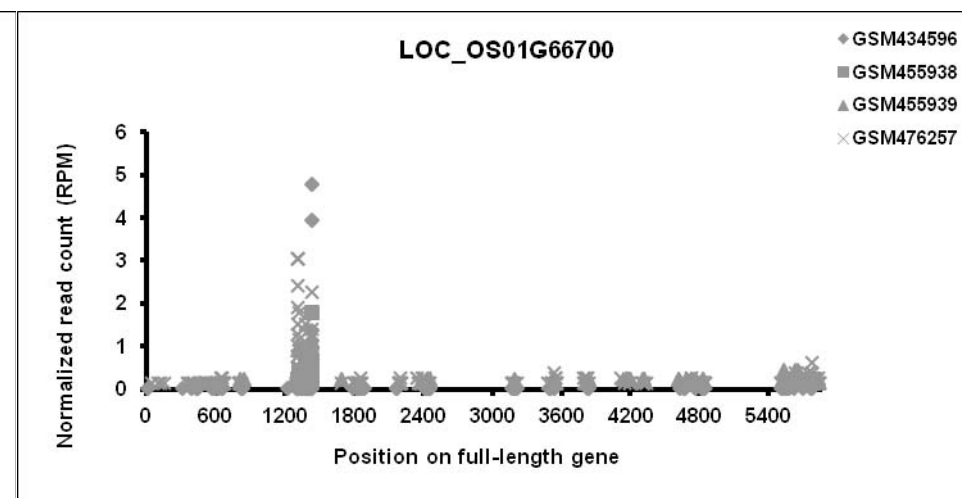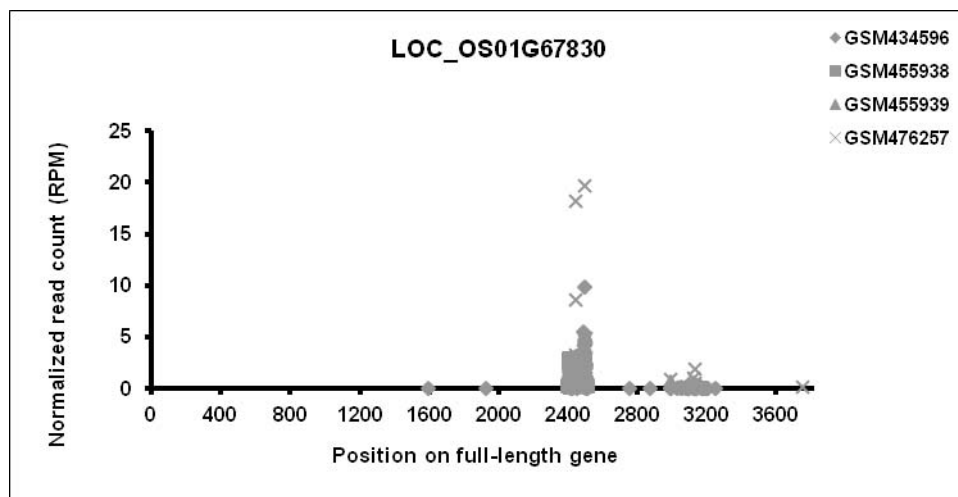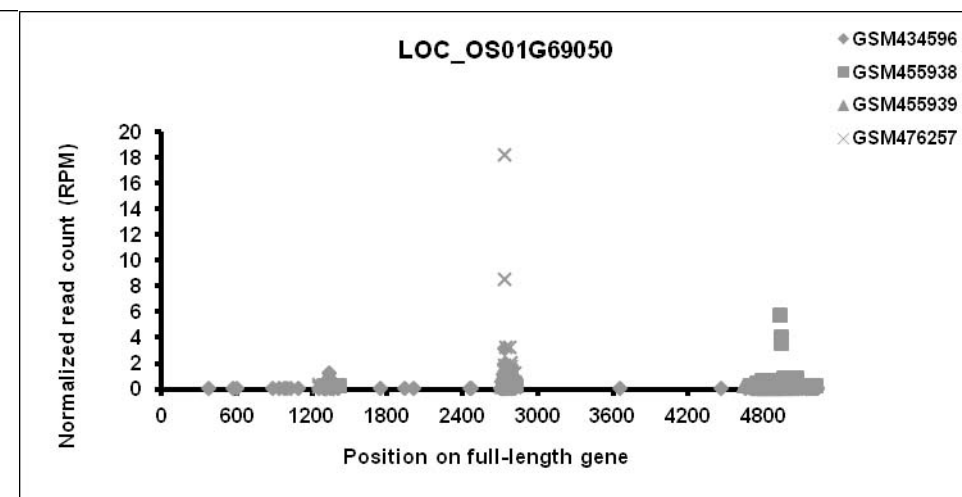

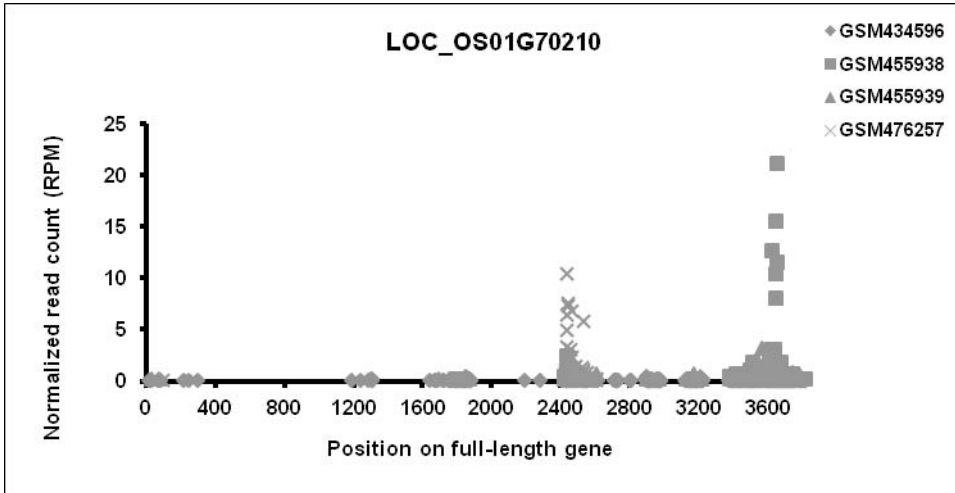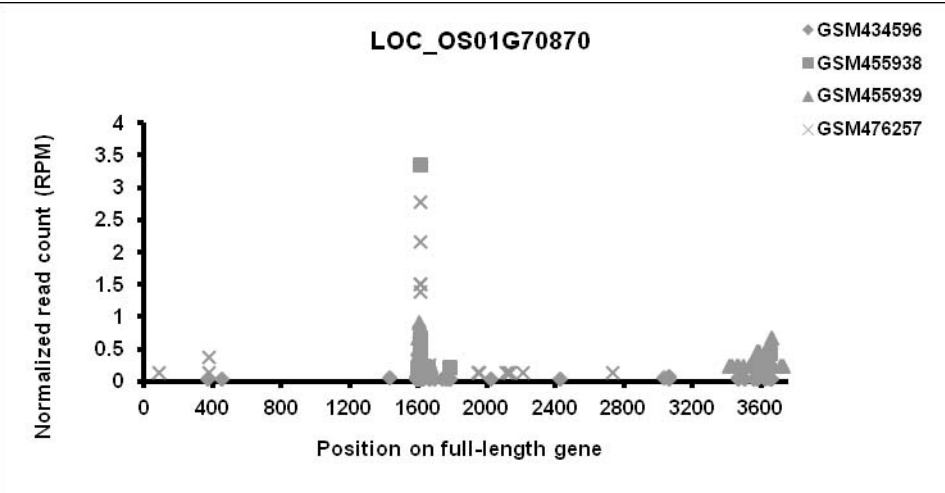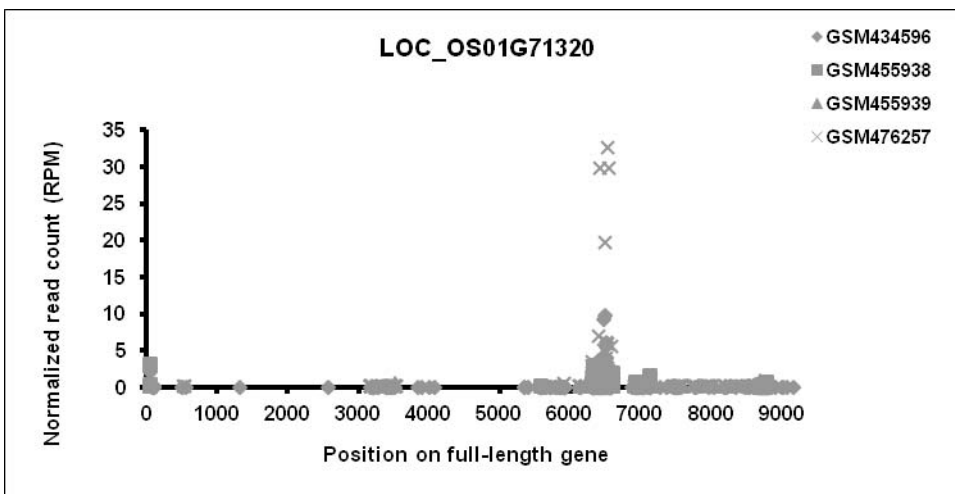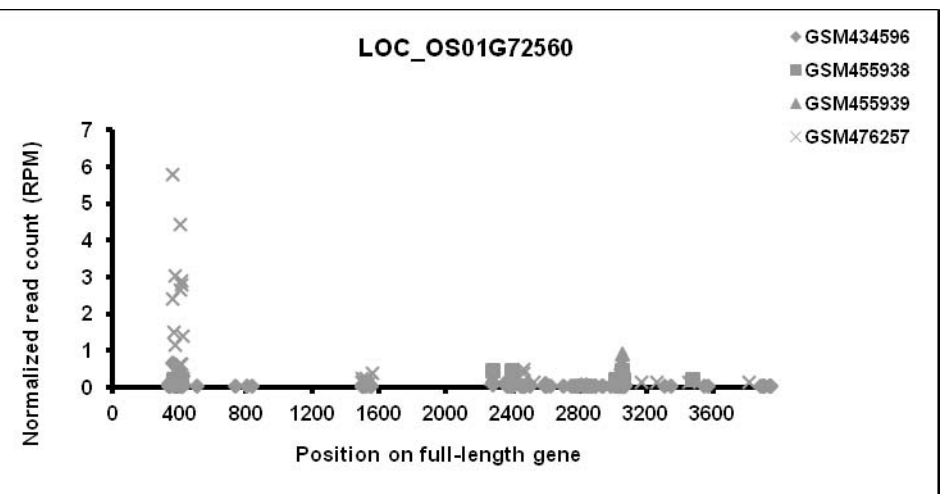

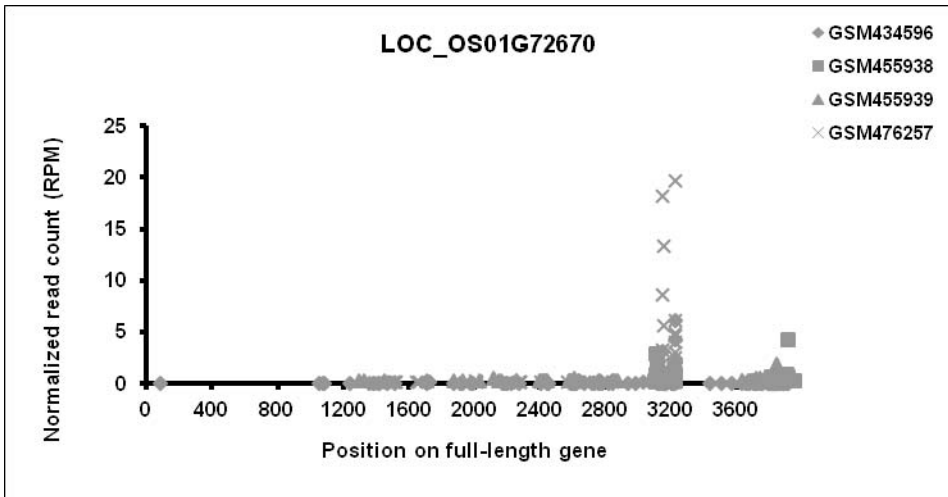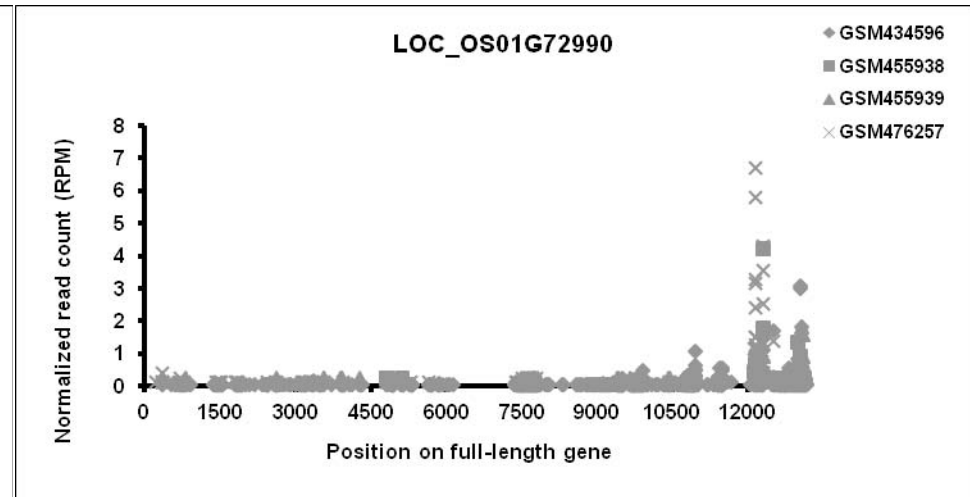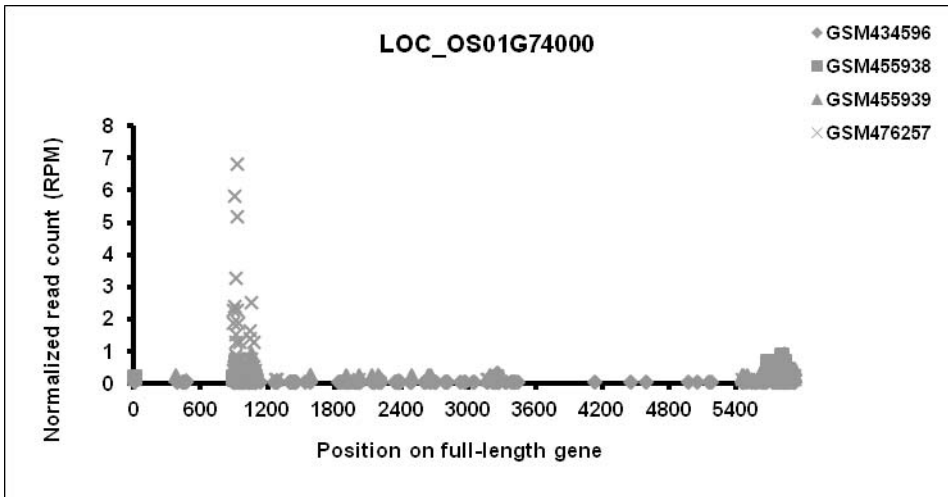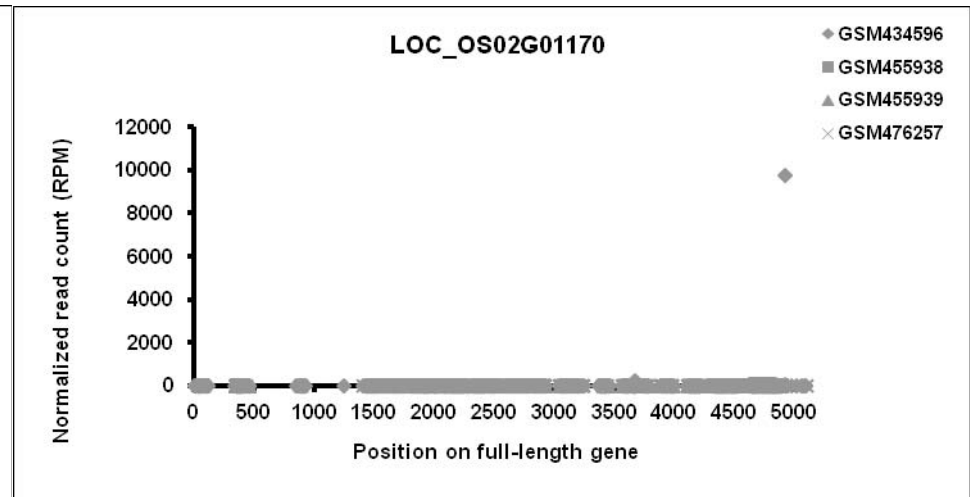

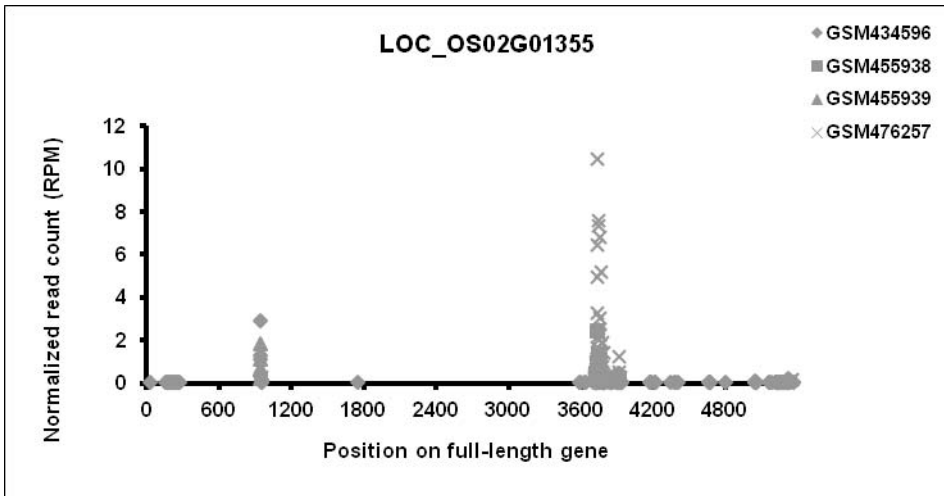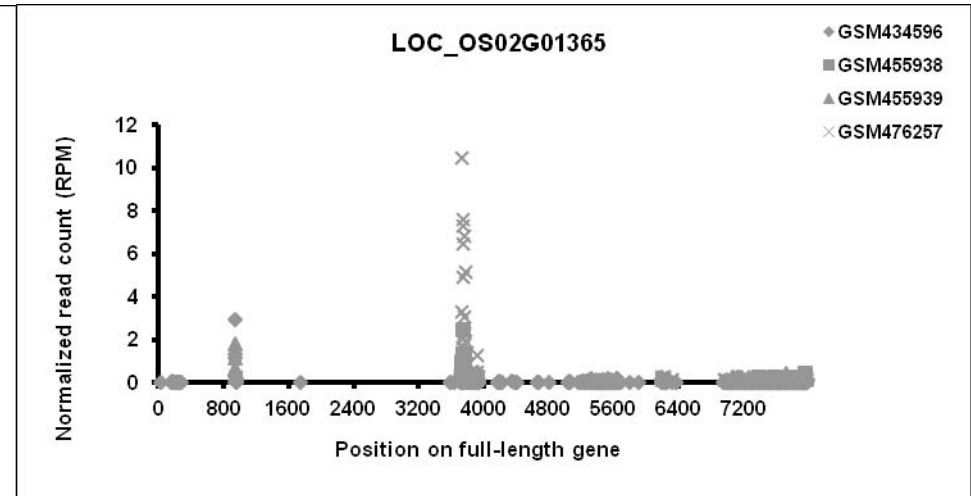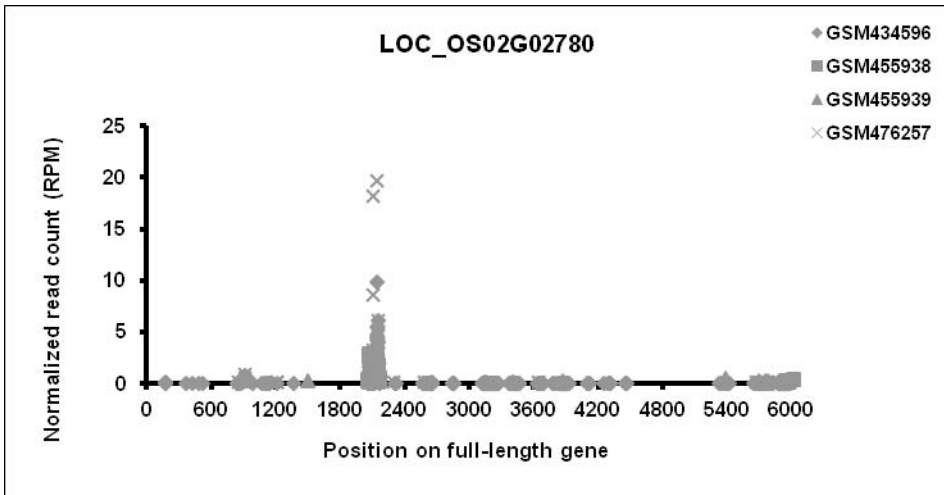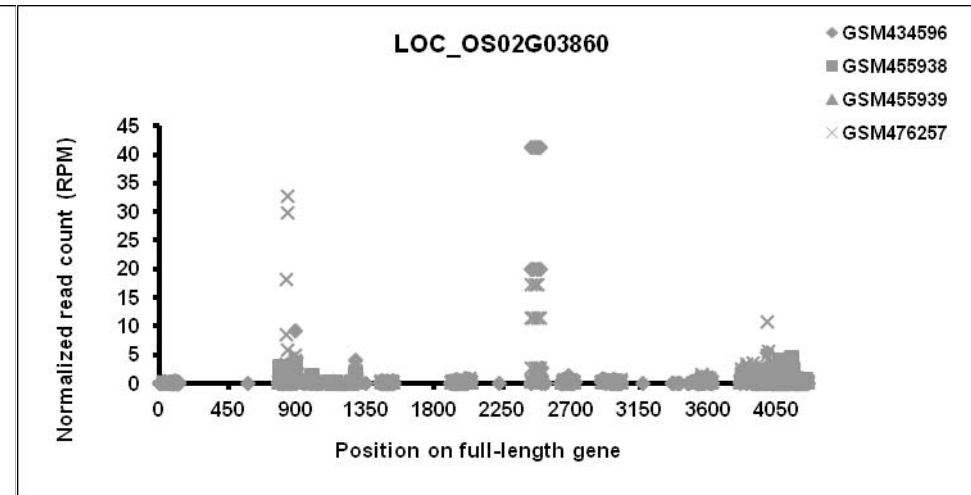

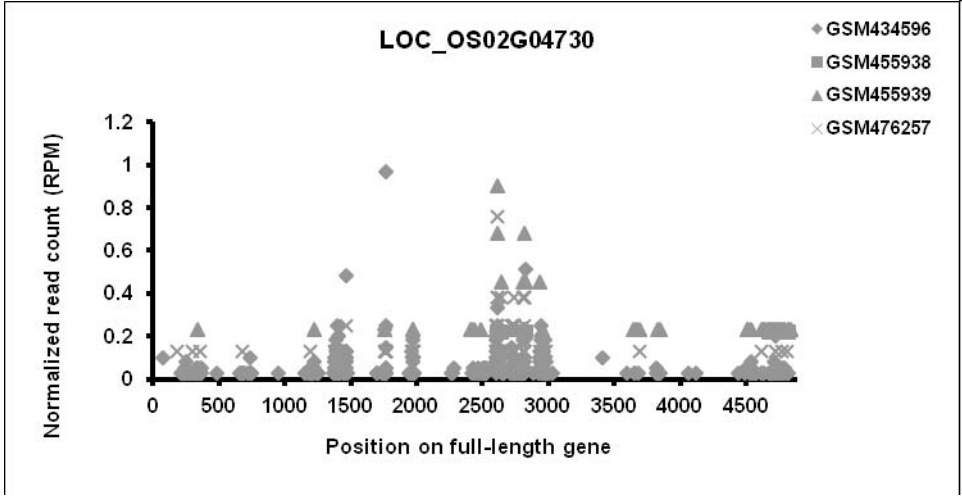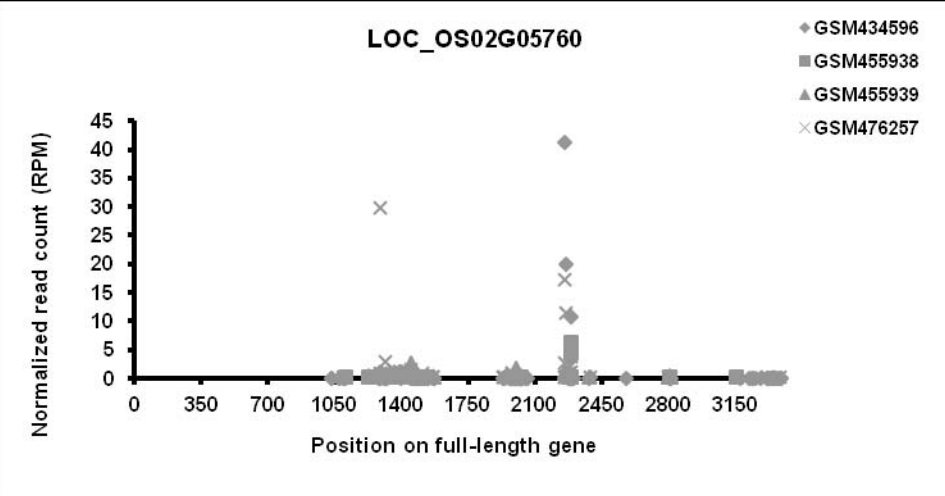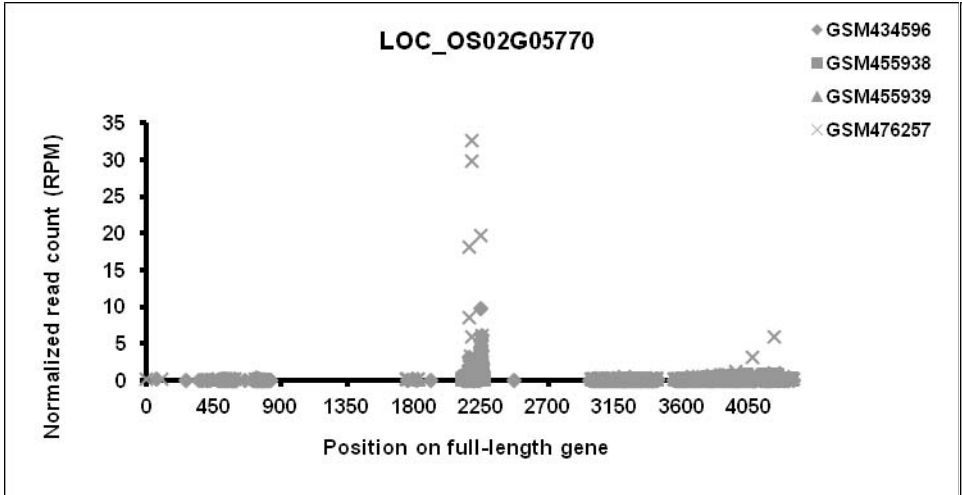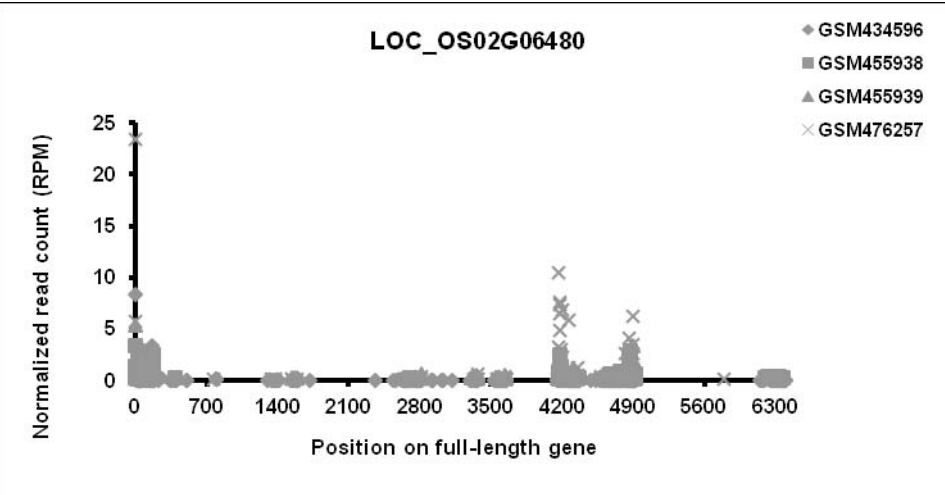

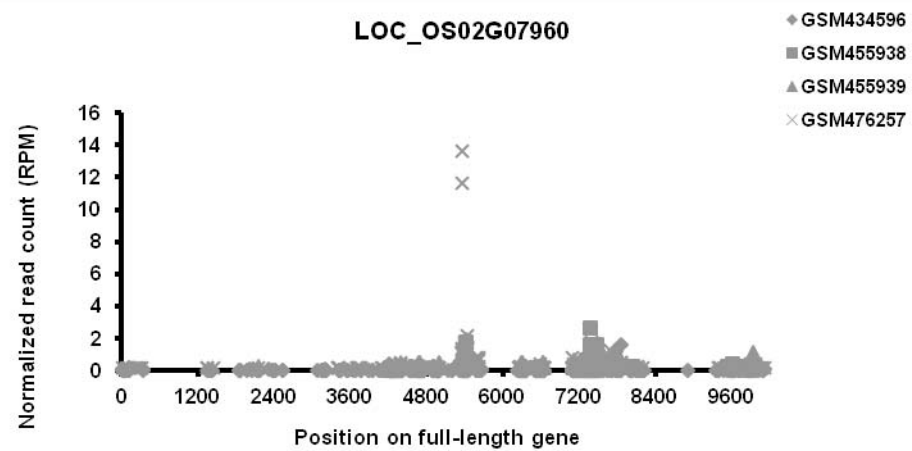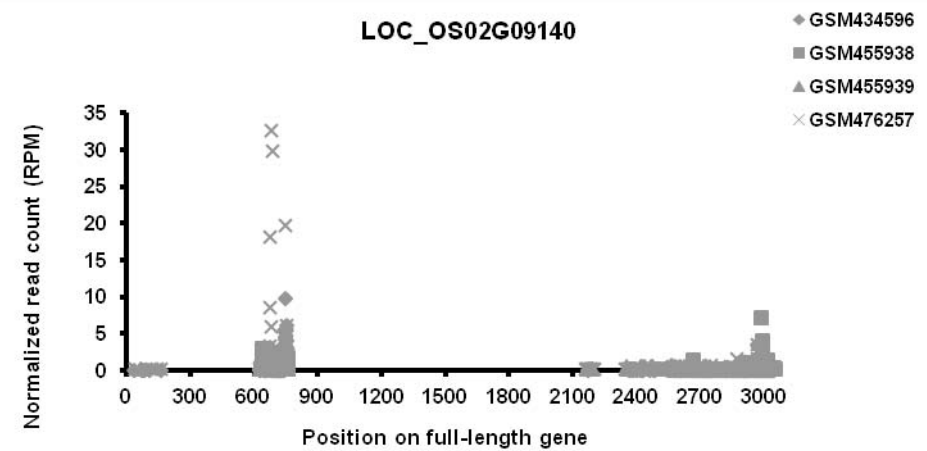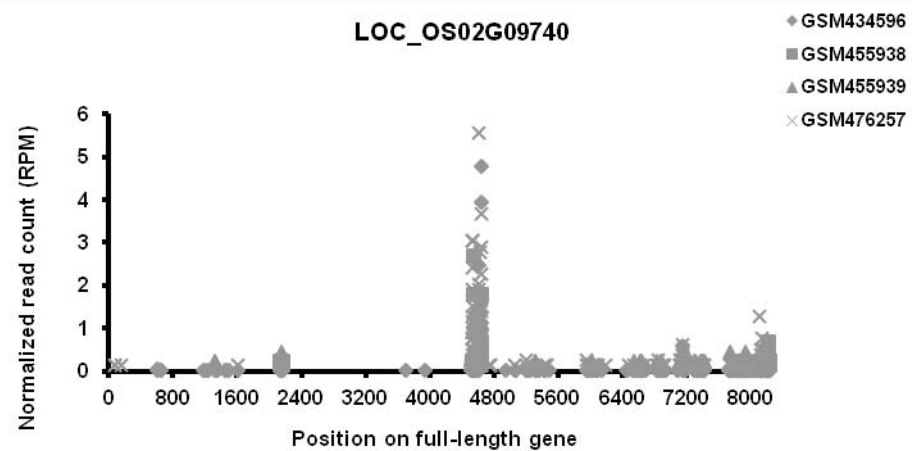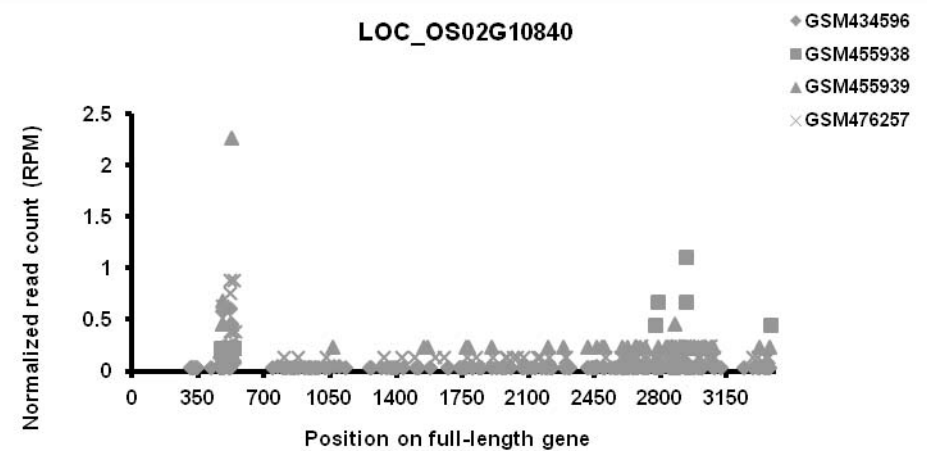

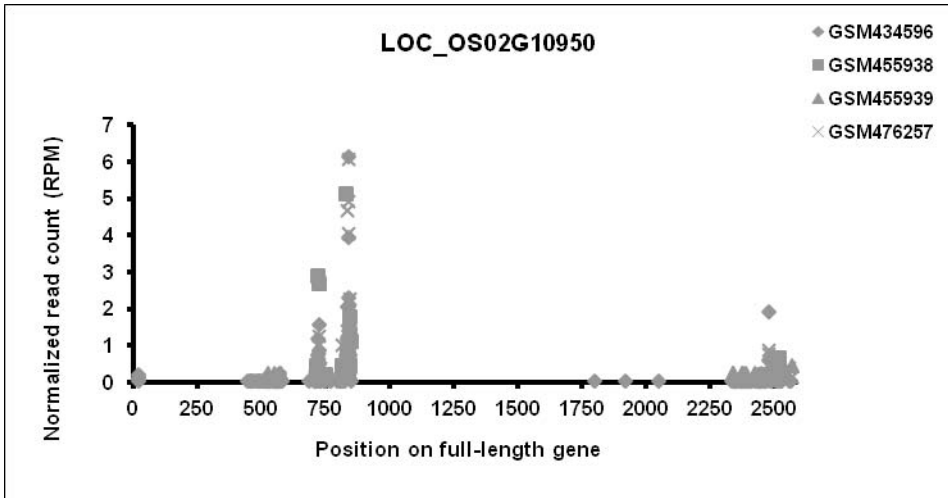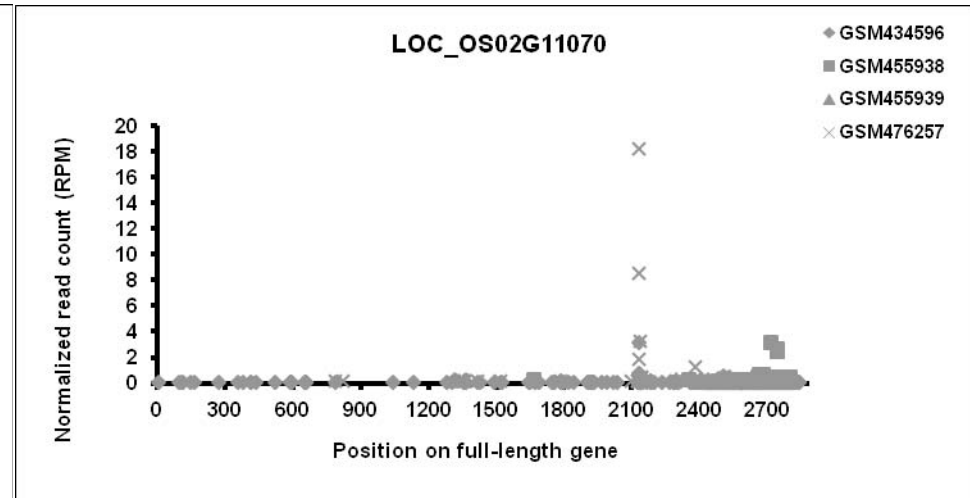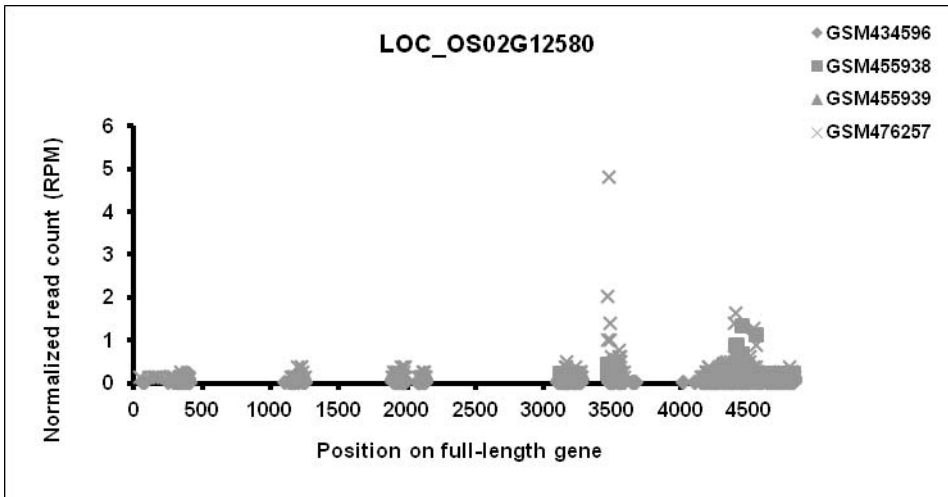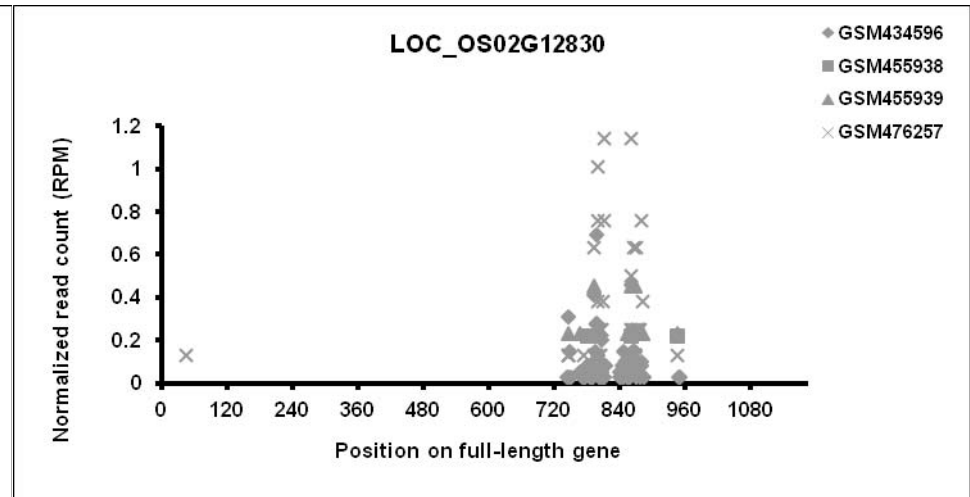

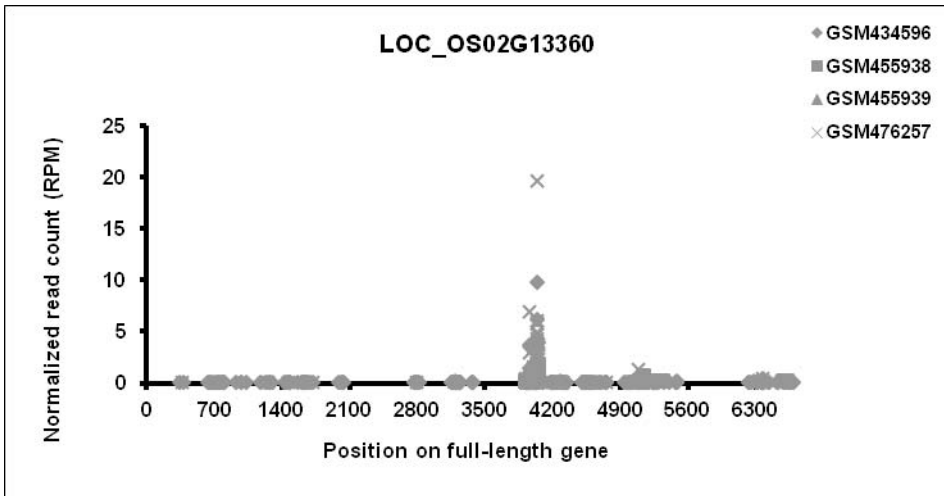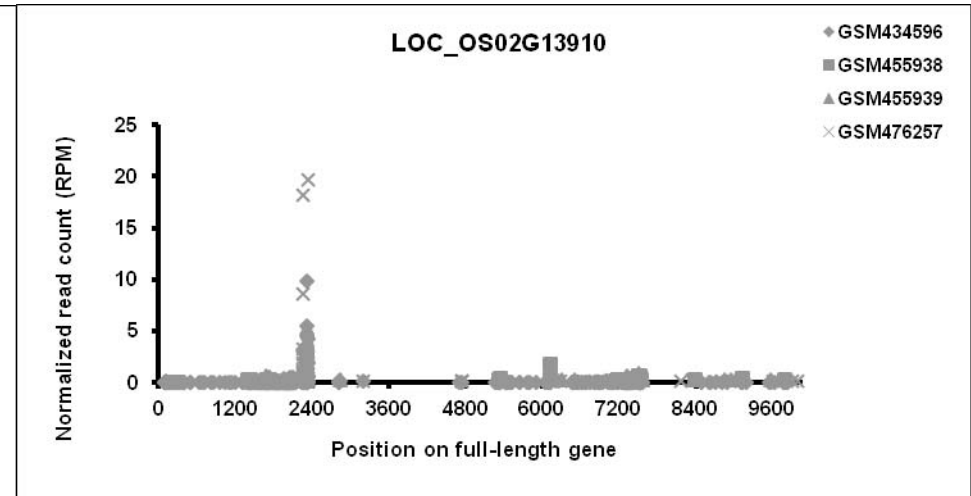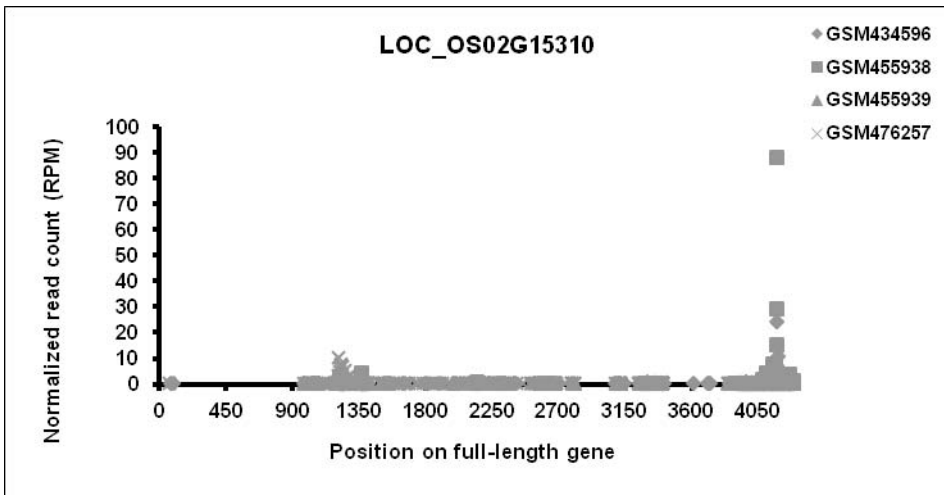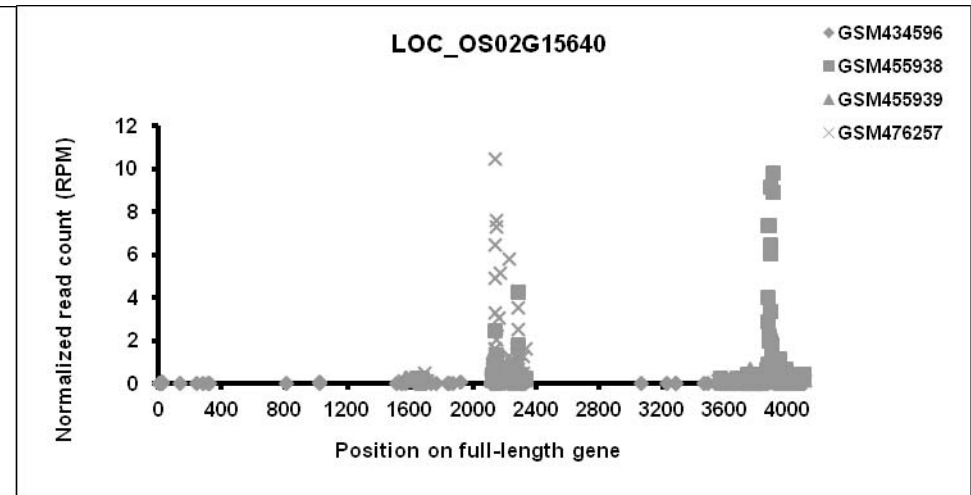

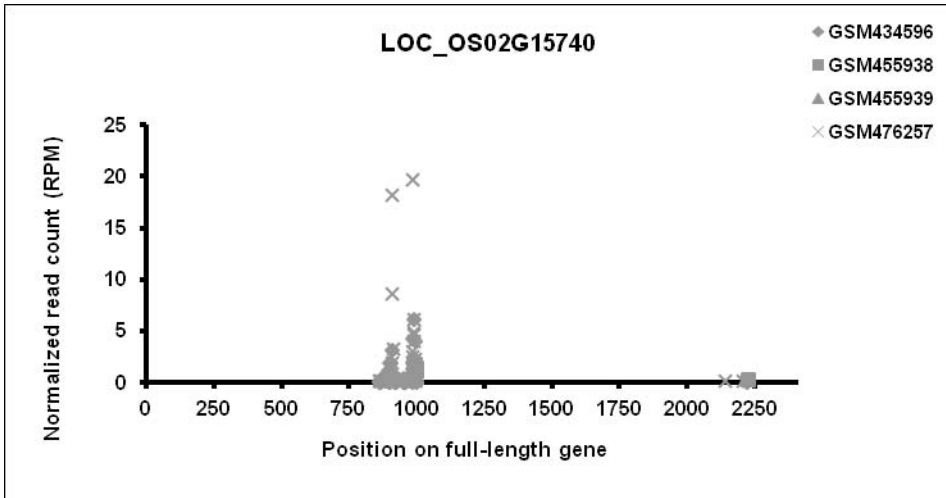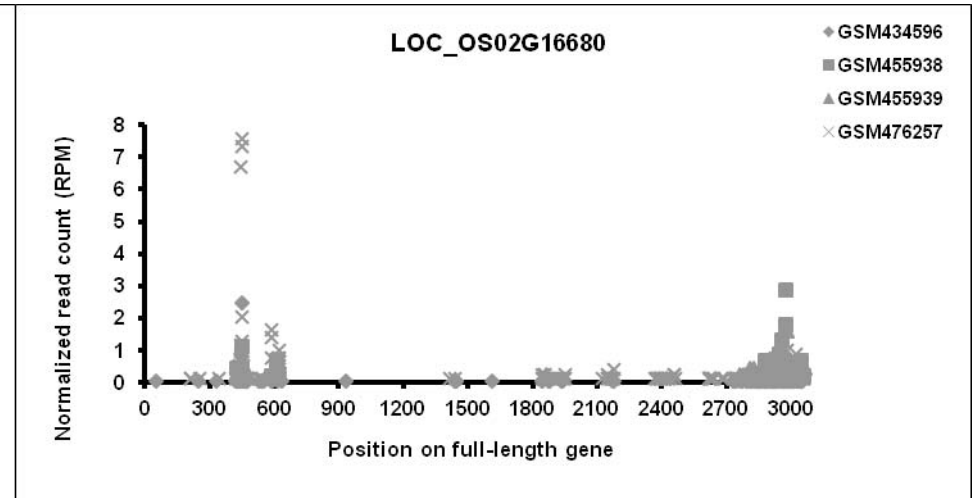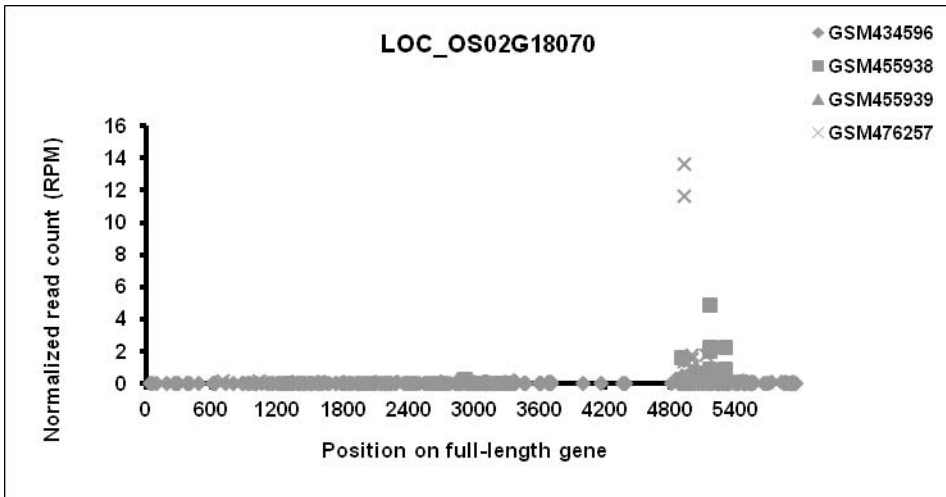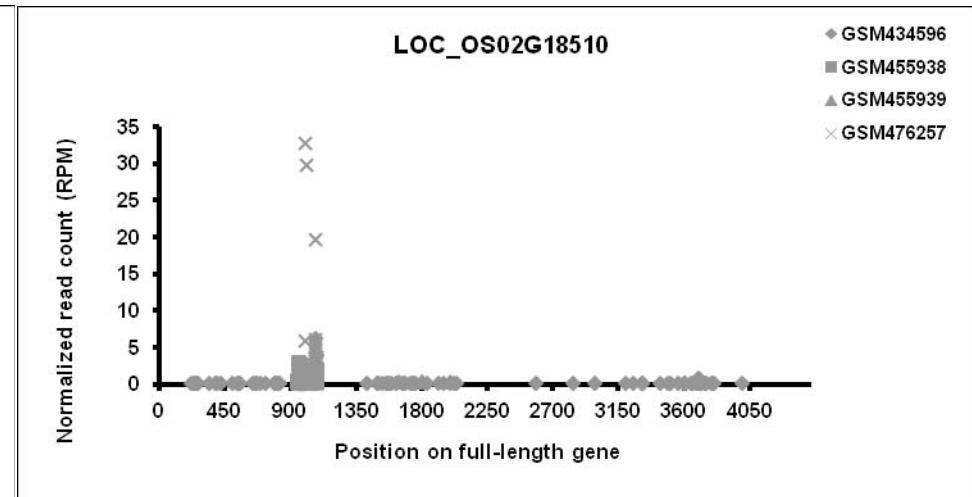

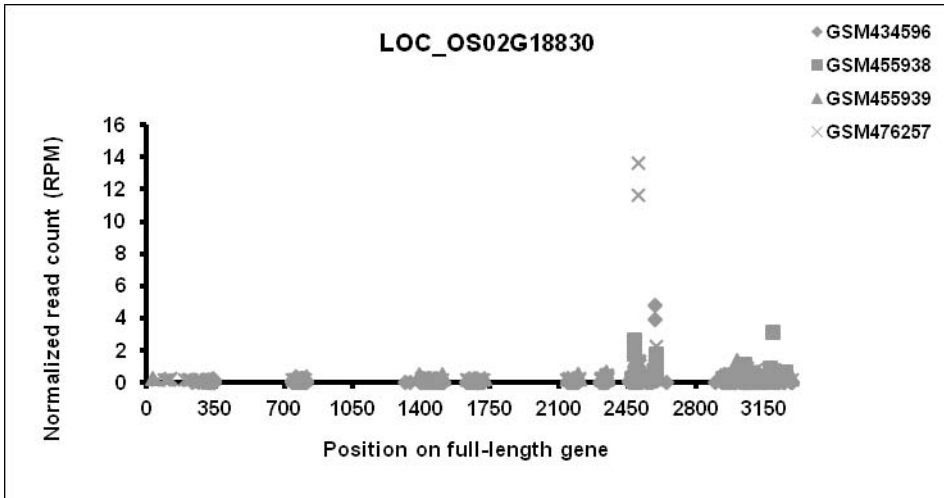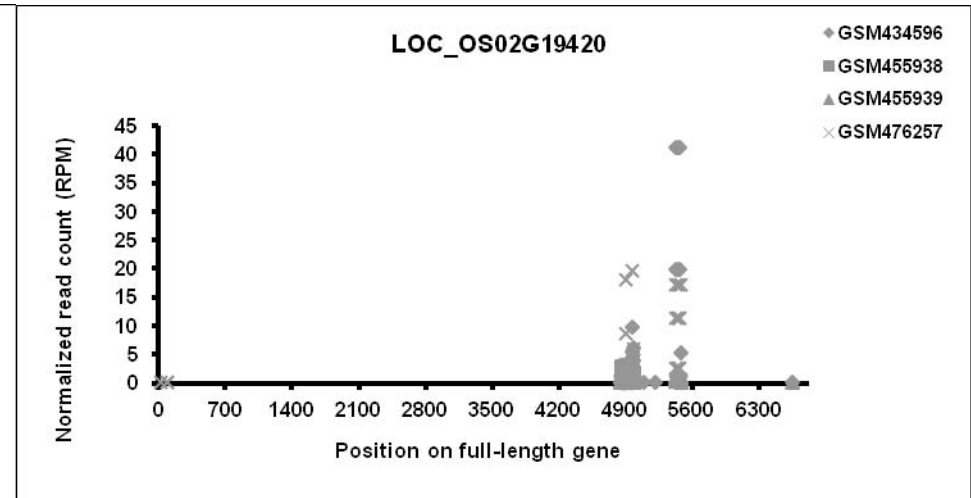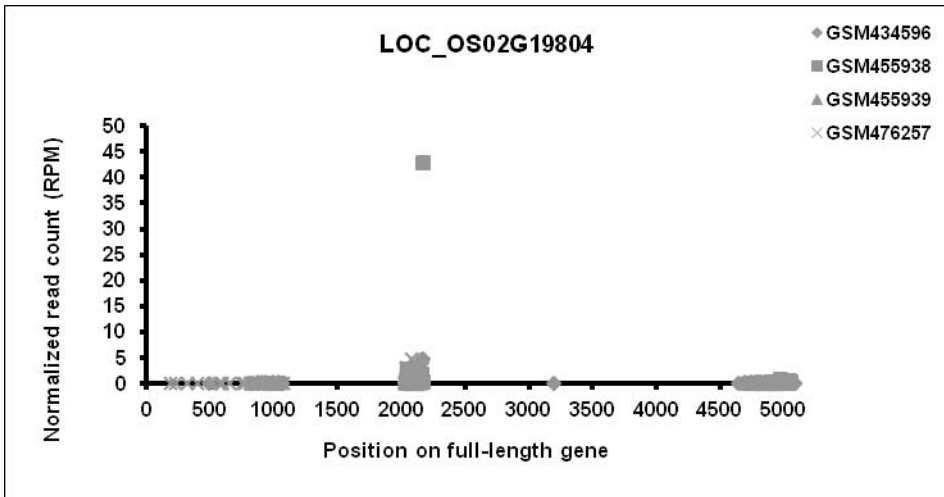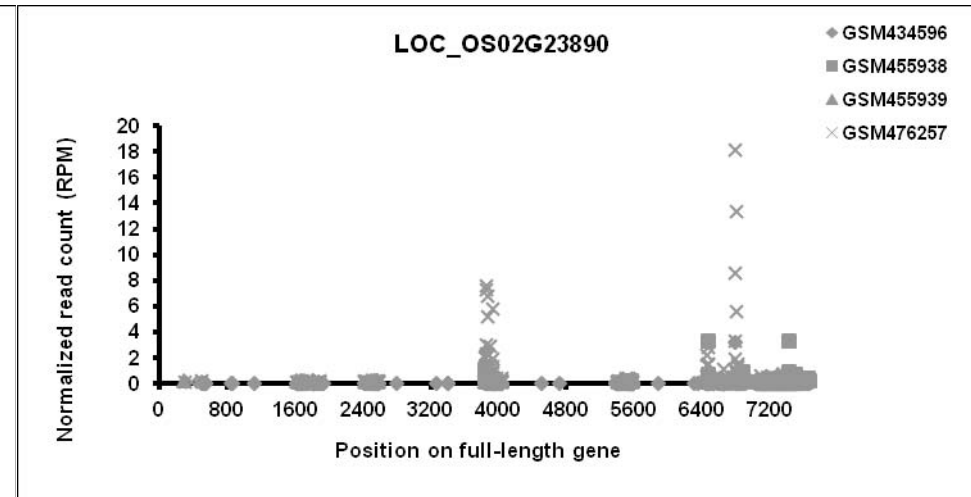

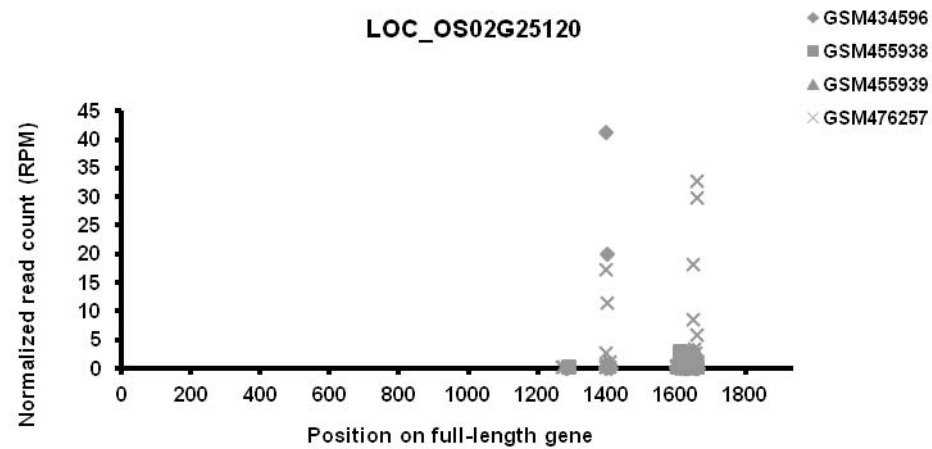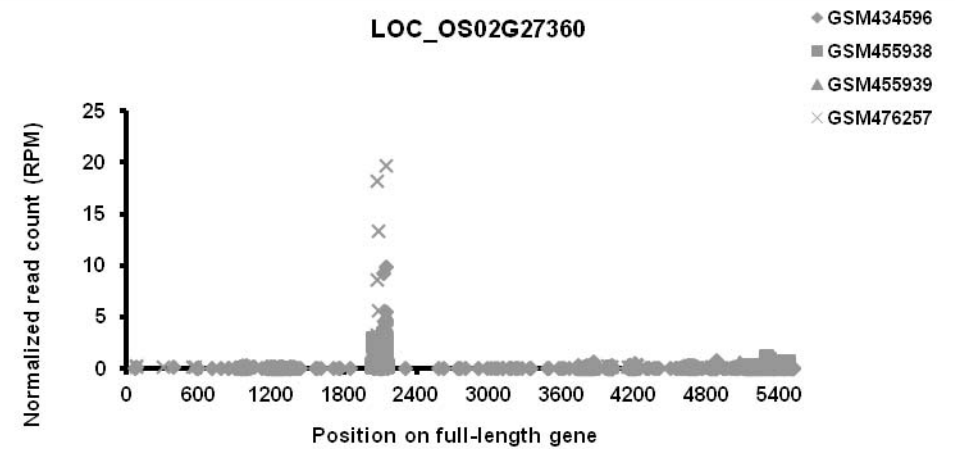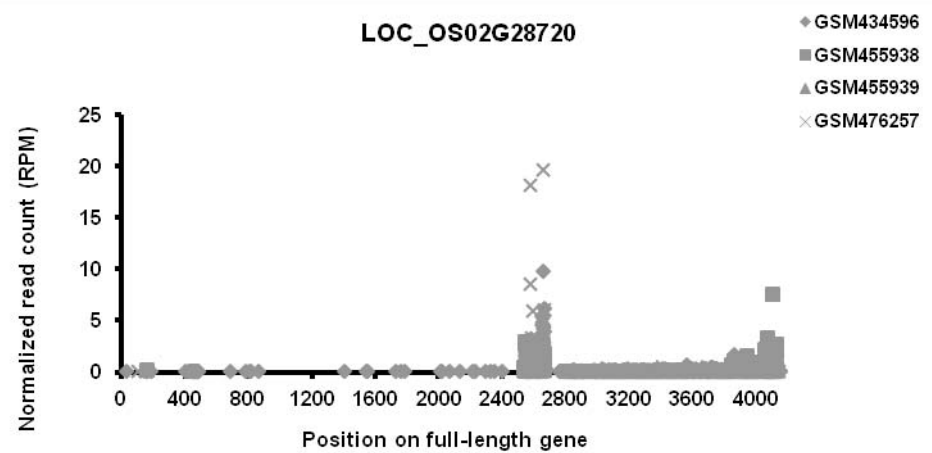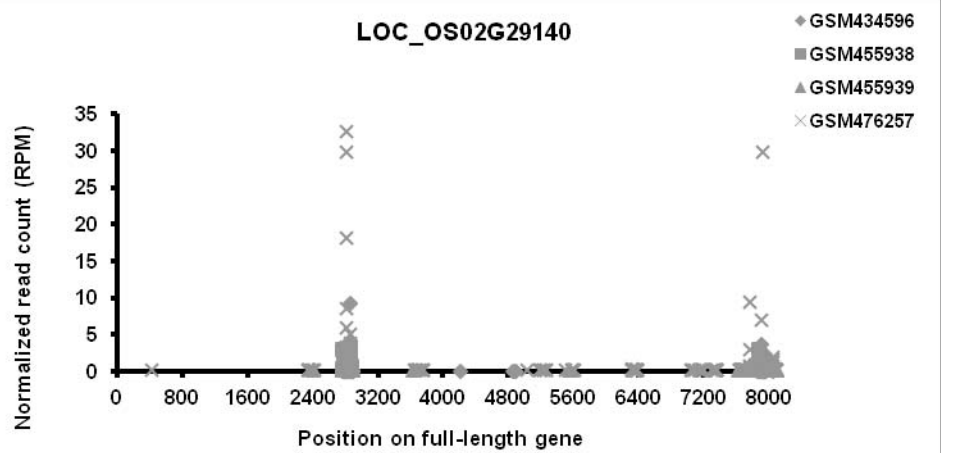

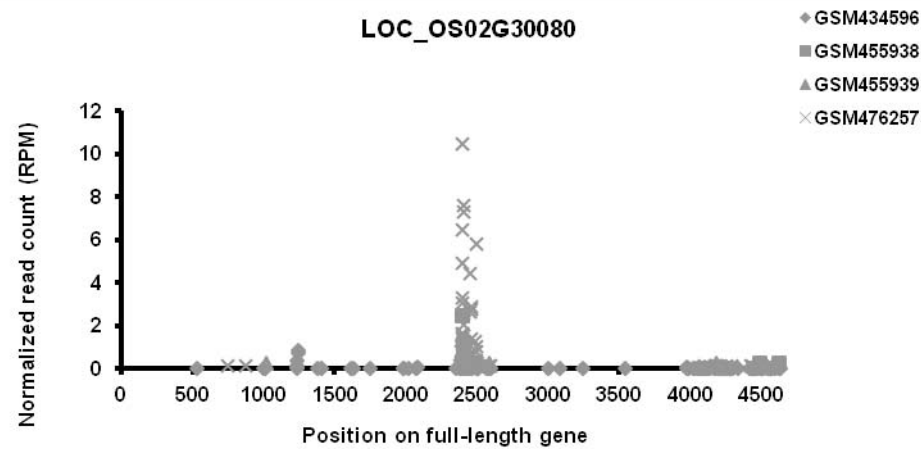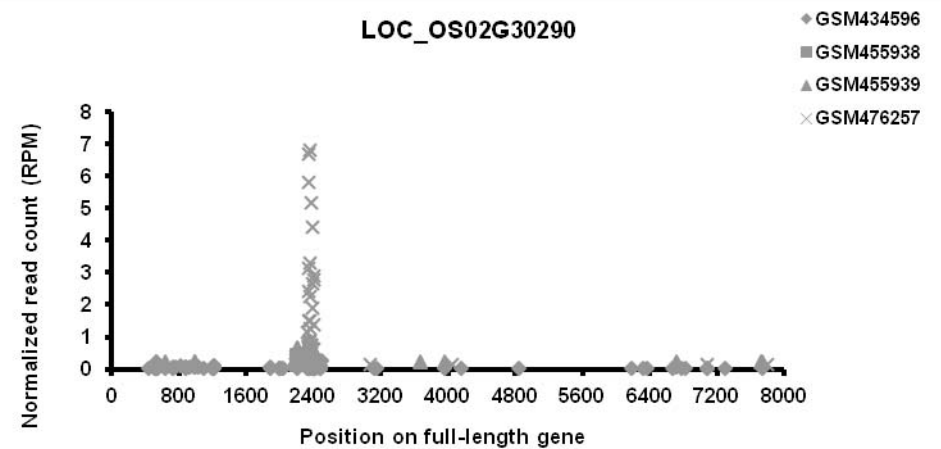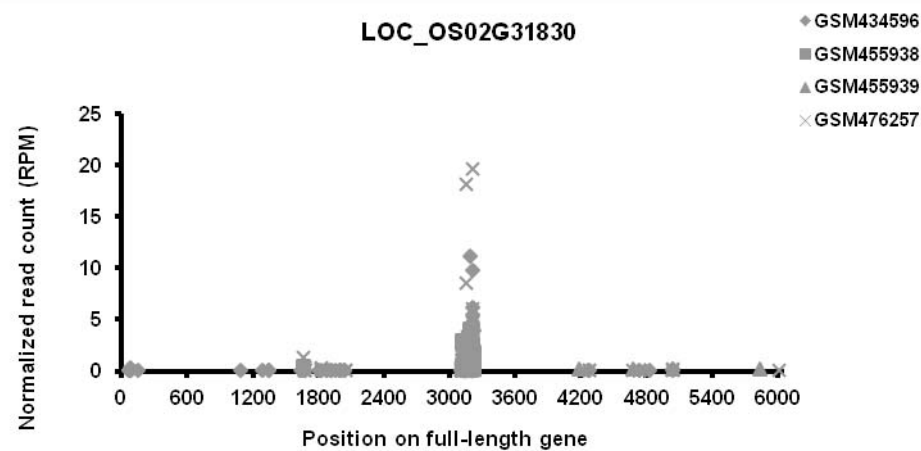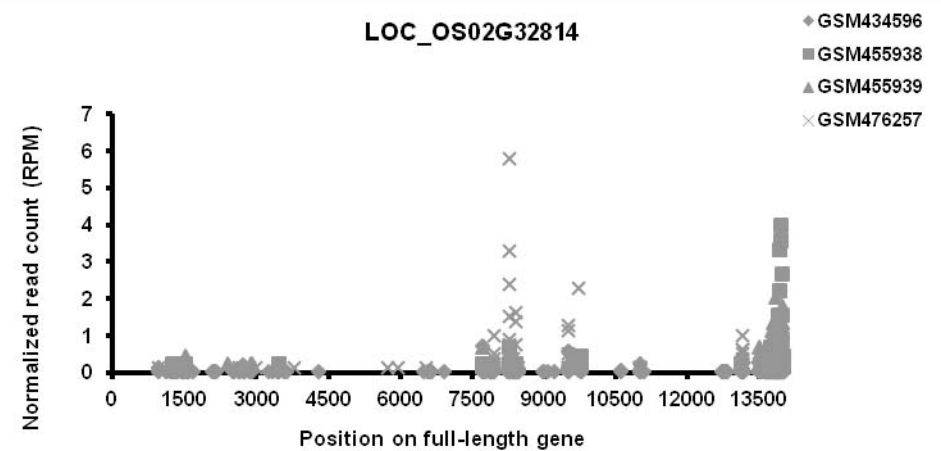

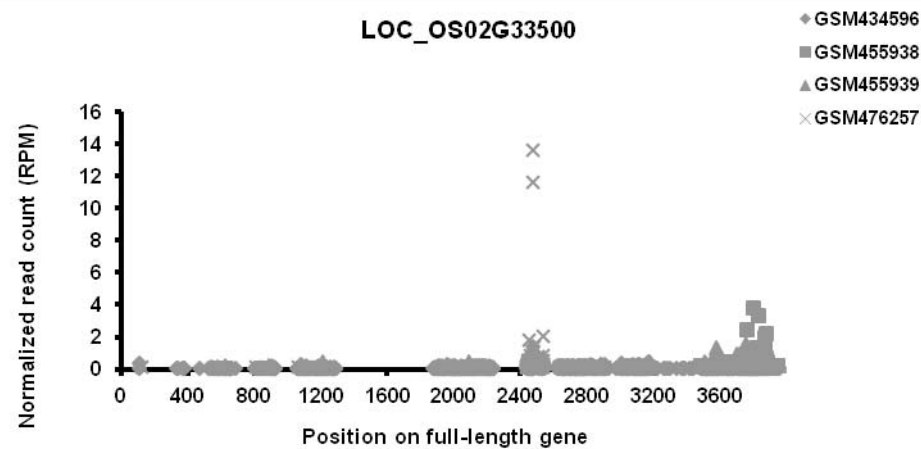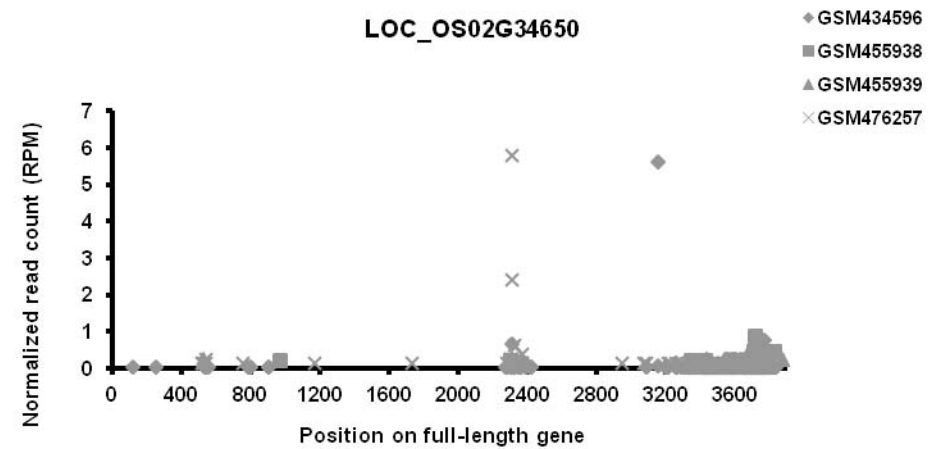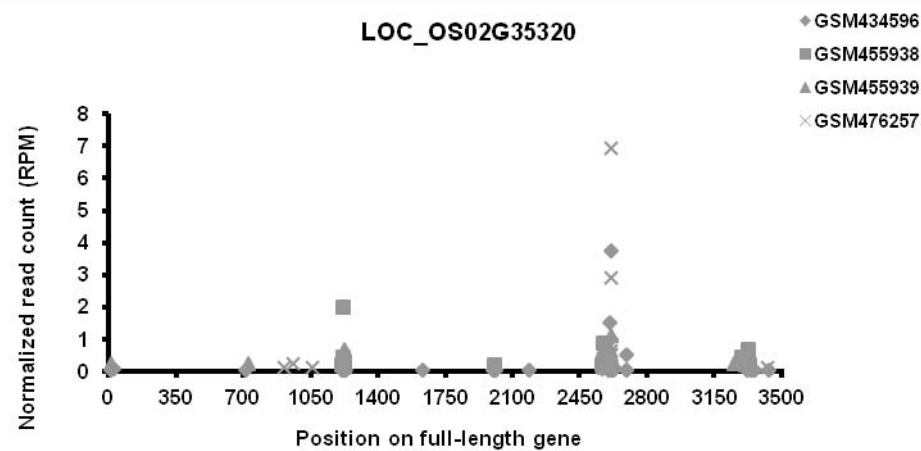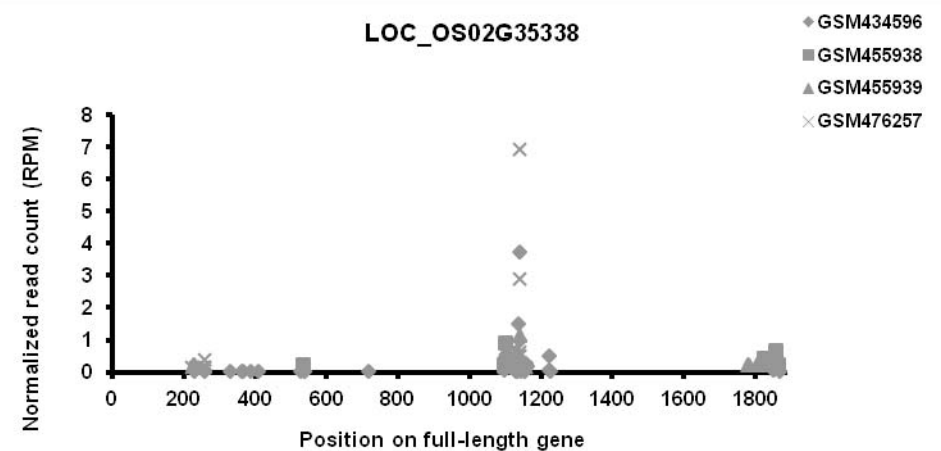

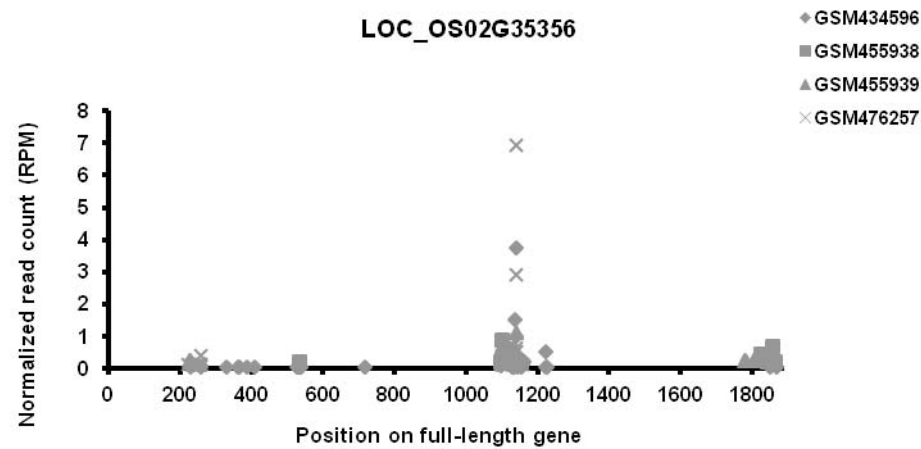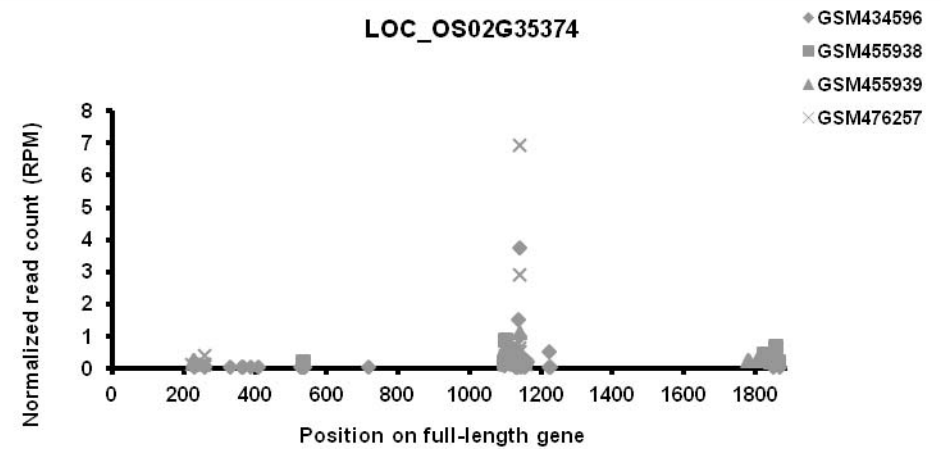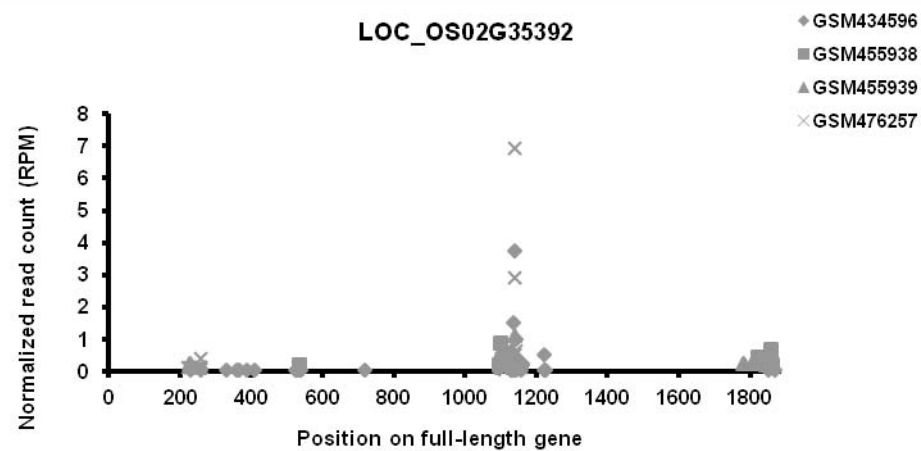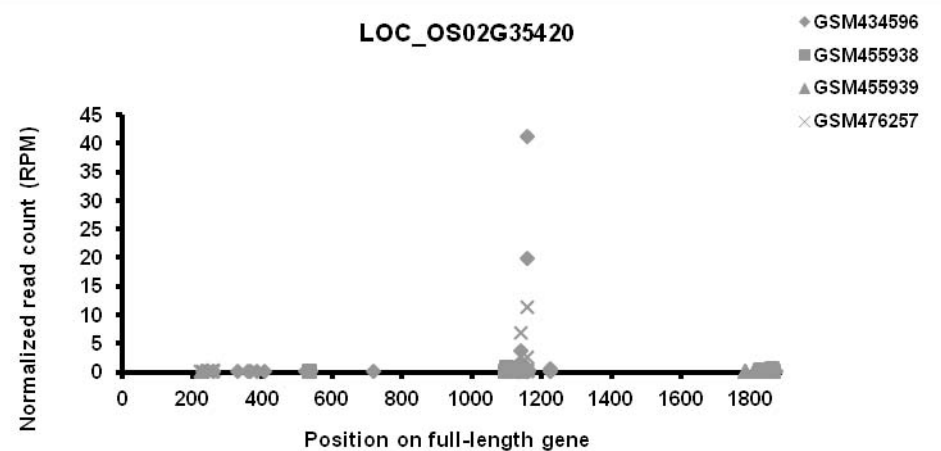

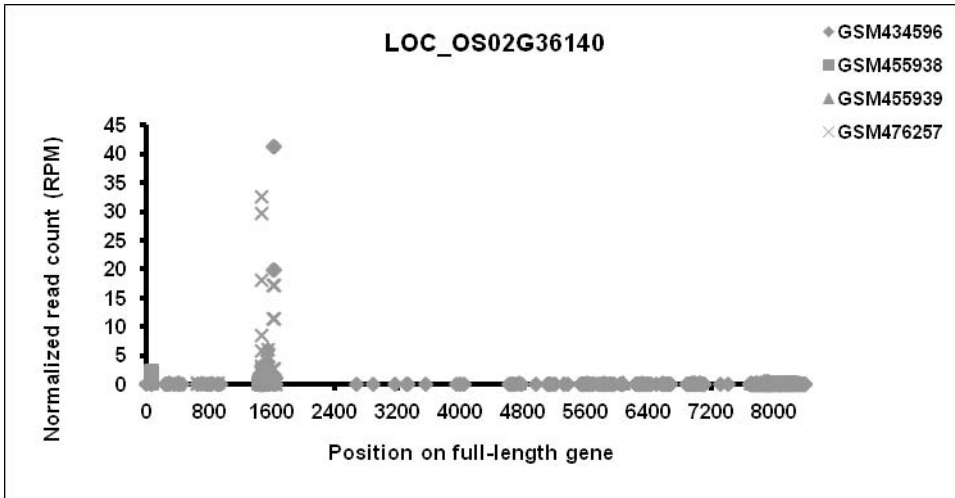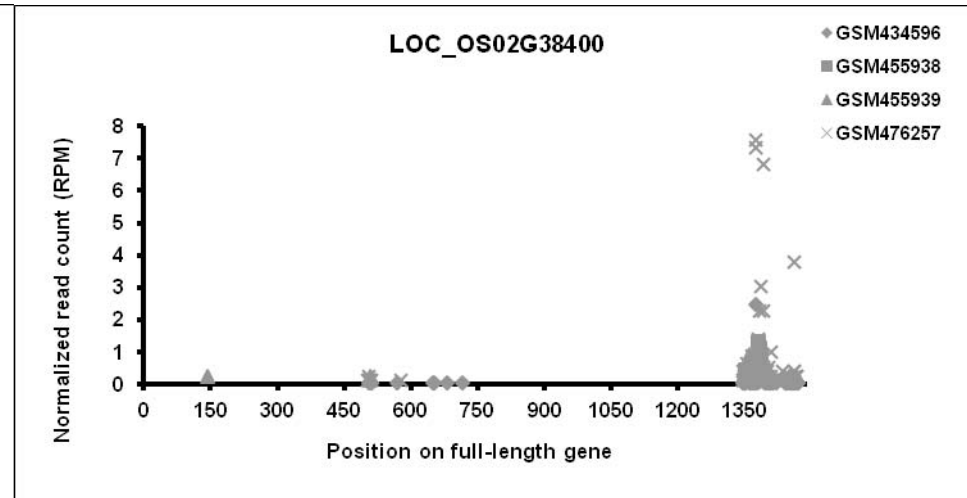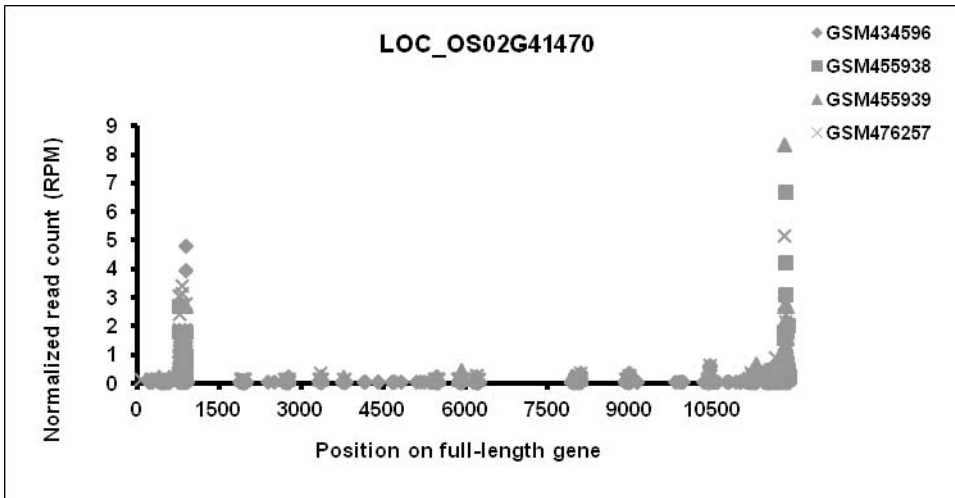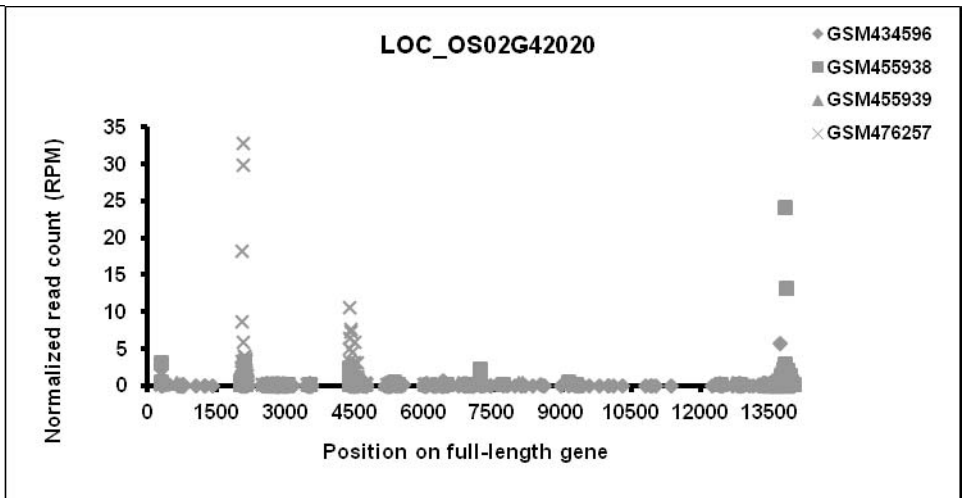

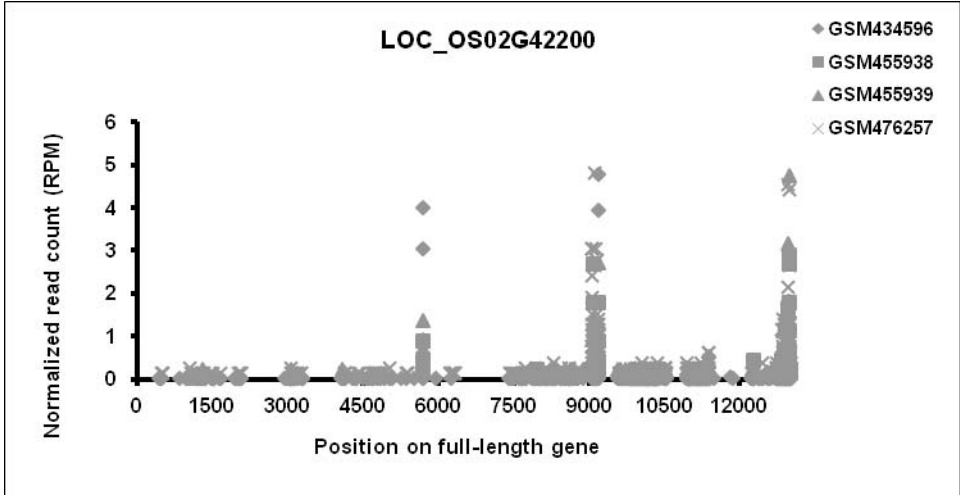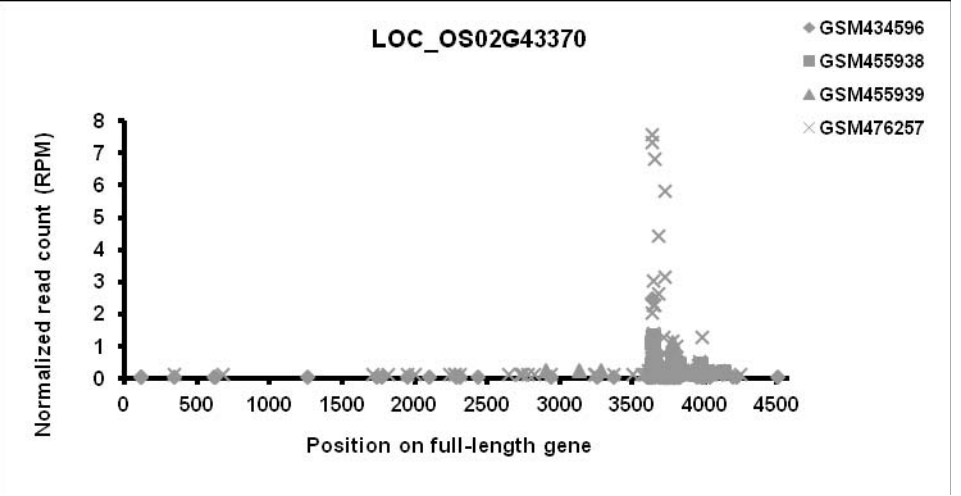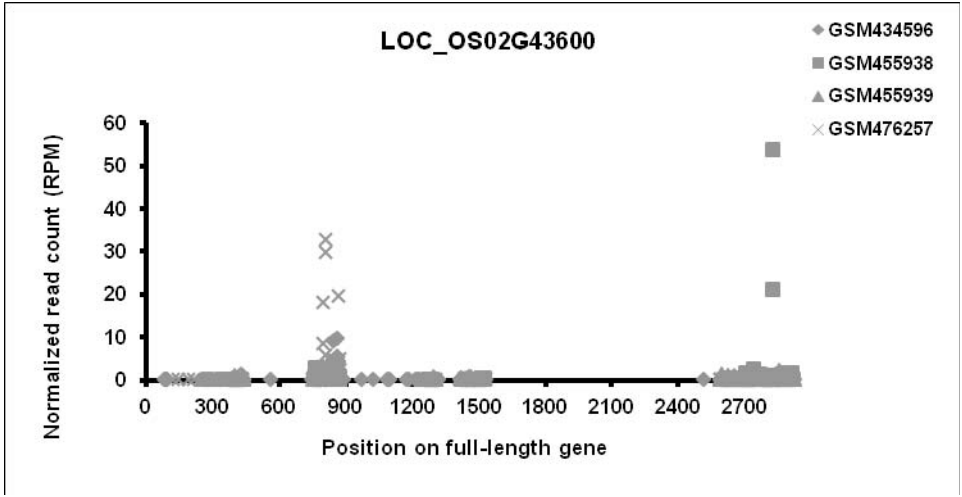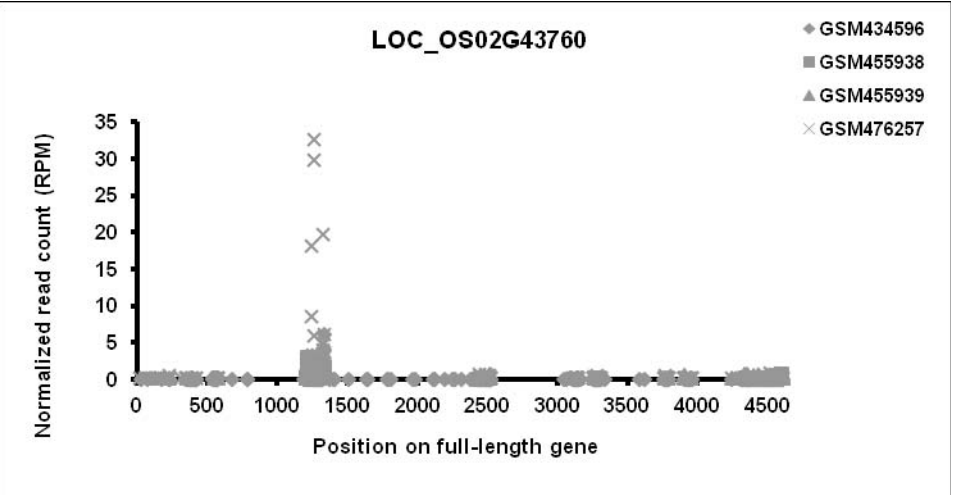

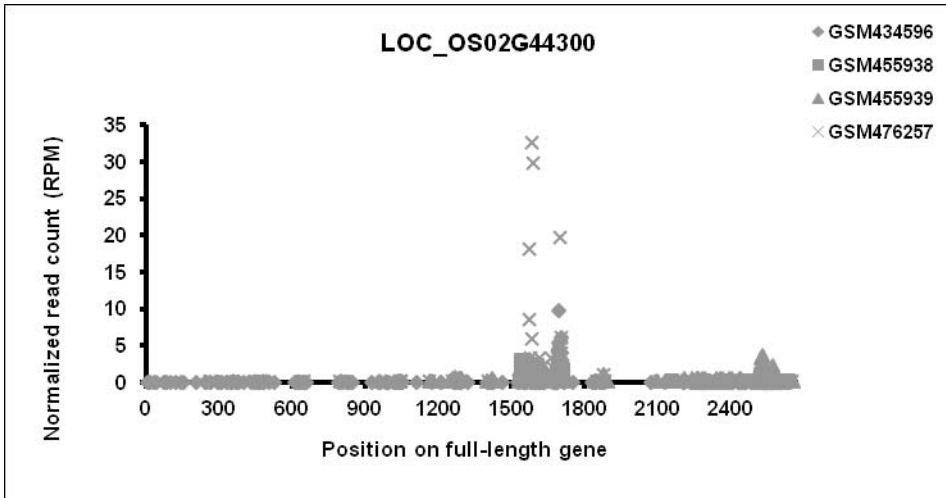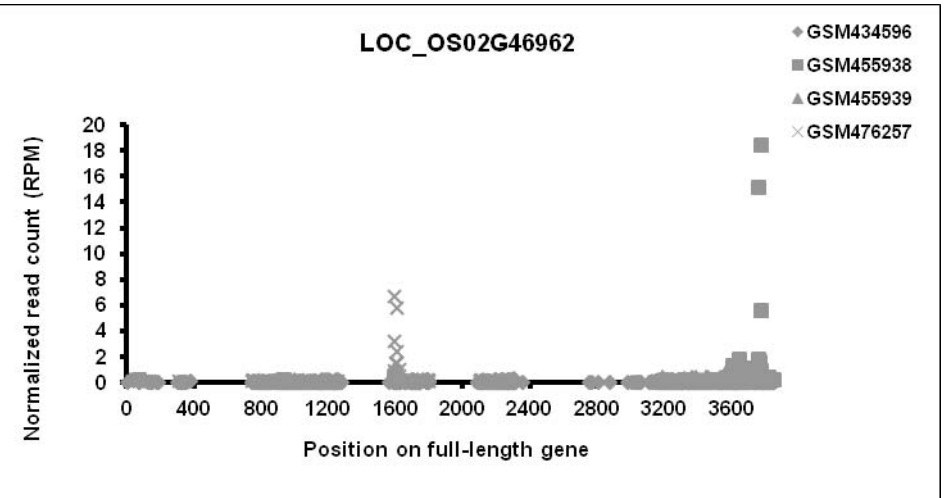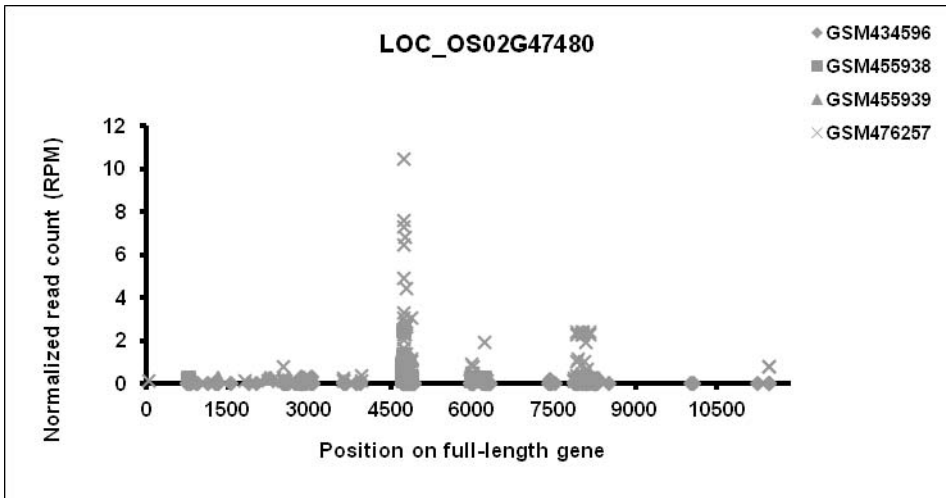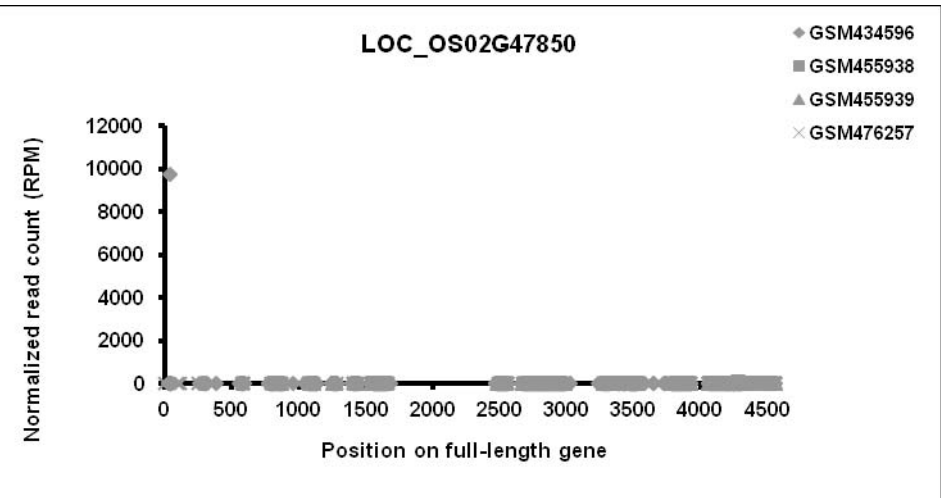

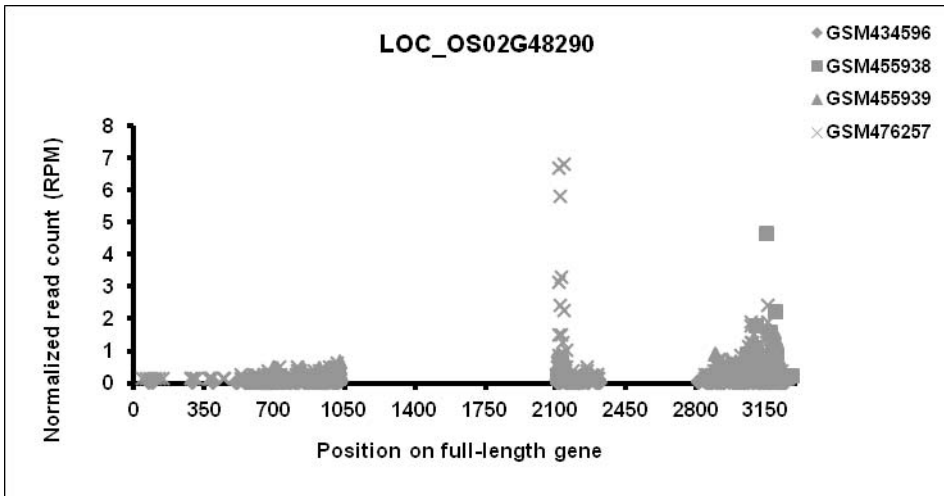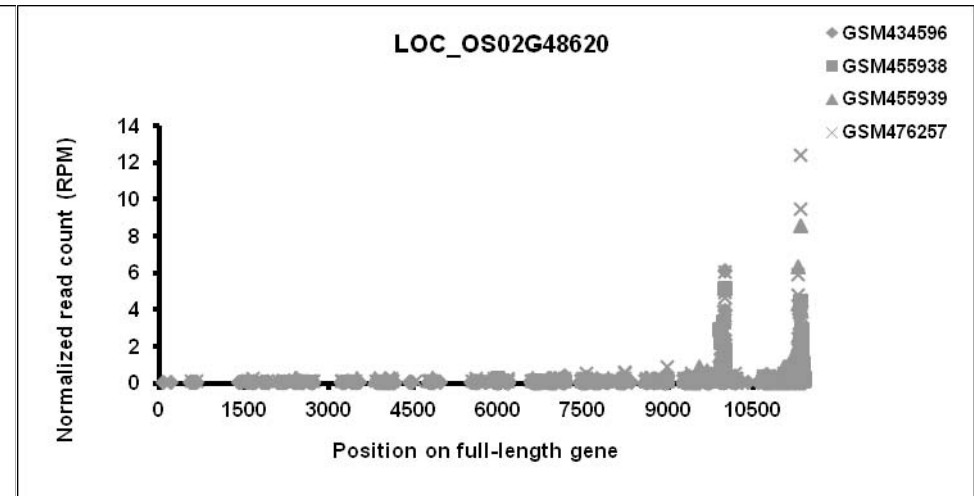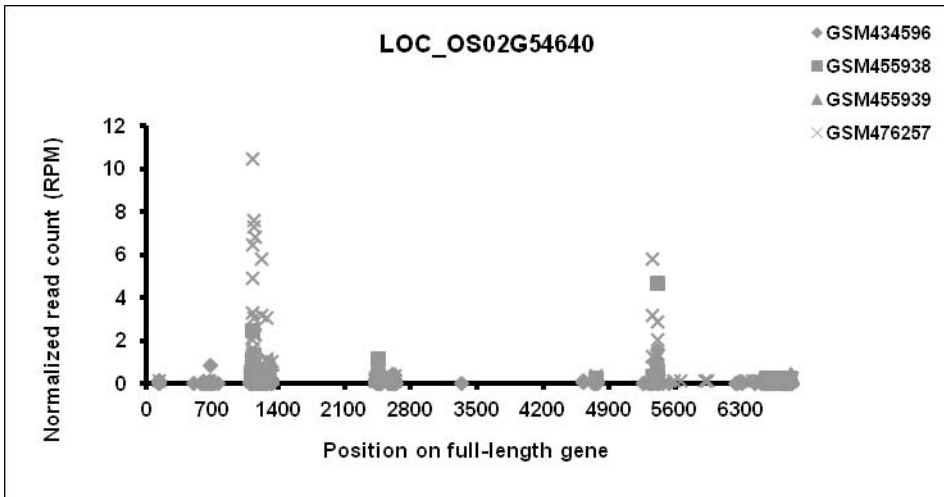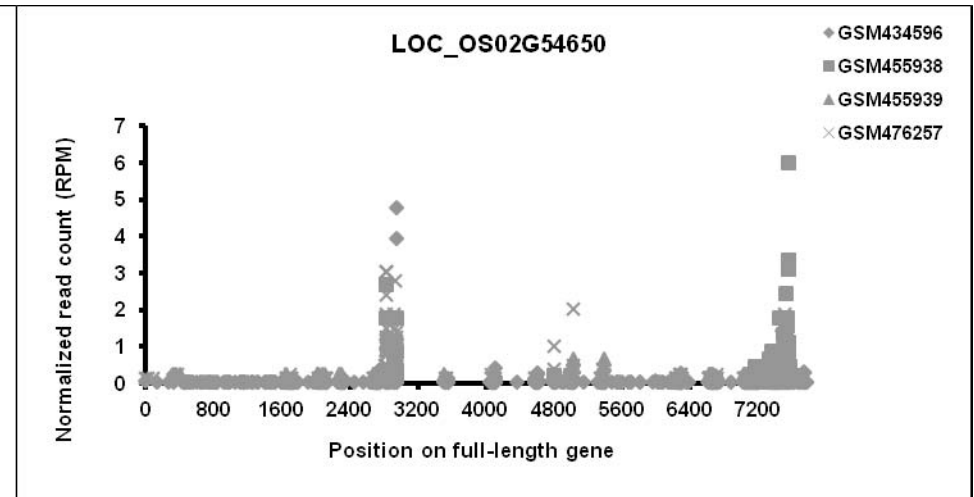

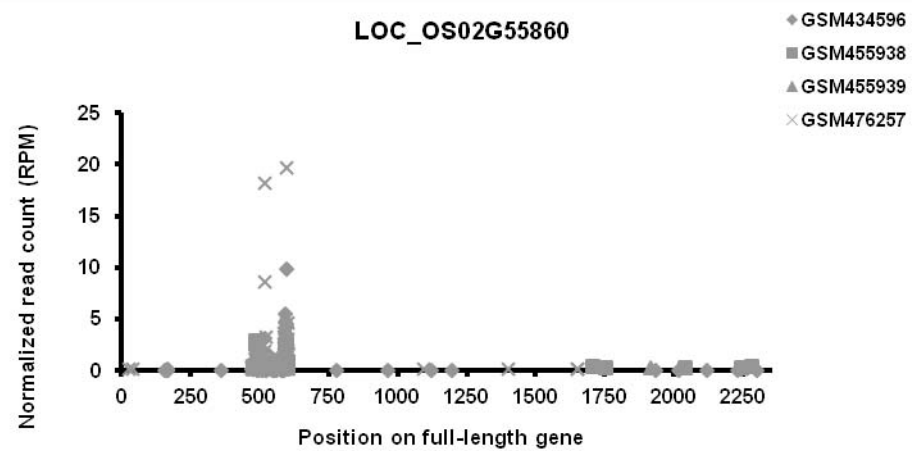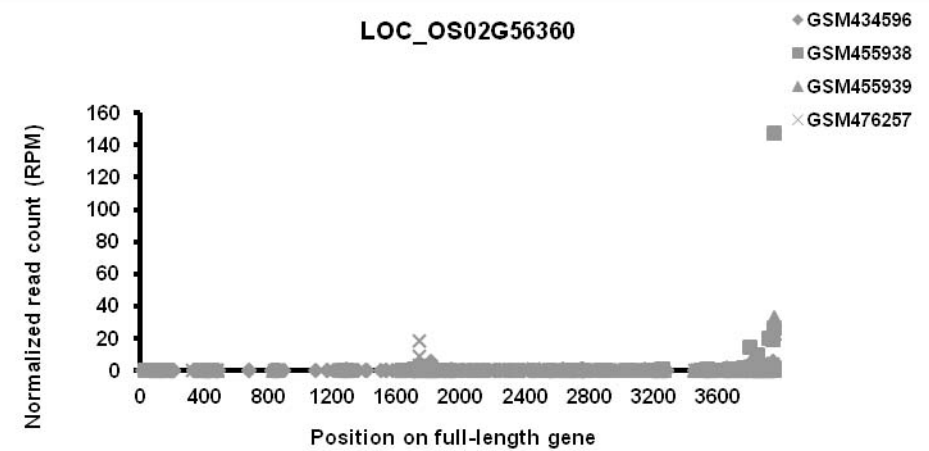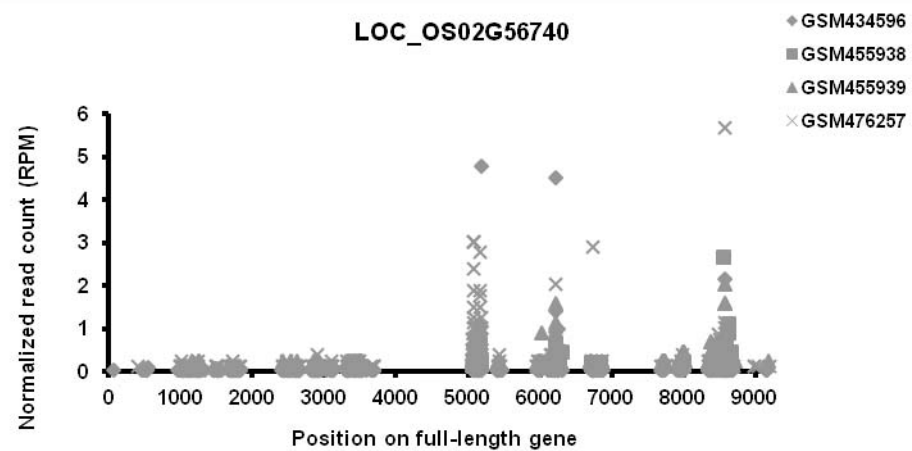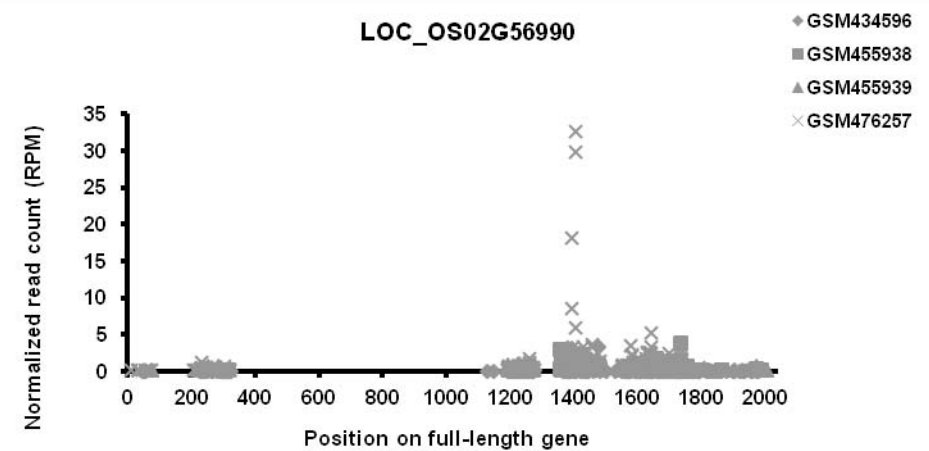

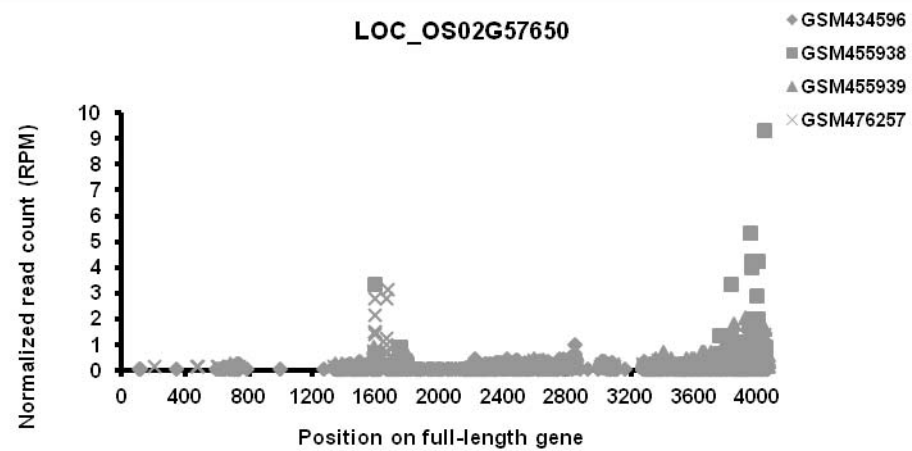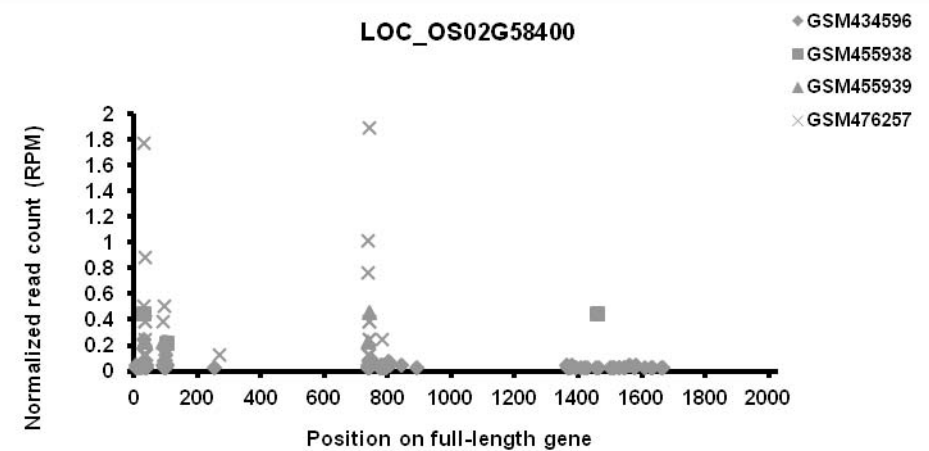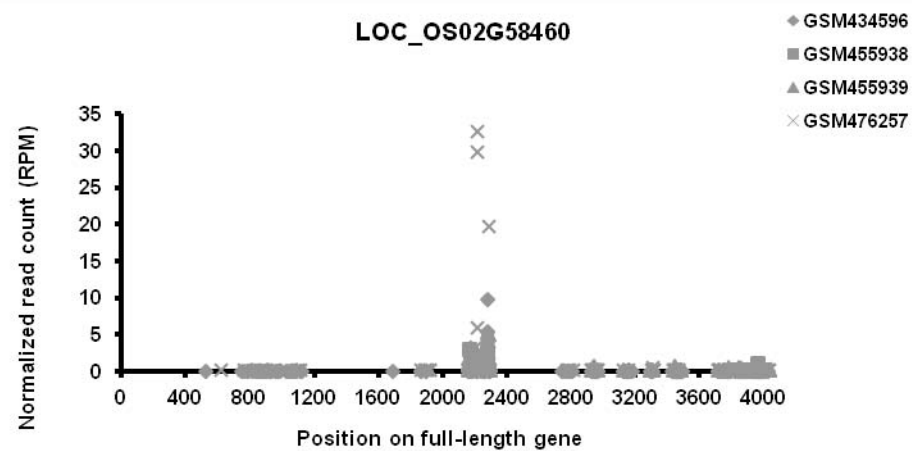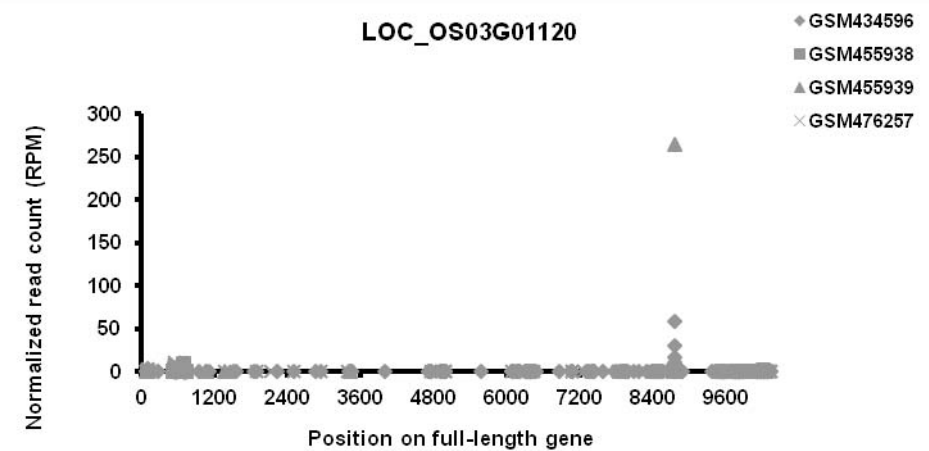

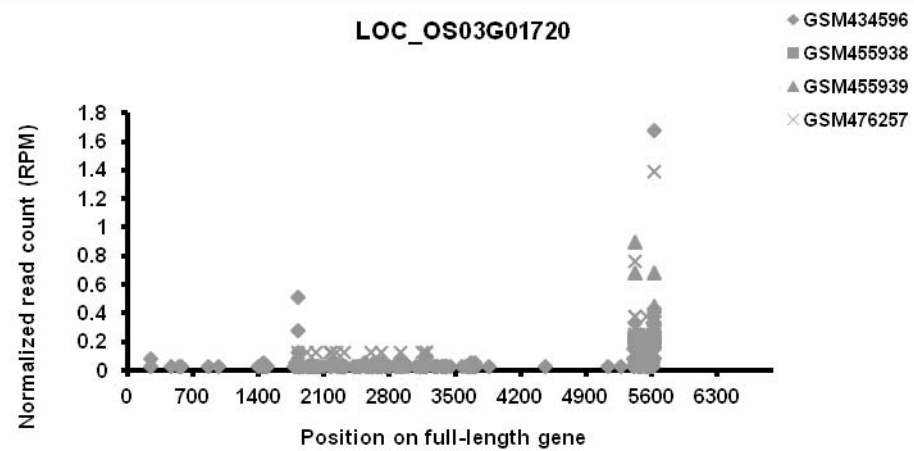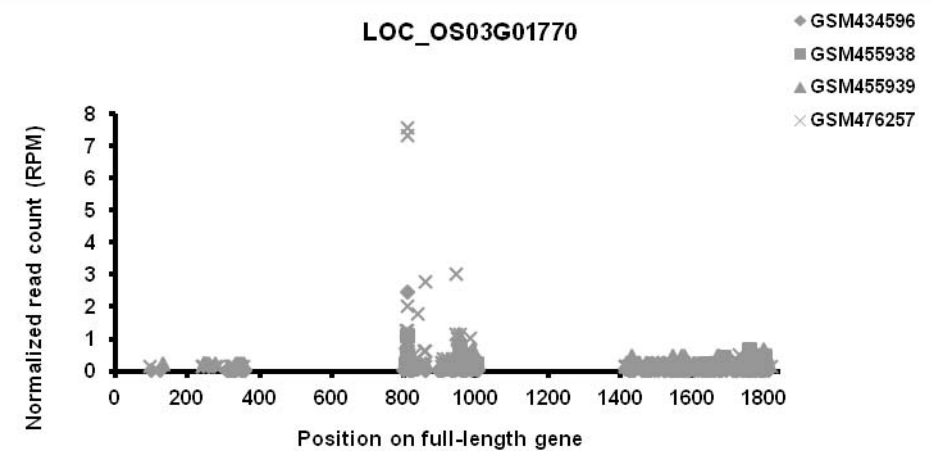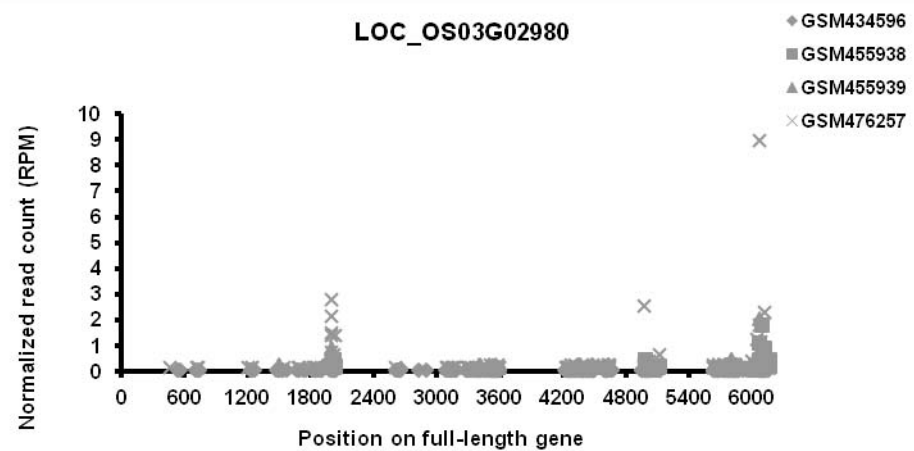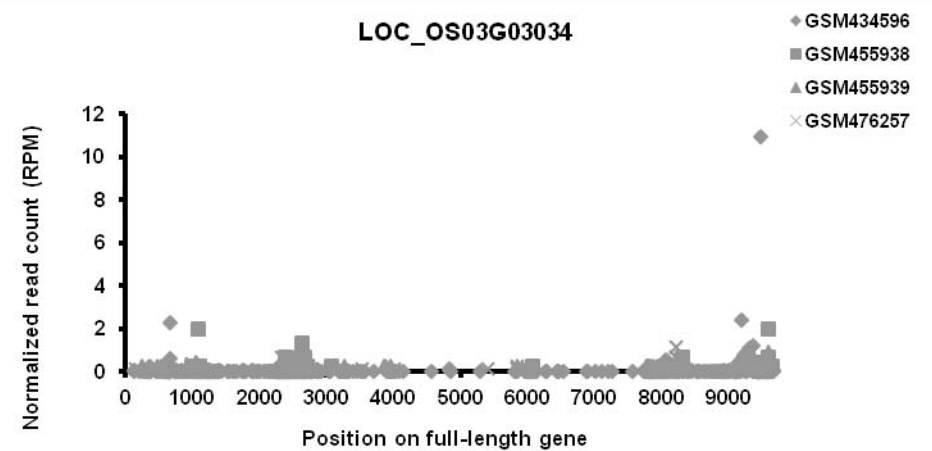

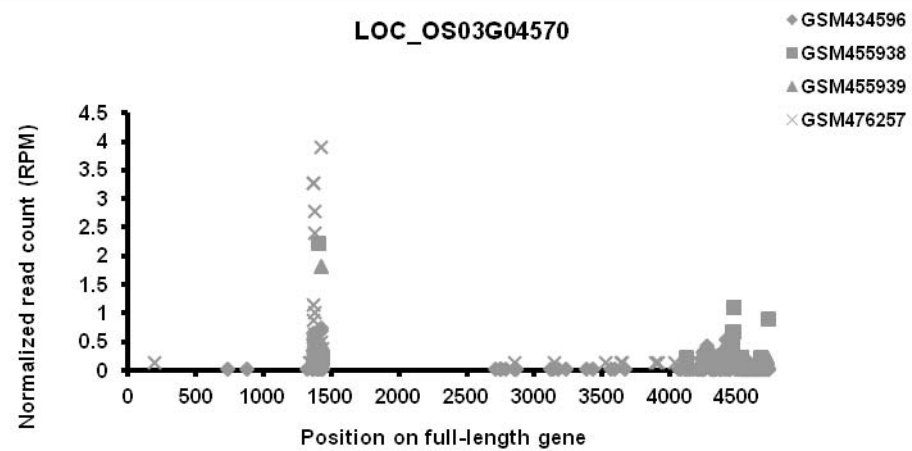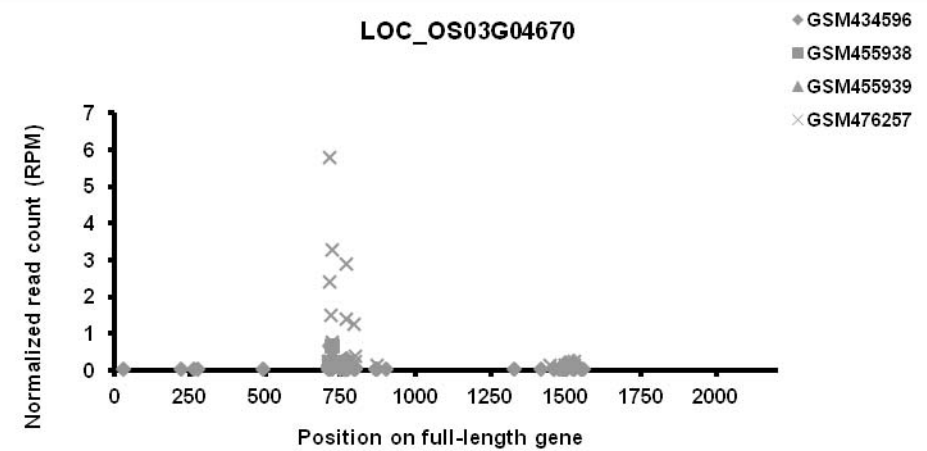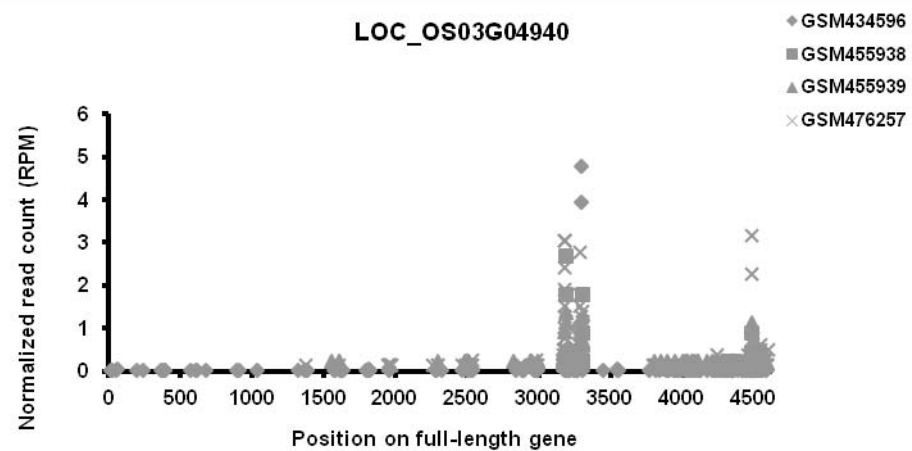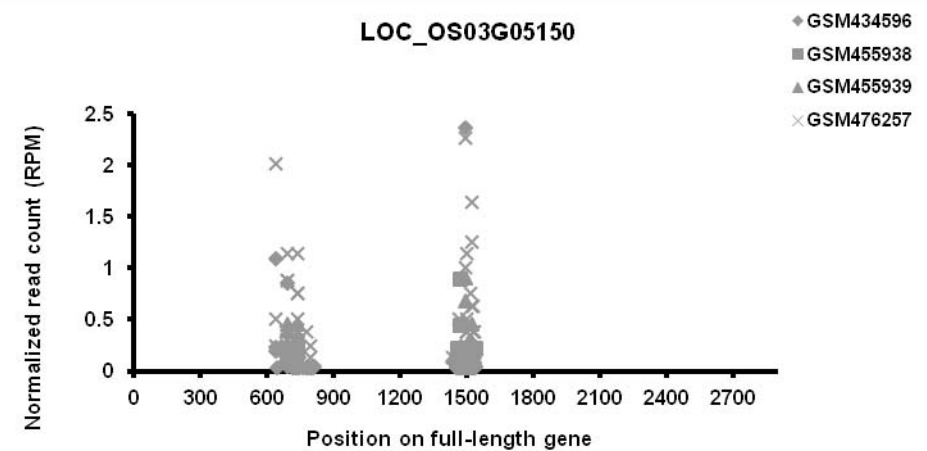

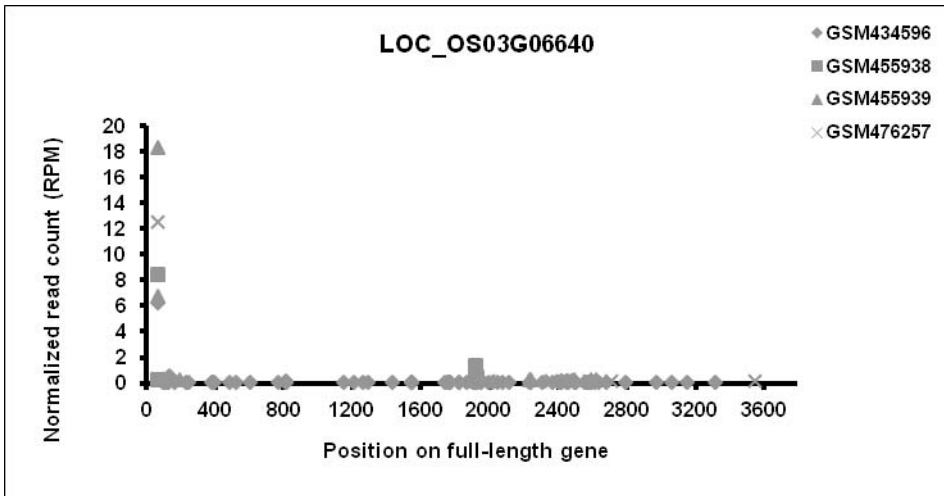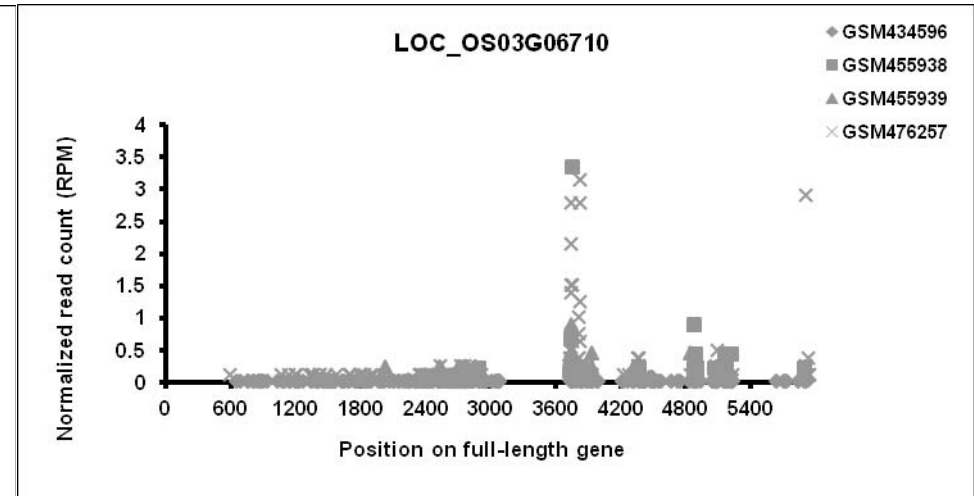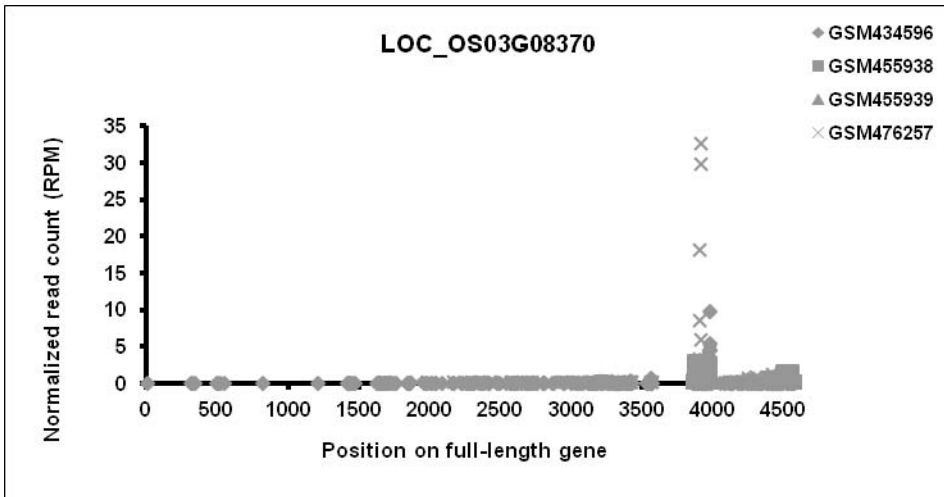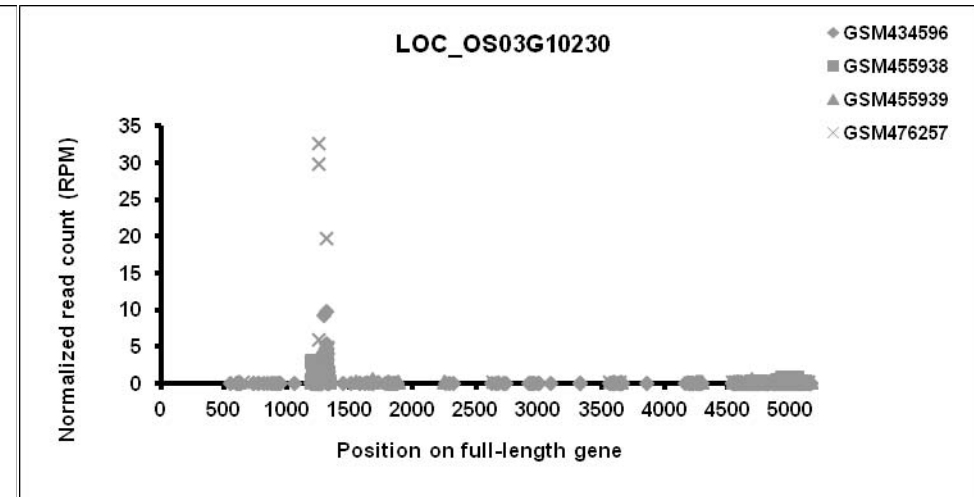

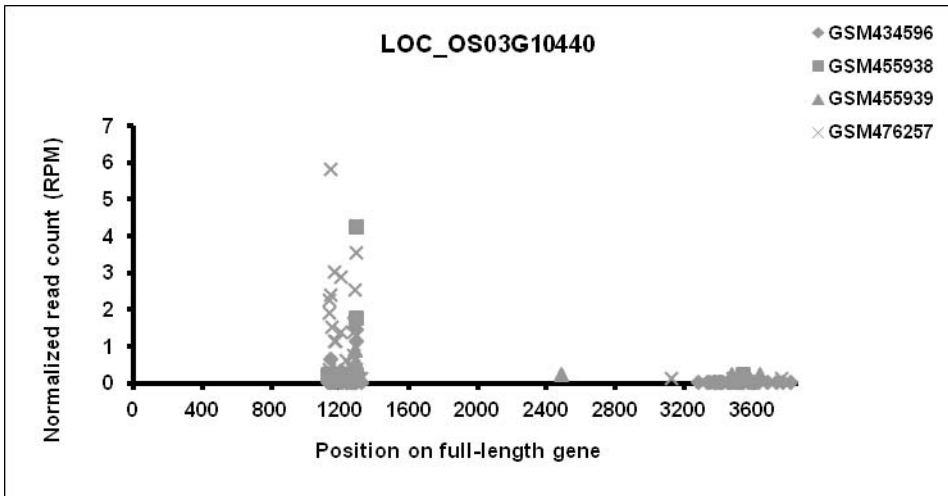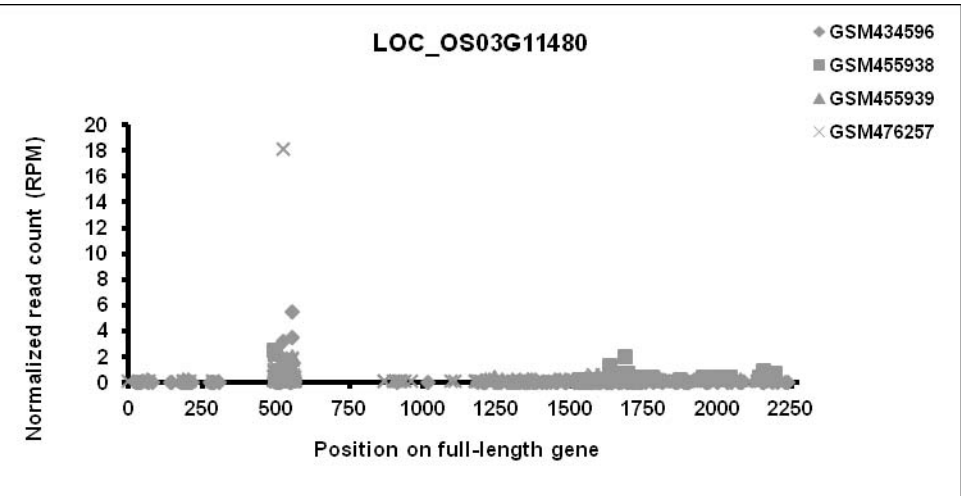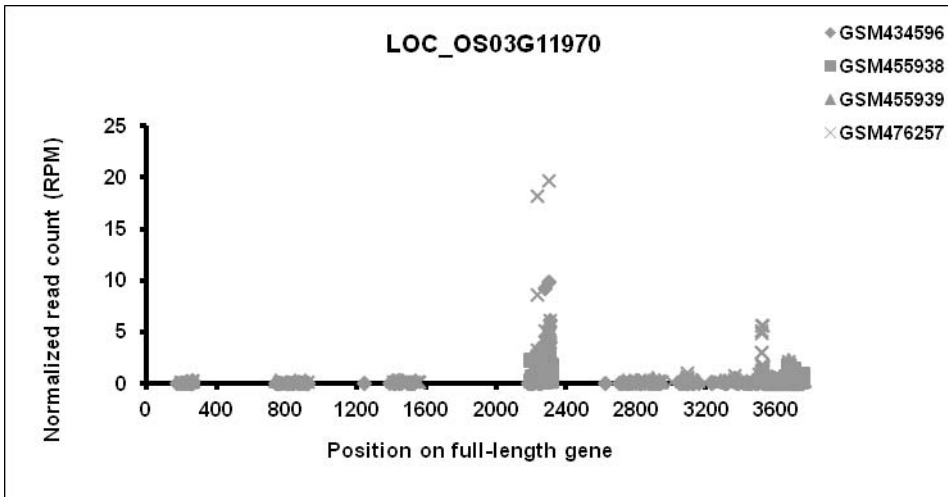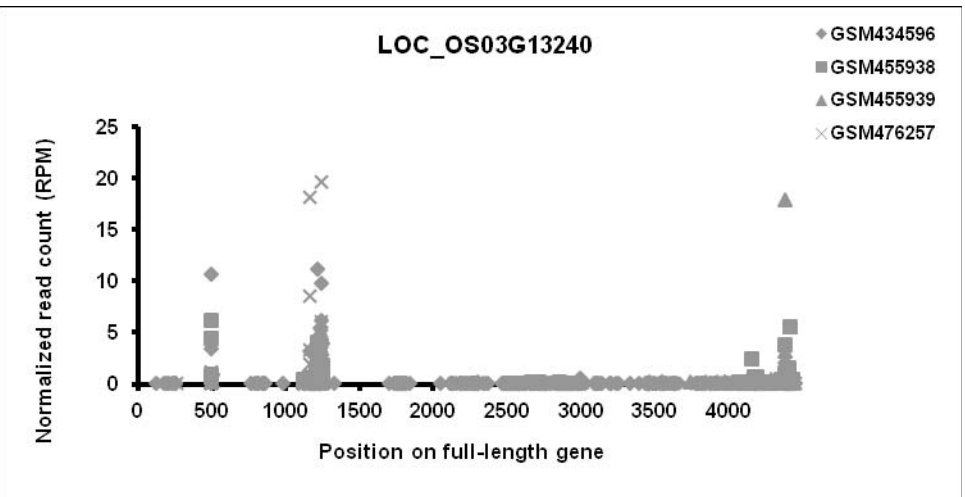

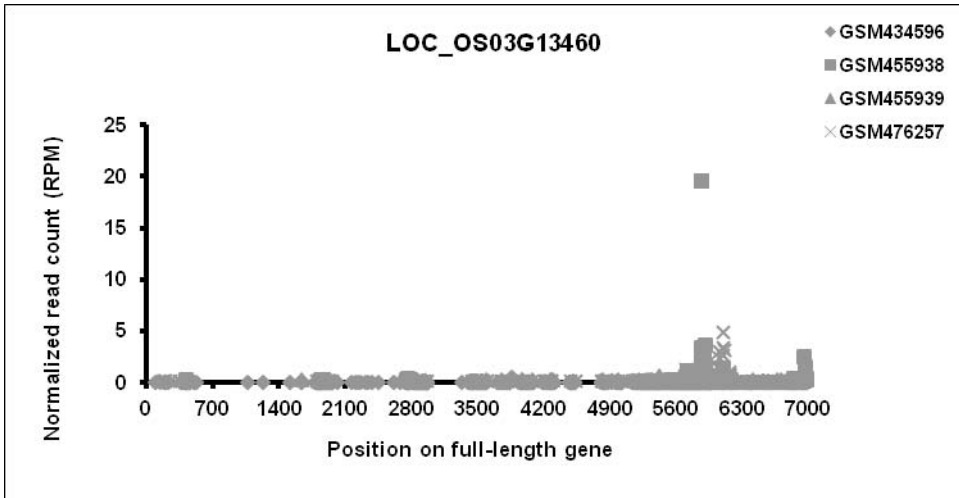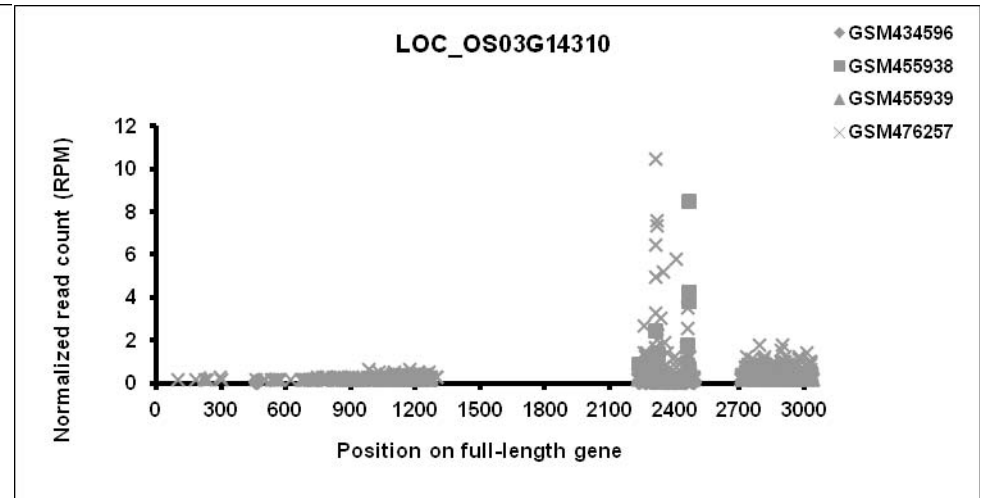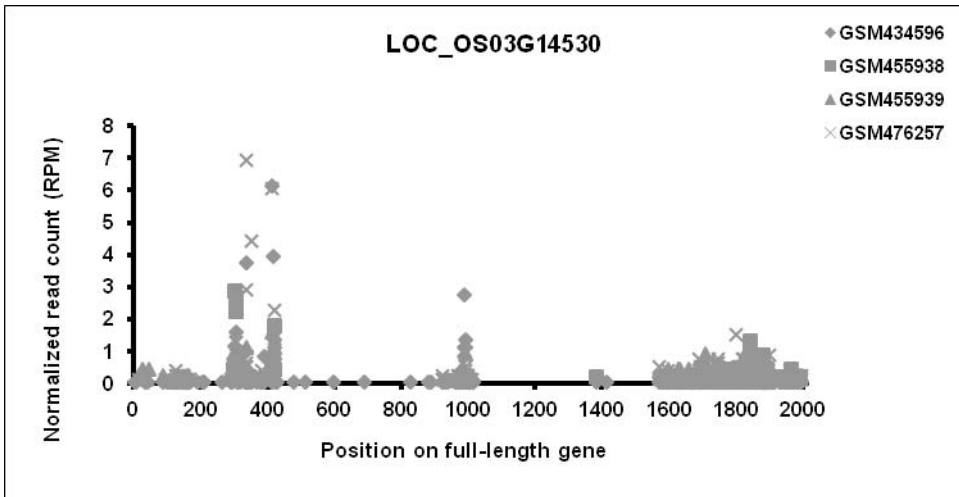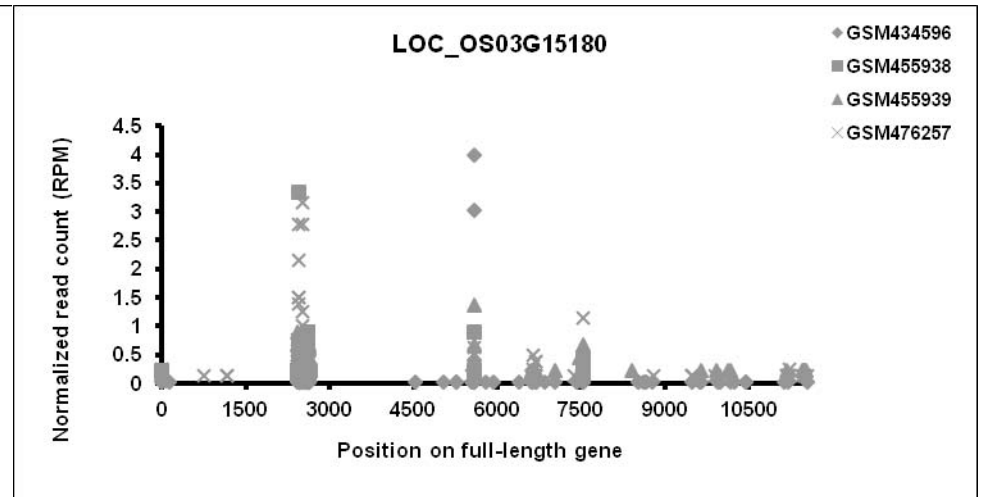

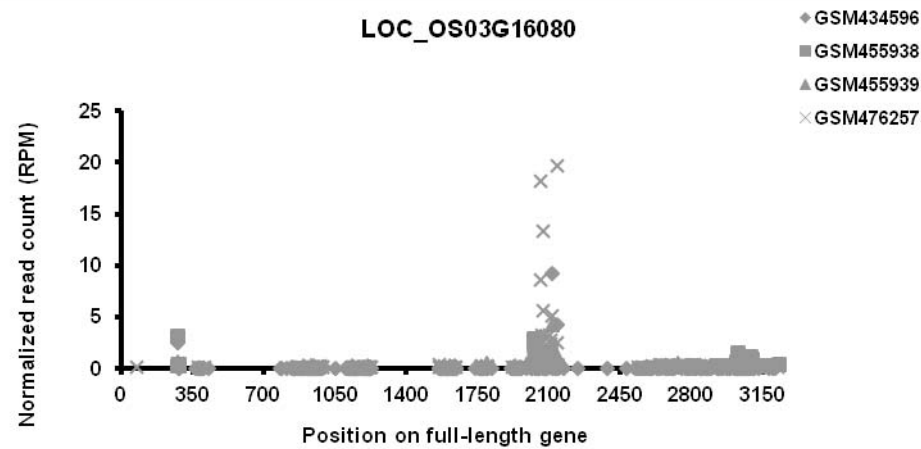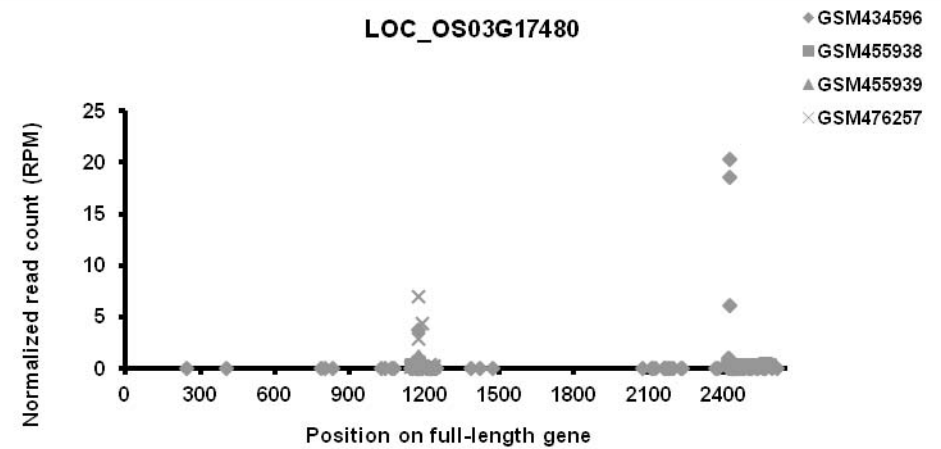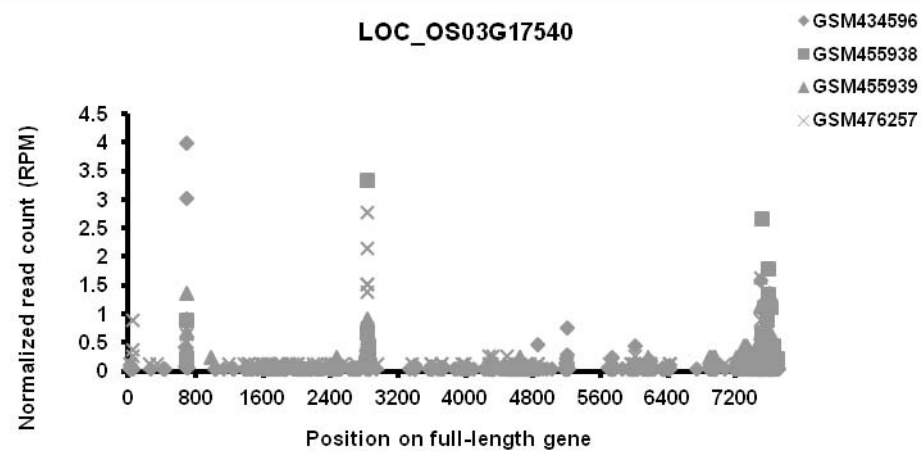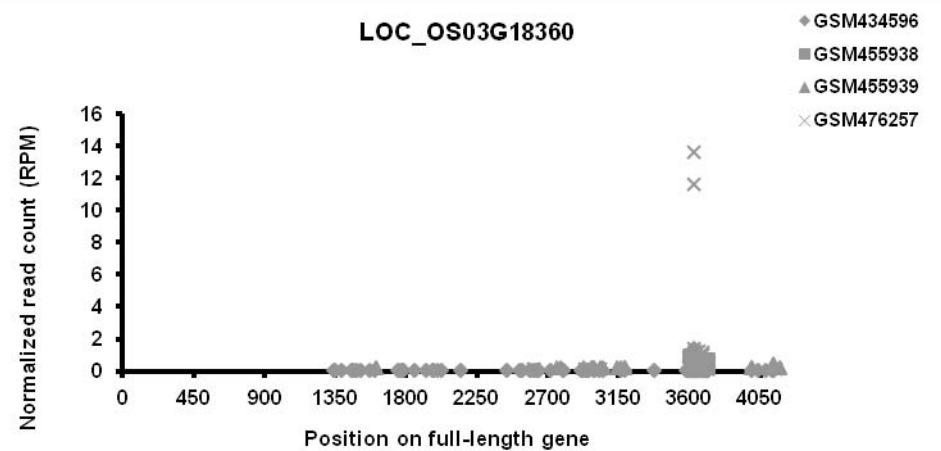

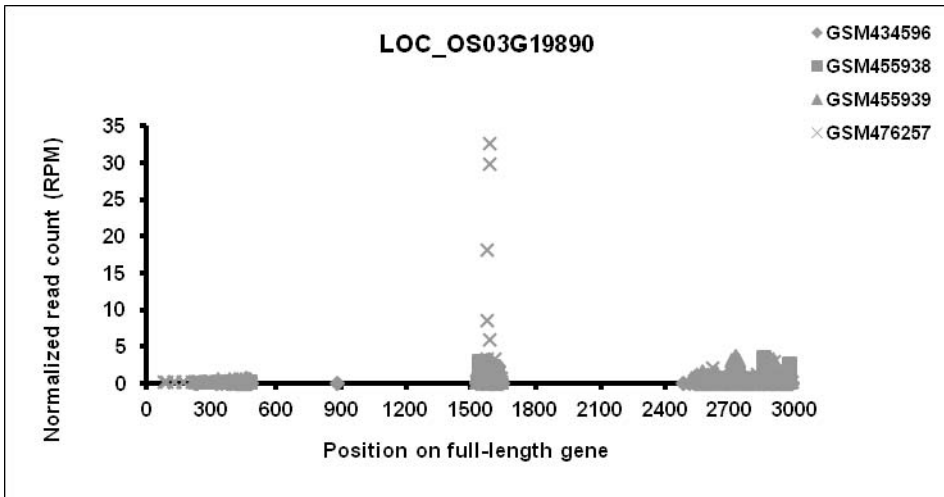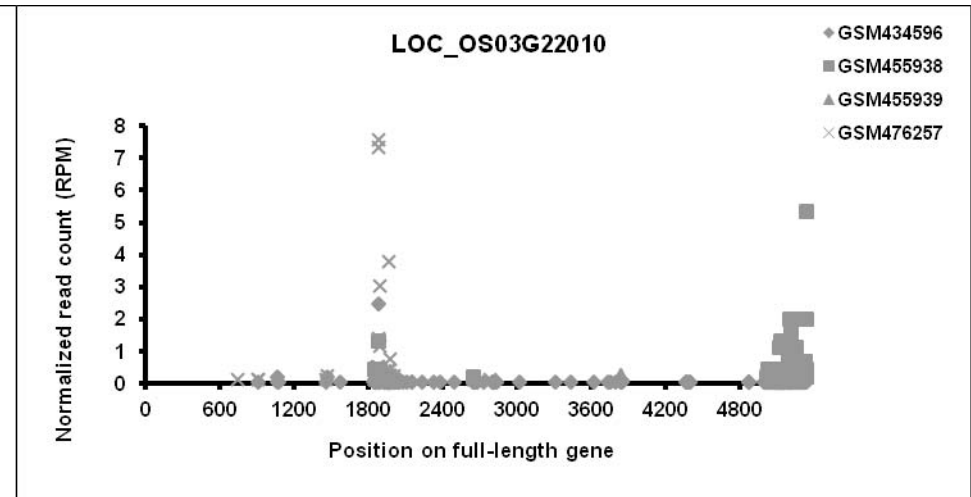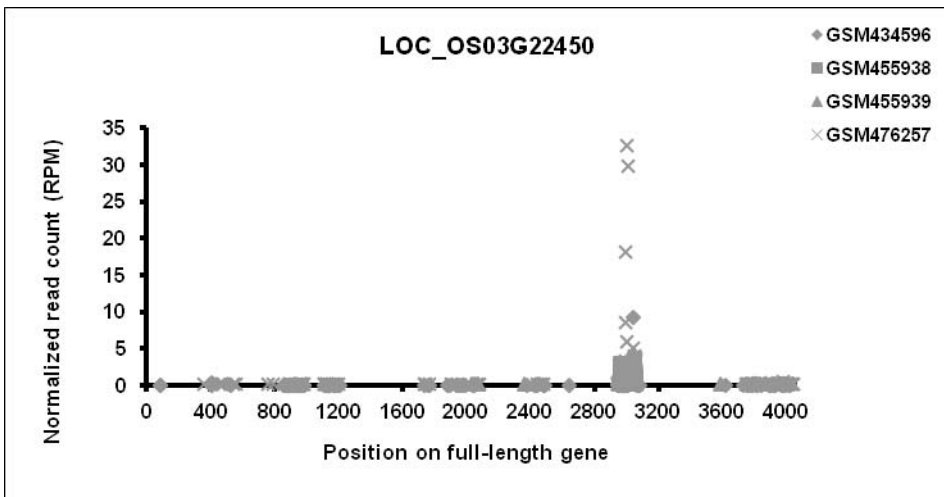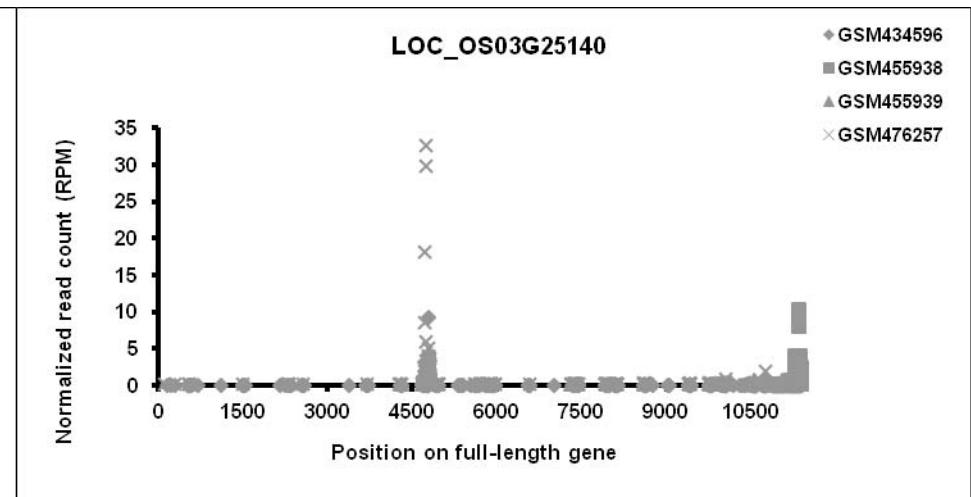

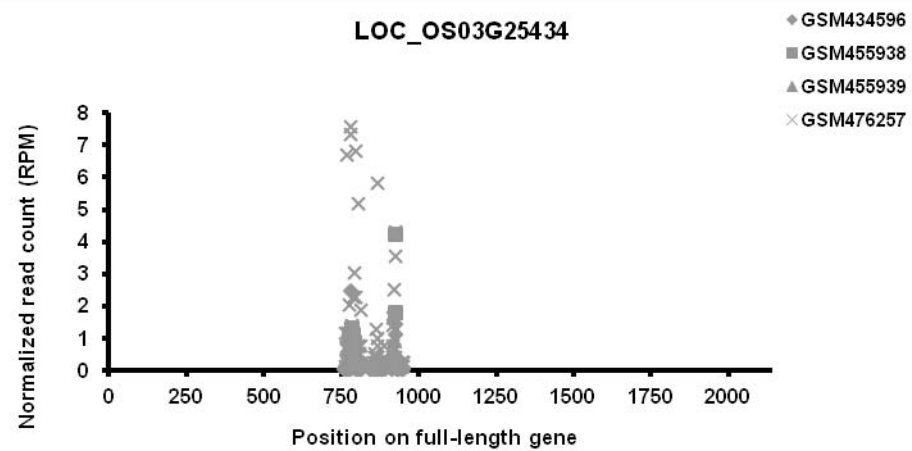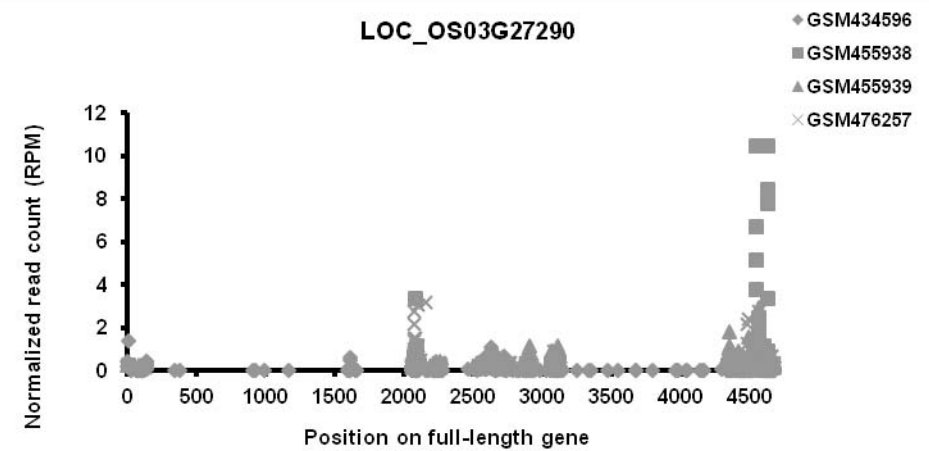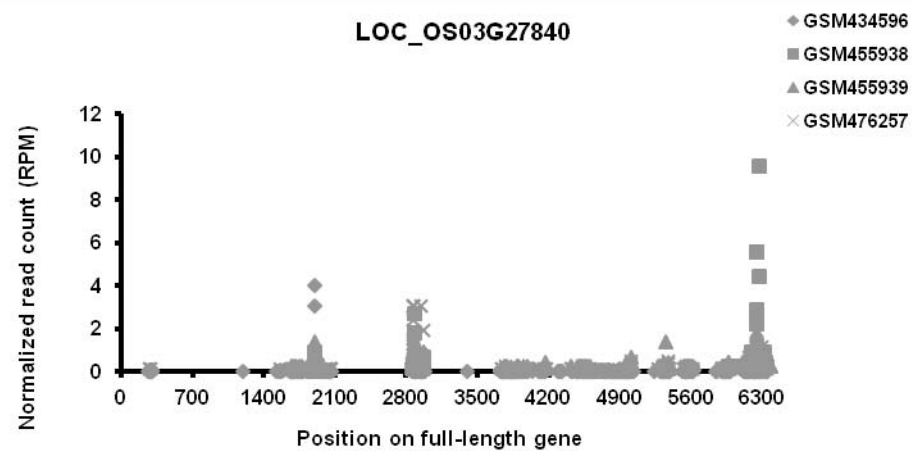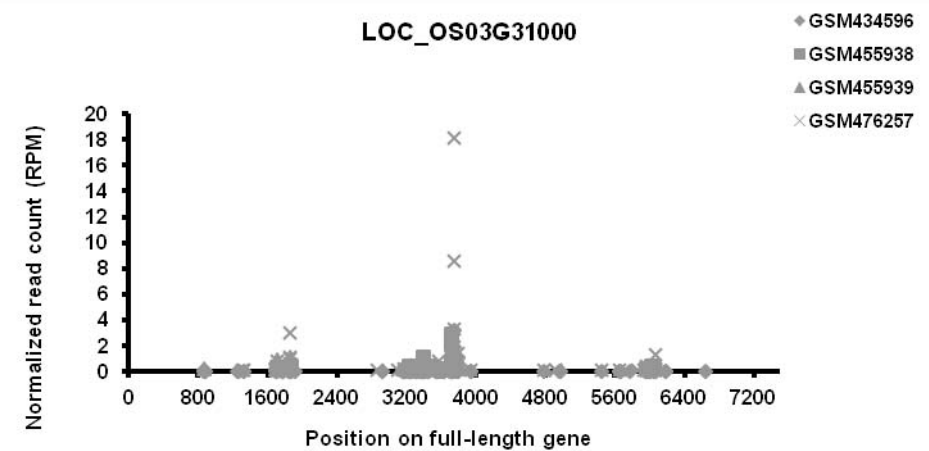

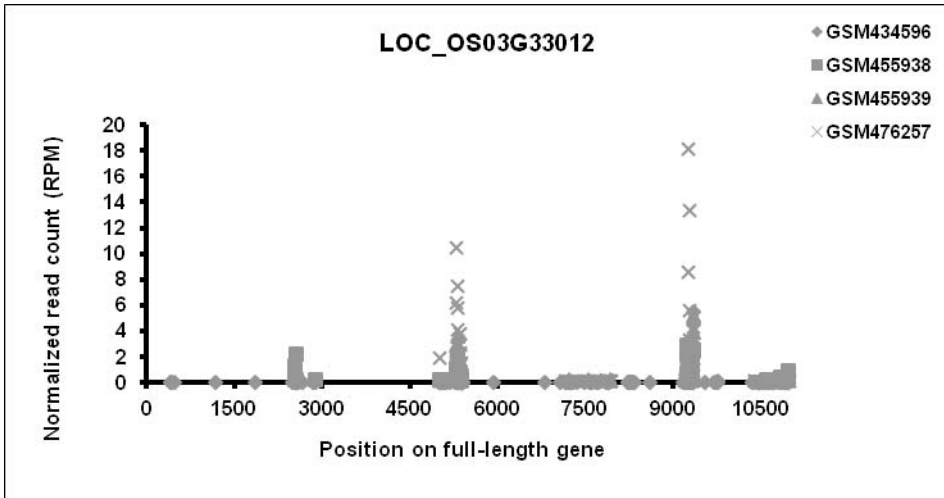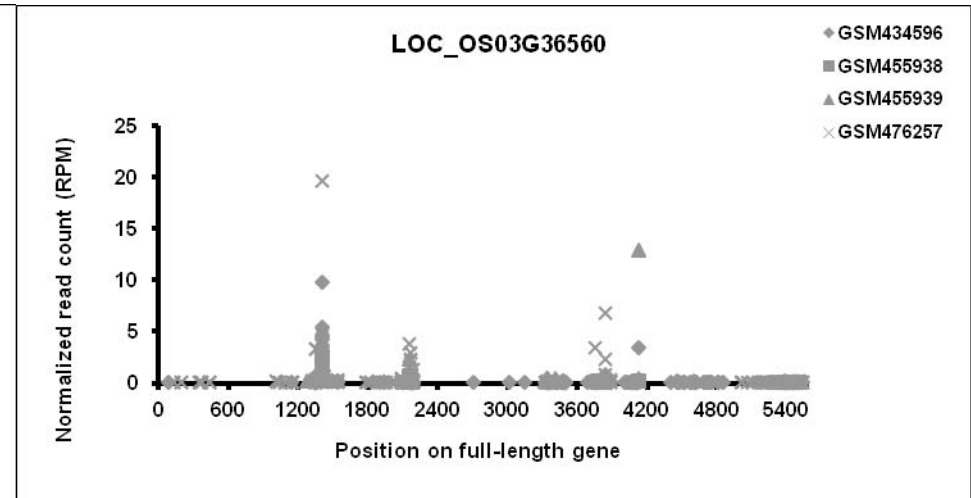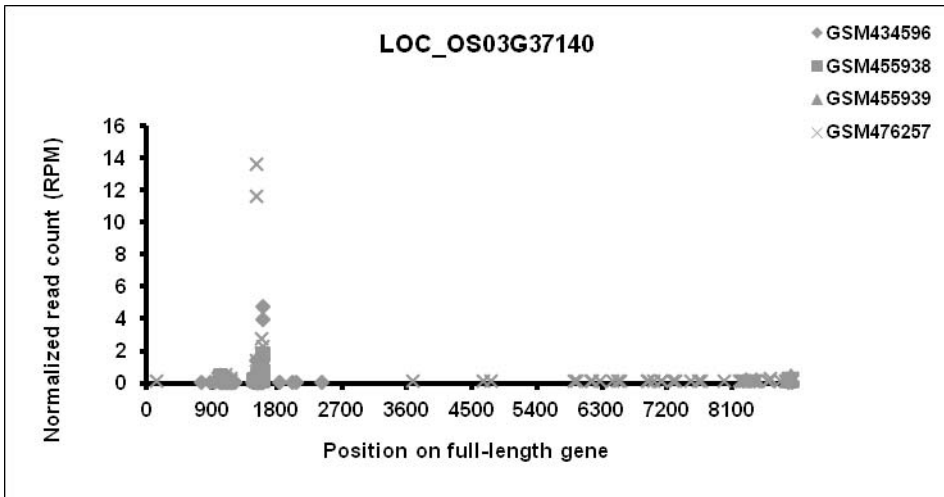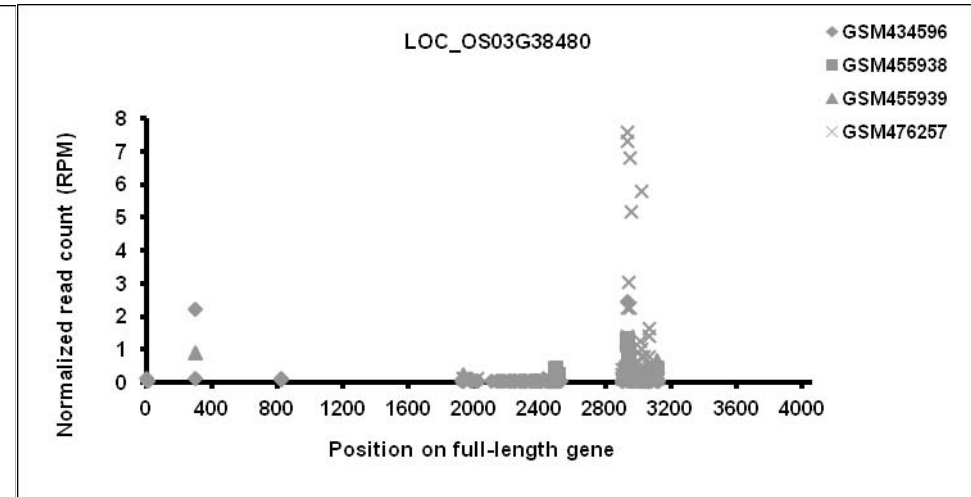

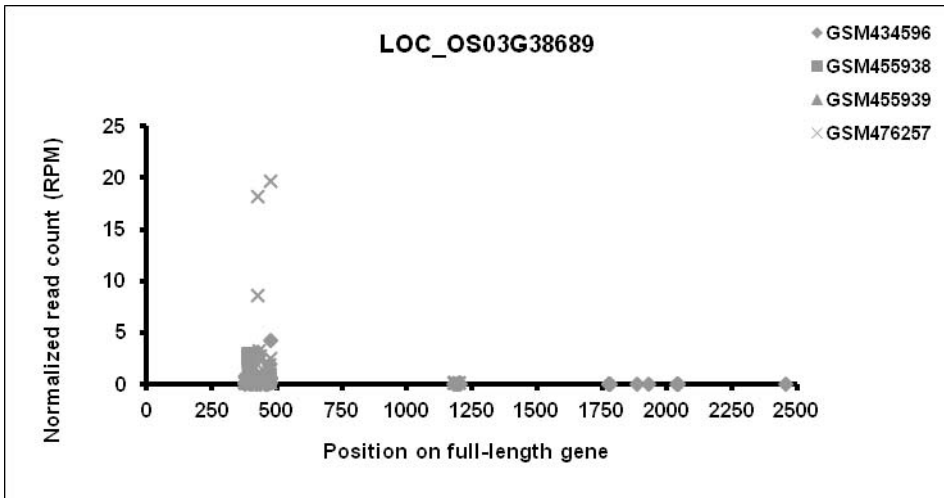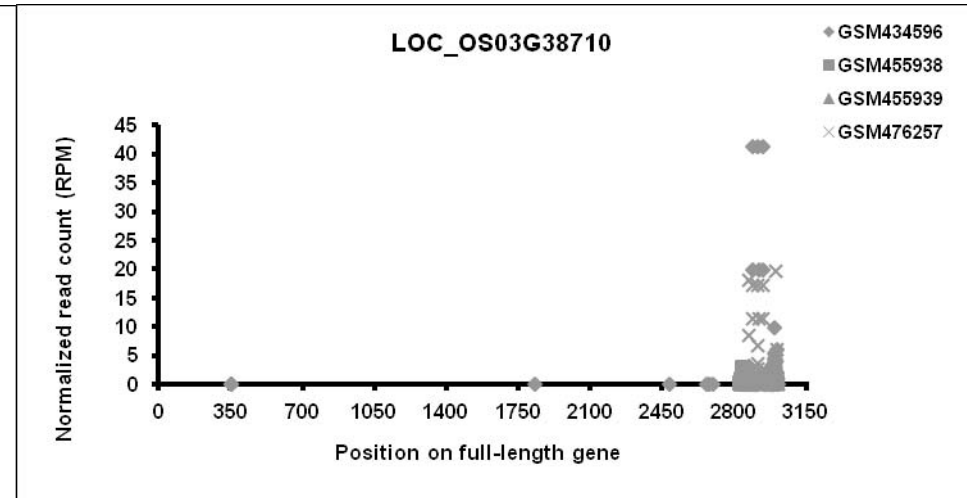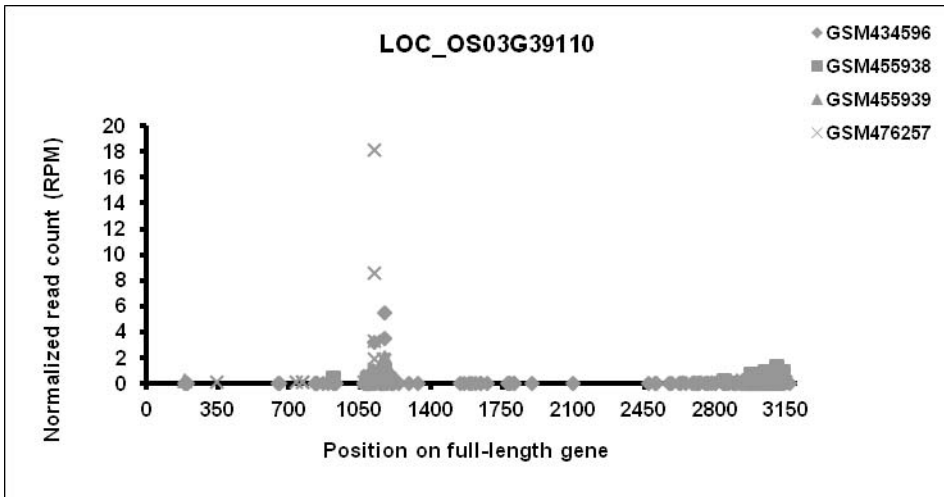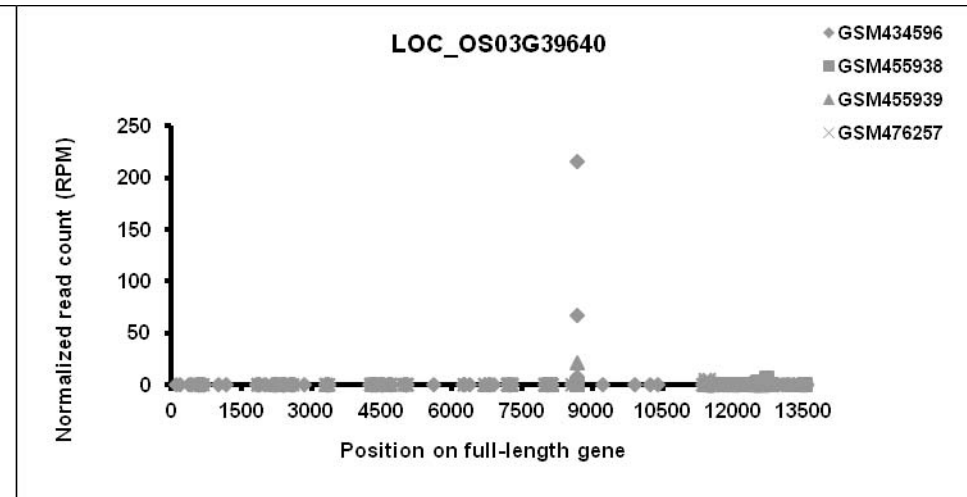

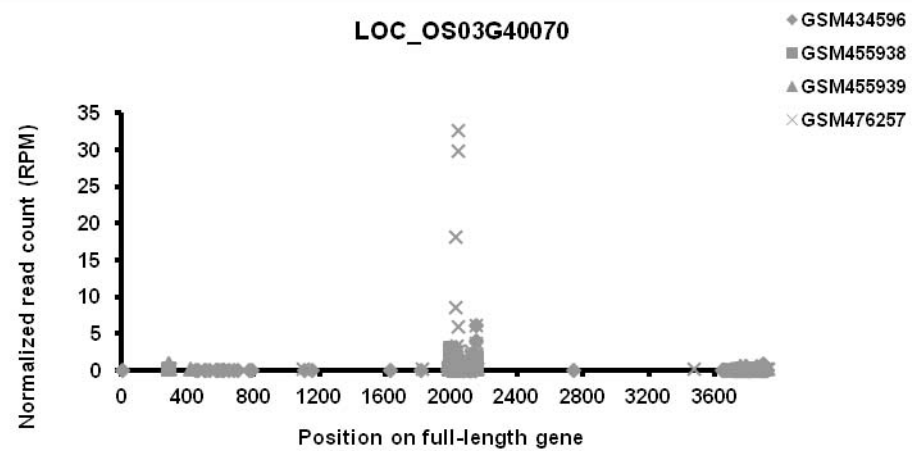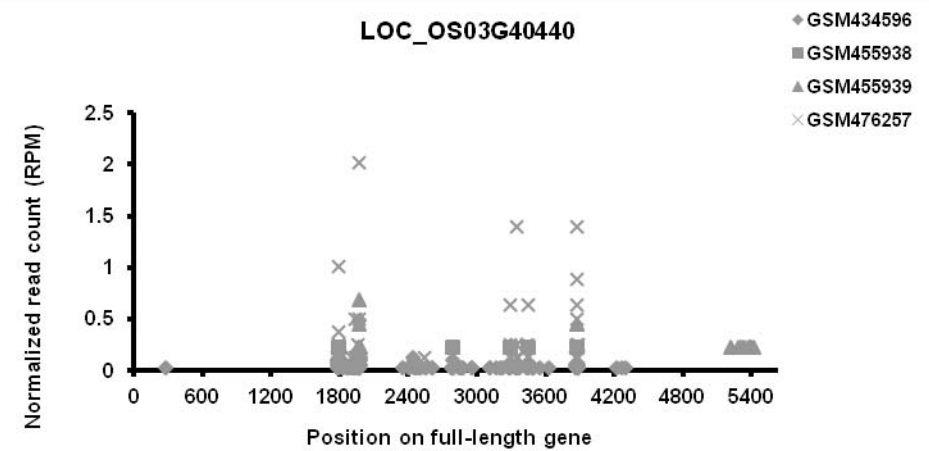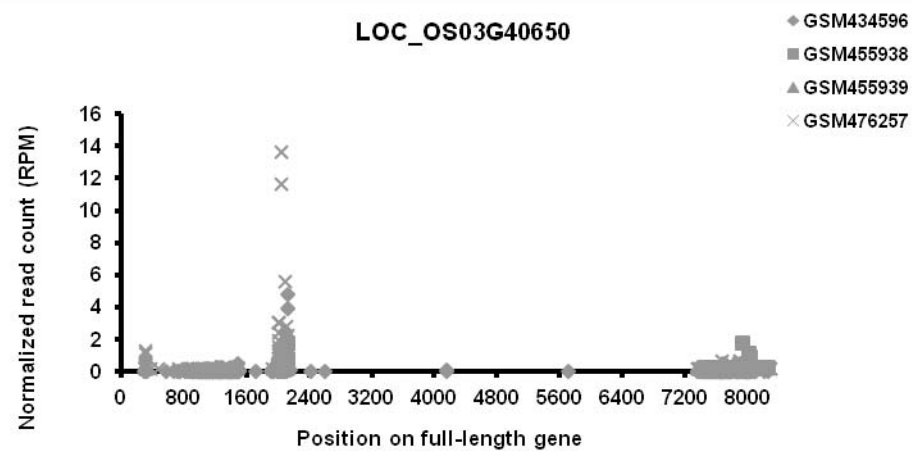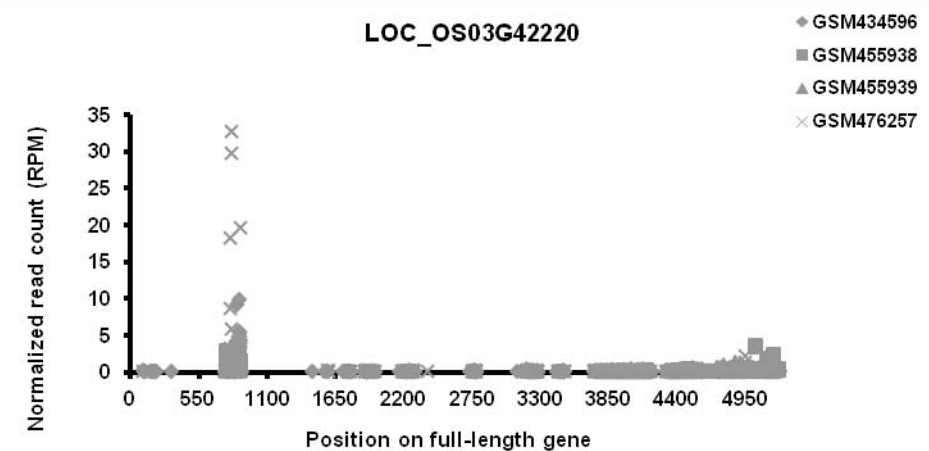

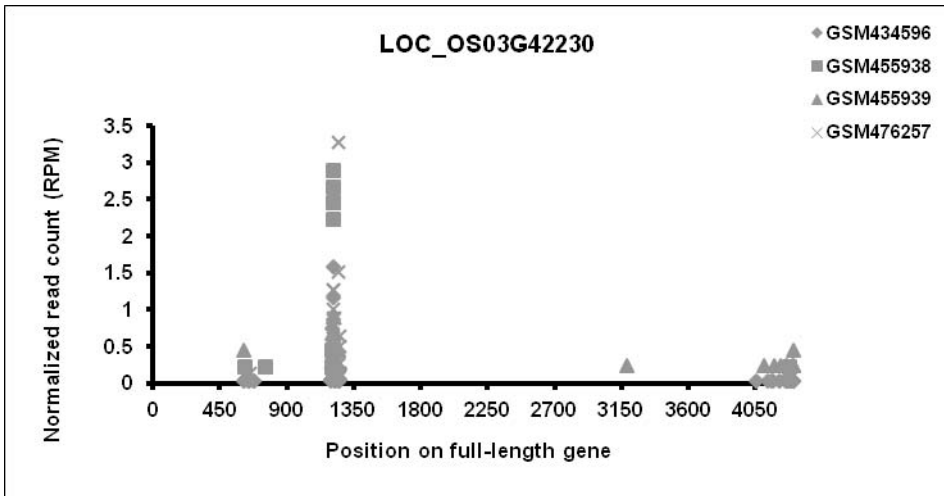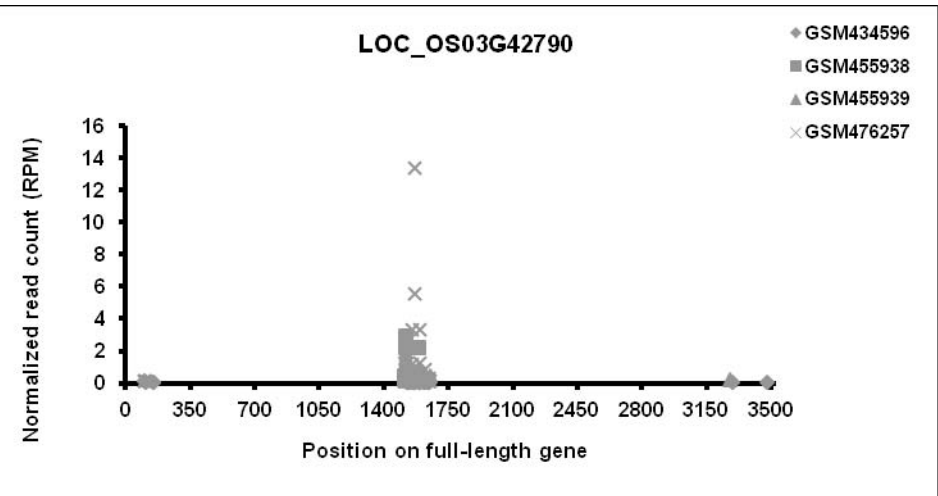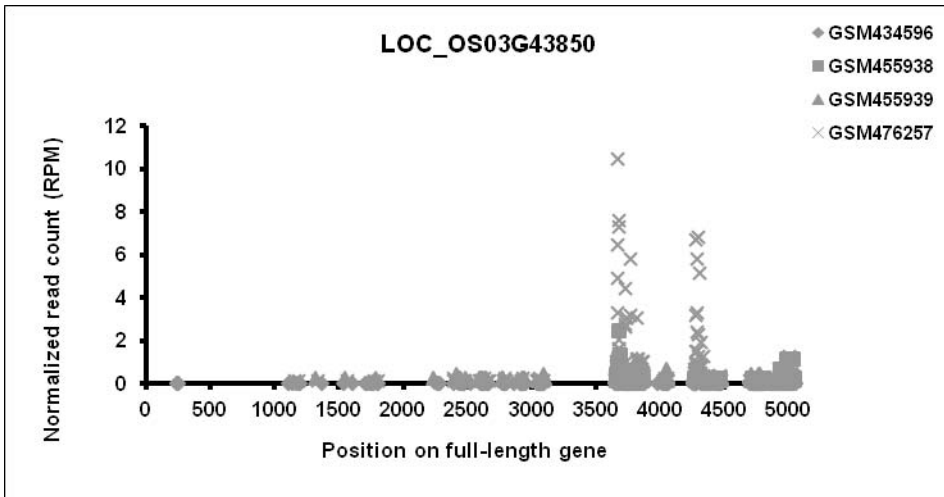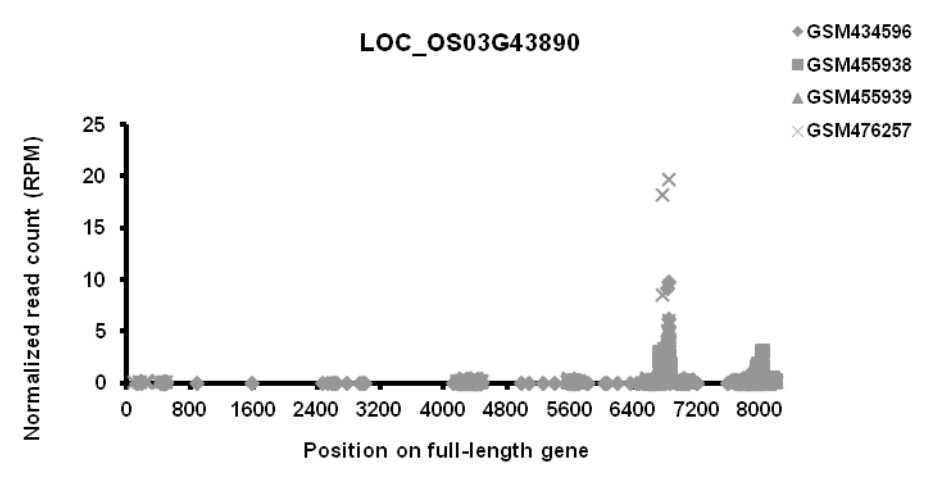

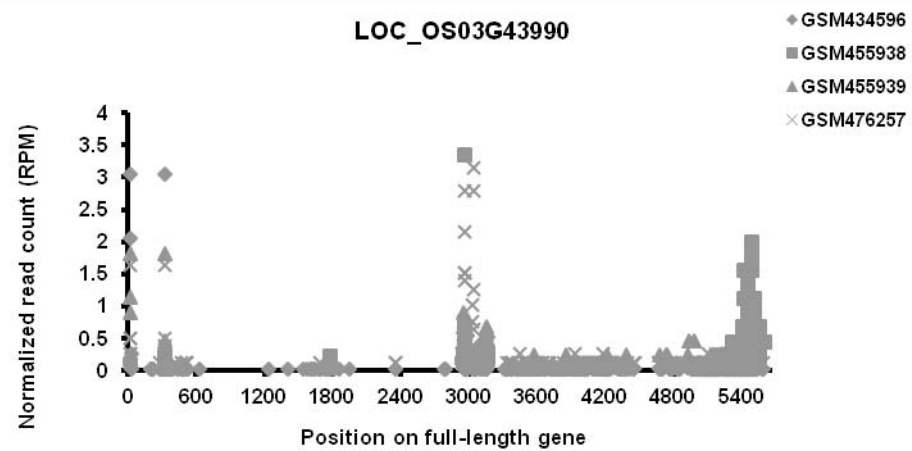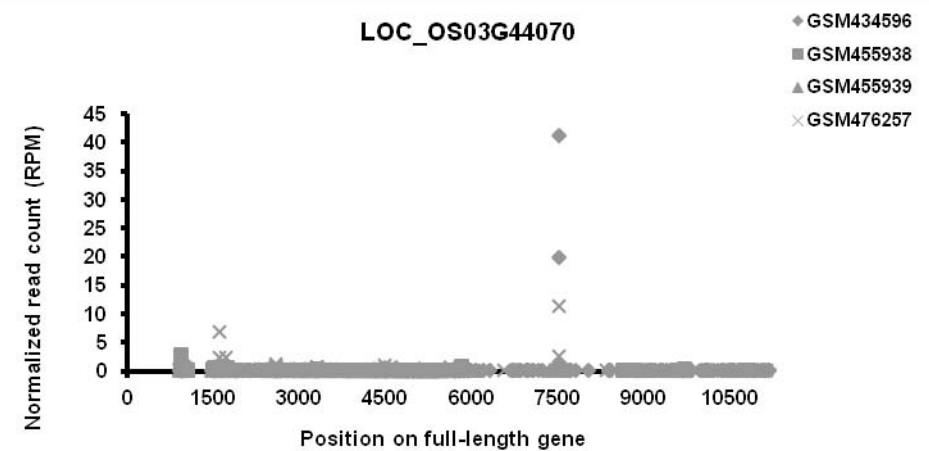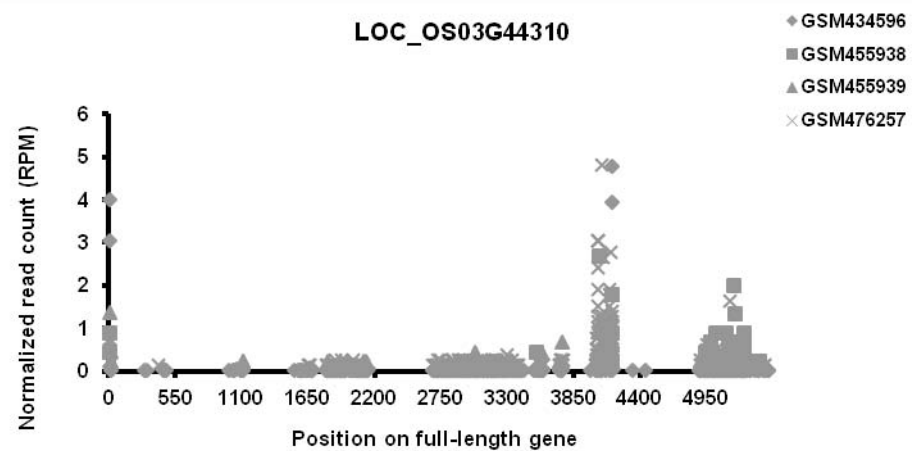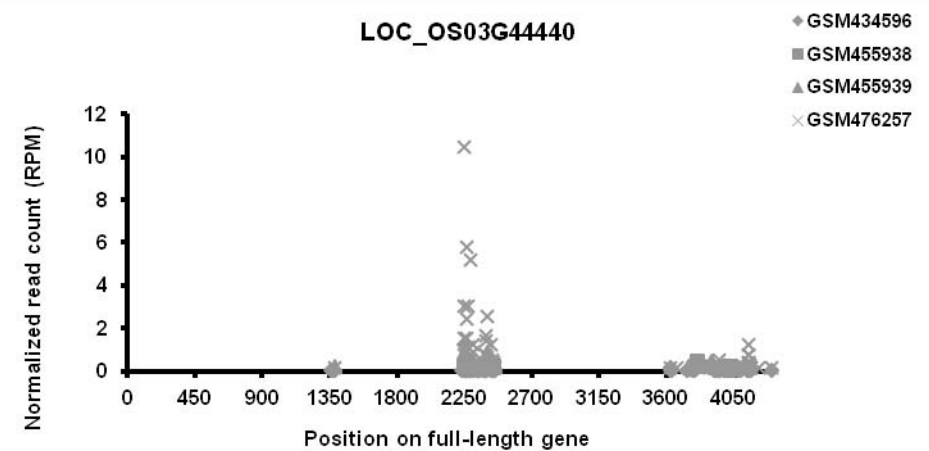

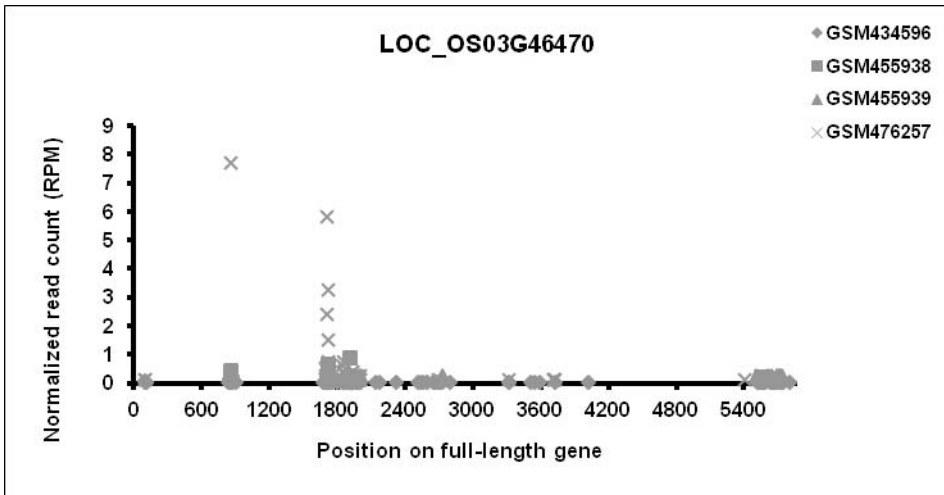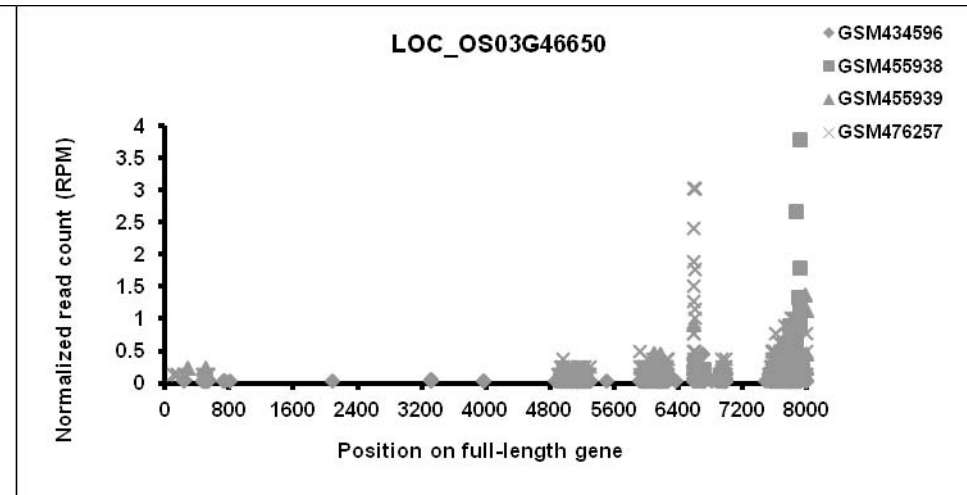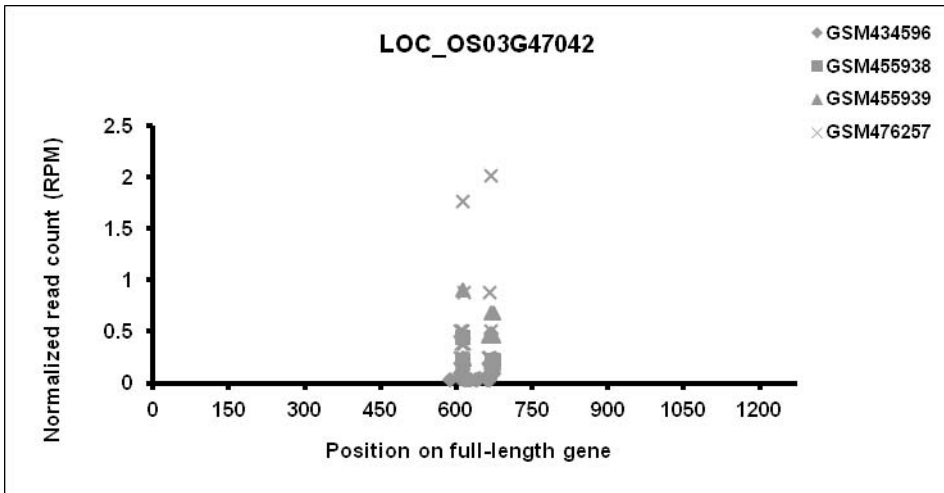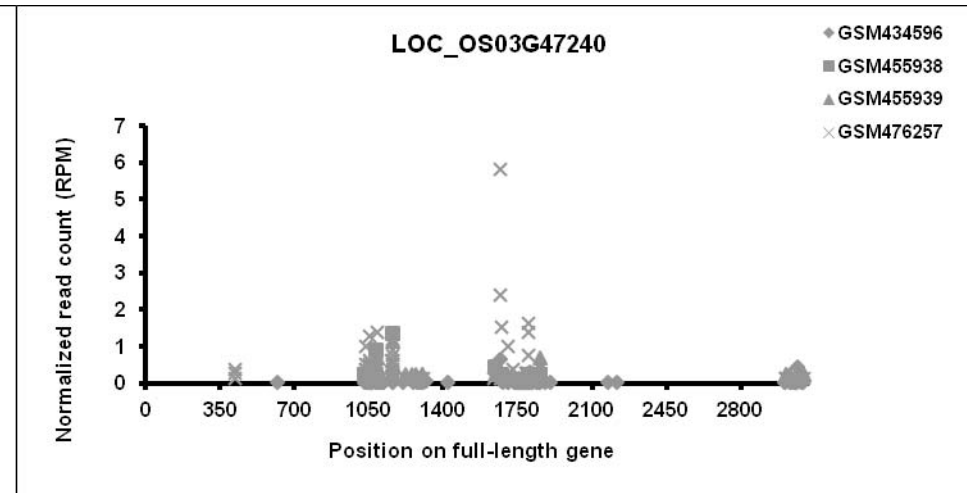

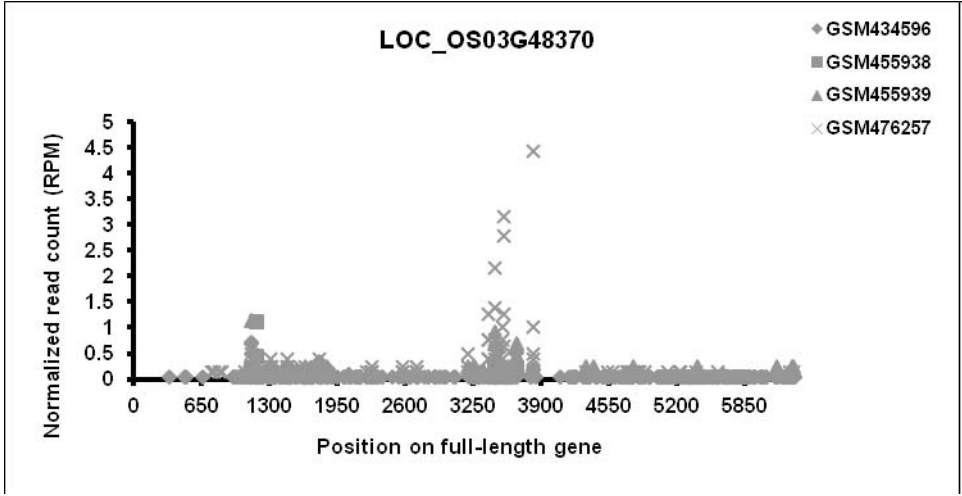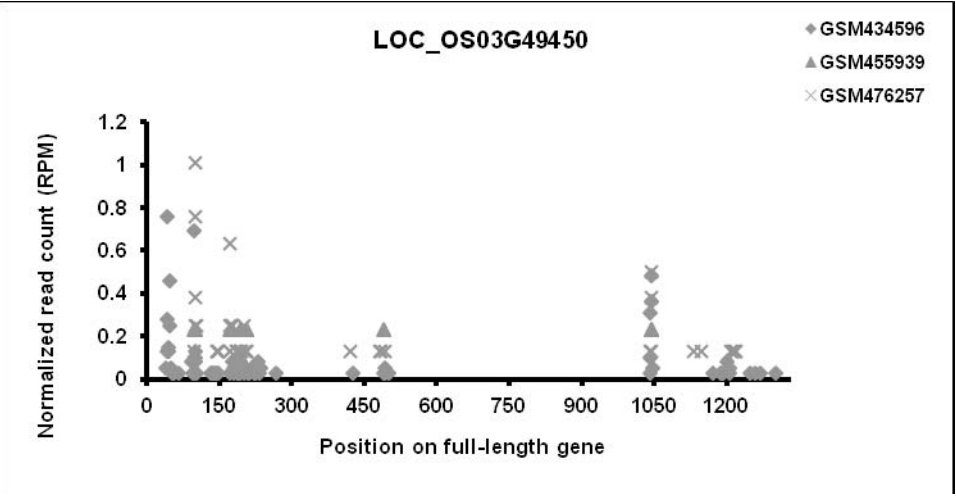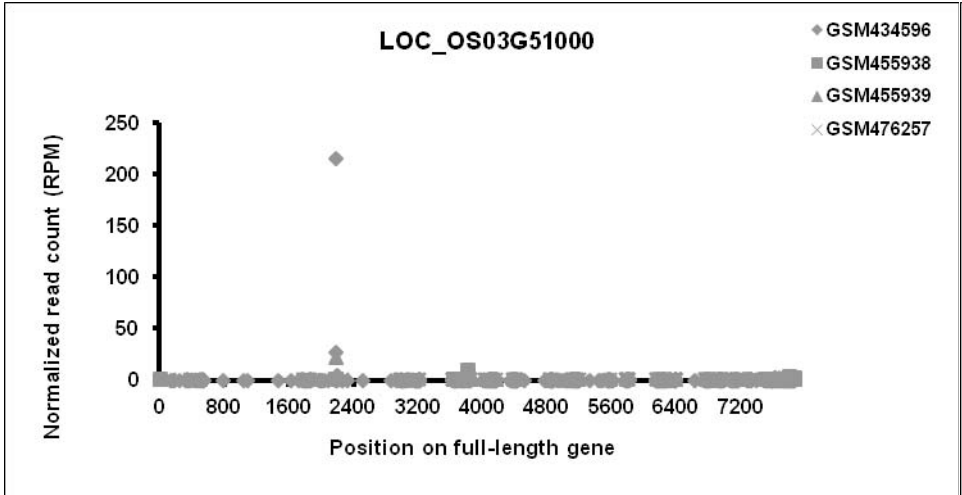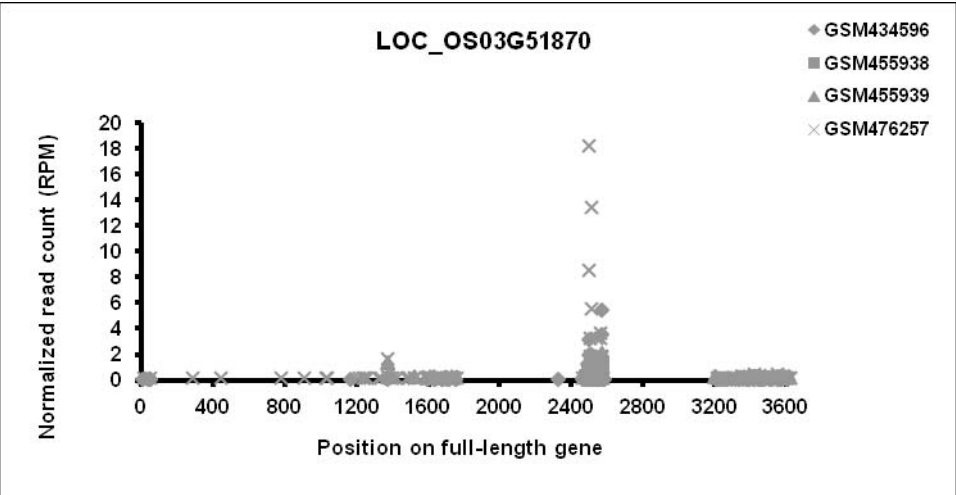

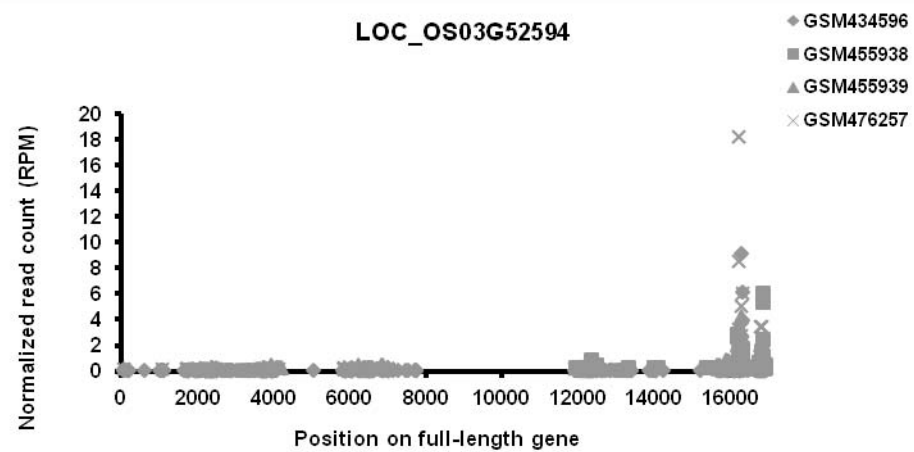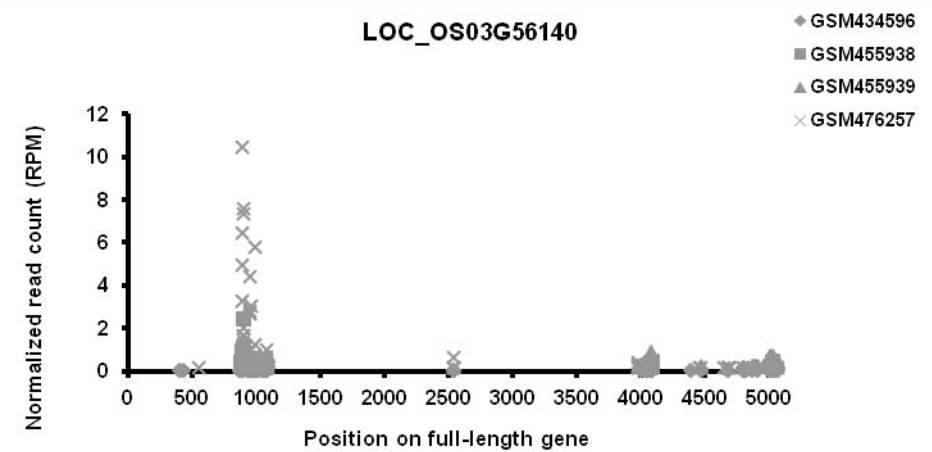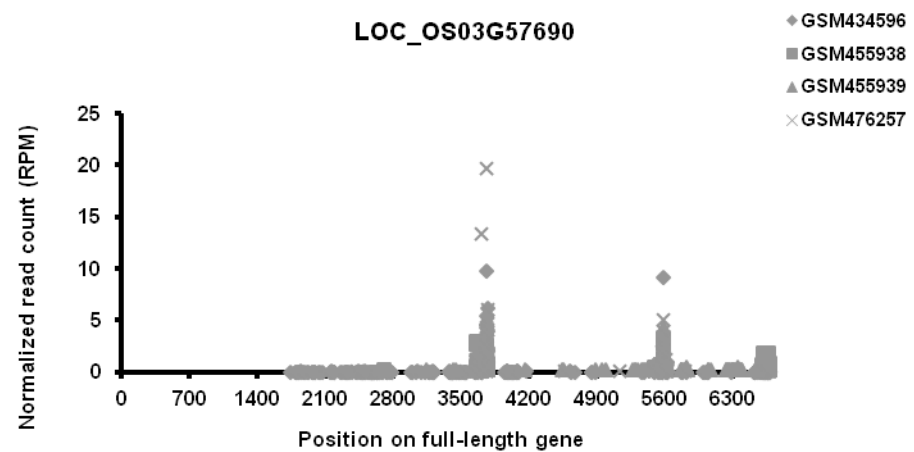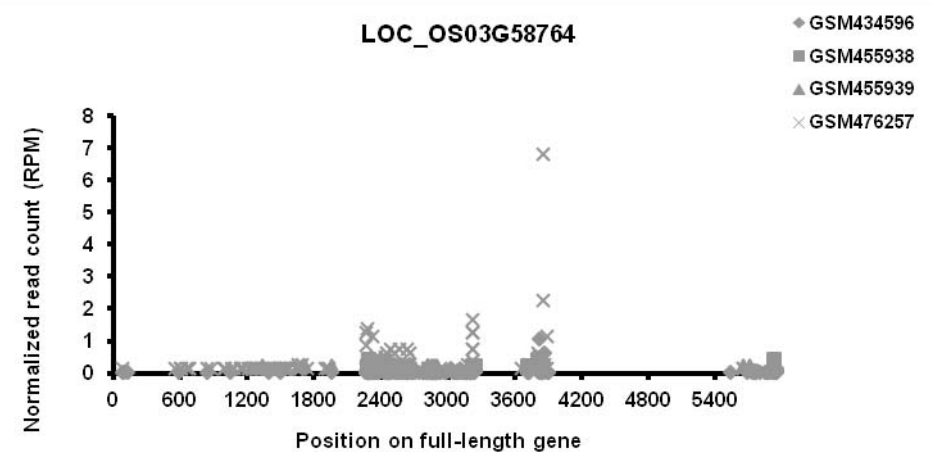

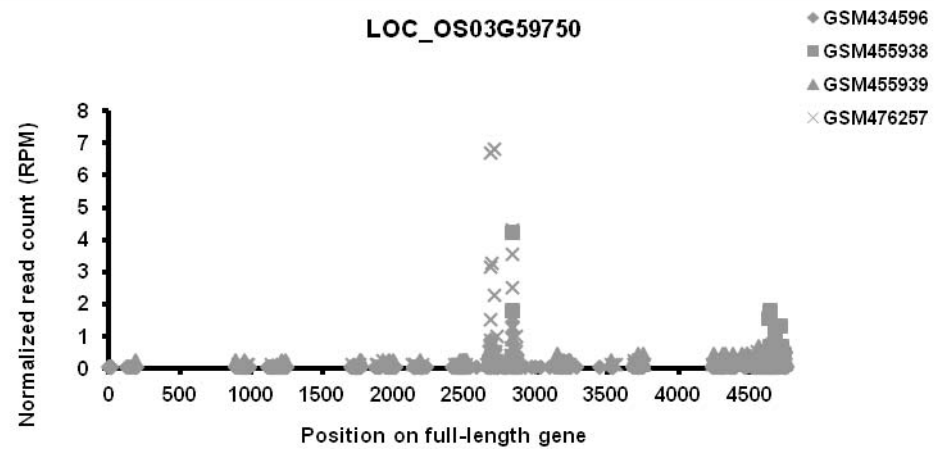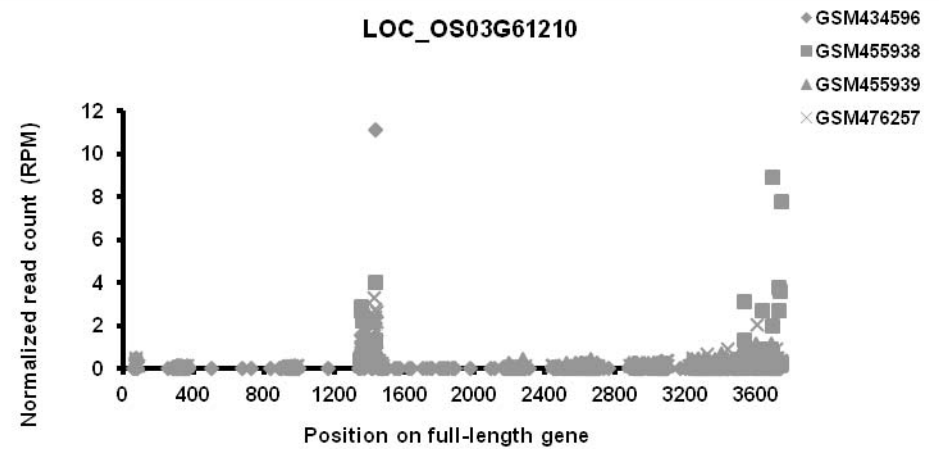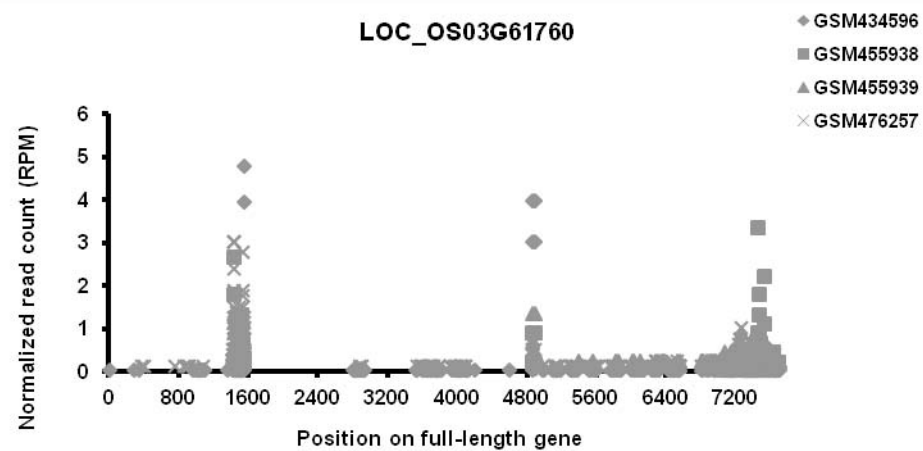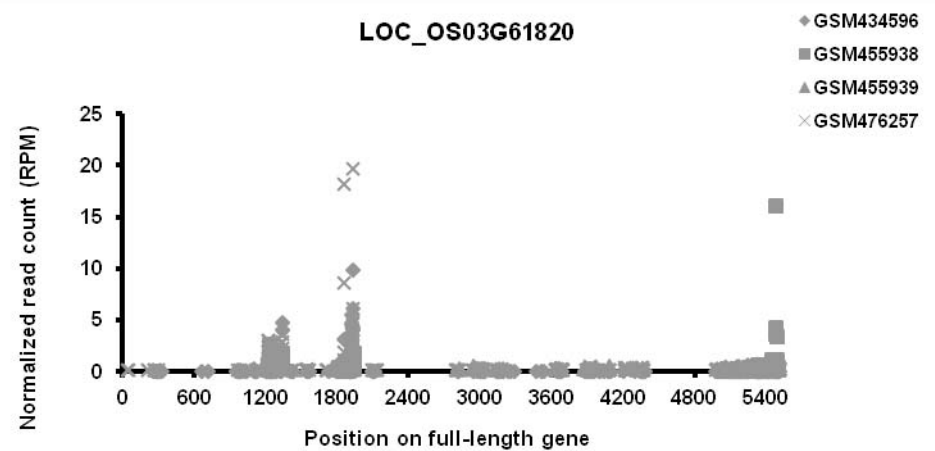

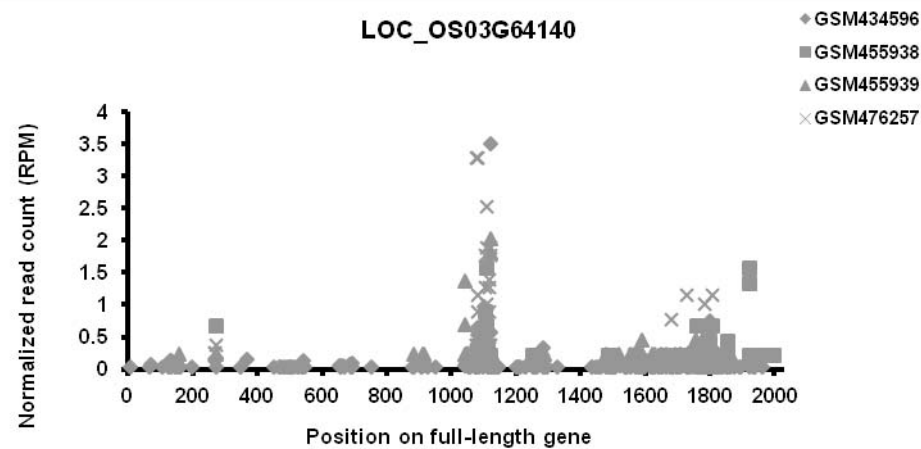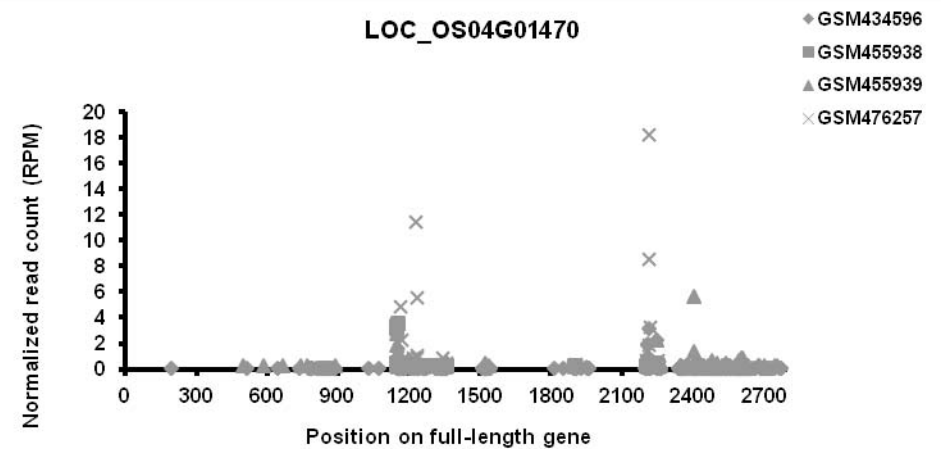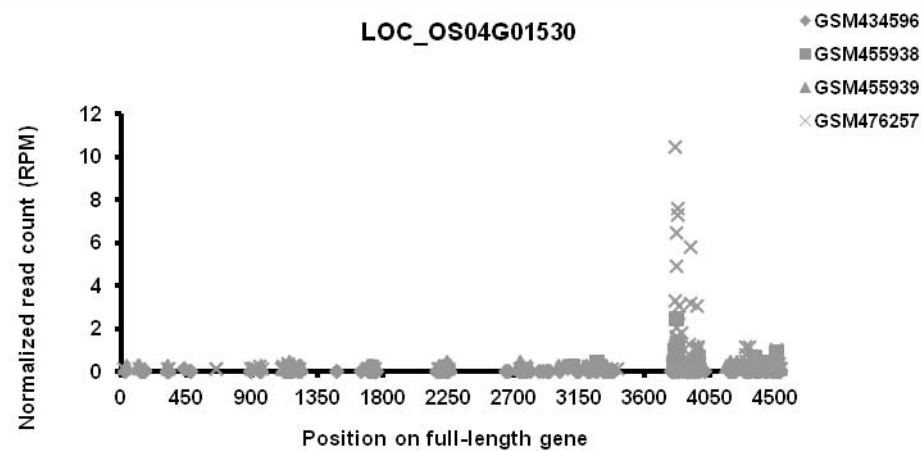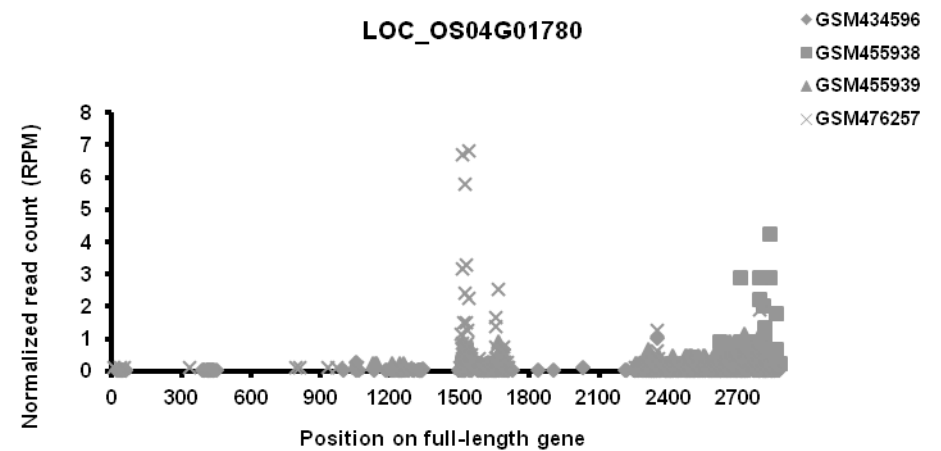

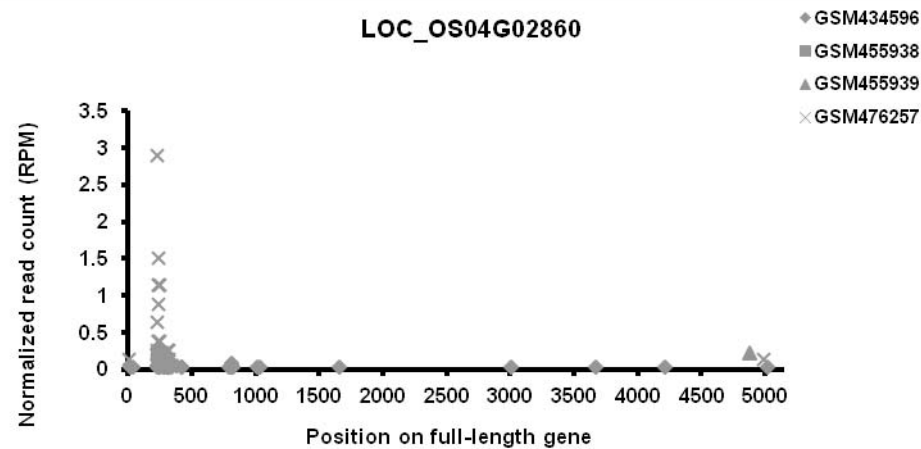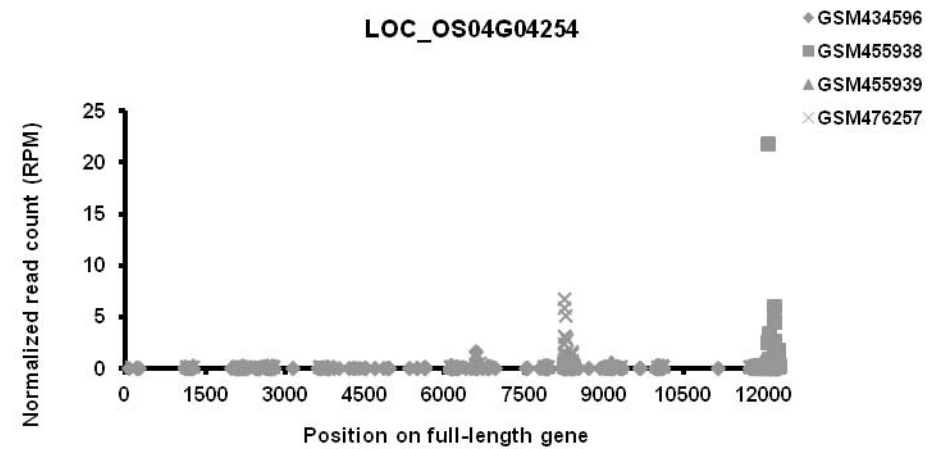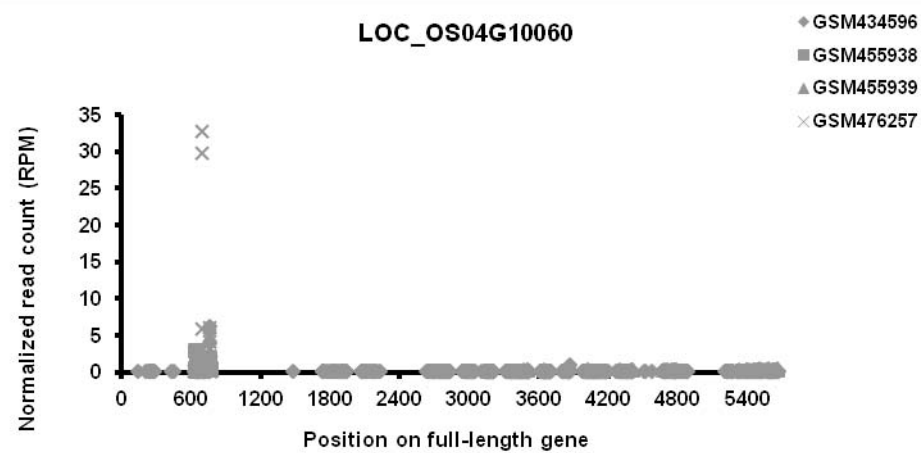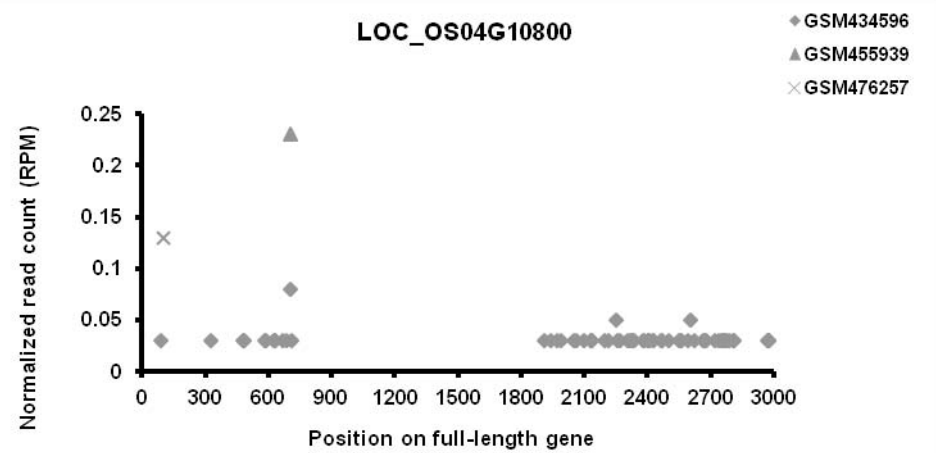

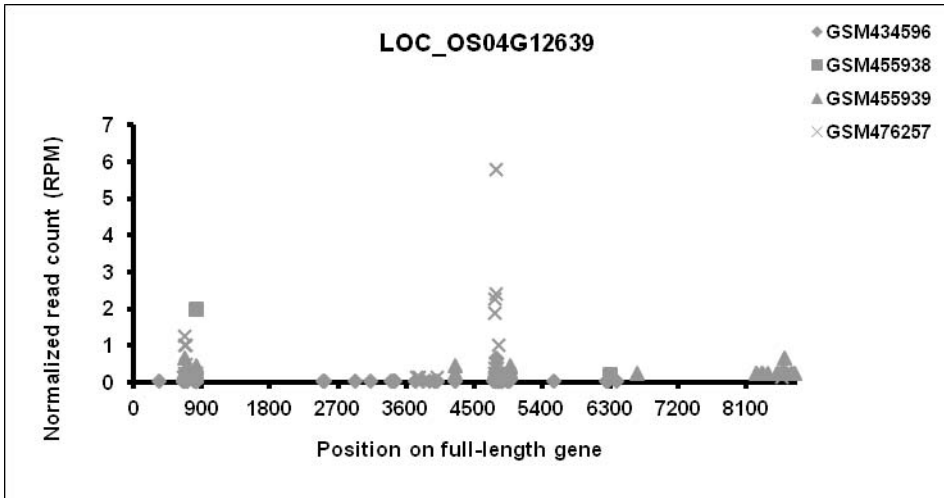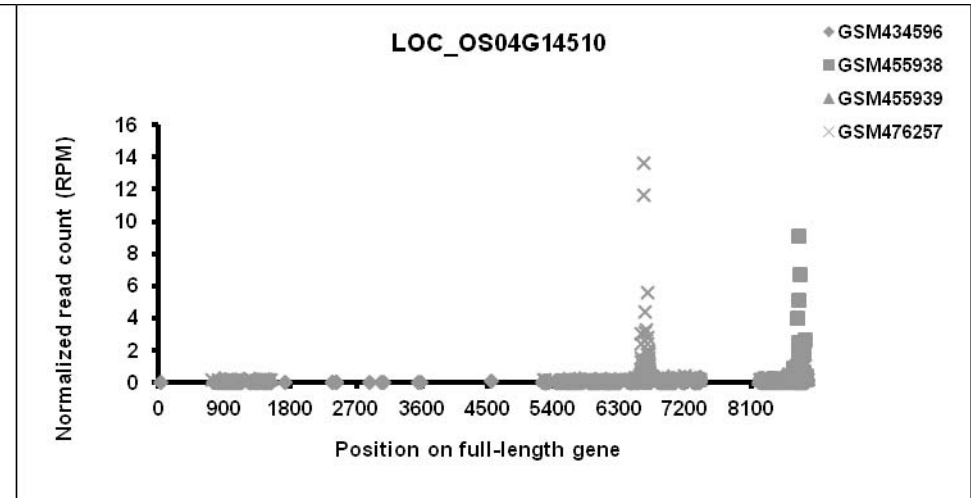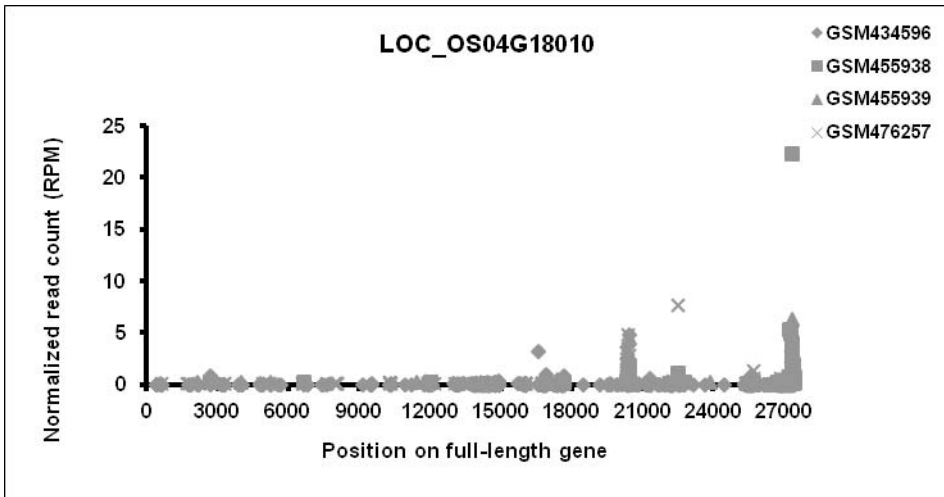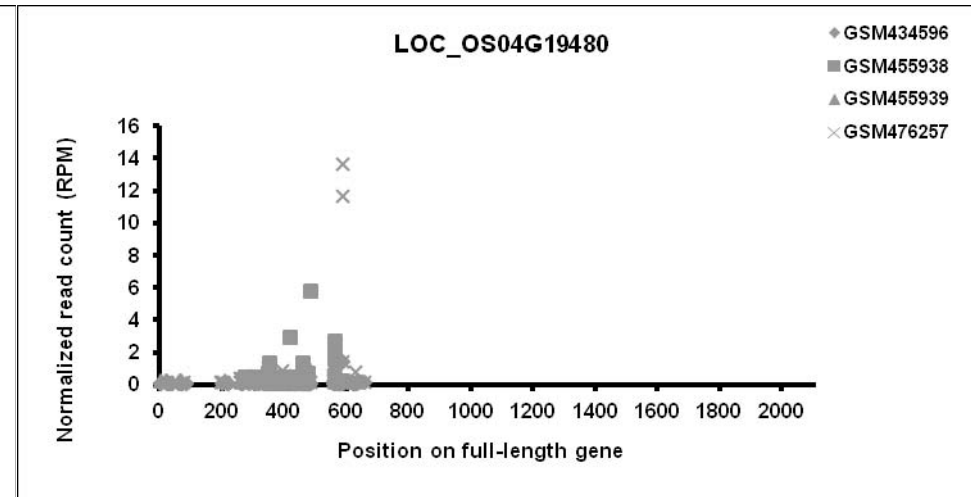

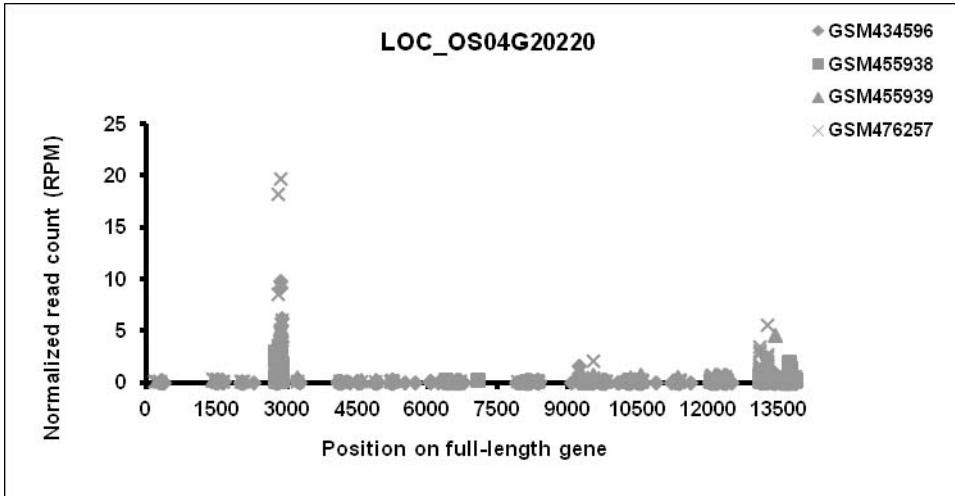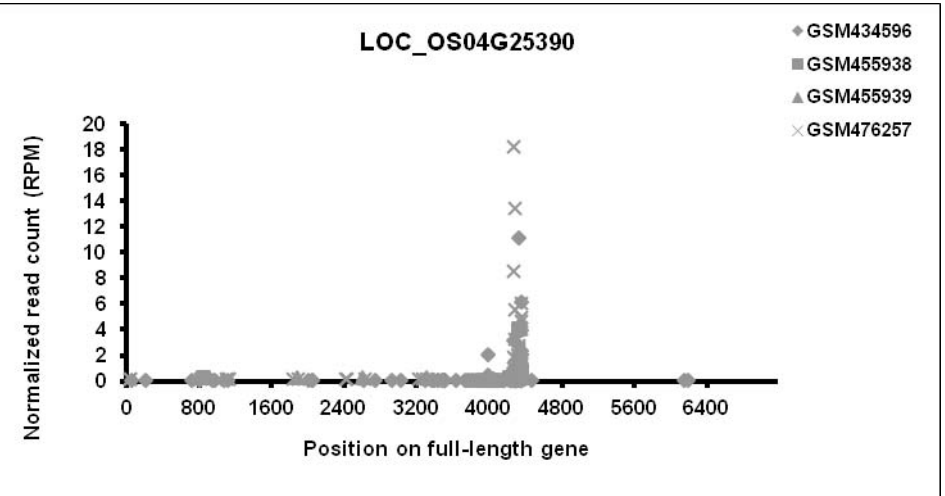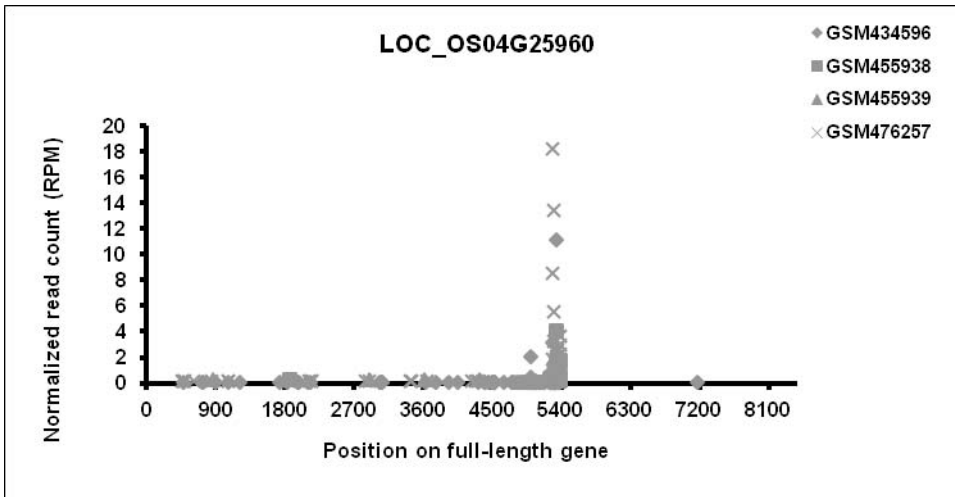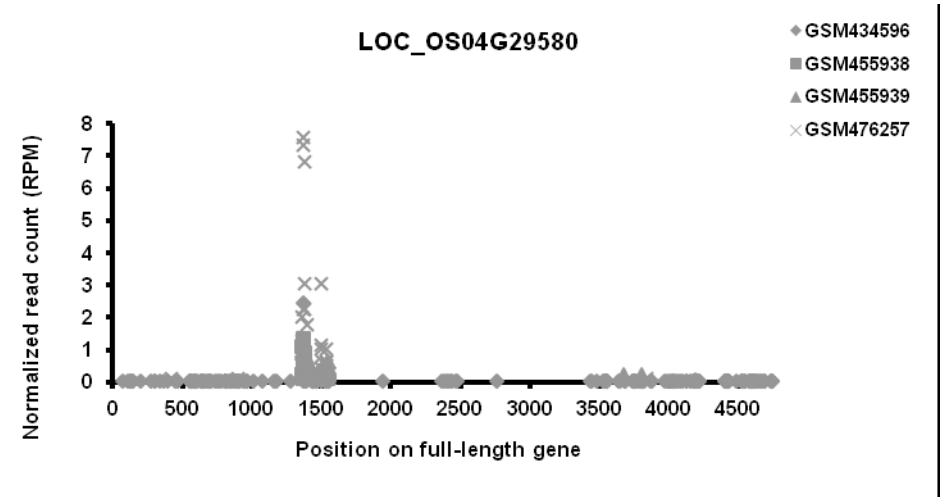

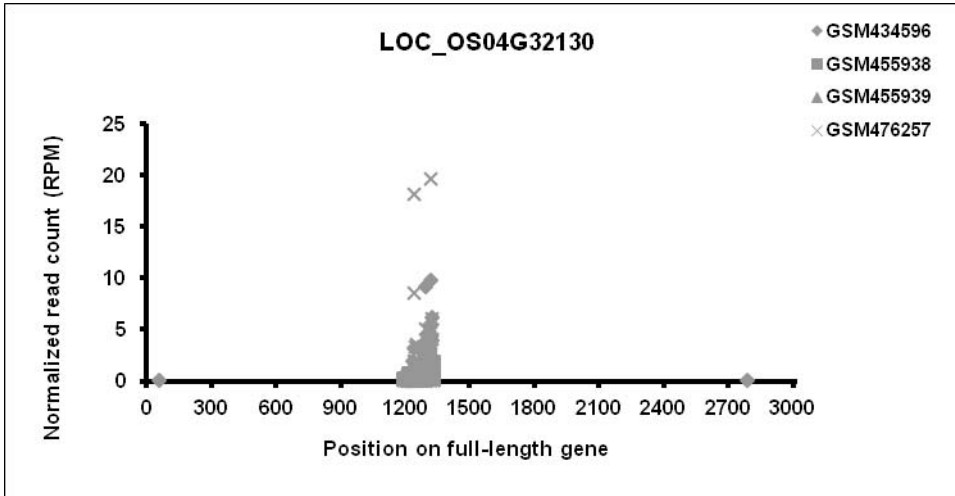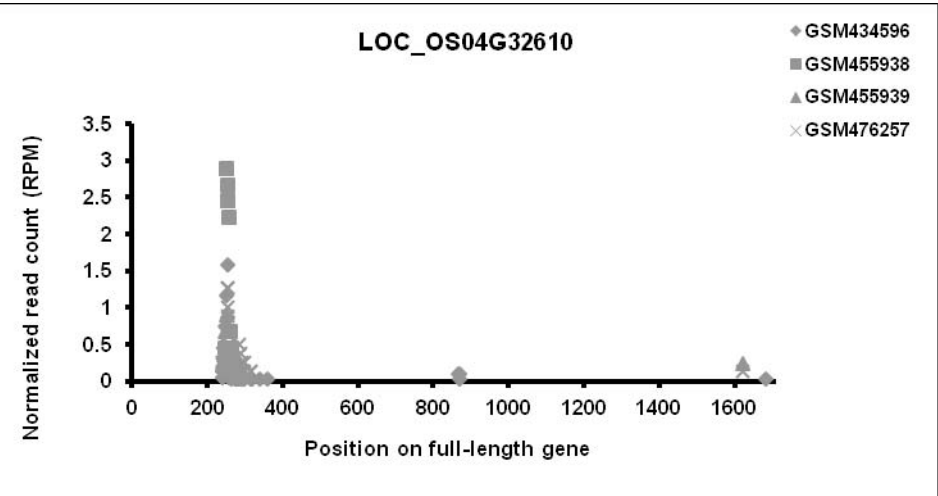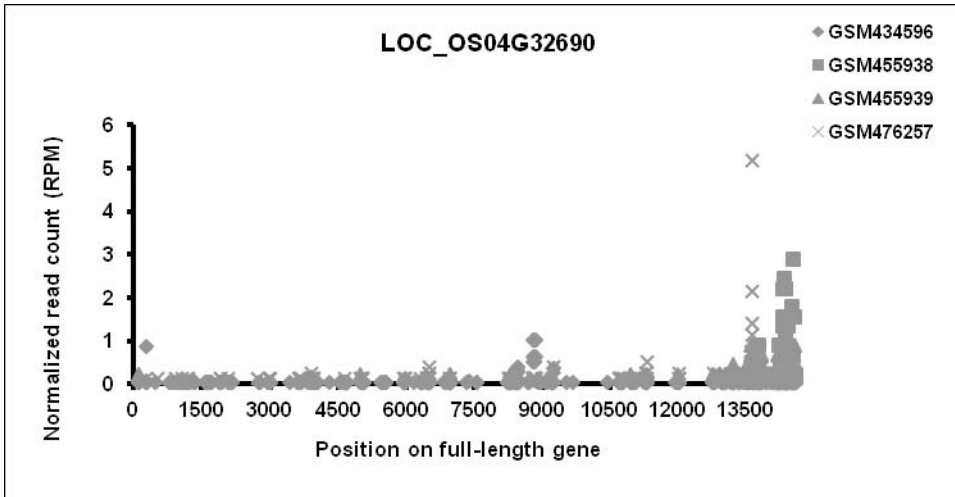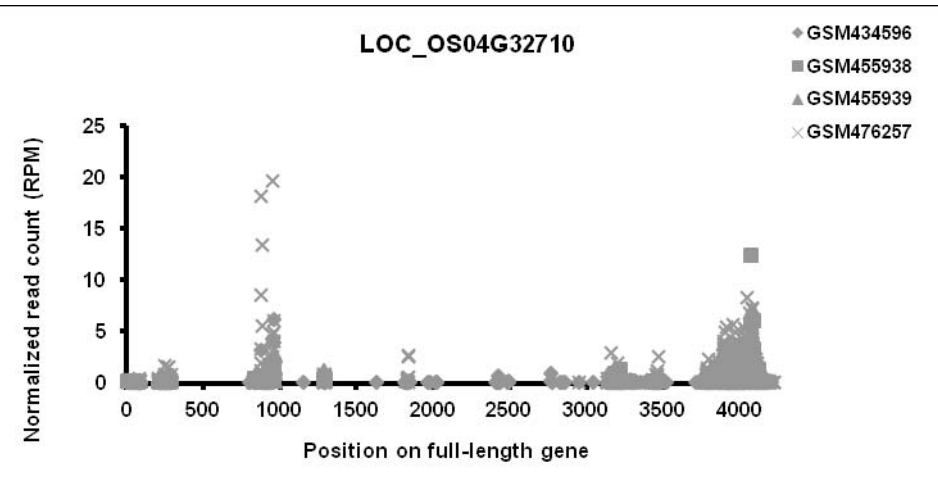

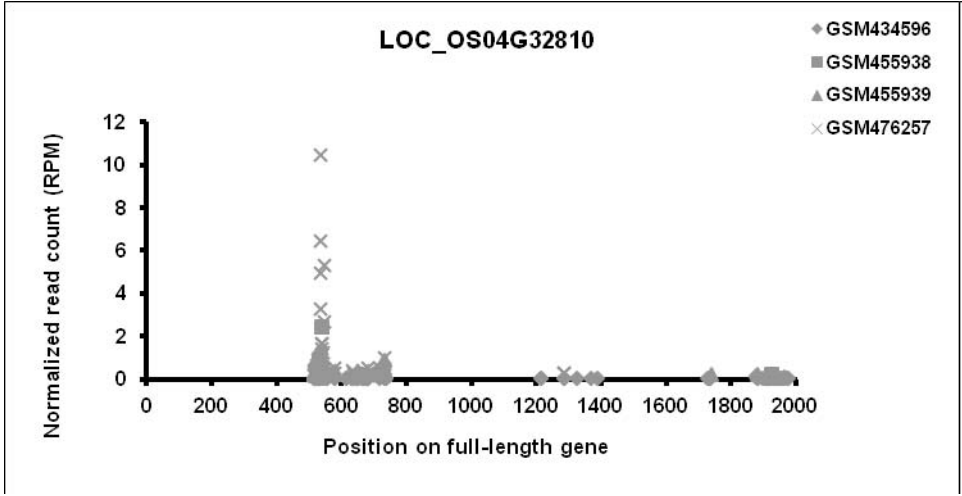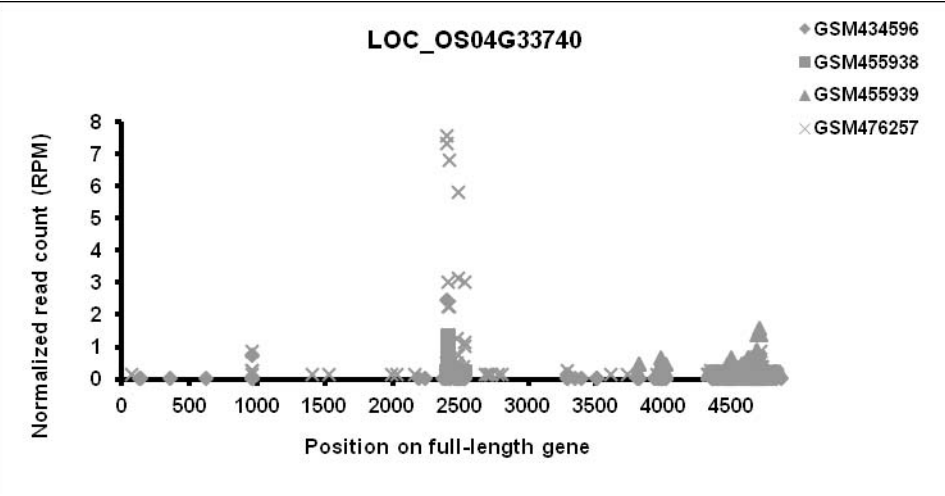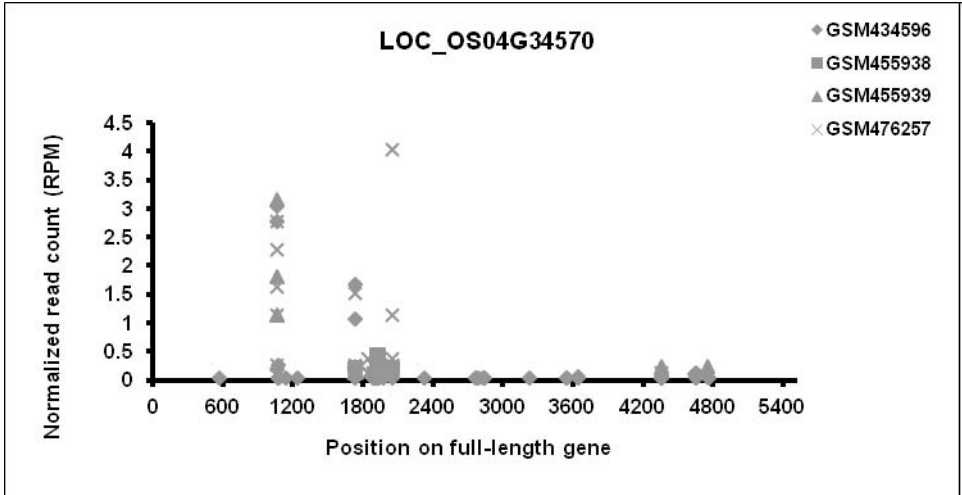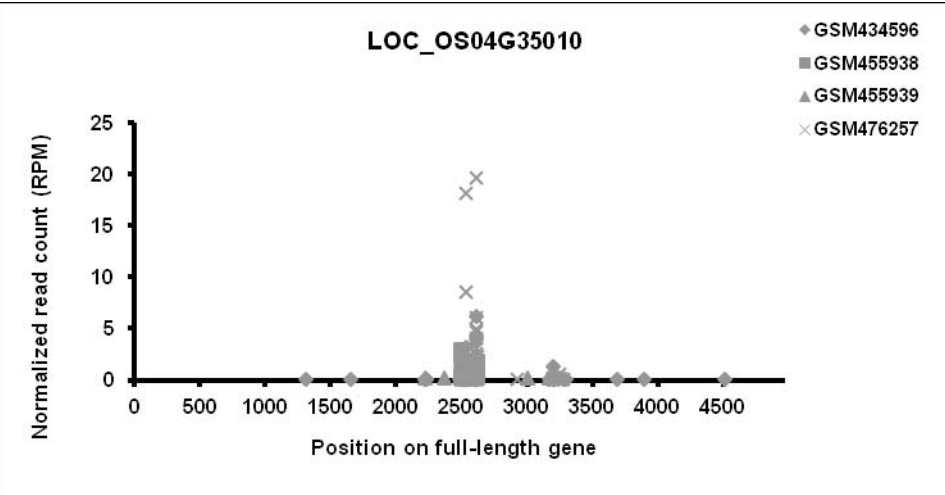

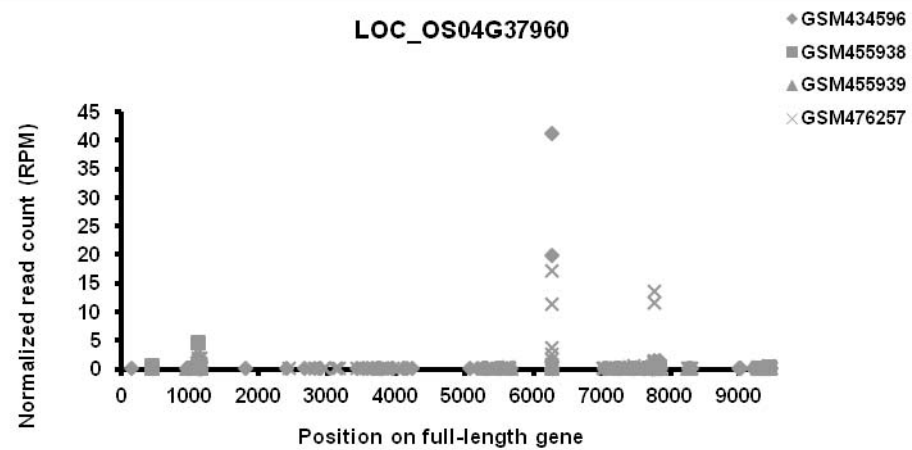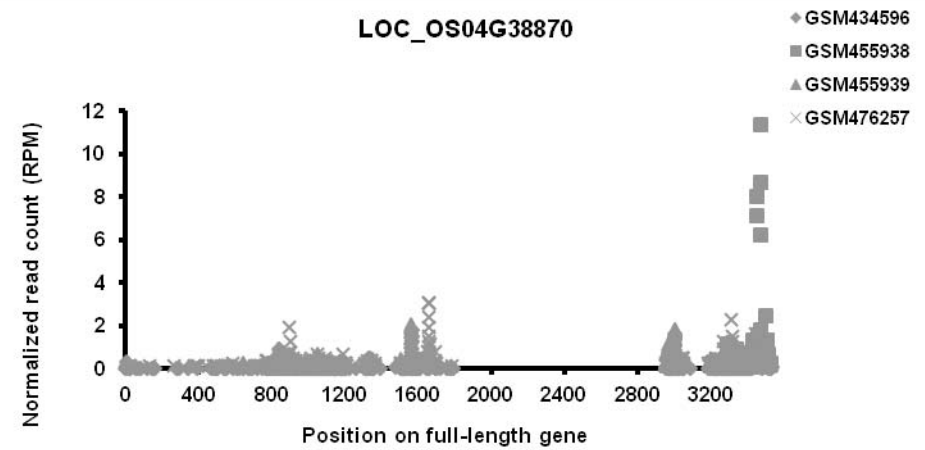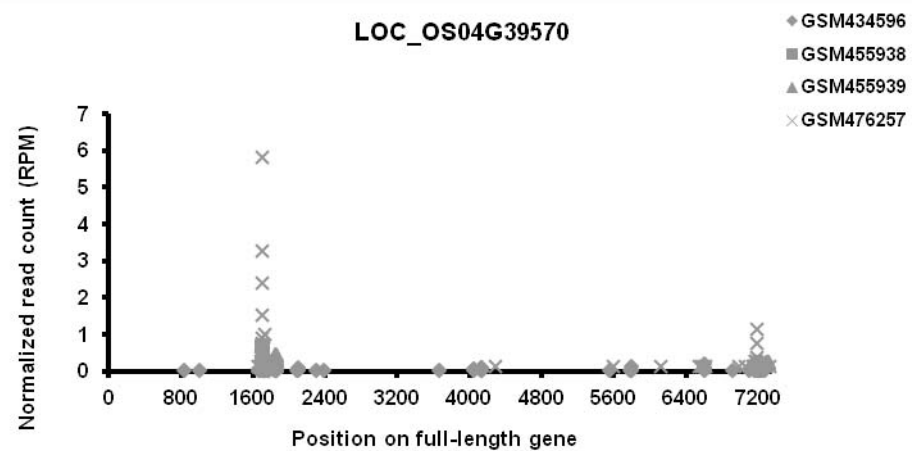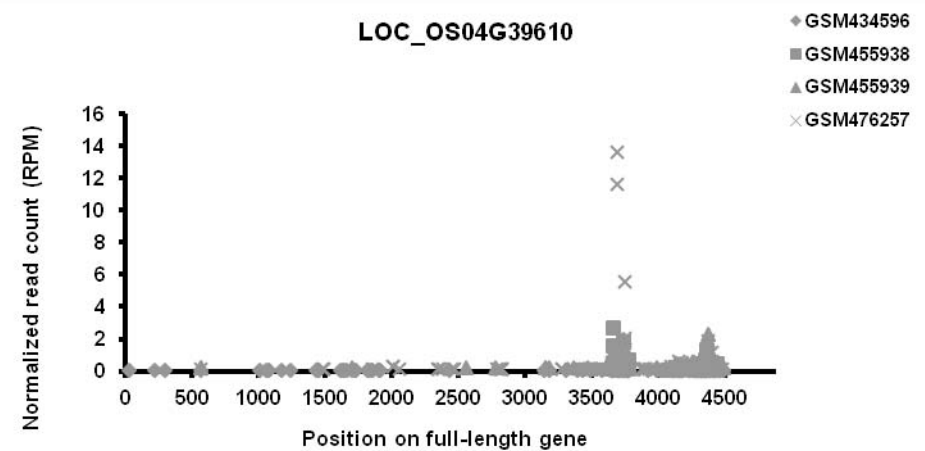

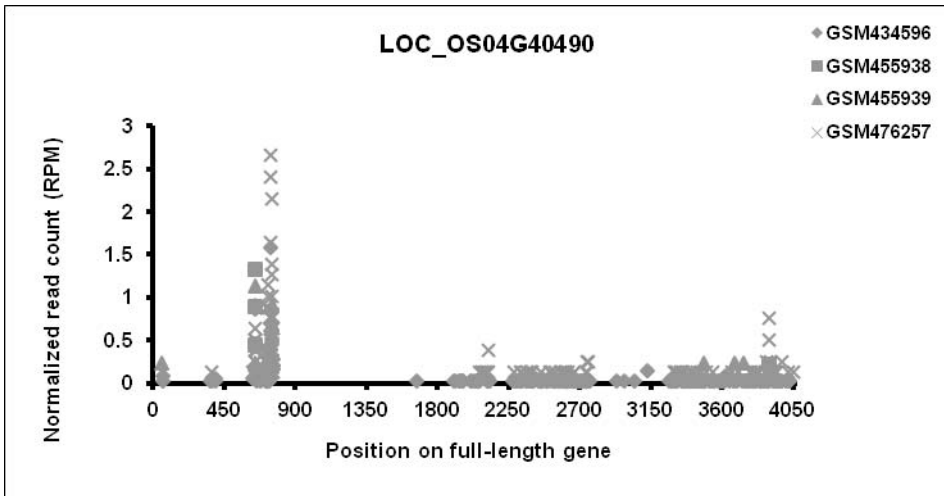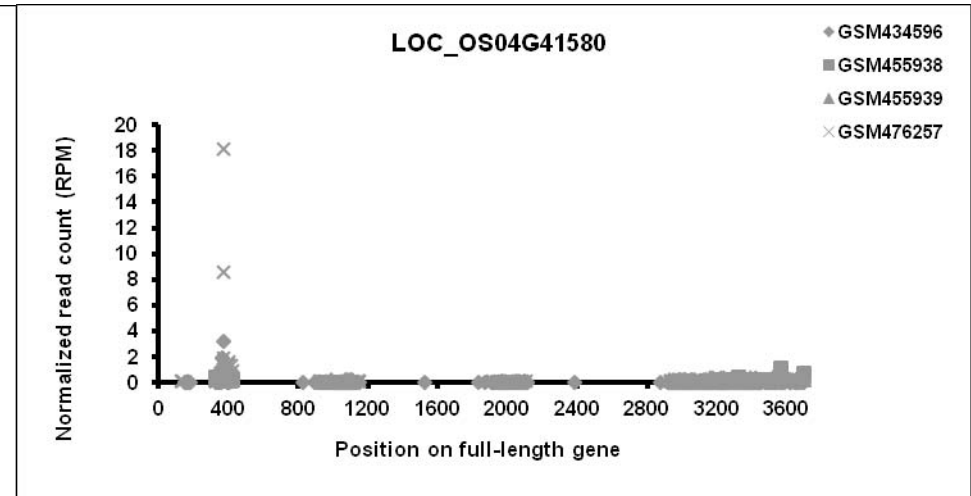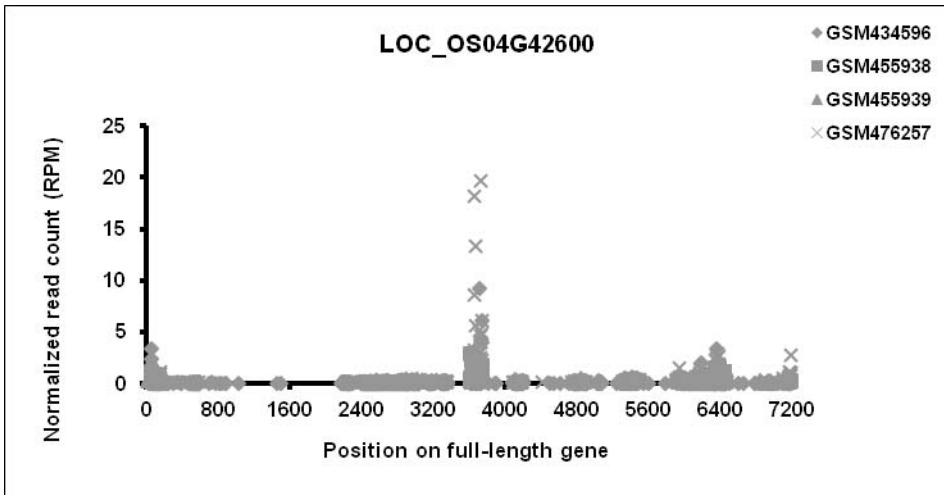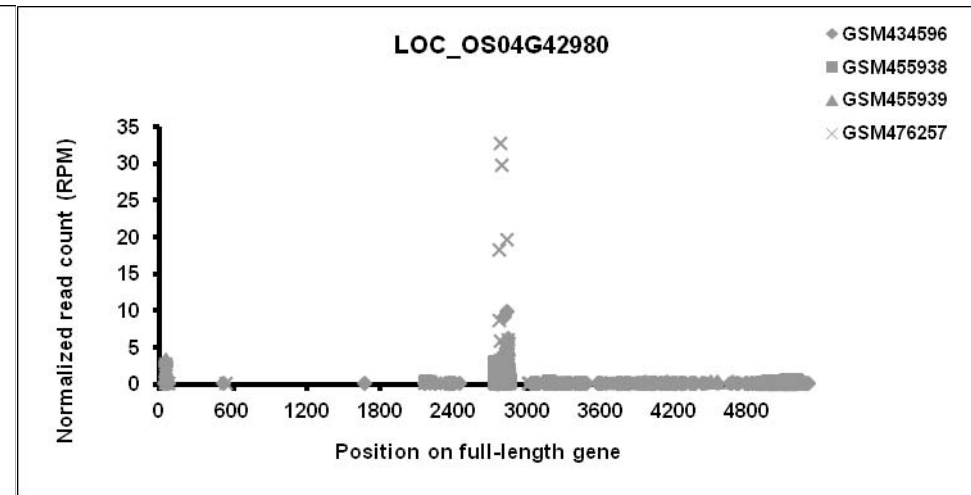

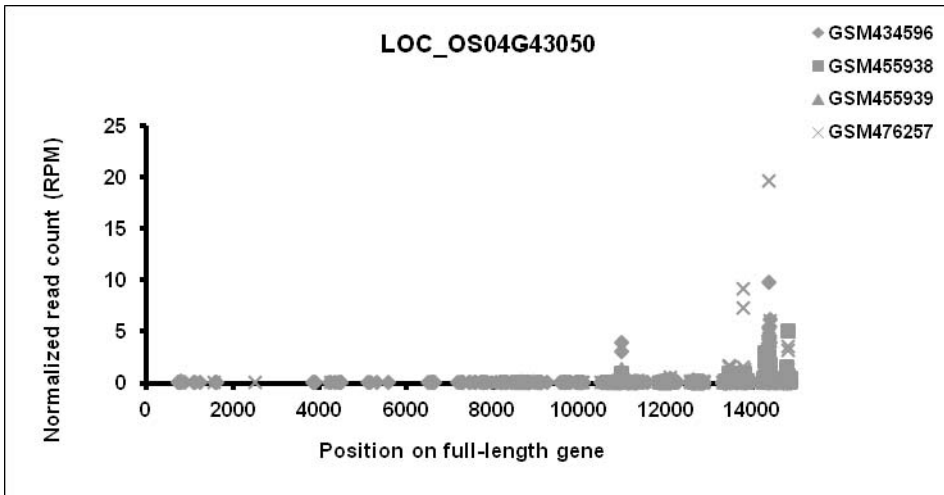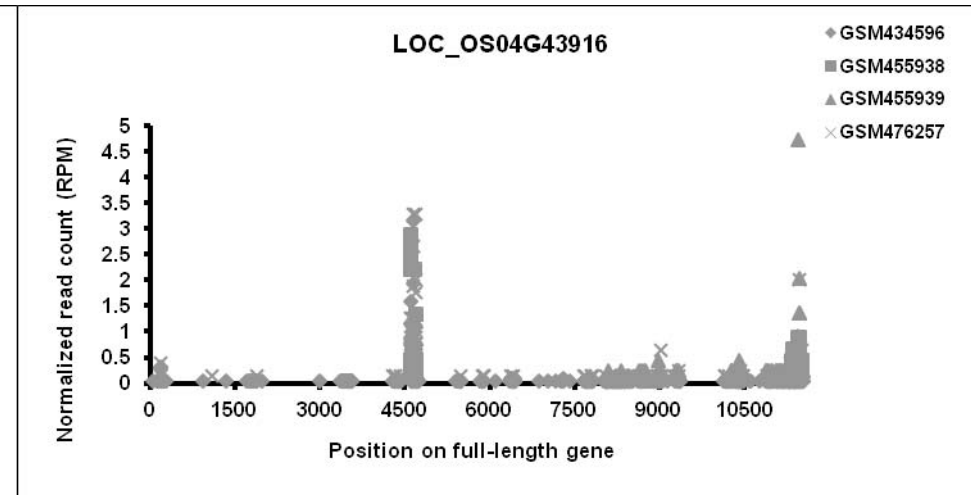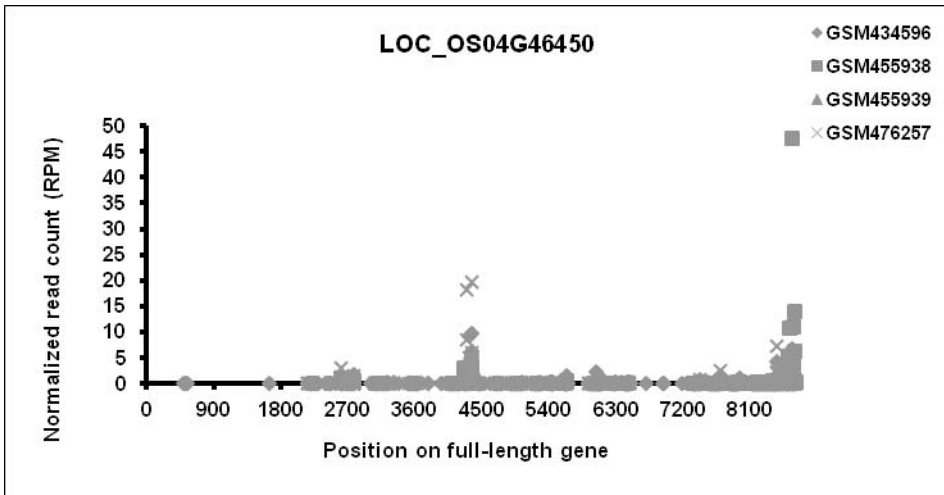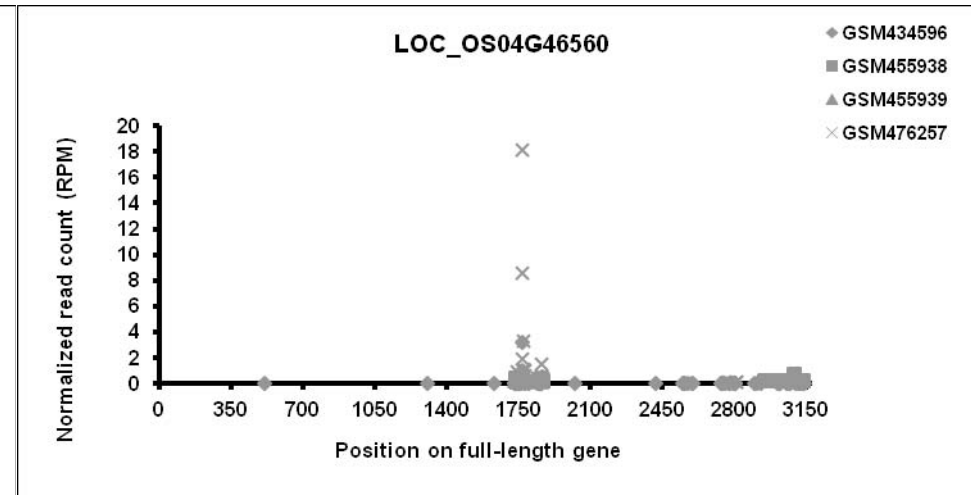

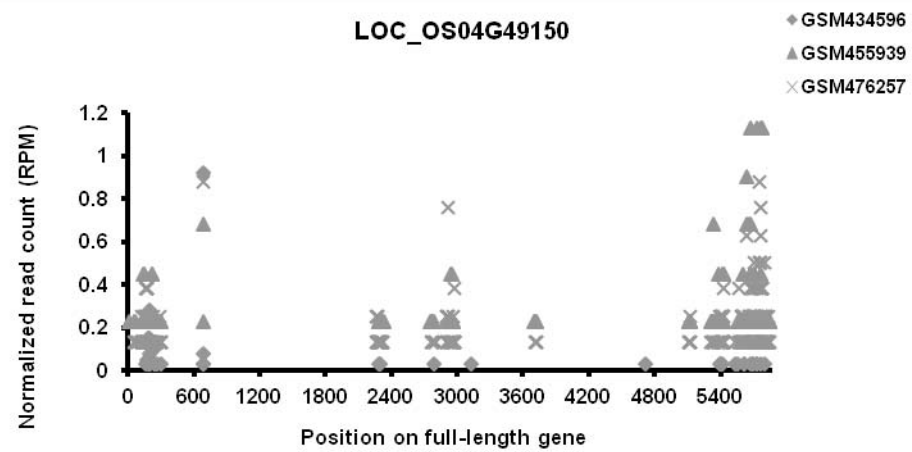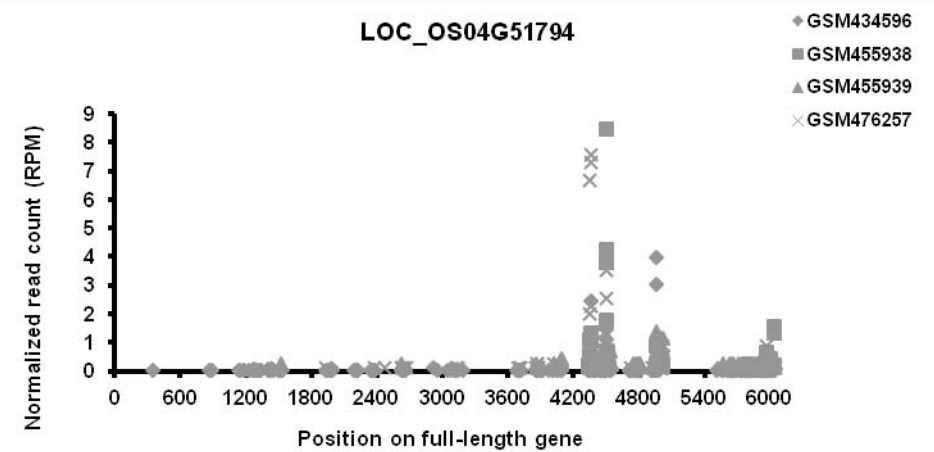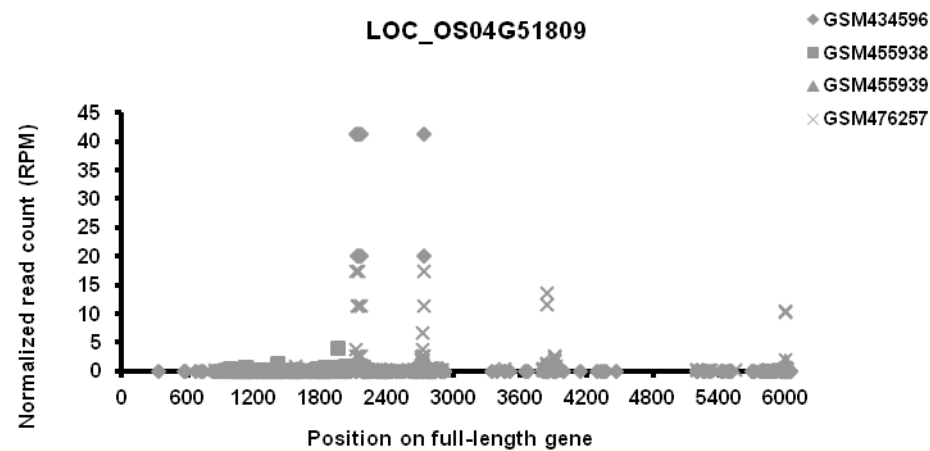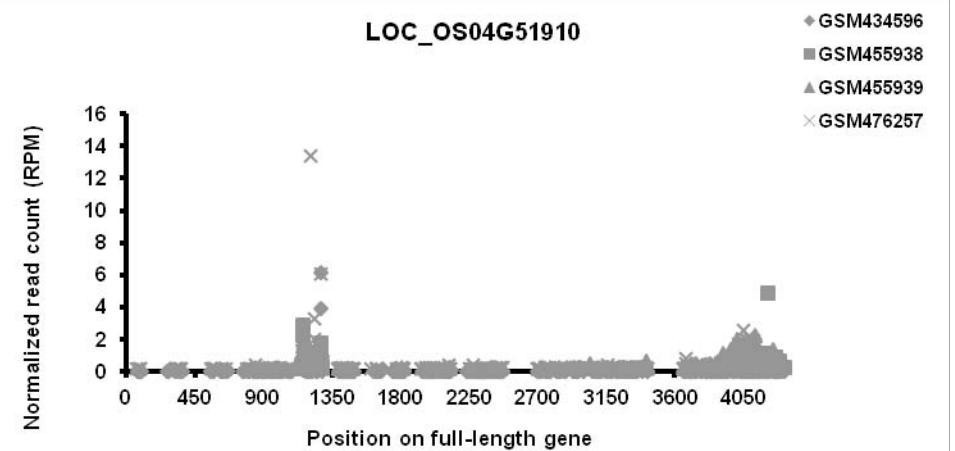

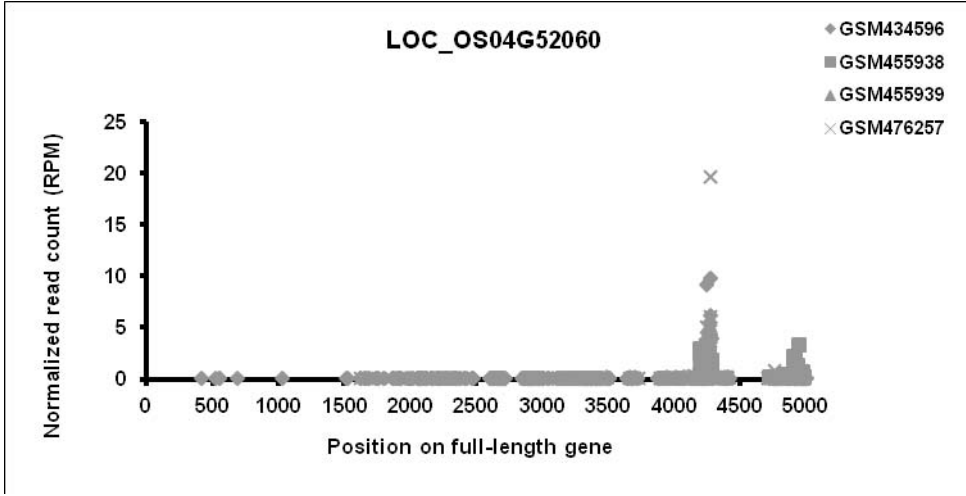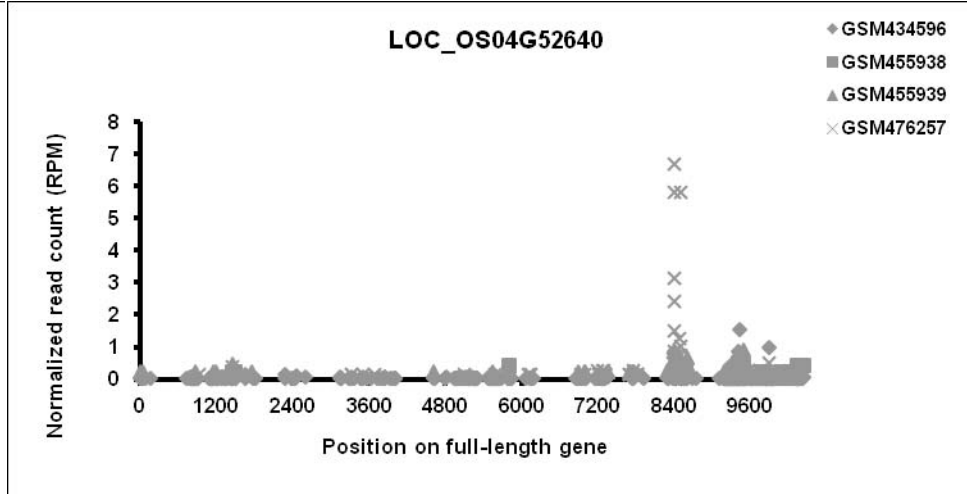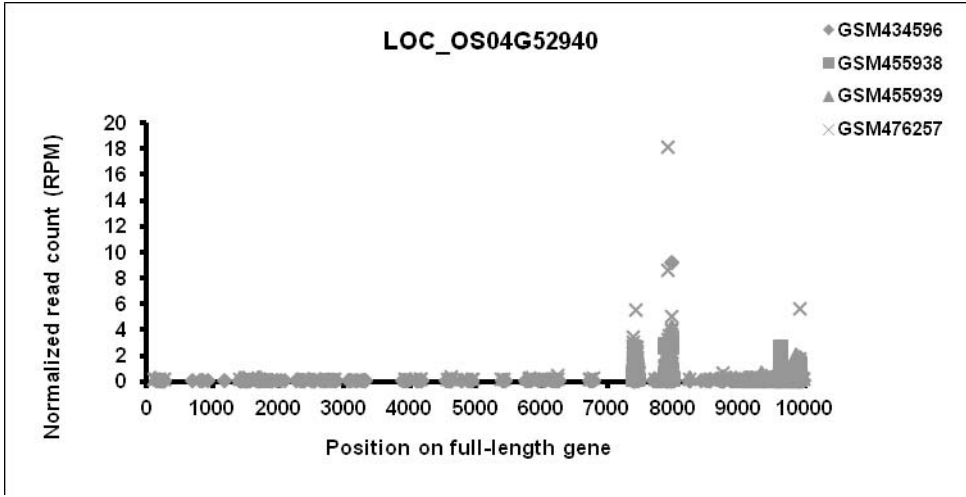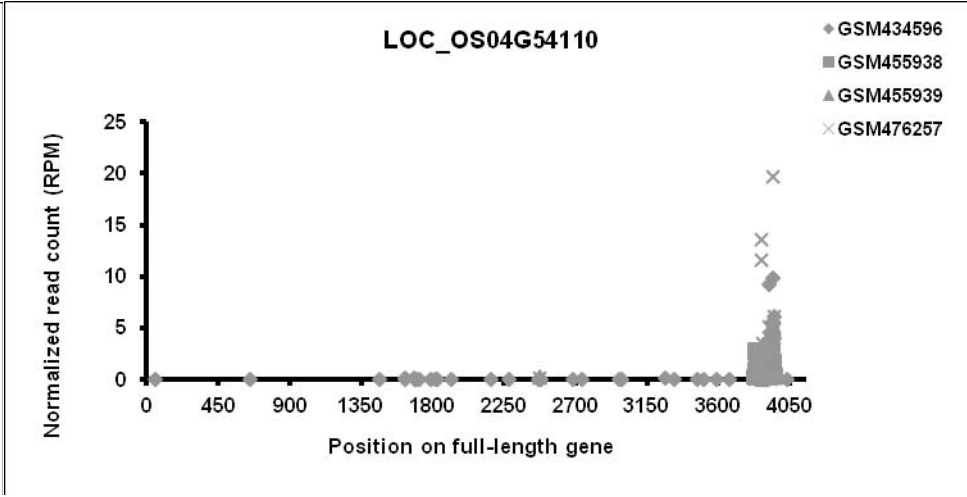

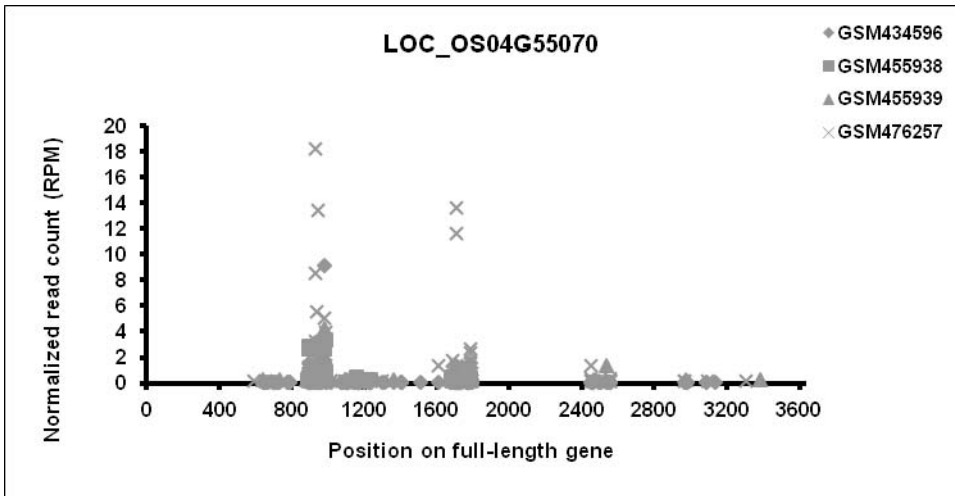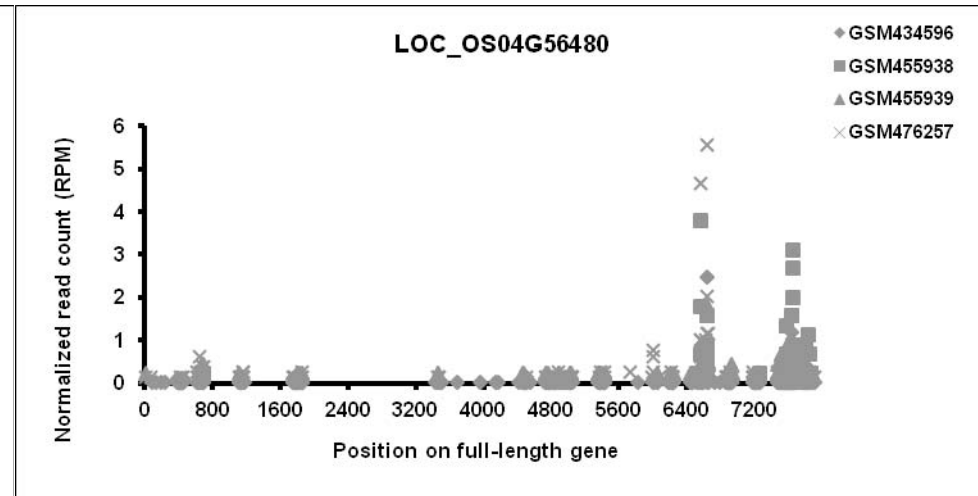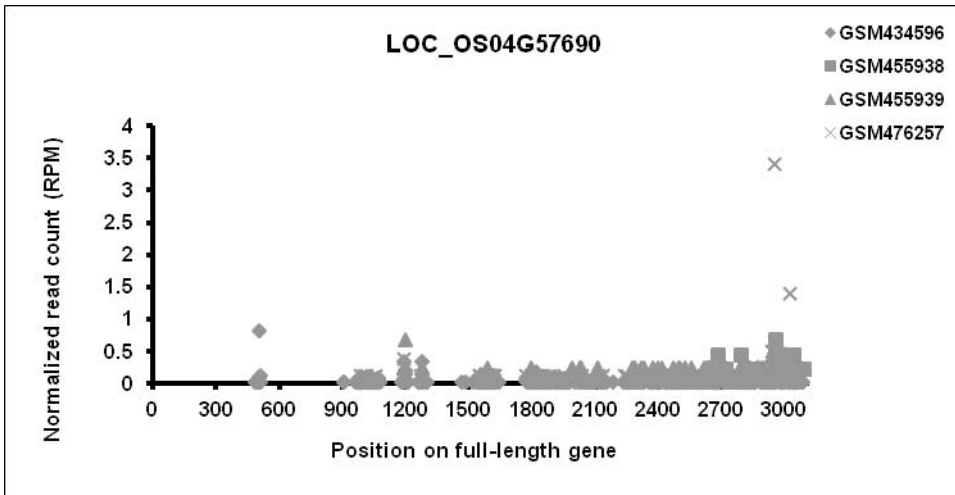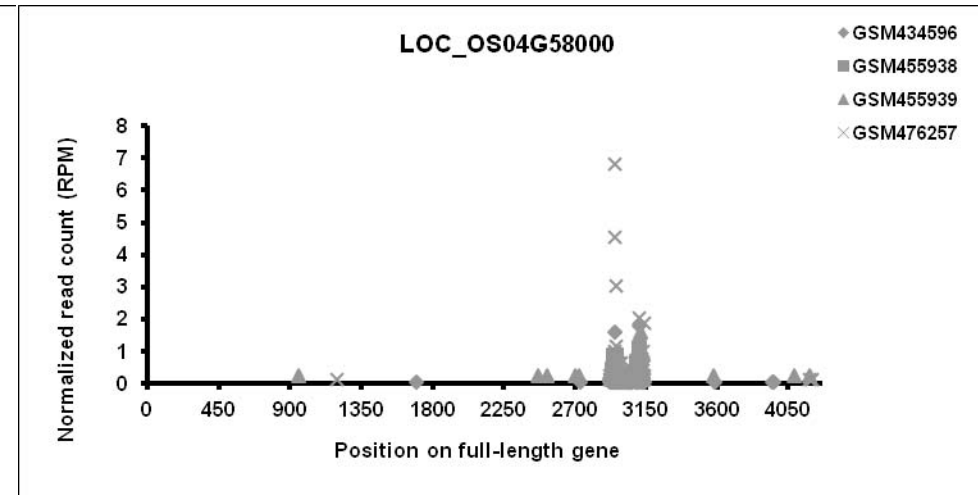

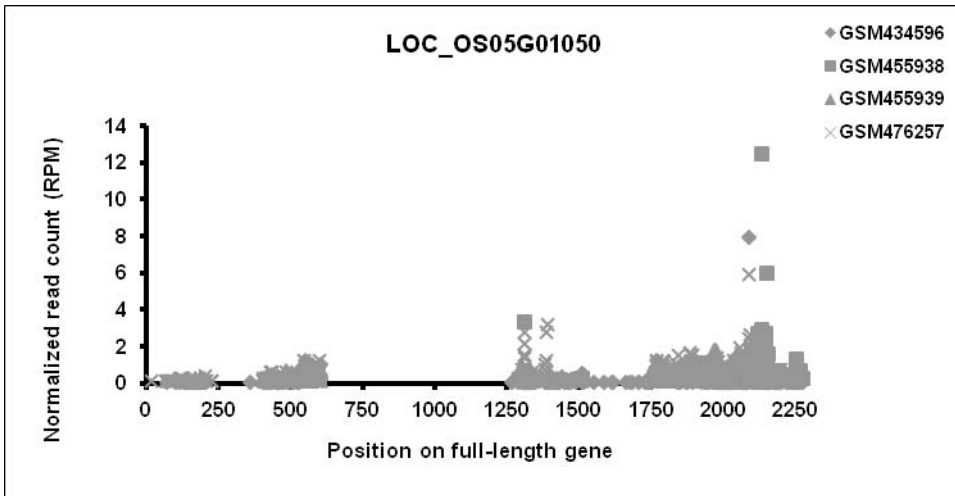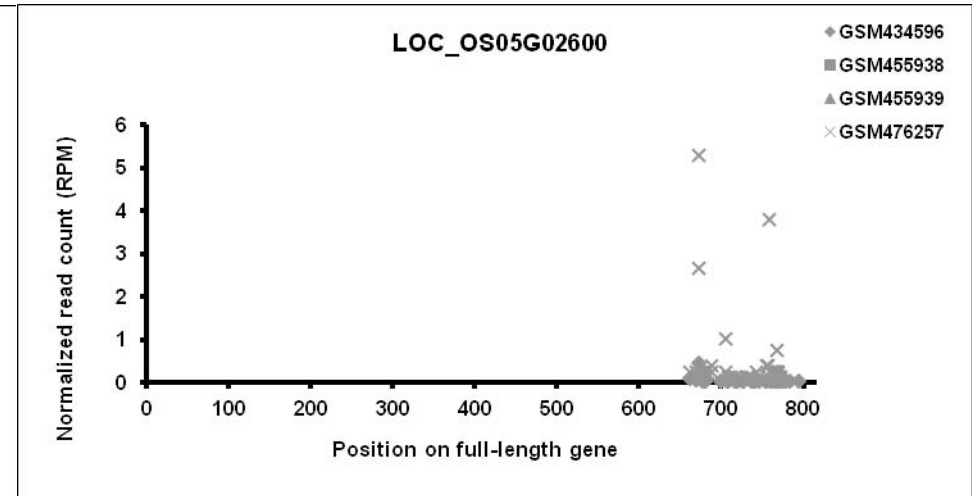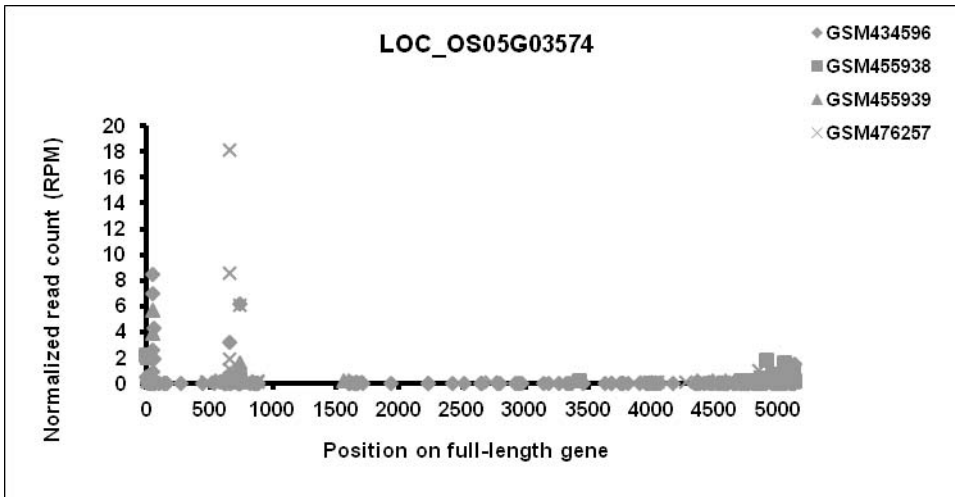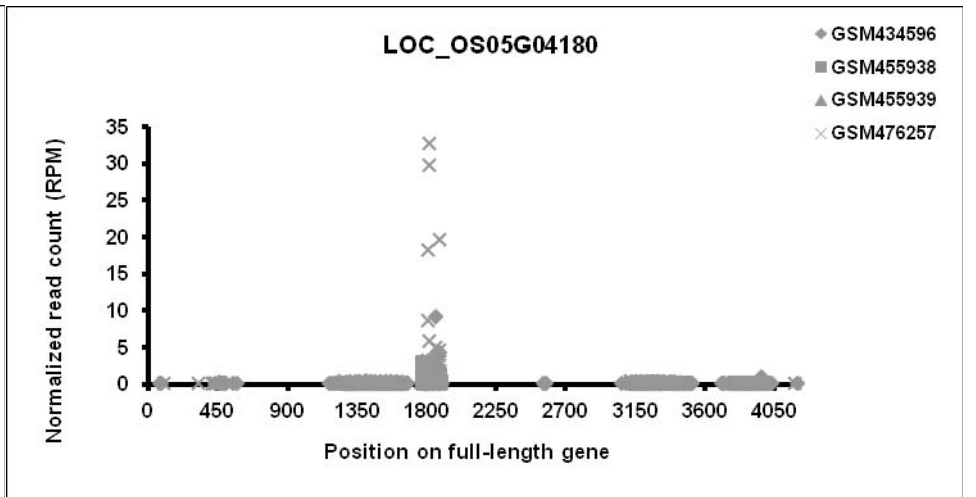

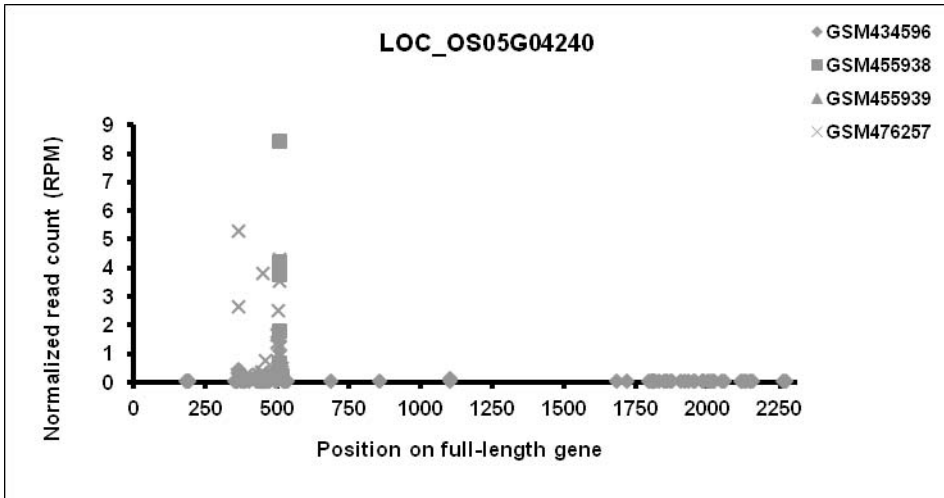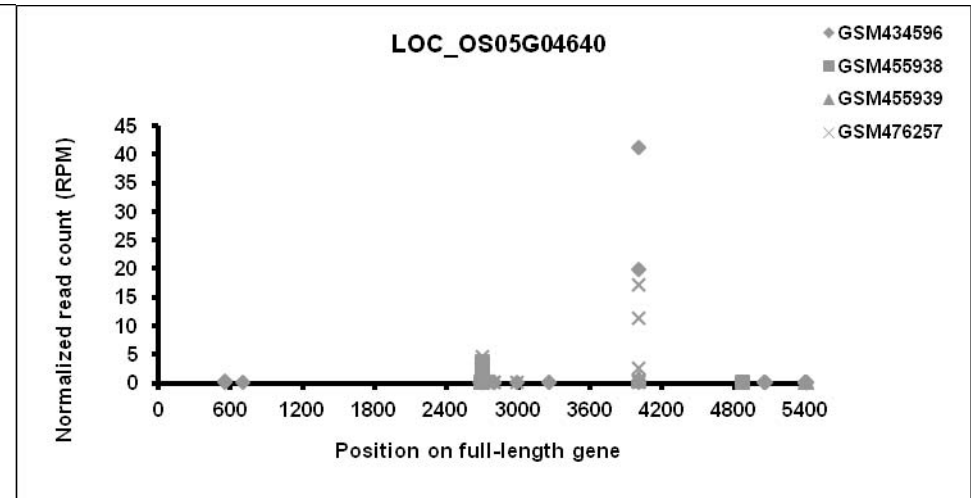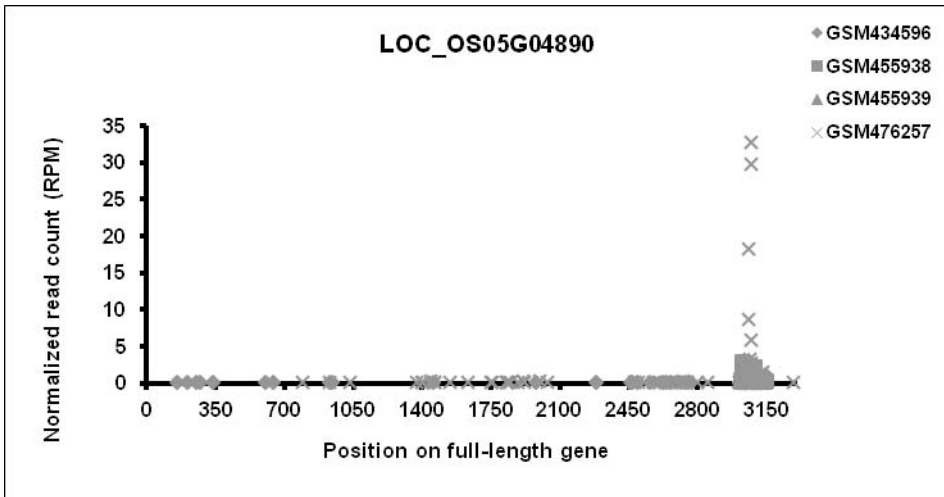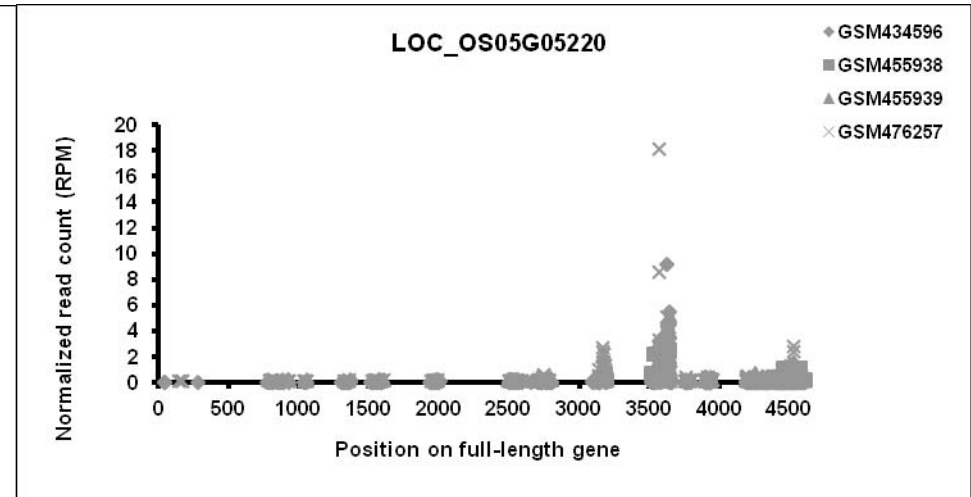

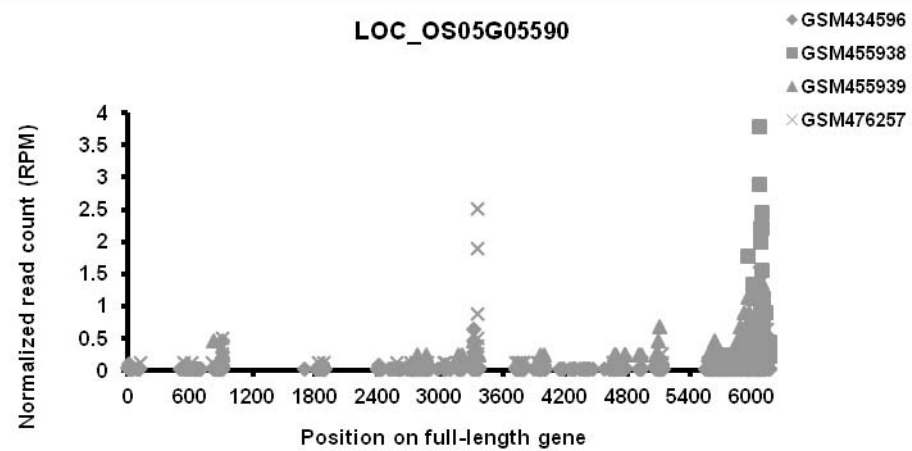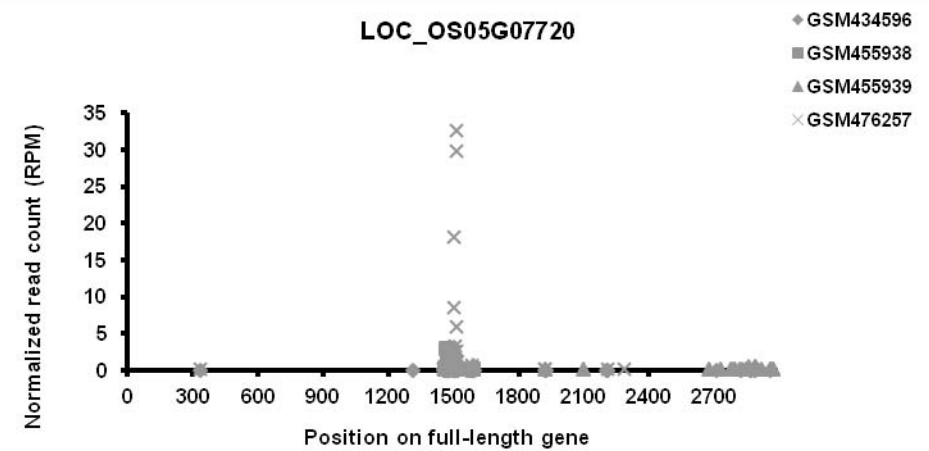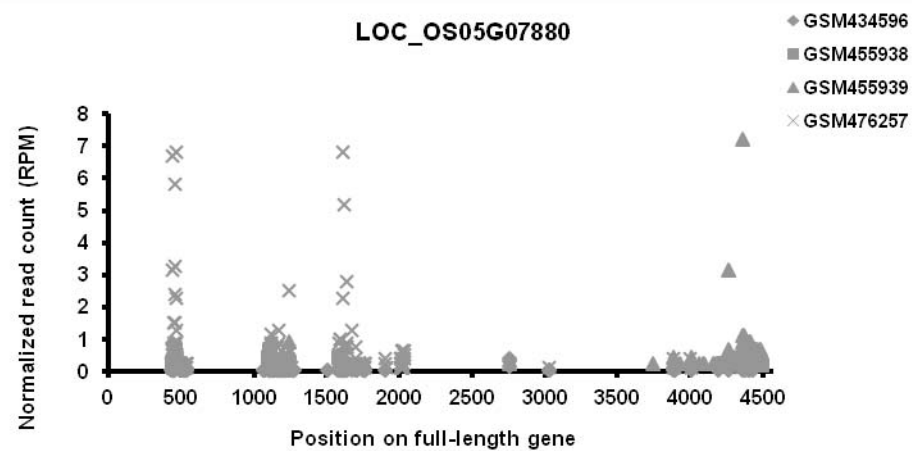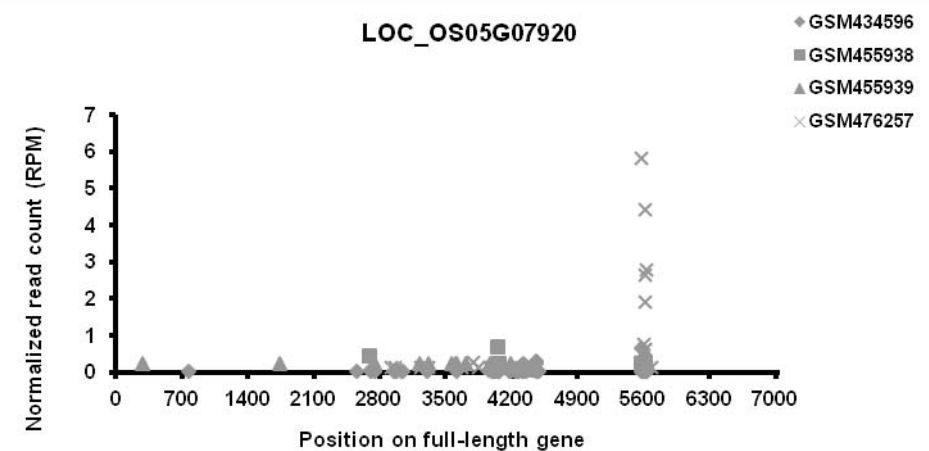

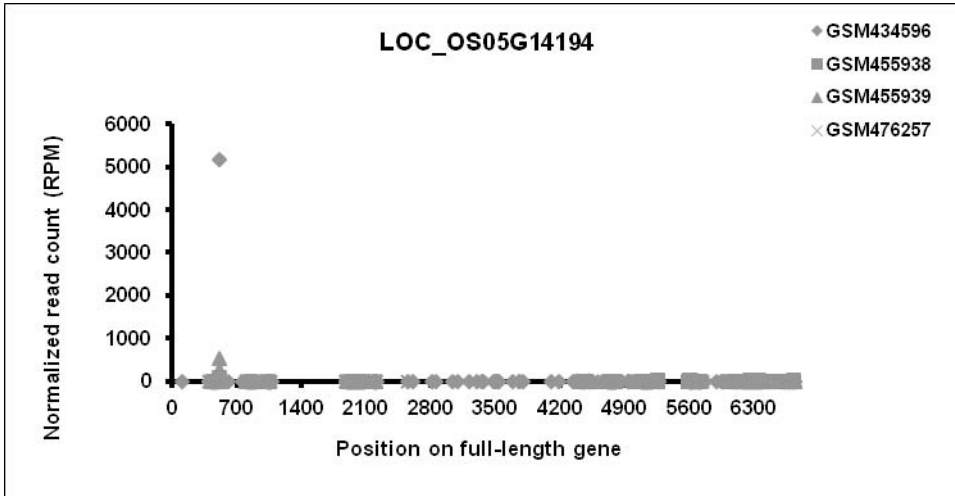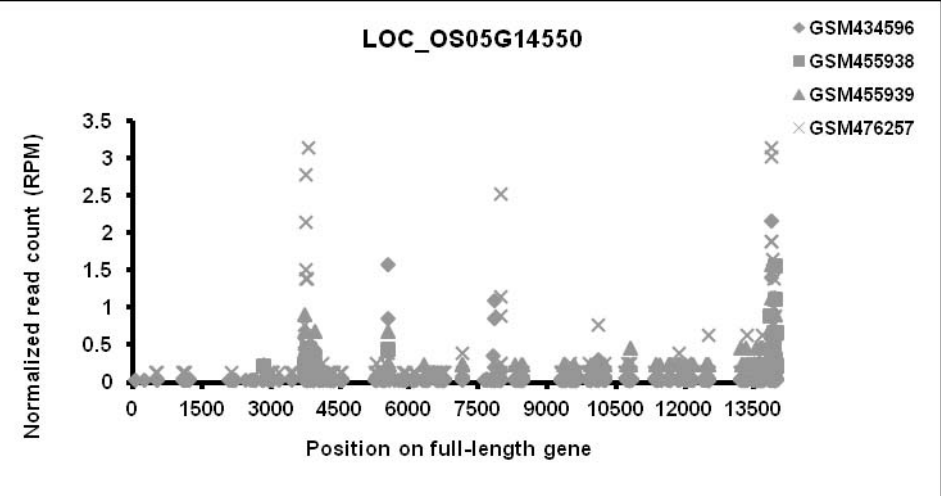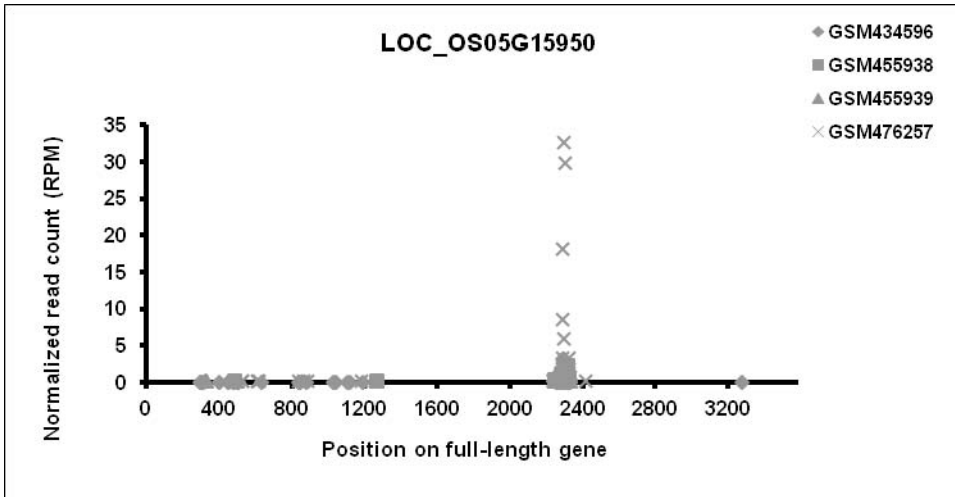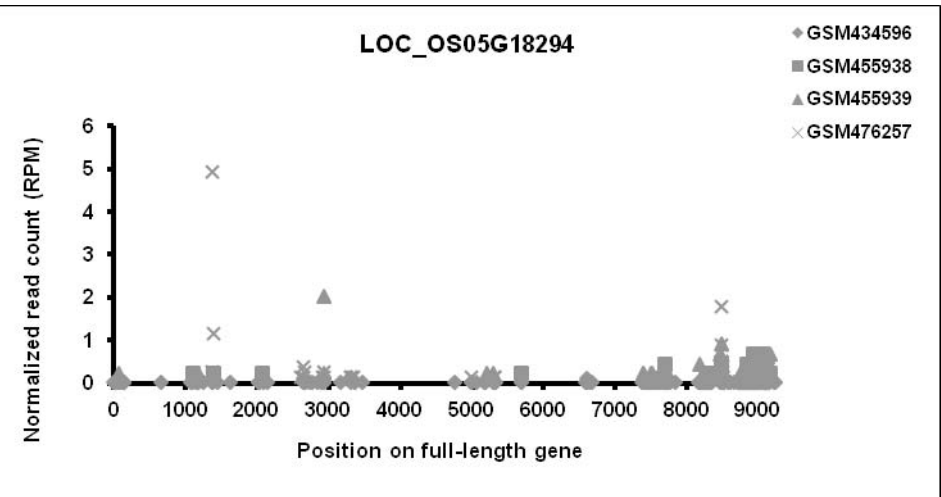

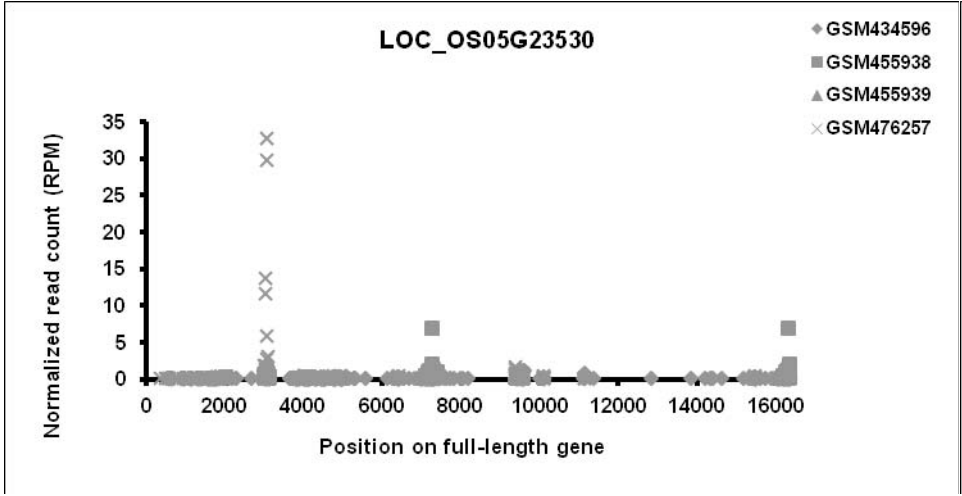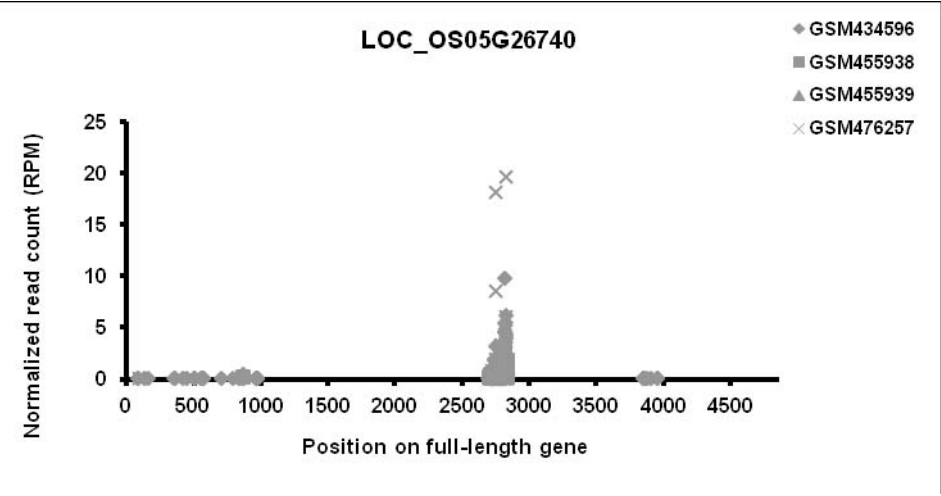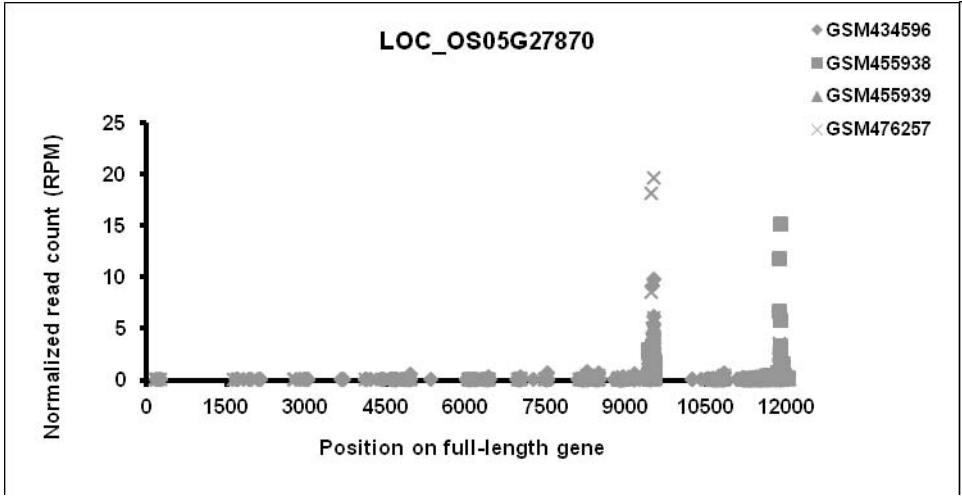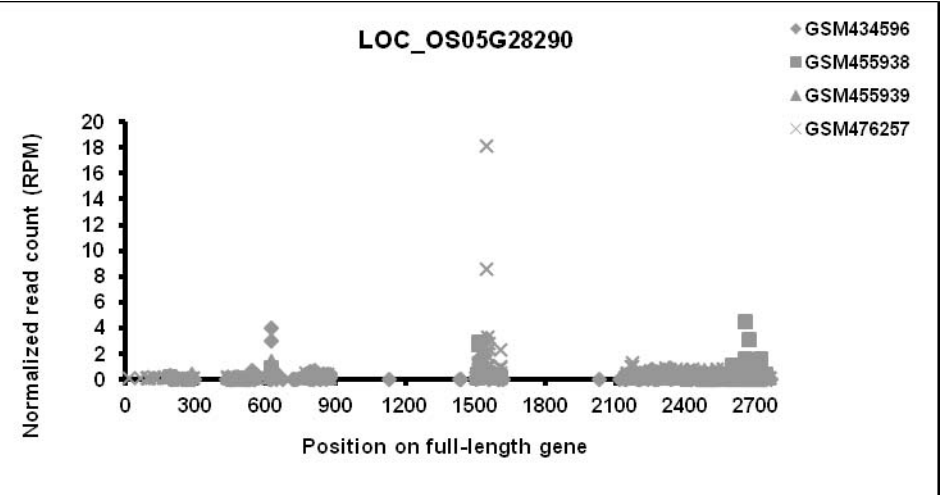

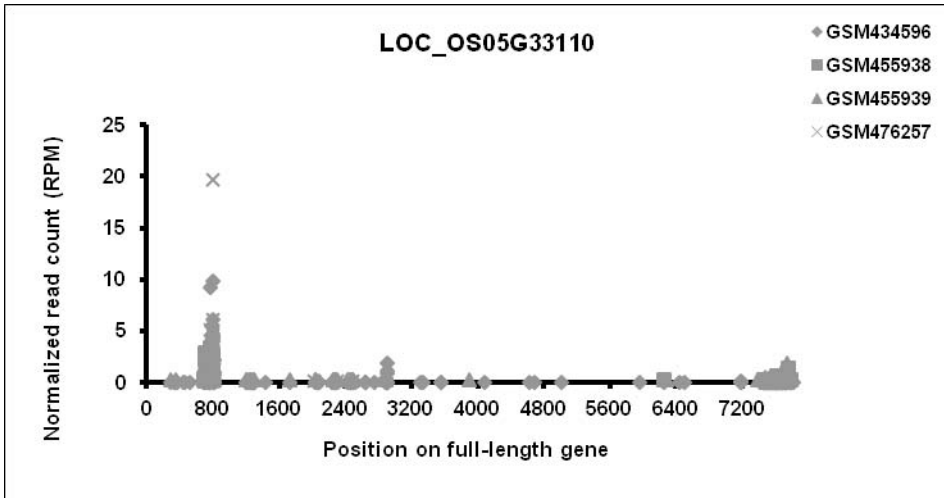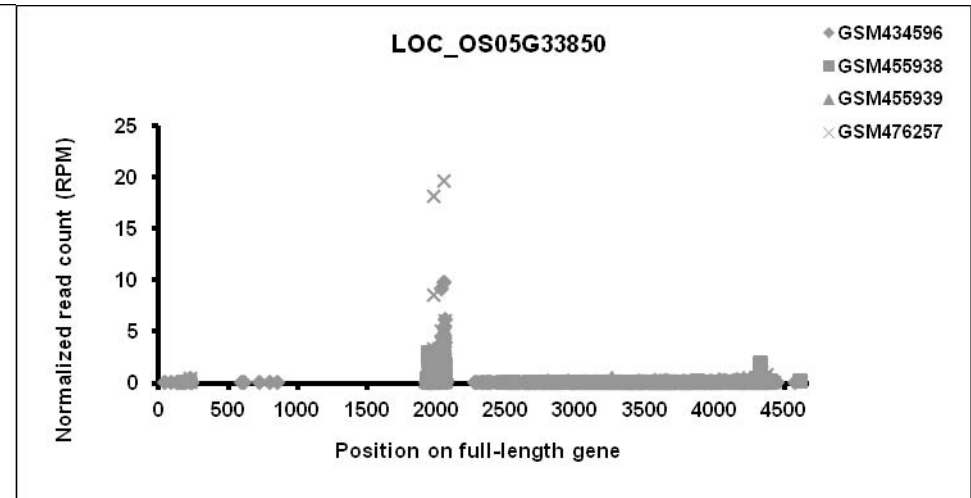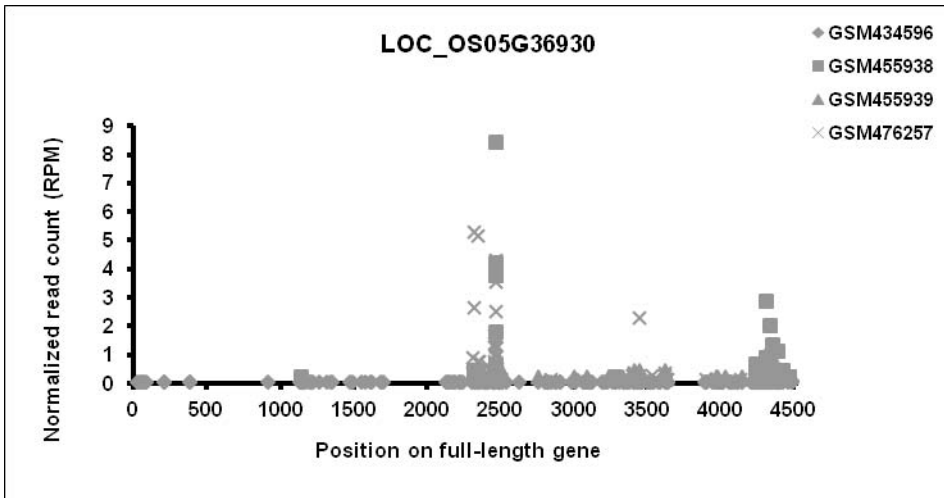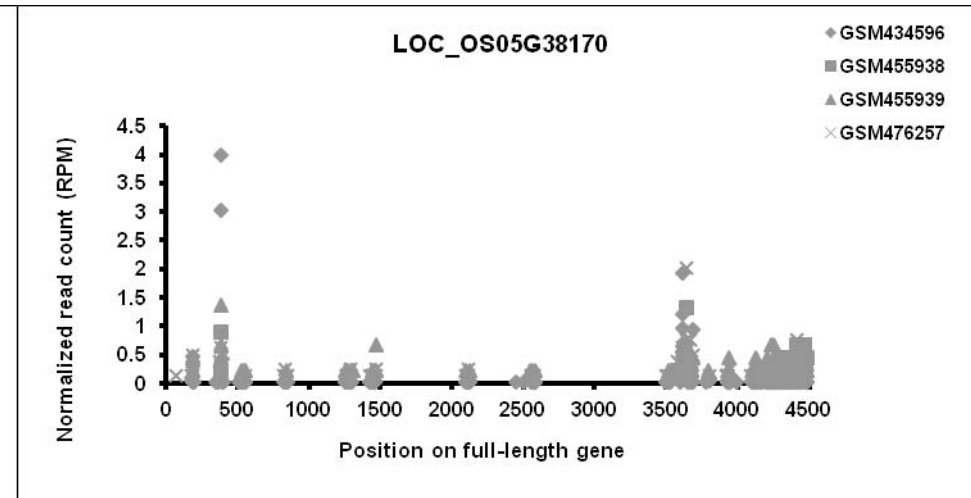

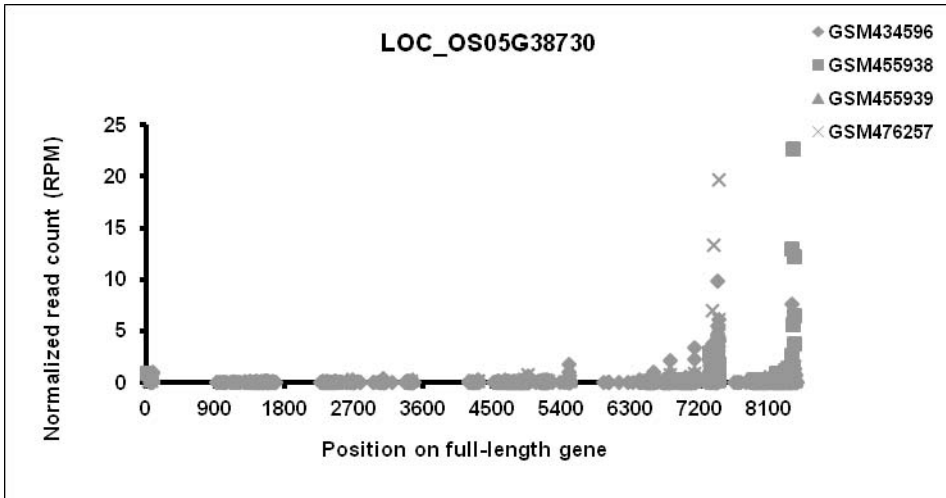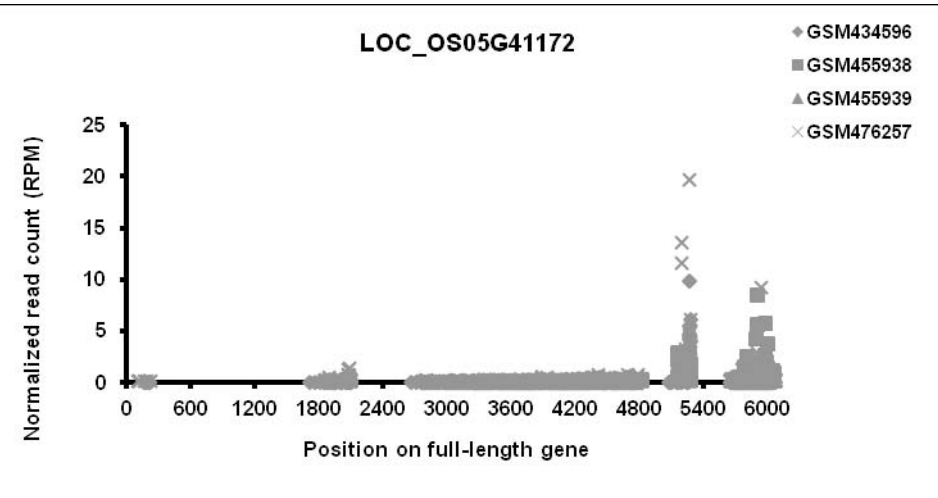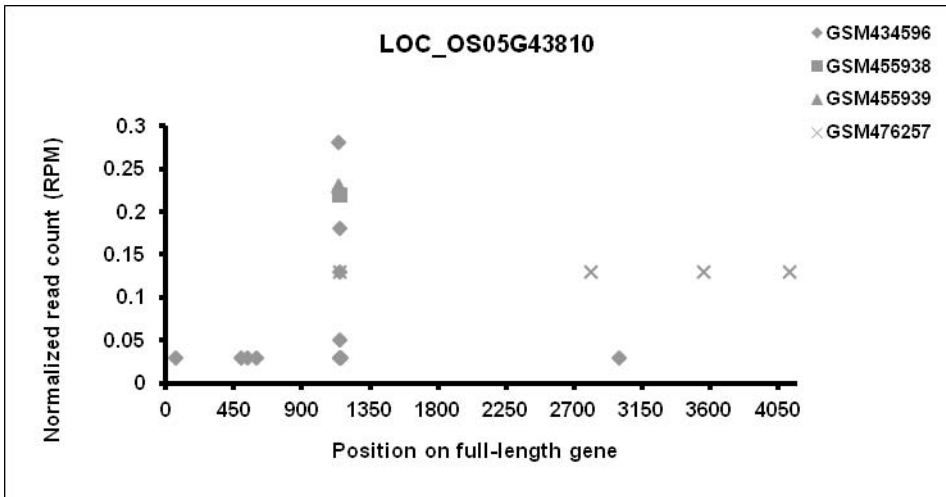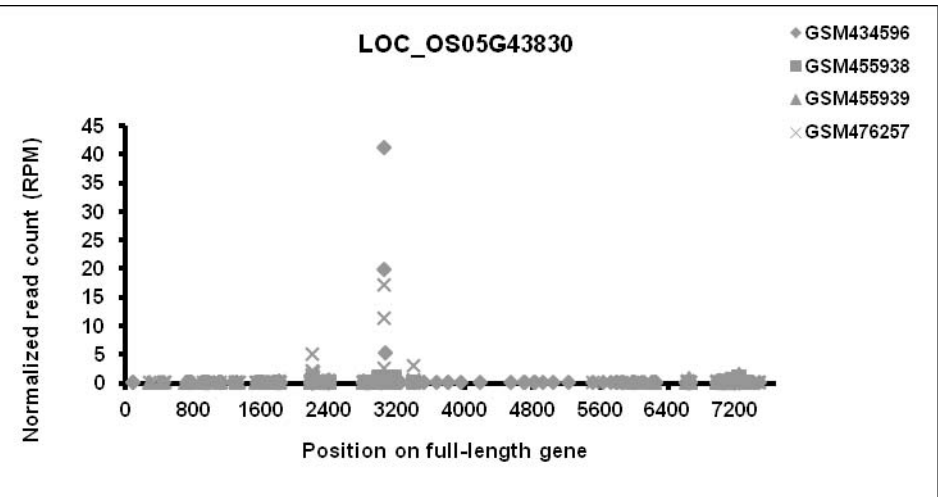

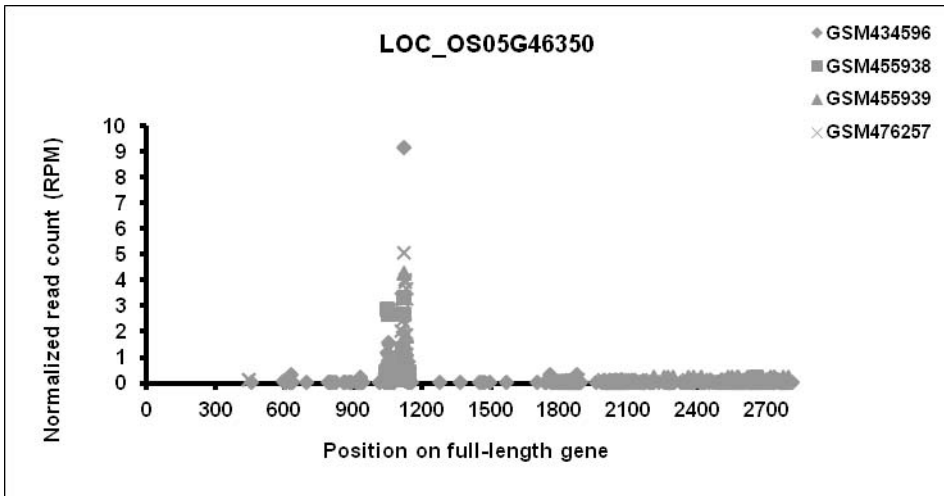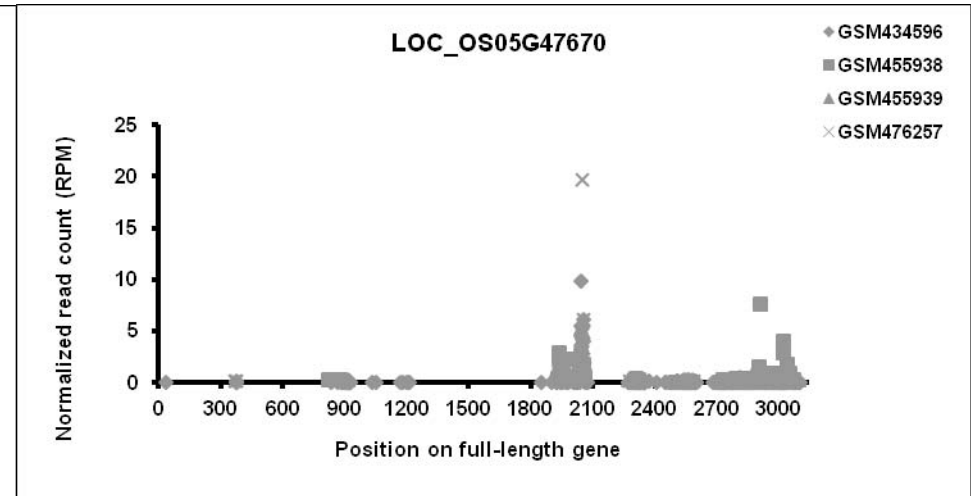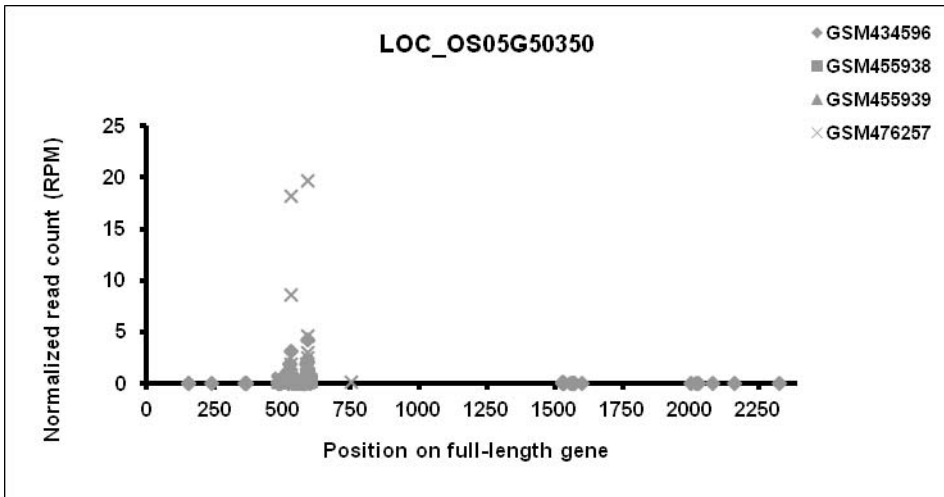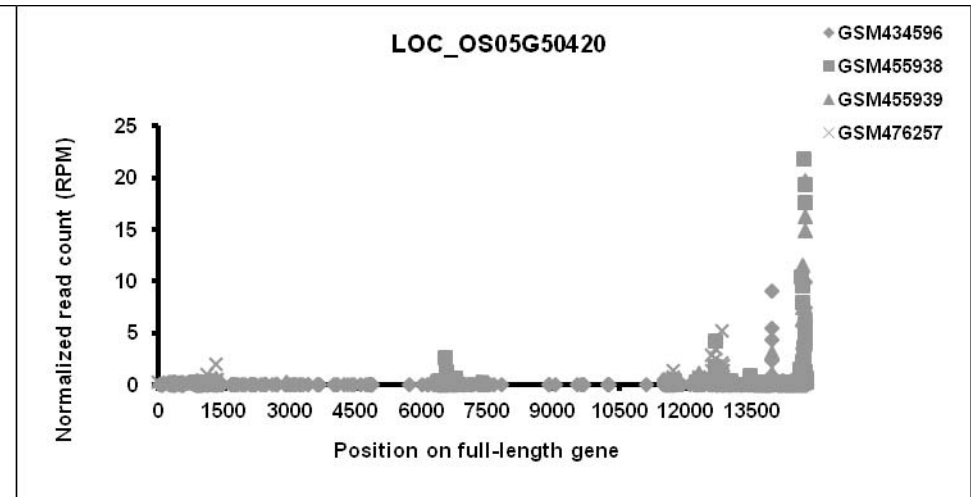

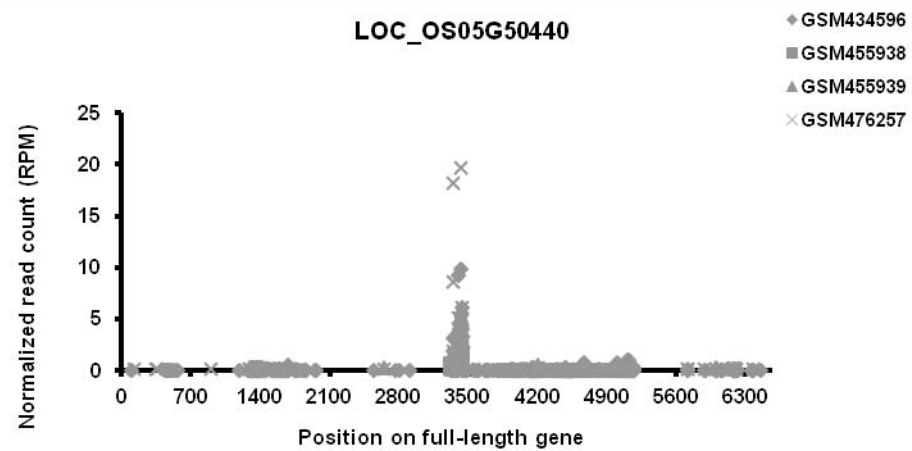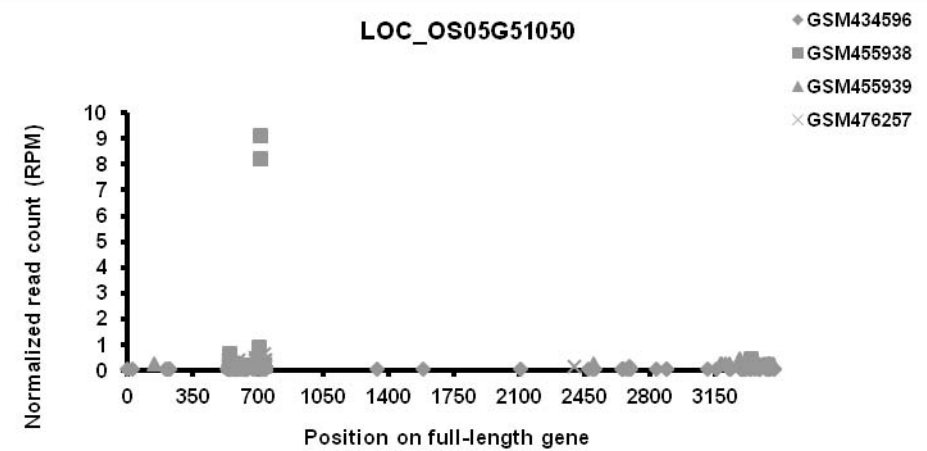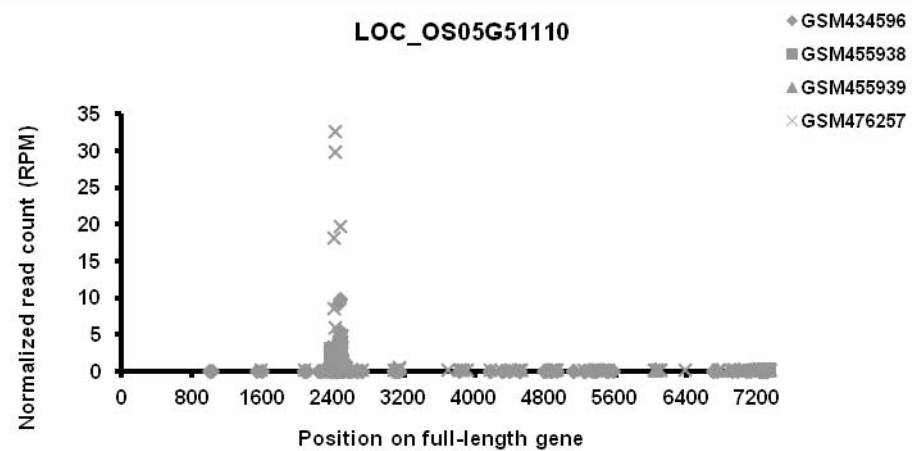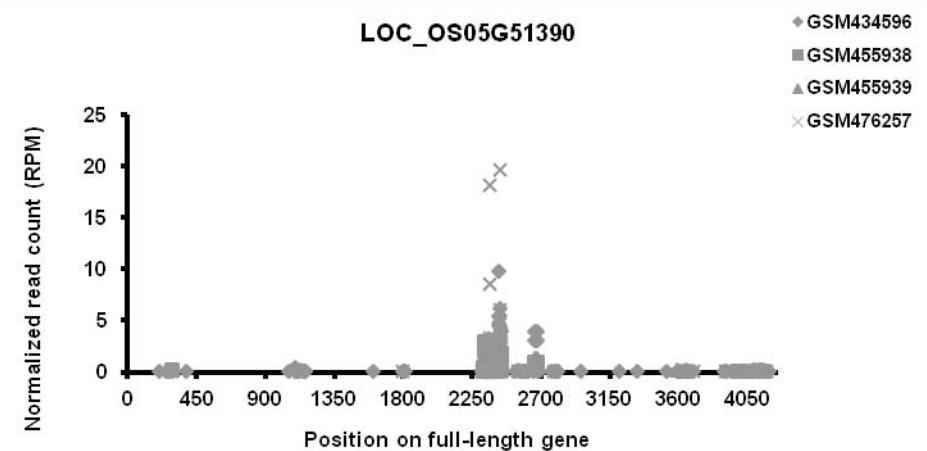

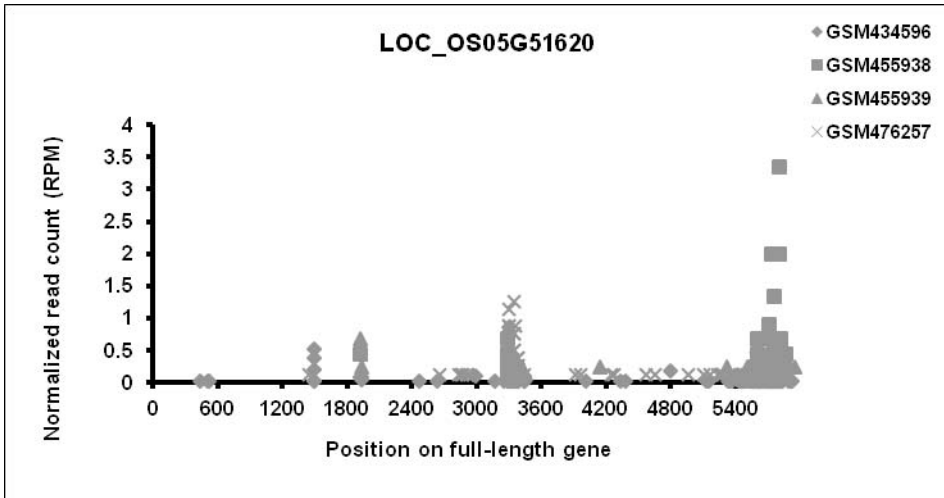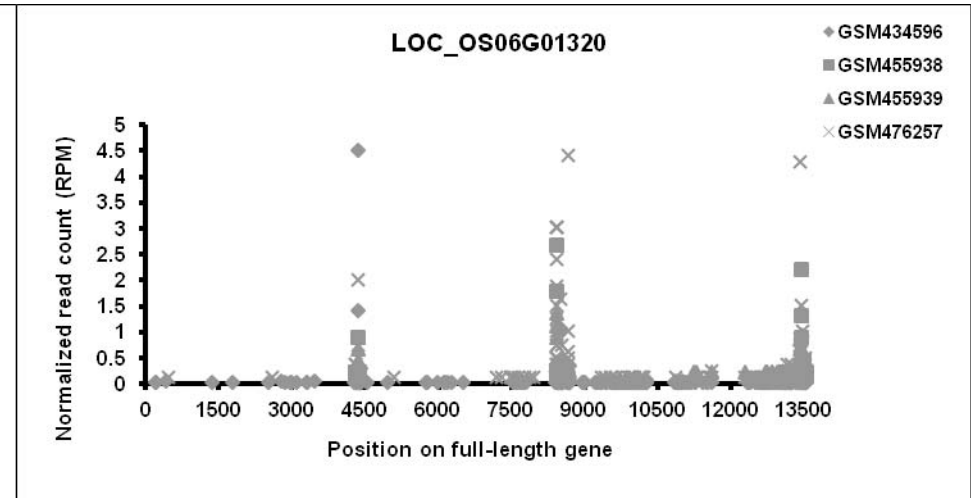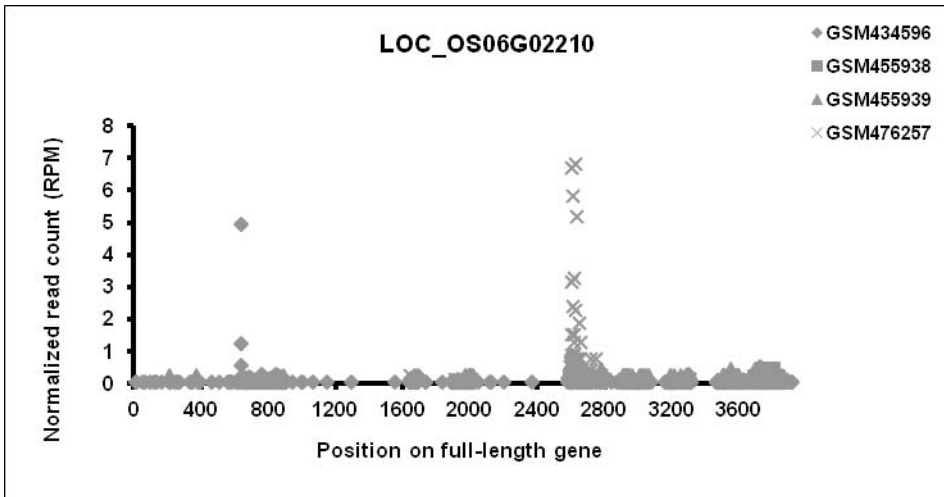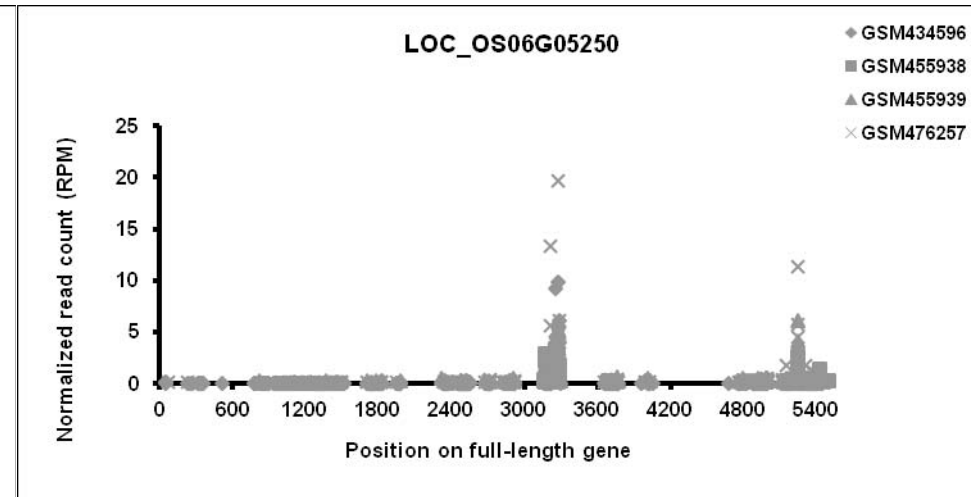

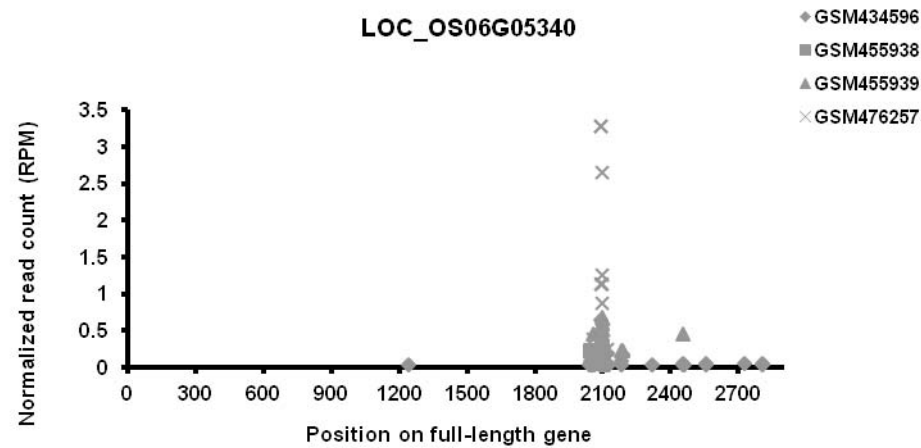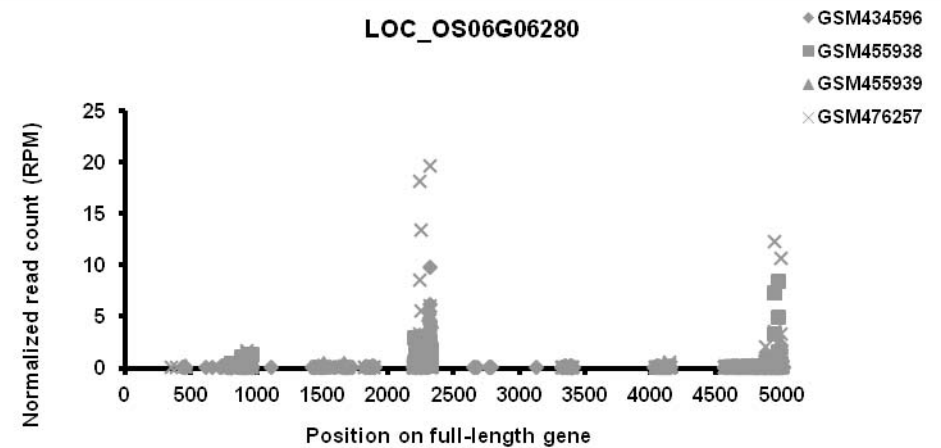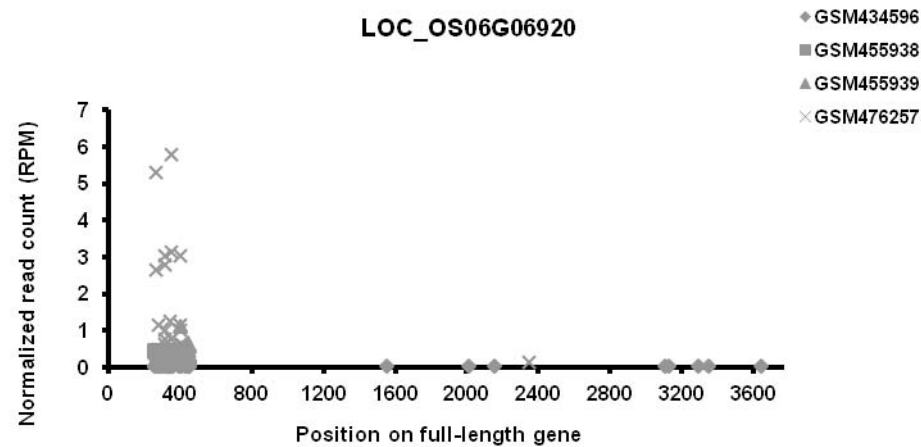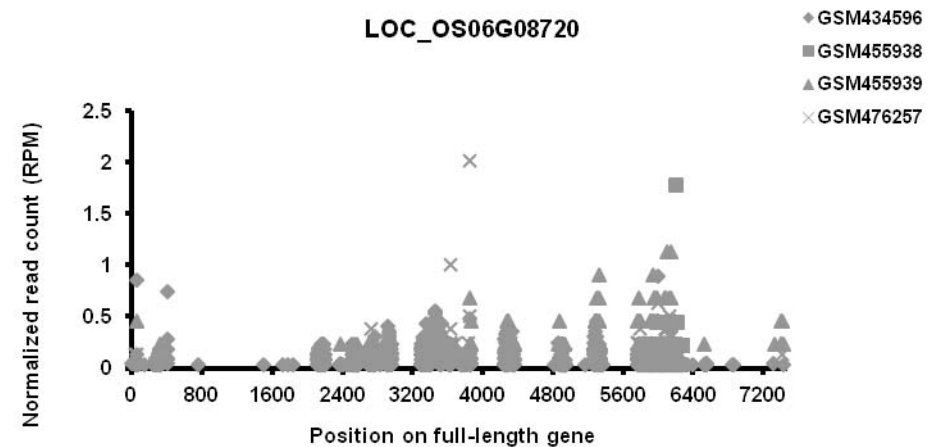

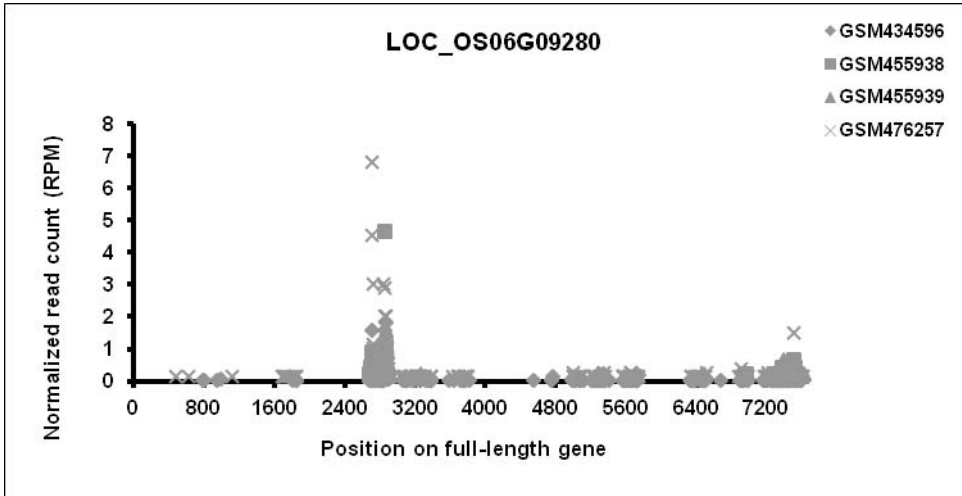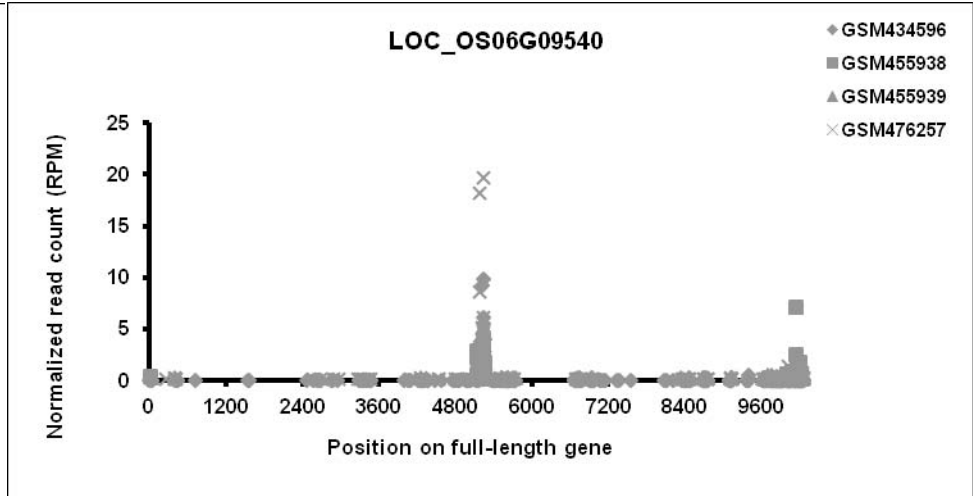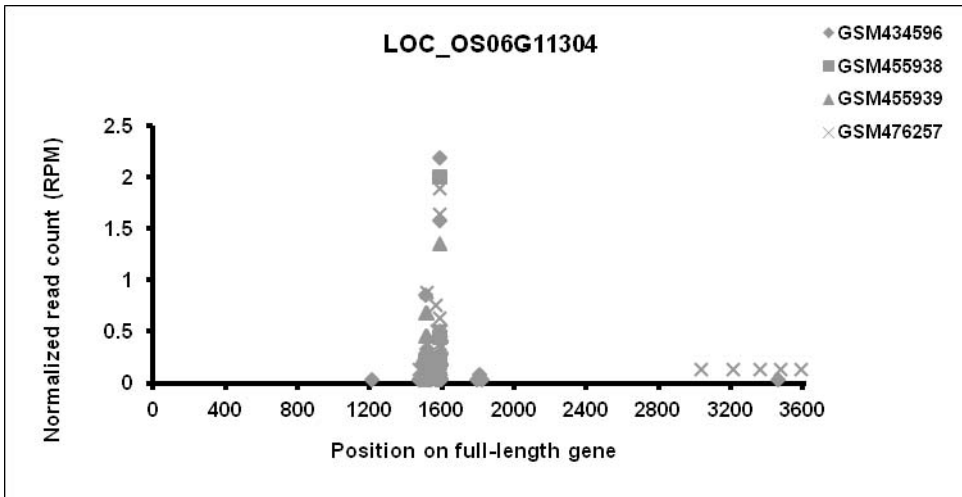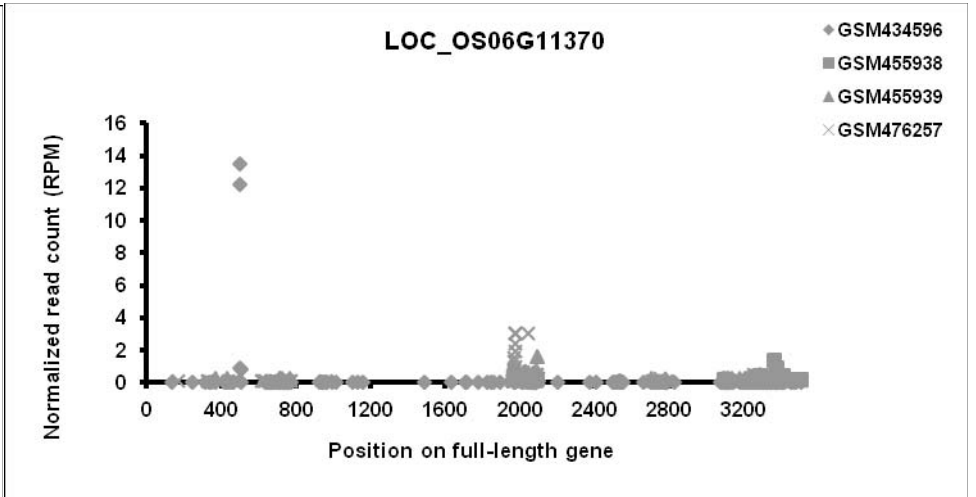

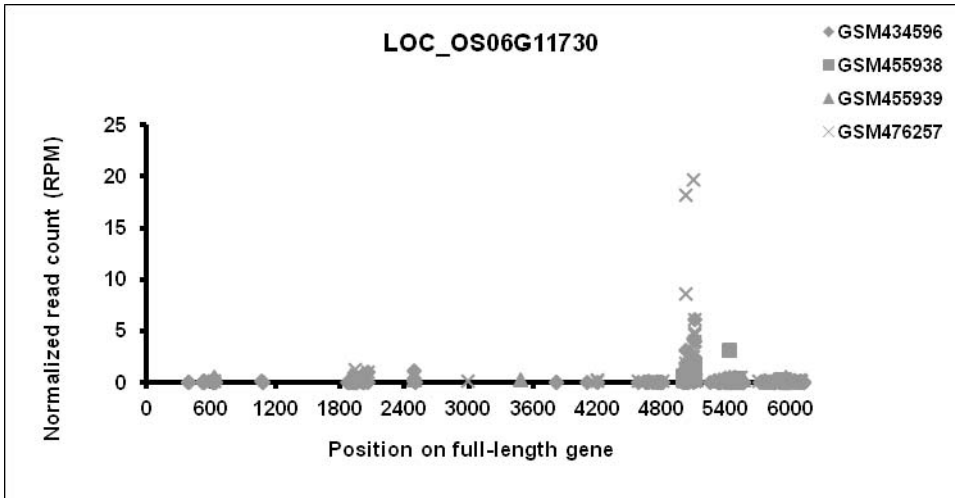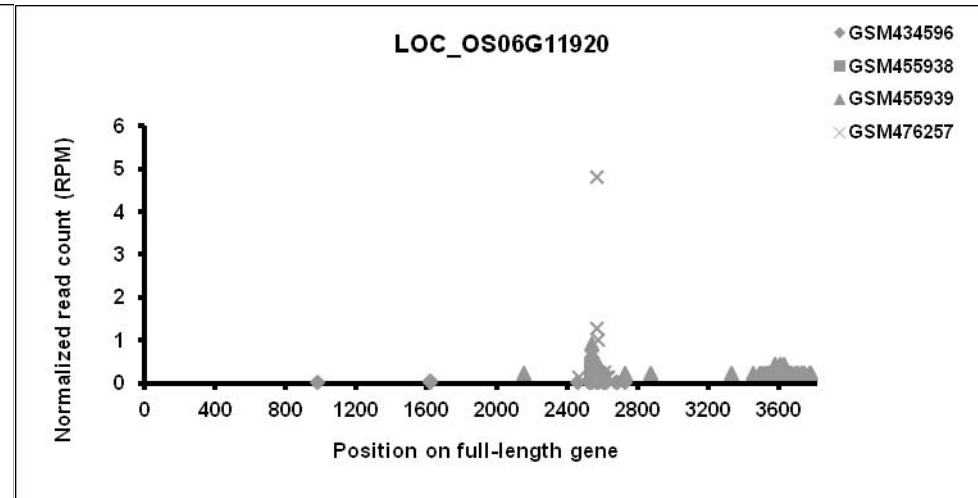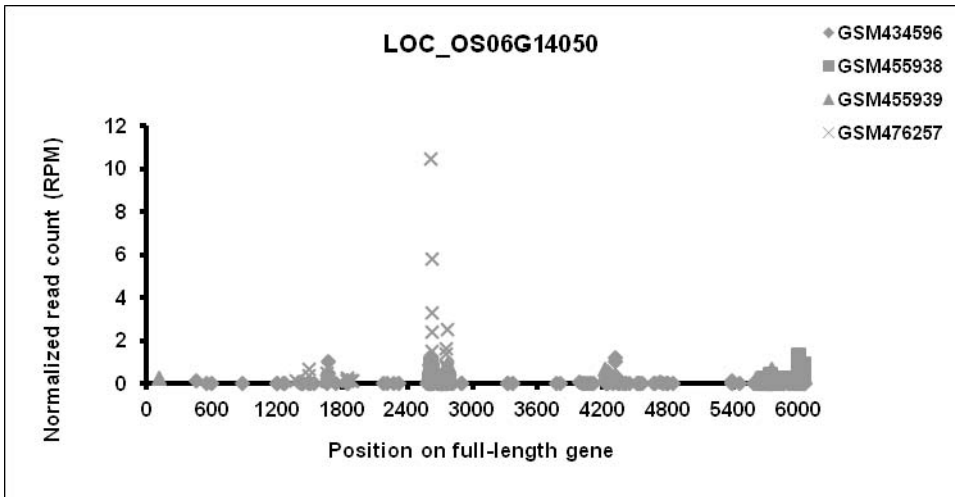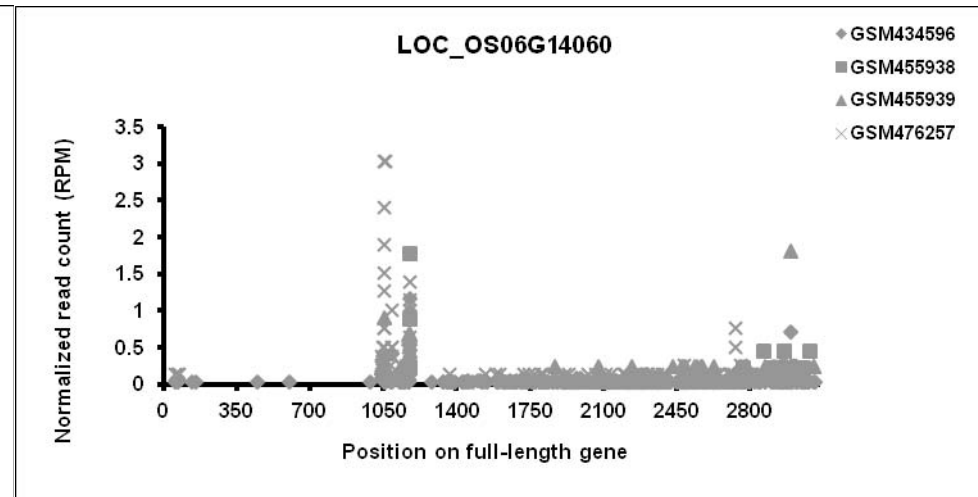

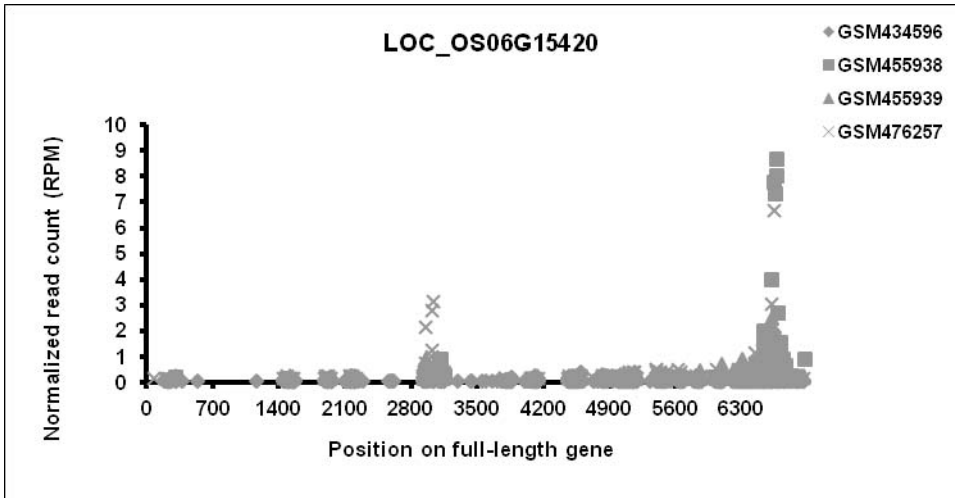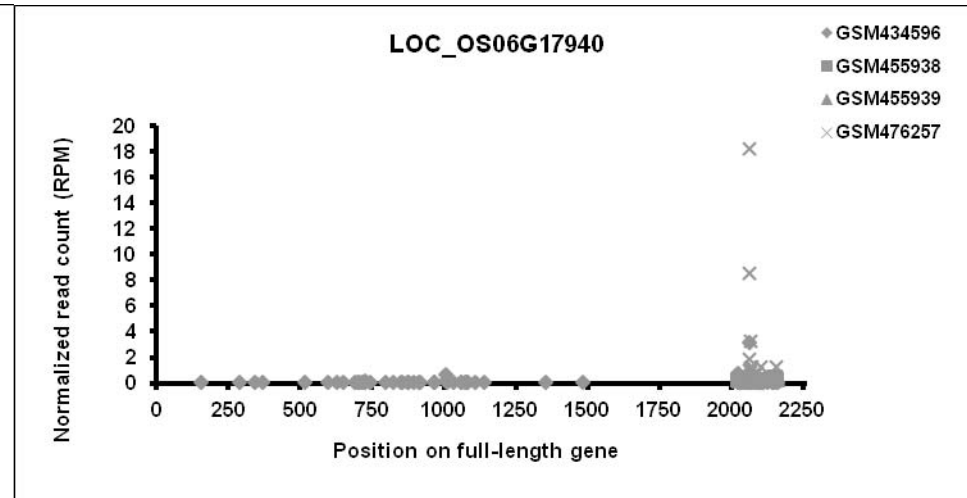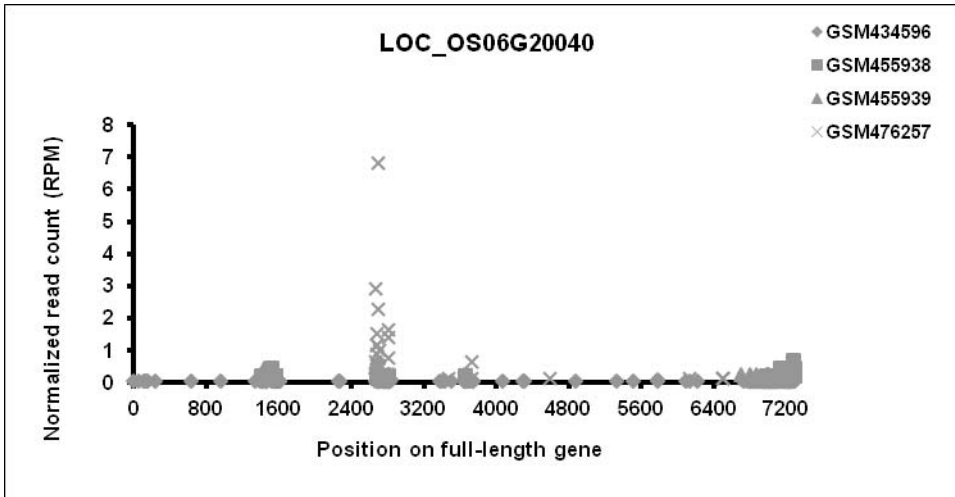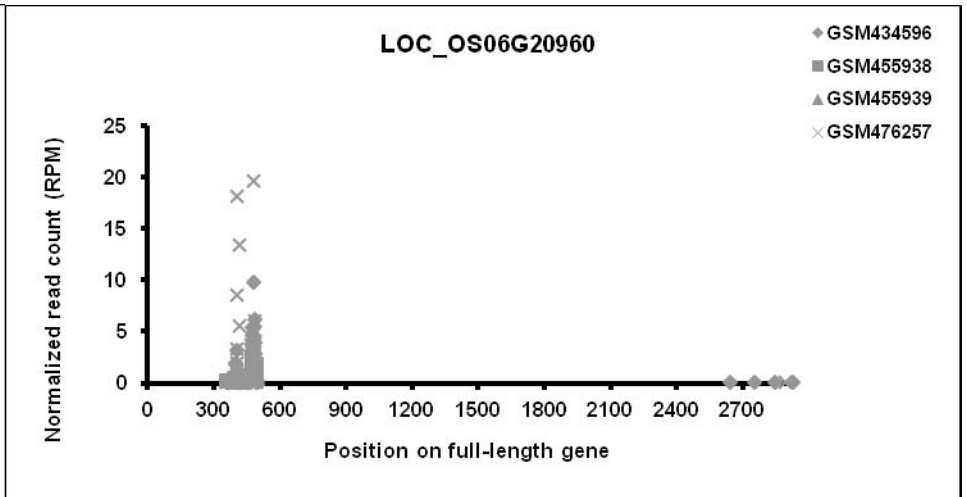

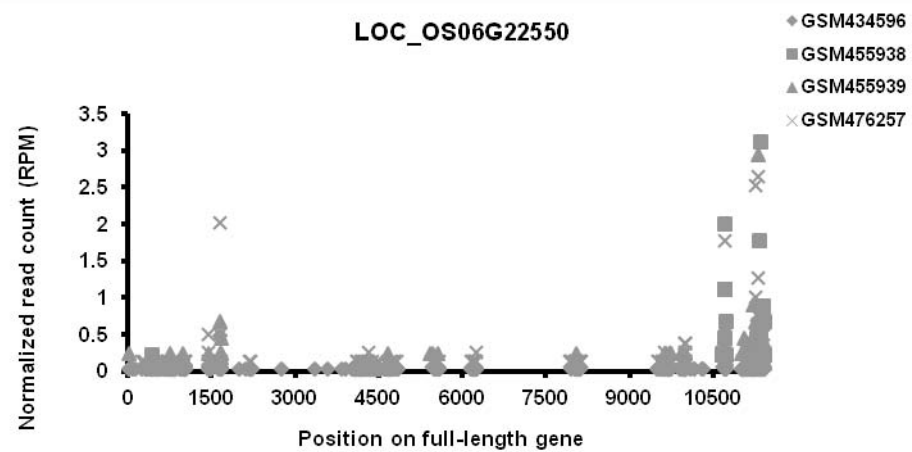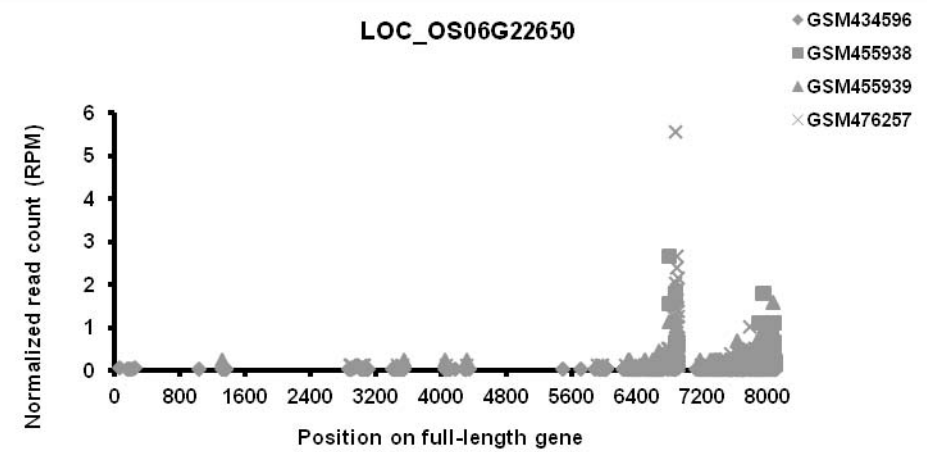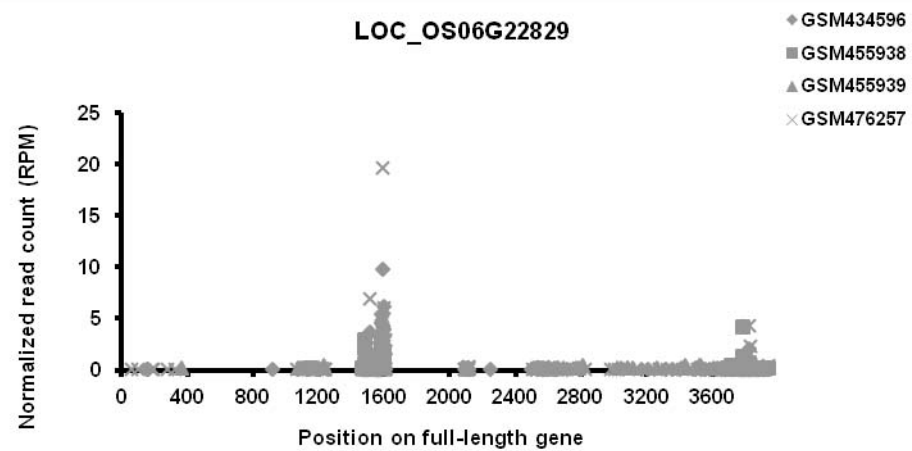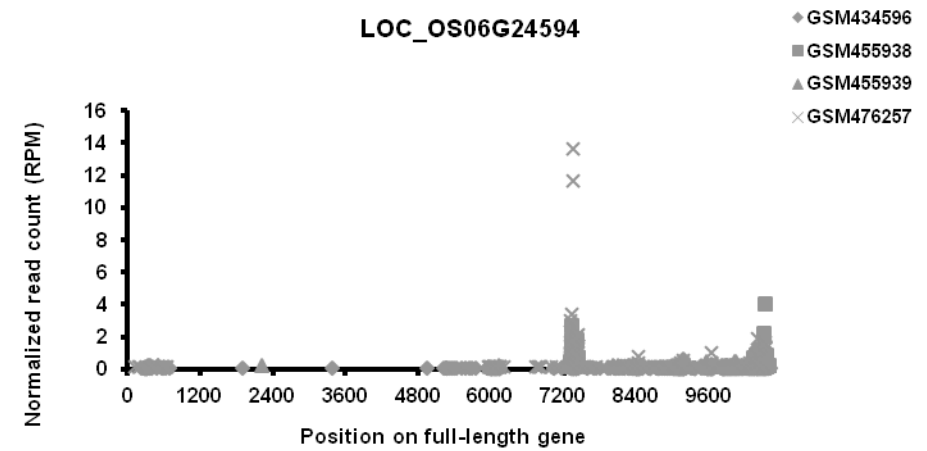

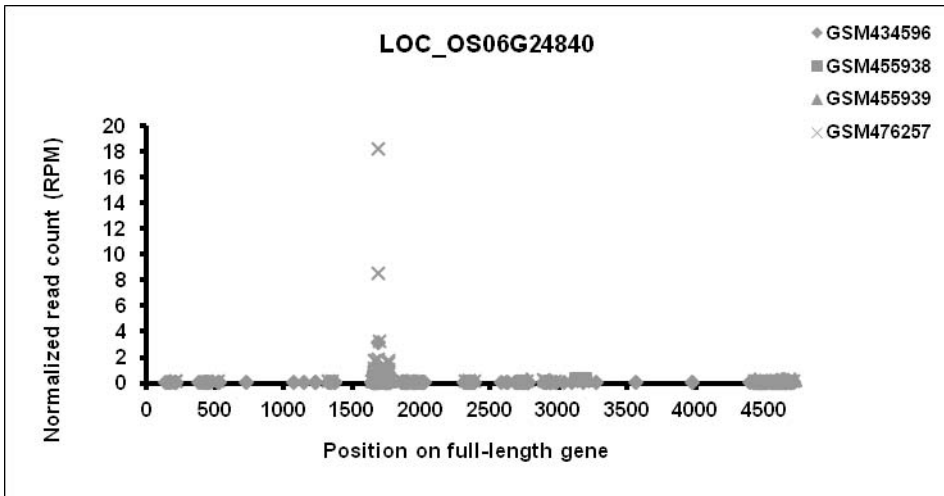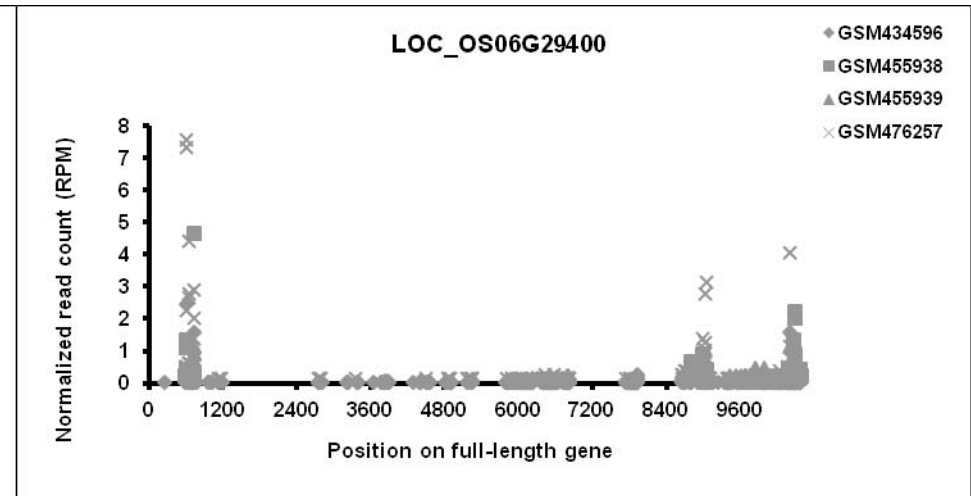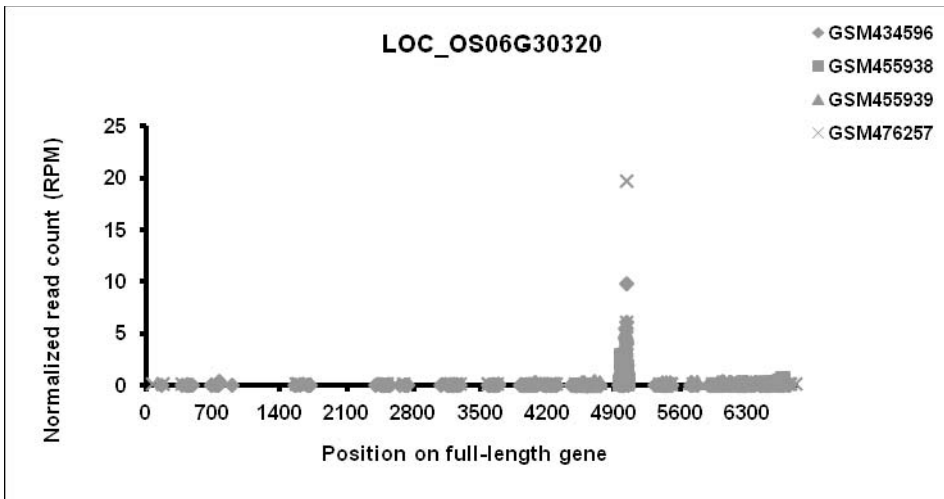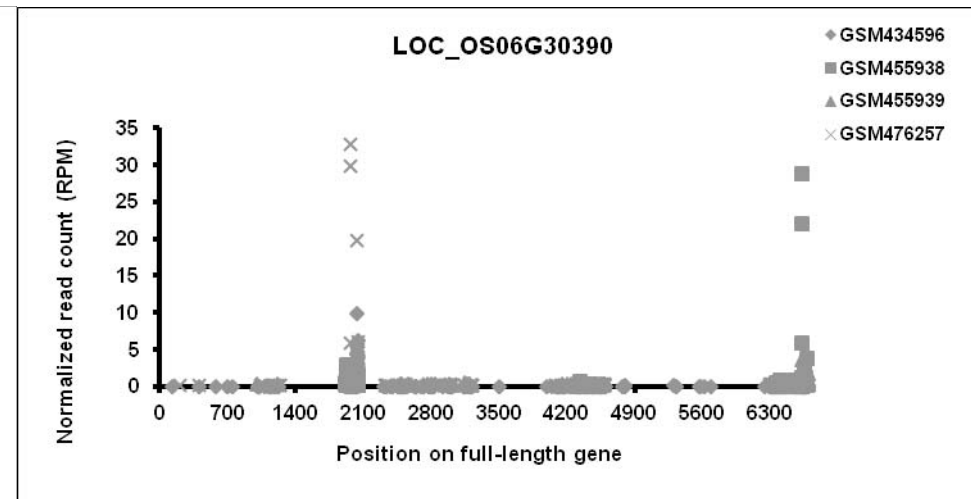

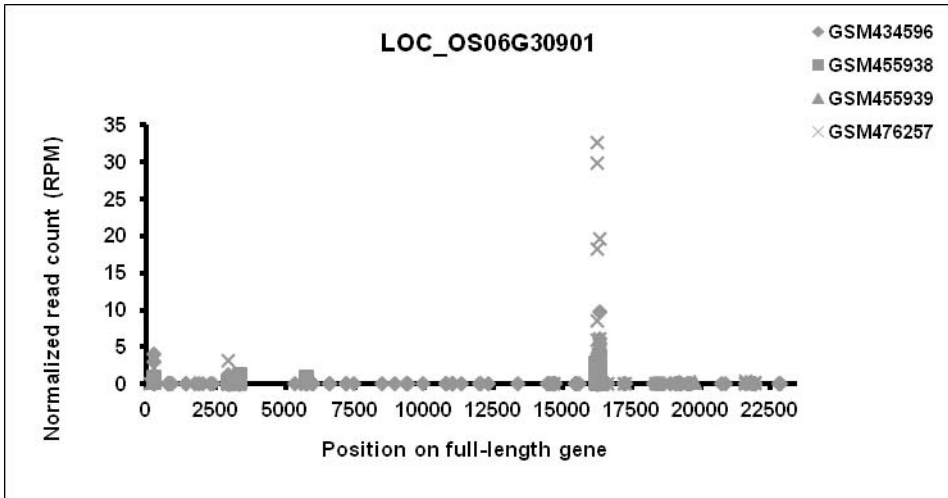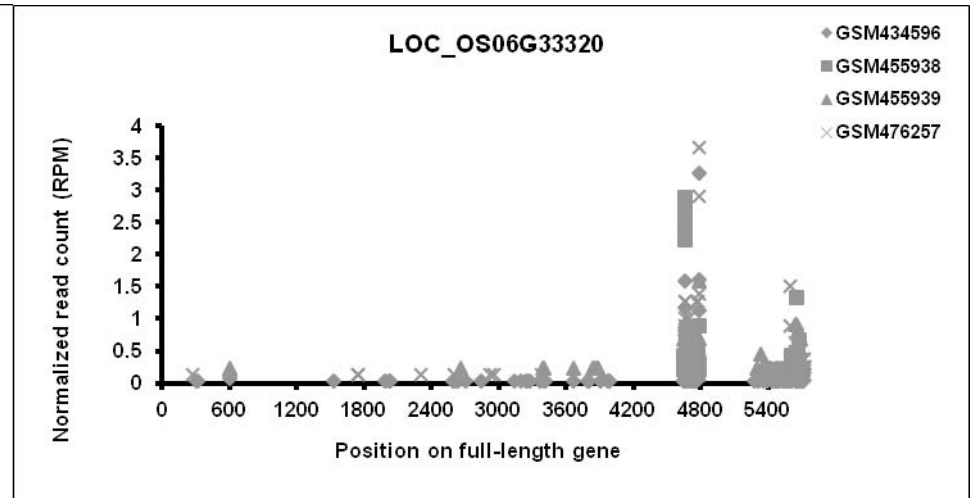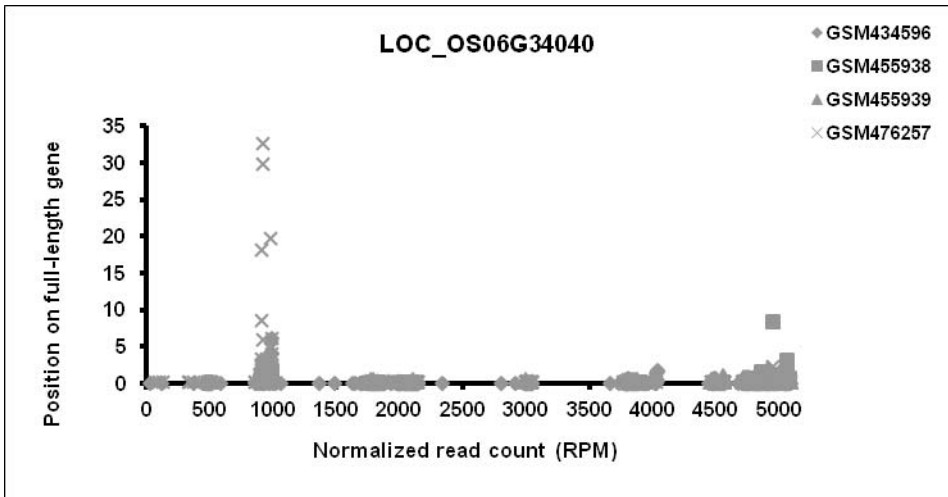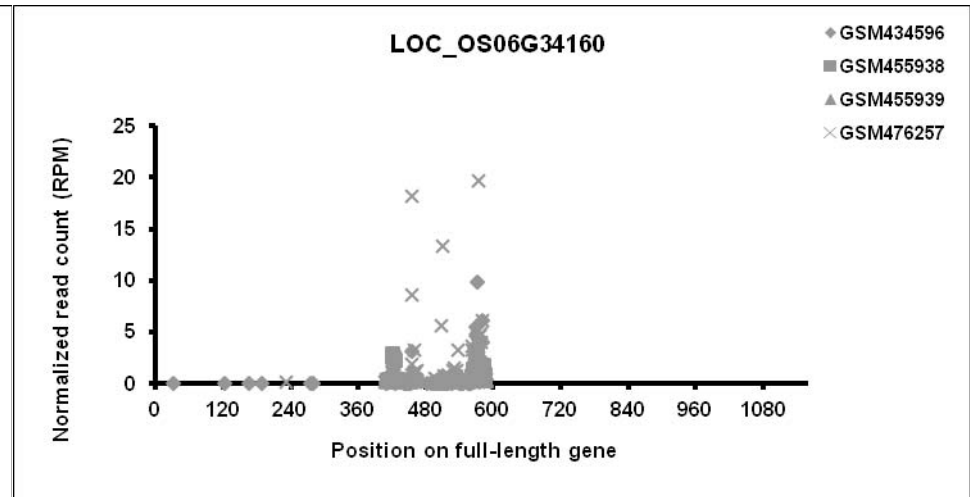

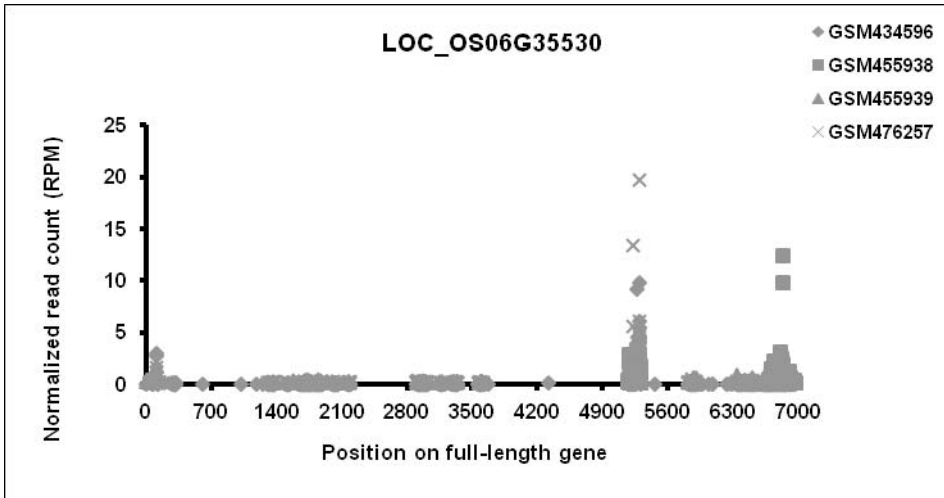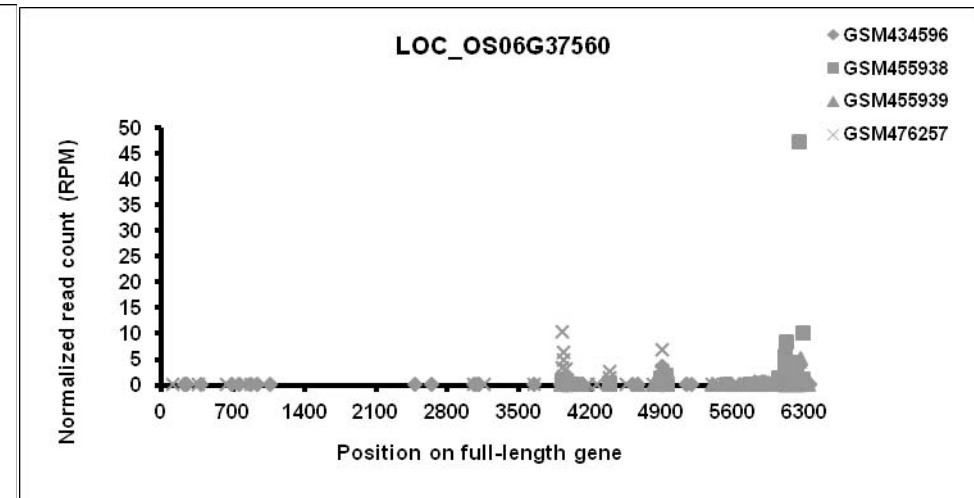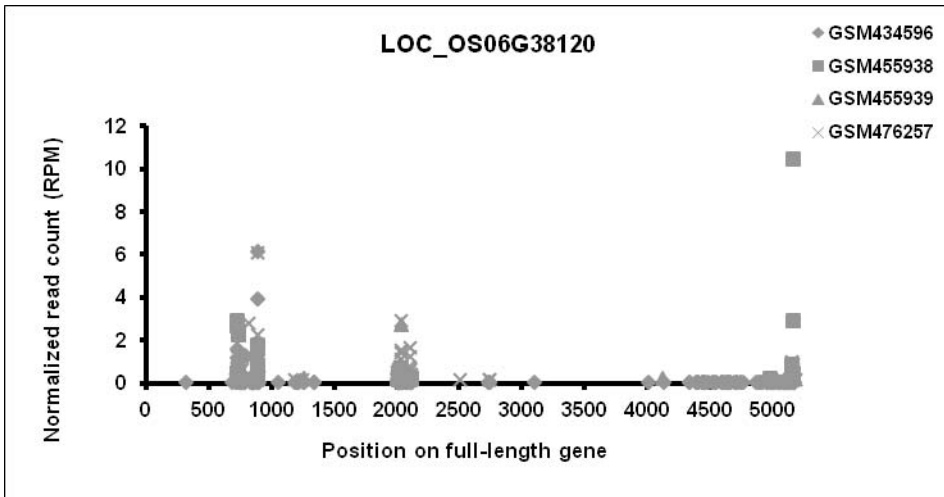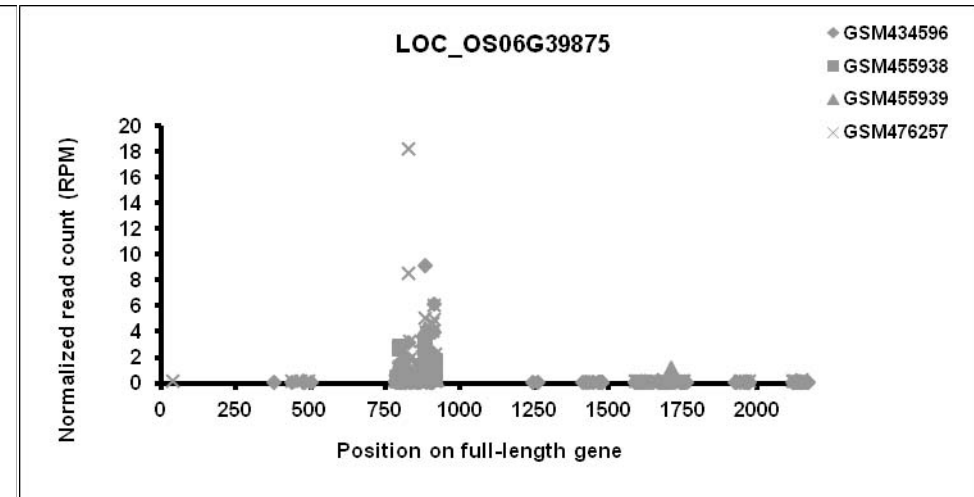

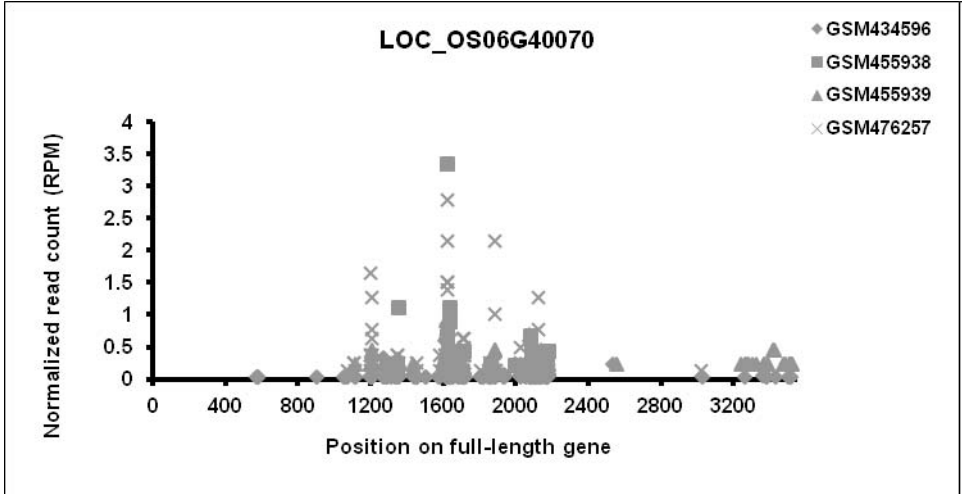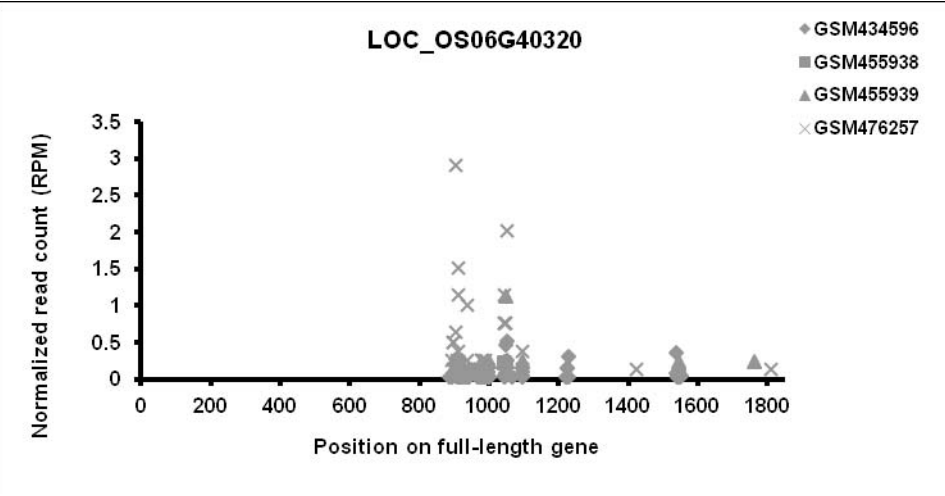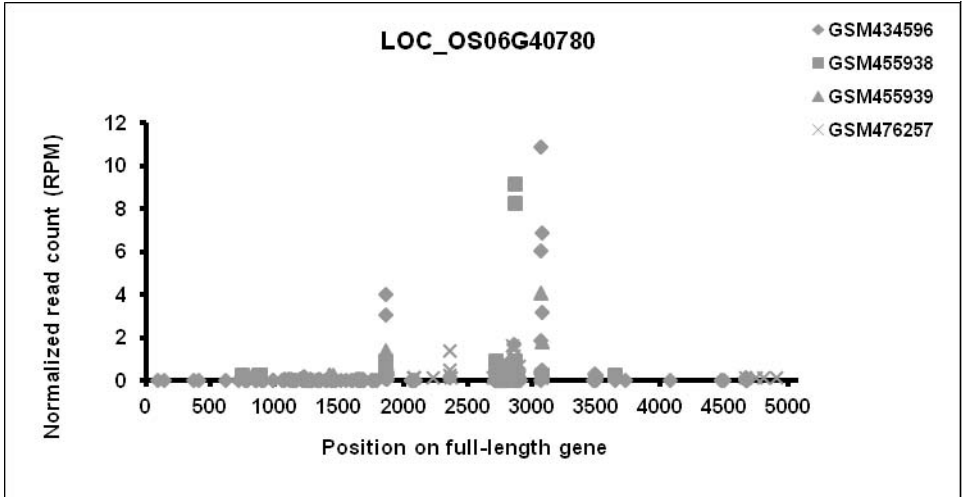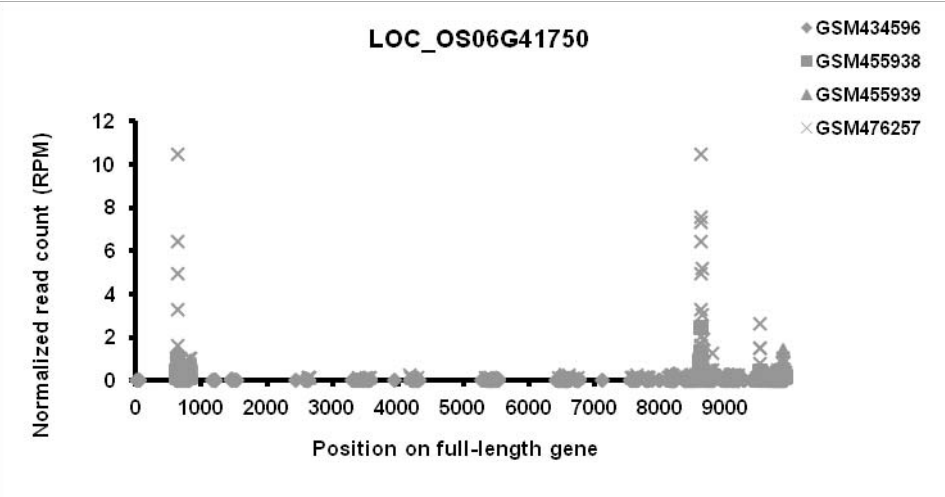

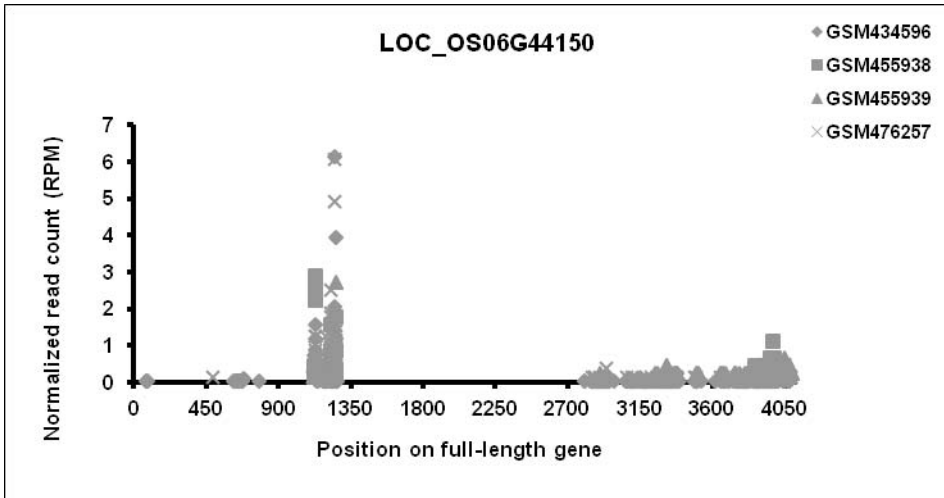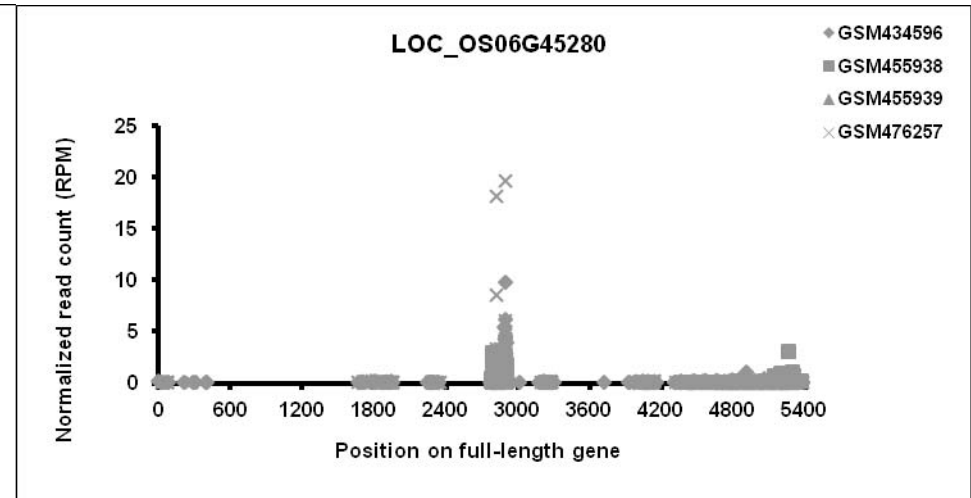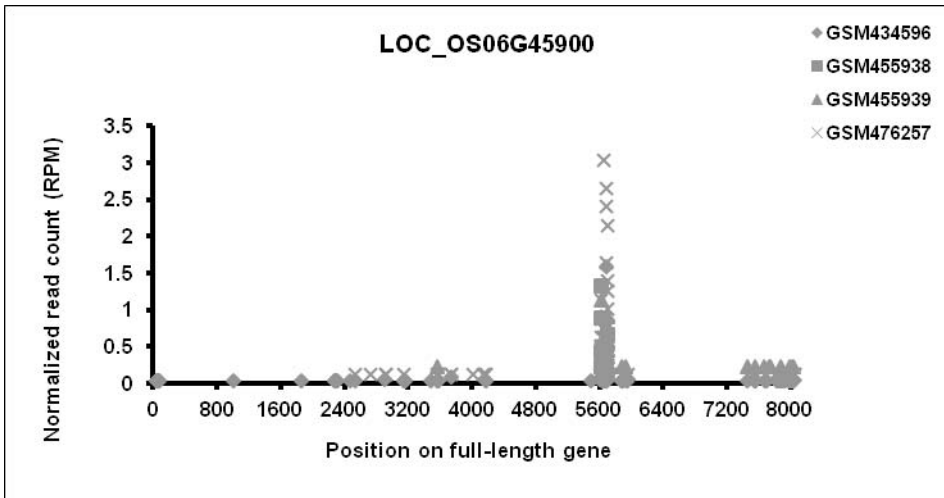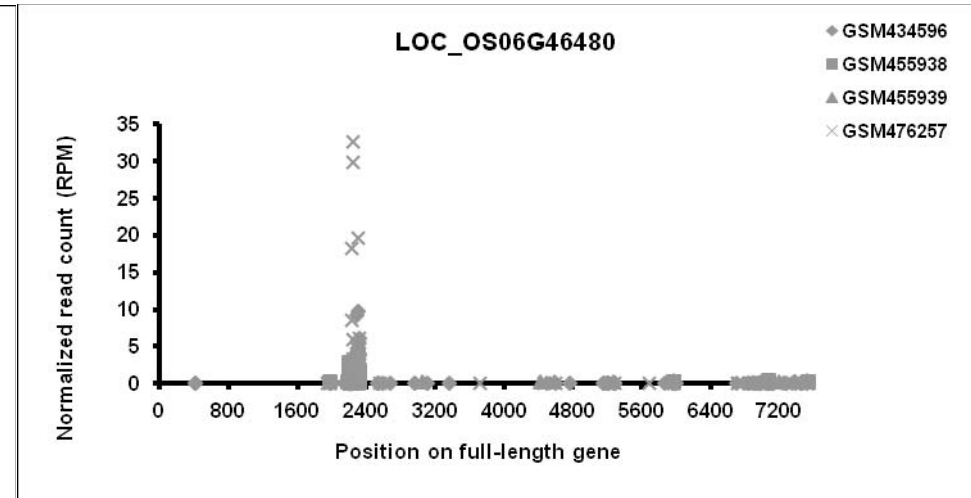

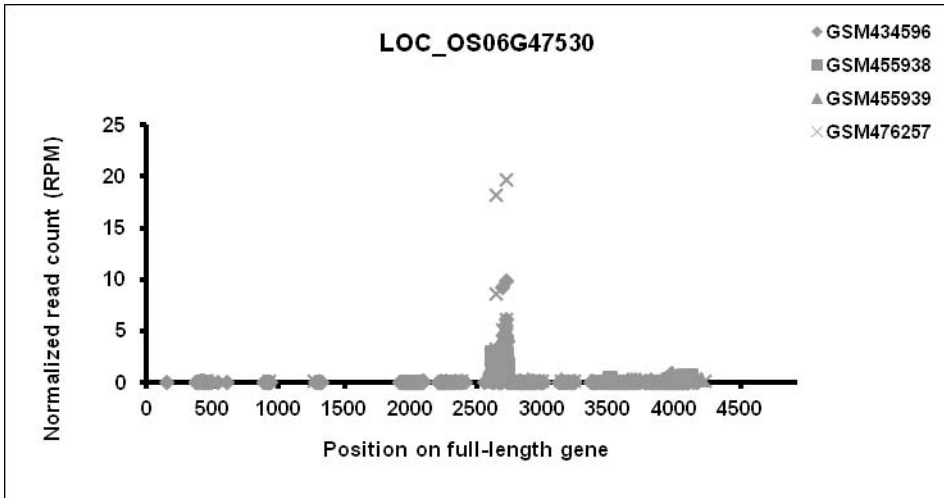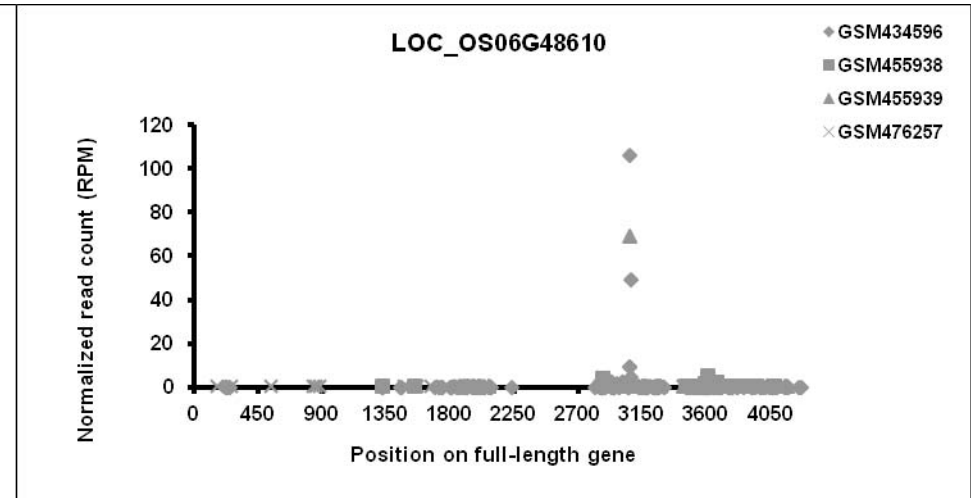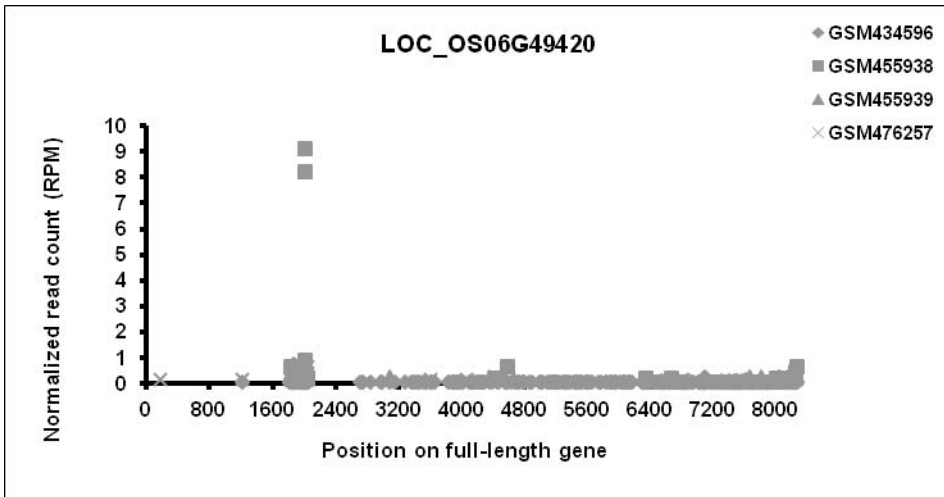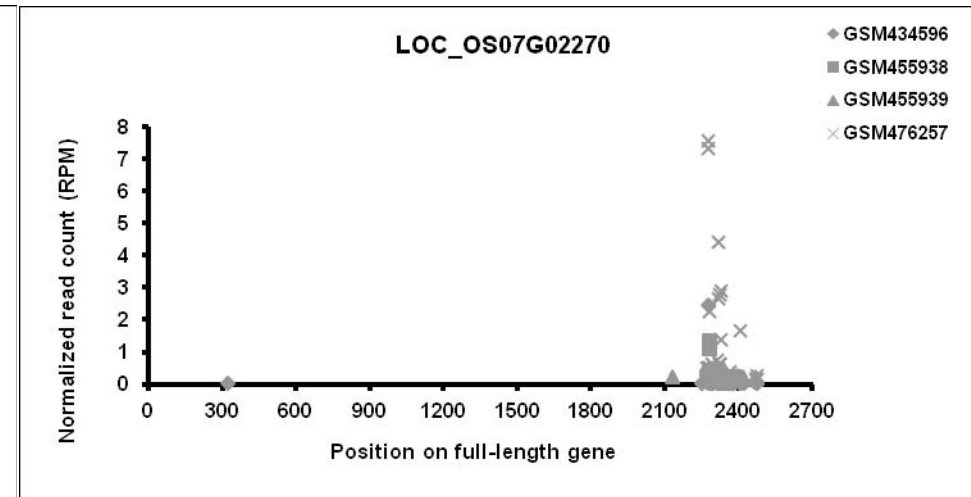

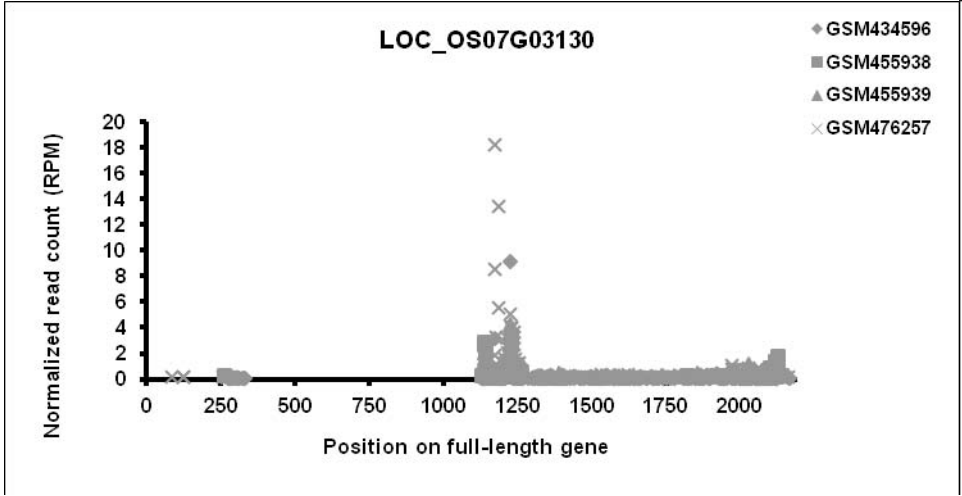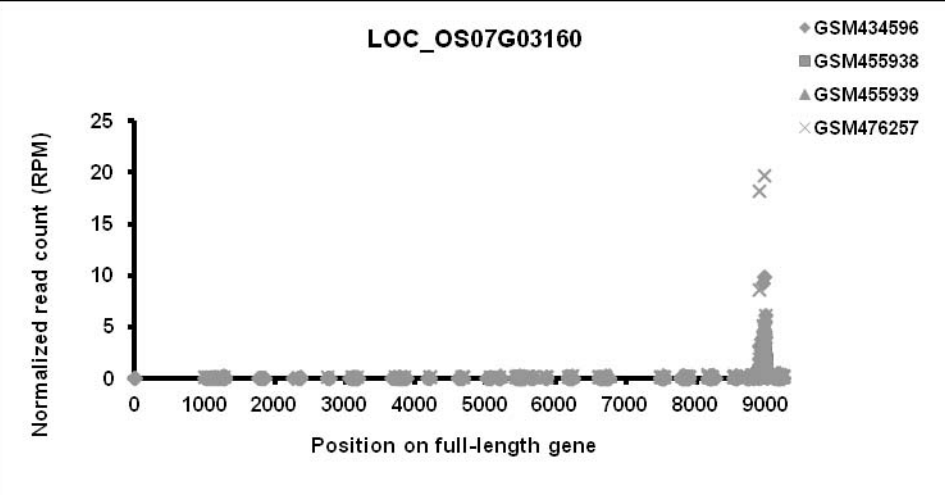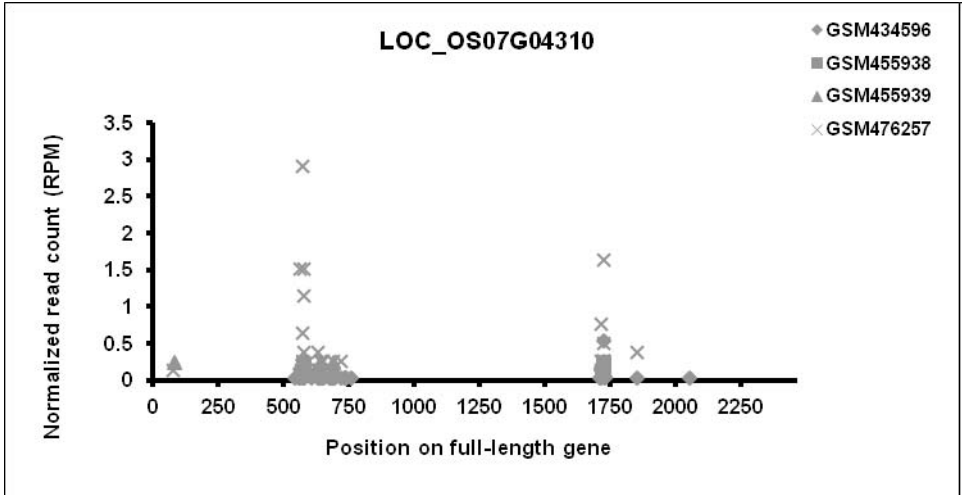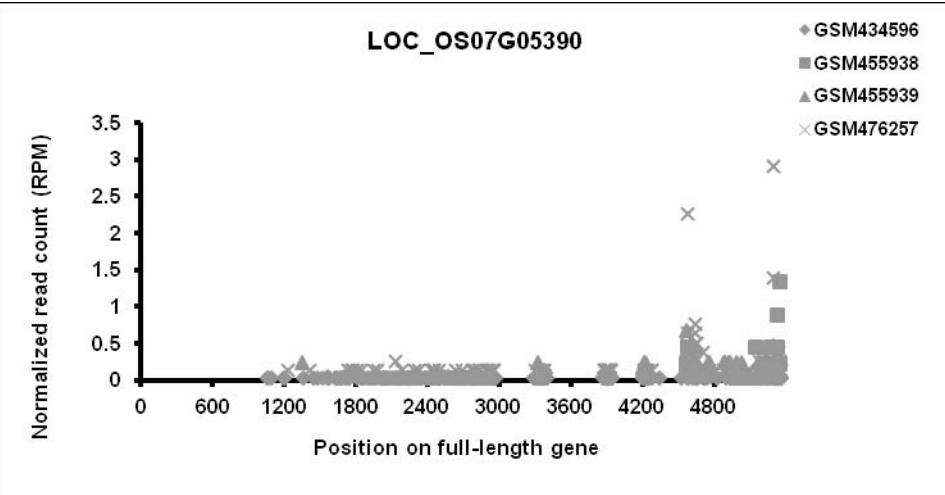

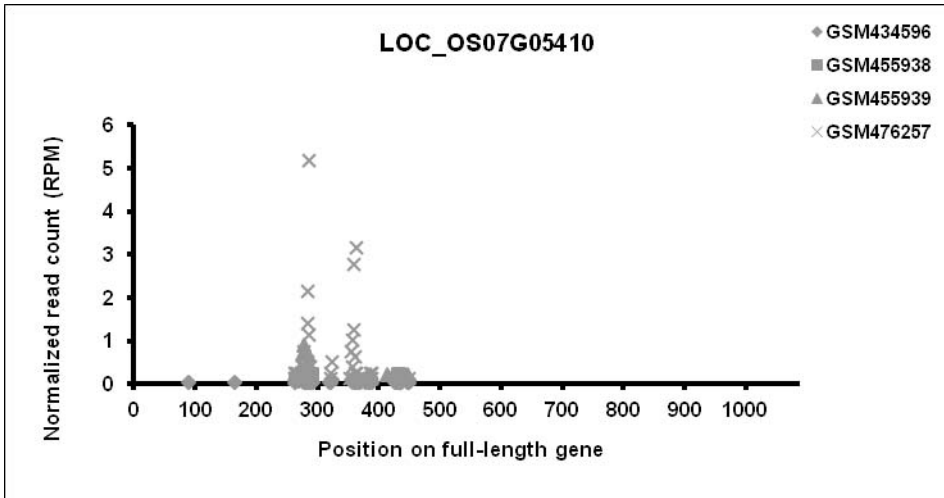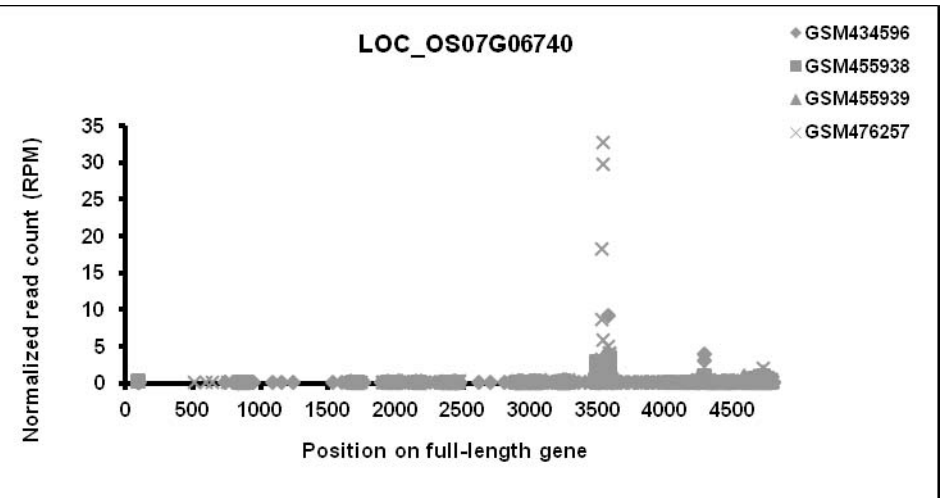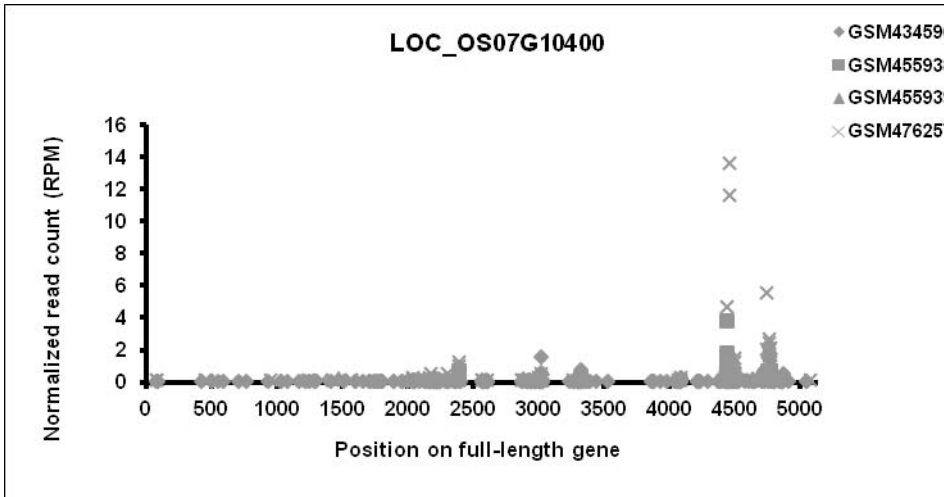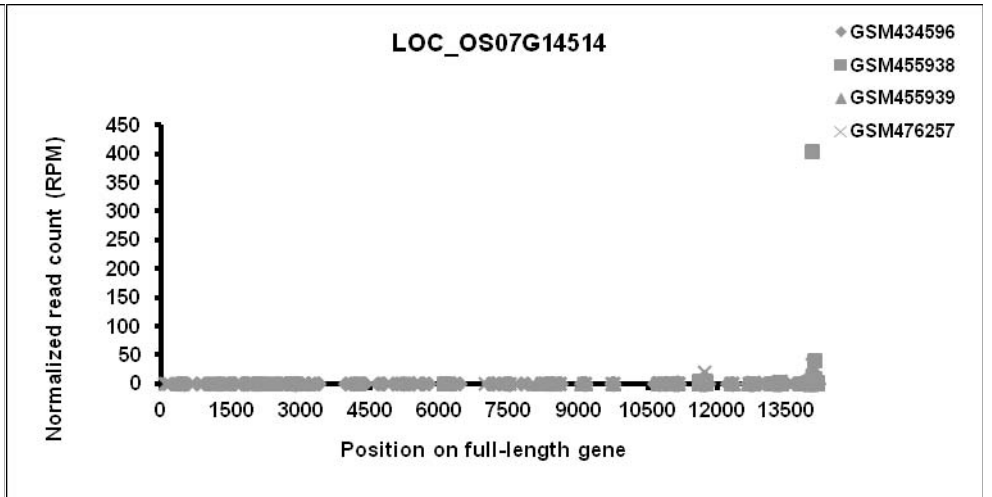

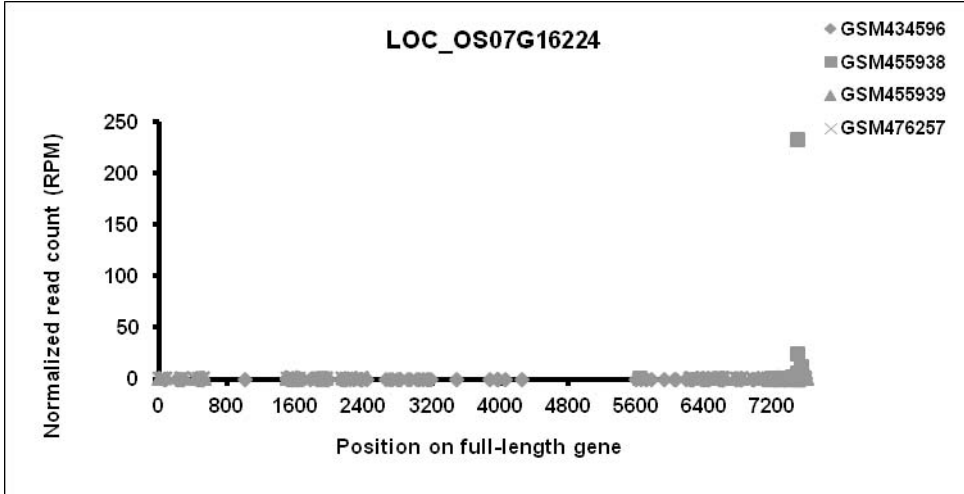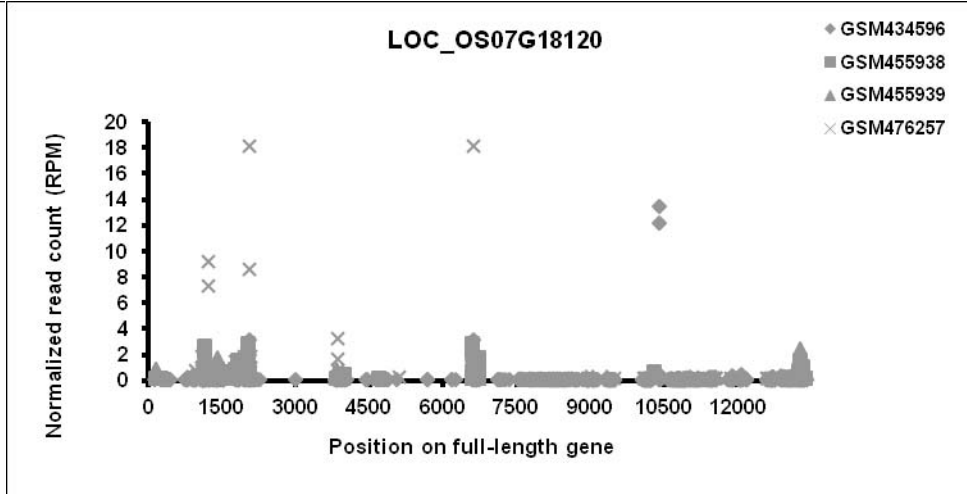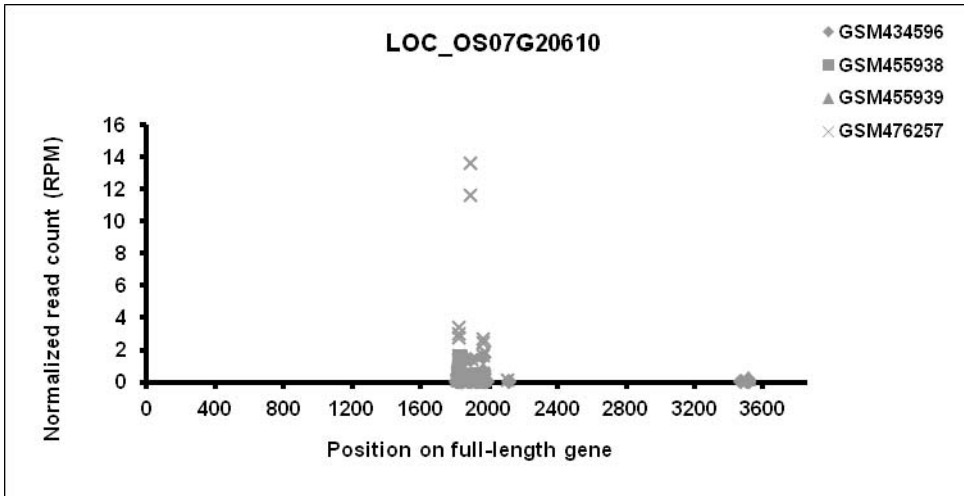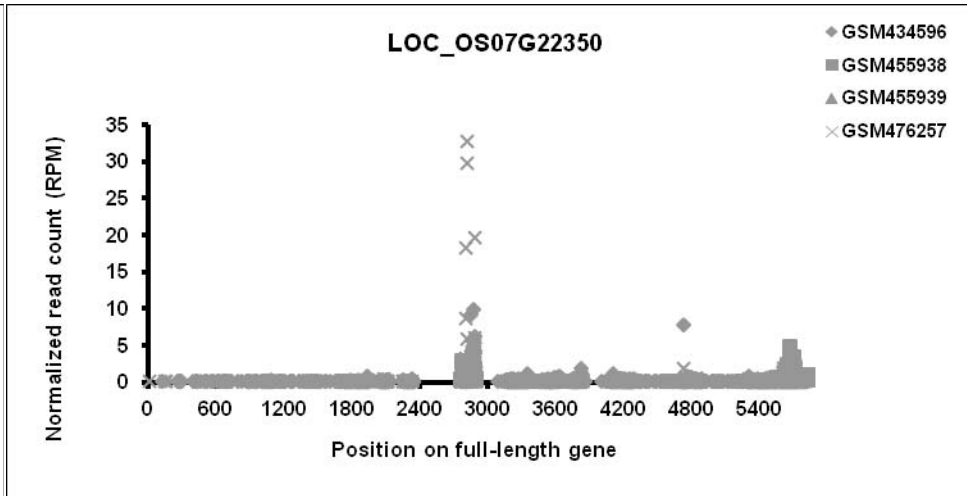

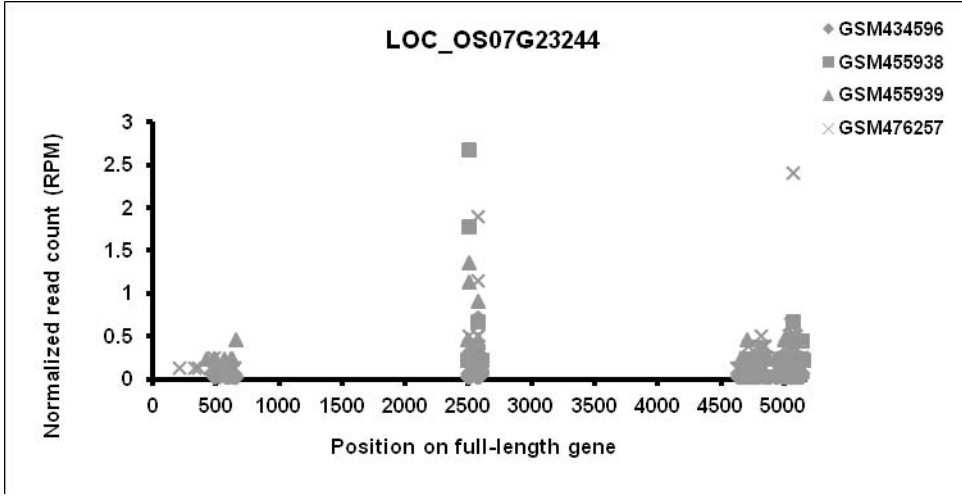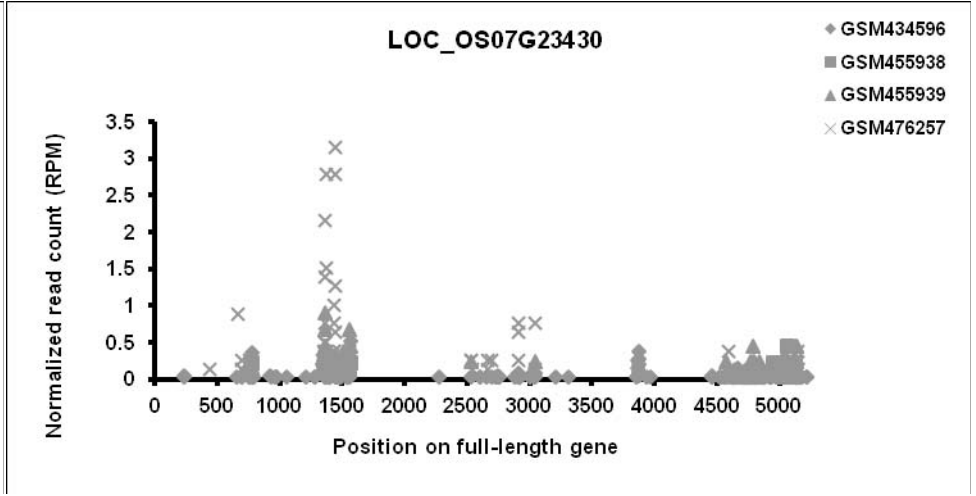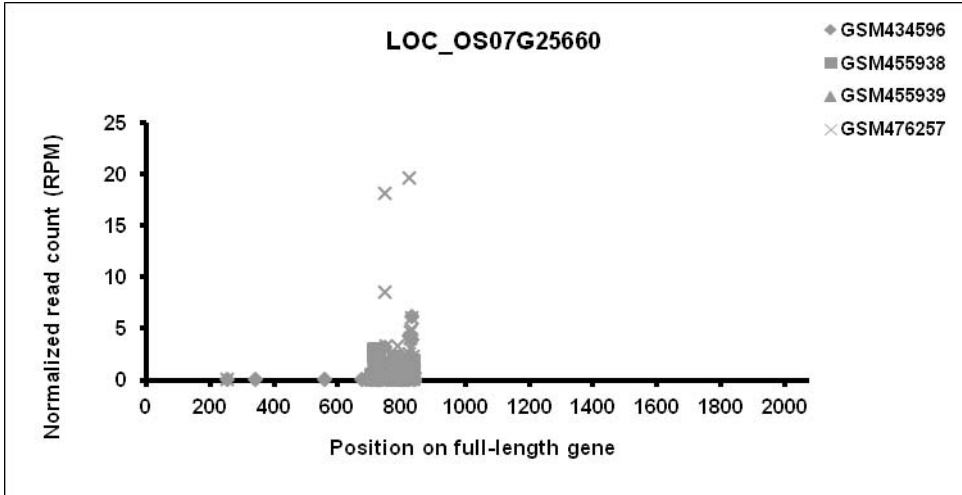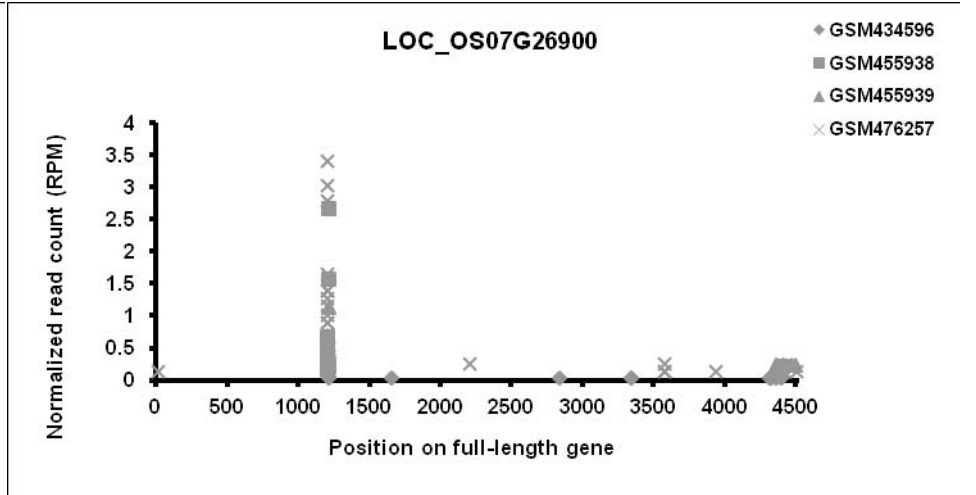

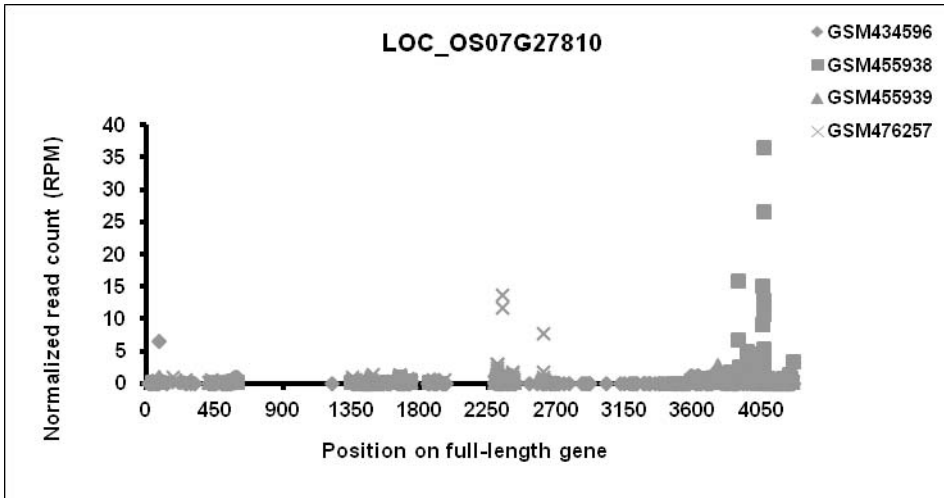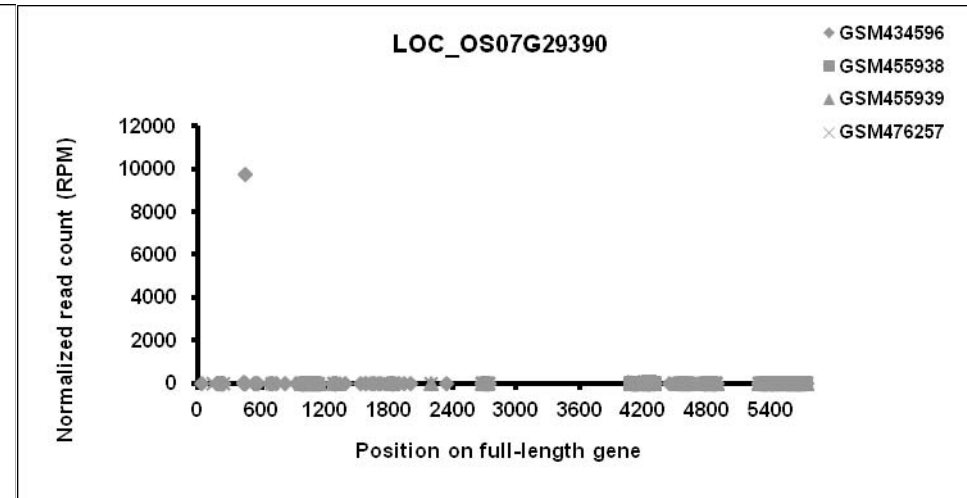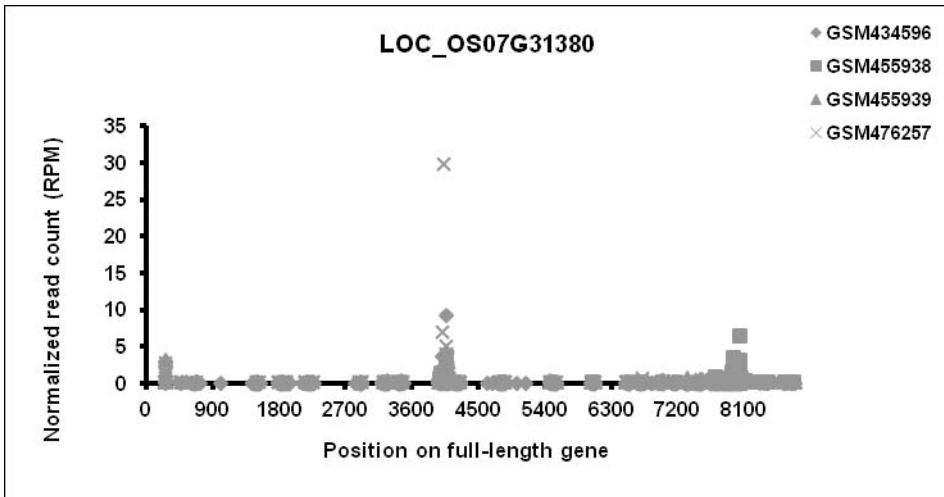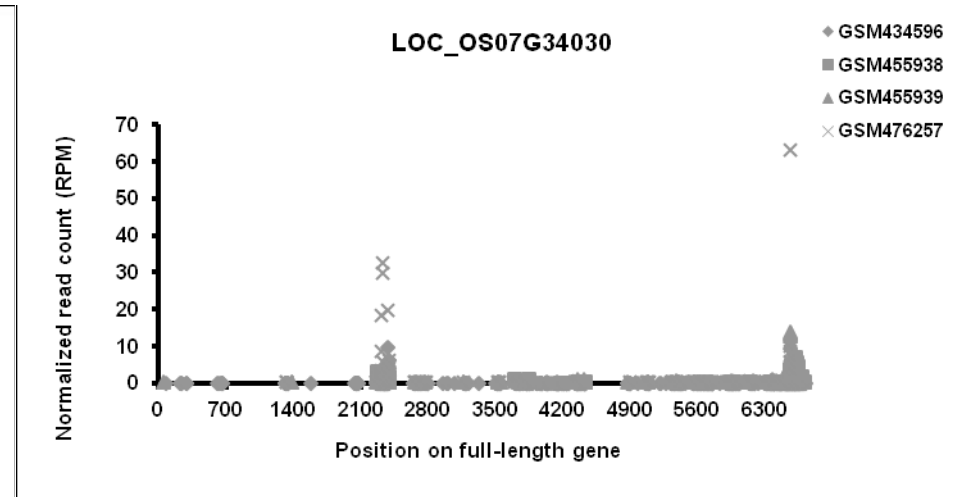

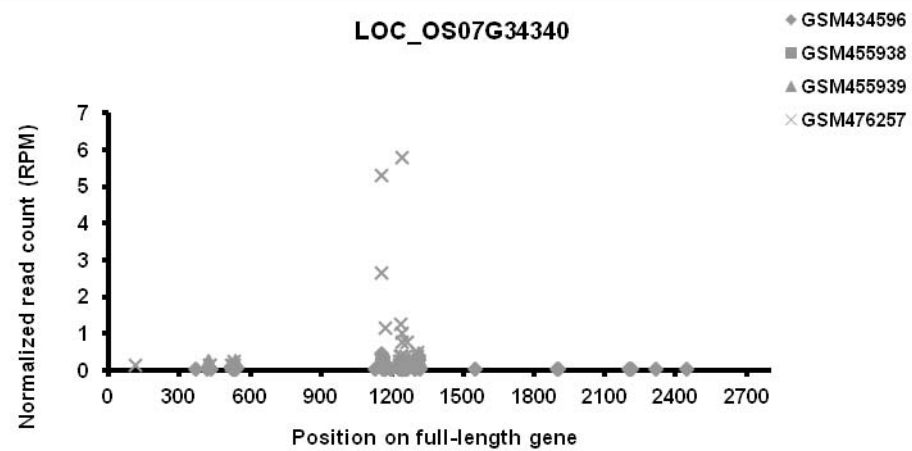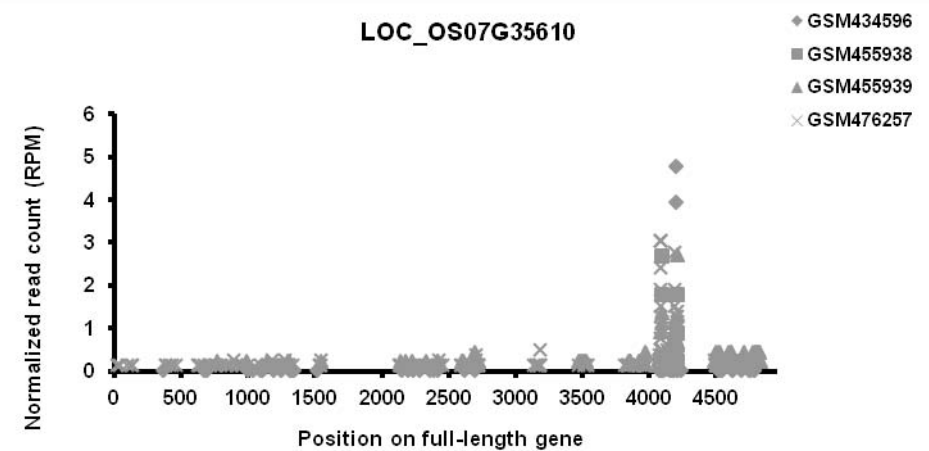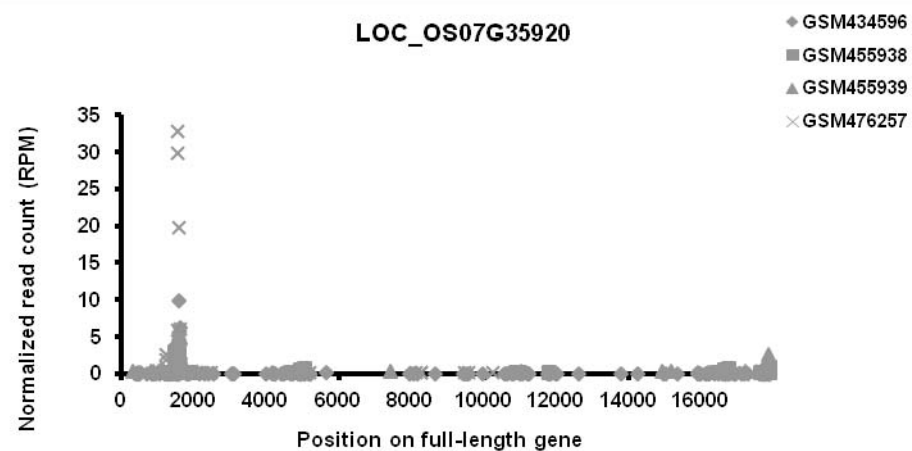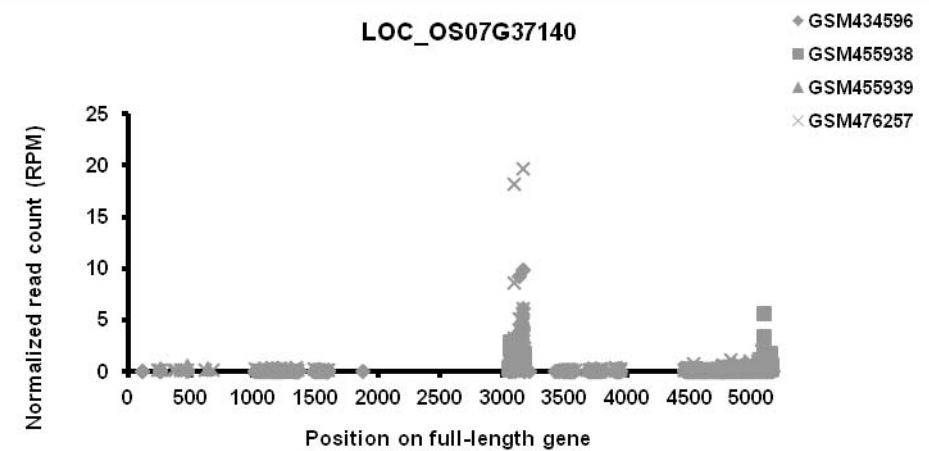

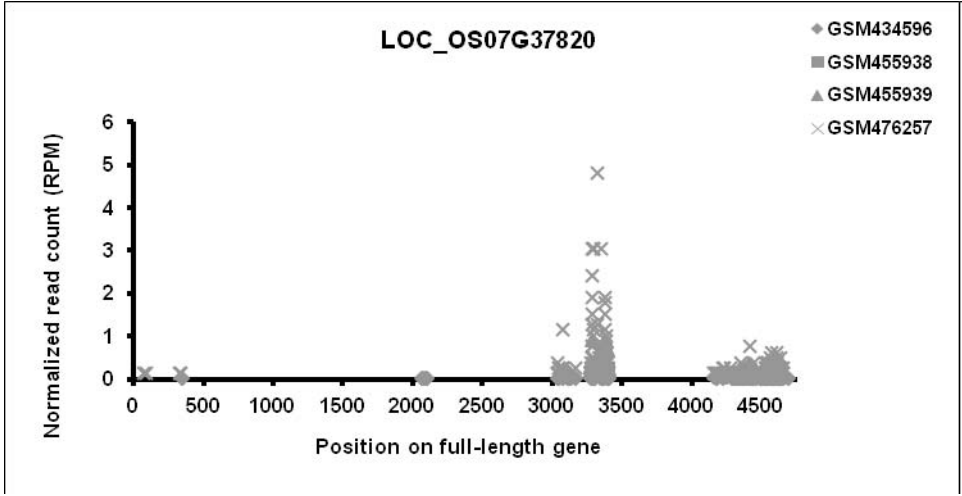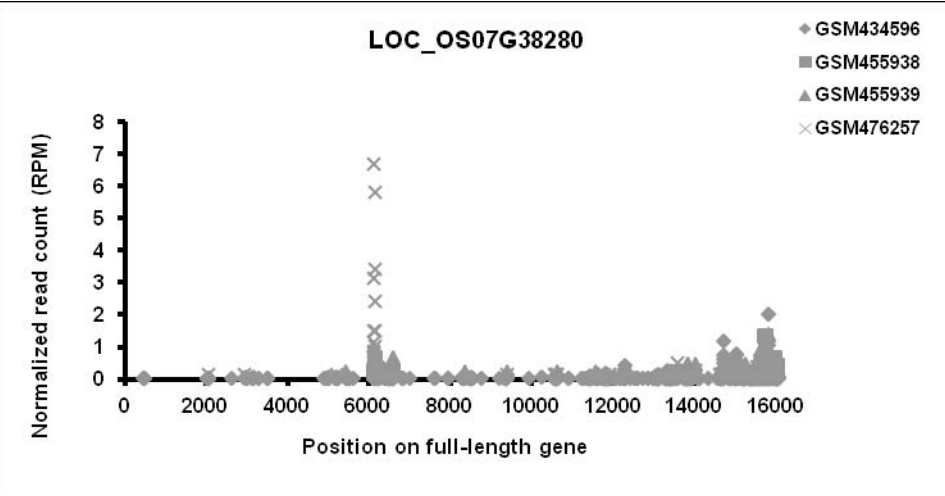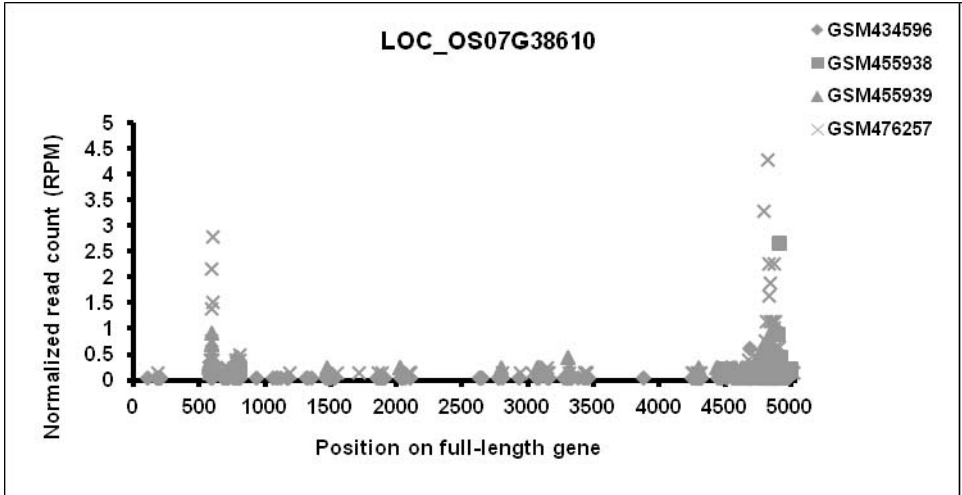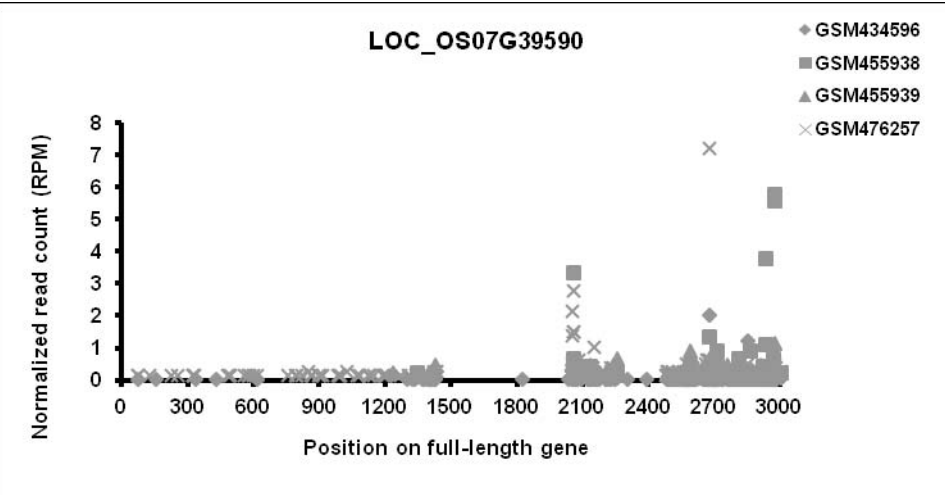

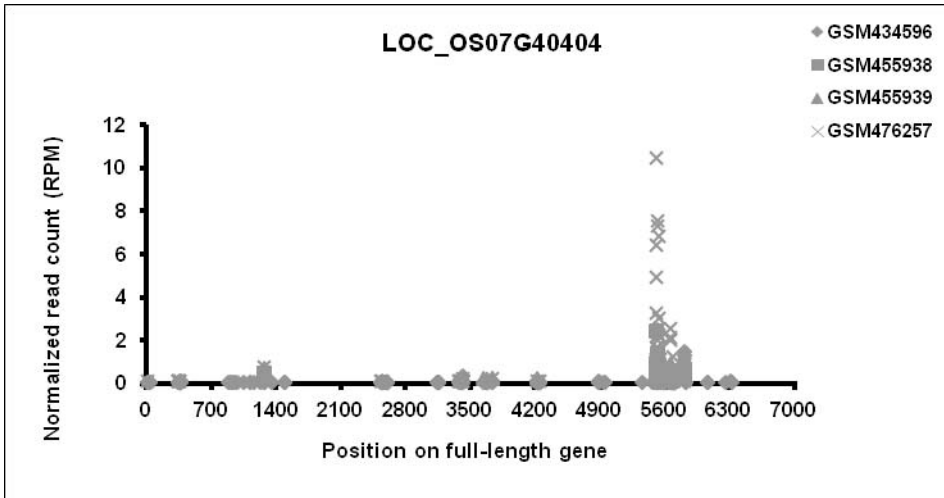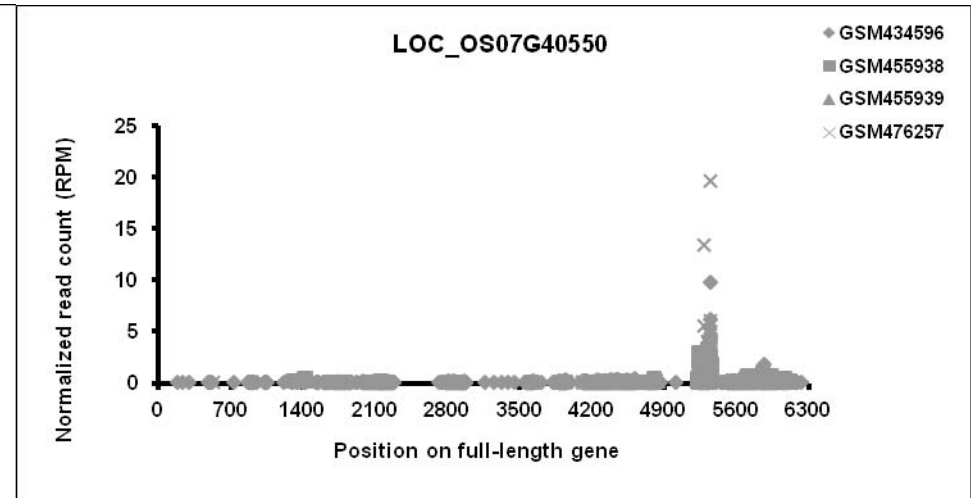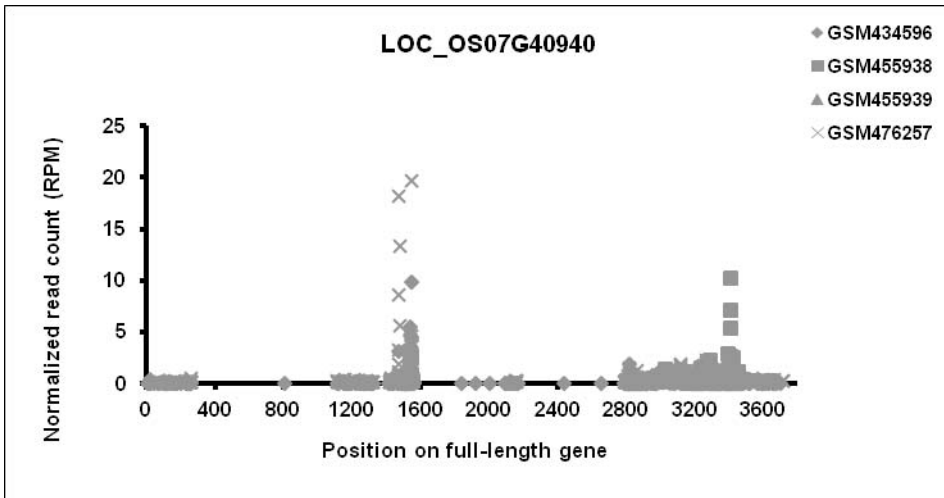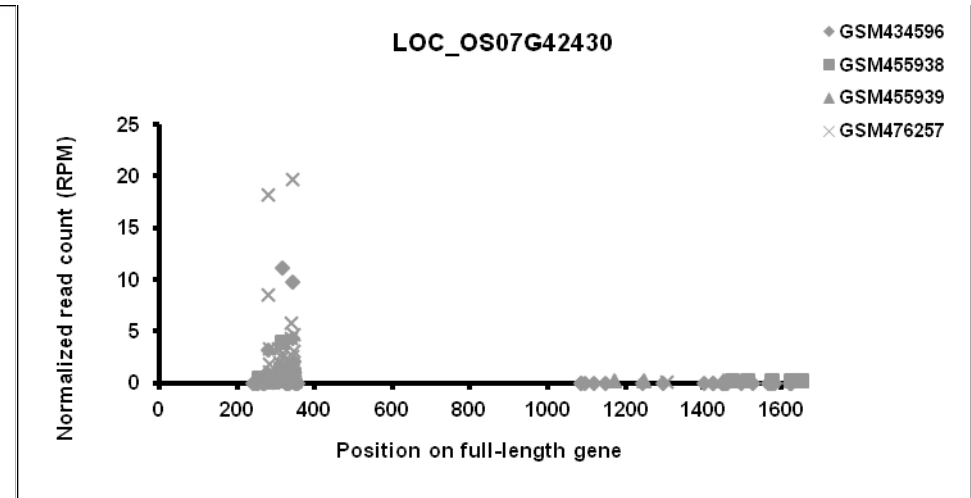

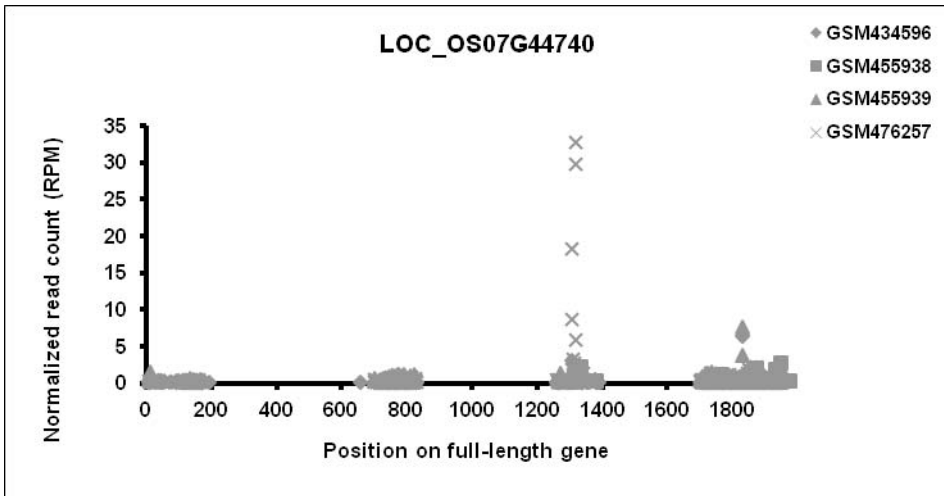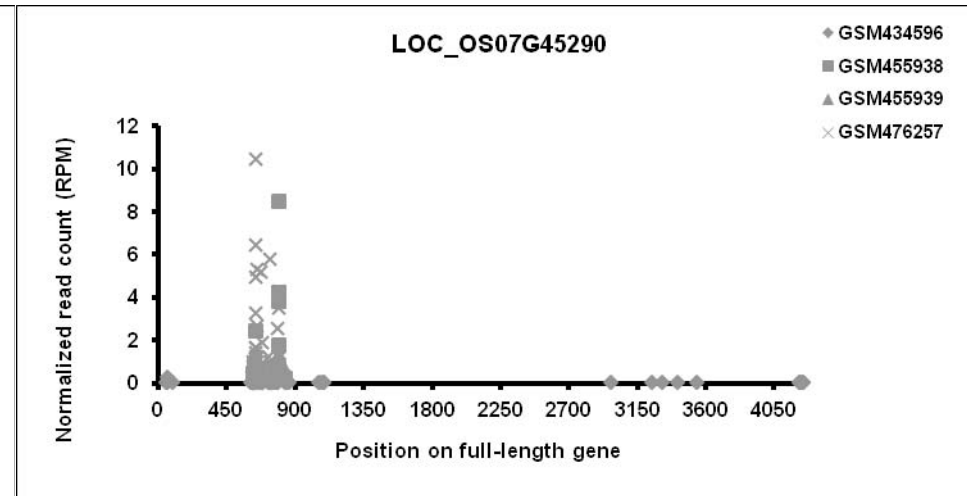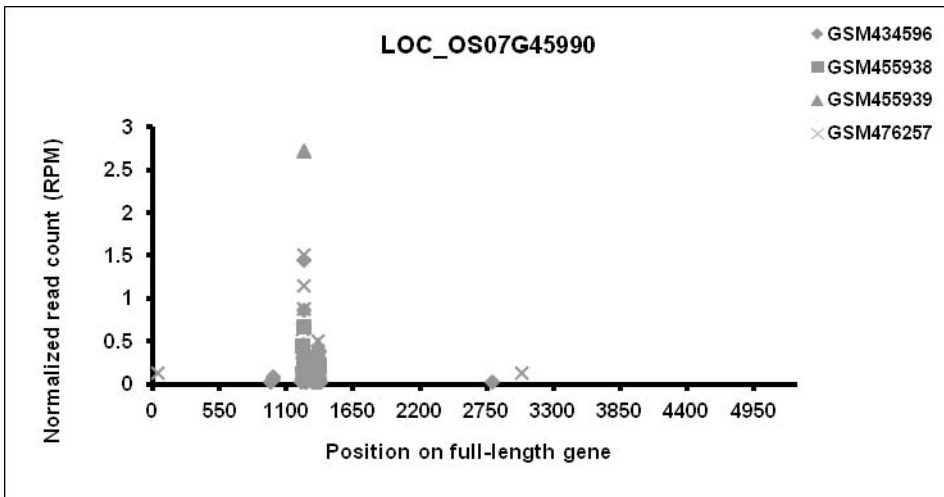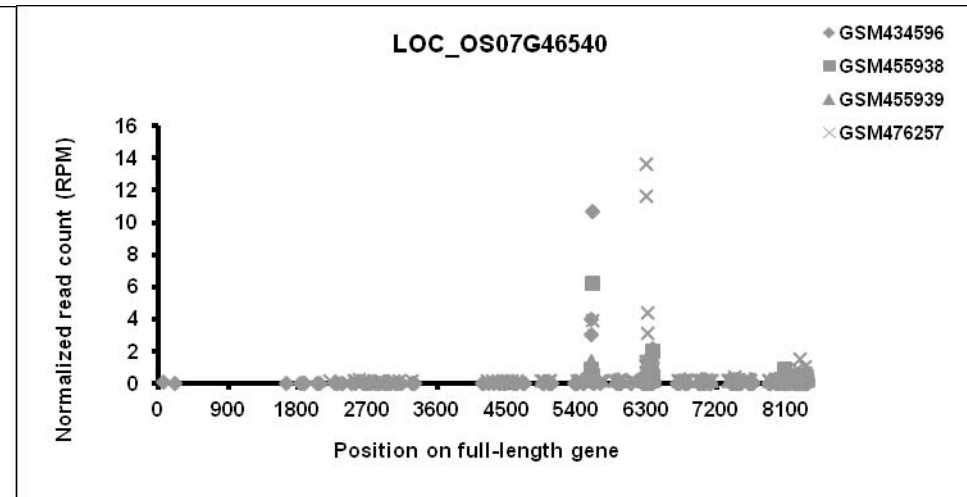

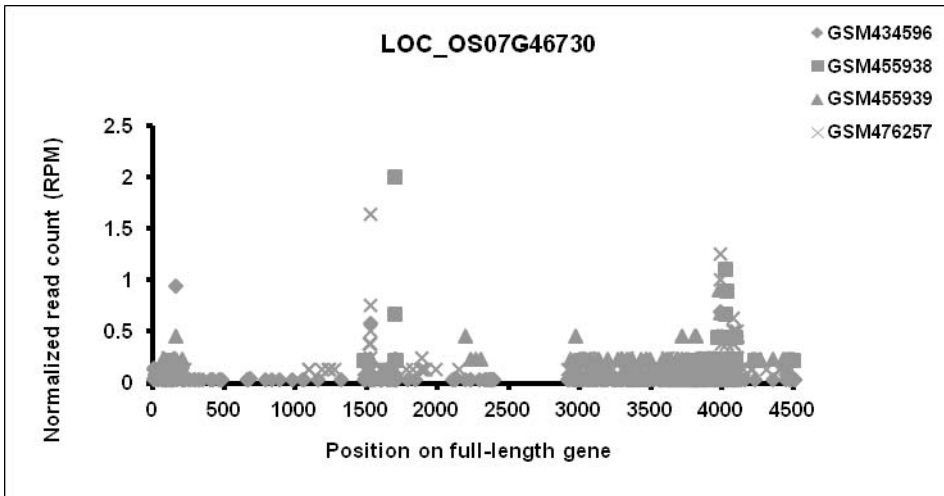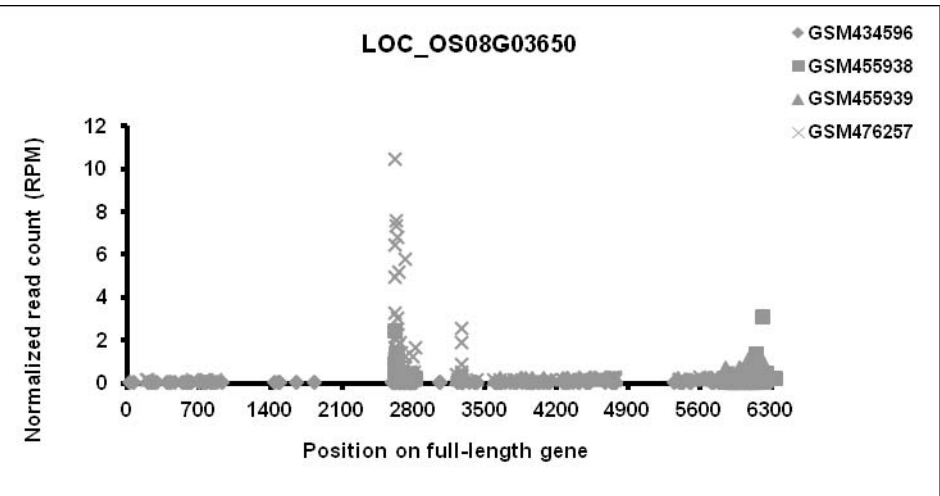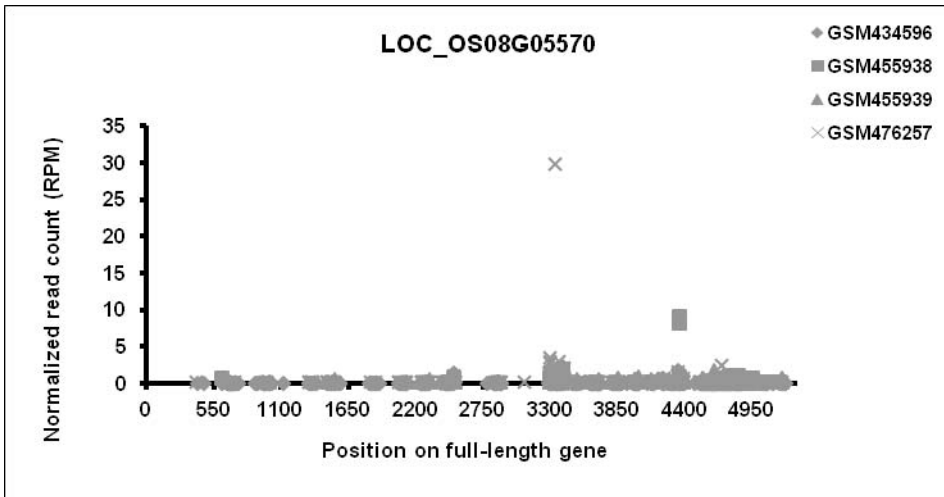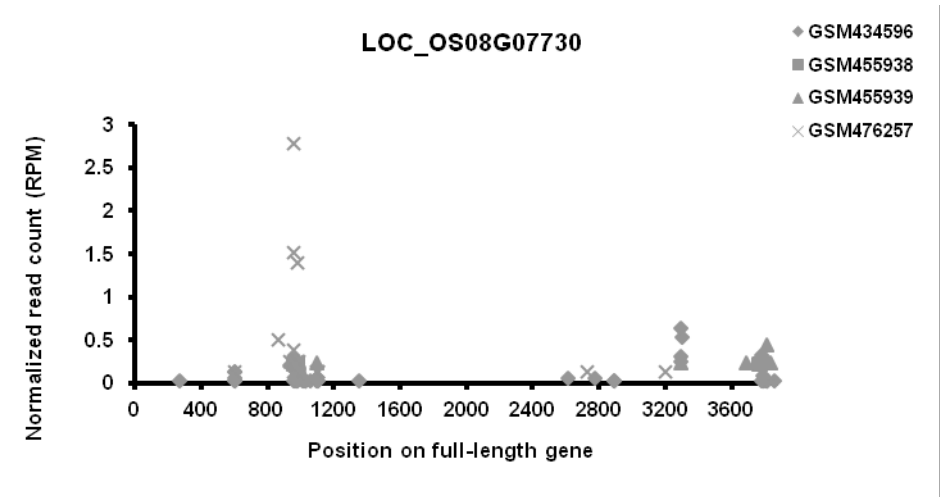

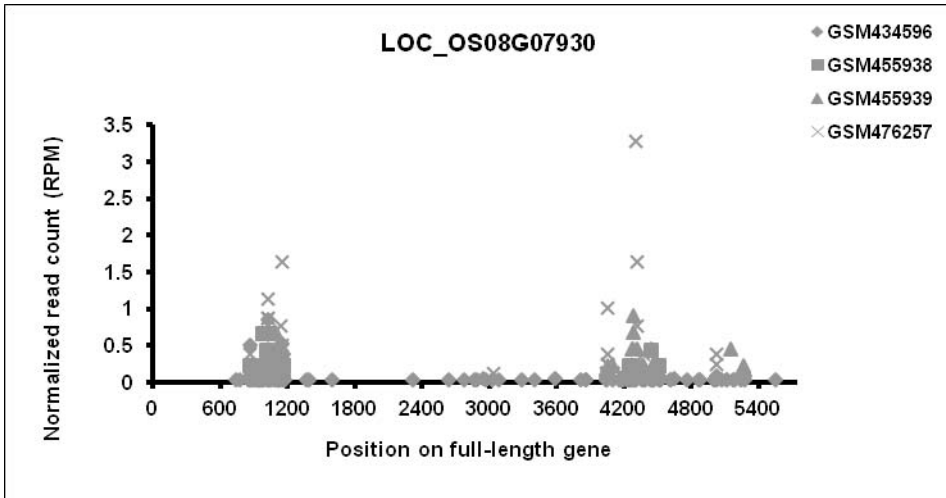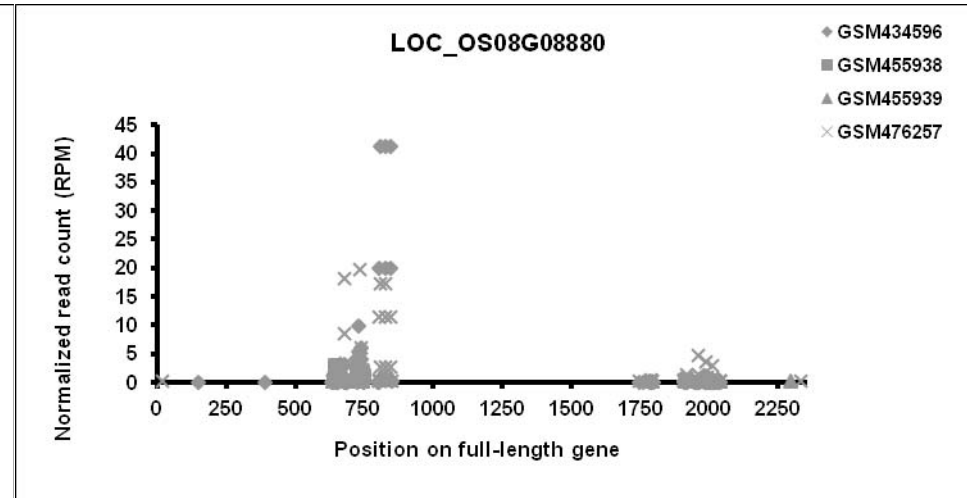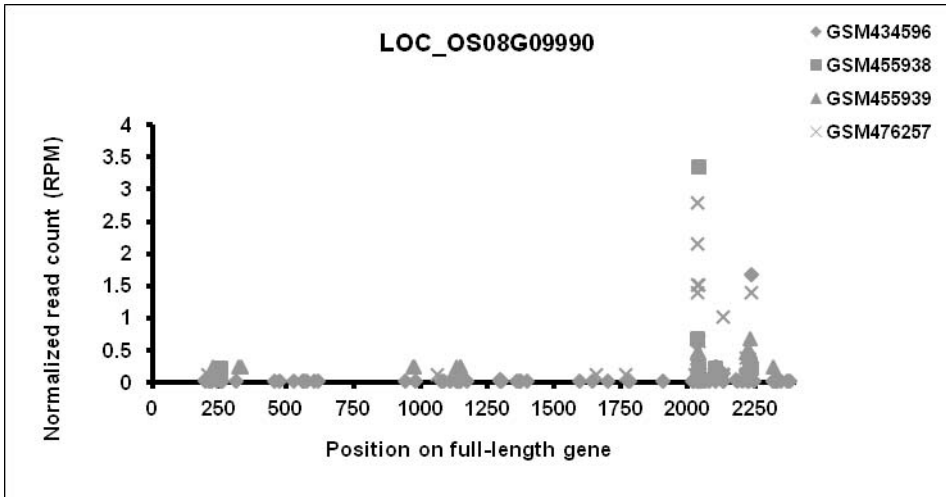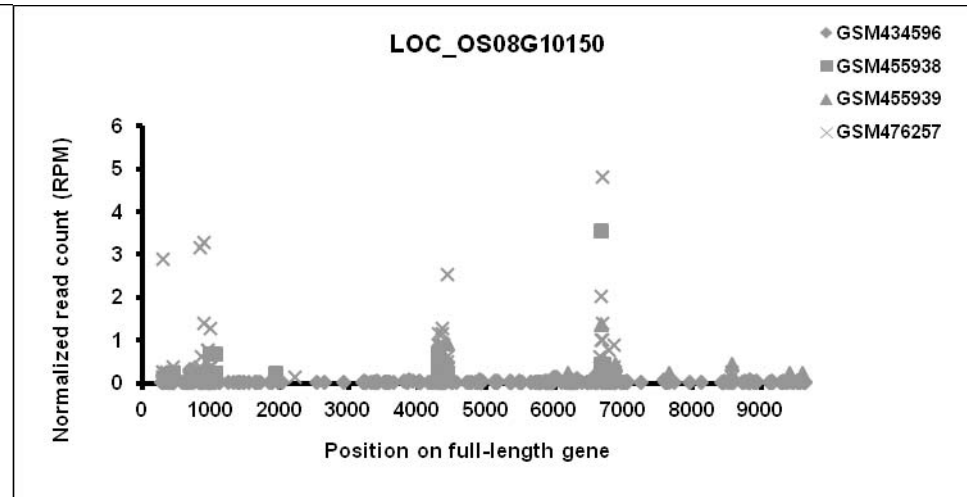

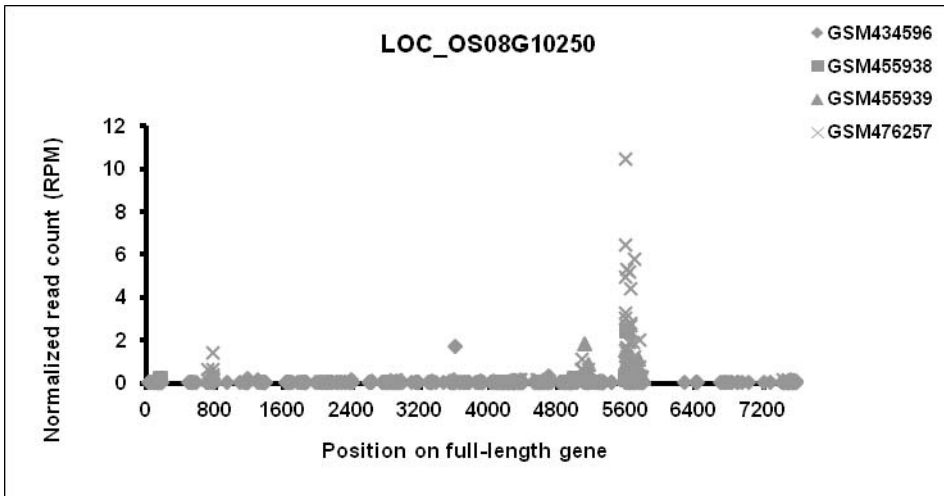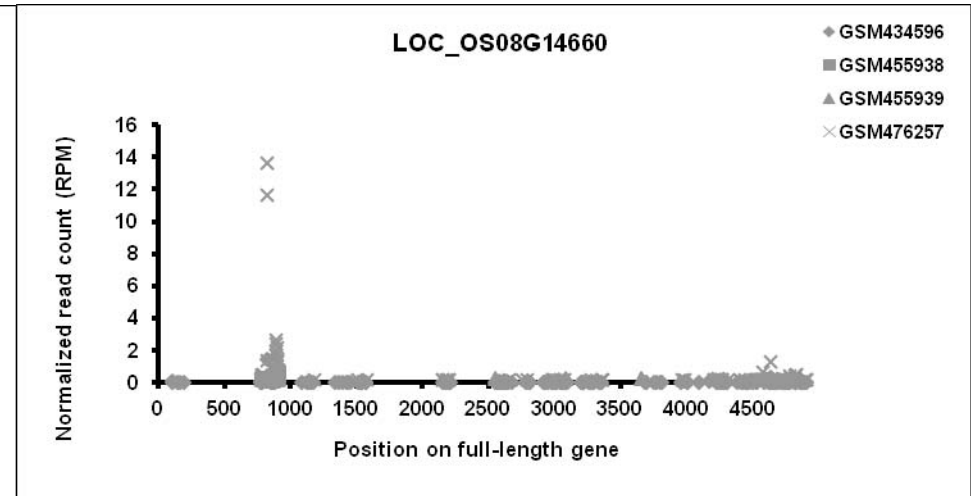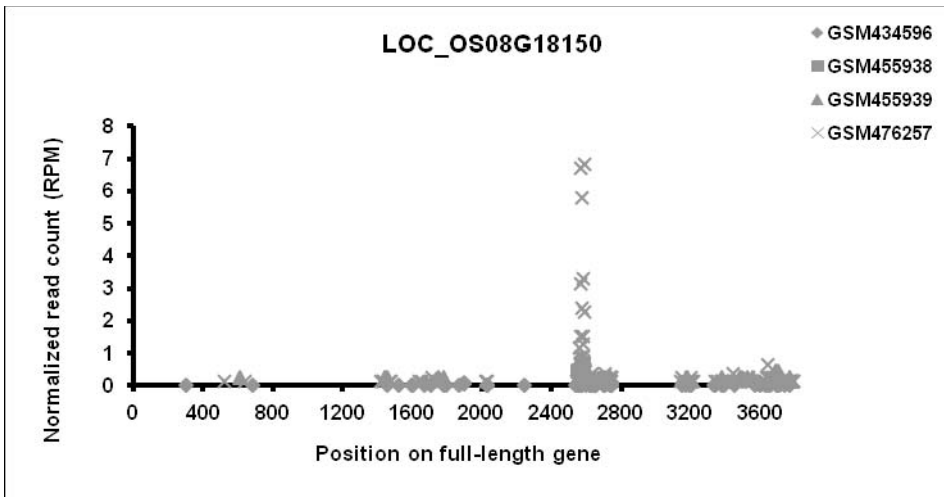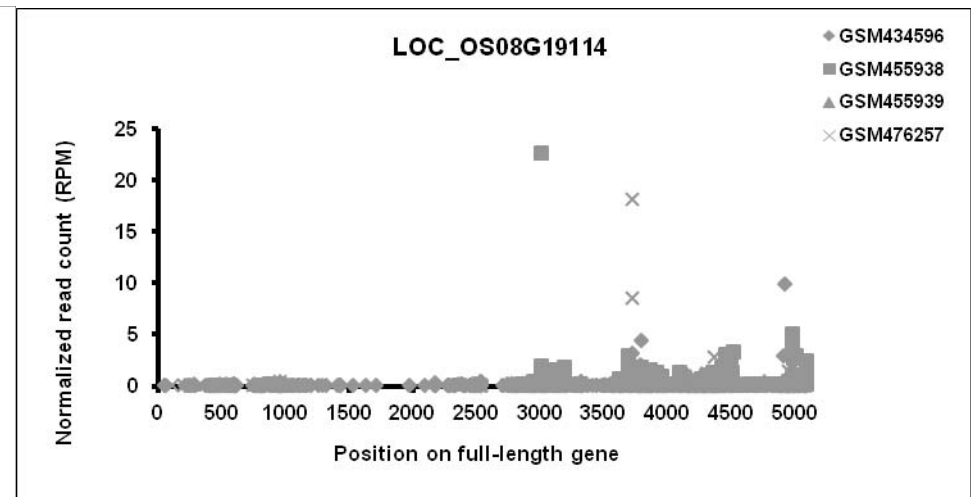

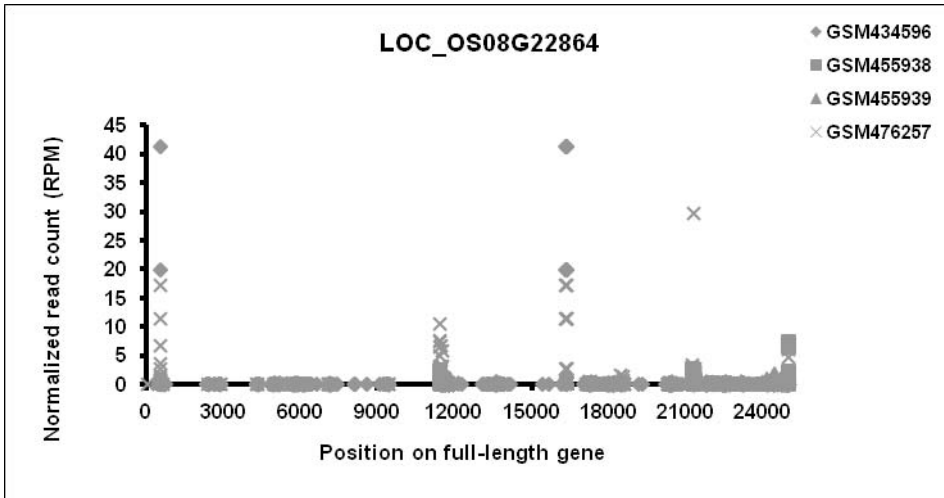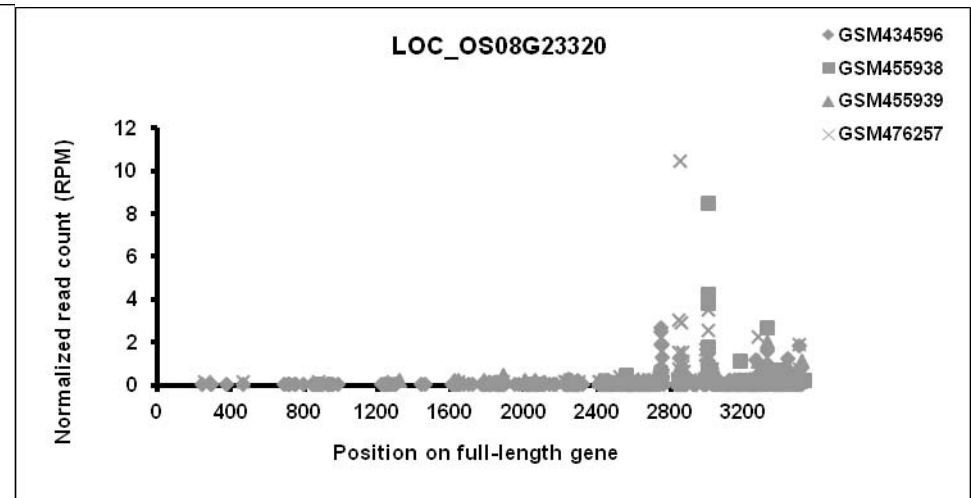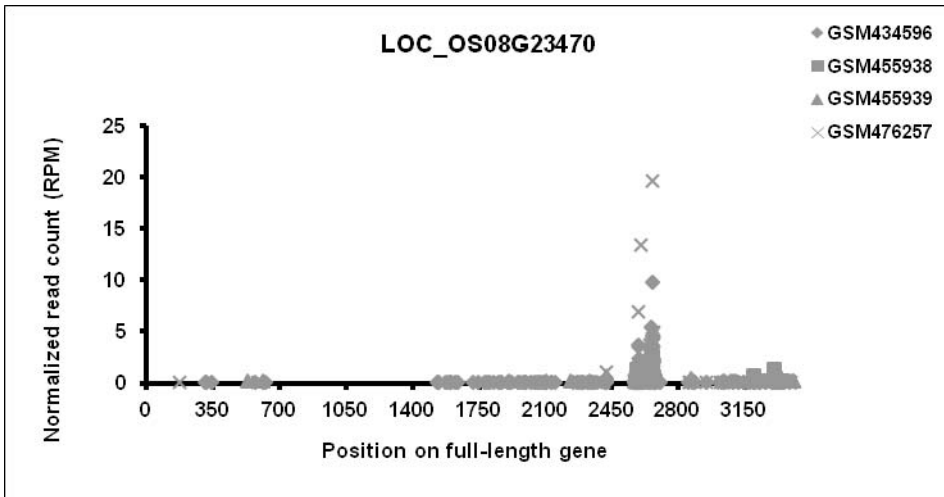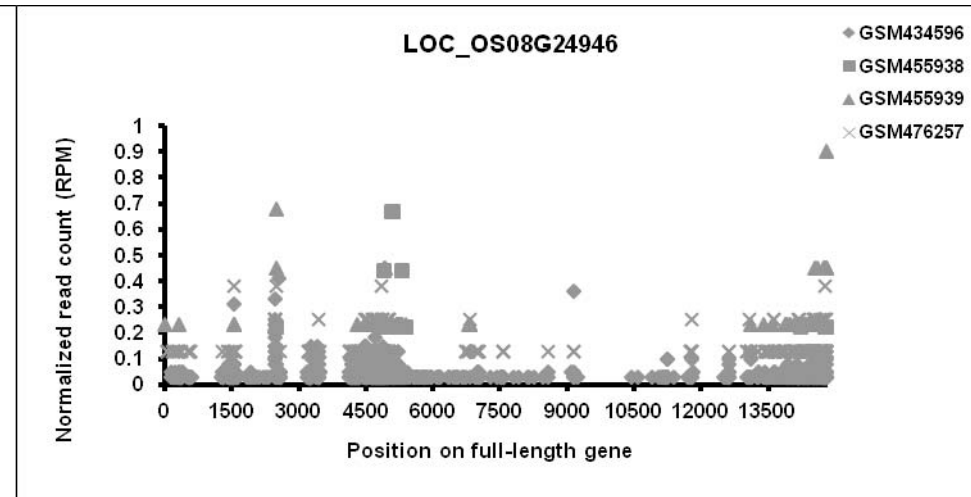

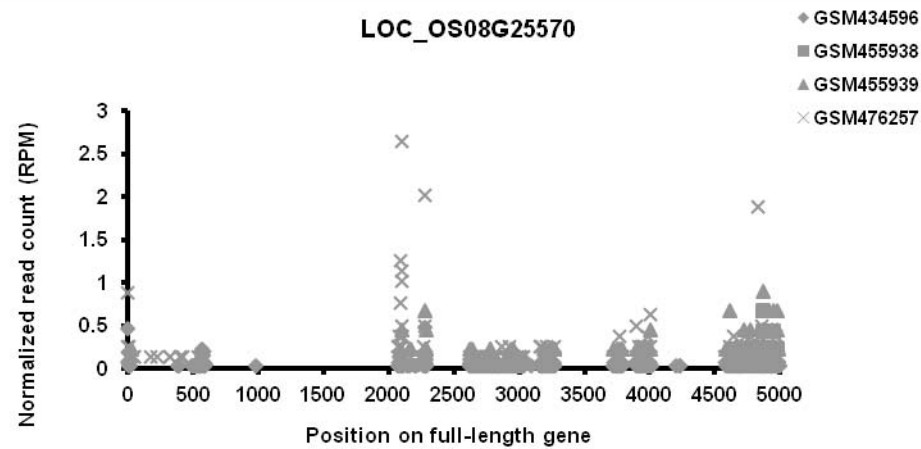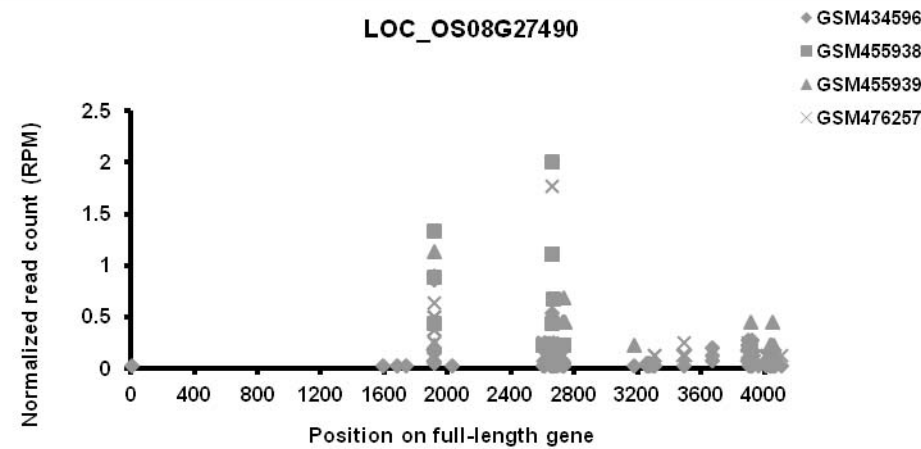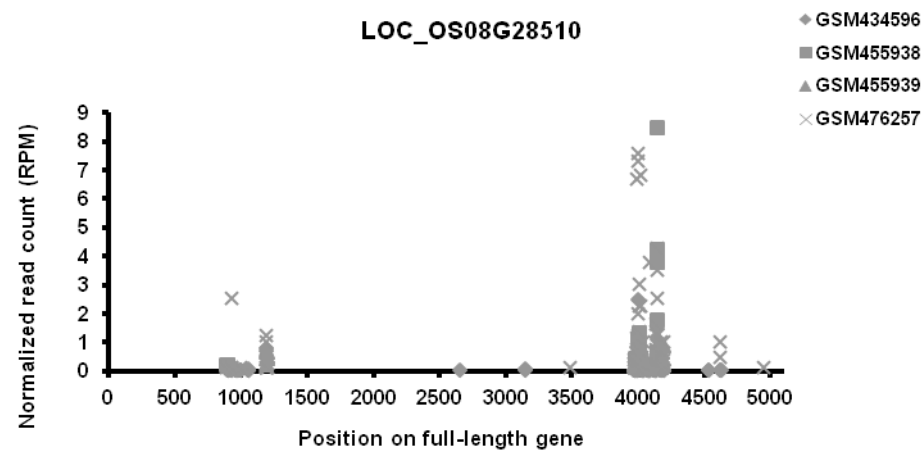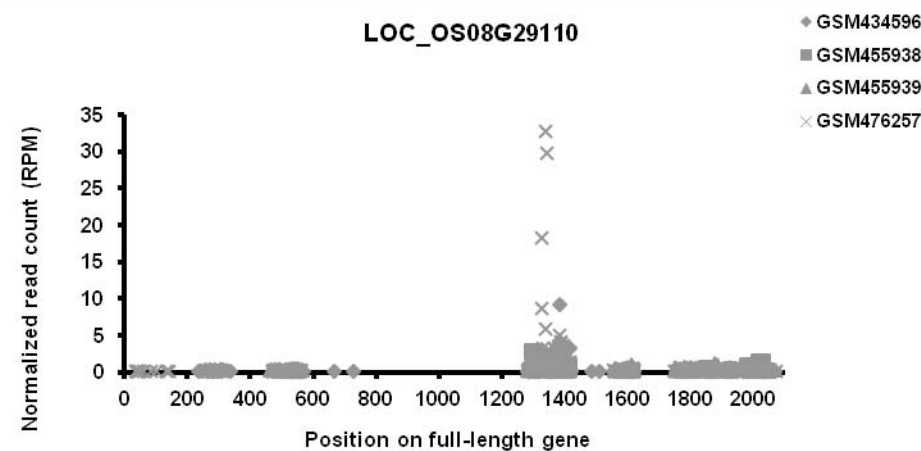

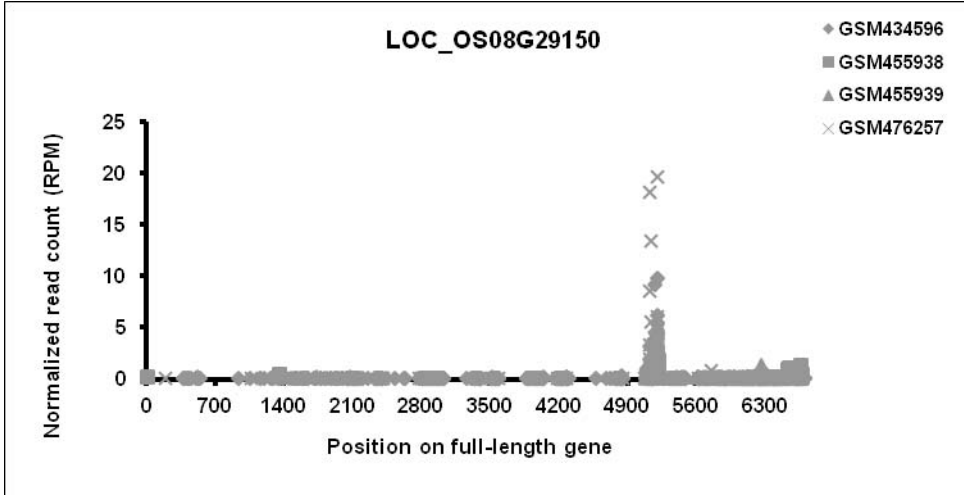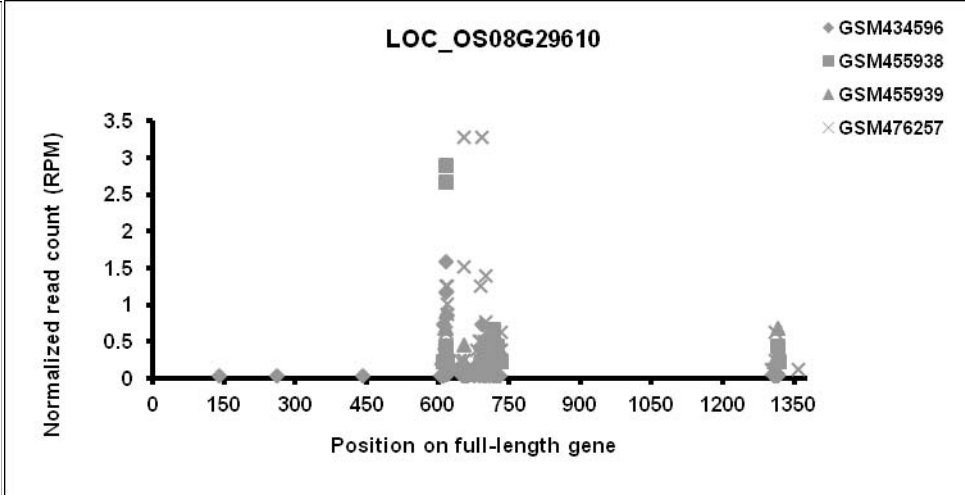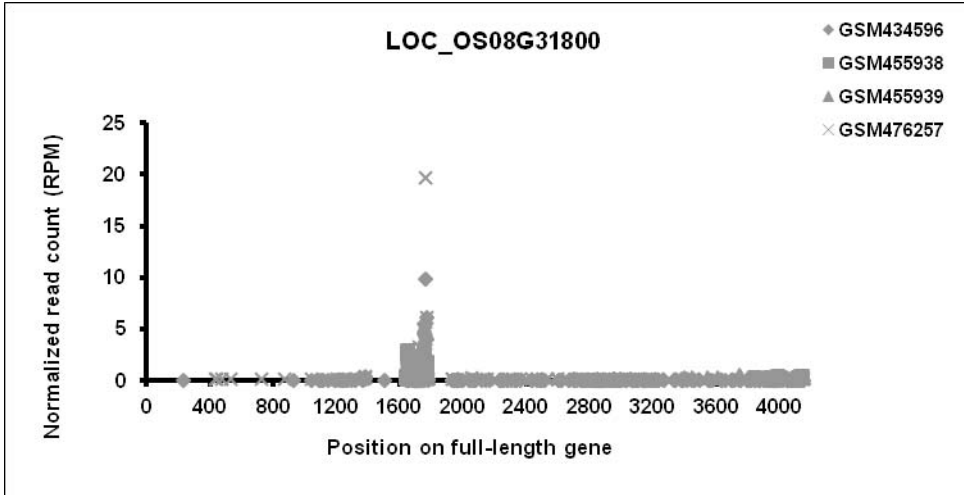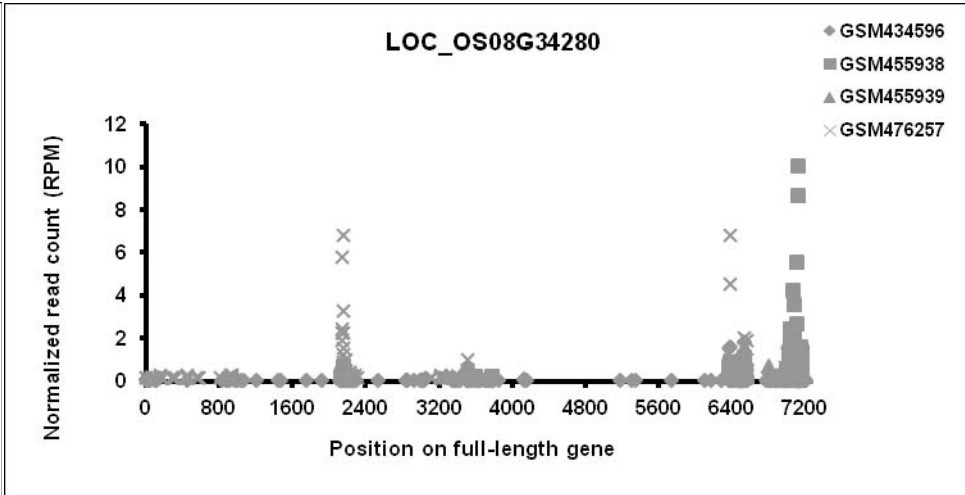

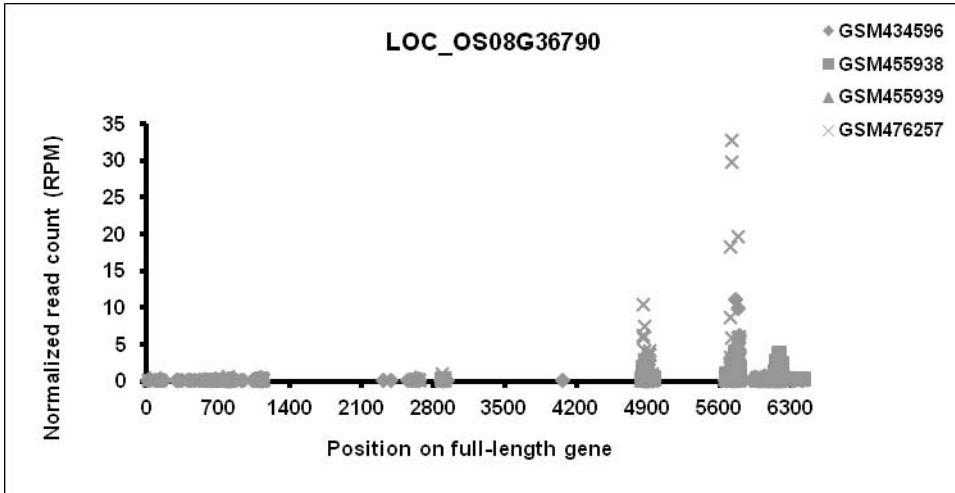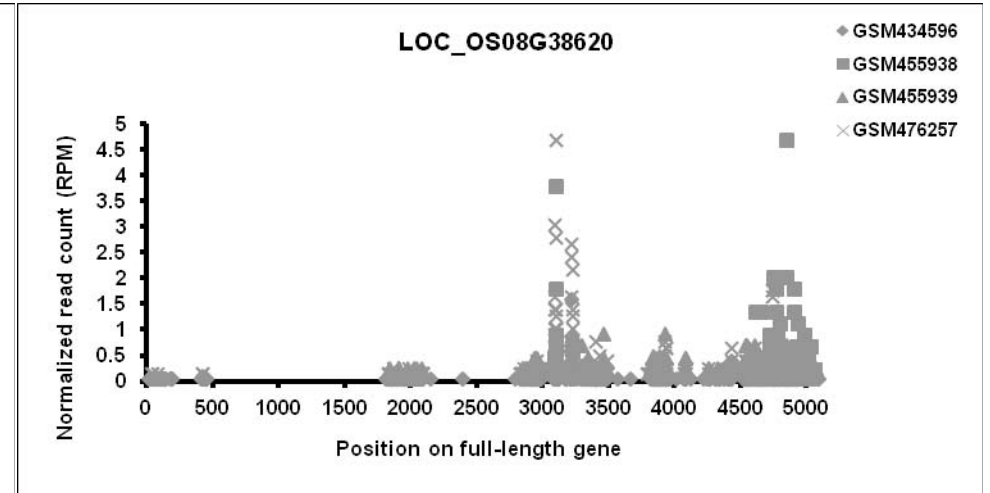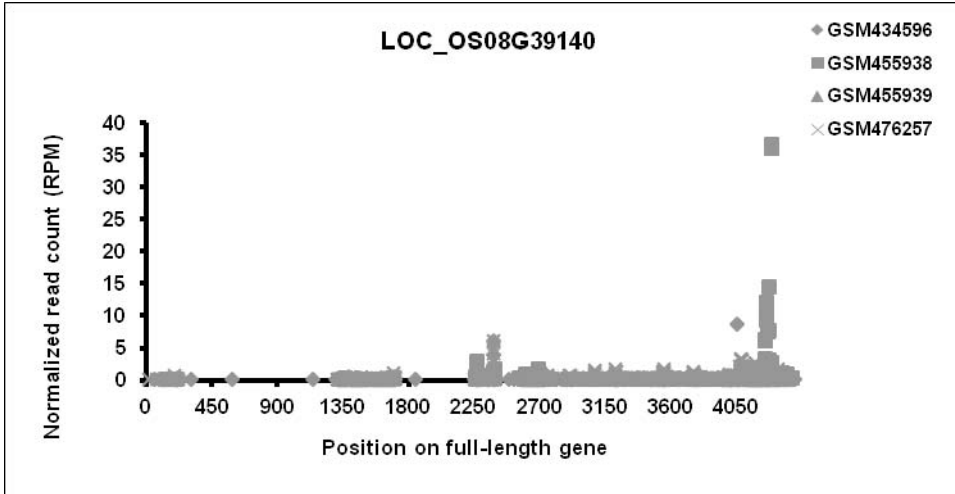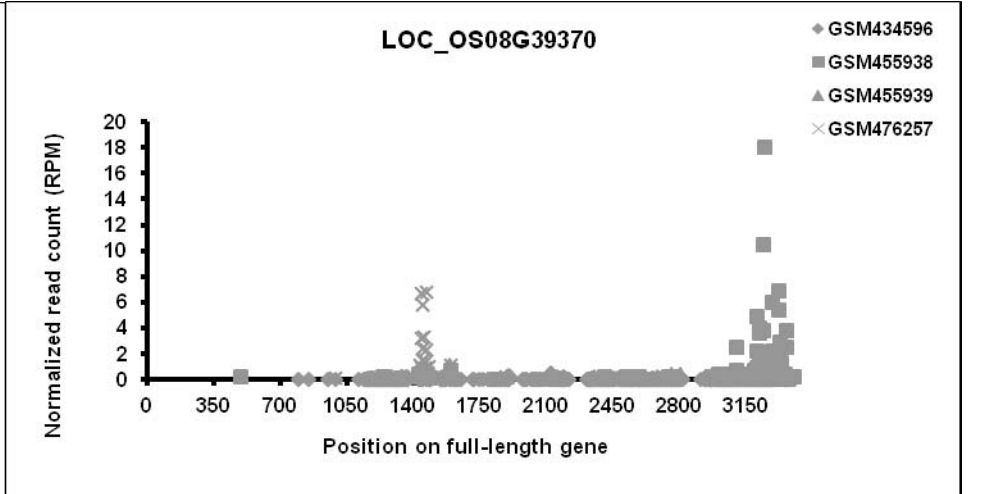

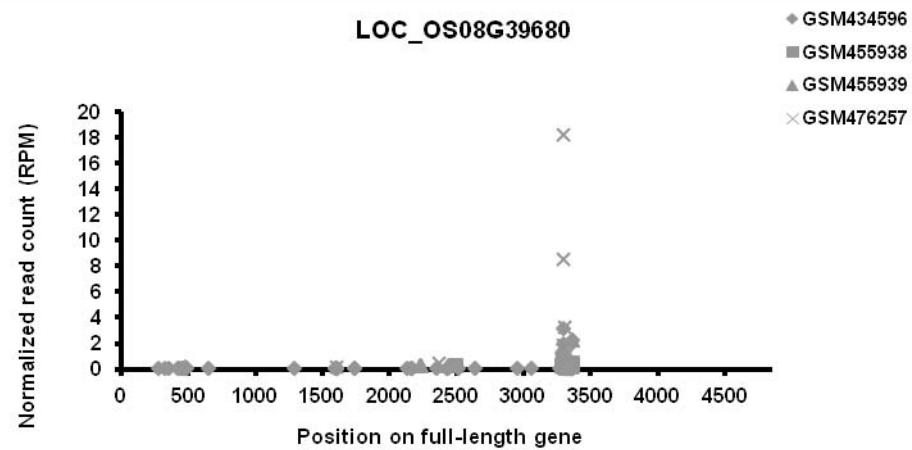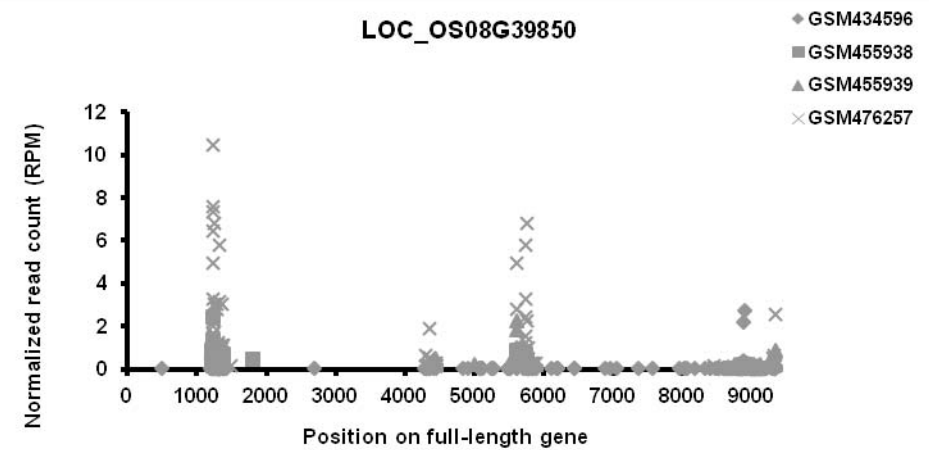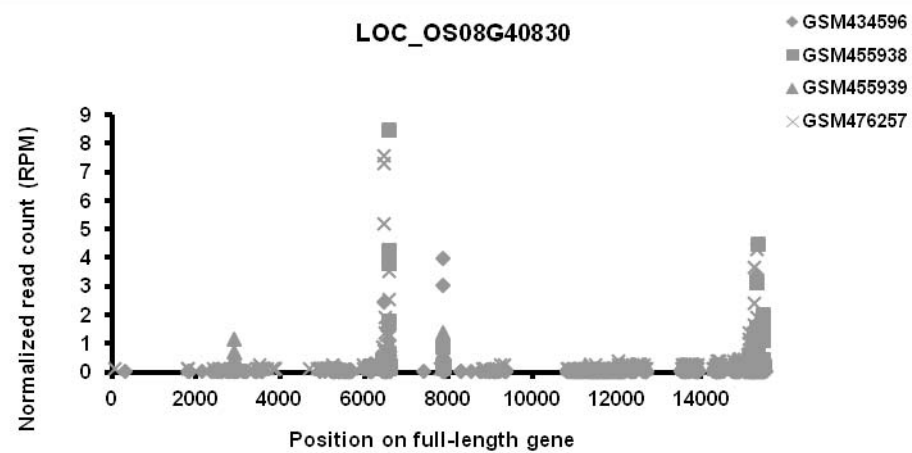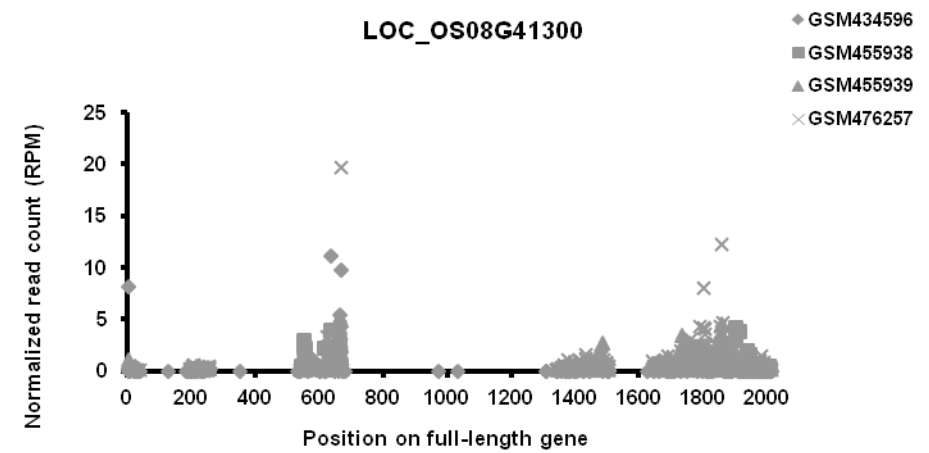

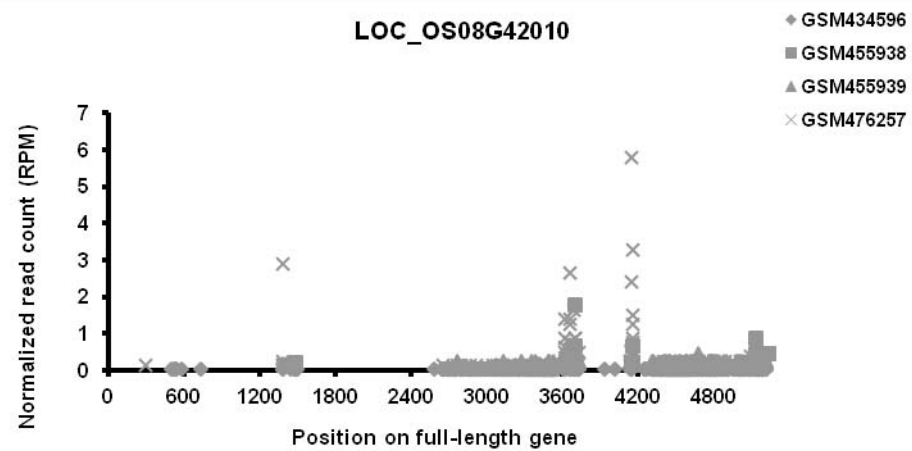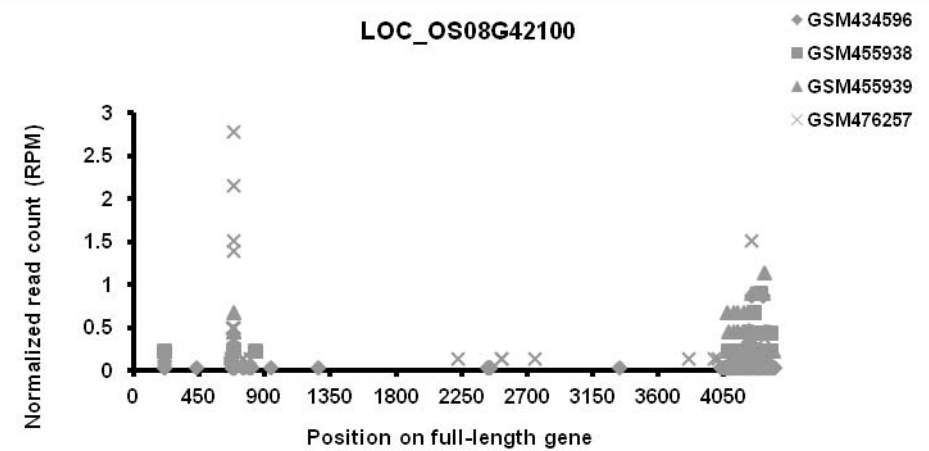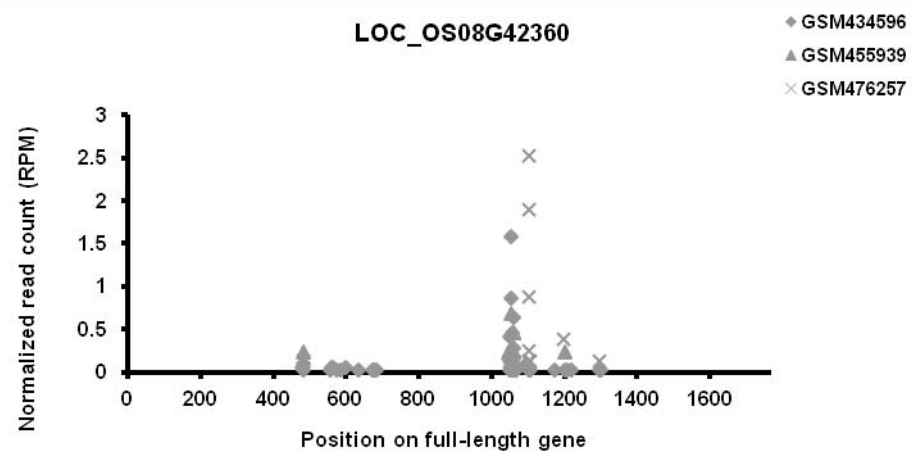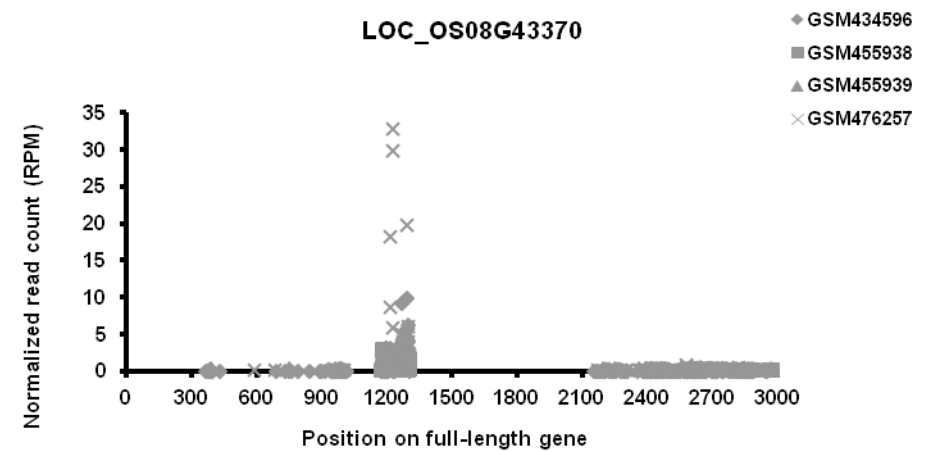

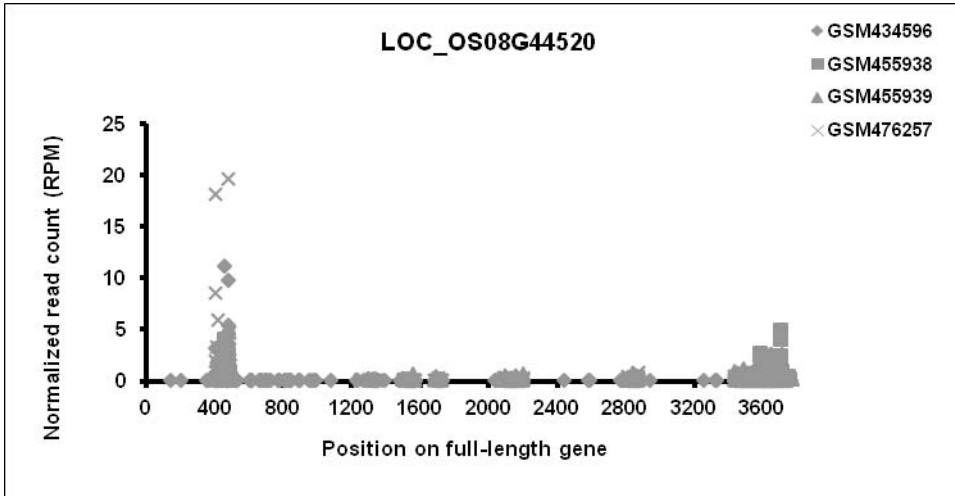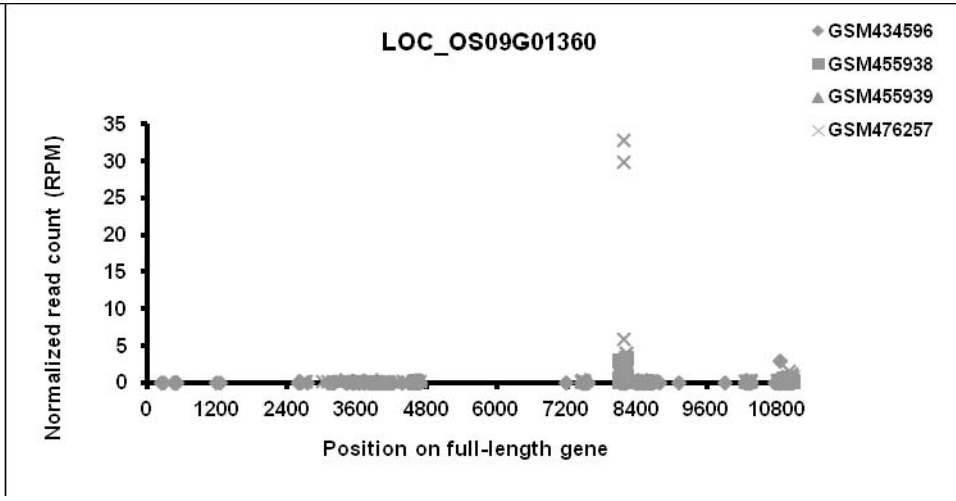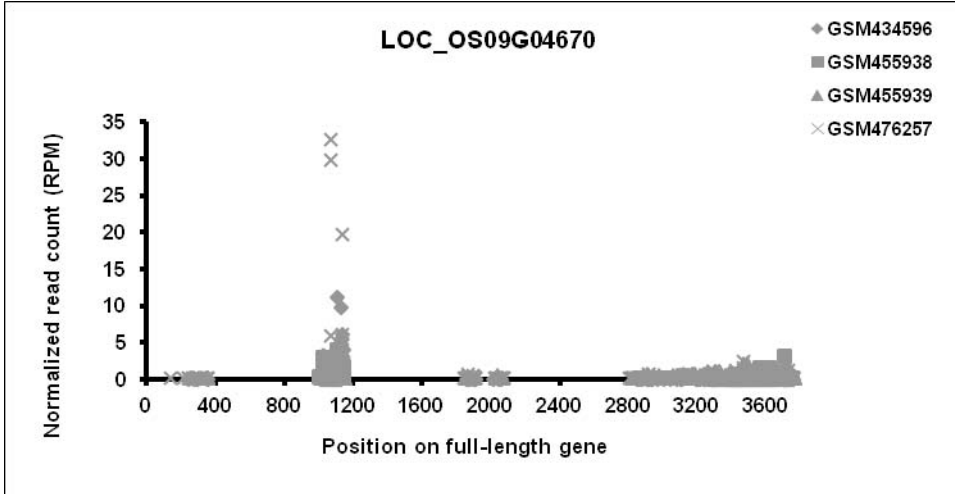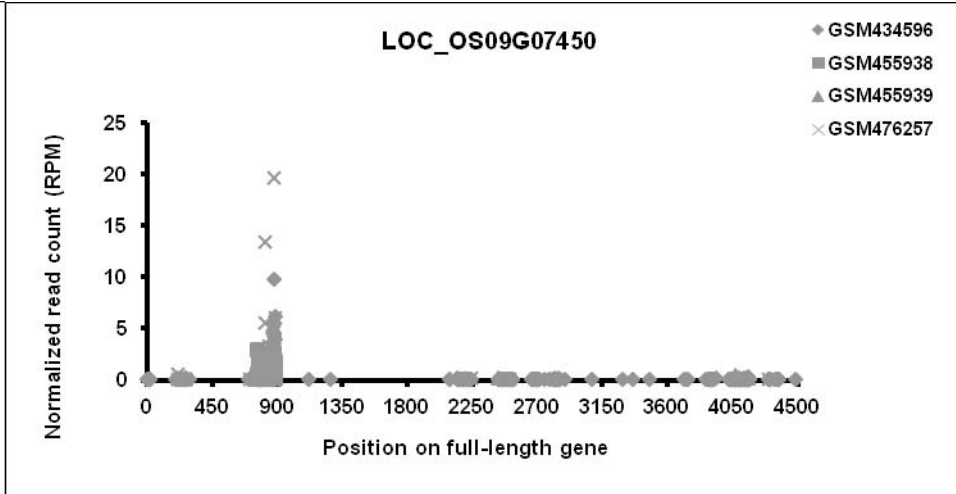

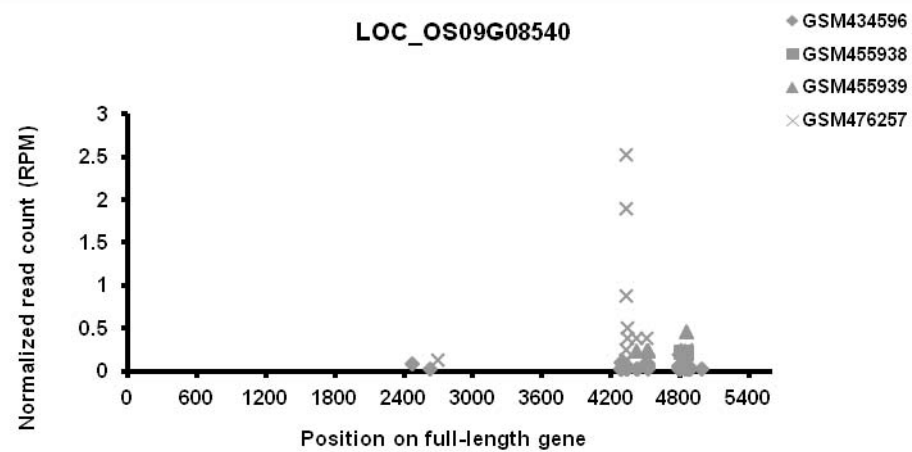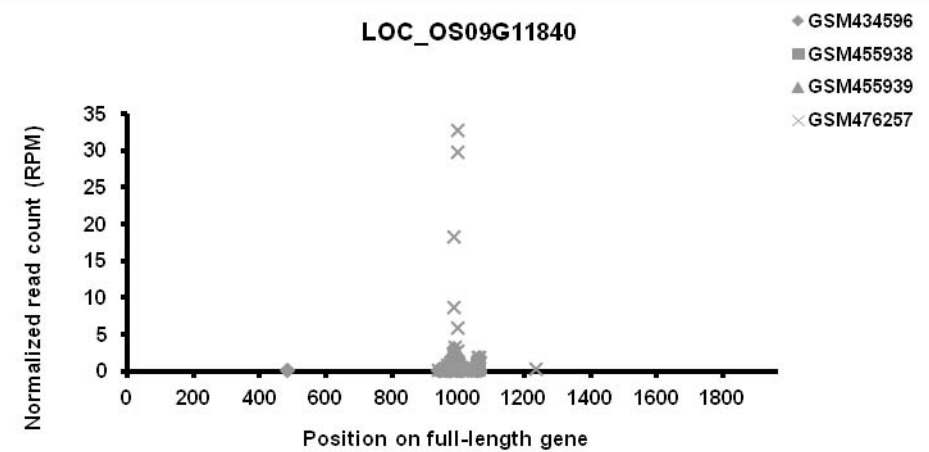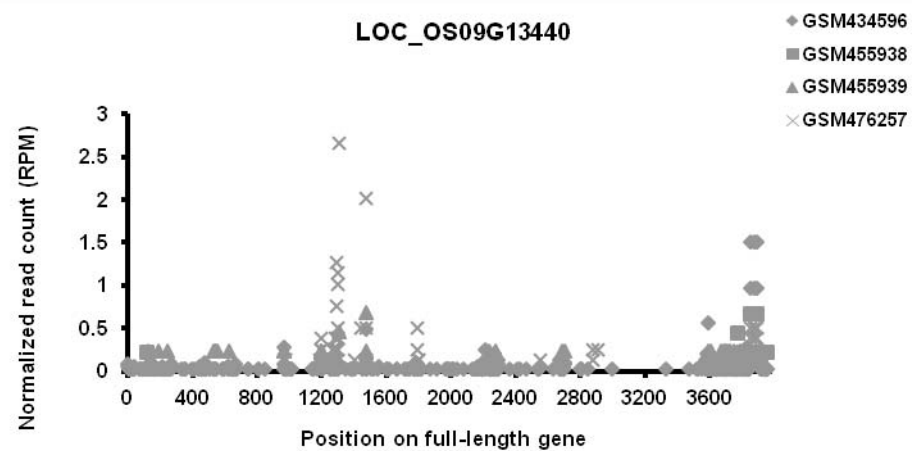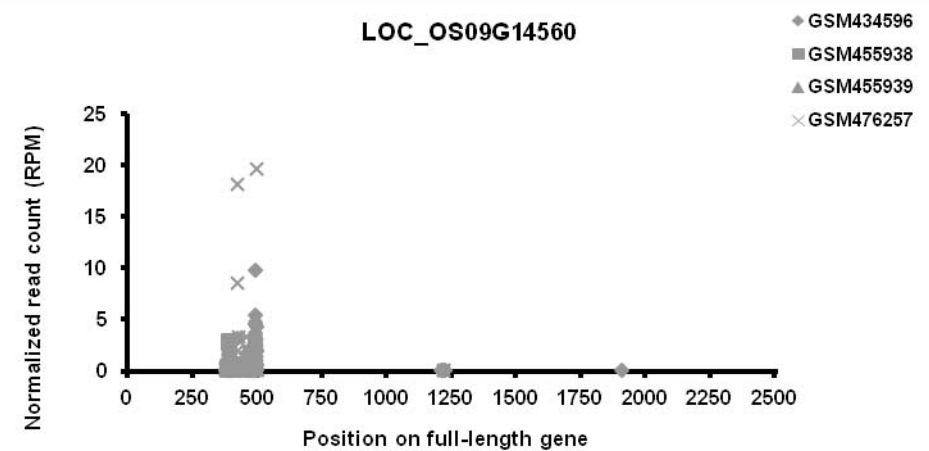

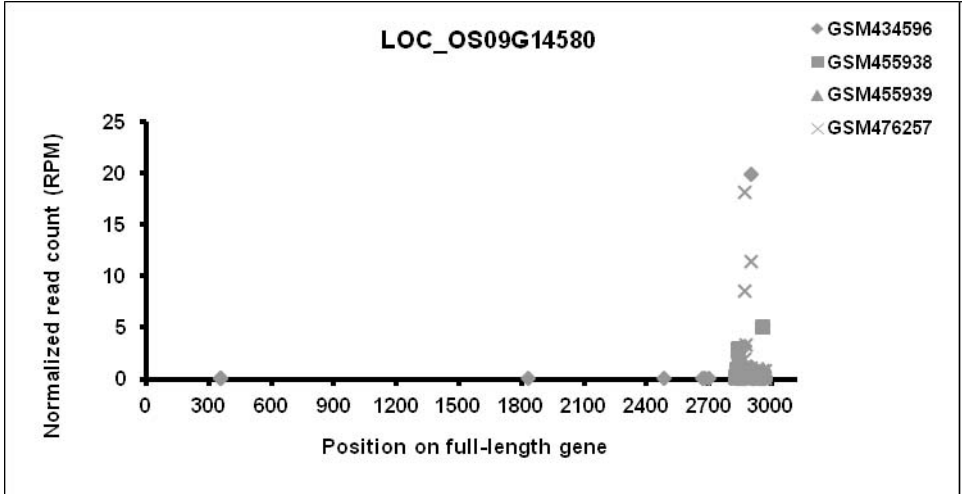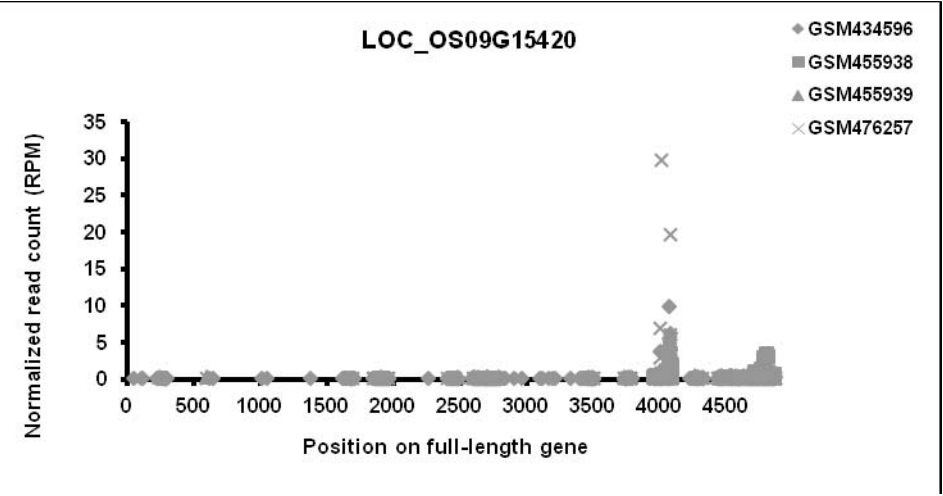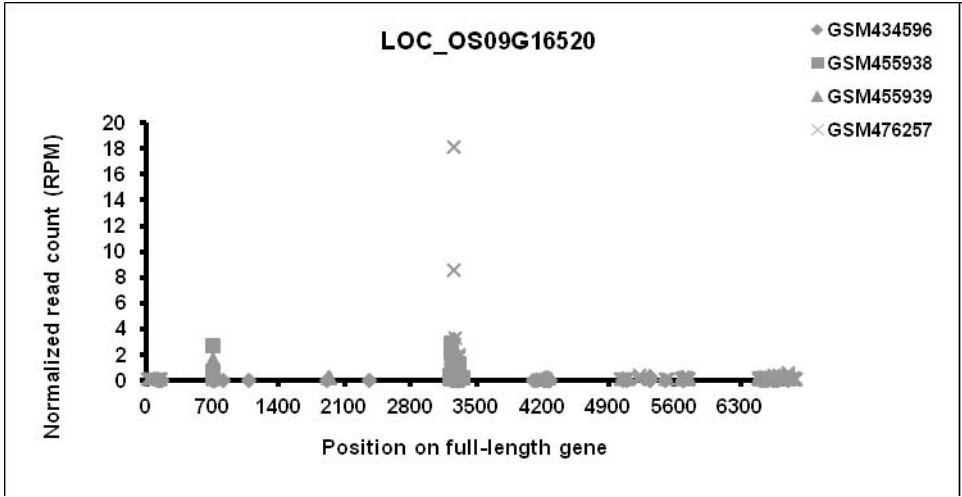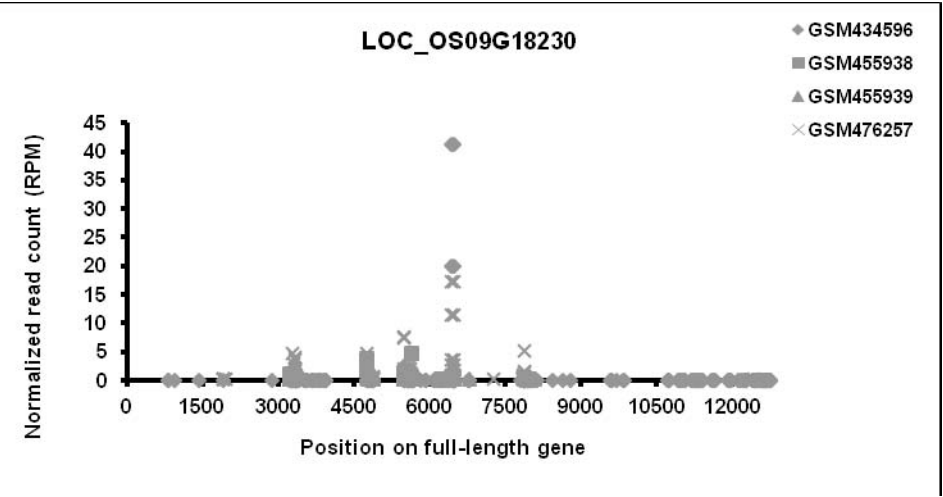

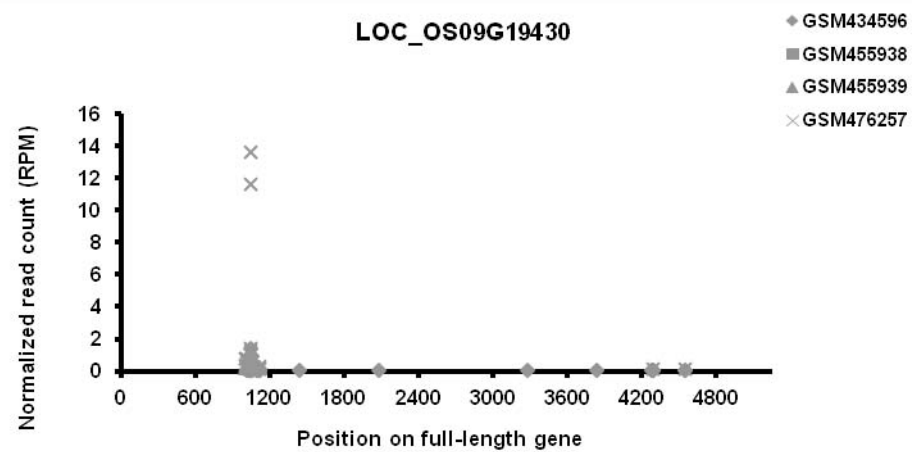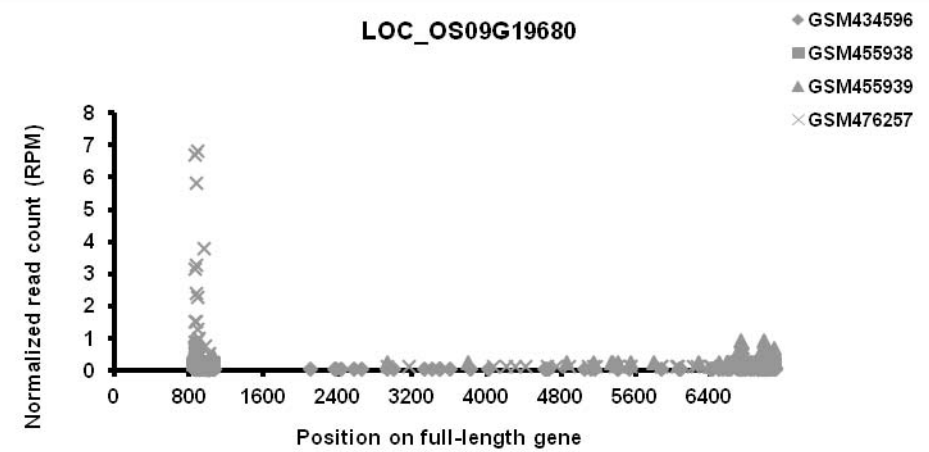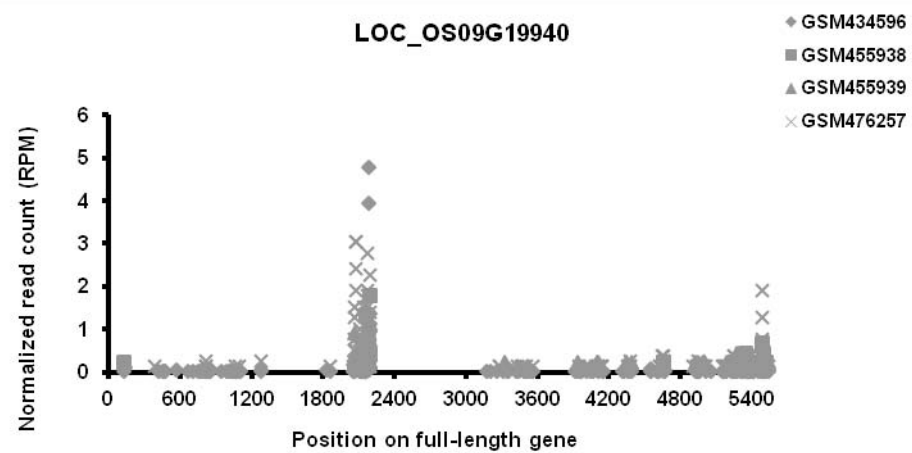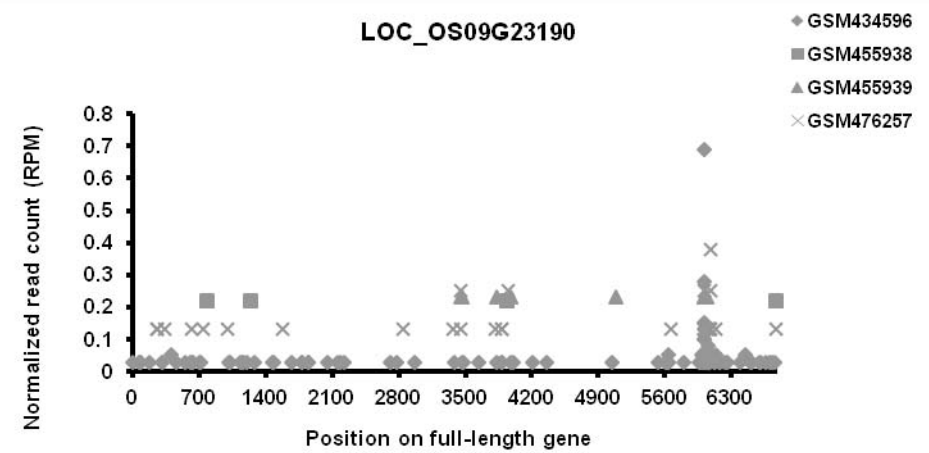

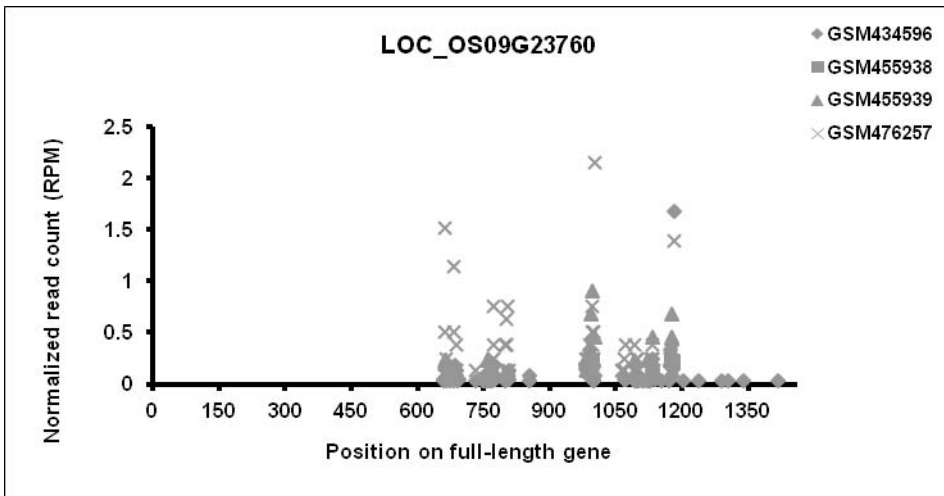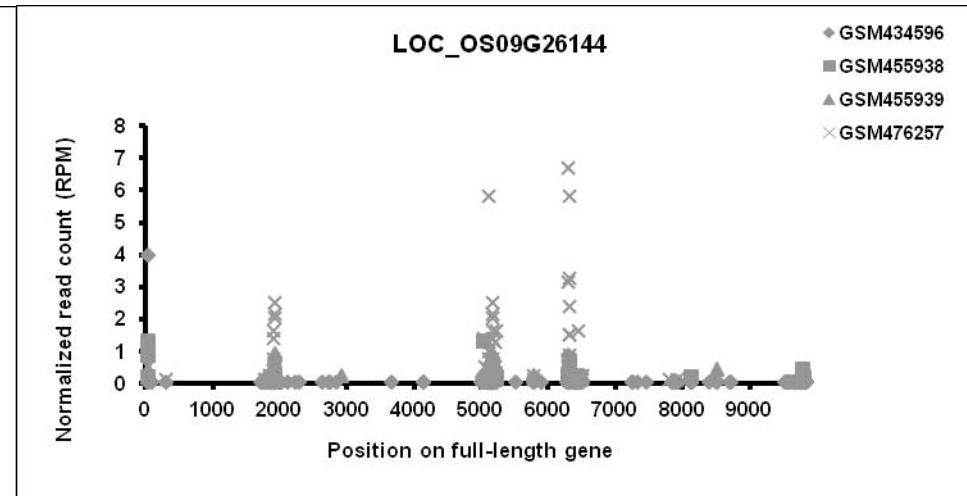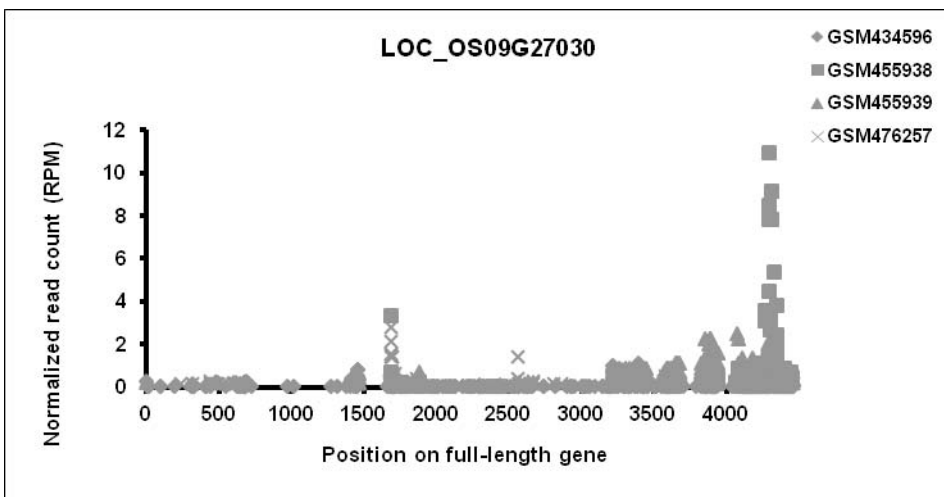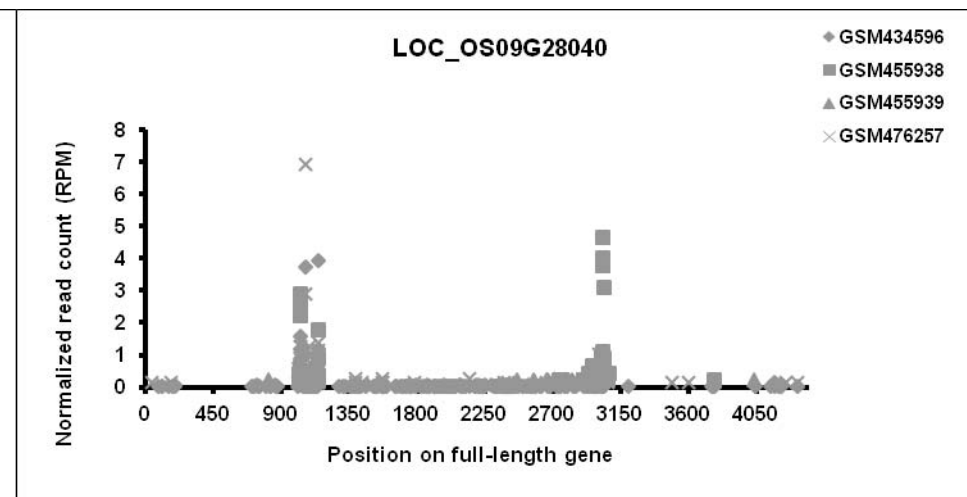

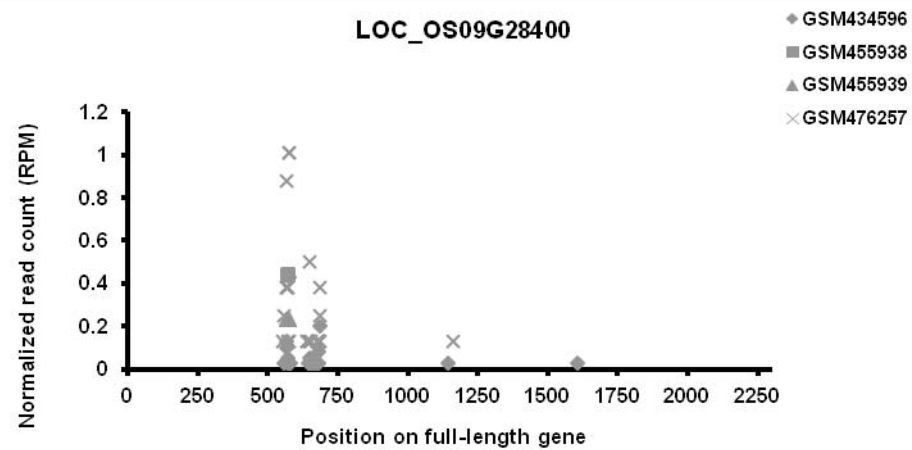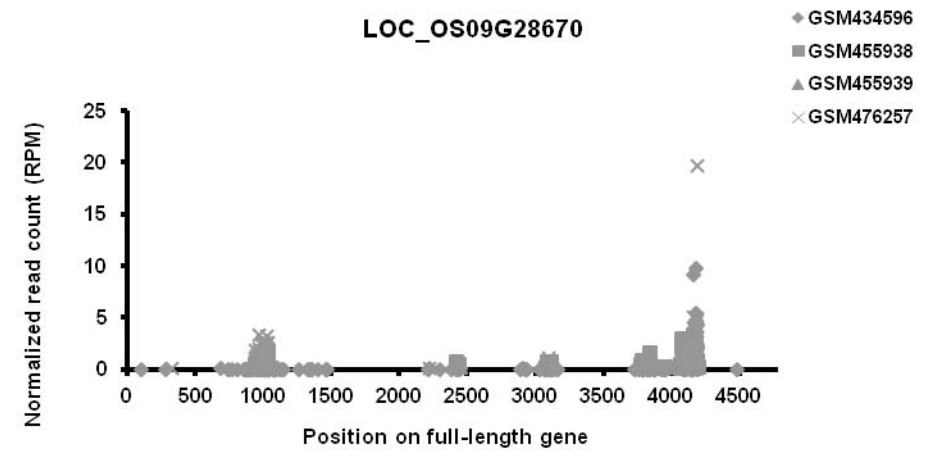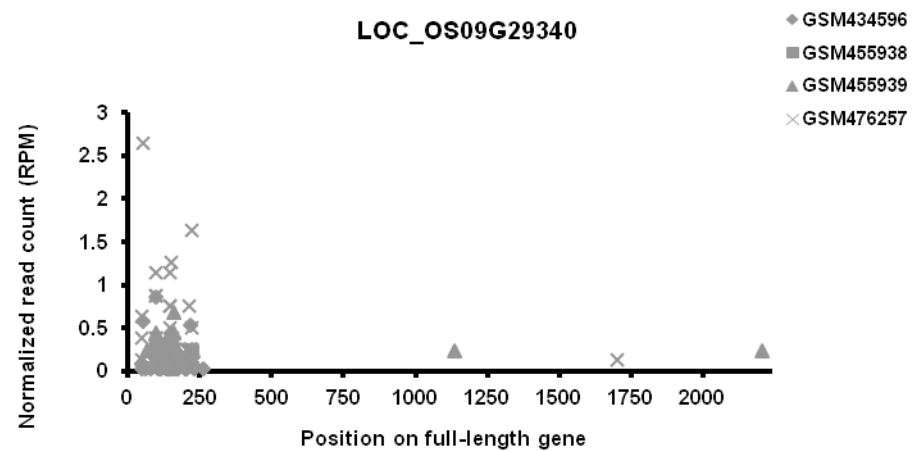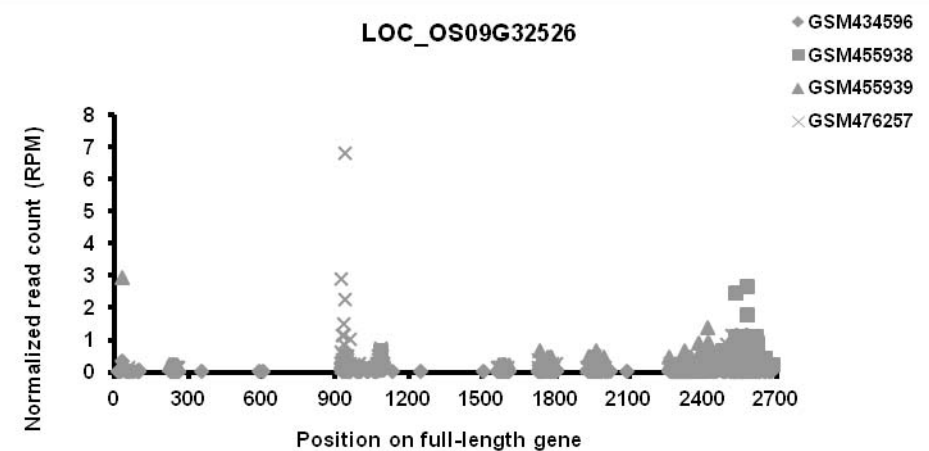

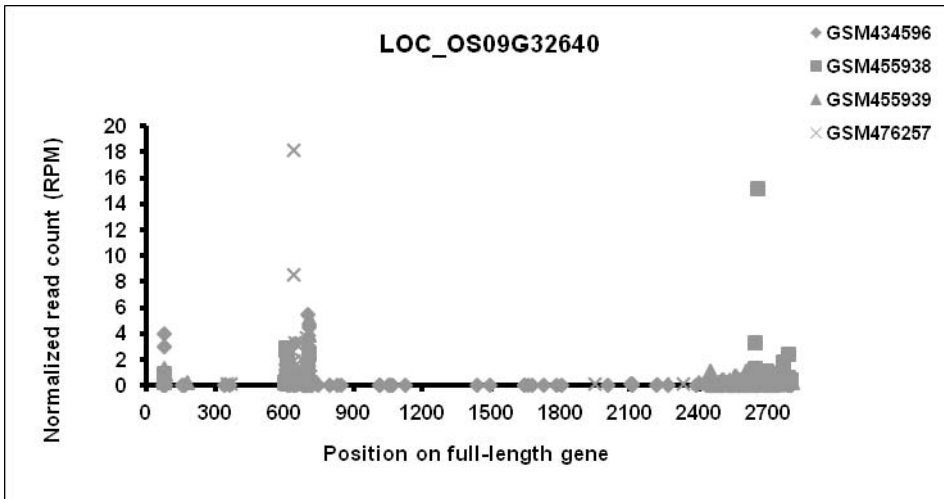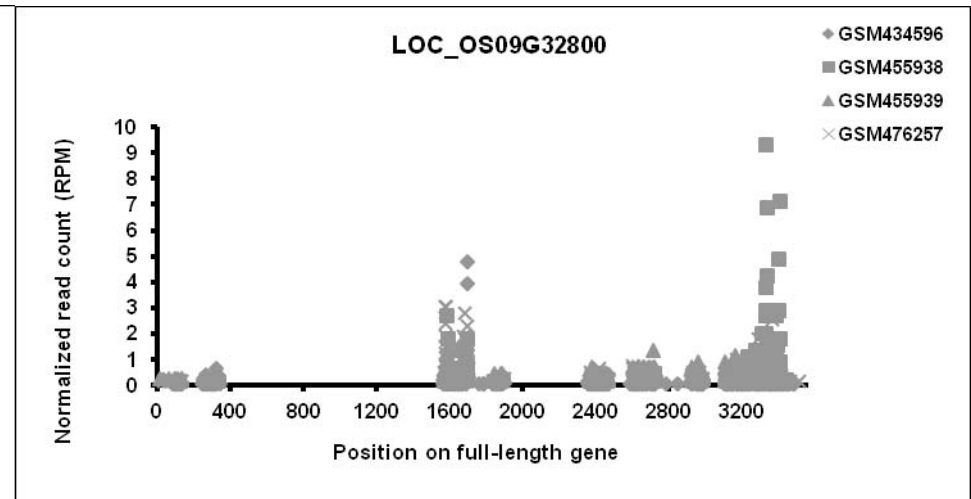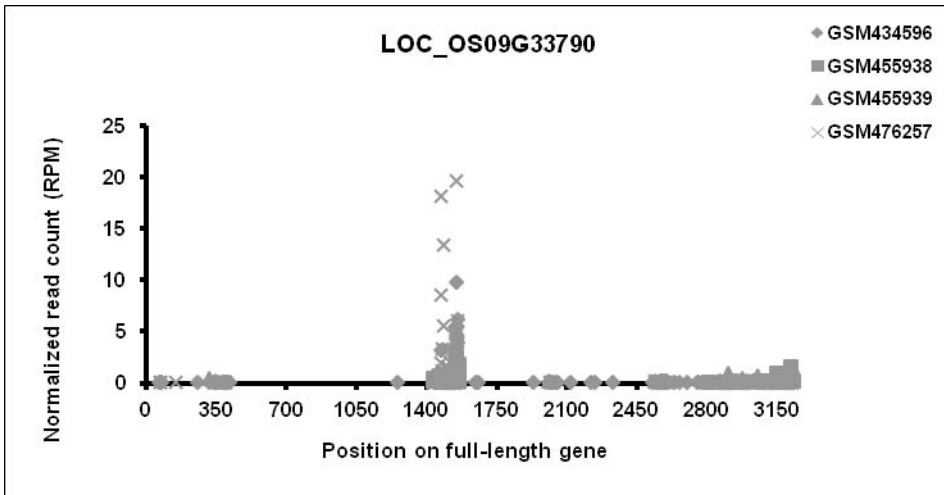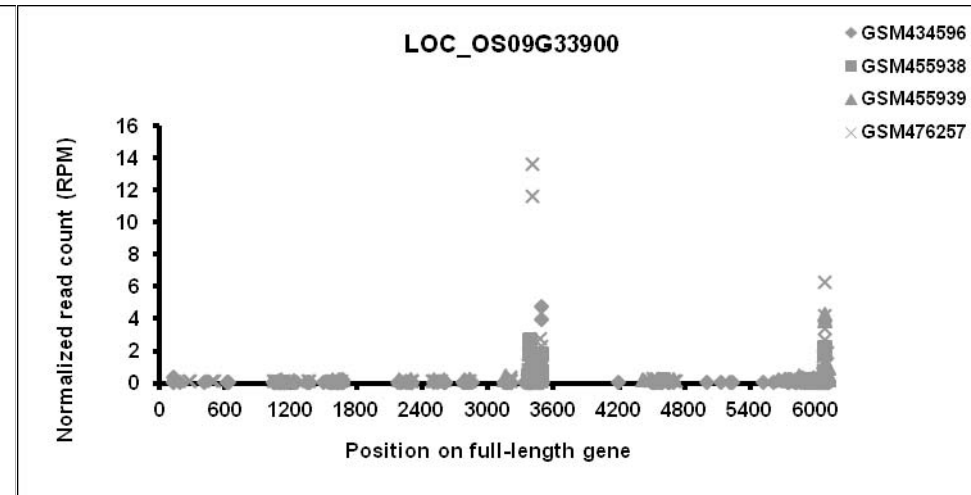

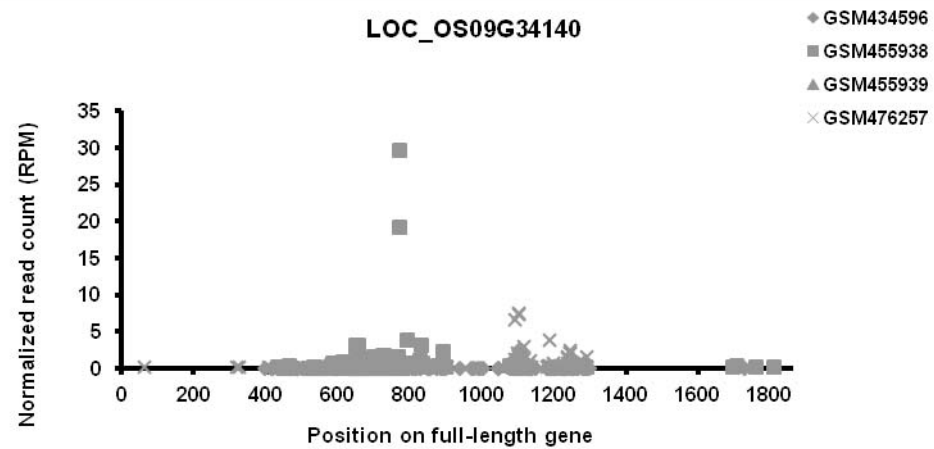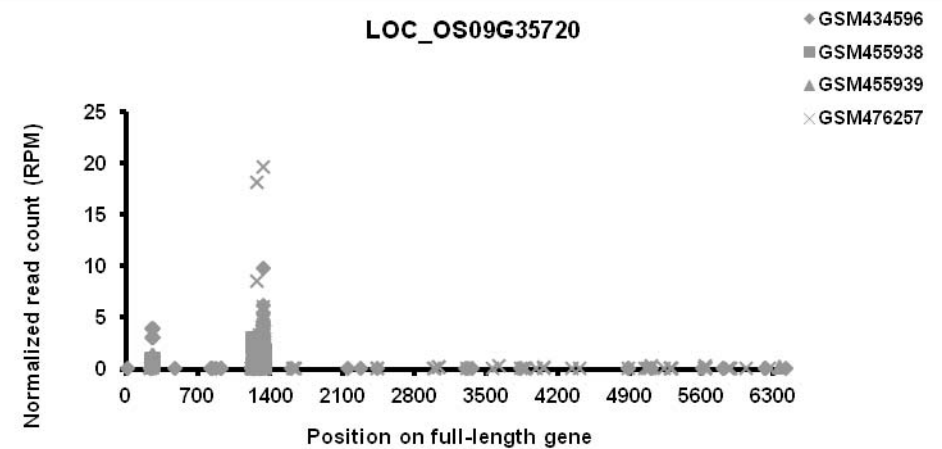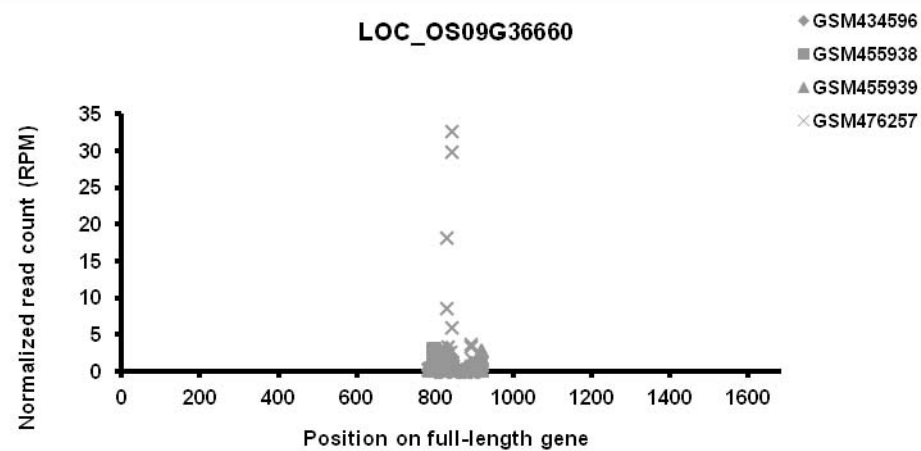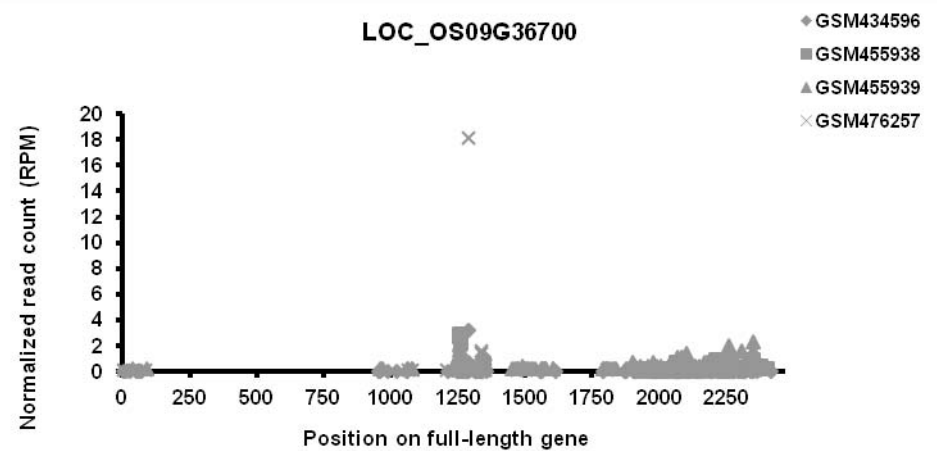

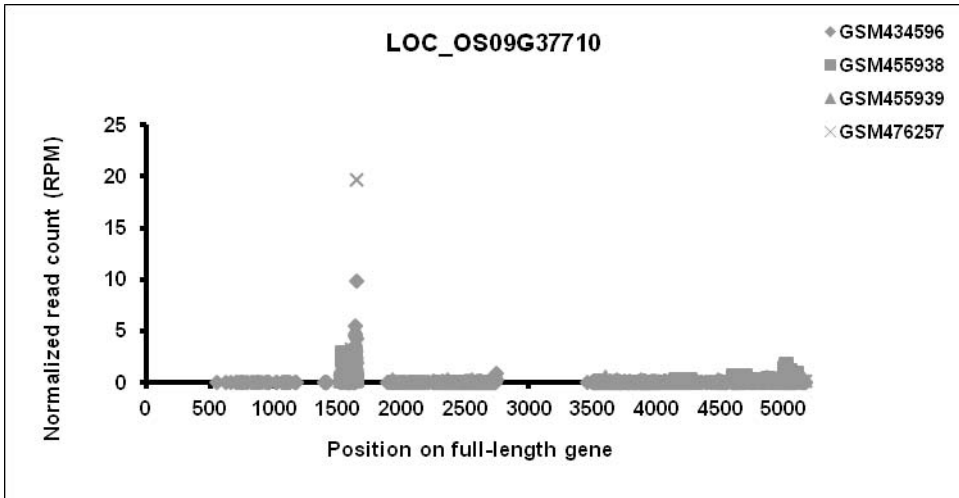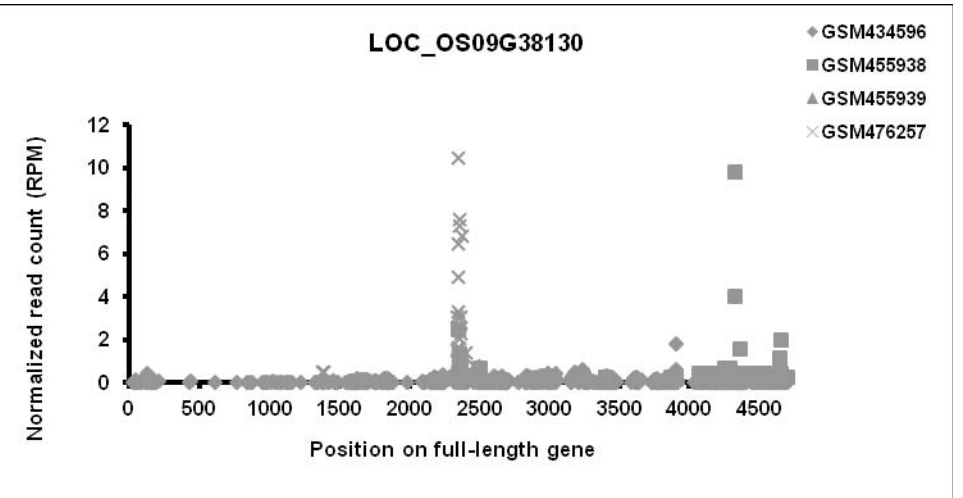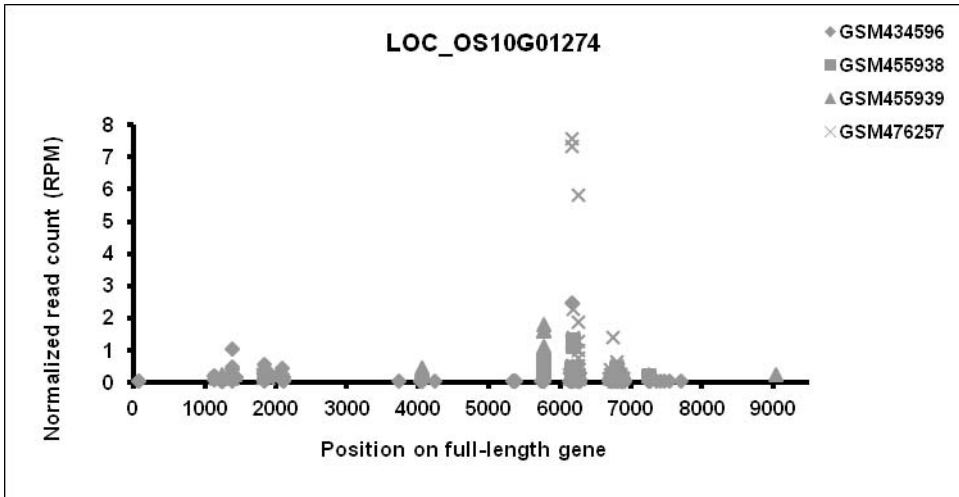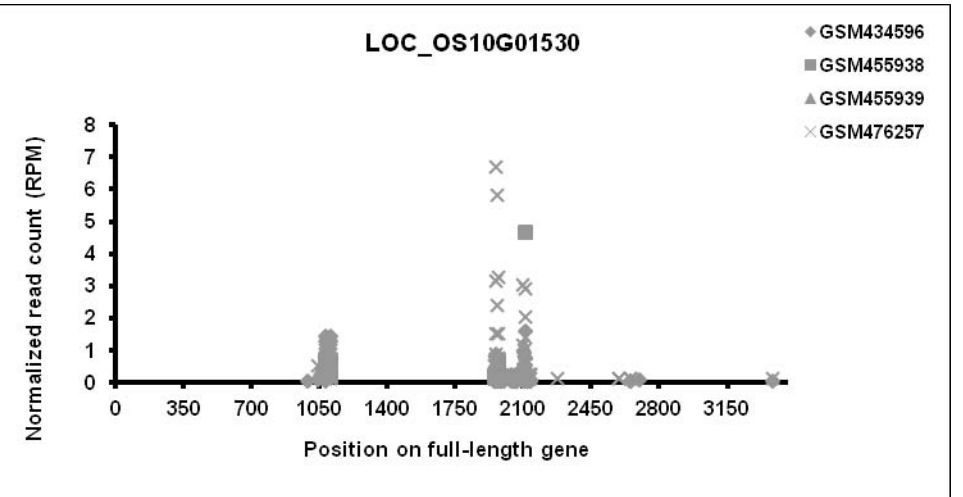

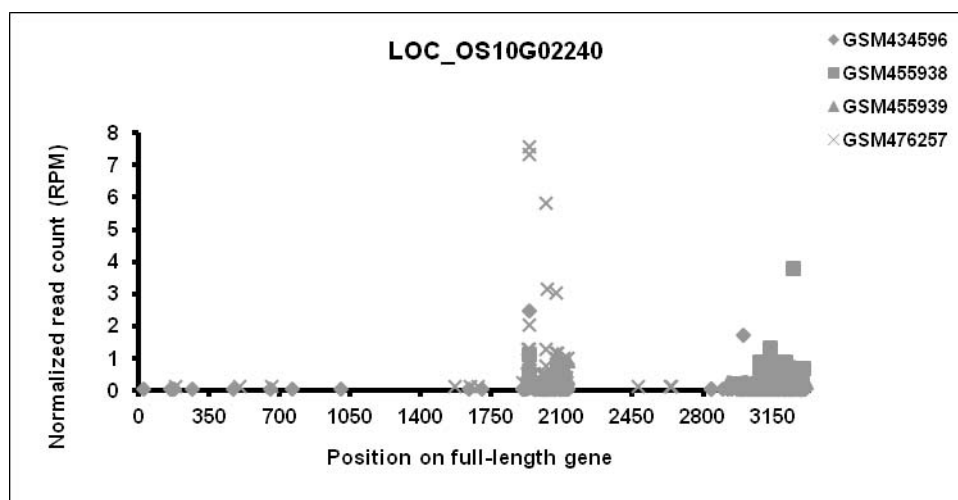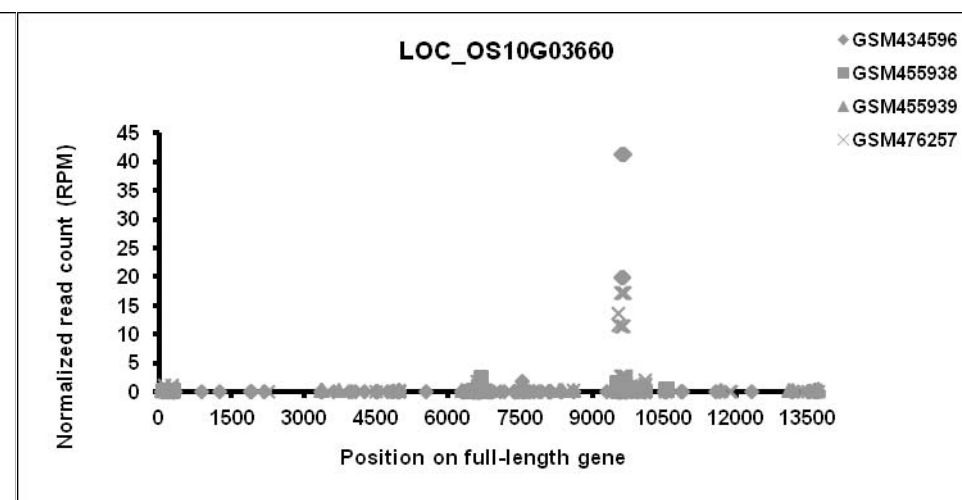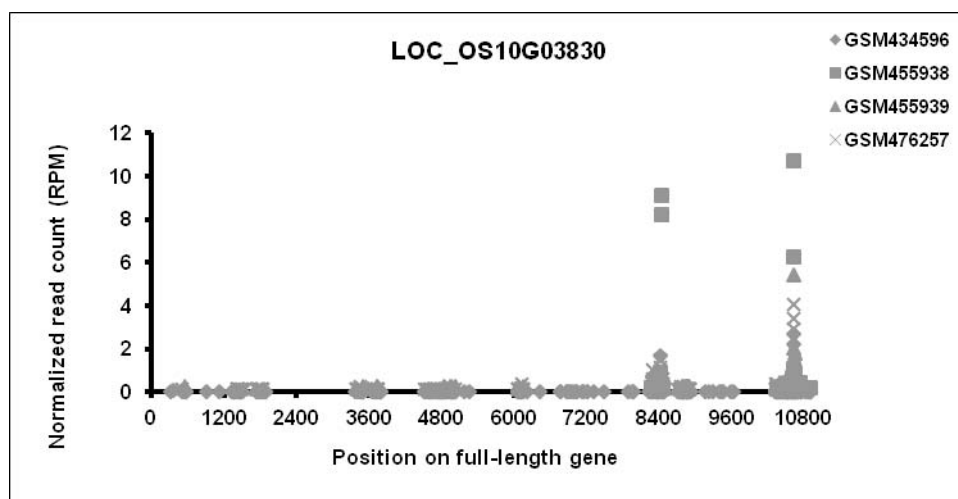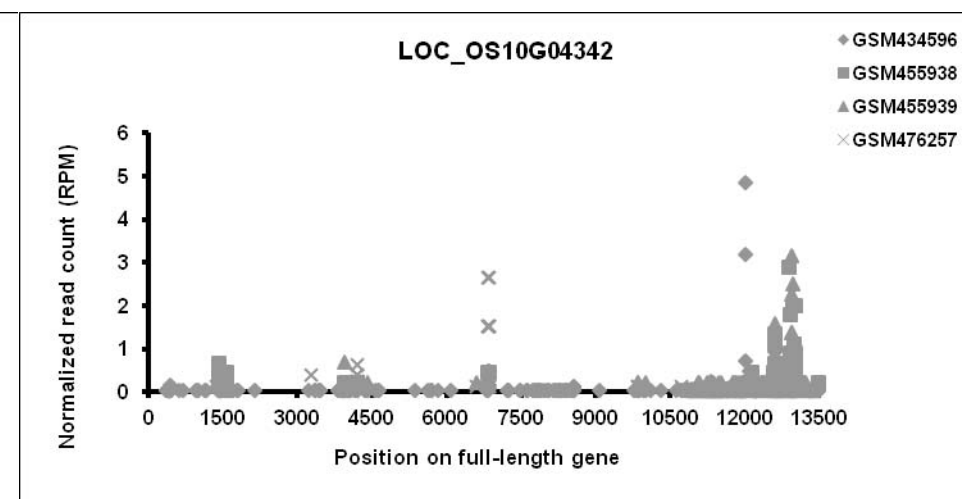

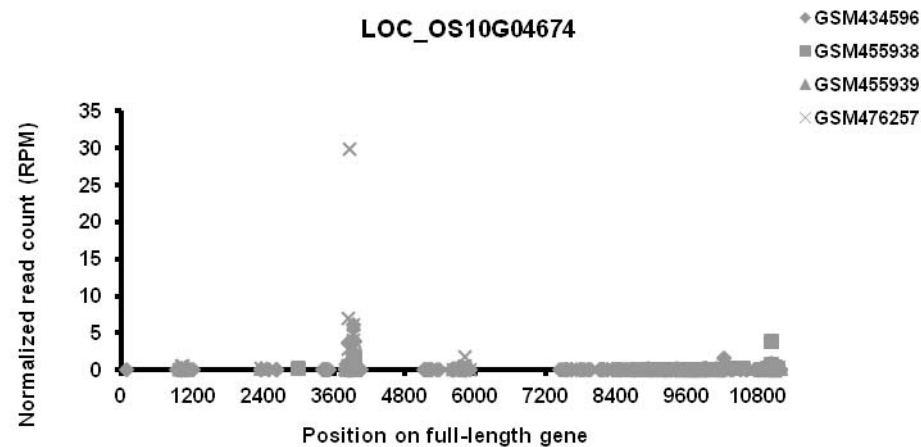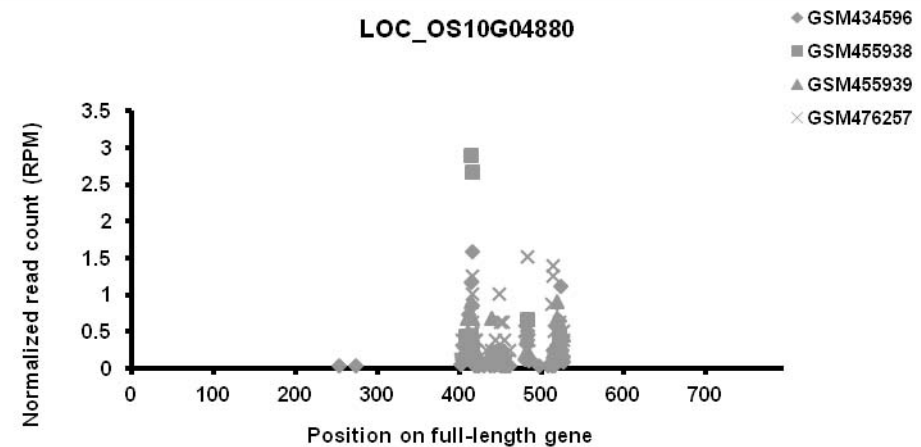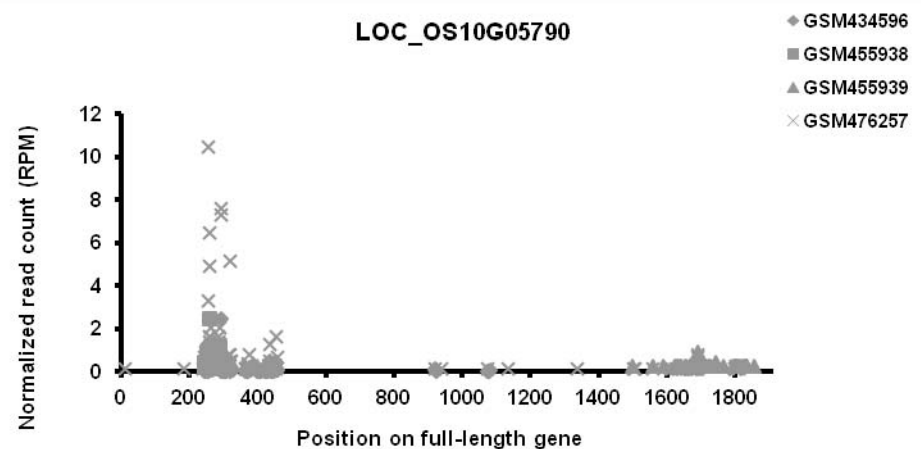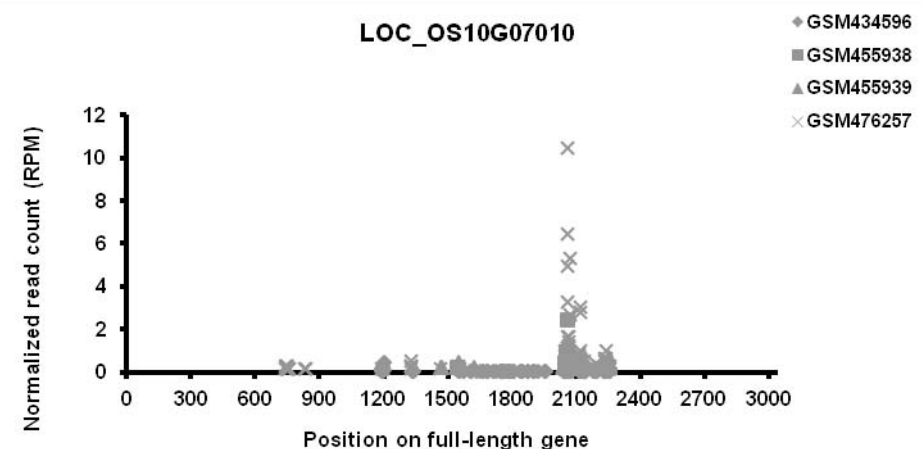

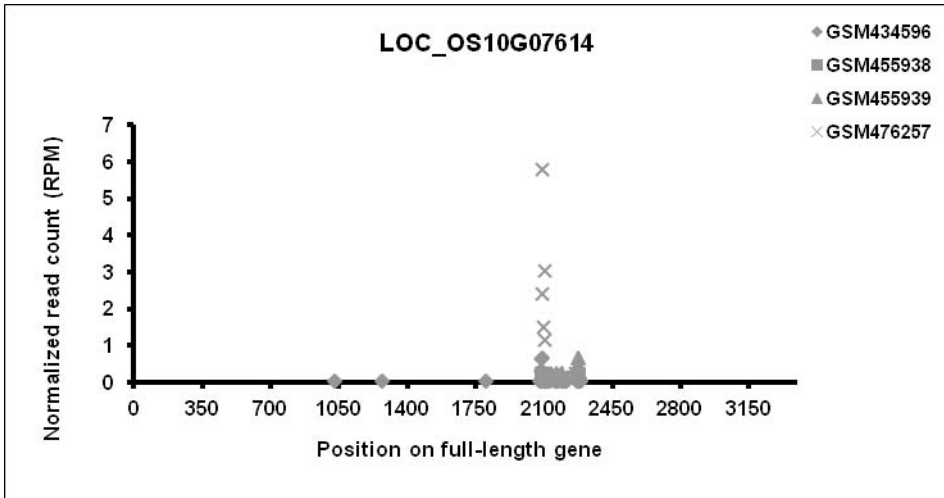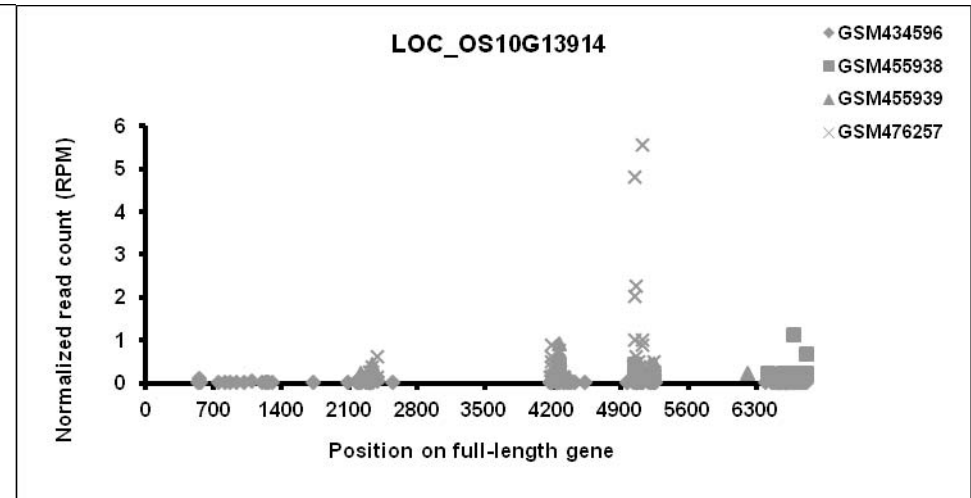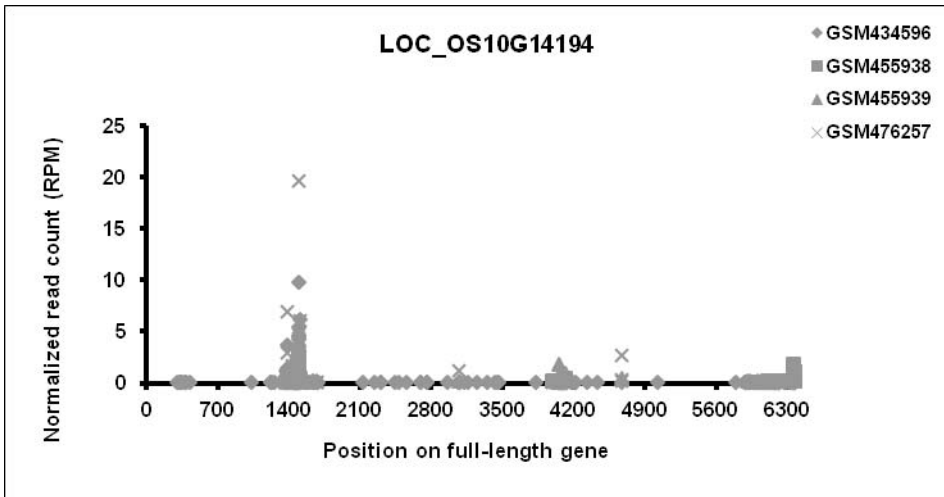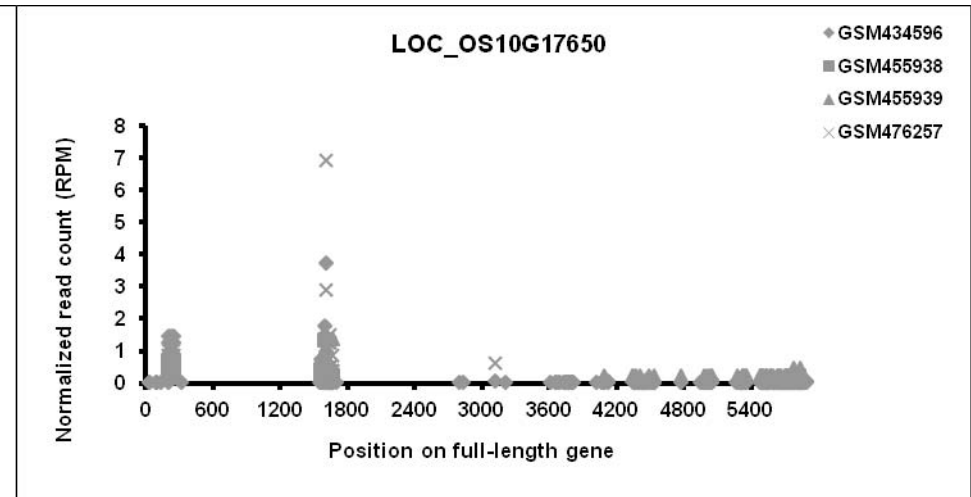

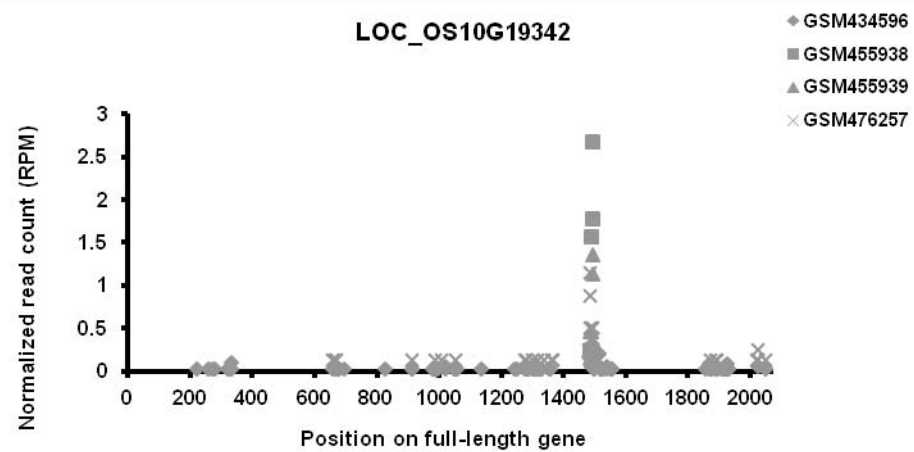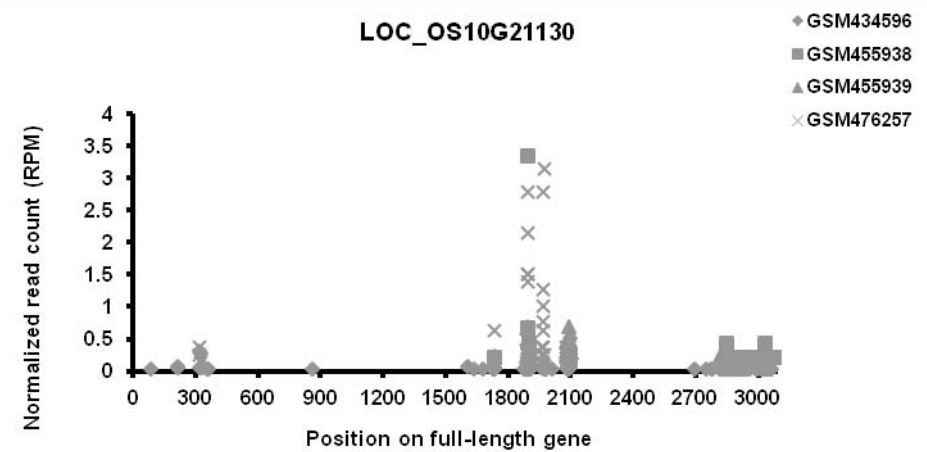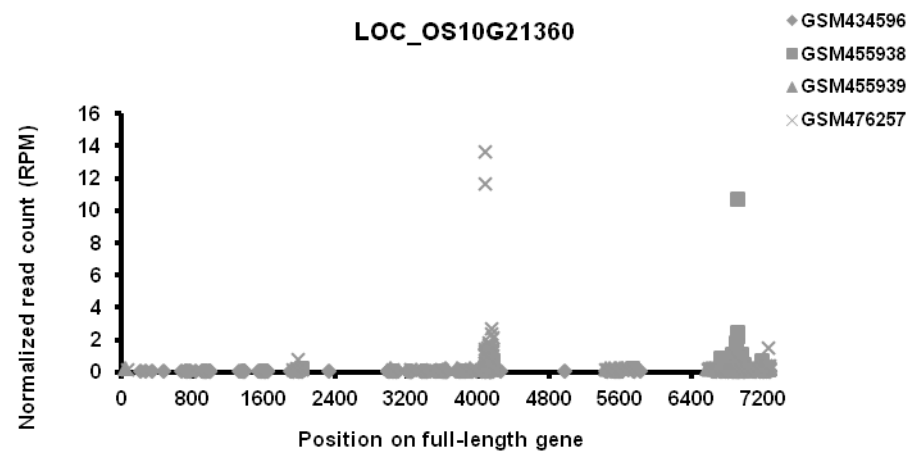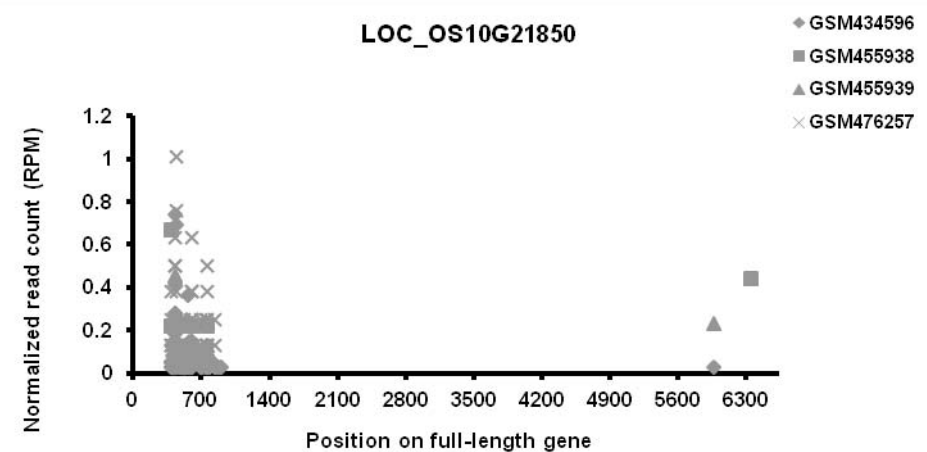

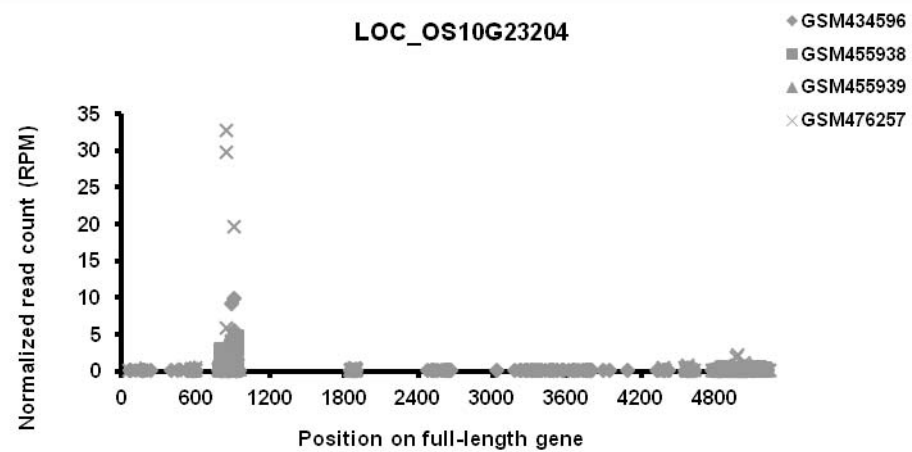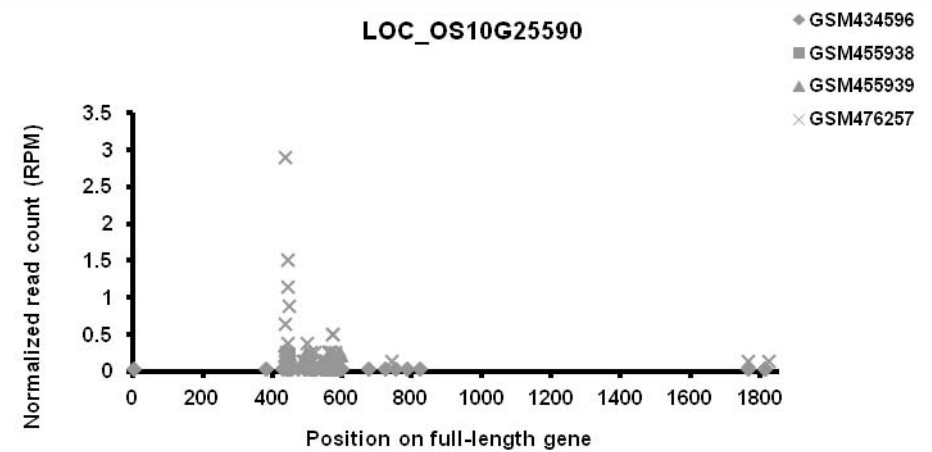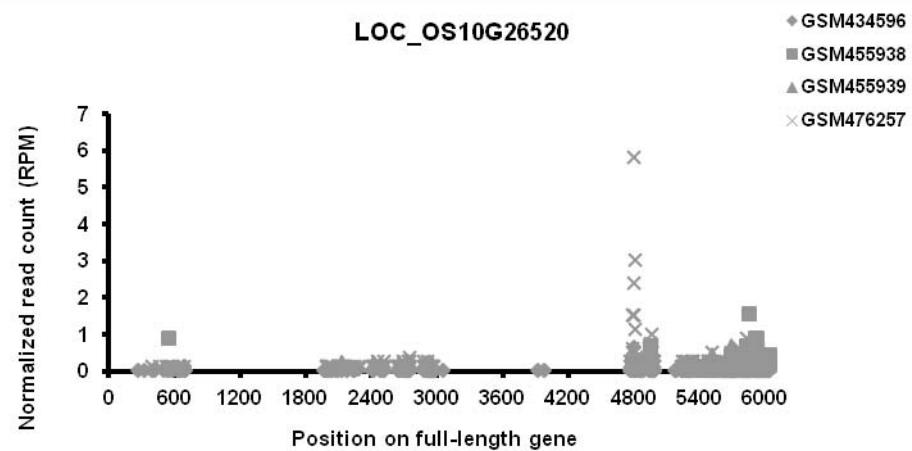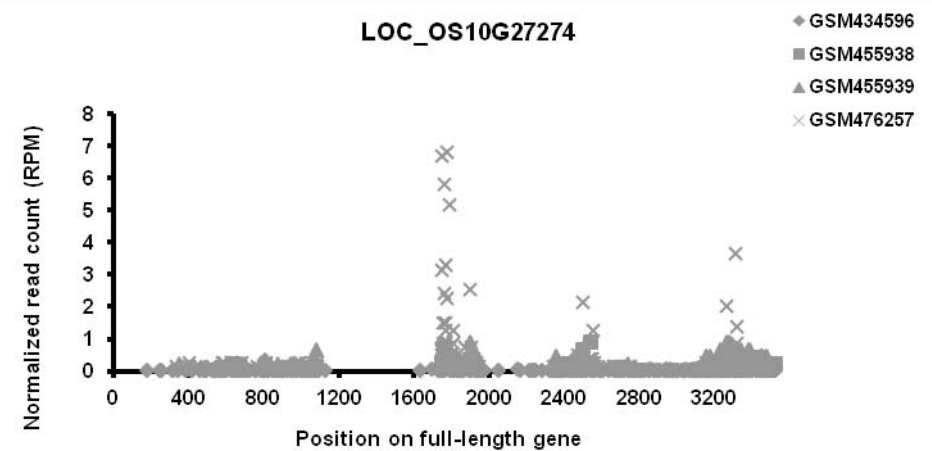

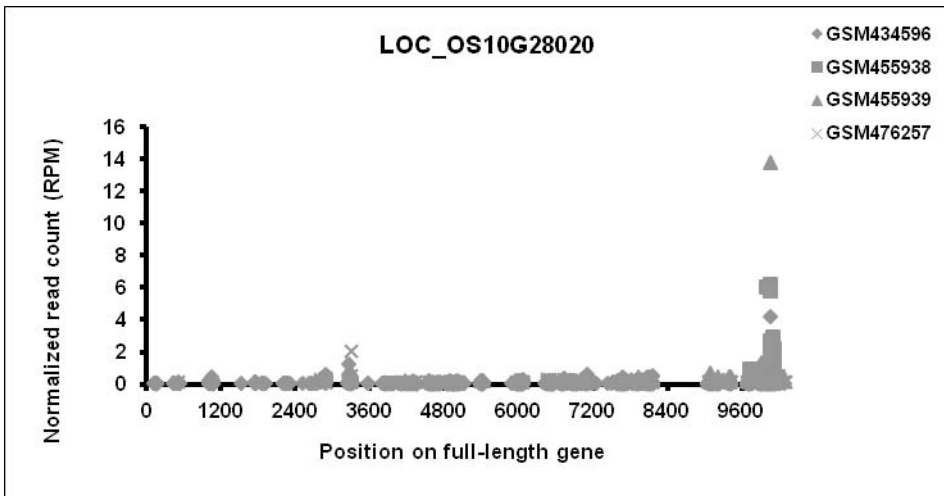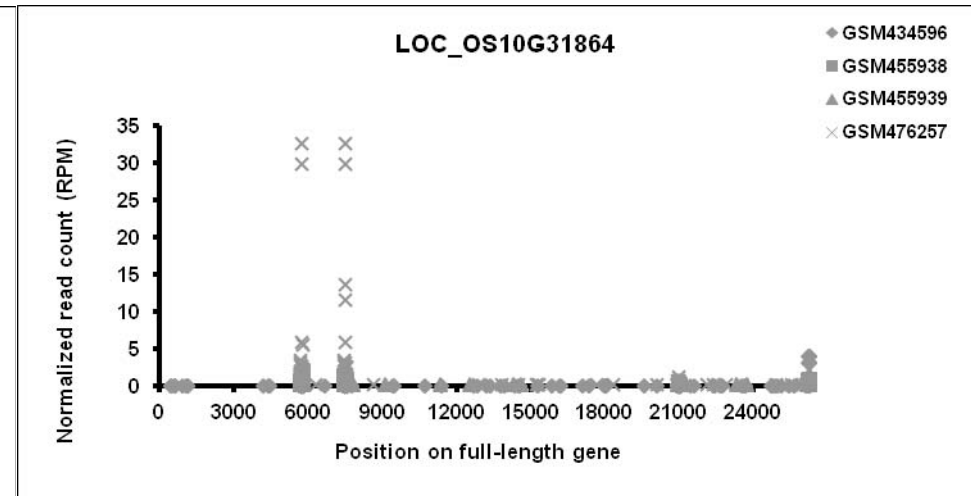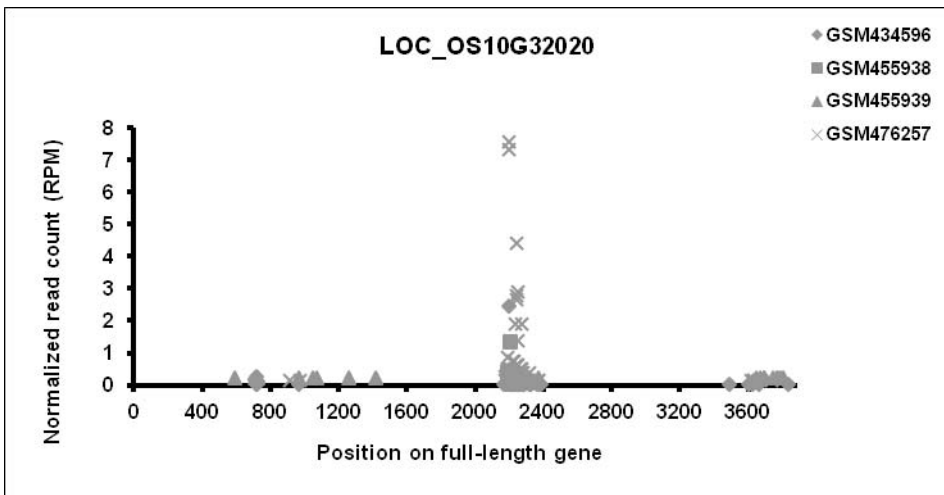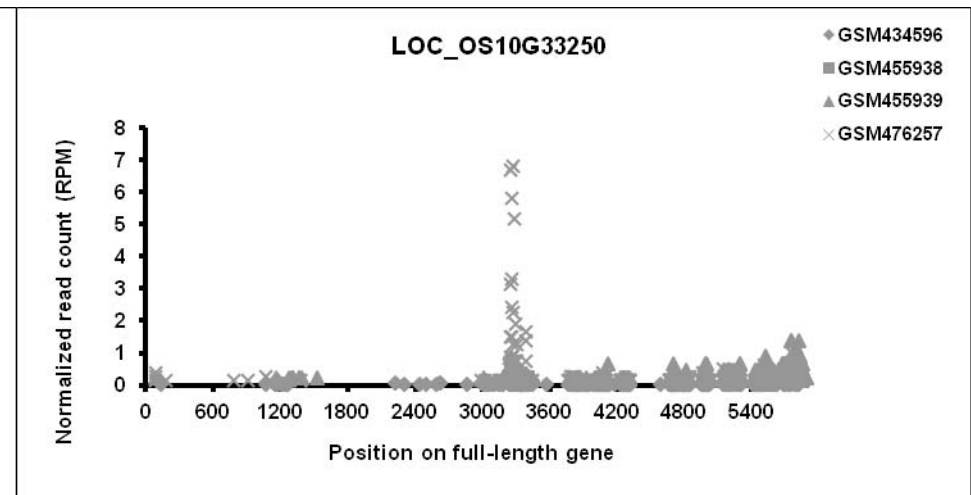

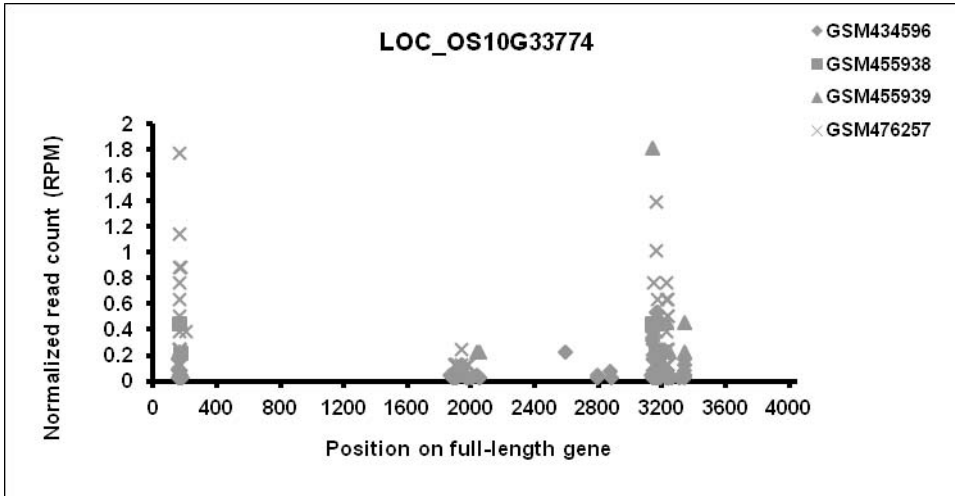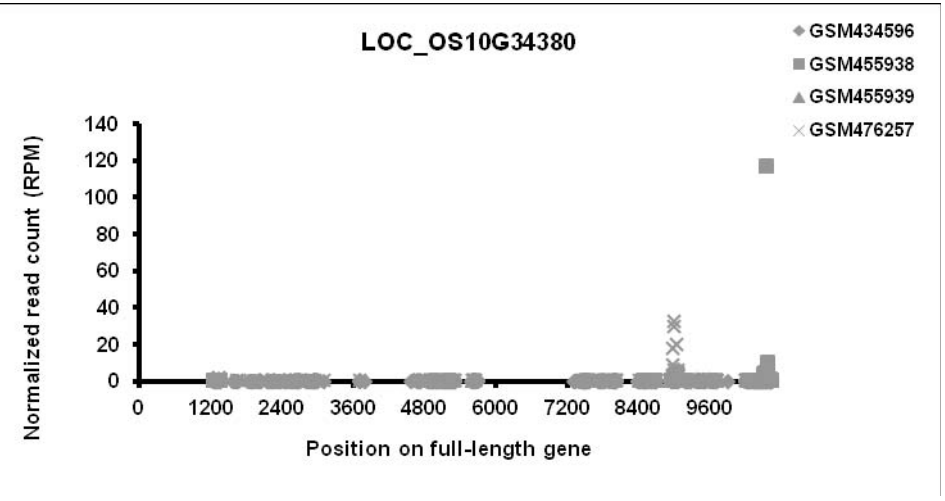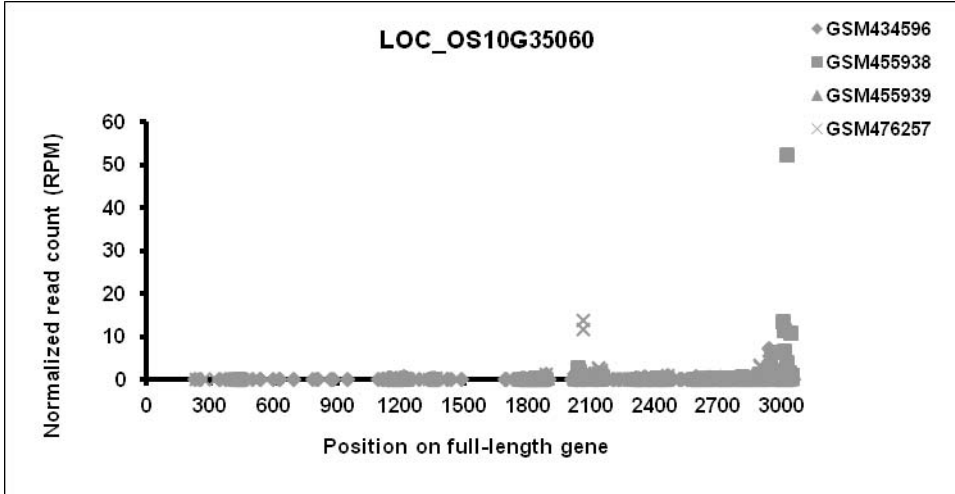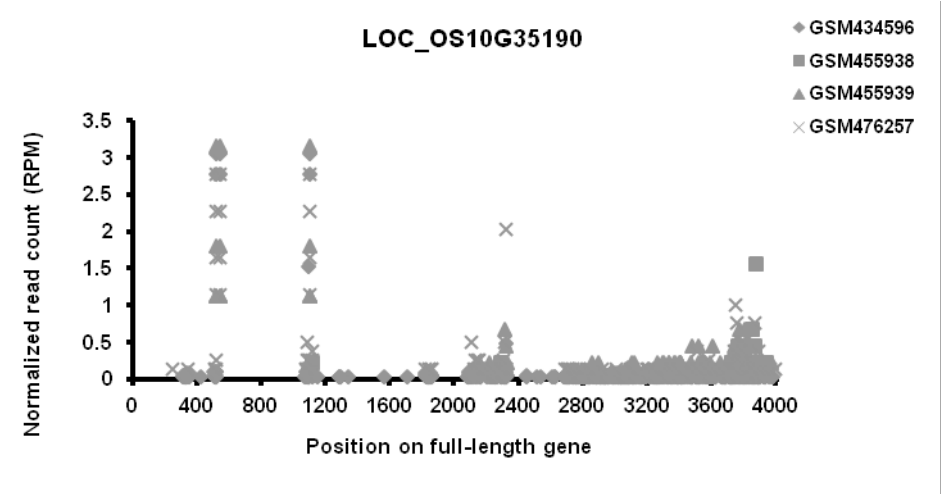

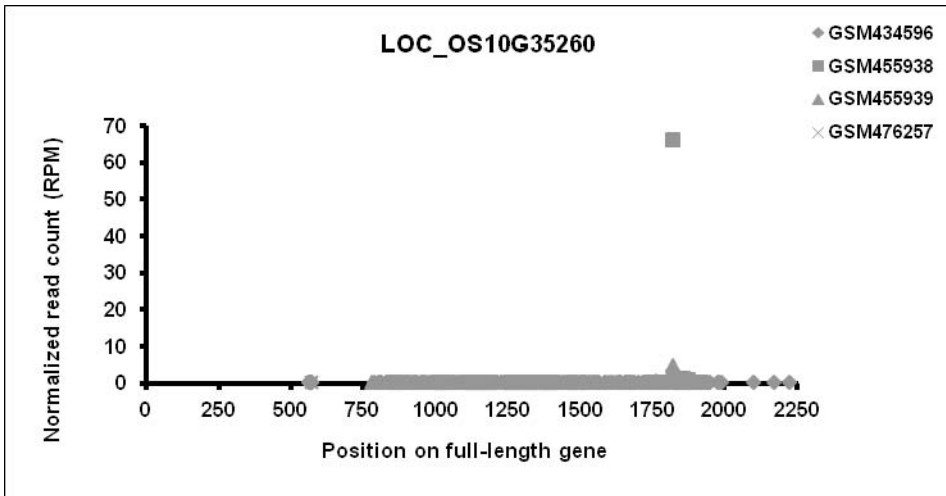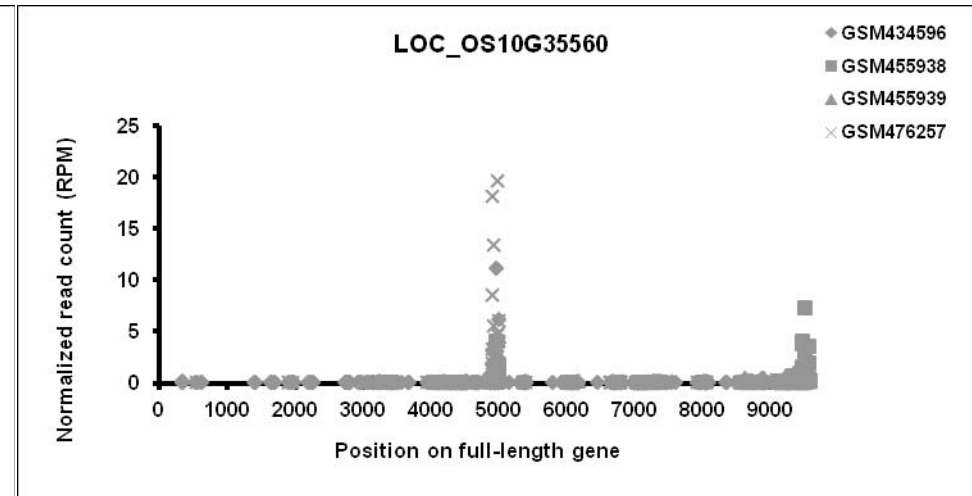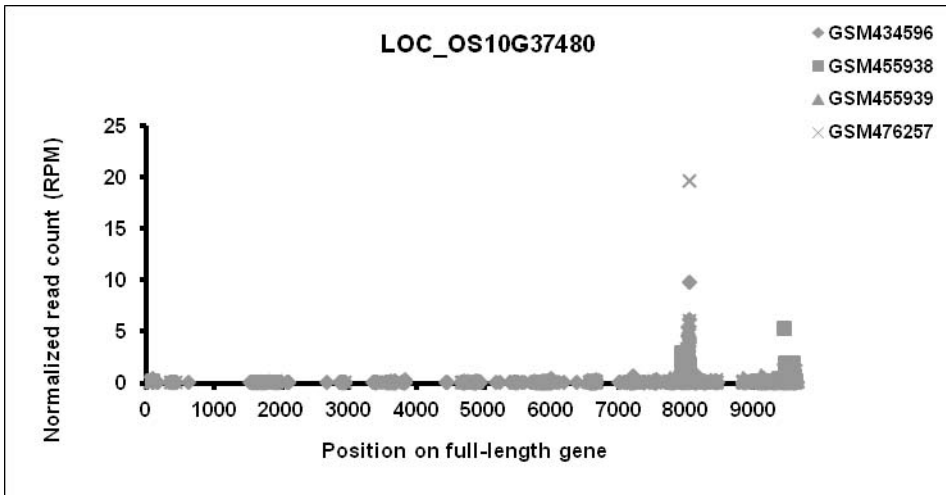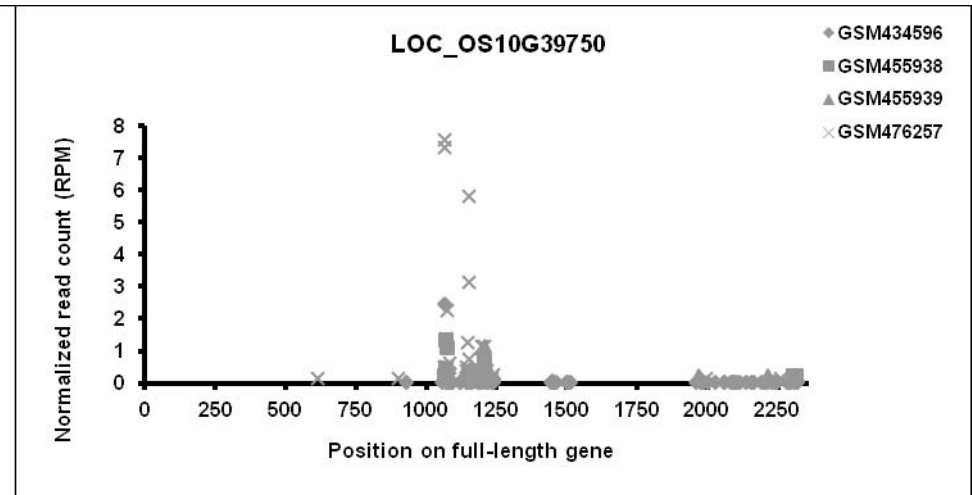

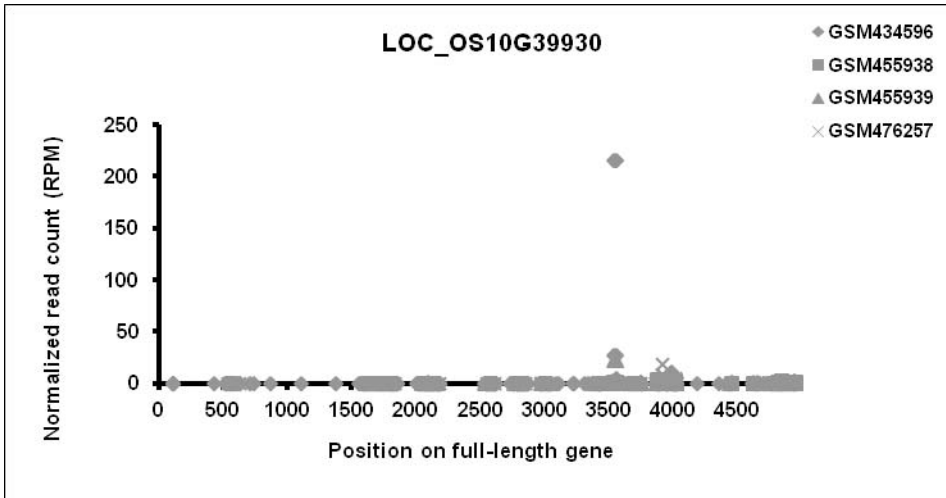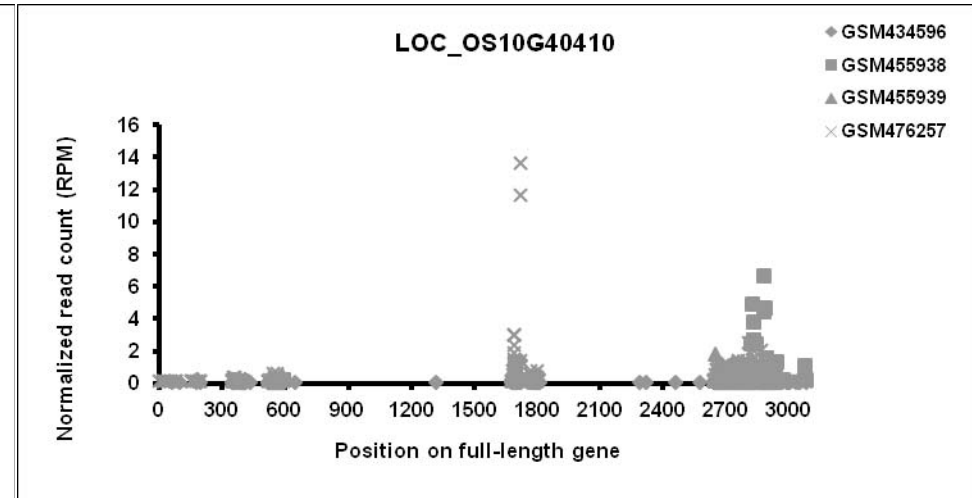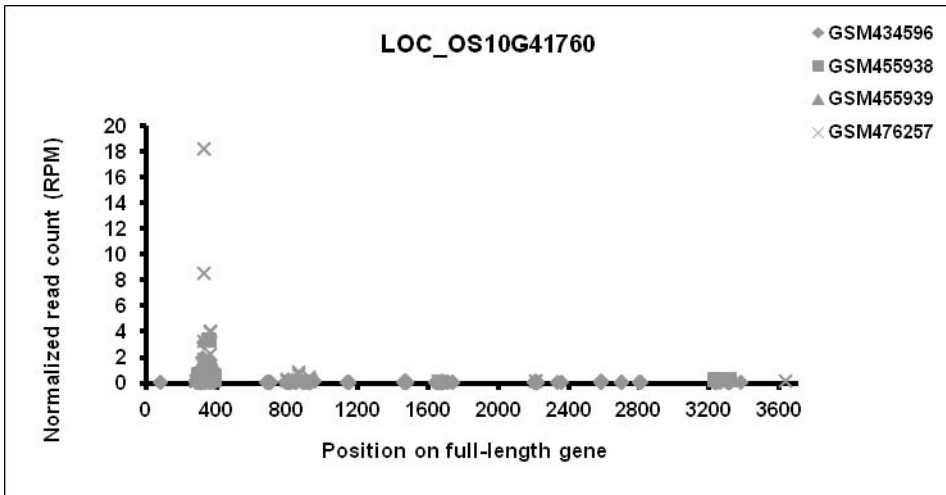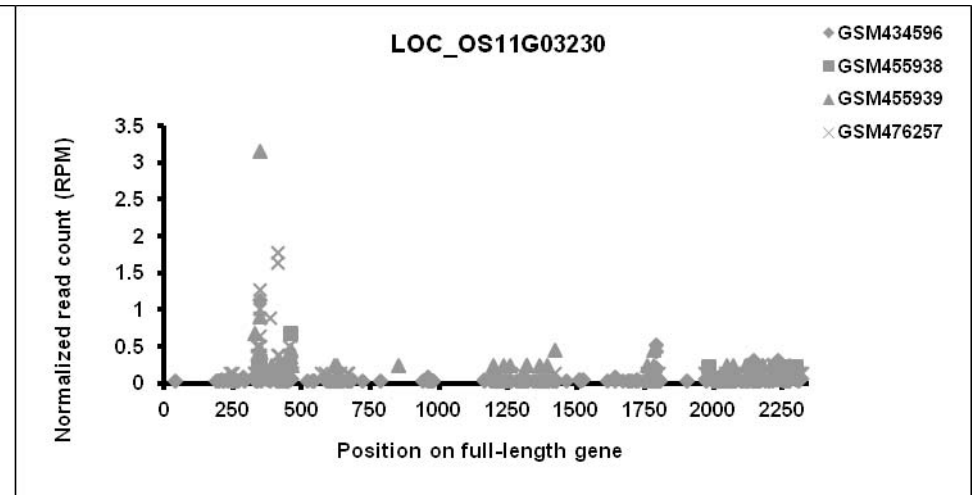

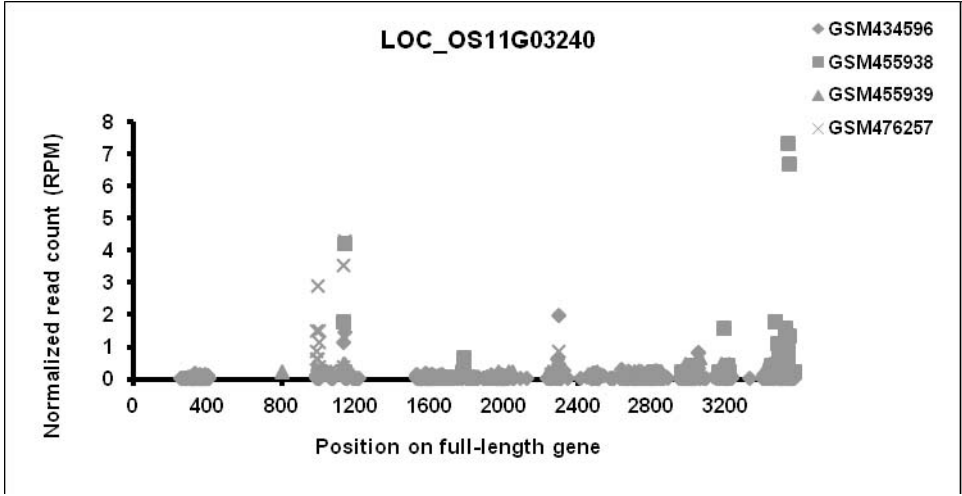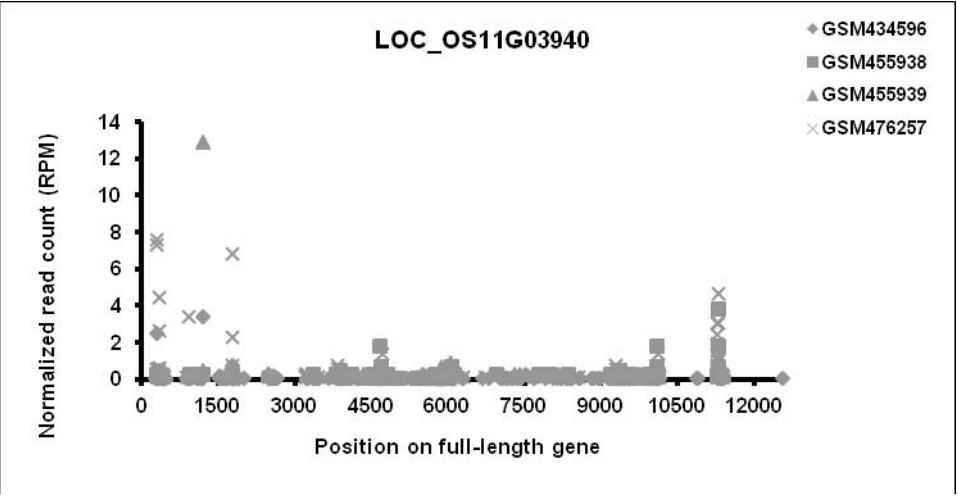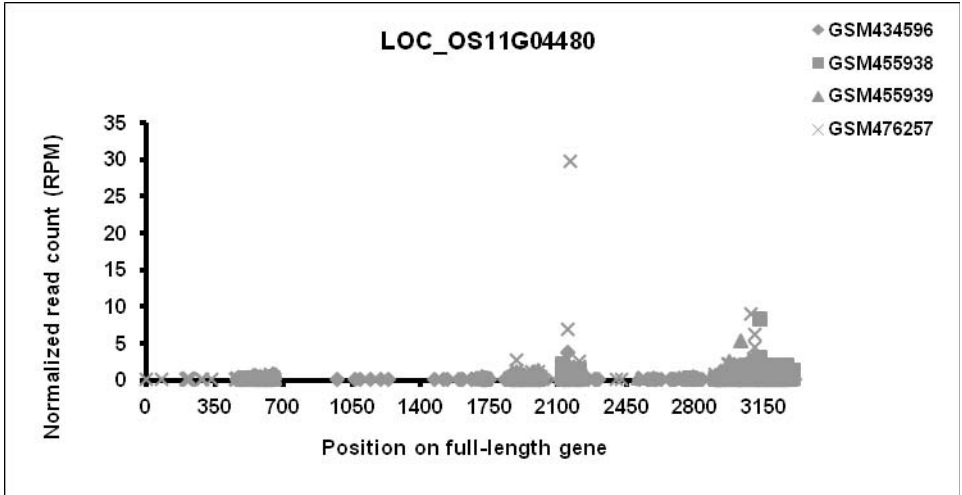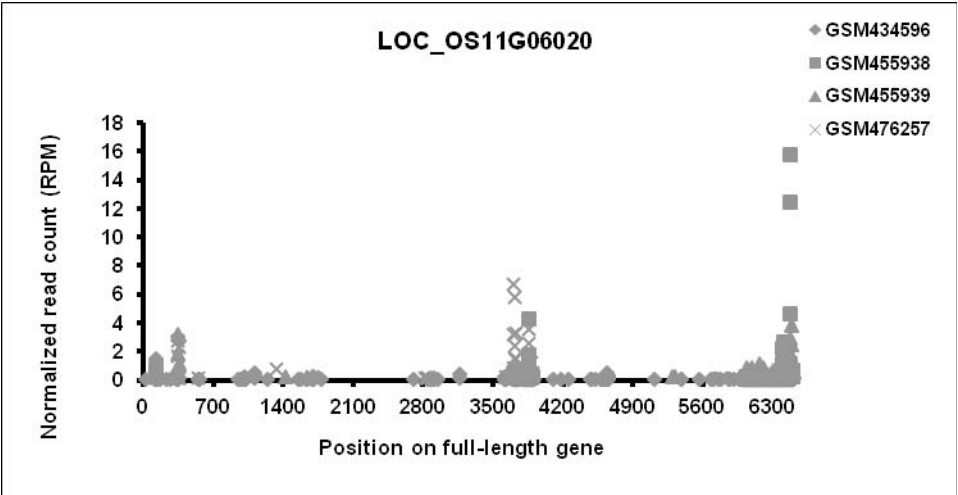

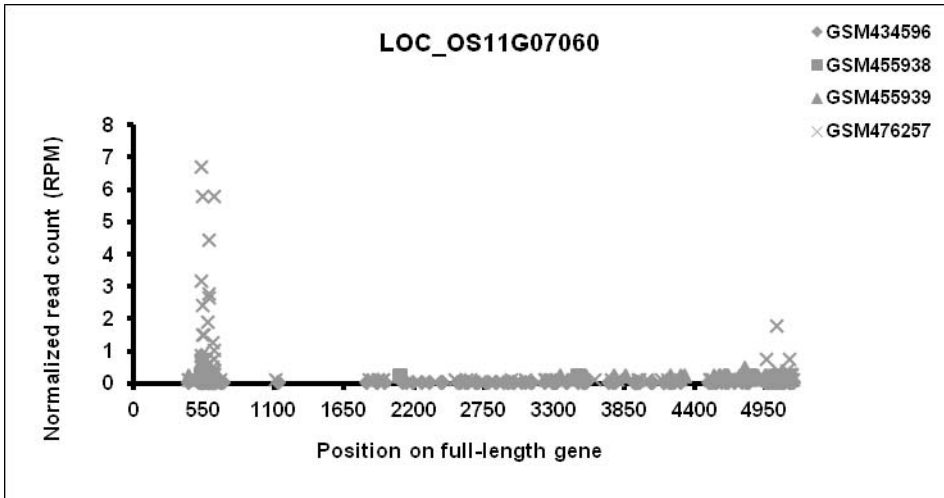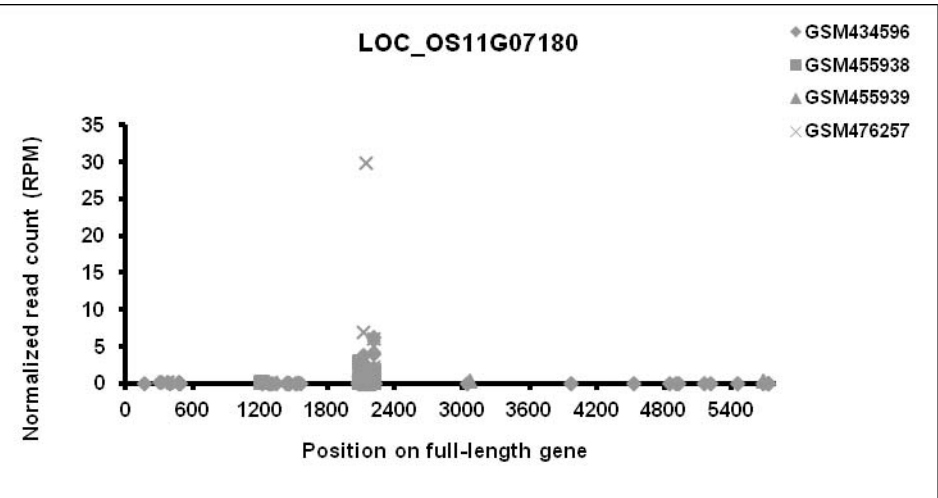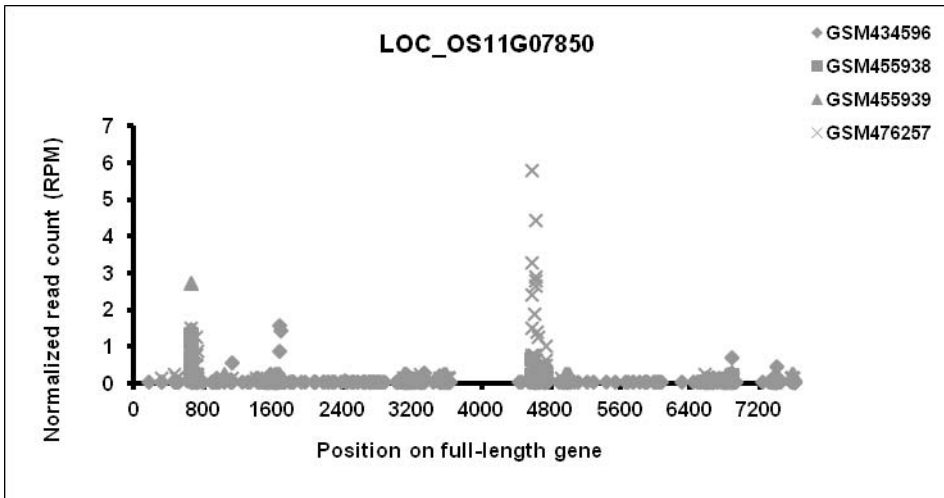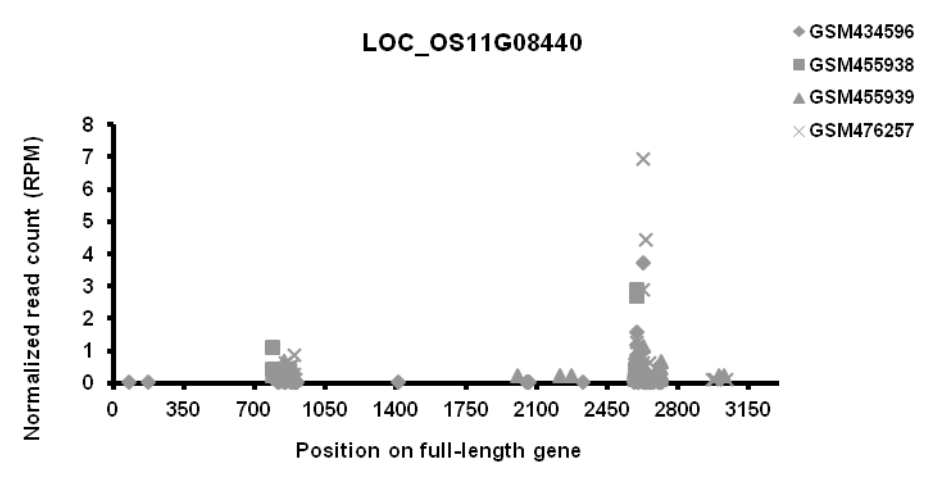

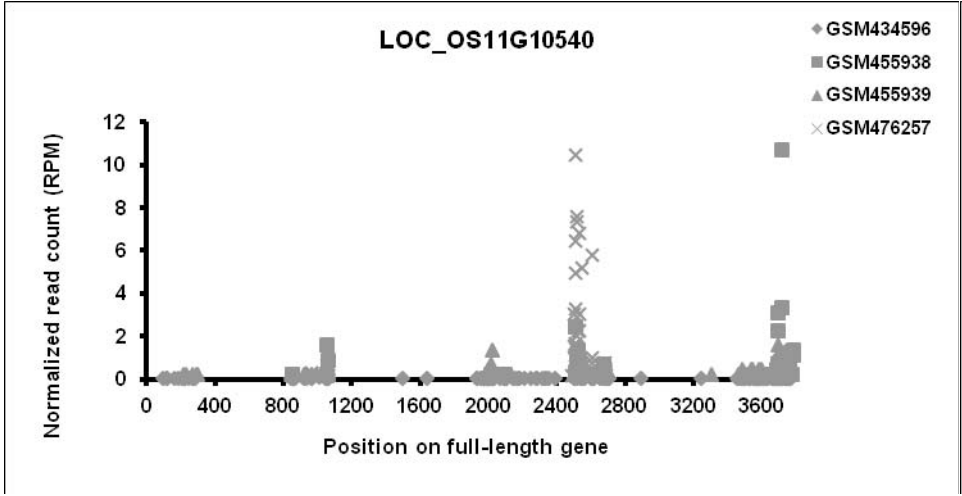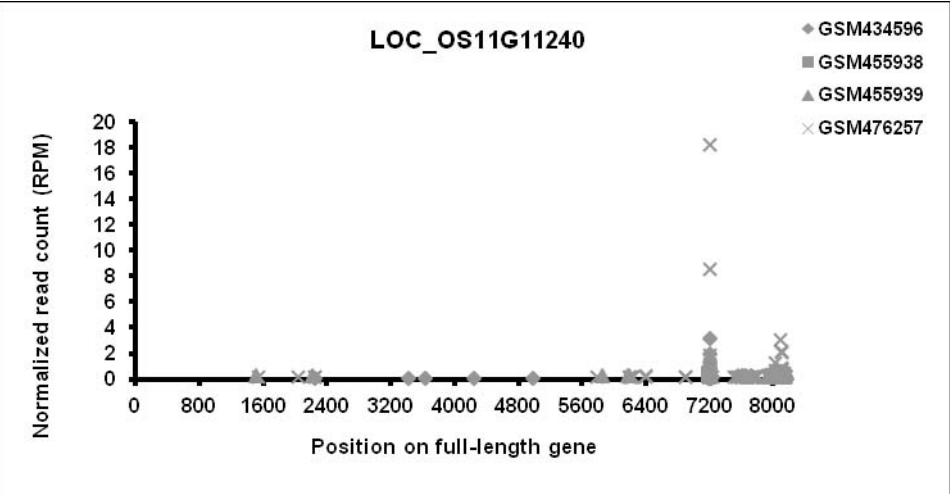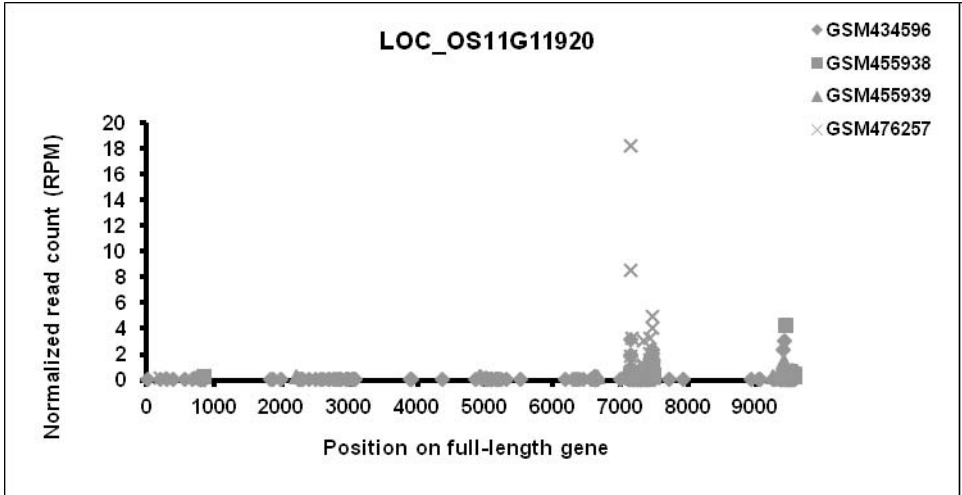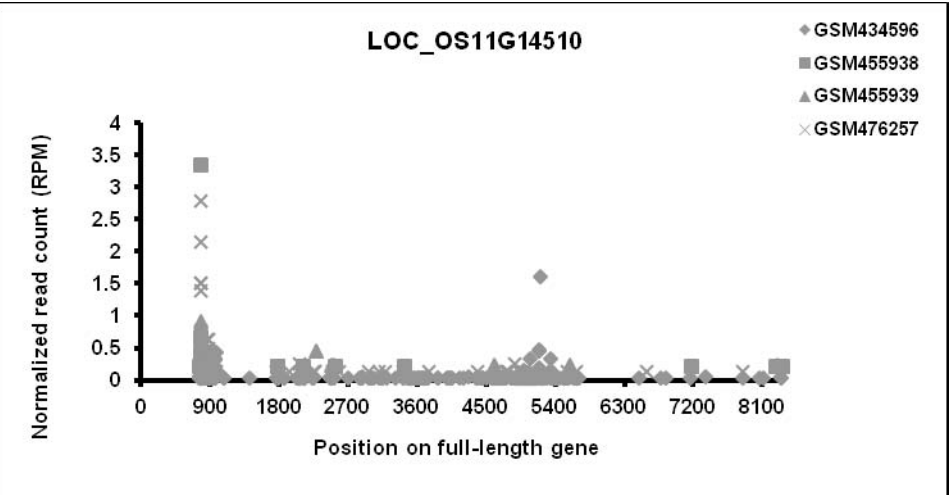

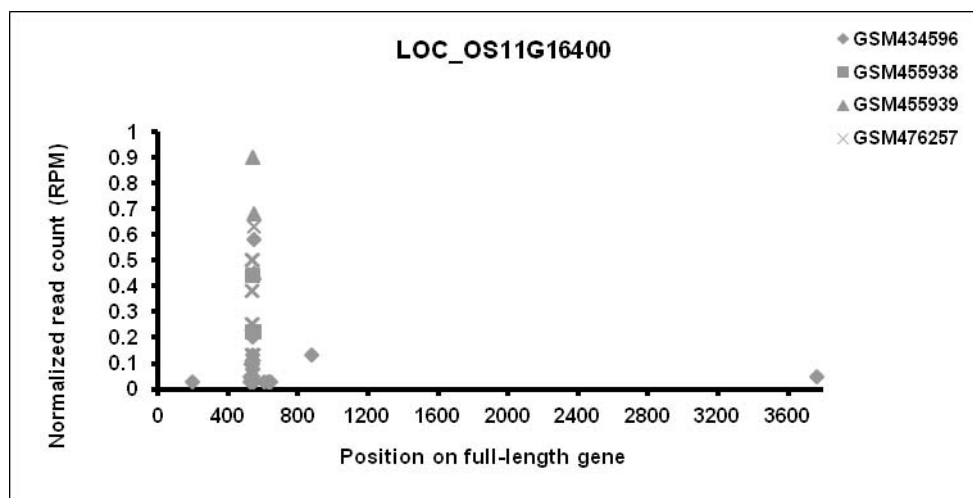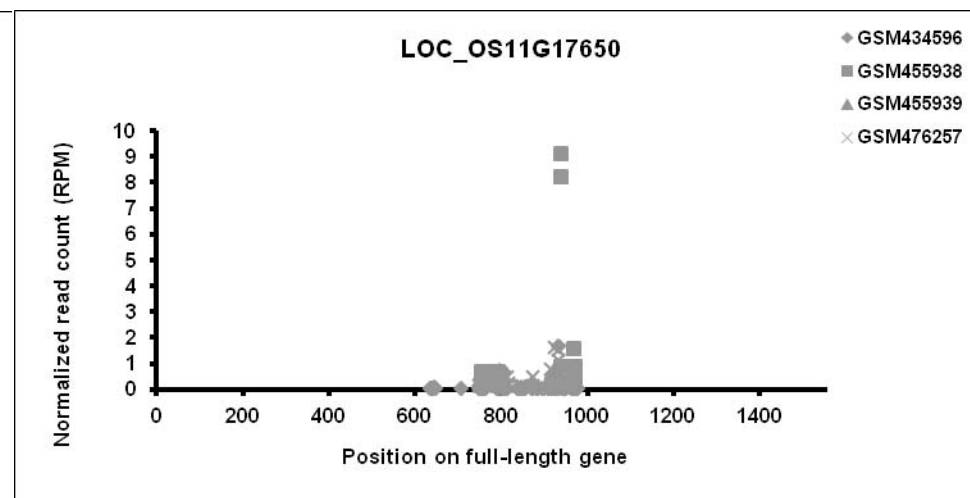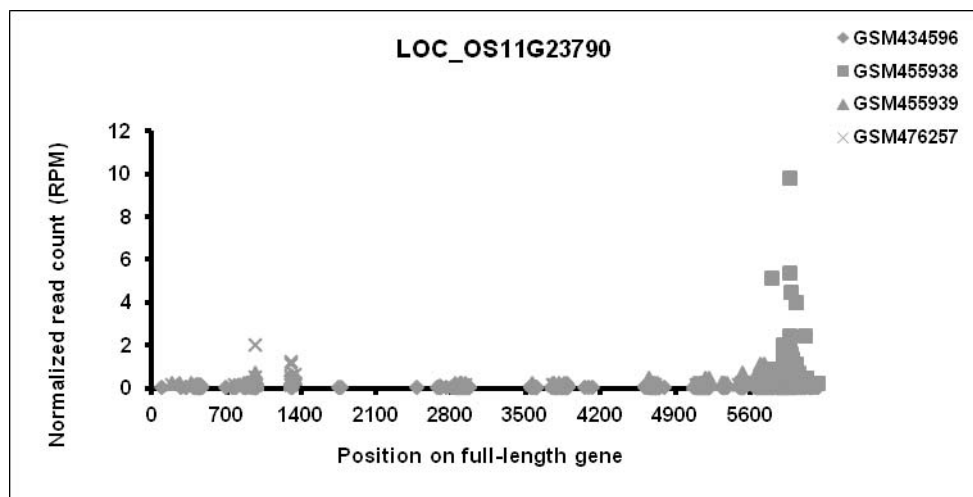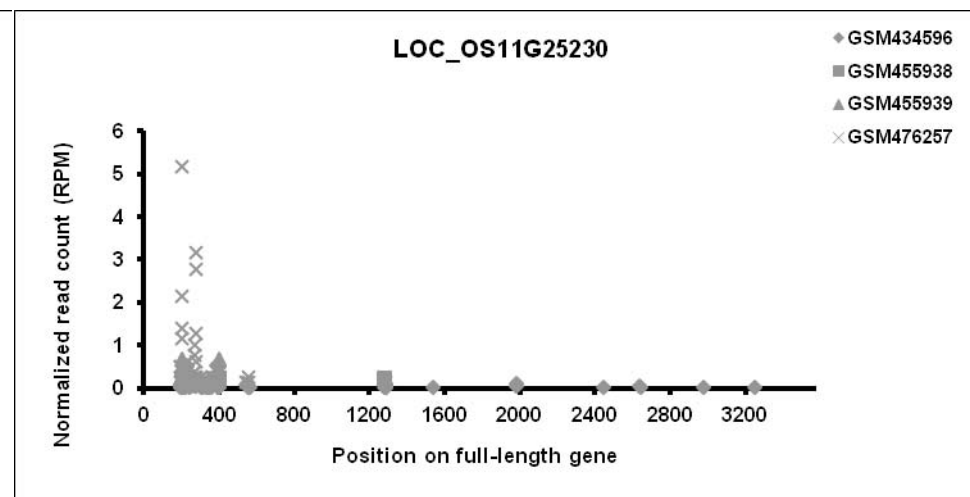

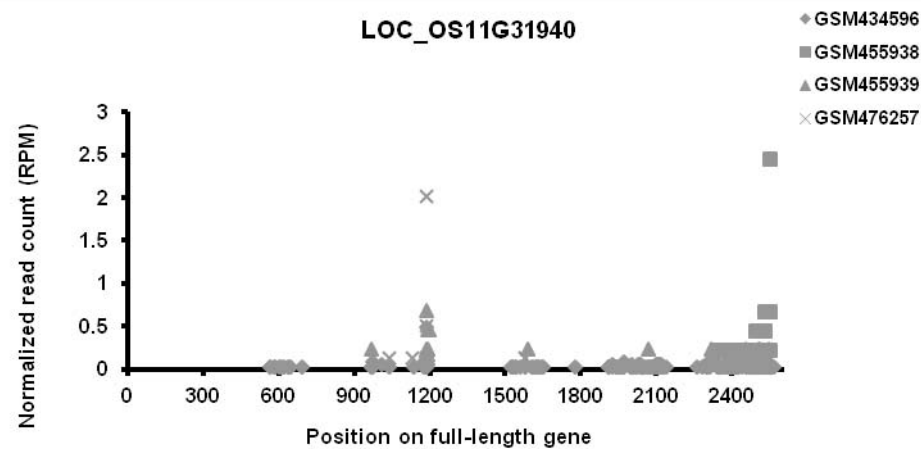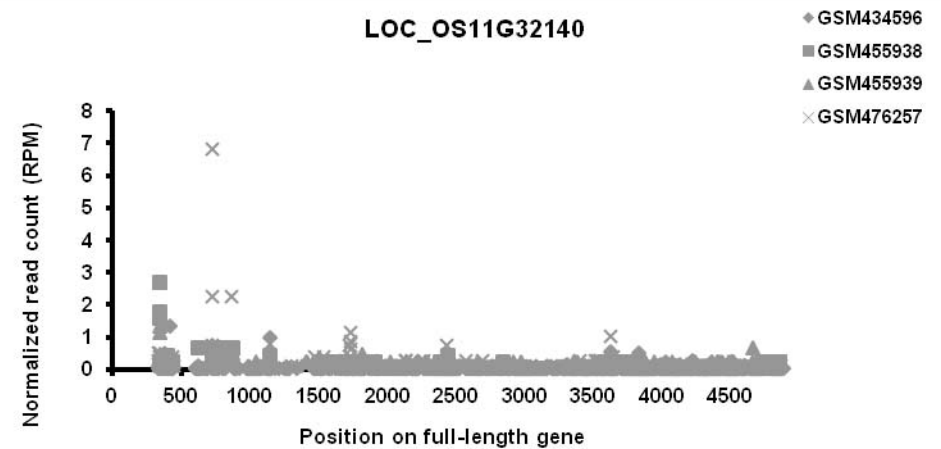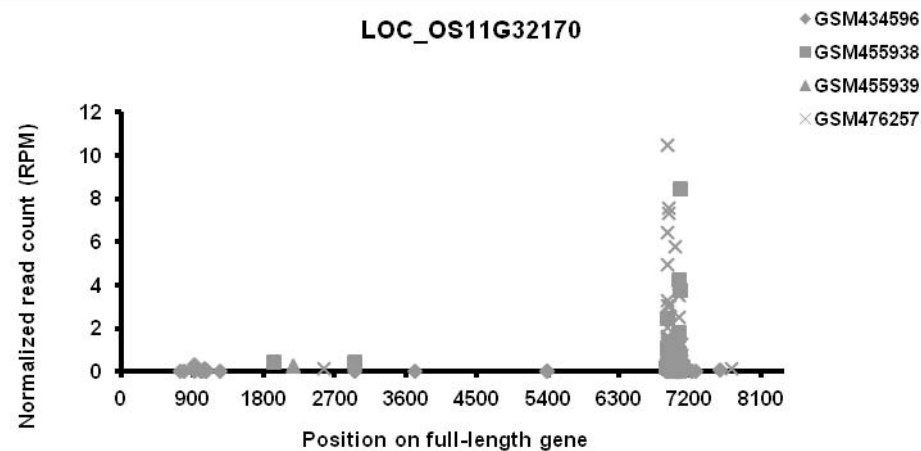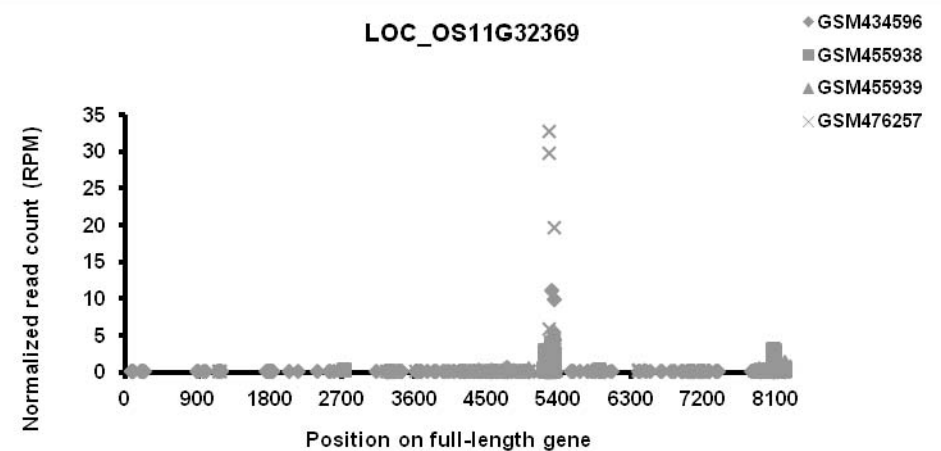

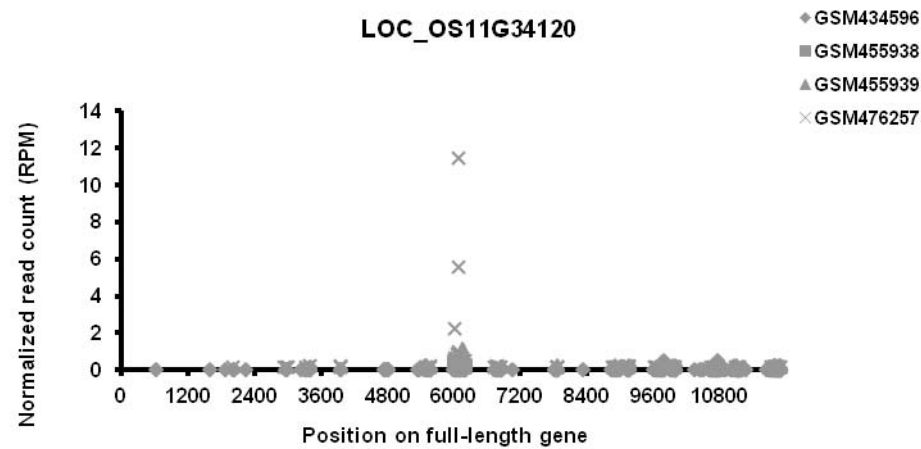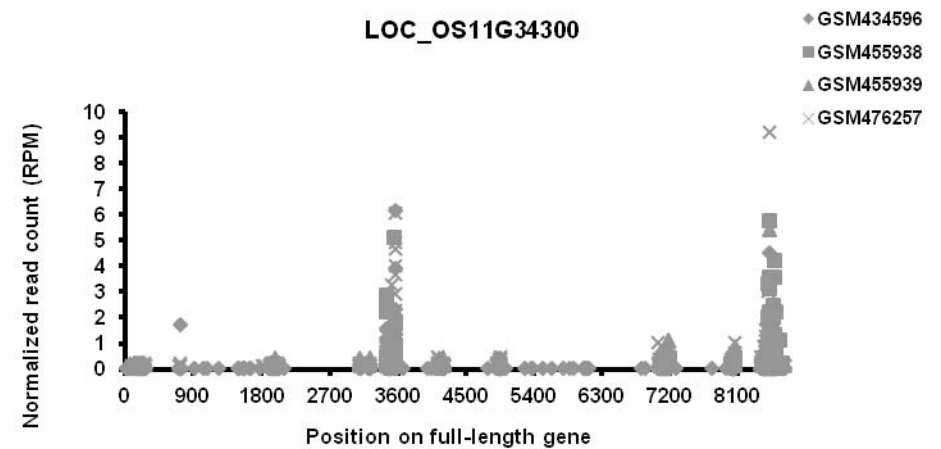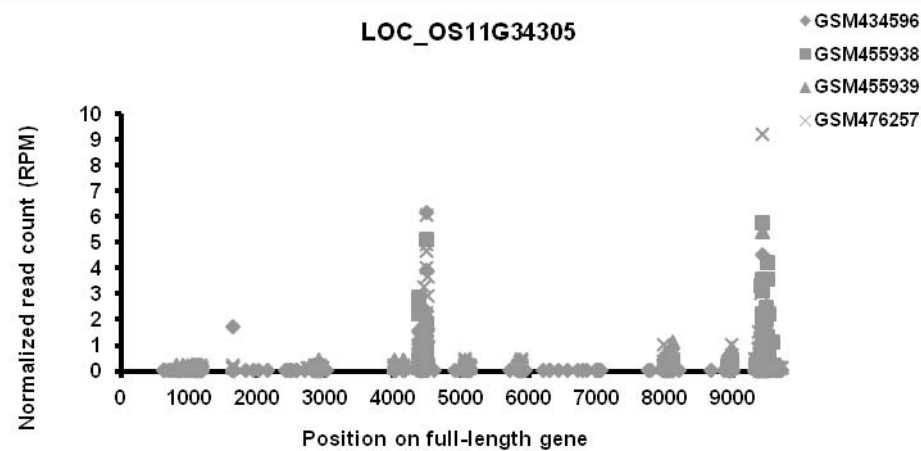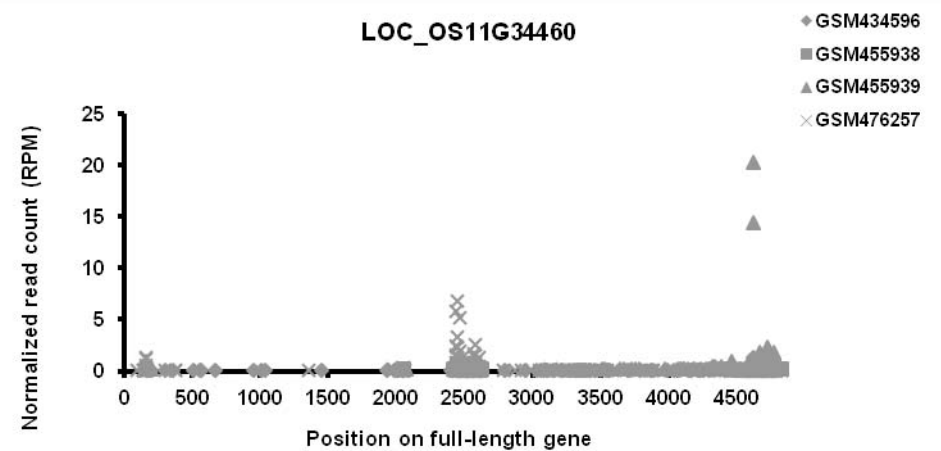

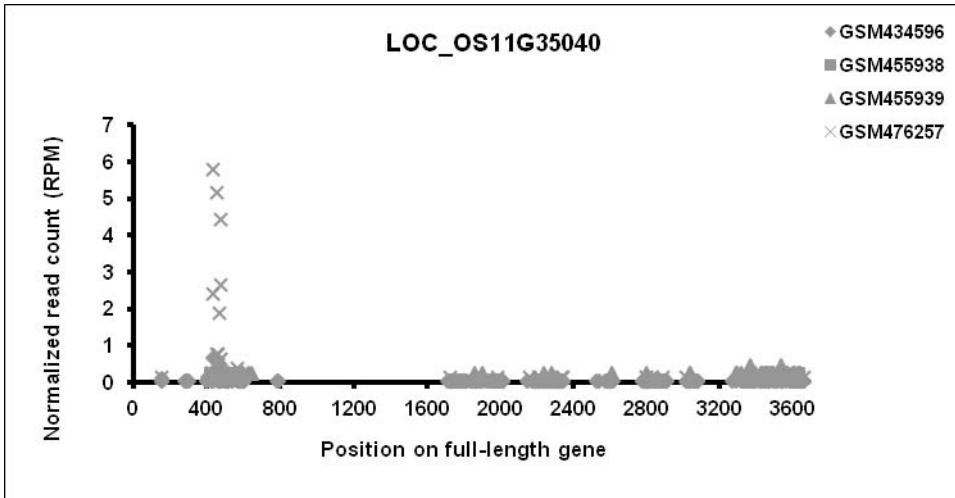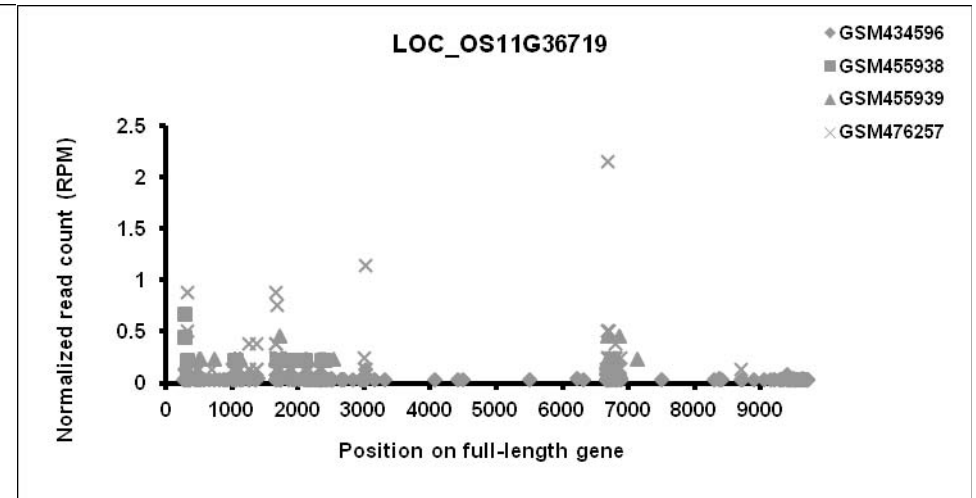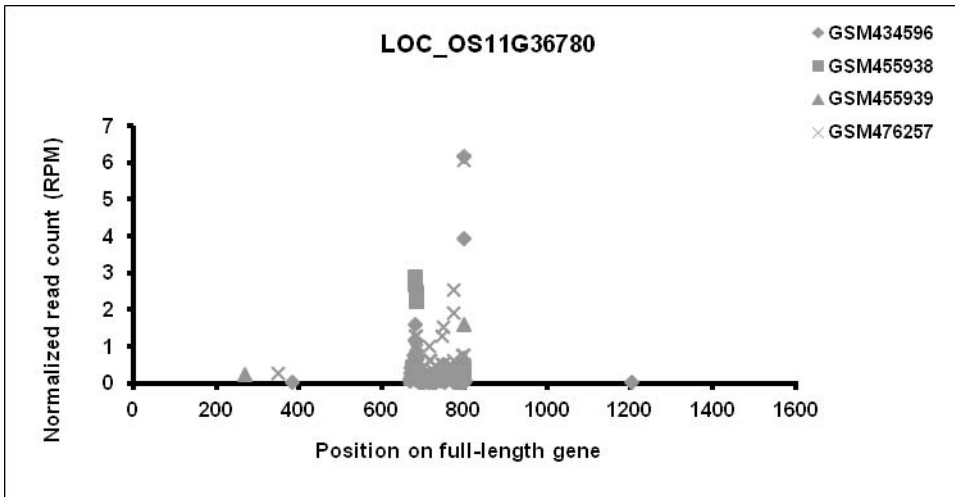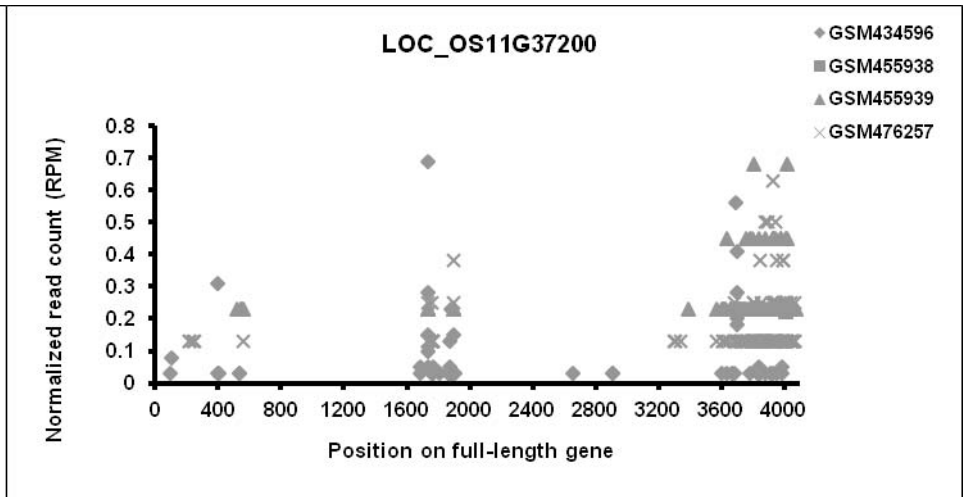

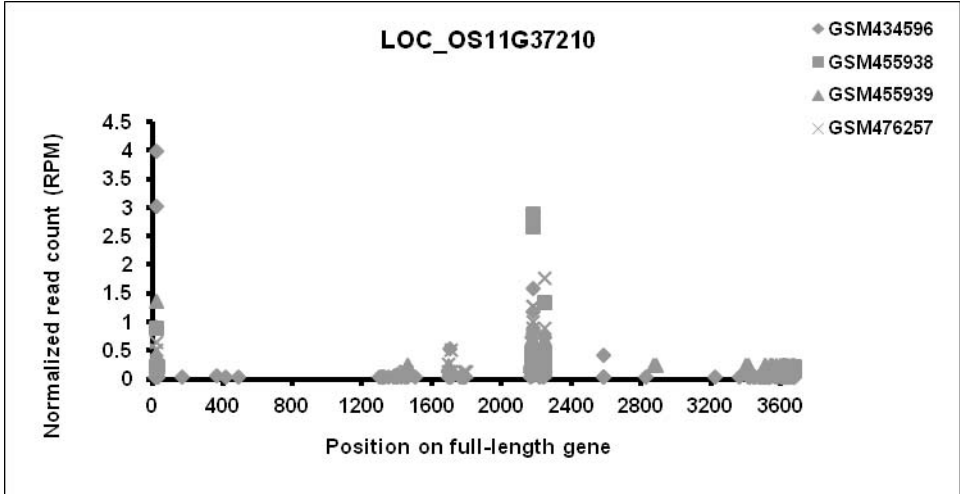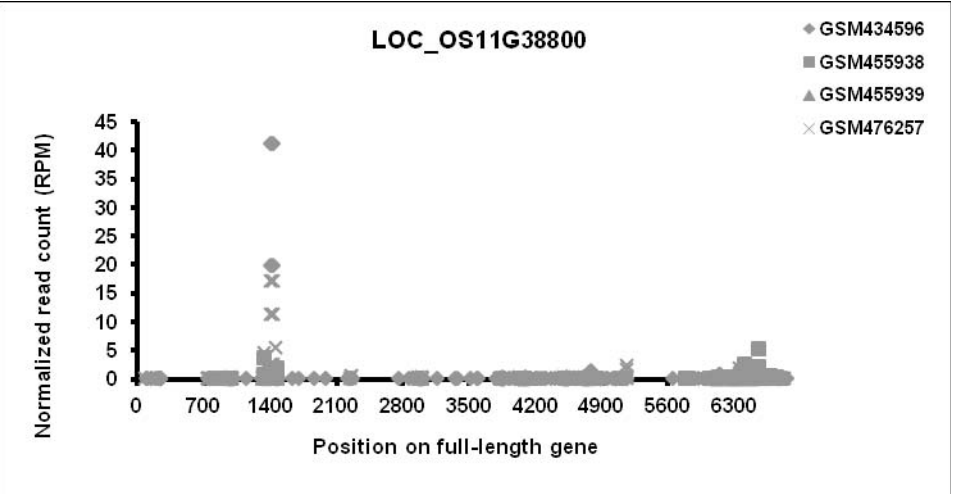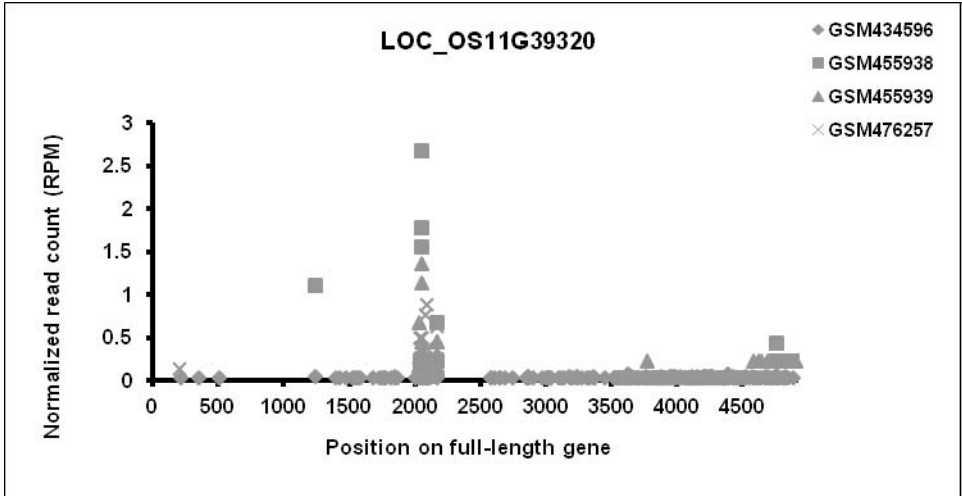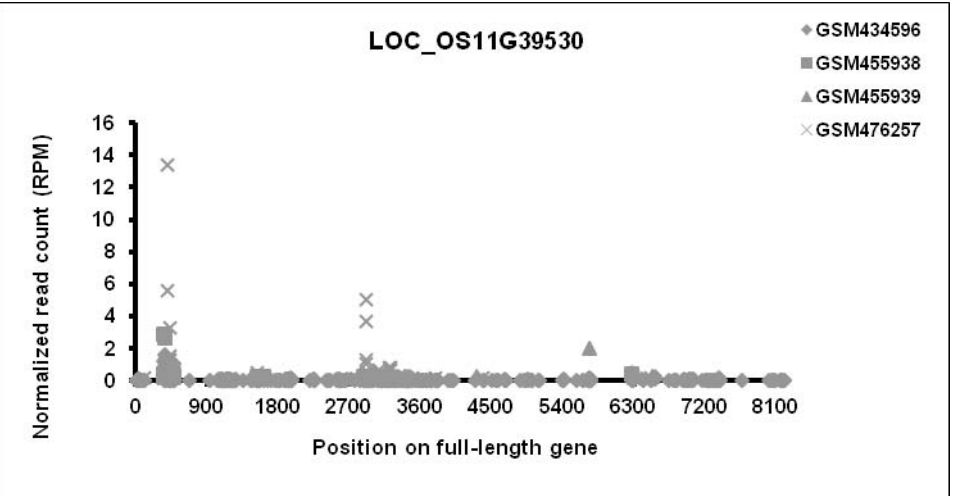

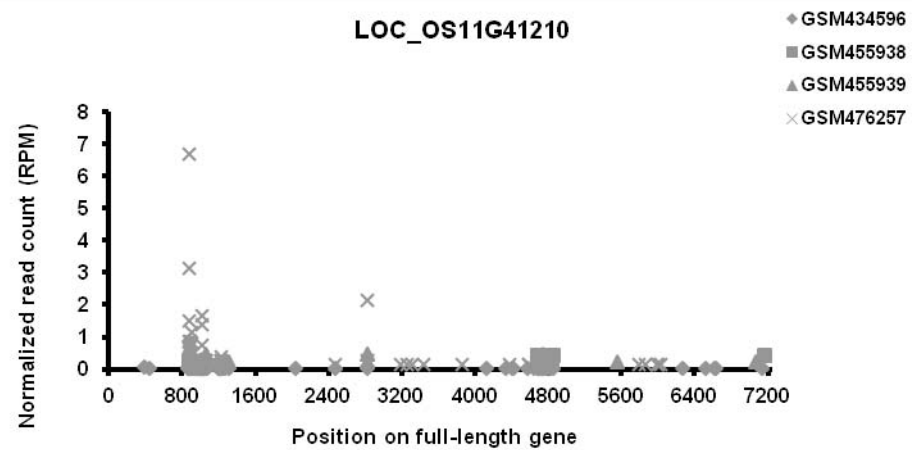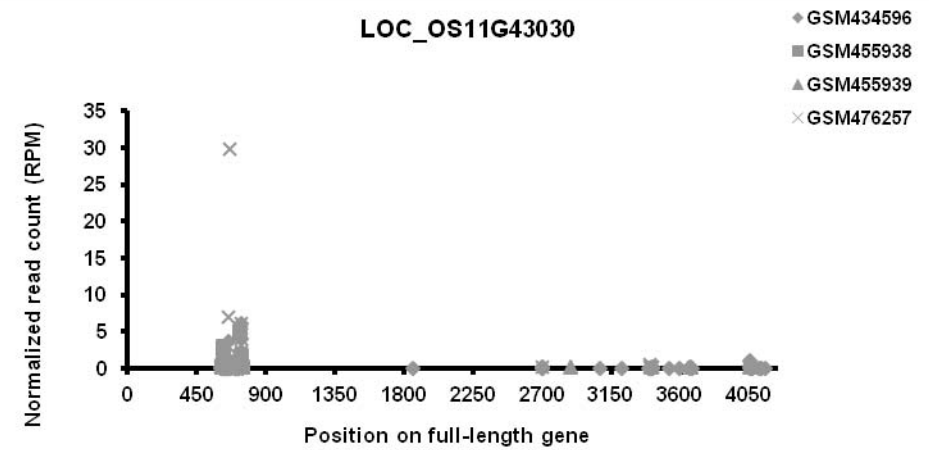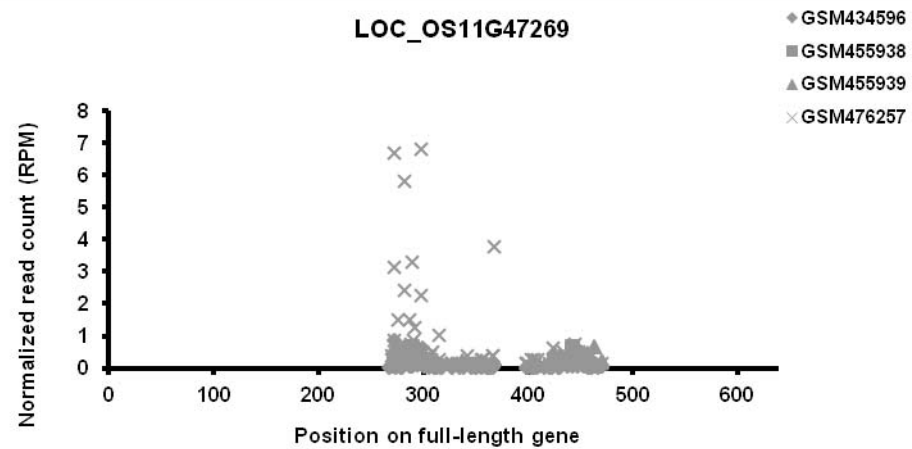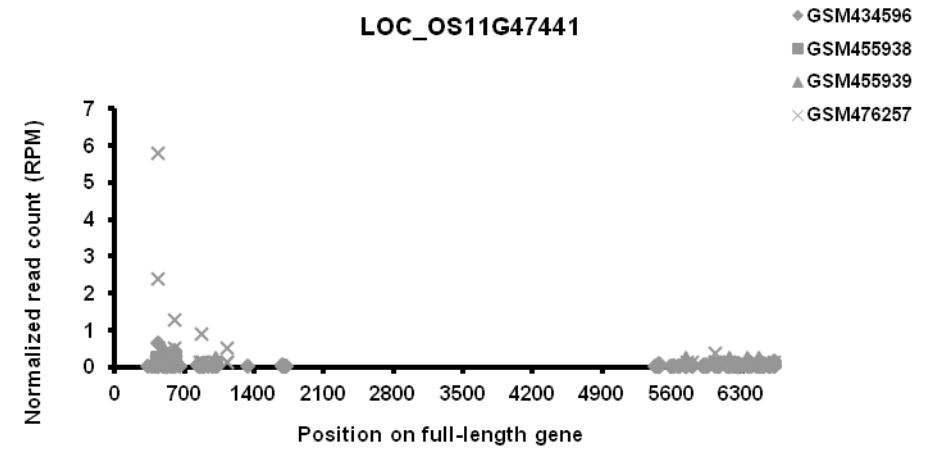

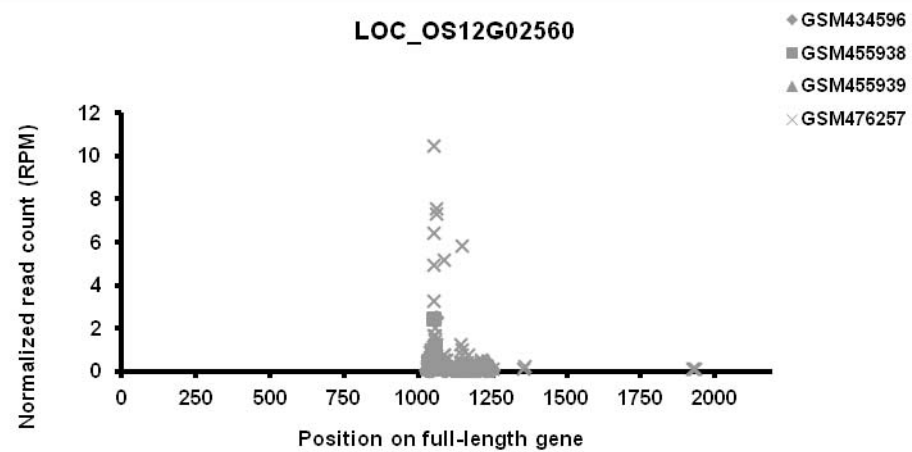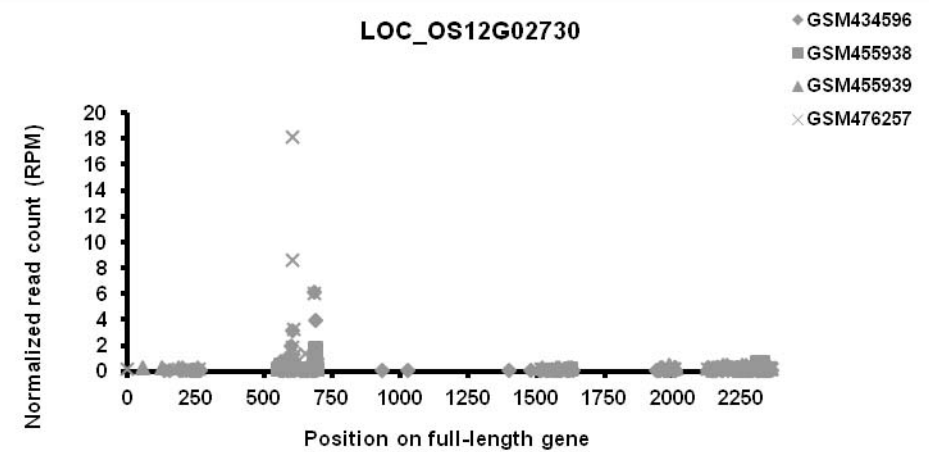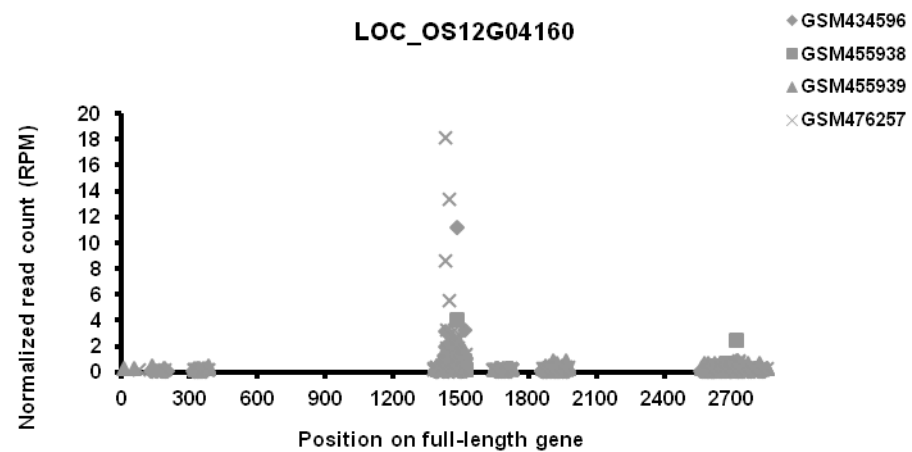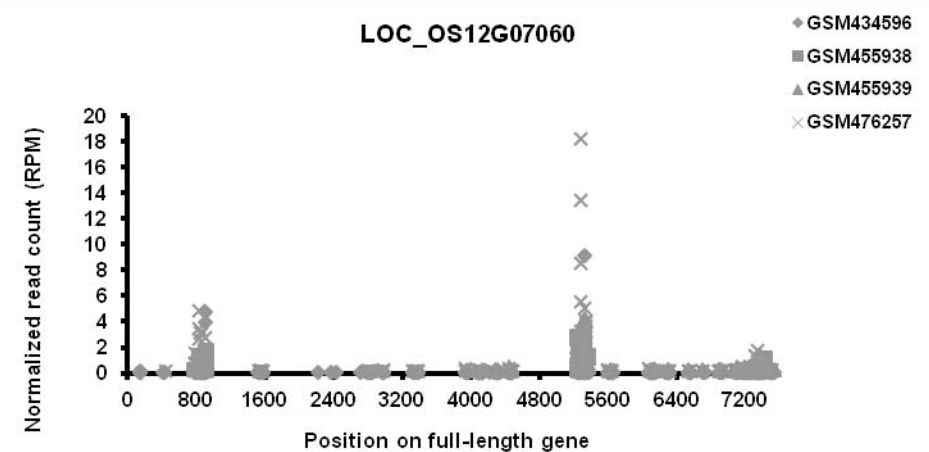

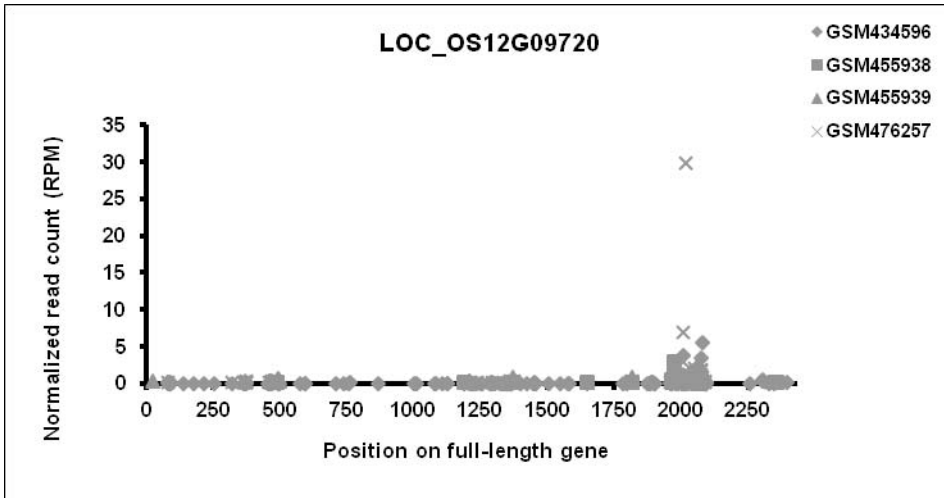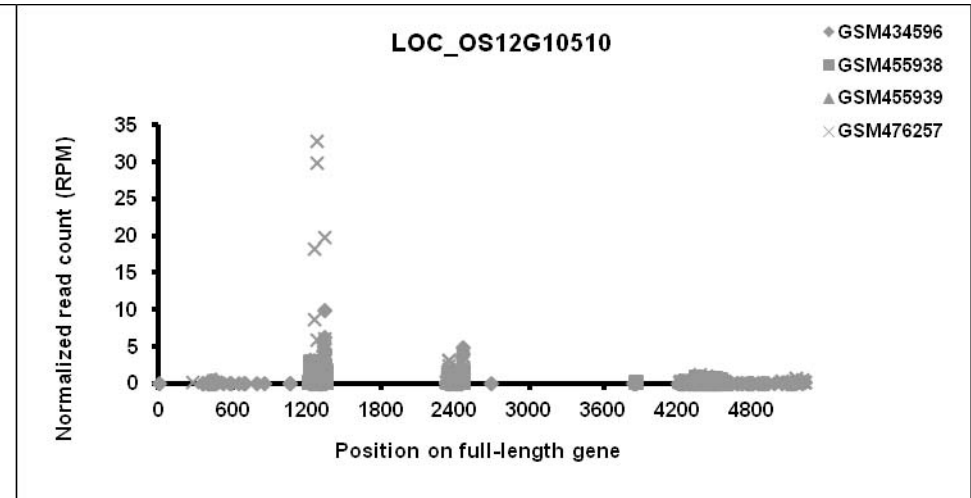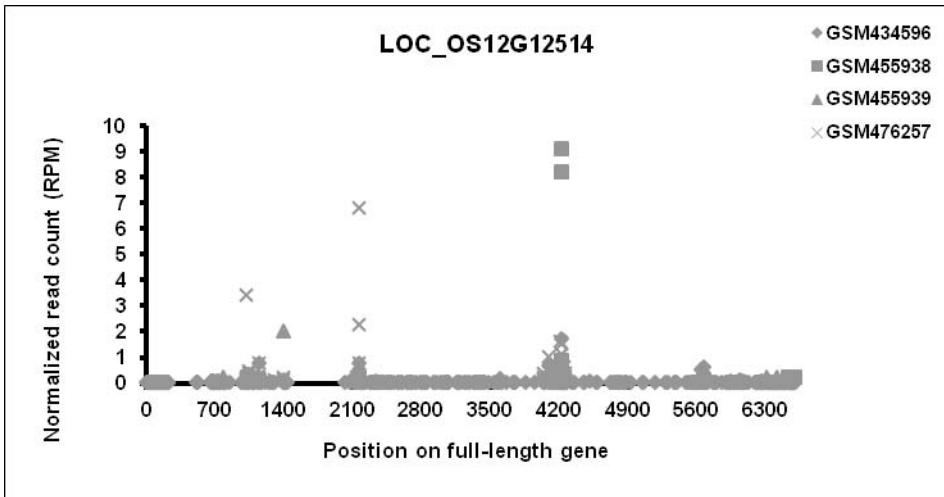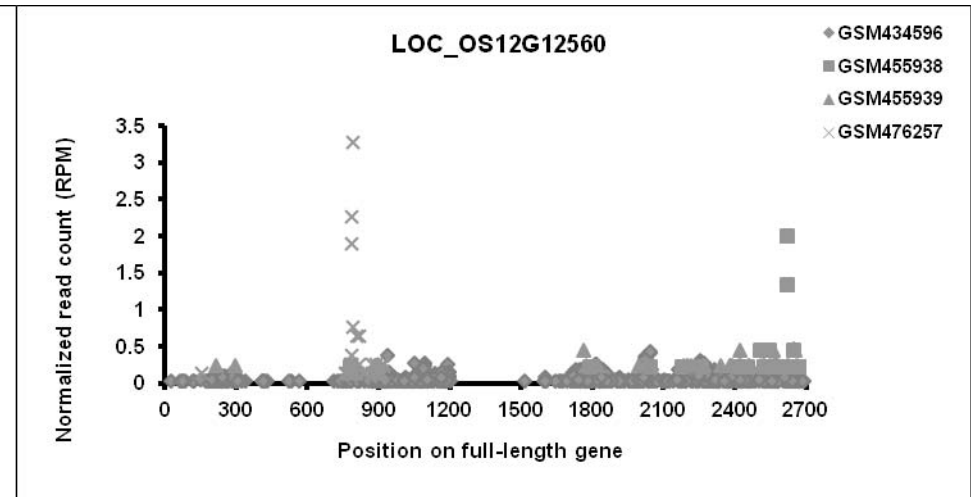

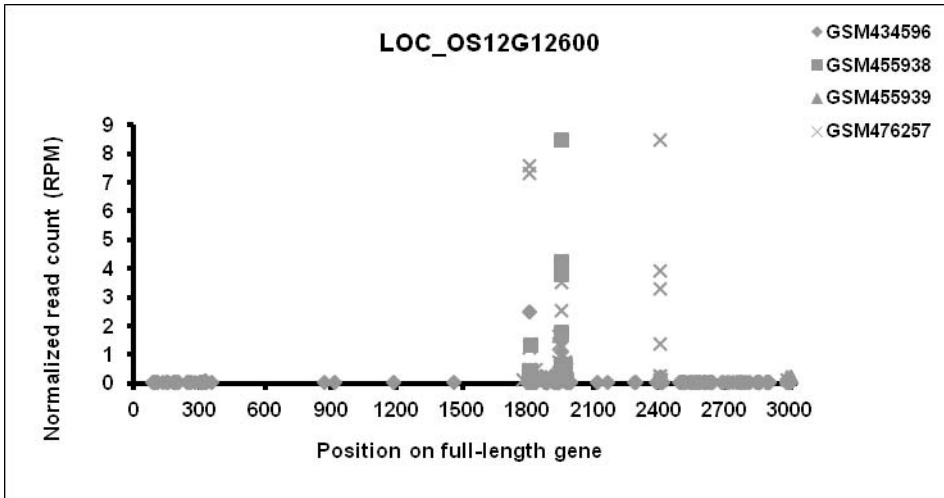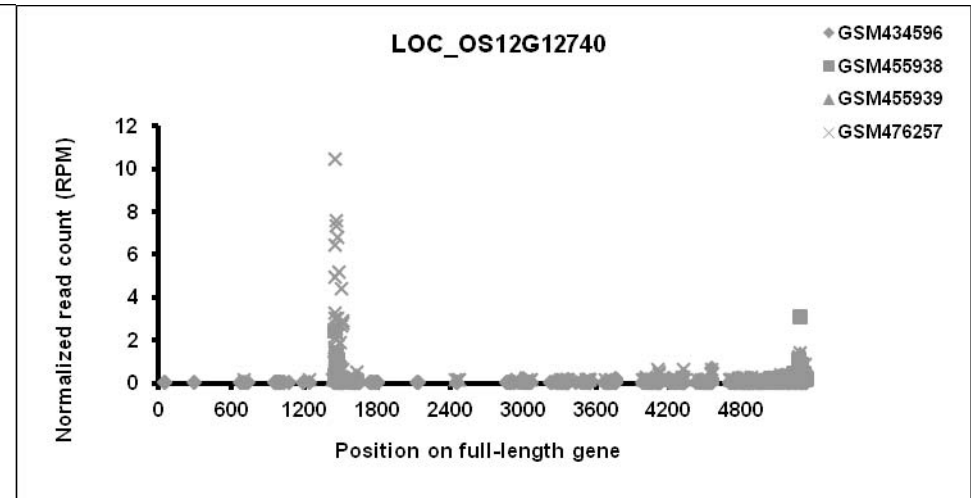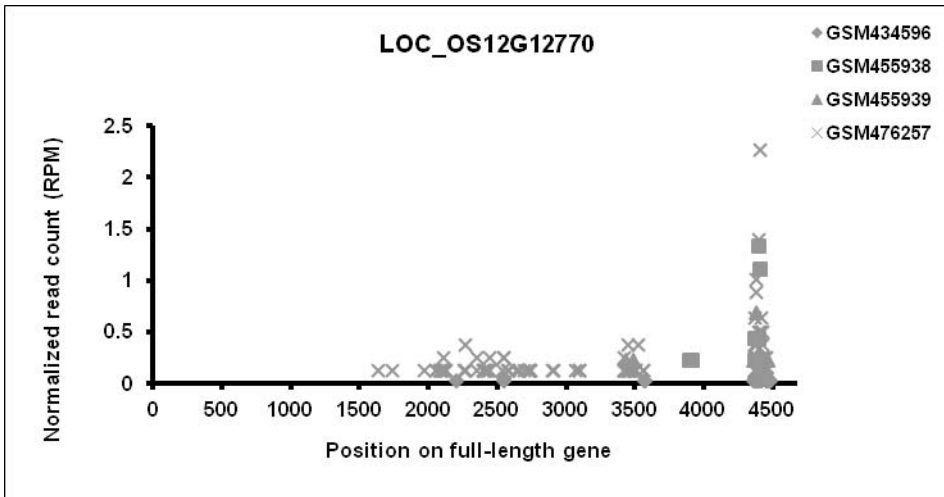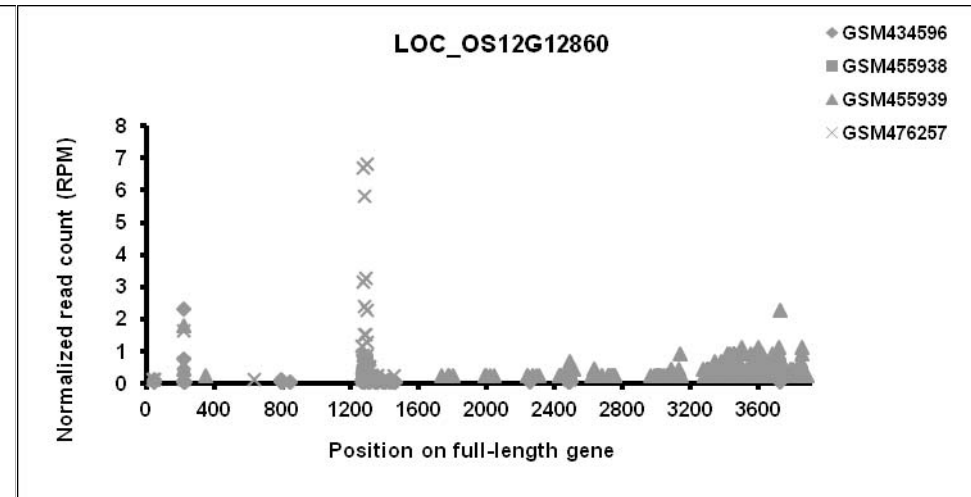

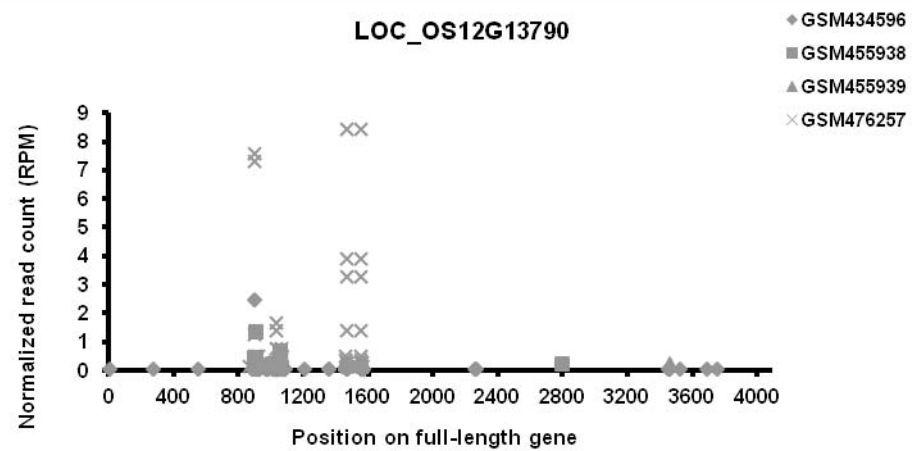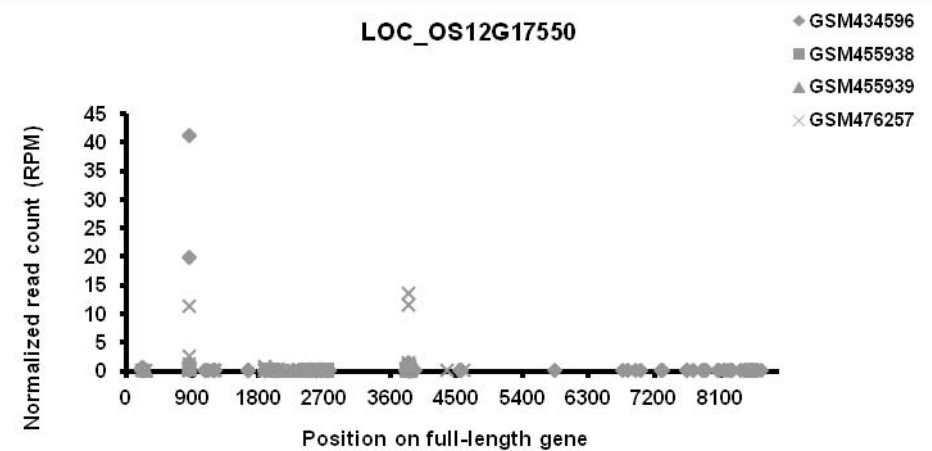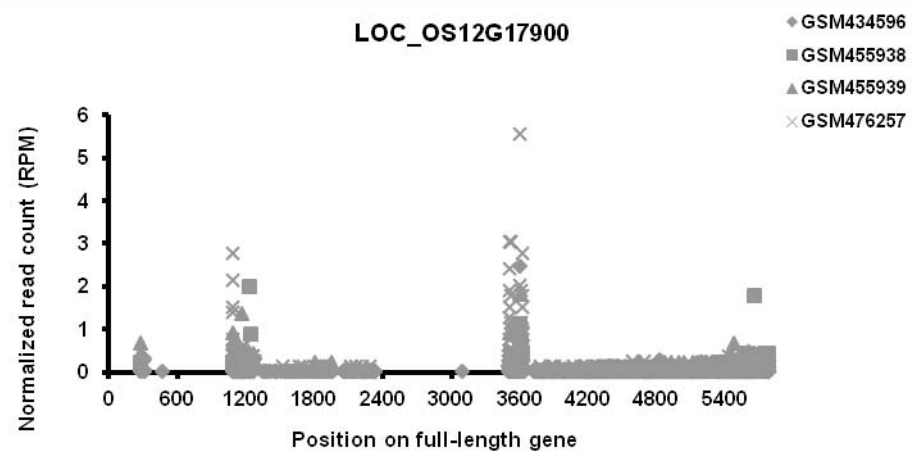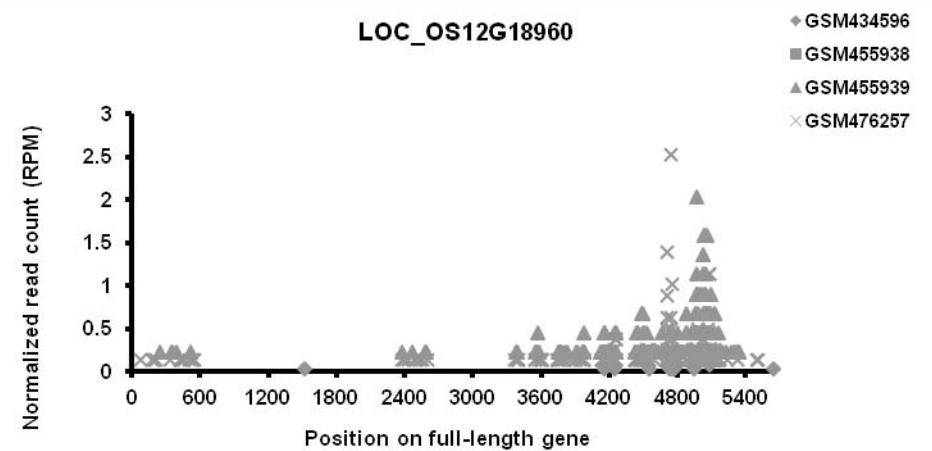

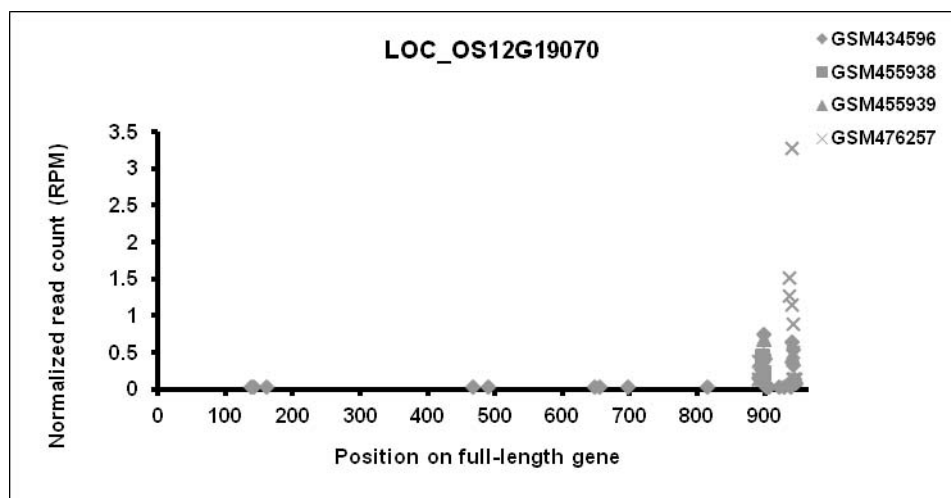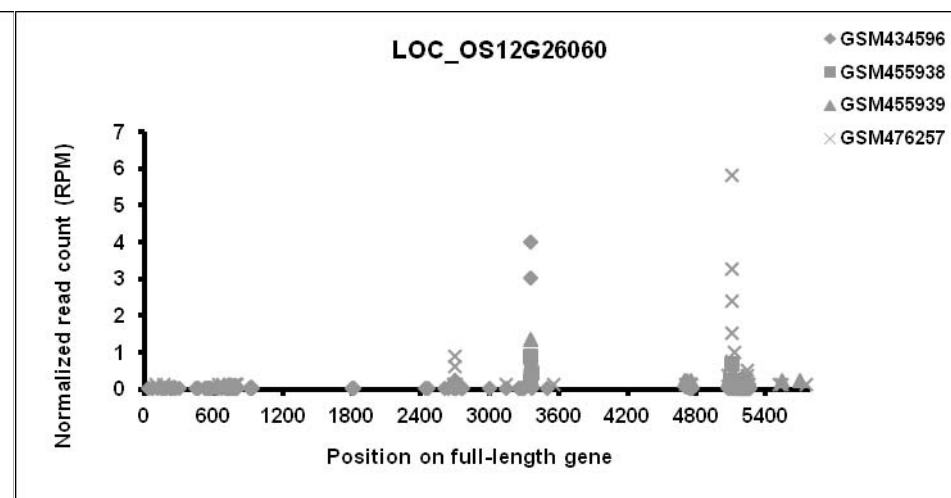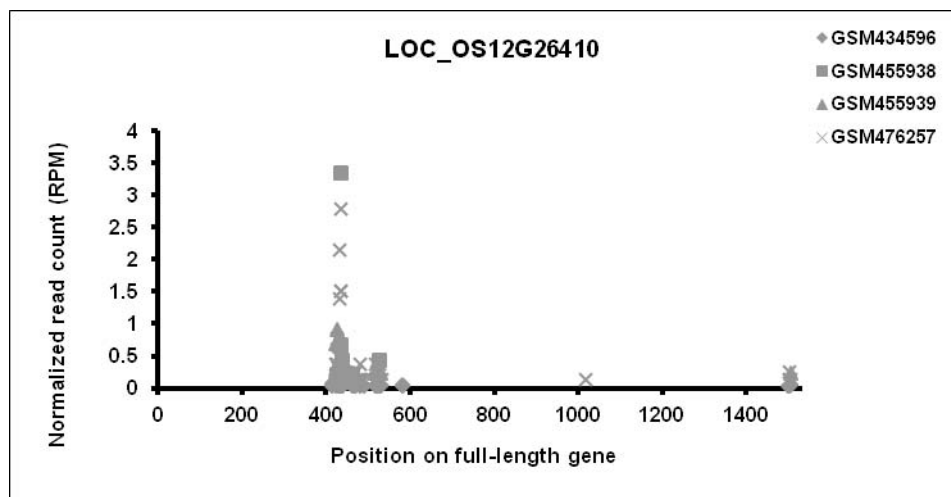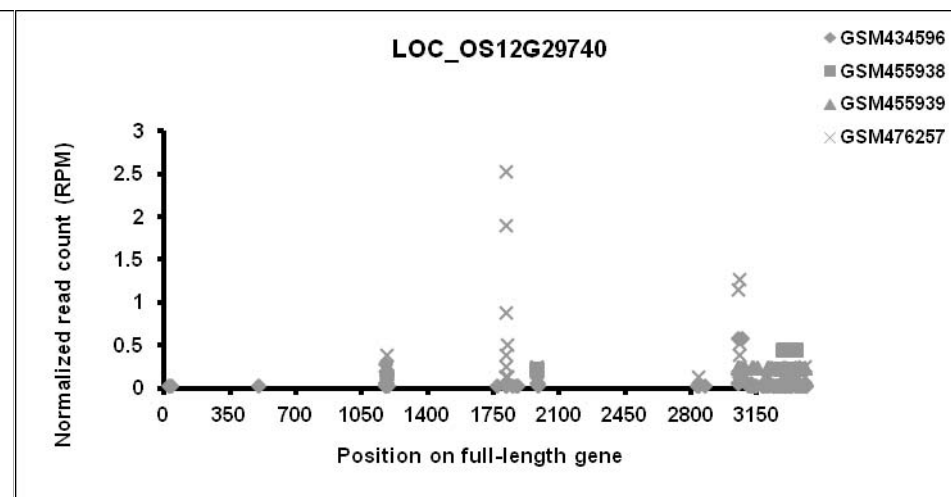

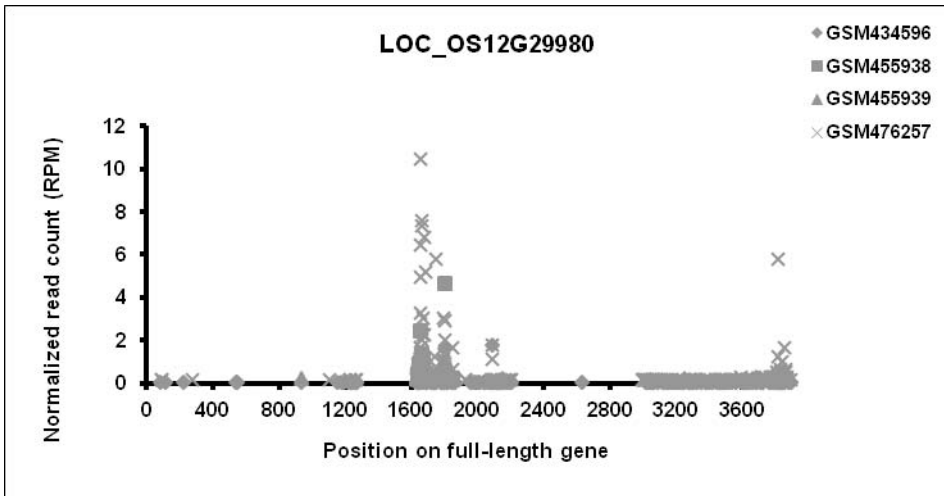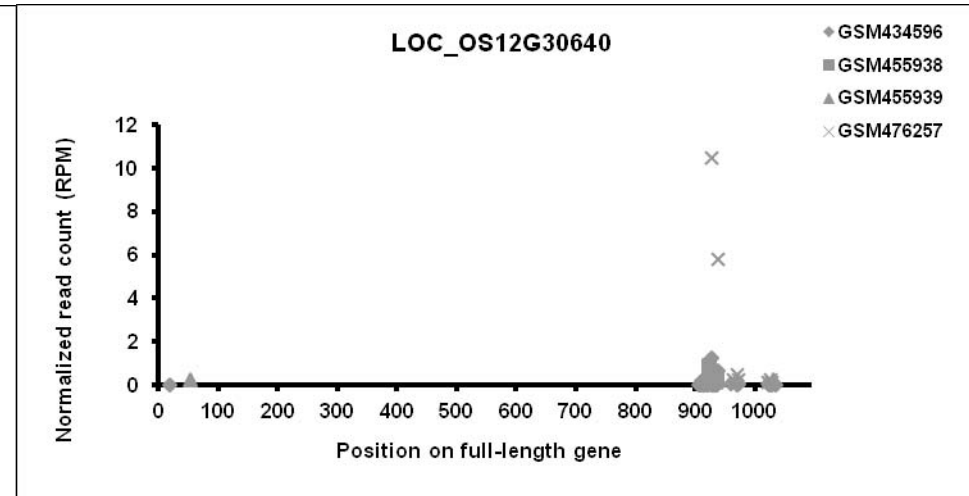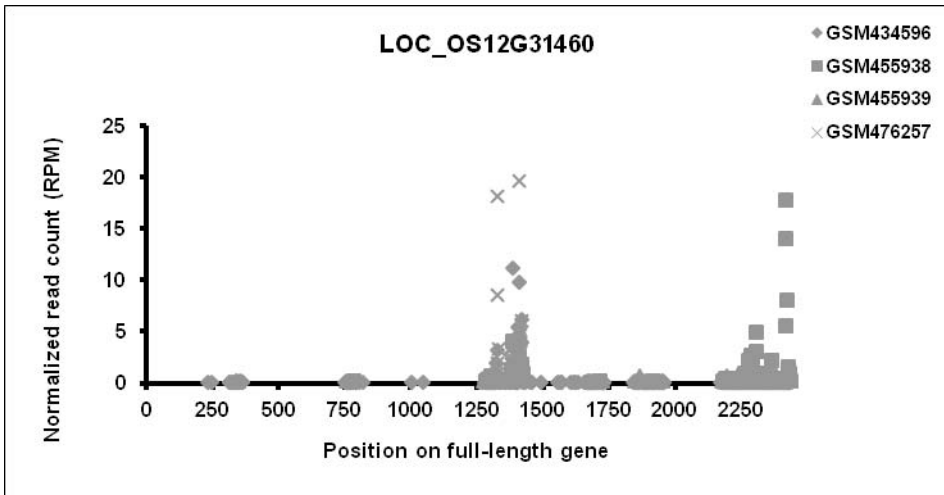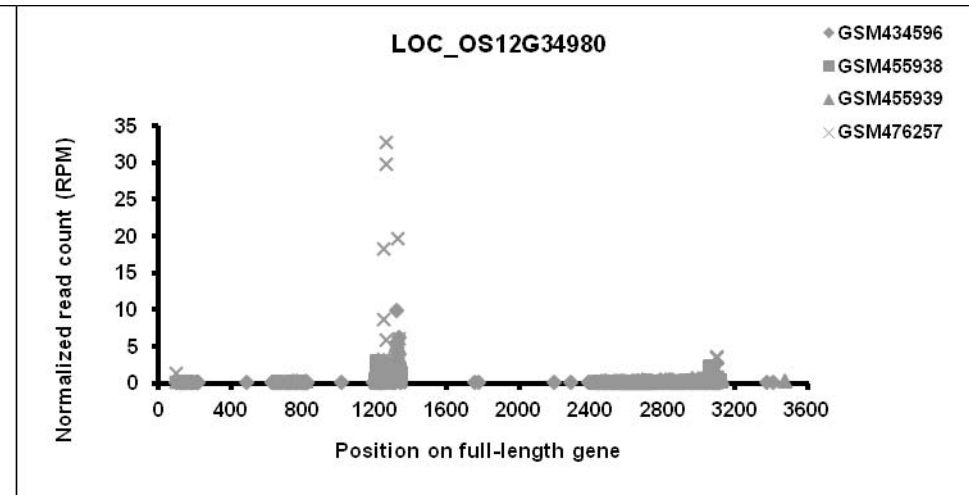

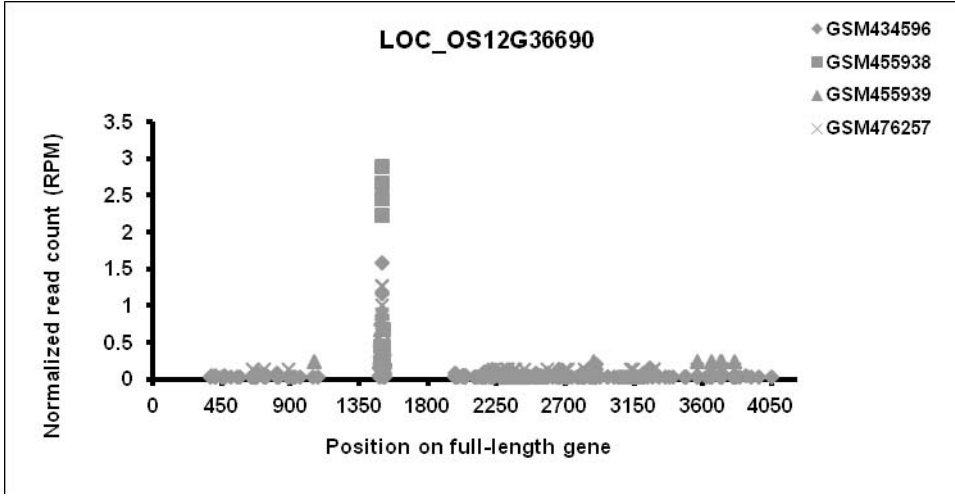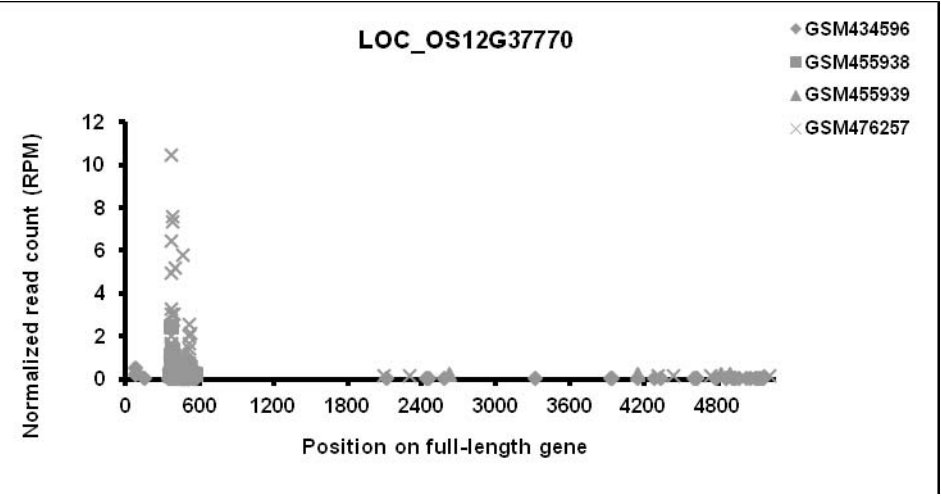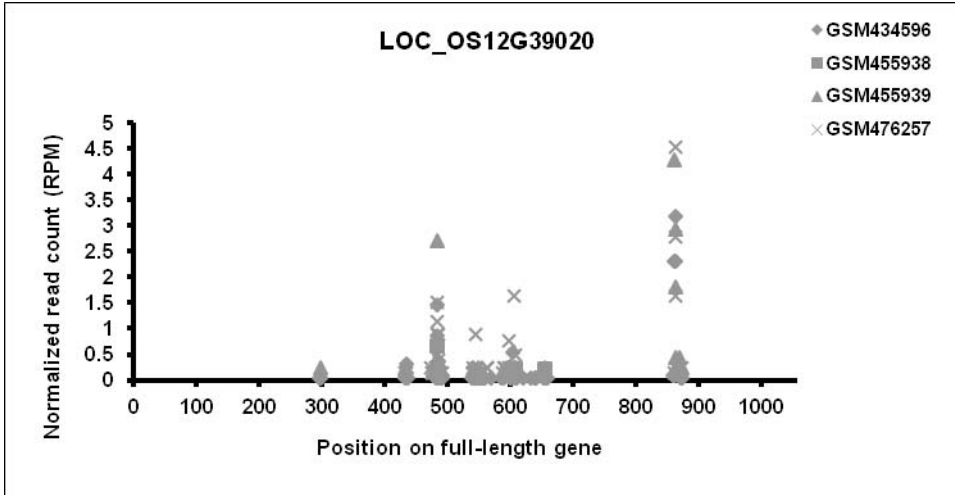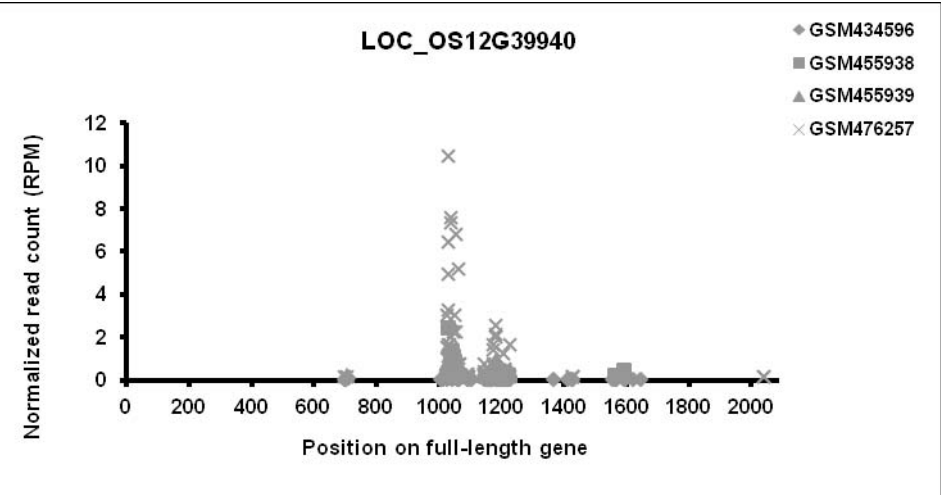

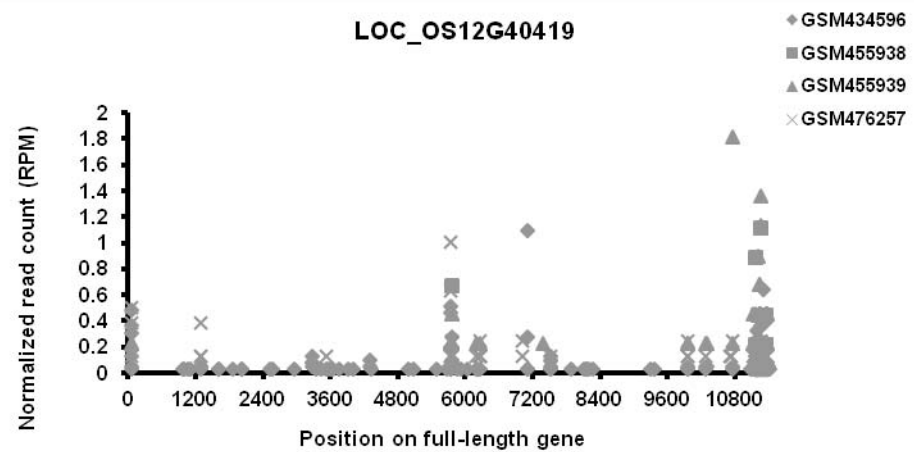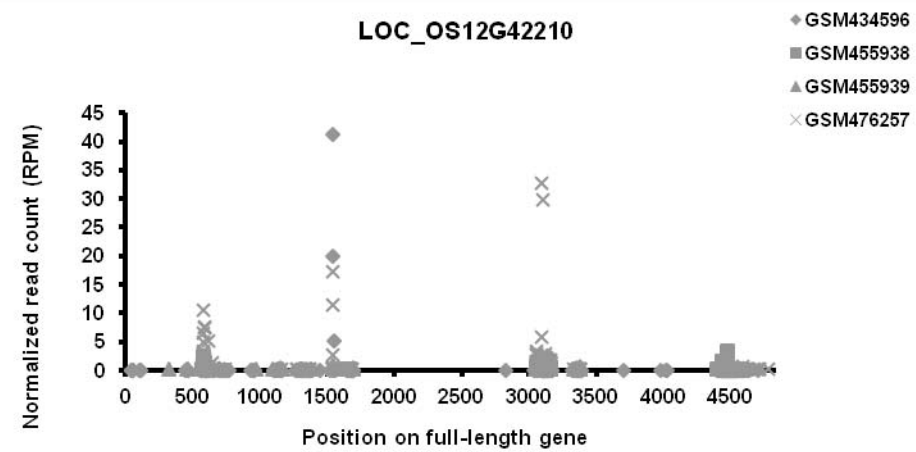

Supplement: Supplementary file 4 — Additional file 4: Figure S4: Global views of the degradome sequencing signatures along the full-length target genes of rice. (PDF 13 MB) [file 12284_2012_45_MOESM4_ESM.pdf]

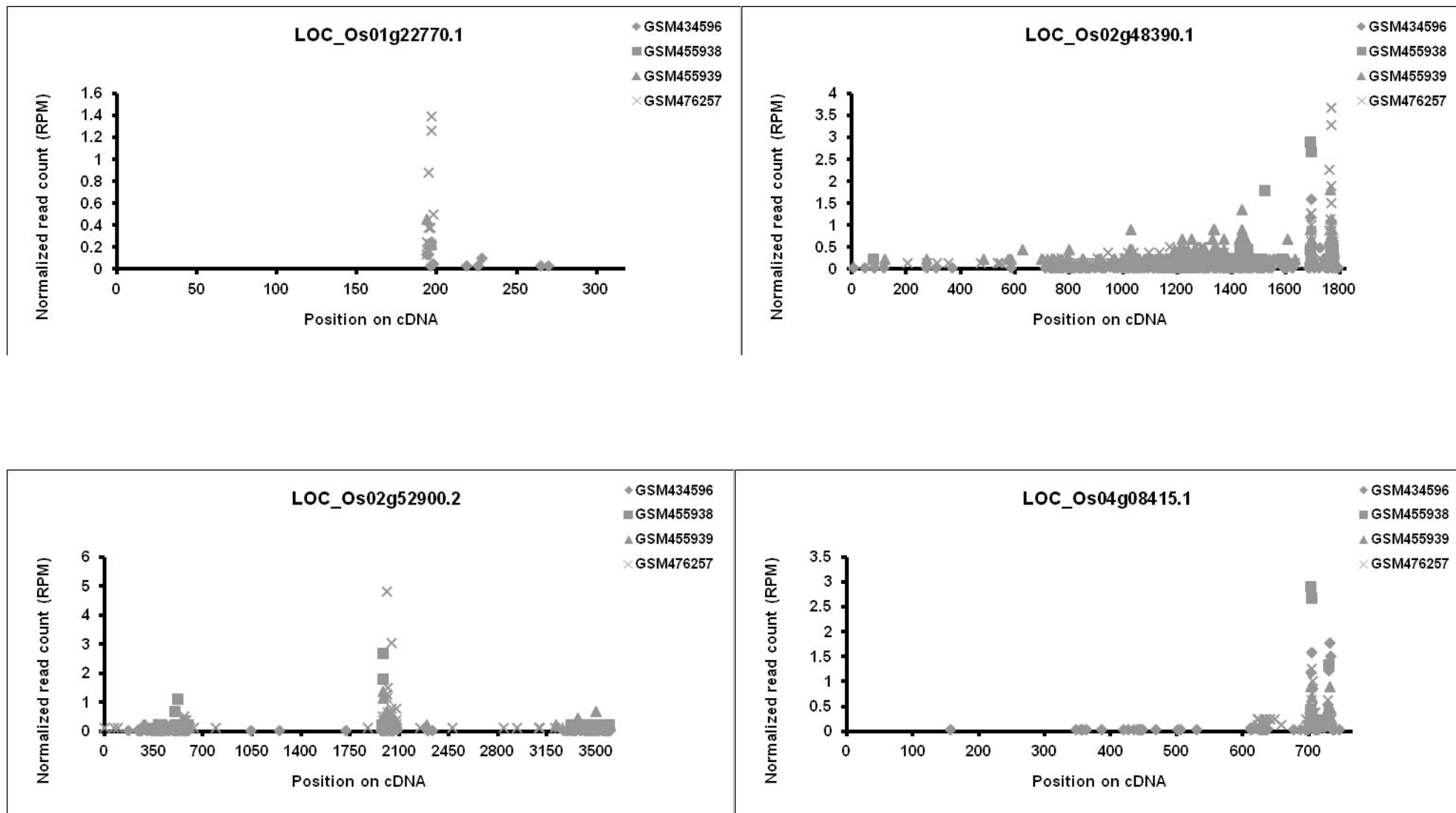

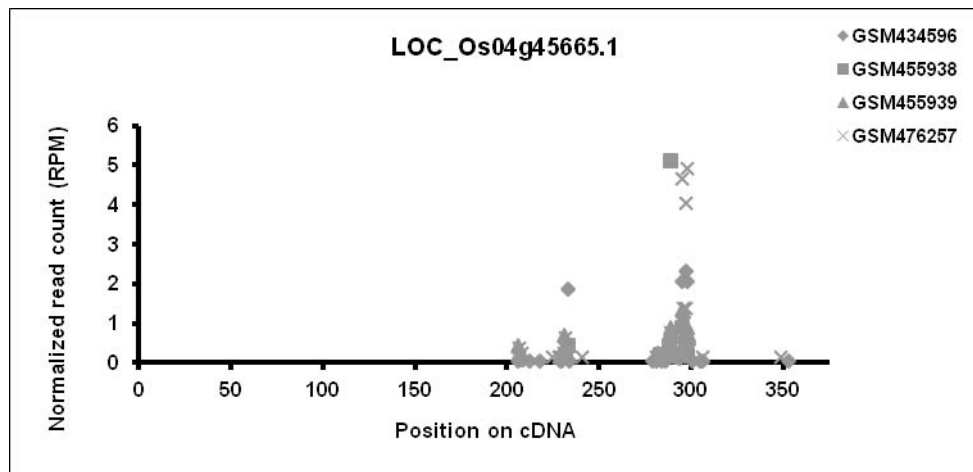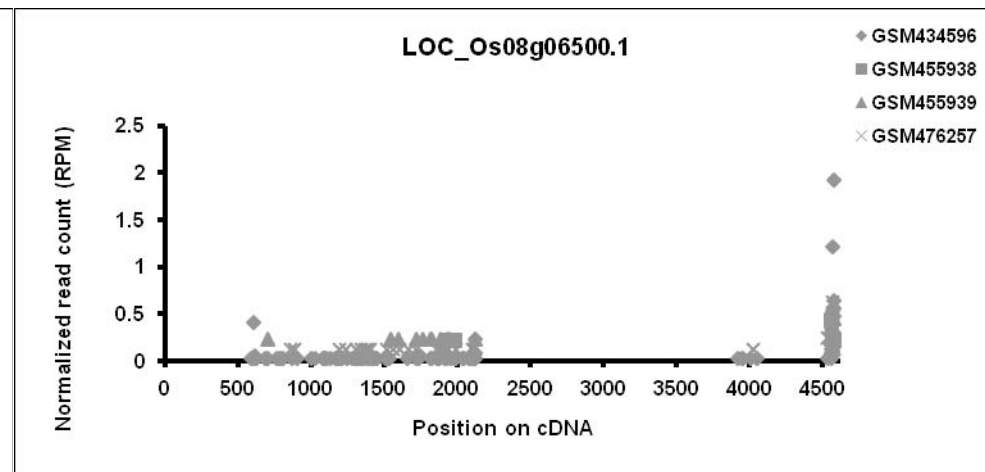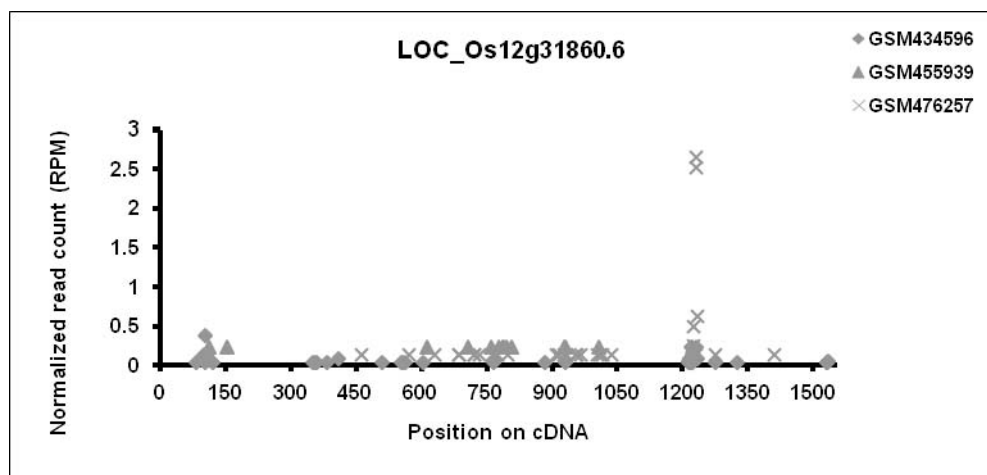

Supplement: Supplementary file 11 — Additional file 11: Figure S6: Global views of the degradome sequencing signatures along the mRNAs targeted by specific phased small RNAs in rice. (PDF 190 KB) [file 12284_2012_45_MOESM11_ESM.pdf]

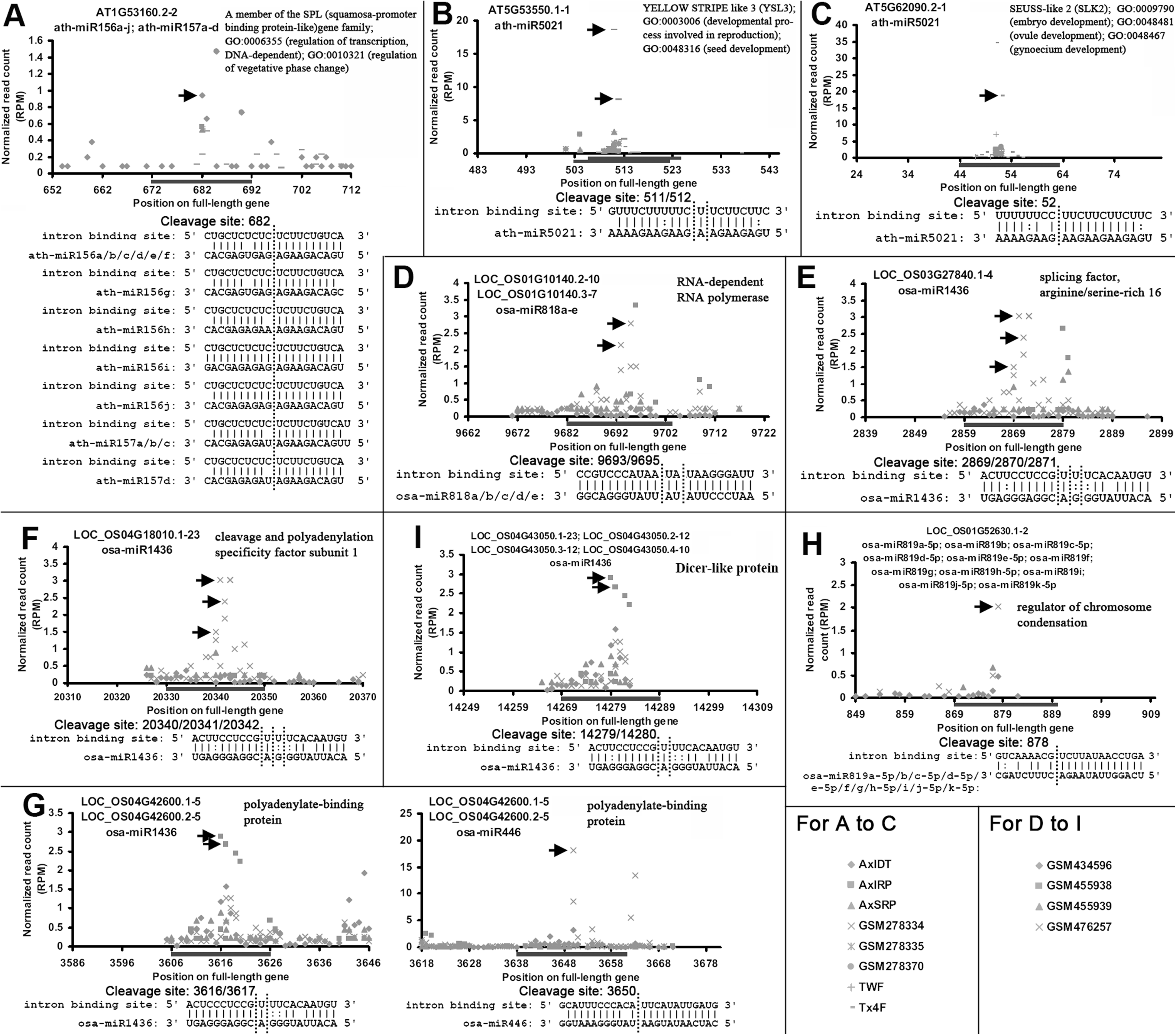

Supplement: Supplementary file 12 — Authors’ original file for figure 1 [file 12284_2012_45_MOESM12_ESM.tiff]

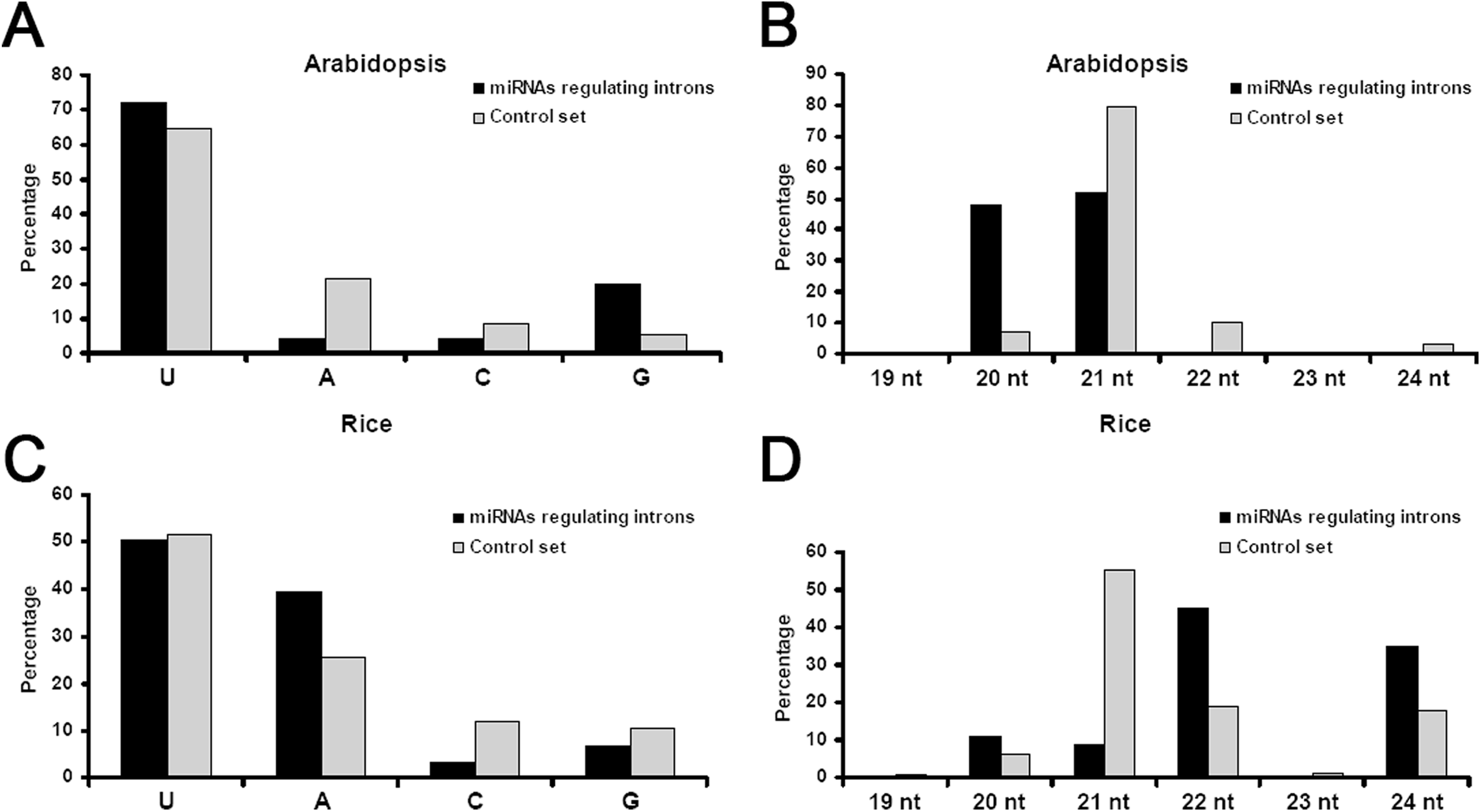

Supplement: Supplementary file 13 — Authors’ original file for figure 2 [file 12284_2012_45_MOESM13_ESM.tiff]

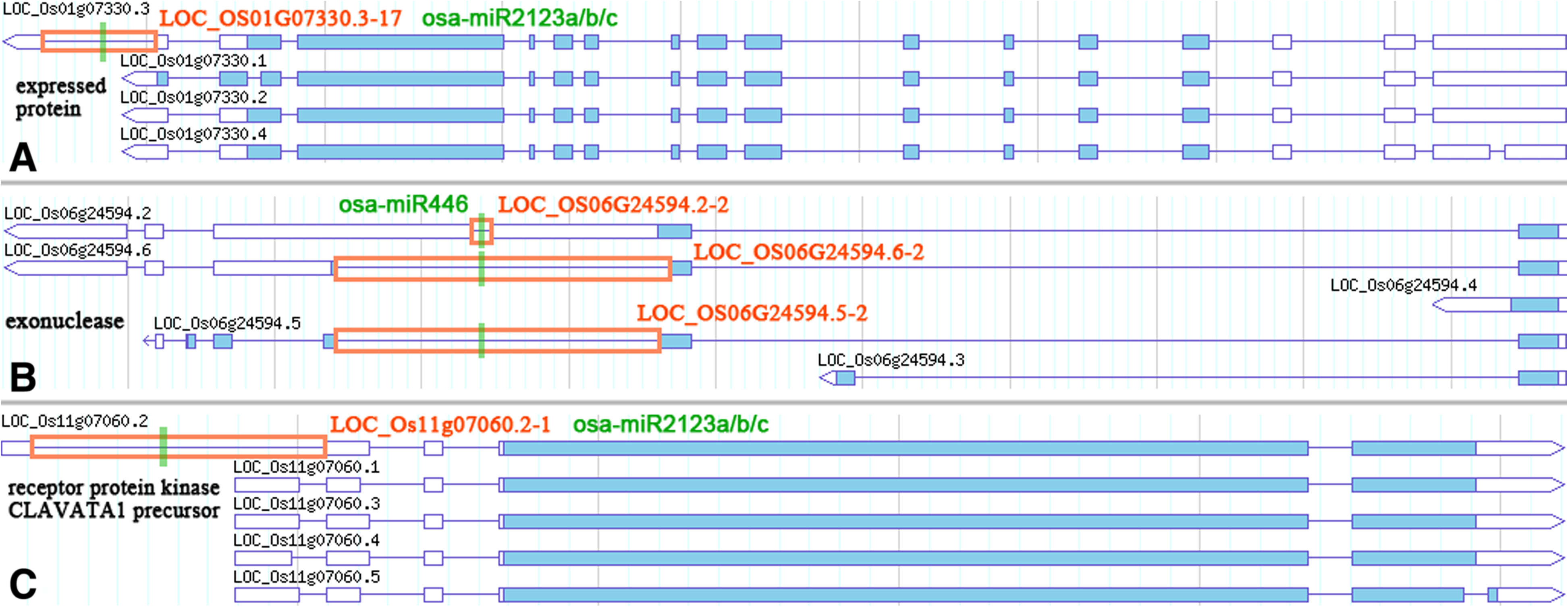

Supplement: Supplementary file 14 — Authors’ original file for figure 3 [file 12284_2012_45_MOESM14_ESM.tiff]

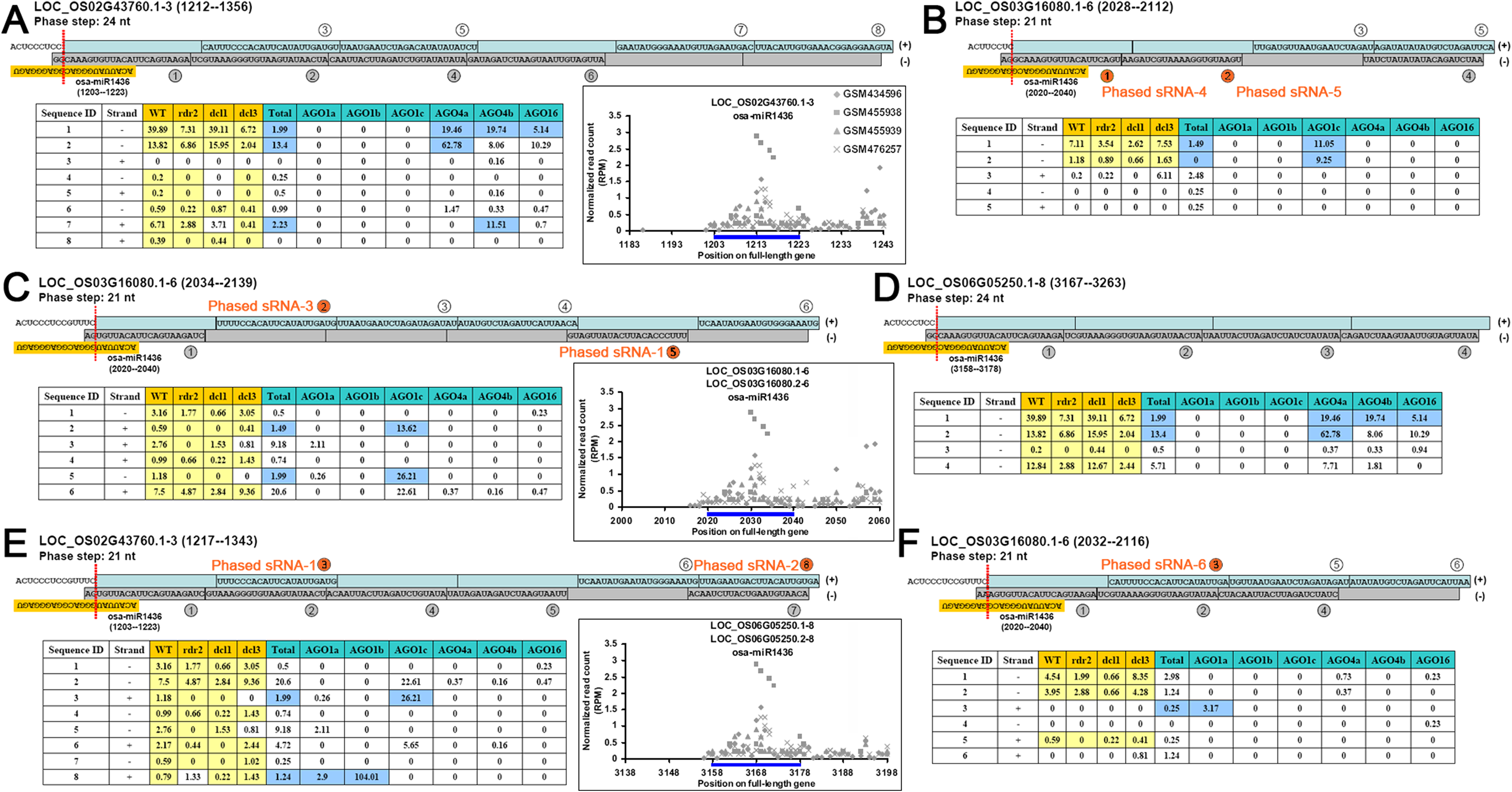

Supplement: Supplementary file 15 — Authors’ original file for figure 4 [file 12284_2012_45_MOESM15_ESM.tiff]

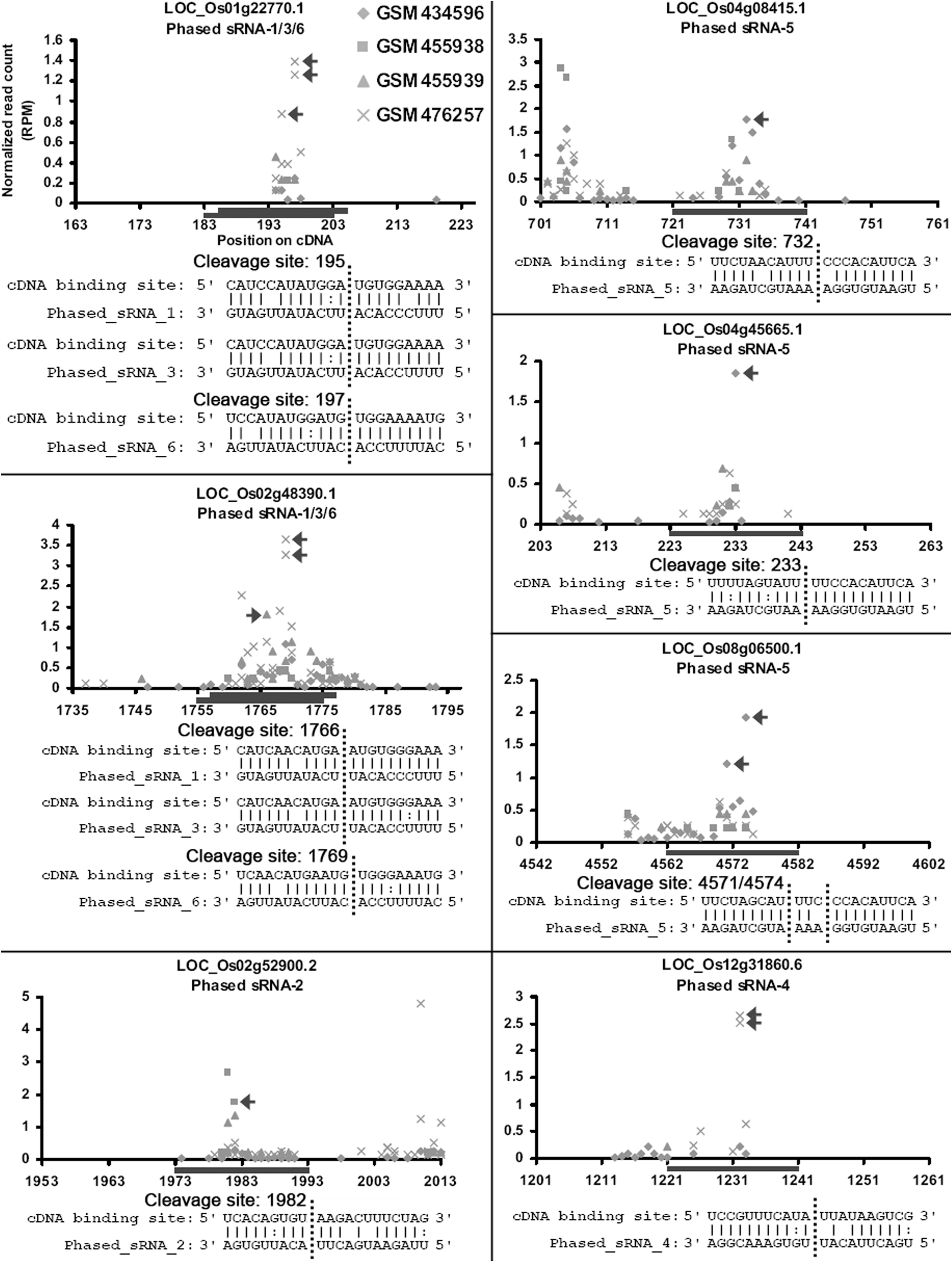

Supplement: Supplementary file 16 — Authors’ original file for figure 5 [file 12284_2012_45_MOESM16_ESM.tiff]

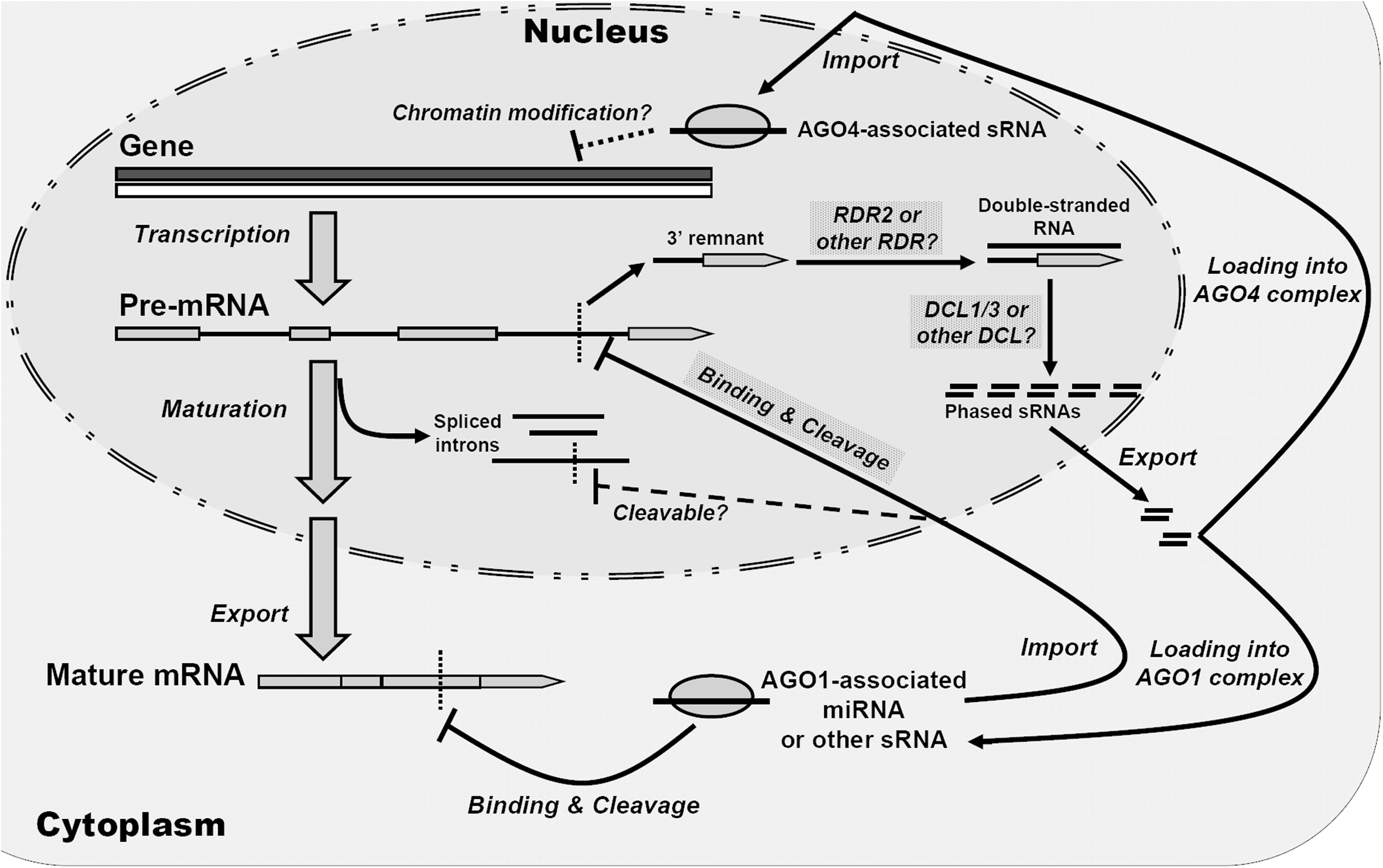

Supplement: Supplementary file 17 — Authors’ original file for figure 6 [file 12284_2012_45_MOESM17_ESM.tiff]
