# Supplementary material for: Introns targeted by plant microRNAs: a possible novel mechanism of gene regulation
Source: Rice (N Y). 2013 Apr 15;6:8. doi: 10.1186/1939-8433-6-8 (PMC4883735; doi:10.1186/1939-8433-6-8)
Supplement: Supplementary file 7 — Additional file 7: Figure S5: Observed distinct microRNA—intron regulatory patterns among different transcription models of a rice gene. (PDF 157 KB) [file 12284_2012_45_MOESM7_ESM.pdf]

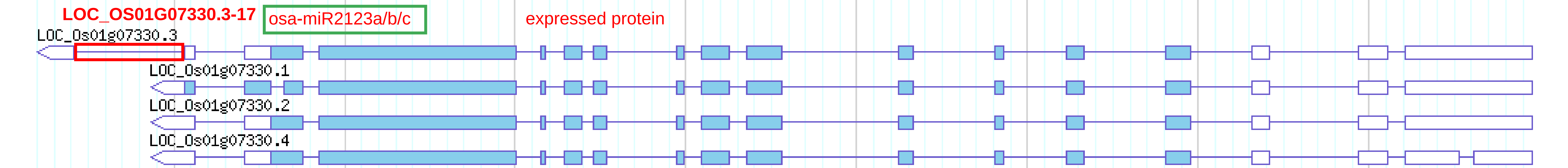

LOC_OS01G07330.3-17
osa-miR2123a/b/c
expressed protein
LOC_Os01g07330.3
LOC_Os01g07330.1
LOC_Os01g07330.2
LOC_Os01g07330.4

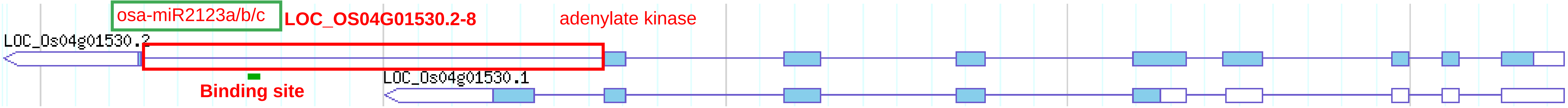
osa-miR2123a/b/c
LOC_OS04G01530.2-8
adenylate kinase
LOC_Os04g01530.2
LOC_Os04g01530.1
Binding site

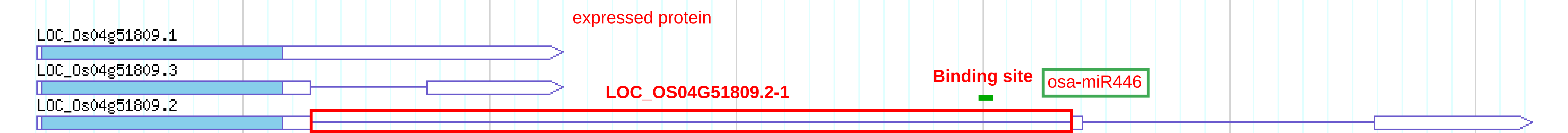
expressed protein
LOC_Os04g51809.1
LOC_Os04g51809.3
Binding site
osa-miR446
LOC_OS04G51809.2-1
LOC_Os04g51809.2

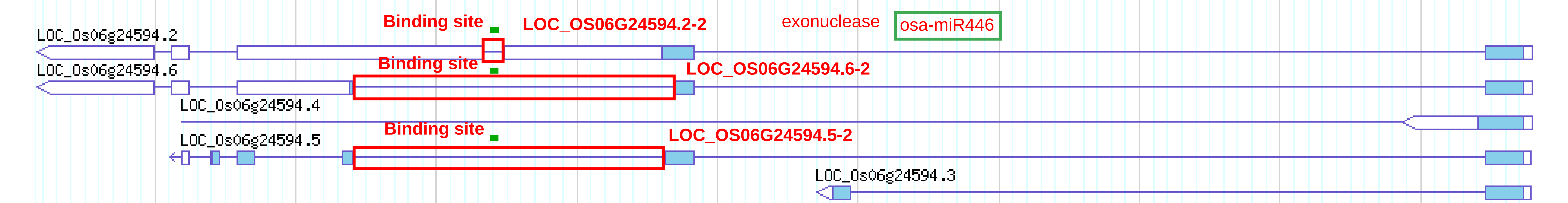
Binding site
LOC_OS06G24594.2-2
exonuclease
osa-miR446
LOC_Os06g24594.2
LOC_Os06g24594.6
Binding site
LOC_OS06G24594.6-2
LOC_Os06g24594.4
Binding site
LOC_OS06G24594.5-2
LOC_Os06g24594.5
LOC_Os06g24594.3

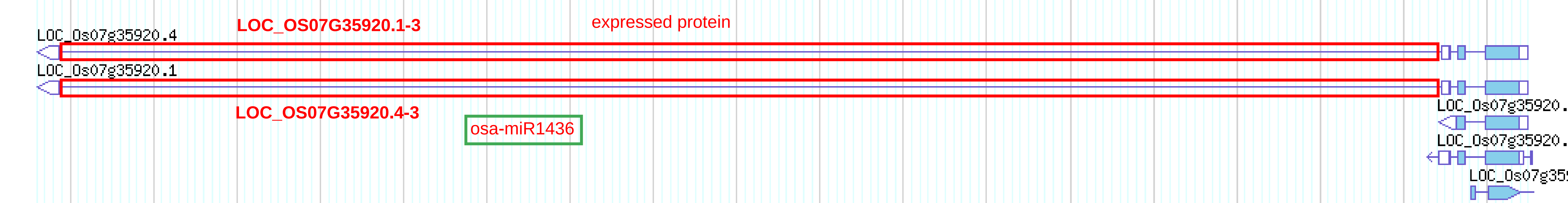

LOC_OS07G35920.1-3
expressed protein
LOC_Os07g35920.4
LOC_Os07g35920.1
LOC_OS07G35920.4-3
osa-miR1436

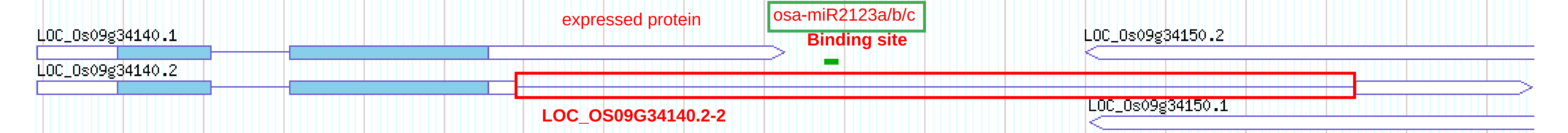
expressed protein
osa-miR2123a/b/c
Binding site
LOC_Os09g34140.1
LOC_Os09g34140.2
LOC_Os09g34150.2
LOC_Os09g34150.1
LOC_OS09G34140.2-2

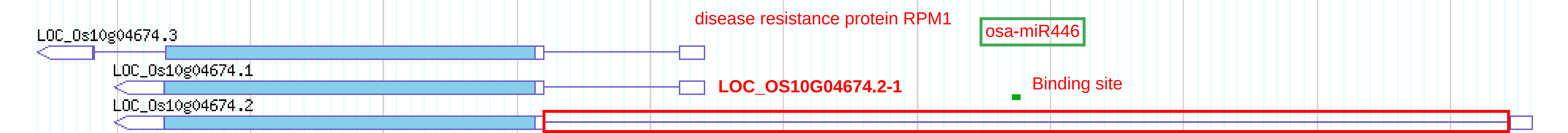
disease resistance protein RPM1
osa-miR446
LOC_Os10g04674.3
LOC_Os10g04674.1
LOC_OS10G04674.2-1
Binding site
LOC_Os10g04674.2

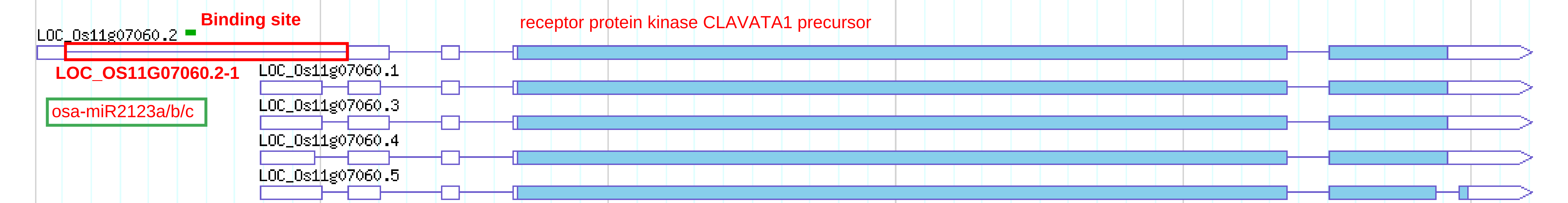
Binding site
receptor protein kinase CLAVATA1 precursor
LOC_Os11g07060.2
LOC_OS11G07060.2-1
LOC_Os11g07060.1
osa-miR2123a/b/c
LOC_Os11g07060.3
LOC_Os11g07060.4
LOC_Os11g07060.5

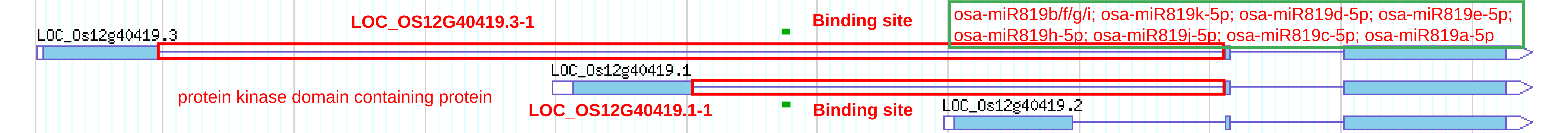
LOC_OS12G40419.3-1
Binding site
osa-miR819b/f/g/i; osa-miR819k-5p; osa-miR819d-5p; osa-miR819e-5p;
osa-miR819h-5p; osa-miR819j-5p; osa-miR819c-5p; osa-miR819a-5p
LOC_Os12g40419.3
LOC_Os12g40419.1
protein kinase domain containing protein
LOC_OS12G40419.1-1
Binding site
LOC_Os12g40419.2
